# Insights in Cellular and Molecular Signatures of the Small Intestinal Graft Posttransplantation: Successful, Recovered, and Rejected—A Case Series

**DOI:** 10.1097/TP.0000000000005411

**Published:** 2025-04-24

**Authors:** Naomi Karmi, Roy Oelen, Emilia Bigaeva, Sofie de Jong, Jan Willem Haveman, Frans van der Heide, Marcela A. Hermoso, Monique G.P. van der Wijst, Gursah Kats-Ugurlu, Rinse K. Weersma, Eleonora A.M. Festen, Werna T.C. Uniken Venema, Gerard Dijkstra, on behalf of the TransplantLines Investigators, C. Annema

**Affiliations:** 1 Department of Gastroenterology and Hepatology, University Medical Center Groningen, University of Groningen, Groningen, the Netherlands; 2 Department of Genetics, University Medical Center Groningen, University of Groningen, Groningen, the Netherlands; 3 Department of Surgery, University Medical Center Groningen, University of Groningen, Groningen, the Netherlands; 4 Laboratory of Innate Immunity, Immunology Program, Institute of Biomedical Sciences, Universidad de Chile, Santiago, Chile; 5 Department of Pathology, University Medical Center Groningen, University of Groningen, Groningen, the Netherlands

## Abstract

**Background.:**

Intestinal transplantation is the treatment for patients with irreversible intestinal failure and complications of parenteral nutrition. Five-year graft survival is only 56%, possibly due to an imbalance in immunosuppression, aiming to prevent rejection while maintaining protection against pathogens. Studying the graft’s mucosal cell populations and regulation of donor and recipient cells posttransplantation offers a unique opportunity to address this (im)balance leading to rejection.

**Methods.:**

We performed single-cell mRNA sequencing of longitudinally sampled ileal graft biopsies from surgery up to 6 mo after transplantation, althrough the TransplantLines Biobank and Cohort Study to characterize the composition and function of donor and recipient cell populations.

**Results.:**

A rapid influx of recipient immune cells was observed in the rejected transplant. Induction therapy using anti-thymocyte globulin did not achieve complete T-cell depletion. Instead, during moderate rejection, apoptotic pathways in epithelial cells preceded pathology-defined severe rejection, indicating potential prognostic information in the transcriptomic profiles.

**Conclusions.:**

This first longitudinal cellular-molecular study of the total ileal graft mucosa and recipient cells within, shows a variable clinical course and response to medication, which align with heterogeneous signatures before intestinal transplantation rejection.

## INTRODUCTION

Intestinal failure (IF) is a life-threatening condition occurring in 5–80 patients per million Europeans,^1^ characterized by the disability of the bowel to regulate electrolytes and absorb nutrients and water. Most patients with IF require parenteral nutrition, commonly leading to complications such as thrombosis and infection. Yearly, 150–250 patients worldwide receive intestinal transplantation (ITx) to improve quality of life and survival.^2^

Despite screening and immunosuppression, graft failure after ITx is 44% at 5 y,^2^ marking the highest failure rate among solid organ transplants.

Shifts in cellular composition, such as increased immune cell presence and loss of epithelium, are seen in graft failure. The gut hosts more immune cells than any other organ, rendering CD4^+^ and CD8^+^ T cells the main players in the proposed mechanisms for graft rejection.^3-6^ Activated T cells initiate B-cell signaling, leading to the formation of donor-specific antibodies (DSA), utilized as clinical markers for humoral rejection.^7^ In nontransplanted intestinal studies, various mucosal antigen-presenting cells are identified as potential rejection mediators.^8-10^ Despite this knowledge, accurate prediction of rejection remains a challenge.

To prevent graft rejection, patients receive initial immunosuppressive induction therapy, such as rabbit anti-thymocyte globulin (rATG) or biologics targeting T and B lymphocytic cell activation, like anti-CD52 (alemtuzumab) and anti-IL2 (basiliximab),^2^ and maintenance therapy, eg, with tacrolimus. The balance between effective immunosuppression and minimal infection rates, posttransplantation lymphoproliferative disorder, and graft-versus-host disease poses a persistent challenge. If initial therapy proves ineffective, rejection is addressed with additional immunosuppressants. In such cases, infliximab (IFX) has shown promise, especially in patients with an enrichment of T-helper (Th) 17 cells.^11^ A comprehensive understanding of small bowel engraftment and medication effects at a cellular level is essential for selecting the most optimal therapeutic regimen optimizing graft survival and minimizing rejection.

Recent studies using single-cell mRNA sequencing (scRNAseq) have characterized the small intestinal immune system in health,^9^ disease,^12,13^ and development.^12,13^ Some studies have explored intragraft subsets of T cells and macrophages and described repopulation by the recipient cells.^14-16^ However, the interplay between the total immunogenic transplanted bowel and the recipient body in the first months posttransplantation remains elusive. Our hypothesis posits that the constitution of an imbalanced recipient’s immune compartment is a key factor in rejection. In this case series, we investigated immune cell repopulation of the small intestinal graft mucosa from 3 patients treated at the University Medical Center Groningen (UMCG) from the time of surgery up to 6 mo after transplantation by scRNAseq. This study represents a detailed examination of longitudinal changes within the small intestinal graft on cellular, chimeric, and molecular levels.

## MATERIALS AND METHODS

### Patient Cohort

This case series was conducted at the Department of Gastroenterology and Hepatology of the UMCG, the Netherlands. Patients were included in the ongoing TransplantLines Biobank and Cohort study (NCT03272841), which has been approved by the local Institutional Review Board (METc 2014/077), adheres to the UMCG Biobank Regulation, and is in accordance with the World Medical Association Declaration of Helsinki and the Declaration of Istanbul. This study prospectively included 3 patients undergoing ITx due to advanced IF.

**FIGURE 1.**
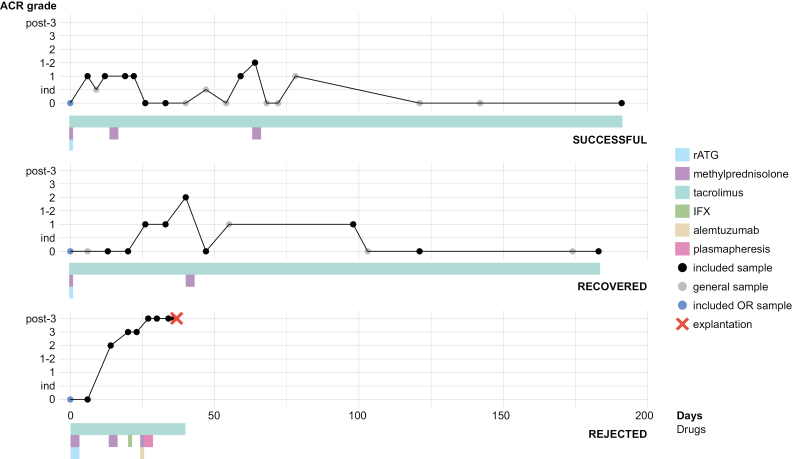
An overview of acute cellular rejection and treatment intervention over time in the successful, recovered, and rejected transplant. ACR grades over the number of days after transplantation per transplant (successful, recovered, and rejected). Follow-up between transplants varied from 34 to 191 d. Each dot represents a sample and time point: blue, only the donor ileum collected during transplantation; blue and black, used for scRNAseq. Stacked colored beams represent the relevant medication interventions (rATG, methylprednisolone, tacrolimus, IFX, alemtuzumab, and plasmapheresis) for the prevention and treatment of rejection. ACR, acute cellular rejection; IFX, infliximab; ind, indeterminate; OR, operation room; rATG, rabbit anti-thymocyte globulin; scRNAseq, single-cell mRNA sequencing.

Treatment regimens can be found in Figure 1. Rejection was monitored based on clinical performance, blood sample collection, and graft gut mucosal biopsies. Biopsies taken within the study sampling field were evaluated by a pathologist. Histology was assessed using acute cellular rejection (ACR) grading (Figure 1 and Table 1). Mild rejection was treated with intensified tacrolimus, while moderate rejection received methylprednisolone (500 mg IV <40 kg or 1000 mg IV >40 kg for 3 d). In severe rejection, a combination of these treatments was administered. Nonresolving cases were managed with a single dose of alemtuzumab (30 mg). For therapy-resistant rejection, IFX (600 mg) was administered according to described treatment strategies.^11^

**TABLE 1.** Histology rating of acute cellular rejection in intestinal transplantation

	Grade
No evidence of acute rejection	0
Indeterminate for acute rejection	ind
Acute cellular rejection	1 (mild)
Acute cellular rejection	2 (moderate)
Acute cellular rejection	3 (severe)

This table represents the general annotation by the pathologist used in this study.

ACR, acute cellular rejection; ind, indeterminate.

### Sample Collection and Cryopreservation

From the day of transplantation (ie, T0, day 0, or baseline) up to 6 mo posttransplantation, 8–10 time points per patient were selected based on clinical relevance. All 28 samples (3 biopsies/sample) were cryopreserved in cold freezing medium and processed according to the “one-step collagenase” protocol.^17^

### Generation of Transcriptomes and Genotypes

In short, cryopreserved biopsies were incubated with collagenase, EDTA, and TrypLE express (Thermo Fisher) and washed.^17^ Consecutively, cells were filtered and resuspended. Viability and cell counts were assessed using a Bürker-Türk counting chamber and Trypan Blue staining. Libraries were prepared with the 10x genomics Chromium Next GEM Single-Cell 3′ Kit v3.1. Up to 3 samples per 10x chip channel were multiplexed to limit batch effects, aiming for a total recovery of 4000 cells/sample. All libraries were indexed (10x Genomics, PN-3000431), pooled, and sequenced on an MGISEQ-2000 platform (BGI, Hong Kong) featuring 28bp, 10bp, 10bp, and 90 bp (read 1, index, read 2) paired-end reads. The cDNA libraries were sequenced at 60 000 reads/cell. DNA was isolated with an Allprep DNA/RNA mini kit (Qiagen) Genotypes from donors and recipients were generated per Illumina Global Screening Array (GSA) Arrays “Infinium iSelect 24x1 HTS Custom Beadchip Kit” and called using GenomeStudio software and imputed using the sc-eQTLgen consortium pipeline (https://github.com/sc-eQTLgen-consortium/WG1-pipeline-QC).^18^

### Preprocessing of Single-cell Transcriptomics

ScRNAseq data were aligned using the hg38 human reference genome and processed using CellRanger (v7.0.0), which rendered 4457 cells per sample on average (129 252 total). Souporcell (v2.0) was next used to identify doublets and assign cells to genotypic clusters, represented by both donor and recipient cells.^19^ These genotypic clusters were subsequently assigned to donors or recipients by correlating the cluster genotypes to the GSA genotypes.

Gene expression data were loaded into Seurat (v4.1.1),^20^ where dimensional reduction using principal component (PC) analysis was performed and Uniform Manifold Approximation and Projection (UMAP), and K-Nearest Neighbors-clustering at a resolution of 1.2 was performed using the first 30 PC. After filtering 3213 cells per sample on average remained (90 282 total). Batch effects were controlled for by checking if identified clusters consisted largely of singular experimental lanes (**Figure S1, SDC**, http://links.lww.com/TP/D263).

scRNAseq count data and cell-type annotations from Elmentaite et al^9^ were loaded in Seurat and filtered to only contain cells from healthy ileal samples. Thes data were subsequently normalized and clustered in the same way as described in the previous section. The resulting dimensional reduction data were used to perform cell-type annotation of the data in this case series, by projecting cells onto the Elmentaite et al^9^ data as a reference dataset, allowing cell-type assignment label transfer.

Since this reference dataset does not include regulatory T cells (Treg) and Th17 cells, the activated CD4^+^ T-cell superset was taken as a subset of the data, and *AREG, IKZF2, RORC, FOXP3, IL7R* high, and *IL2RA* low expressing cells and *IL17A, RORA,* and *TNF* high expressing cells were again clustered (PC 1:10, resolution 0.4) separately (**Figure S2, SDC**, http://links.lww.com/TP/D263).^21,22^ Cells were then annotated to a lower granularity annotation to allow for sufficient observations in some analyses (**Table S1, SDC**, http://links.lww.com/TP/D263; https://github.com/WeersmaLabIBD/DDTX_suppl_materials).

### Cross-check Genotype Calling

Given the novelty of scRNAseq analysis for both the recipient and donor cells in the graft and the lack of reference data, we conducted a thorough cross-check of the genotype calling of single cells. To ensure the integrity of the data, we confirmed that the loss of cells during the mitochondrial and doublet filter steps did not predominantly affect recipient or donor cells.

### Differential Composition Analysis

To put the cell-type composition of the ITx graft and changes over time into the perspective of a “normal” range the dataset of Elmentaite et al^9^ was used to retrieve healthy adult ileum cell counts. Percentages of CD45^−^ and CD45^+^ cell subtypes were calculated to represent the average range of healthy ileal cell count in this case series.

To address data proportionality caused by differences in cell count recovery, we used random downsampling to the lowest number of cells per sample (1949) for compositional analyses, resulting in a total of 54 224 cells. Unfortunately, statistical analysis of cell abundances was not reliable due to the small cohort size.

### Differential Expression and Pathway Analysis

Differentially expressed genes were identified using the “FindAllMarkers” function in the Seurat R package with “MAST.”^23^ Adjusted *P* were considered significant at < 0.05 after Bonferroni multiple testing correction, and only log2 fold change > 0.1 was deemed significant. The statistically significant differentially expressed genes of cell types of interest per transplant per time point were used as input for Reactome pathway analysis (https://reactome.org/).

## RESULTS

### Three Small Intestinal Transplants Show Distinct Clinical Outcomes

This case series features the cellular landscape of the small intestinal transplant of 3 patients over 6 mo postsurgery consisting of 8–10 time points per patient (28 total). The patients showed variable clinical disease courses: 1 patient endured a short period of mild rejection with a maximum of ACR grade 1–2, thus representing the general course of successful transplantation (hereafter named “successful transplant”). The second patient experienced moderate rejection and showed resolution after treatment (“recovered transplant”). The third patient faced therapy-resistant rejection where ACR grade 2 developed into severe rejection and eventually explantation (“rejected transplant”) (Figure 1).

Patient characteristics are listed in Table 2, where obesity was observed in the rejected patient, however, this was not further explored within the study. The presence of nucleotide-binding oligomerization domain-containing protein 2 Crohn’s disease associated alternative alleles was assessed, as these were shown to increase the risk of ITx allograft rejection (a hazard ratio of 4.449).^24-26^ We identified no alternative alleles in the rejected recipient: R702W is homozygous reference in all transplants, G908R is only heterozygous in the donor of the rejected transplant, and fs1007insC was not identified in our genotype data.

**TABLE 2.** Patient cohort characteristics

	Transplants		
	Successful	Recovered	Rejected
Age range at transplantation			
Donor	31–35	21–25	21–25
Recipient	31–35	21–25	46–50
Sex	Female	Female	Male
Transplant type	Combined small intestinal and abdominal wall transplant	Isolated small intestinal transplant	Combined small intestinal and abdominal wall transplant
Indication	Ultra-short bowel syndrome	Short bowel syndrome and motility	Short bowel syndrome and malabsorption
PN complications or malabsorption	Osteoporosis, IFALD	Osteoporosis, IFALD	Mg, P, NaCl dependency
Other diseases	DM	Hemochromatosis	SLE, APS, HT, CD
BMI^*a*^ at transplantation (kg/m^2^)	Normal weight (22.9)	Underweight (18.4)	Obesity (34.2)
CMV status			
Donor	Positive	Negative	Negative
Recipient	Positive	Positive	Negative
EBV status recipient	Positive	Positive	Positive
HLA-antibodies recipient	No	No	Yes

a BMI according to WHO 2023. APS, antiphospholipid syndrome; BMI, body mass index; CD, Crohn’s disease; CMV, cytomegalovirus; DM, diabetes mellitus; EBV, Epstein-Barr virus; HT, hypertension; IFALD, intestinal failure-associated liver disease; Mg, magnesium; NaCl, sodium chloride; P, potassium; PN, parenteral nutrition; SLE, systemic lupus erythematosus.

### High-resolution Cell-type Profiling of the Ileal Recipient and Donor Cells in Intestinal Transplants Shows Intragraft Recipient Immune Cell Repopulation

**FIGURE 2.**
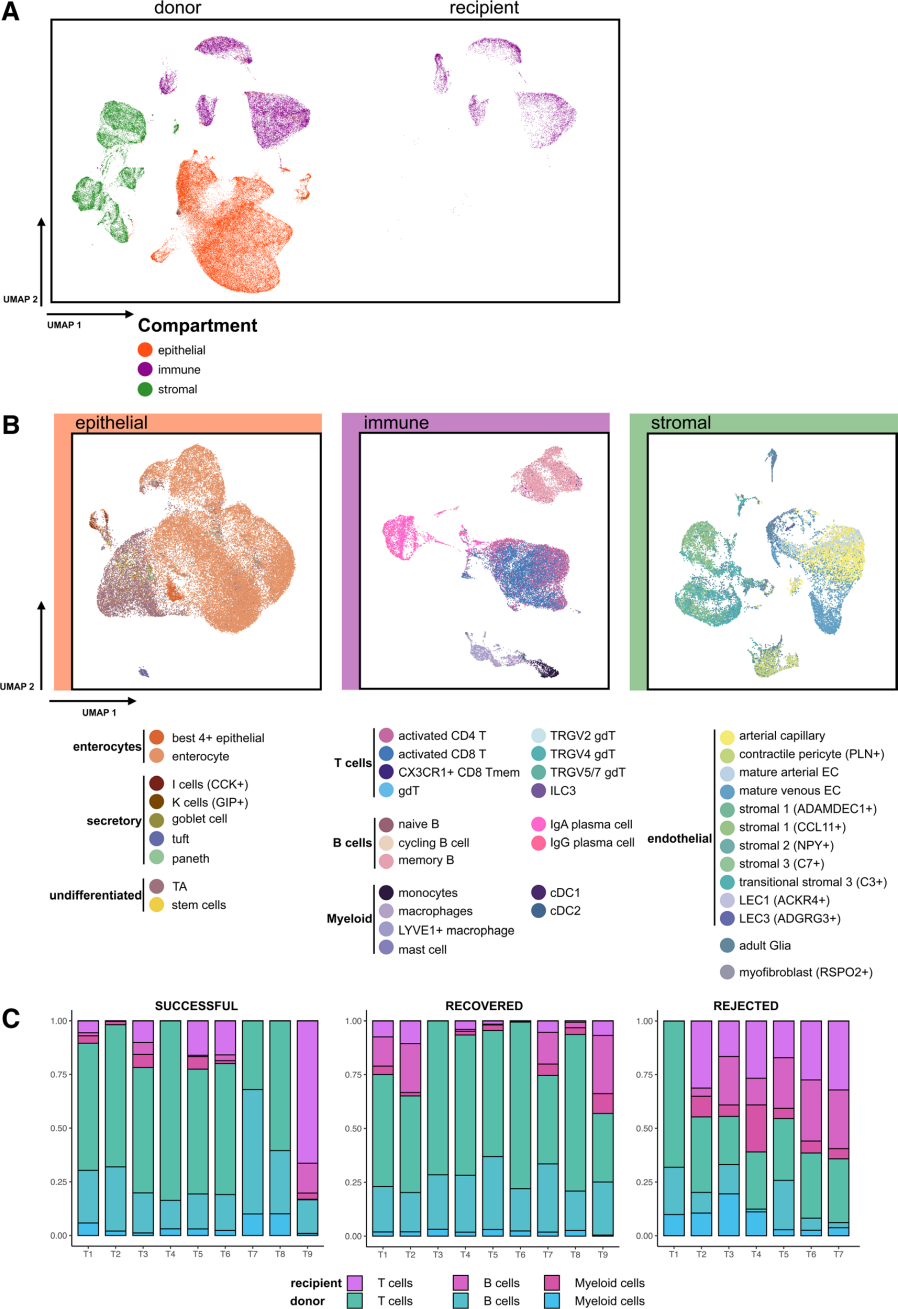
High-resolution single-cell profiling of mucosal ileal graft in intestinal transplantation. A, UMAP view of 90 282 ileal mucosal single-cell transcriptomes colored by compartment and grouped by origin (on the left donor and the right recipient). All cells, irrespective of time point are included in the UMAPs. B, Cells are annotated at a high granularity resulting in 41 cell types in total. Cells are grouped by the epithelial compartment on the left, the immune compartment in the middle and the stromal compartment on the right. C, Stacked bargraphs show percentages of the immune compartment, donor in shades of turquois and recipient in pinks, of each sampled time point (T) per transplant (successful, recovered and rejected). x-Axis shows time points between 0 and 191 d, where T7 is 34 d after transplantation in the rejected transplant. y-Axis shows the percentage of the T, B, and myeloid donor and recipient cells. T, time point; UMAP, Uniform Manifold Approximation and Projection.

To characterize the cell composition of the 28 ileal graft samples, we generated 90 282 high-quality gut mucosal single-cell transcriptomes, which have been annotated into 3 major cell compartments: epithelial, immune, and stromal. As expected, recipient cells were mostly of immune origin (Figure 2A).

Based on a reference-mapping approach, further subclassification of donor and recipient cells identified 41 distinct cell types (ie, high granularity) (Figure 2B). Immune cell proportions of the recipient and donor were plotted per transplant over time. Studying recipient and donor cells separately, we confirmed previous observations of gut mucosal chimerism in all patients within the first 2 wk (T1–T2) after transplantation (Figure 2C).^3,27^ A rapid and progressive influx of recipient immune cells was observed in the rejected transplant within 34 d (T1–T7), mostly consisting of T and B cells. Meanwhile, the presence of recipient immune cells in other transplants generally fluctuated. This is in line with Zuber et al,^28^ who showed that a slower repopulation of recipient T cells in the intestinal graft was associated with a reduced risk of rejection. The high percentage of recipient immune cells at T9 (day 191) was linked to cytomegalovirus reactivation and not rejection based on pathology and microbiology reports (Figure 2C).

It should be noted that certain immune cell types could not be included in our downstream analysis due to their low quantities across samples (Th17 cells, Treg, innate lymphoid cells,^29^ and dendritic cells) or not being captured by our dissociation protocol (neutrophils).^30^

### Nonreversible Depletion of Epithelial Cells Upon Acute Cellular Rejection Grade 2 in the Rejected Transplant

Next, we compared the graft cell-type composition to that of a healthy adult ileum^9^ and described the dynamics that take place shortly after transplantation up to 6 mo when a more stable clinical state is achieved.

**FIGURE 3.**
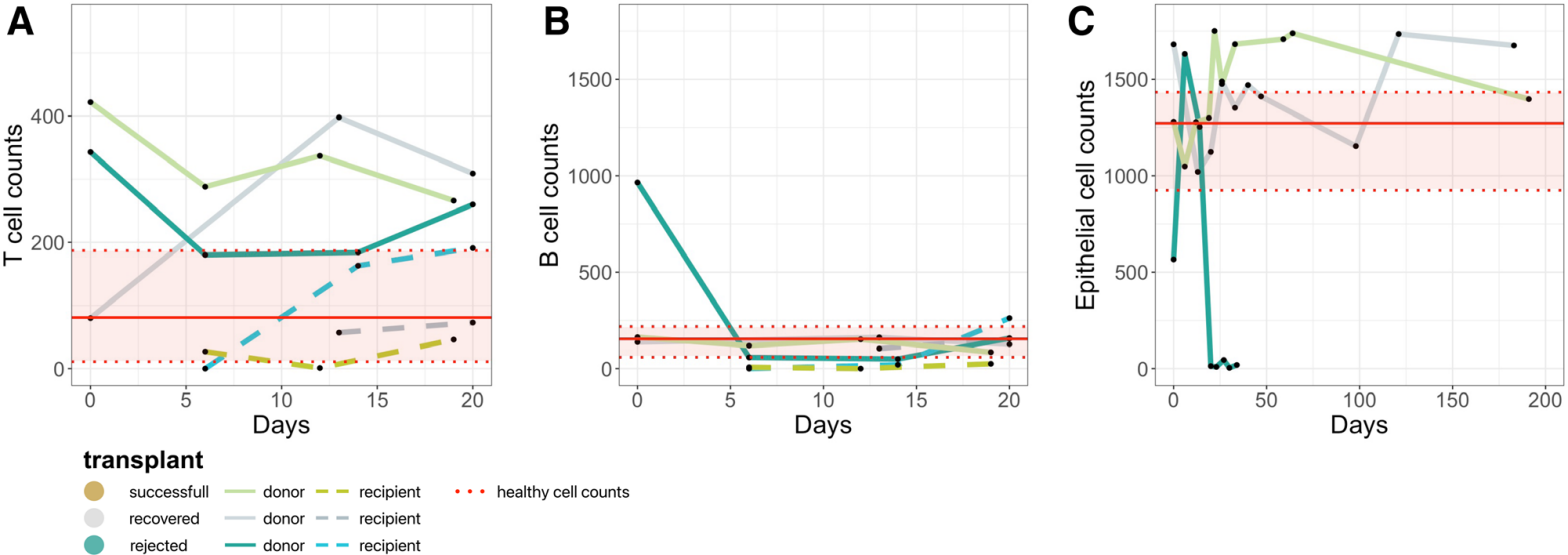
Cell count dynamics vary over 6 mo posttransplantation in transplants. A–C, x-Axis presents days and y-axis presents cell counts of a given cell population in the downsampled dataset. Each dot represents 1 of 28 samples. Colors indicate distinct transplants. Red solid lines indicate the average and red dotted lines indicate the “normal” range of healthy ileal cell counts.^9^ A, Samples split by origin (donor in straight vs recipient in dashed lines), y-axis represents activated T-cell counts. B, Samples split by origin (donor in straight vs recipient in dashed lines), y-axis represents B-cell counts. C, y-Axis represents epithelial cell counts.

First, we focused on the immune compartment. At transplantation, the initial T-cell count of the successful transplant was outside the healthy ileum T-cell count range. The T- and B-cell counts of the recovered transplant were within the normal range, while those of the rejected transplant were outside the normal range (Figure 3A and B).

Next, we investigated endothelial and epithelial cell populations. In the successful and recovered transplants, we observed relatively stable donor epithelial and endothelial cell counts, aligning with normal values and showing stabilization over 6 mo (Figure 3C; **Figure S3, SDC**, http://links.lww.com/TP/D263). Conversely, in the rejected transplant, baseline epithelial cell counts were not within the healthy range, followed by a sharp change in epithelial cell count (at ACR grade 0) and subsequent sustained complete depletion without signs of regeneration as ACR grade increased to 2 and 3 (Figure 3C). Anti-rejection therapy could not stop or reverse the depletion. Neither could a change in stem cells in the rejected transplant during ACR grade 2 (day 14) followed by complete stem cell depletion (**Table S1, SDC, sheet 2**, http://links.lww.com/TP/D263). In contrast, the rejected transplant displayed a continuous trend of expansion of the endothelial compartment (**Figure S3, SDC**, http://links.lww.com/TP/D263). During this trend an increase in the presence of DSA was observed, full overview available in **Table S2, SDC**, http://links.lww.com/TP/D263, suggesting a possible relation between endothelial damage and DSA formation. These observations were not present in the recovered and successful transplants.

A full overview of the cell counts at all time points can be found in **Table S1, SDC**, http://links.lww.com/TP/D263.

### rATG Induction Does Not Totally Deplete Donor T Cells in the Intestinal Graft

To uncover markers of therapy resistance, we investigated the impact of immunosuppressants on cell-type composition in each transplant (Figure 1; **Table S1, SDC**, http://links.lww.com/TP/D263).

rATG induction immunosuppression is designed to deplete T cells pre- and intraoperatively.^31^ The successful and recovered transplants received a single high dose of rATG, while the rejected transplant received a daily lower dose on postoperative days 0, 1, 2, and 3, as per a clinical protocol update. Results showed heterogeneous responses: a decreasing trend in donor T cells after treatment in both the successful (days 0–6) and rejected (days 0–6) transplants and an expansion in the recovered transplant (days 0–13). Contrary to its commonly known working mechanism, rATG did not achieve complete donor T-cell depletion regardless of the time or dosage.

Next, we investigated the effect of methylprednisolone which has an immunosuppressive, anti-inflammatory, and lymphocytic effect. All 3 patients received at least 1 treatment cycle. Although the successful transplant only experienced a short period of mild rejection, the patient received 2 cycles of methylprednisolone (days 14–16 and 59–61) resulting in a reduction of T and B cells as observed as trend in the first but not the second cycle. In the recovered transplant, however, trends of increasing donor T cells, and decreasing donor B cells after methylprednisolone (days 41–43) were observed. Recipient cell counts of these patients were generally too low to study. In the rejected transplant, we observed an expansion in donor and recipient B and T cells after administration of methylprednisolone (days 15–18), which may be a sign of therapy resistance.

Following the treatment scheme of Kroemer et al^11^ for therapy-resistant ITx rejection, the rejected transplant was treated with IFX at day 7 postrejection. T-cell abundance was not affected (451–447) by IFX on day 20. Moreover, examining the trends in the presence of Th17 cells in the activated CD4^+^ T-cell subset, we found an abundance of 16.2% in nonrejection compared with 8.2% in rejection (ACR grade 1–3), a contrast to what has been described previously.^11^ At day 3 post-IFX, the patient still suffered from an ongoing graft rejection (as confirmed by histological analysis). Consequently, treatment with alemtuzumab which targets T cells, B cells, natural killer cells, monocytes, and macrophages was initiated in the rejected transplant (day 24),^32^ whereafter we noticed trends of rising B-cell counts, and declining T and myeloid cell counts. This treatment coincided with IVIG administration (days 25–28). IVIG theoretically neutralizes pathogenic autoantibodies, regulates B cells and antibody synthesis, disturbs the composition of Th cells and downregulates their cytokine production.^33^ Despite extensive immunosuppressive therapy, healing was not achieved in the rejected transplant leading to graft explantation.

### Apoptotic Pathways in Acute Cellular Rejection Grade 2 Precede Severe Rejection

It remains unclear why moderate rejection (ACR grade 2) resolves in the recovered but not in the rejected transplant. We therefore studied differentially overexpressed pathways in multiple cell types important in rejection to uncover relevant pathway changes (**Table S3, SDC**, http://links.lww.com/TP/D263).

**FIGURE 4.**
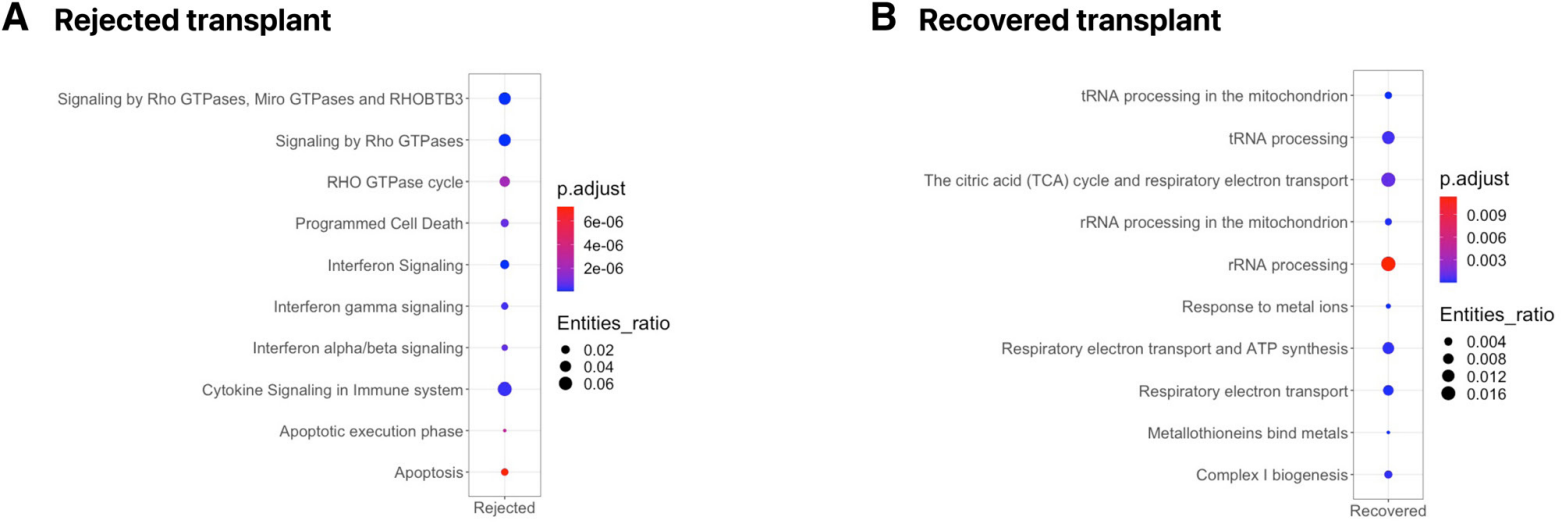
Gene expression pathways and not traditional apoptotic body counts precede severe rejection. A shows the rejected transplant, and B shows the recovered transplant during moderate (ACR grade 2) rejection. Dot plot images of the top overexpressed pathways recovered by Reactome shown by their adjusted *P* (Benjamini–Hochberg) and entity ratio. ACR, acute cellular rejection.

In the rejected transplant at ACR grade 2, epithelial cells regulated pathways such as inflammation (interferon signaling), apoptosis, and barrier function regulation (ρ GTPase signaling) (Figure 4A), whereas pathways in the recovered transplant reflected cellular metabolic homeostasis (tRNA processing and ATP synthesis) (Figure 4B). In the rejected transplant, activated CD4^+^ and CD8^+^ T cells showed interferon signaling while this was not the case in the recovered transplant. Signaling by ρ GTPase was present in the activated CD8^+^ T cells of both rejected and recovered transplants during ACR grade 2. Activated CD4^+^ T cells of the rejected transplant expressed the RUNX1 pathway that regulates the differentiation of hematopoietic stem cells and the development of Treg. Overexpressed pathways in endothelial cells in the rejected transplant during ACR grade 2 were related to extracellular matrix organization, collagen formation, and interferon and cytokine signaling. Gene expression suggests activation (*VCAM11, BATF2, BATF3,* and *MMP31*). A sign of nonresponse to corticosteroids was the absence of *PRDM1* upregulation in B cells of the rejected transplant after methylprednisolone, compared with the recovered transplant which expressed this corticosteroid-responsive gene required for terminal differentiation and reduced proliferation of B cells.^34^

Apoptosis of the crypt epithelium is considered an objective feature of ACR.^35^ Based on the pathology reports one could stipulate that the rejected and recovered transplant (>6 and >7 apoptotic bodies in the crypt epithelium per 10 consecutive crypts, respectively; **Figure S4** and **Table S4, SDC**, http://links.lww.com/TP/D263) endure similar severity of rejection, while they have disparate graft survival. Gene expression and not pathology analyses show a discrepancy between rejected and recovered transplants at ACR grade 2, suggesting that the presence of apoptotic pathways can indicate a worse rejection course.

## DISCUSSION

The primary objective of this case series was to elucidate individual cell dynamics in ITx over 6 mo postsurgery and to evaluate graft changes associated with ACR or medical therapy. We aimed to enhance our understanding of the cellular and molecular mechanisms of engraftment to ultimately improve transplant outcomes. To achieve this, we compiled a dataset comprising recipient and donor gut mucosal cells obtained from 3 intestinal transplants with different clinical outcomes, showing early signs of severe rejection in molecular data before it was detected by endoscopy or pathology.

First, we see gut mucosal chimerism of the ileal graft within 2 wk after transplantation in all transplants. This aligns with the current understanding that recipient T cells migrate to the intestine and replace donor T cells at an early point.^36-38^ We hypothesized that the constitution of an imbalanced recipient’s immune compartment contributes to rejection, which was confirmed in the rejected transplant by the early onset of immune recipient cell influx. Zuber et al^28^ suggested that a slower replacement of donor T cells in the intestinal graft by recipient T cells is associated with a lower risk of rejection. Notably, tissue-resident memory CD4^+^ and CD8^+^ T cells can persist for up to 5 y in small bowel grafts.^39^ Consistent with this, our dataset showed that T cells were predominantly of donor origin at 6 mo for the successful and recovered patient. B cells can be activated when antigen engages their receptors within the transplant leading to alloantibody formation against donor HLA antigens, however, this was not reflected by their pathways.^40^ Nevertheless, the observed high B-cell count in the donor graft at baseline in the rejected transplant could be an early indicator of severe rejection and should be explored in further studies.

Rejection is thought to be marked by the presence of CD4^+^ and CD8^+^ T cells^3-6^; yet we find an increased presence of donor T cells in both the successful and rejected transplants at baseline. Recipient-derived lymphocytes in the graft have been shown to produce interferon(-gamma), a critical cytokine in acute rejection.^3^ We see interferon pathways only in the rejected, and not in the recovered transplant at ACR grade 2, leading to the hypothesis that interferon pathways may precede severe rejection. Although we could not analyze Treg specifically, we do see overexpression of the pathway involved in the development of Treg (RUNX1) in activated CD4^+^ T cells. This pathway is overexpressed in the recovered and rejected transplant 1 wk before the onset of ACR grade 2, possibly indicating an attempt to limit acute allograft rejection.

ρ GTPases are involved in multiple T-cell functions and regulation of the intestinal epithelial barrier functions, which could avoid the development of local immune reactions and therefore inflammatory bowel disease.^41^ Signaling by ρ GTPase is observed in the epithelial cells of the rejected transplant only, and in the activated CD8^+^ T cells of both transplants during ACR grade 2. In theory, if inflammation in inflammatory bowel disease resembles ACR in ITx, dysregulation of ρ GTPase can also lead to the development of ACR and be a possible indicator of rejection outcome. Pathway analysis of B cells of the rejected transplant did not suggest terminal differentiation and reduced proliferation.

Severe rejection in our cohort is characterized by a trend of expansion of the endothelial compartment, mostly mature venous endothelial and transitional stromal cells, which express collagen formation pathways and activation markers. This may be explained as the development of granular tissue in the rejected graft. Compositional changes are solely observed in this compartment and therefore likely to be a biological effect. Another marker of rejection is the total depletion of epithelial cells due to apoptosis and damage to the crypt epithelium as shown in the rejected transplant. Importantly, we show that gene expression changes in epithelial cells precede histologic signs of severe rejection, an observation that should be confirmed in a larger cohort.

All 3 transplants had rATG as induction therapy, which did not result in the complete depletion of (donor) T cells. This was also observed in mouse models.^42^ Causal could be the antigen-experienced character of the tissue-resident T cells as suggested by Park et al^43^ Because of our study design, the initial effect of rATG on recipient cells specifically could not be analyzed. When interpreting these results, we should consider polypharmacy in transplantation which could not be accounted for in this cohort. Altogether, rATG is still considered the safest form of induction therapy in ITx.^44^ The authors of this article propose to explore the addition of the gut-selective drug vedolizumab and low-dosage tacrolimus as part of the ITx induction protocol to optimize (gut) T-cell depletion. ScRNAseq data did not reveal why the rejected transplant endured therapy resistance. One possible explanation could be that genetic predisposition to Crohn’s disease increases the risk of rejection, although we could not confirm recipients carried nucleotide-binding oligomerization domain-containing protein 2 risk alleles.

This case series only focused on ileal samples, while rejection could differ throughout the transplanted small bowel. Another limitation of this study is the observational aspect of the data, as a result, we could not analyze time points between days of sample collection. However, the current approach of using engraftment profiles to test our hypothesis effectively addresses patient-specific effects, which are crucial confounders in our data analysis. We used a downsampled dataset to account for proportionality. Due to the small cohort size, we could not draw any conclusions on small subpopulations like Treg and Th17 cells.

To the best of our knowledge, this is the first prospective longitudinal study in ITx that specifically addresses the early episodes of ACR and immunosuppression over 6 mo follow-up and demonstrates successful separation of all donor and recipient cells within an ileal graft biopsy, using computational methods. This case series can function as a foundation for larger studies through international collaborations to better understand rejection and eventually design gut-specific treatment strategies. We found molecular signs for severe rejection preceding histologic features, providing interesting leads for further large-scale studies requiring international collaborations.

## ACKNOWLEDGMENTS

The authors thank the participants who contributed their biological materials, and all involved from the intestinal prehabilitation/transplant Center, UMCG in the patient care. They acknowledge the team under the guidance of Prof Dr S.J.L. Bakker, which provides the TransplantLines infrastructure and standard procedures for the establishment, expansion, and optimization of clinical biobanks for scientific research, and which allowed for the inclusion of part of their patients. The authors also thank the investigators who are involved in TransplantLines: C. Annema, S.P. Berger, H. Blokzijl, F.A.J.A. Bodewes, M.T. de Boer, K. Damman, M.H. de Borst, A. Diepstra, G. Dijkstra, R.M. Douwes, C.S.E. Doorenbos, M.F. Eisenga, M.E. Erasmus, C.T. Gan, A.W. Gomes Neto, E. Hak, B.G. Hepkema, F. Klont, T.J. Knobbe, D. Kremer, H.G.D. Leuvenink, W.S. Lexmond, V.E. de Meijer, G.J. Nieuwenhuis-Moeke, H.G.M. Niesters, L.J. van Pelt, R.A. Pol, R.J. Porte, A.V. Ranchor, J.S.F. Sanders, M.J. Siebelink, R.J.H.J.A. Slart, J.C. Swarte, D.J. Touw, M.C. van den Heuvel, C. van Leer-Buter, M. van Londen, C.A. te Velde-Keyzer, E.A.M. Verschuuren, M.J. Vos, and R.K. Weersma. They acknowledge those involved in the collection: E. de Jong for inclusion, the endoscopy center (UMCG), Prometheus, A. Bangma, and the lab technicians for collection, processing, and storing of samples. Their single-cell RNA sequencing generation methods were based on research performed by Drs W.T.C. Uniken Venema and M.G.P. van der Wijst who is supported by a NWO Vidi grant (VI.Vidi.223.041). M.X.L. Dijkema was involved in the dissociation lab experiments. They thank the Genomics Coordination Center (UMCG and RUG) for their support and for providing access to the Gearshift high-performance computing cluster. They thank J.C. Swarte and Dr R. Gacesa for the generation and initial exploration of the 16S rRNA sequencing ITx data.

## Supplementary Material
