# Supplementary material for: Insights in Cellular and Molecular Signatures of the Small Intestinal Graft Posttransplantation: Successful, Recovered, and Rejected—A Case Series
Source: Transplantation. 2025 Apr 24;109(11):1792–801. doi: 10.1097/TP.0000000000005411 (PMC12539520; doi:10.1097/TP.0000000000005411)
Supplement: Supplementary file 1 [file tpa-109-1792-s001.pdf]

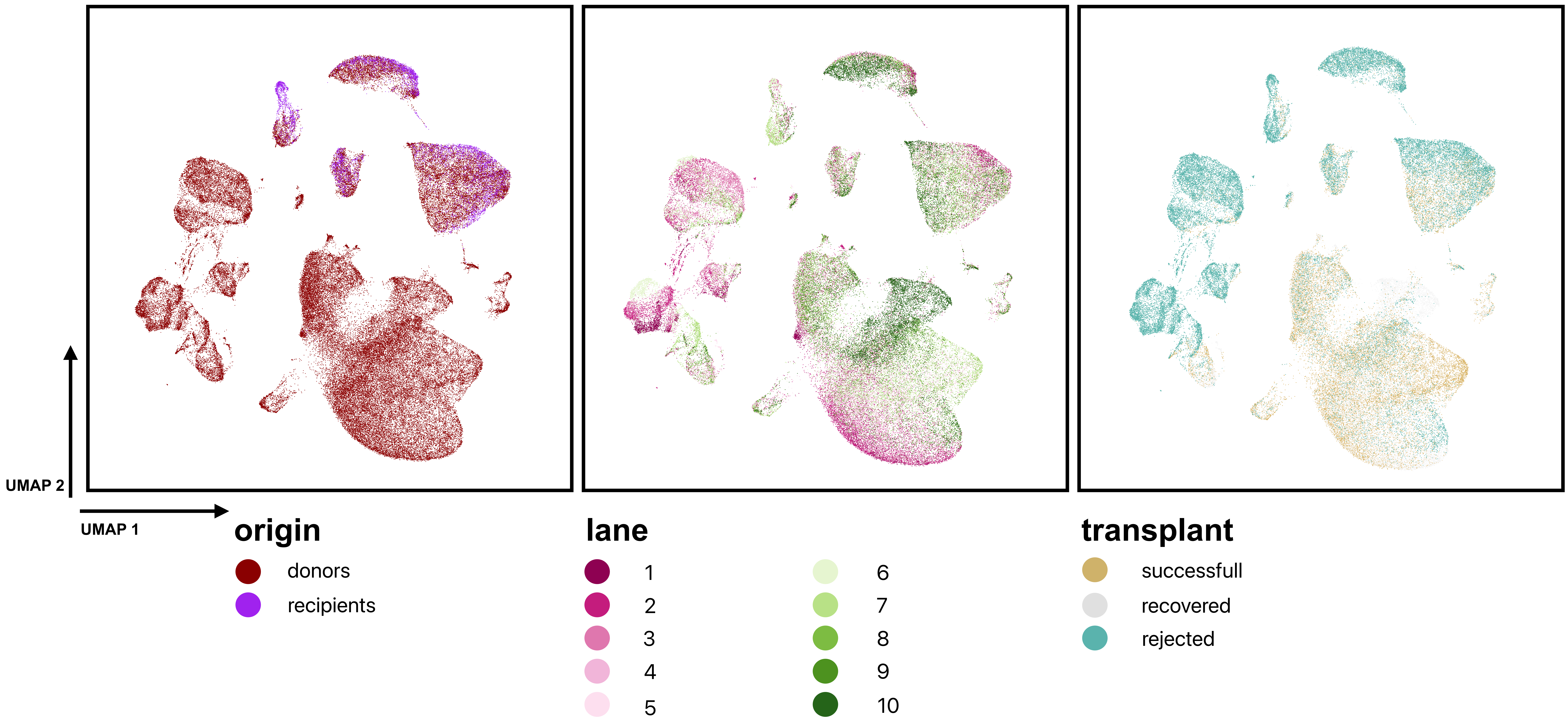

Figure S1. Exploring patient- and sample-specific parameters in the intra-graft cell landscape in the mucosa of transplanted patients

UMAP representations of 90,282 ileal mucosal single cells, where each cell is color coded for the corresponding origin, lane, and transplant. No batch effects were identified.

UMAP, Uniform Manifold Approximation and Projection.

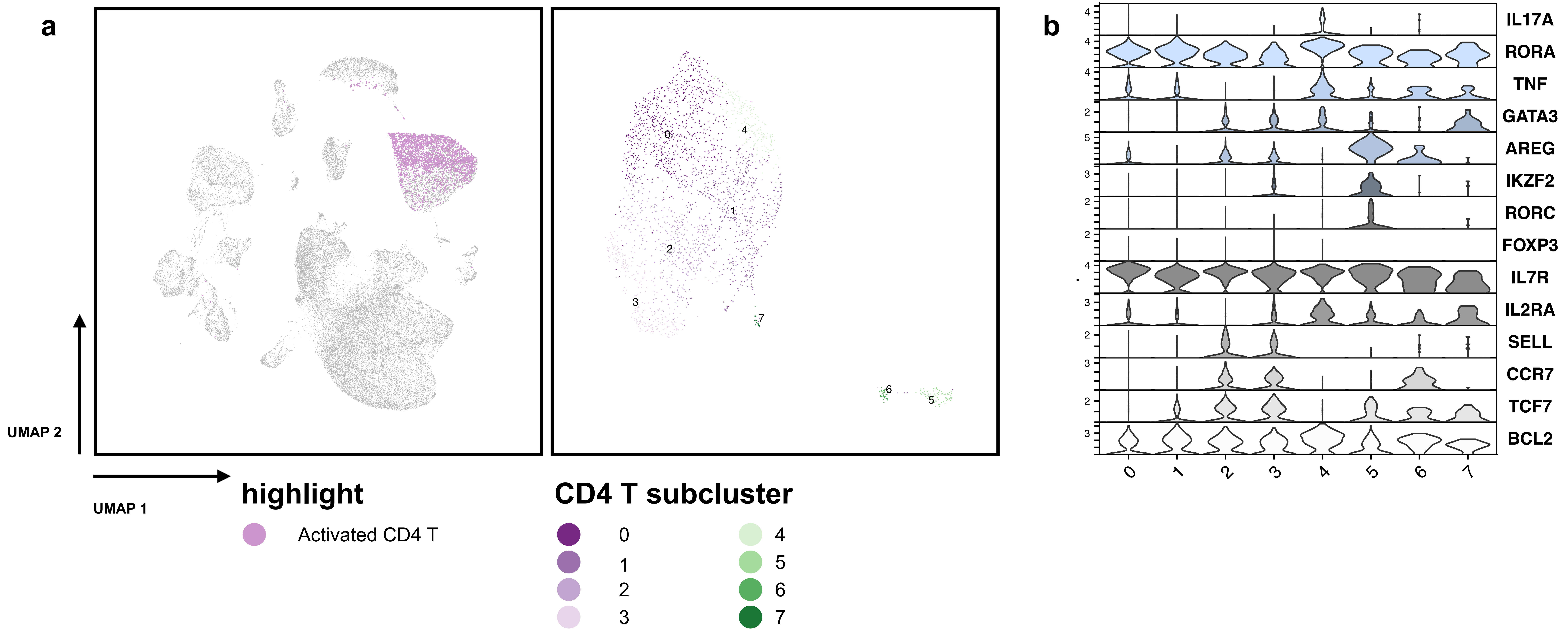

Figure S2. Subclustering of activated CD4+ T cells to identify T regulatory and T helper 17 cells
a. UMAP representations of 90,282 ileal mucosal single cells. Activated CD4+ T cells were subclustered. b. A violin plot was generated with known marker genes *IL17A*, *RORA* and *TNF* expressing cells for Th17 cells (cluster 4) and *AREG*, *IKZF2*, *RORC*, *FOXP3*, *IL7R high* and *IL2RA low* expressing cells for Tregs (cluster 5).
UMAP, Uniform Manifold Approximation and Projection; Th17, T helper 17; Tregs, T regulatory cells.

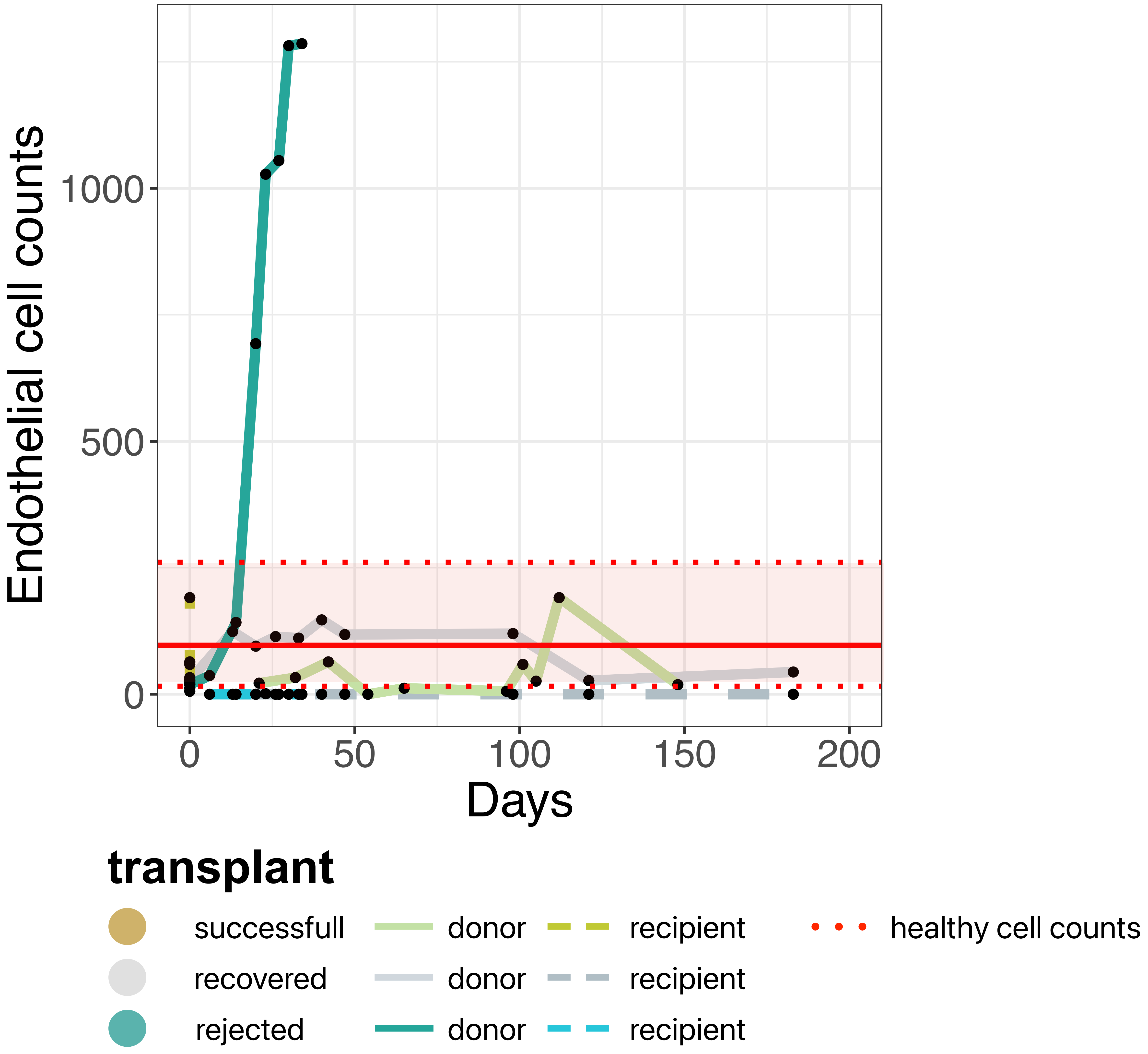

Figure S3. The increase in endothelial cell counts can not be explained by cell composition changes
X-axis presents days and y-axis presents endothelial cell count in the downsampled dataset. Each dot represents one of 28 samples. Colors indicate distinct transplants. Red solid lines indicate the average and red dotted lines indicate the 'normal' range of healthy ileal cell counts 4. Samples split by origin (donor in straight vs recipient in dashed lines).

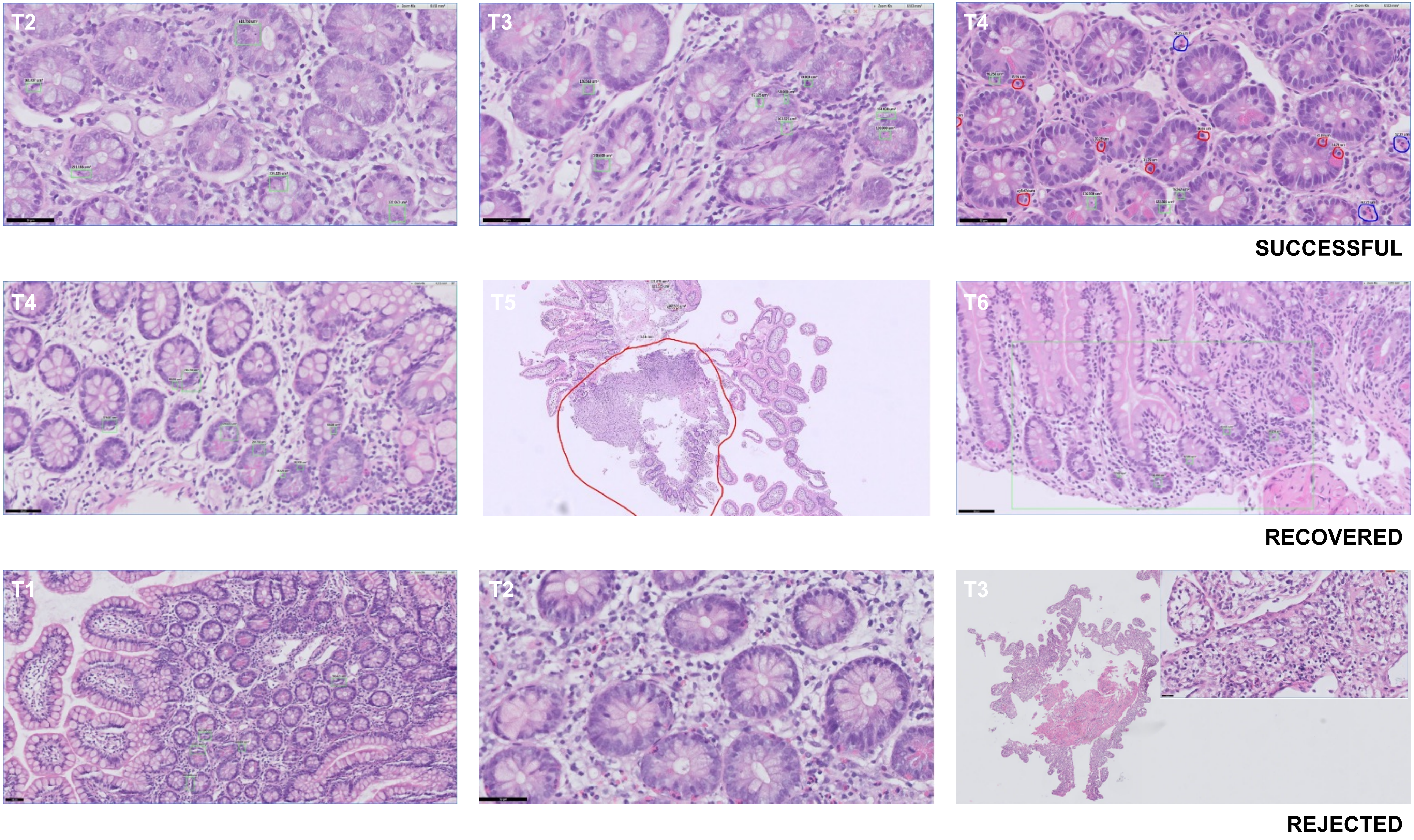

Figure S4. Histological findings of three consecutive and clinically relevant timepoints show different transplantation outcomes

Successful transplant image T2-T4 show dilated vessels circled light blue, the number of apoptotic bodies in green, intra-eptihelial lymphocytes in red and eosinophils in dark blue circle. Recovered transplant image T4 shows some lightly dilated vessels, T5 (ACR grade 2) shows a focal, red-lined region with crypt loss and intraepithelial lymphocytes, T6 shows various apoptotic bodies. Rejected transplant images T1 and T2 show focally dilated lymph vessels, T2 (ACR grade 2) shows an increase in eosinophils, neutrophils and the detachment of the epithelium/lamina propria, T3 shows an overview image and a detailed image of total detachment of the epithelium and denudation of the villi.

Supplemental Digital Content, Table S1

Full Excel overview available on:
github.com/WeersmaLabIBD/DDTX_suppl_materials/tree/main

Transplant	Origin	Time	Day	B cell	Endothelial	Epithelial	Fibroblasts	Glia	Myeloid	T cell
Successful	donor	T0	0	163	54	1280	1	20	9	422
Successful	donor	T1	6	119	96	1048	5	25	28	288
Successful	recipient	T1	6	7	0	0	0	0	17	27
Successful	donor	T2	12	152	65	1278	2	37	10	337
Successful	recipient	T2	12	0	0	0	0	0	8	1
Successful	donor	T3	19	85	148	1299	6	39	5	266
Successful	recipient	T3	19	25	0	0	0	2	28	46
Successful	donor	T4	22	22	21	1751	0	11	5	139
Successful	recipient	T4	22	0	0	0	0	0	0	0
Successful	donor	T5	26	55	105	1476	5	26	10	196
Successful	recipient	T5	26	2	0	0	0	0	20	54
Successful	donor	T6	33	37	32	1682	1	13	5	135
Successful	recipient	T6	33	6	0	0	0	0	3	35
Successful	donor	T7	59	58	101	1708	5	35	10	32
Successful	recipient	T7	59	0	0	0	0	0	0	0
Successful	donor	T8	64	38	42	1739	2	37	13	78
Successful	recipient	T8	64	0	0	0	0	0	0	0
Successful	donor	T9	191	54	112	1397	5	90	3	1
Successful	recipient	T9	191	48	0	0	0	0	10	229
Recovered	donor	T0	0	138	28	1681	0	5	17	80
Recovered	donor	T1	13	162	124	1020	0	40	14	398

Transplant	Origin	Time	Day	B cell	Endothelial	Epithelial	Fibroblasts	Glia	Myeloid	T cell
Recovered	recipient	T1	13	104	0	0	0	0	30	57
Recovered	donor	T2	20	126	95	1123	3	38	13	309
Recovered	recipient	T2	20	156	0	0	0	2	11	73
Recovered	donor	T3	26	74	114	1489	2	53	9	208
Recovered	recipient	T3	26	0	0	0	0	0	0	0
Recovered	donor	T4	33	121	111	1352	1	29	8	297
Recovered	recipient	T4	33	4	0	0	0	0	8	18
Recovered	donor	T5	40	91	147	1470	3	61	8	157
Recovered	recipient	T5	40	1	0	0	0	0	7	4
Recovered	donor	T6	47	77	118	1411	1	28	9	303
Recovered	recipient	T6	47	0	0	0	0	0	2	0
Recovered	donor	T7	98	196	120	1153	0	56	11	254
Recovered	recipient	T7	98	91	0	0	0	2	33	33
Recovered	donor	T8	121	29	27	1735	2	27	4	115
Recovered	recipient	T8	121	4	0	0	0	0	5	1
Recovered	donor	T9	183	51	44	1675	1	22	1	66
Recovered	recipient	T9	183	56	0	0	0	0	19	14
Rejected	donor	T0	0	965	19	566	0	3	53	343
Rejected	donor	T1	6	58	37	1631	4	13	26	180
Rejected	recipient	T1	6	0	0	0	0	0	0	0
Rejected	donor	T2	14	50	142	1253	5	26	55	184

Transplant	Origin	Time	Day	B cell	Endothelial	Epithelial	Fibroblasts	Glia	Myeloid	T cell
Rejected	recipient	T2	14	20	0	0	0	1	50	163
Rejected	donor	T3	20	158	693	13	6	75	225	260
Rejected	recipient	T3	20	262	0	0	0	5	61	191
Rejected	donor	T4	23	11	1028	9	0	69	93	223
Rejected	recipient	T4	23	104	1	0	0	4	183	224
Rejected	donor	T5	27	143	1055	45	12	206	17	179
Rejected	recipient	T5	27	147	0	0	0	10	29	106
Rejected	donor	T6	30	30	1282	4	17	118	13	160
Rejected	recipient	T6	30	150	0	0	0	1	29	145
Rejected	donor	T7	34	13	1286	19	5	94	20	162
Rejected	recipient	T7	34	149	0	0	0	0	26	175

Transplant	Origin	Time point	Day	Activated CD4 T	Activated CD8 T	Adult Glia	rterial capillary	BEST4+ epithelial	cDC1	cDC2
Successful	donor	T0	0	68	352	20	19	13	0	2
Successful	donor	T1	6	102	184	25	8	21	0	3
Successful	recipien	T1	6	9	12	0	0	0	0	1
Successful	donor	T2	12	54	278	37	9	33	2	1
Successful	recipien	T2	12	1	0	0	0	0	0	0
Successful	donor	T3	19	108	153	39	12	50	0	0
Successful	recipien	T3	19	10	24	2	0	0	0	1
Successful	donor	T4	22	31	106	11	1	51	0	0
Successful	recipien	T4	22	0	0	0	0	0	0	0
Successful	donor	T5	26	31	165	26	9	24	0	0
Successful	recipien	T5	26	15	34	0	0	0	0	1
Successful	donor	T6	33	17	118	13	3	31	0	0
Successful	recipien	T6	33	14	20	0	0	0	0	0
Successful	donor	T7	59	12	19	35	7	32	0	0
Successful	recipien	T7	59	0	0	0	0	0	0	0
Successful	donor	T8	64	26	49	37	6	36	0	3
Successful	recipien	T8	64	0	0	0	0	0	0	0
Successful	donor	T9	191	0	1	90	15	10	0	0
Successful	recipien	T9	191	46	180	0	0	0	0	0
Recovered	donor	T0	0	29	50	5	2	10	0	5
Recovered	donor	T1	13	86	299	40	15	32	1	0
Recovered	recipien	T1	13	29	26	0	0	0	0	2
Recovered	donor	T2	20	53	251	38	23	10	1	1
Recovered	recipien	T2	20	32	41	2	0	0	2	2
Recovered	donor	T3	26	93	112	53	23	19	0	0
Recovered	recipien	T3	26	0	0	0	0	0	0	0
Recovered	donor	T4	33	83	213	29	16	13	0	0
Recovered	recipien	T4	33	9	8	0	0	0	0	0
Recovered	donor	T5	40	31	124	61	27	27	0	0
Recovered	recipien	T5	40	1	2	0	0	0	0	0
Recovered	donor	T6	47	38	262	28	29	10	0	0

Recovered	recipien	T6	47	0	0	0	0	0	0	0
Recovered	donor	T7	98	156	95	56	23	23	1	1
Recovered	recipien	T7	98	26	6	2	0	0	0	12
Recovered	donor	T8	121	26	89	27	13	10	0	0
Recovered	recipien	T8	121	1	0	0	0	0	0	1
Recovered	donor	T9	183	11	54	22	4	16	0	0
Recovered	recipien	T9	183	2	12	0	0	0	0	2
Rejected	donor	T0	0	241	99	3	5	4	4	23
Rejected	donor	T1	6	67	108	13	10	27	0	0
Rejected	recipien	T1	6	0	0	0	0	0	0	0
Rejected	donor	T2	14	26	152	26	16	17	0	1
Rejected	recipien	T2	14	36	115	1	0	0	2	0
Rejected	donor	T3	20	153	105	75	133	0	0	0
Rejected	recipien	T3	20	82	77	5	0	0	2	4
Rejected	donor	T4	23	91	130	69	179	0	0	0
Rejected	recipien	T4	23	132	66	4	0	0	1	2
Rejected	donor	T5	27	132	45	206	357	0	0	0
Rejected	recipien	T5	27	77	27	10	0	0	0	3
Rejected	donor	T6	30	54	104	118	263	1	0	0
Rejected	recipien	T6	30	80	62	1	0	0	1	0
Rejected	donor	T7	34	23	138	94	131	0	0	0
Rejected	recipien	T7	34	55	117	0	0	0	0	2

Contractile pericyte (PLN+	CX3CR1+ CD8 Tmem	Cycling B cell	Enterocyte	gdT	Goblet cell	I cells (CCK+)	IgA plasma cell	IgG plasma cell
6	0	2	1065	0	2	2	83	0
3	1	0	514	0	22	2	109	4
0	2	0	0	0	0	0	6	1
1	2	0	824	0	2	6	76	3
0	0	0	0	0	0	0	0	0
3	1	0	906	0	26	4	78	2
0	1	0	0	0	0	0	9	1
0	1	0	1487	0	3	14	18	0
0	0	0	0	0	0	0	0	0
2	0	0	984	0	5	5	46	0
0	2	0	0	0	0	0	2	0
7	0	0	1349	0	7	11	33	0
0	0	0	0	0	0	0	3	1
15	0	0	1328	0	10	10	51	1
0	0	0	0	0	0	0	0	0
3	0	0	1552	0	0	5	28	2
0	0	0	0	0	0	0	0	0
11	0	0	926	0	4	4	53	0
0	0	0	0	0	0	0	36	3
3	1	2	1554	0	11	2	43	0
14	2	1	596	2	11	20	110	7
0	0	1	0	0	0	0	5	0
7	0	0	655	1	9	4	56	2
0	0	0	0	0	0	0	3	0
7	0	0	1199	0	3	3	56	3
0	0	0	0	0	0	0	0	0
2	0	0	1046	0	1	5	108	3
0	1	0	0	0	0	0	1	0
8	0	0	1123	0	5	5	82	1
0	0	0	0	0	0	0	0	0
4	0	0	1172	0	9	6	66	1

0	0	0	0	0	0	0	0	0
4	0	1	857	0	0	2	84	2
0	0	0	0	0	0	0	11	2
2	0	0	1489	0	3	6	29	0
0	0	0	0	0	0	0	3	1
3	0	0	1480	0	1	8	40	2
0	0	1	0	0	0	0	21	1
2	0	17	485	0	3	0	86	9
5	1	0	1355	1	2	2	35	2
0	0	0	0	0	0	0	0	0
13	2	0	689	0	21	9	47	2
0	0	0	0	0	0	0	4	0
42	0	0	3	0	0	0	21	2
0	0	0	0	0	0	0	53	13
68	0	0	1	0	0	0	8	0
0	0	0	0	0	0	0	39	8
86	0	0	0	0	0	0	25	16
0	0	0	0	0	0	0	45	16
150	0	0	1	0	0	0	28	0
0	0	0	0	0	0	0	46	3
170	0	0	2	0	0	0	9	0
0	0	0	0	0	0	0	39	14

ILC3	K cells (GIP+)	LEC1 (ACKR4+)	LEC3 (ADGRG3+)	LYVE1+ Macrophage	Macrophages	Mast cell	Mature arterial EC	Mature venous EC
2	0	0	1	1	3	3	2	8
1	1	0	3	2	19	4	3	4
0	0	0	0	1	7	0	0	0
0	1	0	0	0	4	2	3	9
0	0	0	0	1	3	0	0	0
3	2	2	2	0	2	1	11	20
0	0	0	0	3	14	0	0	0
0	7	1	4	0	3	1	1	4
0	0	0	0	0	0	0	0	0
0	3	0	6	0	3	7	4	13
0	0	0	0	3	10	0	0	0
0	2	1	2	0	1	3	0	2
0	0	0	0	0	3	0	0	0
0	12	3	14	0	3	7	5	12
0	0	0	0	0	0	0	0	0
0	2	0	4	1	9	0	0	4
0	0	0	0	0	0	0	0	0
0	1	0	7	0	1	2	6	15
0	0	0	0	1	7	0	0	0
0	2	0	3	1	7	1	1	2
0	2	1	0	0	9	2	4	23
0	0	0	0	5	20	0	0	0
1	3	4	0	2	4	5	4	3
0	0	0	0	3	1	0	0	0
2	1	1	2	3	4	1	18	8
0	0	0	0	0	0	0	0	0
1	1	1	3	0	4	2	2	9
0	0	0	0	1	6	0	0	0
0	2	1	3	3	1	2	8	8
0	0	0	0	1	5	0	0	0
0	1	1	2	1	3	5	7	12

0	0	0	0	1	1	0	0	0
2	2	2	2	0	4	4	4	13
0	0	0	0	3	12	0	0	0
0	2	0	0	0	0	3	3	0
0	0	0	0	2	2	0	0	0
0	0	1	0	0	1	0	8	2
0	0	0	0	5	8	1	0	0
3	1	0	2	8	12	1	0	5
0	2	0	0	3	20	3	1	7
0	0	0	0	0	0	0	0	0
0	0	0	1	14	38	2	0	18
1	0	0	0	10	21	0	0	0
1	0	0	0	141	83	1	77	124
0	0	0	0	20	19	0	0	0
2	0	0	0	52	40	1	97	261
0	0	0	0	14	17	5	0	0
2	0	0	0	7	8	2	143	342
2	0	0	0	4	9	1	0	0
2	0	0	0	4	7	0	97	301
1	0	0	0	4	2	0	0	0
0	0	0	0	4	16	0	78	116
1	0	0	0	4	7	0	0	0

Memory B	Monocytes	nyofibroblast (RSPO2+)	Naive B	Paneth	Stem cells	Stromal 1 (ADAMDEC1+)	Stromal 1 (CCL11+)	Stromal 2 (NPY+)
78	0	1	0	0	1	5	12	0
6	0	5	0	18	24	8	59	0
0	8	0	0	0	0	0	0	0
70	1	2	3	46	5	13	29	0
0	4	0	0	0	0	0	0	0
5	2	6	0	17	19	17	74	0
15	10	0	0	0	0	0	0	0
4	1	0	0	12	3	1	9	0
0	0	0	0	0	0	0	0	0
8	0	5	1	33	8	19	50	0
0	6	0	0	0	0	0	0	0
4	1	1	0	21	14	3	13	0
2	0	0	0	0	0	0	0	0
6	0	5	0	17	20	12	20	0
0	0	0	0	0	0	0	0	0
8	0	2	0	1	9	4	21	0
0	0	0	0	0	0	0	0	0
1	0	5	0	67	6	8	44	0
9	2	0	0	0	0	0	0	0
93	3	0	0	3	3	4	4	0
43	2	0	1	32	37	21	37	0
97	3	0	1	0	0	0	0	0
67	0	3	1	64	22	17	28	0
150	3	0	3	0	0	0	0	0
14	1	2	1	17	5	25	20	0
0	0	0	0	0	0	0	0	0
10	2	1	0	17	9	37	37	0
3	1	0	0	0	0	0	0	0
8	2	3	0	13	24	50	32	0
1	1	0	0	0	0	0	0	0
9	0	1	1	12	15	20	38	0

0	0	0	0	0	0	0	0	0
108	1	0	1	17	7	46	23	0
78	6	0	0	0	0	0	0	0
0	1	2	0	20	6	2	6	0
0	0	0	0	0	0	0	0	0
8	0	1	1	26	6	6	20	0
33	3	0	0	0	0	0	0	0
853	5	0	0	1	1	1	3	0
20	0	4	1	8	1	4	10	0
0	0	0	0	0	0	0	0	0
1	0	5	0	15	55	17	67	0
16	17	0	0	0	0	0	0	0
135	0	6	0	3	0	18	112	0
195	16	0	1	0	0	0	0	0
3	0	0	0	2	0	29	102	0
57	144	0	0	0	0	0	0	0
102	0	12	0	4	0	33	9	0
86	12	0	0	0	0	0	0	0
2	2	17	0	1	0	70	67	1
101	22	0	0	0	0	0	0	0
4	0	5	0	4	0	262	257	1
95	13	0	1	0	0	0	0	0

Stromal 3 (C7+)	TA	Transitional Stromal 3 (C3+	TRGV2 gdT	TRGV5/7 gdT	Tuft
0	179	1	0	0	18
0	444	8	0	0	2
0	0	0	4	0	0
0	352	1	3	0	9
0	0	0	0	0	0
5	265	2	1	0	10
0	0	0	11	0	0
0	171	0	1	0	3
0	0	0	0	0	0
1	411	1	0	0	3
0	0	0	3	0	0
1	242	0	0	0	5
0	0	0	1	0	0
2	270	11	1	0	9
0	0	0	0	0	0
0	130	0	3	0	4
0	0	0	0	0	0
3	374	3	0	0	5
0	0	0	2	1	0
0	87	9	0	0	9
1	285	8	9	0	5
0	0	0	2	0	0
2	345	7	3	0	11
0	0	0	0	0	0
1	234	9	1	0	8
0	0	0	0	0	0
2	258	2	0	0	2
0	0	0	0	0	0
5	268	5	2	0	3
0	0	0	1	0	0
3	185	2	1	2	1

0	0	0	0	0	0
2	237	1	1	0	8
0	0	0	1	0	0
1	192	0	0	0	7
0	0	0	0	0	0
0	133	0	1	0	5
0	0	0	0	0	0
0	62	1	0	0	9
0	225	0	3	0	9
0	0	0	0	0	0
0	420	10	3	1	27
0	0	0	11	0	0
4	7	183	1	0	0
0	0	0	32	0	0
6	6	286	0	0	0
0	0	1	26	0	0
1	41	84	0	0	0
0	0	0	0	0	0
2	1	331	0	0	0
0	0	0	2	0	0
3	13	268	1	0	0
0	0	0	2	0	0

Table S2: Cumulative overview of Donor Specific Antibodies (DSA) at post-operative days (POD) of the rejected transplant

POD	Type	Value
-1 (pre-ITx)	B44	1878
2	All	negative
13	B62	1981
	B44	17066
	C10	2483
22	B62	5045
	B44	6393
	C10	4044
29	B62	2294
	B44	4564
	C10	1121
30	B62	2965
	B44	5032
	C10	1113

Only values greater than 500 and positive in the Lifecodes Single Antigen are shown in this table.

Supplemental Digital Content, Table S3

Full Excel overview available on:
github.com/WeersmaLabIBD/DDTX_suppl_materials/tree/main

Supplemental Digital Content, Table S3

Transplan	Day	p_val	avg_log2FC	pct.1	pct.2	p_val_adj	Gene
Successfu	0	3.19230681945387e-81	2.20401829457814	0.672	0.044	1.07063586110844e-76	REG1B
Successfu	0	6.87297212738452e-40	1.430625802682	0.962	0.626	2.30505739208222e-35	PHGR1
Successfu	0	2.44950348904837e-39	1.46998840397444	0.969	0.855	8.21514480157041e-35	IGKC
Successfu	0	9.80206632880385e-34	1.48440596105017	0.954	0.765	3.28741700535423e-29	FABP6
Successfu	0	9.58816733414027e-30	1.58915074237728	0.794	0.406	3.21567956052396e-25	APOA4
Successfu	0	1.11464766131806e-26	1.36503640875532	0.565	0.176	3.73830532652851e-22	PDE3A
Successfu	0	5.77524705233742e-25	0.63309249678667	1	1	1.93690235641293e-20	MT-CO2
Successfu	0	7.14967335322528e-23	1.78524943175428	0.855	0.65	2.3978574492047e-18	TXNIP
Successfu	0	4.31819456507042e-22	1.05008795797256	0.519	0.16	1.44823609323332e-17	GUCA2A
Successfu	0	1.46859334666758e-20	1.24296568728188	0.832	0.563	4.92536836605371e-16	FKBP5
Successfu	0	5.0722610244754e-19	0.51693817203631	1	1	1.70113490238856e-14	MT-CO3
Successfu	0	9.51896838658086e-19	1.05800666975977	0.702	0.36	3.19247161749149e-14	JCHAIN
Successfu	0	2.68356015413929e-18	1.29984364712057	0.832	0.529	9.00012404495235e-14	HSPH1
Successfu	0	4.92468059791466e-18	0.94625083397283	0.878	0.573	1.65163937892862e-13	IGHA1
Successfu	0	8.73298998591986e-18	1.10293134305495	0.534	0.223	2.9288701814778e-13	CYP3A5
Successfu	0	9.50665757981946e-18	0.88665086073783	0.84	0.585	3.18834281911985e-13	REG1A
Successfu	0	1.04347702634384e-16	1.01661632760494	0.763	0.486	3.49961325095198e-12	ALDOB
Successfu	0	2.35818223076302e-16	1.07300173667707	0.802	0.602	7.90887156553303e-12	SMAP2
Successfu	0	3.23045383819822e-16	1.05936596156263	0.725	0.456	1.08342960825492e-11	FABP1
Successfu	0	3.53799134066003e-16	0.94523219248028	0.969	0.917	1.18657153583056e-11	HSP90AA1
Successfu	0	8.04698876366237e-16	1.48731931303399	0.725	0.439	2.69879909155709e-11	CRYBG1
Successfu	0	1.26588862973294e-15	1.12262462041001	0.466	0.17	4.24553728639834e-11	ZBTB16
Successfu	0	5.70510416139673e-15	1.00123085982073	0.656	0.362	1.91337783364923e-10	APOC3
Successfu	0	7.72258946579503e-15	1.37153803932274	0.687	0.436	2.59000205503834e-10	ATP1B3
Successfu	0	1.08954158736976e-14	1.04935154573575	0.725	0.406	3.6541045757207e-10	HSPA1A
Successfu	0	1.54950918139419e-14	0.956111816237847	0.58	0.284	5.19674389255983e-10	KRT19
Successfu	0	2.40415304827927e-14	1.01706299195292	0.443	0.176	8.06304849331902e-10	SLC5A1
Successfu	0	1.70036539834367e-13	1.06420142360089	0.504	0.24	5.70268547296499e-09	MYO1E
Successfu	0	2.03711808700014e-13	0.85586285315493	0.397	0.149	6.83208664018108e-09	MUC17

Successfu	0	5.17435787408592e-13	0.43148348605301	0.985	0.986	1.73537614381094e-08	MT-ND3
Successfu	0	1.14442657869161e-12	0.84068541827925	0.344	0.116	3.83817785961591e-08	AMN
Successfu	0	1.15845498214861e-12	0.91153543151420	0.664	0.4	3.88522631913e-08	IGLC2
Successfu	0	1.55715749118967e-12	0.46906992788896	1	0.997	5.22239479395192e-08	MT-ATP6
Successfu	0	2.07895891969284e-12	0.89099996018308	0.595	0.347	6.97241242486586e-08	KRT8
Successfu	0	2.15203688033991e-12	0.92503545060459	0.588	0.306	7.21750128928399e-08	IGLC3
Successfu	0	3.08624652836144e-12	0.85039298959484	0.695	0.475	1.03506536068186e-07	CD74
Successfu	0	5.33763702553141e-12	0.44497061379928	1	0.995	1.79013670562272e-07	MT-CYB
Successfu	0	6.45966232998404e-12	1.17235166950485	0.382	0.146	2.16644155223005e-07	APBA2
Successfu	0	7.99291022824596e-12	0.76845260626976	0.198	0.039	2.68066223234913e-07	BFSP2
Successfu	0	1.26308729755331e-11	0.57228980769332	0.244	0.06	4.2361421785343e-07	PDE9A
Successfu	0	4.08682568730641e-11	0.60215779551548	0.137	0.018	1.37063959900882e-06	MS4A1
Successfu	0	7.13244817106874e-11	1.13798067625792	0.718	0.466	2.39208046761303e-06	ZNF331
Successfu	0	9.99664595284482e-11	0.80965958963617	0.282	0.089	3.3526751196651e-06	CYP3A4
Successfu	0	1.52696604820709e-10	0.68215659407243	0.855	0.638	5.12113873247695e-06	DNAJB1
Successfu	0	1.68846549375636e-10	0.89857291606332	0.672	0.443	5.66277557296008e-06	MTRNR2L8
Successfu	0	2.18207722141831e-10	0.73017667819539	0.382	0.157	7.31825058519273e-06	ADIRF
Successfu	0	2.22200232774809e-10	0.82692402645093	0.504	0.271	7.45215140680156e-06	FABP2
Successfu	0	3.56307831059874e-10	0.90015154055830	0.45	0.219	1.1949852038086e-05	SLC15A1
Successfu	0	5.64925811733004e-10	0.77184094438767	0.542	0.306	1.89464818739015e-05	SELENOP
Successfu	0	6.10209837171748e-10	0.66089123515184	0.176	0.038	2.04652175190661e-05	IGHG1
Successfu	0	1.13613344834833e-09	1.26482750619961	0.573	0.357	3.81036435907063e-05	JMY
Successfu	0	1.58305614670157e-09	1.02239760417502	0.183	0.042	5.30925370480772e-05	GFPT2
Successfu	0	1.78130197435562e-09	0.68195645601593	0.206	0.056	5.97413056159389e-05	TM4SF20
Successfu	0	2.90387361398426e-09	0.72287418467214	0.809	0.688	9.7390113265804e-05	MTRNR2L12
Successfu	0	3.27796036562982e-09	0.31912946222328	1	0.997	0.000109936234742493	MT-ND4
Successfu	0	6.35205169935389e-09	0.73881042652494	0.351	0.151	0.000213035109892931	HLA-DRB1
Successfu	0	7.10230096296887e-09	0.81787797065790	0.412	0.201	0.00023819696969605	HLA-DRA
Successfu	0	9.25653965471867e-09	0.81989713056863	0.313	0.127	0.000310445826939955	SMIM24
Successfu	0	1.39847392657492e-08	0.76784740126915	0.427	0.225	0.000469020185494695	RBP2
Successfu	0	2.38129678810981e-08	0.51197409856786	0.947	0.857	0.000798639316796266	FTL

Successfu	0	4.31041069393885e-08	0.75879153380063[illegible]	0.55	0.357	0.00144562553853321	ATP5ME
Successfu	0	4.50448562809888e-08	0.99896476389622[illegible]	0.618	0.449	0.0015107143899518	ELL2
Successfu	0	5.30261181251545e-08	0.96107714064711[illegible]	0.718	0.564	0.00177838994968143	CXCR4
Successfu	0	5.64145404808037e-08	1.17413493126856	0.603	0.449	0.00189203085864519	KIF13B
Successfu	0	5.76959747120147e-08	0.59470520397340[illegible]	0.267	0.1	0.00193500759989155	FRMD3
Successfu	0	8.41331453749232e-08	0.68702264529408[illegible]	0.328	0.145	0.00282165742958417	CST3
Successfu	0	8.62537529999609e-08	1.27361453699179	0.687	0.54	0.00289277836811269	CEMIP2
Successfu	0	1.00623510058568e-07	0.61205476186527[illegible]	0.351	0.163	0.00337471128034425	CLDN4
Successfu	0	1.02570775256371e-07	0.76405073300504[illegible]	0.878	0.766	0.00344001866054817	CNOT6L
Successfu	0	1.20226274506884e-07	0.8139905344393	0.412	0.228	0.00403214879441188	PIK3IP1
Successfu	0	1.37668796416136e-07	0.66122795776222[illegible]	0.298	0.125	0.00461713609420436	PTPRD
Successfu	0	1.40384659558596e-07	0.42474419068876[illegible]	0.168	0.045	0.00470822071227618	SLC6A19
Successfu	0	1.4467512991773e-07	0.45121682430869[illegible]	0.107	0.018	0.00485211450718084	IGHG4
Successfu	0	1.4578764114733e-07	0.71689374280705[illegible]	0.305	0.131	0.00488942590879915	EPB41L3
Successfu	0	1.53431427930754e-07	0.35770005569665[illegible]	0.107	0.018	0.00514578322994164	C11orf86
Successfu	0	1.83450032460447e-07	0.67503903463274[illegible]	0.305	0.134	0.00615254718865846	DPEP1
Successfu	0	1.84725664666543e-07	0.55574134363455[illegible]	0.672	0.439	0.00619532934158651	HSPA1B
Successfu	0	1.88894954208639e-07	0.70210362628001[illegible]	0.611	0.443	0.00633515897424934	PCSK5
Successfu	0	1.90732264174916e-07	0.75829892905214[illegible]	0.298	0.133	0.00639677867589834	TMC5
Successfu	0	3.39338527574704e-07	0.57293632471832	0.206	0.071	0.0113807355378004	HLA-DRB5
Successfu	0	3.51479498268666e-07	0.76912370756443[illegible]	0.382	0.198	0.0117879194129345	CYB5A
Successfu	0	3.5236736969042e-07	0.57715010318113[illegible]	0.84	0.783	0.0118176968446773	UBC
Successfu	0	3.74366157632322e-07	0.71452608861582[illegible]	0.275	0.118	0.0125554921946728	NR1H4
Successfu	0	4.25224672286152e-07	0.29772229531080[illegible]	0.107	0.02	0.014261185059133	CIDEC
Successfu	0	4.75923558133511e-07	0.83216893689751[illegible]	0.359	0.181	0.0159615242926817	MTRNR2L1
Successfu	0	6.27909356428878e-07	0.57503114891627[illegible]	0.252	0.101	0.0210588239959117	CDHR2
Successfu	0	6.42477165058799e-07	0.61388340256733[illegible]	0.366	0.184	0.021547399161742	FGD4
Successfu	0	7.24420565834014e-07	0.73454274647732[illegible]	0.58	0.403	0.0242956169369412	ANPEP
Successfu	0	7.24428842238931e-07	0.63082834819110[illegible]	0.221	0.081	0.0242958945110093	HHLA2
Successfu	0	9.86264167541746e-07	0.50802063693249[illegible]	0.947	0.894	0.0330773276510151	FTH1
Successfu	0	1.03094882940798e-06	0.59923205241147[illegible]	0.298	0.136	0.0345759618406849	NR5A2

Successfu	0	1.21160946619569e-06	0.71772538797683!	0.405	0.226	0.040634958277271	TSPAN8
Successfu	0	1.25551153140629e-06	0.49309363746580€	0.244	0.098	0.0421073457403042	COBL
Successfu	0	1.46132052352144e-06	0.71635507081684!	0.931	0.896	0.049009767717862	BTG1
Successfu	6	7.22105643526807e-26	1.27160646317227	0.946	0.739	2.42179790726021e-21	RPL19
Successfu	6	1.39457276097582e-22	0.98441618395304!	0.982	0.836	4.67711812576071e-18	RPL13
Successfu	6	4.7797019140299e-22	1.04645343237775	0.964	0.878	1.60301642792735e-17	RPL10
Successfu	6	6.39299760876023e-22	1.39350207818739	0.892	0.581	2.14408353802601e-17	ZFP36
Successfu	6	3.98987401575121e-21	0.91677217465454	0.991	0.971	1.33812394740264e-16	EEF1A1
Successfu	6	5.04685823874504e-21	0.98893710113089:	0.982	0.811	1.69261531611031e-16	RPS3
Successfu	6	2.14045855566511e-20	1.00908441439329	0.955	0.795	7.17866990398964e-16	RPS27A
Successfu	6	2.73598447917956e-19	0.99860650322458	0.946	0.748	9.17594474627242e-15	RPS13
Successfu	6	3.27841289773932e-19	0.89890047850468	0.955	0.713	1.09951411764381e-14	RPL23A
Successfu	6	5.03925383057042e-19	0.89741976388812	0.982	0.827	1.69006494969671e-14	RPS4X
Successfu	6	5.89105443782756e-19	0.96326821919132	0.928	0.681	1.97574183735861e-14	RPS5
Successfu	6	6.05049243661148e-19	0.73482397611908!	1	0.987	2.02921415339076e-14	B2M
Successfu	6	8.59225252346845e-19	0.90133784601070€	0.946	0.796	2.88166965132085e-14	RPL5
Successfu	6	3.58381916260082e-18	0.86847697635604€	0.955	0.836	1.20194127075306e-13	RPS3A
Successfu	6	4.09459365168346e-18	0.89906486907182	0.964	0.817	1.3732448189016e-13	RPL3
Successfu	6	4.91986412115615e-18	0.84776881651373	0.964	0.833	1.65002402895335e-13	RPL30
Successfu	6	5.70101884821231e-18	0.85299787540053	0.991	0.859	1.91200770131344e-13	RPL13A
Successfu	6	1.36370801910663e-17	0.87612124296509	0.982	0.772	4.57360395447981e-13	RPL18A
Successfu	6	2.46371032511232e-17	0.81540794697450!	0.937	0.783	8.2627916883617e-13	RPS16
Successfu	6	2.68579778179551e-17	0.85756337104262	0.955	0.769	9.00762860058578e-13	RPL21
Successfu	6	2.79307738763038e-17	1.01436864073034	0.802	0.457	9.36742294263477e-13	RPS10
Successfu	6	5.17068885991067e-17	1.27045920385608	0.928	0.719	1.73414562983684e-12	NFKBIA
Successfu	6	1.12624737380421e-16	0.85043150631434!	0.955	0.754	3.77720844226456e-12	RPL7A
Successfu	6	4.25131909535136e-16	0.80359342242877	0.946	0.777	1.42580739819894e-11	RPS25
Successfu	6	5.05704990654784e-16	0.75409892512113	0.973	0.808	1.69603339765802e-11	RPL9
Successfu	6	5.42606601972426e-16	0.83255246124309(	0.955	0.775	1.81979402169512e-11	RPL8
Successfu	6	6.98924702342481e-16	0.85443947335207	0.955	0.685	2.34405366671621e-11	RACK1
Successfu	6	7.13381773997633e-16	0.77713179691747	0.964	0.763	2.39253979363326e-11	RPS7

Successfu	6	8.84225840241044e-16	0.75563306603162€	0.973	0.788	2.96551662300041e-11	RPL29
Successfu	6	2.10201651938458e-15	0.90146899057840€	0.946	0.726	7.049743002712e-11	RPLP0
Successfu	6	2.13629046222274e-15	0.77226525007510€	0.964	0.851	7.16469095220262e-11	RPS18
Successfu	6	2.72769055185356e-15	0.82384336168914∶	0.946	0.808	9.14812857280647e-11	RPS23
Successfu	6	2.77379439850293e-15	0.740311153706173	0.928	0.673	9.30275165369914e-11	RPL7
Successfu	6	2.93499569900398e-15	0.78381055594382	0.946	0.714	9.84338857531954e-11	RPL26
Successfu	6	3.14501412643986e-15	0.73615520401562€	0.964	0.848	1.0547748377254e-10	RPS6
Successfu	6	3.55103163387905e-15	0.75010793131519€	0.955	0.83	1.19094498937036e-10	RPL32
Successfu	6	3.90361605457866e-15	0.76005561801476€	0.973	0.889	1.30919475238459e-10	RPS2
Successfu	6	4.15244672898163e-15	0.78582245795284€	0.964	0.874	1.39264758396586e-10	RPS12
Successfu	6	5.96765557395097e-15	0.74320481055767↑	0.928	0.731	2.00143232639167e-10	NACA
Successfu	6	8.09574298992268e-15	0.80510856820577€	0.937	0.758	2.71515028396027e-10	RPS9
Successfu	6	1.02521992941158e-14	0.77386743131133€	0.964	0.854	3.43838259926054e-10	RPL11
Successfu	6	1.34730166427146e-14	0.72622204803696∶	0.982	0.77	4.51858032163361e-10	RPS20
Successfu	6	1.38648238578024e-14	0.76920787394000∶	0.964	0.861	4.64998462542978e-10	RPS19
Successfu	6	1.46807856157456e-14	0.76478031061944↑	0.955	0.775	4.92364187980877e-10	RPL6
Successfu	6	2.5529097710242e-14	0.83453069785744€	0.883	0.657	8.56194879006096e-10	EEF1B2
Successfu	6	2.67719346898979e-14	0.67137416639146∶	0.973	0.887	8.97877145629794e-10	RPL41
Successfu	6	2.8097655908094e-14	0.70922604778311∶	0.964	0.785	9.42339183845657e-10	RPL14
Successfu	6	2.81134230167448e-14	1.02491805562516	0.928	0.732	9.42867981135588e-10	PPP1R15A
Successfu	6	4.84597861199471e-14	0.73029854892829	0.937	0.757	1.62524430689079e-09	RPL24
Successfu	6	6.30977601391067e-14	0.72427487380675	0.973	0.741	2.11617267954536e-09	RPL35A
Successfu	6	6.46491779745398e-14	0.79694005752756	0.811	0.501	2.16820413091012e-09	OAZ1
Successfu	6	6.71051532746969e-14	0.77325068817377∶	0.928	0.668	2.25057263052679e-09	RPL10A
Successfu	6	8.45274349402214e-14	0.72789527350714∶	0.892	0.615	2.83488111302515e-09	MYL12A
Successfu	6	8.61179498851175e-14	0.66615843734979€	0.982	0.89	2.88822380324707e-09	PTMA
Successfu	6	1.23120828406351e-13	0.72447482112236∶	0.973	0.924	4.12922634309219e-09	RPLP1
Successfu	6	2.66056145442392e-13	0.84091444128860€	0.928	0.709	8.92299100584693e-09	RPSA
Successfu	6	3.46278386530558e-13	0.67876694609885€	0.982	0.95	1.16134845274618e-08	TPT1
Successfu	6	4.55723307332564e-13	0.71200227240229↑	0.946	0.817	1.52840482813195e-08	RPL18
Successfu	6	5.02078585500584e-13	0.84079750681362⁊	0.91	0.662	1.68387116005186e-08	PNRC1

Successfu	6	5.09273976005551e-13	0.67060368699439	0.955	0.821	1.70800306072742e-08	RPS15A
Successfu	6	8.30758821441303e-13	0.65326056347816	0.973	0.827	2.78619893534984e-08	RPS14
Successfu	6	9.15036689392558e-13	0.70119919218714	0.667	0.328	3.06885004888476e-08	NOP53
Successfu	6	1.61663795931865e-12	0.71778579492576	0.991	0.928	5.42188038796289e-08	JUND
Successfu	6	1.71051043149822e-12	0.65669692383697	0.973	0.785	5.73670988515874e-08	RPS11
Successfu	6	1.95669169919633e-12	0.72995630314751	0.883	0.673	6.56235262076466e-08	RPS26
Successfu	6	2.51927960473676e-12	1.08379579411287	0.982	0.87	8.44915993836615e-08	JUN
Successfu	6	3.48035361022138e-12	0.99671452796859	0.91	0.669	1.16724099379605e-07	BTG2
Successfu	6	5.52548725596684e-12	0.70206692640121	0.901	0.637	1.85313791590616e-07	RPL27
Successfu	6	6.42062009410486e-12	0.77966543817707	0.838	0.536	2.15334756716089e-07	CD48
Successfu	6	7.83220271155487e-12	0.67373365504323	0.928	0.755	2.62676414540127e-07	HLA-C
Successfu	6	8.90270661599497e-12	0.61461424473186	0.964	0.808	2.98578974487239e-07	RPLP2
Successfu	6	1.30679391135449e-11	0.61876012036663	0.937	0.805	4.3827254199007e-07	RPS15
Successfu	6	2.55079134697765e-11	0.58135370780922	0.955	0.824	8.55484401949365e-07	RPL12
Successfu	6	3.43534983077883e-11	0.68985460591531	0.847	0.587	1.1521476262466e-06	RPL31
Successfu	6	4.18488105895824e-11	0.60054226623473	0.883	0.656	1.40352540955342e-06	GAPDH
Successfu	6	6.36637520754472e-11	0.57213071547963	0.982	0.892	2.13515491710635e-06	EIF1
Successfu	6	6.51474932365597e-11	0.59794716093176	0.955	0.862	2.18491662816774e-06	RPL28
Successfu	6	7.14385708486022e-11	0.61042053259207	0.874	0.592	2.39590678912042e-06	RPL36AL
Successfu	6	7.38211764419057e-11	0.61274809028398	0.964	0.837	2.47581461550863e-06	RPS8
Successfu	6	9.93953158170684e-11	0.60493270971813	0.928	0.726	3.33352010187284e-06	FAU
Successfu	6	1.03368266230825e-10	0.59513130479428	0.847	0.602	3.46676491284942e-06	EEF1D
Successfu	6	1.04244246829859e-10	0.63066774900221	0.937	0.769	3.4961435501798e-06	RPL15
Successfu	6	1.06203475509725e-10	0.60870934702913	0.523	0.237	3.56185216164515e-06	UBXN1
Successfu	6	1.15007567175831e-10	0.61558305827994	0.928	0.802	3.85712378794302e-06	HLA-A
Successfu	6	1.22533156217048e-10	0.66807328678601	0.847	0.555	4.10951699320736e-06	HNRNPA1
Successfu	6	1.79004898386044e-10	0.85380290988368	0.739	0.47	6.00346628207113e-06	MYADM
Successfu	6	1.96135174857661e-10	0.67537926590862	0.829	0.6	6.57798149437623e-06	RPL4
Successfu	6	2.0581638373196e-10	0.64689535895458	0.523	0.246	6.90266987760249e-06	HNRNPA0
Successfu	6	2.27254428751597e-10	0.59052442310472	0.946	0.772	7.62165903147105e-06	RPL27A
Successfu	6	2.42369333633708e-10	0.61970814024520	0.45	0.192	8.12858271140728e-06	DRAP1

Successfu	6	3.07154247879505e-10	0.64426995620391	0.883	0.575	1.03013391653829e-05	YBX1
Successfu	6	5.71357964843702e-10	0.66897939895530	0.73	0.448	1.91622034249281e-05	TAGLN2
Successfu	6	5.84948292291296e-10	0.51472397772095	0.468	0.201	1.96179958268655e-05	PAXX
Successfu	6	8.61689696845572e-10	0.57210805213709	0.901	0.64	2.88993490528068e-05	NPM1
Successfu	6	1.03027255613385e-09	0.56766590217266	0.964	0.89	3.45532809876169e-05	HLA-B
Successfu	6	1.05804732760816e-09	0.85949919338418	0.946	0.832	3.54847912733224e-05	DUSP1
Successfu	6	1.22282907655598e-09	0.60753983762153	0.829	0.612	4.10112415695345e-05	BTF3
Successfu	6	1.39146192745385e-09	0.50795533422733	0.631	0.319	4.66668501229473e-05	TRMT112
Successfu	6	1.44336683821398e-09	0.61159142027792	0.622	0.335	4.84076370200205e-05	CLIC1
Successfu	6	1.50757523091763e-09	0.78868029577028	0.928	0.761	5.05610580945156e-05	JUNB
Successfu	6	2.17444990382763e-09	0.51704534641781	0.883	0.622	7.29267008745709e-05	PFDN5
Successfu	6	2.40307282573886e-09	0.50927212028250	0.964	0.878	8.05942564296298e-05	RPS27
Successfu	6	3.090354505548e-09	0.56911768937847	0.937	0.681	0.000103644309407069	RPL22
Successfu	6	3.25249228327981e-09	0.52784346746062	0.928	0.716	0.000109082086196638	UBA52
Successfu	6	4.31602120572097e-09	0.58246475956219	0.964	0.857	0.00014475071919747	FTL
Successfu	6	4.60226558991753e-09	0.60130174129946	0.874	0.672	0.000154350783354654	HLA-E
Successfu	6	7.66417834282112e-09	0.83932359978490	0.766	0.564	0.000257041213261535	IRF1
Successfu	6	7.81810854038297e-09	0.46290861862763	0.27	0.088	0.000262203724227364	EPB41L4A-AS1
Successfu	6	8.6638359909918e-09	0.50474585343221	0.486	0.233	0.000290567731465883	SRSF9
Successfu	6	1.20956465415107e-08	0.48318758902108	0.414	0.171	0.000405663793709184	ARPC1B
Successfu	6	1.37189444429554e-08	0.63193359189394	0.604	0.335	0.000460105958727839	IER5
Successfu	6	1.53790248888339e-08	0.58019510185932	0.991	0.969	0.000515781736721712	CD69
Successfu	6	1.94694857640381e-08	0.52291709917346	0.82	0.556	0.000652967613554309	SH3BGRL3
Successfu	6	2.08430086912659e-08	0.501772861101111	0.505	0.237	0.000699032825487676	RABAC1
Successfu	6	2.28475233707948e-08	0.50308287804356	0.333	0.129	0.000766260238809717	NXT1
Successfu	6	2.48078254971451e-08	0.45680079245936	0.748	0.452	0.000832004851523251	TRAC
Successfu	6	3.62858208276175e-08	0.51448762096256	0.676	0.414	0.00121695385891664	CD37
Successfu	6	3.89382479237151e-08	0.65539526719622	0.667	0.448	0.00130591095886556	SLC25A6
Successfu	6	4.1196358708056e-08	0.38893663482624	0.802	0.508	0.00138164347835078	TMA7
Successfu	6	4.73949786457565e-08	0.37150268064939	0.162	0.037	0.00158953279382138	ADPRHL2
Successfu	6	5.34505794390091e-08	0.44251638193264	0.324	0.126	0.00179262553322549	ATRAID

Successfu	6	6.24044496999062e-08	0.642493272989751	0.865	0.701	0.00209292043403545	HMGB1
Successfu	6	8.36329245900352e-08	0.598162184498378	0.928	0.816	0.0028048810249006	HSP90AB1
Successfu	6	1.09817084697324e-07	0.462884532469652	0.91	0.725	0.00368304538657885	H3F3A
Successfu	6	1.44268719119058e-07	0.554857603082562	0.477	0.245	0.00483848430181498	SERTAD1
Successfu	6	1.50241504428655e-07	0.501514252875656	0.369	0.16	0.00503879957552824	LAT
Successfu	6	1.53625357813677e-07	0.503575160506889	0.865	0.697	0.00515228725035508	ITM2B
Successfu	6	1.67837667281464e-07	0.476375172585054	0.811	0.537	0.00562893968528574	COX4I1
Successfu	6	1.70075843823369e-07	0.565186641178015	0.55	0.302	0.00570400365014815	TLN1
Successfu	6	2.20349591627046e-07	0.503351480795641	0.396	0.183	0.00739008460398787	NFKBIB
Successfu	6	2.50624980573815e-07	0.393412555305574	0.955	0.833	0.0084054605984846	RPS24
Successfu	6	2.68295126989244e-07	0.437006709703543	0.955	0.846	0.00899808196896526	H3F3B
Successfu	6	2.71597883199213e-07	0.45232966259704	0.514	0.286	0.00910884980673522	SF3B5
Successfu	6	2.85229658907714e-07	0.48039139240856	0.946	0.794	0.0095660323004469	CALM1
Successfu	6	3.31231145566056e-07	0.409891536230448	0.946	0.839	0.0111088301599944	RPL34
Successfu	6	3.81458162136374e-07	0.424090282793762	0.333	0.141	0.0127933438417297	LYAR
Successfu	6	4.67821709836482e-07	0.560266794545417	0.523	0.305	0.0156898045044959	MAP1LC3B
Successfu	6	4.79581141667894e-07	0.527739540686783	0.541	0.299	0.0160841923292578	CMPK1
Successfu	6	5.43204578729397e-07	0.411815236870976	0.405	0.186	0.0182179951614265	SVIP
Successfu	6	5.69293309294273e-07	0.468583740486122	0.387	0.182	0.0190929590071113	REEP5
Successfu	6	6.61108502169014e-07	0.608867368510747	0.667	0.449	0.0221722569457444	STAT1
Successfu	6	7.17423313066812e-07	0.390227420503293	0.937	0.766	0.0240609430736348	RPL35
Successfu	6	7.29855689359798e-07	0.465965795020413	0.91	0.698	0.0244779001097489	CFL1
Successfu	6	8.21543293557252e-07	0.417033791105812	0.261	0.098	0.0275529189793231	LYSMD2
Successfu	6	8.37439829524892e-07	0.481143976625945	0.514	0.287	0.0280860570026058	GUK1
Successfu	6	9.78491019146654e-07	0.491416362765406	0.541	0.322	0.0328166318001405	ANP32B
Successfu	6	9.96411344014963e-07	0.502262344377534	0.712	0.477	0.0334176436555738	SUMO2
Successfu	6	1.04887113672741e-06	1.0499473785141	0.775	0.597	0.035177040183564	IER2
Successfu	6	1.16765186183991e-06	0.463600109601611	0.766	0.529	0.0391607081423869	MYL12B
Successfu	6	1.16832988376381e-06	0.478524691078442	0.604	0.363	0.0391834476416707	C11orf58
Successfu	6	1.34829254879601e-06	0.448021353046728	0.91	0.773	0.0452190355015204	UBC
Successfu	6	1.44383554202178e-06	0.429230428993539	0.64	0.394	0.0484233564083265	ATP6V1G1

Successfu	6	1.45741111709325e-06	0.57923002962551	0.622	0.403	0.0488786540450733	PPP1R15B
Successfu	12	4.9747893235035e-07	0.71334175840629	0.982	0.846	0.016684448433166	DNAJA1
Successfu	12	5.23429992820446e-07	0.38459830677042	0.255	0.065	0.0175547950992121	RPF2
Successfu	12	6.18653453723941e-07	0.76139537869405	0.455	0.169	0.0207483995309935	AFF3
Successfu	12	6.89791427959978e-07	0.54623624236156	0.273	0.076	0.0231342249109218	SEPHS2
Successfu	12	1.18930843525943e-06	0.55442431557563	0.309	0.095	0.0398870263017307	HBB
Successfu	12	1.19734744905024e-06	0.30623200176404	0.127	0.019	0.0401566387462469	SETMAR
Successfu	12	1.34904819655911e-06	1.17821611074905	0.4	0.145	0.0452443784161993	AC105402.3
Successfu	19	3.32684576558961e-23	1.07652933404905	0.833	0.515	1.11575753286344e-18	MT1G
Successfu	19	2.8146310354003e-16	0.81239074561916	0.194	0.026	9.43970956652552e-12	C1QB
Successfu	19	1.50476005256929e-13	0.86947511999854	0.769	0.584	5.04666426430688e-09	REG1A
Successfu	19	3.13724967394476e-13	0.84278976135493	0.844	0.78	1.05217079564759e-08	ACTG1
Successfu	19	2.46733759267571e-12	0.61856251480569	0.919	0.859	8.2749568183158e-08	TMSB10
Successfu	19	4.15252195081285e-12	0.84173167575603	0.656	0.488	1.39267281186361e-07	MT2A
Successfu	19	1.1148354317093e-11	0.62819689740728	0.941	0.888	3.73893507086665e-07	RPS2
Successfu	19	1.42871882189711e-11	0.72094791933176	0.919	0.893	4.79163718487852e-07	RPL41
Successfu	19	1.78863178603863e-11	0.72185677148234	0.86	0.844	5.99871328401634e-07	RPS14
Successfu	19	1.85199318269007e-11	0.80845300713984	0.194	0.046	6.21121473610596e-07	C1QA
Successfu	19	2.23387308810646e-11	0.65695715799261	0.925	0.86	7.49196356289143e-07	RPS19
Successfu	19	2.26044808482289e-11	0.82182060895036	0.919	0.895	7.581090786879e-07	ACTB
Successfu	19	2.70457430825983e-11	0.71875041754610	0.801	0.73	9.07060131504181e-07	RPL26
Successfu	19	3.45011070730954e-11	0.84349880340911	0.484	0.268	1.15709812901747e-06	MT1H
Successfu	19	5.89023433299402e-11	0.65085784738327	0.914	0.87	1.97546679059953e-06	RPS28
Successfu	19	6.15312736028812e-11	0.51429149293788	0.919	0.857	2.06363585409343e-06	FTL
Successfu	19	7.11392247411604e-11	0.73267039096768	0.909	0.88	2.38586731936904e-06	RPS12
Successfu	19	1.07591483927528e-10	0.41535998662757	0.124	0.016	3.60840318796142e-06	S100A9
Successfu	19	1.4980095940642e-10	0.69307232211544	0.78	0.697	5.02402457657253e-06	S100A6
Successfu	19	1.53813602948253e-10	0.65927316511862	0.86	0.812	5.1586006156785e-06	RPS15
Successfu	19	2.083286523077e-10	0.75060059975497	0.941	0.928	6.98692634109566e-06	RPLP1
Successfu	19	7.1398126710498e-10	0.87419120891243	0.462	0.283	2.39455037361668e-05	MT1X
Successfu	19	1.54098579742916e-09	0.6137850457295	0.876	0.875	5.16815816741791e-05	RPL28

Successfu	19	2.95201930443788e-09	0.61294510019036[illegible]	0.855	0.829	9.90048234322376e-05	RPL18
Successfu	19	3.78466432367955e-09	0.70082530479158[illegible]	0.78	0.727	0.000126930072087565	RPSA
Successfu	19	3.80031789789128e-09	0.57444361532996[illegible]	0.871	0.817	0.000127455061659478	RPLP2
Successfu	19	3.91755884753359e-09	0.61966636296269[illegible]	0.828	0.827	0.000131387088628581	RPS23
Successfu	19	8.72907747825001e-09	0.64852933006195[illegible]	0.876	0.849	0.000292755800465549	RPS8
Successfu	19	1.10648194998932e-08	0.63121393498602[illegible]	0.817	0.794	0.000371091916387419	RPL8
Successfu	19	1.3108881908554e-08	0.80870600592687[illegible]	0.333	0.163	0.000439645681449085	AGR2
Successfu	19	2.62742689870568e-08	0.73862292733658[illegible]	0.715	0.635	0.00088118643328791	MYL12A
Successfu	19	4.20963302257239e-08	0.57157329835397	0.855	0.801	0.00141182672311033	RPL29
Successfu	19	4.61285845372758e-08	0.57210856124675	0.882	0.865	0.00154706046821116	RPL11
Successfu	19	5.17570483067612e-08	0.53779185049078[illegible]	0.887	0.896	0.00173582788611216	RPL39
Successfu	19	6.21785060029876e-08	0.68102354462457[illegible]	0.532	0.398	0.0020853427343282	SNRPD2
Successfu	19	1.17077783485568e-07	0.603451805611143	0.785	0.748	0.00392655470253898	RPLP0
Successfu	19	1.70262856413759e-07	0.68433710108052	0.435	0.288	0.00571027567840465	NDUFB2
Successfu	19	1.85629223098814e-07	0.53402191053792[illegible]	0.887	0.842	0.00622563288428803	RPS3A
Successfu	19	2.13302520193146e-07	0.59914908761734[illegible]	0.742	0.724	0.00715373992223774	CFL1
Successfu	19	2.16905348320637e-07	0.59199597267396[illegible]	0.72	0.704	0.00727457157197752	SERF2
Successfu	19	2.34254755786607e-07	0.56614181169630[illegible]	0.774	0.732	0.00785643599957121	RPL37A
Successfu	19	2.62632266396276e-07	0.59090574346843[illegible]	0.812	0.796	0.00880816095039832	RPL6
Successfu	19	2.68020753822865e-07	0.53504001662272[illegible]	0.871	0.865	0.00898888004171126	RPS18
Successfu	19	2.68742241322874e-07	0.53520807356263[illegible]	0.855	0.824	0.00901307728948656	RPL9
Successfu	19	2.9360796770901e-07	0.56891928120686[illegible]	0.796	0.775	0.00984702402102479	HLA-C
Successfu	19	3.5017970833928e-07	0.65262678903153[illegible]	0.828	0.847	0.0117443270582828	RPL12
Successfu	19	3.55406902824173e-07	0.50617918187294[illegible]	0.871	0.831	0.0119196367069171	RPS15A
Successfu	19	3.55536831103796e-07	0.60994861200299[illegible]	0.71	0.643	0.0119239942415591	PPIA
Successfu	19	3.57761952481152e-07	0.53836334795446[illegible]	0.457	0.309	0.0119986203623129	PSMB1
Successfu	19	3.76322227278365e-07	0.53540409194151[illegible]	0.871	0.845	0.0126210948584618	RPL30
Successfu	19	5.30784042648409e-07	0.49323903422202[illegible]	0.828	0.77	0.0178014352223423	RPS9
Successfu	19	5.75040975893398e-07	0.73205650773056[illegible]	0.409	0.258	0.0192857242495128	ALOX5AP
Successfu	19	5.75069502464892e-07	0.54371144319804[illegible]	0.823	0.808	0.0192866809736675	RPS11
Successfu	19	6.94954513567198e-07	0.51840255380818[illegible]	0.14	0.041	0.0233073844760167	MT1B

Successfu	19	7.54826741720441e-07	0.50660133974928	0.882	0.821	0.0253153792638202	RPS3
Successfu	19	7.98327257789634e-07	0.55521621532503[illegible]	0.774	0.692	0.0267742995717488	VIM
Successfu	19	8.16716415225997e-07	0.74045527861494[illegible]	0.554	0.451	0.0273910351338495	IFITM2
Successfu	19	9.84139191470231e-07	0.64601272200646[illegible]	0.538	0.424	0.0330060602035286	PPIB
Successfu	19	1.02991416717485e-06	0.53922330809903[illegible]	0.806	0.775	0.0345412613387102	RPL7A
Successfu	19	1.14008280570434e-06	0.73008489452188	0.511	0.395	0.0382360971377122	OLFM4
Successfu	19	1.22503400393547e-06	0.52779983201875[illegible]	0.817	0.801	0.0410851904239876	RPS16
Successfu	19	1.37562977118174e-06	0.56823817151791[illegible]	0.72	0.678	0.0461358712658933	GAPDH
Successfu	19	1.44142929810752e-06	1.00223765684289	0.323	0.189	0.0483426557999299	LGALS1
Successfu	22	5.79377094311856e-07	0.84722892409075[illegible]	0.811	0.552	0.019431148989031	SSBP2
Successfu	22	8.85465054652365e-07	1.01961736896915	0.925	0.856	0.029696727002931	JMJD1C
Successfu	26	2.11787351275457e-38	2.00720333414516	1	0.985	7.10292418707629e-34	MT-ND3
Successfu	26	8.02771590641933e-30	1.33289067055822	1	0.992	2.69233536069491e-25	MT-ND2
Successfu	26	7.90386000926106e-24	1.10271806365176	1	0.982	2.65079656990598e-19	MT-ND1
Successfu	26	2.51238088687961e-17	0.97123613271380[illegible]	0.963	0.546	8.42602301841684e-13	MT1G
Successfu	26	3.06983573954357e-15	1.28310708914514	0.854	0.465	1.02956151032812e-10	MT-ATP8
Successfu	26	2.65515310877337e-14	1.27957211663088	0.841	0.486	8.90485249620411e-10	OSBPL3
Successfu	26	6.55382143252722e-12	0.65832781730536[illegible]	0.695	0.275	2.19802063204098e-07	MT1H
Successfu	26	7.29257197634383e-12	0.71598876436565[illegible]	0.878	0.504	2.44578278942619e-07	PLCG2
Successfu	26	1.45418315494787e-11	0.57744428787942[illegible]	1	1	4.87703946506417e-07	MT-CO3
Successfu	26	5.14789183730085e-11	0.85211214492039[illegible]	0.793	0.445	1.72649996439396e-06	MTRNR2L8
Successfu	26	1.09677387424852e-10	0.54350258479177[illegible]	0.329	0.084	3.67836021945467e-06	MT1M
Successfu	26	1.35532334787938e-10	1.04665108038602	0.878	0.588	4.54548344411785e-06	MT-ND4L
Successfu	26	3.92173464097016e-10	0.76095633849692[illegible]	0.854	0.625	1.31527136388857e-05	DNAJB6
Successfu	26	6.48812700320953e-10	0.58936116751316[illegible]	1	0.997	2.17598803433641e-05	MT-ND4
Successfu	26	1.8966296446408e-09	0.60693464078998[illegible]	0.768	0.403	6.36091650219631e-05	DEFA5
Successfu	26	2.32733922148566e-09	0.60352501641243[illegible]	0.512	0.198	7.80543028101861e-05	AC090627.1
Successfu	26	4.84616966745857e-09	0.74794468106823	0.354	0.117	0.000162530838307225	RAP1GAP2
Successfu	26	8.10655560301617e-09	0.69320235805895	0.634	0.288	0.000271877661813956	APOB
Successfu	26	2.06973504064196e-08	0.50407209646316[illegible]	1	1	0.000694147737930499	MT-CO1
Successfu	26	2.58183024758525e-08	0.66690766083490[illegible]	0.683	0.364	0.000865894228435141	CD28

Successfu	26	3.87464388460535e-08	0.56889455490020	0.976	0.888	0.00129947806601894	CDC42SE2
Successfu	26	4.16819662739302e-08	0.45707520488726	0.988	0.973	0.00139792978489507	MT-ND5
Successfu	26	4.96496639733828e-08	0.36452200270402	0.171	0.034	0.00166515043033931	HNF4A-AS1
Successfu	26	6.15021144353843e-08	0.42821940188281	1	1	0.00206265791393392	MT-CO2
Successfu	26	6.57205948571434e-08	0.28623276768448	0.159	0.029	0.00220413731031888	AC073529.1
Successfu	26	8.93258775425526e-08	0.56346115860264	0.427	0.171	0.00299581128102213	NMT2
Successfu	26	1.15609554720637e-07	0.52205059991089	0.488	0.218	0.00387731324622073	AGPAT4
Successfu	26	1.35016433483751e-07	0.62194068955041	0.976	0.848	0.00452818114617803	NEAT1
Successfu	26	1.4059859667589e-07	0.27086262618484	0.146	0.027	0.00471539573531599	PRKG2
Successfu	26	1.66288372515682e-07	0.29530923084212	0.22	0.056	0.00557697943743096	RNF185
Successfu	26	2.18968516546721e-07	0.64648354516343	0.439	0.184	0.00734376610794392	MTRNR2L1
Successfu	26	2.30466705188657e-07	0.26813166986271	0.366	0.125	0.00772939235861717	PBX1
Successfu	26	2.32358091026784e-07	0.65383596877034	0.915	0.723	0.00779282565685628	DPYD
Successfu	26	2.37354337486384e-07	0.52338055101341	0.768	0.417	0.00796038977061836	PTPN4
Successfu	26	2.42017989882911e-07	0.32151021932368	0.183	0.042	0.00811679934469308	POFUT2
Successfu	26	2.44921247255472e-07	0.51942369932527	0.244	0.072	0.00821416879045403	SLC14A1
Successfu	26	2.74333779073014e-07	0.58481832916918	0.5	0.228	0.00920060628255073	BRWD3
Successfu	26	2.9191709425979e-07	0.25052224920072	0.207	0.052	0.00979031550728485	C8orf33
Successfu	26	3.28139563877907e-07	0.54911637160788	0.439	0.188	0.0110051446933373	C11orf49
Successfu	26	3.38933102585329e-07	0.50039225099970	0.988	0.997	0.0113671383945068	MT-CYB
Successfu	26	4.59296253092758e-07	0.47679527186060	0.817	0.499	0.0154038777362249	ALDOB
Successfu	26	4.77988245679765e-07	0.36714350072282	0.159	0.034	0.016030769783608	SNHG22
Successfu	26	4.94691090889222e-07	0.30803900777271	0.183	0.044	0.0165909498062427	MLXIPL
Successfu	26	5.02904256862484e-07	0.55573418690401	0.415	0.177	0.016866402966654	P2RY8
Successfu	26	7.1007673544818e-07	0.54287169820945	0.573	0.289	0.0238145535534611	HIVEP1
Successfu	26	7.2108917009613e-07	0.38322881968062	0.293	0.097	0.024183888586684	POF1B
Successfu	26	7.92238119131726e-07	0.69650734482495	0.207	0.056	0.0265700820394398	FAM19A1
Successfu	26	8.92175573665298e-07	0.65137802609926	0.61	0.337	0.0299217843895868	PIBF1
Successfu	26	9.48425184710393e-07	0.25314893984998	0.207	0.056	0.0318082838448172	LIPE-AS1
Successfu	26	9.98236927031495e-07	0.47096543652541	0.293	0.1	0.0334788700587823	SLC41A2
Successfu	26	1.07018913622281e-06	0.51096536694275	0.476	0.219	0.0358920032506404	ZNF33A

Successfu	26	1.1991633869764e-06	0.45374225771862(	0.207	0.059	0.0402175416724146	PKD2L2
Successfu	26	1.22250960547174e-06	0.52352370136317	0.537	0.264	0.0410005271483111	DGKH
Successfu	26	1.24417101476734e-06	0.53862355529151	0.707	0.412	0.0417270074932669	RNGTT
Successfu	26	1.28156624756683e-06	0.74824761167205	0.756	0.513	0.0429811688108964	RUNX1
Successfu	26	1.37459258234131e-06	0.47136959298624	0.256	0.081	0.0461010860265629	PPARG
Successfu	26	1.39348445361221e-06	0.59081456131268	0.89	0.614	0.0467346816052463	ELOVL5
Successfu	33	6.70815460015263e-13	0.44409120064064	0.161	0.008	2.24978088979919e-08	LCT
Successfu	33	1.74781145702601e-09	0.50717042909926	0.226	0.026	5.86181006457382e-05	RNF207
Successfu	33	2.05289416873344e-09	0.97425790918071	0.935	0.451	6.88499646309822e-05	APOA4
Successfu	33	1.55887933086385e-08	0.31604562916174	0.161	0.014	0.000522816949985117	AC008537.4
Successfu	33	4.24026553197209e-08	0.65505618864104	0.419	0.097	0.0014221002541128	TBC1D8
Successfu	33	1.42372213858951e-07	0.64880751886214	0.258	0.043	0.00477487930840151	KLHDC1
Successfu	33	1.4830124330419e-07	0.29976794325097(	0.161	0.017	0.00497372709793591	AC004253.1
Successfu	33	1.50195767730497e-07	0.51374191556601	0.194	0.025	0.0050372656581454	AP003721.2
Successfu	33	2.87323223531342e-07	0.42056903655477	0.194	0.026	0.00963624627079414	EXD2
Successfu	33	3.01043186666128e-07	0.92555016078560	0.677	0.27	0.0100963863944086	VEZT
Successfu	33	4.7294347339889e-07	0.36605395406748	0.129	0.012	0.015861578210852	KIAA1257
Successfu	33	5.11929889540611e-07	0.73562374195043	0.258	0.048	0.017169104635413	NUP85
Successfu	33	7.76801783008944e-07	1.02710765512853	0.903	0.558	0.026052378198554	FBXL17
Successfu	59	5.3032614108538e-10	1.30013231058188	0.227	0.019	1.77860781197215e-05	NCAPG
Successfu	59	2.20703065251042e-09	2.05581572986092	0.182	0.013	7.40193940238946e-05	RRAD
Successfu	59	5.92085918152427e-09	0.82126816856892	0.227	0.022	0.000198573775229961	ASPM
Successfu	59	3.9271604586559e-08	0.55630030634322(	0.182	0.016	0.00131709107462402	IQGAP3
Successfu	59	1.44358226854811e-06	0.81057761266555	0.182	0.021	0.0484148621225666	HMBS
Successfu	64	1.67761837652527e-33	0.79997240631228	0.268	0.005	5.62639651119046e-29	TFPI2
Successfu	64	5.92832997136759e-25	1.29290710037923	0.634	0.098	1.98824330579726e-20	NRG1
Successfu	64	3.89858017582355e-16	1.187867526892	0.61	0.153	1.3075058193677e-11	TIMP1
Successfu	64	4.29877244344097e-16	0.54839810653979	0.22	0.015	1.44172230208123e-11	ARHGAP6
Successfu	64	4.17243942609474e-15	0.95448789277866	0.634	0.155	1.39935273472365e-10	TSPAN14
Successfu	64	1.05497196893914e-13	0.90113446247617	0.39	0.066	3.53816498942808e-09	GRB10
Successfu	64	3.06443855406021e-13	0.57218692144242	0.244	0.025	1.02775140226071e-08	IGFBP5

Successfu	64	3.89969989910983e-13	1.14021118356234	0.293	0.039	1.30788135216346e-08	EPHA4
Successfu	64	8.06335839165036e-12	0.299264926551789	0.122	0.005	2.7042891373917e-07	PREX2
Successfu	64	1.38472561364423e-11	1.38346669490997	0.683	0.224	4.64409276304002e-07	A2M
Successfu	64	1.08975799709439e-09	1.07691179472747	0.415	0.108	3.65483037065518e-05	AL589990.1
Successfu	64	1.64059856377781e-09	0.683652563356006	0.293	0.054	5.50223946319803e-05	IGFBP4
Successfu	64	1.67365459048628e-09	0.997519345128811	0.512	0.149	5.61310276557287e-05	RASA3
Successfu	64	3.53958914835264e-09	1.12356273515173	0.317	0.066	0.000118710740857451	CSGALNACT1
Successfu	64	4.41151924362813e-09	1.25972120722214	0.854	0.509	0.0001479535323928	BACH2
Successfu	64	1.17925431631619e-08	1.14788730800418	0.561	0.198	0.000395498312606125	FAM69A
Successfu	64	1.18642608596432e-08	0.907741818530757	0.634	0.246	0.000397903580710713	KLF2
Successfu	64	2.11179896891247e-08	0.565787971399345	0.195	0.028	0.000708255138193865	ADGRG5
Successfu	64	2.90266463799886e-08	0.858223514836515	0.488	0.15	0.000973495666292057	IFITM3
Successfu	64	2.94808003087571e-08	0.33935674551983	0.195	0.028	0.000988727080755096	COL1A1
Successfu	64	3.47492184827005e-08	0.876156982117698	0.634	0.259	0.00116541928947281	CA5B
Successfu	64	3.49250550506525e-08	0.598765403158203	0.317	0.07	0.00117131649628878	SOX5
Successfu	64	4.17432617014761e-08	1.04109000009135	0.61	0.255	0.0013999855109441	LMNA
Successfu	64	4.41672239252968e-08	1.02676738757647	0.902	0.635	0.00148128035600661	IQGAP2
Successfu	64	4.50175017666912e-08	1.26199491643152	0.878	0.595	0.00150979697425129	PDE3B
Successfu	64	5.80559442315496e-08	0.598888758083561	0.171	0.023	0.00194708025763771	URB2
Successfu	64	5.93952216090781e-08	0.95375372347292	0.463	0.145	0.00199199694232526	SVIL
Successfu	64	6.03601718686422e-08	0.891860750591838	0.293	0.061	0.00202435944413052	ST6GALNAC3
Successfu	64	6.12005338672823e-08	0.360031511027136	0.171	0.023	0.00205254350484091	FGFR1
Successfu	64	7.35596418491414e-08	0.526045990384955	0.171	0.023	0.0024670432683365	LDB2
Successfu	64	7.52563337099585e-08	0.47866472342969	0.317	0.073	0.00252394691996459	GGA3
Successfu	64	1.0237097500998e-07	0.740530920671653	0.439	0.138	0.00343331775988471	AC005920.2
Successfu	64	1.43633148488735e-07	0.975085393268103	0.854	0.486	0.00481716853401519	ITGA4
Successfu	64	1.47174860098596e-07	0.36185092273481	0.146	0.017	0.00493595045798671	MAP1B
Successfu	64	1.58678406664553e-07	0.348474580720138	0.171	0.024	0.00532175640271579	FGF7
Successfu	64	1.7540614921439e-07	1.12792387077839	0.659	0.301	0.0058827714323522	ITGB1
Successfu	64	2.30974705043816e-07	1.05064297387957	0.854	0.506	0.00774642965775951	AP001011.1
Successfu	64	2.51915247093574e-07	0.812711856202324	0.634	0.272	0.00844873355702428	IGF2R

Successfu	64	2.59833616505304e-07	0.48089737916292	0.171	0.025	0.00871429983035489	AC015795.1
Successfu	64	3.17153949135575e-07	0.54683635216850	0.366	0.102	0.0106367091461089	AL035634.1
Successfu	64	3.60569147371268e-07	0.57478012745459	0.293	0.069	0.0120927680645376	COL3A1
Successfu	64	3.77107620295924e-07	0.32206628603341	0.171	0.025	0.0126474353694847	ACTN1
Successfu	64	4.09607285748408e-07	0.65557576435056	0.341	0.092	0.0137374091494301	RFX2
Successfu	64	4.32820943469914e-07	0.73439947034343	0.537	0.203	0.014515948802094	FAM117A
Successfu	64	4.88148347669627e-07	1.05739156198082	0.659	0.299	0.016371519284144	SPTBN1
Successfu	64	5.08744236237562e-07	1.22909782754274	0.878	0.689	0.0170622641949353	CDC14A
Successfu	64	5.88108979091871e-07	1.45410398040792	0.756	0.428	0.0197239989407832	FNDC3B
Successfu	64	8.01801697098597e-07	0.35868572772198	0.171	0.027	0.0268908253172928	COL6A1
Successfu	64	8.52922506673129e-07	0.48946501598584	0.146	0.02	0.0286053150288034	PLEKHG3
Successfu	64	8.98958853492562e-07	0.59782427713617	1	0.91	0.0301492820284335	AKAP13
Successfu	64	9.71959989580591e-07	0.46974222149954	0.171	0.028	0.0325975941305539	COL1A2
Successfu	191	3.12096254027563e-23	0.75613354369421	0.488	0.084	1.04670841675764e-18	NRG1
Successfu	191	1.09447628165492e-20	0.30775651256567	0.146	0.003	3.67065455341428e-16	PAPPA
Successfu	191	2.40224299947667e-20	0.47744221515995	0.293	0.034	8.05664257164486e-16	PRR16
Successfu	191	3.6898508935898e-19	0.55795921041056	0.341	0.051	1.23750219269215e-14	COL3A1
Successfu	191	1.07949508326536e-18	1.66752790152723	0.634	0.204	3.62041061025537e-14	A2M
Successfu	191	2.3079957573056e-18	1.15214764274282	0.5	0.129	7.74055617085152e-14	RASA3
Successfu	191	1.4478573906895e-17	1.43058456817621	0.878	0.499	4.85582411689445e-13	RUNX1
Successfu	191	3.48383255943515e-16	1.32947802027469	0.439	0.11	1.16840776378336e-11	PZP
Successfu	191	6.0864481905176e-15	1.01080907154638	0.524	0.164	2.04127299413579e-10	P2RY8
Successfu	191	2.23458065771988e-14	1.05318012312293	0.878	0.462	7.49433660986095e-10	PAG1
Successfu	191	4.39342094919954e-14	1.0182108616471	0.817	0.412	1.47346551794254e-09	XYLT1
Successfu	191	9.79412260323322e-14	1.29413677112138	0.866	0.576	3.28475283867236e-09	ATP2B1
Successfu	191	1.44937422909065e-13	0.31206324654028	0.183	0.02	4.86091128952421e-09	IGFBP5
Successfu	191	3.77995335298372e-13	0.96460625242596	0.878	0.482	1.26772075552368e-08	OSBPL3
Successfu	191	6.30408591370978e-13	0.88352645120880	0.976	0.924	2.11426433373999e-08	ARHGAP15
Successfu	191	7.57501245005444e-13	1.04169877161057	0.976	0.792	2.54050767549926e-08	EML4
Successfu	191	9.97994153811075e-13	0.42761698468621	0.256	0.045	3.34707279305158e-08	COL4A1
Successfu	191	1.04838316836557e-12	1.63901027342549	0.378	0.107	3.51606747006444e-08	IFNG-AS1

Successfu	191	1.61071352077693e-12	0.86690978869689	0.683	0.292	5.40201100598165e-08	SLCO3A1
Successfu	191	3.60657631190499e-12	1.0001100454609	0.902	0.552	1.20957356348669e-07	TNIK
Successfu	191	5.59576407000871e-12	1.08021262369479	0.927	0.67	1.87670735379952e-07	CDK17
Successfu	191	1.15637364520693e-11	0.87290511146886	0.817	0.48	3.878245931295e-07	MAP3K5
Successfu	191	1.90510903718703e-11	0.88946679190201	0.854	0.552	6.38935468891787e-07	NAMPT
Successfu	191	2.08883758488339e-11	0.51669982394903	0.244	0.048	7.00554349218191e-07	ZNF346
Successfu	191	9.66975275072194e-11	0.85155721400953	0.902	0.681	3.24304167753713e-06	CYTH1
Successfu	191	2.26106488129937e-10	0.91340487928384	0.805	0.462	7.58315939890184e-06	CNOT2
Successfu	191	2.38658979132499e-10	0.45105477816958	0.232	0.046	8.00414484214575e-06	PRKX-AS1
Successfu	191	2.83144181839046e-10	0.65137718119295	0.451	0.163	9.49608957051791e-06	TSIX
Successfu	191	5.26266764593728e-10	0.89145766778084	0.793	0.452	1.76499347509445e-05	CAMK1D
Successfu	191	5.89259395388227e-10	0.40920866764766	0.159	0.022	1.97625816025304e-05	AL365275.1
Successfu	191	6.08029946742574e-10	0.70121127726875	0.927	0.722	2.03921083538525e-05	FOXN3
Successfu	191	7.39714804539237e-10	0.80157319490898	0.402	0.131	2.48085551146369e-05	MYBL1
Successfu	191	7.71162999577294e-10	0.51902423636633	0.488	0.176	2.58632646798233e-05	PIK3CB
Successfu	191	9.6062803315409e-10	0.75265921818919	0.915	0.633	3.22175429759219e-05	ELMO1
Successfu	191	1.05274668336276e-09	0.32600336757079	0.146	0.02	3.53070182666203e-05	C1orf112
Successfu	191	1.08204207057703e-09	0.40860993331205	0.268	0.065	3.62895269630123e-05	MCTP1
Successfu	191	1.26938349598044e-09	0.58621525656337	0.366	0.115	4.25725836881919e-05	CRIM1
Successfu	191	1.39819591294675e-09	0.74162097299340	1	0.909	4.6892694528408e-05	SIK3
Successfu	191	1.71040781440097e-09	0.41805104303115	0.256	0.06	5.73636572793798e-05	AC003681.1
Successfu	191	1.90546683224222e-09	0.50728314761295	0.5	0.191	6.39055466197395e-05	ACSL4
Successfu	191	2.07555366477074e-09	0.73069281036454	0.305	0.086	6.96099188090812e-05	FRY
Successfu	191	2.19281912652858e-09	1.15353414789667	0.78	0.524	7.35427678655155e-05	PHACTR2
Successfu	191	2.19589715826782e-09	0.74981014271091	0.939	0.697	7.36459988939862e-05	ZFAND3
Successfu	191	2.39335514892561e-09	0.78636324128631	0.768	0.404	8.02683449846671e-05	SMAD3
Successfu	191	2.45253002728475e-09	0.86122767328473	0.695	0.383	8.2252952055076e-05	GPHN
Successfu	191	2.70698759577546e-09	0.64786486934743	0.793	0.442	9.07869499871172e-05	CARD11
Successfu	191	2.71961969530392e-09	0.99572419009788	0.622	0.315	9.12106053411028e-05	BTBD11
Successfu	191	2.86046990196309e-09	0.87365747562960	0.476	0.198	9.5934439572038e-05	WDFY2
Successfu	191	3.52868379163794e-09	0.43386230227296	0.28	0.073	0.000118344997003953	C22orf34

Successfu	191	3.83309989438328e-09	0.67161178366114	0.902	0.647	0.000128554504257826	ANKRD11
Successfu	191	4.02738352846604e-09	0.80065399188939	0.78	0.468	0.000135070388777694	NR3C2
Successfu	191	4.33476087332037e-09	0.70520730318058	0.951	0.77	0.000145379210169419	UTRN
Successfu	191	5.14631798360973e-09	0.26366912345917	0.244	0.056	0.000172597212534303	RBPMS
Successfu	191	5.48039789985505e-09	0.64630311652714	0.89	0.66	0.000183801584765339	DYRK1A
Successfu	191	5.54116716133148e-09	0.43369129989779	0.293	0.08	0.000185839664256735	PSTPIP2
Successfu	191	5.84202781399109e-09	0.66161462015864	0.72	0.39	0.000195929928825633	DOCK9
Successfu	191	6.12999196170017e-09	0.85540217717402	0.854	0.598	0.0002055876704115	NFAT5
Successfu	191	6.29491251866483e-09	0.65154854479722	0.683	0.333	0.000211118776050981	KAT2B
Successfu	191	6.67758205220501e-09	0.37654125608418	0.11	0.011	0.000223952746866852	TRPA1
Successfu	191	7.32198028286273e-09	0.40036404650547	0.415	0.142	0.00024556457472665	XRRA1
Successfu	191	7.51760214873458e-09	0.61650523059750	0.512	0.202	0.00025212534086426	LINC00861
Successfu	191	9.80915201270887e-09	0.71693309116750	0.695	0.362	0.00032897934020223	CD28
Successfu	191	9.91027609447346e-09	0.56136021869397	0.988	0.883	0.000332370839656451	ANKRD44
Successfu	191	1.05424888245265e-08	0.28142800473239	0.183	0.035	0.000353573990196969	SDK1
Successfu	191	1.20346101099288e-08	0.69680282098048	0.732	0.386	0.000403616753866793	STAT5B
Successfu	191	1.21051968505878e-08	0.63842712230256	0.659	0.319	0.000405984091975014	MTFR1
Successfu	191	1.2397037771691e-08	0.36326410282971	0.195	0.041	0.000415771852786974	PHACTR2-AS1
Successfu	191	1.4352927247575e-08	0.69196156198638	0.732	0.412	0.000481368474029171	FNDC3B
Successfu	191	1.46677235794534e-08	0.70224782984496	0.89	0.558	0.000491926113407708	ZEB1
Successfu	191	1.52344088551073e-08	0.71505903205593	0.829	0.52	0.000510931604182589	COG5
Successfu	191	2.13965179123065e-08	0.77908540704737	0.659	0.327	0.000717596417742937	RANBP9
Successfu	191	2.24540875383377e-08	0.74791268262660	0.939	0.685	0.000753065187860768	PDE7A
Successfu	191	2.29256333086544e-08	0.91517828756238	0.793	0.486	0.000768879889905652	ARHGEF3
Successfu	191	2.40860669153095e-08	0.429182963411147	0.537	0.225	0.000807798512205651	STRN
Successfu	191	2.60954823836381e-08	0.66591650767341	0.549	0.247	0.000875190288182453	CA5B
Successfu	191	2.72018449869201e-08	0.78226687238316	0.817	0.584	0.000912295477171328	PLCB1
Successfu	191	2.75376739692572e-08	0.64243254893781	0.537	0.242	0.000923558509580949	ATR
Successfu	191	3.07949696851097e-08	0.55172577643040	0.451	0.181	0.00103280169329921	TGFBR1
Successfu	191	3.11069145367551e-08	0.35405890816761	0.195	0.042	0.00104326369973369	AL355916.2
Successfu	191	3.1857234996788e-08	0.96705760382801	0.78	0.451	0.00106842794732228	POLR2A

Successfu	191	3.27341899948269e-08	0.32842295564673‹	0.207	0.048	0.00109783926404651	GLIS3
Successfu	191	3.44752939596318e-08	0.67091923592050‹	0.512	0.215	0.00115623240881813	AGPAT4
Successfu	191	3.83476164370097e-08	0.68689840903302‹	0.829	0.521	0.00128610236006443	ATRX
Successfu	191	3.98905930004496e-08	0.66171521473807	0.793	0.478	0.00133785070804908	GPRIN3
Successfu	191	3.99221344994683e-08	0.32090467496119‹	0.232	0.058	0.00133890854684317	ADGRL2
Successfu	191	4.2364175085043e-08	0.64540058289202‹	0.829	0.604	0.00142080970400217	CEP85L
Successfu	191	4.32701797790127e-08	0.97896746254656‹	0.561	0.27	0.00145119528942853	LYST
Successfu	191	5.04148976559771e-08	0.70063880416618‹	0.805	0.478	0.00169081483758616	ZHX2
Successfu	191	5.16995866949531e-08	0.53620032508440‹	0.744	0.388	0.00173390073857534	RBM33
Successfu	191	5.48131530279841e-08	0.48208240098831‹	0.244	0.065	0.00183832352625253	RGPD2
Successfu	191	5.76476771651851e-08	0.43514166917701‹	0.195	0.044	0.00193338779676598	LONRF3
Successfu	191	6.45470442398604e-08	0.40761452128495‹	0.354	0.122	0.00216477876971644	EXT2
Successfu	191	7.06519394225024e-08	0.91110919066327	0.72	0.427	0.00236952474435189	NCALD
Successfu	191	7.19287041429849e-08	0.66597808496931‹	0.915	0.711	0.00241234487954743	RAB8B
Successfu	191	8.66337148696418e-08	0.34033575530530‹	0.268	0.076	0.00290552152929805	ZNF337
Successfu	191	1.0500217016887e-07	0.55904822297044‹	0.915	0.625	0.00352156278312355	NIPBL
Successfu	191	1.1273344937245e-07	0.65513127741533‹	0.732	0.456	0.00378085442505322	NEK7
Successfu	191	1.17693781672295e-07	0.77894625909504‹	0.329	0.119	0.00394721404972545	IGF1R
Successfu	191	1.17732002546747e-07	0.65432009874956‹	0.878	0.603	0.00394849590141281	INPP4B
Successfu	191	1.21424163735749e-07	0.71124023727697‹	0.841	0.581	0.00407232360336954	RFX3
Successfu	191	1.26534127677167e-07	0.82525338226581‹	0.878	0.678	0.00424370157403682	CDC14A
Successfu	191	1.37143261239237e-07	0.63392617313296‹	0.585	0.303	0.00459951069544152	ST6GAL1
Successfu	191	1.52165687234227e-07	0.69925616369956‹	0.756	0.448	0.00510333281846152	GPCPD1
Successfu	191	1.53154869339488e-07	0.37032185823660‹	0.232	0.063	0.00513650800790775	TRABD2A
Successfu	191	1.56005940071467e-07	0.56254410297692‹	0.39	0.149	0.00523212721811686	PELI2
Successfu	191	1.57546958907166e-07	0.31682067683169	0.329	0.108	0.00528380990782854	ANAPC4
Successfu	191	1.61197860179631e-07	0.54211716209937‹	0.951	0.787	0.00540625383470447	KMT2E
Successfu	191	1.74064830362925e-07	0.57700217529974‹	0.585	0.279	0.00583778628071178	OXSR1
Successfu	191	1.75520274926677e-07	1.04416366632886	0.476	0.223	0.00588659898049091	PCAT1
Successfu	191	1.82892301846156e-07	0.74977260819105‹	1	0.831	0.00613384201931639	CD44
Successfu	191	1.8318854063348e-07	0.26210537100983‹	0.402	0.152	0.00614377727576565	KDM5B

Successfu	191	1.9756112616464e-07	1.20712704176355	0.89	0.697	0.00662580504930969	FAM107B
Successfu	191	2.19806963727767e-07	1.06682561164569	0.5	0.247	0.00737188594950184	LMNA
Successfu	191	2.23786181451579e-07	0.584776927398811	0.756	0.447	0.00750534095352306	TBC1D22A
Successfu	191	2.28622954882805e-07	0.792921871553856	0.915	0.763	0.00766755666085952	PRKCH
Successfu	191	2.44174065826296e-07	0.578388394565811	0.878	0.633	0.0081891098196823	EXOC4
Successfu	191	2.53175358532814e-07	0.50432562261687	0.988	0.979	0.0084909951744735	MBNL1
Successfu	191	2.61647784455427e-07	0.393229957181712	0.561	0.268	0.00877514339506611	ANKH
Successfu	191	2.66797786497039e-07	0.531455934843089	0.951	0.788	0.00894786416353768	SYNE2
Successfu	191	2.78166133738675e-07	0.818416614184357	0.805	0.545	0.00932913579332769	RIPOR2
Successfu	191	2.845726978437e-07	0.624268404604794	0.585	0.305	0.00954399914028201	NRF1
Successfu	191	3.07519048088345e-07	0.636576578375191	0.829	0.625	0.0103135738347869	ARL15
Successfu	191	3.2650760625769e-07	0.501822622063967	0.366	0.139	0.0109504120986704	SMAD7
Successfu	191	3.31687172874324e-07	0.399981694945815	0.122	0.02	0.0111241244038591	ZDHHC11B
Successfu	191	3.42869952699199e-07	0.58024025757353	0.817	0.515	0.0114991724736257	RNF149
Successfu	191	3.75791400960583e-07	0.579820845951034	0.841	0.56	0.012603292005416	FRYL
Successfu	191	4.01014851872658e-07	0.358479828103399	0.354	0.129	0.0134492361021052	IQSEC1
Successfu	191	4.02682387780156e-07	0.571886284596111	0.329	0.119	0.0135051619213709	RAP1GAP2
Successfu	191	4.06328675399056e-07	0.281490222213965	0.207	0.053	0.0136274511155335	LAMB1
Successfu	191	4.60877581830624e-07	0.316432174040161	0.171	0.038	0.0154569123394355	AC097634.3
Successfu	191	4.65042853224202e-07	0.695008376607871	0.427	0.192	0.0155966072114333	ZDHHC14
Successfu	191	4.80817093826436e-07	0.710733448217806	0.427	0.18	0.016125643692751	SLC39A8
Successfu	191	4.96759874068283e-07	0.7093266542639	0.793	0.539	0.0166603326565021	ARHGAP26
Successfu	191	4.97590209147865e-07	0.404559644832482	0.902	0.591	0.0166881804344011	IGHA1
Successfu	191	5.0954567329825e-07	0.584928392846625	0.415	0.176	0.0170891427910767	BICD1
Successfu	191	5.41184601457063e-07	0.610451766986199	0.341	0.135	0.018150249163667	ATP11A
Successfu	191	5.45866311100145e-07	0.586908690249465	0.366	0.145	0.0183072643416767	AC079793.1
Successfu	191	5.7628778696052e-07	0.510211185083563	0.561	0.271	0.0193275397990819	SAFB2
Successfu	191	5.87119931547116e-07	0.66493086305672	0.854	0.567	0.0196908282642272	KAT6A
Successfu	191	5.9817168325511e-07	0.410361089386494	0.207	0.056	0.0200614819130099	EPB41L4A
Successfu	191	6.17862122566364e-07	0.504414619071874	0.366	0.139	0.0207218598666307	SLC45A4
Successfu	191	6.19950293271408e-07	0.420207774882085	0.671	0.357	0.0207918929357365	NFATC3

Successfu	191	6.27670378166767e-07	0.27393916080945	0.146	0.029	0.021050809142957	TSPAN18
Successfu	191	6.74188897070061e-07	0.56027391410293	0.463	0.204	0.0226109472299357	AC090627.1
Successfu	191	6.84728499913837e-07	0.50136725212580	0.89	0.638	0.0229644244301103	HNRNPH1
Successfu	191	6.92471851800675e-07	0.55998711414186	0.854	0.573	0.023224120965691	FAF1
Successfu	191	7.09131152639448e-07	0.25482344248302	0.134	0.025	0.0237828405972218	AC034195.1
Successfu	191	7.55722739158683e-07	0.57572763680727	0.354	0.139	0.0253454292259039	SVIL
Successfu	191	7.77983608197147e-07	0.54290464237942	0.598	0.296	0.0260920142517159	PTBP2
Successfu	191	7.81295483786282e-07	0.36705779907972	0.402	0.163	0.0262030879352243	RNF24
Successfu	191	7.84464213175357e-07	0.48312241220022	0.329	0.124	0.0263093607814751	DNAJC5
Successfu	191	8.29221691972763e-07	0.47555118730734	0.439	0.191	0.0278104371053825	DPP4
Successfu	191	8.38250904323629e-07	0.51224645666057	0.451	0.19	0.0281132588292059	CLYBL
Successfu	191	8.40607657023285e-07	0.47030608647998	0.841	0.563	0.0281922996012469	COP1
Successfu	191	8.43923839275503e-07	0.65680612660694	0.89	0.629	0.0283035177216218	DOCK2
Successfu	191	8.44915579735958e-07	0.47091552074248	0.268	0.087	0.0283367787131846	ZMIZ1
Successfu	191	8.50144235507889e-07	0.39384164666919	0.244	0.074	0.0285121373704636	CDC37L1
Successfu	191	8.65521936088573e-07	0.50211362003416	0.622	0.319	0.0290278746925386	TBC1D15
Successfu	191	9.36984508973399e-07	0.63175419597166	0.695	0.41	0.0314245864619499	TUT4
Successfu	191	9.58508966587442e-07	0.53204565883024	0.622	0.31	0.0321464737214096	NDRG1
Successfu	191	9.60640684850948e-07	0.54583561973556	0.78	0.517	0.0322179672885311	NSD3
Successfu	191	9.84700106863683e-07	0.43341509926533	0.366	0.146	0.0330248721839942	KIF16B
Successfu	191	1.02889052172473e-06	0.53020299944497	0.549	0.278	0.0345069303176041	SEC24B
Successfu	191	1.03426286145154e-06	0.89099698421952	0.39	0.174	0.0346871078473619	PTPRM
Successfu	191	1.05936511346318e-06	0.53340845837722	0.817	0.49	0.0355289871753282	EMB
Successfu	191	1.09047801062145e-06	0.26017336851399	0.232	0.067	0.0365724515202223	MMP25-AS1
Successfu	191	1.14751445921608e-06	0.73518751972609	0.744	0.5	0.038485339933189	SRPK2
Successfu	191	1.15753627127055e-06	0.29692973383644	0.488	0.209	0.0388214514658718	DDX60L
Successfu	191	1.24241686181198e-06	0.53924369633988	0.866	0.562	0.0416681767114503	TC2N
Successfu	191	1.26435414330733e-06	1.03493098650187	0.902	0.711	0.0424039092582412	RNF19A
Successfu	191	1.26495054513185e-06	0.775215511531147	0.549	0.299	0.0424239113826319	LUZP1
Successfu	191	1.3549558798582e-06	0.40823781634063	0.439	0.192	0.0454425102986844	GLMN
Successfu	191	1.35862726229673e-06	0.40931457599277	0.39	0.162	0.045565641122907	DDX39B

Successfu	191	1.37656802766679e-06	0.66575981950983[illegible]	0.671	0.402	0.0461673385118888	NCK2
Recoverec	0	4.26019506808792e-49	1.68616813482948	0.62	0.054	1.42878422193533e-44	REG1B
Recoverec	0	1.07044547618394e-20	1.52287375577924	0.96	0.703	3.5900600380257e-16	IGKC
Recoverec	0	2.04887172702804e-17	0.72672568225098[illegible]	0.36	0.054	6.87150599810665e-13	SERPINA1
Recoverec	0	8.07258633010805e-17	1.2612620830162	0.96	0.59	2.70738400339164e-12	FABP6
Recoverec	0	1.13526191006035e-16	1.1195623143125	0.5	0.113	3.8074413939604e-12	SLC5A1
Recoverec	0	2.90660764207773e-16	0.87795467481723[illegible]	0.48	0.101	9.74818071000028e-12	MUC17
Recoverec	0	5.77392621935558e-16	1.10998323271865	0.62	0.178	1.93645937544747e-11	KRT19
Recoverec	0	1.68309617569593e-15	1.04148716085186	0.78	0.271	5.644767954049e-11	JCHAIN
Recoverec	0	3.23167676357664e-15	0.76309985309627	0.1	0.003	1.08383975296833e-10	CRB1
Recoverec	0	4.84500767798332e-15	0.34097287319238[illegible]	0.14	0.008	1.62491867504205e-10	LCN2
Recoverec	0	1.05064588433827e-14	0.81539986439476[illegible]	0.56	0.145	3.52365616689369e-10	GDA
Recoverec	0	1.3172162395432e-14	1.22699227538733	0.74	0.303	4.41767982417999e-10	APOA4
Recoverec	0	5.20179749520616e-14	0.623725649211182	0.16	0.013	1.74457884394224e-09	BLK
Recoverec	0	8.24858164009468e-14	0.85165620457557[illegible]	0.5	0.126	2.76640931045495e-09	PDE3A
Recoverec	0	3.86065613091662e-13	0.99819258668131[illegible]	0.66	0.229	1.29478685318682e-08	APOC3
Recoverec	0	3.92661325941623e-13	0.69638640124219[illegible]	0.32	0.056	1.31690755494301e-08	CYP3A4
Recoverec	0	6.76669208721372e-13	0.80448730754071[illegible]	0.52	0.134	2.26941319220974e-08	ADIRF
Recoverec	0	1.00354188203106e-12	1.12564758217976	0.78	0.325	3.36567876395577e-08	CD74
Recoverec	0	1.21389984754855e-12	0.36525454017250[illegible]	0.14	0.011	4.07117730870831e-08	TEDC1
Recoverec	0	2.49815464320749e-12	0.28563321537657[illegible]	0.1	0.005	8.37831104238927e-08	CLCA4
Recoverec	0	8.98044841814197e-12	1.0542162983005	0.66	0.237	3.01186279047645e-07	SELENOP
Recoverec	0	2.34587834821413e-11	0.98519982751231[illegible]	0.8	0.37	7.86760680424055e-07	FABP1
Recoverec	0	2.59447862337616e-11	0.38852013983120[illegible]	0.2	0.026	8.70136240707895e-07	IGHA2
Recoverec	0	4.70659289945153e-11	0.27567801990453[illegible]	0.12	0.009	1.57849712661805e-06	CD79A
Recoverec	0	7.22501859672047e-11	1.05363768436405	0.7	0.282	2.42312673696811e-06	IGLC2
Recoverec	0	7.6621876427367e-11	0.60297029704748[illegible]	0.36	0.082	2.56974449162103e-06	PCK1
Recoverec	0	1.16555329635787e-10	0.90901945232594[illegible]	0.86	0.462	3.90903264532503e-06	IGHA1
Recoverec	0	1.7878337369009e-10	0.89830316774819[illegible]	0.42	0.116	5.99603678681825e-06	FGD4
Recoverec	0	2.44146788609539e-10	0.52229967720836[illegible]	0.38	0.093	8.18819499638671e-06	CTSD
Recoverec	0	2.97147119144178e-10	0.71541817691447[illegible]	0.52	0.162	9.96572008185745e-06	LMO7

Recoverec	0	3.18364787387731e-10	1.02596943517495	0.86	0.497	1.06773182394097e-05	PHGR1
Recoverec	0	3.74497936371768e-10	0.84713347249315	0.68	0.263	1.25599117900363e-05	LGALS3
Recoverec	0	1.36043691202682e-09	0.29861680709037	0.1	0.008	4.56263331555556e-05	TMEM37
Recoverec	0	1.45168014368559e-09	0.695281933001326	0.38	0.103	4.86864486589274e-05	PMP22
Recoverec	0	1.45632607577447e-09	0.830770645812442	0.44	0.134	4.88422639293241e-05	HLA-DRA
Recoverec	0	1.61762935643425e-09	0.339560447709017	0.18	0.026	5.42520533560918e-05	H1F0
Recoverec	0	2.18462895044332e-09	0.34205334424363	0.18	0.026	7.32680857399681e-05	FLVCR2
Recoverec	0	2.35370383500022e-09	0.736748238620587	0.36	0.095	7.89385192182374e-05	PDLIM1
Recoverec	0	3.39377917810876e-09	0.524360314126673	0.48	0.148	0.000113820566075411	SLC15A1
Recoverec	0	5.68881673582787e-09	0.699515637360457	0.46	0.145	0.000190791535686195	HIST1H2AC
Recoverec	0	6.01242111859309e-09	0.417778796865624	0.14	0.017	0.000201644579475375	MS4A1
Recoverec	0	7.25354193134309e-09	0.872900448612052	0.76	0.361	0.000243269289293385	ALDOB
Recoverec	0	7.85618015338995e-09	0.513698413925826	0.36	0.096	0.000263480569984392	EPB41L3
Recoverec	0	7.88463216671929e-09	0.634795148743083	0.4	0.118	0.000264434793607431	GUCA2A
Recoverec	0	9.27899320644712e-09	0.595245236009026	0.34	0.092	0.000311198874157824	MYH14
Recoverec	0	1.23661833496228e-08	0.498221960899738	0.16	0.024	0.000414737057179649	HSD11B2
Recoverec	0	1.28546141020902e-08	0.682600471822868	0.36	0.101	0.00043111804775590	TMC5
Recoverec	0	1.51572884171646e-08	0.577685212708003	0.28	0.066	0.000508345138934865	CXADR
Recoverec	0	1.66619020917215e-08	0.620313360938172	0.26	0.059	0.000558806872352154	CYBRD1
Recoverec	0	1.88266096793181e-08	0.46164680533144	0.28	0.065	0.000631406835424972	NT5C
Recoverec	0	2.75352821858697e-08	0.686724187487973	0.3	0.077	0.000923478293949699	PASK
Recoverec	0	4.01241137934496e-08	0.689546234287474	0.24	0.052	0.00134568252840471	HLA-DRB5
Recoverec	0	5.36570625038704e-08	0.658787248329512	0.36	0.106	0.0017995505622548	ELF3
Recoverec	0	5.91262448116428e-08	0.709971651821994	0.38	0.115	0.00198297599849288	C1orf21
Recoverec	0	5.99549135544624e-08	0.45293716742386	0.2	0.039	0.00201076789078956	TMPRSS2
Recoverec	0	6.11138009323257e-08	0.657422003773292	0.46	0.163	0.00204963465566834	MYO1E
Recoverec	0	6.74966300969328e-08	0.756806653589855	0.32	0.087	0.00226370198019093	AMN
Recoverec	0	8.72572669679408e-08	0.745235798773148	0.46	0.167	0.0029264342195708	CYB5A
Recoverec	0	8.94196043842309e-08	0.259657842546881	0.1	0.01	0.00299895469183834	G6PC
Recoverec	0	9.19738929102129e-08	0.97908947995898	0.6	0.287	0.00308462042042272	H1FX
Recoverec	0	9.36414529056059e-08	0.58247780752103	0.26	0.063	0.00314054704754821	CLDN3

Recoverec	0	1.12929101368324e-07	0.65605758280010	0.28	0.073	0.00378741620169085	COBL
Recoverec	0	1.31708316120578e-07	0.66590266730783	0.6	0.239	0.00441723350605193	MALRD1
Recoverec	0	1.43739402642765e-07	0.39095311380995	0.36	0.107	0.00482073208583305	KRT20
Recoverec	0	1.49480111033757e-07	0.34163515026039	0.18	0.033	0.00501326396385014	L3MBTL2
Recoverec	0	1.50298871027833e-07	0.33921920577392	1	1	0.00504072353653146	MT-CO3
Recoverec	0	1.52771164188092e-07	0.53807770015241	0.24	0.055	0.00512363930454022	SLC7A9
Recoverec	0	1.55318025177797e-07	0.56154171482834	0.22	0.047	0.00520905592841294	BANK1
Recoverec	0	1.64918184453971e-07	0.91102806182697	0.22	0.05	0.00553102607021727	AC097103.2
Recoverec	0	1.9848322825134e-07	0.63609634920483	0.28	0.075	0.00665673050909345	DPEP1
Recoverec	0	2.11573333521788e-07	0.51582149884852	0.9	0.529	0.00709574645965372	REG1A
Recoverec	0	2.32241421883203e-07	0.38953319847588	0.2	0.04	0.00778891280711885	SLC10A2
Recoverec	0	2.65733292176186e-07	0.56166504010228	0.32	0.092	0.00891216315300491	ERRFI1
Recoverec	0	2.80381917174118e-07	0.68176478039042	0.68	0.306	0.00940344873818557	PCSK5
Recoverec	0	2.86775588124423e-07	0.30636295362759	0.26	0.064	0.00961787967451691	TMEM45B
Recoverec	0	3.06816629347437e-07	0.31312019010411	0.16	0.028	0.0102900161150544	ANKRD9
Recoverec	0	3.35259989121213e-07	0.45170090350323	0.34	0.102	0.0112439495151473	C7orf50
Recoverec	0	3.89847427119268e-07	0.63624776019000	0.28	0.076	0.013074703010726	LYN
Recoverec	0	4.16484146006578e-07	0.28988549465177	0.2	0.041	0.0139680452887686	PLA2G16
Recoverec	0	6.2184138106732e-07	0.26769007862563	0.12	0.017	0.0208553162382358	PLEKHG6
Recoverec	0	6.87487227904773e-07	0.44522073081538	0.42	0.146	0.0230569466494703	SERPINB6
Recoverec	0	7.97538305951221e-07	0.63145764828521	0.44	0.165	0.0267478397049921	RBP2
Recoverec	0	9.07901467740733e-07	0.81397428498955	0.78	0.404	0.0304491994250887	PLCG2
Recoverec	0	1.02988964740519e-06	0.32735585414130	0.18	0.037	0.0345404389946753	PRKAB1
Recoverec	0	1.19560376904513e-06	0.50132397591588	0.2	0.045	0.0400981592062355	PRAP1
Recoverec	0	1.21408839858341e-06	0.38819867402800	0.16	0.03	0.0407180967116905	TK2
Recoverec	0	1.26881308049488e-06	0.53521203473897	0.44	0.167	0.0425534530936373	CYP3A5
Recoverec	13	1.12928275541178e-18	1.26729056398632	0.887	0.664	3.78738850510001e-14	RPL19
Recoverec	13	1.69766981267164e-16	0.70815241899392	0.574	0.22	5.69364501773816e-12	EIF3F
Recoverec	13	5.45360736288894e-16	1.03535647946849	0.73	0.393	1.82903083736569e-11	RPS10
Recoverec	13	1.19426586566932e-14	0.89285198891874	0.922	0.749	4.00532886028175e-10	RPL14
Recoverec	13	2.50556665640638e-14	0.64028435102372	0.609	0.258	8.40316945225572e-10	NOP53

Recoverec	13	4.51774859576667e-14	0.89204717060225‹	0.635	0.285	1.51516252404823e-09	DDIT4
Recoverec	13	8.67203524674965e-14	0.89204531136669‹	0.574	0.262	2.9084271810549e-09	PPDPF
Recoverec	13	1.34927709795171e-13	0.75704279095756‹	0.861	0.486	4.52520553111043e-09	GAPDH
Recoverec	13	1.35675450053558e-13	0.87982025510891	0.835	0.545	4.55028324389624e-09	EEF1B2
Recoverec	13	4.51545684343098e-13	0.85093539970990‹	0.913	0.707	1.51439391614988e-08	RPL8
Recoverec	13	5.23767506632991e-13	0.43826893407196‹	0.348	0.105	1.75661146374572e-08	ALKBH7
Recoverec	13	7.33727882346503e-13	1.01227038645302	0.93	0.764	2.4607765718137e-08	JUNB
Recoverec	13	1.61011156312802e-12	0.53326761506158‹	0.383	0.127	5.39999216041874e-08	GIMAP1
Recoverec	13	1.93034143752568e-12	0.49474524638187‹	0.339	0.106	6.47397911317364e-08	VAMP2
Recoverec	13	2.33487425118617e-12	0.81709776269268‹	0.887	0.719	7.83070126362819e-08	RPL7A
Recoverec	13	2.48120742038943e-12	0.97432950648917‹	0.922	0.765	8.32147344650207e-08	RPS4X
Recoverec	13	2.71156960863619e-12	0.71105276502444‹	0.53	0.23	9.09406215344406e-08	GIMAP4
Recoverec	13	2.73298897205537e-12	0.60426735678657‹	0.417	0.153	9.16589841447931e-08	EVI2B
Recoverec	13	2.94122046602692e-12	0.73675492307233‹	0.904	0.701	9.86426519896108e-08	RPS9
Recoverec	13	3.25547687230744e-12	0.84158444035372‹	0.957	0.835	1.09182183343447e-07	RPL13
Recoverec	13	5.13902375740221e-12	0.66672703586327‹	0.904	0.734	1.72352578775755e-07	HLA-A
Recoverec	13	5.16998775911901e-12	0.45130136295515‹	0.235	0.057	1.73391049465333e-07	BLOC1S4
Recoverec	13	5.402238078245e-12	0.89852769023616	0.939	0.758	1.81180260668181e-07	RPS3
Recoverec	13	1.10342358856971e-11	0.87598404653559‹	0.896	0.731	3.70066203134509e-07	RPL3
Recoverec	13	1.17859699535999e-11	0.28346121136425	0.13	0.019	3.95277860303832e-07	GRAP
Recoverec	13	1.19881476634944e-11	0.79007303209920‹	0.965	0.845	4.02058496338277e-07	FTH1
Recoverec	13	1.24269950450038e-11	0.61708944782228‹	0.487	0.199	4.16776559819336e-07	TPI1
Recoverec	13	1.24993983597386e-11	0.80330651169942‹	0.939	0.864	4.19204822188912e-07	RPL10
Recoverec	13	1.47435082794366e-11	0.33264768955265‹	0.261	0.068	4.94467780675744e-07	NDUFS7
Recoverec	13	1.60996356328633e-11	0.66502589193220‹	0.983	0.938	5.39949579854971e-07	TPT1
Recoverec	13	4.67140212986095e-11	0.46170452024609‹	0.487	0.2	1.56669484631276e-06	CUTA
Recoverec	13	5.05205342304413e-11	0.76937668471364‹	0.887	0.66	1.69435767702054e-06	RPLP0
Recoverec	13	5.15064632250282e-11	0.66038903417005‹	0.557	0.252	1.72742376364099e-06	TUBB4B
Recoverec	13	6.05589846422066e-11	0.57967447158184‹	0.583	0.273	2.03102722693033e-06	CORO1A
Recoverec	13	6.28261502239207e-11	0.73564546447730‹	0.913	0.758	2.10706342620985e-06	RPL6
Recoverec	13	6.80121698142552e-11	0.61877748155012‹	0.904	0.574	2.28099215123049e-06	FABP6

Recoverec	13	7.34898362790026e-11	0.28861380168727(	0.461	0.177	2.46470212912519e-06	UBALD2
Recoverec	13	7.41479158779511e-11	0.80992550626303	0.826	0.627	2.48677280271472e-06	RACK1
Recoverec	13	7.87319698470304e-11	0.76157969669509:	0.913	0.732	2.6405128047297e-06	RPL18A
Recoverec	13	8.72884931417822e-11	0.68331418436354	0.452	0.19	2.92748148298909e-06	GIMAP7
Recoverec	13	9.98392574198407e-11	0.59425808177852	0.53	0.244	3.34840901534662e-06	SERTAD1
Recoverec	13	1.44555214995371e-10	0.40686924983077:	0.487	0.204	4.84809280051476e-06	ERP29
Recoverec	13	1.57182993012526e-10	0.65639824609332(	0.809	0.544	5.27160321965408e-06	BTF3
Recoverec	13	1.88383778171125e-10	0.38931970781287:	0.583	0.262	6.3180151523032e-06	EDF1
Recoverec	13	1.91338949164346e-10	0.53591535494405:	0.583	0.277	6.41712567707384e-06	MPHOSPH8
Recoverec	13	2.22491716550944e-10	0.56784665470354	0.522	0.24	7.46192718968557e-06	NSA2
Recoverec	13	2.23029996279877e-10	0.69805745042968(	0.765	0.466	7.47998001523451e-06	SH3BGRL3
Recoverec	13	2.26553827753988e-10	0.60657712115044	0.922	0.821	7.59816227521325e-06	HLA-B
Recoverec	13	2.58590975454417e-10	0.71036158529839	0.852	0.691	8.67262413479022e-06	RPL23A
Recoverec	13	3.74228981926338e-10	0.50955618492069:	1	0.997	1.25508915958455e-05	MT-CYB
Recoverec	13	5.62779054987176e-10	0.68669705128686:	0.861	0.684	1.88744839461599e-05	RPL26
Recoverec	13	9.78665535763514e-10	0.60595914924672(	0.739	0.446	3.28224847384367e-05	COX4I1
Recoverec	13	1.09895086687118e-09	0.62486048588410(	0.887	0.724	3.68566141731255e-05	CALM1
Recoverec	13	1.24996554312527e-09	0.32944861720843(	0.296	0.098	4.19213443853353e-05	SELENOH
Recoverec	13	2.16566164146532e-09	0.74820466863790!	0.93	0.842	7.26319601314637e-05	RPS2
Recoverec	13	2.18110511542808e-09	0.70971414243254	0.887	0.81	7.3149903361227e-05	RPL11
Recoverec	13	2.53419604490144e-09	0.36860506703021	0.33	0.121	8.49918669539044e-05	TAF10
Recoverec	13	2.54931336179551e-09	0.51687230055985(	0.504	0.237	8.54988715278979e-05	CLIC1
Recoverec	13	2.61924406604443e-09	0.38889648255161(	0.243	0.075	8.7844207486998e-05	VEGFB
Recoverec	13	2.638743689874e-09	0.67433288880966:	0.617	0.302	8.84981858709943e-05	SAP18
Recoverec	13	2.82844972455285e-09	0.32778699457063	0.348	0.129	9.48605468620535e-05	SCAND1
Recoverec	13	3.01241474125829e-09	0.52741926076537	0.635	0.344	0.000101030365592321	SLC25A6
Recoverec	13	3.86199612670429e-09	0.64833016685019	0.974	0.864	0.000129523626097408	H3F3B
Recoverec	13	4.29098590056331e-09	0.44215481014710(	0.948	0.783	0.000143911085133092	FTL
Recoverec	13	4.80587525845938e-09	0.44566029794746!	0.791	0.479	0.000161179444418211	SERP1
Recoverec	13	5.02443622742658e-09	0.69997075349531(	0.983	0.94	0.000168509542195433	EEF1A1
Recoverec	13	5.95912528800336e-09	0.59641124586809(	0.922	0.761	0.000199857143909057	RPS25

Recoverec	13	6.00032496687348e-09	0.55905194040121ɩ	0.774	0.491	0.000201238898739003	YBX1
Recoverec	13	6.14737651330249e-09	0.52454292737184ī	0.591	0.31	0.000206170713503139	HMGN1
Recoverec	13	6.71849755861673e-09	0.57916393863781ɩ	0.53	0.272	0.000225324971120888	LDHB
Recoverec	13	6.74673622876396e-09	0.38578866755571ī	0.478	0.212	0.000226272039640286	VAMP8
Recoverec	13	9.50085857766193e-09	0.59641686241080ɩ	0.557	0.291	0.000318639794977626	EIF3K
Recoverec	13	9.85332564903311e-09	0.33765114914991ɩ	0.165	0.04	0.000330460835617272	UTP3
Recoverec	13	1.00145691632118e-08	0.68257074524711ɩ	0.861	0.704	0.000335868620595796	RPL21
Recoverec	13	1.08467466709581e-08	0.25928042445195ɩ	0.296	0.104	0.000363778189850592	TSTD1
Recoverec	13	1.12224904886446e-08	0.66158944720840ɩ	0.861	0.676	0.000376379886008164	PNRC1
Recoverec	13	1.3838699738717e-08	0.82088893249924ɩ	0.783	0.584	0.00046412231183709	RPS5
Recoverec	13	1.40374138085181e-08	0.72608656421605ɩ	0.635	0.348	0.000470786784310079	H2AFZ
Recoverec	13	1.42361902857773e-08	0.32285263024564ɩ	0.2	0.056	0.000477453349804399	RIC3
Recoverec	13	1.50763544788494e-08	0.44791466472329ɩ	0.339	0.133	0.000505630776511652	NSMCE3
Recoverec	13	1.56454615661265e-08	0.62178661991830ɩ	0.887	0.742	0.000524717490004751	RPL29
Recoverec	13	1.58734096017062e-08	0.62151468288295ɩ	0.93	0.822	0.000532362411222022	RPS14
Recoverec	13	1.62540973175251e-08	0.37675981107227ɩ	0.748	0.419	0.000545129915835158	OAZ1
Recoverec	13	1.77498858640655e-08	0.59419123389714ɩ	0.852	0.596	0.000595295672109028	NPM1
Recoverec	13	2.12514436909834e-08	0.30697330065456ɩ	0.391	0.163	0.0007127309185082	SBDS
Recoverec	13	2.41318607872123e-08	0.39283371377690ɩ	0.409	0.176	0.000809334347081525	HIGD2A
Recoverec	13	2.43485233365602e-08	0.42360933782599	0.391	0.168	0.000816600775661557	CCDC12
Recoverec	13	2.46155379432846e-08	0.50754497947260ɩ	0.748	0.494	0.000825555911541878	RPL4
Recoverec	13	2.51444299857803e-08	0.42654295002797ɩ	0.513	0.249	0.0008432938928631	RAC2
Recoverec	13	2.67491337244231e-08	0.27246978452119ɩ	0.252	0.083	0.000897112446849701	SNRPN
Recoverec	13	2.85188964376986e-08	0.45600581585134ɩ	0.983	0.9	0.000956466748727534	PTMA
Recoverec	13	2.87071983193952e-08	0.33236801992044ɩ	0.47	0.212	0.000962782017235875	COX6A1
Recoverec	13	3.1464351690922e-08	0.56689969747293ī	0.939	0.826	0.00105525142701014	RPL32
Recoverec	13	3.19512062517515e-08	0.4527114587932210	0.443	0.208	0.00107157955527124	TMEM50A
Recoverec	13	3.45046460938329e-08	0.41215403338663ɩ	0.452	0.212	0.00115721682069497	SLC25A5
Recoverec	13	3.56680786185365e-08	0.53441044910439ī	0.904	0.673	0.00119623602070848	HLA-C
Recoverec	13	3.66553096698783e-08	0.46054458832112ɩ	0.6	0.335	0.00122934577570838	TUBA1B
Recoverec	13	3.79860228618405e-08	0.33559418824654ī	0.33	0.129	0.00127397523474041	PRDX2

Recoverec	13	3.85728105126223e-08	0.32052997249775	0.278	0.1	0.00129365491897233	EMC7
Recoverec	13	4.13946018787658e-08	0.47336663705323	0.591	0.313	0.00138829215781005	LTB
Recoverec	13	4.90146269522293e-08	0.41159892648476	0.365	0.153	0.00164385255872387	PSMB8
Recoverec	13	5.05220109376097e-08	0.36036688762300	0.383	0.163	0.00169440720282555	CHURC1
Recoverec	13	5.10644824219386e-08	0.51126760581721	0.913	0.755	0.00171260061146698	RPL24
Recoverec	13	5.17968948583206e-08	0.35541477456216	0.452	0.212	0.00173716425975836	BUB3
Recoverec	13	5.49846475206049e-08	0.26073785552247	0.183	0.051	0.00184407510854605	SIT1
Recoverec	13	5.53669716953381e-08	0.2942091955848	0.522	0.248	0.00185689749671825	MZT2B
Recoverec	13	5.63956206565378e-08	0.53033395804283	0.757	0.497	0.00189139632557897	HNRNPA1
Recoverec	13	5.71766434588743e-08	0.39476211843448	0.278	0.1	0.00191759026832373	CBX4
Recoverec	13	5.78920227710724e-08	0.32994526944072	0.391	0.164	0.00194158265969623	NXT1
Recoverec	13	5.92820893125045e-08	0.48143247217463	0.513	0.265	0.00198820271136278	MAP1LC3B
Recoverec	13	6.23468615662233e-08	0.31869989503395	0.348	0.141	0.002090989043208	C1QBP
Recoverec	13	6.85681384076141e-08	0.50173433977339	0.452	0.219	0.00229963822591456	IL2RG
Recoverec	13	6.94105967993916e-08	0.30059564008718	0.4	0.176	0.002327892595458	NDUFB11
Recoverec	13	6.97140170279112e-08	0.38479613451943	0.252	0.088	0.00233806870308208	LYAR
Recoverec	13	7.14418221285099e-08	0.34607534169275	0.287	0.107	0.00239601583054597	FCMR
Recoverec	13	7.42553278382162e-08	0.58750336489062	0.757	0.557	0.0024903751850381	HLA-E
Recoverec	13	8.11143358735985e-08	0.54195704094855	0.757	0.512	0.00272041259652875	EEF1D
Recoverec	13	8.45762201952997e-08	0.52302581311422	0.417	0.196	0.00283651727290996	EIF3M
Recoverec	13	8.51314374543049e-08	0.37610571457755	0.33	0.133	0.00285513814934248	MEAF6
Recoverec	13	8.98283198979379e-08	0.27545600458796	0.191	0.057	0.00301266219273704	BTN3A3
Recoverec	13	9.46170899377654e-08	0.57691750042710	0.887	0.793	0.00317326796233278	RPL30
Recoverec	13	9.76788232852429e-08	0.35415088538386	0.33	0.132	0.00327595237534048	C8orf59
Recoverec	13	9.92606126224009e-08	0.48432198556528	0.626	0.367	0.00332900242613008	ATP5MC2
Recoverec	13	1.03806071827214e-07	0.54529555333188	0.87	0.741	0.00348144803694111	RPS7
Recoverec	13	1.0411872449472e-07	0.39497581151700	0.496	0.246	0.00349193378210392	GSTK1
Recoverec	13	1.09874659603023e-07	0.34214143274953	0.27	0.101	0.00368497633376617	PGLS
Recoverec	13	1.15753839777032e-07	0.39871964490951	0.374	0.164	0.0038821522784421	RPL17
Recoverec	13	1.19198984614387e-07	0.36584491023191	0.391	0.178	0.0039976955459973	LAMTOR4
Recoverec	13	1.24005612539483e-07	0.39089102544388	0.487	0.236	0.00415890023334917	EIF1B

Recoverec	13	1.30229870526061e-07	0.66780327856988	0.783	0.613	0.00436764939770302	RPL10A
Recoverec	13	1.32386301061764e-07	0.55802443981687	0.922	0.825	0.00443997176500945	RPS18
Recoverec	13	1.39464418780744e-07	0.47640939087807	0.452	0.231	0.00467735767706859	GPSM3
Recoverec	13	1.4048884030392e-07	0.28084204756369	0.261	0.093	0.00471171472611286	UCHL3
Recoverec	13	1.42833936179365e-07	0.65462608293181	0.887	0.797	0.00479036455158354	RPS3A
Recoverec	13	1.584007161987e-07	0.59241602758805	0.974	0.864	0.005312443219872	RPS12
Recoverec	13	1.59511474382822e-07	0.54766164825468	0.922	0.793	0.00534969582785107	RPS27A
Recoverec	13	1.63041996442593e-07	0.45178329285618	0.922	0.785	0.00546810247669167	RPS24
Recoverec	13	1.66341187336814e-07	0.38601248673224	0.226	0.078	0.00557875074090207	PAIP1
Recoverec	13	1.71895757524136e-07	0.46598813047708	0.687	0.417	0.00576503991584446	CALM2
Recoverec	13	1.72577545483324e-07	0.52507459685003	0.861	0.588	0.00578790572041973	PFN1
Recoverec	13	1.8561127535597e-07	0.39640688488620	0.835	0.543	0.00622503095288852	PPIA
Recoverec	13	1.88870749268238e-07	0.48686543711281	0.365	0.165	0.00633434718895815	TOB1
Recoverec	13	1.93476569699911e-07	0.37877866422234	0.478	0.231	0.00648881719459562	RGS10
Recoverec	13	1.96093497434489e-07	0.47434628128764	0.922	0.815	0.00657658371695788	RPL12
Recoverec	13	2.07144927872116e-07	0.49424891854041	0.739	0.473	0.00694722659097504	IRF1
Recoverec	13	2.11662657213685e-07	0.51524874834716	0.965	0.881	0.00709874219763256	RPL41
Recoverec	13	2.14081736932155e-07	0.62808904747068	0.896	0.807	0.00717987329323061	RPS8
Recoverec	13	2.16635380622355e-07	0.31485620872734	0.287	0.11	0.00726551739531254	MRPL34
Recoverec	13	2.17224661681195e-07	0.32618912712247	0.165	0.046	0.00728528070346392	CFL2
Recoverec	13	2.3809660170466e-07	0.59441870105888	0.817	0.618	0.0079852838279709	RPL7
Recoverec	13	2.38170510079022e-07	0.37304054192978	0.478	0.238	0.00798776256703024	APRT
Recoverec	13	2.39818874682564e-07	0.41858746927454	0.139	0.035	0.00804304541910382	SELL
Recoverec	13	2.40512692034769e-07	0.47623232995698	0.426	0.204	0.0080663146654621	PLAC8
Recoverec	13	2.42176529050197e-07	0.36495648780056	0.478	0.235	0.00812211643128551	HNRNPA0
Recoverec	13	2.62468439217151e-07	0.45178797403645	0.635	0.376	0.00880266651446483	EIF3E
Recoverec	13	2.74965008142436e-07	0.29014143377493	0.339	0.144	0.00922177644308103	GNB2
Recoverec	13	2.93394900212515e-07	0.36151186603707	0.4	0.188	0.00983987816332734	UBXN1
Recoverec	13	2.95006504316829e-07	0.25929230098548	0.261	0.097	0.00989392814177782	LSM2
Recoverec	13	2.95291145515227e-07	0.26784910661940	0.252	0.092	0.00990347443828967	GRK6
Recoverec	13	2.96345019783547e-07	0.38645622183953	0.261	0.1	0.00993881927350061	TBCC

Recoverec	13	3.00256878189654e-07	0.48687316494365€	0.948	0.832	0.0100700151807246	RPL28
Recoverec	13	3.03892421332908e-07	0.41993762820448	0.991	0.972	0.0101919440266631	B2M
Recoverec	13	3.06546143376053e-07	0.35695478675271‡	0.296	0.116	0.0102809445565461	GBP5
Recoverec	13	3.10897649485164e-07	0.31532328555744	0.2	0.065	0.0104268853684334	GBP4
Recoverec	13	3.10978266663924e-07	0.55131465777139‹	0.87	0.79	0.0104295891073747	RPS23
Recoverec	13	3.13840980816141e-07	0.26164315746379€	0.183	0.055	0.0105255988146117	DKC1
Recoverec	13	3.15881246681127e-07	0.30762768622593‡	0.235	0.083	0.0105940252511916	AIP
Recoverec	13	3.21009622474718e-07	0.62651584759487‹	0.661	0.409	0.0107660207185571	TRBC2
Recoverec	13	3.35993423310152e-07	0.33117210127363‡	0.339	0.146	0.0112685474309759	LMAN2
Recoverec	13	3.5777276662833e-07	0.46470867209821	0.47	0.234	0.0119989830471809	EID1
Recoverec	13	3.7569937942856e-07	0.46778922131239	0.843	0.662	0.012600205787275	RPL22
Recoverec	13	3.93493822077121e-07	0.91815470374039€	0.435	0.234	0.0131969958048225	KLF2
Recoverec	13	4.21825749997453e-07	0.40493092154650‹	0.391	0.18	0.0141471920034146	GPX4
Recoverec	13	4.89109910224426e-07	0.38743839928328	0.365	0.166	0.0164037681691068	VDAC2
Recoverec	13	5.01676638464745e-07	0.39281190414227‡	0.278	0.11	0.0168252311008306	RAD23A
Recoverec	13	5.0698108896339e-07	0.26853253433572	0.217	0.074	0.0170031317616542	CD27
Recoverec	13	5.20026001912095e-07	0.25282712588002‹	0.217	0.075	0.0174406320521278	GTF3C6
Recoverec	13	5.26897820997064e-07	0.30542061750515	0.27	0.106	0.0176710991205995	RPAIN
Recoverec	13	5.27986008689378e-07	0.31268218368360‡	0.443	0.217	0.0177075947594243	ATP5F1D
Recoverec	13	5.42378585663687e-07	0.32578563914066‹	0.226	0.081	0.0181902930059887	C3orf38
Recoverec	13	5.64508612700526e-07	0.42232390815658‡	0.53	0.288	0.0189324898527502	RARRES3
Recoverec	13	5.68651461227231e-07	0.31187931522126‡	0.278	0.111	0.0190714327066389	PSMA2
Recoverec	13	5.80126592860149e-07	0.28589051321375	0.2	0.067	0.0194562856713437	NUDCD2
Recoverec	13	6.17540015891525e-07	0.60041185752732€	0.609	0.361	0.02071105705297	TRAC
Recoverec	13	6.36944571037563e-07	0.35706534132594‹	0.409	0.199	0.0213618470234578	EIF3G
Recoverec	13	6.36976368890368e-07	0.72985604234542€	0.539	0.293	0.0213629134598452	DUSP2
Recoverec	13	6.49631723848269e-07	0.25218799807922	0.417	0.195	0.0217873487544232	AC058791.1
Recoverec	13	6.56345208303701e-07	0.28839454667169‡	0.243	0.09	0.0220125055960895	NDUFB5
Recoverec	13	6.68853063438328e-07	0.55288286015602‡	0.878	0.774	0.0224319940415946	RPL18
Recoverec	13	6.82754813067281e-07	0.33785225451660	0.609	0.341	0.0228982309206505	CYBA
Recoverec	13	6.91040729910668e-07	0.60492333114851‡	0.504	0.267	0.023176123999744	UBE2S

Recoverec	13	6.99597547010393e-07	0.66783887256205€	0.861	0.729	0.0234631025316346	RPL5
Recoverec	13	7.07878756992209e-07	0.45485847191070€	0.913	0.797	0.0237408377520047	RPL9
Recoverec	13	7.18553798127256e-07	0.27959073443052	0.513	0.263	0.0240988572815919	CIB1
Recoverec	13	7.18701947908892e-07	0.32877174778433[illegible]	0.313	0.133	0.0241038259289684	NRBF2
Recoverec	13	7.50393811715467e-07	0.31585433194049[illegible]	0.296	0.124	0.0251667076573133	BTN3A1
Recoverec	13	7.65733724610223e-07	0.40446605376719	0.4	0.194	0.0256811776559777	MIDN
Recoverec	13	8.2978485438843e-07	0.27497062904388[illegible]	0.2	0.068	0.0278293244464792	PHB2
Recoverec	13	8.45821216438317e-07	0.58765169907282[illegible]	0.452	0.249	0.0283671519569083	FXYD5
Recoverec	13	8.47971398271758e-07	0.34410422610477[illegible]	0.357	0.164	0.0284392647552382	CTSS
Recoverec	13	8.50582485434515e-07	0.41378617711694[illegible]	0.765	0.519	0.0285268353965028	MYL12A
Recoverec	13	8.53232596886533e-07	0.41816056614780[illegible]	0.348	0.162	0.0286157148343806	GYPC
Recoverec	13	9.0314541105156e-07	0.52404934266706	0.861	0.764	0.0302896907958472	RPS15
Recoverec	13	9.70472126971827e-07	0.37613844634126[illegible]	0.435	0.217	0.0325476941943811	ATP5MC3
Recoverec	13	9.7619687279817e-07	0.27954073851779[illegible]	0.409	0.197	0.032739690719905	RTRAF
Recoverec	13	1.03397504325832e-06	0.36039598627775[illegible]	0.287	0.12	0.0346774550007974	PLIN2
Recoverec	13	1.09520301050612e-06	0.34735453653121[illegible]	0.426	0.212	0.0367309185663542	JPT1
Recoverec	13	1.11462540481436e-06	0.53503659820137[illegible]	0.522	0.276	0.037382306826664	H1FX
Recoverec	13	1.13600793276423e-06	0.49953375465393[illegible]	0.922	0.804	0.0380994340490467	RPL13A
Recoverec	13	1.15114998261412e-06	0.33551216378558[illegible]	0.496	0.261	0.0386072681169122	HLA-F
Recoverec	13	1.21789204064806e-06	0.34300916441613[illegible]	0.591	0.331	0.0408456632592547	GMFG
Recoverec	13	1.28157291518711e-06	0.29869144852494[illegible]	0.687	0.405	0.0429813924295451	OST4
Recoverec	13	1.28954867253172e-06	0.32251799844088[illegible]	0.287	0.122	0.0432488833793688	ZNF394
Recoverec	13	1.38858836116239e-06	0.44268807137840[illegible]	0.391	0.191	0.0465704764566641	SOCS3
Recoverec	13	1.38876718709403e-06	0.26504878617966[illegible]	0.304	0.128	0.0465764739207597	LAT
Recoverec	13	1.39838680185608e-06	0.39478902008404[illegible]	0.496	0.26	0.0468990965606493	ITM2A
Recoverec	13	1.42945306737543e-06	0.34432140000455[illegible]	0.191	0.066	0.0479409969736371	TMEM238
Recoverec	13	1.44947530430679e-06	0.32280884214997[illegible]	0.183	0.06	0.048612502755841	PCNA
Recoverec	13	1.47133557257175e-06	0.26436925002245[illegible]	0.157	0.046	0.0493456524329112	MRPL16
Recoverec	20	5.31132798559849e-27	1.84432988879691	0.577	0.149	1.78131317981002e-22	AC105402.3
Recoverec	20	5.08298596543004e-20	0.61889820633970[illegible]	0.33	0.06	1.70473183308593e-15	RASA3
Recoverec	20	1.40326138714827e-19	0.59910921541894[illegible]	0.227	0.029	4.70625804021786e-15	SELL

Recoverec	20	3.21274725271552e-19	0.777364593112837	0.289	0.049	1.07749117361573e-14	CCR7
Recoverec	20	3.26792459039802e-18	0.755048580725545	0.227	0.031	1.09599654912769e-13	SLC16A10
Recoverec	20	5.39871484027621e-16	0.562863848319699	0.722	0.27	1.81062098313183e-11	OLFM4
Recoverec	20	1.2830385813244e-15	0.376733863766722	0.144	0.014	4.30305479404579e-11	CACHD1
Recoverec	20	1.32851502822454e-15	0.574370158740191	0.247	0.044	4.45557370165946e-11	FGFR1
Recoverec	20	5.08275572136409e-15	1.00781628139351	0.32	0.076	1.70465461383109e-10	LEF1
Recoverec	20	4.56535694229171e-14	0.308004372129706	0.124	0.011	1.53112941130579e-09	STIL
Recoverec	20	9.35671864525006e-14	0.286643127749409	0.155	0.019	3.13805629924397e-09	MYB
Recoverec	20	1.968793858266e-13	1.4571222617918	0.247	0.054	6.60294084185249e-09	NELL2
Recoverec	20	2.0760140463876e-13	0.664236487854594	0.557	0.197	6.96253590877475e-09	TXK
Recoverec	20	3.62037929978829e-13	0.341409903122531	0.588	0.205	1.214202809563e-08	MT1H
Recoverec	20	4.59846532257806e-13	0.474165444353496	0.165	0.024	1.54223329988623e-08	CCDC141
Recoverec	20	6.07032482563859e-13	0.561290063426468	0.186	0.031	2.03586554002267e-08	CNKSR2
Recoverec	20	1.27400416232741e-12	0.506714607100092	0.866	0.409	4.27275515961368e-08	MT1G
Recoverec	20	1.49254905887602e-12	1.35564271995795	0.732	0.367	5.00571103365841e-08	MTRNR2L8
Recoverec	20	3.29669289354259e-12	0.442363480179037	0.299	0.075	1.10564486263631e-07	LINC01550
Recoverec	20	5.60559777651525e-12	0.47842968238718	0.371	0.111	1.88000538228768e-07	TBC1D4
Recoverec	20	6.06593977218071e-12	0.503423324337436	0.361	0.107	2.03439488079397e-07	CHKA
Recoverec	20	7.47981243555634e-12	0.466564916682409	0.536	0.199	2.50857949463689e-07	USP10
Recoverec	20	1.03688908885548e-11	0.440774947430424	0.515	0.19	3.4775186262035e-07	ATF2
Recoverec	20	1.10815701884076e-11	0.414487892243021	0.278	0.07	3.71653700978815e-07	PASK
Recoverec	20	1.50408672883178e-11	1.0038291632565	0.763	0.372	5.04440607115601e-07	SERINC5
Recoverec	20	1.51536139205336e-11	0.443444177483804	0.505	0.181	5.08221903666856e-07	SAMHD1
Recoverec	20	1.73202524553035e-11	0.260378823055503	0.351	0.1	5.80886626845968e-07	TSPOAP1-AS1
Recoverec	20	1.90774745540643e-11	0.282134350953016	0.175	0.031	6.3982034159421e-07	C1orf162
Recoverec	20	3.09705000431445e-11	0.702539503533299	0.351	0.113	1.03868863044698e-06	IGF1R
Recoverec	20	3.22475414387048e-11	0.65696350280168	0.443	0.16	1.08151804477128e-06	P2RY8
Recoverec	20	3.72059897540915e-11	0.266187156411816	0.216	0.046	1.24781448437272e-06	NBPF12
Recoverec	20	4.60517159459229e-11	1.23021483610541	0.814	0.544	1.54448244939436e-06	BACH2
Recoverec	20	8.97052259349958e-11	2.90044735654303	0.371	0.115	3.00853386740789e-06	HBB
Recoverec	20	1.02023378611468e-10	0.464106135799232	0.278	0.075	3.42166007187142e-06	TIAM1

Recoverec	20	1.17975818337704e-10	0.31241221957125	0.309	0.087	3.95667299540991e-06	ID3
Recoverec	20	2.28747107995243e-10	0.50068940687127	0.361	0.12	7.67172050794444e-06	KLHL2
Recoverec	20	2.72439847035503e-10	0.70400826867345	0.763	0.43	9.13708758987669e-06	ZHX2
Recoverec	20	2.84151549265614e-10	0.26039918848328	0.155	0.027	9.52987465927017e-06	ABCB8
Recoverec	20	2.91882004482326e-10	0.44184722674952	0.557	0.227	9.78913866632824e-06	SAFB2
Recoverec	20	3.3352548696873e-10	0.47336125363555	0.546	0.223	1.11857777819573e-05	PSEN1
Recoverec	20	4.10728362377245e-10	0.39374910913765	0.443	0.158	1.3775007817408e-05	GTPBP1
Recoverec	20	4.25360121275401e-10	0.85014662857786	0.381	0.136	1.42657277473344e-05	SESN3
Recoverec	20	4.36410469152156e-10	0.34081035098874	0.268	0.073	1.4636334314425e-05	RUBCN
Recoverec	20	5.3238392317985e-10	0.36268553880645	0.155	0.028	1.78550920156058e-05	SH3RF3
Recoverec	20	5.33651902514259e-10	0.28917150184790	0.268	0.073	1.78976175065232e-05	AP000787.1
Recoverec	20	5.44919182607016e-10	0.37245543972534	0.32	0.099	1.82754995462741e-05	C9orf72
Recoverec	20	7.40210183660229e-10	0.45097694564919	0.423	0.154	2.48251691395967e-05	BTBD10
Recoverec	20	7.60764178958093e-10	0.47606585076901	0.474	0.184	2.55145090338965e-05	ZFX
Recoverec	20	8.26785367952338e-10	0.45410209729697	0.505	0.206	2.77287276703855e-05	IRS2
Recoverec	20	9.29134500019818e-10	0.54001251770271	0.216	0.054	3.11613128616647e-05	KIAA1958
Recoverec	20	1.04648594611969e-09	0.52041256279320	0.546	0.227	3.50970456609623e-05	APLP2
Recoverec	20	1.1128104602432e-09	0.42033377476464	0.515	0.208	3.73214372156363e-05	HMBOX1
Recoverec	20	1.19986713138871e-09	0.49268039382490	0.412	0.15	4.02411438525145e-05	FAM117B
Recoverec	20	1.2235010646355e-09	0.34351868526404	0.423	0.152	4.10337787057455e-05	PIK3CA
Recoverec	20	1.26351119848814e-09	0.59955109637205	0.443	0.172	4.23756385748953e-05	SERTAD2
Recoverec	20	1.3347765487701e-09	0.31286529384861	0.423	0.151	4.47657358926517e-05	SSBP3
Recoverec	20	1.5503544584221e-09	0.451258490111603	0.577	0.248	5.19957878265605e-05	FBXW11
Recoverec	20	1.56601183580241e-09	0.37411837744923	0.227	0.059	5.25209049491411e-05	ZNF589
Recoverec	20	1.60975078265616e-09	0.36135208353172	0.536	0.221	5.39878217487224e-05	USP53
Recoverec	20	1.70537106103254e-09	0.29667648253786	0.227	0.058	5.71947346449092e-05	AL592183.1
Recoverec	20	1.81295591485006e-09	0.69824105732018	0.959	0.715	6.08029154722412e-05	FOXP1
Recoverec	20	1.82493830285934e-09	0.43948697430720	0.412	0.153	6.12047808012965e-05	PARN
Recoverec	20	1.83144635094159e-09	0.49278329500106	0.66	0.317	6.14230477178791e-05	USP36
Recoverec	20	1.87907580427221e-09	0.36891067864412	0.412	0.15	6.30204443236814e-05	TRAK1
Recoverec	20	1.96029774424649e-09	0.32309586840593	0.196	0.045	6.57444657465387e-05	MAL

Recoverec	20	1.98548524883127e-09	0.38781702631961	0.443	0.167	6.65892042753032e-05	STK26
Recoverec	20	2.00885200562429e-09	0.40636212014987	0.33	0.108	6.73728785646273e-05	RABGGTB
Recoverec	20	2.10231079419887e-09	0.37382335568494	0.351	0.119	7.05072994158418e-05	MTERF4
Recoverec	20	2.22507541884559e-09	0.42493571549807	0.423	0.161	7.46245793972435e-05	TMEM243
Recoverec	20	2.48139725555218e-09	0.61822042251208	0.814	0.493	8.3221101156709e-05	PRKCA
Recoverec	20	2.49171679977739e-09	0.6158251498445	0.629	0.297	8.35671980309341e-05	ADD3
Recoverec	20	2.52208627322708e-09	0.45703478243322	0.536	0.234	8.45857294314897e-05	TLK2
Recoverec	20	2.59685650679214e-09	0.38620916926978	0.186	0.042	8.70933735247949e-05	NBEA
Recoverec	20	2.71245866062129e-09	0.45983763652243	0.546	0.234	9.09704385599168e-05	CDK12
Recoverec	20	3.27164197107289e-09	0.49700916900425	0.412	0.164	0.000109724328425843	TOB1
Recoverec	20	3.50394075441117e-09	0.34906009139034	0.361	0.127	0.000117515165021442	BNIP3L
Recoverec	20	3.68656618036437e-09	0.37909300239747	0.247	0.069	0.00012364005655706	HPS3
Recoverec	20	3.85705435187237e-09	0.47454997736118	0.68	0.321	0.000129357888853096	FBXO34
Recoverec	20	4.1597028738459e-09	0.49949985958269	0.794	0.424	0.000139508114983044	ATM
Recoverec	20	4.38375595030622e-09	0.48900994015988	0.68	0.338	0.00014702240706137	IL6ST
Recoverec	20	4.63660446761531e-09	0.38702279878898	0.526	0.221	0.000155502440634882	TBCK
Recoverec	20	4.70895530330904e-09	0.52155596204017	0.557	0.255	0.000157928942962379	ITPKB
Recoverec	20	4.74869722992434e-09	0.50188968971121	0.268	0.081	0.000159261807697203	TTN
Recoverec	20	5.09205808778618e-09	0.70091829017512	0.186	0.044	0.000170777444148173	CSGALNACT1
Recoverec	20	5.31236769931058e-09	0.44617226673686	0.567	0.251	0.000178166187899478	TBL1X
Recoverec	20	5.76262598286718e-09	0.45636128352589	0.526	0.227	0.0001932669502134	TET2
Recoverec	20	5.829869330915e-09	0.25617368288777	0.206	0.052	0.000195522157620227	CEP70
Recoverec	20	5.93574572840221e-09	0.42025813598666	0.464	0.188	0.000199073040239153	CARMIL1
Recoverec	20	6.23403647354528e-09	0.42128381596127	0.309	0.102	0.000209077115249762	RBM10
Recoverec	20	6.6688907432152e-09	0.33475165069840	0.608	0.273	0.000223661257745952	CHD6
Recoverec	20	6.90237174691979e-09	1.01604098376078	0.237	0.069	0.000231491743648196	TSHZ2
Recoverec	20	6.94239036304374e-09	0.63972041054690	0.515	0.215	0.000232833887995761	SIDT1
Recoverec	20	7.06175795023248e-09	0.58614614947753	0.629	0.316	0.000236837238134897	ALG13
Recoverec	20	7.4684596439966e-09	0.40522101201535	0.546	0.24	0.000250477199540358	SLC9A9
Recoverec	20	7.47573344000587e-09	0.45351381933284	0.515	0.221	0.000250721148110917	PDE8A
Recoverec	20	8.24123644464272e-09	0.33805871862871	0.608	0.267	0.000276394587880427	RNF10

Recoverec	20	8.46769286666738e-09	0.84442463652197	0.845	0.5	0.000283989483362291	MT-ND4L
Recoverec	20	8.65473328601863e-09	0.593933949067601	0.588	0.285	0.000290262444946493	SYAP1
Recoverec	20	8.78753779072031e-09	0.394616941710823	0.423	0.162	0.000294716442425178	DYNC1LI1
Recoverec	20	1.00757277716596e-08	0.51574837288746	0.474	0.206	0.000337919758005919	MAT2A
Recoverec	20	1.12018487251507e-08	0.43323136707507	0.814	0.477	0.000375687602544105	STAT3
Recoverec	20	1.23278571861654e-08	0.523372700377459	0.784	0.437	0.000413451674309615	MLLT3
Recoverec	20	1.30047262514048e-08	0.305706974988789	0.485	0.201	0.000436152509019613	RPS6KB1
Recoverec	20	1.3015145938722e-08	0.362855031813424	0.227	0.064	0.000436501964492859	ARRDC2
Recoverec	20	1.38668918944018e-08	0.941369566562832	0.33	0.118	0.000465067820354448	FAAH2
Recoverec	20	1.41735714221895e-08	0.399195263245538	0.485	0.204	0.000475353238357392	CERS6
Recoverec	20	1.47214059057884e-08	0.85420592463367	0.371	0.151	0.000493726511268332	XIST
Recoverec	20	1.53278428565721e-08	0.342735032897413	0.175	0.041	0.000514065193723714	PML
Recoverec	20	1.54966377425102e-08	0.366215319621572	0.155	0.033	0.000519726236608308	LINC00476
Recoverec	20	1.75731618966523e-08	0.281132449489501	0.278	0.088	0.000589368703689924	MASTL
Recoverec	20	1.87831181223558e-08	0.330429079756282	0.753	0.37	0.000629948215587569	HNRNPF
Recoverec	20	1.93656630136385e-08	0.550463354696517	0.402	0.164	0.000649485606151407	NFATC1
Recoverec	20	2.06214836380492e-08	0.439162486501834	0.299	0.101	0.000691603318252896	IQCN
Recoverec	20	2.11659942940991e-08	0.357899089882013	0.536	0.239	0.000709865116635495	YY1
Recoverec	20	2.12357149308168e-08	0.460980949118224	0.33	0.117	0.000712203407349733	KCNQ1OT1
Recoverec	20	2.15548386712661e-08	0.390529293064039	0.289	0.095	0.000722906179356921	POLA1
Recoverec	20	2.25411069280063e-08	0.440515432472566	0.639	0.294	0.000755983644151474	PCSK5
Recoverec	20	2.65368262933184e-08	0.382197835461045	0.66	0.319	0.000889992080225312	ADNP
Recoverec	20	2.68691899976961e-08	0.292303926593702	0.443	0.176	0.000901138894142733	NLRC5
Recoverec	20	2.77888819376901e-08	0.601335420385217	0.742	0.424	0.000931983522426251	MT-ATP8
Recoverec	20	3.05040314081002e-08	0.312988467225933	0.371	0.139	0.00102304420536486	HSD17B11
Recoverec	20	3.62483308832035e-08	0.3367274143078	0.557	0.247	0.00121569652116088	OXSR1
Recoverec	20	3.73617671111464e-08	0.292530304668931	0.577	0.261	0.00125303894537363	LARP4B
Recoverec	20	3.82443297797119e-08	0.678764108658348	0.784	0.475	0.00128263833215198	RIPOR2
Recoverec	20	3.94990960684445e-08	0.368001917114367	0.464	0.193	0.00132472068394349	ZMYM4
Recoverec	20	4.30509532695135e-08	0.49940745761418	0.649	0.329	0.00144384287075294	ATP9B
Recoverec	20	4.49869691759773e-08	0.254841984084429	0.32	0.11	0.00150877297222393	LY9

Recoverec	20	4.67595933699231e-08	0.25697080528282‹	0.144	0.031	0.00156822324244048	MYH10
Recoverec	20	4.84356714638137e-08	0.45182822562341‹	0.464	0.202	0.00162443554955338	TFRC
Recoverec	20	5.03292405100547e-08	0.26058656065748‹	0.536	0.233	0.00168794206822621	PIGR
Recoverec	20	5.41427278591062e-08	0.44222404030787‹	0.897	0.575	0.0018158388069387	ELMO1
Recoverec	20	5.42838989707266e-08	0.29512690203027‹	0.186	0.048	0.00182057340368023	ZNF627
Recoverec	20	5.75740468750441e-08	0.31430924946267‹	0.454	0.188	0.00193091838409523	PIK3IP1
Recoverec	20	6.11755387876634e-08	0.49611853052134‹	0.711	0.37	0.00205170521986065	EPC2
Recoverec	20	6.43291429526099e-08	0.32702551985479‹	0.402	0.16	0.00215747079634463	APC
Recoverec	20	6.61095357785803e-08	0.47588902610742	0.588	0.291	0.00221718161094203	STAT1
Recoverec	20	6.70305000548952e-08	0.27625414422369‹	0.309	0.108	0.00224806891084108	BANP
Recoverec	20	6.94114473209837e-08	0.27756631196384‹	0.351	0.129	0.00232792112025115	HDAC8
Recoverec	20	6.9829207745055e-08	0.25713244425863‹	0.299	0.103	0.00234193196935365	ZNF124
Recoverec	20	7.3990434570001e-08	0.28890822294513	0.474	0.199	0.00248149119460869	ZBTB44
Recoverec	20	7.55445921183707e-08	0.77942697293427‹	0.567	0.288	0.00253361453046592	TRPS1
Recoverec	20	7.59461198281671e-08	0.46684152959675‹	0.392	0.161	0.00254708096679707	CTCF
Recoverec	20	7.86654401234985e-08	0.35086650599525‹	0.474	0.204	0.00263828153086189	TPP2
Recoverec	20	8.30421306128088e-08	0.30887785047684‹	0.33	0.119	0.00278506697649238	CCDC14
Recoverec	20	8.86424562979035e-08	0.34777368843208‹	0.371	0.145	0.00297289069931909	LCLAT1
Recoverec	20	8.89105625888156e-08	0.59506851251477‹	0.918	0.54	0.0029818824481037	MTRNR2L12
Recoverec	20	9.19001683669239e-08	0.40982467455815‹	0.392	0.163	0.00308214784668989	DTNBP1
Recoverec	20	9.34295232017119e-08	0.25194802030281‹	0.278	0.094	0.00313343934913901	TCTN3
Recoverec	20	9.46119650576832e-08	0.38807971166189‹	0.155	0.036	0.00317309608410458	GSTCD
Recoverec	20	9.63568371939337e-08	0.39401236255653‹	0.454	0.194	0.00323161560581015	CRTC3
Recoverec	20	9.83197336703293e-08	0.40715939294503‹	0.268	0.089	0.00329744722783551	PLK2
Recoverec	20	1.02746351790969e-07	0.55125794145742‹	0.299	0.109	0.00344590714636551	UBE2E2
Recoverec	20	1.12204216981065e-07	0.90580929350712‹	0.155	0.037	0.00376310502911096	PDE7B
Recoverec	20	1.13882004548028e-07	0.39422679014846‹	0.485	0.221	0.00381937466853175	R3HDM1
Recoverec	20	1.13963013762856e-07	0.31980365331775‹	0.351	0.132	0.00382209155557866	NLRP1
Recoverec	20	1.1621606109231e-07	0.25877393934778‹	0.237	0.073	0.0038976542569139	ZNF44
Recoverec	20	1.17177360695907e-07	0.62169636966065‹	0.814	0.463	0.00392989432301933	PRDM2
Recoverec	20	1.19935723574199e-07	0.26468585833385‹	0.505	0.221	0.00402240429723148	CNTRL

Recoverec	20	1.21008239852009e-07	0.35921272997761ℇ	0.423	0.18	0.00405837434815667	TAF4B
Recoverec	20	1.25519806262232e-07	0.44227351158743ℇ	0.887	0.525	0.00420968326242274	ASH1L
Recoverec	20	1.26058596470902e-07	0.37894073532015ℇ	0.485	0.208	0.0042277532084411	BRD1
Recoverec	20	1.34840588149335e-07	0.40316188544309ℇ	0.711	0.374	0.00452228364535241	TUT4
Recoverec	20	1.34987294507262e-07	0.91937316860817ℇ	0.32	0.125	0.00452720388318456	HOMER1
Recoverec	20	1.41696517838035e-07	0.27812568337749ℇ	0.186	0.05	0.00475221781525201	EPHX2
Recoverec	20	1.64613931822917e-07	0.48775940068391ℇ	0.412	0.178	0.005520822045477	SP4
Recoverec	20	1.65635685846612e-07	0.34184467074744ℇ	0.619	0.307	0.00555508963192367	AFF1
Recoverec	20	1.75011073504451e-07	0.28363370162113	0.268	0.09	0.00586952138319229	PRCC
Recoverec	20	1.77122446609452e-07	0.28093328217292ℇ	0.485	0.207	0.00594033261438779	SNW1
Recoverec	20	1.80051958226945e-07	0.26452887024572ℇ	0.247	0.079	0.00603858257501527	RARA
Recoverec	20	1.8458151889497e-07	0.42812455071283ℇ	0.691	0.363	0.00619049498069952	GNAQ
Recoverec	20	1.85620815246951e-07	0.42931789639589ℇ	0.351	0.141	0.00622535090175225	ABHD17B
Recoverec	20	1.89350398310584e-07	0.29094678194646ℇ	0.371	0.147	0.00635043365854037	MSRA
Recoverec	20	2.00027613311889e-07	0.35699866153229ℇ	0.278	0.098	0.00670852609525414	PHF6
Recoverec	20	2.02893431284385e-07	0.56652352264423ℇ	0.711	0.407	0.00680463989841571	RGCC
Recoverec	20	2.08810631138504e-07	0.45546036288949ℇ	0.546	0.262	0.00700309094712313	ZBTB10
Recoverec	20	2.1616297656448e-07	0.45031163149793ℇ	0.649	0.346	0.00724967390801954	MSI2
Recoverec	20	2.17815453523481e-07	0.33567733434223ℇ	0.361	0.144	0.0073050946802705	FLNB
Recoverec	20	2.27223030807288e-07	0.51289308673393ℇ	0.134	0.03	0.00762060600721483	KLHL32
Recoverec	20	2.38774776249539e-07	0.47465188882588ℇ	0.619	0.331	0.00800802844585703	BORCS5
Recoverec	20	2.50664756300496e-07	0.67183466741543ℇ	0.423	0.186	0.00840679459680603	LYST
Recoverec	20	2.56885360630762e-07	0.26218252447039	0.505	0.226	0.00861542122483449	PDXDC1
Recoverec	20	2.66334668308879e-07	0.26927928744046ℇ	0.268	0.094	0.00893233210574319	USP38
Recoverec	20	2.7309121288667e-07	0.6215443937849	0.443	0.206	0.00915893309779315	FHIT
Recoverec	20	2.77425743998742e-07	0.38168189273165ℇ	0.577	0.286	0.00930430460222981	NDEL1
Recoverec	20	2.98831821076341e-07	0.31041153164717ℇ	0.34	0.129	0.0100222216152583	CDKN2AIP
Recoverec	20	3.24047300485471e-07	0.35024524584267	0.619	0.307	0.0108678983636817	FARS2
Recoverec	20	3.28551376473114e-07	0.73651830472206ℇ	0.474	0.234	0.0110189560641553	KLF2
Recoverec	20	3.37966116867169e-07	0.53085491696414ℇ	0.423	0.188	0.0113347076274911	TMEM184C
Recoverec	20	3.46845735949652e-07	0.26257121198465ℇ	0.155	0.038	0.0116325122922794	DTWD2

Recoverec	20	3.59748142260871e-07	0.42633183448206[illegible]	0.546	0.259	0.0120652331951451	ANKIB1
Recoverec	20	3.64213982807328e-07	0.31320382719211[illegible]	0.258	0.087	0.0122150085553922	PIP5K1B
Recoverec	20	3.67945828004501e-07	0.53325801167525[illegible]	0.619	0.358	0.012340167179615	RCOR1
Recoverec	20	3.98992877786247e-07	0.42413618810756[illegible]	0.443	0.196	0.0133814231351952	KPNA1
Recoverec	20	4.16643378860268e-07	0.32333143034622[illegible]	0.546	0.26	0.0139733856402157	NUMB
Recoverec	20	4.30870762684298e-07	0.29515914993974[illegible]	0.588	0.287	0.014450543638906	UBR3
Recoverec	20	4.52084870363514e-07	0.25959256401583[illegible]	0.371	0.153	0.0151620223822515	CASP3
Recoverec	20	4.65530102778755e-07	0.25440191248954[illegible]	0.247	0.081	0.0156129485869939	ECE1
Recoverec	20	4.67023950659287e-07	0.41349325758729[illegible]	0.711	0.388	0.0156630492572112	ABI1
Recoverec	20	4.68569016227978e-07	0.26312630269312[illegible]	0.227	0.071	0.0157148676662539	HSF2
Recoverec	20	4.80333500953642e-07	0.64272119807648[illegible]	0.361	0.156	0.0161094249549833	ZC3H12D
Recoverec	20	4.86791096633297e-07	0.32023959738559[illegible]	0.464	0.207	0.0163259997988875	TXLNG
Recoverec	20	4.89041244868552e-07	0.67900520848938	0.722	0.466	0.0164014652704015	CD55
Recoverec	20	4.93544882149309e-07	0.31995055704562[illegible]	0.546	0.267	0.0165525082575235	NDUFV2
Recoverec	20	5.22214710206857e-07	0.31485100189635[illegible]	0.33	0.131	0.0175140369509176	TSHZ1
Recoverec	20	5.52817305911723e-07	0.38996205922392[illegible]	0.526	0.251	0.0185403868056674	UBE2R2
Recoverec	20	5.77456718196011e-07	0.26067101390753[illegible]	0.216	0.066	0.0193667434148578	SLAIN1
Recoverec	20	6.06795636509753e-07	0.38758784134214[illegible]	0.907	0.569	0.0203507120572641	CYTH1
Recoverec	20	6.09594046413391e-07	0.31783371805839[illegible]	0.433	0.195	0.0204445651286123	PCNX4
Recoverec	20	6.2658803485802e-07	0.28990899318230[illegible]	0.124	0.027	0.0210145095130683	C8orf37-AS1
Recoverec	20	6.29614277706673e-07	0.38654940294835[illegible]	0.773	0.448	0.0211160036457264	PAPOLA
Recoverec	20	6.32482706185455e-07	0.43100765058597[illegible]	0.402	0.178	0.0212122050000478	FER
Recoverec	20	6.36458315570293e-07	0.30156075774981	0.113	0.023	0.0213455389875965	EPHA4
Recoverec	20	6.66393184260617e-07	0.39434567254906[illegible]	0.649	0.346	0.0223494946137326	ABLIM1
Recoverec	20	6.66554259120574e-07	0.27843126257250[illegible]	0.392	0.161	0.0223548967423858	IMMT
Recoverec	20	6.68965825749386e-07	0.34752659878307[illegible]	0.412	0.181	0.0224357758639829	PRKN
Recoverec	20	6.9291257583808e-07	0.26719138082107[illegible]	0.412	0.178	0.0232389019684575	FBXW4
Recoverec	20	7.28121649257117e-07	0.34483264689256[illegible]	0.711	0.397	0.0244197438727852	LINC01004
Recoverec	20	7.31471505174038e-07	0.31529476621950[illegible]	0.216	0.07	0.0245320913405269	CD84
Recoverec	20	7.89213343546962e-07	0.290111179902969	0.258	0.09	0.026468637115878	LRRC8B
Recoverec	20	7.97193165411975e-07	0.27445562021223[illegible]	0.423	0.181	0.0267362643815868	FAM168A

Recoverec	20	8.08446606381729e-07	0.31562577070079	0.402	0.174	0.0271136822848304	AQR
Recoverec	20	8.49324814922802e-07	0.26662931218774	0.258	0.091	0.0284846556428809	KIAA1324
Recoverec	20	8.96137809562999e-07	0.27618083835625	0.433	0.195	0.0300546698571239	TRRAP
Recoverec	20	8.96919620376848e-07	0.38051075037775	0.485	0.234	0.0300808902281987	ST6GAL1
Recoverec	20	9.16019708795113e-07	0.32903798462048	0.722	0.393	0.0307214689935705	TRIP12
Recoverec	20	9.49503690916375e-07	0.28477971631727	0.588	0.304	0.0318444547859534	DOCK9
Recoverec	20	9.92047277879773e-07	0.31293960993323	0.474	0.213	0.0332712816055318	SNX1
Recoverec	20	9.95112791699786e-07	0.26824194911923	0.567	0.265	0.0333740928080274	MAX
Recoverec	20	1.04326563562779e-06	0.48392247573394	0.381	0.167	0.0349890428876849	TPK1
Recoverec	20	1.061522148658e-06	0.2673188222666	0.701	0.363	0.0356013298216918	URI1
Recoverec	20	1.08666914221297e-06	0.25499070242716	0.34	0.136	0.0364447096915387	ZKSCAN1
Recoverec	20	1.14721277105086e-06	0.40735547845895	1	1	0.0384752219155039	MT-CO3
Recoverec	20	1.15132647955092e-06	0.26728028726940	0.588	0.298	0.0386131874711786	GSPT1
Recoverec	20	1.26153281396619e-06	0.32568009105310	0.381	0.164	0.042309287514798	ZC3H7A
Recoverec	20	1.27221028598759e-06	0.44997109573422	0.608	0.316	0.0426673885714517	ERC1
Recoverec	20	1.34446478737286e-06	0.44935110895563	0.649	0.353	0.0450906600389111	FUT8
Recoverec	20	1.35925476989023e-06	0.52519006440327	0.691	0.403	0.0455866864725786	POLR2A
Recoverec	20	1.40117981842961e-06	0.411151250666065	0.423	0.195	0.0469927687504924	MCU
Recoverec	20	1.48481454606674e-06	0.26428925458497	0.33	0.13	0.0497977102459862	CHIC1
Recoverec	26	7.13360729236512e-28	0.90750266893092	0.246	0.026	2.39246921371341e-23	C1QB
Recoverec	26	3.63364907024115e-23	1.13661055566645	0.802	0.404	1.21865322517748e-18	MT1G
Recoverec	26	1.00413535770973e-20	0.99716262267093	0.968	0.917	3.36766916268689e-16	HSP90AA1
Recoverec	26	5.45607950800979e-15	0.87004332195272	0.833	0.519	1.82985994539632e-10	HSPA1A
Recoverec	26	1.76610381190889e-14	0.94889791577445	0.341	0.108	5.92315896438003e-10	AGR2
Recoverec	26	5.75487846538247e-14	0.83600853493260	0.905	0.689	1.93007113971997e-09	DNAJB1
Recoverec	26	5.42787686662192e-12	0.82179499297269	0.77	0.519	1.82040134352766e-07	REG1A
Recoverec	26	8.93131516621918e-12	0.84659603034322	0.46	0.209	2.99538448044659e-07	MT1H
Recoverec	26	5.09199069819762e-11	0.74553083864955	0.675	0.395	1.70775184036152e-06	MT2A
Recoverec	26	3.04287860855984e-10	0.53847812844447	0.151	0.031	1.0205206277388e-05	C1QA
Recoverec	26	4.177096757604e-10	0.83662036830847	0.802	0.556	1.40091471056523e-05	HSPH1
Recoverec	26	4.32598768018638e-09	0.59229075270930	0.873	0.695	0.00014508497481809	IGKC

Recoverec	26	2.14288164337011e-08	0.47666365250081	0.905	0.786	0.000718679645553467	FTL
Recoverec	26	1.39544817280017e-07	0.59642522964688	0.762	0.524	0.00468005408193721	HSPD1
Recoverec	26	1.60608964470246e-07	0.46141920383796	0.944	0.833	0.00538650345040313	DNAJA1
Recoverec	26	2.03839398551108e-07	0.60675702540577	0.333	0.162	0.00683636574860707	MT1E
Recoverec	26	2.05488821617125e-07	0.63809438270637	0.897	0.799	0.00689168409939515	HSP90AB1
Recoverec	26	3.56517594628297e-07	0.43780889029744	0.802	0.583	0.0119568870886438	FABP6
Recoverec	26	5.77573639829649e-07	0.69719982832234	0.476	0.286	0.0193706647326068	OLFM4
Recoverec	26	7.9165040273693e-07	0.48721694968123	0.159	0.05	0.0265503712069911	TFF3
Recoverec	33	3.78950209025817e-22	2.48877256209417	0.333	0.094	1.27092321103078e-17	HBB
Recoverec	33	1.17853097979737e-13	0.50856367652565	1	0.955	3.95255720004441e-09	MT-ND2
Recoverec	33	7.70323361621258e-12	0.61439151813892	0.836	0.632	2.58351049020537e-07	PLCB1
Recoverec	33	1.73563904830601e-11	0.54101380266010	0.408	0.193	5.82098624020871e-07	MT-ND6
Recoverec	33	2.02990349142215e-11	0.51221948327612	0.812	0.613	6.80789032953159e-07	PPP3CA
Recoverec	33	2.71316942980362e-11	0.48374502776525	1	0.887	9.09942763367538e-07	MT-ND5
Recoverec	33	7.62860857304219e-11	0.53100318199726	0.643	0.409	2.55848274322689e-06	MT-ATP8
Recoverec	33	2.19796211479984e-10	0.60108455381431	0.84	0.703	7.37152534061569e-06	THEMIS
Recoverec	33	7.47522548017977e-10	0.63902487615457	0.526	0.319	2.50704112154269e-05	RFX7
Recoverec	33	1.22497289780297e-09	0.62181735108834	0.221	0.085	4.1083141046516e-05	HIST1H1E
Recoverec	33	1.67022084203654e-09	0.65542179577586	0.728	0.516	5.60158666002216e-05	IVNS1ABP
Recoverec	33	1.81128460549281e-09	0.46532910761969	0.789	0.606	6.07468630990179e-05	RAB8B
Recoverec	33	1.09120328620036e-08	0.51831351805304	0.225	0.09	0.000365967758125877	IL15
Recoverec	33	1.80102032378786e-08	0.52318360756936	0.751	0.554	0.000604026196191971	HNRNPU
Recoverec	33	3.99174406864713e-08	0.42037837333513	0.709	0.509	0.00133875112574287	SRRM2
Recoverec	33	4.38590501980259e-08	0.66486589047981	0.901	0.745	0.00147094482554139	ANXA1
Recoverec	33	7.88843571050759e-08	0.83100062074774	0.39	0.229	0.00264562356859004	IL2RA
Recoverec	33	1.06924214847771e-07	0.57797476768017	0.648	0.463	0.00358602431756456	SOS1
Recoverec	33	1.69549615337886e-07	0.75581266886115	0.207	0.088	0.00568635499920204	AC009299.3
Recoverec	33	1.83496143352211e-07	0.44361465203850	0.531	0.348	0.00615409365574646	CHIC2
Recoverec	33	1.88391402620432e-07	0.43910702345150	0.93	0.77	0.00631827086108406	PARP8
Recoverec	33	2.48134763235198e-07	0.50452973201859	0.399	0.233	0.00832194368938207	IL18R1
Recoverec	33	2.59110327178559e-07	0.51370775077706	0.906	0.819	0.00869004215291453	STAT4

Recoverec	33	2.62661294032136e-07	0.44583115027239{	0.934	0.829	0.00880913447924979	RAB11A
Recoverec	33	3.73845294914668e-07	0.39260558191902{	0.526	0.321	0.0125380235008481	ERCC1
Recoverec	33	5.96874575765863e-07	0.51272830235372{	0.385	0.231	0.0200179795220355	NCK1
Recoverec	33	7.27183302844572e-07	0.47383577368039{	0.911	0.849	0.0243882736108012	DENND4A
Recoverec	33	7.88863562312738e-07	0.44378773065244{	0.23	0.107	0.0264569061528446	AC097376.2
Recoverec	33	9.02953305661199e-07	0.49265185100239{	0.836	0.683	0.0302832479652653	BTG2
Recoverec	33	9.50249573886308e-07	0.50759585789728{	0.479	0.306	0.031869470208999	TTC33
Recoverec	33	9.81460285753723e-07	0.41769615804381{	0.704	0.546	0.0329162150636084	DDX3Y
Recoverec	33	1.08516754168286e-06	0.40009689186814{	0.789	0.611	0.0363943490129598	HIVEP2
Recoverec	33	1.163645752656e-06	0.46360516261575{	0.901	0.796	0.0390263512525768	HNRNPA2B1
Recoverec	40	8.83367604598944e-32	2.20070156826041	1	0.986	2.96263827230394e-27	MT-ND3
Recoverec	40	8.31816531280934e-26	1.65362409753684	1	0.961	2.78974628261e-21	MT-ND2
Recoverec	40	3.37180303520733e-23	0.78521280530613{	0.436	0.062	1.13083530194784e-18	MT1M
Recoverec	40	4.58913454571542e-23	1.9524880563844	1	0.424	1.53910394394204e-18	MT-ATP8
Recoverec	40	4.94429215043997e-23	1.49615391824587	1	0.949	1.65821670141456e-18	MT-ND1
Recoverec	40	8.37866244767954e-22	1.20339855787962	0.764	0.21	2.81003581170276e-17	MT1H
Recoverec	40	2.7280883060385e-20	1.22396707529582	1	0.981	9.14946256079191e-16	MT-ND4
Recoverec	40	3.03790230658807e-17	1.1851962256515	0.909	0.423	1.01885167558351e-12	MT1G
Recoverec	40	1.59297890679226e-16	1.16938257405975	1	0.902	5.3425326575999e-12	MT-ND5
Recoverec	40	2.81826145841475e-16	1.32270858813996	0.909	0.397	9.45188527923137e-12	PLCG2
Recoverec	40	4.39320544940738e-16	0.97298673594068	0.891	0.353	1.47339324362225e-11	ALDOB
Recoverec	40	5.02137307848715e-16	0.30060335102859{	0.109	0.004	1.68406810306302e-11	CDC42BPG
Recoverec	40	8.13208700520962e-16	1.07811793908601	1	1	2.7273393398072e-11	MT-CO3
Recoverec	40	6.22145145351244e-15	1.47207869573166	0.927	0.508	2.086550388479e-10	MT-ND4L
Recoverec	40	1.08878318449547e-14	0.53218179330811{	0.345	0.06	3.6515610441609e-10	SLC5A12
Recoverec	40	2.15646015233012e-14	1.31597221709585	0.745	0.268	7.23233605888477e-10	CROCC
Recoverec	40	4.05337643551956e-14	0.54947633008217{	0.382	0.076	1.35942138894455e-09	MGAM
Recoverec	40	1.68759121932887e-13	1.18022254775086	0.873	0.395	5.65984343138516e-09	AC022217.3
Recoverec	40	2.40270050878672e-13	1.18388272739955	0.818	0.376	8.05817696636891e-09	MTRNR2L8
Recoverec	40	2.76582175896045e-13	1.021190505871	1	0.997	9.27601301520154e-09	MT-CYB
Recoverec	40	3.52059339856188e-13	0.58986458310019{	0.364	0.074	1.18073661400968e-08	MTTP

Recoverec	40	1.24900401885143e-12	0.5856390409002	0.309	0.057	4.18890967842393e-08	MBNL1-AS1
Recoverec	40	1.40200132248845e-12	0.91629180044688€	1	0.998	4.70203203536177e-08	MT-CO1
Recoverec	40	1.7808091063539e-12	0.66612830037356₂	0.291	0.052	5.97247758088969e-08	HIST1H2BD
Recoverec	40	1.89655510727938e-12	0.53467436841016€	0.345	0.07	6.36066651879358e-08	ESRRG
Recoverec	40	1.98939341219999e-12	0.39897825442309⁄	0.109	0.007	6.67202762583632e-08	SEMA3F-AS1
Recoverec	40	3.29987025127813e-12	0.71854946500432!	0.364	0.079	1.10671048487366e-07	AL360012.1
Recoverec	40	3.47704755586162e-12	1.1110508699624	0.6	0.213	1.16613220928487e-07	MT-ND6
Recoverec	40	4.31252458657265e-12	0.36352243015845	0.255	0.041	1.44633449584473e-07	FRK
Recoverec	40	5.05479389310233e-12	0.58666902433393	0.491	0.133	1.69527677586866e-07	HNF4G
Recoverec	40	5.51207010572880e-12	0.71433529874282‧	0.473	0.126	1.84863807205933e-07	SOX6
Recoverec	40	8.94708337787517e-12	0.497539608455	0.236	0.037	3.00067282327177e-07	ADGRG7
Recoverec	40	9.28381480531052e-12	0.38971716090297⁄	0.236	0.037	3.11360580940504e-07	HIST1H4H
Recoverec	40	1.47214999002522e-11	0.69248746276266⁄	0.491	0.14	4.93729663654657e-07	OPRM1
Recoverec	40	1.67164087870339e-11	0.47337889520451‧	0.436	0.111	5.60634917899542e-07	PTPN9
Recoverec	40	2.35934689059211e-11	0.79186172876855‧	0.309	0.064	7.91277760166781e-07	PLEKHG2
Recoverec	40	2.77542322143484e-11	0.60573619746284‧	0.364	0.085	9.30821440004815e-07	DENND5B
Recoverec	40	3.71335738762454e-11	0.27626991335294‧	0.218	0.033	1.24538580066152e-06	SLC39A5
Recoverec	40	5.29047555066244e-11	0.47704942434937!	0.218	0.035	1.77431969018117e-06	MT1B
Recoverec	40	5.32573505906419e-11	0.70301162400931	0.564	0.19	1.78614502410895e-06	APOB
Recoverec	40	5.59079323887706e-11	1.17297161924545	0.273	0.054	1.87504023645459e-06	MTRNR2L1
Recoverec	40	6.73023076145599e-11	0.43206313926045‧	0.291	0.059	2.25718479277711e-06	MEP1A
Recoverec	40	7.65431641115638e-11	0.40563494767515‧	0.273	0.052	2.56710463797363e-06	SLC40A1
Recoverec	40	7.9587970422491e-11	0.49252864876305€	0.418	0.111	2.6692213520295e-06	PLS1
Recoverec	40	8.80712306641287e-11	0.51430736644331‧	0.527	0.159	2.95373293401355e-06	MBTD1
Recoverec	40	9.17925377650203e-11	0.77861236335234‧	0.436	0.122	3.07853813156325e-06	TET3
Recoverec	40	9.59866371806449e-11	0.80799415822602‧	0.382	0.101	3.21919983776447e-06	TESK2
Recoverec	40	1.1361709337135e-10	0.35297309867513‧	0.164	0.02	3.81049007748834e-06	SPIRE2
Recoverec	40	1.49460136034303e-10	0.43389521218387!	0.327	0.073	5.01259404231846e-06	MYO7B
Recoverec	40	1.79145824175573e-10	0.49402370172175‧	0.255	0.048	6.00819265120037e-06	UGT2B17
Recoverec	40	3.0259336675051e-10	0.30210940601728‧	0.2	0.031	1.01483763340786e-05	KALRN
Recoverec	40	3.5608054001895e-10	0.34397229532309‧	0.182	0.026	1.19422291511556e-05	PRUNE2

Recoverec	40	3.84121929628859e-10	0.76027866177563ℇ	0.727	0.302	1.28826812758927e-05	PCSK5
Recoverec	40	4.09953005448359e-10	0.27830523269521ℇ	0.2	0.031	1.37490038967271e-05	TOM1L1
Recoverec	40	4.25816553188411e-10	0.52968995143985ℇ	0.455	0.132	1.42810355608329e-05	ENPEP
Recoverec	40	4.82049435393669e-10	0.47696644040816ℇ	0.4	0.108	1.61669739642329e-05	POLG2
Recoverec	40	5.17972506051976e-10	0.56760484782452ℇ	0.509	0.165	1.73717619079712e-05	MT1E
Recoverec	40	5.56418986188116e-10	0.35102507076760ℇ	0.2	0.032	1.8661179958777e-05	KLHL25
Recoverec	40	5.74273279048017e-10	0.46347805527199ℇ	0.273	0.057	1.92599772327124e-05	CYP3A4
Recoverec	40	5.90416795307728e-10	0.39923954943396ℇ	0.291	0.063	1.98013984810306e-05	ACOX1
Recoverec	40	7.18316491221294e-10	0.71700909112936ℇ	0.382	0.105	2.40908984825797e-05	IQCN
Recoverec	40	7.32444817381151e-10	0.58076281942415ℇ	0.509	0.163	2.45647342853291e-05	CYP3A5
Recoverec	40	7.54863452971626e-10	1.23787988213661	0.909	0.554	2.53166104857624e-05	MTRNR2L12
Recoverec	40	9.3117300843547e-10	0.31366929650099ℇ	0.145	0.018	3.12296803569088e-05	SCN9A
Recoverec	40	1.01153266852043e-09	0.34022596496595ℇ	0.309	0.07	3.39247826368382e-05	CPS1
Recoverec	40	1.06618923419667e-09	0.37235120160865ℇ	0.182	0.028	3.57578545364878e-05	GPA33
Recoverec	40	1.06681833518348e-09	0.60497589805946ℇ	0.327	0.082	3.57789533253837e-05	SLC25A25
Recoverec	40	1.13811583739089e-09	0.69145396424509ℇ	0.473	0.151	3.81701289544155e-05	SNTB2
Recoverec	40	1.25157666681588e-09	0.81233513393428ℇ	0.745	0.34	4.19753782516711e-05	CAMKMT
Recoverec	40	1.30247365363538e-09	0.73587342070160ℇ	0.618	0.234	4.36823613956232e-05	ZDHHC14
Recoverec	40	1.30518747271999e-09	0.42357801164547ℇ	0.2	0.033	4.3773377460083e-05	ACVR1C
Recoverec	40	1.4200097856509e-09	0.35694015524825ℇ	0.236	0.046	4.76242881911598e-05	ACE2
Recoverec	40	1.44458818105727e-09	0.57857443513280ℇ	0.382	0.103	4.84485984162988e-05	MME
Recoverec	40	1.45463011668128e-09	0.62869069169299ℇ	0.218	0.041	4.87853848532569e-05	HIST1H2BC
Recoverec	40	1.92469883211499e-09	0.31857983226186ℇ	0.236	0.046	6.45505494314724e-05	CYP4F12
Recoverec	40	2.01846459694176e-09	0.50993311129253ℇ	0.582	0.204	6.76952656522328e-05	ANKH
Recoverec	40	2.02926963443866e-09	0.46820131561824ℇ	0.218	0.041	6.80576449998038e-05	GCNT2
Recoverec	40	2.16530584455583e-09	0.31371054494880ℇ	0.145	0.019	7.26200274147133e-05	FP236383.1
Recoverec	40	2.22979449073537e-09	0.53356838653494ℇ	0.382	0.105	7.47828476302829e-05	PARD3
Recoverec	40	2.44109186960171e-09	1.11612425414275	0.782	0.374	8.1869339122702e-05	GALM
Recoverec	40	2.63467876476344e-09	0.61427292578983ℇ	0.327	0.082	8.83618564126362e-05	TBC1D17
Recoverec	40	2.78843204407606e-09	0.31156935573190ℇ	0.182	0.029	9.35184338942231e-05	MAOB
Recoverec	40	2.90691466605205e-09	0.46670632912851ℇ	0.255	0.053	9.74921040700536e-05	EFCAB13

Recoverec	40	3.16252628653987e-09	0.343281342093117	0.218	0.041	0.000106064806597974	FBXL12
Recoverec	40	3.59994189743664e-09	1.10528619561687	0.491	0.173	0.00012073485135623	CCDC97
Recoverec	40	3.81641216671607e-09	0.53756762673746	0.709	0.289	0.000127994831247324	ARHGAP25
Recoverec	40	4.37651871194104e-09	0.428828160406287	0.382	0.108	0.000146779684561079	LLGL2
Recoverec	40	4.70924851701886e-09	0.55400070796586	0.345	0.094	0.000157938776763778	MEPCE
Recoverec	40	5.22784649217208e-09	0.31975259170133	0.182	0.03	0.000175331515654467	AC116366.1
Recoverec	40	5.54951099169748e-09	0.32002107951833	0.164	0.024	0.00018611949963955	TM6SF2
Recoverec	40	5.99247699503173e-09	0.47109437826632	0.436	0.134	0.000200975693459374	AK3
Recoverec	40	6.42332036650209e-09	0.79863210475719	0.582	0.23	0.000215425318451747	TBCK
Recoverec	40	7.44841578075996e-09	0.28245815988367	0.218	0.041	0.000249804968455128	CDC42EP5
Recoverec	40	7.45637031449181e-09	0.26700569880457	0.182	0.03	0.000250071747607426	AL121985.1
Recoverec	40	8.44081148485668e-09	0.73400637215907	1	1	0.000283087935579123	MT-CO2
Recoverec	40	8.68356347558252e-09	0.576110532689404	0.364	0.103	0.000291229351844087	SMIM24
Recoverec	40	8.90598412458279e-09	0.28474053570914	0.2	0.036	0.000298688895570258	BCL2L14
Recoverec	40	9.23786310968236e-09	0.55246162792878	0.291	0.071	0.000309819452972527	ASTL
Recoverec	40	9.36002974092574e-09	0.56529618040573	0.418	0.128	0.000313916677451167	PDE3A
Recoverec	40	1.10797994916832e-08	0.29083150196034	0.2	0.036	0.000371594315352071	TRHDE
Recoverec	40	1.1217391343499e-08	0.25657534346282	0.145	0.02	0.000376208870878268	LRP1B
Recoverec	40	1.29346262738683e-08	0.443511262920624	0.618	0.237	0.000433801495972994	SELENOP
Recoverec	40	1.37551967810757e-08	0.47929508492181	0.545	0.193	0.000461321789643716	MAPKAPK2
Recoverec	40	1.56551694404063e-08	0.47697477866431	0.364	0.105	0.000525043072692346	MUC17
Recoverec	40	1.66980144522737e-08	0.36175325975355	0.127	0.016	0.000560018008700354	LINC01924
Recoverec	40	1.82812367854643e-08	0.41411946417700	0.255	0.057	0.000613116119310903	VAMP1
Recoverec	40	1.90885519266946e-08	0.60378406384015	0.509	0.182	0.000640191854517485	MFSD6
Recoverec	40	1.93660137605069e-08	0.55291142948260	0.673	0.284	0.000649497369499881	ANPEP
Recoverec	40	1.9676324154175e-08	0.59200724389447	0.327	0.089	0.00065990455948272	CSRNP2
Recoverec	40	2.09393933723365e-08	0.57519704688739	0.473	0.166	0.000702265374921422	MUC20-OT1
Recoverec	40	2.12833988764712e-08	0.27558195942261	0.127	0.016	0.00071380263151909	ENPP3
Recoverec	40	2.642317143348e-08	0.64033458960693	0.509	0.183	0.000886180323536052	PTPRM
Recoverec	40	2.88328030471876e-08	0.35437905917569	0.345	0.095	0.000966994548596578	MT1F
Recoverec	40	2.88694641535616e-08	0.30378353438712	0.218	0.045	0.000968224088782149	ABO

Recoverec	40	2.93993456882293e-08	0.51890618706672	0.836	0.404	0.000985995255691836	MT2A
Recoverec	40	3.63523607614031e-08	0.66814403964263	0.418	0.136	0.00121918547521594	TMEM164
Recoverec	40	3.92762057221196e-08	0.66231827110370	0.782	0.403	0.00131724538750845	NABP1
Recoverec	40	4.36335634606103e-08	0.53241242223598	0.927	0.492	0.00146338245134195	PHGR1
Recoverec	40	4.43320113673637e-08	1.81812611938824	0.673	0.35	0.00148680699723864	TGFB1
Recoverec	40	4.88287863367435e-08	0.44499643940163	0.345	0.1	0.0016376198361617	MAOA
Recoverec	40	5.43530409700125e-08	0.53464261050897	0.636	0.268	0.00182289228805228	DCAF6
Recoverec	40	5.82781069683417e-08	0.33336738380478	0.164	0.028	0.00195453115150424	CA3
Recoverec	40	6.04103368547266e-08	0.41505993513622	0.255	0.061	0.00202604187743382	B3GNTL1
Recoverec	40	6.7588466472771e-08	0.91133770250379	0.691	0.341	0.00226678198856379	ERCC1
Recoverec	40	6.87826287381876e-08	0.25758796965692	0.109	0.013	0.00230683180262134	BYSL
Recoverec	40	8.10478309648295e-08	0.64566999165095	0.455	0.165	0.00271818215489845	CLEC16A
Recoverec	40	8.28336500597904e-08	0.41687098466262	0.582	0.23	0.00277807495570525	APOC3
Recoverec	40	8.46669040641461e-08	0.37337324346068	0.382	0.117	0.00283955862850333	PATL2
Recoverec	40	8.53370440554553e-08	0.52494310142141	0.382	0.122	0.00286203378353186	MLXIP
Recoverec	40	8.69251903174294e-08	0.43617276333464	0.418	0.138	0.00291529703286595	KIF16B
Recoverec	40	8.75655573137528e-08	0.80863537155568	0.964	0.592	0.00293677366118864	IKZF1
Recoverec	40	8.9854447095271e-08	0.28389489802005	0.145	0.023	0.0030135384466812	PAFAH2
Recoverec	40	9.8962022370125e-08	0.72975415957345	0.291	0.083	0.00331898830624925	AP002336.2
Recoverec	40	9.98113709869088e-08	0.43680907384861	0.455	0.156	0.00334747376015895	AC090627.1
Recoverec	40	9.98866562072966e-08	0.45039220339833	0.364	0.114	0.00334999867588031	PARD3B
Recoverec	40	1.01756448175111e-07	0.29899614546681	0.2	0.041	0.00341270775889687	GUCY2C
Recoverec	40	1.03709183645938e-07	0.37566521278657	0.218	0.048	0.00347819860111747	RMND1
Recoverec	40	1.12561929822773e-07	0.52326569944847	0.564	0.217	0.00377510200239614	HMBOX1
Recoverec	40	1.17456490468292e-07	0.47428606110566	0.2	0.042	0.00393925577732558	HIST4H4
Recoverec	40	1.19080905443228e-07	0.44474683019617	0.309	0.087	0.00399373540675499	ACE
Recoverec	40	1.20356522902995e-07	0.38224830459609	0.218	0.048	0.00403651706512066	XDH
Recoverec	40	1.22222827178771e-07	0.45897532123694	0.455	0.159	0.00409910917792161	CTNNA1
Recoverec	40	1.23007770523893e-07	0.64412920288293	0.836	0.419	0.00412543460783032	AMD1
Recoverec	40	1.25067876966437e-07	0.33270140138530	0.236	0.055	0.00419452645770035	RAB11FIP4
Recoverec	40	1.38553601063766e-07	0.51959044988923	0.236	0.057	0.00464681067247659	C22orf46

Recoverec	40	1.49541155007031e-07	0.49500946211750¢	0.436	0.154	0.0050153112566258	DIP2A
Recoverec	40	1.50500868988342e-07	0.39402094541931¢	0.164	0.03	0.00504749814413101	SLC1A1
Recoverec	40	1.55649834201675e-07	0.63693977359251¢	0.418	0.147	0.00522018413945577	ESR2
Recoverec	40	1.64849721169366e-07	0.73464991438423¢	0.818	0.408	0.00552872994857821	RUNX1
Recoverec	40	1.66232392242309e-07	0.51092326573838¢	0.455	0.164	0.00557510197102255	LMO7
Recoverec	40	1.66915429298475e-07	0.28144677155686¢	0.109	0.014	0.00559800966781225	PDZD3
Recoverec	40	1.67740794887934e-07	0.38412665309307¢	0.745	0.338	0.00562569077895152	APOA1
Recoverec	40	1.72491942692967e-07	0.41331309888256¢	0.182	0.035	0.00578503477403673	SLC52A1
Recoverec	40	1.72509603942081e-07	0.69626792410382¢	0.618	0.284	0.00578562709700953	SLC12A2
Recoverec	40	1.78898037909443e-07	0.40094729345287¢	0.6	0.244	0.00599988239540692	USP24
Recoverec	40	1.7898343122139e-07	0.63109693151396¢	0.564	0.231	0.00600274631630298	CUL5
Recoverec	40	1.86313583411672e-07	0.44679736846363¢	0.273	0.073	0.00624858496046066	NF2
Recoverec	40	2.01757042806589e-07	0.33910201865066¢	0.2	0.042	0.00676652770164739	SEMA5A
Recoverec	40	2.02333869235202e-07	0.59285237304461¢	0.691	0.315	0.0067858733064102	FARS2
Recoverec	40	2.08058390872241e-07	0.43684502076372¢	0.455	0.161	0.00697786231307323	CATSPERB
Recoverec	40	2.13133705241655e-07	0.65666601032074¢	0.527	0.205	0.00714807820639464	NUDT4
Recoverec	40	2.21889882958603e-07	0.85264877655493¢	0.382	0.127	0.00744174289466562	GAB2
Recoverec	40	2.22934219309009e-07	0.42171156295265¢	0.255	0.064	0.00747676784718553	SLC26A3
Recoverec	40	2.33953828517508e-07	0.41562406637354¢	0.327	0.097	0.00784634350082019	CNNM2
Recoverec	40	2.62308539377991e-07	0.41941827166650¢	0.236	0.058	0.00879730379365905	OTUD7B
Recoverec	40	2.6367883373082e-07	0.45828858522423¢	0.327	0.099	0.00884326072566423	GAREM1
Recoverec	40	2.86614595324883e-07	0.74341078623750¢	0.491	0.202	0.00961248029800591	RSU1
Recoverec	40	3.04976025868354e-07	0.39818457298468¢	0.455	0.159	0.0102282859555728	MBOAT1
Recoverec	40	3.0599739838343e-07	0.28371713626303¢	0.236	0.057	0.0102625407469835	LINC01135
Recoverec	40	3.06964346599505e-07	0.55910471827165¢	0.582	0.253	0.0102949702562542	UBE2E1
Recoverec	40	3.07958759299652e-07	0.87739312644323¢	0.909	0.659	0.0103283208693917	MGAT5
Recoverec	40	3.0857235706916e-07	0.58498392640548	0.327	0.104	0.0103488997113855	EFL1
Recoverec	40	3.13652270455971e-07	0.25129464086390¢	0.182	0.036	0.0105192698465524	HTR4
Recoverec	40	3.21877950226884e-07	0.46879072747241¢	0.364	0.12	0.0107951426947092	AC109587.1
Recoverec	40	3.23830321020144e-07	0.69398456073496¢	0.327	0.102	0.0108606213063736	AC137810.1
Recoverec	40	3.25997725740746e-07	0.34939772863588¢	0.2	0.043	0.0109333117258931	GNAO1

Recoverec	40	3.26637817707502e-07	0.600554857116987	0.273	0.077	0.0109547791302742	TNFRSF10D
Recoverec	40	3.34894756739651e-07	0.29766325625346?	0.364	0.114	0.0112317003515344	AC079793.1
Recoverec	40	3.3611798688067e-07	0.58107821794409?	0.4	0.142	0.0112727250440039	BMPR1A
Recoverec	40	3.39437230137643e-07	0.64896559677653?	0.564	0.238	0.0113840458243563	CD58
Recoverec	40	3.71224516406742e-07	0.30649114488542?	0.309	0.089	0.0124501278312493	PIEZO1
Recoverec	40	3.71334170529598e-07	0.39089092011713?	0.255	0.066	0.0124538054112217	SPINK1
Recoverec	40	3.74294169851311e-07	0.85179440331317	1	0.747	0.0125530778684733	NEAT1
Recoverec	40	3.82166008925582e-07	0.55803705807187?	0.491	0.188	0.0128170836073462	MFHAS1
Recoverec	40	4.00979384679512e-07	0.35109179447592?	0.182	0.038	0.0134480466033815	ADTRP
Recoverec	40	4.05587001186452e-07	0.76300791846284?	0.655	0.316	0.0136025768457912	NFATC3
Recoverec	40	4.26905103047367e-07	0.31849296790750?	0.309	0.09	0.0143175433460026	RARS
Recoverec	40	4.27843426425165e-07	0.55333807912391?	0.345	0.109	0.0143490128354472	AL035530.2
Recoverec	40	4.33304941211472e-07	0.56875260292662?	0.309	0.095	0.0145321811183503	COLQ
Recoverec	40	4.38846921020006e-07	0.47797265770904?	0.545	0.22	0.014718048037169	PIK3R5
Recoverec	40	4.56568203793234e-07	0.56158881008239?	0.309	0.096	0.0153123844188175	HSD17B4
Recoverec	40	4.6294717894634e-07	0.46163793686117?	0.418	0.151	0.0155263224875023	EHD1
Recoverec	40	4.6527778919047e-07	0.41309266911741?	0.673	0.3	0.01560448649387	RNF111
Recoverec	40	4.78947791962624e-07	0.62066904684623?	0.927	0.619	0.0160629510468425	PCNX1
Recoverec	40	5.13665860997252e-07	0.37527788853891?	0.255	0.068	0.0172273256461258	ERMP1
Recoverec	40	5.16422602572734e-07	0.53246464557320?	0.6	0.253	0.0173197812450843	MAGI3
Recoverec	40	5.2723987619694e-07	0.44175550152025?	0.364	0.122	0.017682570967893	IBTK
Recoverec	40	5.40639696757671e-07	0.26214863400954?	0.164	0.031	0.0181319741498588	HIST1H2BE
Recoverec	40	5.79589755659574e-07	0.33048289606899?	0.255	0.067	0.0194382812253108	A1CF
Recoverec	40	5.99522840239362e-07	0.64965222303774?	0.727	0.392	0.0201067970159477	EGR1
Recoverec	40	5.99877309795954e-07	0.27068938228179	0.2	0.045	0.0201186852159367	WNK2
Recoverec	40	6.00178976327037e-07	0.35444581127907?	0.164	0.032	0.0201288025080562	AC008735.2
Recoverec	40	6.02305782985491e-07	0.59648882670739?	0.564	0.243	0.0202001313497674	RPRD2
Recoverec	40	6.22504514042644e-07	0.49030117723502?	0.473	0.185	0.0208775563919622	MAPK14
Recoverec	40	6.48379293640222e-07	0.35360415366962?	0.236	0.061	0.0217453447501058	MYRFL
Recoverec	40	6.54799647395565e-07	0.61966813283914	0.473	0.187	0.0219606705743525	FAM168A
Recoverec	40	6.82207780622655e-07	0.37622663931164?	0.455	0.167	0.0228798845465226	TRANK1

Recoverec	40	6.87297451270436e-07	0.30815656967759٤	0.127	0.02	0.0230505819207079	AC009226.1
Recoverec	40	6.88775124739958e-07	0.343119105879191	0.273	0.075	0.0231001401335287	FARSB
Recoverec	40	7.01264198300828e-07	0.26149345565318З	0.4	0.132	0.0235189986826132	PTK2
Recoverec	40	7.25424503333366e-07	0.56947260715314З	0.564	0.246	0.0243292869927944	MAPKAP1
Recoverec	40	7.55963237441538e-07	0.32513562745004٤	0.182	0.039	0.0253534950573143	PARP16
Recoverec	40	7.91475135858959e-07	0.49010662771575٤	0.545	0.227	0.0265444931064378	ZNF33A
Recoverec	40	8.06055764239228e-07	0.31854479700123٦	0.364	0.12	0.0270334982210552	TCHP
Recoverec	40	8.18718664825371e-07	0.47332799970002᷊	0.327	0.104	0.0274581865809133	VPS45
Recoverec	40	8.74454391622146e-07	0.56752396552185٤	0.527	0.217	0.0293274513862235	SPON1
Recoverec	40	8.77430290487671e-07	0.46055137526359٤	0.309	0.097	0.0294272570823755	MYO1A
Recoverec	40	8.95153296145605e-07	0.41310276005177٤	0.255	0.071	0.0300216512461313	KIAA1671
Recoverec	40	9.47598912066449e-07	0.48927825419407٤	0.564	0.245	0.0317805723128846	SI
Recoverec	40	9.80965699922032e-07	0.37346507427428٦	0.455	0.17	0.0328996276439851	AGPAT3
Recoverec	40	1.01501394635543e-06	0.25815203271530٤	0.164	0.032	0.0340415377328683	SRSF12
Recoverec	40	1.03901216470517e-06	0.50554241744342٤	0.745	0.376	0.0348463899798822	STIM1
Recoverec	40	1.04478902350904e-06	0.281341995741191	0.236	0.061	0.035040134270446	NUP85
Recoverec	40	1.05552234380031e-06	0.402756017950311	0.236	0.062	0.0354001083663748	MYO5B
Recoverec	40	1.05811333983491e-06	0.45212285797780٤	0.527	0.218	0.0354870051913833	FABP2
Recoverec	40	1.05834190246412e-06	0.31345073209448٦	0.2	0.046	0.0354946707248418	MGAM2
Recoverec	40	1.06610673941625e-06	0.45223209356315٦	0.509	0.207	0.0357550878265422	PPP6R2
Recoverec	40	1.10662546159716e-06	0.38011543865245	0.2	0.048	0.0371140047310455	TAF4
Recoverec	40	1.11801027418019e-06	0.40398802794338٤	0.364	0.121	0.0374958285754551	DAB1
Recoverec	40	1.15735648141914e-06	0.38335621371321٤	0.691	0.312	0.038815421673835	PIBF1
Recoverec	40	1.17578530010034e-06	0.25921216542325	0.2	0.046	0.0394334873947651	PIK3C2G
Recoverec	40	1.19199683454367e-06	0.39454226255381	0.582	0.255	0.0399771898369257	PTK2B
Recoverec	40	1.19716242254373e-06	0.271150278066751	0.436	0.156	0.0401504333272717	GIT2
Recoverec	40	1.20383750584373e-06	0.59285932765387٤	0.364	0.128	0.0403743022709871	CCNYL1
Recoverec	40	1.22209501940815e-06	0.32842088593685	0.273	0.077	0.0409866227609105	TULP2
Recoverec	40	1.24548025266558e-06	0.42050888614962٤	0.473	0.181	0.0417709167138981	ZSWIM5
Recoverec	40	1.2509137507426e-06	0.43768548634404З	0.236	0.063	0.0419531453724053	COL10A1
Recoverec	40	1.26096837734441e-06	0.30845562331494	0.309	0.095	0.0422903574393769	PRKD2

Recoverec	40	1.2650328652725e-06	0.29326472986208	0.236	0.062	0.0424266722355092	POF1B
Recoverec	40	1.27656395239599e-06	0.60439230873476	0.582	0.271	0.0428134018354568	ZBTB10
Recoverec	40	1.2769992805865e-06	0.32126535429477	0.182	0.041	0.0428280018723101	CUL4B
Recoverec	40	1.3038554919934e-06	0.44330656502528	0.418	0.152	0.0437287054904746	PLB1
Recoverec	40	1.3275596967001e-06	0.56541654410235	0.6	0.295	0.0445236971079278	GGNBP2
Recoverec	40	1.35844368023696e-06	0.31064382023464	0.8	0.366	0.0455594841477873	RNF38
Recoverec	40	1.37952020380548e-06	0.28824816277905	0.145	0.027	0.0462663485952283	GIN1
Recoverec	40	1.40848609102668e-06	0.39702074263198	0.236	0.064	0.0472378065208526	TMEM45B
Recoverec	40	1.42697503862052e-06	0.36020872361664	0.618	0.266	0.047857888845255	AZIN1
Recoverec	40	1.48461396374598e-06	0.67354814756347	0.818	0.479	0.0497909831161126	SMARCA2
Recoverec	40	1.48845184679686e-06	0.41335794355428	0.618	0.275	0.0499196980378731	SORL1
Recoverec	47	1.28600217390627e-24	0.32252734911460	0.171	0.005	4.31299409084684e-20	LCT
Recoverec	47	1.00028991688836e-19	0.34564249225513	0.171	0.008	3.35477232326019e-15	EPDR1
Recoverec	47	9.51883584985483e-16	0.83754510057892	0.659	0.159	3.19242716732431e-11	RBP2
Recoverec	47	1.75880206534524e-13	0.70474586229462	0.512	0.115	5.89867036675486e-09	SLC5A1
Recoverec	47	1.01597264343713e-12	1.02160510503185	0.488	0.111	3.40736905155944e-08	TTC39C-AS1
Recoverec	47	1.86195987749487e-12	0.91882838914631	0.659	0.191	6.24464103714229e-08	APOB
Recoverec	47	4.22730529021659e-12	0.41434220475945	0.244	0.031	1.41775364823284e-07	AC079209.2
Recoverec	47	4.16777342030878e-11	0.47073020336484	0.293	0.048	1.39778784970316e-06	RORC
Recoverec	47	6.0355758741484e-11	0.49131469923532	0.22	0.028	2.02421143667189e-06	DLEU7
Recoverec	47	1.83227783222641e-10	0.35455928733423	0.195	0.023	6.14509339372093e-06	GPRASP1
Recoverec	47	4.91819860542903e-10	0.40600726237651	0.195	0.025	1.64946544828879e-05	FP671120.1
Recoverec	47	6.93827081228661e-10	0.55197408535947	0.341	0.07	2.32695726502468e-05	AL391839.1
Recoverec	47	8.36395972561076e-10	0.66643809516156	0.78	0.305	2.80510481277534e-05	APOA4
Recoverec	47	1.59535076158311e-09	1.04490485699329	0.488	0.141	5.35048738419743e-05	AL356272.1
Recoverec	47	4.84241330990904e-09	0.38438940010790	0.244	0.042	0.000162404857587729	IDH1
Recoverec	47	6.53989301112828e-09	0.54168831069639	0.366	0.088	0.00021933493180722	TUBGCP3
Recoverec	47	1.57070300700361e-08	0.72456365783240	0.463	0.135	0.000526782374488869	AL157402.2
Recoverec	47	1.96211518707685e-08	0.56470849800740	0.268	0.053	0.000658054191441835	AC022973.3
Recoverec	47	2.85707111724819e-08	1.01330259085073	0.756	0.371	0.000958204511302696	GPHN
Recoverec	47	3.92648915416964e-08	0.30238632610627	0.268	0.054	0.00131686593252541	CASZ1

Recoverec	47	4.10389282485497e-08	0.63898661765074	0.805	0.344	0.00137636357559986	TANC2
Recoverec	47	7.78841558488349e-08	0.51446507020372	0.22	0.04	0.00261207881885822	LATS2
Recoverec	47	7.90247586604801e-08	0.44509169865886	0.293	0.065	0.00265033235595518	RHBDD2
Recoverec	47	8.59331933904192e-08	0.63930286482246	0.195	0.033	0.00288202743992788	AC018511.2
Recoverec	47	9.6284874695034e-08	0.51064425209144	0.537	0.181	0.00322920212752205	SBNO1
Recoverec	47	9.97239216508481e-08	0.56849063816029	0.415	0.122	0.00334454088432614	DAB1
Recoverec	47	1.037766296092e-07	0.90172797532766	0.78	0.375	0.00348046060383335	RUNX2
Recoverec	47	1.22615052110177e-07	0.38889655339437	0.244	0.049	0.00411226361767112	UGT2A3
Recoverec	47	1.63561107709897e-07	0.69160372320396	0.463	0.151	0.00548551243037454	PPM1D
Recoverec	47	1.94407454909971e-07	0.70523595145502	0.707	0.308	0.00652003722277062	NFKBID
Recoverec	47	2.07882715902095e-07	0.28409637012559	0.195	0.033	0.00697197052592445	AC084200.1
Recoverec	47	2.17816107392302e-07	0.48168432929874	0.268	0.06	0.00730511660972304	MALL
Recoverec	47	2.1923596850685e-07	0.78458267078384	0.488	0.162	0.00735273591178273	PTPN13
Recoverec	47	2.92655368177465e-07	0.39318145459890	0.171	0.027	0.00981507573793581	AL138720.1
Recoverec	47	2.971638077757e-07	0.47602949148559	0.195	0.035	0.00996627978518142	NGEF
Recoverec	47	3.28223750455019e-07	1.29033439973645	0.976	0.656	0.0110079681427604	RORA-AS1
Recoverec	47	3.37198552368098e-07	0.51972654640319	0.366	0.103	0.0113089650493213	ZNF512
Recoverec	47	3.90393431354399e-07	0.51149097541142	0.317	0.083	0.0130930149007638	MIR29B2CHG
Recoverec	47	4.09340908376735e-07	0.51644499682180	0.293	0.071	0.0137284753851389	TMLHE
Recoverec	47	4.83619795391027e-07	0.78691921189187	0.707	0.319	0.0162196406978243	FARS2
Recoverec	47	5.30206194626773e-07	0.81662130467107	0.805	0.397	0.0177820553553927	FOXO3
Recoverec	47	5.82167061113496e-07	0.59214241372755	0.293	0.075	0.0195247188956244	AL031428.1
Recoverec	47	6.72702024050257e-07	0.36329330714599	0.268	0.063	0.0225610804825975	MYO5B
Recoverec	47	6.81490440833734e-07	1.00408243736156	1	0.809	0.0228558264046818	RORA
Recoverec	47	7.04444321551676e-07	0.72874848175082	0.439	0.15	0.0236256536562001	MCF2L2
Recoverec	47	7.73440836641182e-07	0.50238833964442	0.22	0.046	0.025939658779272	FBXO25
Recoverec	47	8.07489681110636e-07	0.51638277507361	0.293	0.076	0.0270815889250885	B3GNT5
Recoverec	47	8.26953171045325e-07	0.70481351494854	0.683	0.298	0.0277343554505181	PRKAR2A
Recoverec	47	8.29402328713231e-07	0.71718610685345	0.341	0.098	0.0278164953003844	AP003086.2
Recoverec	47	9.35923284799125e-07	0.43675455730274	0.22	0.047	0.031388995125593	RAD52
Recoverec	47	9.42340564371618e-07	0.53292159418762	0.439	0.146	0.0316042178478953	SDCCAG8

Recoverec	47	1.0138783677889e-06	0.63277892756588€	0.366	0.112	0.0340034526989042	ELOVL6
Recoverec	47	1.0995804714594e-06	0.71681338874443£	0.927	0.634	0.0368777298518052	AKT3
Recoverec	47	1.1033094854668e-06	0.85646340853359	0.659	0.312	0.0370027935235857	SCFD2
Recoverec	47	1.1259880712423e-06	0.77697703313933€	0.805	0.387	0.0377633879333242	PHIP
Recoverec	47	1.19563795140344e-06	0.96967041779895€	0.512	0.185	0.0400993056141687	SLC4A10
Recoverec	47	1.25938571437803e-06	0.83346220183963Ž	0.268	0.068	0.0422372780888102	PLCG1-AS1
Recoverec	47	1.35783230109406e-06	0.31249264337739:	0.146	0.023	0.0455389797140924	AF127577.4
Recoverec	47	1.38331406153944e-06	0.67304642028676Ž	0.415	0.143	0.0463935869959097	ATG2B
Recoverec	47	1.47307912658945e-06	0.39064932222041€	0.244	0.056	0.0494041277475571	AF127577.2
Recoverec	98	3.08234086142582e-44	1.11487195068939	0.904	0.888	1.03375547810499e-39	TMSB4X
Recoverec	98	2.1209672965278e-40	0.91957188654956	0.906	0.87	7.11330011909494e-36	RPL39
Recoverec	98	4.95808188114742e-29	0.75402056838719£	0.927	0.947	1.66284150129922e-24	JUND
Recoverec	98	5.65725253728299e-28	0.81715362982021:	0.876	0.83	1.89732935595397e-23	RPS28
Recoverec	98	2.08015719103476e-27	0.65564581161738€	0.965	0.979	6.97643118729239e-23	B2M
Recoverec	98	2.92248492377495e-23	0.91785246225206	0.772	0.758	9.80142993735642e-19	RPS29
Recoverec	98	8.59903871319045e-23	0.72767026662050£	0.853	0.837	2.88394560362981e-18	RPLP2
Recoverec	98	1.88934564953353e-22	0.64027740068589	0.929	0.933	6.33648743940556e-18	RPLP1
Recoverec	98	1.73578019444564e-20	0.61826668432662£	0.902	0.88	5.82145961613178e-16	RPL41
Recoverec	98	2.44852530021903e-20	0.74242026385253€	0.845	0.85	8.21186415187457e-16	RPS27
Recoverec	98	1.10315638847558e-19	0.69581325554505	0.849	0.816	3.69976589566939e-15	RPL34
Recoverec	98	6.67284218405178e-19	0.67091595634414€	0.847	0.825	2.23793781168729e-14	RPS18
Recoverec	98	1.56251979783715e-18	0.64023310866174€	0.872	0.876	5.24037889798625e-14	RPS12
Recoverec	98	4.40303917223681e-18	0.69673722450208	0.815	0.8	1.47669127758478e-13	RPL37
Recoverec	98	1.13938278377252e-17	0.76539006992274€	0.786	0.785	3.82126198021629e-13	RPS11
Recoverec	98	6.58199483916327e-17	0.63876319456754	0.825	0.792	2.20746942915858e-12	RPS27A
Recoverec	98	3.66888492087499e-16	0.58692384075458Ž	0.849	0.8	1.23047062476305e-11	RPS15A
Recoverec	98	2.18589235803438e-15	0.73643457828155	0.764	0.757	7.33104579037569e-11	RPL27A
Recoverec	98	2.30820666232873e-15	0.76506624887314	0.723	0.718	7.74126350411809e-11	RPL37A
Recoverec	98	3.68760336527794e-15	0.64910898577981Ž	0.802	0.834	1.23674841664691e-10	TMSB10
Recoverec	98	7.97187156125858e-15	0.56904220774004:	0.851	0.837	2.6736062842149e-10	RPL28
Recoverec	98	9.50820347133099e-15	0.56093007218479€	0.833	0.781	3.18886128021499e-10	RPL30

Recoverec	98	2.81679374621381e-14	0.56037109138094!	0.825	0.78	9.44696286605188e-10	RPS23
Recoverec	98	4.5970633754907e-14	0.94324834299435!	0.615	0.586	1.54176311487207e-09	ATP5F1E
Recoverec	98	7.10492262143747e-14	0.60337785995292	0.788	0.796	2.3828489487777e-09	RPS20
Recoverec	98	8.4361829470854e-14	0.54172213162919!	0.851	0.842	2.8293270367935e-09	RPS19
Recoverec	98	1.77825910275593e-13	0.58120120325206	0.792	0.765	5.96392537882284e-09	RPS25
Recoverec	98	4.85982320939429e-13	1.0100062950621	0.564	0.505	1.62988750796666e-08	CD52
Recoverec	98	1.89004543999142e-12	0.59040312558973	0.77	0.74	6.33883439664321e-08	RPS13
Recoverec	98	2.03407114868295e-12	0.82486488296682!	0.65	0.64	6.82186781845289e-08	RPL27
Recoverec	98	2.1159452256516e-12	0.65046263927775	0.78	0.826	7.09645709779034e-08	RPL9
Recoverec	98	2.46132428736596e-12	0.55526555124581	0.802	0.754	8.25478939496795e-08	RPS15
Recoverec	98	3.85415338634357e-11	0.51931003501162!	0.819	0.864	1.29260596271191e-06	ACTB
Recoverec	98	4.04725059295578e-11	0.63966406519194	0.713	0.718	1.35736690386551e-06	UBA52
Recoverec	98	4.39073586792388e-11	0.53875039914048	0.794	0.801	1.47256499538431e-06	RPS16
Recoverec	98	7.38832636493612e-11	0.46736334024249!	0.821	0.796	2.47789689627227e-06	RPS3A
Recoverec	98	3.36708947500544e-10	0.58173927777935	0.733	0.738	1.12925446812732e-05	RPL36
Recoverec	98	5.32100890915835e-10	0.52596742003804!	0.77	0.734	1.78455996795353e-05	RPL15
Recoverec	98	5.51396729904485e-10	0.47115254863037	0.823	0.81	1.84927435275366e-05	RPL13A
Recoverec	98	5.70952463112739e-10	0.3874606452915	0.896	0.916	1.9148603707875e-05	PTMA
Recoverec	98	5.787729099316e-10	0.54691137228927	0.747	0.784	1.9410885853286e-05	RPL24
Recoverec	98	8.90654893289194e-10	0.78329446998410	0.64	0.653	2.9870783811133e-05	RPL23
Recoverec	98	1.22866082927823e-09	0.36134653249891	0.874	0.869	4.12068268923334e-05	RPL10
Recoverec	98	6.17712524494305e-09	0.43369158569605	0.813	0.833	0.0002071684264649	RPL12
Recoverec	98	6.55712501890169e-09	0.61926382207232!	0.701	0.72	0.000219912858883925	RPL35
Recoverec	98	1.15749388411484e-08	0.83397154184336!	0.161	0.067	0.000388200298854434	C1orf56
Recoverec	98	1.68363135003703e-08	0.39815808636608	0.835	0.83	0.000564656282175418	RPS14
Recoverec	98	1.86081157322698e-08	0.42230591873975	0.884	0.944	0.000624078985428863	KLF6
Recoverec	98	2.16528872517772e-08	0.66413968717643	0.611	0.654	0.000726194532650104	RPL38
Recoverec	98	2.59150184319894e-08	0.35191188915735!	0.821	0.814	0.000869137888172062	RPL11
Recoverec	98	2.69178261687283e-08	0.47604431759333	0.756	0.752	0.00090277005404681	RPS7
Recoverec	98	4.27860067930961e-08	0.47985270748374!	0.719	0.733	0.00143495709582686	RPL35A
Recoverec	98	6.71293287500209e-08	0.68324609580452!	0.621	0.629	0.0022513834276182	RPS21

Recoverec	98	1.02348066607654e-07	0.41671125660562	0.782	0.785	0.00343254945788749	RPL18
Recoverec	98	1.19294971336071e-07	0.63098618793907	0.627	0.692	0.00400091474866915	SERF2
Recoverec	98	3.54750656721311e-07	0.88783137801638	0.517	0.514	0.0118976275251193	CD3D
Recoverec	98	3.7215131962659e-07	0.46951155349930	0.684	0.676	0.0124812109576366	RPL22
Recoverec	98	3.90199540391822e-07	0.45196059503879	0.701	0.709	0.0130865121856609	RPL23A
Recoverec	98	4.00374605733076e-07	0.37459840508482	0.811	0.834	0.0134277635270759	RPS6
Recoverec	98	9.37257099657309e-07	0.48186448877061	0.701	0.714	0.0314337286083068	FAU
Recoverec	98	9.69289035428042e-07	0.29946334567386	0.817	0.814	0.0325080156701857	RPS8
Recoverec	121	5.9607874170414e-51	0.87121500262736	0.297	0.005	1.99912888392734e-46	TFPI2
Recoverec	121	6.99497891939194e-22	0.50328032525489	0.162	0.005	2.34597602998567e-17	ANO2
Recoverec	121	4.31872242986622e-19	0.79843509698107	0.459	0.063	1.44841312852853e-14	NRG1
Recoverec	121	2.94142630677519e-15	0.40740144081965	0.162	0.009	9.86495554766263e-11	AL158068.2
Recoverec	121	4.76390186941489e-15	0.26836130929844	0.108	0.003	1.59771740896436e-10	GALNT15
Recoverec	121	2.20574188888684e-13	0.26244093281068	0.108	0.004	7.39761714694869e-09	TPO
Recoverec	121	1.25851807412243e-12	0.46941273176062	0.243	0.027	4.22081791699181e-08	EXTL3
Recoverec	121	1.81973023691996e-11	1.23968592487705	0.811	0.332	6.10301126858218e-07	LDLR
Recoverec	121	1.97198084417272e-11	0.44131445801885	0.27	0.037	6.61362935518648e-07	LDB2
Recoverec	121	2.72950699217926e-11	0.93881246791825	0.649	0.19	9.1542205503708e-07	KLF9
Recoverec	121	3.93292258457395e-11	0.87337322550145	0.486	0.118	1.31902357641441e-06	VSIR
Recoverec	121	7.95099883143937e-11	1.04963044070971	0.784	0.283	2.66660598808814e-06	IGLC2
Recoverec	121	2.68209661209723e-10	0.48196604960276	0.189	0.021	8.9952156176517e-06	COL5A2
Recoverec	121	3.08848090782458e-10	0.99632932872844	0.595	0.165	1.03581472686621e-05	A2M
Recoverec	121	4.91491310132295e-10	0.36752680643029	0.108	0.007	1.64836355592169e-05	CDH11
Recoverec	121	9.25742392131059e-10	0.63520043110133	0.27	0.044	3.10475483472915e-05	SLC25A43
Recoverec	121	1.11192572617972e-09	0.41141912908988	0.27	0.043	3.72917650046153e-05	PID1
Recoverec	121	1.16588402396218e-09	0.41407594380954	0.216	0.029	3.91014183956436e-05	COL6A3
Recoverec	121	1.8194664286745e-09	1.47975705379036	0.946	0.621	6.10212650848854e-05	RASGEF1B
Recoverec	121	2.56133401538004e-09	0.82531234206465	0.514	0.145	8.59020202078159e-05	LRIG1
Recoverec	121	2.63156883782221e-09	0.52876022928999	0.324	0.063	8.82575556828813e-05	IGFBP4
Recoverec	121	3.41866832288388e-09	0.26060692617070	0.108	0.007	0.000114655298212879	TM4SF4
Recoverec	121	4.17105268059998e-09	0.68505306903680	0.378	0.087	0.000139888764801962	SAP30L-AS1

Recoverec	121	4.30286078023605e-09	0.30638184127222	0.162	0.017	0.000144309344847557	TPM2
Recoverec	121	6.56853493801546e-09	0.25394137222929	0.135	0.012	0.000220295524751163	BCL2L2
Recoverec	121	7.33266653785408e-09	0.78123676355160	0.405	0.103	0.00024592297034655	SH2B3
Recoverec	121	7.60523920752534e-09	0.40960947883585	0.27	0.047	0.000255064512541985	RBPMS
Recoverec	121	1.38917411239894e-08	0.38848787173608	0.216	0.032	0.000465901213816356	CLMP
Recoverec	121	1.61153082192503e-08	0.39173722488182	0.162	0.019	0.000540475207057216	SLCO2A1
Recoverec	121	2.07867324986855e-08	0.39387008626463	0.243	0.042	0.000697145434540913	SOX5
Recoverec	121	2.07928418531165e-08	0.74267505581072	0.405	0.103	0.00069735033006982	SMAD7
Recoverec	121	2.59064307405199e-08	0.48962571799932	0.459	0.13	0.000868849874175555	DDX39B
Recoverec	121	3.31075781468716e-08	0.40056321829378	0.162	0.02	0.00111036195588978	RCAN2
Recoverec	121	3.61784575561269e-08	0.68153693460088	0.378	0.091	0.00121335310951738	SPARCL1
Recoverec	121	4.32909433907061e-08	0.28440342405593	0.162	0.02	0.0014518916594375	CTGF
Recoverec	121	4.37761891122725e-08	0.36320880192372	0.243	0.043	0.0014681658304474	COL3A1
Recoverec	121	5.92492739143994e-08	0.35152453334624	0.189	0.027	0.00198710214854113	RGS5
Recoverec	121	7.48635133776381e-08	1.00310819459416	0.757	0.374	0.00251077251165923	RAPGEF1
Recoverec	121	9.36615592457887e-08	0.70746113820156	0.649	0.258	0.00314122137398526	PTK2B
Recoverec	121	9.46000578940261e-08	0.60068639018033	0.568	0.19	0.00317269674164985	MYO1F
Recoverec	121	9.73282862917033e-08	0.70155125269331	0.486	0.147	0.00326419606565115	IGHM
Recoverec	121	1.01403334111239e-07	0.84508806249280	0.811	0.421	0.00340086501942273	MYH9
Recoverec	121	1.16670215523648e-07	0.74696842844205	0.514	0.166	0.00391288568823211	HDAC4
Recoverec	121	1.54780320879709e-07	0.66809113483191	0.514	0.172	0.00519102240166369	DNM2
Recoverec	121	1.68983244423877e-07	0.62750229462921	0.432	0.125	0.00566736005148798	IRF4
Recoverec	121	1.87056024227505e-07	1.01935988999862	0.811	0.411	0.00627348494054207	ZNF407
Recoverec	121	1.99340988493624e-07	0.47639048471108	0.243	0.047	0.00668549807209916	DLC1
Recoverec	121	2.10641170561772e-07	0.75416836569963	0.514	0.179	0.00706448357830072	ARHGAP35
Recoverec	121	2.14915557423727e-07	0.25242286469698	0.108	0.01	0.00720783796487694	FGD5
Recoverec	121	2.21122587210455e-07	0.75607692558023	0.622	0.246	0.00741600932986422	RAB3GAP2
Recoverec	121	2.36622866471526e-07	0.29106795546695	0.135	0.016	0.00793585769572205	FGF14
Recoverec	121	2.44834399461942e-07	0.40352928985372	0.243	0.047	0.00821125608915461	TPST1
Recoverec	121	2.82461660322809e-07	0.25021130940775	0.135	0.016	0.00947319916390637	VWF
Recoverec	121	2.89658801986883e-07	0.64803201520727	0.459	0.147	0.0097145769010361	ELL

Recoverec	121	3.17143213747552e-07	0.82428006787496	0.973	0.732	0.0106363491026654	UTRN
Recoverec	121	3.36853929417877e-07	0.79569427446235	0.378	0.106	0.0112974070848168	ABCC4
Recoverec	121	3.38433411098392e-07	0.60755166731364	0.216	0.04	0.0113503797414179	SLC13A1
Recoverec	121	3.57028183229268e-07	0.37295117472076	0.243	0.048	0.0119740112091432	KLF7
Recoverec	121	3.77611838081049e-07	0.80325676759605	0.946	0.6	0.0126643458255622	MACF1
Recoverec	121	4.10753582163061e-07	0.55567211051173	0.189	0.032	0.0137758536385847	NNMT
Recoverec	121	4.300424739564e-07	0.66736386752988	0.297	0.071	0.0144227644915497	NCKAP5
Recoverec	121	4.41077350797343e-07	0.26332989538556	0.135	0.017	0.0147928521910413	AC093110.1
Recoverec	121	4.55023243811789e-07	0.49695226827580	0.162	0.024	0.0152605695509598	COL1A1
Recoverec	121	5.3777025217092e-07	0.41557425889945	0.108	0.011	0.0180357387173083	SEMA3E
Recoverec	121	5.40043578893884e-07	0.84479493124645	0.973	0.608	0.0181119815489431	COG5
Recoverec	121	5.50859797871917e-07	0.40217682976939	0.108	0.011	0.0184747359010284	NR2F2
Recoverec	121	6.60720775510261e-07	0.52950419423401	0.622	0.234	0.0221592533690631	TMEM117
Recoverec	121	6.64350017927499e-07	0.66445818556912	0.784	0.343	0.0222809709012525	SLFN12L
Recoverec	121	6.8211677866605e-07	0.33223408427099	0.162	0.024	0.022876832522902	SLC27A1
Recoverec	121	7.11051191229844e-07	0.39049173898672	0.27	0.06	0.0238472348514665	IGFBP7
Recoverec	121	7.26082051312613e-07	0.44711823396910	0.135	0.017	0.0243513398369224	MFGE8
Recoverec	121	7.86723876814128e-07	0.37376733973307	0.243	0.051	0.0263851453805922	AC025180.1
Recoverec	121	8.04972102424814e-07	0.37444679482398	0.27	0.061	0.0269971543711234	TMEM140
Recoverec	121	8.48693590927016e-07	0.83783827207923	0.703	0.309	0.0284634856525103	NFKBID
Recoverec	121	8.54354366014705e-07	0.30624595997781	0.162	0.024	0.0286533367274012	GNG11
Recoverec	121	8.71780154904828e-07	0.28953304777605	0.135	0.017	0.0292377628351981	AC016910.1
Recoverec	121	8.9574308380291e-07	0.65234412109402	0.595	0.227	0.030041431544582	EXOC2
Recoverec	121	1.07963704147082e-06	0.66816213734847	0.595	0.235	0.0362088670968483	GALNT10
Recoverec	121	1.09463819717616e-06	0.58488554378006	0.297	0.075	0.0367119758568939	TG
Recoverec	121	1.09856846974042e-06	0.35131429615800	0.297	0.073	0.0368437893381544	TNFRSF1A
Recoverec	121	1.09959618415096e-06	0.47419703910775	0.378	0.109	0.0368782568240548	GTPBP4
Recoverec	121	1.1292166030139e-06	0.43602954525117	0.27	0.062	0.0378716664318803	RDH13
Recoverec	121	1.24139138520721e-06	0.87221815413892	0.919	0.53	0.0416337842770793	ANKRD11
Recoverec	121	1.24545217613914e-06	0.46457367439799	0.297	0.075	0.0417699750833546	RAVER2
Recoverec	121	1.38423634555408e-06	0.5455454095577	0.351	0.098	0.0464245185571927	AC136475.3

Recoverec	121	1.41632450108305e-06	0.77422318639624	0.703	0.345	0.0475006911173232	MAN1A2
Recoverec	121	1.43557350395507e-06	0.66200254182860	0.486	0.174	0.048146264175645	AGPAT3
Recoverec	121	1.44656257846901e-06	0.82192933952177	0.73	0.383	0.0485148157566937	STK24
Recoverec	183	2.94055192880618e-25	0.41582949455135	0.133	0.001	9.86202305883016e-21	ARAP1-AS2
Recoverec	183	3.51526004688093e-25	0.3442710609429	0.133	0.001	1.17894791452293e-20	AC107982.3
Recoverec	183	1.07616268963765e-18	0.32843464202572	0.2	0.005	3.60923442850677e-14	CREB3L1
Recoverec	183	2.71720804281085e-18	0.71498112014924	0.267	0.01	9.11297233397901e-14	AC134669.1
Recoverec	183	3.24889707043399e-16	1.42110428741127	0.467	0.038	1.08961509948215e-11	CRTC3-AS1
Recoverec	183	2.47731998857223e-15	0.33758980065516	0.2	0.007	8.30843577767353e-11	AL138930.1
Recoverec	183	5.61705540127239e-13	0.32558851576681	0.133	0.003	1.88384804047873e-08	IGF1
Recoverec	183	5.61705540127239e-13	0.26296056667202	0.133	0.003	1.88384804047873e-08	AL596202.1
Recoverec	183	6.13725527015327e-13	0.26502293882694	0.133	0.003	2.058312672504e-08	LINC02267
Recoverec	183	6.41480721341324e-13	0.25726491922046	0.133	0.003	2.15139804323453e-08	TMEM74B
Recoverec	183	2.18647879074055e-11	0.39286625701885	0.2	0.01	7.33301256838566e-07	AGER
Recoverec	183	3.48987538250785e-11	0.30433074954571	0.133	0.004	1.17043440578548e-06	AC138811.1
Recoverec	183	3.51764436877316e-11	1.02331240583467	0.733	0.129	1.17974756839914e-06	CLSTN1
Recoverec	183	9.57149025367564e-11	0.56970034974923	0.267	0.019	3.21008640127774e-06	IGFBP5
Recoverec	183	3.94844824059983e-10	0.54371959196946	0.2	0.011	1.32423057093237e-05	LINC00923
Recoverec	183	5.59099980840475e-10	0.36879007608775	0.133	0.005	1.87510951574279e-05	LINC01980
Recoverec	183	1.29148154083749e-09	0.43702264331293	0.267	0.021	4.33137079166078e-05	ZSWIM9
Recoverec	183	3.69541546656365e-09	0.76077773529081	0.4	0.05	0.000123936843917612	RBPMS
Recoverec	183	5.20130210263821e-09	0.44218355430172	0.2	0.013	0.00017444126991828	AC006213.3
Recoverec	183	6.7337515686261e-09	0.30192999172190	0.133	0.006	0.000225836560108582	LINC01281
Recoverec	183	7.35410738191847e-09	0.34931196301206	0.133	0.006	0.000246642053374782	PEAK3
Recoverec	183	1.01053864577924e-08	0.83634921094538	0.467	0.07	0.000338914451021442	MCTP1
Recoverec	183	1.1221772384899e-08	0.72285200537440	0.4	0.053	0.000376355802244744	SLC37A3
Recoverec	183	1.32053062781426e-08	0.88173509096220	0.467	0.069	0.000442879561956345	KNTC1
Recoverec	183	1.33678502670897e-08	0.70573213377407	0.467	0.07	0.000448330962257654	NRG1
Recoverec	183	1.68254491703316e-08	0.29500814984711	0.2	0.014	0.00056429191427458	PLEKHH1
Recoverec	183	1.7497152763394e-08	1.27156093919002	0.467	0.074	0.000586819509378707	PIK3C2B
Recoverec	183	2.54281317914696e-08	0.39614924266057	0.267	0.025	0.000852808684022309	AC008764.8

Recoverec	183	3.12903541836652e-08	0.36178064045511:	0.2	0.015	0.00104941589861176	CDCA7L
Recoverec	183	3.78618970286251e-08	0.89379410291059(	0.6	0.116	0.00126981230254603	MBNL3
Recoverec	183	4.25629873160073e-08	0.784601135931104	0.4	0.056	0.00142747746860425	TOX2
Recoverec	183	4.26145114564273e-08	0.93577702885647!	0.6	0.123	0.00142920548522566	YEATS2
Recoverec	183	4.45702171763997e-08	0.49587781577229{	0.133	0.007	0.00149479594366209	PIEZO2
Recoverec	183	4.45702171763997e-08	0.40338357437072:	0.133	0.007	0.00149479594366209	PRRG4
Recoverec	183	4.69874515264702e-08	0.40071252011390:	0.133	0.007	0.00157586514929476	KATNAL2
Recoverec	183	5.08531890528599e-08	0.31523527141576:	0.133	0.007	0.00170551425445482	IPO13
Recoverec	183	5.08531890528599e-08	0.29140861596161:	0.133	0.007	0.00170551425445482	SLC44A5
Recoverec	183	5.22089693573046e-08	0.33689920612390{	0.133	0.007	0.00175098441430528	TP63
Recoverec	183	5.22089693573046e-08	0.27229580719179:	0.133	0.007	0.00175098441430528	AL031963.3
Recoverec	183	7.83245775007416e-08	0.49250679907114!	0.2	0.016	0.00262684968021987	TELO2
Recoverec	183	7.97144605328333e-08	0.44180998383727	0.2	0.016	0.00267346357735016	B4GALNT3
Recoverec	183	8.86635829034015e-08	1.17732657723951	0.733	0.189	0.00297359924341428	RNF144A
Recoverec	183	1.10977739292239e-07	1.12030299232006	0.533	0.111	0.00372197142038312	FBXL4
Recoverec	183	1.58325693206792e-07	0.57869251241141(	0.2	0.016	0.00530992709876939	EPHB6
Recoverec	183	1.70932677319377e-07	0.54898716605834{	0.333	0.043	0.00573274013193726	TMEM236
Recoverec	183	2.00271236871735e-07	0.50169847892218!	0.133	0.007	0.00671669674220425	GALNT8
Recoverec	183	2.10121907358703e-07	0.52296084225143:	0.133	0.007	0.00704706852899618	AP002383.2
Recoverec	183	2.20440047830068e-07	0.3710325382599	0.133	0.007	0.00739311832412482	FYB2
Recoverec	183	2.31246979946484e-07	0.35466723376259!	0.133	0.007	0.00775556121344518	TSPAN18
Recoverec	183	2.36840659196166e-07	0.32895753841960:	0.133	0.007	0.00794316202812103	AL355916.3
Recoverec	183	3.5433154407127e-07	0.77020989610325:	0.467	0.085	0.0118835713250623	ZFYVE9
Recoverec	183	3.84355144603395e-07	0.66966166307394!	0.2	0.017	0.0128905028397087	AC099063.1
Recoverec	183	4.81916147664448e-07	0.87727619164537:	0.467	0.086	0.0161625037603703	STYX
Recoverec	183	6.06076789373483e-07	1.50798359844165	0.267	0.032	0.0203266033620079	AL109930.1
Recoverec	183	6.47658478877035e-07	1.93256588474154	0.933	0.399	0.021721170064578	TOX
Recoverec	183	6.69390242215068e-07	0.68352771633966	0.2	0.018	0.0224500099434089	AC010320.4
Recoverec	183	7.65872125206234e-07	0.27903991093736:	0.333	0.046	0.0256858193351667	COL3A1
Recoverec	183	8.38088028031585e-07	0.41882268386267!	0.133	0.008	0.0281077962841233	PRICKLE2
Recoverec	183	8.9488441591725e-07	0.38800027879673!	0.133	0.008	0.0300126335410327	RAI2

Recoverec	183	9.14625748316488e-07	0.32719854119884€	0.133	0.008	0.0306747183470384	BOP1
Recoverec	183	9.1462700115606e-07	0.49924852637162‑	0.133	0.008	0.0306747603647719	BLOC1S5-TXNDC!
Recoverec	183	9.1462700115606e-07	0.28061425295800	0.133	0.008	0.0306747603647719	AL024508.2
Recoverec	183	9.34788606948418e-07	0.277871119833169	0.133	0.008	0.031350940299836	POMGNT2
Recoverec	183	9.553777498339e-07	0.28207291832444€	0.133	0.008	0.0320414589739293	FGF12
Recoverec	183	1.07010324867355e-06	0.83309157030929€	0.933	0.288	0.0358891227540135	EWSR1
Recoverec	183	1.27666974920363e-06	0.42930627479101	0.2	0.019	0.0428169500487913	SH3BP5-AS1
Recoverec	183	1.35749163528906e-06	0.75055250830945	0.533	0.113	0.0455275544643246	MFN1
Recoverec	183	1.4196809151881e-06	0.45703622843959	0.267	0.033	0.0476132585335786	PDK4
Rejected	0	2.90873024215188e-18	1.35455124223025	0.908	0.298	9.75529948612897e-18(	APOA4
Rejected	0	1.09933064875594e-17	1.33820799515804	0.997	0.734	3.68693512979768e-17!	IGKC
Rejected	0	1.84552194074671e-17	1.40933848133703	0.99	0.582	6.1895114848763e-170	FABP6
Rejected	0	5.55914650895991e-16!	1.31784895183301	0.969	0.509	1.86442655617497e-16(	PHGR1
Rejected	0	1.91420100272673e-15!	0.93034667492294	0.624	0.106	6.4198473229449e-155	GUCA2A
Rejected	0	2.51000887264678e-15€	1.00784834139261	0.631	0.116	8.41806775708278e-15	PDE3A
Rejected	0	3.1232138054677e-142	1.10873080083773	0.774	0.225	1.04746344607776e-137	APOC3
Rejected	0	2.66684804451923e-12	1.22118536401797	0.906	0.415	8.9440749717086e-119	AC105402.3
Rejected	0	1.0978018605073e-117	1.55799356945016	0.88	0.437	3.68180787976937e-11	ZNF331
Rejected	0	8.87842573161129e-117	0.71964181167268	0.382	0.046	2.97764642186779e-11	CYP3A4
Rejected	0	1.02525199584994e-11€	0.81970174567400	0.542	0.112	3.43849014368152e-11	SLC5A1
Rejected	0	5.97475160884734e-11€	1.08185024754013	0.977	0.879	2.00381219457522e-111	RPS4X
Rejected	0	9.2220734776442e-116	0.88155950129066!	0.619	0.153	3.09289900293231e-111	GDA
Rejected	0	1.6718648995385e-113	1.16702483664743	0.98	0.891	5.60710050007224e-10!	RPS8
Rejected	0	2.4074284855598e-111	1.04239607244432	0.96	0.856	8.07403365487047e-107	RPS29
Rejected	0	9.94938577227276e-111	0.98304417555694	0.835	0.335	3.33682500030484e-10€	ALDOB
Rejected	0	8.82874003853149e-11(	0.87474550236891€	0.672	0.191	2.96098283412269e-10!	KRT19
Rejected	0	9.84229981998232e-10	0.73755708810913	0.476	0.095	3.30091051362567e-97	CLDN4
Rejected	0	1.92846085323263e-98	0.97690310688643	0.985	0.915	6.4676720095716e-94	RPS27
Rejected	0	3.66938312957615e-97	0.53821964844490!	1	1	1.23063771399725e-92	MT-CO2
Rejected	0	4.19062991673859e-97	0.70589259298848	0.455	0.091	1.40545346147579e-92	MUC17
Rejected	0	6.5424518185773e-96	0.50347283004375	1	1	2.19420749091446e-91	MT-CO3

Rejected	0	1.07509948310552e-93	0.89550008708147(	0.988	0.899	3.6056686464393e-89	RPL11
Rejected	0	1.3067719719776e-93	0.92350546384776:	0.96	0.859	4.38265183961849e-89	RPL29
Rejected	0	1.62774962727648e-92	0.92525294600402(	0.987	0.914	5.45914669995987e-88	RPL13
Rejected	0	1.10150326329895e-90	0.80980252646396	0.852	0.378	3.69422164445203e-86	FABP1
Rejected	0	4.87414055548538e-90	0.94536590914837(	0.962	0.865	1.63468925949869e-85	RPL3
Rejected	0	2.18320418972043e-89	0.87203688465475	0.96	0.879	7.32203021148439e-85	RPL37
Rejected	0	1.64301182624392e-87	0.86415644973317!	0.944	0.822	5.51033306285684e-83	RPL36
Rejected	0	3.19460225263089e-87	0.94070370401329(	0.931	0.779	1.07140570348735e-82	RPS21
Rejected	0	3.3220171666489e-87	0.74129564766999	0.621	0.195	1.11413811735071e-82	FABP2
Rejected	0	1.22780785690997e-86	0.91091585862309(	0.98	0.901	4.11782199050466e-82	RPS18
Rejected	0	3.32551956029711e-86	0.94742383476142!	0.954	0.835	1.11531275013244e-81	RPL19
Rejected	0	7.17600787505924e-86	0.87008972528401(	0.969	0.903	2.40668952113737e-81	RPL34
Rejected	0	9.14823265650064e-86	0.55057012976661:	0.371	0.064	3.06813426833719e-81	CLDN3
Rejected	0	8.94576283270773e-84	0.63587221878166	0.239	0.021	3.00022993883352e-79	MYOM2
Rejected	0	9.2062491152549e-83	1.83179273092766	0.722	0.342	3.08759182827419e-78	ATP1B3
Rejected	0	2.01154853659205e-82	0.54466748986552	0.412	0.086	6.74633148202243e-78	PMP22
Rejected	0	9.67005916656258e-82	0.88348201902616:	0.965	0.895	3.24314444328176e-77	RPL12
Rejected	0	6.79072790393101e-81	0.66765526906154:	0.471	0.119	2.27747432442038e-76	FGD4
Rejected	0	1.30036779940013e-80	0.66030233151002!	0.57	0.171	4.36117352562817e-76	CYP3A5
Rejected	0	1.91935402821466e-80	0.73620647237332:	0.511	0.137	6.43712953982631e-76	HLA-DRA
Rejected	0	1.10795654842122e-79	0.37496451745967	1	1	3.71586467209508e-75	MT-CO1
Rejected	0	3.82620496504794e-79	1.10929857384202	0.728	0.32	1.28323262117778e-74	LTB
Rejected	0	2.545127663318e-78	0.88057723212471:	0.932	0.797	8.53584915723592e-74	RPL22
Rejected	0	6.90147742597932e-78	0.83225312952819	0.957	0.824	2.31461749912494e-73	RPL8
Rejected	0	7.16291130200296e-78	0.90225220913081	0.965	0.854	2.40229719246575e-73	RPL18A
Rejected	0	4.22879074857814e-76	1.06835107125484	0.964	0.768	1.41825184125814e-71	TXNIP
Rejected	0	4.73045922972951e-76	0.62316542688340:	0.504	0.141	1.58650141646668e-71	SLC15A1
Rejected	0	7.34487558116334e-76	0.58013918193818!	0.392	0.085	2.46332437241056e-71	THSD4
Rejected	0	1.55968124443561e-75	0.87069241678517(	0.951	0.592	5.23085895758816e-71	MTRNR2L8
Rejected	0	2.10228678707469e-75	0.60603500039238	0.478	0.126	7.05064942649108e-71	ADIRF
Rejected	0	8.6858067183746e-75	0.80989807816508	0.952	0.819	2.91304585720847e-70	NACA

Rejected	0	1.54832869254544e-74	0.74861017412320?	0.96	0.868	5.19278476905889e-70	RPS15
Rejected	0	3.18575220257644e-74	0.93808462392304?	0.942	0.851	1.06843757370009e-69	RPL21
Rejected	0	6.82246407306904e-74	0.82761496320946?	0.969	0.889	2.2881180008259e-69	RPL32
Rejected	0	1.80972765328019e-73	0.76087549294240?	0.962	0.899	6.06946460357109e-69	RPS15A
Rejected	0	3.27588919562321e-73	0.55680327240876	0.443	0.111	1.09866771842811e-68	MUC13
Rejected	0	6.76297536052961e-73	0.80620236619877?	0.946	0.846	2.26816667641442e-68	RPL35A
Rejected	0	1.00191621758438e-72	0.69580310529781?	0.646	0.243	3.36022661053448e-68	LIMD2
Rejected	0	2.359684902901e-72	0.74721815276547?	0.682	0.275	7.91391122734938e-68	LAPTM5
Rejected	0	4.60615030642016e-72	0.71803174605144?	0.977	0.914	1.54481068976719e-67	RPL28
Rejected	0	6.65673844029341e-72	0.80846942850650?	0.96	0.86	2.2325369381056e-67	RPL6
Rejected	0	1.06799358630851e-71	0.58776225954696?	0.474	0.132	3.58183688976148e-67	NEDD4L
Rejected	0	8.21924685069964e-71	0.69541058638207?	0.957	0.847	2.75657100878765e-66	RPL24
Rejected	0	1.99534388082303e-70	0.76337794577421?	0.929	0.8	6.69198430750428e-66	RPL37A
Rejected	0	2.59469905173344e-70	0.45445979478780?	0.257	0.035	8.70210167970362e-66	TM4SF20
Rejected	0	1.20291553037188e-69	0.63017720202913?	0.614	0.222	4.0343381057612e-65	SELENOP
Rejected	0	1.95850080479917e-69	0.53935414102143?	0.377	0.086	6.56841999913545e-65	HLA-DRB1
Rejected	0	6.74220264493351e-69	0.75837065420493?	0.98	0.907	2.2611999230578e-64	RPS14
Rejected	0	1.91565125091714e-68	0.82258904005456?	0.776	0.358	6.4247111653259e-64	CD74
Rejected	0	2.8752807892502e-67	0.59845489835950?	0.722	0.289	9.64311671098732e-63	JCHAIN
Rejected	0	4.47804279888643e-67	0.59891159419820?	0.486	0.147	1.50184599389053e-62	RBP2
Rejected	0	1.51279923496285e-66	0.49848968071095?	0.343	0.074	5.07362607421841e-62	CDHR2
Rejected	0	1.57442659938654e-66	0.50370343501216?	0.361	0.082	5.28031192902257e-62	AMN
Rejected	0	2.19758457259352e-66	0.73063970578229?	0.965	0.895	7.37025913956413e-62	RPS23
Rejected	0	2.26464837635965e-66	0.86877589487787?	0.929	0.787	7.59517772463499e-62	RPL10A
Rejected	0	4.43343919198024e-66	0.74341279482088?	0.954	0.85	1.48688683620633e-61	RPS7
Rejected	0	5.01332668818428e-66	0.84002033146689?	0.928	0.781	1.68136950468324e-61	RACK1
Rejected	0	1.80671588982745e-65	0.75383448567048?	0.516	0.171	6.0593637513033e-61	HOOK2
Rejected	0	3.93231985353995e-65	0.57238486081433?	0.909	0.49	1.31882143248023e-60	REG1A
Rejected	0	5.52874622273047e-65	0.78914532908110?	0.913	0.708	1.85423090817935e-60	EEF1D
Rejected	0	4.88614069888336e-64	0.84497716709104?	0.977	0.929	1.6387138675915e-59	RPS12
Rejected	0	5.1509791544069e-64	0.48684389803805?	1	0.994	1.72753538880499e-59	MT-CYB

Rejected	0	7.01557168774538e-64	0.685143567917231	0.985	0.918	2.35288243263605e-59	RPS28
Rejected	0	2.00314273184071e-63	0.729267259911852	0.987	0.923	6.71814009404736e-59	RPS2
Rejected	0	3.49765059122665e-63	0.491406190260153	0.389	0.098	1.17304205528559e-58	ELF3
Rejected	0	4.77756034639614e-63	0.492448558000805	0.366	0.088	1.60229818897434e-58	TMC5
Rejected	0	7.48610776591152e-63	0.847646485501079	0.504	0.171	2.5106908225314e-58	PRMT9
Rejected	0	2.26056938666739e-62	0.465945251737736	0.306	0.062	7.58149760900508e-58	PDE9A
Rejected	0	4.14857604892139e-62	0.508609333474053	0.321	0.067	1.39134943528726e-57	KCNQ5
Rejected	0	4.24987361374859e-62	0.688960407299435	1	0.98	1.425322612579e-57	MT-ND3
Rejected	0	5.78182570522494e-62	0.674966780851307	0.959	0.845	1.93910870501834e-57	RPL15
Rejected	0	6.10056069568689e-62	0.705030573924189	0.951	0.87	2.04600604611947e-57	RPL7A
Rejected	0	3.58387676928503e-61	0.745153926531828	0.956	0.902	1.20196059088281e-56	RPS3A
Rejected	0	7.39789277703382e-61	0.718379506986442	0.985	0.929	2.4811052795616e-56	RPL10
Rejected	0	1.42000829919106e-60	0.67054397864136	0.489	0.159	4.76242383382699e-56	MYO1E
Rejected	0	2.02484815508004e-60	0.878554789822566	0.932	0.774	6.79093574250744e-56	RPS5
Rejected	0	2.20580541934807e-59	0.421756661449764	0.297	0.06	7.39783021540957e-55	SERPINA1
Rejected	0	2.76399795591552e-59	0.354646709063716	0.138	0.006	9.26989634454946e-55	CD79A
Rejected	0	2.92335709752281e-59	0.998151498622892	0.929	0.771	9.804355033672e-55	JUNB
Rejected	0	4.55913658776895e-59	0.449956596146509	0.18	0.017	1.52904322880595e-54	MS4A1
Rejected	0	1.27856466705131e-58	0.51114315816547	0.442	0.131	4.28805018035668e-54	LGALS4
Rejected	0	1.43704350724832e-58	1.0097088811968	0.539	0.212	4.81955651460941e-54	TBC1D4
Rejected	0	1.92711043413634e-58	0.667950101379481	0.937	0.825	6.46314297400647e-54	RPL35
Rejected	0	2.25909207642437e-58	0.54371767520687	0.507	0.172	7.57654300591204e-54	ACSL5
Rejected	0	2.2744291385433e-58	0.662032515954291	0.975	0.897	7.62798044484652e-54	RPL30
Rejected	0	3.46224147253999e-58	0.584364985797878	0.718	0.312	1.16116654506046e-53	PCSK5
Rejected	0	1.06854389220616e-57	0.607634631642133	0.626	0.254	3.58368250568103e-53	KRT8
Rejected	0	1.15378855700311e-57	0.614438150382446	0.98	0.923	3.86957606247702e-53	RPL39
Rejected	0	1.23084788660159e-57	0.630206744374784	0.939	0.808	4.12801764208441e-53	UBA52
Rejected	0	2.78798796250514e-57	0.724576137506728	0.954	0.848	9.35035402864974e-53	RPS9
Rejected	0	3.18380866484986e-57	0.727911461341301	0.979	0.907	1.06778575001735e-52	RPS6
Rejected	0	4.09052530578942e-57	0.740055687531375	0.982	0.887	1.37188037705566e-52	ACTB
Rejected	0	5.88926138120485e-57	0.728587887675074	0.909	0.778	1.97514048202848e-52	RPL7

Rejected	0	3.74915337651576e-56	0.690336365362639	0.904	0.701	1.25739105941586e-51	RPL31
Rejected	0	9.57346291877067e-56	0.989958115734588	0.295	0.069	3.21074799369731e-51	ICA1
Rejected	0	2.4077788633284e-55	0.62731736916478	0.666	0.295	8.07520875183079e-51	ATP5ME
Rejected	0	3.31405323248865e-55	0.409941803929324	0.236	0.04	1.11146717311204e-50	TMPRSS2
Rejected	0	5.32424861150324e-55	0.610404595088209	0.954	0.865	1.78564649932596e-50	RPS11
Rejected	0	1.40840575552867e-54	0.397718416165638	0.362	0.096	4.72351122289204e-50	EPB41L3
Rejected	0	2.90572974239658e-54	0.60725199974251	0.959	0.871	9.74523641004965e-50	RPS16
Rejected	0	4.63362996127982e-54	0.805414905745276	0.86	0.523	1.55402681641403e-49	GAPDH
Rejected	0	8.6152571868496e-54	0.747341216714711	0.687	0.305	2.88938495532562e-49	IPCEF1
Rejected	0	1.14899760861811e-53	0.60599811697838	0.974	0.922	3.85350817978343e-49	RPL41
Rejected	0	3.09178878815184e-53	0.576407906845107	0.975	0.899	1.03692412377036e-48	RPLP2
Rejected	0	6.17737340064249e-53	0.612779768053272	0.863	0.496	2.07176749110748e-48	MTRNR2L12
Rejected	0	2.74981567708167e-52	0.980568253153914	0.845	0.516	9.22233181779651e-48	CEMIP2
Rejected	0	3.40615971207559e-52	0.621086512684509	0.932	0.818	1.14235784423591e-47	FAU
Rejected	0	3.77482838535524e-52	0.408876391750176	0.269	0.057	1.26600194388044e-47	HHLA2
Rejected	0	5.21427195865576e-52	0.388446041436119	0.259	0.053	1.74876252949397e-47	PRAP1
Rejected	0	9.69011012689455e-52	0.64820485660722	0.959	0.865	3.24986913435789e-47	RPS25
Rejected	0	2.63579058412668e-51	0.360428594500653	0.168	0.019	8.83991446104407e-47	CXorf40A
Rejected	0	2.64278054217487e-51	0.644155982191017	0.975	0.892	8.86335738234606e-47	RPS27A
Rejected	0	4.1373207274029e-51	0.474071630360217	0.473	0.163	1.38757462555638e-46	EPCAM
Rejected	0	5.90708811600125e-51	0.460460333177501	0.316	0.082	1.9811192123445e-46	DPEP1
Rejected	0	7.24619464758346e-51	0.727269676520176	0.824	0.491	2.43022876090654e-46	CD52
Rejected	0	1.94097924846765e-50	0.656578366060989	0.924	0.82	6.50965620351082e-46	RPL26
Rejected	0	2.5233775519006e-50	0.302143836184707	0.11	0.004	8.46290363356424e-46	VPREB3
Rejected	0	5.71733853238025e-50	0.592118253098462	0.975	0.892	1.91748099698969e-45	RPL18
Rejected	0	7.71672295861164e-50	0.410375154025622	0.283	0.066	2.58803454585917e-45	COBL
Rejected	0	1.10806174821839e-49	0.409116115655541	0.422	0.134	3.71621749117485e-45	CST3
Rejected	0	3.23037780374502e-49	0.270082673528588	0.135	0.01	1.08340410782e-44	C20orf204
Rejected	0	5.24998912317548e-49	0.582231076987523	0.671	0.317	1.76074135213059e-44	PIK3IP1
Rejected	0	6.7175303402192e-49	0.417079761179147	0.349	0.1	2.25292532550271e-44	MME
Rejected	0	7.78010536466801e-49	0.47372686283036	0.532	0.206	2.60929173720236e-44	LMO7

Rejected	0	8.27270441715573e-49	0.596283659709187	0.951	0.851	2.77449960742622e-44	RPL27A
Rejected	0	1.33506139137547e-48	0.375951248441056	0.407	0.128	4.47752889439507e-44	DAB1
Rejected	0	1.50249673957581e-48	0.415280803708789	0.333	0.09	5.03907356518934e-44	SMIM24
Rejected	0	1.51759462274362e-48	0.554237270981387	0.409	0.136	5.08970884575754e-44	C9orf72
Rejected	0	2.18406356153672e-48	0.648083840129203	0.853	0.559	7.32491237268184e-44	COX4I1
Rejected	0	4.57342670222524e-48	0.363881913022344	0.221	0.041	1.5338358473923e-43	HLA-DRB5
Rejected	0	5.97395286981028e-48	0.617949552757921	0.201	0.033	2.00354431347697e-43	BANK1
Rejected	0	2.45564284551467e-47	0.444749886242268	0.425	0.142	8.23573497528709e-43	CDHR5
Rejected	0	3.30261432605109e-47	0.541264246566628	0.878	0.505	1.10763079267101e-42	IGHA1
Rejected	0	3.68676217016447e-47	0.61981890319194	0.326	0.095	1.23646629662976e-42	PASK
Rejected	0	5.14530484752907e-47	0.383529205663115	0.229	0.045	1.7256323397643e-42	SLC7A9
Rejected	0	1.02851406138436e-46	0.32864652722721	0.209	0.038	3.44943045907088e-42	SLC6A19
Rejected	0	1.21055447097843e-46	0.356134252283208	0.259	0.058	4.05995758476746e-42	CYBRD1
Rejected	0	1.62307272195422e-46	0.637622443200604	0.967	0.873	5.44346129489008e-42	RPS13
Rejected	0	2.47441594489717e-46	0.402604315753351	0.56	0.225	8.29869619599614e-42	PIGR
Rejected	0	3.82150015792804e-46	0.501930512101487	0.643	0.292	1.28165472296591e-41	ANPEP
Rejected	0	1.3941837014428e-45	0.83191410083431	0.845	0.578	4.67581329789886e-41	SLC2A3
Rejected	0	1.75811756125264e-45	1.15769741720936	0.362	0.127	5.8963746769291e-41	CTLA4
Rejected	0	1.86529960384684e-45	0.403805039849737	0.292	0.076	6.25584181138153e-41	NCKAP5
Rejected	0	2.85109819623501e-45	0.720648878968493	0.883	0.725	9.56201313053298e-41	EEF1B2
Rejected	0	3.56758107363172e-45	0.514377069753829	0.657	0.312	1.19649534047461e-40	EDF1
Rejected	0	5.55529309913361e-45	0.458029702347399	0.544	0.218	1.86313419958743e-40	MALRD1
Rejected	0	2.06965063008499e-44	0.404287422173695	0.437	0.153	6.94119428317903e-40	TSPAN8
Rejected	0	2.15229208542865e-44	0.391657751530543	0.267	0.066	7.21835719611062e-40	NR1H4
Rejected	0	2.26671070272034e-44	0.960019189246154	0.59	0.295	7.60209435478347e-40	TRBC1
Rejected	0	2.74468162343392e-44	0.374696859561909	0.275	0.069	9.20511322867267e-40	SHROOM3
Rejected	0	4.92704761310005e-44	0.346427275418432	0.222	0.046	1.65243322848149e-39	KHK
Rejected	0	5.88640272359912e-44	0.437220041087659	0.325	0.094	1.97418174544067e-39	HLA-DPB1
Rejected	0	1.39933216640049e-43	0.604662546735783	0.974	0.882	4.69308021967395e-39	RPS3
Rejected	0	1.67874242109499e-43	0.429256818275733	0.629	0.28	5.63016633186839e-39	LGALS3
Rejected	0	2.17634128850471e-43	0.305184211570401	0.196	0.035	7.29901341338708e-39	GUCA2B

Rejected	0	4.39188393427429e-43	0.518338005909742	0.9	0.663	1.47295003387691e-38	COX7C
Rejected	0	6.39238024887357e-43	0.535818047369776	0.985	0.938	2.14387648786722e-38	TMSB4X
Rejected	0	1.57563010304022e-42	0.334134508000604	0.272	0.07	5.28434823957631e-38	DNPH1
Rejected	0	1.80757813848478e-42	0.557205334392191	0.895	0.674	6.06225556085025e-38	TOMM7
Rejected	0	2.45440492623435e-42	0.386082145116917	0.374	0.123	8.23158324160476e-38	ITGB2
Rejected	0	3.76066457502818e-42	0.408435596149501	0.247	0.06	1.26125168517295e-37	CASS4
Rejected	0	3.82018268276663e-42	0.57073286377784	0.987	0.92	1.28121286814627e-37	RPS19
Rejected	0	4.08050043143338e-42	0.363680962700452	0.155	0.021	1.36851823469413e-37	ANKRD55
Rejected	0	4.72718302033803e-42	0.536920503916299	0.575	0.267	1.58540264136097e-37	GYPC
Rejected	0	7.11094279778521e-42	0.339080086335291	0.229	0.051	2.3848679955212e-37	TMIGD1
Rejected	0	8.09915726796523e-42	0.495150380355259	0.494	0.205	2.71629536453018e-37	CCR7
Rejected	0	1.19787100222117e-41	0.324783506124348	0.308	0.088	4.01741976724936e-37	PCK1
Rejected	0	2.1076681208811e-41	0.366579729012657	0.13	0.014	7.06869734381103e-37	AC018754.1
Rejected	0	2.75340436820901e-41	0.550423516941034	0.923	0.811	9.23436757009938e-37	RPL23A
Rejected	0	2.79597078087379e-41	0.424926236909863	0.438	0.166	9.37712680489452e-37	ALKBH7
Rejected	0	3.1504090201518e-41	0.370836477992154	0.446	0.169	1.05658417717851e-36	CYB5A
Rejected	0	4.17822141667572e-41	0.491917317277393	0.631	0.302	1.4012918987247e-36	PPDPF
Rejected	0	5.20343316878669e-41	0.57069174347566	0.509	0.218	1.74512741614768e-36	TNFSF8
Rejected	0	6.4290077364539e-41	0.600467394613032	0.82	0.572	2.15616061465191e-36	RPS10
Rejected	0	1.0340232428835e-40	0.538511347668463	0.964	0.887	3.46790715198268e-36	RPL9
Rejected	0	1.07002550434611e-40	0.570404431359639	0.702	0.361	3.58865153647599e-36	PLCG2
Rejected	0	1.6822820989471e-40	0.546381799017202	0.949	0.865	5.64203770344878e-36	RPL14
Rejected	0	3.42756779481116e-40	0.526037964989692	0.834	0.531	1.14953768702377e-35	UQCRB
Rejected	0	4.19666800451489e-40	0.334066890685364	0.189	0.037	1.40747851535421e-35	PDK4
Rejected	0	6.4408460142848e-40	0.806182994551663	0.618	0.323	2.16013093627084e-35	VPS37B
Rejected	0	6.66374826586407e-40	0.594836310300801	0.377	0.137	2.23488789340549e-35	TMEM184C
Rejected	0	7.93605997256646e-40	0.270459628259452	0.237	0.057	2.66159579359934e-35	MEP1A
Rejected	0	1.23106353107838e-39	0.455442227737952	0.313	0.098	4.12874087053068e-35	SCML1
Rejected	0	1.5640557236862e-39	0.499494838669319	0.993	0.955	5.24553008609878e-35	RPLP1
Rejected	0	2.20927113538588e-39	0.493580761788861	0.506	0.213	7.40945353385715e-35	C9orf16
Rejected	0	4.5795563727721e-39	0.294974073415924	0.209	0.046	1.53589161630031e-34	PRSS3

Rejected	0	1.03828430281059e-38	0.338477530201239	0.384	0.136	3.48219789476614e-34	NDUFB1
Rejected	0	1.10886553565458e-38	0.591833428694237	0.745	0.446	3.71891323347832e-34	LDHB
Rejected	0	1.89382608228278e-38	0.349353373014455	0.234	0.058	6.35151391475999e-34	ACTN1
Rejected	0	2.27101848750097e-38	0.661410250521362	0.901	0.778	7.61654180338074e-34	RPL23
Rejected	0	6.30963425892257e-38	0.472901623905002	0.967	0.911	2.11612513775745e-33	RPL13A
Rejected	0	7.60616397431651e-38	0.412661518101916	0.598	0.281	2.55095527370627e-33	ATP5IF1
Rejected	0	7.79315545466924e-38	0.40164730416578	0.98	0.85	2.61366847638697e-33	FTL
Rejected	0	9.68134424355571e-38	0.67503835041066	0.956	0.764	3.24692923240372e-33	ACTG1
Rejected	0	1.04860086053616e-37	0.405339930798697	0.514	0.221	3.51679756606616e-33	GPX4
Rejected	0	1.46840265837511e-37	0.626857385618794	0.554	0.258	4.92472883565843e-33	LEF1
Rejected	0	2.05560096261929e-37	0.502069615552192	0.573	0.279	6.89407450843258e-33	RGS10
Rejected	0	2.38954721719843e-37	0.292960164893253	0.247	0.065	8.0140634570401e-33	ST14
Rejected	0	2.52211739937535e-37	0.655748745485598	0.903	0.651	8.45867733402506e-33	DNAJB1
Rejected	0	2.61115169743547e-37	0.351654880809303	0.227	0.057	8.7572805628591e-33	MATN2
Rejected	0	2.72697121412324e-37	0.518048046886435	0.916	0.737	9.14571605792653e-33	RPL38
Rejected	0	2.89929897708021e-37	0.378420514884607	0.558	0.247	9.7236689093316e-33	ELOB
Rejected	0	2.96208227258236e-37	0.302883158379862	0.247	0.065	9.93423152578672e-33	CDH1
Rejected	0	2.9701384721786e-37	0.38314289430243	0.356	0.122	9.9612504079926e-33	AC026979.2
Rejected	0	4.69165018494418e-37	0.309218943364724	0.18	0.035	1.57348563902658e-32	AIF1
Rejected	0	6.07464995701172e-37	0.495833863992259	0.997	0.964	2.03731610258259e-32	EEF1A1
Rejected	0	7.40003772114798e-37	0.267210827579773	0.231	0.058	2.48182465091861e-32	SLC5A12
Rejected	0	8.46378326773435e-37	0.322722817254322	0.252	0.069	2.83858363233275e-32	FRMD3
Rejected	0	9.34065349512442e-37	0.604907273409559	0.932	0.788	3.13266836919483e-32	RPLP0
Rejected	0	1.05144364273203e-36	0.403886313738762	0.563	0.259	3.52633168899467e-32	GPSM3
Rejected	0	1.54524470292784e-36	0.328470271219531	0.249	0.068	5.18244168467938e-32	ERRFI1
Rejected	0	1.892247973599e-36	0.562933441316519	0.82	0.557	6.34622125385632e-32	CD3D
Rejected	0	2.00700820393047e-36	0.503655261274909	0.54	0.245	6.73110411434201e-32	CERS6
Rejected	0	2.08883720534448e-36	0.474207532311988	0.69	0.374	7.00554221928432e-32	C12orf57
Rejected	0	3.05902415125031e-36	0.372683439185043	0.407	0.158	1.02593551984633e-31	MZT2A
Rejected	0	5.16785760572541e-36	0.65503764162315	0.237	0.064	1.73319608380819e-31	ARHGAP24
Rejected	0	6.58074308759993e-36	0.288204187959997	0.227	0.058	2.20704961671926e-31	CXADR

Rejected	0	7.59823693304326e-36	0.286267554438537	0.306	0.097	2.54829670260405e-31	CTSD
Rejected	0	1.12262753384166e-35	0.523670609649707	0.942	0.857	3.76506822299817e-31	RPL5
Rejected	0	2.01036609680566e-35	0.320109207389103	0.201	0.047	6.74236581546683e-31	STXBP6
Rejected	0	2.34402557271066e-35	0.281120185986964	0.29	0.09	7.86139296575701e-31	PTPRD
Rejected	0	2.35107282715758e-35	0.321070025358456	0.239	0.066	7.88502804772108e-31	MYO15B
Rejected	0	2.40983542491421e-35	0.490394092936073	0.74	0.431	8.08210604807728e-31	EIF3K
Rejected	0	4.029774602628e-35	0.288445610379298	0.217	0.054	1.35150580622938e-30	GNG12
Rejected	0	4.6343383199907e-35	0.385464695150915	0.756	0.39	1.55426438575848e-30	IGLC2
Rejected	0	8.56345066536515e-35	0.316575652469271	0.468	0.196	2.87201008415016e-30	PRKG1
Rejected	0	9.19309393434121e-35	0.467609204075109	0.911	0.728	3.08317984369935e-30	PFDN5
Rejected	0	9.44926075862786e-35	0.253547380854271	0.163	0.031	3.16909307322861e-30	KCNK5
Rejected	0	9.54621568495623e-35	0.280619468739039	0.163	0.031	3.20160981642062e-30	MDK
Rejected	0	1.44949874191085e-34	0.463626640974453	0.998	0.965	4.8613288806206e-30	TPT1
Rejected	0	2.61529057578137e-34	0.408377343221729	0.343	0.126	8.77116153305557e-30	CD27
Rejected	0	3.32326160548379e-34	0.522727190748467	0.997	0.92	1.11455547724715e-29	BTG1
Rejected	0	3.32884689746425e-34	0.425414221873868	0.675	0.363	1.11642867247156e-29	NDUFS5
Rejected	0	3.89739170589202e-34	0.500025313443469	0.738	0.432	1.30710723032207e-29	CD37
Rejected	0	6.22353745932167e-34	0.445299864333651	0.244	0.07	2.0872499931073e-29	LYN
Rejected	0	6.27391452518503e-34	0.475933139543695	0.911	0.665	2.10414545345656e-29	UBB
Rejected	0	1.7288804904557e-33	0.269339830015534	0.199	0.048	5.79831938889031e-29	SLC2A2
Rejected	0	2.88682987519236e-33	0.669096419340072	0.74	0.463	9.68185003542015e-29	TRBC2
Rejected	0	2.998045388907e-33	0.402224490913909	0.442	0.185	1.00548446253163e-28	TUBA4A
Rejected	0	3.35605659252264e-33	0.351438806399159	0.476	0.208	1.12555426000024e-28	APOB
Rejected	0	3.38429024363798e-33	0.452401923120646	0.595	0.29	1.13502326191131e-28	TXK
Rejected	0	3.52361956747154e-33	0.356797266728737	0.219	0.059	1.18175153053861e-28	MYO7B
Rejected	0	4.57969395805152e-33	0.446847704330434	0.946	0.855	1.53593775965132e-28	RPS20
Rejected	0	5.48336162690369e-33	0.507101210809136	0.522	0.255	1.83900982243096e-28	UBE2S
Rejected	0	6.60921482444544e-33	0.374847290539392	0.641	0.331	2.21659846782251e-28	MZT2B
Rejected	0	7.78834385189585e-33	0.30097108672719	1	1	2.61205476104883e-28	MT-ATP6
Rejected	0	1.26019403702593e-32	0.33061393991779	0.325	0.115	4.22643876137755e-28	FCGRT
Rejected	0	3.10329261749134e-32	0.280018599227515	0.209	0.055	1.04078227805424e-27	CLDN7

Rejected	0	3.1766218605588e-32	0.350609394045852	0.506	0.225	1.06537543959421e-27	ATP5PO
Rejected	0	4.65271769445265e-32	0.468016021367303	0.949	0.876	1.56042846036553e-27	RPS24
Rejected	0	5.07715674183898e-32	0.528716208613844	0.906	0.686	1.70277682807796e-27	ARHGDIB
Rejected	0	6.27740207448267e-32	0.329384670379771	0.262	0.082	2.10531510774e-27	ACE
Rejected	0	8.49357720963217e-32	0.303197416260456	0.148	0.028	2.84857592456644e-27	COBLL1
Rejected	0	8.94832918989129e-32	0.274081010464458	0.249	0.076	3.00109064370574e-27	CES2
Rejected	0	9.10573285171938e-32	0.532136505055651	0.694	0.401	3.05388068380964e-27	TRAC
Rejected	0	9.57158032040337e-32	0.390387046113549	0.57	0.287	3.21011660785688e-27	ATP5F1D
Rejected	0	1.02172342352829e-31	0.434140171225077	0.829	0.544	3.42665601782917e-27	SRP14
Rejected	0	1.59943606871139e-31	0.264200229475372	0.185	0.044	5.36418868724425e-27	MYO5B
Rejected	0	1.67947458138227e-31	0.526778195150878	0.984	0.877	5.63262185103985e-27	H3F3B
Rejected	0	1.72147303590212e-31	0.28888285759748	0.329	0.12	5.77347626780854e-27	SPINT2
Rejected	0	1.82919561595019e-31	0.305196380788407	0.129	0.021	6.13475625677375e-27	ADAM28
Rejected	0	2.00439137418479e-31	0.440199655960462	0.651	0.357	6.72232779074094e-27	SLC25A3
Rejected	0	2.16597851707894e-31	0.335362352364986	0.478	0.21	7.26425875057936e-27	VAMP8
Rejected	0	2.62308551539642e-31	0.374346716095238	0.438	0.189	8.79730420153651e-27	ISG15
Rejected	0	2.63824233071401e-31	0.656749769208235	0.918	0.729	8.84813712874866e-27	YPEL5
Rejected	0	4.47741835654316e-31	0.474079421775675	0.817	0.523	1.50163656841745e-26	HSPH1
Rejected	0	4.78454323328397e-31	0.27190419162298	0.323	0.116	1.60464010957878e-26	C15orf48
Rejected	0	6.405141428554e-31	0.369177674271897	0.412	0.174	2.14815633230844e-26	PRDX2
Rejected	0	9.88000737800689e-31	0.255587331880058	0.226	0.065	3.31355687443595e-26	B3GNT5
Rejected	0	1.04208495758861e-30	0.340867750292838	0.282	0.097	3.49494453076067e-26	HIST1H1C
Rejected	0	1.04208773018277e-30	0.422503675219332	0.45	0.205	3.49495382948697e-26	NOSIP
Rejected	0	1.06838931309961e-30	0.291481430074489	0.44	0.188	3.58316407827347e-26	CHCHD10
Rejected	0	1.08763824165584e-30	0.283075680242815	0.389	0.155	3.64772113486535e-26	DGAT1
Rejected	0	1.28110733133646e-30	0.292823132339413	0.377	0.152	4.2965777678362e-26	FIS1
Rejected	0	1.53147987774886e-30	0.295068978908884	0.252	0.08	5.13627721399412e-26	DENND5B
Rejected	0	1.6238628437164e-30	0.2823471217199	0.203	0.054	5.44611120525607e-26	HLA-DQB1
Rejected	0	1.97852170684519e-30	0.448345085994027	0.168	0.039	6.63556610041742e-26	TOX2
Rejected	0	2.57158097400045e-30	0.540703479636512	0.934	0.755	8.6245682706027e-26	UBC
Rejected	0	3.59374283606799e-30	0.384332979828532	0.435	0.189	1.20526947236048e-25	FCMR

Rejected	0	4.10129679721151e-30	0.427857715719736	0.651	0.348	1.3754929198488e-25	HSPA1B
Rejected	0	4.27639323288687e-30	0.44520664439122	0.662	0.371	1.4342167624456e-25	FXYD5
Rejected	0	4.46460019397427e-30	0.283547866147973	0.252	0.081	1.49733761305509e-25	ARF5
Rejected	0	6.44050045800617e-30	0.365627035342047	0.308	0.113	2.16001504360611e-25	LARGE1
Rejected	0	7.8444545244424e-30	0.561205751274142	0.417	0.184	2.63087315840749e-25	OPRM1
Rejected	0	1.20775946792557e-29	0.439116246475328	0.7	0.403	4.05058370352879e-25	HSPA1A
Rejected	0	1.347753715561e-29	0.426102129832624	0.731	0.432	4.5200964112485e-25	HSPE1
Rejected	0	1.8023951724717e-29	0.439910392165632	0.608	0.329	6.04487292943557e-25	DDIT4
Rejected	0	1.82310607485253e-29	0.349493945007152	0.567	0.282	6.11433315384042e-25	C19orf53
Rejected	0	1.95034271899169e-29	0.35759375760027	0.54	0.267	6.54105941095433e-25	SPCS1
Rejected	0	2.00264517858205e-29	0.260742303289228	0.213	0.061	6.71647139992849e-25	IFITM1
Rejected	0	2.06598845576443e-29	0.263106723050403	0.201	0.055	6.92891208294275e-25	NET1
Rejected	0	3.53722227042388e-29	0.407073930614298	0.883	0.663	1.18631360505476e-24	ATP5F1E
Rejected	0	3.58966709775782e-29	0.397073723330888	0.923	0.704	1.20390255124602e-24	SERF2
Rejected	0	3.63208028631121e-29	0.303947113405415	0.526	0.25	1.21812708642305e-24	UQCRQ
Rejected	0	3.86441601738142e-29	0.497080722939715	0.715	0.448	1.29604784390938e-24	SLC25A6
Rejected	0	4.04958103365619e-29	0.711197509341912	0.659	0.394	1.35814848706761e-24	BCAS2
Rejected	0	7.93058614457943e-29	0.326317591306952	0.554	0.275	2.65975998116905e-24	RPL36A
Rejected	0	8.33667876179348e-29	0.326481630514278	1	0.991	2.7959553231303e-24	MT-ND4
Rejected	0	8.67402787773556e-29	0.511134968663173	0.84	0.554	2.90909546963495e-24	NR4A2
Rejected	0	1.09054416446883e-28	0.290996212554735	0.176	0.044	3.65746701879556e-24	GNG7
Rejected	0	1.45272544725162e-28	0.466007799808082	0.86	0.648	4.87215060499249e-24	RPL4
Rejected	0	1.58114471828925e-28	0.39811266150739	0.572	0.299	5.30284315619848e-24	ST13
Rejected	0	2.19542948742307e-28	0.436197371138084	0.72	0.446	7.36303141491951e-24	NOP53
Rejected	0	2.33464742017222e-28	0.279165084240506	0.329	0.128	7.82994051777358e-24	WDR74
Rejected	0	2.562653521092e-28	0.274789743711026	0.241	0.077	8.59462737903836e-24	CDH17
Rejected	0	2.75871978347209e-28	0.325539093913591	0.377	0.156	9.25219440980869e-24	PLB1
Rejected	0	2.87707204057679e-28	0.385097404728608	0.608	0.322	9.64912420968643e-24	NSA2
Rejected	0	4.15710743221956e-28	0.388241452459964	0.628	0.344	1.3942106906178e-23	SNHG8
Rejected	0	5.38545066076684e-28	0.276255196813841	0.196	0.054	1.80617244260798e-23	TMEM45B
Rejected	0	6.07609907013242e-28	0.265283568616269	0.357	0.144	2.03780210614101e-23	ICAM3

Rejected	0	6.32034477543077e-28	0.275998983860439	0.213	0.063	2.11971723078397e-23	HLA-DPA1
Rejected	0	7.01801517471905e-28	0.275689863563418	0.433	0.189	2.35370192929728e-23	SMIM26
Rejected	0	7.04321996226484e-28	0.260827641141617	0.428	0.188	2.36215511094438e-23	EPS8
Rejected	0	7.08578987599505e-28	0.408329090769856	0.73	0.439	2.37643220861122e-23	GMFG
Rejected	0	7.20244854241603e-28	0.53450404531489	0.629	0.351	2.41555719215549e-23	JMY
Rejected	0	9.0731135030591e-28	0.442441602140652	0.904	0.784	3.04294080665596e-23	RPL27
Rejected	0	1.09340004084426e-27	0.274659409142099	0.301	0.113	3.66704505698348e-23	SELENOH
Rejected	0	1.56122797223452e-27	0.615092683571095	0.758	0.486	5.23604637328012e-23	IL6ST
Rejected	0	1.70101845613607e-27	0.300304034779661	0.56	0.283	5.70487569818914e-23	COX6C
Rejected	0	1.70628419353904e-27	0.506490160608847	0.909	0.819	5.72253592829124e-23	RPSA
Rejected	0	1.86375566143107e-27	0.516020629743566	0.992	0.919	6.25066373730753e-23	HSP90AA1
Rejected	0	2.16706295131537e-27	0.475902661011444	0.87	0.691	7.2678957261215e-23	BTF3
Rejected	0	3.13194454349797e-27	0.420242787715515	0.555	0.287	1.05039156099835e-22	CD7
Rejected	0	3.40424083064714e-27	0.273572128049827	0.395	0.17	1.14171428978244e-22	DDX27
Rejected	0	4.5932650728315e-27	0.312662544707396	0.507	0.247	1.54048924012623e-22	DDT
Rejected	0	4.5941924307765e-27	0.312701145961406	0.293	0.109	1.54080025743382e-22	SC5D
Rejected	0	4.60299897436671e-27	0.351374692422864	0.532	0.271	1.54375379602311e-22	EIF3G
Rejected	0	7.17882649815477e-27	0.302750281845161	0.422	0.19	2.40763483095115e-22	C1QBP
Rejected	0	8.50927688943222e-27	0.266926039465978	0.458	0.211	2.85384128317778e-22	OCIAD2
Rejected	0	8.57544362010452e-27	0.271550810506889	0.364	0.15	2.87603228131065e-22	CHRM3-AS2
Rejected	0	9.7236490835067e-27	0.421248053460535	0.484	0.239	3.26111742962648e-22	EIF4A3
Rejected	0	1.04761507662658e-26	0.447209267120422	0.916	0.73	3.51349144399021e-22	HLA-A
Rejected	0	1.07221930073294e-26	0.267025682565107	0.28	0.102	3.59600909079814e-22	KRT20
Rejected	0	1.40850054268357e-26	0.302419946522555	0.229	0.074	4.72382912005217e-22	FAM184A
Rejected	0	1.6583499512942e-26	0.265350836776028	0.325	0.129	5.56177406665049e-22	TMEM256
Rejected	0	1.66769840965441e-26	0.367944988553265	0.185	0.053	5.59312692629898e-22	LINC01891
Rejected	0	1.721029989898e-26	0.878944186907481	0.763	0.491	5.7719903801199e-22	CRYBG1
Rejected	0	1.7797585021055e-26	0.319183122636596	0.247	0.086	5.96895406436142e-22	IL6R
Rejected	0	2.72671779049572e-26	0.419850829567586	0.807	0.571	9.14486612576454e-22	CD48
Rejected	0	2.97618018787265e-26	0.324715357805358	0.524	0.265	9.98151311408729e-22	ABRACL
Rejected	0	3.14744359981918e-26	0.324143309719573	0.557	0.288	1.05558963450736e-21	APRT

Rejected	0	4.39675227790281e-26	0.315694984036827	0.209	0.065	1.47458277896304e-21	HKDC1
Rejected	0	5.98564798950079e-26	0.621356601687646	0.239	0.086	2.00746662271878e-21	TIGIT
Rejected	0	7.55355490853781e-26	0.336596063348692	0.596	0.315	2.53331124522541e-21	PRR13
Rejected	0	8.77949089708629e-26	0.506087058224521	0.784	0.517	2.9444656570648e-21	HSPD1
Rejected	0	1.03720589253463e-25	0.373270872900102	0.591	0.323	3.47858112238265e-21	RAC2
Rejected	0	1.24018610222216e-25	0.431762008606455	0.585	0.327	4.15933614963268e-21	CORO1A
Rejected	0	1.3964459192094e-25	0.761959592473219	0.722	0.458	4.68340032384447e-21	RANBP2
Rejected	0	2.01336228707408e-25	0.302032302432252	0.675	0.379	6.75241443838904e-21	PEBP1
Rejected	0	2.23142733547376e-25	0.344430192857697	0.456	0.221	7.4837609977119e-21	HIGD2A
Rejected	0	3.57528239102459e-25	0.285762281615566	0.236	0.082	1.19907820830183e-20	PHLDB3
Rejected	0	4.37062660392848e-25	0.34343898967662	0.638	0.366	1.46582075042553e-20	RPS17
Rejected	0	4.48807394107691e-25	0.338462078689772	0.537	0.28	1.50521023835837e-20	SLC25A5
Rejected	0	5.72223914625703e-25	0.395589038812261	0.585	0.323	1.91912456487168e-20	TCF7
Rejected	0	7.01966554353741e-25	0.32237424422578	0.634	0.354	2.35425542999158e-20	COX5B
Rejected	0	7.02290307380137e-25	0.374850947020315	0.152	0.038	2.3553412328915e-20	MAGEH1
Rejected	0	7.19999599164449e-25	0.274502324520202	0.474	0.234	2.41473465567773e-20	NDUFB11
Rejected	0	7.27909230202976e-25	0.263874305370102	0.369	0.159	2.44126197625474e-20	TAF10
Rejected	0	1.05057802372983e-24	0.348683661515319	0.575	0.309	3.52342857598509e-20	RARRES3
Rejected	0	1.3377288438091e-24	0.32586639036143	0.511	0.259	4.48647499636697e-20	ERP29
Rejected	0	1.39825265184458e-24	0.253543442383271	0.27	0.102	4.68945974375636e-20	MGAM
Rejected	0	2.41967294644017e-24	0.277727192723351	0.433	0.204	8.11509912777104e-20	TECR
Rejected	0	2.89453928155468e-24	0.525409046600096	0.316	0.136	9.70770584247808e-20	GK
Rejected	0	2.9012967364998e-24	0.316373546742847	0.456	0.222	9.73036899487302e-20	TMEM243
Rejected	0	3.87119304240498e-24	0.379632505333062	0.664	0.396	1.29832072256178e-19	CD3G
Rejected	0	4.31619037944858e-24	0.318165951565825	0.647	0.374	1.44756392945947e-19	UXT
Rejected	0	5.26086744894779e-24	0.336599572793992	0.572	0.316	1.76438972502811e-19	CIB1
Rejected	0	5.3707329059716e-24	0.461971284645677	0.942	0.825	1.80123640200476e-19	CXCR4
Rejected	0	6.88055878620801e-24	0.397393060973443	0.516	0.275	2.30760180571844e-19	DUSP2
Rejected	0	7.22934050040722e-24	0.403228042948927	0.893	0.65	2.42457621702657e-19	HINT1
Rejected	0	7.73696602203011e-24	0.263594632851914	0.371	0.164	2.59482366446846e-19	CARMIL1
Rejected	0	8.71985171585694e-24	0.275193678143153	0.329	0.139	2.9244638684641e-19	NUCB2

Rejected	0	1.19583150523509e-23	0.268483424080225	0.329	0.139	4.01057970225744e-19	HIST1H4C
Rejected	0	1.80276733036018e-23	0.26084601201512	0.348	0.154	6.04612107256197e-19	SCAND1
Rejected	0	2.15007027133626e-23	0.383831997065116	0.781	0.512	7.21090567600754e-19	MT-ND4L
Rejected	0	2.59563859249334e-23	0.262641000443315	0.445	0.217	8.70525271150418e-19	VPS28
Rejected	0	3.13152650933917e-23	0.447392203531005	0.715	0.441	1.05025136070217e-18	SOD1
Rejected	0	3.191893168741e-23	0.318751081383932	0.313	0.132	1.07049713093236e-18	MED29
Rejected	0	3.85604113599671e-23	0.266891051019901	0.245	0.09	1.29323907619058e-18	ABTB2
Rejected	0	7.74886490899741e-23	0.343130997610192	0.329	0.142	2.59881431317955e-18	CCSER1
Rejected	0	7.82706858616544e-23	0.385585842224823	0.824	0.622	2.62504226242816e-18	ATP5MG
Rejected	0	7.84829466139416e-23	0.349153772541051	0.764	0.519	2.63216106353837e-18	EIF3E
Rejected	0	1.2064651071364e-22	0.275692390951554	0.224	0.079	4.04624267631407e-18	ANKRD37
Rejected	0	1.55510228826521e-22	0.252647631884973	0.143	0.037	5.21550205438385e-18	AL627171.1
Rejected	0	1.56163824751283e-22	0.393098490702164	0.598	0.345	5.23742235450854e-18	LBH
Rejected	0	1.68512284803097e-22	0.608653050192022	0.72	0.47	5.65156500772625e-18	ELL2
Rejected	0	1.81175102029685e-22	0.494094037345668	0.829	0.606	6.07625057187158e-18	FYB1
Rejected	0	1.8325110412538e-22	0.416854346784924	0.381	0.184	6.14587553015699e-18	NAF1
Rejected	0	1.91229082677705e-22	0.301080525889407	0.605	0.339	6.41344097484486e-18	HMGN1
Rejected	0	2.23484222915463e-22	0.276269400600374	0.405	0.194	7.49521386813881e-18	PTP4A1
Rejected	0	2.6875897960428e-22	0.525227059710232	0.629	0.376	9.01363865796835e-18	SYAP1
Rejected	0	2.79141444961945e-22	0.298005581496224	0.652	0.383	9.36184578113373e-18	COX6B1
Rejected	0	3.24536134198538e-22	0.255212252155528	0.493	0.253	1.08842928687506e-17	ATP5MC3
Rejected	0	3.66146835709272e-22	0.300980171465198	0.507	0.271	1.22798325760176e-17	SMDT1
Rejected	0	3.72374889038061e-22	0.391783793562738	0.713	0.461	1.24887090285585e-17	SEPT6
Rejected	0	3.97574524644874e-22	0.379804773144572	0.501	0.27	1.33338544075398e-17	NR4A1
Rejected	0	5.32382036274507e-22	0.310865859676081	0.366	0.167	1.78550287325744e-17	SOCS1
Rejected	0	9.26584852533081e-22	0.362366030993819	0.366	0.174	3.10758027842545e-17	UCP2
Rejected	0	1.68637024184189e-21	0.254915960825982	0.484	0.249	5.65574851708934e-17	COX6A1
Rejected	0	1.84031052302963e-21	0.365246301356166	0.3	0.132	6.17203343213677e-17	MAL
Rejected	0	1.88573931130022e-21	0.262525350555096	0.443	0.219	6.32439250223869e-17	SNHG7
Rejected	0	1.93343929319475e-21	0.260563419741997	0.42	0.205	6.48436870151655e-17	YPEL3
Rejected	0	2.17568915903277e-21	0.314818178619596	0.743	0.479	7.2968263015641e-17	SNRPD2

Rejected	0	2.45507394926415e-21	0.286157683428557	0.703	0.426	8.2338270110421e-17	ANAPC16
Rejected	0	2.67466598172844e-21	0.423236400535325	0.652	0.402	8.97029476952085e-17	SELENOK
Rejected	0	3.35585865296345e-21	0.292498603786519	0.507	0.27	1.12548787503088e-16	DYNLT1
Rejected	0	4.46729933507756e-21	0.373987682079748	0.88	0.685	1.49824285099831e-16	NPM1
Rejected	0	5.47730017860837e-21	0.280889431057782	0.349	0.161	1.83697693390167e-16	POR
Rejected	0	5.98067918256501e-21	0.262609538299178	0.219	0.081	2.00580018424865e-16	RHBDD2
Rejected	0	7.86110633840206e-21	0.31442186313199	0.272	0.113	2.63645784377328e-16	ZDBF2
Rejected	0	8.26249972476102e-21	0.280392958961766	0.273	0.113	2.77107715769035e-16	LY9
Rejected	0	1.45660012822554e-20	0.303082970272784	0.27	0.111	4.8851455100428e-16	DNAJB4
Rejected	0	2.47430364063412e-20	0.265253327630382	0.189	0.065	8.29831954995872e-16	ARG2
Rejected	0	2.70305993257175e-20	0.251892284364868	0.333	0.153	9.06552240185913e-16	SGK1
Rejected	0	2.73568083377577e-20	0.285497306877745	0.71	0.446	9.17492638031718e-16	ISG20
Rejected	0	3.5160195838145e-20	0.264806012092327	0.241	0.094	1.17920264801971e-15	AC025164.1
Rejected	0	3.65328823533661e-20	0.264801667939684	0.331	0.155	1.22523980836719e-15	CNN2
Rejected	0	4.05329793702492e-20	0.344629956687593	0.735	0.481	1.35939506211942e-15	CD3E
Rejected	0	5.42733611316712e-20	0.260123013193804	0.26	0.108	1.82021998563399e-15	CKS2
Rejected	0	7.24262010658098e-20	0.336490040260745	0.311	0.144	2.42902993134513e-15	BCL2L11
Rejected	0	8.24774815398845e-20	0.260262546127228	0.45	0.231	2.76612977588465e-15	RNASET2
Rejected	0	8.85112671634168e-20	0.290908260689271	0.755	0.501	2.96849087812667e-15	CHCHD2
Rejected	0	8.89951906943811e-20	0.302500813157513	0.501	0.269	2.98472070550815e-15	PARK7
Rejected	0	1.02021691480709e-19	0.377609558912117	0.17	0.056	3.42160348888002e-15	MEF2C
Rejected	0	1.32537630273362e-19	0.399157018155355	0.558	0.327	4.445047044108e-15	TRMO
Rejected	0	2.5861475650708e-19	0.519937329952058	0.603	0.381	8.67342170373445e-15	USP36
Rejected	0	3.15302379373447e-19	0.479842042142424	0.689	0.448	1.05746111994267e-14	CYCS
Rejected	0	3.59046013149444e-19	0.428997301153584	0.876	0.687	1.2041685189006e-14	PFN1
Rejected	0	3.68850808718888e-19	0.279603277365308	0.807	0.548	1.2370518422814e-14	CIRBP
Rejected	0	7.6377901776977e-19	0.280298687674086	0.369	0.184	2.56156206979625e-14	NSMCE3
Rejected	0	1.01469543995601e-18	0.269079570690101	0.717	0.443	3.40308556652448e-14	HMGN2
Rejected	0	1.13302230725961e-18	0.580862555638085	0.558	0.332	3.79993021408729e-14	MRPL1
Rejected	0	1.93848751109416e-18	0.305985835312088	0.214	0.086	6.50129941470759e-14	CKAP2
Rejected	0	2.19983594446502e-18	0.623168268137587	0.682	0.489	7.37780979054677e-14	KLF2

Rejected	0	2.3676993682634e-18	0.46508305155864	0.896	0.703	7.94079014128179e-14	CCNH
Rejected	0	2.44230097707571e-18	0.285233084196036	0.672	0.411	8.19098901691653e-14	UQCRH
Rejected	0	2.67979631675751e-18	0.269479864571025	0.402	0.208	8.98750088714134e-14	FAM102A
Rejected	0	2.77806794006102e-18	0.363426786265215	0.957	0.878	9.31708425737665e-14	TMSB10
Rejected	0	2.84273464721844e-18	0.265910021906283	0.221	0.089	9.5339634598412e-14	CHPT1
Rejected	0	2.90229253126729e-18	0.328436629916661	0.257	0.113	9.73370869136425e-14	GADD45A
Rejected	0	3.21696308955346e-18	0.269385089878602	0.652	0.409	1.07890508097444e-13	EIF3F
Rejected	0	4.51997921958701e-18	0.324328678115416	0.738	0.512	1.51591063066509e-13	EEF2
Rejected	0	5.06300990557469e-18	0.344305104361486	0.903	0.742	1.69803226213164e-13	H3F3A
Rejected	0	9.19415056659658e-18	0.277771652761122	0.577	0.346	3.08353421702516e-13	SNU13
Rejected	0	9.83574355926733e-18	0.256732623473319	0.188	0.07	3.29871167490708e-13	MAP7
Rejected	0	1.43826175605317e-17	0.336950187823605	0.965	0.863	4.82364227745112e-13	SARAF
Rejected	0	3.321873308185e-17	0.267961804607066	0.74	0.496	1.11408987009909e-12	ATP5MC2
Rejected	0	5.07814785795135e-17	0.269129780835319	0.3	0.144	1.70310922859972e-12	HIST1H1D
Rejected	0	5.72923696783183e-17	0.329275181935457	0.951	0.763	1.92147149427144e-12	SMAP2
Rejected	0	1.31086845401392e-16	0.2891775699276	1	0.978	4.39639062107189e-12	MT-ND1
Rejected	0	2.2864212662016e-16	0.268084604517747	0.743	0.517	7.66819964258691e-12	COMMD6
Rejected	0	2.95336661197025e-16	0.328541343512262	0.285	0.138	9.90500094322583e-12	TIAM1
Rejected	0	3.28883786568138e-16	0.269458936642021	0.318	0.158	1.10301044339222e-11	WDR45B
Rejected	0	4.40028119950197e-16	0.303650375923297	0.348	0.182	1.47576630868897e-11	CD40LG
Rejected	0	5.59234043605409e-16	0.347483621736902	0.539	0.331	1.87555913544382e-11	COTL1
Rejected	0	1.70039216523987e-15	0.254775621606315	0.595	0.37	5.70277524378146e-11	ARPC5
Rejected	0	2.73823160407533e-15	0.357018775683559	0.45	0.262	9.18348115374784e-11	NR4A3
Rejected	0	3.41137625662034e-15	0.541236547605114	0.509	0.307	1.14410736894533e-10	TOX
Rejected	0	3.65228067309157e-15	0.303875352021149	0.71	0.473	1.22490189214145e-10	EVL
Rejected	0	3.92765514816955e-15	0.293209150002518	0.414	0.235	1.3172569835931e-10	CHMP1B
Rejected	0	4.18825553177731e-15	0.338898408485102	0.209	0.093	1.40465714024748e-10	DEAF1
Rejected	0	1.00222625337954e-14	0.298759538439139	0.142	0.051	3.36126640858431e-10	EBF1
Rejected	0	1.41700151853504e-14	0.375917968765232	0.755	0.523	4.75233969286281e-10	SAT1
Rejected	0	3.81517071673063e-14	0.433807710471636	0.944	0.76	1.27953195497712e-09	EZR
Rejected	0	1.55150326414121e-13	0.262275347341235	0.269	0.136	5.2034316472768e-09	AC008892.1

Rejected	0	1.66201055744911e-13	0.29409617508155[illegible]	0.285	0.149	5.57405100757284e-09	AGPAT5
Rejected	0	8.77041621783648e-13	0.25592592748886[illegible]	0.489	0.306	2.941422191138e-08	GIMAP7
Rejected	0	9.54420433533029e-13	0.26978445792042[illegible]	0.554	0.358	3.20093524998307e-08	SLA
Rejected	0	1.02092403401452e-12	0.26896470380857[illegible]	0.39	0.228	3.4239750252779e-08	SNAPC1
Rejected	0	1.32267887818278e-12	0.27696821138495[illegible]	0.13	0.049	4.43600042164939e-08	CDK14
Rejected	0	1.9893457871117e-12	0.39940125528943[illegible]	0.329	0.188	6.67186790081523e-08	MIR4435-2HG
Rejected	0	2.67917194397516e-12	0.25374224109269[illegible]	0.201	0.094	8.98540686570389e-08	CCDC138
Rejected	0	4.12473791568689e-12	0.25896932912526[illegible]	0.105	0.036	1.38335460216307e-07	AL138720.1
Rejected	0	5.82847247749026e-11	0.27048625026928[illegible]	0.901	0.701	1.95475309950068e-06	ETS1
Rejected	0	8.84613566030064e-11	0.25851531018987[illegible]	0.643	0.439	2.96681697775163e-06	NAP1L4
Rejected	0	1.93558656174083e-10	0.26295539462841[illegible]	0.969	0.87	6.49157021076641e-06	DUSP1
Rejected	0	6.93558757532535e-10	0.25435567852622[illegible]	0.843	0.637	2.32605736101262e-05	STAT3
Rejected	0	2.89699099159892e-09	0.42178817119007[illegible]	0.964	0.918	9.71592838762447e-05	SRGN
Rejected	0	3.1986830207839e-09	0.30816670080587	0.512	0.356	0.000107277431151051	FOXN2
Rejected	0	9.51722038985996e-09	0.26540482711239[illegible]	0.193	0.106	0.000319188537435123	RAB9A
Rejected	0	2.42165404902998e-08	0.38902334982880[illegible]	0.522	0.364	0.000812174334963674	CMSS1
Rejected	0	1.95781148138125e-07	0.33364314201047[illegible]	0.471	0.341	0.00656610814625643	MAF
Rejected	6	2.25877646881806e-22	2.38960383185847	0.215	0.026	7.57548452112201e-18	IL17A
Rejected	6	2.55174983460878e-22	0.98457548209855[illegible]	0.291	0.045	8.55805859531094e-18	IL23R
Rejected	6	8.09308360983105e-21	0.54132028239912[illegible]	0.228	0.03	2.71425838106514e-16	TNFSF14
Rejected	6	9.7310710706909e-21	1.47457298204686	0.392	0.087	3.26360661568831e-16	HDAC9
Rejected	6	2.31653300415032e-15	1.2421268031545	0.987	0.945	7.76918838931934e-11	CD69
Rejected	6	1.15991621703773e-13	0.81194394714614[illegible]	1	0.995	3.89012700870114e-09	MT-CYB
Rejected	6	2.83333413940122e-13	0.33947045917532[illegible]	0.177	0.029	9.50243603672382e-09	CAPN12
Rejected	6	5.7103906291053e-13	1.04109076506294	0.139	0.019	1.91515080918934e-08	IL2
Rejected	6	2.81328857913681e-12	0.90029822340465[illegible]	0.987	0.896	9.43520723670902e-08	H3F3B
Rejected	6	6.18344472538783e-12	1.31932428195916	0.481	0.184	2.07380369200057e-07	TNF
Rejected	6	6.53010107310848e-12	1.230836278535	0.873	0.602	2.19006529789912e-07	ZFP36
Rejected	6	8.22333018109342e-12	1.29327565452753	0.886	0.682	2.75794047613511e-07	BTG2
Rejected	6	2.87222783347182e-11	0.87165109414962[illegible]	0.924	0.785	9.63287770789778e-07	PPP1R15A
Rejected	6	1.5724152198188e-10	0.67951132868131[illegible]	0.228	0.055	5.27356616422829e-06	SLC4A10

Rejected	6	1.94907488637055e-10	0.850195378333722	0.658	0.372	6.53680735390954e-06	CORO1A
Rejected	6	3.04541459454957e-10	0.611937062855355	1	1	1.02137114672004e-05	MT-CO1
Rejected	6	3.08139603298405e-10	0.476703879041693	0.291	0.085	1.03343860154219e-05	PRR5
Rejected	6	4.23867060164138e-10	0.887911146430095	0.43	0.16	1.42156534637849e-05	ITGA1
Rejected	6	5.52985884628856e-10	0.9025791060577	0.291	0.089	1.85460405986826e-05	IL4I1
Rejected	6	5.85713411329673e-10	0.429806911353277	0.228	0.056	1.96436563891746e-05	HOPX
Rejected	6	7.34166329275162e-10	0.833268990346716	0.418	0.165	2.46224703512304e-05	BCL2A1
Rejected	6	1.09355927313758e-09	0.533240752920059	0.127	0.021	3.66757909024881e-05	KIT
Rejected	6	1.32663934798123e-09	0.881386369989035	0.987	0.966	4.44928304525945e-05	FOS
Rejected	6	1.88450021381504e-09	0.835213280354156	0.772	0.47	6.3202368170929e-05	KLRB1
Rejected	6	2.17280756428747e-09	0.908137672092872	0.494	0.22	7.28716200910731e-05	MAP3K8
Rejected	6	3.11245643206279e-09	0.455621066830277	0.228	0.062	0.000104385563818522	LAIR1
Rejected	6	3.66612766158556e-09	0.485204010223944	0.165	0.036	0.000122954589514256	IER5L
Rejected	6	6.25273809510616e-09	0.72019937776088	0.38	0.142	0.000209704330233671	CCR6
Rejected	6	7.59606670077304e-09	0.662077508235916	1	0.992	0.000254756885010526	MT-ND4
Rejected	6	8.12866824838041e-09	0.267584440726429	0.101	0.015	0.000272619275714182	LRRC49
Rejected	6	1.45631605623296e-08	0.603860386686562	0.646	0.361	0.00048841927893941	PPDPF
Rejected	6	1.46702804027333e-08	0.410087766590971	0.291	0.096	0.000492011864146869	LYSMD2
Rejected	6	1.72420536245213e-08	0.696219013948579	0.975	0.943	0.000578263994459194	KLF6
Rejected	6	2.05984389185892e-08	0.707522817618326	0.823	0.589	0.000690830444451645	S100A4
Rejected	6	3.60964072043295e-08	0.726484703276918	0.43	0.185	0.0012106013048188	C21orf91
Rejected	6	4.84785726932109e-08	0.769405186212189	0.785	0.575	0.00162587437098491	SH3BGRL3
Rejected	6	5.01420799176598e-08	0.373329520051775	0.152	0.034	0.00168166507627848	HAUS8
Rejected	6	8.39843866791025e-08	0.285840448220863	0.165	0.04	0.00281666836044374	ELK1
Rejected	6	9.08595475246156e-08	0.878231301507736	0.57	0.292	0.00304724750488056	CCL5
Rejected	6	1.32467394221633e-07	0.482004252995925	0.38	0.154	0.00444269146740511	MYO1F
Rejected	6	1.53840977543881e-07	0.263854296270468	0.101	0.018	0.00515951870486667	NCR3
Rejected	6	1.96235187644174e-07	0.653611037576618	0.684	0.444	0.00658133572321032	CD3G
Rejected	6	2.50931979381363e-07	0.568697193387225	0.392	0.172	0.00841575672449216	DYNLT3
Rejected	6	2.97620165584588e-07	0.554263137042352	1	1	0.0099815851133759	MT-ATP6
Rejected	6	4.11584031491947e-07	0.251539228599341	0.19	0.054	0.0138037052481769	CAPG

Rejected	6	5.25912217875476e-07	0.58596299169774	0.873	0.763	0.0176380439631077	PNRC1
Rejected	6	7.08249188585804e-07	0.83867266304110	0.557	0.31	0.0237532612867907	NR4A1
Rejected	6	7.82236841774538e-07	0.36847641766742	0.139	0.034	0.0262346591994345	PLEKHF1
Rejected	6	8.97438757028822e-07	0.31118418174569	0.468	0.218	0.0300983010332326	TUBA1C
Rejected	6	9.62267075593575e-07	1.04887719207898	0.785	0.556	0.0322725131812573	IER2
Rejected	6	1.24272884076949e-06	0.51198116835448	0.43	0.209	0.0416786398617273	TRAPPC1
Rejected	14	2.28238623901828e-28	1.45339453294655	0.8	0.243	7.65466696841951e-24	STAT1
Rejected	14	2.62977488103194e-18	0.81460284176469	0.462	0.108	8.81973899600491e-14	GBP5
Rejected	14	1.10088954198482e-17	0.63978792338342	0.292	0.049	3.69216334590869e-13	AL645465.1
Rejected	14	1.73875519675187e-17	2.88784466963411	0.477	0.114	5.83143717886644e-13	HBB
Rejected	14	1.29681578871936e-16	0.75497695420239	0.385	0.086	4.349260792207e-12	GBP4
Rejected	14	4.5678698628983e-14	0.33608106309358	0.108	0.009	1.53197219461883e-09	DTL
Rejected	14	1.37238350667473e-13	0.67524943684686	0.615	0.209	4.60269980468572e-09	EPG5
Rejected	14	3.02670875460465e-13	0.87679225609930	0.615	0.216	1.01509758211931e-08	ARL5B
Rejected	14	5.87364825530971e-13	1.02644906425482	0.969	0.658	1.96990415186577e-08	MTRNR2L8
Rejected	14	1.96644672255154e-12	0.45768415749664	0.246	0.047	6.59506901809335e-08	IFI44L
Rejected	14	3.13562965379307e-12	0.65895075982649	0.738	0.291	1.05162747328912e-07	OLFM4
Rejected	14	7.8001991460968e-12	0.85249793007125	0.662	0.272	2.61603078961794e-07	EPSTI1
Rejected	14	1.46278705991785e-11	0.43993054573579	0.323	0.079	4.9058952415525e-07	PUS10
Rejected	14	1.74427271657201e-11	0.51146023827075	0.415	0.122	5.84994183683921e-07	PARP9
Rejected	14	3.01816341360255e-11	0.82412042655881	0.415	0.13	1.01223164565402e-06	GBP1
Rejected	14	4.11456437456785e-11	0.50686878768544	0.585	0.209	1.37994259994256e-06	MT1H
Rejected	14	1.08847490014027e-10	0.31583684139390	0.4	0.115	3.65052712009045e-06	DEFA6
Rejected	14	1.23412352848284e-10	0.57436158103274	0.4	0.124	4.13900348982576e-06	XAF1
Rejected	14	1.31081776714781e-10	0.82946818373038	0.815	0.41	4.39622062746033e-06	RCOR1
Rejected	14	1.44129904252363e-10	0.70897670546225	0.831	0.414	4.83382872881575e-06	CD28
Rejected	14	4.48174991977386e-10	0.93094917836401	0.769	0.43	1.50308928809376e-05	TIPARP
Rejected	14	5.07826695759317e-10	0.56492477533414	0.4	0.132	1.7031491722376e-05	N4BP2L1
Rejected	14	5.82595257890877e-10	0.56591440217584	0.492	0.177	1.95390797591442e-05	IL4R
Rejected	14	7.56336241293302e-10	0.95699875887484	0.754	0.394	2.53660048604948e-05	PDE4B
Rejected	14	8.02727614327e-10	1.00639567087892	0.892	0.559	2.69218787292989e-05	MT-ND4L

Rejected	14	1.44149843813186e-09	0.72045639267651	0.446	0.162	4.83449746180663e-05	FAM122C
Rejected	14	1.46291464816499e-09	0.43356002884655	0.415	0.137	4.90632314701574e-05	PURB
Rejected	14	1.82402826263995e-09	0.64845075824135	0.123	0.018	6.11742598724186e-05	HIST1H2BH
Rejected	14	1.91356764357862e-09	0.27744551965985	0.785	0.337	6.41772316303398e-05	DEFA5
Rejected	14	3.82957241995605e-09	0.37172440543008	0.354	0.106	0.000128436199820486	DTX3L
Rejected	14	6.47551282501002e-09	0.60497178882041	0.323	0.098	0.000217175749125186	MIR222HG
Rejected	14	6.58745028794113e-09	0.36965066557340	0.308	0.088	0.00022092990775697	INTS7
Rejected	14	7.27301730588086e-09	0.37418951927037	0.277	0.076	0.000243922454404632	BCLAF3
Rejected	14	8.40176546707191e-09	0.88296183278474	0.4	0.141	0.000281778410234658	MIR155HG
Rejected	14	9.07432416659404e-09	0.60063932950585	0.938	0.747	0.000304334683899231	RHOH
Rejected	14	1.23075267270425e-08	0.42021551927993	0.246	0.063	0.000412769831371551	NETO2
Rejected	14	1.56595067693501e-08	1.0913640913048	0.431	0.155	0.000525188538030463	SOX6
Rejected	14	2.09636116402024e-08	1.00085220069556	0.554	0.265	0.000703077607189109	ADAM19
Rejected	14	2.47715981434448e-08	0.35868445230664	0.246	0.065	0.000830789858534853	SLC25A28
Rejected	14	3.8353910875158e-08	0.54153639929868	0.492	0.207	0.00128631346293105	EFHD2
Rejected	14	3.86410620707631e-08	0.54515819069559	0.662	0.318	0.00129594393972925	TUT7
Rejected	14	4.0888372812648e-08	0.26356080932708	0.108	0.016	0.00137131424739059	LINC02341
Rejected	14	4.58961270216753e-08	0.34021414099390	0.231	0.06	0.00153926430805295	SLC25A17
Rejected	14	5.71349436271533e-08	1.22911614004182	0.877	0.563	0.00191619173936747	MTRNR2L12
Rejected	14	8.24695874304447e-08	0.92661571316133	0.831	0.507	0.00276586502324226	AC105402.3
Rejected	14	1.08789164894604e-07	0.32261344921606	0.492	0.193	0.00364857101223522	AGPAT3
Rejected	14	1.18172933435976e-07	0.75766485524388	0.4	0.154	0.00396328384157576	BHLHE40
Rejected	14	1.18698996170078e-07	0.38873653113867	0.769	0.378	0.00398092693355209	OSBPL3
Rejected	14	1.20850638681502e-07	0.28213525434975	0.415	0.151	0.00405308872010022	PSMD5
Rejected	14	1.34864138562365e-07	0.52225839198734	0.354	0.126	0.0045230734791046	COX10
Rejected	14	1.35006893223374e-07	0.75123896902768	0.415	0.169	0.00452786118492553	FRMD4B
Rejected	14	1.49611339236196e-07	0.53526467086046	0.323	0.111	0.00501766509530355	OSTM1
Rejected	14	1.84613559779453e-07	0.52846928950816	0.354	0.129	0.00619156956788329	ZC3H12A
Rejected	14	1.86339095580489e-07	0.57998609410812	0.754	0.382	0.00624944058757843	PNPLA8
Rejected	14	1.90186719271022e-07	0.43433950041133	0.431	0.167	0.00637848219091152	VCL
Rejected	14	2.05256504343839e-07	0.53106333440627	0.569	0.272	0.00688389264268368	ADSS

Rejected	14	2.29428390839437e-07	0.32815914423554	0.308	0.1	0.00769456937197303	TXLNA
Rejected	14	2.44743099507763e-07	0.43297571395085	0.477	0.203	0.00820819407129136	APOL6
Rejected	14	2.95934904897805e-07	0.63574350514048	0.754	0.43	0.0099250648404626	TRAF3
Rejected	14	3.27974440367059e-07	0.73041787564472	0.831	0.512	0.0109996067810304	IRF1
Rejected	14	4.1203697586342e-07	0.47316774044391	0.385	0.146	0.0138188960965074	TTC21B
Rejected	14	4.163437587938e-07	0.60114986816888	0.585	0.295	0.0139633369824265	PRKX
Rejected	14	4.21070403532295e-07	0.271558891113162	0.123	0.023	0.0141218591936661	SYCP2
Rejected	14	4.76886531709497e-07	0.40665099695611	0.185	0.047	0.0159938205004731	HRH2
Rejected	14	5.4696353026384e-07	0.62843131485536	0.354	0.131	0.0183440628779887	PGAP1
Rejected	14	5.56704373621917e-07	0.31864452008833	0.292	0.098	0.0186707512825318	ZNF124
Rejected	14	5.82900776441788e-07	0.31762502414617	0.369	0.135	0.0195493262403047	RECQL
Rejected	14	7.05890007563244e-07	0.39136487588550	0.308	0.107	0.0236741390736561	NDFIP2
Rejected	14	7.2222057545092e-07	0.38367320563077	0.385	0.151	0.0242218336594729	TRIM37
Rejected	14	7.33759009431709e-07	0.611163996571557	0.138	0.03	0.0246088096583206	MKI67
Rejected	14	9.58609078732943e-07	0.39258450452739	0.369	0.14	0.0321498312825454	PDCL3
Rejected	14	1.0057858561843e-06	0.25475466532144	0.323	0.112	0.033732046044709	E2F3
Rejected	14	1.03066027811946e-06	0.80775580394444	0.769	0.459	0.0345662844075704	MT-ATP8
Rejected	14	1.0776817687585e-06	0.36784201938326	0.708	0.367	0.0361432911606227	PSMB9
Rejected	14	1.13316797543456e-06	0.711203687905084	0.2	0.055	0.0380041875601244	CSF1
Rejected	14	1.36082089375479e-06	0.64087802653507	0.492	0.221	0.0456392111347483	MAP3K8
Rejected	14	1.42623351924492e-06	0.60311689394593	0.538	0.266	0.0478330197684362	SLAMF1
Rejected	20	1.89901953973716e-11	1.46825367974554	0.791	0.405	6.36893173237048e-11	MT1G
Rejected	20	7.81744416444339e-64	1.07975373940422	0.448	0.162	2.62181442387102e-59	MT1H
Rejected	20	3.26034620061413e-58	0.82390863075534	0.218	0.033	1.09345490876197e-53	C1QB
Rejected	20	4.52876597382928e-53	0.90885178201100	0.794	0.522	1.51885753230286e-48	REG1A
Rejected	20	4.16746441407488e-48	0.66296248073877	0.184	0.028	1.39768421519243e-43	C1QA
Rejected	20	7.9669810695421e-41	0.63032550752590	0.935	0.862	2.67196611110303e-36	FTL
Rejected	20	5.20611603331609e-40	0.97984554296202	0.309	0.109	1.74602719525355e-35	AGR2
Rejected	20	2.41405831008228e-35	0.45023241788135	0.115	0.013	8.09626876035396e-31	C1QC
Rejected	20	5.42154603895554e-32	0.83790519813047	0.36	0.171	1.81827811054491e-27	MT1E
Rejected	20	2.34784734698556e-31	0.42899139997602	0.99	0.982	7.87421043232017e-27	B2M

Rejected	20	1.68774362832309e-29	0.785071321085116	0.631	0.468	5.66035458066999e-25	MT2A
Rejected	20	2.96066710707614e-26	0.55304424377645	0.94	0.922	9.92948534371197e-22	IL7R
Rejected	20	2.90348042590497e-25	0.523267313446078	0.923	0.887	9.73769265240011e-21	TMSB10
Rejected	20	1.2962162965929e-24	0.596761402046021	0.17	0.05	4.34725021551328e-20	MT1M
Rejected	20	3.10734503796363e-23	0.810964828119315	0.432	0.268	1.04214137883224e-18	OLFM4
Rejected	20	9.05307983154356e-23	0.722453201689456	0.376	0.218	3.03622191390308e-18	MT1X
Rejected	20	1.10091223023258e-22	0.590310752184298	0.774	0.702	3.69223943775403e-18	S100A6
Rejected	20	1.34035549301553e-22	0.399408318438961	0.962	0.949	4.49528425247547e-18	PTMA
Rejected	20	4.14365691762148e-20	0.366124552960354	0.95	0.929	1.38969965703189e-15	RPL41
Rejected	20	3.32706362886841e-19	0.547796640269618	0.806	0.724	1.11583059984989e-14	VIM
Rejected	20	8.37325721503328e-17	0.461653190819395	0.93	0.937	2.80822300477786e-12	BTG1
Rejected	20	8.27509941328657e-16	0.440316690352999	0.955	0.943	2.77530284122805e-11	CD69
Rejected	20	4.51831761114743e-15	0.572347234294119	0.895	0.888	1.51535336042663e-10	ZFP36L2
Rejected	20	9.42711996548103e-15	0.615909261657357	0.29	0.167	3.16166749402303e-10	FABP5
Rejected	20	2.01305174857892e-14	0.627572686123672	0.302	0.184	6.75137295438397e-10	IFI27
Rejected	20	4.90438542299252e-14	0.717321319316819	0.799	0.778	1.64483278316323e-09	NFKBIA
Rejected	20	8.01321104065559e-14	0.361072498373811	0.877	0.832	2.68747071881507e-09	RPL26
Rejected	20	1.94164999312142e-13	0.48902837721202	0.515	0.421	6.51190574693063e-09	SEC61B
Rejected	20	4.099963117306e-13	0.617492374910463	0.22	0.116	1.37504563028209e-08	CEBPD
Rejected	20	1.86868364842768e-12	0.342163392822764	0.799	0.765	6.26719122009677e-08	HMGB1
Rejected	20	3.36492351921093e-12	0.370859562001649	0.763	0.742	1.12852804987296e-07	RPL36AL
Rejected	20	9.13147722781086e-12	0.412054276247498	0.684	0.644	3.0625148326632e-07	S100A10
Rejected	20	2.08535506162564e-11	1.10255382315715	0.266	0.157	6.99386380568007e-07	CCL4
Rejected	20	2.60541466859436e-11	0.337666199462332	0.849	0.855	8.73803971553176e-07	CALM1
Rejected	20	6.67048998989949e-11	0.406899267965838	0.904	0.893	2.23714893281249e-06	TSC22D3
Rejected	20	2.19095076715613e-10	0.320755493544733	0.78	0.74	7.34801068288822e-06	SERF2
Rejected	20	5.1312394711701e-10	0.533027196149486	0.388	0.301	1.72091509384103e-05	CD63
Rejected	20	1.20522698200724e-09	0.735614158029383	0.269	0.173	4.04209025225588e-05	TNF
Rejected	20	1.96277177677791e-09	0.379646459835115	0.365	0.266	6.58274398495775e-05	CKLF
Rejected	20	3.39038190866286e-09	0.44391599417589	0.455	0.383	0.000113706628452735	RPS27L
Rejected	20	4.17257970580404e-09	0.297874785400001	0.645	0.578	0.000139939978173256	GAPDH

Rejected	20	7.07122248619255e-09	0.581812220965255	0.626	0.588	0.000237154659741926	S100A4
Rejected	20	7.46941828449935e-09	0.261424248621758	0.111	0.049	0.000250509350425539	HOPX
Rejected	20	8.23993810689062e-09	0.357324940482146	0.76	0.747	0.000276351044228897	HLA-C
Rejected	20	8.74931066368921e-09	0.439449562727587	0.165	0.091	0.000293434381038809	TFF3
Rejected	20	9.12872825187493e-09	0.28363970850114	0.852	0.848	0.000306159288111382	HLA-B
Rejected	20	1.41064109553053e-08	0.374639403302944	0.208	0.127	0.000473100810619029	STMN1
Rejected	20	1.4210216873304e-08	0.432061972233382	0.412	0.342	0.000476582253496868	UQCR10
Rejected	20	1.49148405810314e-08	0.380497467725415	0.897	0.888	0.000500213923406631	DUSP1
Rejected	20	2.48876172249097e-08	0.304770181688919	0.756	0.704	0.000834680906489021	UBB
Rejected	20	3.25485260741228e-08	0.404954714312575	0.511	0.472	0.00109161246747393	PPIB
Rejected	20	4.60012170195419e-08	0.423049396662011	0.487	0.422	0.0015427888164014	H2AFZ
Rejected	20	9.38550731092588e-08	0.372448981843645	0.153	0.085	0.00314771144193832	SAP30
Rejected	20	1.00851638043384e-07	0.419844490050691	0.389	0.323	0.00338236223669902	TXN
Rejected	20	1.01178570974979e-07	0.537819523728948	0.576	0.53	0.00339332691335886	SQSTM1
Rejected	20	1.01519474185483e-07	0.437525069506646	0.451	0.39	0.00340476012523272	TUBA1B
Rejected	20	1.03098564184004e-07	0.692081369391625	0.427	0.356	0.00345771964560312	GADD45B
Rejected	20	1.24449837421604e-07	0.434874075208081	0.484	0.424	0.00417379864744575	HCST
Rejected	20	1.45595511037417e-07	0.334334543553284	0.688	0.676	0.00488298224917288	MYL12A
Rejected	20	2.52518308524254e-07	0.423756410297808	0.513	0.475	0.00846895903128642	CYBA
Rejected	20	5.05037412886449e-07	0.305690004688453	0.712	0.7	0.0169379447533857	ITM2B
Rejected	20	5.37260670206059e-07	0.320952035083886	0.643	0.586	0.0180186483573708	TAGAP
Rejected	20	5.47172153712874e-07	0.32356482259062	0.19	0.12	0.0183510596912224	NTS
Rejected	20	6.12654705279589e-07	0.434364511828116	0.101	0.05	0.0205472135056669	C11orf96
Rejected	20	7.42962869958097e-07	0.400288642965431	0.309	0.24	0.0249174887326547	RABAC1
Rejected	20	9.05842026377603e-07	0.402065027194722	0.365	0.283	0.030380129880652	CCL5
Rejected	20	9.51685942214369e-07	0.406162565399655	0.187	0.118	0.0319176431299855	ABCA1
Rejected	23	2.97064183200147e-67	1.6219290434692	0.859	0.665	9.96293857616652e-63	ANK3
Rejected	23	1.45632653949641e-51	3.83468651703038	0.327	0.083	4.88422794816308e-47	HBB
Rejected	23	4.50367464037015e-48	0.949751349449764	0.741	0.414	1.51044240088734e-43	MT-ATP8
Rejected	23	2.70534508709072e-42	0.752080025603501	0.96	0.784	9.07318635308485e-38	PDE3B
Rejected	23	6.45710461943449e-37	0.842189755336257	0.485	0.208	2.16558374726594e-32	DDX3Y

Rejected	23	3.75905794624418e-34	0.56380058106914[illegible]	0.932	0.684	1.26071285401137e-29	SYNE2
Rejected	23	2.25541265065965e-31	0.51485652032871[illegible]	0.987	0.934	7.56420294778235e-27	ARHGAP15
Rejected	23	1.15866728123052e-30	0.62509822656126[illegible]	0.487	0.223	3.88593832779092e-26	UTY
Rejected	23	8.3329740648574e-30	0.62578583848389[illegible]	0.538	0.278	2.79471284187188e-25	RASA3
Rejected	23	5.92877069130724e-29	0.67000159726703[illegible]	0.483	0.236	1.98839111445062e-24	RPS4Y1
Rejected	23	1.28321585620415e-27	0.55309728584099[illegible]	0.994	0.975	4.30364933853749e-23	MBNL1
Rejected	23	3.04099613389883e-27	0.58029397941255[illegible]	0.928	0.774	1.01988928338699e-22	ANKRD12
Rejected	23	1.35753024922815e-25	0.492016854719111	0.892	0.725	4.55288494986137e-21	PRRC2C
Rejected	23	9.89147071362912e-24	0.6174622389272	0.778	0.575	3.31740144793693e-19	ZEB1
Rejected	23	1.10169916847072e-23	0.57378146638989[illegible]	0.835	0.657	3.69487867121711e-19	CHD2
Rejected	23	1.39409810443454e-23	0.48415624116260[illegible]	0.595	0.35	4.67552622265255e-19	TMEM87A
Rejected	23	3.21702000577582e-23	1.01721580789854	0.496	0.287	1.07892416953709e-18	FAAH2
Rejected	23	1.53273808272798e-22	0.52695506128769[illegible]	0.939	0.817	5.14049698185309e-18	SMCHD1
Rejected	23	2.72058120824419e-22	0.36560532178779[illegible]	0.994	0.961	9.12428525620938e-18	PTPRC
Rejected	23	6.73711595122026e-22	0.43771522118952[illegible]	0.962	0.874	2.25949394772025e-17	CD44
Rejected	23	1.39613801265757e-21	0.52029931394574	0.857	0.658	4.68236766685097e-17	SLC38A1
Rejected	23	2.23913602031217e-21	0.50923651702114[illegible]	0.806	0.598	7.50961438492295e-17	BCLAF1
Rejected	23	6.91492282726782e-21	0.50702796237349[illegible]	0.671	0.458	2.31912681780908e-16	DHX9
Rejected	23	1.0710604884712e-20	0.50931317692922	0.295	0.126	3.5921226662347e-16	USP9Y
Rejected	23	1.34928289670646e-19	0.373979321711571	0.981	0.906	4.52522497897412e-15	SIK3
Rejected	23	2.93665404971147e-19	0.50983044311340[illegible]	0.819	0.647	9.84895035192232e-15	RFX3
Rejected	23	3.02180595174325e-19	0.56301616360820[illegible]	0.808	0.677	1.01345328009565e-14	RPS26
Rejected	23	3.28207680185222e-19	0.43975740185680[illegible]	0.344	0.161	1.1007429178052e-14	CD44-AS1
Rejected	23	3.70106598945683e-19	0.46142224397327[illegible]	0.932	0.82	1.24126351154403e-14	PARP8
Rejected	23	6.89628121291939e-19	0.42657489184764	0.774	0.584	2.3128747931889e-14	SRRM2
Rejected	23	1.17656575342756e-18	0.45942778456100[illegible]	0.84	0.663	3.94596622384536e-14	BRAF
Rejected	23	1.20967533968008e-18	0.55499493467605[illegible]	0.616	0.413	4.05700915421906e-14	HSPA5
Rejected	23	2.28597068955313e-18	0.51934516586725[illegible]	0.854	0.683	7.66668849862328e-14	ANXA1
Rejected	23	2.77089209648792e-18	0.40916303091802	0.882	0.691	9.2930179132012e-14	IQGAP2
Rejected	23	3.78812036821076e-18	0.35725852622688[illegible]	0.407	0.209	1.27045980909052e-13	ESR2
Rejected	23	4.01758978250429e-18	0.45097566760840[illegible]	0.802	0.619	1.34741926125629e-13	NCL

Rejected	23	5.93527258978838e-18	0.461032681860561	0.692	0.483	1.99057172116323e-13	USP47
Rejected	23	7.1617968271755e-18	0.496856238929077	0.551	0.33	2.40192341989812e-13	FAM129A
Rejected	23	8.28447072857808e-18	0.468035499760325	0.755	0.585	2.77844579295052e-13	ADK
Rejected	23	8.91122326368873e-18	0.439309926657382	0.703	0.489	2.98864605817593e-13	FUBP1
Rejected	23	2.02964185277336e-17	0.463639633186103	0.741	0.53	6.80701284583131e-13	SCAF11
Rejected	23	3.91826353567999e-17	0.411239087886907	0.622	0.408	1.31410722459636e-12	DDX21
Rejected	23	4.94131819449313e-17	0.395857359923503	0.886	0.77	1.65721929606911e-12	PPP1R15A
Rejected	23	6.46861614698488e-17	0.415381189640924	0.895	0.726	2.16944448337579e-12	CCNL1
Rejected	23	6.89620934279556e-17	0.577811582688668	0.39	0.216	2.31285068938678e-12	RNF130
Rejected	23	1.20803383205508e-16	0.398355539190541	0.939	0.834	4.05150386594632e-12	PABPC1
Rejected	23	1.28036310019755e-16	0.407978874201186	0.949	0.859	4.29408176544254e-12	HNRNPC
Rejected	23	1.5867956287135e-16	0.450469754307184	0.57	0.364	5.32179517957933e-12	NUP153
Rejected	23	1.75271627066984e-16	0.402870224802865	0.907	0.77	5.8782598285725e-12	KMT2E
Rejected	23	1.82680164818425e-16	0.357968360366358	0.314	0.155	6.12672736768032e-12	AL354733.3
Rejected	23	1.83350425941498e-16	0.388882400250965	0.268	0.125	6.14920658522595e-12	SH3BP5
Rejected	23	1.96734695402904e-16	0.407017297988353	0.884	0.721	6.59808821442258e-12	LRBA
Rejected	23	3.45232889855473e-16	0.300011117690005	0.154	0.052	1.15784206599729e-11	TTTY15
Rejected	23	3.51899082718567e-16	0.455323646703849	0.69	0.492	1.18019914362153e-11	AHNAK
Rejected	23	4.20748505358542e-16	0.521783568011609	0.895	0.743	1.41110633727148e-11	GLS
Rejected	23	5.00592694249194e-16	0.361224578744466	0.502	0.304	1.67888777797295e-11	SLC25A26
Rejected	23	6.84989964837466e-16	0.386927151580734	0.603	0.394	2.29731934407189e-11	CASP8
Rejected	23	6.96258809822469e-16	0.448625913434696	0.804	0.623	2.3351127963826e-11	KAT6A
Rejected	23	7.39756254728283e-16	0.43099847663743	0.435	0.247	2.48099452710771e-11	LMNA
Rejected	23	7.93031733462917e-16	0.45540804843668	0.46	0.277	2.65966982768793e-11	NAA15
Rejected	23	8.83982129278407e-16	0.377044490556616	0.595	0.397	2.96469926517392e-11	EIF3A
Rejected	23	9.71299766662228e-16	0.422629431512056	0.764	0.581	3.25754515743178e-11	USP3
Rejected	23	1.27914922636994e-15	0.415894542577374	0.46	0.276	4.29001067539951e-11	TP53BP2
Rejected	23	2.16898885283282e-15	0.351144080033091	0.555	0.351	7.2743548146307e-11	FAM133B
Rejected	23	2.61757707342804e-15	0.399125929176959	0.741	0.563	8.77882998886296e-11	CSDE1
Rejected	23	3.43445326960103e-15	0.466672040165054	0.73	0.568	1.15184693755879e-10	BTBD9
Rejected	23	3.51062611752518e-15	0.502516504803787	0.348	0.194	1.17739378729559e-10	MT-ND6

Rejected	23	3.6776444244305e-15	0.475687971543492	0.778	0.631	1.2334083870655e-10	LRRFIP1
Rejected	23	4.12204358861393e-15	0.391637220367981	0.319	0.165	1.38245097874934e-10	CCDC173
Rejected	23	4.27446134470613e-15	0.376603441978407	0.842	0.666	1.43356884578754e-10	DPYD
Rejected	23	5.05637366987615e-15	0.369766055949996	0.759	0.569	1.69580660140306e-10	ZNF644
Rejected	23	5.135732880141e-15	0.358424038837551	0.734	0.536	1.72242209334169e-10	SF3B1
Rejected	23	7.24654349614646e-15	0.46721476645304	0.618	0.437	2.4303457577376e-10	TBC1D15
Rejected	23	1.08350497492813e-14	0.430361216049865	0.829	0.654	3.63385898491396e-10	USP15
Rejected	23	1.14525027896083e-14	0.363866063727664	0.69	0.5	3.84094038557882e-10	RBM25
Rejected	23	1.28880647029565e-14	0.362533698701831	0.943	0.857	4.32239914007755e-10	DENND4A
Rejected	23	2.78016047494331e-14	0.391093281039663	0.89	0.775	9.32410220086487e-10	GNG2
Rejected	23	3.76913884430759e-14	0.395962668972196	0.776	0.598	1.26409378560388e-09	SFPQ
Rejected	23	4.52308683513391e-14	0.398477412918518	0.785	0.595	1.51695286276721e-09	FBXW7
Rejected	23	4.84870776118802e-14	0.372321856748264	0.766	0.594	1.62615960894724e-09	FAM172A
Rejected	23	5.05596263728234e-14	0.380242307467334	0.823	0.654	1.69566874929175e-09	NFKB1
Rejected	23	5.08776092979991e-14	0.368505128619331	0.684	0.507	1.70633326063629e-09	RPS6KA5
Rejected	23	5.34548603231203e-14	0.352324777003313	0.757	0.599	1.79276910551681e-09	HNRNPDL
Rejected	23	9.50038815676668e-14	0.27864938641153	0.253	0.123	3.18624018001641e-09	DNAJB11
Rejected	23	1.33375648966229e-13	0.33100450628192	0.489	0.309	4.4731525150294e-09	HBS1L
Rejected	23	1.73829593004706e-13	0.32967206563582	0.791	0.619	5.82989689019183e-09	NIPBL
Rejected	23	1.98449680603352e-13	0.436939627334665	0.624	0.448	6.65560538807521e-09	KIAA1109
Rejected	23	2.06404319166165e-13	0.251622089718795	0.165	0.064	6.92238805619483e-09	AC009299.3
Rejected	23	2.21030768326172e-13	0.312972393031354	0.941	0.815	7.41292990812316e-09	JMJD1C
Rejected	23	2.57387908061419e-13	0.473233424605128	0.58	0.406	8.63227566056387e-09	TNKS
Rejected	23	2.772995521914e-13	0.311649536333151	0.861	0.675	9.30007238139518e-09	STAG1
Rejected	23	3.00957782071659e-13	0.384026675395192	0.53	0.346	1.00935220951193e-08	TRAF1
Rejected	23	3.18072915982953e-13	0.39655480499129	0.624	0.451	1.06675294562363e-08	YTHDC1
Rejected	23	3.6617026931231e-13	0.378241948389632	0.705	0.521	1.22806184921962e-08	TTC39C
Rejected	23	4.73463285125557e-13	0.35026275532508	0.757	0.577	1.58790116565409e-08	PIAS1
Rejected	23	5.25067056642801e-13	0.319886749098305	0.582	0.394	1.76096989456863e-08	ASCC3
Rejected	23	5.38930971956732e-13	0.390730918124979	0.667	0.476	1.80746669374849e-08	MAP3K5
Rejected	23	5.46325241227433e-13	0.368365375194617	0.726	0.543	1.83226559402856e-08	MORF4L2

Rejected	23	5.76771285742891e-13	0.336948938650434	0.705	0.522	1.93437553812451e-08	GOLGA4
Rejected	23	7.45730490408316e-13	0.314927787368262	0.899	0.764	2.50103091873141e-08	EML4
Rejected	23	7.78018180383289e-13	0.337378647079719	0.608	0.424	2.60931737336947e-08	AP3B1
Rejected	23	7.87084376576004e-13	0.345758902568375	0.407	0.246	2.6397235821606e-08	TCERG1
Rejected	23	1.08423701882399e-12	0.306812749364819	0.943	0.836	3.63631411373191e-08	ARIH1
Rejected	23	1.14999194552155e-12	0.321651464734286	0.865	0.705	3.85684298689017e-08	GPBP1
Rejected	23	1.41209954921708e-12	0.341713151598716	0.576	0.392	4.73589946816425e-08	CDC73
Rejected	23	1.41974150734318e-12	0.433583025862948	0.58	0.415	4.76152906732757e-08	PCGF5
Rejected	23	1.55228654440648e-12	0.306106208876932	0.757	0.57	5.20605861263045e-08	CDKAL1
Rejected	23	1.69656935987031e-12	0.326293481163689	0.257	0.133	5.68995431913306e-08	GARS
Rejected	23	1.72934954215282e-12	0.406986490506643	0.498	0.337	5.79989249447212e-08	CA5B
Rejected	23	2.09399451608593e-12	0.336400419679872	0.502	0.334	7.02283880804899e-08	RASGRP1
Rejected	23	2.24512329014275e-12	0.369599165862797	0.686	0.502	7.52969449048075e-08	KDM2A
Rejected	23	2.40958138324274e-12	0.382420794057508	0.641	0.478	8.0812540431195e-08	EPC2
Rejected	23	2.71614675188507e-12	0.330553630192186	0.399	0.243	9.10941297647215e-08	TNKS2
Rejected	23	2.81696408522922e-12	0.457105031820061	0.466	0.31	9.44753414904176e-08	ITGB1
Rejected	23	3.30400179752383e-12	0.345671323268708	0.897	0.796	1.10809612285354e-07	ARID4B
Rejected	23	3.76500448908602e-12	0.324131607565055	0.572	0.394	1.26270720554967e-07	PRPF4B
Rejected	23	3.96517371495411e-12	0.295282009473106	0.257	0.132	1.32983996052131e-07	GTPBP8
Rejected	23	4.31861663457607e-12	0.339157914263609	0.802	0.655	1.44837764690412e-07	BIRC6
Rejected	23	4.37154593035979e-12	0.383231167167305	0.734	0.551	1.46612907412407e-07	COG5
Rejected	23	4.64786115868212e-12	0.366054554505417	0.414	0.258	1.55879967539881e-07	FBXO33
Rejected	23	7.10823173486181e-12	0.336510329044359	0.612	0.445	2.38395875923795e-07	SENP6
Rejected	23	7.41264424913516e-12	0.324305220864142	0.418	0.261	2.48605262827495e-07	DCTN4
Rejected	23	9.95929737358554e-12	0.438455777443964	0.703	0.537	3.34014915315312e-07	BICDL1
Rejected	23	1.02806107479887e-11	0.275154965910963	0.842	0.66	3.44791123266044e-07	PCNX1
Rejected	23	1.08819721816681e-11	0.408257850276472	0.58	0.408	3.64959583028786e-07	QKI
Rejected	23	1.1180827367161e-11	0.319281943744958	0.565	0.383	3.74982588239846e-07	ATP9B
Rejected	23	1.12119525582083e-11	0.283069846532598	0.639	0.462	3.76026464897191e-07	MIER1
Rejected	23	1.1363570055332e-11	0.339352619968693	0.73	0.572	3.81111412515724e-07	SMYD3
Rejected	23	1.35721249019606e-11	0.331260227088405	0.576	0.4	4.55181924961953e-07	MON2

Rejected	23	1.45408848167939e-11	0.35295690105546	0.574	0.412	4.87672194985634e-07	SMARCA5
Rejected	23	1.56340444893138e-11	0.298200427239901	0.378	0.229	5.24334584082605e-07	RAB22A
Rejected	23	1.68826934537083e-11	0.323986840362481	0.629	0.456	5.6621177305047e-07	PDS5A
Rejected	23	1.73947373648347e-11	0.307414073228592	0.783	0.589	5.83384701741828e-07	AP001011.1
Rejected	23	1.90920182128875e-11	0.40314717366712	0.787	0.637	6.40308106823822e-07	TANK
Rejected	23	2.03390434985173e-11	0.275333470303674	0.281	0.151	6.82130840853273e-07	TGFBR3
Rejected	23	2.08888631578415e-11	0.456166204824139	0.728	0.567	7.0057069258769e-07	RGCC
Rejected	23	2.10138472393999e-11	0.336252739014567	0.403	0.247	7.04762408714995e-07	ANKRD36
Rejected	23	2.19965757984066e-11	0.357679329130578	0.648	0.475	7.37721159126961e-07	PCM1
Rejected	23	2.54984082477695e-11	0.304910348062506	0.791	0.629	8.55165615813693e-07	MYCBP2
Rejected	23	2.80157213095721e-11	0.312821595875954	0.939	0.872	9.3959126128043e-07	CDC42SE2
Rejected	23	2.80567696755642e-11	0.329427172178447	0.719	0.568	9.40967941379072e-07	RSRC2
Rejected	23	3.05508922238817e-11	0.325025646815024	0.787	0.621	1.02461582340454e-06	LPP
Rejected	23	3.16591227528879e-11	0.392387228827371	0.705	0.557	1.06178365888635e-06	PPP3CA
Rejected	23	3.22398894983407e-11	0.368492640163035	0.39	0.248	1.08126141399535e-06	ATR
Rejected	23	3.34607472008658e-11	0.356982941725148	0.58	0.418	1.12220653962264e-06	SMARCC1
Rejected	23	3.8705197903316e-11	0.276703116936701	0.43	0.271	1.29809492728141e-06	ZNF267
Rejected	23	4.17836461857418e-11	0.368649220372439	0.43	0.276	1.40133992577741e-06	AC001226.2
Rejected	23	4.89401088519258e-11	0.337779551997226	0.422	0.271	1.64135337067589e-06	TP53BP1
Rejected	23	5.26040650923399e-11	0.367795972888329	0.532	0.371	1.7642351350669e-06	WDR70
Rejected	23	5.42941236956237e-11	0.320035589622139	0.629	0.463	1.82091632050383e-06	XRN1
Rejected	23	6.44961613356812e-11	0.283912542695648	0.388	0.24	2.16307225887607e-06	WARS2
Rejected	23	7.35766290266345e-11	0.323984383148061	0.454	0.295	2.46761298429527e-06	SPATA5
Rejected	23	7.54230241159156e-11	0.285255752020211	0.295	0.167	2.52953738279958e-06	GOLGA8A
Rejected	23	8.34125107865649e-11	0.284726269531028	0.797	0.619	2.79748878675981e-06	USP34
Rejected	23	9.66659627669785e-11	0.309782622201685	0.576	0.409	3.24198305927892e-06	FNBP4
Rejected	23	1.02882375888103e-10	0.29439521949316	0.331	0.198	3.45046912253521e-06	CHD8
Rejected	23	1.12161920456514e-10	0.385530844393619	0.331	0.196	3.76168648827058e-06	FAM102B
Rejected	23	1.12806578810744e-10	0.265697733631018	0.224	0.115	3.78330704015473e-06	WDR36
Rejected	23	1.14716223743827e-10	0.284048800367918	0.703	0.537	3.84735271192046e-06	BPTF
Rejected	23	1.19906062972683e-10	0.368830527708002	0.435	0.287	4.02140953997784e-06	GRK5

Rejected	23	1.24741605887381e-10	0.31645027904007	0.424	0.274	4.183583978251e-06	SREK1
Rejected	23	1.44821419376875e-10	0.32169034548242	0.8	0.623	4.85702076306162e-06	WNK1
Rejected	23	1.54620769561156e-10	0.32739147025474	0.692	0.519	5.18567136954204e-06	PPP1R12A
Rejected	23	1.54745734024422e-10	0.26680103163359	0.504	0.344	5.18986242771106e-06	SP3
Rejected	23	1.54776504972069e-10	0.30506387564182	0.458	0.311	5.19089442375325e-06	CNTRL
Rejected	23	1.71105891396298e-10	0.29442015414893	0.441	0.292	5.73854938564903e-06	SCFD1
Rejected	23	1.73446118769227e-10	0.29118416849353	0.719	0.536	5.81703593128235e-06	GOLGB1
Rejected	23	1.76775849447764e-10	0.37451397015117	0.502	0.347	5.92870843877911e-06	RSRC1
Rejected	23	1.85200900626229e-10	0.28246239501423	0.319	0.185	6.21126780520247e-06	AC092652.2
Rejected	23	1.92931353051763e-10	0.31980109042413	0.637	0.479	6.47053171865002e-06	CYLD
Rejected	23	1.99092137193476e-10	0.31690023520851	0.451	0.304	6.67715209719481e-06	MTREX
Rejected	23	2.09175041829801e-10	0.31829389547988	0.46	0.309	7.01531255288786e-06	SUCO
Rejected	23	2.31094540942413e-10	0.28438438244453	0.724	0.559	7.75044871412665e-06	TOP1
Rejected	23	2.44734101564226e-10	0.3366989769361	0.546	0.391	8.20789229826101e-06	RNGTT
Rejected	23	2.60437000272187e-10	0.32384261781395	0.673	0.522	8.7345361151286e-06	COP1
Rejected	23	2.70611416641296e-10	0.26575772843234	0.629	0.463	9.07576569131578e-06	SLTM
Rejected	23	2.86947623384018e-10	0.35694457915457	0.749	0.617	9.6236493930532e-06	HERC1
Rejected	23	2.97806547386304e-10	0.52519858712285	0.485	0.343	9.98783598624186e-06	ANTXR2
Rejected	23	2.98236496526729e-10	0.33629602999425	0.802	0.653	1.00022556205134e-05	DYRK1A
Rejected	23	3.05876244978006e-10	0.31778440948631	0.781	0.632	1.02584775040724e-05	CDK17
Rejected	23	3.16988842959797e-10	0.27182541800661	0.65	0.47	1.06311718151857e-05	EMB
Rejected	23	3.24129871125839e-10	0.34508025222693	0.475	0.329	1.08706676178184e-05	MAN1A2
Rejected	23	3.24420886141558e-10	0.34144125715207	0.555	0.392	1.08804276794156e-05	PPP4R3A
Rejected	23	3.41161763811431e-10	0.29384279735399	0.511	0.35	1.14418832347078e-05	PBXIP1
Rejected	23	3.41799256875617e-10	0.27786092324778	0.838	0.699	1.14632634770944e-05	FUS
Rejected	23	3.48427091676632e-10	0.26551416742883	0.673	0.515	1.16855478006509e-05	EIF4G2
Rejected	23	3.78477954158525e-10	0.37893810665131	0.513	0.364	1.26933936265686e-05	SP140
Rejected	23	3.83054510176116e-10	0.32465462280405	0.321	0.196	1.28468821622866e-05	OTUD4
Rejected	23	3.86431432098124e-10	0.30755393158647	0.713	0.569	1.29601373697069e-05	ARL4C
Rejected	23	4.02145821034598e-10	0.28176281743699	0.196	0.097	1.34871665458583e-05	TAF1B
Rejected	23	4.03159740541165e-10	0.33048075327937	0.654	0.483	1.35211713782696e-05	ROCK1

Rejected	23	4.14328316085227e-10	0.28709893980818	0.882	0.745	1.38957430648664e-05	PPP1CB
Rejected	23	4.19639551696655e-10	0.27564315924355	0.338	0.205	1.40738712848024e-05	ECD
Rejected	23	4.28585171571936e-10	0.32685581540055	0.757	0.576	1.43738894841796e-05	ARID5B
Rejected	23	4.32808005531928e-10	0.31127994440222	0.831	0.695	1.45155148895298e-05	DOCK10
Rejected	23	4.48464545523284e-10	0.36167514010125	0.534	0.38	1.50406039277599e-05	XPR1
Rejected	23	4.61417113383382e-10	0.26389665533899	0.643	0.449	1.54750071486519e-05	MT1G
Rejected	23	4.6564721610849e-10	0.31525296610218	0.411	0.262	1.56168763338466e-05	SLCO3A1
Rejected	23	4.72889353494734e-10	0.29808123048669	0.757	0.604	1.58597631375064e-05	ASH1L
Rejected	23	5.52243306716252e-10	0.27275079861561	0.344	0.208	1.85211360206497e-05	ATXN2L
Rejected	23	5.7267897901549e-10	0.28680124018659	0.238	0.131	1.92065075982215e-05	ADPGK
Rejected	23	5.90086585015156e-10	0.324361114503232	0.416	0.277	1.97903238882383e-05	CDC37
Rejected	23	6.63493993766554e-10	0.29156619642503	0.483	0.34	2.22522615629427e-05	MAP3K1
Rejected	23	6.87268752473982e-10	0.28284417927956	0.715	0.564	2.30496194204724e-05	SON
Rejected	23	6.98526796203282e-10	0.25304734258522	0.504	0.34	2.34271916910657e-05	AGO3
Rejected	23	7.18709888556121e-10	0.29978074509848	0.513	0.36	2.41040922423952e-05	PBRM1
Rejected	23	7.59401014325041e-10	0.34699709503391	0.774	0.628	2.54687912184332e-05	CDC14A
Rejected	23	8.03553997288668e-10	0.334769384117767	0.278	0.162	2.69495939610673e-05	AC079793.1
Rejected	23	8.28646438575716e-10	0.37990492281855	0.812	0.682	2.77911442569524e-05	ZC3HAV1
Rejected	23	9.31051574306671e-10	0.26799452822843	0.732	0.567	3.12256076990971e-05	CUL3
Rejected	23	9.3344367917892e-10	0.34137990146091	0.502	0.36	3.13058341123026e-05	WDR26
Rejected	23	9.71933578415237e-10	0.33702410323797	0.405	0.268	3.25967083528902e-05	SENP5
Rejected	23	1.03215478706683e-09	0.29654466182352	0.228	0.122	3.46164072486475e-05	AL157938.2
Rejected	23	1.18075284343716e-09	0.27263139315874	0.464	0.317	3.96000888631956e-05	BDP1
Rejected	23	1.22291472013586e-09	0.29251725667776	0.451	0.297	4.10141138839165e-05	PIK3R5
Rejected	23	1.38479010615549e-09	0.32974312053856	0.373	0.244	4.64430905802428e-05	RAB18
Rejected	23	1.46273366036341e-09	0.27943994856997	0.557	0.409	4.90571615012681e-05	MARCH7
Rejected	23	1.62201068560084e-09	0.27453921336589	0.289	0.174	5.43989943736808e-05	SNIP1
Rejected	23	1.80650226151758e-09	0.27858853394507	0.833	0.684	6.05864728467766e-05	IKZF1
Rejected	23	1.93435971083628e-09	0.26403451631111	0.441	0.296	6.48745559820271e-05	AP1G1
Rejected	23	1.93652721269161e-09	0.33038263893884	0.819	0.702	6.49472496592513e-05	INPP4B
Rejected	23	1.95986528596176e-09	0.26357860112179	0.525	0.365	6.57299619605856e-05	PNPLA8

Rejected	23	1.99119790623721e-09	0.302208117058662	0.376	0.244	6.67807953793837e-05	SEC14L1
Rejected	23	2.08697090178707e-09	0.360725924697908	0.698	0.559	6.99928301041348e-05	NR3C1
Rejected	23	2.21319897746158e-09	0.35633227276621	0.523	0.381	7.42262673061065e-05	LMBRD1
Rejected	23	2.40671780526728e-09	0.274157226981648	0.451	0.305	8.07165017530541e-05	WBP11
Rejected	23	2.69691628842406e-09	0.303240925330645	0.73	0.568	9.04491784811663e-05	CHD1
Rejected	23	2.72789095244394e-09	0.337428819524732	0.367	0.238	9.14880067630648e-05	FAM69A
Rejected	23	2.93354098264322e-09	0.262408900743594	0.443	0.303	9.83850974758884e-05	TOP2B
Rejected	23	3.23567500008925e-09	0.370604991075071	0.384	0.254	0.000108518068152993	NEU1
Rejected	23	3.46312084397145e-09	0.268508332874424	0.137	0.06	0.000116146146865114	ZFY
Rejected	23	4.00430784374544e-09	0.264551031865219	0.281	0.165	0.000134296476463535	ABCE1
Rejected	23	4.35689139431106e-09	0.385656308693919	0.243	0.136	0.000146121423582404	MLF1
Rejected	23	4.63765240185965e-09	0.274260376555711	0.66	0.498	0.000155537586253569	TLE4
Rejected	23	4.70993209304475e-09	0.282456358445402	0.439	0.291	0.000157961702536535	KIAA2026
Rejected	23	4.72256325787097e-09	0.283696658757465	0.508	0.359	0.000158385326542477	KPNA4
Rejected	23	4.90255858956812e-09	0.281912606734882	0.616	0.465	0.000164422009976935	PLCL2
Rejected	23	5.00828047409697e-09	0.318061180943349	0.411	0.275	0.000167967710540264	SLFN12L
Rejected	23	5.11383251358335e-09	0.279043107068832	0.519	0.371	0.000171507714840559	GBP2
Rejected	23	5.18191945095968e-09	0.307646711608187	0.584	0.432	0.000173791214546286	RSF1
Rejected	23	5.2479027052731e-09	0.322139124904807	0.662	0.511	0.000176004160929449	PAPOLA
Rejected	23	5.39276844373216e-09	0.308171272501615	0.451	0.314	0.000180862668065889	PREX1
Rejected	23	5.82873698842522e-09	0.326835959037549	0.563	0.42	0.000195484181117805	STAT5B
Rejected	23	5.89092067359101e-09	0.259104554706918	0.371	0.245	0.000197569697550895	UBE4A
Rejected	23	5.93057643574637e-09	0.279460900527091	0.546	0.393	0.000198899672502062	XRN2
Rejected	23	6.09654197849819e-09	0.302902626335094	0.7	0.555	0.000204465824874872	FAF1
Rejected	23	6.1102881469148e-09	0.299529833376539	0.53	0.392	0.000204926843871228	SCAF8
Rejected	23	6.24725481105308e-09	0.401943702337567	0.989	0.938	0.000209520431853098	MT-ND5
Rejected	23	6.48839868177154e-09	0.28599338882048	0.475	0.316	0.000217607914989254	AC009313.1
Rejected	23	6.68991430064571e-09	0.253677639153948	0.93	0.873	0.000224366345815056	RAB11A
Rejected	23	6.97692982260541e-09	0.348919906796238	0.724	0.562	0.00023399227239054	FBXO11
Rejected	23	7.22729808142609e-09	0.252610218590188	0.498	0.358	0.000242389123054868	KDM5A
Rejected	23	7.35927713603012e-09	0.277822981771168	0.694	0.527	0.000246815436588178	CEP85L

Rejected	23	7.81180067461008e-09	0.31570006005081	0.797	0.666	0.000261992171025073	CAMK4
Rejected	23	7.84272208656328e-09	0.265197215923436	0.62	0.466	0.000263029213339159	ITSN2
Rejected	23	7.97181929165024e-09	0.412195516435731	0.559	0.417	0.000267358875403366	NEK7
Rejected	23	8.03321509810481e-09	0.611348812950628	0.437	0.3	0.000269417967960239	FILIP1L
Rejected	23	8.64199615343598e-09	0.317957451668047	0.418	0.281	0.000289835266993936	CHD7
Rejected	23	8.66978039047923e-09	0.250700095291103	0.603	0.447	0.000290767094735892	DLG1
Rejected	23	9.07101414945404e-09	0.26142901834405	0.7	0.532	0.00030422367254439	VTI1A
Rejected	23	9.41973949292186e-09	0.257079758511141	0.502	0.365	0.000315919223113613	TMEM131
Rejected	23	9.79209209671597e-09	0.272149471966298	0.629	0.48	0.00032840718473966	FNDC3A
Rejected	23	9.86663656179862e-09	0.309800661639635	0.487	0.346	0.000330907257009602	WWP1
Rejected	23	1.08647544918739e-08	0.251467583605315	0.378	0.254	0.000364382136148465	RLIM
Rejected	23	1.21367630413941e-08	0.253869150288659	0.276	0.164	0.000407042758882274	RPGR
Rejected	23	1.24793799993547e-08	0.610385767991439	0.357	0.238	0.000418533446418356	TAF4B
Rejected	23	1.26436968147301e-08	0.267792657707323	0.793	0.668	0.000424044303772419	HIVEP2
Rejected	23	1.28575838683468e-08	0.296733593571636	0.681	0.533	0.000431217647776616	MKLN1
Rejected	23	1.32906643314046e-08	0.314047884842549	0.804	0.68	0.000445742300346646	OSBPL8
Rejected	23	1.35187060537738e-08	0.273174390185199	0.312	0.198	0.000453390363631465	DNTTIP2
Rejected	23	1.35843343311512e-08	0.275345180954467	0.869	0.785	0.00045559140479815	STAT4
Rejected	23	1.38622608075415e-08	0.26830237692974	0.527	0.375	0.000464912502963327	DENND1A
Rejected	23	1.42626630317458e-08	0.298295225990199	0.593	0.447	0.00047834119275869	NUFIP2
Rejected	23	1.4545933292265e-08	0.259284043367317	0.762	0.614	0.000487841510755983	HNRNPA3
Rejected	23	1.4842311674228e-08	0.276244374917746	0.289	0.174	0.000497781448930259	UHRF1BP1L
Rejected	23	1.60071472546852e-08	0.308118382714007	0.441	0.311	0.000536847704627631	WDR7
Rejected	23	1.73695902074337e-08	0.357409349918765	0.614	0.478	0.000582541316376912	PIK3R1
Rejected	23	1.75075498762938e-08	0.26483949685932	0.612	0.46	0.000587168207751142	BIRC3
Rejected	23	1.89640193175847e-08	0.299573903481495	0.658	0.518	0.000636015279873157	FBXL17
Rejected	23	1.91525455576074e-08	0.294683810379275	0.272	0.165	0.000642338072911038	NUS1
Rejected	23	1.95409625361463e-08	0.257687038716742	0.728	0.564	0.000655364801537275	LCP1
Rejected	23	1.96765685618532e-08	0.376886695097681	0.333	0.214	0.000659912756427432	CEP128
Rejected	23	2.0013702461713e-08	0.362682572831635	0.639	0.521	0.00067121955316093	NSD3
Rejected	23	2.20868816913067e-08	0.501380294269458	0.255	0.155	0.000740749838163043	AOAH

Rejected	23	2.33899036309904e-08	0.283973348911373	0.441	0.307	0.000784450587976156	NUP58
Rejected	23	2.43311584640119e-08	0.253784345140168	0.901	0.817	0.000816018392566031	HNRNPA2B1
Rejected	23	2.59637020004577e-08	0.272776191614624	0.586	0.435	0.000870770637691352	MYLIP
Rejected	23	2.71603432902285e-08	0.284361859847167	0.27	0.162	0.000910903593267682	GOLGA8B
Rejected	23	2.73820391998329e-08	0.261366034497553	0.329	0.211	0.000918338830683995	SRP72
Rejected	23	2.85812206990319e-08	0.272011678795804	0.595	0.447	0.000958556979804131	CREBBP
Rejected	23	3.03869891882815e-08	0.25602631629479	0.532	0.384	0.00101911884339659	UBR1
Rejected	23	3.09557810797941e-08	0.27169343396876	0.561	0.416	0.00103819498585413	DNAJC1
Rejected	23	3.23675926471322e-08	0.269200609678462	0.498	0.362	0.00108554432219952	RNF111
Rejected	23	3.24763582834017e-08	0.272960473476041	0.572	0.422	0.00108919210410873	IKZF3
Rejected	23	3.26601046365313e-08	0.270407917622244	0.578	0.439	0.00109535458929999	SOS1
Rejected	23	3.2752272603533e-08	0.28398935124996	0.281	0.175	0.00109844571857729	SLC36A4
Rejected	23	3.39905869349424e-08	0.373888346168505	0.487	0.355	0.0011399763046241	MBNL2
Rejected	23	3.415021585261e-08	0.315253840174989	0.58	0.44	0.00114532993926483	DIAPH1
Rejected	23	3.73136946347757e-08	0.343222443144572	0.437	0.306	0.00125142669066111	DNAJB14
Rejected	23	3.89911110907484e-08	0.338868919723051	0.654	0.52	0.00130768388376152	CRY1
Rejected	23	4.12660350880748e-08	0.260718947088033	0.508	0.372	0.00138398028478385	GNAI3
Rejected	23	4.18407585553518e-08	0.409529885604187	0.329	0.217	0.00140325536042939	FER
Rejected	23	4.18544763266107e-08	0.341433808494171	0.384	0.263	0.00140371542704187	AGTPBP1
Rejected	23	4.1945615986159e-08	0.302653110953919	0.515	0.391	0.0014067720689438	PPP4R3B
Rejected	23	4.21878228100613e-08	0.291924554180007	0.422	0.292	0.00141489520140384	SCFD2
Rejected	23	4.24135063029837e-08	0.281277087780918	0.316	0.204	0.00142246417438947	TAF2
Rejected	23	4.3190666216599e-08	0.277628132583521	0.618	0.477	0.0014485285635723	TAB2
Rejected	23	4.34194712362467e-08	0.28023274139384	0.549	0.409	0.00145620222632124	CEP350
Rejected	23	4.47668292412277e-08	0.342129185494439	0.327	0.215	0.00150138991909229	CSPP1
Rejected	23	4.6263670114713e-08	0.283803529100805	0.734	0.585	0.00155159096830724	CCDC91
Rejected	23	4.96658271666489e-08	0.29814936441006	0.253	0.152	0.00166569251151507	ALMS1
Rejected	23	5.10585676245174e-08	0.278689867539497	0.27	0.167	0.00171240224099107	ICE1
Rejected	23	5.19599313104274e-08	0.281788614874092	0.352	0.236	0.00174263217628911	SEC63
Rejected	23	5.36244160145593e-08	0.360009695609788	0.599	0.473	0.00179845566429629	ARID2
Rejected	23	5.41304674180758e-08	0.260460778240071	0.287	0.179	0.00181542761626743	ARMH4

Rejected	23	5.45758628591898e-08	0.367660552849971	0.346	0.229	0.00183036528857151	AGPAT4
Rejected	23	5.46515308950239e-08	0.422105890120749	0.278	0.177	0.00183290304315731	SELL
Rejected	23	5.50266440379182e-08	0.273312305456493	0.591	0.441	0.0018454835877437	IFI16
Rejected	23	5.52879141917958e-08	0.263719087091177	0.865	0.748	0.00185424606616445	UTRN
Rejected	23	6.2542081815762e-08	0.251131059048117	0.323	0.207	0.00209753633993703	DTNB
Rejected	23	6.27583108657893e-08	0.319176837924681	0.378	0.258	0.00210478822981684	HDAC4
Rejected	23	6.30815622040322e-08	0.298088779911173	0.589	0.468	0.00211562943319883	XRCC5
Rejected	23	6.40416359610154e-08	0.281460996476983	0.27	0.167	0.00214782838686053	NUP160
Rejected	23	6.5243898278178e-08	0.274549257977301	0.414	0.29	0.00218814986045353	CCNK
Rejected	23	6.74796869932556e-08	0.277430067916376	0.344	0.226	0.0022631337423798	EXOC6
Rejected	23	7.80641374462948e-08	0.263608212240656	0.599	0.447	0.00261811504167383	DHX36
Rejected	23	8.45874150453963e-08	0.319675251402231	0.713	0.562	0.0028368927257925	CAST
Rejected	23	9.51041579482277e-08	0.351474802326309	0.517	0.396	0.00318960324926766	ITFG1
Rejected	23	1.02166535357982e-07	0.263356784107811	0.249	0.148	0.003426461262836	AC096558.2
Rejected	23	1.05828250151522e-07	0.269064055459986	0.707	0.56	0.00354926785358174	ATRX
Rejected	23	1.14305866195674e-07	0.270929839923641	0.338	0.226	0.00383359014047051	PIK3CA
Rejected	23	1.26927349238413e-07	0.274575543000816	0.791	0.684	0.00425688943875789	EIF4A2
Rejected	23	1.31390361332871e-07	0.254672238213743	0.553	0.419	0.00440656993838183	GNAQ
Rejected	23	1.4102466578756e-07	0.25586328682178	0.589	0.46	0.00472968524118319	BABAM2
Rejected	23	1.49897655674815e-07	0.270011499367413	0.89	0.825	0.00502726757602195	HSP90AB1
Rejected	23	1.59052802020963e-07	0.34117611128029	0.473	0.344	0.00533431287417904	TAF3
Rejected	23	1.68757133818786e-07	0.360965516159097	1	0.974	0.00565977675401444	MT-ND2
Rejected	23	1.69661147001012e-07	0.329450965680226	0.348	0.232	0.00569009554811993	SRBD1
Rejected	23	1.7221665635985e-07	0.297420672623476	0.576	0.457	0.00577580222099665	RAB7A
Rejected	23	1.93963942021981e-07	0.255121317680782	0.582	0.455	0.00650516268753318	SERBP1
Rejected	23	1.97892525050345e-07	0.255700439358851	0.622	0.479	0.00663691950513846	YWHAB
Rejected	23	2.11192527214016e-07	0.261241324387532	0.648	0.518	0.00708297497770367	ATP11B
Rejected	23	2.27224895608707e-07	0.254724564297104	0.346	0.234	0.00762066854892482	BRD1
Rejected	23	2.46801028972467e-07	0.363364584265521	0.346	0.238	0.00827721290967859	CPQ
Rejected	23	2.60697738256677e-07	0.319570962961414	0.289	0.181	0.00874328074565244	RNF24
Rejected	23	2.74029932523507e-07	0.395745296510285	0.544	0.421	0.00919041587697336	CHORDC1

Rejected	23	2.98772681133394e-07	0.309115046997001	0.504	0.383	0.0100202381798518	RB1CC1
Rejected	23	2.99307694122374e-07	0.32664676611109	0.745	0.626	0.0100381814454762	ATP2B1
Rejected	23	3.27997311194233e-07	0.252043495419322	0.403	0.284	0.0110003738228322	DNM1L
Rejected	23	3.3487025043895e-07	0.374275210125089	0.3	0.196	0.0112308784592215	SPDYA
Rejected	23	3.58561976927367e-07	0.25262663587979	0.791	0.647	0.01202545158219	RIPOR2
Rejected	23	3.61169052647708e-07	0.30658987149101	0.329	0.225	0.0121128876876988	P2RY8
Rejected	23	3.94407972758592e-07	0.285808685583323	0.297	0.196	0.0132276545903776	NSMAF
Rejected	23	3.99350529248781e-07	0.277231933001277	0.635	0.505	0.0133934180499456	BBX
Rejected	23	4.94211689966212e-07	0.279412763027842	0.762	0.651	0.0165748716580868	AKT3
Rejected	23	5.04406772862354e-07	0.25359156842606	0.544	0.411	0.0169167943482576	CDK13
Rejected	23	5.11298027132652e-07	0.300273383898841	0.738	0.623	0.0171479132339749	MAPRE2
Rejected	23	5.97056682476044e-07	0.290153537798823	0.464	0.345	0.0200240870168816	HIVEP1
Rejected	23	6.04926426167119e-07	0.25050989231161	0.215	0.13	0.0202880224807928	NOLC1
Rejected	23	6.54430853201279e-07	0.314837119282305	0.397	0.284	0.0219483019546645	VEZT
Rejected	23	6.92562212969223e-07	0.253845127331074	0.274	0.176	0.0232271514985618	VRK2
Rejected	23	7.6940573031365e-07	0.283182864875039	0.517	0.398	0.0258043293832592	PKN2
Rejected	23	7.99782064344387e-07	0.309158182804277	0.359	0.253	0.0268230908739821	ZBTB11
Rejected	23	8.02434634593298e-07	0.429240978547301	0.327	0.226	0.02691205277499	PELI2
Rejected	23	8.65434830631693e-07	0.314398187645782	0.511	0.393	0.0290249533497257	CLASP2
Rejected	23	8.69387999534072e-07	0.256315488137568	0.696	0.591	0.0291575347283737	PAN3
Rejected	23	8.88930135825724e-07	0.256416077112472	0.454	0.333	0.0298129388953231	NUTM2B-AS1
Rejected	23	9.06565348297507e-07	0.263093488018648	0.629	0.497	0.0304043886512018	NCOR1
Rejected	23	1.01111164824272e-06	0.277969439455188	0.684	0.564	0.0339106624587642	CD55
Rejected	23	1.02066330711694e-06	0.326079606081564	0.376	0.268	0.0342310059940878	ELK3
Rejected	23	1.09609820888683e-06	0.261740972913631	0.565	0.44	0.0367609417296464	ZNF407
Rejected	23	1.11908080207002e-06	0.359830014012992	0.253	0.164	0.0375317319398243	CSGALNACT1
Rejected	23	1.18138203326229e-06	0.256740399794498	0.696	0.557	0.0396211906315507	SIPA1L1
Rejected	27	2.25718798876674e-41	0.581344823383465	0.933	0.935	7.57015707672589e-37	RPL39
Rejected	27	1.50619933671822e-21	0.450706552761488	0.935	0.958	5.05149133548555e-17	JUND
Rejected	27	1.10228446270871e-18	0.279570665676598	1	1	3.69684163103247e-14	MALAT1
Rejected	27	4.55723427832415e-18	0.34319561756619	0.921	0.935	1.52840523226435e-13	RPS28

Rejected	27	4.79580870755251e-16	0.308916033600089	0.965	0.989	1.60841832433896e-11	B2M
Rejected	27	6.24052771070381e-16	0.423237989487942	0.853	0.892	2.09294818361584e-11	RPS11
Rejected	27	5.89211977661958e-15	0.394206678782041	0.925	0.954	1.97609913068267e-10	TMSB4X
Rejected	27	3.95618928925154e-13	0.323859761911729	0.893	0.921	1.32682676382918e-08	RPLP2
Rejected	27	4.08213104820217e-12	0.284326954476008	0.956	0.964	1.36906511094604e-07	RPLP1
Rejected	27	4.84834862777789e-12	0.324474871691153	0.837	0.889	1.62603916278415e-07	RPS29
Rejected	27	9.32402537869463e-12	0.300784786826128	0.893	0.919	3.12709163150661e-07	RPL30
Rejected	27	1.0805399700035e-11	0.362562360351648	0.839	0.88	3.62391495139774e-07	RPL27A
Rejected	27	1.7826453051173e-11	0.296630369640794	0.868	0.904	5.97863582430239e-07	RPL37
Rejected	27	2.31202000071877e-11	0.404917298373124	0.824	0.856	7.7540526784106e-07	CXCR4
Rejected	27	4.19298342389646e-11	0.270623473214339	0.906	0.936	1.40624278070639e-06	RPS27
Rejected	27	7.90194790142485e-11	0.302892930945537	0.908	0.941	2.65015528717987e-06	RPS19
Rejected	27	2.41415137665288e-10	0.282958927742811	0.894	0.913	8.09658088701844e-06	RPS27A
Rejected	27	2.92584916487946e-10	0.252998624260959	0.919	0.937	9.81271292917273e-06	RPL41
Rejected	27	1.25193644165793e-09	0.335778048937527	0.195	0.359	4.19874443803236e-05	EGR1
Rejected	27	1.68121649567996e-09	0.415874326530822	0.76	0.823	5.63846388321145e-05	RPL27
Rejected	27	3.42990100568914e-09	0.263812739144742	0.891	0.931	0.000115032019928802	RPL13A
Rejected	27	6.93591591041847e-09	0.343910799518287	0.903	0.956	0.000232616747803615	KLF6
Rejected	27	9.09954693225405e-09	0.667823321666909	0.516	0.466	0.000305180605013936	KLRB1
Rejected	27	1.27661786986435e-08	0.305154456641628	0.957	0.984	0.000428152101195106	MBNL1
Rejected	27	1.77406698558121e-08	0.250495590266596	0.897	0.922	0.000594986585624226	RPL34
Rejected	27	3.45295372403512e-08	0.291196679654443	0.868	0.913	0.0011580516199669	RPL9
Rejected	27	9.90246941223139e-08	0.312470839351602	0.799	0.844	0.00332109019147416	RPL23A
Rejected	27	2.76154710733662e-07	0.296285241675641	0.767	0.822	0.00926167668858556	RPS21
Rejected	27	3.47898029583114e-07	0.600939919154532	0.482	0.433	0.0116678041161585	PLCB1
Rejected	27	6.27330691009362e-07	0.257094290600197	0.85	0.894	0.021039416715072	RPS25
Rejected	27	1.47671796525541e-06	0.268189099281471	0.887	0.961	0.0495261671187358	DDX5
Rejected	30	3.46236777737496e-149	0.765955412230489	0.287	0.005	1.16120890517601e-144	TFPI2
Rejected	30	1.52706575469994e-105	0.723175935049982	0.486	0.084	5.12147312811264e-101	NRG1
Rejected	30	2.2079397899165e-80	0.623515993615826	0.249	0.023	7.40498846742197e-76	IGFBP5
Rejected	30	4.36902246650653e-78	0.73064796120431	0.3	0.038	1.46528275481696e-73	COL4A1

Rejected	30	1.17328129480997e-66	0.558509216937214	0.252	0.031	3.93495080653369e-62	LDB2
Rejected	30	4.74134524091899e-61	0.86923258003288	0.992	0.906	1.59015236689941e-56	SIK3
Rejected	30	3.28360564042995e-47	0.713498110526598	0.947	0.689	1.1012556596874e-42	SYNE2
Rejected	30	3.44090072957275e-46	0.537069071265054	0.252	0.048	1.15400928668411e-41	COL4A2
Rejected	30	1.40353795886526e-44	0.775700312752825	1	0.979	4.70718560644232e-40	AC016831.7
Rejected	30	1.50120993918399e-44	0.451830669571516	0.204	0.032	5.03475789403527e-40	ADAMTS9
Rejected	30	2.86493852566755e-43	0.783889320490089	0.975	0.854	9.60843082738384e-39	DENND4A
Rejected	30	4.36608231864152e-43	1.02621075693646	0.534	0.224	1.46429668802599e-38	UTY
Rejected	30	6.3302008901069e-43	0.746409426052311	0.967	0.869	2.12302277452405e-38	RAB11A
Rejected	30	1.34632754311233e-41	0.791522712015227	0.97	0.876	4.51531331409015e-37	CD44
Rejected	30	1.33104725644838e-40	0.530900973055052	0.262	0.059	4.46406628867656e-36	ADGRL2
Rejected	30	3.59576689149675e-40	0.783986213027263	0.907	0.642	1.20594830007018e-35	DYRK1A
Rejected	30	1.91706665778747e-39	0.773294597963655	0.537	0.221	6.42945815688762e-35	A2M
Rejected	30	2.65141068728134e-38	0.394579595578341	0.196	0.035	8.89230116300416e-34	SPARC
Rejected	30	3.02761525170497e-38	0.509126641596039	0.249	0.057	1.01540160311681e-33	COL3A1
Rejected	30	1.02415359787041e-37	0.63686406795218	0.506	0.206	3.43480633653779e-33	TIMP1
Rejected	30	1.13759664024668e-36	0.723667672802118	0.975	0.901	3.8152716120593e-32	FYN
Rejected	30	8.49820713239376e-36	0.325731149099895	0.156	0.023	2.85012870806222e-31	COL1A2
Rejected	30	5.77605675435243e-35	0.436659462970381	0.169	0.028	1.93717391427472e-30	CEMIP
Rejected	30	9.55884817695018e-35	0.504101804889366	0.264	0.069	3.20584650158555e-30	AC008937.2
Rejected	30	9.63647002001733e-35	0.375455763551184	0.161	0.026	3.23187931531341e-30	COL6A3
Rejected	30	1.1258290430665e-34	0.767908564188315	0.776	0.484	3.77580544463643e-30	AHNAK
Rejected	30	3.92019173153928e-34	0.48597754058194	0.191	0.038	1.31475390292364e-29	PRR16
Rejected	30	4.05127747613436e-34	0.992387937108919	0.829	0.588	1.35871743994594e-29	AP001011.1
Rejected	30	4.69442938269255e-34	0.430796313103874	0.234	0.055	1.57441772636743e-29	IGFBP7
Rejected	30	1.45812037205936e-33	0.478412483799192	0.34	0.107	4.89024410381267e-29	SPARCL1
Rejected	30	7.61254049981124e-33	0.656253104417841	0.602	0.299	2.55309383282669e-28	AC058822.1
Rejected	30	9.65061840930039e-33	0.713657587753813	0.897	0.701	3.23662440211116e-28	CYTH1
Rejected	30	2.86320975208336e-32	0.388404466652912	0.249	0.064	9.60263286653718e-28	IGFBP4
Rejected	30	3.17078165453524e-32	0.646815078035608	0.771	0.47	1.06341675129803e-27	SND1
Rejected	30	4.06359437844758e-32	0.371170356970193	0.108	0.011	1.36284828264375e-27	MAP1B

Rejected	30	4.36230940762923e-32	0.681181799338045	0.408	0.157	1.46303132913069e-27	CD44-AS1
Rejected	30	6.83529426774816e-32	0.877508113040196	0.839	0.615	2.29242099151738e-27	ATP2B1
Rejected	30	1.03098016736076e-31	0.780067419805947	0.864	0.645	3.45770128529451e-27	RFX3
Rejected	30	1.65001638008419e-31	0.539806666629867	0.688	0.366	5.53382493552637e-27	IGLC3
Rejected	30	3.17679251357292e-30	0.457989929423968	0.224	0.057	1.06543267320209e-25	RBPMS
Rejected	30	6.04653533900497e-30	0.547682289313211	0.962	0.836	2.02788702199549e-25	ARIH1
Rejected	30	1.19574666222577e-29	0.802631669294094	0.479	0.217	4.01029515577278e-25	DDX3Y
Rejected	30	1.25090215120666e-29	0.704386926873239	0.521	0.25	4.19527563471688e-25	SLCO3A1
Rejected	30	1.89042394205275e-29	0.612383763512177	0.826	0.591	6.3401038168565e-25	ANKRD11
Rejected	30	2.50456481703011e-29	0.764650908860974	0.864	0.657	8.39980948335558e-25	CHD2
Rejected	30	3.9614874461122e-29	0.728394809379949	0.917	0.756	1.32860365967711e-24	ESYT2
Rejected	30	4.28865770910767e-29	0.287874233672111	0.121	0.017	1.43833002248053e-24	NXN
Rejected	30	1.56115284315495e-28	0.844742020893613	0.773	0.552	5.23579440537308e-24	PRKCA
Rejected	30	1.72756004797668e-28	0.608160404127002	0.469	0.215	5.79389088890419e-24	WDR48
Rejected	30	2.16433479805178e-28	0.595860141992057	0.277	0.087	7.25874604570608e-24	AL589990.1
Rejected	30	2.39061099223325e-28	0.570301652632262	0.955	0.79	8.01763114575189e-24	ARID4B
Rejected	30	2.94377851778843e-28	0.836160141060835	0.751	0.511	9.87284439295882e-24	ZHX2
Rejected	30	6.83879685609107e-28	0.279173723397188	0.111	0.015	2.29359568959582e-23	SELE
Rejected	30	6.96840704263534e-28	0.615072031550633	0.166	0.035	2.33706435395904e-23	RGS5
Rejected	30	7.72166678859447e-28	0.844697073906211	0.547	0.302	2.58969260755881e-23	USP3-AS1
Rejected	30	9.76333570630229e-28	0.265676672696486	0.134	0.022	3.27442752917966e-23	COL1A1
Rejected	30	1.97718807458884e-27	0.463719907104383	0.985	0.936	6.63109336455604e-23	ARHGAP15
Rejected	30	3.43788525573803e-27	0.478551211069807	0.945	0.833	1.15299795706942e-22	ANKRD44
Rejected	30	5.99398917126688e-27	0.513199346341824	0.365	0.146	2.01026408825949e-22	AC124319.2
Rejected	30	6.19802245099814e-27	0.689503534198981	0.861	0.665	2.07869276961576e-22	BRAF
Rejected	30	7.43337900813535e-27	0.581546565528868	0.325	0.122	2.49300665174843e-22	AF241726.2
Rejected	30	1.08120016419811e-26	0.689395754198369	0.61	0.335	3.62612911068761e-22	FNDC3B
Rejected	30	2.27076781311563e-26	0.70536277399838	0.534	0.287	7.61570109162719e-22	RASA3
Rejected	30	2.5475917083927e-26	0.512210081044305	0.703	0.428	8.54411307160743e-22	IGLC2
Rejected	30	4.59990479187057e-26	0.572387706087283	0.861	0.624	1.54271606909755e-21	CDK17
Rejected	30	5.13321391086173e-26	0.364827879961346	0.154	0.032	1.72157728142481e-21	TMTC1

Rejected	30	7.33055314040002e-26	0.57923882339175[illegible]	0.829	0.588	2.45852091222736e-21	KDM4C
Rejected	30	7.49034359411081e-26	0.61490664985520[illegible]	0.771	0.499	2.51211143459288e-21	RPS6KA5
Rejected	30	9.70359706759685e-26	0.32856344902369[illegible]	0.176	0.042	3.25439238453063e-21	ADGRL3
Rejected	30	1.06705476969785e-25	0.63089344715615[illegible]	0.448	0.209	3.57868828661266e-21	ESR2
Rejected	30	1.13814796130192e-25	0.53618651311645[illegible]	0.851	0.621	3.81712063261438e-21	KAT6A
Rejected	30	1.44939148102581e-25	0.48986032266901	0.917	0.726	4.86096914906437e-21	PRRC2C
Rejected	30	1.47670686236175e-25	0.50324635322483[illegible]	0.36	0.144	4.95257947498885e-21	AC090627.1
Rejected	30	3.0286609710815e-25	0.49526265201289[illegible]	0.413	0.178	1.01575231648132e-20	IFITM3
Rejected	30	3.16415396967694e-25	0.56522345039812[illegible]	0.947	0.843	1.06119395835025e-20	ZBTB20
Rejected	30	3.34907856690109e-25	0.38883786505895[illegible]	0.204	0.056	1.12321396976729e-20	CALD1
Rejected	30	3.42978368380425e-25	0.54888768685759[illegible]	0.569	0.312	1.15028085187427e-20	RELB
Rejected	30	3.94278108063396e-25	0.53249703152553[illegible]	0.368	0.152	1.32232991882302e-20	AC079793.1
Rejected	30	4.42365313780163e-25	0.59255313378343[illegible]	0.919	0.743	1.48360478935591e-20	UTRN
Rejected	30	4.82245870860277e-25	0.83852656129683[illegible]	0.766	0.539	1.6173562016912e-20	RORA-AS1
Rejected	30	5.84112219134531e-25	0.53844320944428[illegible]	0.501	0.249	1.95899556053339e-20	AC008569.1
Rejected	30	6.46365416298722e-25	0.60567180787914[illegible]	0.836	0.608	2.16778033318265e-20	HERC1
Rejected	30	8.48969317613501e-25	0.58875840992112[illegible]	0.411	0.186	2.84727329741216e-20	ACSL4
Rejected	30	1.37720875400416e-24	0.70700279968801[illegible]	0.783	0.583	4.61888271917914e-20	USP3
Rejected	30	1.48477854944432e-24	0.3946980719019	0.214	0.063	4.97965029912637e-20	PLCG1-AS1
Rejected	30	1.5944247864782e-24	0.73047108878799[illegible]	0.844	0.664	5.3473818488906e-20	HIVEP2
Rejected	30	2.11732674453887e-24	0.63653569430393[illegible]	0.919	0.774	7.10109043583445e-20	GNG2
Rejected	30	2.18363856399158e-24	0.61885152997674[illegible]	0.572	0.306	7.32348701591497e-20	AC009313.1
Rejected	30	3.61220227827461e-24	0.52955242749605	0.781	0.52	1.21146040008774e-19	DOCK2
Rejected	30	3.61846021571439e-24	0.63958829664114[illegible]	0.861	0.7	1.21355918714629e-19	IQGAP2
Rejected	30	4.9358064940562e-24	0.62470244150457[illegible]	0.693	0.434	1.65537078197657e-19	TRAPPC9
Rejected	30	6.28172235807488e-24	0.58375960278602[illegible]	0.806	0.567	2.10676404445115e-19	ZNF644
Rejected	30	7.07665841685247e-24	0.72164324900259[illegible]	0.582	0.334	2.37336969984398e-19	LMBR1
Rejected	30	7.38565072581015e-24	0.64870272579146[illegible]	0.579	0.331	2.47699954042221e-19	SORL1
Rejected	30	7.45590670811186e-24	0.65884369298565	0.768	0.532	2.50056199176655e-19	BICDL1
Rejected	30	8.1164933156519e-24	0.56803139509306[illegible]	0.645	0.378	2.72210952820333e-19	SCAF8
Rejected	30	1.01864098575263e-23	0.56383120010720[illegible]	0.877	0.692	3.41631813801716e-19	DOCK10

Rejected	30	1.18789229307801e-23	0.48735363590685	0.927	0.794	3.98395317252503e-19	TNRC6B
Rejected	30	1.35058252493609e-23	0.52407012559971	0.869	0.662	4.52958367213066e-19	SLC38A1
Rejected	30	1.3788524942703e-23	0.74513291560750	0.809	0.577	4.62439549528373e-19	LINC00513
Rejected	30	3.55822073074241e-23	0.61298603657624	0.809	0.596	1.19335606867639e-18	ZSWIM6
Rejected	30	3.69750424739085e-23	0.75105840188486	0.831	0.67	1.24006897448994e-18	BACH2
Rejected	30	3.84726205142019e-23	0.65395237693943	0.564	0.308	1.2902947468053e-18	AL031772.1
Rejected	30	4.25399719433979e-23	0.59589952102024	0.846	0.672	1.42670557903768e-18	ANK3
Rejected	30	4.95957196467625e-23	0.45854273124192	0.466	0.229	1.66334124551312e-18	IGHM
Rejected	30	6.25588212869518e-23	0.41024091854431	0.224	0.072	2.09809774832179e-18	GLIS3
Rejected	30	6.29618711319797e-23	0.54389008499634	0.63	0.37	2.11161523402434e-18	XPR1
Rejected	30	6.75279099466313e-23	0.54309645396026	0.393	0.177	2.26475104379012e-18	HIF1A-AS2
Rejected	30	7.00562141958229e-23	0.53400746497380	0.741	0.512	2.34954531169951e-18	GNB1
Rejected	30	7.60616568763461e-23	0.55423287575172	0.846	0.66	2.5509558483189e-18	ELMO1
Rejected	30	7.79246569020517e-23	0.38911379376502	0.214	0.066	2.61343714318101e-18	FAM217A
Rejected	30	2.49262832028245e-22	0.44865532640146	0.355	0.149	8.35977686056329e-18	PTPRM
Rejected	30	2.5817528342114e-22	0.53270231851911	0.607	0.363	8.65868265537819e-18	AGO2
Rejected	30	2.8544115563446e-22	0.62372810183473	0.738	0.491	9.57312547766852e-18	RAPGEF2
Rejected	30	3.28419646085402e-22	0.54713901438054	0.929	0.855	1.10145380904122e-17	FOXP1
Rejected	30	3.54751704517375e-22	0.43523100208231	0.97	0.859	1.18976626661037e-17	HNRNPC
Rejected	30	4.25255298519197e-22	0.45044903979737	0.992	0.976	1.42622122017368e-17	MBNL1
Rejected	30	4.29440785693553e-22	0.54867343943131	0.819	0.623	1.44025850705904e-17	KMT2C
Rejected	30	5.49275390561841e-22	0.46824250082725	0.305	0.124	1.8421598048663e-17	AC109587.1
Rejected	30	7.41545543349685e-22	0.63246589356245	0.909	0.782	2.48699544328617e-17	STAT4
Rejected	30	8.032066132291e-22	0.62043905716214	0.683	0.45	2.69379433944776e-17	BABAM2
Rejected	30	8.24052969950553e-22	0.63908712746961	0.859	0.701	2.76370885062017e-17	PITPNC1
Rejected	30	9.21616591650718e-22	0.55947744217927	0.509	0.274	3.09091772507818e-17	TP53BP2
Rejected	30	1.01166481417157e-21	0.51585349868844	0.801	0.561	3.39292145376862e-17	CASK
Rejected	30	1.29907758781984e-21	0.56540572051182	0.64	0.394	4.35684641403019e-17	USP36
Rejected	30	1.31906702124179e-21	0.51848274890872	0.927	0.794	4.42388697584073e-17	PDE3B
Rejected	30	1.54973509366238e-21	0.64125223418327	0.773	0.566	5.1975015571249e-17	BTBD9
Rejected	30	1.93446925627343e-21	0.32736623918530	0.275	0.101	6.48782299168983e-17	TCF4

Rejected	30	3.36628576532707e-21	0.545885674581938	0.819	0.596	1.12898491997539e-16	FBXW7
Rejected	30	3.54608313967068e-21	0.617944381760562	0.819	0.616	1.18928536338275e-16	CHST11
Rejected	30	3.69340058365185e-21	0.508245274869459	0.69	0.437	1.23869268774516e-16	CREBBP
Rejected	30	3.74156167583802e-21	0.645364880437323	0.539	0.316	1.25484495484256e-16	RFTN1
Rejected	30	4.33619203295043e-21	0.531802079514782	0.645	0.4	1.45427208401091e-16	MARCH7
Rejected	30	5.79227865855738e-21	0.512351983297284	0.95	0.817	1.94261441650697e-16	JMJD1C
Rejected	30	1.0196332617555e-20	0.566926471985129	0.695	0.474	3.41964603327561e-16	EPC2
Rejected	30	1.15546306177404e-20	0.512853985693904	0.897	0.723	3.87519201657778e-16	ARID1B
Rejected	30	1.26427991572106e-20	0.567526803799816	0.834	0.643	4.24014198134529e-16	AKT3
Rejected	30	1.29209693508319e-20	0.459239299525927	0.869	0.679	4.33343470088199e-16	STAG1
Rejected	30	1.3001640240218e-20	0.644153542135815	0.395	0.191	4.3604901037643e-16	DPYD-AS1
Rejected	30	2.03046261399064e-20	0.421495898747164	0.451	0.226	6.80976551480182e-16	TACC1
Rejected	30	2.13829911783481e-20	0.460795232260554	0.322	0.136	7.17142758139438e-16	EPAS1
Rejected	30	2.30084610592856e-20	0.528923272084254	0.95	0.819	7.71657767006319e-16	SMCHD1
Rejected	30	2.57501468138998e-20	0.765308347743357	0.574	0.362	8.63608423844572e-16	TBL1X
Rejected	30	2.59190456144547e-20	0.757335333202971	0.811	0.626	8.69272951817581e-16	CDC14A
Rejected	30	2.81120955745873e-20	0.593685191790684	0.423	0.214	9.42823461380507e-16	PELI2
Rejected	30	2.84747400507579e-20	0.433341805302547	0.806	0.558	9.54985831822318e-16	CSDE1
Rejected	30	3.59814043452679e-20	0.366238368301007	0.171	0.049	1.2067443389316e-15	DOCK4
Rejected	30	3.85139666804105e-20	0.486022029060218	0.907	0.723	1.29168141452761e-15	LRBA
Rejected	30	3.85702290248647e-20	0.539233037020707	0.753	0.536	1.29356834103591e-15	GOLGB1
Rejected	30	3.88856954784172e-20	0.57630704074723	0.814	0.63	1.30414845495516e-15	LRRFIP1
Rejected	30	4.46945961061219e-20	0.627452062330569	0.824	0.659	1.49896736420712e-15	NFKB1
Rejected	30	4.48458562420664e-20	0.535606944264533	0.819	0.618	1.50404032664642e-15	MACF1
Rejected	30	4.76897013342035e-20	0.621825069996072	0.778	0.566	1.59941720334652e-15	RBMS1
Rejected	30	4.77759101148635e-20	0.666124773846931	0.302	0.127	1.60230847343229e-15	DOK6
Rejected	30	4.89026837379116e-20	0.483604378804318	0.61	0.372	1.64009820720208e-15	SMG6
Rejected	30	5.97676613674645e-20	0.784918565771168	0.761	0.562	2.00448782694202e-15	ELOVL5
Rejected	30	6.61147147716655e-20	0.453731097760863	0.952	0.82	2.21735530401212e-15	PARP8
Rejected	30	6.66988148733229e-20	0.491852441402033	0.73	0.477	2.2369448532215e-15	UBR5
Rejected	30	6.89916455698291e-20	0.260561530847379	0.113	0.023	2.31384180912093e-15	RNF32

Rejected	30	7.12992079480986e-20	0.47352475194450[illegible]	0.499	0.275	2.39123283616333e-15	SAFB
Rejected	30	7.2409602772304e-20	0.2640388069006	0.116	0.024	2.42847325777753e-15	HPSE2
Rejected	30	7.84836799164292e-20	0.68296132889917[illegible]	0.824	0.636	2.6321856570372e-15	TANK
Rejected	30	8.21106590250868e-20	0.53289680047767[illegible]	0.902	0.775	2.75382728238336e-15	KMT2E
Rejected	30	8.91786145445731e-20	0.46565977653936[illegible]	0.307	0.13	2.99087237459589e-15	AL035634.1
Rejected	30	1.04944410699775e-19	0.48401681153483[illegible]	0.607	0.367	3.51962564604905e-15	MARK3
Rejected	30	1.41562288384923e-19	0.43474518252772[illegible]	0.305	0.128	4.74771602785353e-15	AC009554.1
Rejected	30	1.76829839621108e-19	0.53343914967209[illegible]	0.602	0.368	5.93051916121271e-15	DOCK9
Rejected	30	1.91206595512271e-19	0.49127926916538[illegible]	0.305	0.13	6.41268680029054e-15	USP9Y
Rejected	30	2.16940158814462e-19	0.46997020708240[illegible]	0.733	0.5	7.27573904631941e-15	KDM2A
Rejected	30	2.27340901767678e-19	0.50308070552734[illegible]	0.693	0.461	7.6245591634844e-15	DHX9
Rejected	30	2.58144739028558e-19	0.43648364804360[illegible]	0.695	0.449	8.65765825753978e-15	RAB1A
Rejected	30	2.62206311184946e-19	0.52788143790368[illegible]	0.788	0.58	8.79387526452072e-15	ZEB1
Rejected	30	2.77289351652781e-19	0.56343055521060[illegible]	0.69	0.465	9.29973027573098e-15	ABCC1
Rejected	30	3.23970758226122e-19	0.52335444027282[illegible]	0.861	0.668	1.08653312893877e-14	DPYD
Rejected	30	3.58855956668604e-19	0.50283472850226[illegible]	0.343	0.157	1.20353110747517e-14	RPGR
Rejected	30	4.12046731271567e-19	0.50973564903855[illegible]	0.317	0.14	1.38192232733858e-14	AC096558.2
Rejected	30	4.3929658904116e-19	0.44421780758279[illegible]	0.365	0.173	1.47331290032624e-14	SLC9A8
Rejected	30	5.00823080894886e-19	0.47474820965568[illegible]	0.738	0.507	1.67966044870527e-14	PUM1
Rejected	30	5.8044842274966e-19	0.43898596047235[illegible]	0.491	0.271	1.94670792021781e-14	AC001226.2
Rejected	30	7.7211015925765e-19	0.43863486131003[illegible]	0.793	0.563	2.58950305211831e-14	CUL3
Rejected	30	8.07724791966241e-19	0.44411889473658	0.242	0.092	2.70894740729638e-14	AC026202.2
Rejected	30	9.07202466841968e-19	0.54571166070886[illegible]	0.917	0.765	3.04257563329459e-14	EML4
Rejected	30	9.71189594350076e-19	0.42579022918860[illegible]	0.441	0.231	3.25717566153129e-14	PI4KA
Rejected	30	9.80084174653156e-19	0.56008597955074[illegible]	0.62	0.395	3.28700630495175e-14	MAP4K4
Rejected	30	9.82881055533505e-19	0.53870987439064[illegible]	0.763	0.55	3.29638648404827e-14	PRKCB
Rejected	30	1.41184886962031e-18	0.49431558184336[illegible]	0.866	0.662	4.73505873893259e-14	PCNX1
Rejected	30	1.43874023086925e-18	0.47951527792026[illegible]	0.169	0.051	4.82524698628929e-14	DLC1
Rejected	30	1.52181812972203e-18	0.42310527380629[illegible]	0.912	0.745	5.10387364346174e-14	PPP1CB
Rejected	30	1.62561071848102e-18	0.51066098473585	0.599	0.377	5.45197322764165e-14	CNST
Rejected	30	1.67645771733566e-18	0.46393526028429[illegible]	0.786	0.554	5.62250389240033e-14	TOP1

Rejected	30	1.76942450326618e-18	0.54484018895981{	0.615	0.389	5.93429589905413e-14	RCOR1
Rejected	30	2.23708073219774e-18	0.51348549110975€	0.748	0.543	7.5027213596448e-14	RAB8B
Rejected	30	2.26481582263564e-18	0.44888360007823⁄	0.509	0.29	7.59573930595542e-14	AP1G1
Rejected	30	2.49866461393064e-18	0.43571521501702⁄	0.826	0.62	8.3800213822006e-14	USP34
Rejected	30	2.50245165214043e-18	0.4770961913939	0.816	0.588	8.39272235094857e-14	NCOA2
Rejected	30	2.78203040913623e-18	0.482938111153683	0.738	0.511	9.33037358616109e-14	RNF213
Rejected	30	2.81097805981011e-18	0.467143995387397	0.514	0.294	9.42745821699115e-14	KLHL24
Rejected	30	2.84645779375015e-18	0.470546984068667	0.788	0.592	9.54645014867925e-14	TCF12
Rejected	30	3.52088732825468e-18	0.61984369894505	0.851	0.713	1.18083519215005e-13	SNX9
Rejected	30	3.67190433613526e-18	0.47235188903711ᶓ	0.972	0.868	1.23148327625304e-13	CDC42SE2
Rejected	30	4.44895649122168e-18	0.50605347170609€	0.662	0.451	1.49209102802593e-13	ASAP1
Rejected	30	4.51219658659204e-18	0.489100584381321	0.627	0.408	1.51330049121124e-13	TRAF3
Rejected	30	5.55473096095916e-18	0.411495914788942	0.632	0.397	1.86294566968648e-13	EIF3A
Rejected	30	5.6110627945371e-18	0.33228937194850⁄	0.202	0.069	1.88183824003185e-13	LRMDA
Rejected	30	5.92253160273885e-18	0.278960325543102	0.128	0.032	1.98629864892656e-13	WWTR1
Rejected	30	5.98975732356291e-18	0.646208211358532	0.411	0.218	2.00884481117653e-13	RNF130
Rejected	30	6.25905219711108e-18	0.53567959462771€	0.637	0.413	2.09916092586711e-13	ITPR2
Rejected	30	7.70015733848554e-18	0.53781923654332€	0.71	0.498	2.58247876818128e-13	BBX
Rejected	30	8.24189143457551e-18	0.41248858980918	0.428	0.222	2.76416554932793e-13	SIN3A
Rejected	30	9.49158225856669e-18	0.54755523183571{	0.763	0.56	3.1832868578781e-13	FBXO11
Rejected	30	9.51134870933048e-18	0.489783248390267	0.829	0.652	3.18991613013526e-13	ZFAND3
Rejected	30	1.1220183717128e-17	0.463518987965003	0.829	0.635	3.7630252150504e-13	ADGRE5
Rejected	30	1.15739280368404e-17	0.45858410974889⁄	0.897	0.747	3.88166398499554e-13	GLS
Rejected	30	1.29149985457431e-17	0.550806194243767	0.778	0.607	4.33143221227131e-13	EXOC4
Rejected	30	1.29223715561816e-17	0.53800835035488€	0.617	0.408	4.3339049725122e-13	HUWE1
Rejected	30	1.51131861328085e-17	0.325092464600007	0.99	0.94	5.06866036522132e-13	MT-ND5
Rejected	30	1.51859456434448e-17	0.61438446354420ᶓ	0.71	0.525	5.09306244989853e-13	CAMK1D
Rejected	30	1.51993469381428e-17	0.37528770158509⁄	0.275	0.115	5.09755697611433e-13	AC090772.2
Rejected	30	1.64546917010598e-17	0.43453843375343ᶓ	0.67	0.444	5.51857450270145e-13	HBP1
Rejected	30	1.67247404488516e-17	0.43198779878506€	0.655	0.408	5.60914345173584e-13	LUC7L3
Rejected	30	1.81730549364476e-17	0.370230206753793	0.829	0.595	6.09487916458581e-13	SFPQ

Rejected	30	1.90195084139027e-17	0.45450927888168	0.627	0.404	6.3787627318547e-13	TNKS
Rejected	30	2.14461630087237e-17	0.420988333111981	0.516	0.301	7.19261414986577e-13	YLPM1
Rejected	30	2.2466974638628e-17	0.475755059315192	0.574	0.345	7.53497395430305e-13	TRAF1
Rejected	30	2.27354561674191e-17	0.445095490187096	0.567	0.331	7.62501728942902e-13	CA5B
Rejected	30	2.37560240074498e-17	0.462928167298206	0.594	0.366	7.9672953316185e-13	BTAF1
Rejected	30	2.70672081503423e-17	0.420487078359666	0.874	0.67	9.07780026946181e-13	DOCK8
Rejected	30	2.78072409600738e-17	0.341411381137265	0.514	0.286	9.32599247318954e-13	RAF1
Rejected	30	2.84032182962124e-17	0.459057105198318	0.695	0.473	9.52587135218373e-13	PCM1
Rejected	30	2.89182124468002e-17	0.686171360097787	0.877	0.742	9.69859009040786e-13	FAM107B
Rejected	30	3.02246403673264e-17	0.581548950951776	0.479	0.278	1.01367398863939e-12	PRKCE
Rejected	30	3.07722346037375e-17	0.541947077581258	0.673	0.473	1.03203920414015e-12	TAB2
Rejected	30	3.58998977859616e-17	0.446993061590791	0.506	0.301	1.20401077194558e-12	UBR4
Rejected	30	3.85221347829219e-17	0.375695713745013	0.809	0.577	1.29195535634964e-12	PAN3
Rejected	30	4.09067437030719e-17	0.378416306780131	0.179	0.059	1.37193037031363e-12	CLIC4
Rejected	30	4.9694110468135e-17	0.461764054519063	0.577	0.353	1.66664107688031e-12	RNF216
Rejected	30	5.78395994605319e-17	0.487806006868218	0.657	0.446	1.93982448670732e-12	GSK3B
Rejected	30	5.84590267193152e-17	0.378033706604385	0.191	0.066	1.96059883811239e-12	AP002518.2
Rejected	30	6.34905355032748e-17	0.395924097976837	0.38	0.193	2.12934557970883e-12	KDM3A
Rejected	30	6.39480029113915e-17	0.541690333013686	0.781	0.59	2.14468812164225e-12	GPCPD1
Rejected	30	6.62627298558057e-17	0.479500981247335	0.897	0.771	2.22231943390401e-12	SYTL3
Rejected	30	6.73128978890576e-17	0.50088916193539	0.773	0.573	2.25753996940321e-12	CDKAL1
Rejected	30	7.03340466280675e-17	0.662856111043355	0.778	0.595	2.35886325581213e-12	LDLRAD4
Rejected	30	7.23154017965541e-17	0.456410284896939	0.725	0.518	2.42531394545283e-12	COP1
Rejected	30	7.71562053820473e-17	0.503232556901489	0.793	0.591	2.5876648161031e-12	CD247
Rejected	30	7.83895371309999e-17	0.404795998247745	0.327	0.155	2.62902829629948e-12	FAM196B
Rejected	30	9.27835129988412e-17	0.352969789274983	0.202	0.074	3.11177345895514e-12	PECAM1
Rejected	30	9.77900534586069e-17	0.716992548591715	0.872	0.713	3.27968281289476e-12	RORA
Rejected	30	1.031434526877e-16	0.299903765564371	0.678	0.429	3.45922511624007e-12	YWHAE
Rejected	30	1.04436505913489e-16	0.419340745912829	0.476	0.268	3.50259153532661e-12	DHX15
Rejected	30	1.05628756556434e-16	0.423646018026089	0.662	0.436	3.54257723738968e-12	MT-ATP8
Rejected	30	1.12596595436237e-16	0.509320941595309	0.685	0.459	3.77626461774053e-12	PLCL2

Rejected	30	1.18981159094708e-16	0.542295855263417	0.428	0.234	3.9903901137183e-12	SIDT1
Rejected	30	1.30961416000121e-16	0.534846491833182	0.486	0.283	4.39218396981207e-12	GRK5
Rejected	30	1.53567309553837e-16	0.381304909132862	0.184	0.064	5.15034042781658e-12	AC009299.3
Rejected	30	1.57862770839708e-16	0.443078968966591	0.567	0.354	5.29440160842213e-12	INPP5D
Rejected	30	1.58306825236016e-16	0.472802557811106	0.804	0.631	5.3092943047655e-12	KDM6A
Rejected	30	1.62294247852377e-16	0.427674358985422	0.249	0.104	5.44302448447301e-12	ABCC4
Rejected	30	1.8672001262132e-16	0.480786532419889	0.69	0.49	6.26221578329384e-12	USP47
Rejected	30	1.90999083100927e-16	0.523222297083975	0.428	0.241	6.40572724903889e-12	RELL1
Rejected	30	2.0776535467468e-16	0.51000936938098	0.751	0.568	6.96803446507941e-12	PPP6R3
Rejected	30	2.08314870239156e-16	0.377101719517275	0.829	0.624	6.98646411808082e-12	WNK1
Rejected	30	2.23082974455117e-16	0.474906586382907	0.489	0.281	7.48175679727572e-12	ZC3H12D
Rejected	30	2.38833485653362e-16	0.516876751794922	0.254	0.109	8.00999744184244e-12	IFIH1
Rejected	30	2.53633076357577e-16	0.39499686654538	0.582	0.364	8.50634611488041e-12	BRD4
Rejected	30	2.58083561934601e-16	0.394868577134677	0.31	0.144	8.65560650016264e-12	IFFO2
Rejected	30	2.73203921835995e-16	0.434825489021578	0.718	0.511	9.1627131305356e-12	PICALM
Rejected	30	2.97853873542765e-16	0.77644005365246	0.549	0.362	9.98942321087724e-12	OSBPL3
Rejected	30	3.14530704009615e-16	0.389754961748934	0.71	0.494	1.05487307510745e-11	FUBP1
Rejected	30	3.20875201080408e-16	0.609676353741186	0.748	0.561	1.07615124938347e-11	MPP7
Rejected	30	3.25073172638774e-16	0.533125588673826	0.761	0.572	1.09023040639592e-11	TBC1D5
Rejected	30	3.47313478629482e-16	0.389433370426765	0.479	0.273	1.16481994462756e-11	MTFR1
Rejected	30	3.59298383919266e-16	0.555862838342324	0.741	0.555	1.20501491998843e-11	SIPA1L1
Rejected	30	3.73694575251255e-16	0.457758678754978	0.574	0.36	1.25329686647766e-11	TMEM87A
Rejected	30	3.82425895099089e-16	0.473602616609655	0.678	0.489	1.28257996698332e-11	SUCLG2
Rejected	30	4.82339372354187e-16	0.377647995520367	0.746	0.507	1.61766978700147e-11	ATP11B
Rejected	30	4.95247114172914e-16	0.438552830222574	0.411	0.224	1.66095977151312e-11	MTMR3
Rejected	30	5.16338548186486e-16	0.353158425567094	0.753	0.52	1.73169622290784e-11	GOLGA4
Rejected	30	5.30226504261515e-16	0.729093707161288	0.476	0.283	1.77827364999227e-11	PCAT1
Rejected	30	5.59923771397296e-16	0.677357302002805	0.713	0.54	1.87787234451225e-11	IMMP2L
Rejected	30	5.69047998270548e-16	0.391120564845876	0.627	0.398	1.90847317659976e-11	MON2
Rejected	30	5.9379636721288e-16	0.594744194280906	0.937	0.818	1.99147425635856e-11	CBLB
Rejected	30	6.54535782634048e-16	0.457693062157272	0.171	0.058	2.19518210779807e-11	SOX5

Rejected	30	7.51326340045536e-16	0.301915219912966	0.217	0.084	2.51979827924472e-11	AL137779.1
Rejected	30	7.70759236461939e-16	0.450490257318493	0.617	0.412	2.58497232724605e-11	TBC1D22A
Rejected	30	8.10663352629602e-16	0.460953293344053	0.756	0.536	2.71880275204916e-11	MAP3K2
Rejected	30	8.30227607746218e-16	0.431847003815994	0.448	0.25	2.78441735085926e-11	LMNA
Rejected	30	8.47614455909503e-16	0.428831711088398	0.564	0.354	2.84272936222929e-11	FLI1
Rejected	30	8.64864681868125e-16	0.356085310771919	0.547	0.326	2.90058317004932e-11	INO80D
Rejected	30	8.72721394359787e-16	0.393089439551524	0.655	0.433	2.92693301240386e-11	DIAPH1
Rejected	30	9.14062754015679e-16	0.368625248192621	0.312	0.15	3.06558366441779e-11	ATP11A
Rejected	30	9.17332295814707e-16	0.584320809405902	0.615	0.414	3.07654905370336e-11	GNAQ
Rejected	30	1.10136914624228e-15	0.481691144510086	0.718	0.516	3.69377184266736e-11	EXT1
Rejected	30	1.1679689897041e-15	0.42949472377614	0.884	0.698	3.91713439766959e-11	FOXN3
Rejected	30	1.22217770659001e-15	0.451263944164482	0.355	0.181	4.09893959236158e-11	AC073167.1
Rejected	30	1.27931038009551e-15	0.364696954110849	0.841	0.643	4.29055115276433e-11	RIPOR2
Rejected	30	1.3863400058289e-15	0.418577244076751	0.816	0.63	4.64950711154895e-11	MYCBP2
Rejected	30	1.4006507856086e-15	0.529148932176559	0.836	0.666	4.69750260477412e-11	MGAT5
Rejected	30	1.49161075106202e-15	0.553187892051176	0.504	0.309	5.00256413691182e-11	ITGB1
Rejected	30	1.67353570107872e-15	0.493212165381322	0.652	0.435	5.61270403427783e-11	RYBP
Rejected	30	1.6748912397054e-15	0.360592840961834	0.521	0.317	5.61725023972396e-11	ACIN1
Rejected	30	1.70858166196193e-15	0.420614870764458	0.524	0.32	5.73024117788792e-11	EP300
Rejected	30	1.74039384108376e-15	0.442632814305268	0.574	0.363	5.8369328642267e-11	GATAD2B
Rejected	30	1.75709564467775e-15	0.387378584534882	0.678	0.465	5.89294737312024e-11	ARID2
Rejected	30	1.81129036588118e-15	0.355588681521547	0.35	0.177	6.07470562909229e-11	AGPAT3
Rejected	30	1.83366843547795e-15	0.366475707465962	0.368	0.191	6.14975719890595e-11	DIDO1
Rejected	30	1.94783713749932e-15	0.379991832365624	0.242	0.101	6.53265619174522e-11	MCC
Rejected	30	2.00015946435935e-15	0.429621740915413	0.846	0.687	6.70813481156840e-11	JAK1
Rejected	30	2.0458203492053e-15	0.397229770225431	0.846	0.656	6.86127228716473e-11	USP15
Rejected	30	2.06675275027841e-15	0.373100672057396	0.909	0.781	6.93147537388372e-11	ANKRD12
Rejected	30	2.36117881884287e-15	0.382812398155611	0.557	0.336	7.91892152263522e-11	ANTXR2
Rejected	30	2.38215611931516e-15	0.368292147707967	0.937	0.808	7.9892751929592e-11	AKAP13
Rejected	30	2.40733782613405e-15	0.394040807603961	0.408	0.221	8.07372960128838e-11	TMEM181
Rejected	30	2.63019312469822e-15	0.412231898806349	0.584	0.378	8.82114170161290e-11	STK10

Rejected	30	2.64804710629921e-15	0.38354174153355	0.874	0.697	8.88102038510628e-11	INPP4B
Rejected	30	2.70187986626941e-15	0.47705260585974	0.625	0.411	9.06156469549435e-11	NSMCE2
Rejected	30	2.71765633209533e-15	0.43948817304788	0.932	0.777	9.11447580658131e-11	EZR
Rejected	30	2.73207820061659e-15	0.42436892531664	0.824	0.656	9.16284386922793e-11	BIRC6
Rejected	30	2.77033177444236e-15	0.35391852461462	0.567	0.352	9.29113870512478e-11	KDM5A
Rejected	30	2.94087820524477e-15	0.48160197483897	0.406	0.221	9.86311732474992e-11	ULK4
Rejected	30	2.94881207323391e-15	0.34339996001989	0.864	0.699	9.8897259312119e-11	FUS
Rejected	30	3.02023446972685e-15	0.45796268733438	0.363	0.189	1.01292623645699e-10	SPDYA
Rejected	30	3.08301862796613e-15	0.45732260470723	0.594	0.393	1.03398278744728e-10	RAPGEF1
Rejected	30	3.12688463588288e-15	0.45230897174602	0.433	0.246	1.0486945691824e-10	DGKH
Rejected	30	3.19306216809533e-15	0.42313121858013	0.463	0.275	1.07088918993581e-10	TBC1D1
Rejected	30	3.27867698306864e-15	0.43232947428016	0.786	0.596	1.09960268658156e-10	FAM172A
Rejected	30	3.49011255538302e-15	0.48167420454523	0.668	0.46	1.17051394882436e-10	MBD5
Rejected	30	3.59821521829169e-15	0.33715918031146	0.275	0.124	1.20676941991067e-10	KSR1
Rejected	30	3.60583788200887e-15	0.45658847253912	0.67	0.466	1.20932590886813e-10	NUP98
Rejected	30	3.67946424256356e-15	0.35130373640013	0.786	0.549	1.23401871767097e-10	IGHA1
Rejected	30	3.82921030677721e-15	0.38418891381128	0.715	0.494	1.28424055268694e-10	MYH9
Rejected	30	3.87747601079003e-15	0.40252825531893	0.463	0.272	1.30042790449876e-10	ATF2
Rejected	30	4.1139869062486e-15	0.36204354295794	0.166	0.058	1.37974892861766e-10	BNC2
Rejected	30	4.15130361386429e-15	0.56146884977854	0.542	0.338	1.39226420601781e-10	FAM129A
Rejected	30	4.36767654843668e-15	0.44101797326151	0.584	0.386	1.46483136081469e-10	ATP9B
Rejected	30	4.89575118078141e-15	0.40872448508874	0.317	0.158	1.64193703101047e-10	TTC7A
Rejected	30	5.2856000714339e-15	0.41838175689074	0.403	0.223	1.7726845519575e-10	NPC1
Rejected	30	5.69365774922485e-15	0.34373975572395	0.144	0.045	1.90953893593503e-10	AC025034.1
Rejected	30	5.76996528774671e-15	0.57025126887788	0.403	0.224	1.93513095820449e-10	AGPAT4
Rejected	30	5.84948537141881e-15	0.59938870478047	0.635	0.45	1.96180040386644e-10	SSBP2
Rejected	30	6.51329534174675e-15	0.26491684491591	1	0.992	2.18442899171502e-10	MT-ND4
Rejected	30	6.59043837611017e-15	0.39341171825427	0.597	0.388	2.21030122257983e-10	SRSF4
Rejected	30	6.6865993376283e-15	0.43091561235661	0.713	0.508	2.24255168585378e-10	KAT6B
Rejected	30	7.38788273397602e-15	0.42265912969947	0.771	0.583	2.47774811132088e-10	CCDC91
Rejected	30	7.83834413602741e-15	0.35691164050869	0.811	0.606	2.62882385634087e-10	IQGAP1

Rejected	30	7.94198622423121e-15	0.37529709693082	0.748	0.527	2.66358333988266e-10	MKLN1
Rejected	30	8.01481255958468e-15	0.31930461738865	0.322	0.159	2.68800783623351e-10	AL354733.3
Rejected	30	8.27113255604964e-15	0.49053817936335	0.705	0.496	2.77397243664793e-10	TLE4
Rejected	30	8.41576827775272e-15	0.44372904950805	0.935	0.831	2.82248036499271e-10	CELF2
Rejected	30	8.62688107580261e-15	0.47350037642568	0.569	0.373	2.89328337520268e-10	DENND1A
Rejected	30	8.70388729651522e-15	0.47802241069262	0.418	0.237	2.91910972150527e-10	IRS2
Rejected	30	8.84436230330076e-15	0.30442206230418	0.146	0.047	2.96622222928101e-10	GPR82
Rejected	30	9.03649078973589e-15	0.41240295128982	0.826	0.645	3.03065828106162e-10	MED13L
Rejected	30	1.05621461448301e-14	0.33852810687255	0.229	0.098	3.54233257405311e-10	BCR
Rejected	30	1.08151176731799e-14	0.37738106893577	0.61	0.399	3.62717416523108e-10	CNOT1
Rejected	30	1.08241468633228e-14	0.34914747200633	0.826	0.613	3.63020237502121e-10	VPS13B
Rejected	30	1.08642637598818e-14	0.37156473793055	0.69	0.474	3.64365677978917e-10	RLF
Rejected	30	1.20059667817291e-14	0.39220529215864	0.605	0.392	4.0265611392563e-10	RBM33
Rejected	30	1.20491935398328e-14	0.30820772887155	0.244	0.106	4.04105852938913e-10	AC010260.1
Rejected	30	1.22545937630104e-14	0.40254280309730	0.534	0.331	4.10994565623844e-10	MLLT10
Rejected	30	1.28382346768157e-14	0.39515549412571	0.506	0.315	4.30568714591044e-10	CMIP
Rejected	30	1.33368250458328e-14	0.31863720549781	0.461	0.264	4.4729043838714e-10	C16orf72
Rejected	30	1.33874657444221e-14	0.44308990858002	0.713	0.515	4.48988826136429e-10	CRY1
Rejected	30	1.34189458750159e-14	0.48928377427129	0.547	0.356	4.50044606756284e-10	FARS2
Rejected	30	1.49308386185166e-14	0.44321861232447	0.441	0.255	5.00750465587811e-10	SMAD2
Rejected	30	1.50966286163143e-14	0.40641261542418	0.451	0.264	5.0631073053395e-10	HOOK3
Rejected	30	1.53532783241304e-14	0.46467020975523	0.348	0.186	5.14918248434687e-10	AL157402.2
Rejected	30	1.7027010948521e-14	0.27459258071386	0.164	0.057	5.71051893191497e-10	AC124014.1
Rejected	30	1.7683198882684e-14	0.42658432748358	0.446	0.26	5.93059124127455e-10	ELK3
Rejected	30	1.78142225762037e-14	0.38816880013245	0.516	0.316	5.97453396760721e-10	UBL3
Rejected	30	2.06555496495837e-14	0.39383159043919	0.652	0.437	6.92745824147738e-10	TBC1D15
Rejected	30	2.12821194461437e-14	0.42047533242047	0.796	0.626	7.13759721984768e-10	FOXO1
Rejected	30	2.1335318779867e-14	0.46332101153231	0.438	0.259	7.15543921239181e-10	FBXO33
Rejected	30	2.32744874233223e-14	0.64351053302254	0.38	0.213	7.80579759203384e-10	FER
Rejected	30	2.33242377671282e-14	0.66074616075864	0.511	0.339	7.82248286233945e-10	MAP3K1
Rejected	30	2.38193888773752e-14	0.40246648200052	0.793	0.606	7.98854664169408e-10	BCLAF1

Rejected	30	2.44761059278936e-14	0.392484021106176	0.791	0.604	8.20879640609694e-10	ASH1L
Rejected	30	2.49072478114539e-14	0.385621388570396	0.652	0.441	8.35339277100541e-10	KMT2A
Rejected	30	2.66841498836918e-14	0.452243466200565	0.625	0.421	8.94933018799255e-10	DYM
Rejected	30	2.81627511834932e-14	0.473112463708274	0.914	0.829	9.44522349191994e-10	RABGAP1L
Rejected	30	2.82730105582173e-14	0.428981061400909	0.403	0.228	9.48220228101492e-10	TRRAP
Rejected	30	2.85716720177763e-14	0.406749647031291	0.521	0.323	9.58236736132181e-10	VPS13D
Rejected	30	2.89240380090686e-14	0.414451021125765	0.453	0.271	9.70054386748144e-10	TP53BP1
Rejected	30	2.95448843999516e-14	0.388488889512836	0.877	0.711	9.90876333005576e-10	RAP1B
Rejected	30	3.18201784697467e-14	0.318008589701071	0.441	0.254	1.06718514551836e-09	WIPF2
Rejected	30	3.38536213973443e-14	0.470059656415551	0.499	0.297	1.13538275442413e-09	RASGRF2
Rejected	30	3.48294163681114e-14	0.37638128069546	0.297	0.145	1.16810896615372e-09	ZRANB1
Rejected	30	3.6206333418172e-14	0.292831619640127	0.156	0.053	1.21428801017865e-09	CLMP
Rejected	30	3.7107408619534e-14	0.397135828060984	0.428	0.245	1.24450827028193e-09	IGF2R
Rejected	30	3.75715175001441e-14	0.335338324133687	0.574	0.369	1.26007355391983e-09	NUP153
Rejected	30	3.77605534531382e-14	0.395316331203615	0.776	0.59	1.26641344171135e-09	SRRM2
Rejected	30	3.80497364706149e-14	0.387283914090286	0.589	0.389	1.27611206175148e-09	RNGTT
Rejected	30	4.02100266520538e-14	0.36131643709518	0.27	0.127	1.34856387385658e-09	KCNQ1
Rejected	30	4.20755354184287e-14	0.375923196887494	0.423	0.246	1.41112930686326e-09	APLP2
Rejected	30	4.37275831265277e-14	0.489930433586856	0.39	0.22	1.46653568289748e-09	EZH2
Rejected	30	4.43672473198309e-14	0.591393249697095	0.28	0.139	1.48798874061249e-09	LINC02320
Rejected	30	4.58152010737018e-14	0.35393003147626	0.287	0.14	1.53655021360981e-09	EIF4G1
Rejected	30	4.74950840487146e-14	0.534545085241355	0.312	0.158	1.59289012882579e-09	CSGALNACT1
Rejected	30	4.76776933917887e-14	0.359626730045055	0.343	0.175	1.59901448097381e-09	AL606489.1
Rejected	30	4.85108540398197e-14	0.368132725957392	0.554	0.357	1.62695702278747e-09	RNF111
Rejected	30	4.86683319123014e-14	0.41268883620238	0.882	0.71	1.63223851567476e-09	PRKCH
Rejected	30	4.96959570748538e-14	0.386220746595252	0.665	0.451	1.66670300837645e-09	TRIP12
Rejected	30	5.08588051497769e-14	0.542228687640248	0.655	0.473	1.70570260711322e-09	NEDD9
Rejected	30	5.26912297794387e-14	0.365986820662477	0.599	0.389	1.76715846434281e-09	XRN2
Rejected	30	5.3948051538238e-14	0.402174129293138	0.66	0.464	1.80930975248943e-09	ARFGEF1
Rejected	30	5.49257738790336e-14	0.425110932931207	0.557	0.353	1.84210060435503e-09	CHD6
Rejected	30	5.78362241689303e-14	0.340521797947001	0.292	0.141	1.93971128617758e-09	SKAP1-AS1

Rejected	30	5.84845133837624e-14	0.45266171042685(	0.657	0.467	1.96145360986462e-09	PPP2R2A
Rejected	30	5.9770339175294e-14	0.32068260930241	0.861	0.661	2.00457763526101e-09	RASA2
Rejected	30	6.60619513645197e-14	0.50262048468988(	0.494	0.311	2.21558572486326e-09	SLC25A26
Rejected	30	6.66709618286117e-14	0.47590977421243	0.642	0.454	2.23601071780798e-09	PTPN1
Rejected	30	6.83921489156866e-14	0.36177349390045	0.544	0.34	2.2937358903343e-09	EWSR1
Rejected	30	8.58376054648536e-14	0.26015437610407	0.128	0.04	2.87882161208026e-09	AC008735.2
Rejected	30	8.63972561012669e-14	0.25625329127163	0.327	0.166	2.89759117512429e-09	PLPP1
Rejected	30	9.30199711965585e-14	0.28650246533952	0.373	0.196	3.11970379399018e-09	FAM193A
Rejected	30	1.05648768623896e-13	0.42689869809574	0.506	0.318	3.54324840210821e-09	DIP2B
Rejected	30	1.06427925883581e-13	0.37391108957292	0.406	0.23	3.56937977828353e-09	ARMH3
Rejected	30	1.08609684292683e-13	0.50921797863922	0.338	0.181	3.64255159180802e-09	SVIL
Rejected	30	1.09609824181032e-13	0.49573078382782	0.423	0.241	3.67609428338344e-09	DISC1
Rejected	30	1.09966749782314e-13	0.43020636023205(	0.496	0.304	3.68806485419925e-09	PHF21A
Rejected	30	1.13451304242812e-13	0.42312889023201	0.322	0.165	3.80492984169541e-09	AL139807.1
Rejected	30	1.20554346663295e-13	0.43597719830993	0.678	0.478	4.04315167839358e-09	CYLD
Rejected	30	1.22178892430187e-13	0.33759652455379	0.763	0.582	4.09763569432361e-09	PIAS1
Rejected	30	1.28612632313814e-13	0.38774833826290	0.582	0.38	4.31341046254068e-09	NFATC2
Rejected	30	1.29524873004157e-13	0.35511466748781	0.635	0.42	4.34400519081342e-09	LCOR
Rejected	30	1.30542323010375e-13	0.39069322277401	0.584	0.386	4.37812842912194e-09	KIF13B
Rejected	30	1.35016663916902e-13	0.34798538443424	0.385	0.21	4.52818887444507e-09	NR2C2
Rejected	30	1.37952354621039e-13	0.48300839668405	0.703	0.514	4.6266460692804e-09	NSD3
Rejected	30	1.3991841610082e-13	0.39017340702933	0.66	0.452	4.6925838391893e-09	ZNF609
Rejected	30	1.44536158888339e-13	0.44677475709338	0.617	0.428	4.8474536967971e-09	MSI2
Rejected	30	1.48629695819978e-13	0.65947451568156	0.504	0.34	4.98474273841043e-09	BTBD11
Rejected	30	1.55096859133946e-13	0.40710424626763	0.62	0.413	5.20163846163427e-09	CLASP1
Rejected	30	1.55581609140205e-13	0.44938615194146	0.547	0.35	5.21789600734421e-09	MBNL2
Rejected	30	1.74424564196828e-13	0.41287546178034	0.932	0.835	5.84985103403323e-09	SKAP1
Rejected	30	1.74725843137344e-13	0.35217761645745	0.501	0.308	5.85995532714024e-09	DCP1A
Rejected	30	1.77318739555165e-13	0.40862522254932	0.725	0.538	5.94691588720111e-09	BPTF
Rejected	30	1.91489716925315e-13	0.41195968623595	0.514	0.325	6.4221821262412e-09	NBAS
Rejected	30	2.08258578464196e-13	0.30133822543532	0.239	0.108	6.9845762045322e-09	TBCD

Rejected	30	2.09998933941494e-13	0.317331104788677	0.171	0.065	7.04294424652981e-09	TTC28
Rejected	30	2.22985429380611e-13	0.37873170474455[illegible]	0.461	0.281	7.47848533056693e-09	UBE3C
Rejected	30	2.44907701904611e-13	0.414021097920657	0.436	0.255	8.21371450647685e-09	DIS3L2
Rejected	30	2.47759099885298e-13	0.41004574803001[illegible]	0.642	0.451	8.30934469195313e-09	RAB7A
Rejected	30	2.62452458632164e-13	0.339117653109027	0.234	0.104	8.8021305576055e-09	SH3RF3
Rejected	30	2.63410227823663e-13	0.43891002407753[illegible]	0.67	0.478	8.83425222075e-09	FNDC3A
Rejected	30	2.82130787869574e-13	0.38681888234757[illegible]	0.343	0.183	9.46210236356978e-09	TRANK1
Rejected	30	2.82972586465387e-13	0.359032554995	0.625	0.417	9.49033460487616e-09	PSPC1
Rejected	30	2.98434706956726e-13	0.38571988136024[illegible]	0.816	0.621	1.00089032019147e-08	NIPBL
Rejected	30	3.10024621007134e-13	0.32784436143307	0.453	0.271	1.03976057393373e-08	PSMA3-AS1
Rejected	30	3.15252418574706e-13	0.26517837265371[illegible]	0.204	0.085	1.05729356141585e-08	STK35
Rejected	30	3.21383700283752e-13	0.547141961798207	0.285	0.142	1.07785665401165e-08	SAMD4A
Rejected	30	3.46351156441016e-13	0.35609488055365[illegible]	0.156	0.056	1.16159250847188e-08	EBF1
Rejected	30	3.51243360526432e-13	0.378878531596887	0.456	0.277	1.17799998253355e-08	EXOC2
Rejected	30	3.74376058722409e-13	0.46010849920663[illegible]	0.486	0.306	1.25558242574322e-08	PRKAR2A
Rejected	30	3.80008633476027e-13	0.46289612125117[illegible]	0.476	0.293	1.2744729549519e-08	SBF2
Rejected	30	3.84807280092893e-13	0.39000053098564	0.65	0.455	1.29056665597554e-08	RBM6
Rejected	30	4.17668176967663e-13	0.407627384364007	0.489	0.311	1.40077553191415e-08	PTBP2
Rejected	30	4.18846372444721e-13	0.48244995803922[illegible]	0.461	0.29	1.40472696390511e-08	SREBF2
Rejected	30	4.23843860022211e-13	0.36905562630838	0.69	0.506	1.42148753774249e-08	SETD2
Rejected	30	4.31152499734619e-13	0.38118969692386[illegible]	0.753	0.56	1.44599925360997e-08	CCSER2
Rejected	30	4.42527655628464e-13	0.34577760847746	0.738	0.548	1.48414925144674e-08	NAMPT
Rejected	30	4.78831108751846e-13	0.40371515906328[illegible]	0.307	0.158	1.60590377253194e-08	BMPR2
Rejected	30	4.85584309455546e-13	0.410035077876597	0.423	0.252	1.62855265705201e-08	ERO1B
Rejected	30	4.95507552850196e-13	0.373111963786855	0.368	0.204	1.66183323074899e-08	GALNT10
Rejected	30	4.9897841076268e-13	0.47019382798752[illegible]	0.317	0.17	1.67347379401588e-08	IGF1R
Rejected	30	5.93905866224062e-13	0.47383390690387[illegible]	0.61	0.428	1.99184149414226e-08	AFF1
Rejected	30	6.91777900513949e-13	0.49817272041684[illegible]	0.773	0.621	2.32008472274368e-08	MAPRE2
Rejected	30	6.92421460752588e-13	0.42204570533676	0.798	0.624	2.32224309507203e-08	SH3KBP1
Rejected	30	7.15622123163291e-13	0.39055245085409[illegible]	0.438	0.263	2.40005347666504e-08	EFR3A
Rejected	30	7.26117033636879e-13	0.36038324807473[illegible]	0.378	0.213	2.43525130741136e-08	KLHL18

Rejected	30	7.30163975016816e-13	0.364856598636655	0.423	0.246	2.4488239394114e-08	IP6K1
Rejected	30	7.40832336223881e-13	0.381146822918707	0.537	0.351	2.48460348922765e-08	OSBPL9
Rejected	30	7.46626015221424e-13	0.344513815830274	0.607	0.417	2.50403432984961e-08	SEMA4D
Rejected	30	7.52638360307521e-13	0.434197897613436	0.499	0.323	2.52419853279936e-08	FTO
Rejected	30	7.67082612740671e-13	0.352883095556106	0.448	0.274	2.57264166660966e-08	SPTBN1
Rejected	30	7.82522665222735e-13	0.433475189157122	0.662	0.491	2.62442451462401e-08	HIPK1
Rejected	30	7.95380556228893e-13	0.352479180759021	0.945	0.848	2.66754730948046e-08	NEAT1
Rejected	30	7.95519231749321e-13	0.491274944801857	0.741	0.578	2.66801239944087e-08	PACS1
Rejected	30	8.34637859936612e-13	0.31919948177328	0.214	0.095	2.79920845465541e-08	PRCC
Rejected	30	8.5333510240213e-13	0.437103569091873	0.509	0.326	2.86191526643626e-08	SIK2
Rejected	30	8.57730073291861e-13	0.412348395325573	0.685	0.507	2.87665511980624e-08	STAG2
Rejected	30	8.80419211977557e-13	0.341328980275117	0.733	0.539	2.95274995313033e-08	PAFAH1B1
Rejected	30	8.96370379671955e-13	0.355319265554593	0.164	0.063	3.0062469793438e-08	TPST1
Rejected	30	9.40161274773252e-13	0.380616806223317	0.428	0.257	3.15311288333453e-08	SAFB2
Rejected	30	9.60962832129915e-13	0.287724732465759	0.264	0.128	3.22287714639731e-08	CALU
Rejected	30	9.74913083155396e-13	0.450138073100555	0.683	0.518	3.26966349828657e-08	UBE2H
Rejected	30	1.03373124961891e-12	0.352365223265129	0.705	0.493	3.46692786497192e-08	EPS15
Rejected	30	1.04607929403957e-12	0.415010826736361	0.421	0.249	3.50834073634992e-08	HMBOX1
Rejected	30	1.0794921603924e-12	0.309375425726332	0.461	0.283	3.62040080752402e-08	ETF1
Rejected	30	1.08285593130592e-12	0.488824840742117	0.612	0.437	3.63168222241379e-08	ZNF407
Rejected	30	1.12109064042545e-12	0.45429114575877	0.411	0.245	3.75991378985888e-08	ATG7
Rejected	30	1.13348846261493e-12	0.375345486632981	0.297	0.156	3.80149360591796e-08	PACSIN2
Rejected	30	1.17773312933336e-12	0.33775315152581	0.607	0.404	3.94988136915822e-08	WDR33
Rejected	30	1.1865631761171e-12	0.401300304759802	0.564	0.371	3.97949558006154e-08	DOCK11
Rejected	30	1.23312436558556e-12	0.310368089867806	0.146	0.052	4.13565249730086e-08	SLC24A1
Rejected	30	1.24745985491471e-12	0.353212464430201	0.353	0.193	4.18373086141295e-08	UBE4B
Rejected	30	1.26076975280352e-12	0.278217390907785	0.27	0.131	4.22836959695244e-08	AREL1
Rejected	30	1.35612054651097e-12	0.375537069909771	0.375	0.216	4.5481570888885e-08	WHAMM
Rejected	30	1.38204025566662e-12	0.376559585165724	0.461	0.282	4.63508660945472e-08	NAA15
Rejected	30	1.38888633963297e-12	0.419201557763469	0.325	0.18	4.65804700586106e-08	ADARB1
Rejected	30	1.42034392328373e-12	0.414632493022554	0.708	0.517	4.76354944990897e-08	NFAT5

Rejected	30	1.45619261919767e-12	0.30137020070317	0.159	0.06	4.88377880626515e-08	PID1
Rejected	30	1.47580496479283e-12	0.31511724252337	0.683	0.473	4.9495546909222e-08	NFE2L2
Rejected	30	1.50851896208355e-12	0.37850808777182	0.511	0.327	5.05927089503582e-08	RAB3GAP1
Rejected	30	1.69226777439114e-12	0.33423804537571	0.128	0.043	5.67552766175302e-08	AP005131.1
Rejected	30	1.71983213698883e-12	0.30543281742308	0.118	0.037	5.76797302103315e-08	GPC6
Rejected	30	1.72155739866868e-12	0.39242666212758	0.65	0.464	5.77375920365501e-08	CTNNB1
Rejected	30	1.7424849196118e-12	0.42448922181879	0.426	0.256	5.84394592339406e-08	PDE8A
Rejected	30	1.78192119722613e-12	0.37298113283313	0.494	0.318	5.97620731125699e-08	ADAR
Rejected	30	1.78702841400845e-12	0.52728129129436	0.471	0.313	5.99333589490154e-08	STX8
Rejected	30	1.79501551756095e-12	0.31647940828900	0.491	0.302	6.02012304279591e-08	SEC24B
Rejected	30	1.92164024355122e-12	0.53270813366294	0.395	0.241	6.44479704882207e-08	ZBTB16
Rejected	30	1.94388456154131e-12	0.34359956831781	0.469	0.29	6.51940004249726e-08	TLK2
Rejected	30	1.96273978998064e-12	0.35559549935051	0.514	0.333	6.58263670763708e-08	ATAD2B
Rejected	30	2.02220496869795e-12	0.37044437182228	0.955	0.871	6.7820710240192e-08	CNOT6L
Rejected	30	2.37992816128322e-12	0.39800646824256	0.446	0.275	7.98180306731166e-08	SREK1
Rejected	30	2.38829383892268e-12	0.31184062220981	0.506	0.319	8.00985987697887e-08	LARP4B
Rejected	30	2.43111222951282e-12	0.38059202423190	0.62	0.427	8.15346419534008e-08	RNF38
Rejected	30	2.61286889946635e-12	0.37805752036416	0.202	0.088	8.76303971503023e-08	CRIM1
Rejected	30	2.70147043827996e-12	0.33524417926047	0.489	0.307	9.06019155590334e-08	XPO6
Rejected	30	2.72835612385204e-12	0.28891987710602	0.413	0.242	9.15036076817498e-08	SNX1
Rejected	30	2.84556649183454e-12	0.35073318643255	0.499	0.324	9.54346090031468e-08	GATAD2A
Rejected	30	3.03661007173533e-12	0.37001076302538	0.501	0.323	1.0184182858586e-07	PPP1R15B
Rejected	30	3.04085663280272e-12	0.34505124789245	0.552	0.369	1.01984249750938e-07	PSME4
Rejected	30	3.10356963155064e-12	0.37124802928508	0.65	0.466	1.04087518302945e-07	ITSN2
Rejected	30	3.22737685174943e-12	0.29299942179474	0.693	0.5	1.08239764853972e-07	IVNS1ABP
Rejected	30	3.26846307829141e-12	0.35869530478126	0.574	0.376	1.09617714719737e-07	RB1CC1
Rejected	30	3.30621842718745e-12	0.38035772025208	0.544	0.358	1.10883953611013e-07	COA1
Rejected	30	3.31059755025328e-12	0.49259934588238	0.62	0.443	1.11030820640394e-07	PPP1R16B
Rejected	30	3.70895439157446e-12	0.45829636586479	0.501	0.33	1.24390912384624e-07	SMAD3
Rejected	30	3.87537311935358e-12	0.54340326274047	0.831	0.708	1.2997226367688e-07	MAML2
Rejected	30	4.01272461776963e-12	0.61386273360642	0.683	0.507	1.34578758230758e-07	PELI1

Rejected	30	4.05425389882356e-12	0.488117575126462	0.599	0.425	1.35971567258744e-07	SPPL3
Rejected	30	4.23503958089718e-12	0.392068901115944	0.725	0.552	1.4203475746413e-07	PRDM2
Rejected	30	4.29085315263679e-12	0.364840672768698	0.668	0.485	1.43906633033133e-07	SMARCA2
Rejected	30	4.51058168243904e-12	0.471896767449691	0.514	0.347	1.5127588846564e-07	FCHSD2
Rejected	30	4.67994249509998e-12	0.380388463967917	0.521	0.34	1.56955911400663e-07	SLMAP
Rejected	30	4.69649315639713e-12	0.54269977164752	0.63	0.469	1.57510987479247e-07	ARHGAP26
Rejected	30	4.80136961370938e-12	0.320648508653175	0.101	0.029	1.61028334104585e-07	PLXNA4
Rejected	30	5.19797727028833e-12	0.286176909772083	0.312	0.164	1.7432976169093e-07	IL4R
Rejected	30	5.24669438216591e-12	0.325327217869801	0.922	0.786	1.7596363618908e-07	NAP1L1
Rejected	30	5.30097444397058e-12	0.408173053199122	0.685	0.518	1.77784080901885e-07	FBXL17
Rejected	30	5.42040848552134e-12	0.317859450817636	0.348	0.194	1.81789659787415e-07	KDM2B
Rejected	30	5.42990181468331e-12	0.390093400732731	0.484	0.306	1.82108047060849e-07	ZFR
Rejected	30	5.69211705999536e-12	0.301122381177561	0.599	0.404	1.90902221958124e-07	UBAP1
Rejected	30	6.23687121067245e-12	0.454469511596567	0.829	0.68	2.09172186663533e-07	OSBPL8
Rejected	30	6.78981693455247e-12	0.425048384442049	0.637	0.462	2.27716880351021e-07	TUT4
Rejected	30	6.93528786292417e-12	0.344425370245682	0.715	0.529	2.32595684346751e-07	CEP85L
Rejected	30	7.09879472456249e-12	0.368845840781183	0.63	0.439	2.38079377472377e-07	MED13
Rejected	30	7.26780153684906e-12	0.342763087212151	0.315	0.172	2.43747527942844e-07	FAM168B
Rejected	30	7.35174012408737e-12	0.361625663146568	0.494	0.316	2.46562660281642e-07	ST3GAL1
Rejected	30	7.52489709941354e-12	0.387309480332806	0.388	0.232	2.52369998920131e-07	RAB22A
Rejected	30	7.70866035515304e-12	0.36852907159613	0.401	0.241	2.58533050991123e-07	APOB
Rejected	30	7.73721165848475e-12	0.314943198238747	0.217	0.1	2.59490604602262e-07	CRY2
Rejected	30	7.9494003452224e-12	0.369061470841099	0.34	0.193	2.66606988778069e-07	RRP12
Rejected	30	7.96171032748253e-12	0.368968083454764	0.877	0.757	2.67019840963109e-07	KLF12
Rejected	30	8.10893370149722e-12	0.387909139568369	0.69	0.516	2.71957418480814e-07	MARCH6
Rejected	30	8.14871067464754e-12	0.326481182147327	0.207	0.093	2.73291458606329e-07	PBX1
Rejected	30	8.26484824750393e-12	0.318422588296124	0.438	0.267	2.77186480524787e-07	SENP5
Rejected	30	8.28522121665814e-12	0.49029438209907	0.579	0.411	2.77869749164281e-07	MAPK8
Rejected	30	8.55850809798596e-12	0.392113002720709	0.612	0.434	2.87035244590253e-07	PATJ
Rejected	30	8.78426727341214e-12	0.369516392719566	0.325	0.18	2.94606755815696e-07	ATG13
Rejected	30	8.93481309014696e-12	0.343222313233641	0.534	0.353	2.99655761417349e-07	KIAA0232

Rejected	30	1.00280563637401e-11	0.344978166459149	0.499	0.318	3.36320954327114e-07	INPP4A
Rejected	30	1.02159638029108e-11	0.404213171605044	0.32	0.172	3.42622994022021e-07	NFIA
Rejected	30	1.06558411715629e-11	0.391558530609292	0.416	0.249	3.57375601211876e-07	MYO9B
Rejected	30	1.06743508558312e-11	0.35680386716831	0.229	0.11	3.57996379002868e-07	CYREN
Rejected	30	1.0934686716033e-11	0.363302133177194	0.572	0.38	3.66727523082316e-07	CELF1
Rejected	30	1.11002514752469e-11	0.363814693660783	0.476	0.302	3.72280233976831e-07	TOP2B
Rejected	30	1.1303247267942e-11	0.345627222191506	0.892	0.745	3.79088306872238e-07	FNBP1
Rejected	30	1.15346959368703e-11	0.385411907316	0.574	0.414	3.86850632330757e-07	QKI
Rejected	30	1.16301478187748e-11	0.314305139693081	0.189	0.081	3.90051897546068e-07	AC010275.1
Rejected	30	1.17378747378003e-11	0.32880914904627	0.597	0.417	3.93664842956347e-07	PCGF5
Rejected	30	1.17572729242674e-11	0.335203509975714	0.71	0.514	3.94315419334079e-07	EIF4G2
Rejected	30	1.23491492014168e-11	0.376218352433283	0.809	0.677	4.14165765917118e-07	ATXN1
Rejected	30	1.2687739270811e-11	0.372067384793375	0.594	0.415	4.25521399664459e-07	MECP2
Rejected	30	1.27792880432784e-11	0.305938208498182	0.317	0.173	4.2859176239547e-07	UHRF1BP1L
Rejected	30	1.33427030076451e-11	0.271761554239195	0.28	0.142	4.47487573470402e-07	DHX40
Rejected	30	1.3393992174737e-11	0.375344953699096	0.254	0.126	4.49207709556328e-07	JAM3
Rejected	30	1.35378123507769e-11	0.393326570803097	0.678	0.516	4.54031150620355e-07	USP9X
Rejected	30	1.37285569012964e-11	0.376064889675229	0.406	0.247	4.6042834135568e-07	RPTOR
Rejected	30	1.37840943842035e-11	0.445554999014851	0.715	0.56	4.62290957457418e-07	ARL15
Rejected	30	1.40800156021758e-11	0.325002006171028	0.605	0.425	4.72215563265773e-07	CNOT4
Rejected	30	1.52104889928059e-11	0.331192519848588	0.222	0.105	5.10129379840724e-07	TTN
Rejected	30	1.54937764581866e-11	0.28540792522829	0.355	0.197	5.19630274854662e-07	DUSP10
Rejected	30	1.58261404393505e-11	0.51250714217274	0.436	0.279	5.30777098054936e-07	SNX25
Rejected	30	1.71676080051527e-11	0.253200103907273	0.159	0.063	5.75767237276812e-07	KDM5D
Rejected	30	1.72210296336057e-11	0.356291906004604	0.793	0.624	5.77558891851869e-07	LPP
Rejected	30	1.73549111805944e-11	0.356287645043346	0.61	0.415	5.82049011174775e-07	STIM1
Rejected	30	2.01106364134678e-11	0.389027970645282	0.61	0.447	6.74470524034884e-07	TNRC6A
Rejected	30	2.09790366805867e-11	0.398819848349054	0.28	0.143	7.03594932193518e-07	KIF21A
Rejected	30	2.11864967447558e-11	0.28407287062585	0.564	0.375	7.1055272782562e-07	EHMT1
Rejected	30	2.1479963873297e-11	0.301186601028581	0.378	0.219	7.20395028382633e-07	QRICH1
Rejected	30	2.26332772717383e-11	0.344813328707296	0.501	0.326	7.5907485313956e-07	NRF1

Rejected	30	2.43369803782525e-11	0.330037130875448	0.202	0.091	8.16213647925831e-07	AC005037.1
Rejected	30	2.46210250519813e-11	0.431606208496031	0.665	0.494	8.25739938193349e-07	RUNX1
Rejected	30	2.7274771894599e-11	0.394375736820031	0.708	0.536	9.14741299801062e-07	VTI1A
Rejected	30	2.82202702324208e-11	0.253362097084455	0.156	0.062	9.46451423054928e-07	FARP1
Rejected	30	2.88184865885402e-11	0.255340046603467	0.131	0.048	9.6651440320646e-07	TSPAN18
Rejected	30	2.91422036886025e-11	0.363544556469339	0.678	0.493	9.77371227308352e-07	ASXL1
Rejected	30	2.94384481056083e-11	0.342304187898529	0.698	0.524	9.8730667256589e-07	ANKRD17
Rejected	30	2.96445320966779e-11	0.339858298792153	0.486	0.309	9.94218317458383e-07	UBR3
Rejected	30	3.01968325625417e-11	0.362579919093227	0.506	0.333	1.01274137048252e-06	CUX1
Rejected	30	3.04621018206049e-11	0.299503256148255	0.474	0.297	1.02163797085945e-06	KBTBD2
Rejected	30	3.08767891160312e-11	0.251123243465608	0.249	0.123	1.03554575337345e-06	AC016831.1
Rejected	30	3.11983061017265e-11	0.351124273536035	0.257	0.134	1.0463287900397e-06	SAMD4B
Rejected	30	3.24327973888972e-11	0.455130521594498	0.446	0.278	1.08773115882884e-06	AC010609.1
Rejected	30	3.24893879854391e-11	0.342100245057407	0.217	0.101	1.08962909425566e-06	RFX2
Rejected	30	3.27366390304411e-11	0.318819898371538	0.242	0.119	1.09792139980293e-06	C11orf65
Rejected	30	3.36074045851989e-11	0.325628705675511	0.408	0.246	1.1271251349784e-06	VPS8
Rejected	30	3.72025815626356e-11	0.251484296280066	0.144	0.055	1.24770018044767e-06	RASAL2
Rejected	30	3.87812824070715e-11	0.35842982500622	0.305	0.165	1.30064664936836e-06	ANKUB1
Rejected	30	3.96958868900521e-11	0.423740836297122	0.335	0.192	1.33132065451857e-06	DHRSX
Rejected	30	3.99698489395616e-11	0.319463914997204	0.67	0.468	1.34050879373502e-06	ABI1
Rejected	30	4.0025944656367e-11	0.426719596684926	0.751	0.587	1.34239013188524e-06	TMEM131L
Rejected	30	4.31572837412368e-11	0.422950744797629	0.693	0.507	1.4474089821136e-06	RASGEF1B
Rejected	30	4.37077126122564e-11	0.351220542968403	0.718	0.528	1.46586926558986e-06	TBL1XR1
Rejected	30	4.385649316451e-11	0.350580782995629	0.617	0.427	1.47085906775134e-06	USP25
Rejected	30	4.39459505009466e-11	0.326729740781325	0.189	0.084	1.47385928790075e-06	MYO1B
Rejected	30	4.60746888984015e-11	0.257752528299709	0.154	0.06	1.54525291627459e-06	ZFY
Rejected	30	4.79749634806917e-11	0.518924087084392	0.113	0.038	1.60898432521544e-06	EDA
Rejected	30	4.83111333729719e-11	0.404744425690703	0.416	0.259	1.62025879106273e-06	OPA1
Rejected	30	4.93910211826404e-11	0.393723606063091	0.718	0.55	1.65647606842339e-06	PHF20
Rejected	30	5.23974638440122e-11	0.381419283499077	0.474	0.311	1.75730614240048e-06	SUCO
Rejected	30	5.26372565547323e-11	0.368568550567358	0.401	0.249	1.76534831033261e-06	LDLR

Rejected	30	5.35801169764954e-11	0.39534655492001[illegible]	0.519	0.357	1.7969699631577e-06	GBF1
Rejected	30	5.44364003760031e-11	0.40604172469373[illegible]	0.416	0.263	1.82568799581039e-06	LUZP1
Rejected	30	5.47347806200331e-11	0.58652955605592[illegible]	0.592	0.451	1.83569507243467e-06	RAD51B
Rejected	30	5.78272341862956e-11	0.49304141816409[illegible]	0.327	0.187	1.93940978013998e-06	MX2
Rejected	30	6.36806246526808e-11	0.41434925801732[illegible]	0.534	0.376	2.13572078960161e-06	STK39
Rejected	30	6.39100397728174e-11	0.36644006831329[illegible]	0.592	0.417	2.14341491390075e-06	POLR2A
Rejected	30	6.44131144423753e-11	0.29278662704860[illegible]	0.335	0.19	2.16028703216838e-06	TNIP1
Rejected	30	6.49771117070097e-11	0.34060100114536[illegible]	0.36	0.212	2.17920237242969e-06	FBXO21
Rejected	30	6.89235190006718e-11	0.324732771114443	0.36	0.211	2.31155698024453e-06	R3HCC1L
Rejected	30	7.23562650053252e-11	0.27246523519394[illegible]	0.474	0.295	2.4266844157486e-06	AEBP2
Rejected	30	7.37472713856183e-11	0.35921574595919	0.438	0.278	2.47333598773087e-06	POU2F1
Rejected	30	7.48114072672787e-11	0.32733717608511	0.433	0.264	2.50902497692999e-06	AHCTF1
Rejected	30	7.5210096271715e-11	0.40528026394818[illegible]	0.315	0.179	2.52239620876078e-06	APP
Rejected	30	7.84516013501118e-11	0.34081299717558[illegible]	0.771	0.599	2.63110980608005e-06	AAK1
Rejected	30	7.85727544995457e-11	0.27504174608746[illegible]	0.317	0.179	2.63517304040576e-06	SZRD1
Rejected	30	7.86808083828538e-11	0.30894250971749[illegible]	0.32	0.177	2.63879695154415e-06	ARMH4
Rejected	30	8.06101411846337e-11	0.33600496998699[illegible]	0.423	0.26	2.70350291505024e-06	RREB1
Rejected	30	8.24998784759739e-11	0.28921816397902[illegible]	0.29	0.158	2.76688092432721e-06	PTPRG
Rejected	30	8.4173770538684e-11	0.26826499647609[illegible]	0.438	0.267	2.82301991632638e-06	CDK12
Rejected	30	8.43923978146124e-11	0.32500601796471[illegible]	0.458	0.292	2.83035223790647e-06	PLEKHA2
Rejected	30	9.0715930776689e-11	0.27060201496952[illegible]	0.272	0.142	3.0424308863886e-06	FAM91A1
Rejected	30	9.22458224009113e-11	0.38353932821430[illegible]	0.537	0.364	3.09374039168176e-06	TMEM131
Rejected	30	9.31550706085149e-11	0.27481914153078[illegible]	0.186	0.084	3.12423475806837e-06	ARID3B
Rejected	30	9.38078112290475e-11	0.30549722265826[illegible]	0.249	0.128	3.14612637299979e-06	KIAA0556
Rejected	30	9.46080110261686e-11	0.48878228600279[illegible]	0.204	0.097	3.17296347379564e-06	MB21D2
Rejected	30	9.68091154169397e-11	0.28678245550467[illegible]	0.237	0.119	3.24678411285332e-06	ZBTB43
Rejected	30	9.81419858222784e-11	0.26291646787472[illegible]	0.421	0.253	3.29148592050757e-06	MAP2K1
Rejected	30	1.0222863229173e-10	0.40965505696297[illegible]	0.544	0.381	3.42854386980003e-06	ERC1
Rejected	30	1.03245932902267e-10	0.39189339664679[illegible]	0.338	0.199	3.46266209767623e-06	MAPKAPK2
Rejected	30	1.05195335266946e-10	0.26776085461023[illegible]	0.32	0.179	3.52804115418283e-06	ELK4
Rejected	30	1.05942261185939e-10	0.38396746408470[illegible]	0.481	0.315	3.55309155565402e-06	MAP4

Rejected	30	1.08717150531757e-10	0.330386567813985	0.567	0.384	3.64615579453408e-06	ADNP
Rejected	30	1.13007974335297e-10	0.329258260641486	0.242	0.124	3.7900614432572e-06	EMP1
Rejected	30	1.13251007854263e-10	0.399972138688013	0.428	0.271	3.79821230141628e-06	AC010894.3
Rejected	30	1.18864509903557e-10	0.275980042679256	0.602	0.419	3.98647793314549e-06	SMARCC1
Rejected	30	1.20168733940583e-10	0.317067947744713	0.738	0.556	4.03021899889928e-06	COG5
Rejected	30	1.24903308467013e-10	0.305433578549263	0.312	0.174	4.18900715936668e-06	SCYL2
Rejected	30	1.31092162140044e-10	0.277696224383349	0.736	0.554	4.39656893385281e-06	FAF1
Rejected	30	1.31628692499932e-10	0.321622077329628	0.378	0.229	4.41456308906271e-06	HDLBP
Rejected	30	1.32448742858522e-10	0.27395214610725	0.408	0.249	4.4420659379891e-06	USP7
Rejected	30	1.34280953538675e-10	0.354738178148505	0.741	0.568	4.50351461978007e-06	ZCCHC7
Rejected	30	1.37662772624709e-10	0.339448223505528	0.529	0.36	4.61693406828748e-06	WDR26
Rejected	30	1.41444708124076e-10	0.33959550211692	0.398	0.247	4.74377262106527e-06	CHD4
Rejected	30	1.46479407243177e-10	0.287848323769347	0.494	0.322	4.91262636012167e-06	SOS2
Rejected	30	1.53881892664572e-10	0.332813665413022	0.441	0.281	5.16089091618441e-06	OPTN
Rejected	30	1.53903152608292e-10	0.325602200180759	0.176	0.077	5.16160393217689e-06	ERP27
Rejected	30	1.60648145212205e-10	0.282879849561769	0.521	0.353	5.38781749412693e-06	ITCH
Rejected	30	1.6422158204208e-10	0.260286946548914	0.458	0.287	5.50766341852728e-06	CCNK
Rejected	30	1.73635191483975e-10	0.30739248367784	0.438	0.274	5.82337705198956e-06	ZNF267
Rejected	30	1.7369383305056e-10	0.317225520630894	0.285	0.155	5.82534377284969e-06	VCL
Rejected	30	1.75396859316859e-10	0.31254871497855	0.111	0.038	5.88245986776881e-06	C9orf84
Rejected	30	1.76879292235561e-10	0.346769024752218	0.531	0.365	5.93217770299624e-06	UVRAG
Rejected	30	1.76969876715194e-10	0.292016511324076	0.227	0.112	5.93521572527418e-06	GSE1
Rejected	30	1.79907052604536e-10	0.455927469456462	0.567	0.406	6.03372273025092e-06	ATP8A1
Rejected	30	1.80773396154624e-10	0.3233755147471	0.297	0.165	6.06277816023377e-06	ANKS1A
Rejected	30	1.87493508080604e-10	0.444685972505272	0.489	0.324	6.28815727400729e-06	RUNX2
Rejected	30	1.91540887234355e-10	0.310660141771268	0.287	0.154	6.42389827606579e-06	TGFBR3
Rejected	30	1.99197942314959e-10	0.322529299910021	0.529	0.361	6.6807005893591e-06	PTPRA
Rejected	30	2.0448872374906e-10	0.334391099794126	0.453	0.291	6.85814281709597e-06	GLUD1
Rejected	30	2.09431696719407e-10	0.382132778058898	0.343	0.205	7.02392024457548e-06	PCCA
Rejected	30	2.09575765958817e-10	0.37630188593619	0.509	0.341	7.02875203872681e-06	HIVEP1
Rejected	30	2.11748585565377e-10	0.388592316056645	0.73	0.573	7.10162406269162e-06	TNIK

Rejected	30	2.16035752829745e-10	0.36088693878320(	0.73	0.548	7.24540707840398e-06	ARAP2
Rejected	30	2.16795277811178e-10	0.36535439930900{	0.66	0.483	7.27088002723129e-06	MAP3K5
Rejected	30	2.25875935649551e-10	0.30674800930108	0.28	0.151	7.57542712981463e-06	ALMS1
Rejected	30	2.27274595934097e-10	0.42434124716172	0.3	0.17	7.62233539843775e-06	LRIG1
Rejected	30	2.30615154222878e-10	0.33717344967828	0.579	0.41	7.73437104232689e-06	GLG1
Rejected	30	2.42232355446998e-10	0.31990488383594	0.695	0.535	8.12398873698142e-06	CSNK1A1
Rejected	30	2.51202254633927e-10	0.35198770725353	0.466	0.3	8.42482121591265e-06	PIK3R5
Rejected	30	2.53809377069212e-10	0.33012451813358	0.355	0.216	8.51225888814722e-06	SUPT6H
Rejected	30	2.54989670654035e-10	0.41381904938996	0.491	0.332	8.55184357439503e-06	PTPRJ
Rejected	30	2.62175518684222e-10	0.74622098583493	0.552	0.437	8.79284254563143e-06	WWOX
Rejected	30	2.70845361048032e-10	0.32653223438052	0.36	0.213	9.0836117188289e-06	CSPP1
Rejected	30	2.72384227435835e-10	0.29570351357639	0.368	0.218	9.13522221974305e-06	AC087286.2
Rejected	30	2.79865671245406e-10	0.29906499367489	0.295	0.166	9.38613488222843e-06	IKBKB
Rejected	30	2.81144011076591e-10	0.38779923074176	0.33	0.195	9.42900784348671e-06	BBS9
Rejected	30	2.94965014833197e-10	0.44873836842993	0.547	0.386	9.89253666747575e-06	BACH1
Rejected	30	2.98949922892618e-10	0.32642861896575	0.32	0.184	1.00261825139726e-05	DAAM1
Rejected	30	3.04047164493669e-10	0.35936953183765	0.443	0.285	1.01971338027887e-05	LRCH3
Rejected	30	3.07158889487016e-10	0.33687953865141	0.212	0.102	1.03014948356155e-05	ANGPT2
Rejected	30	3.20033882980401e-10	0.35350709543750	0.479	0.318	1.07332963673967e-05	SFMBT2
Rejected	30	3.24208067091124e-10	0.27400344229412	0.302	0.169	1.08732901541021e-05	MBTD1
Rejected	30	3.43172009946331e-10	0.32680672810163	0.678	0.493	1.15093028695801e-05	NCOR1
Rejected	30	3.43463071682892e-10	0.32297312236041	0.264	0.144	1.15190644981008e-05	BIN3
Rejected	30	3.45479655346721e-10	0.26962848186180	0.35	0.206	1.15866966810183e-05	PHF12
Rejected	30	3.8435996789307e-10	0.35432960462110	0.368	0.225	1.28906646031978e-05	PIK3CA
Rejected	30	3.92770974631157e-10	0.28043495594948(	0.554	0.372	1.31727529471797e-05	MKL1
Rejected	30	3.95391220932218e-10	0.28831950777411	0.212	0.102	1.32606307676247e-05	AC005920.2
Rejected	30	4.10985207123352e-10	0.28561624245608	0.358	0.21	1.3783621876503e-05	PKP4
Rejected	30	4.25310773060105e-10	0.34147479690208	0.582	0.415	1.42640727068898e-05	SMC5
Rejected	30	4.32997989342978e-10	0.29033563022673	0.358	0.214	1.45218865665848e-05	KAT7
Rejected	30	4.3934961159923e-10	0.27005050457229	0.378	0.228	1.4734907273815e-05	ATP11C
Rejected	30	4.54923341255333e-10	0.29187430469713	0.297	0.169	1.52572190190214e-05	OSBP

Rejected	30	4.83470731604828e-10	0.37579325278205	0.557	0.391	1.62146413965627e-05	RALY
Rejected	30	4.85866988446605e-10	0.329479183284801	0.345	0.204	1.62950070585222e-05	ZNFX1
Rejected	30	4.98588480408038e-10	0.267348238257954	0.292	0.158	1.67216604559248e-05	PRKD3
Rejected	30	5.01272845368072e-10	0.303901313448587	0.315	0.182	1.68116886879544e-05	PPP1R13B
Rejected	30	5.24105349649154e-10	0.368538086279331	0.272	0.148	1.75774452165333e-05	EPB41L2
Rejected	30	5.40358252523466e-10	0.322143653421115	0.453	0.302	1.8122535073132e-05	USP10
Rejected	30	5.63000937863483e-10	0.303067793309587	0.31	0.18	1.88819254540655e-05	SMARCC2
Rejected	30	5.66934513657864e-10	0.352605604913816	0.466	0.306	1.90138497190574e-05	RALGAPA2
Rejected	30	5.72951146505136e-10	0.39150385824989	0.559	0.394	1.92156355514892e-05	FUT8
Rejected	30	5.994256229295e-10	0.422148631479021	0.161	0.071	2.01035365418096e-05	DIAPH2-AS1
Rejected	30	6.00167021203595e-10	0.312568638141585	0.423	0.272	2.01284015571262e-05	CYFIP2
Rejected	30	6.45342285086509e-10	0.324631340623917	0.32	0.186	2.16434895572314e-05	ERAP1
Rejected	30	6.52762532561811e-10	0.256482367072317	0.249	0.13	2.1892349817058e-05	CDS2
Rejected	30	6.53558881588476e-10	0.255581642402655	0.161	0.07	2.19190577707143e-05	MTMR2
Rejected	30	7.00444664638299e-10	0.451904183476396	0.703	0.581	2.34915131626393e-05	SMYD3
Rejected	30	7.00654711939386e-10	0.353648308732591	0.529	0.366	2.34985577290231e-05	FAM214A
Rejected	30	7.31314682207785e-10	0.37807973055064	0.511	0.349	2.45268318118847e-05	PVT1
Rejected	30	7.34112485680842e-10	0.283102218042653	0.3	0.166	2.46206645447641e-05	TTLL5
Rejected	30	7.50991882807495e-10	0.294840586150122	0.295	0.165	2.51867657655978e-05	CCDC93
Rejected	30	7.86918196803105e-10	0.339628708324346	0.514	0.356	2.63916624843825e-05	SNRK
Rejected	30	8.21083269252492e-10	0.281724517339941	0.859	0.711	2.75374906841901e-05	GPBP1
Rejected	30	8.49731274403695e-10	0.27524362472919	0.421	0.27	2.84982874809511e-05	IPO7
Rejected	30	8.86509864666341e-10	0.423771443332397	0.453	0.305	2.97317678411798e-05	N4BP1
Rejected	30	9.35051554368227e-10	0.343836576485653	0.411	0.261	3.13597590304016e-05	AC104365.1
Rejected	30	9.44145534600493e-10	0.364213713920114	0.398	0.255	3.16647529394314e-05	XPO4
Rejected	30	9.45597912039241e-10	0.256039499179818	0.506	0.342	3.17134627739721e-05	PRRC2B
Rejected	30	9.78540500160795e-10	0.280798745376322	0.511	0.335	3.28182912943928e-05	CARD11
Rejected	30	1.02247185312207e-09	0.267202839965967	0.61	0.435	3.42916610100079e-05	RAB2A
Rejected	30	1.07421250169075e-09	0.28520779329621	0.272	0.152	3.60269388817045e-05	NCOR2
Rejected	30	1.08535542609762e-09	0.39592542120081	0.499	0.345	3.64006502804619e-05	TNRC6C
Rejected	30	1.12208207008688e-09	0.329369615929912	0.388	0.24	3.76323884665738e-05	SMURF1

Rejected	30	1.14408897865995e-09	0.293405805436679	0.249	0.133	3.83704561662973e-05	SLX4IP
Rejected	30	1.15031890115036e-09	0.275488680157271	0.312	0.179	3.85793953067808e-05	VPS41
Rejected	30	1.15538703715231e-09	0.330624376392354	0.705	0.55	3.87493704520143e-05	PUM2
Rejected	30	1.17054142845142e-09	0.289017232880218	0.446	0.294	3.92576184274038e-05	ILF3
Rejected	30	1.21738349769145e-09	0.278164108578513	0.698	0.529	4.08286077455759e-05	CAMK2D
Rejected	30	1.21768911583206e-09	0.329933953240914	0.531	0.376	4.08388575667757e-05	WDR70
Rejected	30	1.23943710991561e-09	0.415848714402085	0.355	0.221	4.15682417923497e-05	SEPT11
Rejected	30	1.26936587447526e-09	0.271499263764739	0.673	0.509	4.25719926981514e-05	RBM25
Rejected	30	1.38498732724461e-09	0.32014534003686	0.599	0.43	4.64497049811297e-05	AP3B1
Rejected	30	1.38709773916106e-09	0.298743845391467	0.3	0.173	4.65204839759835e-05	SF3B3
Rejected	30	1.44401999232268e-09	0.349701731700729	0.426	0.278	4.84295425025181e-05	CRTC3
Rejected	30	1.51968570046844e-09	0.31684176899165	0.577	0.41	5.09672190223107e-05	CDK13
Rejected	30	1.5210083570598e-09	0.319950728387732	0.708	0.559	5.10115782790717e-05	SERINC5
Rejected	30	1.5348446205244e-09	0.412186021982527	0.458	0.312	5.14756188831474e-05	WDR7
Rejected	30	1.54001966823592e-09	0.323869249029868	0.897	0.796	5.16491796332962e-05	STK4
Rejected	30	1.54479354173158e-09	0.350169282047183	0.861	0.734	5.18092858025939e-05	RHOH
Rejected	30	1.55051991829629e-09	0.32164445691449	0.39	0.245	5.20013370198208e-05	ANO6
Rejected	30	1.55858609999468e-09	0.456070581281979	0.431	0.282	5.22718606216215e-05	FAM13A
Rejected	30	1.60107169721081e-09	0.333635143391854	0.345	0.209	5.36967425810562e-05	CLTC
Rejected	30	1.60459371920907e-09	0.289008155590676	0.572	0.41	5.38148641548339e-05	CEP350
Rejected	30	1.61497253253362e-09	0.327144782392228	0.748	0.594	5.41629487961124e-05	WAC
Rejected	30	1.63186717853784e-09	0.268640867204552	0.194	0.094	5.47295614338021e-05	HEG1
Rejected	30	1.69590432662469e-09	0.391387765190682	0.398	0.251	5.68772393063388e-05	NFATC1
Rejected	30	1.70573776842443e-09	0.34524089046393	0.537	0.374	5.72070332774184e-05	ELF2
Rejected	30	1.7376791753826e-09	0.309818457783188	0.597	0.415	5.82782841839816e-05	CREBRF
Rejected	30	1.78629205336332e-09	0.28830028135561	0.272	0.152	5.99086628856989e-05	FAM117A
Rejected	30	1.82529630935072e-09	0.281348586397115	0.438	0.288	6.12167876230046e-05	MAPK1
Rejected	30	1.82643421128934e-09	0.297437206452256	0.526	0.365	6.1254950578222e-05	STIM2
Rejected	30	1.82813905204774e-09	0.367156418139281	0.529	0.361	6.13121275275772e-05	ITPKB
Rejected	30	1.8661376744631e-09	0.366691496711884	0.202	0.102	6.25865253261433e-05	AP002336.2
Rejected	30	1.94320884436875e-09	0.344867719741092	0.431	0.282	6.51713382224391e-05	OXSR1

Rejected	30	1.96394836500439e-09	0.52249042306275	0.373	0.242	6.58669002655172e-05	PRKN
Rejected	30	2.04823214860282e-09	0.34579380016832	0.469	0.318	6.86936097998413e-05	STXBP5
Rejected	30	2.08117162063555e-09	0.35614171943979	0.307	0.182	6.9798333812875e-05	RNF24
Rejected	30	2.12471212340521e-09	0.39804297196309	0.597	0.438	7.12585951947638e-05	ARHGEF3
Rejected	30	2.18445264346547e-09	0.38269847134167	0.542	0.384	7.3262172756545e-05	SMURF2
Rejected	30	2.1889329608854e-09	0.28060975042284	0.239	0.126	7.34124336421747e-05	EDRF1
Rejected	30	2.23898081174603e-09	0.27672935768655	0.29	0.164	7.50909384643383e-05	MAD1L1
Rejected	30	2.26014043440359e-09	0.36971293629969	0.657	0.493	7.58005898890277e-05	SRPK2
Rejected	30	2.45546773778895e-09	0.30740017629758	0.254	0.14	8.23514769899657e-05	ECE1
Rejected	30	2.49070304787854e-09	0.35682633710983	0.395	0.251	8.35331988197504e-05	TRERF1
Rejected	30	2.51805247555882e-09	0.41054080779074	0.126	0.05	8.44504439252918e-05	EDARADD
Rejected	30	2.5350772128195e-09	0.27113118279580	0.504	0.338	8.50214195635405e-05	SETD5
Rejected	30	2.57234210884338e-09	0.33216786906919	0.373	0.235	8.62712096463893e-05	ABCF1
Rejected	30	2.58919465972283e-09	0.32880137136934	0.353	0.215	8.68364104977842e-05	GPATCH2L
Rejected	30	2.59288809980632e-09	0.37931665937538	0.552	0.407	8.69602810913045e-05	SPIDR
Rejected	30	2.62616894650595e-09	0.29852834927091	0.343	0.208	8.80764541279164e-05	TAF1
Rejected	30	2.65976869661868e-09	0.28293230468159	0.156	0.069	8.92033225471972e-05	AL513188.1
Rejected	30	2.69913442515716e-09	0.36801469346896	0.617	0.474	9.05235703509209e-05	HIF1A
Rejected	30	2.71021611260664e-09	0.29033516343861	0.499	0.328	9.08952279846016e-05	ZDHHC20
Rejected	30	2.7113400962011e-09	0.31600025588208	0.65	0.501	9.09329241463924e-05	FRYL
Rejected	30	2.98448228255642e-09	0.32102554941630	0.453	0.301	0.000100093566792377	GTF2I
Rejected	30	2.99000318514109e-09	0.28447497636564	0.642	0.468	0.000100278726823262	HNRNPM
Rejected	30	3.07507765345867e-09	0.33975641538468	0.431	0.28	0.000103131954341697	PAM
Rejected	30	3.10646270161964e-09	0.29160696569315	0.398	0.253	0.000104184546086919	CD226
Rejected	30	3.15897637159078e-09	0.27183649361742	0.297	0.173	0.000105945749550412	OSER1
Rejected	30	3.16586894006167e-09	0.38024987419966	0.534	0.379	0.000106176912511788	FBXO34
Rejected	30	3.20894343131859e-09	0.27070255799085	0.466	0.309	0.000107621544799563	USP24
Rejected	30	3.21736784182013e-09	0.34125756340781	0.287	0.163	0.000107904082678963	DDX60L
Rejected	30	3.37848322733785e-09	0.29430806762236	0.751	0.591	0.000113307570478457	ADK
Rejected	30	3.39168880406802e-09	0.27459581694870	0.38	0.237	0.000113750459110833	METTL15
Rejected	30	3.4243419392213e-09	0.27995368353703	0.39	0.243	0.000114845579957604	NR1D2

Rejected	30	3.6090329705666e-09	0.266915693251482	0.327	0.196	0.000121039747766863	MDM2
Rejected	30	3.71093722135287e-09	0.320021467545923	0.3	0.173	0.000124457412529733	ADAMTS17
Rejected	30	3.82954635244559e-09	0.31023541706662	0.627	0.446	0.00012843532556832	OGT
Rejected	30	3.87905670782648e-09	0.32055784839082	0.406	0.266	0.000130095803867085	SNX29
Rejected	30	4.13768876007028e-09	0.285192033167624	0.335	0.205	0.000138769805635237	RALGAPB
Rejected	30	4.19923959091563e-09	0.271839455515052	0.176	0.083	0.000140834097400128	AC093019.2
Rejected	30	4.32945142636626e-09	0.257231582679007	0.383	0.243	0.000145201141937472	RAD23B
Rejected	30	4.34931177047777e-09	0.314491096843908	0.776	0.624	0.000145867218158284	PDE7A
Rejected	30	4.4938873475859e-09	0.252513000115252	0.766	0.605	0.000150715993863336	TRA2B
Rejected	30	4.51734690447925e-09	0.28265965324609	0.35	0.214	0.000151502780482425	NLRC5
Rejected	30	4.583649571454e-09	0.250618102284947	0.302	0.175	0.000153726439327424	SLC36A4
Rejected	30	4.58530411664961e-09	0.308126674874956	0.358	0.221	0.000153781929464195	DDX10
Rejected	30	4.7944062319414e-09	0.273156034150625	0.282	0.161	0.000160794796206851	RPN1
Rejected	30	4.89870157835824e-09	0.278121928431389	0.128	0.052	0.000164292653534979	SRGAP1
Rejected	30	4.92788470007968e-09	0.263071843695618	0.322	0.191	0.000165271397071272	SBNO1
Rejected	30	4.95338111458527e-09	0.354551056901596	0.285	0.164	0.000166126495820961	KLHL2
Rejected	30	4.98504366884443e-09	0.307460389947971	0.317	0.191	0.000167188394565705	DGKD
Rejected	30	5.09602342552478e-09	0.305010704578778	0.778	0.617	0.00017091043364525	FTX
Rejected	30	5.26806095736799e-09	0.290414954378546	0.572	0.405	0.000176680228388208	CASP8
Rejected	30	5.42776879475283e-09	0.327463936520797	0.438	0.294	0.000182036509838421	PITPNB
Rejected	30	5.64354179395969e-09	0.46111235466373	0.426	0.295	0.00018927310468582	SCFD2
Rejected	30	5.69042241606976e-09	0.313914910416453	0.481	0.331	0.000190845386990148	NDEL1
Rejected	30	5.69098886437791e-09	0.304748452399416	0.625	0.453	0.000190864384533506	KIAA1109
Rejected	30	5.78735345343653e-09	0.266749002918053	0.282	0.161	0.000194096260121354	PTK2
Rejected	30	5.94746265419615e-09	0.279996275969456	0.471	0.312	0.000199466002496431	STRN3
Rejected	30	5.97223483706459e-09	0.308846507237741	0.436	0.288	0.000200296811965472	AZIN1
Rejected	30	5.99082659413604e-09	0.26758817312219	0.448	0.3	0.000200920342314134	TRIM33
Rejected	30	6.00072527869986e-09	0.291263059884186	0.27	0.154	0.000201252324397036	SLC23A2
Rejected	30	6.03896370881701e-09	0.337615066153792	0.448	0.302	0.000202534764866305	MAP2K4
Rejected	30	6.20550472956589e-09	0.279364762984733	0.965	0.863	0.000208120217620181	PPP2R5C
Rejected	30	6.4134791390308e-09	0.299496929158566	0.587	0.425	0.000215095263364815	UBE2K

Rejected	30	6.65035103691768e-09	0.31139839232038	0.481	0.333	0.000223039473076145	PCNX2
Rejected	30	6.70817225080051e-09	0.34300336413007	0.496	0.353	0.000224978680947347	TAOK1
Rejected	30	6.77348826358769e-09	0.28910446099369	0.461	0.307	0.000227169249384204	MTREX
Rejected	30	6.98804445558605e-09	0.35394816014123	0.559	0.397	0.000234365034951445	DIAPH2
Rejected	30	7.17715425724239e-09	0.36006422620425	0.365	0.227	0.000240707399479395	MFSD6
Rejected	30	7.38339198113593e-09	0.26154284572937	0.97	0.898	0.000247624200263337	TNFAIP3
Rejected	30	7.40815334451582e-09	0.34580752736012	0.481	0.338	0.000248454646868372	OGDH
Rejected	30	7.64177804408184e-09	0.36596140887033	0.401	0.264	0.000256289952042417	AGTPBP1
Rejected	30	7.90843864392583e-09	0.29156312106852	0.705	0.542	0.000265233215239985	SCAF11
Rejected	30	8.32911462474117e-09	0.28191341650635	0.569	0.415	0.00027934184628457	SMG1
Rejected	30	8.72618622102270e-09	0.36611957243401	0.33	0.205	0.000292658833480659	INVS
Rejected	30	8.77139594283479e-09	0.31769280868897	0.378	0.245	0.000294175077130793	NDRG1
Rejected	30	8.79596679196655e-09	0.25029591516439	0.501	0.346	0.000294999134268974	FAM13B
Rejected	30	8.97579874620283e-09	0.32246441021619	0.607	0.451	0.000301030338350151	DLG1
Rejected	30	9.15141908006869e-09	0.26045005661192	0.214	0.111	0.000306920293107344	SPRED2
Rejected	30	9.48846314380312e-09	0.35482146164882	0.768	0.641	0.000318224076916869	RALGAPA1
Rejected	30	9.6009171670249e-09	0.47126819284838	0.189	0.094	0.00032199555994768	FMN1
Rejected	30	9.71360032988698e-09	0.33331264960954	0.753	0.603	0.000325774727863749	KANSL1
Rejected	30	9.7937874519212e-09	0.25859648176656	0.552	0.397	0.000328464043562533	CCDC88C
Rejected	30	1.02185064441601e-08	0.37960419755276	0.37	0.239	0.000342708269124241	WWP2
Rejected	30	1.08127527774381e-08	0.29378431161385	0.383	0.246	0.000362638102649719	SUPT5H
Rejected	30	1.15946812193404e-08	0.29763582645276	0.655	0.489	0.000388862418734239	ACAP2
Rejected	30	1.17491507661413e-08	0.27569064964154	0.385	0.244	0.000394043018394849	LRCH1
Rejected	30	1.17503104796708e-08	0.31129317227794	0.363	0.235	0.000394081912867199	BRD1
Rejected	30	1.19248982125334e-08	0.30771481004768	0.65	0.478	0.000399937236251944	NCK2
Rejected	30	1.21647406890225e-08	0.30680980538725	0.476	0.325	0.000407981073228437	SYNCRIP
Rejected	30	1.25040883725721e-08	0.29654277568779	0.262	0.15	0.000419362115839324	SFMBT1
Rejected	30	1.26708470145386e-08	0.26297812182763	0.229	0.124	0.000424954867173596	RPRD1B
Rejected	30	1.27291590576624e-08	0.27214197711551	0.338	0.207	0.000426910536475882	TRPC4AP
Rejected	30	1.27640214686328e-08	0.30041727487749	0.469	0.316	0.000428079752015008	PREX1
Rejected	30	1.28379718813684e-08	0.26779991744578	0.322	0.196	0.000430559900957332	ARHGAP17

Rejected	30	1.40472331075841e-08	0.393616977034001	0.574	0.442	0.000471116103962156	NR3C2
Rejected	30	1.42302728209734e-08	0.275104537859555	0.395	0.259	0.000477254889869806	UBA6
Rejected	30	1.43820719516304e-08	0.254027446705779	0.262	0.151	0.000482345929113781	TENT4A
Rejected	30	1.44205887213332e-08	0.316983899995633	0.537	0.388	0.000483637704536073	UBR1
Rejected	30	1.6159243493814e-08	0.314669425612043	0.431	0.297	0.000541948708295532	USP12
Rejected	30	1.64501137086325e-08	0.308280554405358	0.403	0.268	0.000551703913560116	DCTN4
Rejected	30	1.70842222882832e-08	0.300616029333763	0.627	0.453	0.000572970647104442	STT3B
Rejected	30	1.72926165690486e-08	0.37994926187086	0.262	0.152	0.000579959774492751	HLCS
Rejected	30	1.76914225485019e-08	0.306246421182591	0.458	0.319	0.000593334929431657	CUL1
Rejected	30	1.79430785665575e-08	0.258682053494997	0.272	0.159	0.000601774968965205	BSDC1
Rejected	30	1.80850706289628e-08	0.26017526714937	0.401	0.262	0.000606537098754154	POGZ
Rejected	30	1.82313305759359e-08	0.353685942461581	0.398	0.266	0.000611442364855738	PPP2R5A
Rejected	30	1.84511659836547e-08	0.355022703943779	0.272	0.162	0.000618815204759813	IPMK
Rejected	30	1.8956637062876e-08	0.265825587733266	0.123	0.051	0.000635767693814736	AC024145.1
Rejected	30	1.90789738234956e-08	0.351119528757456	0.65	0.502	0.000639870624092396	JARID2
Rejected	30	2.00282502225785e-08	0.700462040253014	0.267	0.156	0.000671707455964838	AOAH
Rejected	30	2.03463637909638e-08	0.317493842905091	0.411	0.27	0.000682376348821345	UHRF2
Rejected	30	2.14401048195118e-08	0.255104222868751	0.395	0.26	0.000719058235436785	KPNA1
Rejected	30	2.15549816349451e-08	0.316826344520301	0.584	0.439	0.000722910974072788	ZFAND6
Rejected	30	2.21719829782165e-08	0.254297268110152	0.242	0.134	0.000743603965123425	TRIM56
Rejected	30	2.24531429040396e-08	0.313186789038299	0.322	0.202	0.000753033506715681	HECTD4
Rejected	30	2.29919896280646e-08	0.313355633036285	0.302	0.185	0.00077110534814603	MCTP2
Rejected	30	2.33494101239628e-08	0.311696195203079	0.418	0.283	0.000783092516737465	PRKX
Rejected	30	2.36493051059666e-08	0.436853906313079	0.577	0.429	0.000793150394643907	BCAS3
Rejected	30	2.41351337168227e-08	0.257899934568463	0.605	0.446	0.0008094441145948	GPATCH8
Rejected	30	2.52798134310539e-08	0.315819210003383	0.627	0.468	0.000847834382850686	XRN1
Rejected	30	2.52972639249963e-08	0.354048852035047	0.31	0.19	0.000848419637516526	AC092652.2
Rejected	30	2.59546378466051e-08	0.42988298402865	0.511	0.371	0.000870466644099441	XYLT1
Rejected	30	2.66465885896739e-08	0.368856724922069	0.406	0.272	0.000893673288120483	PDSS2
Rejected	30	2.70792873902252e-08	0.298928259512276	0.222	0.122	0.000908185140493372	GGA2
Rejected	30	2.75575179034691e-08	0.256466483059766	0.557	0.404	0.000924224035446546	ASCC3

Rejected	30	2.79190267816753e-08	0.330030674877305	0.385	0.259	0.000936348320203827	SNX13
Rejected	30	3.08174651588391e-08	0.254675911783023	0.123	0.053	0.00103355614649715	SEMA5A
Rejected	30	3.19222440079621e-08	0.285079512238633	0.526	0.372	0.00107060821953903	BRWD1
Rejected	30	3.47546623541525e-08	0.327191135263495	0.312	0.193	0.00116560186603357	FAM222B
Rejected	30	3.54295416303672e-08	0.250942830860198	0.401	0.26	0.00118823596719925	CANX
Rejected	30	3.6402669019104e-08	0.319312980591786	0.547	0.391	0.00122087271356271	CLASP2
Rejected	30	3.90364038349431e-08	0.29990664559729	0.307	0.192	0.00130920291181632	HPCAL1
Rejected	30	3.97633915456374e-08	0.312568297200047	0.257	0.147	0.00133358462565759	CCDC7
Rejected	30	4.03739889417604e-08	0.284183926110911	0.504	0.353	0.00135406284112876	VWA8
Rejected	30	4.08405705853385e-08	0.315144639388312	0.552	0.412	0.00136971105629108	PBX4
Rejected	30	4.20563390783137e-08	0.282536680448655	0.27	0.158	0.00141048550000849	INKA2
Rejected	30	4.30695128695271e-08	0.268662933198837	0.438	0.3	0.0014444653226182	WAPL
Rejected	30	4.40372403797233e-08	0.255613562893388	0.547	0.391	0.00147692096785516	SAMHD1
Rejected	30	4.47381041229496e-08	0.311048465341629	0.34	0.219	0.00150042653607548	ARNTL
Rejected	30	4.5957290906815e-08	0.310572545488328	0.489	0.346	0.00154131562243276	TAF3
Rejected	30	4.77483531585329e-08	0.37811390401162	0.27	0.16	0.00160138426823088	NLK
Rejected	30	4.93527859551959e-08	0.328590573541907	0.373	0.244	0.00165519373536536	PFKFB3
Rejected	30	5.04733043216846e-08	0.273776593851424	0.438	0.304	0.00169277368034066	ZZEF1
Rejected	30	5.12565238772927e-08	0.306346376052652	0.408	0.277	0.00171904129779664	XKR6
Rejected	30	5.27283077140395e-08	0.256106102448945	0.388	0.254	0.00176840198411346	ANKRD36
Rejected	30	5.29321716205879e-08	0.283362760034453	0.393	0.254	0.00177523917181128	ARHGAP25
Rejected	30	5.51444428066347e-08	0.268767835184643	0.295	0.176	0.00184943432284891	ZNF107
Rejected	30	5.93142412568541e-08	0.342151248541036	0.365	0.24	0.00198928102327237	TRIM44
Rejected	30	5.93976038201446e-08	0.256958054318058	0.322	0.2	0.00199207683692001	CDC5L
Rejected	30	5.95492191734449e-08	0.25992557072005	0.63	0.468	0.00199716171263899	SLTM
Rejected	30	5.99741557877779e-08	0.286133664150847	0.642	0.49	0.00201141323681049	RNF149
Rejected	30	6.02071101049483e-08	0.304186617042701	0.345	0.221	0.00201922605869976	TASP1
Rejected	30	6.28136372102151e-08	0.368308309698565	0.594	0.441	0.00210664376475619	SOS1
Rejected	30	6.62838290139954e-08	0.293879839690153	0.426	0.297	0.00222302705747138	RBM5
Rejected	30	6.66200522676911e-08	0.25310611071223	0.322	0.202	0.00223430331295382	FBXW4
Rejected	30	6.72421952657078e-08	0.437153650052122	0.385	0.265	0.00225516874482131	SUMF1

Rejected	30	6.89361399496241e-08	0.25165655524555€	0.547	0.401	0.00231198026163049	FOXO3
Rejected	30	7.32314465541651e-08	0.28770248231740€	0.703	0.564	0.00245603625453359	CD55
Rejected	30	7.33916223721253e-08	0.25152453912417⁄	0.589	0.435	0.00246140823111634	RSF1
Rejected	30	7.35103247187821e-08	0.27656904067068	0.673	0.527	0.00246538927041851	PPP1R12A
Rejected	30	7.43708073183013e-08	0.27300554625181́1	0.378	0.253	0.00249424813584119	WDR43
Rejected	30	7.46173764351301e-08	0.30105301270593	0.587	0.453	0.00250251757088139	CCNY
Rejected	30	7.4823460518988e-08	0.27909556461984	0.38	0.247	0.00250942921888582	SEC14L1
Rejected	30	8.35948724579203e-08	0.28175147244931	0.212	0.117	0.00280360483249373	KAZN
Rejected	30	8.70565170440047e-08	0.27771262447465€	0.592	0.449	0.00291970146862183	PAG1
Rejected	30	8.73097429472625e-08	0.25247382745356	0.181	0.094	0.00292819415896529	TOM1L2
Rejected	30	8.79444417752533e-08	0.34057884868185€	0.612	0.463	0.00294948068825845	LPIN2
Rejected	30	8.89367840790704e-08	0.28149651090465	0.38	0.252	0.00298276186444386	ATXN10
Rejected	30	9.15345975116527e-08	0.30623779834794	0.373	0.248	0.00306988733134581	PRKAG2
Rejected	30	9.2905009167515e-08	0.33512047644751	0.244	0.138	0.00311584819746012	MLF1
Rejected	30	9.52521189762581e-08	0.25437749904359	0.338	0.213	0.00319456556622575	PPTC7
Rejected	30	9.59971881856941e-08	0.36184953159367	0.479	0.348	0.00321955369737181	CHCHD3
Rejected	30	9.60007403288189e-08	0.29629169624755	0.232	0.131	0.00321967282914793	PIAS2
Rejected	30	9.62293135627125e-08	0.30180441632625	0.325	0.209	0.00322733871826625	MDN1
Rejected	30	9.74163968644397e-08	0.30810605337142⁄	0.27	0.159	0.00326715111803958	ZBTB20-AS5
Rejected	30	9.89097597305754e-08	0.40804971188381€	0.531	0.399	0.00331723552184404	COMMD10
Rejected	30	1.01848891644087e-07	0.28599224161383	0.38	0.25	0.00341580812795938	TNKS2
Rejected	30	1.11366597170587e-07	0.31520078022579	0.456	0.321	0.00373501293590713	ZBTB10
Rejected	30	1.12249229942484e-07	0.30350506295852	0.458	0.323	0.00376461467381104	TGFB1
Rejected	30	1.12637063403593e-07	0.53983207043707€	0.202	0.111	0.00377762183242969	MX1
Rejected	30	1.13732688091723e-07	0.28632637361518€	0.7	0.54	0.00381436689322021	ERBIN
Rejected	30	1.14112854460703e-07	0.36297450435344	0.3	0.19	0.00382711691290307	FAM126B
Rejected	30	1.18808432296998e-07	0.31399039902686⁄	0.537	0.397	0.00398459720237671	ITFG1
Rejected	30	1.229435597705e-07	0.32729263598190	0.683	0.565	0.00412328110758304	PPP3CA
Rejected	30	1.22952946319554e-07	0.27439578705617€	0.28	0.169	0.00412359591366519	NUP160
Rejected	30	1.29972325746854e-07	0.34636093695093⁄	0.655	0.516	0.00435901186089799	BAZ1A
Rejected	30	1.33826160112778e-07	0.40799364790949	0.426	0.291	0.00448826175786234	JAZF1

Rejected	30	1.36803840016228e-07	0.280708349503342	0.348	0.223	0.00458812718646426	ATP2C1
Rejected	30	1.41970775626587e-07	0.276919495849874	0.194	0.104	0.00476141587296447	PSD3
Rejected	30	1.42994162739543e-07	0.266075398649995	0.29	0.18	0.00479573822995878	CTNNA1
Rejected	30	1.42996283206956e-07	0.43345680269215	0.715	0.611	0.0047958093461949	BCL2
Rejected	30	1.44278704540978e-07	0.260533327789163	0.458	0.32	0.00483881919289533	HBS1L
Rejected	30	1.57330097399022e-07	0.296123891278613	0.373	0.251	0.00527653680656842	TYW1
Rejected	30	1.62452156857412e-07	0.382551316085349	0.426	0.302	0.00544832043668389	MYO1D
Rejected	30	1.63161974851736e-07	0.348173411805411	0.327	0.217	0.00547212631257751	REPS1
Rejected	30	1.68146128542896e-07	0.34676380945359	0.582	0.441	0.00563928485907165	ABLIM1
Rejected	30	1.6853793196764e-07	0.325312473913759	0.688	0.563	0.0056524251623307	GMDS
Rejected	30	1.78393628473013e-07	0.259728335974872	0.557	0.397	0.00598296551172789	PPP4R3A
Rejected	30	1.78507381813909e-07	0.403744774151399	0.766	0.622	0.00598678057127488	SCML4
Rejected	30	1.84371493007088e-07	0.284537377945442	0.219	0.124	0.00618345113247171	TRIO
Rejected	30	1.90035650372062e-07	0.302142704786056	0.33	0.215	0.0063734156421782	MAP4K3
Rejected	30	1.92340292298728e-07	0.305096009458641	0.65	0.512	0.00645070872311474	EIF4B
Rejected	30	2.12240583382211e-07	0.253906762367015	0.237	0.138	0.00711812468547258	GMPS
Rejected	30	2.24510726296285e-07	0.321979795962458	0.212	0.12	0.00752964073852482	FGGY
Rejected	30	2.28251272277449e-07	0.260989104481821	0.312	0.2	0.00765509116964108	PPM1D
Rejected	30	2.28656510204751e-07	0.291087476082697	0.572	0.428	0.00766868203924694	CENPC
Rejected	30	2.36523868000386e-07	0.312042229197206	0.259	0.158	0.00793253748499694	CEP83
Rejected	30	2.37269792648213e-07	0.296455078549121	0.504	0.373	0.00795755430583577	PNPLA8
Rejected	30	2.40276617352122e-07	0.344066518809719	0.108	0.046	0.00805839719275545	SEMA4A
Rejected	30	2.41044513098346e-07	0.251253615707634	0.29	0.181	0.00808415088029233	ENTPD4
Rejected	30	2.47806733927306e-07	0.306013132374478	0.242	0.143	0.00831094224245398	GALNT2
Rejected	30	2.50461065413367e-07	0.295197713741499	0.189	0.1	0.00839996321183351	MYBL1
Rejected	30	2.51725213465673e-07	0.353365599995887	0.554	0.421	0.00844236020921175	DNAJC1
Rejected	30	2.55609021604961e-07	0.339230521139596	0.801	0.682	0.00857261536658717	CCND3
Rejected	30	2.56314911159294e-07	0.268827058853854	0.217	0.122	0.00859628949046039	ETFDH
Rejected	30	2.56495491154627e-07	0.351489087936282	0.275	0.174	0.00860234578234387	AGAP1
Rejected	30	2.58669387515056e-07	0.305495539629128	0.229	0.132	0.00867525391847995	AC026347.1
Rejected	30	2.5957630441535e-07	0.296156302670549	0.327	0.214	0.00870567009748199	GNB5

Rejected	30	2.60438754520519e-07	0.35146910280702[illegible]	0.332	0.214	0.00873459494910918	HIVEP3
Rejected	30	2.8799169793197e-07	0.31691990673275[illegible]	0.358	0.243	0.00965866556524241	HSD17B12
Rejected	30	2.8884985510514e-07	0.311192790761735	0.297	0.189	0.00968744644051618	FRS2
Rejected	30	3.04822102991572e-07	0.27164505360242[illegible]	0.136	0.065	0.0102231236901313	DOCK7
Rejected	30	3.07402973117955e-07	0.31660186911600[illegible]	0.474	0.344	0.01030968091243	THADA
Rejected	30	3.19154165347039e-07	0.28072861014277	0.353	0.233	0.010703792397409	PIK3C2A
Rejected	30	3.30009804271095e-07	0.26104864939458[illegible]	0.385	0.261	0.011067868815644	ZC3H7A
Rejected	30	3.30712603711859e-07	0.31850703258028	0.524	0.393	0.0110914393032883	ATP2B4
Rejected	30	3.48789421476909e-07	0.34668641162937[illegible]	0.662	0.533	0.0116976996174926	TTC39C
Rejected	30	3.60297621679777e-07	0.25144521562939[illegible]	0.481	0.348	0.0120836616358964	LSM14A
Rejected	30	3.61887700476422e-07	0.34660369615404	0.34	0.225	0.0121369896985782	TMCC1
Rejected	30	3.7690849341537e-07	0.27665216669631[illegible]	0.844	0.693	0.0126407570521647	PIP4K2A
Rejected	30	4.12036124322416e-07	0.28977518258074[illegible]	0.484	0.356	0.0138188675375252	TRAPPC10
Rejected	30	4.15454840269762e-07	0.26788499333268	0.567	0.427	0.0139335244329673	SPAG9
Rejected	30	4.2462679126357e-07	0.29861157109156[illegible]	0.332	0.221	0.0142411333253976	SLC30A7
Rejected	30	4.27666823795188e-07	0.25385510503504	0.322	0.205	0.014343089936443	DGLUCY
Rejected	30	4.60644831165271e-07	0.318584611443181	0.32	0.209	0.0154491063476208	ATXN7L1
Rejected	30	4.74370737246646e-07	0.28559315918357[illegible]	0.426	0.302	0.015909445785778	CAMTA1
Rejected	30	4.74645144202533e-07	0.26242466823972	0.31	0.199	0.0159186488462646	ADAM17
Rejected	30	4.81510212411821e-07	0.43775685672166[illegible]	0.463	0.351	0.0161488895038676	UMAD1
Rejected	30	4.91839429962548e-07	0.2568098834078	0.798	0.669	0.0164953108020839	CAMK4
Rejected	30	4.92274277019726e-07	0.36952806597875[illegible]	0.688	0.546	0.0165098947026876	GPRIN3
Rejected	30	4.98069710414448e-07	0.26161061457692[illegible]	0.569	0.422	0.0167042619478798	NASP
Rejected	30	5.00960771117882e-07	0.26393042755557[illegible]	0.33	0.212	0.0168012223417515	AHCYL2
Rejected	30	5.03503254416091e-07	0.25614395487877[illegible]	0.844	0.681	0.0168864921466068	ZC3HAV1
Rejected	30	5.05786334333239e-07	0.26133609682157[illegible]	0.426	0.299	0.0169630620808682	SCFD1
Rejected	30	5.28201201672874e-07	0.30146608985477	0.116	0.052	0.0177148119017048	PHLDB2
Rejected	30	5.37243059652958e-07	0.38613370463800[illegible]	0.358	0.241	0.0180180577346409	TAF4B
Rejected	30	5.39948115003666e-07	0.28024126899220[illegible]	0.239	0.144	0.018108779880993	ZC3H18
Rejected	30	5.46009196373187e-07	0.25564579207098[illegible]	0.207	0.117	0.018312056427964	SART3
Rejected	30	5.72729366543792e-07	0.34366041835704[illegible]	0.486	0.354	0.0192081974951457	CAMKMT

Rejected	30	6.01424000010822e-07	0.251218567318612	0.272	0.168	0.0201705581123629	NUS1
Rejected	30	6.17983413626653e-07	0.303889774586756	0.831	0.703	0.0207259277262107	EPC1
Rejected	30	6.44613821834996e-07	0.293252318764146	0.264	0.163	0.0216190583567021	USF3
Rejected	30	6.69153565172589e-07	0.369005397461814	0.66	0.54	0.0224420722687583	ANKRD28
Rejected	30	7.2685497898947e-07	0.297447038374494	0.37	0.247	0.0243772622853488	WARS2
Rejected	30	7.4328334839112e-07	0.353069112729842	0.171	0.092	0.0249282369383414	PAQR3
Rejected	30	7.48240466270466e-07	0.298774051285719	0.471	0.351	0.0250944887577789	FAM208B
Rejected	30	8.2704301572511e-07	0.33531419564135	0.375	0.267	0.0277373686613888	ANKH
Rejected	30	8.52861127797808e-07	0.357777292566952	0.516	0.376	0.0286032565040829	GBP2
Rejected	30	8.75450207932989e-07	0.310519054896592	0.257	0.158	0.0293608490736566	RNF157
Rejected	30	8.89928346069173e-07	0.314713244599971	0.219	0.128	0.0298464168704679	ZDHHC14
Rejected	30	9.05029547756437e-07	0.30931729343079	0.463	0.343	0.0303528809726554	TULP4
Rejected	30	9.13439543190658e-07	0.253577165389337	0.479	0.356	0.0306349353995283	RC3H1
Rejected	30	1.01021405781694e-06	0.388210380793203	0.292	0.194	0.0338805590710644	AC008014.1
Rejected	30	1.0270602346058e-06	0.268309870536286	0.635	0.491	0.0344455461482095	ROCK1
Rejected	30	1.04833485626547e-06	0.277207455192449	0.345	0.235	0.0351590544094314	WDR20
Rejected	30	1.04942547861935e-06	0.37150254018036	0.275	0.173	0.0351956317019357	NELL2
Rejected	30	1.05084216378025e-06	0.256958266300089	0.441	0.313	0.0352431444888619	CAB39
Rejected	30	1.06593652926069e-06	0.319721655470003	0.385	0.273	0.0357493793183452	ACBD6
Rejected	30	1.08336358516932e-06	0.269753379585972	0.141	0.071	0.0363338479194087	RAPH1
Rejected	30	1.12450773699135e-06	0.281683739955403	0.237	0.141	0.0377137404832157	ACVR2A
Rejected	30	1.1723111083619e-06	0.4061416914884	0.428	0.306	0.0393169699522413	FILIP1L
Rejected	30	1.18022943335656e-06	0.257390059862683	0.413	0.293	0.0395825347359124	RPRD2
Rejected	30	1.18881310548699e-06	0.327970949253062	0.315	0.206	0.0398704139318226	WDPCP
Rejected	30	1.25907929557865e-06	0.274158306216952	0.32	0.211	0.0422270014151168	DTNB
Rejected	30	1.29083899746436e-06	0.27219603460733	0.365	0.25	0.0432921582969596	MSH3
Rejected	30	1.30043352108244e-06	0.262211312891696	0.237	0.143	0.043613939430063	MARF1
Rejected	30	1.30853743966986e-06	0.266733654268812	0.617	0.48	0.0438857286516478	EMB
Rejected	30	1.38629728869023e-06	0.298161873851011	0.408	0.287	0.046493638468093	CHD7
Rejected	30	1.39763440140362e-06	0.319184046679621	0.249	0.153	0.0468738625542746	KCNQ1OT1
Rejected	30	1.40446120773181e-06	0.287711522188001	0.315	0.214	0.0471028199849094	MAPK14

Rejected	30	1.41535967195737e-06	0.320694499974675	0.373	0.258	0.0474683326781063	ARL8B
Rejected	30	1.4194563807423e-06	0.273817652099755	0.345	0.234	0.0476057280973353	TBC1D23
Rejected	30	1.42021229416534e-06	0.26416226679273	0.383	0.267	0.0476310799217171	BAZ2B
Rejected	30	1.45564907455506e-06	0.259744394925343	0.652	0.514	0.0488195586624277	DUSP16
Rejected	30	1.47698140870878e-06	0.341702560271873	0.307	0.199	0.049535002485275	GTDC1
Rejected	34	5.618279207799e-39	0.563809128285147	0.564	0.12	1.88425848071163e-34	NRG1
Rejected	34	7.57262647989008e-27	1.15477018533112	0.94	0.698	2.53970746882553e-22	IKZF1
Rejected	34	2.00984411988675e-26	0.861098984564714	0.718	0.259	6.74061520927617e-22	HDAC4
Rejected	34	2.45673507271931e-24	1.35123683237875	0.949	0.711	8.23939808688601e-20	INPP4B
Rejected	34	8.91780853123347e-24	1.1277705859997	0.658	0.249	2.99085462520508e-19	UTY
Rejected	34	1.29346373458073e-23	1.19213971890277	0.863	0.502	4.33801867303687e-19	RUNX1
Rejected	34	5.24060702997757e-23	0.475674050581695	0.308	0.062	1.75759478571388e-18	AC124014.1
Rejected	34	8.19340782233454e-23	0.329380666502894	0.274	0.05	2.74790511545456e-18	PRR16
Rejected	34	4.58529280783057e-22	0.882912327376276	0.974	0.941	1.53781550189022e-17	ARHGAP15
Rejected	34	1.36663204520836e-21	0.712728060861686	0.453	0.133	4.58341055321981e-17	KCNQ1
Rejected	34	2.94468553823233e-21	0.890160905716219	0.632	0.236	9.8758863581236e-17	DDX3Y
Rejected	34	3.59037237357385e-21	0.907392354347007	0.846	0.442	1.2041390866492e-16	PATJ
Rejected	34	3.81165311657764e-21	0.441799030698698	0.231	0.04	1.27835222223781e-16	AL023584.2
Rejected	34	4.86563918117608e-21	0.969129476306802	0.974	0.805	1.63183806858283e-16	FKBP5
Rejected	34	5.70922610118304e-21	0.95471301194239	0.453	0.138	1.91476024981477e-16	DOK6
Rejected	34	4.38235412475068e-20	1.1470264396059	0.795	0.48	1.46975392635888e-15	ETV6
Rejected	34	4.64235299396493e-20	0.888362063287712	0.957	0.674	1.55695234711596e-15	ELMO1
Rejected	34	5.27397534202498e-20	1.06149217380963	0.846	0.493	1.76878585020834e-15	MAP3K5
Rejected	34	5.8731141525123e-20	1.28116373535615	0.573	0.229	1.96972502446958e-15	PELI2
Rejected	34	6.24345432756622e-20	0.661321936099329	0.462	0.14	2.09392971237916e-15	USP9Y
Rejected	34	3.47626054250579e-19	1.01037819014494	0.923	0.659	1.16586826074559e-14	RIPOR2
Rejected	34	1.21147272732381e-18	0.77667030312414	0.427	0.136	4.06303723289859e-14	ZNRF1
Rejected	34	1.40160068150251e-18	0.759985490132398	0.974	0.842	4.70068836562313e-14	ANKRD44
Rejected	34	4.3732088674794e-18	0.701591497151946	0.735	0.32	1.46668678997524e-13	PREX1
Rejected	34	4.95472855159484e-18	0.833211230613041	0.658	0.283	1.66171686163388e-13	CRTC3
Rejected	34	5.07436899359426e-18	0.852762480340083	0.94	0.688	1.70184187307164e-13	CCND3

Rejected	34	5.75160604183169e-18	0.670669854263191	0.957	0.738	1.92897363430951e-13	LRBA
Rejected	34	8.69091545992765e-18	0.346526048382726	0.316	0.077	2.91475922695054e-13	ADGRL2
Rejected	34	9.01031413256617e-18	1.0819279125237	0.906	0.808	3.02187915378004e-13	PDE3B
Rejected	34	5.01610136184395e-17	0.519107881268436	0.504	0.174	1.68230007473522e-12	GOLGA8A
Rejected	34	5.36418390394165e-17	0.787450938710488	0.769	0.419	1.79903999770395e-12	GLG1
Rejected	34	6.15423460008432e-17	0.53256431761365	0.359	0.104	2.06400720017628e-12	STX2
Rejected	34	1.31244833919626e-16	0.911025602052436	0.607	0.269	4.40168923999643e-12	UBASH3B
Rejected	34	1.31720849045083e-16	0.556136179800774	0.496	0.175	4.417653835274e-12	NOL10
Rejected	34	1.34667697301311e-16	1.08514521171599	0.632	0.305	4.51648523209137e-12	LEF1
Rejected	34	1.55268588992972e-16	0.646599086634597	0.667	0.292	5.20739793764631e-12	GIGYF2
Rejected	34	2.90253058336559e-16	0.51836044349231	0.462	0.154	9.73450707049153e-12	VAV1
Rejected	34	3.22145769062566e-16	0.739032654322189	0.701	0.318	1.08041248028203e-11	UBR3
Rejected	34	6.19471740837778e-16	0.786170275272461	0.838	0.501	2.07758432442174e-11	SUCLG2
Rejected	34	9.09011156052237e-16	0.652276170061874	0.778	0.381	3.04864161516799e-11	STK39
Rejected	34	1.09034785581416e-15	0.753281180008503	0.829	0.475	3.65680863882952e-11	PLCL2
Rejected	34	1.27779373836107e-15	0.724665179290886	0.803	0.426	4.28546463971535e-11	PCGF5
Rejected	34	1.37125867894234e-15	0.78243329174995	0.393	0.126	4.59892735743682e-11	ST8SIA1
Rejected	34	1.44203678437212e-15	0.639585329835011	0.444	0.16	4.83630296742721e-11	MTMR1
Rejected	34	2.35340388810194e-15	0.621180863848219	0.496	0.188	7.89284595991628e-11	PIK3CD
Rejected	34	3.31039035875963e-15	0.609727433295285	0.607	0.258	1.11023871852081e-10	HMBOX1
Rejected	34	3.86737084656484e-15	0.592792922801782	0.692	0.315	1.29703883452092e-10	USP24
Rejected	34	3.95476005813916e-15	1.10038069201898	0.863	0.609	1.32634742829871e-10	LDLRAD4
Rejected	34	5.23882256985486e-15	0.765222098274017	0.915	0.679	1.75699631347792e-10	HIVEP2
Rejected	34	5.3825670499186e-15	0.444395007490263	0.538	0.203	1.8052053372017e-10	ACSL4
Rejected	34	5.73160133176808e-15	0.956379822018149	0.889	0.581	1.92226445464838e-10	CASK
Rejected	34	6.98764343196215e-15	0.803116225725629	0.65	0.329	2.34351585421147e-10	SLC16A7
Rejected	34	7.29576781461633e-15	0.723304309723172	0.829	0.543	2.44685460966603e-10	DOCK2
Rejected	34	7.96532986790846e-15	0.783190137895932	0.889	0.596	2.67141233109914e-10	RPS6KA3
Rejected	34	1.11158403235448e-14	0.585718907918531	0.368	0.119	3.72803052771046e-10	TG
Rejected	34	1.24366098021753e-14	0.7744102958825	0.974	0.829	4.17099019545355e-10	JMJD1C
Rejected	34	1.24411252268464e-14	0.63338165401905	0.726	0.349	4.17250457857976e-10	PRRC2B

Rejected	34	1.80899180504686e-14	0.700267892655499	0.966	0.853	6.06699671576618e-10	ZBTB20
Rejected	34	2.09380137878591e-14	1.04290097275539	0.829	0.538	7.02219106417218e-10	CAMK1D
Rejected	34	2.13902979547221e-14	0.680657714830987	0.65	0.299	7.17387812805471e-10	SCFD2
Rejected	34	2.5644744993881e-14	0.832849700102331	0.692	0.353	8.60073457604781e-10	LMBR1
Rejected	34	2.62058112078641e-14	0.683333999049832	0.906	0.614	8.78890496289345e-10	FBXW7
Rejected	34	2.70347048999885e-14	0.754310189074494	0.915	0.678	9.06689932935815e-10	RASA2
Rejected	34	2.8649226706612e-14	0.292459279852621	0.214	0.048	9.60837765286354e-10	AL024508.1
Rejected	34	3.008279201802e-14	0.747676754541242	0.838	0.487	1.00891667870035e-09	NCK2
Rejected	34	3.28128930900406e-14	0.781896876917966	0.915	0.637	1.10047880845378e-09	FOXO1
Rejected	34	3.95729434750202e-14	0.921430042710414	0.906	0.717	1.32719737826523e-09	MAML2
Rejected	34	4.72015517905124e-14	0.55912470152941	0.598	0.258	1.58304564395021e-09	ANKRD36
Rejected	34	5.7002258568075e-14	0.918093666136815	0.829	0.571	1.9117417478561e-09	PRKCA
Rejected	34	5.87381561046329e-14	0.469962023392541	0.513	0.2	1.96996027943718e-09	EP400
Rejected	34	7.40287083156871e-14	0.443505088418104	0.453	0.162	2.48277481949151e-09	INKA2
Rejected	34	7.60497288611862e-14	0.607007677519916	0.607	0.271	2.55055580654646e-09	ABHD15-AS1
Rejected	34	7.86292218901668e-14	0.850614097419062	0.701	0.387	2.63706684375241e-09	DOCK9
Rejected	34	9.27853243230958e-14	0.753893139997409	0.889	0.633	3.11183420714799e-09	CHST11
Rejected	34	9.66484407138722e-14	0.571927788217782	0.436	0.165	3.24139540466185e-09	IARS
Rejected	34	1.10867245788228e-13	0.252374342970887	0.145	0.025	3.71826568924558e-09	AC022146.2
Rejected	34	1.19981036615496e-13	0.951365350043558	0.829	0.614	4.02392400601050e-09	THEMIS
Rejected	34	1.27429268841785e-13	0.848402781409305	0.462	0.191	4.27372281841580e-09	SVIL
Rejected	34	1.62982779741613e-13	0.377828931870395	0.316	0.096	5.46611646697421e-09	DENND6A-AS1
Rejected	34	1.63857485189024e-13	0.493532245645864	0.393	0.137	5.49545233826949e-09	SH3BP5
Rejected	34	1.85647168576996e-13	0.638041314191853	0.692	0.345	6.22623473973528e-09	CARD11
Rejected	34	1.86871069645729e-13	0.784877784192165	0.872	0.638	6.26728193377846e-09	LPP
Rejected	34	1.92289199270075e-13	0.6829885292361	0.726	0.369	6.44899516511977e-09	ITPKB
Rejected	34	2.04806255837711e-13	0.839051386714219	0.897	0.597	6.86879220828515e-09	TMEM131L
Rejected	34	2.11912371691375e-13	0.596483931678669	0.692	0.32	7.10711712178532e-09	USP3-AS1
Rejected	34	2.20163425619647e-13	0.771466595161748	0.436	0.163	7.38384096843171e-09	FAM19A2
Rejected	34	2.21263422911858e-13	0.955720058911226	0.359	0.125	7.42073267761788e-09	AC139720.1
Rejected	34	2.4600581875779e-13	0.377634406640219	0.162	0.032	8.25054314949876e-09	HDGFL3

Rejected	34	2.52839516281588e-13	0.774378446646786	0.632	0.307	8.47973169705191e-09	RASA3
Rejected	34	2.56665635189268e-13	0.307040083416476	0.239	0.061	8.60805207297766e-09	TTTY15
Rejected	34	2.87889759285663e-13	0.615384424219255	0.658	0.318	9.65524674692258e-09	XPO6
Rejected	34	3.77603750860839e-13	0.667351418219762	0.932	0.679	1.26640745963708e-08	PCNX1
Rejected	34	4.08555972937464e-13	0.64102138208966	0.684	0.355	1.37021502203767e-08	WWP1
Rejected	34	4.10186569434559e-13	0.425283214101357	0.453	0.171	1.37568371656963e-08	MUC20-OT1
Rejected	34	4.36235423813441e-13	0.591308844762251	0.94	0.666	1.46304636438552e-08	DYRK1A
Rejected	34	5.89435620108865e-13	0.682260087719668	0.718	0.403	1.97684918272111e-08	ITFG1
Rejected	34	6.0134796756033e-13	0.659583056183598	0.889	0.634	2.01680081360383e-08	MACF1
Rejected	34	6.70249107964986e-13	0.740393146178123	0.701	0.369	2.24788145829297e-08	FLI1
Rejected	34	6.84377536136371e-13	0.748589569559072	0.829	0.577	2.29526538069416e-08	UBAC2
Rejected	34	7.68497750672065e-13	0.655939819946615	0.94	0.779	2.57738775620397e-08	EML4
Rejected	34	8.46044480182601e-13	0.628763052067665	0.632	0.309	2.83746397763641e-08	PIK3R5
Rejected	34	1.03373053095116e-12	0.624829103597151	0.821	0.477	3.46692545470401e-08	ITSN2
Rejected	34	1.05422155548715e-12	0.737388422949008	0.615	0.298	3.5356482527928e-08	GRK5
Rejected	34	1.05816315420095e-12	0.505606748595721	0.547	0.245	3.54886758655914e-08	TAF4B
Rejected	34	1.20300339160005e-12	1.14267446894959	0.41	0.169	4.03463277474825e-08	CSGALNACT1
Rejected	34	1.28898110003994e-12	0.699863313486099	0.564	0.268	4.32298481331394e-08	PTK2B
Rejected	34	1.29016194121356e-12	0.481983933440521	0.53	0.219	4.32694511844202e-08	RYK
Rejected	34	1.32513216929772e-12	0.607895807802814	0.838	0.48	4.44422826939068e-08	ABCC1
Rejected	34	1.35031434958561e-12	0.596877617533124	0.923	0.63	4.52868426564021e-08	VPS13B
Rejected	34	1.44722482061613e-12	0.563812882053131	0.957	0.759	4.85370260338239e-08	UTRN
Rejected	34	1.69300074531761e-12	0.839614357632207	0.821	0.527	5.67798589964619e-08	ATP11B
Rejected	34	1.74981312078033e-12	0.63312772498135	0.419	0.161	5.86852324447308e-08	LPCAT3
Rejected	34	1.83805655717656e-12	0.43550751116948	0.632	0.283	6.16447408145876e-08	SLFN12L
Rejected	34	1.91992941719938e-12	0.434038382468982	0.265	0.077	6.43905927940327e-08	C1S
Rejected	34	2.28567778241113e-12	0.421029372395752	0.402	0.147	7.66570614665044e-08	AKAP7
Rejected	34	2.33670823703447e-12	0.632107367511253	0.974	0.805	7.83685208536621e-08	TNRC6B
Rejected	34	2.48438365205454e-12	0.545278844786802	0.479	0.203	8.33212589226053e-08	SEL1L3
Rejected	34	2.75364367840128e-12	0.614778759082053	0.923	0.695	9.23517016862221e-08	STAG1
Rejected	34	2.90453094629917e-12	0.516349017507666	0.393	0.146	9.74121588769815e-08	ABCB7

Rejected	34	2.96298813885764e-12	0.59886430773333	0.769	0.383	9.93726962010075e-08	AC022217.3
Rejected	34	3.27699460380366e-12	0.504037909113749	0.744	0.391	1.09903845022367e-07	STK10
Rejected	34	3.33885342515418e-12	0.624622058540873	0.684	0.37	1.11978466172821e-07	INPP5D
Rejected	34	3.6916316987587e-12	0.636216468008631	0.915	0.708	1.23809943912969e-07	DOCK10
Rejected	34	3.97328138632921e-12	0.781546060906705	0.915	0.761	1.33255911134709e-07	PPP1CB
Rejected	34	4.07456320234939e-12	0.395699971935953	0.385	0.14	1.36652700680394e-07	IQSEC1
Rejected	34	4.25624958801461e-12	0.602560974395126	0.47	0.199	1.42746098682834e-07	N4BP2
Rejected	34	4.34853083344886e-12	0.676217293635416	0.906	0.64	1.45841027092208e-07	WNK1
Rejected	34	4.61686538722468e-12	0.672923512265275	0.641	0.313	1.54840431356741e-07	ST6GAL1
Rejected	34	4.66234303012975e-12	0.840981837991658	0.427	0.166	1.56365660544492e-07	PTPRM
Rejected	34	4.96375726048125e-12	0.30862526472079	0.308	0.097	1.6647449100202e-07	TOM1L2
Rejected	34	4.97121255563099e-12	0.633516262468868	0.821	0.497	1.66724526690752e-07	SMARCA2
Rejected	34	5.04689435662457e-12	0.661273311436745	0.667	0.339	1.69262742932475e-07	MAN1A2
Rejected	34	5.10000352171544e-12	0.264870997470538	0.248	0.07	1.71043918111292e-07	PLCG1
Rejected	34	5.2107986480433e-12	0.341599034224408	0.325	0.107	1.74759765058076e-07	SBNO2
Rejected	34	5.21686812558003e-12	0.464561248894417	0.684	0.336	1.74963323195703e-07	NBAS
Rejected	34	5.37309417045085e-12	0.670461184488303	0.923	0.597	1.80202832288581e-07	USP3
Rejected	34	5.49025910337144e-12	0.616958448348601	0.641	0.335	1.84132309808871e-07	FTO
Rejected	34	5.61306679199061e-12	0.400774706927063	0.239	0.067	1.88251034069781e-07	SDCBP2-AS1
Rejected	34	6.25386167536038e-12	0.37680011610038	0.333	0.111	2.09742012868236e-07	THOC3
Rejected	34	6.83463666887057e-12	1.00626647115335	0.821	0.596	2.29220044600581e-07	ARID5B
Rejected	34	6.97565931963272e-12	0.808309725349788	0.513	0.233	2.33949662261842e-07	RNF130
Rejected	34	7.52239017701959e-12	0.440594261702523	0.41	0.157	2.52285921756883e-07	BICD1
Rejected	34	7.72908085295655e-12	0.435898618312249	0.427	0.165	2.59217913646457e-07	HIST1H1D
Rejected	34	7.73627199066286e-12	0.331819919270542	0.35	0.119	2.59459090022851e-07	PARP9
Rejected	34	8.78448322788635e-12	0.498181106201152	0.436	0.176	2.94613998496852e-07	NUDCD3
Rejected	34	9.36637492742869e-12	0.590551228046958	0.932	0.738	3.14129482316103e-07	ARID1B
Rejected	34	9.85259517947178e-12	0.377194504880432	0.308	0.099	3.30436337129124e-07	ZNF337
Rejected	34	1.01943145295111e-11	0.498219595646294	0.675	0.338	3.41896920690742e-07	NDEL1
Rejected	34	1.08199958274254e-11	0.774531835034241	0.145	0.03	3.62881020060193e-07	GREM2
Rejected	34	1.09326626861058e-11	0.692319587198475	0.547	0.257	3.66659641166615e-07	RASSF3

Rejected	34	1.14021264843185e-11	0.535046767889007	0.504	0.223	3.82404518031073e-07	RHBDD1
Rejected	34	1.16873334618596e-11	0.653311039635601	0.419	0.168	3.91969789643847e-07	FAM168A
Rejected	34	1.2220857434669e-11	0.358557635365124	0.47	0.188	4.09863116643928e-07	POM121
Rejected	34	1.27191059769683e-11	0.646400342646629	0.744	0.441	4.26573376255564e-07	RNF38
Rejected	34	1.27847181122407e-11	0.776981149741422	0.667	0.35	4.2877387604833e-07	MAP3K1
Rejected	34	1.28516365987481e-11	0.666322490112932	0.573	0.26	4.31018188248813e-07	CD226
Rejected	34	1.45911036995315e-11	0.493522494279561	0.521	0.233	4.89356435874886e-07	MGA
Rejected	34	1.76017121975222e-11	0.789792858807292	0.436	0.182	5.903262236805e-07	NFIA
Rejected	34	1.77833900534159e-11	0.347187891034697	0.282	0.087	5.96419335611463e-07	TNFRSF10A
Rejected	34	1.93216440657200e-11	1.07617407955011	0.88	0.688	6.48009298676119e-07	ANK3
Rejected	34	2.00184734190983e-11	0.562538461779702	0.675	0.353	6.71379561529718e-07	TNRC6C
Rejected	34	2.2655869695019e-11	0.676651429086499	0.923	0.715	7.59832557831548e-07	SYNE2
Rejected	34	2.45684738335323e-11	0.584947997082502	0.274	0.088	8.23977475429005e-07	AC008937.2
Rejected	34	2.78541008106576e-11	0.267911507326826	0.274	0.084	9.34170832987836e-07	TRMT2B
Rejected	34	3.10158356113613e-11	0.510127689439667	0.462	0.197	1.04020909473383e-06	LMTK2
Rejected	34	3.11093970087765e-11	0.512438623847083	0.624	0.315	1.04334695688034e-06	ERCC1
Rejected	34	3.18921893657313e-11	0.552373899760017	0.402	0.161	1.0696002469479e-06	RNF157
Rejected	34	3.52368580689832e-11	0.592942667254842	0.65	0.34	1.18177374591756e-06	TXK
Rejected	34	3.71707799792654e-11	0.778127879101313	0.581	0.295	1.2466336189446e-06	KAT2B
Rejected	34	3.76751667843812e-11	0.5376858383503	0.607	0.306	1.26354974361458e-06	AC118549.1
Rejected	34	3.9220116025059e-11	0.290494886601553	0.291	0.094	1.31536425124843e-06	AC009093.2
Rejected	34	4.01459296123759e-11	0.365821392792845	0.462	0.189	1.34641418733986e-06	AGPAT3
Rejected	34	4.10170600686381e-11	0.544010180991636	0.915	0.617	1.37563016058199e-06	ASH1L
Rejected	34	4.15937169723098e-11	0.563670735324152	0.556	0.262	1.39497007981733e-06	ATF7IP2
Rejected	34	4.29116173059031e-11	0.537850938004884	0.658	0.331	1.43916982120538e-06	LARP4B
Rejected	34	4.34395759467341e-11	0.701865050701754	0.479	0.219	1.45687649810157e-06	GMDS-DT
Rejected	34	4.35999334734179e-11	0.686491160188741	0.863	0.608	1.46225456883149e-06	TCF12
Rejected	34	4.45214292300702e-11	0.406717338803277	0.308	0.104	1.49315969351809e-06	ZCCHC14
Rejected	34	5.08234614952942e-11	0.761402378336232	0.735	0.429	1.70451725162918e-06	STIM1
Rejected	34	5.54695880925005e-11	0.552351026452379	0.53	0.251	1.86033904544628e-06	ITGA6
Rejected	34	6.12175738001273e-11	0.491862278578368	0.632	0.318	2.05311499010867e-06	SP110

Rejected	34	6.13980759788915e-11	0.268164130217153	0.179	0.044	2.05916867218006e-06	ZNF445
Rejected	34	6.36678619504906e-11	0.534058715522485	0.598	0.288	2.13529275409555e-06	PAM
Rejected	34	6.50209655669422e-11	0.505472507604964	0.709	0.378	2.18067314318411e-06	GATAD2B
Rejected	34	6.68551001215954e-11	0.515071153454686	0.333	0.121	2.24218634787807e-06	EVA1C
Rejected	34	7.00589133034354e-11	0.366633688429129	0.59	0.266	2.34963583437062e-06	DENND6A
Rejected	34	7.16253281150604e-11	0.546323061586788	0.658	0.342	2.4021702543229e-06	PIBF1
Rejected	34	7.19082068780308e-11	0.501487212246067	0.59	0.287	2.4116574422754e-06	POU2F1
Rejected	34	7.29073718270937e-11	0.397031598202892	0.436	0.177	2.44516743633707e-06	CLIP4
Rejected	34	7.43543060619547e-11	0.561889144484621	0.88	0.586	2.49369471670584e-06	TBC1D5
Rejected	34	7.44982868719425e-11	0.478070327130759	0.641	0.323	2.49852354511121e-06	SLC25A26
Rejected	34	8.08189836757864e-11	0.599204948977081	0.906	0.638	2.71050707451852e-06	KMT2C
Rejected	34	8.13240206222211e-11	0.535454203349742	0.692	0.406	2.72744500362805e-06	CCDC88C
Rejected	34	8.41800215206572e-11	0.67665418434462	0.838	0.534	2.8232295617598e-06	COP1
Rejected	34	9.06192090017471e-11	0.582511895407245	0.957	0.84	3.03918703150059e-06	CELF2
Rejected	34	9.17572473005019e-11	0.528600417001862	0.427	0.177	3.07735455996423e-06	SLAMF6
Rejected	34	9.51535737775166e-11	0.359148723176476	0.376	0.142	3.19126055735035e-06	MAN1C1
Rejected	34	1.01278784956308e-10	0.408743173775869	0.393	0.156	3.39668788986467e-06	SERGEF
Rejected	34	1.03646377917517e-10	0.456530130022966	0.752	0.405	3.4760922225977e-06	PHC3
Rejected	34	1.04790613882113e-10	0.318769853666665	0.248	0.074	3.51446760837829e-06	SLC16A1-AS1
Rejected	34	1.09154051411833e-10	0.331881508358245	0.419	0.163	3.66080857625004e-06	GNL1
Rejected	34	1.09930180095437e-10	0.442401045785039	0.427	0.177	3.68683838004077e-06	DCAF1
Rejected	34	1.13415920512245e-10	0.511917651822766	0.444	0.195	3.80374314213969e-06	AVL9
Rejected	34	1.15646744830984e-10	0.395759932761359	0.496	0.216	3.87856052814154e-06	MAPK14
Rejected	34	1.24916100023779e-10	0.611217463497781	0.701	0.396	4.1894361625975e-06	TTC17
Rejected	34	1.2505037452566e-10	0.466955236231235	0.41	0.168	4.19393946084158e-06	FAM196B
Rejected	34	1.26447360293838e-10	0.319966159750836	0.385	0.148	4.24079156953475e-06	PXN
Rejected	34	1.29414394135884e-10	0.355846774455899	0.316	0.11	4.34029995052927e-06	TMEM63A
Rejected	34	1.34515343939253e-10	0.509409213148262	0.872	0.559	4.51137560503465e-06	PUM2
Rejected	34	1.3543093337885e-10	0.446357159056991	0.427	0.17	4.54208264365987e-06	EMSY
Rejected	34	1.4037724437433e-10	0.442369987300091	0.359	0.133	4.70797202182628e-06	REG3A
Rejected	34	1.44372949267311e-10	0.387242111275051	0.436	0.179	4.84197997252706e-06	PDK3

Rejected	34	1.45454906641789e-10	0.349523753922235	0.41	0.165	4.87826665895233e-06	TBC1D2B
Rejected	34	1.47512721033859e-10	0.259272785080045	0.179	0.045	4.94728163803357e-06	GRIK1
Rejected	34	1.48311102162803e-10	0.667381908757853	0.761	0.467	4.9740577443361e-06	ASAP1
Rejected	34	1.53807446486039e-10	0.265319920403725	0.171	0.042	5.15839414024876e-06	ZNF208
Rejected	34	1.55901346296464e-10	0.620499145760876	0.769	0.449	5.22861935209081e-06	SOS1
Rejected	34	1.60602934448053e-10	0.284743751419552	0.162	0.039	5.3863012155188e-06	RNF170
Rejected	34	1.73958935076137e-10	0.669849068605366	0.47	0.219	5.83423476458348e-06	TPK1
Rejected	34	1.86110912364608e-10	0.600508584832447	0.889	0.611	6.24178777888424e-06	AAK1
Rejected	34	2.0373994570272e-10	0.322701180654931	0.291	0.099	6.83303029897781e-06	ANAPC4
Rejected	34	2.106791086447e-10	0.538313824741765	0.744	0.441	7.06575594572595e-06	ZNF148
Rejected	34	2.25524014163672e-10	0.515847994767135	0.607	0.302	7.56362438702122e-06	KIAA2026
Rejected	34	2.26327314525893e-10	0.475163600084193	0.692	0.37	7.59056547456941e-06	RNF216
Rejected	34	2.27495366525323e-10	0.35869392451272	0.444	0.187	7.62973960252627e-06	ENOSF1
Rejected	34	2.30479998534879e-10	0.835685117262953	0.308	0.114	7.72983819086278e-06	SH3RF3
Rejected	34	2.33509515107114e-10	0.520314919237	0.547	0.267	7.8314421176624e-06	PDE8A
Rejected	34	2.39852518083405e-10	0.382898422366912	0.667	0.334	8.04417375148123e-06	GATAD2A
Rejected	34	2.59482822572599e-10	0.469218016956219	0.35	0.139	8.70253490343982e-06	ALPK1
Rejected	34	2.73871247596795e-10	0.393623863083235	0.342	0.13	9.1850939019013e-06	PIGL
Rejected	34	3.33990172938696e-10	0.645471735718341	0.444	0.206	1.1201362420018e-05	DTNBP1
Rejected	34	3.41118993119668e-10	0.36352963887609	0.564	0.258	1.14404487912474e-05	FAM120A
Rejected	34	3.56174713966694e-10	0.629459238958686	0.675	0.359	1.1945387557015e-05	TAOK1
Rejected	34	3.67971070495268e-10	0.373855749128414	0.368	0.142	1.23410137622703e-05	HERC3
Rejected	34	3.89941597468737e-10	0.432862524611791	0.376	0.15	1.30778612959065e-05	RIOK1
Rejected	34	3.96303634784049e-10	0.43330055093643	0.35	0.136	1.32912313033874e-05	CRACR2A
Rejected	34	4.12159877226944e-10	0.470410707361275	0.581	0.285	1.38230179624372e-05	XPO1
Rejected	34	4.2548020671466e-10	0.320511746913489	0.393	0.156	1.42697551727963e-05	PARP11
Rejected	34	4.33777549945327e-10	0.625132101457122	0.53	0.26	1.45480314700664e-05	TRERF1
Rejected	34	4.43549953115743e-10	0.307295055814443	0.325	0.118	1.48757783275958e-05	AL645568.1
Rejected	34	4.48689922573901e-10	0.612916176560268	0.923	0.736	1.50481626232835e-05	SSH2
Rejected	34	4.78413340404523e-10	0.615611811190973	0.906	0.726	1.60450266104869e-05	PRKCH
Rejected	34	4.8390625150313e-10	0.617301667957328	0.829	0.554	1.6229247862912e-05	GPRIN3

Rejected	34	4.93904068526265e-10	0.72308218120903	0.513	0.255	1.65645546502339e-05	DISC1
Rejected	34	5.09204400582439e-10	0.70065091304760	0.521	0.262	1.70776971867338e-05	ARHGAP25
Rejected	34	5.13041370490626e-10	0.70697578517247	0.65	0.385	1.72063814835146e-05	TSPAN5
Rejected	34	5.1632637143737e-10	0.40585298737777	0.368	0.142	1.73165538452665e-05	EPM2AIP1
Rejected	34	5.61769711737397e-10	0.61969603665879	0.949	0.844	1.88406325922488e-05	SKAP1
Rejected	34	5.96840566113279e-10	0.36211812901044	0.171	0.044	2.00168389063071e-05	TMLHE-AS1
Rejected	34	6.05094279847421e-10	0.71373390019578	0.564	0.294	2.02936519575228e-05	PRKCE
Rejected	34	6.19717672429687e-10	0.34057599012827	0.453	0.194	2.07840912979469e-05	VPS54
Rejected	34	6.20427162524629e-10	0.37355924830651	0.521	0.242	2.0807886176751e-05	FAM120B
Rejected	34	6.77963203425392e-10	0.33581625415725	0.282	0.097	2.27375299164808e-05	TARBP1
Rejected	34	7.04416062848066e-10	0.35343387749088	0.368	0.146	2.36247059157984e-05	EYA3
Rejected	34	7.10517478194017e-10	0.40282938619156	0.444	0.188	2.38293351836709e-05	ANKRD36C
Rejected	34	7.16248138922771e-10	0.27162553844947	0.179	0.047	2.40215300831919e-05	CC2D1B
Rejected	34	7.62923945374771e-10	0.52847485684296	0.513	0.244	2.55869432799791e-05	CPQ
Rejected	34	7.84553735336549e-10	0.65473281139857	0.889	0.666	2.63123631757172e-05	ZFAND3
Rejected	34	8.41894122402547e-10	0.51186136482777	0.803	0.492	2.82354450771366e-05	FNDC3A
Rejected	34	8.45557673638218e-10	0.27112239217933	0.316	0.114	2.83583132584786e-05	RAB11FIP2
Rejected	34	8.45955723278207e-10	0.33492915578514	0.205	0.059	2.83716630473045e-05	AC027018.1
Rejected	34	8.66668267578814e-10	0.30221821061214	0.385	0.152	2.90663203580583e-05	MLXIP
Rejected	34	9.08551630117686e-10	0.33174181254176	0.427	0.18	3.0471004570887e-05	TANGO6
Rejected	34	9.50009547183828e-10	0.46134693489810	0.615	0.322	3.18614201934512e-05	STRN3
Rejected	34	9.5675644179593e-10	0.42108182736321	0.556	0.264	3.20876975449519e-05	LONP2
Rejected	34	9.6478907668937e-10	0.43264062459730	0.718	0.392	3.23570960540081e-05	CELF1
Rejected	34	9.67354063350855e-10	0.27791053465791	0.162	0.041	3.2443120576661e-05	PER3
Rejected	34	9.69831165158374e-10	0.38814735536680	0.547	0.257	3.25261976170815e-05	RPTOR
Rejected	34	9.91195257220917e-10	0.34806309560508	0.274	0.095	3.32427065366751e-05	IPO11
Rejected	34	1.00170841551774e-09	0.31918012329438	0.53	0.245	3.35952968396341e-05	TMC8
Rejected	34	1.08505484847531e-09	0.46715944732417	0.385	0.163	3.63905695081651e-05	ABL2
Rejected	34	1.09598172004926e-09	0.35696231667356	0.385	0.158	3.67570349270121e-05	NUTM2A-AS1
Rejected	34	1.13959766687865e-09	0.59269551803117	0.769	0.468	3.82198265517761e-05	ZNF609
Rejected	34	1.14390837121989e-09	0.47167244557228	0.402	0.172	3.83643989539725e-05	AC079793.1

Rejected	34	1.17201804407294e-09	0.43286871137258	0.376	0.155	3.93071411621184e-05	AKAP11
Rejected	34	1.18584346408507e-09	0.34868838961137	0.453	0.196	3.97708180984851e-05	AC092652.2
Rejected	34	1.41224286372104e-09	0.64102393700075	0.521	0.257	4.73638011634763e-05	TMEM65
Rejected	34	1.46879766082687e-09	0.33276969054122	0.214	0.064	4.92605359488114e-05	LINC00278
Rejected	34	1.55604097179558e-09	0.46873849034067	0.692	0.401	5.218650211208e-05	RALY
Rejected	34	1.57448226425605e-09	0.31773650121699	0.316	0.118	5.28049861786194e-05	CYREN
Rejected	34	1.70004312684873e-09	0.74274210464934	0.803	0.585	5.70160463882528e-05	TNIK
Rejected	34	1.72148295019286e-09	0.54131575076592	0.709	0.405	5.77350951835681e-05	SCAPER
Rejected	34	1.72899837787724e-09	0.27082831985724	0.393	0.159	5.7987147597247e-05	NCOR2
Rejected	34	1.72908241898611e-09	0.70027792058943	0.769	0.532	5.79899661679562e-05	NCOA1
Rejected	34	1.75880976795042e-09	0.69737983664686	0.376	0.159	5.89869619975213e-05	TIAM1
Rejected	34	1.77991822041607e-09	0.43029072764999	0.513	0.237	5.96948972763142e-05	ZMYND11
Rejected	34	1.96909422287377e-09	0.57351136651324	0.368	0.156	6.60394820467404e-05	AC096558.2
Rejected	34	1.99076288408215e-09	0.34696686139362	0.333	0.126	6.67662056063471e-05	ETFRF1
Rejected	34	2.09352305080826e-09	0.28181333623340	0.188	0.053	7.02125760780073e-05	TSPAN18
Rejected	34	2.12347617098101e-09	0.35439478976772	0.256	0.087	7.1217143822361e-05	MLH3
Rejected	34	2.15133055887491e-09	0.53160083626431	0.658	0.352	7.21513242835467e-05	SORL1
Rejected	34	2.17239459659549e-09	0.58982145377284	0.735	0.448	7.28577699806195e-05	ARHGEF3
Rejected	34	2.17433013672225e-09	0.28024636235184	0.325	0.122	7.29226841253909e-05	UNK
Rejected	34	2.22310582730197e-09	0.39050566031794	0.402	0.168	7.45585232360536e-05	AC006059.1
Rejected	34	2.2387819442816e-09	0.26247178180990	0.342	0.128	7.50842688473163e-05	AC090772.2
Rejected	34	2.31882367502026e-09	0.48390909234112	0.735	0.427	7.77687084128296e-05	TBC1D22A
Rejected	34	2.33992638183614e-09	0.36158209675605	0.462	0.207	7.84764509940206e-05	FAR1
Rejected	34	2.3679392691383e-09	0.42888466449442	0.462	0.213	7.94159472083603e-05	ZNF654
Rejected	34	2.39651025505068e-09	0.44161101895598	0.538	0.269	8.03741609338897e-05	DIS3L2
Rejected	34	2.48264684926778e-09	0.38966548632856	0.368	0.151	8.32630100307427e-05	PRR14L
Rejected	34	2.55511302094816e-09	0.45808050756064	0.718	0.432	8.56933804965595e-05	SMARCC1
Rejected	34	2.63362657761078e-09	0.49278042234015	0.615	0.318	8.83265681599102e-05	CAB39
Rejected	34	2.65964462976899e-09	0.27825320880629	0.41	0.17	8.91991615931925e-05	DDX60L
Rejected	34	2.7020954581966e-09	0.60681410756625	0.786	0.542	9.06228774769976e-05	CAMK2D
Rejected	34	2.85252816907676e-09	0.27393129726620	0.137	0.032	9.56680897344963e-05	AC013437.1

Rejected	34	2.91322346470558e-09	0.25282282486539	0.282	0.1	9.77036885592959e-05	CKAP5
Rejected	34	3.2547364308177e-09	0.34241738585585	0.538	0.263	0.000109157350416764	XPO4
Rejected	34	3.27585256154023e-09	0.41474941021161	0.299	0.109	0.000109865543208936	MIR29B2CHG
Rejected	34	3.28811322078758e-09	0.55657806629282	0.504	0.249	0.000110276741198774	SMURF1
Rejected	34	3.39040951367375e-09	0.43556156946564	0.462	0.209	0.00011370755426959	FAM193A
Rejected	34	3.40348832646365e-09	0.52726877869234	0.709	0.437	0.000114146191492938	DYM
Rejected	34	3.45128424167364e-09	0.48786346253989	0.667	0.37	0.00011574917089725	FARS2
Rejected	34	3.48948980611686e-09	0.51410958145388	0.632	0.35	0.000117030509117547	THADA
Rejected	34	3.59197424825475e-09	0.58432839085252	0.615	0.34	0.000120467632337968	RAB3GAP1
Rejected	34	3.6238944723267e-09	0.39186622840239	0.47	0.224	0.000121538172812893	CCM2
Rejected	34	3.65848427972907e-09	0.52082174797329	0.812	0.536	0.000122698245773553	ANKRD17
Rejected	34	3.90229333663616e-09	0.52188525865425	0.906	0.611	0.000130875113924104	ANKRD11
Rejected	34	4.03874890097731e-09	0.40940830136440	0.47	0.222	0.000135451560640977	NLRC5
Rejected	34	4.08167671766721e-09	0.69071215366369	0.496	0.262	0.000136891273757123	DGKH
Rejected	34	4.16284947354079e-09	0.45483061421696	0.624	0.355	0.000139613645643611	CHCHD3
Rejected	34	4.17804640256165e-09	0.32392899396507	0.308	0.117	0.000140123320249113	CEPT1
Rejected	34	4.25072956278638e-09	0.53894977470777	0.752	0.474	0.00014256096807673	CAPZB
Rejected	34	4.28070761464393e-09	0.42009057310912	0.521	0.25	0.000143566371979928	ZBTB16
Rejected	34	4.35774501971887e-09	0.63445217610817	0.513	0.253	0.000146150052471332	A2M
Rejected	34	4.66974087822052e-09	0.58267750335089	0.419	0.184	0.00015661376957376	AP000787.1
Rejected	34	4.68490657569677e-09	0.45790597656431	0.598	0.302	0.000157122396735718	PLEKHA2
Rejected	34	4.75355925743170e-09	0.29087360435129	0.231	0.076	0.000159424870375744	TTN-AS1
Rejected	34	4.77441296954242e-09	0.36675010277166	0.316	0.124	0.000160124262172514	WRNIP1
Rejected	34	4.81950928872017e-09	0.43209455207286	0.821	0.508	0.000161636702525097	FRYL
Rejected	34	4.86052010390123e-09	0.42919532384653	0.709	0.396	0.000163012123244639	SETX
Rejected	34	4.86178259472519e-09	0.38882824668749	0.444	0.204	0.000163054464661893	ZBTB21
Rejected	34	4.89967723989578e-09	0.59624343673757	0.692	0.405	0.000164325375271625	RNGTT
Rejected	34	5.0422307320982e-09	0.28940310610162	0.308	0.115	0.000169106334293109	MTMR12
Rejected	34	5.22826621242908e-09	0.45216146987919	0.846	0.523	0.000175345592232446	KAT6B
Rejected	34	5.36424729191119e-09	0.59352004311412	0.803	0.571	0.000179906125676118	COG5
Rejected	34	5.48618405978054e-09	0.49546415576014	0.821	0.534	0.00018399564099692	CRYBG1

Rejected	34	5.54784961221258e-09	0.473097786559251	0.393	0.175	0.000186063780294386	ICE1
Rejected	34	5.72037225619701e-09	0.549787144762545	0.829	0.59	0.000191849844728335	PACS1
Rejected	34	5.73873534952655e-09	0.276883925893568	0.274	0.097	0.000192465706152421	LRIG2
Rejected	34	5.77492095096496e-09	0.340987568496133	0.385	0.162	0.000193679298853463	SHPRH
Rejected	34	5.84749555840927e-09	0.354176880105378	0.368	0.156	0.00019611330603793	SUCLA2
Rejected	34	5.88245478207535e-09	0.398839975235872	0.453	0.204	0.000197285768481243	DIDO1
Rejected	34	5.9131867856976e-09	0.329205332520743	0.47	0.215	0.000198316458418726	ARFGEF2
Rejected	34	5.93127187046282e-09	0.525119991572646	0.308	0.12	0.000198922995991582	AL035530.2
Rejected	34	5.98014238060685e-09	0.286018374594973	0.308	0.117	0.000200562015160792	AC010260.1
Rejected	34	6.02872739452027e-09	0.450037145547698	0.846	0.589	0.000202191459357421	CDKAL1
Rejected	34	6.29343459262455e-09	0.385049779539756	0.265	0.095	0.000211069209367442	LDAH
Rejected	34	6.30751911197304e-09	0.576173827053703	0.581	0.328	0.000211541575977352	UBE2G1
Rejected	34	6.44524577585001e-09	0.261694138225416	0.291	0.106	0.000216160652830458	STYX
Rejected	34	6.5184550784896e-09	0.335885145253537	0.47	0.214	0.000218615946422384	SFI1
Rejected	34	6.64577456823525e-09	0.661720559738385	0.538	0.284	0.000222885987469474	XKR6
Rejected	34	6.6603059914526e-09	0.624000574087404	0.872	0.687	0.000223373342341337	DPYD
Rejected	34	6.68242838615085e-09	0.263464012081837	0.581	0.277	0.000224115283214727	NRDC
Rejected	34	6.77788765396928e-09	0.474448328234781	0.487	0.246	0.000227316796138822	RSU1
Rejected	34	6.83708050420136e-09	0.425092703104407	0.291	0.109	0.000229302005949905	DPH6
Rejected	34	6.85850375147926e-09	0.37331254641341	0.521	0.24	0.000230020498817112	FAM208A
Rejected	34	7.0469947972376e-09	0.332332346903063	0.513	0.243	0.000236342111509755	ARMH3
Rejected	34	7.11796181924334e-09	0.329062044841034	0.239	0.081	0.000238722203493783	HACE1
Rejected	34	7.2349066779692e-09	0.497331577085694	0.65	0.362	0.000242644300165731	VWA8
Rejected	34	7.30682473913632e-09	0.461844653706775	0.624	0.338	0.000245056288101154	MYO5A
Rejected	34	7.40018483167473e-09	0.422253744098188	0.513	0.25	0.000248187398884707	SIDT1
Rejected	34	7.56283899831136e-09	0.583003183725231	0.829	0.622	0.000253642494325366	EXOC4
Rejected	34	7.6219243960857e-09	0.339459325601946	0.239	0.081	0.000255624100395922	MMS19
Rejected	34	7.80906148817042e-09	0.483941750831908	0.915	0.705	0.00026190030419026	PIP4K2A
Rejected	34	7.92443472994331e-09	0.310695137114921	0.282	0.103	0.000265769691972839	SNX19
Rejected	34	7.92594265428466e-09	0.43325660416725	0.615	0.326	0.000265820264739399	CUL1
Rejected	34	7.99364470399403e-09	0.282410336048187	0.145	0.037	0.000268090856082552	AC005154.6

Rejected	34	8.26015205353828e-09	0.45642359283966	0.778	0.468	0.000277028979571567	RBM6
Rejected	34	8.33902557038895e-09	0.512967383850089	0.889	0.645	0.000279674239579704	MYCBP2
Rejected	34	9.07895113805193e-09	0.526321454597882	0.487	0.241	0.000304489863267986	SRBD1
Rejected	34	9.130151206867e-09	0.259633409219098	0.291	0.108	0.000306207011175906	RAP1GAP2
Rejected	34	9.2569248762282e-09	0.564739076771287	0.855	0.632	0.000310458746498941	MAPRE2
Rejected	34	9.64142001984616e-09	0.447140854356929	0.197	0.061	0.0003233539446256	DOCK3
Rejected	34	9.68290751542642e-09	0.584266629141863	0.795	0.547	0.000324745352252371	MKLN1
Rejected	34	1.01559925417188e-08	0.709644221396016	0.479	0.251	0.000340611677864164	PRKN
Rejected	34	1.0170120378886e-08	0.548848665094961	0.863	0.639	0.000341085497267078	USP34
Rejected	34	1.02188103653279e-08	0.554579061967241	0.906	0.767	0.000342718462032368	KLF12
Rejected	34	1.02882449539508e-08	0.326993132522693	0.453	0.203	0.000345047159265603	TCF20
Rejected	34	1.03412081288638e-08	0.482208000822856	0.684	0.413	0.000346823438225834	ASCC3
Rejected	34	1.05450502731693e-08	0.391127386428663	0.59	0.308	0.000353659896061551	DENND4C
Rejected	34	1.05468220766925e-08	0.585272472504631	0.786	0.544	0.000353719318808113	CEP85L
Rejected	34	1.0565721063609e-08	0.516604970897619	0.368	0.158	0.000354353153031317	KCNQ1OT1
Rejected	34	1.15101825656538e-08	0.392944402219901	0.521	0.264	0.000386028502886898	DCAF6
Rejected	34	1.18057001021116e-08	0.460574348720524	0.641	0.36	0.000395939570024618	PVT1
Rejected	34	1.24855801608325e-08	0.279784966665889	0.299	0.114	0.000418741387434001	MCM3AP
Rejected	34	1.26344271004206e-08	0.369227670252245	0.547	0.279	0.000423733416093906	SENP5
Rejected	34	1.2945628272182e-08	0.321536015576064	0.385	0.165	0.000434170480992439	ZFYVE28
Rejected	34	1.30025270101663e-08	0.681750226225672	0.769	0.545	0.000436078750866956	TBL1XR1
Rejected	34	1.32835424962168e-08	0.37299465388173	0.47	0.218	0.000445503448238118	GAPVD1
Rejected	34	1.48563392959235e-08	0.335707927608056	0.248	0.088	0.000498251907306682	IDE
Rejected	34	1.49242703430499e-08	0.339032157501	0.299	0.117	0.000500530178765207	ELP3
Rejected	34	1.49774805233633e-08	0.276051253659755	0.188	0.056	0.000502314741792557	AC009126.1
Rejected	34	1.50367190755473e-08	0.298368145999876	0.274	0.101	0.000504301484355704	ATL3
Rejected	34	1.50915995143939e-08	0.512036927912806	0.453	0.222	0.000506142064513742	OPRM1
Rejected	34	1.54084687495294e-08	0.393497796567907	0.983	0.758	0.000516769224921718	GLS
Rejected	34	1.55105481258837e-08	0.292249821719148	0.436	0.198	0.000520192763045889	HPCAL1
Rejected	34	1.5608143762857e-08	0.500607961377797	0.726	0.442	0.000523465925518697	MSI2
Rejected	34	1.56879058795131e-08	0.354395399067297	0.675	0.367	0.000526140987387111	PRKDC

Rejected	34	1.58177477027602e-08	0.306806380858542	0.35	0.145	0.000530495622455171	NAA25
Rejected	34	1.61866953467984e-08	0.377697780763123	0.803	0.488	0.000542869388540926	NKTR
Rejected	34	1.64346130673873e-08	0.434178694416604	0.444	0.209	0.000551184053054036	HECTD4
Rejected	34	1.65419674672458e-08	0.310205901781209	0.359	0.149	0.000554784504916488	ABR
Rejected	34	1.65817399059635e-08	0.266589694874219	0.214	0.069	0.000556118392966204	DOCK7
Rejected	34	1.6786521145579e-08	0.395537094273693	0.718	0.425	0.00056298634618043	SMC5
Rejected	34	1.70600412680831e-08	0.445301408897252	0.393	0.175	0.00057215966404897	UBAP2L
Rejected	34	1.74221468750714e-08	0.479229268114979	0.598	0.312	0.000584303961896145	PCED1B
Rejected	34	1.7474224419404e-08	0.369809617461305	0.342	0.144	0.00058605053857797	HK1
Rejected	34	1.76884278122623e-08	0.311161906805942	0.53	0.26	0.000593234491967654	RAB3GAP2
Rejected	34	1.82659063896613e-08	0.379354656746624	0.197	0.063	0.000612601968496462	COL10A1
Rejected	34	1.83210588972809e-08	0.328169483237872	0.291	0.113	0.000614451673297006	TTN
Rejected	34	1.83903875959571e-08	0.304653613501328	0.35	0.146	0.000616776819193211	BANP
Rejected	34	1.90114262467335e-08	0.34053224206425	0.444	0.208	0.000637605213462947	AATF
Rejected	34	1.91962700616371e-08	0.303424193341898	0.376	0.161	0.000643804505327185	SNX14
Rejected	34	2.03790247757846e-08	0.253581117908223	0.162	0.045	0.000683471732930262	AC009690.1
Rejected	34	2.09940685322424e-08	0.432131317996681	0.726	0.424	0.000704099070434346	SMG1
Rejected	34	2.21387643485e-08	0.306881192993978	0.692	0.38	0.000742489878719994	BRWD1
Rejected	34	2.27548545999656e-08	0.382496283255477	0.248	0.088	0.000763152313573648	WBP1L
Rejected	34	2.29225451648262e-08	0.53816970822782	0.744	0.459	0.00076877631973794	OGT
Rejected	34	2.51939144985284e-08	0.463871030574155	0.538	0.29	0.000844953504451645	TBC1D1
Rejected	34	2.52105525106845e-08	0.403860118956147	0.675	0.387	0.000845511510103337	PPM1B
Rejected	34	2.58582159388976e-08	0.422518842279638	0.453	0.22	0.000867232846158747	GNB5
Rejected	34	2.63536084433295e-08	0.463774134199679	0.333	0.143	0.000883847319972384	RFX3-AS1
Rejected	34	2.76027728710035e-08	0.411415909940963	0.675	0.385	0.000925741796547717	DOCK11
Rejected	34	2.76724247043614e-08	0.580059114315719	0.829	0.584	0.000928077779734872	BTBD9
Rejected	34	2.85807522639722e-08	0.502763508900901	0.368	0.166	0.0009585412694291	ZBTB20-AS5
Rejected	34	2.86305473215259e-08	0.281478214868441	0.393	0.175	0.000960211296069337	IKBKB
Rejected	34	2.91246818784865e-08	0.558667305223135	0.803	0.568	0.000976783580840682	FAF1
Rejected	34	2.94005538359768e-08	0.555790668962433	0.393	0.183	0.000986035774550989	ATP10A
Rejected	34	2.98241437221483e-08	0.387238840208065	0.299	0.117	0.00100024213215341	AL359915.2

Rejected	34	3.02113876192716e-08	0.318384615502721	0.359	0.154	0.00101322951797513	HERC2
Rejected	34	3.09237879397642e-08	0.436470205317219	0.265	0.101	0.00103712199992381	GNA12
Rejected	34	3.09773527193816e-08	0.284316492326337	0.222	0.076	0.00103891845550262	AC139887.2
Rejected	34	3.12288741239136e-08	0.294339270412249	0.419	0.193	0.00104735398036782	KCNAB2
Rejected	34	3.1689497804938e-08	1.37787646284238	0.376	0.179	0.00106280237738201	TSHZ2
Rejected	34	3.17044601796408e-08	0.328002912532851	0.427	0.195	0.00106330418550479	AC073167.1
Rejected	34	3.2269576608807e-08	0.253772094297899	0.274	0.101	0.00108225706030617	PRKD2
Rejected	34	3.3516363516444e-08	0.320511548657968	0.573	0.286	0.0011240717996145	SPTBN1
Rejected	34	3.3613898362058e-08	0.310180461417341	0.368	0.159	0.0011273429232667	STAMBPL1
Rejected	34	3.51106276536134e-08	0.428543686957591	0.726	0.462	0.00117754023024689	DLG1
Rejected	34	3.55887910912028e-08	0.604171226736652	0.684	0.434	0.00119357687561676	EXOC6B
Rejected	34	3.58206982384768e-08	0.34418329539184	0.171	0.051	0.00120135457752203	EGLN3
Rejected	34	3.66256631974709e-08	0.291161612694118	0.41	0.183	0.00122835149231678	DGKZ
Rejected	34	3.79751679889748e-08	0.290208433402154	0.513	0.248	0.00127361118401424	PI4KA
Rejected	34	3.86991394942333e-08	0.382758048118117	0.385	0.174	0.0012978917403576	ANKS1A
Rejected	34	3.98583683554655e-08	0.42987907926183	0.513	0.266	0.0013367699579056	MAP2K1
Rejected	34	3.98875641010992e-08	0.41550424961413	0.607	0.323	0.00133774912482266	RBM26
Rejected	34	4.04148438893499e-08	0.323292283014408	0.419	0.192	0.00135543303436102	NUP210
Rejected	34	4.1984112566606e-08	0.274801697847522	0.325	0.132	0.00140806316725883	GPR107
Rejected	34	4.24055241929795e-08	0.416087660853489	0.538	0.276	0.00142219647038415	AHCTF1
Rejected	34	4.26234246180542e-08	0.416972575196645	0.299	0.123	0.0014295044148403	FBXL4
Rejected	34	4.3067310764704e-08	0.420667930323316	0.761	0.493	0.00144439146842664	EPC2
Rejected	34	4.34038745663657e-08	0.535729769353172	0.726	0.466	0.00145567914520677	KIAA1109
Rejected	34	4.39730106072834e-08	0.344863486443485	0.573	0.289	0.00147476682974707	EXOC2
Rejected	34	4.41568028589129e-08	0.292577196183785	0.333	0.137	0.00148093085428222	ZNF254
Rejected	34	4.46654195195729e-08	0.308965605949213	0.368	0.157	0.00149798883984744	SFMBT1
Rejected	34	4.58194021108571e-08	0.480211308353105	0.487	0.254	0.00153669110799393	UBE2E1
Rejected	34	4.60745249422216e-08	0.455299048496473	0.667	0.391	0.00154524741751223	ERC1
Rejected	34	4.62867857038269e-08	0.411452348128631	0.427	0.2	0.00155236621893495	PCSK7
Rejected	34	4.64751203237627e-08	0.380265428185119	0.581	0.317	0.00155868258541836	SEC24B
Rejected	34	4.77610288359391e-08	0.419618967335628	0.41	0.196	0.00160180938509973	SFXN1

Rejected	34	5.12240603690529e-08	0.374440173525083	0.581	0.303	0.0017179525366573	ZFC3H1
Rejected	34	5.4373124404184e-08	0.489848542919812	0.709	0.458	0.00182356584626752	TRAPPC9
Rejected	34	5.70890487368856e-08	0.267714889612427	0.291	0.113	0.00191465251653767	CEP290
Rejected	34	5.79256548469639e-08	0.453291606874796	0.556	0.312	0.00194271061225748	MAP2K4
Rejected	34	5.8097426890842e-08	0.336817851172673	0.427	0.202	0.00194847150306506	GPATCH2
Rejected	34	6.00489657859975e-08	0.476362751819597	0.521	0.282	0.00201392221453078	CYFIP2
Rejected	34	6.01887920892853e-08	0.437963806812866	0.385	0.179	0.00201861170909045	TTC37
Rejected	34	6.04230888820986e-08	0.573978368594022	0.479	0.254	0.00202646955492782	TPD52
Rejected	34	6.37399663128267e-08	0.327493007076505	0.624	0.329	0.00213771099019958	CMIP
Rejected	34	6.38755845453174e-08	0.387339183205344	0.581	0.31	0.00214225935448085	FBXW11
Rejected	34	6.48740075760264e-08	0.423009599533712	0.419	0.201	0.00217574446608477	ATP6V1H
Rejected	34	6.57673949874516e-08	0.47884417203507	0.795	0.524	0.00220570689308915	RPS6KA5
Rejected	34	6.63671446163649e-08	0.266497545782489	0.179	0.055	0.00222582129614364	GPR82
Rejected	34	6.83242295710172e-08	0.256773925278201	0.145	0.04	0.00229145801135277	LINC00243
Rejected	34	6.93261125592678e-08	0.50250245027958	0.376	0.174	0.00232505916301272	MSRA
Rejected	34	7.42785810433439e-08	0.4284417477832	0.342	0.152	0.00249115505103167	BIN3
Rejected	34	7.78780907444592e-08	0.42218819292397	0.906	0.672	0.00261187540738767	USP15
Rejected	34	7.95725771501894e-08	0.529032848857783	0.598	0.339	0.00266870509246305	VPS13D
Rejected	34	8.2932560631909e-08	0.457046799952055	0.692	0.43	0.00278139221847296	CLASP1
Rejected	34	8.37926967402296e-08	0.500539310399212	0.966	0.794	0.00281023946327382	SMAP2
Rejected	34	8.41162905555952e-08	0.506315848622057	0.855	0.589	0.00282109215265355	ZNF644
Rejected	34	8.42562661152153e-08	0.893050017239026	0.684	0.455	0.00282578665297209	MAN2A1
Rejected	34	8.45556344935547e-08	0.312504608165391	0.385	0.169	0.00283582686964484	NSF
Rejected	34	8.61242573017288e-08	0.347280425103746	0.47	0.229	0.00288843534138538	CCDC6
Rejected	34	8.65892907299094e-08	0.255486939931183	0.573	0.29	0.0029040316324997	MTFR1
Rejected	34	9.00810864492986e-08	0.63631951469794	0.393	0.199	0.00302113947733658	FAM117B
Rejected	34	9.18224829211731e-08	0.368411514064362	0.308	0.126	0.0030795424322103	SUGP2
Rejected	34	9.39760480834337e-08	0.302609474390398	0.154	0.045	0.0031517687006222	UBA6-AS1
Rejected	34	9.8247601312493e-08	0.592917686542704	0.598	0.355	0.00329502805281839	TAF3
Rejected	34	9.93975882337105e-08	0.389375135226055	0.607	0.331	0.00333359631418218	AL031772.1
Rejected	34	1.03502321041269e-07	0.385387942691295	0.402	0.189	0.00347126084308207	APP

Rejected	34	1.04367252320913e-07	0.5457136557349755	0.564	0.33	0.00350026890833877	ST3GAL1
Rejected	34	1.06148517632376e-07	0.3349030498591911	0.991	0.977	0.00356000898435462	MBNL1
Rejected	34	1.10889579878493e-07	0.3944119123274188	0.47	0.236	0.00371901472996489	EXOC6
Rejected	34	1.11337241418784e-07	0.3349885675957222	0.829	0.522	0.00373402840270318	BAZ1A
Rejected	34	1.11488813616187e-07	0.4968530057378799	0.778	0.497	0.00373911183105969	TAOK3
Rejected	34	1.12415069829306e-07	0.2718760209115911	0.231	0.083	0.00377017661193525	STRADB
Rejected	34	1.12752795129314e-07	0.3688830503003844	0.735	0.433	0.00378150324304695	PSPC1
Rejected	34	1.13261451867799e-07	0.2908616636407466	0.103	0.023	0.00379856257274224	PAK3
Rejected	34	1.1550484788149e-07	0.4016885701096511	0.393	0.184	0.00387380158824943	KIAA0586
Rejected	34	1.15666072969815e-07	0.5161791300066822	0.325	0.139	0.00387920875526166	ZNF236
Rejected	34	1.20278376460297e-07	0.5855758008647411	0.521	0.289	0.00403389618972542	CBFA2T2
Rejected	34	1.20676304013451e-07	0.2570302789815633	0.231	0.085	0.00404724188400312	DELE1
Rejected	34	1.2264671733792e-07	0.4757440893861288	0.846	0.612	0.00411332560607915	FAM172A
Rejected	34	1.2401378332652e-07	0.4191690049497411	0.547	0.296	0.00415917426520482	COL4A3BP
Rejected	34	1.26571838016517e-07	0.2631966300081333	0.35	0.152	0.00424496630339794	SETD3
Rejected	34	1.28829372384205e-07	0.6241085139792677	0.547	0.315	0.00432067949102148	RASGRF2
Rejected	34	1.32421959814474e-07	0.4451108276284344	0.436	0.21	0.00444116768825782	DPYD-AS1
Rejected	34	1.35468384848613e-07	0.3024304458991099	0.436	0.212	0.00454333869105278	PHF11
Rejected	34	1.39024273044505e-07	0.4374238463909355	0.299	0.129	0.00466259606936661	NSD2
Rejected	34	1.4121240699938e-07	0.4167910440727322	0.453	0.223	0.0047359817059452	CCAR1
Rejected	34	1.41524145954689e-07	0.5646193561773511	0.479	0.258	0.00474643680702835	ATG7
Rejected	34	1.43820277306874e-07	0.5828880673657177	0.667	0.431	0.00482344446031795	GNAQ
Rejected	34	1.49534476553138e-07	0.4889589413675299	0.598	0.363	0.00501508727463913	PTEN
Rejected	34	1.49618997570821e-07	0.5553844884140877	0.752	0.572	0.00501792194053019	PRKCB
Rejected	34	1.5295690716139e-07	0.7258798141031333	0.487	0.273	0.00512986875237869	MAPKAP1
Rejected	34	1.57622484902792e-07	0.4587934527638266	0.556	0.318	0.00528634289866985	RALGAPA2
Rejected	34	1.580752815748e-07	0.3179899643109633	0.316	0.133	0.00530152879345566	SEC23B
Rejected	34	1.59503596711165e-07	0.3753734497924866	0.496	0.26	0.00534943162649907	WDR43
Rejected	34	1.62927184455914e-07	0.2750220284785988	0.316	0.132	0.00546425191228244	CAMK2G
Rejected	34	1.6411361418593e-07	0.4762010974891588	0.496	0.261	0.00550404239256771	ATR
Rejected	34	1.6508379375435e-07	0.5106417536997733	0.735	0.501	0.0055365802749334	SND1

Rejected	34	1.65747100690675e-07	0.38696903044765[illegible]	0.718	0.44	0.00555882626296387	ZNF638
Rejected	34	1.66208838470115e-07	0.32102635459261[illegible]	0.368	0.165	0.0055743120246107	AC090627.1
Rejected	34	1.72639737146532e-07	0.38885080385954[illegible]	0.829	0.531	0.00578999150442037	VPS13C
Rejected	34	1.74328684895291e-07	0.26227183022846[illegible]	0.308	0.128	0.00584663543401827	CARNMT1
Rejected	34	1.8277614172552e-07	0.42111138072766	0.709	0.445	0.00612994624119048	RSF1
Rejected	34	1.93686913260881e-07	0.41742506443366[illegible]	0.624	0.352	0.00649587169694343	NFATC3
Rejected	34	1.94202220798682e-07	0.34079565766285[illegible]	0.222	0.08	0.00651315408114619	CPNE8
Rejected	34	2.12645233396803e-07	0.25241500155164	0.752	0.431	0.00713169583766198	OGA
Rejected	34	2.13465142093987e-07	0.35182525342005[illegible]	0.35	0.157	0.00715919393554813	TYW1B
Rejected	34	2.13858243398795e-07	0.45080704951126[illegible]	0.863	0.643	0.0071723777671088	KAT6A
Rejected	34	2.16849582005461e-07	0.506631118543632	0.709	0.474	0.00727270128129915	PDS5A
Rejected	34	2.19217090051472e-07	0.27149237206805[illegible]	0.248	0.096	0.00735210276614628	DNASE1
Rejected	34	2.27124997829037e-07	0.55322950586052[illegible]	0.761	0.487	0.00761731817719026	EMB
Rejected	34	2.27968318259158e-07	0.35132765991006[illegible]	0.487	0.244	0.00764560145777563	ELP2
Rejected	34	2.31854206154281e-07	0.44032488629829[illegible]	0.65	0.387	0.00777592636600229	SLA
Rejected	34	2.34850582401076e-07	0.30956215756766[illegible]	0.547	0.288	0.00787641883256728	SREK1
Rejected	34	2.40248531987086e-07	0.27641072918550[illegible]	0.333	0.144	0.00805745526578287	AC009554.1
Rejected	34	2.40581133124162e-07	0.48433868609699[illegible]	0.564	0.322	0.00806861004271815	WDR7
Rejected	34	2.41093122822303e-07	0.31602394890622[illegible]	0.444	0.219	0.00808578115321439	MED27
Rejected	34	2.41195493217524e-07	0.39171306707410[illegible]	0.701	0.425	0.00808921445152932	HUWE1
Rejected	34	2.45657741915828e-07	0.36938081053894[illegible]	0.795	0.505	0.00823886934837303	USP47
Rejected	34	2.49864535988902e-07	0.34217142379089[illegible]	0.436	0.215	0.00837995680799578	PIK3CB
Rejected	34	2.59659969640062e-07	0.30891639699045[illegible]	0.171	0.055	0.0087084760617884	ASB7
Rejected	34	2.64427418123298e-07	0.38575495908769[illegible]	0.179	0.06	0.00886836674901918	LRRN3
Rejected	34	2.6671381433027e-07	0.48766124793992[illegible]	0.915	0.691	0.00894504790500861	OSBPL8
Rejected	34	2.78523997850181e-07	0.36767641966359[illegible]	0.376	0.176	0.00934113783989936	NUP160
Rejected	34	2.81260875909334e-07	0.27880471433482[illegible]	0.274	0.112	0.00943292725624724	ZNF736
Rejected	34	2.84452416127358e-07	0.39061233882070[illegible]	0.923	0.626	0.00953996513207933	HERC1
Rejected	34	2.88926307744787e-07	0.39919481851348[illegible]	0.436	0.217	0.00969001050914466	TAF1
Rejected	34	2.93220693534275e-07	0.30647322429529[illegible]	0.282	0.118	0.00983403561975251	RCL1
Rejected	34	2.95397467298819e-07	0.45493243355115[illegible]	0.581	0.336	0.00990704025826779	SCLT1

Rejected	34	2.96657272192955e-07	0.427171311641761	0.761	0.501	0.00994929159480732	ACAP2
Rejected	34	2.99247151563515e-07	0.260421953731704	0.239	0.09	0.0100361509691372	AC093019.2
Rejected	34	3.10067115021212e-07	0.251503659952635	0.197	0.067	0.0103990309035814	ABCG2
Rejected	34	3.10782714532396e-07	0.714719404505924	0.821	0.571	0.0104230306799875	NR3C1
Rejected	34	3.10910470743519e-07	0.515281074482588	0.496	0.277	0.0104273153677961	SLCO3A1
Rejected	34	3.11578189756339e-07	0.480717988939372	0.615	0.363	0.0104497093280481	HIPK2
Rejected	34	3.19770853847754e-07	0.402545996405502	0.769	0.513	0.010724474896346	AHNAK
Rejected	34	3.20210794951345e-07	0.335447254947777	0.436	0.219	0.0107392296410782	USP14
Rejected	34	3.22231201615132e-07	0.296401833809788	0.368	0.169	0.0108069900397683	MIGA1
Rejected	34	3.23208097301563e-07	0.324277490552415	0.35	0.159	0.0108397531672998	FBXO42
Rejected	34	3.33696859285559e-07	0.252083364994353	0.282	0.114	0.0111915252667191	BMP2K
Rejected	34	3.37658241545492e-07	0.414881103282083	0.487	0.259	0.0113243821049527	VOPP1
Rejected	34	3.40314871877574e-07	0.350709833914716	0.667	0.388	0.0114134801730301	MARK3
Rejected	34	3.4111551427126e-07	0.271338559191818	0.308	0.132	0.0114403321176295	SLC7A1
Rejected	34	3.4773257555362e-07	0.575149078095419	0.256	0.103	0.0116622551189173	MEGF9
Rejected	34	3.48164729870766e-07	0.291741717688656	0.675	0.389	0.0116767487104058	EHMT1
Rejected	34	3.51706451275854e-07	0.308949787792358	0.256	0.101	0.0117955309628896	SLF2
Rejected	34	3.52800962600029e-07	0.39986387132456	0.385	0.184	0.0118322386836798	VRK2
Rejected	34	3.5527534614966e-07	0.400565696924871	0.726	0.463	0.0119152245591673	GSK3B
Rejected	34	3.56911010458956e-07	0.452733444072955	0.368	0.171	0.0119700814687725	LPAR6
Rejected	34	3.6142604508615e-07	0.345097209984633	0.393	0.185	0.0121215067000993	VTA1
Rejected	34	3.62146572778878e-07	0.73253497387309	0.632	0.384	0.012145671757858	TRPS1
Rejected	34	3.62675780326493e-07	0.485953106950747	0.504	0.272	0.0121634203205899	AC104365.1
Rejected	34	3.65666276150099e-07	0.289754331885957	0.564	0.301	0.012263715569522	AP2B1
Rejected	34	3.69145547233112e-07	0.382717738798423	0.726	0.458	0.0123804033631041	TNRC6A
Rejected	34	3.74873227450688e-07	0.447894354892449	0.556	0.303	0.0125724983022412	TLK2
Rejected	34	3.8554619114772e-07	0.461030089017323	0.855	0.661	0.0129304481587122	TNFAIP8
Rejected	34	3.87009345088674e-07	0.402183283053633	0.47	0.247	0.0129795194155839	USP37
Rejected	34	3.89116198465821e-07	0.4793642982216	0.863	0.661	0.0130501790641467	MED13L
Rejected	34	3.906426595918e-07	0.269020640936398	0.658	0.355	0.0131013735173898	MAX
Rejected	34	3.91846210271353e-07	0.361913848534597	0.547	0.289	0.0131417382000806	USP8

Rejected	34	3.97464975873405e-07	0.387146728725325	0.504	0.267	0.0133301803608423	RABEP1
Rejected	34	4.0162543437606e-07	0.279709853862199	0.427	0.204	0.0134697138181043	AUH
Rejected	34	4.02085315117374e-07	0.395732487122817	0.504	0.267	0.0134851372984065	UBA6
Rejected	34	4.06165920038205e-07	0.26710525414753	0.35	0.158	0.0136219926262413	TENT4A
Rejected	34	4.12103711363149e-07	0.301671098296762	0.872	0.58	0.0138211342716973	CSDE1
Rejected	34	4.12597393211936e-07	0.365566806570198	0.641	0.374	0.0138376913735419	NSD1
Rejected	34	4.1339242776185e-07	0.280858840787664	0.299	0.128	0.0138643552422769	NNT
Rejected	34	4.16798684118291e-07	0.352437975409264	0.436	0.217	0.0139785942679592	RNF169
Rejected	34	4.16950648008699e-07	0.323421082371282	0.453	0.231	0.0139836908329158	DDX10
Rejected	34	4.26006572179264e-07	0.334167108654476	0.838	0.598	0.0142874084177482	PAN3
Rejected	34	4.31022314524968e-07	0.583056705710817	0.615	0.38	0.0144556263845384	XYLT1
Rejected	34	4.34446288367343e-07	0.5716312577553	0.709	0.458	0.0145704596192639	PAG1
Rejected	34	4.3831139988107e-07	0.421931294835847	0.786	0.529	0.0147000877292113	MARCH6
Rejected	34	4.51108452711528e-07	0.32694720263836	0.145	0.043	0.0151292752870392	C9orf84
Rejected	34	4.56058256879841e-07	0.446406554942904	0.444	0.238	0.0152952818192361	RETREG1
Rejected	34	4.58100027313429e-07	0.347787780988628	0.744	0.479	0.0153637587160378	ARFGEF1
Rejected	34	4.6196407523266e-07	0.464533394794798	0.872	0.613	0.015493351155153	KANSL1
Rejected	34	4.76516274034998e-07	0.273833598756256	0.137	0.039	0.0159814027985858	AC104596.1
Rejected	34	4.76791289000236e-07	0.348659449203644	0.427	0.214	0.0159906262504899	RABGAP1
Rejected	34	4.79981383550763e-07	0.383096326313873	0.632	0.37	0.0160976156415255	CHD6
Rejected	34	4.99465473512804e-07	0.317564871219856	0.735	0.43	0.0167510730506724	SEMA4D
Rejected	34	5.0595073783836e-07	0.387972189674808	0.274	0.114	0.0169685758456229	DENND5A
Rejected	34	5.07772214705447e-07	0.417078640893206	0.872	0.581	0.0170296645367913	PPP6R3
Rejected	34	5.09807626128338e-07	0.415522860784301	0.282	0.121	0.0170979281650922	SLC44A1
Rejected	34	5.11886593476178e-07	0.369505284060785	0.291	0.125	0.0171676525720041	KATNA1
Rejected	34	5.15474471342497e-07	0.369785527631524	0.487	0.258	0.0172879828198847	TYW1
Rejected	34	5.15648086851321e-07	0.531745198741082	0.846	0.639	0.0172938055368196	SH3KBP1
Rejected	34	5.31809125285796e-07	0.333983468687572	0.692	0.404	0.017835814443835	SRSF4
Rejected	34	5.35517832358675e-07	0.522867672863524	0.402	0.203	0.0179601970616453	APBA2
Rejected	34	5.38849347165276e-07	0.342324707750403	0.684	0.406	0.018071929405229	XRN2
Rejected	34	5.50929556865604e-07	0.624178143504841	0.701	0.479	0.0184770754781586	MBD5

Rejected	34	5.82601019902383e-07	0.25210706495716	0.282	0.117	0.0195392730054861	ABCC4
Rejected	34	5.84194495854789e-07	0.31803758974312	0.419	0.212	0.0195927150019779	DGLUCY
Rejected	34	5.99927942957741e-07	0.32741812395941	0.444	0.225	0.0201203833509167	NR2C2
Rejected	34	6.05926820520431e-07	0.65248463751190	0.872	0.652	0.0203215737066142	ADGRE5
Rejected	34	6.06491592026223e-07	0.28674446510698	0.188	0.065	0.0203405150133755	TCAIM
Rejected	34	6.09964426034128e-07	0.29015843580439	0.769	0.47	0.0204569869203326	RAB1A
Rejected	34	6.1913816460401e-07	0.35638944361753	0.265	0.109	0.0207646557644893	PPARD
Rejected	34	6.2035541191743e-07	0.40930657404932	0.761	0.508	0.0208054798048868	NCOR1
Rejected	34	6.29921360942714e-07	0.28967313317768	0.47	0.238	0.0211263026032967	ANAPC5
Rejected	34	6.39569109044168e-07	0.38905430496746	0.872	0.674	0.0214498687791233	ATF7IP
Rejected	34	6.43275109621232e-07	0.33720105503618	0.573	0.318	0.0215741606264769	WBP11
Rejected	34	6.4401200137024e-07	0.36753545060789	0.675	0.403	0.0215988745019551	SCAF8
Rejected	34	6.45211619624321e-07	0.75312938136340	0.846	0.643	0.0216391072989605	CDC14A
Rejected	34	6.70653390044504e-07	0.36570243057763	0.479	0.257	0.0224923733953126	BTRC
Rejected	34	6.88048705864463e-07	0.27423496514371	0.402	0.193	0.0230757774972823	TRIM14
Rejected	34	6.89320919347885e-07	0.52776428238185	0.769	0.514	0.0231184449930894	RAPGEF2
Rejected	34	7.12197103743766e-07	0.27320579208215	0.496	0.259	0.0238856664653584	ARHGEF1
Rejected	34	7.25947602711916e-07	0.53933259871531	0.752	0.529	0.0243468306997522	USP9X
Rejected	34	7.33688366399259e-07	0.27413811229963	0.222	0.084	0.0246064404322984	TBC1D31
Rejected	34	7.34043423375747e-07	0.41146070756961	0.47	0.256	0.0246183483331758	ANO6
Rejected	34	7.47942089270979e-07	0.25997434617399	0.376	0.178	0.0250844817899701	SEC22B
Rejected	34	7.52433035982628e-07	0.29511861079535	0.752	0.44	0.0252350991607854	USP25
Rejected	34	7.5328689753511e-07	0.37643532136048	0.581	0.324	0.0252637359695325	PTBP2
Rejected	34	7.7374989681366e-07	0.42821824610934	0.453	0.24	0.0259500240393365	ATP11C
Rejected	34	7.73897988888071e-07	0.45262852677412	0.658	0.409	0.0259549907513281	DIAPH2
Rejected	34	7.77773046091434e-07	0.42861719669503	0.427	0.215	0.0260849524198145	DICER1
Rejected	34	7.79715064290845e-07	0.38142333268121	0.496	0.269	0.0261500838261863	KPNA1
Rejected	34	7.87418152653984e-07	0.34288916258850	0.444	0.236	0.0264084300037093	SLC20A2
Rejected	34	7.89447608683442e-07	0.41099029422561	0.538	0.303	0.0264764939000253	TESPA1
Rejected	34	8.11286141538523e-07	0.36067747874159	0.402	0.198	0.027208914614919	BTBD10
Rejected	34	8.21213851015258e-07	0.27099676419058	0.692	0.417	0.0275418701353497	MON2

Rejected	34	8.21435489215386e-07	0.3452974176832	0.453	0.233	0.0275493034373056	STAU2
Rejected	34	8.30840260361601e-07	0.416228082379779	0.675	0.426	0.0278647206520074	PHIP
Rejected	34	8.36512933167668e-07	0.391229532291317	0.632	0.387	0.0280549707525773	WDR70
Rejected	34	8.59061264464365e-07	0.814987514243455	0.462	0.274	0.0288111966876059	CMTM8
Rejected	34	8.67081900364071e-07	0.378376518854605	0.709	0.439	0.0290801927744102	GCC2
Rejected	34	8.7081676657831e-07	0.269375974929419	0.162	0.053	0.0292054527175034	AC008878.3
Rejected	34	8.9856756354716e-07	0.41618356462414	0.667	0.402	0.0301361589462447	ATP9B
Rejected	34	9.22191863781424e-07	0.309937883524793	0.444	0.233	0.0309284707275014	TNPO3
Rejected	34	9.3179979639749e-07	0.44179041410592	0.171	0.059	0.031250701571579	AFG1L
Rejected	34	9.40017066147379e-07	0.37164915125002	0.41	0.204	0.0315262923644508	TTC39B
Rejected	34	9.42641210531505e-07	0.390060135348347	0.479	0.261	0.0316143009188056	RNF115
Rejected	34	9.51174951939511e-07	0.410406216631391	0.872	0.67	0.0319005055381473	BIRC6
Rejected	34	9.69085985107314e-07	0.36006130623394	0.513	0.286	0.0325012057685291	VPS13A
Rejected	34	9.88408696875655e-07	0.420262431946667	0.496	0.276	0.0331492508758157	SNX29
Rejected	34	9.89216544031526e-07	0.289681868894057	0.47	0.246	0.0331763444537293	CDYL
Rejected	34	9.89884878022649e-07	0.426012661154771	0.923	0.688	0.0331987590391236	DOCK8
Rejected	34	9.97272593350091e-07	0.42675864394212	0.632	0.377	0.0334465282357754	TMEM131
Rejected	34	1.00826960081855e-06	0.320803096340939	0.419	0.204	0.0338153458722524	SPDYA
Rejected	34	1.01467586323486e-06	0.320566290163528	0.222	0.087	0.0340301991011707	ACAT2
Rejected	34	1.04182063612254e-06	0.514695418943873	0.342	0.163	0.0349405804942777	ATG10
Rejected	34	1.04185054962994e-06	0.526795711975917	0.581	0.361	0.034941583733489	CD6
Rejected	34	1.04256797491425e-06	0.533143922863383	0.718	0.533	0.0349656447426743	FBXL17
Rejected	34	1.0634745139152e-06	0.586426287241954	0.59	0.34	0.0356668082476878	RBPJ
Rejected	34	1.08505621845426e-06	0.301918125405754	0.436	0.222	0.036390615454519	HELB
Rejected	34	1.10537531194975e-06	0.383641316877764	0.444	0.231	0.0370720772121709	ATP2C1
Rejected	34	1.11908862517689e-06	0.280040536140284	0.239	0.097	0.0375319943111826	MAPKAPK5
Rejected	34	1.12491320608214e-06	0.37165659519314	0.256	0.107	0.037727339105583	DSTYK
Rejected	34	1.13588657777521e-06	0.277010520804985	0.35	0.165	0.038095364045425	ATG2B
Rejected	34	1.13736602363956e-06	0.576671519145696	0.718	0.518	0.0381449817008235	BBX
Rejected	34	1.13784515060595e-06	0.270801929309557	0.547	0.296	0.0381610506610223	NAA15
Rejected	34	1.15148364191657e-06	0.272484337373821	0.179	0.062	0.038618458382598	CMTR1

Rejected	34	1.16389795366864e-06	0.349858813678809	0.615	0.352	0.0390348095701387	CA5B
Rejected	34	1.17591663989502e-06	0.315375220946535	0.538	0.291	0.0394378922687992	PRKX
Rejected	34	1.19751976354806e-06	0.54891559565065	0.761	0.55	0.0401624178298749	VTI1A
Rejected	34	1.20087447265477e-06	0.300172672884159	0.436	0.22	0.0402749280638958	PRKAA1
Rejected	34	1.2110511328425e-06	0.6498030830778	0.453	0.248	0.0406162328932716	WDFY2
Rejected	34	1.21500119207135e-06	0.273365537518779	0.615	0.36	0.040748709979689	UBE3A
Rejected	34	1.22097519383042e-06	0.527792741240731	0.368	0.181	0.0409490660506845	HTT
Rejected	34	1.23478363549517e-06	0.776612294287224	0.821	0.617	0.0414121735672369	BCL2
Rejected	34	1.24368793641146e-06	0.333132427762162	0.658	0.398	0.0417108060113675	ADAM10
Rejected	34	1.24699315460975e-06	0.31857912263506	0.359	0.174	0.0418216564193019	EXOC1
Rejected	34	1.26330356806155e-06	0.261850661234084	0.675	0.384	0.0423686750656482	AGO2
Rejected	34	1.26834238757331e-06	0.375345497355667	0.239	0.098	0.0425376669944335	LARS2
Rejected	34	1.2693211018532e-06	0.256437822056066	0.231	0.09	0.0425704911139525	ZYG11B
Rejected	34	1.28341166582129e-06	0.356392213120251	0.726	0.435	0.0430430604483145	UBE2K
Rejected	34	1.29626660042619e-06	0.415454138745375	0.282	0.126	0.0434741892450937	FGGY
Rejected	34	1.31681598518835e-06	0.510890847088105	0.94	0.792	0.0441633745112469	ANKRD12
Rejected	34	1.34202150786731e-06	0.283678701692695	0.265	0.112	0.0450087173308538	RUBCN
Rejected	34	1.35111708928714e-06	0.395077311087259	0.726	0.483	0.0453137649405121	ARID2
Rejected	34	1.35130372604925e-06	0.294165541910873	0.385	0.187	0.0453200243642398	C2CD5
Rejected	34	1.37576502588116e-06	0.29596737053422	0.41	0.201	0.0461404074380022	DOPEY1
Rejected	34	1.38514686772595e-06	0.43225316254724	0.812	0.612	0.0464550556497931	KDM4C
Rejected	34	1.39269784526431e-06	0.330046712789967	0.607	0.354	0.0467083003344743	SLMAP
Rejected	34	1.43772539311564e-06	0.601525255492422	0.65	0.469	0.0482184342343123	MLLT3
Rejected	34	1.47184867969733e-06	0.45954488578855	0.744	0.505	0.0493628610196892	SRPK2
Rejected	34	1.47346327782666e-06	0.281251717210991	0.436	0.22	0.0494170114117506	ARID5A

Supplemental Digital Conte

Transplan	Day	Pathway identifie
Successfu	0	R-HSA-3371568
Successfu	0	R-HSA-3371571
Successfu	0	R-HSA-3371511
Successfu	0	R-HSA-3371556
Successfu	0	R-HSA-3371453
Successfu	0	R-HSA-8868766
Successfu	0	R-HSA-6785470
Successfu	0	R-HSA-174824
Successfu	0	R-HSA-8963889
Successfu	0	R-HSA-917937
Successfu	0	R-HSA-8963899
Successfu	0	R-HSA-383280
Successfu	0	R-HSA-2262752
Successfu	0	R-HSA-8953897
Successfu	0	R-HSA-2173782
Successfu	0	R-HSA-163200
Successfu	0	R-HSA-3371497
Successfu	0	R-HSA-9613829
Successfu	0	R-HSA-6804760
Successfu	0	R-HSA-977225
Successfu	6	R-HSA-9735869
Successfu	6	R-HSA-975956
Successfu	6	R-HSA-72689
Successfu	6	R-HSA-192823
Successfu	6	R-HSA-1799339
Successfu	6	R-HSA-156827
Successfu	6	R-HSA-72706
Successfu	6	R-HSA-72649
Successfu	6	R-HSA-72702

Successfu	6	R-HSA-156842
Successfu	6	R-HSA-6791226
Successfu	6	R-HSA-927802
Successfu	6	R-HSA-156902
Successfu	6	R-HSA-72764
Successfu	6	R-HSA-975957
Successfu	6	R-HSA-72613
Successfu	6	R-HSA-72737
Successfu	6	R-HSA-8868773
Successfu	6	R-HSA-9711097
Successfu	6	R-HSA-72312
Successfu	12	R-HSA-983231
Successfu	12	R-HSA-1247673
Successfu	12	R-HSA-9613829
Successfu	12	R-HSA-1237044
Successfu	12	R-HSA-1480926
Successfu	12	R-HSA-9615710
Successfu	12	R-HSA-9707616
Successfu	12	R-HSA-3371497
Successfu	12	R-HSA-9707564
Successfu	12	R-HSA-2168880
Successfu	12	R-HSA-2408557
Successfu	12	R-HSA-109582
Successfu	12	R-HSA-9612973
Successfu	12	R-HSA-2173782
Successfu	12	R-HSA-2408522
Successfu	12	R-HSA-2262752
Successfu	12	R-HSA-8953897
Successfu	12	R-HSA-9711123
Successfu	12	R-HSA-6798695
Successfu	12	R-HSA-71291

Successfu	19	R-HSA-975956
Successfu	19	R-HSA-72689
Successfu	19	R-HSA-192823
Successfu	19	R-HSA-1799339
Successfu	19	R-HSA-72706
Successfu	19	R-HSA-72649
Successfu	19	R-HSA-72702
Successfu	19	R-HSA-6791226
Successfu	19	R-HSA-927802
Successfu	19	R-HSA-156902
Successfu	19	R-HSA-975957
Successfu	19	R-HSA-156827
Successfu	19	R-HSA-72764
Successfu	19	R-HSA-72613
Successfu	19	R-HSA-72737
Successfu	19	R-HSA-8868773
Successfu	19	R-HSA-156842
Successfu	19	R-HSA-9735869
Successfu	19	R-HSA-72695
Successfu	19	R-HSA-9754678
Successfu	22	R-HSA-983231
Successfu	22	R-HSA-109582
Successfu	26	R-HSA-8868766
Successfu	26	R-HSA-6785470
Successfu	26	R-HSA-72306
Successfu	26	R-HSA-163200
Successfu	26	R-HSA-611105
Successfu	26	R-HSA-72312
Successfu	26	R-HSA-1428517
Successfu	26	R-HSA-6799198
Successfu	26	R-HSA-5661231

Successfu	26	R-HSA-5660526
Successfu	26	R-HSA-8951911
Successfu	26	R-HSA-8953854
Successfu	26	R-HSA-9022707
Successfu	26	R-HSA-8986944
Successfu	26	R-HSA-5657560
Successfu	26	R-HSA-9707564
Successfu	26	R-HSA-8934593
Successfu	26	R-HSA-9022699
Successfu	26	R-HSA-9013508
Successfu	26	R-HSA-9008059
Successfu	33	R-HSA-8963889
Successfu	33	R-HSA-8963899
Successfu	33	R-HSA-174824
Successfu	33	R-HSA-5659898
Successfu	33	R-HSA-8963888
Successfu	33	R-HSA-8963901
Successfu	33	R-HSA-9708530
Successfu	33	R-HSA-189085
Successfu	33	R-HSA-8963898
Successfu	33	R-HSA-9615933
Successfu	33	R-HSA-5619107
Successfu	33	R-HSA-159227
Successfu	33	R-HSA-4085377
Successfu	33	R-HSA-159230
Successfu	33	R-HSA-170822
Successfu	33	R-HSA-180746
Successfu	33	R-HSA-3301854
Successfu	33	R-HSA-3232142
Successfu	33	R-HSA-180910
Successfu	33	R-HSA-165054

Successfu	59	R-HSA-5626467
Successfu	59	R-HSA-9031628
Successfu	59	R-HSA-9013406
Successfu	59	R-HSA-9013106
Successfu	59	R-HSA-198725
Successfu	59	R-HSA-187037
Successfu	59	R-HSA-8980692
Successfu	59	R-HSA-9013148
Successfu	59	R-HSA-166520
Successfu	59	R-HSA-9013149
Successfu	59	R-HSA-2514853
Successfu	59	R-HSA-195258
Successfu	59	R-HSA-373753
Successfu	59	R-HSA-9012999
Successfu	59	R-HSA-9013424
Successfu	59	R-HSA-6802948
Successfu	59	R-HSA-9013420
Successfu	59	R-HSA-189451
Successfu	59	R-HSA-5674135
Successfu	59	R-HSA-9656223
Successfu	64	R-HSA-3000170
Successfu	64	R-HSA-216083
Successfu	64	R-HSA-9006934
Successfu	64	R-HSA-3000171
Successfu	64	R-HSA-1474244
Successfu	64	R-HSA-8874081
Successfu	64	R-HSA-74751
Successfu	64	R-HSA-3000178
Successfu	64	R-HSA-6806834
Successfu	64	R-HSA-8948216
Successfu	64	R-HSA-1474228

Successfu	64	R-HSA-8875878
Successfu	64	R-HSA-75892
Successfu	64	R-HSA-2219530
Successfu	64	R-HSA-74752
Successfu	64	R-HSA-109704
Successfu	64	R-HSA-112399
Successfu	64	R-HSA-2219528
Successfu	64	R-HSA-2022090
Successfu	64	R-HSA-1839120
Successfu	191	R-HSA-428359
Successfu	191	R-HSA-2173788
Successfu	191	R-HSA-8951911
Successfu	191	R-HSA-186797
Successfu	191	R-HSA-3656532
Successfu	191	R-HSA-1306955
Successfu	191	R-HSA-3656534
Successfu	191	R-HSA-3304356
Successfu	191	R-HSA-2173789
Successfu	191	R-HSA-3304349
Successfu	191	R-HSA-3304351
Successfu	191	R-HSA-164944
Successfu	191	R-HSA-164952
Successfu	191	R-HSA-3000171
Successfu	191	R-HSA-2219530
Successfu	191	R-HSA-9700645
Successfu	191	R-HSA-186763
Successfu	191	R-HSA-2173795
Successfu	191	R-HSA-170834
Successfu	191	R-HSA-1474228
Recoverec	0	R-HSA-202733
Recoverec	0	R-HSA-983695

Recoverec	0	R-HSA-9615017
Recoverec	0	R-HSA-8963899
Recoverec	0	R-HSA-174824
Recoverec	0	R-HSA-8941333
Recoverec	0	R-HSA-2168880
Recoverec	0	R-HSA-8939245
Recoverec	0	R-HSA-8963889
Recoverec	0	R-HSA-5690714
Recoverec	0	R-HSA-983705
Recoverec	0	R-HSA-9632974
Recoverec	0	R-HSA-8939246
Recoverec	0	R-HSA-9024446
Recoverec	0	R-HSA-109582
Recoverec	0	R-HSA-9027277
Recoverec	0	R-HSA-8963888
Recoverec	0	R-HSA-202433
Recoverec	0	R-HSA-5423646
Recoverec	0	R-HSA-9623433
Recoverec	13	R-HSA-72689
Recoverec	13	R-HSA-975956
Recoverec	13	R-HSA-9735869
Recoverec	13	R-HSA-72706
Recoverec	13	R-HSA-156827
Recoverec	13	R-HSA-192823
Recoverec	13	R-HSA-1799339
Recoverec	13	R-HSA-72649
Recoverec	13	R-HSA-72702
Recoverec	13	R-HSA-156842
Recoverec	13	R-HSA-6791226
Recoverec	13	R-HSA-927802
Recoverec	13	R-HSA-156902

Recoverec	13	R-HSA-975957
Recoverec	13	R-HSA-72613
Recoverec	13	R-HSA-72764
Recoverec	13	R-HSA-72737
Recoverec	13	R-HSA-8868773
Recoverec	13	R-HSA-9711097
Recoverec	13	R-HSA-72312
Recoverec	20	R-HSA-1059683
Recoverec	20	R-HSA-1839124
Recoverec	20	R-HSA-6783589
Recoverec	20	R-HSA-5655302
Recoverec	20	R-HSA-1226099
Recoverec	20	R-HSA-1433557
Recoverec	20	R-HSA-3700989
Recoverec	20	R-HSA-1839117
Recoverec	20	R-HSA-9020956
Recoverec	20	R-HSA-9670439
Recoverec	20	R-HSA-6804115
Recoverec	20	R-HSA-9669938
Recoverec	20	R-HSA-8984722
Recoverec	20	R-HSA-1839120
Recoverec	20	R-HSA-1980143
Recoverec	20	R-HSA-6796648
Recoverec	20	R-HSA-9673767
Recoverec	20	R-HSA-9673770
Recoverec	20	R-HSA-8868766
Recoverec	20	R-HSA-2428928
Recoverec	26	R-HSA-3371568
Recoverec	26	R-HSA-3371571
Recoverec	26	R-HSA-3371556
Recoverec	26	R-HSA-3371511

Recoverec	26	R-HSA-3371453
Recoverec	26	R-HSA-8953897
Recoverec	26	R-HSA-5661231
Recoverec	26	R-HSA-2262752
Recoverec	26	R-HSA-5660526
Recoverec	26	R-HSA-3371497
Recoverec	26	R-HSA-9006931
Recoverec	26	R-HSA-8869496
Recoverec	26	R-HSA-5336415
Recoverec	26	R-HSA-9018519
Recoverec	26	R-HSA-2173782
Recoverec	26	R-HSA-9623433
Recoverec	26	R-HSA-3000484
Recoverec	26	R-HSA-399954
Recoverec	26	R-HSA-9613829
Recoverec	26	R-HSA-173623
Recoverec	33	R-HSA-6785470
Recoverec	33	R-HSA-451927
Recoverec	33	R-HSA-428540
Recoverec	33	R-HSA-449147
Recoverec	33	R-HSA-186763
Recoverec	33	R-HSA-8868766
Recoverec	33	R-HSA-6799198
Recoverec	33	R-HSA-1280215
Recoverec	33	R-HSA-8983432
Recoverec	33	R-HSA-163200
Recoverec	33	R-HSA-186797
Recoverec	33	R-HSA-8877330
Recoverec	33	R-HSA-8950505
Recoverec	33	R-HSA-9020591
Recoverec	33	R-HSA-72306

Recoverec	33	R-HSA-373753
Recoverec	33	R-HSA-8953854
Recoverec	33	R-HSA-447115
Recoverec	33	R-HSA-6804115
Recoverec	33	R-HSA-912526
Recoverec	40	R-HSA-6785470
Recoverec	40	R-HSA-8868766
Recoverec	40	R-HSA-5661231
Recoverec	40	R-HSA-5660526
Recoverec	40	R-HSA-72306
Recoverec	40	R-HSA-611105
Recoverec	40	R-HSA-2022377
Recoverec	40	R-HSA-8963888
Recoverec	40	R-HSA-163200
Recoverec	40	R-HSA-6799198
Recoverec	40	R-HSA-8942233
Recoverec	40	R-HSA-8963898
Recoverec	40	R-HSA-8951911
Recoverec	40	R-HSA-72312
Recoverec	40	R-HSA-171306
Recoverec	40	R-HSA-73728
Recoverec	40	R-HSA-8963901
Recoverec	40	R-HSA-8963899
Recoverec	40	R-HSA-5334118
Recoverec	40	R-HSA-1428517
Recoverec	47	R-HSA-8941332
Recoverec	47	R-HSA-8941333
Recoverec	47	R-HSA-9818025
Recoverec	47	R-HSA-6785807
Recoverec	47	R-HSA-8941284
Recoverec	47	R-HSA-8878166

Recoverec	47	R-HSA-8949275
Recoverec	47	R-HSA-8939246
Recoverec	47	R-HSA-198693
Recoverec	47	R-HSA-8963888
Recoverec	47	R-HSA-9614399
Recoverec	47	R-HSA-8963899
Recoverec	47	R-HSA-8963901
Recoverec	47	R-HSA-2032785
Recoverec	47	R-HSA-975634
Recoverec	47	R-HSA-8939902
Recoverec	47	R-HSA-9634638
Recoverec	47	R-HSA-5674400
Recoverec	47	R-HSA-6806667
Recoverec	47	R-HSA-8963898
Recoverec	98	R-HSA-975956
Recoverec	98	R-HSA-72689
Recoverec	98	R-HSA-192823
Recoverec	98	R-HSA-1799339
Recoverec	98	R-HSA-72706
Recoverec	98	R-HSA-156827
Recoverec	98	R-HSA-72649
Recoverec	98	R-HSA-72702
Recoverec	98	R-HSA-6791226
Recoverec	98	R-HSA-927802
Recoverec	98	R-HSA-156902
Recoverec	98	R-HSA-975957
Recoverec	98	R-HSA-72613
Recoverec	98	R-HSA-72764
Recoverec	98	R-HSA-72737
Recoverec	98	R-HSA-8868773
Recoverec	98	R-HSA-156842

Recoverec	98	R-HSA-9735869
Recoverec	98	R-HSA-72695
Recoverec	98	R-HSA-72662
Recoverec	121	R-HSA-9759811
Recoverec	121	R-HSA-9759475
Recoverec	121	R-HSA-9764260
Recoverec	121	R-HSA-9759476
Recoverec	121	R-HSA-9762293
Recoverec	121	R-HSA-418990
Recoverec	121	R-HSA-8948216
Recoverec	121	R-HSA-8875878
Recoverec	121	R-HSA-216083
Recoverec	121	R-HSA-6806834
Recoverec	121	R-HSA-421270
Recoverec	121	R-HSA-1306955
Recoverec	121	R-HSA-3000170
Recoverec	121	R-HSA-1500931
Recoverec	121	R-HSA-2022090
Recoverec	121	R-HSA-1442490
Recoverec	121	R-HSA-8874081
Recoverec	121	R-HSA-186797
Recoverec	121	R-HSA-8951671
Recoverec	121	R-HSA-9662001
Recoverec	183	R-HSA-1306955
Recoverec	183	R-HSA-1358803
Recoverec	183	R-HSA-8847993
Recoverec	183	R-HSA-6785631
Recoverec	183	R-HSA-1963642
Recoverec	183	R-HSA-9665686
Recoverec	183	R-HSA-1250196
Recoverec	183	R-HSA-9664565

Recoverec	183	R-HSA-1227990
Recoverec	183	R-HSA-8863795
Recoverec	183	R-HSA-3656532
Recoverec	183	R-HSA-3304356
Recoverec	183	R-HSA-3656534
Recoverec	183	R-HSA-3304349
Recoverec	183	R-HSA-9013419
Recoverec	183	R-HSA-1227986
Recoverec	183	R-HSA-3304351
Recoverec	183	R-HSA-9715370
Recoverec	183	R-HSA-8848021
Recoverec	183	R-HSA-9006927
Rejected	0	R-HSA-156902
Rejected	0	R-HSA-156842
Rejected	0	R-HSA-975956
Rejected	0	R-HSA-72689
Rejected	0	R-HSA-192823
Rejected	0	R-HSA-72706
Rejected	0	R-HSA-156827
Rejected	0	R-HSA-1799339
Rejected	0	R-HSA-72649
Rejected	0	R-HSA-72702
Rejected	0	R-HSA-6791226
Rejected	0	R-HSA-927802
Rejected	0	R-HSA-975957
Rejected	0	R-HSA-9735869
Rejected	0	R-HSA-72613
Rejected	0	R-HSA-72764
Rejected	0	R-HSA-72737
Rejected	0	R-HSA-8868773
Rejected	0	R-HSA-72312

Rejected	0	R-HSA-72766
Rejected	6	R-HSA-6785807
Rejected	6	R-HSA-449147
Rejected	6	R-HSA-8868766
Rejected	6	R-HSA-6785470
Rejected	6	R-HSA-1280215
Rejected	6	R-HSA-9634638
Rejected	6	R-HSA-9648895
Rejected	6	R-HSA-448424
Rejected	6	R-HSA-6783783
Rejected	6	R-HSA-168256
Rejected	6	R-HSA-380108
Rejected	6	R-HSA-8877330
Rejected	6	R-HSA-450294
Rejected	6	R-HSA-8866910
Rejected	6	R-HSA-9669937
Rejected	6	R-HSA-9669921
Rejected	6	R-HSA-9669934
Rejected	6	R-HSA-9669924
Rejected	6	R-HSA-9669926
Rejected	6	R-HSA-9669936
Rejected	14	R-HSA-1280215
Rejected	14	R-HSA-913531
Rejected	14	R-HSA-877300
Rejected	14	R-HSA-909733
Rejected	14	R-HSA-1462054
Rejected	14	R-HSA-168256
Rejected	14	R-HSA-1221632
Rejected	14	R-HSA-1500620
Rejected	14	R-HSA-449147
Rejected	14	R-HSA-1461973

Rejected	14	R-HSA-8939236
Rejected	14	R-HSA-1059683
Rejected	14	R-HSA-68867
Rejected	14	R-HSA-157118
Rejected	14	R-HSA-3214815
Rejected	14	R-HSA-69002
Rejected	14	R-HSA-5688426
Rejected	14	R-HSA-449836
Rejected	14	R-HSA-201681
Rejected	14	R-HSA-1474165
Rejected	20	R-HSA-1280215
Rejected	20	R-HSA-5661231
Rejected	20	R-HSA-168256
Rejected	20	R-HSA-5660526
Rejected	20	R-HSA-209560
Rejected	20	R-HSA-913531
Rejected	20	R-HSA-1236975
Rejected	20	R-HSA-909733
Rejected	20	R-HSA-193639
Rejected	20	R-HSA-1236974
Rejected	20	R-HSA-449147
Rejected	20	R-HSA-9637628
Rejected	20	R-HSA-1799339
Rejected	20	R-HSA-9664873
Rejected	20	R-HSA-977225
Rejected	20	R-HSA-156902
Rejected	20	R-HSA-209543
Rejected	20	R-HSA-975956
Rejected	20	R-HSA-156842
Rejected	20	R-HSA-8953897
Rejected	23	R-HSA-428359

Rejected	23	R-HSA-383280
Rejected	23	R-HSA-8953854
Rejected	23	R-HSA-170834
Rejected	23	R-HSA-2173789
Rejected	23	R-HSA-2173788
Rejected	23	R-HSA-2990846
Rejected	23	R-HSA-3108232
Rejected	23	R-HSA-4839726
Rejected	23	R-HSA-3247509
Rejected	23	R-HSA-1839117
Rejected	23	R-HSA-2980766
Rejected	23	R-HSA-1169408
Rejected	23	R-HSA-9006936
Rejected	23	R-HSA-3134963
Rejected	23	R-HSA-72203
Rejected	23	R-HSA-5663202
Rejected	23	R-HSA-9837092
Rejected	23	R-HSA-9703465
Rejected	23	R-HSA-2470946
Rejected	27	R-HSA-975956
Rejected	27	R-HSA-72689
Rejected	27	R-HSA-192823
Rejected	27	R-HSA-1799339
Rejected	27	R-HSA-72706
Rejected	27	R-HSA-156827
Rejected	27	R-HSA-927802
Rejected	27	R-HSA-156902
Rejected	27	R-HSA-975957
Rejected	27	R-HSA-72613
Rejected	27	R-HSA-72764
Rejected	27	R-HSA-72737

Rejected	27	R-HSA-156842
Rejected	27	R-HSA-6791226
Rejected	27	R-HSA-9735869
Rejected	27	R-HSA-2408557
Rejected	27	R-HSA-72766
Rejected	27	R-HSA-9633012
Rejected	27	R-HSA-9010553
Rejected	27	R-HSA-8868773
Rejected	30	R-HSA-4839726
Rejected	30	R-HSA-3247509
Rejected	30	R-HSA-194315
Rejected	30	R-HSA-9716542
Rejected	30	R-HSA-5663202
Rejected	30	R-HSA-9022692
Rejected	30	R-HSA-9012999
Rejected	30	R-HSA-9013149
Rejected	30	R-HSA-6802957
Rejected	30	R-HSA-9022537
Rejected	30	R-HSA-9675151
Rejected	30	R-HSA-9005891
Rejected	30	R-HSA-9005895
Rejected	30	R-HSA-9697154
Rejected	30	R-HSA-6802952
Rejected	30	R-HSA-400253
Rejected	30	R-HSA-170834
Rejected	30	R-HSA-8939246
Rejected	30	R-HSA-5683057
Rejected	30	R-HSA-2173788
Rejected	34	R-HSA-9022692
Rejected	34	R-HSA-8986944
Rejected	34	R-HSA-4839726

Rejected	34	R-HSA-3247509
Rejected	34	R-HSA-5684264
Rejected	34	R-HSA-426496
Rejected	34	R-HSA-383280
Rejected	34	R-HSA-975871
Rejected	34	R-HSA-168176
Rejected	34	R-HSA-168142
Rejected	34	R-HSA-350054
Rejected	34	R-HSA-2122947
Rejected	34	R-HSA-2644606
Rejected	34	R-HSA-2644602
Rejected	34	R-HSA-2894862
Rejected	34	R-HSA-2894858
Rejected	34	R-HSA-2644603
Rejected	34	R-HSA-983168
Rejected	34	R-HSA-8939243
Rejected	34	R-HSA-975138

nt, Table S3

Pathway name (only top 20 per transplant and per day is shown)	#Entities foun
Attenuation phase	16
HSF1-dependent transactivation	16
HSF1 activation	13
Cellular response to heat stress	17
Regulation of HSF1-mediated heat shock response	16
rRNA processing in the mitochondrion	6
tRNA processing in the mitochondrion	6
Plasma lipoprotein assembly, remodeling, and clearance	8
Assembly of active LPL and LIPC lipase complexes	5
Iron uptake and transport	7
Plasma lipoprotein remodeling	6
Nuclear Receptor transcription pathway	7
Cellular responses to stress	24
Cellular responses to stimuli	24
Binding and Uptake of Ligands by Scavenger Receptors	9
Respiratory electron transport, ATP synthesis by chemiosmotic coupling, and heat production by uncoupling prc	7
HSP90 chaperone cycle for steroid hormone receptors (SHR) in the presence of ligand	5
Chaperone Mediated Autophagy	3
Regulation of TP53 Activity through Methylation	3
Amyloid fiber formation	5
SARS-CoV-1 modulates host translation machinery	32
Nonsense Mediated Decay (NMD) independent of the Exon Junction Complex (EJC)	75
Formation of a pool of free 40S subunits	75
Viral mRNA Translation	75
SRP-dependent cotranslational protein targeting to membrane	75
L13a-mediated translational silencing of Ceruloplasmin expression	75
GTP hydrolysis and joining of the 60S ribosomal subunit	75
Translation initiation complex formation	30
Ribosomal scanning and start codon recognition	30

Eukaryotic Translation Elongation	78
Major pathway of rRNA processing in the nucleolus and cytosol	75
Nonsense-Mediated Decay (NMD)	75
Peptide chain elongation	76
Eukaryotic Translation Termination	76
Nonsense Mediated Decay (NMD) enhanced by the Exon Junction Complex (EJC)	75
Eukaryotic Translation Initiation	75
Cap-dependent Translation Initiation	75
rRNA processing in the nucleus and cytosol	76
Cellular response to starvation	76
rRNA processing	76
Factors involved in megakaryocyte development and platelet production	2
Erythrocytes take up oxygen and release carbon dioxide	1
Chaperone Mediated Autophagy	1
Erythrocytes take up carbon dioxide and release oxygen	1
O2/CO2 exchange in erythrocytes	1
Late endosomal microautophagy	1
Heme signaling	1
HSP90 chaperone cycle for steroid hormone receptors (SHR) in the presence of ligand	1
Cytoprotection by HMOX1	1
Scavenging of heme from plasma	1
Selenocysteine synthesis	1
Hemostasis	2
Autophagy	1
Binding and Uptake of Ligands by Scavenger Receptors	1
Selenoamino acid metabolism	1
Cellular responses to stress	2
Cellular responses to stimuli	2
Cellular response to chemical stress	1
Neutrophil degranulation	1
Metabolism of amino acids and derivatives	1

Nonsense Mediated Decay (NMD) independent of the Exon Junction Complex (EJC)	40
Formation of a pool of free 40S subunits	40
Viral mRNA Translation	40
SRP-dependent cotranslational protein targeting to membrane	40
GTP hydrolysis and joining of the 60S ribosomal subunit	40
Translation initiation complex formation	17
Ribosomal scanning and start codon recognition	17
Major pathway of rRNA processing in the nucleolus and cytosol	40
Nonsense-Mediated Decay (NMD)	40
Peptide chain elongation	40
Nonsense Mediated Decay (NMD) enhanced by the Exon Junction Complex (EJC)	40
L13a-mediated translational silencing of Ceruloplasmin expression	40
Eukaryotic Translation Termination	40
Eukaryotic Translation Initiation	40
Cap-dependent Translation Initiation	40
rRNA processing in the nucleus and cytosol	40
Eukaryotic Translation Elongation	40
SARS-CoV-1 modulates host translation machinery	17
Formation of the ternary complex, and subsequently, the 43S complex	17
SARS-CoV-2 modulates host translation machinery	18
Factors involved in megakaryocyte development and platelet production	1
Hemostasis	1
rRNA processing in the mitochondrion	11
tRNA processing in the mitochondrion	11
tRNA processing	11
Respiratory electron transport, ATP synthesis by chemiosmotic coupling, and heat production by uncoupling pro	10
Respiratory electron transport	9
rRNA processing	11
The citric acid (TCA) cycle and respiratory electron transport	10
Complex I biogenesis	5
Metallothioneins bind metals	3

Response to metal ions	3
RUNX3 regulates RUNX1-mediated transcription	2
Metabolism of RNA	12
MECP2 regulates transcription factors	2
Transcriptional Regulation by MECP2	4
Hereditary fructose intolerance	1
Cytoprotection by HMOX1	3
Regulation of RUNX1 Expression and Activity	2
MECP2 regulates neuronal receptors and channels	2
NOTCH3 Intracellular Domain Regulates Transcription	2
Interleukin-37 signaling	2
Assembly of active LPL and LIPC lipase complexes	2
Plasma lipoprotein remodeling	2
Plasma lipoprotein assembly, remodeling, and clearance	2
Intestinal saccharidase deficiencies	1
Chylomicron assembly	1
Chylomicron remodeling	1
Regulation of BACH1 activity	1
Digestion of dietary carbohydrate	1
Plasma lipoprotein assembly	1
Postmitotic nuclear pore complex (NPC) reformation	1
Defective TPR may confer susceptibility towards thyroid papillary carcinoma (TPC)	1
Transport of the SLBP independent Mature mRNA	1
SUMOylation of SUMOylation proteins	1
Transport of the SLBP Dependant Mature mRNA	1
Regulation of Glucokinase by Glucokinase Regulatory Protein	1
Nuclear import of Rev protein	1
Nuclear Pore Complex (NPC) Disassembly	1
SUMOylation of ubiquitinylation proteins	1
Vpr-mediated nuclear import of PICs	1
Rev-mediated nuclear export of HIV RNA	1

RHO GTPases activate IQGAPs	2
NGF-stimulated transcription	2
RHOQ GTPase cycle	2
RHOC GTPase cycle	2
Nuclear Events (kinase and transcription factor activation)	2
Signaling by NTRK1 (TRKA)	2
RHOA GTPase cycle	2
CDC42 GTPase cycle	2
Signaling by NTRKs	2
RAC1 GTPase cycle	2
Condensation of Prometaphase Chromosomes	1
RHO GTPase Effectors	2
Nephrin family interactions	1
RHO GTPase cycle	2
RHOV GTPase cycle	1
Signaling by high-kinase activity BRAF mutants	1
RHOU GTPase cycle	1
Heme biosynthesis	1
MAP2K and MAPK activation	1
Signaling by RAF1 mutants	1
Syndecan interactions	5
Integrin cell surface interactions	6
Signaling by Receptor Tyrosine Kinases	13
Non-integrin membrane-ECM interactions	5
Extracellular matrix organization	9
MET activates PTK2 signaling	4
Insulin receptor signalling cascade	5
ECM proteoglycans	5
Signaling by MET	5
Collagen chain trimerization	4
Degradation of the extracellular matrix	6

MET promotes cell motility	4
Platelet Adhesion to exposed collagen	3
Constitutive Signaling by Aberrant PI3K in Cancer	5
Signaling by Insulin receptor	5
PI3K Cascade	4
IRS-mediated signalling	4
PI3K/AKT Signaling in Cancer	5
Assembly of collagen fibrils and other multimeric structures	4
Signaling by FGFR1 amplification mutants	2
Insulin-like Growth Factor-2 mRNA Binding Proteins (IGF2BPs/IMPs/VICKZs) bind RNA	5
Downregulation of TGF-beta receptor signaling	4
RUNX3 regulates RUNX1-mediated transcription	2
Signaling by PDGF	5
TGFBR1 KD Mutants in Cancer	2
GRB7 events in ERBB2 signaling	2
Loss of Function of TGFBR1 in Cancer	2
SMAD2/3 Phosphorylation Motif Mutants in Cancer	2
TGF-beta receptor signaling activates SMADs	4
Loss of Function of SMAD2/3 in Cancer	2
Signaling by TGF-beta Receptor Complex in Cancer	2
Nef and signal transduction	2
The role of Nef in HIV-1 replication and disease pathogenesis	3
Non-integrin membrane-ECM interactions	4
Constitutive Signaling by Aberrant PI3K in Cancer	5
ALK mutants bind TKIs	2
Downstream signal transduction	3
Downregulation of SMAD2/3:SMAD4 transcriptional activity	3
Signaling by TGF-beta Receptor Complex	5
Degradation of the extracellular matrix	6
Cell surface interactions at the vascular wall	9
Antigen activates B Cell Receptor (BCR) leading to generation of second messengers	6

FOXO-mediated transcription of oxidative stress, metabolic and neuronal genes	4
Plasma lipoprotein remodeling	4
Plasma lipoprotein assembly, remodeling, and clearance	5
RUNX2 regulates genes involved in differentiation of myeloid cells	2
Scavenging of heme from plasma	5
RUNX1 regulates transcription of genes involved in BCR signaling	2
Assembly of active LPL and LIPC lipase complexes	3
CD22 mediated BCR regulation	4
Signaling by the B Cell Receptor (BCR)	6
NR1H2 & NR1H3 regulate gene expression linked to gluconeogenesis	2
RUNX1 regulates transcription of genes involved in differentiation of myeloid cells	2
NR1H2 and NR1H3-mediated signaling	4
Hemostasis	13
Erythropoietin activates Phospholipase C gamma (PLCG)	2
Chylomicron assembly	2
Generation of second messenger molecules	3
Aflatoxin activation and detoxification	3
NR1H2 & NR1H3 regulate gene expression to control bile acid homeostasis	2
Formation of a pool of free 40S subunits	60
Nonsense Mediated Decay (NMD) independent of the Exon Junction Complex (EJC)	55
SARS-CoV-1 modulates host translation machinery	21
GTP hydrolysis and joining of the 60S ribosomal subunit	60
L13a-mediated translational silencing of Ceruloplasmin expression	60
Viral mRNA Translation	55
SRP-dependent cotranslational protein targeting to membrane	55
Translation initiation complex formation	24
Ribosomal scanning and start codon recognition	24
Eukaryotic Translation Elongation	58
Major pathway of rRNA processing in the nucleolus and cytosol	56
Nonsense-Mediated Decay (NMD)	55
Peptide chain elongation	56

Nonsense Mediated Decay (NMD) enhanced by the Exon Junction Complex (EJC)	55
Eukaryotic Translation Initiation	60
Eukaryotic Translation Termination	55
Cap-dependent Translation Initiation	60
rRNA processing in the nucleus and cytosol	57
Cellular response to starvation	56
rRNA processing	58
Interleukin-6 signaling	6
FGFR1 mutant receptor activation	7
Interleukin-6 family signaling	6
Signaling by FGFR1 in disease	7
Signaling by FGFR in disease	8
Signaling by SCF-KIT	6
Transcriptional Regulation by TP53	20
Signaling by cytosolic FGFR1 fusion mutants	4
Interleukin-27 signaling	3
Signaling by phosphorylated juxtamembrane, extracellular and kinase domain KIT mutants	4
TP53 regulates transcription of additional cell cycle genes whose exact role in the p53 pathway remain uncertai	4
Signaling by KIT in disease	4
Interleukin-35 Signalling	3
Signaling by FGFR1 amplification mutants	2
Signaling by NOTCH1	6
TP53 Regulates Transcription of DNA Repair Genes	6
Signaling by PDGFRA transmembrane, juxtamembrane and kinase domain mutants	3
Signaling by PDGFRA extracellular domain mutants	3
rRNA processing in the mitochondrion	4
IRS-related events triggered by IGF1R	5
Attenuation phase	11
HSF1-dependent transactivation	11
Cellular response to heat stress	12
HSF1 activation	9

Regulation of HSF1-mediated heat shock response	10
Cellular responses to stimuli	17
Metallothioneins bind metals	4
Cellular responses to stress	13
Response to metal ions	4
HSP90 chaperone cycle for steroid hormone receptors (SHR) in the presence of ligand	5
Signaling by Nuclear Receptors	6
TFAP2A acts as a transcriptional repressor during retinoic acid induced cell differentiation	2
Uptake and function of diphtheria toxin	2
Estrogen-dependent gene expression	4
Binding and Uptake of Ligands by Scavenger Receptors	4
NR1H2 & NR1H3 regulate gene expression to control bile acid homeostasis	2
Scavenging by Class F Receptors	2
Sema3A PAK dependent Axon repulsion	2
Chaperone Mediated Autophagy	2
Classical antibody-mediated complement activation	3
tRNA processing in the mitochondrion	4
Interleukin-2 family signaling	4
Activation of RAC1	3
Signaling by Interleukins	10
Downstream signal transduction	3
rRNA processing in the mitochondrion	3
Complex I biogenesis	3
Cytokine Signaling in Immune system	10
Interleukin-15 signaling	2
Respiratory electron transport, ATP synthesis by chemiosmotic coupling, and heat production by uncoupling prc	4
Signaling by PDGF	3
RUNX1 and FOXP3 control the development of regulatory T lymphocytes (Tregs)	2
Gene and protein expression by JAK-STAT signaling after Interleukin-12 stimulation	3
Interleukin-12 signaling	3
tRNA processing	4

Nephrin family interactions	2
Metabolism of RNA	8
Interleukin-12 family signaling	3
TP53 regulates transcription of additional cell cycle genes whose exact role in the p53 pathway remain uncertai	2
Interleukin receptor SHC signaling	2
tRNA processing in the mitochondrion	12
rRNA processing in the mitochondrion	11
Metallothioneins bind metals	7
Response to metal ions	7
tRNA processing	13
Respiratory electron transport	10
Metabolism of Angiotensinogen to Angiotensins	5
Chylomicron assembly	4
Respiratory electron transport, ATP synthesis by chemiosmotic coupling, and heat production by uncoupling prc	11
Complex I biogenesis	6
Intestinal infectious diseases	2
Plasma lipoprotein assembly	4
RUNX3 regulates RUNX1-mediated transcription	2
rRNA processing	12
Packaging Of Telomere Ends	4
RNA Polymerase I Promoter Opening	4
Chylomicron remodeling	3
Plasma lipoprotein remodeling	5
DNA methylation	4
The citric acid (TCA) cycle and respiratory electron transport	11
RUNX2 regulates genes involved in cell migration	3
RUNX2 regulates genes involved in differentiation of myeloid cells	2
NFE2L2 regulating TCA cycle genes	2
Interleukin-4 and Interleukin-13 signaling	6
RUNX2 regulates chondrocyte maturation	2
Transcriptional regulation by RUNX2	5

RUNX3 Regulates Immune Response and Cell Migration	2
RUNX1 regulates transcription of genes involved in differentiation of myeloid cells	2
AKT phosphorylates targets in the nucleus	2
Chylomicron assembly	2
Regulation of localization of FOXO transcription factors	2
Plasma lipoprotein remodeling	3
Chylomicron remodeling	2
YAP1- and WWTR1 (TAZ)-stimulated gene expression	2
Retinoid metabolism and transport	3
Regulation of RUNX2 expression and activity	3
Estrogen-dependent nuclear events downstream of ESR-membrane signaling	2
Constitutive Signaling by AKT1 E17K in Cancer	2
Metabolism of fat-soluble vitamins	3
Plasma lipoprotein assembly	2
Nonsense Mediated Decay (NMD) independent of the Exon Junction Complex (EJC)	53
Formation of a pool of free 40S subunits	53
Viral mRNA Translation	53
SRP-dependent cotranslational protein targeting to membrane	53
GTP hydrolysis and joining of the 60S ribosomal subunit	53
L13a-mediated translational silencing of Ceruloplasmin expression	53
Translation initiation complex formation	23
Ribosomal scanning and start codon recognition	23
Major pathway of rRNA processing in the nucleolus and cytosol	53
Nonsense-Mediated Decay (NMD)	53
Peptide chain elongation	53
Nonsense Mediated Decay (NMD) enhanced by the Exon Junction Complex (EJC)	53
Eukaryotic Translation Initiation	53
Eukaryotic Translation Termination	53
Cap-dependent Translation Initiation	53
rRNA processing in the nucleus and cytosol	53
Eukaryotic Translation Elongation	53

SARS-CoV-1 modulates host translation machinery	23
Formation of the ternary complex, and subsequently, the 43S complex	23
Activation of the mRNA upon binding of the cap-binding complex and eIFs, and subsequent binding to 43S	23
Regulation of CDH11 mRNA translation by microRNAs	4
Regulation of CDH11 Expression and Function	5
Regulation of Expression and Function of Type II Classical Cadherins	5
Regulation of Homotypic Cell-Cell Adhesion	5
Regulation of CDH11 gene transcription	3
Adherens junctions interactions	5
Collagen chain trimerization	4
MET promotes cell motility	4
Integrin cell surface interactions	5
Signaling by MET	5
Cell-cell junction organization	5
GRB7 events in ERBB2 signaling	2
Syndecan interactions	3
Cell-Cell communication	6
Assembly of collagen fibrils and other multimeric structures	4
Collagen degradation	4
MET activates PTK2 signaling	3
Signaling by PDGF	4
RUNX3 regulates YAP1-mediated transcription	2
Defective factor VIII causes hemophilia A	2
GRB7 events in ERBB2 signaling	2
Downregulation of ERBB2:ERBB3 signaling	2
ERBB2 Activates PTK6 Signaling	2
ERBB2 Regulates Cell Motility	2
PI3K events in ERBB2 signaling	2
Signaling by ERBB2 TMD/JMD mutants	2
SHC1 events in ERBB2 signaling	2
Signaling by ERBB2 KD Mutants	2

Signaling by ERBB2 in Cancer	2
Downregulation of ERBB2 signaling	2
TGFBR1 KD Mutants in Cancer	1
SMAD2/3 Phosphorylation Motif Mutants in Cancer	1
Loss of Function of TGFBR1 in Cancer	1
Loss of Function of SMAD2/3 in Cancer	1
RHOT2 GTPase cycle	1
Signaling by ERBB2	2
Signaling by TGF-beta Receptor Complex in Cancer	1
Miro GTPase Cycle	1
Signaling by PTK6	2
Signaling by Non-Receptor Tyrosine Kinases	2
Peptide chain elongation	85
Eukaryotic Translation Elongation	87
Nonsense Mediated Decay (NMD) independent of the Exon Junction Complex (EJC)	83
Formation of a pool of free 40S subunits	87
Viral mRNA Translation	83
GTP hydrolysis and joining of the 60S ribosomal subunit	87
L13a-mediated translational silencing of Ceruloplasmin expression	87
SRP-dependent cotranslational protein targeting to membrane	85
Translation initiation complex formation	37
Ribosomal scanning and start codon recognition	37
Major pathway of rRNA processing in the nucleolus and cytosol	84
Nonsense-Mediated Decay (NMD)	84
Nonsense Mediated Decay (NMD) enhanced by the Exon Junction Complex (EJC)	84
SARS-CoV-1 modulates host translation machinery	34
Eukaryotic Translation Initiation	87
Eukaryotic Translation Termination	83
Cap-dependent Translation Initiation	87
rRNA processing in the nucleus and cytosol	84
rRNA processing	93

Translation	94
Interleukin-4 and Interleukin-13 signaling	8
Signaling by Interleukins	13
rRNA processing in the mitochondrion	4
tRNA processing in the mitochondrion	4
Cytokine Signaling in Immune system	14
Estrogen-dependent nuclear events downstream of ESR-membrane signaling	3
Response of EIF2AK1 (HRI) to heme deficiency	3
Interleukin-17 signaling	4
Interleukin-10 signaling	4
Immune System	22
Chemokine receptors bind chemokines	3
RUNX1 and FOXP3 control the development of regulatory T lymphocytes (Tregs)	2
MAP kinase activation	3
TFAP2 (AP-2) family regulates transcription of growth factors and their receptors	2
Drug resistance of KIT mutants	1
KIT mutants bind TKIs	1
Sunitinib-resistant KIT mutants	1
Masitinib-resistant KIT mutants	1
Nilotinib-resistant KIT mutants	1
Sorafenib-resistant KIT mutants	1
Cytokine Signaling in Immune system	23
Interferon Signaling	13
Interferon gamma signaling	9
Interferon alpha/beta signaling	7
Alpha-defensins	3
Immune System	32
Meiotic synapsis	4
Meiosis	4
Signaling by Interleukins	11
Defensins	3

RUNX1 regulates transcription of genes involved in differentiation of HSCs	4
Interleukin-6 signaling	2
Assembly of the pre-replicative complex	4
Signaling by NOTCH	6
HDACs deacetylate histones	3
DNA Replication Pre-Initiation	4
Deubiquitination	6
Other interleukin signaling	2
TCF dependent signaling in response to WNT	5
Reproduction	4
Cytokine Signaling in Immune system	27
Metallothioneins bind metals	6
Immune System	42
Response to metal ions	6
NF-kB is activated and signals survival	5
Interferon Signaling	13
Antigen processing-Cross presentation	9
Interferon alpha/beta signaling	9
p75NTR signals via NF-kB	5
ER-Phagosome pathway	8
Signaling by Interleukins	17
Modulation by Mtb of host immune system	4
SRP-dependent cotranslational protein targeting to membrane	8
Pexophagy	4
Amyloid fiber formation	7
Peptide chain elongation	7
p75NTR recruits signalling complexes	4
Nonsense Mediated Decay (NMD) independent of the Exon Junction Complex (EJC)	7
Eukaryotic Translation Elongation	7
Cellular responses to stimuli	20
Insulin-like Growth Factor-2 mRNA Binding Proteins (IGF2BPs/IMPs/VICKZs) bind RNA	5

Nuclear Receptor transcription pathway	11
Metabolism of RNA	45
Signaling by TGF-beta Receptor Complex	12
TGF-beta receptor signaling activates SMADs	8
Downregulation of TGF-beta receptor signaling	6
SUMOylation	16
SUMO E3 ligases SUMOylate target proteins	15
Chromatin organization	18
Chromatin modifying enzymes	18
Signaling by cytosolic FGFR1 fusion mutants	5
Nuclear Envelope Breakdown	8
ISG15 antiviral mechanism	9
Signaling by TGFB family members	12
DEx/H-box helicases activate type I IFN and inflammatory cytokines production	3
Processing of Capped Intron-Containing Pre-mRNA	19
Diseases of signal transduction by growth factor receptors and second messengers	28
FASTK family proteins regulate processing and stability of mitochondrial RNAs	4
Signaling by FLT3 fusion proteins	5
Cohesin Loading onto Chromatin	3
Nonsense Mediated Decay (NMD) independent of the Exon Junction Complex (EJC)	27
Formation of a pool of free 40S subunits	27
Viral mRNA Translation	27
SRP-dependent cotranslational protein targeting to membrane	27
GTP hydrolysis and joining of the 60S ribosomal subunit	27
L13a-mediated translational silencing of Ceruloplasmin expression	27
Nonsense-Mediated Decay (NMD)	27
Peptide chain elongation	27
Nonsense Mediated Decay (NMD) enhanced by the Exon Junction Complex (EJC)	27
Eukaryotic Translation Initiation	27
Eukaryotic Translation Termination	27
Cap-dependent Translation Initiation	27

Eukaryotic Translation Elongation	27
Major pathway of rRNA processing in the nucleolus and cytosol	27
SARS-CoV-1 modulates host translation machinery	10
Selenocysteine synthesis	27
Translation	27
Response of EIF2AK4 (GCN2) to amino acid deficiency	27
Regulation of expression of SLITs and ROBOs	27
rRNA processing in the nucleus and cytosol	27
Chromatin organization	48
Chromatin modifying enzymes	48
Signaling by Rho GTPases	98
Signaling by Rho GTPases, Miro GTPases and RHOBTB3	98
Diseases of signal transduction by growth factor receptors and second messengers	73
Regulation of MECP2 expression and activity	15
RHO GTPase cycle	67
RAC1 GTPase cycle	34
Oncogenic MAPK signaling	21
Loss of MECP2 binding ability to the NCoR/SMRT complex	6
Disorders of Developmental Biology	8
Loss of function of MECP2 in Rett syndrome	8
Pervasive developmental disorders	8
Disorders of Nervous System Development	8
Signaling by BRAF and RAF1 fusions	17
Circadian Clock	21
Signaling by TGF-beta Receptor Complex	21
RUNX1 regulates transcription of genes involved in differentiation of myeloid cells	6
MAPK family signaling cascades	50
Downregulation of TGF-beta receptor signaling	9
Regulation of MECP2 expression and activity	11
Transcriptional Regulation by MECP2	17
Chromatin organization	30

Chromatin modifying enzymes	30
MAP3K8 (TPL2)-dependent MAPK1/3 activation	6
Post-transcriptional silencing by small RNAs	4
Nuclear Receptor transcription pathway	13
MyD88 cascade initiated on plasma membrane	15
Toll Like Receptor 5 (TLR5) Cascade	15
Toll Like Receptor 10 (TLR10) Cascade	15
Notch-HLH transcription pathway	7
NOTCH1 Intracellular Domain Regulates Transcription	10
Constitutive Signaling by NOTCH1 PEST Domain Mutants	11
Signaling by NOTCH1 PEST Domain Mutants in Cancer	11
Constitutive Signaling by NOTCH1 HD+PEST Domain Mutants	11
Signaling by NOTCH1 HD+PEST Domain Mutants in Cancer	11
Signaling by NOTCH1 in Cancer	11
Antigen processing: Ubiquitination & Proteasome degradation	30
RUNX1 interacts with co-factors whose precise effect on RUNX1 targets is not known	8
TRAF6 mediated induction of NFkB and MAP kinases upon TLR7/8 or 9 activation	15

#Entities tota	Entities ratio	Entities pValue	Entities FDR	#Reactions foun	#Reactions tota
47	0.003085609243697479	1.1102230246251565E-1	4.2521541843143495E-1	4	5
59	0.003873424369747899	1.1102230246251565E-1	4.2521541843143495E-1	5	8
43	0.002823004201680672	5.551115123125783E-16	1.4155343563970746E-1	4	7
135	0.008862920168067227	2.2870594307278225E-1	3.6181058149509226E-1	16	29
113	0.00741859243697479	2.3647750424515834E-1	3.6181058149509226E-1	7	14
40	0.002626050420168067	2.6805498135606953E-6	3.404298263222083E-4	1	6
45	0.002954306722689075	5.228237367815858E-6	5.698778730919285E-4	1	3
102	0.006696428571428571	6.782928586468451E-6	6.443782157145028E-4	11	86
30	0.001969537815126050	1.1353777531919462E-5	9.650710902131543E-4	2	12
83	0.005449054621848739	1.63385499376556E-5	0.0012307454776356863	4	34
56	0.003676470588235294	1.7836890980227338E-5	0.0012307454776356863	5	31
86	0.005646008403361344	2.0462929222975212E-5	0.0012891645410474384	2	2
1008	0.0661764705882353	2.695213141534758E-5	0.0015632236220901596	62	455
1026	0.06735819327731092	3.573731543560221E-5	0.0018392364235449987	62	486
168	0.011029411764705883	3.606345928519605E-5	0.0018392364235449987	5	33
153	0.010044642857142858	6.847080344394296E-4	0.03218127761865319	11	31
76	0.004989495798319327	8.376602011438417E-4	0.037694709051472874	22	22
23	0.001509978991596638	0.0014313056857331308	0.05725222742932523	15	19
23	0.001509978991596638	0.0014313056857331308	0.05725222742932523	5	12
89	0.00584296218487395	0.0016735753430978662	0.06359586303771891	7	33
41	0.002691701680672269	1.1102230246251565E-1	1.9984014443252818E-1	3	3
101	0.00663077731092437	1.1102230246251565E-1	1.9984014443252818E-1	1	1
106	0.006959033613445379	1.1102230246251565E-1	1.9984014443252818E-1	2	2
114	0.007484243697478992	1.1102230246251565E-1	1.9984014443252818E-1	2	2
119	0.0078125	1.1102230246251565E-1	1.9984014443252818E-1	5	5
120	0.007878151260504201	1.1102230246251565E-1	1.9984014443252818E-1	3	3
120	0.007878151260504201	1.1102230246251565E-1	1.9984014443252818E-1	3	3
62	0.004070378151260504	1.1102230246251565E-1	1.9984014443252818E-1	2	2
64	0.004201680672268907	1.1102230246251565E-1	1.9984014443252818E-1	2	2

102	0.006696428571428571	1.1102230246251565E-1(	1.9984014443252818E-1!	8	9
189	0.012408088235294117	1.1102230246251565E-1(	1.9984014443252818E-1!	6	7
124	0.008140756302521009	1.1102230246251565E-1(	1.9984014443252818E-1!	5	6
97	0.006368172268907563	1.1102230246251565E-1(	1.9984014443252818E-1!	4	5
106	0.006959033613445379	1.1102230246251565E-1(	1.9984014443252818E-1!	4	5
124	0.008140756302521009	1.1102230246251565E-1(	1.9984014443252818E-1!	4	5
130	0.008534663865546218	1.1102230246251565E-1(	1.9984014443252818E-1!	13	21
130	0.008534663865546218	1.1102230246251565E-1(	1.9984014443252818E-1!	10	18
207	0.013589810924369748	1.1102230246251565E-1(	1.9984014443252818E-1!	8	15
176	0.011554621848739496	1.1102230246251565E-1(	1.9984014443252818E-1!	12	28
247	0.016215861344537816	1.1102230246251565E-1(	1.9984014443252818E-1!	8	21
194	0.012736344537815126	0.004316062414002952	0.06267873770221144	1	43
16	0.001050420168067226	0.008372531453416876	0.06267873770221144	1	6
23	0.001509978991596638	0.012016183342979825	0.06267873770221144	9	19
24	0.00157563025210084(	0.012535747540442288	0.06267873770221144	2	8
24	0.00157563025210084(	0.012535747540442288	0.06267873770221144	3	14
35	0.002297794117647059	0.018235194368245944	0.07294077747298378	3	3
70	0.004595588235294118	0.03617876584809887	0.08393085299838499	2	25
76	0.004989495798319327	0.03922581723775431	0.08393085299838499	3	22
91	0.00597426470588235!	0.04680659843233381	0.08393085299838499	3	22
106	0.006959033613445379	0.05433499021551502	0.08393085299838499	5	12
112	0.007352941176470588	0.057331747528357124	0.08393085299838499	1	7
803	0.05271796218487395	0.06294144752936404	0.08393085299838499	1	338
166	0.010898109243697478	0.08393085299838499	0.08393085299838499	11	108
168	0.011029411764705883	0.08490326094614076	0.08490326094614076	5	33
180	0.011817226890756302	0.09071876438695514	0.09071876438695514	1	33
1008	0.0661764705882353	0.09391726303366443	0.09391726303366443	7	455
1026	0.06735819327731092	0.09683605555379649	0.09683605555379649	7	486
274	0.01798844537815126	0.1351659448711181	0.1351659448711181	3	134
478	0.0313813025210084	0.22514081638184447	0.22514081638184447	2	10
667	0.04378939075630252	0.30107839848560347	0.30107839848560347	1	285

101	0.00663077731092437	1.1102230246251565E-16	8.881784197001252E-16	1	1
106	0.006959033613445379	1.1102230246251565E-16	8.881784197001252E-16	2	2
114	0.007484243697478992	1.1102230246251565E-16	8.881784197001252E-16	2	2
119	0.0078125	1.1102230246251565E-16	8.881784197001252E-16	5	5
120	0.007878151260504201	1.1102230246251565E-16	8.881784197001252E-16	3	3
62	0.0040703781512605045	1.1102230246251565E-16	8.881784197001252E-16	2	2
64	0.004201680672268907	1.1102230246251565E-16	8.881784197001252E-16	2	2
189	0.012408088235294117	1.1102230246251565E-16	8.881784197001252E-16	6	7
124	0.008140756302521009	1.1102230246251565E-16	8.881784197001252E-16	5	6
97	0.006368172268907563	1.1102230246251565E-16	8.881784197001252E-16	4	5
124	0.008140756302521009	1.1102230246251565E-16	8.881784197001252E-16	4	5
120	0.007878151260504201	1.1102230246251565E-16	8.881784197001252E-16	2	3
106	0.006959033613445379	1.1102230246251565E-16	8.881784197001252E-16	3	5
130	0.008534663865546218	1.1102230246251565E-16	8.881784197001252E-16	12	21
130	0.008534663865546218	1.1102230246251565E-16	8.881784197001252E-16	10	18
207	0.013589810924369748	1.1102230246251565E-16	8.881784197001252E-16	7	15
102	0.006696428571428571	1.1102230246251565E-16	8.881784197001252E-16	4	9
41	0.002691701680672269	1.1102230246251565E-16	8.881784197001252E-16	1	3
54	0.0035451680672268907	1.1102230246251565E-16	8.881784197001252E-16	1	3
62	0.0040703781512605045	1.1102230246251565E-16	8.881784197001252E-16	2	6
160	0.01376580917147036	0.02734212084079546	0.05468424168159092	1	43
726	0.06246235911554676	0.12102317192481404	0.12102317192481404	1	338
40	0.0026260504201680674	2.220446049250313E-16	5.728750807065808E-14	1	6
45	0.0029543067226890755	6.661338147750939E-16	8.593126210598712E-14	1	3
184	0.01207983193277311	1.9231191084045918E-9	1.653882433227949E-7	1	54
153	0.010044642857142858	4.7814752157648854E-9	3.0601441380895267E-7	12	31
118	0.0077468487394957986	7.96211452325224E-9	4.060678406858642E-7	9	19
247	0.016215861344537816	3.842135520848444E-8	1.652118273964831E-6	1	21
238	0.015625	2.8476866109627963E-7	1.0251671799466067E-5	12	67
59	0.0038734243697478993	1.2009703742266176E-5	3.843105197525176E-4	6	13
16	0.0010504201680672268	7.395301409474087E-5	0.0020706843946527442	6	27

21	0.00137867647058823[illegible]	1.6427901218096785E-4	0.004106975304524196	6	31
4	2.626050420168067E-4	1.889394591271243E-4	0.004345607559923859	2	2
830	0.054490546218487396	7.196524406154481E-4	0.01511270125292441	10	189
10	6.565126050420169E-4	0.0011585043450526022	0.022011582555999443	2	8
100	0.006565126050420168	0.0015581920488317857	0.028047456878972143	4	77
2	1.3130252100840336E-[illegible]	0.00979999959463096	0.15489086738469426	1	1
91	0.00597426470588235[illegible]	0.010457534521384648	0.15489086738469426	2	22
32	0.00210084033613445[illegible]	0.011063633384621019	0.15489086738469426	18	19
32	0.00210084033613445[illegible]	0.011063633384621019	0.15489086738469426	2	26
36	0.00236344537815126[illegible]	0.013826735734304374	0.1659208288116525	2	22
36	0.00236344537815126[illegible]	0.013826735734304374	0.1659208288116525	1	14
30	0.00196953781512605[illegible]	3.4747924309341816E-4	0.03996011295574309	1	12
56	0.003676470588235294	0.0011943626197370438	0.0680786693250115	3	31
102	0.006696428571428571	0.0038679626527018396	0.10564931591189364	6	86
8	5.252100840336134E-4	0.007327891883032289	0.10564931591189364	1	5
14	9.191176470588235E-4	0.012791054243784683	0.10564931591189364	3	5
17	0.00111607142857142[illegible]	0.015512153477156843	0.10564931591189364	2	3
20	0.00131302521008403[illegible]	0.018226286757706656	0.10564931591189364	3	8
27	0.00177258403361344[illegible]	0.02453226701296818	0.10564931591189364	1	13
30	0.00196953781512605[illegible]	0.027223299158463443	0.10564931591189364	3	19
32	0.00210084033613445[illegible]	0.02901348910053636	0.10564931591189364	6	10
36	0.00236344537815126[illegible]	0.03258469516358142	0.10564931591189364	1	1
39	0.00256039915966386[illegible]	0.03525509160800033	0.10564931591189364	3	3
39	0.00256039915966386[illegible]	0.03525509160800033	0.10564931591189364	2	5
40	0.00262605042016806[illegible]	0.03614370161273861	0.10564931591189364	3	3
40	0.00262605042016806[illegible]	0.03614370161273861	0.10564931591189364	1	5
41	0.002691701680672269	0.037031551547120456	0.10564931591189364	2	7
42	0.00275735294117647[illegible]	0.037918642011301795	0.10564931591189364	2	2
43	0.002823004201680672	0.03880497360499913	0.10564931591189364	1	3
44	0.002888655462184874	0.03969054692749985	0.10564931591189364	1	2
45	0.00295430672268907[illegible]	0.040575362577655705	0.10564931591189364	2	10

36	0.0023634453781512603	1.1638249161061154E-4	0.005692140048279004	5	5
56	0.003676470588235294	2.8038578769462585E-4	0.005692140048279004	3	37
63	0.004136029411764706	3.54319372573797E-4	0.005692140048279004	1	5
79	0.0051864495798319326	5.551942888148975E-4	0.005692140048279004	1	6
80	0.005252100840336135	5.692140048279004E-4	0.005692140048279004	3	48
143	0.00938813025210084	0.0017937654260251623	0.01202503950530065	3	102
154	0.01011029411764706	0.002075325432909114	0.01202503950530065	1	6
159	0.010438550420168067	0.002209846984177699	0.01202503950530065	1	6
166	0.010898109243697478	0.00240500790106013	0.01202503950530065	3	164
191	0.012539390756302522	0.0031665174317114797	0.0158325871585574	1	6
15	9.847689075630252E-4	0.006873050579187745	0.026800924995571074	4	4
325	0.021336659663865547	0.00890175704824181	0.026800924995571074	5	113
25	0.001641281512605042	0.011432555174660797	0.026800924995571074	1	13
460	0.030199579831932773	0.017309587302572038	0.026800924995571074	10	91
39	0.0025603991596638656	0.017785711572062435	0.026800924995571074	1	2
44	0.002888655462184874	0.020046198500919044	0.026800924995571074	4	6
44	0.002888655462184874	0.020046198500919044	0.026800924995571074	1	4
46	0.0030199579831932774	0.0209491438918437	0.026800924995571074	1	20
49	0.003216911764705882	0.0223022248244088	0.026800924995571074	8	12
49	0.003216911764705882	0.0223022248244088	0.026800924995571074	4	7
29	0.0019038865546218487	2.0294880809235138E-7	6.0884642427705415E-5	3	15
86	0.005646008403361344	2.20186562593927E-6	2.3830277393610544E-4	34	55
623	0.04090073529411765	2.3830277393610544E-6	2.3830277393610544E-4	109	746
61	0.004004726890756302	7.511938077242597E-6	5.633953557931948E-4	5	22
328	0.02153361344537815	1.2157484056030121E-5	6.325781175375855E-4	114	319
32	0.0021008403361344537	1.265156235075171E-5	6.325781175375855E-4	1	5
72	0.004726890756302521	1.659078227622679E-5	6.968128556015252E-4	6	25
79	0.0051864495798319326	2.57766683726679E-5	9.537367297887123E-4	11	23
88	0.005777310924369748	4.290491078806635E-5	0.001160846322540099	4	51
44	0.002888655462184874	4.3494908133823174E-5	0.001160846322540099	3	28
148	0.009716386554621849	4.6412400794171305E-5	0.001160846322540099	12	105

45	0.002954306722689075	4.74317171812455E-5	0.001160846322540099	2	12
16	0.001050420168067226	5.0471579240873865E-5	0.001160846322540099	4	6
96	0.006302521008403361	6.45506957653863E-5	0.0013555646110731123	1	2
102	0.006696428571428571	8.56814597010036E-5	0.001713629194020072	6	34
58	0.003807773109243697	1.2549489236679534E-4	0.002133413170235521	3	6
65	0.004267331932773109	1.935173238825083E-4	0.002788010014183717	3	9
124	0.008140756302521009	2.1147905081431162E-4	0.002788010014183717	1	21
67	0.004398634453781513	2.1704668331590238E-4	0.002788010014183717	16	26
5	3.2825630252100843E-4	2.2791634406427175E-4	0.002788010014183717	5	5
13	8.534663865546219E-4	1.2406503931972068E-6	9.044341366407638E-4	2	3
28	0.001838235294117647	6.535146928491331E-4	0.23787934819708445	25	28
4	2.626050420168067E-4	0.0014457104737788384	0.35130764512825774	2	2
70	0.004595588235294118	0.0029948925905332047	0.3865652054892208	5	31
6	3.939075630252101E-4	0.0031947537643737256	0.3865652054892208	1	1
6	3.939075630252101E-4	0.0031947537643737256	0.3865652054892208	1	1
7	4.5955882352941176E-4	0.004309473479495951	0.39216208663413155	2	2
7	4.5955882352941176E-4	0.004309473479495951	0.39216208663413155	1	1
50	0.003282563025210084	0.005263895596171353	0.4016411211559614	31	44
8	5.252100840336134E-4	0.005578348904943908	0.4016411211559614	2	2
9	5.908613445378151E-4	0.006997012041613293	0.4148047113830935	6	7
9	5.908613445378151E-4	0.006997012041613293	0.4148047113830935	1	5
29	0.0019038865546218487	0.007733743457443598	0.4148047113830935	4	20
61	0.004004726890756302	0.010387955538465166	0.4148047113830935	5	22
96	0.006302521008403361	0.01094818788939067	0.4148047113830935	1	2
12	7.878151260504202E-4	0.01210937964130332	0.4148047113830935	1	1
37	0.002429096638655462	0.014836051424955143	0.4148047113830935	4	16
38	0.002494747899159663	0.015913553146259307	0.4148047113830935	22	27
107	0.00702468487394958	0.01672031757641279	0.4148047113830935	81	100
148	0.009716386554621849	0.017153768550116633	0.4148047113830935	12	105
257	0.01687237394957983	7.755242581908295E-5	0.01823757703131257	6	65
103	0.006762079831932773	8.684560491101223E-5	0.01823757703131257	12	26

49	0.003216911764705882	3.9517984508319337E-4	0.05312247341700216	4	34
56	0.003676470588235294	6.498063750003391E-4	0.05312247341700216	5	31
102	0.006696428571428571	7.464356681263773E-4	0.05312247341700216	10	86
6	3.939075630252101E-4	8.246174362118719E-4	0.05312247341700216	2	2
106	0.006959033613445379	8.85374556950036E-4	0.05312247341700216	1	12
7	4.5955882352941176E-4	0.0011173644273869687	0.056702994696435916	2	4
30	0.0019695378151260504	0.0012326737977486069	0.056702994696435916	2	12
72	0.004726890756302521	0.001632742615613414	0.06857518985576339	3	4
189	0.012408088235294117	0.002063319298601596	0.07840613334686064	15	45
10	6.565126050420169E-4	0.0022498372527121058	0.0787443038449237	2	2
11	7.221638655462185E-4	0.0027101248070752204	0.08672399382640705	2	7
85	0.005580357142857143	0.0029630203501201935	0.0888906105036058	4	60
803	0.05271796218487395	0.0035885142372164003	0.09753773623408502	17	338
13	8.534663865546219E-4	0.0037514513936186544	0.09753773623408502	3	3
14	9.191176470588235E-4	0.0043313629100568685	0.10019401793893623	3	5
47	0.003085609243697479	0.0043562616495189665	0.10019401793893623	4	17
49	0.003216911764705882	0.004887735649699798	0.10395065296380324	4	18
15	9.847689075630252E-4	0.00495003109351444	0.10395065296380324	2	6
106	0.006959033613445379	1.1102230246251565E-1	2.7755575615628914E-1	2	2
101	0.00663077731092437	1.1102230246251565E-1	2.7755575615628914E-1	1	1
41	0.002691701680672269	1.1102230246251565E-1	2.7755575615628914E-1	3	3
120	0.007878151260504201	1.1102230246251565E-1	2.7755575615628914E-1	3	3
120	0.007878151260504201	1.1102230246251565E-1	2.7755575615628914E-1	3	3
114	0.007484243697478992	1.1102230246251565E-1	2.7755575615628914E-1	2	2
119	0.0078125	1.1102230246251565E-1	2.7755575615628914E-1	5	5
62	0.0040703781512605045	1.1102230246251565E-1	2.7755575615628914E-1	2	2
64	0.004201680672268907	1.1102230246251565E-1	2.7755575615628914E-1	2	2
102	0.006696428571428571	1.1102230246251565E-1	2.7755575615628914E-1	8	9
189	0.012408088235294117	1.1102230246251565E-1	2.7755575615628914E-1	6	7
124	0.008140756302521009	1.1102230246251565E-1	2.7755575615628914E-1	5	6
97	0.006368172268907563	1.1102230246251565E-1	2.7755575615628914E-1	4	5

124	0.008140756302521009	1.1102230246251565E-1(	2.7755575615628914E-1!	4	5
130	0.008534663865546218	1.1102230246251565E-1(	2.7755575615628914E-1!	13	21
106	0.006959033613445379	1.1102230246251565E-1(	2.7755575615628914E-1!	3	5
130	0.008534663865546218	1.1102230246251565E-1(	2.7755575615628914E-1!	10	18
207	0.013589810924369748	1.1102230246251565E-1(	2.7755575615628914E-1!	8	15
176	0.011554621848739496	1.1102230246251565E-1(	2.7755575615628914E-1!	12	28
247	0.016215861344537816	1.1102230246251565E-1(	2.7755575615628914E-1!	9	21
17	0.0011160714285714285	8.865186311712847E-7	8.723343330725442E-4	17	20
39	0.002560399159663865(	9.082510290747692E-6	0.004468595063047864	20	25
30	0.001969537815126050	2.1987462453187945E-5	0.007211887684645646	21	34
49	0.003216911764705882	3.849223199225982E-5	0.009469089070095915	30	35
82	0.005383403361344538	1.5238493931257757E-4	0.029867448105265204	37	99
51	0.003348214285714285!	3.866993143578634E-4	0.0634186875546896	10	39
484	0.03177521008403361	6.467109926990844E-4	0.09053953897787181	37	259
23	0.0015099789915966387	8.991714859790667E-4	0.1105980927754252	9	14
13	8.534663865546219E-4	0.0018297232232982186	0.15090152327415263	10	20
28	0.001838235294117647	0.0018402624789530808	0.15090152327415263	6	11
28	0.001838235294117647	0.0018402624789530808	0.15090152327415263	6	14
28	0.001838235294117647	0.0018402624789530808	0.15090152327415263	6	26
16	0.0010504201680672268	0.003277622668540481	0.24582170014053606	16	26
5	3.2825630252100843E-4	0.003879221141037781	0.27154547987264466	5	5
84	0.005514705882352941!	0.004714645271575835	0.2848028775711746	19	39
86	0.005646008403361344	0.0052706310896588615	0.2848028775711746	6	17
19	0.0012473739495798318	0.00527412736242916	0.2848028775711746	3	7
19	0.0012473739495798318	0.00527412736242916	0.2848028775711746	3	7
40	0.0026260504201680674	0.006474877538929835	0.3302187544854216	2	6
69	0.004529936974789916	0.009081878002131671	0.41014541958564255	7	12
47	0.003085609243697479	1.1102230246251565E-1(	6.5503158452884236E-1!	4	5
59	0.0038734243697478993	1.1102230246251565E-1(	6.5503158452884236E-1!	5	8
135	0.008862920168067227	1.1102230246251565E-1(	6.5503158452884236E-1!	16	29
43	0.002823004201680672	4.440892098500626E-16	1.9539925233402755E-1·	4	7

113	0.00741859243697479	3.9968028886505635E-1	1.3988810110276972E-12	7	14
1026	0.06735819327731092	4.939271214254859E-12	1.432388652133909E-10	47	486
16	0.00105042016806722	4.861887892282368E-8	1.215471973070592E-6	9	27
1008	0.0661764705882353	7.559247239274924E-8	1.6630343926404834E-6	38	455
21	0.00137867647058823	1.4318428431803198E-7	2.7205014020426077E-6	9	31
76	0.00498949579831932	6.532247529555946E-7	1.1104820800245108E-5	22	22
386	0.02534138655462185	1.6272873975198276E-4	0.00254936937746674	13	196
9	5.908613445378151E-4	1.820978126761963E-4	0.00254936937746674	2	7
10	6.565126050420169E-4	2.2450764722148353E-4	0.00291859941387928	1	5
153	0.01004464285714285	3.3002614294597166E-4	0.00396031371535166	4	66
168	0.01102941176470588	4.689383902454214E-4	0.00515832229269963	5	33
15	9.847689075630252E-4	5.017320802670477E-4	0.00551905288293752	2	6
16	0.00105042016806722	5.700870489048215E-4	0.00570087048904821	2	2
19	0.00124737394957983	8.006531453818022E-4	0.00720587830843622	2	6
23	0.00150997899159663	0.00116692217697511	0.00977068975192629	12	19
97	0.00636817226890756	0.00122133621899078	0.00977068975192629	2	2
45	0.00295430672268907	1.030254125011787E-5	0.00139260517586059	2	3
47	0.00308560924369747	1.2207268195507659E-5	0.00139260517586059	35	59
15	9.847689075630252E-4	1.3137784677930142E-5	0.00139260517586059	4	4
658	0.04319852941176470	1.7921070476822898E-5	0.00141576456766900	102	505
37	0.00242909663865546	1.8842531243001837E-4	0.01187079468309115	3	16
40	0.00262605042016806	2.3660760821975213E-4	0.01254020323564686	1	6
59	0.00387342436974789	7.301539458640649E-4	0.02956836612191771	5	13
1039	0.06821165966386554	7.581632338953259E-4	0.02956836612191771	107	745
16	0.00105042016806722	0.00105999383888388	0.03105415719093951	15	17
153	0.01004464285714285	0.00109205121427347	0.03105415719093951	9	31
70	0.00459558823529411	0.00119214602386874	0.03105415719093951	3	31
17	0.00111607142857142	0.00119439066118998	0.03105415719093951	3	20
73	0.00479254201680672	0.00134377521665429	0.0322506051997031	36	36
84	0.00551470588235294	0.00200163821229759	0.04403604067054711	41	56
184	0.01207983193277311	0.00213773800588712	0.0448924981236296	2	54

25	0.001641281512605042	0.0025445768438346894	0.0483469600328591	2	13
830	0.054490546218487396	0.002732998129703512	0.04919396633466322	18	189
96	0.006302521008403361	0.002915216612947913	0.04955868242011452	55	114
28	0.001838235294117647	0.003174035590286728	0.050784569444587646	3	14
29	0.0019038865546218487	0.0033984333809475853	0.05097650071421378	6	6
45	0.002954306722689075	1.0791711968494155E-1	9.259288868967985E-8	2	3
40	0.002626050420168067	4.736084857626111E-10	2.0317804039216014E-7	1	6
16	0.001050420168067226	3.226091760399896E-8	9.226622434743703E-6	15	27
21	0.001378676470588235	1.9979138465853197E-7	4.275535631692584E-5	15	31
184	0.01207983193277311	5.956812864127414E-5	0.010186149997657878	3	54
118	0.007746848739495798	9.97398244880765E-5	0.01426279490179494	9	19
26	0.001706932773109243	1.523318530646245E-4	0.017181459342060945	10	20
14	9.191176470588235E-4	1.6057438637440136E-4	0.017181459342060945	5	5
153	0.010044642857142858	1.8834343314377833E-4	0.01789262614865894	12	31
59	0.003873424369747899	9.863354668424584E-4	0.08383851468160897	6	13
3	1.9695378151260505E-4	0.0015336690947410636	0.11962618938980296	1	1
30	0.001969537815126050	0.002677361929544375	0.17513448825029998	16	19
4	2.626050420168067E-4	0.00269290253038601	0.17513448825029998	2	2
247	0.016215861344537816	0.002871057184431147	0.17513448825029998	2	21
33	0.002166491596638655	0.0037521306750158967	0.19886292577584253	2	2
33	0.002166491596638655	0.0037521306750158967	0.19886292577584253	1	2
17	0.001116071428571428	0.004281177923621704	0.20752043360003747	3	3
56	0.003676470588235294	0.0044801937641141265	0.20752043360003747	17	31
35	0.002297794117647059	0.004611565191111944	0.20752043360003747	7	7
238	0.015625	0.006072120415988147	0.24537632389316943	12	67
14	9.191176470588235E-4	4.415900991927568E-5	0.012850271886509224	7	7
6	3.939075630252101E-4	3.893804492295372E-4	0.03064559450920834	2	2
6	3.939075630252101E-4	3.893804492295372E-4	0.03064559450920834	2	4
211	0.013852415966386554	5.052538991812128E-4	0.03064559450920834	1	47
7	4.5955882352941176E-4	5.283723191242817E-4	0.03064559450920834	4	4
147	0.009650735294117647	6.848214488703874E-4	0.0328714295457786	81	84

10	6.565126050420169E-4	0.00106847328925197	0.04380740485933077	2	5
11	7.221638655462185E-4	0.001288911702597817	0.04640082129352141	1	7
12	7.878151260504202E-4	0.0015292372356274786	0.048935591540079315	4	4
14	9.191176470588235E-4	0.0020688020712664734	0.05378885385292831	5	5
14	9.191176470588235E-4	0.0020688020712664734	0.05378885385292831	3	5
56	0.003676470588235294	0.0024525364726945	0.058860875344667996	6	31
17	0.001116071428571428[illegible]	0.003022663752449861	0.06649860255389695	3	3
18	0.001181722689075630[illegible]	0.003378424999975538	0.06756849999951076	1	9
79	0.005186449579831932[illegible]	0.00637400679474831	0.11473212230546959	14	28
83	0.005449054621848739	0.0072937540940990475	0.11762992070379608	32	32
29	0.001903886554621848[illegible]	0.008480963885936554	0.11762992070379608	3	12
29	0.001903886554621848[illegible]	0.008480963885936554	0.11762992070379608	2	18
89	0.00584296218487395	0.00881401519554914	0.11762992070379608	14	34
30	0.001969537815126050[illegible]	0.009048455438753544	0.11762992070379608	10	19
101	0.00663077731092437	1.1102230246251565E-1[illegible]	1.3322676295501878E-1[illegible]	1	1
106	0.006959033613445379	1.1102230246251565E-1[illegible]	1.3322676295501878E-1[illegible]	2	2
114	0.007484243697478992	1.1102230246251565E-1[illegible]	1.3322676295501878E-1[illegible]	2	2
119	0.0078125	1.1102230246251565E-1[illegible]	1.3322676295501878E-1[illegible]	5	5
120	0.007878151260504201	1.1102230246251565E-1[illegible]	1.3322676295501878E-1[illegible]	3	3
120	0.007878151260504201	1.1102230246251565E-1[illegible]	1.3322676295501878E-1[illegible]	3	3
62	0.004070378151260504[illegible]	1.1102230246251565E-1[illegible]	1.3322676295501878E-1[illegible]	2	2
64	0.004201680672268907	1.1102230246251565E-1[illegible]	1.3322676295501878E-1[illegible]	2	2
189	0.012408088235294117	1.1102230246251565E-1[illegible]	1.3322676295501878E-1[illegible]	6	7
124	0.008140756302521009	1.1102230246251565E-1[illegible]	1.3322676295501878E-1[illegible]	5	6
97	0.006368172268907563	1.1102230246251565E-1[illegible]	1.3322676295501878E-1[illegible]	4	5
124	0.008140756302521009	1.1102230246251565E-1[illegible]	1.3322676295501878E-1[illegible]	4	5
130	0.008534663865546218	1.1102230246251565E-1[illegible]	1.3322676295501878E-1[illegible]	13	21
106	0.006959033613445379	1.1102230246251565E-1[illegible]	1.3322676295501878E-1[illegible]	3	5
130	0.008534663865546218	1.1102230246251565E-1[illegible]	1.3322676295501878E-1[illegible]	10	18
207	0.013589810924369748	1.1102230246251565E-1[illegible]	1.3322676295501878E-1[illegible]	7	15
102	0.006696428571428571	1.1102230246251565E-1[illegible]	1.3322676295501878E-1[illegible]	4	9

41	0.00269170168067226¢	1.1102230246251565E-1€	1.3322676295501878E-1!	1	3
54	0.0035451680672268907	1.1102230246251565E-1€	1.3322676295501878E-1!	1	3
66	0.004332983193277311	1.1102230246251565E-1€	1.3322676295501878E-1!	2	6
14	9.191176470588235E-4	3.8114729650207124E-6	0.0013462105460521068	4	4
35	0.002297794117647059	6.414427930478972E-6	0.0013462105460521068	25	28
39	0.002560399159663865€	1.0769684368416854E-5	0.0013462105460521068	25	33
39	0.002560399159663865€	1.0769684368416854E-5	0.0013462105460521068	25	33
12	7.878151260504202E-4	9.907341553672122E-5	0.009907341553672122	11	14
66	0.004332983193277311	1.281279484169895E-4	0.010634619718610128	27	49
44	0.002888655462184874	3.149496140860686E-4	0.021245853938455683	4	28
45	0.002954306722689075!	3.4267506352347876E-4	0.021245853938455683	3	12
86	0.005646008403361344	4.295008244203924E-4	0.02362254534312158	6	55
88	0.005777310924369748	4.7637267896472046E-4	0.023818633948236023	7	51
99	0.006499474789915967	8.064403059052649E-4	0.03628981376573692	27	54
6	3.939075630252101E-4	9.042337558768931E-4	0.03707358399095262	1	1
29	0.0019038865546218487	0.0012792411825054417	0.046388200975320815	1	15
165	0.010832457983193277	0.0013253771707234518	0.046388200975320815	28	93
67	0.004398634453781513	0.0014917520727303346	0.0472056344871723	16	26
69	0.004529936974789916	0.0016596644816050388	0.0472056344871723	11	34
32	0.0021008403361344537	0.0016920307475606355	0.0472056344871723	1	5
70	0.004595588235294118	0.0017483568328582333	0.0472056344871723	2	31
9	5.908613445378151E-4	0.0020059947151859214	0.050149867879648036	2	3
9	5.908613445378151E-4	0.0020059947151859214	0.050149867879648036	3	8
6	5.162178439301385E-4	6.109317711811002E-4	0.13012846726157434	1	1
14	0.001204508302503656€	0.003225896041702536	0.1838130357591805	2	8
16	0.001376580917147036′	0.0041813577333075225	0.1838130357591805	2	2
18	0.001548653531790415€	0.005251801021690872	0.1838130357591805	2	2
18	0.001548653531790415€	0.005251801021690872	0.1838130357591805	4	7
26	0.002236943990363933€	0.010628744300016701	0.2926848396402778	6	13
28	0.002409016605007313:	0.01223352097350161	0.2926848396402778	4	6
29	0.002495052912329003	0.013073219480426945	0.2926848396402778	8	17

30	0.00258108921965069[illegible]	0.013937373316203705	0.2926848396402778	14	62
32	0.00275316183429407[illegible]	0.01573780907422262	0.29901837241022977	4	14
6	5.162178439301385E-4	0.03500101358040475	0.42213929257592575	1	1
6	5.162178439301385E-4	0.03500101358040475	0.42213929257592575	1	1
7	6.022541512518283E-4	0.04071595091763025	0.42213929257592575	1	2
7	6.022541512518283E-4	0.04071595091763025	0.42213929257592575	1	2
7	6.022541512518283E-4	0.04071595091763025	0.42213929257592575	1	3
56	0.00481803321001462[illegible]	0.04402687691841878	0.42213929257592575	24	46
8	6.882904585735181E-4	0.04639753077704489	0.42213929257592575	2	7
8	6.882904585735181E-4	0.04639753077704489	0.42213929257592575	1	6
58	0.004990105824658006	0.046875045689903194	0.42213929257592575	2	53
58	0.004990105824658006	0.046875045689903194	0.42213929257592575	2	53
97	0.006368172268907563	1.1102230246251565E-1[illegible]	3.6637359812630166E-1[illegible]	5	5
102	0.006696428571428571	1.1102230246251565E-1[illegible]	3.6637359812630166E-1[illegible]	9	9
101	0.00663077731092437	1.1102230246251565E-1[illegible]	3.6637359812630166E-1[illegible]	1	1
106	0.006959033613445379	1.1102230246251565E-1[illegible]	3.6637359812630166E-1[illegible]	2	2
114	0.007484243697478992	1.1102230246251565E-1[illegible]	3.6637359812630166E-1[illegible]	2	2
120	0.007878151260504201	1.1102230246251565E-1[illegible]	3.6637359812630166E-1[illegible]	3	3
120	0.007878151260504201	1.1102230246251565E-1[illegible]	3.6637359812630166E-1[illegible]	3	3
119	0.0078125	1.1102230246251565E-1[illegible]	3.6637359812630166E-1[illegible]	5	5
62	0.00407037815126050[illegible]	1.1102230246251565E-1[illegible]	3.6637359812630166E-1[illegible]	2	2
64	0.004201680672268907	1.1102230246251565E-1[illegible]	3.6637359812630166E-1[illegible]	2	2
189	0.012408088235294117	1.1102230246251565E-1[illegible]	3.6637359812630166E-1[illegible]	6	7
124	0.008140756302521009	1.1102230246251565E-1[illegible]	3.6637359812630166E-1[illegible]	5	6
124	0.008140756302521009	1.1102230246251565E-1[illegible]	3.6637359812630166E-1[illegible]	4	5
41	0.002691701680672269	1.1102230246251565E-1[illegible]	3.6637359812630166E-1[illegible]	2	3
130	0.008534663865546218	1.1102230246251565E-1[illegible]	3.6637359812630166E-1[illegible]	13	21
106	0.006959033613445379	1.1102230246251565E-1[illegible]	3.6637359812630166E-1[illegible]	3	5
130	0.008534663865546218	1.1102230246251565E-1[illegible]	3.6637359812630166E-1[illegible]	10	18
207	0.013589810924369748	1.1102230246251565E-1[illegible]	3.6637359812630166E-1[illegible]	8	15
247	0.016215861344537816	1.1102230246251565E-1[illegible]	3.6637359812630166E-1[illegible]	9	21

339	0.02225577731092437	1.1102230246251565E-1(	3.6637359812630166E-1!	41	99
211	0.013852415966386554	4.782692832994151E-6	0.0012096865507613686	5	47
658	0.043198529411764705	6.109528034148326E-6	0.0012096865507613686	44	505
40	0.0026260504201680674	3.3865013135847555E-5	0.004470181733931877	1	6
45	0.002954306722689075!	5.3344499102214726E-5	0.005281105411119258	1	3
1039	0.06821165966386554	1.7248118078738717E-4	0.013626013282203586	56	745
29	0.0019038865546218487	3.152723942873026E-4	0.017655254080088945	3	12
29	0.0019038865546218487	3.152723942873026E-4	0.017655254080088945	3	20
78	0.005120798319327731	4.312307576248564E-4	0.021130307123617964	12	35
86	0.005646008403361344	6.204884880125494E-4	0.027301493472552174	1	15
2627	0.1724658613445378	0.0017837796181539023	0.053453777995797314	101	1664
57	0.003742121848739496	0.0021901528114338653	0.053453777995797314	4	19
17	0.0011160714285714285	0.002701889390561707	0.053453777995797314	3	20
69	0.004529936974789916	0.0037401877158940167	0.053453777995797314	11	32
21	0.0013786764705882354	0.004075868822725259	0.053453777995797314	2	18
1	6.565126050420168E-5	0.004454481499649776	0.053453777995797314	7	7
1	6.565126050420168E-5	0.004454481499649776	0.053453777995797314	3	3
1	6.565126050420168E-5	0.004454481499649776	0.053453777995797314	1	1
1	6.565126050420168E-5	0.004454481499649776	0.053453777995797314	1	1
1	6.565126050420168E-5	0.004454481499649776	0.053453777995797314	1	1
1	6.565126050420168E-5	0.004454481499649776	0.053453777995797314	1	1
1039	0.06821165966386554	7.765313525531781E-8	2.981729591056137E-5	138	745
322	0.021139705882352942	1.1647381215063035E-7	2.981729591056137E-5	23	79
177	0.011620273109243698	1.8458188918435425E-6	3.1378921161340223E-4	10	23
129	0.008469012605042016	1.8007658441843155E-5	0.002304980280555924	11	25
11	7.221638655462185E-4	4.9599772528718944E-5	0.005059176797929332	9	12
2627	0.1724658613445378	8.026085588974663E-5	0.0068221727506284635	196	1664
61	0.004004726890756302	6.12582511322679E-4	0.03920528072465146	6	6
91	0.005974264705882352!	0.002633125468417874	0.12373838477132804	10	15
658	0.043198529411764705	0.002689964886333218	0.12373838477132804	77	505
51	0.003348214285714285!	0.004129942772996831	0.16802799270110957	10	19

105	0.006893382352941176	0.0043697002764476744	0.16802799270110957	4	15
17	0.0011160714285714285	0.005191250298065664	0.16802799270110957	6	20
113	0.00741859243697479	0.005645340782641561	0.16802799270110957	3	23
258	0.016938025210084032	0.005692487298176174	0.16802799270110957	10	154
63	0.004136029411764706	0.00737900144319692	0.16802799270110957	5	5
131	0.00860031512605042	0.00937197635467324	0.16802799270110957	3	32
289	0.018973214285714284	0.00965501522109935	0.16802799270110957	8	77
24	0.0015756302521008404	0.010058665535187528	0.16802799270110957	5	17
215	0.014115021008403362	0.011388307612387738	0.16802799270110957	7	71
144	0.009453781512605041	0.012877027790604734	0.16802799270110957	10	43
1039	0.06821165966386554	1.616484723854228E-10	1.2317613595769217E-7	59	745
16	0.0010504201680672268	1.148424577657181E-9	3.3254291187390095E-7	13	27
2627	0.1724658613445378	1.309224062495673E-9	3.3254291187390095E-7	225	1664
21	0.0013786764705882354	5.724148266494922E-9	1.0875881706340351E-6	13	31
15	9.847689075630252E-4	5.253788826209416E-8	7.985759015838312E-6	3	4
322	0.021139705882352942	1.3209676508196821E-7	1.4106778221556482E-5	6	79
128	0.008403361344537815	1.40282719329754E-7	1.4106778221556482E-5	10	23
129	0.008469012605042016	1.4968838069062684E-7	1.4106778221556482E-5	3	25
19	0.0012473739495798318	1.679378359709105E-7	1.4106778221556482E-5	7	13
106	0.006959033613445379	4.232762548239677E-7	3.2168995366621544E-5	6	10
658	0.043198529411764705	7.513746268505983E-7	5.184484925269128E-5	40	505
11	7.221638655462185E-4	8.567869671516348E-7	5.3977578930552994E-5	2	6
119	0.0078125	9.990530560610722E-7	5.794507725154219E-5	5	5
13	8.534663865546219E-4	1.6553354655890473E-6	8.795488280477493E-5	6	13
89	0.00584296218487395	1.7590976560954985E-6	8.795488280477493E-5	8	33
97	0.006368172268907563	3.0849200750582284E-6	1.4499124352773674E-4	4	5
16	0.0010504201680672268	3.7437828720454647E-6	1.6472644637000045E-4	4	9
101	0.00663077731092437	4.010827935663208E-6	1.6845477329785474E-4	1	1
102	0.006696428571428571	4.2753478564083736E-6	1.7101391425633494E-4	4	9
1026	0.06735819327731092	5.3656812506464036E-6	1.8643794593131702E-4	57	486
13	8.406621831350233E-4	4.345244845316287E-5	0.02398367827652359	2	3

86	0.005561303673047077	5.127527140813548E-5	0.02398367827652359	2	2
841	0.05438437661665804	5.61678648162145E-5	0.02398367827652359	89	199
108	0.006983962752198655	8.919811471708172E-5	0.02854339670946615	26	100
51	0.003297982410760476	1.3830759195321285E-4	0.03531451934428431	15	44
28	0.00181065700982980[illegible]	1.844934848221902E-4	0.03531451934428431	11	28
193	0.012480600103466116	1.9297551554253722E-4	0.03531451934428431	52	141
184	0.01189860320744956	3.603227048017965E-4	0.05765163276828744	49	132
254	0.016425245732022763	5.089584239684752E-4	0.06514667826796483	29	85
254	0.016425245732022763	5.089584239684752E-4	0.06514667826796483	29	85
23	0.001487325400931195	5.962139274968692E-4	0.06916081558963683	10	14
65	0.00420331091567511[illegible]	6.820972987925389E-4	0.07184060246518154	7	13
83	0.005367304707708225	7.854560089578522E-4	0.07184060246518154	10	16
138	0.00892395240558717	7.894571699470498E-4	0.07184060246518154	31	125
7	4.526642524573202E-4	0.0011579260670836478	0.09842371570211006	5	5
299	0.019335230212105536	0.001284290685849676	0.10274325486797409	32	34
540	0.034919813760993276	0.0022511317703047817	0.1574521853808588	234	512
19	0.00122866011381272[illegible]	0.002371309698633084	0.1574521853808588	4	4
32	0.00206932229694774[illegible]	0.002522014650079063	0.1574521853808588	17	18
10	6.466632177961717E-4	0.0031684941348003592	0.1574521853808588	2	2
101	0.006531298499741335	1.1102230246251565E-1[illegible]	1.7763568394002505E-1[illegible]	1	1
106	0.006854630108639421	1.1102230246251565E-1[illegible]	1.7763568394002505E-1[illegible]	2	2
114	0.007371960682876358	1.1102230246251565E-1[illegible]	1.7763568394002505E-1[illegible]	2	2
119	0.007695292291774444	1.1102230246251565E-1[illegible]	1.7763568394002505E-1[illegible]	5	5
120	0.007759958613554061	1.1102230246251565E-1[illegible]	1.7763568394002505E-1[illegible]	3	3
120	0.007759958613554061	1.1102230246251565E-1[illegible]	1.7763568394002505E-1[illegible]	3	3
124	0.00801862390067253	1.1102230246251565E-1[illegible]	1.7763568394002505E-1[illegible]	5	6
97	0.006272633212622866	1.1102230246251565E-1[illegible]	1.7763568394002505E-1[illegible]	4	5
124	0.00801862390067253	1.1102230246251565E-1[illegible]	1.7763568394002505E-1[illegible]	4	5
130	0.008406621831350233	1.1102230246251565E-1[illegible]	1.7763568394002505E-1[illegible]	13	21
106	0.006854630108639421	1.1102230246251565E-1[illegible]	1.7763568394002505E-1[illegible]	3	5
130	0.008406621831350233	1.1102230246251565E-1[illegible]	1.7763568394002505E-1[illegible]	10	18

102	0.006595964821520952	1.1102230246251565E-16	1.7763568394002505E-15	4	9
189	0.012221934816347646	1.1102230246251565E-16	1.7763568394002505E-15	3	7
41	0.0026513191929643043	1.1102230246251565E-16	1.7763568394002505E-15	1	3
112	0.007242628039317123	1.1102230246251565E-16	1.7763568394002505E-15	2	7
339	0.02192188308329022	1.1102230246251565E-16	1.7763568394002505E-15	25	99
115	0.007436627004655976	1.1102230246251565E-16	1.7763568394002505E-15	4	16
183	0.011833936885669943	1.1102230246251565E-16	1.7763568394002505E-15	5	20
207	0.013385928608380756	1.1102230246251565E-16	1.7763568394002505E-15	3	15
254	0.016675420168067227	5.573020211979695 7E-8	4.842954564210355 5E-5	42	85
254	0.016675420168067227	5.5730202119796957E-8	4.8429545642103555E-5	42	85
708	0.04648109243697479	8.859649291981242E-8	5.129736940057139E-5	96	203
724	0.047531512605042014	2.4167763423843525E-7	1.048880932594809E-4	97	212
498	0.03269432773109244	5.499640598483069E-7	1.9083752876736249E-4	247	478
39	0.0025603991596638656	8.396624123774288E-7	2.4266243717707692E-4	13	14
460	0.030199579831932773	1.98439500465053E-6	4.921299611533314E-4	47	91
191	0.012539390756302522	1.563475492571964E-5	0.003392741818881162	3	6
93	0.006105567226890756	2.5495603545255818E-5	0.004920651484234373	44	46
8	5.252100840336134E-4	5.014086331178724E-5	0.0063035258947956585	1	1
16	0.0010504201680672268	5.0834886248352085E-5	0.0063035258947956585	5	5
16	0.0010504201680672268	5.0834886248352085E-5	0.0063035258947956585	5	5
16	0.0010504201680672268	5.0834886248352085E-5	0.0063035258947956585	5	5
16	0.0010504201680672268	5.0834886248352085E-5	0.0063035258947956585	5	5
73	0.004792542016806723	1.0081298217956558E-4	0.011593492950650042	5	5
105	0.006893382352941176	1.3627501730240077E-4	0.014717701868659283	58	59
107	0.00702468487394958	1.7517120977184053E-4	0.017867463396727734	87	100
11	7.221638655462185E-4	2.7779827932650214E-4	0.026668634815344205	7	7
380	0.02494747899159664	4.0905572613181285E-4	0.03722407107799497	69	122
28	0.001838235294117647	4.675729732649403E-4	0.040211275700784865	26	28
39	0.0025603991596638656	6.689383256319736E-6	0.00806767158226862	7	14
100	0.006565126050420168	1.9831058684083303E-5	0.00806767158226862	14	77
254	0.016675420168067227	2.3384555310923538E-5	0.00806767158226862	33	85

254	0.016675420168067227	2.3384555310923538E-5	0.00806767158226862	33	85
18	0.00118172268907563[illegible]	3.514864526295014E-4	0.03233675364191413	5	8
7	4.5955882352941176E-4	4.82847106378248E-4	0.03603432054858913	3	3
86	0.005646008403361344	5.441051086844118E-4	0.03603432054858913	2	2
109	0.007155987394957983	5.543741622859866E-4	0.03603432054858913	34	70
109	0.007155987394957983	5.543741622859866E-4	0.03603432054858913	34	71
109	0.007155987394957983	5.543741622859866E-4	0.03603432054858913	34	71
28	0.001838235294117647	6.371485767332485E-4	0.03950321175746141	2	2
57	0.003742121848739496	7.711239990724206E-4	0.04057742158025257	14	18
68	0.004464285714285714	8.281106444949504E-4	0.04057742158025257	14	21
68	0.004464285714285714	8.281106444949504E-4	0.04057742158025257	14	21
68	0.004464285714285714	8.281106444949504E-4	0.04057742158025257	14	21
68	0.004464285714285714	8.281106444949504E-4	0.04057742158025257	14	21
68	0.004464285714285714	8.281106444949504E-4	0.04057742158025257	18	39
315	0.02068014705882353	8.69625585844469E-4	0.040872402534690044	6	9
39	0.00256039915966386[illegible]	9.577781279004327E-4	0.044057793883419905	5	5
116	0.007615546218487395	0.0010269298847819242	0.04415798504562274	34	60

Reactions ratio	Species identifie	Species name	Submitted entities found	
3.494792758789404E-4	9606	Homo sapiens	DNAJB1;HSP90AA1;HSPH1	R-HSA-3371554;R-HSA-5324617;R-HSA-5
5.591668414063046E-4	9606	Homo sapiens	DNAJB1;HSP90AA1;HSPH1	R-HSA-3371554;R-HSA-5324617;R-HSA-5
4.892709862305165E-4	9606	Homo sapiens	DNAJB1;HSP90AA1;HSPH1	R-HSA-3371586;R-HSA-4793819;R-HSA-5
0.00202697980009785422	9606	Homo sapiens	DNAJB1;HSP90AA1;HSPH1	R-HSA-5251955;R-HSA-5082409;R-HSA-5
9.78541972461033E-4	9606	Homo sapiens	DNAJB1;HSPH1;HSPA1B;H	R-HSA-5251955;R-HSA-5251942;R-HSA-5
4.193751310547285E-4	9606	Homo sapiens	MT-ATP6;MT-ND4;MT-CO2;	R-HSA-6785722
2.0968756552736424E-4	9606	Homo sapiens	MT-ATP6;MT-ND4;MT-CO2;	R-HSA-6785722
0.0060110435451177745	9606	Homo sapiens	AMN;UBC;APOC3;APOA4;P	R-HSA-174690;R-HSA-6784676;R-HSA-88
8.38750262109457E-4	9606	Homo sapiens	APOA4;PCSK5;CIDEC	R-HSA-6784676;R-HSA-6784622
0.0023764590759767948	9606	Homo sapiens	FTH1;UBC;FTL	R-HSA-5690886;R-HSA-5691108;R-HSA-1
0.00216677151044943	9606	Homo sapiens	APOC3;APOA4;PCSK5;CID	R-HSA-174690;R-HSA-6784676;R-HSA-17
1.3979171035157615E-4	9606	Homo sapiens	NR5A2;NR1H4	R-HSA-382096;R-HSA-376419
0.03180261410498358	9606	Homo sapiens	FABP1;DNAJB1;HSP90AA1	R-HSA-400143;R-HSA-5324632;R-HSA-97
0.03396938561543301	9606	Homo sapiens	FABP1;DNAJB1;HSP90AA1	R-HSA-400143;R-HSA-5324632;R-HSA-97
0.0023065632208010066	9606	Homo sapiens	HSP90AA1;HSPH1;IGKC;F	R-HSA-2187266;R-HSA-2247514;R-HSA-2
0.00216677151044943	9606	Homo sapiens	MT-ATP6;MT-ND4;MT-CO2;	R-HSA-164834;R-HSA-164832;R-HSA-163
0.0015377088138673377	9606	Homo sapiens	DNAJB1;HSP90AA1;HSPA1	R-HSA-9726580;R-HSA-9706837;R-HSA-5
0.0013280212483399733	9606	Homo sapiens	HSP90AA1;UBC	R-HSA-9626256;R-HSA-9626039;R-HSA-9
8.38750262109457E-4	9606	Homo sapiens	UBC;JMY	R-HSA-6804229;R-HSA-6804242;R-HSA-6
0.0023065632208010066	9606	Homo sapiens	CST3;UBC;APOA4	R-HSA-976734;R-HSA-5667111;R-HSA-56
2.0968756552736424E-4	9606	Homo sapiens	RPS15;RPS4X;RPS14;RPS	R-HSA-9729232;R-HSA-9727869;R-HSA-9
6.989585517578807E-5	9606	Homo sapiens	RPL4;RPL5;RPL30;RPL3;R	R-HSA-927789
1.3979171035157615E-4	9606	Homo sapiens	RPL4;RPL5;RPL30;RPL3;R	R-HSA-72673;R-HSA-72676
1.3979171035157615E-4	9606	Homo sapiens	RPL4;RPL5;RPL30;RPL3;R	R-HSA-192704;R-HSA-192841
3.494792758789404E-4	9606	Homo sapiens	RPL4;RPL5;RPL30;RPL3;R	R-HSA-1799326;R-HSA-1799330;R-HSA-1
2.0968756552736424E-4	9606	Homo sapiens	RPL4;RPL5;RPL30;RPL3;R	R-HSA-156832;R-HSA-156823;R-HSA-156
2.0968756552736424E-4	9606	Homo sapiens	RPL4;RPL5;RPL30;RPL3;R	R-HSA-72672;R-HSA-72619;R-HSA-72671
1.3979171035157615E-4	9606	Homo sapiens	RPS15;RPS4X;RPS14;RPS	R-HSA-156808;R-HSA-157849
1.3979171035157615E-4	9606	Homo sapiens	RPS15;RPS4X;RPS14;RPS	R-HSA-72697;R-HSA-72621

6.290626965820927E-4	9606	Homo sapiens	RPL4;RPL5;RPL30;RPL3;R	R-HSA-156915;R-HSA-156912;R-HSA-156
4.892709862305165E-4	9606	Homo sapiens	RPL4;RPL5;RPL30;RPL3;R	R-HSA-6791223;R-HSA-6791222;R-HSA-6
4.193751310547285E-4	9606	Homo sapiens	RPL4;RPL5;RPL30;RPL3;R	R-HSA-927836;R-HSA-927789;R-HSA-927
3.494792758789404E-4	9606	Homo sapiens	RPL4;RPL5;RPL30;RPL3;R	R-HSA-156915;R-HSA-156912;R-HSA-156
3.494792758789404E-4	9606	Homo sapiens	RPL4;RPL5;RPL30;RPL3;R	R-HSA-141671;R-HSA-141691;R-HSA-141
3.494792758789404E-4	9606	Homo sapiens	RPL4;RPL5;RPL30;RPL3;R	R-HSA-927836;R-HSA-927832;R-HSA-927
0.0014678129586915495	9606	Homo sapiens	RPL4;RPL5;RPL30;RPL3;R	R-HSA-72673;R-HSA-72672;R-HSA-15683
0.0012581253931641854	9606	Homo sapiens	RPL4;RPL5;RPL30;RPL3;R	R-HSA-72673;R-HSA-72672;R-HSA-72691
0.0010484378276368212	9606	Homo sapiens	RPL4;RPL5;RPL30;RPL3;R	R-HSA-6791223;R-HSA-6791222;R-HSA-6
0.001957083944922066	9606	Homo sapiens	RPL4;RPL5;RPL30;RPL3;R	R-HSA-9640195;R-HSA-9633008;R-HSA-9
0.0014678129586915495	9606	Homo sapiens	RPL4;RPL5;RPL30;RPL3;R	R-HSA-6791223;R-HSA-6791222;R-HSA-6
0.0030055217725588872	9606	Homo sapiens	HBB	R-HSA-1008220
4.193751310547285E-4	9606	Homo sapiens	HBB	R-HSA-1247668
0.0013280212483399733	9606	Homo sapiens	HBB	R-HSA-9625188;R-HSA-9620197;R-HSA-9
5.591668414063046E-4	9606	Homo sapiens	HBB	R-HSA-6806831;R-HSA-1237325
9.78541972461033E-4	9606	Homo sapiens	HBB	R-HSA-1247668;R-HSA-6806831;R-HSA-1
2.0968756552736424E-4	9606	Homo sapiens	HBB	R-HSA-9631065;R-HSA-9615721;R-HSA-9
0.0017473963793947019	9606	Homo sapiens	HBB	R-HSA-9707504;R-HSA-9707516
0.0015377088138673377	9606	Homo sapiens	DNAJA1	R-HSA-3371590;R-HSA-3371503;R-HSA-3
0.0015377088138673377	9606	Homo sapiens	HBB	R-HSA-9709317;R-HSA-9709360;R-HSA-9
8.38750262109457E-4	9606	Homo sapiens	HBB	R-HSA-2168883;R-HSA-2168885;R-HSA-2
4.892709862305165E-4	9606	Homo sapiens	SEPHS2	R-HSA-8959510
0.02362479904941637	9606	Homo sapiens	HBB	R-HSA-1008220
0.007548752358985112	9606	Homo sapiens	HBB	R-HSA-9625188;R-HSA-9620197;R-HSA-9
0.0023065632208010066	9606	Homo sapiens	HBB	R-HSA-2168883;R-HSA-2168885;R-HSA-2
0.0023065632208010066	9606	Homo sapiens	SEPHS2	R-HSA-8959510
0.03180261410498358	9606	Homo sapiens	DNAJA1;HBB	R-HSA-9709317;R-HSA-9707504;R-HSA-9
0.03396938561543301	9606	Homo sapiens	DNAJA1;HBB	R-HSA-9709317;R-HSA-9707504;R-HSA-9
0.009366044593555603	9606	Homo sapiens	HBB	R-HSA-9709317;R-HSA-9709360;R-HSA-9
6.989585517578808E-4	9606	Homo sapiens	HBB	R-HSA-6800434;R-HSA-6798745
0.0199203187250996	9606	Homo sapiens	SEPHS2	R-HSA-8959510

6.989585517578807E-5	9606	Homo sapiens	RPL30;RPL12;RPLP1;RPLF	R-HSA-927789
1.3979171035157615E-4	9606	Homo sapiens	RPL30;RPL12;RPLP1;RPLF	R-HSA-72673;R-HSA-72676
1.3979171035157615E-4	9606	Homo sapiens	RPL30;RPL12;RPLP1;RPLF	R-HSA-192704;R-HSA-192841
3.494792758789404E-4	9606	Homo sapiens	RPL30;RPL12;RPLP1;RPLF	R-HSA-1799326;R-HSA-1799330;R-HSA-1
2.0968756552736424E-4	9606	Homo sapiens	RPL30;RPL12;RPLP1;RPLF	R-HSA-72672;R-HSA-72619;R-HSA-72671
1.3979171035157615E-4	9606	Homo sapiens	RPS9;RPS8;RPS3A;RPSA;I	R-HSA-156808;R-HSA-157849
1.3979171035157615E-4	9606	Homo sapiens	RPS9;RPS8;RPS3A;RPSA;I	R-HSA-72697;R-HSA-72621
4.892709862305165E-4	9606	Homo sapiens	RPL30;RPL12;RPLP1;RPLF	R-HSA-6791223;R-HSA-6791222;R-HSA-6
4.193751310547285E-4	9606	Homo sapiens	RPL30;RPL12;RPLP1;RPLF	R-HSA-927836;R-HSA-927789;R-HSA-927
3.494792758789404E-4	9606	Homo sapiens	RPL30;RPL12;RPLP1;RPLF	R-HSA-156915;R-HSA-156912;R-HSA-156
3.494792758789404E-4	9606	Homo sapiens	RPL30;RPL12;RPLP1;RPLF	R-HSA-927836;R-HSA-927832;R-HSA-927
2.0968756552736424E-4	9606	Homo sapiens	RPL30;RPL12;RPLP1;RPLF	R-HSA-156823;R-HSA-156826
3.494792758789404E-4	9606	Homo sapiens	RPL30;RPL12;RPLP1;RPLF	R-HSA-141671;R-HSA-141691;R-HSA-141
0.001467812958691549	9606	Homo sapiens	RPL30;RPL12;RPLP1;RPLF	R-HSA-72673;R-HSA-72672;R-HSA-72691
0.001258125393164185	9606	Homo sapiens	RPL30;RPL12;RPLP1;RPLF	R-HSA-72673;R-HSA-72672;R-HSA-72691
0.001048437827636821	9606	Homo sapiens	RPL30;RPL12;RPLP1;RPLF	R-HSA-6791223;R-HSA-6791222;R-HSA-6
6.290626965820927E-4	9606	Homo sapiens	RPL30;RPL12;RPLP1;RPLF	R-HSA-156915;R-HSA-156912;R-HSA-156
2.0968756552736424E-4	9606	Homo sapiens	RPS9;RPS8;RPS3A;RPSA;I	R-HSA-9729232
2.0968756552736424E-4	9606	Homo sapiens	RPS9;RPS8;RPS3A;RPSA;I	R-HSA-72691
4.193751310547285E-4	9606	Homo sapiens	RPS9;RPS8;RPS3A;RPSA;I	R-HSA-191786;R-HSA-9729849
0.003085091117807433	9606	Homo sapiens	JMJD1C Q15652	R-HSA-997263
0.024250251112067727	9606	Homo sapiens	JMJD1C Q15652	R-HSA-997263
4.193751310547285E-4	9606	Homo sapiens	MT-ND4L;MT-ND4;MT-ND5;	R-HSA-6785722
2.0968756552736424E-4	9606	Homo sapiens	MT-ND4L;MT-ND4;MT-ND5;	R-HSA-6785722
0.003774376179492556	9606	Homo sapiens	MT-ND4L;MT-ND4;MT-ND5;	R-HSA-6785722
0.00216677151044943	9606	Homo sapiens	MT-ND4;MT-ND5;MT-CO1;M	R-HSA-164834;R-HSA-6799191;R-HSA-16
0.001328021248339973	9606	Homo sapiens	MT-ND4;MT-ND5;MT-CO1;M	R-HSA-6799191;R-HSA-163217;R-HSA-67
0.001467812958691549	9606	Homo sapiens	MT-ND4L;MT-ND4;MT-ND5;	R-HSA-6785722
0.004683022296777801	9606	Homo sapiens	MT-ND4;MT-ND5;MT-CO1;M	R-HSA-164834;R-HSA-6799191;R-HSA-16
9.08646117285245E-4	9606	Homo sapiens	MT-ND4;MT-ND5;MT-ND2;M	R-HSA-6799191;R-HSA-6799202;R-HSA-6
0.001887188089746278	9606	Homo sapiens	MT1M;MT1G;MT1H	R-HSA-5662599;R-HSA-5662597;R-HSA-5

0.00216677151044943	9606	Homo sapiens MT1M;MT1G;MT1H	R-HSA-5662599;R-HSA-5662597;R-HSA-5
1.3979171035157615E-4	9606	Homo sapiens RUNX1	R-HSA-8951926;R-HSA-8951910
0.013210316628223947	9606	Homo sapiens MT-ND4L;MT-ND4;RNGTT;I	R-HSA-77073;R-HSA-77090;R-HSA-77077
5.591668414063046E-4	9606	Homo sapiens PPARG	R-HSA-9017441;R-HSA-9017447
0.005381980848535682	9606	Homo sapiens PPARG;PTPN4	R-HSA-9006585;R-HSA-9006588;R-HSA-9
6.989585517578807E-5	9606	Homo sapiens ALDOB	R-HSA-5656438
0.0015377088138673377	9606	Homo sapiens MT-CO1;MT-CO2;MT-CO3	R-HSA-9709406;R-HSA-163214
0.0013280212483399733	9606	Homo sapiens RUNX1	R-HSA-8938440;R-HSA-8935785;R-HSA-8
0.00181729223457049	9606	Homo sapiens PTPN4	R-HSA-9006585;R-HSA-9006588
0.0015377088138673377	9606	Homo sapiens PBX1	R-HSA-9021406;R-HSA-9021412
9.78541972461033E-4	9606	Homo sapiens PTPN4	R-HSA-9008894
8.38750262109457E-4	9606	Homo sapiens APOA4	R-HSA-6784622
0.00216677151044943	9606	Homo sapiens APOA4	R-HSA-174690;R-HSA-174757;R-HSA-678
0.0060110435451177745	9606	Homo sapiens APOA4	R-HSA-174690;R-HSA-174660;R-HSA-174
3.494792758789404E-4	9606	Homo sapiens LCT	R-HSA-5658001
3.494792758789404E-4	9606	Homo sapiens APOA4	R-HSA-174660;R-HSA-174741;R-HSA-174
2.0968756552736424E-4	9606	Homo sapiens APOA4	R-HSA-174690;R-HSA-174757
5.591668414063046E-4	9606	Homo sapiens FBXL17	R-HSA-9708517;R-HSA-9708525;R-HSA-9
9.08646117285245E-4	9606	Homo sapiens LCT	R-HSA-189062
0.0013280212483399733	9606	Homo sapiens APOA4	R-HSA-174660;R-HSA-174741;R-HSA-174
6.989585517578808E-4	9606	Homo sapiens NUP85	R-HSA-9615937;R-HSA-9634219;R-HSA-9
6.989585517578807E-5	9606	Homo sapiens NUP85	R-HSA-5661474
2.0968756552736424E-4	9606	Homo sapiens NUP85	R-HSA-158481;R-HSA-158484;R-HSA-111
3.494792758789404E-4	9606	Homo sapiens NUP85	R-HSA-4551649;R-HSA-4551679
2.0968756552736424E-4	9606	Homo sapiens NUP85	R-HSA-77587;R-HSA-159046;R-HSA-1590
3.494792758789404E-4	9606	Homo sapiens NUP85	R-HSA-170796
4.892709862305165E-4	9606	Homo sapiens NUP85	R-HSA-180710;R-HSA-180732
1.3979171035157615E-4	9606	Homo sapiens NUP85	R-HSA-2990880;R-HSA-2990882
2.0968756552736424E-4	9606	Homo sapiens NUP85	R-HSA-5228523
1.3979171035157615E-4	9606	Homo sapiens NUP85	R-HSA-180622
6.989585517578808E-4	9606	Homo sapiens NUP85	R-HSA-165043;R-HSA-165047

3.494792758789404E-4	9606	Homo sapiens IQGAP3	R-HSA-5626549;R-HSA-5626469;R-HSA-5
0.002586146641504159	9606	Homo sapiens RRAD	R-HSA-9613219;R-HSA-9613210;R-HSA-9
3.494792758789404E-4	9606	Homo sapiens IQGAP3	R-HSA-9018746
4.193751310547285E-4	9606	Homo sapiens IQGAP3	R-HSA-9013110
0.003355001048437828	9606	Homo sapiens RRAD	R-HSA-9613219;R-HSA-9613210;R-HSA-9
0.007129377227930384	9606	Homo sapiens RRAD	R-HSA-9613219;R-HSA-9613210;R-HSA-9
4.193751310547285E-4	9606	Homo sapiens IQGAP3	R-HSA-9013009
4.193751310547285E-4	9606	Homo sapiens IQGAP3	R-HSA-9013157
0.011462920248829245	9606	Homo sapiens RRAD	R-HSA-9613219;R-HSA-9613210;R-HSA-9
4.193751310547285E-4	9606	Homo sapiens IQGAP3	R-HSA-9013145
2.795834207031523E-4	9606	Homo sapiens NCAPG	R-HSA-2514854;R-HSA-2529015;R-HSA-2
0.007898231634864052	9606	Homo sapiens IQGAP3	R-HSA-5626549;R-HSA-5626469;R-HSA-5
9.08646117285245E-4	9606	Homo sapiens IQGAP3	R-HSA-451377
0.006360522820996715	9606	Homo sapiens IQGAP3	R-HSA-9013145;R-HSA-9018746;R-HSA-9
1.3979171035157615E-4	9606	Homo sapiens IQGAP3	R-HSA-9018794
4.193751310547285E-4	9606	Homo sapiens IQGAP3	R-HSA-6802912;R-HSA-6802911;R-HSA-6
2.795834207031523E-4	9606	Homo sapiens IQGAP3	R-HSA-9018766
0.0013979171035157615	9606	Homo sapiens HMBS	R-HSA-189406
8.38750262109457E-4	9606	Homo sapiens IQGAP3	R-HSA-9657603;R-HSA-5672980;R-HSA-5
4.892709862305165E-4	9606	Homo sapiens IQGAP3	R-HSA-9656211;R-HSA-9656209;R-HSA-9
0.0010484378276368212	9606	Homo sapiens COL1A1;ITGB1;COL3A1;C(	R-HSA-2731149;R-HSA-2731075;R-HSA-2
0.0038442720346683443	9606	Homo sapiens ITGB1;COL1A1;COL3A1;C(	R-HSA-2484965;R-HSA-114563;R-HSA-24
0.052142307961137904	9606	Homo sapiens ITGB1;COL1A1;COL3A1;F(	R-HSA-5654614;R-HSA-5654615;R-HSA-8
0.0015377088138673377	9606	Homo sapiens COL1A1;ITGB1;COL3A1;C(	R-HSA-2731149;R-HSA-2465890;R-HSA-2
0.022296777801076396	9606	Homo sapiens ITGB1;COL1A1;COL3A1;C(	R-HSA-2002460;R-HSA-2473511;R-HSA-2
3.494792758789404E-4	9606	Homo sapiens COL1A1;ITGB1;COL3A1;C(	R-HSA-8874079
0.0017473963793947019	9606	Homo sapiens FGF7;PDE3B;GRB10;FGFF	R-HSA-74707;R-HSA-109699;R-HSA-7474
0.0016076046690431257	9606	Homo sapiens COL1A1;ITGB1;COL3A1;C(	R-HSA-4086204;R-HSA-2426471;R-HSA-2
0.003564688613965192	9606	Homo sapiens COL1A1;ITGB1;COL3A1;G(	R-HSA-8874079;R-HSA-8875661;R-HSA-8
0.001957083944922066	9606	Homo sapiens COL1A1;COL3A1;COL1A2;(	R-HSA-8944240;R-HSA-8944214;R-HSA-8
0.007339064793457748	9606	Homo sapiens COL1A1;COL3A1;COL1A2;(	R-HSA-1454822;R-HSA-2485111;R-HSA-1

8.38750262109457E-4	9606	Homo sapiens	COL1A1;ITGB1;COL3A1;CO	R-HSA-8874079;R-HSA-8875531
4.193751310547285E-4	9606	Homo sapiens	COL1A1;ITGB1;COL1A2	R-HSA-114577;R-HSA-114563;R-HSA-114
1.3979171035157615E-4	9606	Homo sapiens	FGF7;NRG1;FGFR1	R-HSA-2400009
0.0023764590759767948	9606	Homo sapiens	FGF7;PDE3B;GRB10;FGFR	R-HSA-74707;R-HSA-109699;R-HSA-7474
4.193751310547285E-4	9606	Homo sapiens	FGF7;PDE3B;FGFR1	R-HSA-109699;R-HSA-162363;R-HSA-162
6.290626965820927E-4	9606	Homo sapiens	FGF7;PDE3B;FGFR1	R-HSA-109699;R-HSA-162363;R-HSA-162
0.0014678129586915495	9606	Homo sapiens	FGF7;NRG1;FGFR1	R-HSA-2400009
0.00181729223457049	9606	Homo sapiens	COL1A1;COL3A1;COL1A2;	R-HSA-2395302;R-HSA-2395340;R-HSA-1
3.494792758789404E-4	9606	Homo sapiens	FGFR1	R-HSA-5654544;R-HSA-5654545;R-HSA-2
2.0968756552736424E-4	9606	Homo sapiens	CD44	R-HSA-428287;R-HSA-428296
0.001957083944922066	9606	Homo sapiens	SMAD3;EMB;TGFBR1;SMA	R-HSA-178178;R-HSA-2176417;R-HSA-21
1.3979171035157615E-4	9606	Homo sapiens	RUNX1	R-HSA-8951926;R-HSA-8951910
0.00216677151044943	9606	Homo sapiens	STAT5B;COL3A1;COL4A1;N	R-HSA-186800;R-HSA-382054;R-HSA-380
6.989585517578807E-5	9606	Homo sapiens	SMAD3;TGFBR1	R-HSA-3656523
6.989585517578807E-5	9606	Homo sapiens	NRG1	R-HSA-1306953
1.3979171035157615E-4	9606	Homo sapiens	SMAD3;TGFBR1	R-HSA-3656382;R-HSA-3656523
6.989585517578807E-5	9606	Homo sapiens	SMAD3;TGFBR1	R-HSA-3304394
0.0030754176277346754	9606	Homo sapiens	SMAD3;EMB;TGFBR1;SMA	R-HSA-178178;R-HSA-2176417;R-HSA-21
1.3979171035157615E-4	9606	Homo sapiens	SMAD3;TGFBR1	R-HSA-3304394;R-HSA-3315483
4.892709862305165E-4	9606	Homo sapiens	SMAD3;TGFBR1	R-HSA-3645780;R-HSA-3311014;R-HSA-3
3.494792758789404E-4	9606	Homo sapiens	ELMO1;DOCK2	R-HSA-200952
0.0013979171035157615	9606	Homo sapiens	ELMO1;CD28;DOCK2	R-HSA-167633;R-HSA-167637;R-HSA-200
0.0015377088138673377	9606	Homo sapiens	COL3A1;COL4A1;LAMB1	R-HSA-2328129;R-HSA-2426355;R-HSA-2
1.3979171035157615E-4	9606	Homo sapiens	CD28;STRN;NRG1;PIK3CB	R-HSA-2400009
6.989585517578807E-5	9606	Homo sapiens	EML4;STRN	R-HSA-9700131
0.0011183336828126092	9606	Homo sapiens	STAT5B;NCK2;PIK3CB	R-HSA-186800;R-HSA-380782;R-HSA-186
0.001887188089746278	9606	Homo sapiens	SMAD3;SMAD7	R-HSA-2187395;R-HSA-2187330;R-HSA-9
0.0069895855175788075	9606	Homo sapiens	SMAD3;EMB;TGFBR1;SMA	R-HSA-178178;R-HSA-2186785;R-HSA-21
0.007339064793457748	9606	Homo sapiens	COL3A1;COL4A1;LAMB1;A	R-HSA-1454791;R-HSA-2485111;R-HSA-1
0.004543230586426225	9606	Homo sapiens	LYN;CD74;CXADR;IGKC;SL	R-HSA-210291;R-HSA-114577;R-HSA-567
0.00181729223457049	9606	Homo sapiens	LYN;BLK;CD79A;IGKC;PLC	R-HSA-9606162;R-HSA-9606163;R-HSA-9

0.0023764590759767948	9606	Homo sapiens	G6PC;PCK1	R-HSA-9623280;R-HSA-9623168;R-HSA-9
0.0021667715104494 3	9606	Homo sapiens	APOC3;APOA4;PCSK5	R-HSA-174690;R-HSA-6784676;R-HSA-17
0.0060110435451177745	9606	Homo sapiens	AMN;APOC3;APOA4;PCSK	R-HSA-174690;R-HSA-6784676;R-HSA-26
1.3979171035157615E-4	9606	Homo sapiens	LGALS3	R-HSA-8938382;R-HSA-8938371
8.38750262109457E-4	9606	Homo sapiens	IGKC;IGLC2;IGHA1;IGHA2;	R-HSA-2203516
2.795834207031523E-4	9606	Homo sapiens	BLK	R-HSA-8938987;R-HSA-8938965
8.38750262109457E-4	9606	Homo sapiens	APOA4;PCSK5	R-HSA-6784676;R-HSA-6784622
2.795834207031523E-4	9606	Homo sapiens	LYN;CD79A;IGKC;IGLC2	R-HSA-5690740;R-HSA-5690702;R-HSA-5
0.003145313482910463	9606	Homo sapiens	LYN;BLK;CD79A;IGKC;PLC	R-HSA-9606162;R-HSA-9606163;R-HSA-5
1.3979171035157615E-4	9606	Homo sapiens	PCK1	R-HSA-9028524;R-HSA-9605051
4.892709862305165E-4	9606	Homo sapiens	LGALS3	R-HSA-8938382;R-HSA-8938389
0.004193751310547285	9606	Homo sapiens	FABP6;PCK1	R-HSA-9631296;R-HSA-9028524;R-HSA-9
0.02362479904941637	9606	Homo sapiens	LYN;CD74;SERPINA1;CXAI	R-HSA-139842;R-HSA-114689;R-HSA-432
2.0968756552736424E-4	9606	Homo sapiens	LYN;PLCG2	R-HSA-9027373;R-HSA-9032478;R-HSA-9
3.494792758789404E-4	9606	Homo sapiens	APOC3;APOA4	R-HSA-174660;R-HSA-174741;R-HSA-174
0.0011882295379883974	9606	Homo sapiens	HLA-DRB5;PLCG2;HLA-DR	R-HSA-202407;R-HSA-202245;R-HSA-202
0.0012581253931641854	9606	Homo sapiens	DPEP1;CYP3A4;CYP3A5	R-HSA-5423664;R-HSA-5433067;R-HSA-5
4.193751310547285E-4	9606	Homo sapiens	FABP6	R-HSA-9631296;R-HSA-9607342
1.3979171035157615E-4	9606	Homo sapiens	RPL4;RPL5;RPL30;RPL3;R	R-HSA-72673;R-HSA-72676
6.989585517578807E-5	9606	Homo sapiens	RPL4;RPL5;RPL30;RPL3;R	R-HSA-927789
2.0968756552736424E-4	9606	Homo sapiens	RPS9;RPS7;RPS8;RPS5;RI	R-HSA-9729232;R-HSA-9727869;R-HSA-9
2.0968756552736424E-4	9606	Homo sapiens	RPL4;RPL5;RPL30;RPL3;R	R-HSA-72672;R-HSA-72619;R-HSA-72671
2.0968756552736424E-4	9606	Homo sapiens	RPL4;RPL5;RPL30;RPL3;R	R-HSA-156832;R-HSA-156823;R-HSA-156
1.3979171035157615E-4	9606	Homo sapiens	RPL4;RPL5;RPL30;RPL3;R	R-HSA-192704;R-HSA-192841
3.494792758789404E-4	9606	Homo sapiens	RPL4;RPL5;RPL30;RPL3;R	R-HSA-1799326;R-HSA-1799330;R-HSA-1
1.3979171035157615E-4	9606	Homo sapiens	RPS9;RPS7;RPS8;RPS5;RI	R-HSA-156808;R-HSA-157849
1.3979171035157615E-4	9606	Homo sapiens	RPS9;RPS7;RPS8;RPS5;RI	R-HSA-72697;R-HSA-72621
6.290626965820927E-4	9606	Homo sapiens	RPL4;RPL5;RPL30;RPL3;R	R-HSA-156915;R-HSA-156912;R-HSA-156
4.892709862305165E-4	9606	Homo sapiens	RPL4;RPL5;RPL30;RPL3;R	R-HSA-6791223;R-HSA-6791222;R-HSA-6
4.193751310547285E-4	9606	Homo sapiens	RPL4;RPL5;RPL30;RPL3;R	R-HSA-927836;R-HSA-927789;R-HSA-927
3.494792758789404E-4	9606	Homo sapiens	RPL4;RPL5;RPL30;RPL3;R	R-HSA-156915;R-HSA-156912;R-HSA-156

3.494792758789404E-4	9606	Homo sapiens	RPL4;RPL5;RPL30;RPL3;R	R-HSA-927836;R-HSA-927832;R-HSA-927
0.00146781295869154951	9606	Homo sapiens	RPL4;RPL5;RPL30;RPL3;R	R-HSA-72673;R-HSA-72672;R-HSA-15683
3.494792758789404E-4	9606	Homo sapiens	RPL4;RPL5;RPL30;RPL3;R	R-HSA-141671;R-HSA-141691;R-HSA-141
0.00125812539316418541	9606	Homo sapiens	RPL4;RPL5;RPL30;RPL3;R	R-HSA-72673;R-HSA-72672;R-HSA-72691
0.00104843782763682121	9606	Homo sapiens	RPL4;RPL5;RPL30;RPL3;R	R-HSA-6791223;R-HSA-6791222;R-HSA-6
0.001957083944922066	9606	Homo sapiens	RPL4;RPL5;RPL30;RPL3;R	R-HSA-9640195;R-HSA-9633008;R-HSA-9
0.00146781295869154951	9606	Homo sapiens	RPL4;RPL5;RPL30;RPL3;R	R-HSA-6791223;R-HSA-6791222;R-HSA-6
0.00139791710351576151	9606	Homo sapiens	STAT1;STAT3;IL6ST	R-HSA-1112690;R-HSA-1112755;R-HSA-1
0.00174739637939470191	9606	Homo sapiens	PIK3CA;STAT1;CNTRL;STA	R-HSA-2023455;R-HSA-1839102;R-HSA-1
0.00237645907597679481	9606	Homo sapiens	STAT1;STAT3;IL6ST	R-HSA-1112690;R-HSA-6783524;R-HSA-6
0.00244635493115258251	9606	Homo sapiens	PIK3CA;STAT1;CNTRL;STA	R-HSA-2023455;R-HSA-2023451;R-HSA-1
0.006919689662403019	9606	Homo sapiens	PIK3CA;POLR2A;STAT1;CN	R-HSA-2023455;R-HSA-5655252;R-HSA-2
0.002725938351855735	9606	Homo sapiens	FER;PIK3CA;STAT1;STAT3;	R-HSA-1433508;R-HSA-205328;R-HSA-14
0.01810302649052911	9606	Homo sapiens	BNIP3L;ATF2;BRD1;PLK2;F	R-HSA-6797606;R-HSA-6801637;R-HSA-6
9.78541972461033E-4	9606	Homo sapiens	PIK3CA;STAT1;CNTRL;STA	R-HSA-1839102;R-HSA-1839039;R-HSA-1
0.00139791710351576151	9606	Homo sapiens	STAT1;STAT3;IL6ST	R-HSA-8950537;R-HSA-8950441;R-HSA-8
7.688544069336689E-4	9606	Homo sapiens	PIK3CA;STAT1;STAT3	R-HSA-9670433;R-HSA-9670417;R-HSA-9
9.78541972461033E-4	9606	Homo sapiens	RGCC;PLK2	R-HSA-6801637;R-HSA-6801666;R-HSA-6
0.00181729223457049	9606	Homo sapiens	PIK3CA;STAT1;STAT3	R-HSA-9670433;R-HSA-9670417;R-HSA-9
0.00181729223457049	9606	Homo sapiens	STAT1;STAT3;IL6ST	R-HSA-8983983;R-HSA-8984014;R-HSA-8
3.494792758789404E-4	9606	Homo sapiens	FGFR1	R-HSA-5654544;R-HSA-5654545;R-HSA-2
0.002725938351855735	9606	Homo sapiens	SNW1;NBEA;NUMB;TBL1X	R-HSA-9667638;R-HSA-157353;R-HSA-19
0.00118822953798839741	9606	Homo sapiens	ATF2;POLR2A;ATM;CDK12	R-HSA-6797606;R-HSA-6797090;R-HSA-6
4.892709862305165E-4	9606	Homo sapiens	PIK3CA;STAT1;STAT3	R-HSA-9672177;R-HSA-9672176;R-HSA-9
4.892709862305165E-4	9606	Homo sapiens	PIK3CA;STAT1;STAT3	R-HSA-9672162;R-HSA-9672178;R-HSA-9
4.193751310547285E-4	9606	Homo sapiens	MT-ND4L;MTERF4;MT-CO3	R-HSA-6793057;R-HSA-6785722
8.38750262109457E-4	9606	Homo sapiens	PIK3CA;IRS2;FGFR1;IGF1F	R-HSA-74737;R-HSA-74736;R-HSA-10969
3.494792758789404E-4	9606	Homo sapiens	DNAJB1;HSP90AA1;HSPH1	R-HSA-3371554;R-HSA-5324617;R-HSA-5
5.591668414063046E-4	9606	Homo sapiens	DNAJB1;HSP90AA1;HSPH1	R-HSA-3371554;R-HSA-5324617;R-HSA-5
0.00202697980009785421	9606	Homo sapiens	DNAJB1;HSP90AA1;HSPH1	R-HSA-5251955;R-HSA-5082409;R-HSA-5
4.892709862305165E-4	9606	Homo sapiens	DNAJB1;HSP90AA1;HSP90	R-HSA-3371586;R-HSA-4793819;R-HSA-5

9.78541972461033E-4	9606	Homo sapiens	DNAJB1;HSPH1;HSPA1A	R-HSA-5251955;R-HSA-5251942;R-HSA-5
0.03396938561543301	9606	Homo sapiens	DNAJA1;DNAJB1;MT2A;HS	R-HSA-5662614;R-HSA-9706837;R-HSA-5
0.001887188089746278	9606	Homo sapiens	MT2A;MT1G;MT1H;MT1E	R-HSA-5662598;R-HSA-5662614;R-HSA-5
0.03180261410498358	9606	Homo sapiens	DNAJA1;DNAJB1;HSP90AA	R-HSA-9706837;R-HSA-5324632;R-HSA-9
0.00216677151044943	9606	Homo sapiens	MT2A;MT1G;MT1H;MT1E	R-HSA-5662598;R-HSA-5662614;R-HSA-5
0.0015377088138673377	9606	Homo sapiens	DNAJA1;DNAJB1;HSP90AA	R-HSA-9726580;R-HSA-9706837;R-HSA-5
0.013699587614454462	9606	Homo sapiens	HSP90AA1;HSP90AB1;FAB	R-HSA-9716913;R-HSA-9032751;R-HSA-9
4.892709862305165E-4	9606	Homo sapiens	HSPD1	R-HSA-8869558;R-HSA-8869542
3.494792758789404E-4	9606	Homo sapiens	HSP90AA1;HSP90AB1	R-HSA-5336420
0.004613126441602013	9606	Homo sapiens	HSP90AA1;HSP90AB1;TFF	R-HSA-9018494;R-HSA-9038161;R-HSA-9
0.0023065632208010066	9606	Homo sapiens	HSP90AA1;HSPH1;IGKC;F	R-HSA-2187266;R-HSA-2247514;R-HSA-2
4.193751310547285E-4	9606	Homo sapiens	FABP6	R-HSA-9631296;R-HSA-9607342
1.3979171035157615E-4	9606	Homo sapiens	HSP90AA1;HSPH1	R-HSA-2247514;R-HSA-2197645
4.193751310547285E-4	9606	Homo sapiens	HSP90AA1;HSP90AB1	R-HSA-419644;R-HSA-419645
0.0013280212483399733	9606	Homo sapiens	HSP90AA1;HSP90AB1	R-HSA-9626242;R-HSA-9626256;R-HSA-9
1.3979171035157615E-4	9606	Homo sapiens	C1QB;C1QA;IGKC	R-HSA-173626;R-HSA-173631
2.0968756552736424E-4	9606	Homo sapiens	MT-ND6;MT-ND5;MT-ND2;M	R-HSA-6786854;R-HSA-6785722
0.004123855455371497	9606	Homo sapiens	IL15;IL2RA;STAT4;SOS1	R-HSA-450054;R-HSA-8983371;R-HSA-89
2.795834207031523E-4	9606	Homo sapiens	SOS1;NCK1	R-HSA-428535;R-HSA-428515;R-HSA-428
0.03529740686377298	9606	Homo sapiens	ANXA1;IL15;IL2RA;HNRNPA	R-HSA-6785895;R-HSA-450054;R-HSA-45
0.0011183336828126092	9606	Homo sapiens	SOS1;NCK1	R-HSA-186834;R-HSA-186826;R-HSA-382
4.193751310547285E-4	9606	Homo sapiens	MT-ND5;MT-ND2;MT-ATP8	R-HSA-6785722
9.08646117285245E-4	9606	Homo sapiens	MT-ND6;MT-ND5;MT-ND2	R-HSA-6799202;R-HSA-6799199;R-HSA-6
0.05207241210596212	9606	Homo sapiens	ANXA1;IL15;IL2RA;HNRNPA	R-HSA-6785895;R-HSA-450054;R-HSA-45
0.0011882295379883974	9606	Homo sapiens	IL15;SOS1	R-HSA-8983371;R-HSA-8983307;R-HSA-8
0.00216677151044943	9606	Homo sapiens	MT-ND6;MT-ND5;MT-ND2;M	R-HSA-164834;R-HSA-164832;R-HSA-163
0.00216677151044943	9606	Homo sapiens	SOS1;NCK1	R-HSA-186834;R-HSA-186826;R-HSA-382
0.0013979171035157615	9606	Homo sapiens	IL2RA	R-HSA-8877396;R-HSA-8877391;R-HSA-8
0.0025162507863283707	9606	Homo sapiens	HNRNPA2B1;STAT4	R-HSA-8950344;R-HSA-8950218;R-HSA-8
0.003914167889844132	9606	Homo sapiens	HNRNPA2B1;STAT4	R-HSA-8950344;R-HSA-8950218;R-HSA-8
0.003774376179492556	9606	Homo sapiens	MT-ND6;MT-ND5;MT-ND2;M	R-HSA-6786854;R-HSA-6785722

9.08646117285245E-4	9606	Homo sapiens	NCK1	R-HSA-532603;R-HSA-373724
0.013210316628223947	9606	Homo sapiens	SRRM2;MT-ND6;MT-ND5;H	R-HSA-9770132;R-HSA-6786854;R-HSA-9
0.00796812749003984	9606	Homo sapiens	HNRNPA2B1;STAT4	R-HSA-8950415;R-HSA-9617941;R-HSA-8
9.78541972461033E-4	9606	Homo sapiens	BTG2	R-HSA-6798020;R-HSA-6798031;R-HSA-6
4.193751310547285E-4	9606	Homo sapiens	IL2RA;SOS1	R-HSA-508247;R-HSA-453111;R-HSA-912
2.0968756552736424E-4	9606	Homo sapiens	MT-ND6;MT-ND4L;MT-ND4;	R-HSA-6786854;R-HSA-6785722
4.193751310547285E-4	9606	Homo sapiens	MT-ND4L;MT-ND4;MT-ND5;	R-HSA-6785722
0.001887188089746278	9606	Homo sapiens	MT2A;MT1M;MT1F;MT1G;M	R-HSA-5662614;R-HSA-5662610;R-HSA-5
0.0021667715104494З	9606	Homo sapiens	MT2A;MT1M;MT1F;MT1G;M	R-HSA-5662614;R-HSA-5662610;R-HSA-5
0.003774376179492556	9606	Homo sapiens	MT-ND6;MT-ND4L;MT-ND4;	R-HSA-6786854;R-HSA-6783483;R-HSA-6
0.00132802124833997З	9606	Homo sapiens	MT-ND6;MT-ND4;MT-ND5;M	R-HSA-6799191;R-HSA-163217;R-HSA-67
0.00139791710351576l	9606	Homo sapiens	ACE2;ENPEP;ACE;MME;AN	R-HSA-2022398;R-HSA-2022399;R-HSA-2
3.494792758789404E-4	9606	Homo sapiens	MTTP;APOA1;APOC3;APO	R-HSA-174786;R-HSA-174660;R-HSA-174
0.00216677151044943	9606	Homo sapiens	MT-ND6;MT-ND4;MT-ND5;M	R-HSA-164834;R-HSA-6799191;R-HSA-16
9.08646117285245E-4	9606	Homo sapiens	MT-ND6;MT-ND4;MT-ND5;M	R-HSA-6799191;R-HSA-6799202;R-HSA-6
6.989585517578807E-5	9606	Homo sapiens	GUCY2C;PDZD3	R-HSA-8936356
0.00132802124833997З	9606	Homo sapiens	MTTP;APOA1;APOC3;APO	R-HSA-174786;R-HSA-8866308;R-HSA-17
1.3979171035157615E-4	9606	Homo sapiens	RUNX1	R-HSA-8951926;R-HSA-8951910
0.00146781295869154l	9606	Homo sapiens	MT-ND4L;MT-ND4;MT-ND5;	R-HSA-6791223;R-HSA-6785722
1.3979171035157615E-4	9606	Homo sapiens	HIST4H4;HIST1H4H;HIST1I	R-HSA-181450;R-HSA-176700
1.3979171035157615E-4	9606	Homo sapiens	HIST4H4;HIST1H4H;HIST1I	R-HSA-73718
2.0968756552736424E-4	9606	Homo sapiens	APOA1;APOC3;APOB	R-HSA-174690;R-HSA-174739;R-HSA-174
0.00216677151044943	9606	Homo sapiens	MTTP;APOA1;APOC3;APO	R-HSA-9737780;R-HSA-174690;R-HSA-26
4.892709862305165E-4	9606	Homo sapiens	HIST4H4;HIST1H4H;HIST1I	R-HSA-5334097;R-HSA-5334160;R-HSA-5
0.004683022296777801	9606	Homo sapiens	MT-ND6;MT-ND4;MT-ND5;M	R-HSA-164834;R-HSA-6799191;R-HSA-16
4.892709862305165E-4	9606	Homo sapiens	AKT3;RUNX2	R-HSA-8939833;R-HSA-8939963;R-HSA-8
1.3979171035157615E-4	9606	Homo sapiens	RUNX2	R-HSA-8938382;R-HSA-8938371
2.795834207031523E-4	9606	Homo sapiens	IDH1	R-HSA-9761844;R-HSA-9761822
0.00328510519326203l	9606	Homo sapiens	RORC;RORA;FOXO3	R-HSA-6790036
2.795834207031523E-4	9606	Homo sapiens	RUNX2	R-HSA-8878257;R-HSA-8878266;R-HSA-9
0.005871251834766198	9606	Homo sapiens	AKT3;PPM1D;RUNX2	R-HSA-9008137;R-HSA-8937864;R-HSA-8

3.494792758789404E-4	9606	Homo sapiens	RORC	R-HSA-8949276;R-HSA-8949301
4.892709862305165E-4	9606	Homo sapiens	RUNX2	R-HSA-8938382
2.795834207031523E-4	9606	Homo sapiens	AKT3;FOXO3	R-HSA-199299;R-HSA-199298;R-HSA-199
3.494792758789404E-4	9606	Homo sapiens	APOA4;APOB	R-HSA-174786;R-HSA-174660;R-HSA-174
3.494792758789404E-4	9606	Homo sapiens	AKT3;FOXO3	R-HSA-199299;R-HSA-9614562;R-HSA-96
0.00216677151044943	9606	Homo sapiens	APOA4;APOB	R-HSA-174690;R-HSA-174739;R-HSA-174
2.0968756552736424E-4	9606	Homo sapiens	APOA4;APOB	R-HSA-174690;R-HSA-174739;R-HSA-174
6.290626965820927E-4	9606	Homo sapiens	RUNX2	R-HSA-2064932
0.001957083944922066	9606	Homo sapiens	RBP2;APOA4;APOB	R-HSA-975629;R-HSA-2187332;R-HSA-24
0.0022366673656252184	9606	Homo sapiens	RUNX2	R-HSA-8985293;R-HSA-9008076;R-HSA-9
8.38750262109457E-4	9606	Homo sapiens	AKT3;FOXO3	R-HSA-199298;R-HSA-9624526;R-HSA-96
0.0012581253931641854	9606	Homo sapiens	AKT3;FOXO3	R-HSA-2399992;R-HSA-2400010
0.0023764590759767948	9606	Homo sapiens	RBP2;APOA4;APOB	R-HSA-975629;R-HSA-2187332;R-HSA-24
0.0013280212483399733	9606	Homo sapiens	APOA4;APOB	R-HSA-8866327;R-HSA-174786;R-HSA-88
6.989585517578807E-5	9606	Homo sapiens	RPL30;RPL34;RPLP1;RPL9	R-HSA-927789
1.3979171035157615E-4	9606	Homo sapiens	RPL30;RPL34;RPLP1;RPL9	R-HSA-72673;R-HSA-72676
1.3979171035157615E-4	9606	Homo sapiens	RPL30;RPL34;RPLP1;RPL9	R-HSA-192704;R-HSA-192841
3.494792758789404E-4	9606	Homo sapiens	RPL30;RPL34;RPLP1;RPL9	R-HSA-1799326;R-HSA-1799330;R-HSA-1
2.0968756552736424E-4	9606	Homo sapiens	RPL30;RPL34;RPLP1;RPL9	R-HSA-72672;R-HSA-72619;R-HSA-72671
2.0968756552736424E-4	9606	Homo sapiens	RPL30;RPL34;RPLP1;RPL9	R-HSA-156832;R-HSA-156823;R-HSA-156
1.3979171035157615E-4	9606	Homo sapiens	RPS7;RPS8;RPS6;RPS3A;R	R-HSA-156808;R-HSA-157849
1.3979171035157615E-4	9606	Homo sapiens	RPS7;RPS8;RPS6;RPS3A;R	R-HSA-72697;R-HSA-72621
4.892709862305165E-4	9606	Homo sapiens	RPL30;RPL34;RPLP1;RPL9	R-HSA-6791223;R-HSA-6791222;R-HSA-6
4.193751310547285E-4	9606	Homo sapiens	RPL30;RPL34;RPLP1;RPL9	R-HSA-927836;R-HSA-927789;R-HSA-927
3.494792758789404E-4	9606	Homo sapiens	RPL30;RPL34;RPLP1;RPL9	R-HSA-156915;R-HSA-156912;R-HSA-156
3.494792758789404E-4	9606	Homo sapiens	RPL30;RPL34;RPLP1;RPL9	R-HSA-927836;R-HSA-927832;R-HSA-927
0.0014678129586915495	9606	Homo sapiens	RPL30;RPL34;RPLP1;RPL9	R-HSA-72673;R-HSA-72672;R-HSA-15683
3.494792758789404E-4	9606	Homo sapiens	RPL30;RPL34;RPLP1;RPL9	R-HSA-141671;R-HSA-141691;R-HSA-141
0.0012581253931641854	9606	Homo sapiens	RPL30;RPL34;RPLP1;RPL9	R-HSA-72673;R-HSA-72672;R-HSA-72691
0.0010484378276368212	9606	Homo sapiens	RPL30;RPL34;RPLP1;RPL9	R-HSA-6791223;R-HSA-6791222;R-HSA-6
6.290626965820927E-4	9606	Homo sapiens	RPL30;RPL34;RPLP1;RPL9	R-HSA-156915;R-HSA-156912;R-HSA-156

2.0968756552736424E-4	9606	Homo sapiens	RPS7;RPS8;RPS6;RPS3A;I		R-HSA-9729232
2.0968756552736424E-4	9606	Homo sapiens	RPS7;RPS8;RPS6;RPS3A;I		R-HSA-72691
4.193751310547285E-4	9606	Homo sapiens	RPS7;RPS8;RPS6;RPS3A;I		R-HSA-156808;R-HSA-157849
2.795834207031523E-4	9606	Homo sapiens	CDH11		R-HSA-9759809;R-HSA-9759516;R-HSA-9
0.001957083944922066	9606	Homo sapiens	CDH11		R-HSA-9761110;R-HSA-9761047;R-HSA-9
0.002306563220801006	9606	Homo sapiens	CDH11		R-HSA-9761110;R-HSA-9761047;R-HSA-9
0.002306563220801006	9606	Homo sapiens	CDH11		R-HSA-9761110;R-HSA-9761047;R-HSA-9
9.78541972461033E-4	9606	Homo sapiens	CDH11		R-HSA-9760965;R-HSA-9761110;R-HSA-9
0.003424896903613615	9606	Homo sapiens	CDH11		R-HSA-9761110;R-HSA-9761047;R-HSA-9
0.001957083944922066	9606	Homo sapiens	COL1A1;COL3A1;COL5A2;(		R-HSA-8944223;R-HSA-8944240;R-HSA-8
8.38750262109457E-4	9606	Homo sapiens	COL1A1;COL3A1;COL5A2;I		R-HSA-8875558;R-HSA-8875568;R-HSA-8
0.003844272034668344	9606	Homo sapiens	COL1A1;COL3A1;VWF;COL		R-HSA-114563;R-HSA-265425;R-HSA-408
0.003564688613965192	9606	Homo sapiens	COL1A1;COL3A1;COL5A2;I		R-HSA-8875431;R-HSA-8875558;R-HSA-8
0.003774376179492556	9606	Homo sapiens	CDH11		R-HSA-9761110;R-HSA-9761047;R-HSA-9
6.989585517578807E-5	9606	Homo sapiens	NRG1		R-HSA-1306953
0.001048437827636821	9606	Homo sapiens	COL1A1;COL3A1;COL5A2		R-HSA-2731075
0.006500314531348291	9606	Homo sapiens	CDH11;PTK2B		R-HSA-9761110;R-HSA-9761047;R-HSA-9
0.00181729223457049	9606	Homo sapiens	COL1A1;COL3A1;COL5A2;(		R-HSA-2395302;R-HSA-2395340;R-HSA-1
0.002376459075976794	9606	Homo sapiens	COL1A1;COL3A1;COL5A2;(		R-HSA-1454822;R-HSA-2485111;R-HSA-1
3.494792758789404E-4	9606	Homo sapiens	COL1A1;COL3A1;COL5A2		R-HSA-8874079
0.00216677151044943	9606	Homo sapiens	COL3A1;COL5A2;RAPGEF		R-HSA-382052;R-HSA-382054
2.0968756552736424E-4	9606	Homo sapiens	CTGF		R-HSA-8951695;R-HSA-1989766
5.591668414063046E-4	9606	Homo sapiens	TPST1;VWF		R-HSA-9665809;R-HSA-9668148;R-HSA-9
7.174630506528914E-5	9606	Homo sapiens	NRG1	Q02297-10;Q022	R-HSA-1306953
5.739704405223131E-4	9606	Homo sapiens	NRG1	Q02297-10;Q022	R-HSA-1358798;R-HSA-1358792
1.4349261013057828E-4	9606	Homo sapiens	NRG1	Q02297-10;Q022	R-HSA-8848005;R-HSA-8847995
1.4349261013057828E-4	9606	Homo sapiens	NRG1	Q02297-10;Q022	R-HSA-6785636;R-HSA-6785648
5.02224135457024E-4	9606	Homo sapiens	NRG1	Q02297-10;Q022	R-HSA-1306979;R-HSA-1250346;R-HSA-1
9.327019658487588E-4	9606	Homo sapiens	NRG1	Q02297-10;Q022	R-HSA-9665699;R-HSA-9665697;R-HSA-9
4.3047783039173483E-4	9606	Homo sapiens	NRG1	Q02297-10;Q022	R-HSA-1250195;R-HSA-1250486;R-HSA-1
0.001219687186109915	9606	Homo sapiens	NRG1	Q02297-10;Q022	R-HSA-9664976;R-HSA-9664567;R-HSA-9

0.004448270914047927	9606	Homo sapiens	NRG1	Q02297-10;Q022	R-HSA-9664976;R-HSA-9664567;R-HSA-9
0.001004448270914048	9606	Homo sapiens	NRG1	Q02297-10;Q022	R-HSA-1963563;R-HSA-1358798;R-HSA-8
7.174630506528914E-5	9606	Homo sapiens	ZFYVE9	O95405-1	R-HSA-3656523
7.174630506528914E-5	9606	Homo sapiens	ZFYVE9	O95405-1	R-HSA-3304394
1.4349261013057828E-4	9606	Homo sapiens	ZFYVE9	O95405-1	R-HSA-3656523
1.4349261013057828E-4	9606	Homo sapiens	ZFYVE9	O95405-1	R-HSA-3304394
2.1523891519586742E-4	9606	Homo sapiens	MFN1	Q8IWA4	R-HSA-9018824
0.003300330033003300	9606	Homo sapiens	NRG1	Q02297-10;Q022	R-HSA-1250195;R-HSA-8848005;R-HSA-6
5.02224135457024E-4	9606	Homo sapiens	ZFYVE9	O95405-1	R-HSA-3304394;R-HSA-3656523
4.3047783039173483E-4	9606	Homo sapiens	MFN1	Q8IWA4	R-HSA-9018824
0.003802554168460324	9606	Homo sapiens	NRG1	Q02297-10;Q022	R-HSA-8848005;R-HSA-8847995
0.003802554168460324	9606	Homo sapiens	NRG1	Q02297-10;Q022	R-HSA-8848005;R-HSA-8847995
3.494792758789404E-4	9606	Homo sapiens	RPL4;RPL5;RPL30;RPL3;R		R-HSA-156930;R-HSA-156915;R-HSA-156
6.290626965820927E-4	9606	Homo sapiens	RPL4;RPL5;RPL30;RPL3;R		R-HSA-156930;R-HSA-156915;R-HSA-156
6.989585517578807E-5	9606	Homo sapiens	RPL4;RPL5;RPL30;RPL3;R		R-HSA-927789
1.3979171035157615E-4	9606	Homo sapiens	RPL4;RPL5;RPL30;RPL3;R		R-HSA-72673;R-HSA-72676
1.3979171035157615E-4	9606	Homo sapiens	RPL4;RPL5;RPL30;RPL3;R		R-HSA-192704;R-HSA-192841
2.0968756552736424E-4	9606	Homo sapiens	RPL4;RPL5;RPL30;RPL3;R		R-HSA-72672;R-HSA-72619;R-HSA-72671
2.0968756552736424E-4	9606	Homo sapiens	RPL4;RPL5;RPL30;RPL3;R		R-HSA-156832;R-HSA-156823;R-HSA-156
3.494792758789404E-4	9606	Homo sapiens	RPL4;RPL5;RPL30;RPL3;R		R-HSA-1799326;R-HSA-1799330;R-HSA-1
1.3979171035157615E-4	9606	Homo sapiens	RPS15;RPS4X;RPS14;RPS		R-HSA-156808;R-HSA-157849
1.3979171035157615E-4	9606	Homo sapiens	RPS15;RPS4X;RPS14;RPS		R-HSA-72697;R-HSA-72621
4.892709862305165E-4	9606	Homo sapiens	RPL4;RPL5;RPL30;RPL3;R		R-HSA-6791223;R-HSA-6791222;R-HSA-6
4.193751310547285E-4	9606	Homo sapiens	RPL4;RPL5;RPL30;RPL3;R		R-HSA-927836;R-HSA-927789;R-HSA-927
3.494792758789404E-4	9606	Homo sapiens	RPL4;RPL5;RPL30;RPL3;R		R-HSA-927836;R-HSA-927832;R-HSA-927
2.0968756552736424E-4	9606	Homo sapiens	RPS15;RPS4X;RPS14;RPS		R-HSA-9729232;R-HSA-9727944
0.001467812958691549	9606	Homo sapiens	RPL4;RPL5;RPL30;RPL3;R		R-HSA-72673;R-HSA-72672;R-HSA-15683
3.494792758789404E-4	9606	Homo sapiens	RPL4;RPL5;RPL30;RPL3;R		R-HSA-141671;R-HSA-141691;R-HSA-141
0.001258125393164185	9606	Homo sapiens	RPL4;RPL5;RPL30;RPL3;R		R-HSA-72673;R-HSA-72672;R-HSA-72691
0.001048437827636821	9606	Homo sapiens	RPL4;RPL5;RPL30;RPL3;R		R-HSA-6791223;R-HSA-6791222;R-HSA-6
0.001467812958691549	9606	Homo sapiens	RPL4;MT-ND4L;RPL5;MT-N		R-HSA-6791223;R-HSA-6791222;R-HSA-6

0.006919689662403019	9606	Homo sapiens RPL4;RPL5;RPL30;RPL3;R	R-HSA-5389842;R-HSA-156930;R-HSA-54
0.003285105193262039(	9606	Homo sapiens IL23R;FOS;TNF;IL17A	R-HSA-6790022;R-HSA-6789524;R-HSA-6
0.03529740686377298	9606	Homo sapiens CCL5;IL23R;MAP3K8;FOS;T	R-HSA-450054;R-HSA-912527;R-HSA-452
4.193751310547285E-4	9606	Homo sapiens MT-ATP6;MT-ND4;MT-CO1;	R-HSA-6785722
2.0968756552736424E-4	9606	Homo sapiens MT-ATP6;MT-ND4;MT-CO1;	R-HSA-6785722
0.05207241210596212	9606	Homo sapiens TNFSF14;CCL5;IL23R;MAP	R-HSA-450054;R-HSA-912527;R-HSA-452
8.38750262109457E-4	9606	Homo sapiens FOS;ELK1	R-HSA-9625479;R-HSA-198731;R-HSA-96
0.001397917103515761!	9606	Homo sapiens PPP1R15A	R-HSA-9654752;R-HSA-9650710;R-HSA-9
0.002446354931152582!	9606	Homo sapiens MAP3K8;FOS;ELK1;IL17A	R-HSA-450292;R-HSA-450325;R-HSA-568
0.001048437827636821:	9606	Homo sapiens CCL5;TNF	R-HSA-6784160
0.11630670301251136	9606	Homo sapiens PRR5;TRAPPC1;KLRB1;TN	R-HSA-450054;R-HSA-452100;R-HSA-202
0.001328021248339973:	9606	Homo sapiens CCL5;CCR6	R-HSA-373061;R-HSA-443986;R-HSA-373
0.001397917103515761!	9606	Homo sapiens IL2	R-HSA-8877348;R-HSA-8877345;R-HSA-8
0.002236667365625218	9606	Homo sapiens MAP3K8;FOS;ELK1	R-HSA-450292;R-HSA-450325;R-HSA-568
0.001258125393164185	9606	Homo sapiens KIT	R-HSA-8864690;R-HSA-8864698
4.892709862305165E-4	9606	Homo sapiens KIT	R-HSA-9669859;R-HSA-9669874;R-HSA-9
2.0968756552736424E-4	9606	Homo sapiens KIT	R-HSA-9681382;R-HSA-9669898;R-HSA-9
6.989585517578807E-5	9606	Homo sapiens KIT	R-HSA-9669878
6.989585517578807E-5	9606	Homo sapiens KIT	R-HSA-9669863
6.989585517578807E-5	9606	Homo sapiens KIT	R-HSA-9669868
6.989585517578807E-5	9606	Homo sapiens KIT	R-HSA-9669874
0.05207241210596212	9606	Homo sapiens GBP5;IL4R;CSF1;STAT1;PS	R-HSA-9730967;R-HSA-1112602;R-HSA-8
0.005521772558887258	9606	Homo sapiens GBP5;STAT1;IRF1;XAF1;GE	R-HSA-1678841;R-HSA-997326;R-HSA-97
0.001607604669043125	9606	Homo sapiens GBP5;STAT1;IRF1;GBP1;GI	R-HSA-873917;R-HSA-997326;R-HSA-973
0.001747396379394701!	9606	Homo sapiens STAT1;IRF1;XAF1	R-HSA-909725;R-HSA-997309;R-HSA-909
8.38750262109457E-4	9606	Homo sapiens DEFA6;DEFA5	R-HSA-1462039;R-HSA-1462005;R-HSA-1
0.11630670301251136	9606	Homo sapiens GBP5;IL4R;DEFA6;DTX3L;(	R-HSA-1112602;R-HSA-8987266;R-HSA-1
4.193751310547285E-4	9606	Homo sapiens TUT7;SYCP2;HIST1H2BH	R-HSA-912505;R-HSA-912408;R-HSA-912
0.001048437827636821:	9606	Homo sapiens TUT7;SYCP2;HIST1H2BH	R-HSA-912505;R-HSA-912408;R-HSA-912
0.03529740686377298	9606	Homo sapiens TXLNA;IL4R;CSF1;STAT1;F	R-HSA-6788582;R-HSA-6793958;R-HSA-8
0.001328021248339973:	9606	Homo sapiens DEFA6;DEFA5	R-HSA-1462039;R-HSA-1462023;R-HSA-1

0.0010484378276368212	9606	Homo sapiens	PSMD5;HIST1H2BH;PSMB9	R-HSA-8865491;R-HSA-8865505;R-HSA-8
0.0013979171035157615	9606	Homo sapiens	STAT1	R-HSA-1112565;R-HSA-1112727;R-HSA-11
0.0016076046690431257	9606	Homo sapiens	PSMD5;HIST1H2BH;PSMB9	R-HSA-68825;R-HSA-9749197;R-HSA-974
0.010763961697071363	9606	Homo sapiens	STAT1;PSMD5;HIST1H2BH	R-HSA-9604834;R-HSA-9604642;R-HSA-9
3.494792758789404E-4	9606	Homo sapiens	HIST1H2BH;RCOR1	R-HSA-3777129;R-HSA-3782637;R-HSA-3
0.0022366673656252184	9606	Homo sapiens	PSMD5;HIST1H2BH;PSMB9	R-HSA-68825;R-HSA-9749197;R-HSA-974
0.005381980848535682	9606	Homo sapiens	PSMD5;TRAF3;OSBPL3;HIS	R-HSA-936381;R-HSA-5689539;R-HSA-56
0.0011882295379883974	9606	Homo sapiens	TXLNA;CSF1	R-HSA-9014074;R-HSA-9009485;R-HSA-9
0.004962605717480953	9606	Homo sapiens	PSMD5;HIST1H2BH;SOX6;	R-HSA-1504193;R-HSA-3364026;R-HSA-4
0.0030055217725588872	9606	Homo sapiens	TUT7;SYCP2;HIST1H2BH	R-HSA-912505;R-HSA-912408;R-HSA-912
0.05207241210596212	9606	Homo sapiens	CEBPD;HLA-B;HLA-C;HMG	R-HSA-1169307;R-HSA-9750942;R-HSA-1
0.001887188089746278	9606	Homo sapiens	MT2A;MT1M;MT1G;MT1X;M	R-HSA-5662614;R-HSA-5662615;R-HSA-5
0.11630670301251136	9606	Homo sapiens	C1QB;C1QA;CD63;CEBPD;	R-HSA-741386;R-HSA-199169;R-HSA-688
0.00216677151044943	9606	Homo sapiens	MT2A;MT1M;MT1G;MT1X;M	R-HSA-5662614;R-HSA-5662615;R-HSA-5
2.795834207031523E-4	9606	Homo sapiens	NFKBIA;UBB;SQSTM1	R-HSA-209536;R-HSA-193703;R-HSA-193
0.005521772558887258	9606	Homo sapiens	MT2A;IFI27;UBB;HLA-B;HLA	R-HSA-1015702;R-HSA-1169307;R-HSA-9
0.0016076046690431257	9606	Homo sapiens	UBB;HLA-B;HLA-C;CYBA;S	R-HSA-8863895;R-HSA-1236947;R-HSA-8
0.0017473963793947019	9606	Homo sapiens	IFI27;UBB;HLA-B;HLA-C	R-HSA-1015702;R-HSA-909719;R-HSA-10
9.08646117285245E-4	9606	Homo sapiens	NFKBIA;UBB;SQSTM1	R-HSA-209536;R-HSA-193703;R-HSA-193
6.989585517578808E-4	9606	Homo sapiens	UBB;HLA-B;HLA-C;SEC61B	R-HSA-8863895;R-HSA-1236947;R-HSA-8
0.03529740686377298	9606	Homo sapiens	NFKBIA;UBB;CEBPD;CCL5	R-HSA-507719;R-HSA-6784160;R-HSA-97
4.193751310547285E-4	9606	Homo sapiens	UBB;B2M	R-HSA-9637635;R-HSA-9636461
3.494792758789404E-4	9606	Homo sapiens	RPL41;UBB;RPL36AL;RPS2	R-HSA-1799326;R-HSA-1799330;R-HSA-1
9.08646117285245E-4	9606	Homo sapiens	UBB;SQSTM1	R-HSA-9664881;R-HSA-9664880;R-HSA-9
0.0023065632208010066	9606	Homo sapiens	UBB;H2AFZ;B2M;ITM2B	R-HSA-976734;R-HSA-5667111;R-HSA-56
3.494792758789404E-4	9606	Homo sapiens	RPL41;UBB;RPL36AL;RPS2	R-HSA-156915;R-HSA-156912;R-HSA-156
6.290626965820927E-4	9606	Homo sapiens	UBB;SQSTM1	R-HSA-193684;R-HSA-193641;R-HSA-193
6.989585517578807E-5	9606	Homo sapiens	RPL41;UBB;RPL36AL;RPS2	R-HSA-927789
6.290626965820927E-4	9606	Homo sapiens	RPL41;UBB;RPL36AL;RPS2	R-HSA-156915;R-HSA-156912;R-HSA-156
0.03396938561543301	9606	Homo sapiens	RPL41;MT1M;RPS27L;CYB	R-HSA-9766677;R-HSA-9766687;R-HSA-8
2.0376282007742986E-4	9606	Homo sapiens	CD44	R-HSA-428287;R-HSA-428296

1.3584188005161992E-4	9606	Homo sapiens	NCOR1;NR3C1;ESR2	R-HSA-382096;R-HSA-376419
0.013516267065136181	9606	Homo sapiens	MT-ND6;EIF4A2;HBS1L;TC	R-HSA-9770132;R-HSA-8849157;R-HSA-9
0.006792094002580996	9606	Homo sapiens	ITGB1;PPP1R15A;PPP1CB	R-HSA-178178;R-HSA-2186785;R-HSA-87
0.002988521361135638	9606	Homo sapiens	ITGB1;PPP1CB;TGFBR3;PI	R-HSA-178178;R-HSA-178215;R-HSA-178
0.001901786320722678	9606	Homo sapiens	PPP1CB;PPP1R15A;STAG1	R-HSA-178178;R-HSA-2167924;R-HSA-17
0.009576852543639203	9606	Homo sapiens	HDAC4;TOP2B;CREBBP;NU	R-HSA-4615872;R-HSA-4641345;R-HSA-4
0.008965564083406914	9606	Homo sapiens	HDAC4;TOP2B;CREBBP;NU	R-HSA-4615872;R-HSA-4641345;R-HSA-4
0.005773279902193847	9606	Homo sapiens	KDM5A;BRD1;CREBBP;PB	R-HSA-4754187;R-HSA-5159245;R-HSA-5
0.005773279902193847	9606	Homo sapiens	KDM5A;BRD1;CREBBP;PB	R-HSA-4754187;R-HSA-5159245;R-HSA-5
9.508931603613394E-4	9606	Homo sapiens	STAT5B;PIK3CA;CNTRL;PI	R-HSA-1839102;R-HSA-1839039;R-HSA-1
8.829722203355294E-4	9606	Homo sapiens	NEK7;LMNA;NUP153;VRK2	R-HSA-5229194;R-HSA-2993898;R-HSA-2
0.001086735040412959	9606	Homo sapiens	EIF4A2;KPNA4;NUP153;AR	R-HSA-1169395;R-HSA-1678843;R-HSA-1
0.008490117503226244	9606	Homo sapiens	ITGB1;PPP1R15A;PPP1CB	R-HSA-178178;R-HSA-2186785;R-HSA-87
3.396047001290498E-4	9606	Homo sapiens	DHX9;DHX36;NFKB1	R-HSA-9604480;R-HSA-9604487;R-HSA-3
0.002309311960877538	9606	Homo sapiens	SRRM2;TCERG1;HNRNPA3	R-HSA-9770132;R-HSA-8849157;R-HSA-9
0.0347755212932147	9606	Homo sapiens	HDAC4;TNKS;YWHAB;ZC3	R-HSA-9664664;R-HSA-1220610;R-HSA-1
2.7168376010323984E-4	9606	Homo sapiens	MT-ND6;MT-ND5;MT-ND2;M	R-HSA-9837087;R-HSA-9837212;R-HSA-9
0.001222576920464579	9606	Homo sapiens	STAT5B;PIK3CA;GOLGB1;F	R-HSA-9698033;R-HSA-9703440;R-HSA-9
1.3584188005161992E-4	9606	Homo sapiens	NIPBL;STAG1;PDS5A	R-HSA-2470935;R-HSA-2468040
6.792094002580996E-5	9606	Homo sapiens	RPL41;RPL30;RPL34;RPLF	R-HSA-927789
1.3584188005161992E-4	9606	Homo sapiens	RPL41;RPL30;RPL34;RPLF	R-HSA-72673;R-HSA-72676
1.3584188005161992E-4	9606	Homo sapiens	RPL41;RPL30;RPL34;RPLF	R-HSA-192704;R-HSA-192841
3.396047001290498E-4	9606	Homo sapiens	RPL41;RPL30;RPL34;RPLF	R-HSA-1799326;R-HSA-1799330;R-HSA-1
2.0376282007742986E-4	9606	Homo sapiens	RPL41;RPL30;RPL34;RPLF	R-HSA-72672;R-HSA-72619;R-HSA-72671
2.0376282007742986E-4	9606	Homo sapiens	RPL41;RPL30;RPL34;RPLF	R-HSA-156832;R-HSA-156823;R-HSA-156
4.0752564015485973E-4	9606	Homo sapiens	RPL41;RPL30;RPL34;RPLF	R-HSA-927836;R-HSA-927789;R-HSA-927
3.396047001290498E-4	9606	Homo sapiens	RPL41;RPL30;RPL34;RPLF	R-HSA-156915;R-HSA-156912;R-HSA-156
3.396047001290498E-4	9606	Homo sapiens	RPL41;RPL30;RPL34;RPLF	R-HSA-927836;R-HSA-927832;R-HSA-927
0.001426339740542009	9606	Homo sapiens	RPL41;RPL30;RPL34;RPLF	R-HSA-72673;R-HSA-72672;R-HSA-15683
3.396047001290498E-4	9606	Homo sapiens	RPL41;RPL30;RPL34;RPLF	R-HSA-141671;R-HSA-141691;R-HSA-141
0.001222576920464579	9606	Homo sapiens	RPL41;RPL30;RPL34;RPLF	R-HSA-72673;R-HSA-72672;R-HSA-72691

6.112884602322896E-4	9606	Homo sapiens	RPL41;RPL30;RPL34;RPLF	R-HSA-156915;R-HSA-156912;R-HSA-156
4.754465801806697E-4	9606	Homo sapiens	RPL41;RPL30;RPL34;RPLF	R-HSA-6791223;R-HSA-6791221;R-HSA-6
2.0376282007742986E-4	9606	Homo sapiens	RPS25;RPS28;RPS27;RPS	R-HSA-9729232
4.754465801806697E-4	9606	Homo sapiens	RPL41;RPL30;RPL34;RPLF	R-HSA-2408529;R-HSA-5333615
0.00672417306255518€	9606	Homo sapiens	RPL41;RPL30;RPL34;RPLF	R-HSA-1799326;R-HSA-156808;R-HSA-15
0.0010867350404129594	9606	Homo sapiens	RPL41;RPL30;RPL34;RPLF	R-HSA-9633008;R-HSA-9633742;R-HSA-9
0.0013584188005161992	9606	Homo sapiens	RPL41;RPL30;RPL34;RPLF	R-HSA-9014652;R-HSA-9011313;R-HSA-8
0.0010188141003871494	9606	Homo sapiens	RPL41;RPL30;RPL34;RPLF	R-HSA-6791223;R-HSA-6791221;R-HSA-6
0.00594114768994198€	9606	Homo sapiens	KDM5A;ATF2;SETD2;KDM3	R-HSA-8865605;R-HSA-4754187;R-HSA-5
0.00594114768994198€	9606	Homo sapiens	KDM5A;ATF2;SETD2;KDM3	R-HSA-8865605;R-HSA-4754187;R-HSA-5
0.01418885860068498	9606	Homo sapiens	YWHAE;ITGB1;CYFIP2;ITS	R-HSA-3858489;R-HSA-2029469;R-HSA-2
0.014817921297267072	9606	Homo sapiens	YWHAE;ITGB1;CYFIP2;ITS	R-HSA-9706390;R-HSA-3858489;R-HSA-5
0.0334102187740267	9606	Homo sapiens	GSK3B;WDR48;MAML2;CL	R-HSA-9664664;R-HSA-5339713;R-HSA-1
9.78541972461033E-4	9606	Homo sapiens	TNRC6C;NCOR2;MECP2;C	R-HSA-9023592;R-HSA-9005995;R-HSA-9
0.006360522820996715	9606	Homo sapiens	CYFIP2;ITGB1;ITSN2;UHRI	R-HSA-9013004;R-HSA-9018766;R-HSA-9
4.193751310547285E-4	9606	Homo sapiens	CYFIP2;ITGB1;DOCK4;TRI	R-HSA-9013145;R-HSA-9013144;R-HSA-9
0.0032152093380862514	9606	Homo sapiens	MAP2K1;CAMK2D;KSR1;AI	R-HSA-6802919;R-HSA-9656211;R-HSA-6
6.989585517578807E-5	9606	Homo sapiens	NCOR2;MECP2;NCOR1;TB	R-HSA-9005585
3.494792758789404E-4	9606	Homo sapiens	NCOR2;MECP2;NCOR1;TB	R-HSA-9005561;R-HSA-9022462;R-HSA-9
3.494792758789404E-4	9606	Homo sapiens	NCOR2;MECP2;NCOR1;TB	R-HSA-9005561;R-HSA-9022462;R-HSA-9
3.494792758789404E-4	9606	Homo sapiens	NCOR2;MECP2;NCOR1;TB	R-HSA-9005561;R-HSA-9022462;R-HSA-9
3.494792758789404E-4	9606	Homo sapiens	NCOR2;MECP2;NCOR1;TB	R-HSA-9005561;R-HSA-9022462;R-HSA-9
3.494792758789404E-4	9606	Homo sapiens	MAP2K1;CAMK2D;KSR1;AI	R-HSA-6802935;R-HSA-6802934;R-HSA-6
0.004123855455371497	9606	Homo sapiens	ATF2;NCOA2;CREBBP;CR	R-HSA-549385;R-HSA-1368087;R-HSA-13
0.0069895855175788075	9606	Homo sapiens	ITGB1;SMAD2;WWTR1;CC	R-HSA-178178;R-HSA-2186785;R-HSA-21
4.892709862305165E-4	9606	Homo sapiens	CREBBP;PRKCB;RUNX2;R	R-HSA-8939066;R-HSA-8938251;R-HSA-8
0.008527294331446146	9606	Homo sapiens	APP;CAMK2D;CUL3;RASG	R-HSA-5687126;R-HSA-5692754;R-HSA-9
0.001957083944922066	9606	Homo sapiens	PPP1CB;SMAD2;USP15;ST	R-HSA-178178;R-HSA-2128994;R-HSA-21
9.78541972461033E-4	9606	Homo sapiens	NCOR2;TNRC6C;CAMK2D;	R-HSA-9023592;R-HSA-8986939;R-HSA-9
0.005381980848535682	9606	Homo sapiens	CAMK2D;PTEN;HTT;OPRM	R-HSA-9615570;R-HSA-9022728;R-HSA-9
0.00594114768994198€	9606	Homo sapiens	SETD3;KMT2C;EHMT1;NSI	R-HSA-8865605;R-HSA-4754187;R-HSA-5

0.005941147689941986	9606	Homo sapiens	SETD3;KMT2C;EHMT1;NSI	R-HSA-8865605;R-HSA-4754187;R-HSA-5
5.591668414063046E-4	9606	Homo sapiens	IKBKB;MAP2K4;MAP2K1;FI	R-HSA-5684275;R-HSA-451649;R-HSA-56
2.0968756552736424E-4	9606	Homo sapiens	TNRC6C;AGO2;TNRC6A;TI	R-HSA-426522;R-HSA-426520;R-HSA-426
1.3979171035157615E-4	9606	Homo sapiens	NCOR2;NCOR1;NR2C2;NR	R-HSA-382096;R-HSA-376419
0.004892709862305165	9606	Homo sapiens	APP;MAP2K4;USP14;MAP2	R-HSA-937034;R-HSA-9750226;R-HSA-93
0.004962605717480953	9606	Homo sapiens	APP;MAP2K4;USP14;MAP2	R-HSA-937034;R-HSA-9750226;R-HSA-93
0.004962605717480953	9606	Homo sapiens	APP;MAP2K4;USP14;MAP2	R-HSA-937034;R-HSA-9750226;R-HSA-93
1.3979171035157615E-4	9606	Homo sapiens	NCOR2;HDAC4;KAT2B;NC	R-HSA-212356;R-HSA-350058
0.0012581253931641854	9606	Homo sapiens	NCOR2;HDAC4;KAT2B;NC	R-HSA-4396371;R-HSA-1980078;R-HSA-1
0.0014678129586915495	9606	Homo sapiens	NCOR2;HDAC4;KAT2B;NC	R-HSA-2220964;R-HSA-2220967;R-HSA-2
0.0014678129586915495	9606	Homo sapiens	NCOR2;HDAC4;KAT2B;NC	R-HSA-2220964;R-HSA-2220967;R-HSA-2
0.0014678129586915495	9606	Homo sapiens	NCOR2;HDAC4;KAT2B;NC	R-HSA-2220964;R-HSA-2220967;R-HSA-2
0.0014678129586915495	9606	Homo sapiens	NCOR2;HDAC4;KAT2B;NC	R-HSA-2220964;R-HSA-2220967;R-HSA-2
0.002725938351855735	9606	Homo sapiens	NCOR2;HDAC4;KAT2B;NC	R-HSA-2220964;R-HSA-2220967;R-HSA-2
6.290626965820927E-4	9606	Homo sapiens	PRKN;PATJ;UBA6;CUL1;UE	R-HSA-983147;R-HSA-983157;R-HSA-983
3.494792758789404E-4	9606	Homo sapiens	SMARCC1;PCGF5;ARID2;A	R-HSA-8878054;R-HSA-8938217;R-HSA-8
0.004193751310547285	9606	Homo sapiens	APP;MAP2K4;USP14;MAP2	R-HSA-975119;R-HSA-9750226;R-HSA-45

5082369;R-HSA-5082384
5082356;R-HSA-5082369;R-HSA-5082384
5082409;R-HSA-5324632
5251959;R-HSA-5324632;R-HSA-5082369;R-HSA-3371518;R-HS
5251959;R-HSA-3371467;R-HSA-5252041;R-HSA-3371518;R-HS

354628;R-HSA-264848;R-HSA-174660;R-HSA-264834;R-HSA-17

562626;R-HSA-5691107
74757;R-HSA-6784622;R-HSA-266303

726621;R-HSA-9725855;R-HSA-8932355;R-HSA-163214;R-HSA-
726621;R-HSA-9725855;R-HSA-8932355;R-HSA-163214;R-HSA-
2197645;R-HSA-2203516;R-HSA-2299677
3217;R-HSA-6799202;R-HSA-6799199;R-HSA-164651;R-HSA-67
5618099;R-HSA-5618098;R-HSA-9725885;R-HSA-9726621;R-HS
9626235;R-HSA-9622840;R-HSA-9624158;R-HSA-9626242;R-HS
6804383;R-HSA-6804253;R-HSA-6804379
67107;R-HSA-5658496;R-HSA-5660752;R-HSA-5660753;R-HSA
9727944

799329;R-HSA-1799335;R-HSA-1799332
5826

913;R-HSA-156923;R-HSA-156907;R-HSA-156910;R-HSA-1569
791221;R-HSA-6791218;R-HSA-6791228;R-HSA-6791227
832;R-HSA-927813;R-HSA-927889
923;R-HSA-156907
673;R-HSA-6800138
813;R-HSA-927889
2;R-HSA-72676;R-HSA-72619;R-HSA-156808;R-HSA-72621;R-
;R-HSA-72676;R-HSA-72697;R-HSA-72619;R-HSA-156808;R-H
790982;R-HSA-6791221;R-HSA-6791218;R-HSA-6791228;R-HS
640167;R-HSA-9639286;R-HSA-9646468;R-HSA-9640168;R-HS
790982;R-HSA-6791221;R-HSA-6791218;R-HSA-6791228;R-HS

622840;R-HSA-9615721;R-HSA-9624158;R-HSA-9622831;R-HS

237325
631068

371422
707504
168884;R-HSA-2168889;R-HSA-2230938

622840;R-HSA-9615721;R-HSA-9631065;R-HSA-9624158;R-HS
168884;R-HSA-2168889;R-HSA-2230938

709360;R-HSA-3371590;R-HSA-9707516;R-HSA-3371503;R-HS
709360;R-HSA-3371590;R-HSA-9707516;R-HSA-3371503;R-HS
707504

799329;R-HSA-1799335;R-HSA-1799332

791221;R-HSA-6791218;R-HSA-6791228;R-HSA-6791227
832;R-HSA-927813;R-HSA-927889
923;R-HSA-156907
813;R-HSA-927889

673
;R-HSA-72676;R-HSA-156823;R-HSA-72697;R-HSA-156826;R-I
;R-HSA-72676;R-HSA-72697;R-HSA-72619;R-HSA-156808;R-H
791221;R-HSA-6791218;R-HSA-6791228;R-HSA-6791227;R-HS
923;R-HSA-156907

4832;R-HSA-163217;R-HSA-6799202;R-HSA-6799199;R-HSA-1
99202;R-HSA-6799199;R-HSA-164651;R-HSA-6799197;R-HSA-

4832;R-HSA-163217;R-HSA-6799202;R-HSA-6799199;R-HSA-1
799199;R-HSA-6799197;R-HSA-6799196;R-HSA-6799179
662621;R-HSA-5662618;R-HSA-5662619;R-HSA-5662617

5662621;R-HSA-5662618;R-HSA-5662619;R-HSA-5662617

7;R-HSA-77078;R-HSA-77081;R-HSA-77083;R-HSA-77069;R-HS

9017441;R-HSA-9017447

8938507;R-HSA-8937134;R-HSA-8937728;R-HSA-8938853;R-HS

84622
4757;R-HSA-174741;R-HSA-174587;R-HSA-6784622

4587

9708557

4587
9618378;R-HSA-9634169;R-HSA-9615945;R-HSA-9615901

439

950

5672304;R-HSA-5626507;R-HSA-5672329
9613213

9613213
9613213

9613213

2520883;R-HSA-2529020
5672304;R-HSA-5626507;R-HSA-5672329

9018794;R-HSA-9018766;R-HSA-9013009;R-HSA-9013024;R-HS

6802910;R-HSA-6803227

5672978;R-HSA-9657606;R-HSA-5672972;R-HSA-5672973;R-HS
9656215;R-HSA-9656214
2731074
173511;R-HSA-216064;R-HSA-4539779;R-HSA-216068;R-HSA-2
8848005;R-HSA-5654612;R-HSA-6785636;R-HSA-5654608;R-HS
2327738;R-HSA-2731075;R-HSA-2731074
216064;R-HSA-216068;R-HSA-2002440;R-HSA-1458433;R-HSA-

40;R-HSA-162363;R-HSA-110011;R-HSA-162425
2327909;R-HSA-2396370;R-HSA-2424243;R-HSA-2424252;R-HS
8875659;R-HSA-8875531
8944247
1454757;R-HSA-1474213;R-HSA-1474210;R-HSA-2473584;R-HS

671;R-HSA-114670

0;R-HSA-162363;R-HSA-110011;R-HSA-162425
425
425

474266;R-HSA-2396234;R-HSA-2213201;R-HSA-2395223;R-HS
023462;R-HSA-1982066;R-HSA-1982065

76416;R-HSA-2128994;R-HSA-2187368;R-HSA-173512;R-HSA-

782;R-HSA-186780;R-HSA-382058

76416;R-HSA-2128994;R-HSA-2187368;R-HSA-173512;R-HSA-

304394;R-HSA-3315483;R-HSA-3656382;R-HSA-3656523

952;R-HSA-167630
465890;R-HSA-2327738;R-HSA-2731075

780;R-HSA-382058
730967;R-HSA-870538;R-HSA-2176491;R-HSA-173481;R-HSA-
76417;R-HSA-2176416;R-HSA-2160931;R-HSA-9730967;R-HS
474213;R-HSA-1454757;R-HSA-1474210;R-HSA-2533874;R-HS
6133;R-HSA-199093;R-HSA-8858428;R-HSA-375131
83709;R-HSA-983707;R-HSA-9606151;R-HSA-983703;R-HSA-1

623281;R-HSA-9623192
4757;R-HSA-6784622;R-HSA-266303
4848;R-HSA-174660;R-HSA-264834;R-HSA-174757;R-HSA-174

690701
690740;R-HSA-1112666;R-HSA-9691421;R-HSA-983709;R-HSA

605051;R-HSA-9607342
295;R-HSA-481007;R-HSA-114600;R-HSA-210291;R-HSA-1145
027425
587
248;R-HSA-202216
423672;R-HSA-156526

727944

826

799329;R-HSA-1799335;R-HSA-1799332

913;R-HSA-156923;R-HSA-156907;R-HSA-156910;R-HSA-1569
791221;R-HSA-6791218;R-HSA-6791228;R-HSA-6791227
832;R-HSA-927813;R-HSA-927889
923;R-HSA-156907

'813;R-HSA-927889
;2;R-HSA-72676;R-HSA-72619;R-HSA-156808;R-HSA-72621;R-I
673
;R-HSA-72676;R-HSA-72697;R-HSA-72619;R-HSA-156808;R-H
i791221;R-HSA-6791218;R-HSA-6791228;R-HSA-6791227;R-HS
)640167;R-HSA-9639286;R-HSA-9646468;R-HSA-9640168;R-HS
i791221;R-HSA-6791218;R-HSA-6791228;R-HSA-6791227;R-HS
l12565;R-HSA-1112727;R-HSA-1112602;R-HSA-1112538;R-HSA
839039;R-HSA-2023451;R-HSA-1839065;R-HSA-5654544;R-HS
783556;R-HSA-1112755;R-HSA-1112565;R-HSA-1112727;R-HSA
839065;R-HSA-5654544;R-HSA-5654545;R-HSA-5655326;R-HS
!023451;R-HSA-1839065;R-HSA-5654544;R-HSA-5654545;R-HS
-33456;R-HSA-1562641;R-HSA-205289;R-HSA-1470012;R-HSA-
i799332;R-HSA-6798372;R-HSA-6797090;R-HSA-5631903;R-HS
839080;R-HSA-1839065;R-HSA-1888198;R-HSA-1839078;R-HS
;950733;R-HSA-8950782;R-HSA-8950210;R-HSA-8950340;R-HS
)670416;R-HSA-9670426;R-HSA-9670431;R-HSA-9670412
i803917;R-HSA-6801675;R-HSA-6803914;R-HSA-6801641
)670416;R-HSA-9670426;R-HSA-9670431;R-HSA-9670412
;983841;R-HSA-8984963;R-HSA-8983845;R-HSA-8950405;R-HS
!023462;R-HSA-1982066;R-HSA-1982065
)80112;R-HSA-4396371;R-HSA-1980078;R-HSA-1980047;R-HSA
i797616;R-HSA-6797712;R-HSA-6806412;R-HSA-6806394
)672172
)672159

)9;R-HSA-2428930;R-HSA-109817;R-HSA-2428926;R-HSA-2428
i082369;R-HSA-5082384
i082356;R-HSA-5082369;R-HSA-5082384
i251959;R-HSA-5324632;R-HSA-5082369;R-HSA-3371518;R-HS
i082409;R-HSA-5324632

5251959;R-HSA-3371467;R-HSA-5252041;R-HSA-3371518;R-HS
5662608;R-HSA-5324632;R-HSA-9726621;R-HSA-9725855;R-HS
5662597;R-HSA-5662595;R-HSA-5662608;R-HSA-5662618;R-HS
9726621;R-HSA-9725855;R-HSA-5618073;R-HSA-5082369;R-HS
5662597;R-HSA-5662595;R-HSA-5662608;R-HSA-5662618;R-HS
5618099;R-HSA-5618098;R-HSA-9725885;R-HSA-9726621;R-HS
9716947;R-HSA-9018497;R-HSA-9018499;R-HSA-8939203;R-HS

9018497;R-HSA-9018499
2197645;R-HSA-2203516;R-HSA-2299677

9626039;R-HSA-9626276;R-HSA-9625188;R-HSA-9626235;R-HS

983307;R-HSA-912527;R-HSA-452100;R-HSA-508292;R-HSA-89
3531;R-HSA-428511
52100;R-HSA-452097;R-HSA-8950415;R-HSA-8983425;R-HSA-8
2058

6799197;R-HSA-6799196;R-HSA-6799179
52100;R-HSA-452097;R-HSA-8950415;R-HSA-8983425;R-HSA-8
8983309;R-HSA-8983374;R-HSA-8983425;R-HSA-8983424;R-HS
3217;R-HSA-6799202;R-HSA-6799199;R-HSA-6799197;R-HSA-1
2058
3877385
8950732;R-HSA-8950415;R-HSA-9617941;R-HSA-8950721;R-HS
8950732;R-HSA-8950415;R-HSA-9617941;R-HSA-8950721;R-HS

)27789;R-HSA-9770129;R-HSA-9770131;R-HSA-72103;R-HSA-9
3984963;R-HSA-8950533;R-HSA-8984964;R-HSA-8950681;R-HS
6798044
527;R-HSA-913374;R-HSA-453104;R-HSA-913424

5662608;R-HSA-5662623;R-HSA-5662621;R-HSA-5662618;R-HS
5662608;R-HSA-5662623;R-HSA-5662621;R-HSA-5662618;R-HS
6785722
'99202;R-HSA-6799199;R-HSA-164651;R-HSA-6799197;R-HSA-
2022396;R-HSA-2022378;R-HSA-2065355;R-HSA-2022379;R-HS
-741;R-HSA-174587;R-HSA-174731
64832;R-HSA-163217;R-HSA-6799202;R-HSA-6799199;R-HSA-1
6799199;R-HSA-6799197;R-HSA-6799196;R-HSA-6799179

'4660;R-HSA-8866304;R-HSA-174731;R-HSA-8866327;R-HSA-2

-757
66310;R-HSA-264678;R-HSA-6784676;R-HSA-174757;R-HSA-26
5334099;R-HSA-5334179;R-HSA-5334164;R-HSA-5334151;R-HS
64832;R-HSA-163217;R-HSA-6799202;R-HSA-6799199;R-HSA-1
3940001;R-HSA-8939667;R-HSA-8939829;R-HSA-8939670;R-HS

)008326;R-HSA-8986294
3985227;R-HSA-8985485;R-HSA-8937869;R-HSA-9008015;R-HS

)863;R-HSA-199839
-741;R-HSA-174587;R-HSA-174731
514414
-757;R-HSA-6784622;R-HSA-266350;R-HSA-176879
-757

-04131;R-HSA-2404140;R-HSA-2395784;R-HSA-2399913;R-HSA
)008015;R-HSA-9007759;R-HSA-9016526;R-HSA-8939904;R-HS
524527

-04131;R-HSA-2404140;R-HSA-2395784;R-HSA-2399913;R-HSA
366308;R-HSA-8866321;R-HSA-174660;R-HSA-8866304;R-HSA-

799329;R-HSA-1799335;R-HSA-1799332

5826

5791221;R-HSA-6791218;R-HSA-6791228;R-HSA-6791227
'832;R-HSA-927813;R-HSA-927889
5923;R-HSA-156907
'813;R-HSA-927889
32;R-HSA-72676;R-HSA-72619;R-HSA-156808;R-HSA-72621;R-H
673
;R-HSA-72676;R-HSA-72697;R-HSA-72619;R-HSA-156808;R-H
5791221;R-HSA-6791218;R-HSA-6791228;R-HSA-6791227;R-HS
5923;R-HSA-156907

759950;R-HSA-9759513
759516;R-HSA-9762333;R-HSA-9759513;R-HSA-9833671;R-HS
759516;R-HSA-9762333;R-HSA-9759513;R-HSA-9833671;R-HS
759516;R-HSA-9762333;R-HSA-9759513;R-HSA-9833671;R-HS
761047;R-HSA-9761120;R-HSA-9760641;R-HSA-9761025;R-HS
759516;R-HSA-9762333;R-HSA-9759513;R-HSA-9833671;R-HS
944214;R-HSA-8944247
874079
4903;R-HSA-216072;R-HSA-2327695;R-HSA-216045
875443;R-HSA-8875490;R-HSA-8875568;R-HSA-8874079;R-HS
759516;R-HSA-9762333;R-HSA-9759513;R-HSA-9833671;R-HS

759516;R-HSA-9762333;R-HSA-9759513;R-HSA-9833671;R-HS
474266;R-HSA-2396234;R-HSA-2213201;R-HSA-2395223;R-HS
564164;R-HSA-1474213;R-HSA-1454757;R-HSA-1474210;R-HS

666383

250462;R-HSA-1250189
665701;R-HSA-9665700;R-HSA-9665705;R-HSA-9665709
963578;R-HSA-1250463
664983;R-HSA-9664950;R-HSA-9664646;R-HSA-9664664;R-HS

)664983;R-HSA-9664950;R-HSA-9664664;R-HSA-9664991;R-HS
;863804;R-HSA-1358792

;785636;R-HSA-1306969;R-HSA-1250463;R-HSA-1250462;R-HS

;912;R-HSA-156923;R-HSA-156907
;912;R-HSA-156913;R-HSA-156923;R-HSA-156907;R-HSA-1569

;826
799329;R-HSA-1799335;R-HSA-1799332

;791221;R-HSA-6791218;R-HSA-6791228;R-HSA-6791227
'832;R-HSA-927813;R-HSA-927889
'813;R-HSA-927889

;2;R-HSA-72676;R-HSA-72619;R-HSA-156808;R-HSA-72621;R-I
673
;R-HSA-72676;R-HSA-72697;R-HSA-72619;R-HSA-156808;R-H
;791221;R-HSA-6791218;R-HSA-6791228;R-HSA-6791227;R-HS
;791221;R-HSA-6791218;R-HSA-6791228;R-HSA-6791227;R-HS

19281;R-HSA-1799326;R-HSA-156808;R-HSA-5389848;R-HSA-
790036;R-HSA-6789615;R-HSA-6789325
100;R-HSA-508292;R-HSA-452097;R-HSA-6784160;R-HSA-451

100;R-HSA-508292;R-HSA-452097;R-HSA-6789615;R-HSA-447
25465
654774
4261;R-HSA-451634;R-HSA-5684275;R-HSA-451649;R-HSA-56

245;R-HSA-452097;R-HSA-447246;R-HSA-450063;R-HSA-4521
087;R-HSA-3296301
877338
4261;R-HSA-451634;R-HSA-5684275;R-HSA-451649;R-HSA-56

669863;R-HSA-9669878;R-HSA-9669855;R-HSA-9669870;R-HS
669900

984963;R-HSA-8987266;R-HSA-1112604;R-HSA-8985988;R-HS
30967;R-HSA-9710963;R-HSA-873927;R-HSA-1031716;R-HSA-
0967;R-HSA-873927;R-HSA-1031716;R-HSA-873921;R-HSA-87
726;R-HSA-913529;R-HSA-909721;R-HSA-909722;R-HSA-9710
462003;R-HSA-1461971;R-HSA-1461982;R-HSA-1462014;R-HS
112604;R-HSA-8987270;R-HSA-936475;R-HSA-5668417;R-HSA
389;R-HSA-912470;R-HSA-912467;R-HSA-912450
458;R-HSA-912389;R-HSA-912503;R-HSA-912470;R-HSA-9124
987150;R-HSA-1112727;R-HSA-1112602;R-HSA-8984963;R-HS
462005;R-HSA-1462003;R-HSA-1461971;R-HSA-1461982;R-HS

;957265;R-HSA-8865498
112602;R-HSA-1112538;R-HSA-1112587;R-HSA-1112604
-9098
021325;R-HSA-9013567;R-HSA-9014129;R-HSA-4395231;R-HS
782655;R-HSA-9679787;R-HSA-3769447
-9098
96960;R-HSA-5690080;R-HSA-5665854;R-HSA-5665871;R-HSA
009488;R-HSA-449117;R-HSA-9014052
641260;R-HSA-3640874;R-HSA-4641256;R-HSA-3451147;R-HS
458;R-HSA-912389;R-HSA-912503;R-HSA-912470;R-HSA-9124
295519;R-HSA-1295516;R-HSA-5668417;R-HSA-9758604;R-HS
662608;R-HSA-5662622;R-HSA-5662621;R-HSA-5662618;R-HS
136;R-HSA-688137;R-HSA-936960;R-HSA-8863895;R-HSA-936
662608;R-HSA-5662622;R-HSA-5662621;R-HSA-5662618;R-HS
705
09719;R-HSA-1031716;R-HSA-1015699;R-HSA-1031713
863973;R-HSA-1236935;R-HSA-1236967;R-HSA-1236964;R-HS
15699
684;R-HSA-193705;R-HSA-193641;R-HSA-193694;R-HSA-2095
863973;R-HSA-1236935;R-HSA-1236965;R-HSA-1236971
50942;R-HSA-1295519;R-HSA-5684250;R-HSA-1295516;R-HSA

799329;R-HSA-1799335;R-HSA-1799332
664855;R-HSA-9664888;R-HSA-9674127;R-HSA-9664892
67107;R-HSA-5658496;R-HSA-5660752;R-HSA-977224;R-HSA-
923;R-HSA-156907
694;R-HSA-209566

923;R-HSA-156907
932355;R-HSA-3788724;R-HSA-9766532;R-HSA-3371554;R-HS

)770129;R-HSA-429955;R-HSA-9770131;R-HSA-9770141;R-HSA
'0479;R-HSA-2160931;R-HSA-2160932;R-HSA-2160935;R-HSA-
3218;R-HSA-2167917;R-HSA-178189;R-HSA-170861;R-HSA-216
'8215;R-HSA-178218;R-HSA-2179291;R-HSA-2187355;R-HSA-2
-719424;R-HSA-4090288;R-HSA-4719448;R-HSA-5228508;R-HS
-719424;R-HSA-4090288;R-HSA-4719448;R-HSA-5228508;R-HS
5244692;R-HSA-3321975;R-HSA-3697008;R-HSA-4754181;R-HS
5244692;R-HSA-3321975;R-HSA-3697008;R-HSA-4754181;R-HS
839080;R-HSA-1839112;R-HSA-1839065;R-HSA-1839114;R-HS
2990880;R-HSA-5244669;R-HSA-2980720;R-HSA-2990882;R-HS
169394;R-HSA-1678842;R-HSA-1169398;R-HSA-1176059;R-HSA
'0479;R-HSA-2160931;R-HSA-2160932;R-HSA-2160935;R-HSA-
3134954;R-HSA-3134953;R-HSA-3134962
)770129;R-HSA-159046;R-HSA-9770131;R-HSA-75079;R-HSA-9
220611;R-HSA-1220614;R-HSA-1839112;R-HSA-9664646;R-HS
)837149;R-HSA-9837179
)703441;R-HSA-9703442;R-HSA-9698043;R-HSA-9698041;R-HS

799329;R-HSA-1799335;R-HSA-1799332

5826
'832;R-HSA-927813;R-HSA-927889
5923;R-HSA-156907
'813;R-HSA-927889
32;R-HSA-72676;R-HSA-72619;R-HSA-156808;R-HSA-72621;R-H
673
;R-HSA-72676;R-HSA-72697;R-HSA-72619;R-HSA-156808;R-H

923;R-HSA-156907
791218

6823;R-HSA-156826;R-HSA-157849;R-HSA-72671;R-HSA-7267
634669;R-HSA-9633005
986083;R-HSA-9014610;R-HSA-9011300
791218
159245;R-HSA-5244692;R-HSA-3321975;R-HSA-3301237;R-HS
159245;R-HSA-5244692;R-HSA-3321975;R-HSA-3301237;R-HS
029466;R-HSA-5668947;R-HSA-3858495;R-HSA-9013004;R-HS
668947;R-HSA-3858495;R-HSA-9018766;R-HSA-5666129;R-HS
839112;R-HSA-9664646;R-HSA-1839114;R-HSA-1839110;R-HS
022314;R-HSA-9022625;R-HSA-9021888;R-HSA-9018594;R-HS
018754;R-HSA-8981637;R-HSA-9018756;R-HSA-9018758;R-HS
013143
802918;R-HSA-9656209;R-HSA-6802916;R-HSA-9652816;R-HS

005585;R-HSA-9022465;R-HSA-9023461
005585;R-HSA-9022465;R-HSA-9023461
005585;R-HSA-9022465;R-HSA-9023461
005585;R-HSA-9022465;R-HSA-9023461
802933;R-HSA-6802932;R-HSA-6802927
68081;R-HSA-400256;R-HSA-400267;R-HSA-5663174;R-HSA-1
60931;R-HSA-2160932;R-HSA-2187303;R-HSA-870538;R-HSA-
939054;R-HSA-8938382;R-HSA-8938228;R-HSA-8938389;R-HS
635739;R-HSA-5692764;R-HSA-9635743;R-HSA-9657603;R-HS
87368;R-HSA-173512;R-HSA-2167917;R-HSA-2187375;R-HSA-
022314;R-HSA-9021183;R-HSA-9021182;R-HSA-9006992;R-HS
615571;R-HSA-9023592;R-HSA-9022314;R-HSA-9022734;R-HS
159245;R-HSA-5244692;R-HSA-3321975;R-HSA-3301237;R-HS

[illegible]159245;R-HSA-5244692;R-HSA-3321975;R-HSA-3301237;R-HS
[illegible]84250;R-HSA-5684267;R-HSA-5684248
[illegible]489

[illegible]7032;R-HSA-451649;R-HSA-9750942;R-HSA-9749471;R-HSA-5
[illegible]7032;R-HSA-451649;R-HSA-9750942;R-HSA-9749471;R-HSA-5
[illegible]7032;R-HSA-451649;R-HSA-9750942;R-HSA-9749471;R-HSA-5

980047;R-HSA-1852623;R-HSA-1912393;R-HSA-1912394;R-HS
[illegible]220966;R-HSA-2220971;R-HSA-4396393;R-HSA-4396392;R-HS
[illegible]220966;R-HSA-2220971;R-HSA-4396393;R-HSA-4396392;R-HS
[illegible]220966;R-HSA-2220971;R-HSA-4396393;R-HSA-4396392;R-HS
[illegible]220966;R-HSA-2220971;R-HSA-4396393;R-HSA-4396392;R-HS
[illegible]220966;R-HSA-2730752;R-HSA-2220971;R-HSA-4396393;R-HS
[illegible]156;R-HSA-983140;R-HSA-983153;R-HSA-983152
[illegible]878050;R-HSA-8937989;R-HSA-8878056
[illegible]1649;R-HSA-9750942;R-HSA-9749471;R-HSA-5684250;R-HSA-

Supplemental Digital Content, Table S3

Transplan	Day	p_val	avg_log2FC	pct.1	pct.2	p_val_adj	Gene
Successfu	0	6.32124430504111e-264	1.7415206761126	0.758	0.087	2.12001891502469e-259	REG1B
Successfu	0	9.51283279754866e-224	1.49651668284256	0.997	0.889	3.19041386364187e-219	IGKC
Successfu	0	6.79260615733857e-222	2.52326209786788	0.963	0.671	2.27810425304821e-217	TXNIP
Successfu	0	9.30593643679813e-165	1.29463856643743	0.987	0.803	3.12102496217336e-160	FABP6
Successfu	0	1.26176904580332e-156	1.37368792952952	0.877	0.449	4.23172102581517e-152	APOA4
Successfu	0	3.65524962399564e-155	1.27322013208564	0.949	0.718	1.22589761889566e-150	PHGR1
Successfu	0	4.63660177085639e-139	1.36846750848522	0.927	0.568	1.55502350190982e-134	FKBP5
Successfu	0	1.3499625337197e-129	1.05922841119322	0.985	0.884	4.52750434558911e-125	FTL
Successfu	0	2.58979929523844e-116	1.01949520133908	0.919	0.623	8.68566887637069e-112	REG1A
Successfu	0	1.46740543818214e-113	1.21503713206254	0.978	0.866	4.92138435857527e-109	IL7R
Successfu	0	1.6498062976966e-111	1.07242358051232	0.939	0.857	5.53312036121487e-107	RPS29
Successfu	0	3.57615748765429e-111	1.25920097652641	0.984	0.926	1.1993716982095e-106	BTG1
Successfu	0	5.35699566241507e-108	1.02173701897403	0.972	0.916	1.79662920526076e-103	RPS27
Successfu	0	3.08250339720598e-102	1.42328551230353	0.816	0.425	1.03380998935494e-97	HSPA1B
Successfu	0	2.21830111906284e-99	0.930175781959035	0.979	0.912	7.43973829311295e-95	RPL39
Successfu	0	9.46916092717756e-97	0.966467918176117	0.967	0.888	3.17576719175681e-92	RPL34
Successfu	0	1.47793348746215e-96	1.03851012460771	0.743	0.363	4.95669333025056e-92	JCHAIN
Successfu	0	7.90063095581452e-95	0.934037109731217	0.985	0.918	2.64971360996108e-90	RPL10
Successfu	0	1.17578547675277e-93	0.900756161429138	0.973	0.895	3.94334933193346e-89	RPL11
Successfu	0	1.38209057062193e-91	0.890097922965751	0.963	0.868	4.63525535575183e-87	RPL30
Successfu	0	1.21418373252957e-90	0.497123504750292	1	1	4.07212940215767e-86	MT-CO2
Successfu	0	1.38796108238745e-90	0.894841747022658	0.952	0.869	4.65494387811101e-86	RPL37
Successfu	0	2.6861144160824e-90	0.886679214568896	0.973	0.91	9.00869052865714e-86	RPL13
Successfu	0	4.94428098917152e-89	0.890968576936417	0.961	0.88	1.65821295814835e-84	RPS15A
Successfu	0	5.03462811435798e-89	0.975474060868341	0.916	0.71	1.68851357699338e-84	SMAP2
Successfu	0	5.33531036331221e-89	0.962119949142893	0.939	0.862	1.78935638964765e-84	RPL21
Successfu	0	2.19530952437619e-88	0.93113873613247	0.96	0.884	7.36262908285287e-84	RPS8
Successfu	0	4.8281417104078e-88	0.868479069293957	0.979	0.901	1.61926216683657e-83	RPL28
Successfu	0	6.34913430272074e-88	0.903754535690349	0.972	0.903	2.12937266244648e-83	RPS18

Successfu	0	6.80481535458261e-88	1.83929841147779	0.773	0.45	2.28219897361992e-83	HSPA1A
Successfu	0	1.78333689356336e-86	0.943575445768075	0.759	0.419	5.98095527363279e-82	APOC3
Successfu	0	8.9532042440116e-86	0.862765264689467	0.969	0.888	3.00272563935661e-81	RPS4X
Successfu	0	2.43359860874044e-85	0.816494916777714	0.973	0.903	8.1618030139937e-81	RPS28
Successfu	0	7.81376423246131e-85	0.943602765471048	0.662	0.285	2.62058024828287e-80	KRT19
Successfu	0	8.75706320340012e-85	0.845235794082932	0.955	0.871	2.93694385715633e-80	RPL9
Successfu	0	3.40828405022656e-84	0.81748221021338	0.981	0.924	1.14307030476498e-79	RPL41
Successfu	0	4.72553398424694e-84	0.861425645999453	0.951	0.866	1.58484958763674e-79	RPL3
Successfu	0	1.87468477909505e-83	1.34141199723165	0.851	0.603	6.28731781212897e-79	CXCR4
Successfu	0	4.98257248539348e-83	0.830709916578049	0.984	0.896	1.67105516015127e-78	RPS14
Successfu	0	5.2089287868431e-83	0.898211219441255	0.922	0.828	1.74697053653144e-78	RPL35A
Successfu	0	2.25687239622957e-82	1.28207203489286	0.975	0.948	7.56909864247474e-78	HSP90AA1
Successfu	0	1.70522394090619e-81	0.812604299341551	0.975	0.905	5.71898005301116e-77	RPS19
Successfu	0	3.63980503922273e-81	0.820517948090755	0.949	0.861	1.22071781405452e-76	RPL29
Successfu	0	1.3550558392609e-80	0.870875625127453	0.979	0.92	4.54458627371322e-76	RPS12
Successfu	0	3.44462550475397e-80	0.980283554504109	0.903	0.768	1.15525850178439e-75	UBB
Successfu	0	1.38590844091044e-79	0.836402616479869	0.954	0.851	4.64805972912545e-75	RPS25
Successfu	0	1.95372581567196e-79	0.899546229350654	0.564	0.206	6.55240564060062e-75	GUCA2A
Successfu	0	8.04536536355633e-79	0.851366356517673	0.952	0.873	2.69825463562952e-74	RPS27A
Successfu	0	2.98398423191916e-78	0.762020895089413	0.963	0.886	1.00076863170105e-73	RPLP2
Successfu	0	4.17214568630631e-77	0.874180111400313	0.925	0.819	1.39925422027341e-72	RPL36
Successfu	0	4.20466842075256e-77	0.746172415874139	0.984	0.921	1.41016169495199e-72	RPS2
Successfu	0	7.43526651278536e-76	1.36476345567952	0.883	0.676	2.49363968305795e-71	DNAJB1
Successfu	0	7.59130764852582e-76	0.813288160887617	0.925	0.839	2.54597275916259e-71	RPL35
Successfu	0	1.08170706510493e-75	0.818419181687976	0.958	0.883	3.62782915494893e-71	RPL12
Successfu	0	1.29037114899186e-74	0.971291704470289	0.581	0.24	4.32764675948891e-70	PDE3A
Successfu	0	1.74924326267898e-74	0.820849481221858	0.951	0.874	5.86661205437277e-70	RPL32
Successfu	0	1.00769973383022e-73	0.797237292702982	0.94	0.848	3.3796233673198e-69	RPS13
Successfu	0	1.44598279022435e-73	0.78477525410516	0.952	0.877	4.84953708185441e-69	RPS15
Successfu	0	2.50401604954683e-73	0.824144401625591	0.91	0.799	8.39796902697016e-69	RPL22
Successfu	0	9.14044101513123e-73	0.798935428649671	0.922	0.809	3.06552110765471e-68	FAU

Successfu	0	1.09160301785839e-72	0.781388064265939	0.934	0.853	3.66101820129347e-68	RPS7
Successfu	0	1.17278999612518e-72	0.892763691096623	0.901	0.767	3.93330308900462e-68	RPS21
Successfu	0	3.51594846311533e-72	0.763341033314785	0.945	0.844	1.17917879555962e-67	RPL8
Successfu	0	5.86505098753248e-72	1.24578906874078	0.827	0.586	1.96702080019864e-67	CRYBG1
Successfu	0	2.35711089273478e-71	0.836764904196719	0.83	0.569	7.90527851205392e-67	ALDOB
Successfu	0	7.68381970844898e-70	0.740093610585344	0.964	0.866	2.57699945381962e-65	RPS23
Successfu	0	1.81517289257693e-69	0.797682202418339	0.927	0.814	6.0877268471245e-65	UBA52
Successfu	0	8.69751497836322e-69	0.757924841895438	0.933	0.846	2.91697257344346e-64	RPL27A
Successfu	0	8.80980473981201e-69	0.843181270266889	0.816	0.544	2.95463231363815e-64	FABP1
Successfu	0	1.12264421705839e-68	0.776872184881485	0.874	0.665	3.76512417517044e-64	IGHA1
Successfu	0	2.93376417828664e-68	0.744511205362725	0.958	0.884	9.83925830113773e-64	RPS3A
Successfu	0	4.02805716326436e-68	0.727428439867488	0.982	0.945	1.3509298114156e-63	RPLP1
Successfu	0	8.36554009462974e-68	0.803851995338325	0.927	0.783	2.80563483693692e-63	RPS26
Successfu	0	1.06135925528603e-67	0.780476190808641	0.425	0.124	3.55958667037829e-63	CYP3A4
Successfu	0	1.0769153271266e-66	0.711207697174298	0.942	0.853	3.61175862411719e-62	RPL15
Successfu	0	1.84060404802021e-66	0.651634731030853	0.994	0.967	6.17301785625019e-62	TPT1
Successfu	0	6.11748673485158e-66	0.715036544994796	0.937	0.861	2.05168270113452e-61	RPS20
Successfu	0	1.09219119875911e-65	0.830547239808589	0.939	0.845	3.66299084239831e-61	UBC
Successfu	0	4.96898284710333e-64	0.732416246768584	0.916	0.813	1.66649746726152e-59	RPL37A
Successfu	0	3.13731079443084e-62	0.358745445133752	1	1	1.05219129423622e-57	MT-CO3
Successfu	0	3.76531193519038e-62	0.704019506656723	0.939	0.855	1.26281031682415e-57	RPL18A
Successfu	0	3.81787115246584e-62	0.64974839611781	0.954	0.9	1.28043762711399e-57	RPL13A
Successfu	0	1.1200334514272e-61	0.928620273404831	0.966	0.896	3.75636818939655e-57	CCL5
Successfu	0	2.05827762409734e-61	0.804623250529718	0.984	0.92	6.90305149569764e-57	FTH1
Successfu	0	7.03396942431111e-61	0.723842294633706	0.717	0.402	2.35905266552546e-56	IGLC2
Successfu	0	1.76369561802127e-60	0.740068195700257	0.912	0.789	5.91508236371974e-56	RPL10A
Successfu	0	2.66840868343063e-60	0.675119277694685	0.949	0.872	8.94930904248966e-56	RPL18
Successfu	0	4.15640151301827e-60	0.733725212964503	0.907	0.771	1.39397393943607e-55	RPS5
Successfu	0	1.98732438125846e-59	0.639395206869778	0.958	0.868	6.66508850986463e-55	RPS16
Successfu	0	5.68796933461246e-59	0.700002208288447	0.933	0.849	1.90763115544233e-54	RPL19
Successfu	0	5.75595070064551e-59	0.901828836137361	0.586	0.299	1.93043074598249e-54	PIK3IP1

Successfu	0	2.7855062772233e-58	0.68366344536683[illegible]	0.943	0.877	9.34203095255149e-54	RPS3
Successfu	0	6.91103651122622e-58	0.67830992374248[illegible]	0.925	0.824	2.31782342513505e-53	NACA
Successfu	0	2.47644067255584e-56	0.71957761200123[illegible]	0.894	0.744	8.30548672761776e-52	PFDN5
Successfu	0	3.22840407934333e-56	0.73624235596560[illegible]	0.876	0.737	1.08274216013017e-51	EEF1D
Successfu	0	2.31072332023134e-55	0.78466001942243[illegible]	0.964	0.91	7.74970387139188e-51	H3F3B
Successfu	0	6.09225278406137e-55	0.89517858476217[illegible]	0.833	0.618	2.0432197387185e-50	HSPH1
Successfu	0	2.0913273912069e-54	0.38846304410364[illegible]	1	1	7.01389380462971e-50	MT-ATP6
Successfu	0	7.45740888712956e-54	0.68149512028798[illegible]	0.978	0.94	2.50106579256551e-49	PTMA
Successfu	0	3.07115663532221e-53	0.59833678828961[illegible]	0.948	0.865	1.03000451235436e-48	RPS11
Successfu	0	3.49571192608109e-53	0.79847806396776[illegible]	0.513	0.23	1.17239186576908e-48	SLC5A1
Successfu	0	7.16912009379438e-53	0.68812259415591[illegible]	0.502	0.211	2.40437949705676e-48	HLA-DRA
Successfu	0	4.27405101380417e-52	0.69203845763070[illegible]	0.854	0.683	1.43343122900964e-47	TOMM7
Successfu	0	7.26249564544326e-52	0.85718645098624[illegible]	0.96	0.835	2.43569578956876e-47	TSC22D3
Successfu	0	3.09691747557838e-51	0.61235731094940[illegible]	0.925	0.85	1.03864418295948e-46	RPL24
Successfu	0	3.47701919539037e-51	0.53963452553727[illegible]	0.996	0.982	1.16612269775002e-46	EEF1A1
Successfu	0	4.054619054038e-51	0.62756733052976[illegible]	0.946	0.875	1.35983813834326e-46	RPL7A
Successfu	0	4.42530228536401e-51	0.67223682276602[illegible]	0.909	0.815	1.48415788046538e-46	RPL23A
Successfu	0	3.46879877416528e-50	0.67340059629907[illegible]	0.883	0.715	1.16336573287955e-45	EEF1B2
Successfu	0	8.64562222588968e-50	0.68818325908628[illegible]	0.496	0.218	2.89956878211888e-45	ADIRF
Successfu	0	2.74082126574087e-49	0.67909192833068[illegible]	0.604	0.304	9.19216636104172e-45	IGLC3
Successfu	0	2.79763130452044e-49	0.59764484595537[illegible]	0.958	0.912	9.38269586910065e-45	RPS6
Successfu	0	4.26669911935958e-49	0.57612801080355[illegible]	0.925	0.858	1.43096555065082e-44	RPS9
Successfu	0	4.38840495354555e-49	0.89217339457655[illegible]	0.62	0.362	1.47178325332011e-44	AC058791.1
Successfu	0	5.82421021485081e-49	0.66499259176838[illegible]	0.907	0.809	1.95332362185666e-44	RPL26
Successfu	0	9.97360392051632e-49	0.65600900244022[illegible]	0.886	0.774	3.34494728286276e-44	RPL7
Successfu	0	1.11129087437853e-48	0.74897002417488[illegible]	0.575	0.31	3.72704733449072e-44	GDA
Successfu	0	1.44700738781841e-48	0.39345847738568[illegible]	1	0.999	4.85297337726537e-44	MT-CYB
Successfu	0	6.311151015693e-48	0.61224696551602[illegible]	0.909	0.817	2.11663382764312e-43	RACK1
Successfu	0	3.15656203684908e-47	0.60178764383183[illegible]	0.949	0.872	1.05864777591845e-42	RPL6
Successfu	0	4.03642223986233e-47	0.68451543739579[illegible]	0.484	0.214	1.35373529080503e-42	FGD4
Successfu	0	1.67433277478561e-46	0.61590459490968[illegible]	0.942	0.879	5.61537726007597e-42	RPS24

Successfu	0	2.24118938628889e-44	0.657640090073313	0.441	0.187	7.51650096373569e-40	MUC17
Successfu	0	3.97717312522076e-44	0.641704734542328	0.876	0.747	1.33386432273654e-39	RPL38
Successfu	0	3.46068823909458e-43	0.599493955039721	0.81	0.607	1.16064562162754e-38	CD74
Successfu	0	3.85069531923191e-43	0.954490715077563	0.523	0.256	1.291446196164e-38	APBA2
Successfu	0	3.89836685752961e-43	0.695982006200722	0.643	0.39	1.30743427667828e-38	SELENOP
Successfu	0	7.17099616081215e-43	0.791165430697321	0.927	0.858	2.40500869241318e-38	HSP90AB1
Successfu	0	2.33533858826765e-42	0.641270201375478	0.848	0.73	7.83225855733205e-38	RPL31
Successfu	0	4.72561959539397e-42	0.440553837053918	1	0.988	1.58487829990323e-37	B2M
Successfu	0	6.53687647293181e-42	0.625338582987566	0.785	0.573	2.19233763149187e-37	MTRNR2L8
Successfu	0	1.46772330441096e-41	0.531949785159558	0.268	0.068	4.92245041833348e-37	BFSP2-AS1
Successfu	0	2.63076058732771e-41	0.570905950983676	0.892	0.775	8.82304485777969e-37	RPL27
Successfu	0	3.1920898074059e-41	0.625114658254522	0.731	0.483	1.07056307960779e-36	PCSK5
Successfu	0	4.42630894051846e-41	0.614351594235752	0.65	0.408	1.48449549247108e-36	KRT8
Successfu	0	1.97041659899888e-40	0.610597661456459	0.454	0.21	6.60838318972245e-36	CLDN4
Successfu	0	2.01108316777275e-40	0.651440193491516	0.596	0.349	6.74477072807626e-36	FABP2
Successfu	0	3.66800796710878e-40	0.615131309760072	0.843	0.701	1.23017651200894e-35	COX7C
Successfu	0	8.9330483059582e-40	0.639617485384095	0.879	0.757	2.99596574085226e-35	RPL23
Successfu	0	5.81157067741442e-39	0.695110430683009	0.928	0.855	1.94908457379125e-34	ZFP36L2
Successfu	0	6.13775894346719e-39	0.524653242112596	0.924	0.828	2.05848159446003e-34	RPLP0
Successfu	0	6.48887190279487e-39	0.70259475391512	0.466	0.209	2.17623785875934e-34	ZBTB16
Successfu	0	1.26817972347053e-38	0.597719433853121	0.827	0.666	4.25322115657547e-34	COX4I1
Successfu	0	2.07932342756758e-38	0.523016467405211	0.945	0.876	6.97363491137617e-34	RPL14
Successfu	0	2.43292824276652e-38	0.663155900305759	0.762	0.615	8.15955474059036e-34	CIRBP
Successfu	0	4.2195925739427e-38	0.499711130867262	0.957	0.903	1.4151669574489e-33	TMSB10
Successfu	0	4.60438488568137e-38	0.625759232884704	0.493	0.255	1.54421860295982e-33	NEDD4L
Successfu	0	5.86445770414158e-38	0.560246740339019	0.354	0.137	1.966821824815e-33	PMP22
Successfu	0	6.61214052957887e-38	0.79886604939447	0.62	0.399	2.21757969081016e-33	H1FX
Successfu	0	1.91352921959653e-37	0.43576646611311	0.984	0.934	6.41759429668283e-33	TMSB4X
Successfu	0	2.6070391104982e-37	0.628257911100963	0.619	0.387	8.74348776878887e-33	LGALS3
Successfu	0	2.89587943295257e-37	0.647749526046098	0.556	0.325	9.71220044223633e-33	CYP3A5
Successfu	0	8.86559345140095e-37	0.672238849138188	0.712	0.524	2.97334273173085e-32	ISG20

Successfu	0	2.02080767158897e-36	0.69690698533413[illegible]	0.957	0.884	6.77738476897507e-32	JUN
Successfu	0	2.78873426450755e-36	0.58889390620708[illegible]	0.244	0.064	9.35285697630541e-32	BFSP2
Successfu	0	3.76358703978398e-36	0.57573578518548[illegible]	0.964	0.945	1.26223182140275e-31	SRGN
Successfu	0	1.15719675711244e-35	0.47832143076511[illegible]	0.924	0.87	3.88100648400369e-31	RPL5
Successfu	0	2.74798372775755e-35	0.76505066189610[illegible]	0.643	0.437	9.21618782615326e-31	GPR171
Successfu	0	1.93412237038779e-34	0.85409391800738[illegible]	0.886	0.786	6.48665960580657e-30	JUNB
Successfu	0	3.07546367543049e-34	0.68860364394851[illegible]	0.837	0.696	1.03144900746588e-29	CD52
Successfu	0	7.07929978598773e-34	0.53105998393764	0.303	0.109	2.37425556222457e-29	CLDN3
Successfu	0	1.17296157319973e-33	0.56726916599222[illegible]	0.868	0.758	3.93387852419725e-29	CD3D
Successfu	0	4.02954005452179e-33	0.87721301647168[illegible]	0.874	0.753	1.35142714348552e-28	LINC00513
Successfu	0	7.28989987936504e-33	0.62157769314181[illegible]	0.88	0.812	2.44488662154145e-28	PNRC1
Successfu	0	1.81663910522679e-32	0.58298469113715[illegible]	0.495	0.278	6.09264423110962e-28	SLC15A1
Successfu	0	2.18757804473684e-32	0.44013416038753[illegible]	0.897	0.796	7.33669924643843e-28	SERF2
Successfu	0	2.56485115450618e-32	0.63317047717922[illegible]	0.931	0.895	8.60199780198284e-28	DNAJA1
Successfu	0	3.04322633962061e-32	0.48736090155520[illegible]	0.299	0.11	1.02063724978196e-27	SMIM3
Successfu	0	4.50116748973164e-32	0.79745091617793[illegible]	0.797	0.676	1.5096015527062e-27	ID2
Successfu	0	5.25012179818674e-32	0.47724160684746[illegible]	0.259	0.085	1.76078584867587e-27	TMPRSS2
Successfu	0	1.07327778301939e-31	0.47681646806471[illegible]	0.882	0.766	3.59955902869043e-27	GAPDH
Successfu	0	1.10604390897289e-31	0.46328666967793[illegible]	0.894	0.779	3.70945006191328e-27	MTRNR2L12
Successfu	0	7.38819746910916e-31	0.50615236105233[illegible]	0.286	0.104	2.47785366718983e-26	SERPINA1
Successfu	0	9.53661615330541e-31	0.47521887865959[illegible]	0.937	0.869	3.19839032549557e-26	HLA-C
Successfu	0	1.84696116362162e-30	0.43874175672182[illegible]	0.241	0.077	6.1943383505542e-26	TM4SF20
Successfu	0	2.04103756210879e-30	0.54918148254578	0.502	0.29	6.84523177580046e-26	RBP2
Successfu	0	4.45244424019334e-30	0.78378659886077[illegible]	0.578	0.384	1.49326074927604e-25	PRDM1
Successfu	0	5.33596665576003e-30	0.68185889014073[illegible]	0.731	0.587	1.7895764970088e-25	CDV3
Successfu	0	5.6793927400258e-30	0.55585093798946[illegible]	0.71	0.545	1.90475473714985e-25	COMMD6
Successfu	0	7.32246085179403e-30	0.44462709534506[illegible]	0.904	0.829	2.45580692047468e-25	ITM2B
Successfu	0	1.43119232892591e-29	0.51254347101177	0.401	0.198	4.79993283275173e-25	CST3
Successfu	0	2.05971572816703e-29	0.53587589648430[illegible]	0.661	0.475	6.90787460912659e-25	ANPEP
Successfu	0	3.52690870268309e-29	0.76528708547583	0.791	0.651	1.18285464070586e-24	KIF13B
Successfu	0	3.97345503668027e-29	0.44218145714663[illegible]	0.275	0.101	1.33261735020183e-24	PRAP1

Successfu	0	4.45848679980202e-29	0.33406799913743	0.176	0.04	1.4952873029176e-24	BANK1
Successfu	0	5.78382137573476e-29	0.256136073438841	1	0.994	1.93977801299392e-24	MT-ND3
Successfu	0	6.48080460647447e-29	0.475038586969415	0.401	0.19	2.17353224891941e-24	IFI27
Successfu	0	2.23639598171853e-28	0.254482511424662	0.127	0.019	7.50042484348762e-24	IGHG4
Successfu	0	3.39039615889681e-28	0.281128525697367	0.132	0.021	1.13707106377081e-23	LCN2
Successfu	0	3.63843846629502e-28	0.290421039261563	0.135	0.022	1.22025949282602e-23	CIDEC
Successfu	0	4.35467733179279e-28	0.485932233436975	0.365	0.171	1.46047168353666e-23	TMC5
Successfu	0	8.079342121242e-28	0.50356424649198	0.691	0.52	2.70964976062214e-23	PEBP1
Successfu	0	2.54980527692626e-27	0.476779457233777	0.342	0.157	8.55153693775529e-23	DPEP1
Successfu	0	6.90279715162932e-27	0.593672338199613	0.417	0.223	2.31506010871344e-22	SOCS1
Successfu	0	1.43369464415362e-26	0.456523656136693	0.84	0.733	4.80832509756241e-22	BTF3
Successfu	0	1.98652416170912e-26	0.461865172172637	0.274	0.108	6.66240473354003e-22	PDE9A
Successfu	0	2.27081324598395e-26	0.408149478563891	0.96	0.941	7.61585346438096e-22	EIF1
Successfu	0	2.81729527897056e-26	0.262440199686338	1	0.999	9.44864490661148e-22	MT-ND4
Successfu	0	3.03653191108134e-26	0.638633286123356	0.607	0.395	1.01839207233846e-21	KLRD1
Successfu	0	4.71968588494211e-26	0.540592400659377	0.457	0.27	1.58288825209188e-21	CHCHD10
Successfu	0	2.12149916159303e-25	0.553377237890512	0.921	0.843	7.11508388815072e-21	DUSP1
Successfu	0	2.2778275186819e-25	0.58508541910339	0.544	0.367	7.63937793215536e-21	HERPUD1
Successfu	0	2.3686848202583e-25	0.619283502216411	0.816	0.727	7.94409515018227e-21	ZFP36
Successfu	0	2.40717020958713e-25	0.508022802185515	0.399	0.214	8.07316744891333e-21	IGHM
Successfu	0	3.6486248404544e-25	0.451474232304353	0.873	0.77	1.2236757989916e-20	ATP5F1E
Successfu	0	4.03971020977739e-25	0.708156582931996	0.354	0.172	1.35483801015514e-20	TXK
Successfu	0	4.07312239873471e-25	0.499446126601624	0.625	0.453	1.36604379008765e-20	PPDPF
Successfu	0	4.65872399638156e-25	0.502943656298928	0.471	0.283	1.56244285390645e-20	EPCAM
Successfu	0	1.0202025622673e-24	0.469994986478011	0.405	0.222	3.42155535333208e-20	MUC13
Successfu	0	1.04588252856875e-24	0.461194283679658	0.375	0.19	3.50768082431386e-20	TMIGD2
Successfu	0	2.29289383168677e-24	0.334733728017192	0.211	0.07	7.68990733271108e-20	GUCA2B
Successfu	0	4.15028646306816e-24	0.554051703009399	0.698	0.559	1.3919230739838e-19	HSPE1
Successfu	0	4.71422477643886e-24	0.493070170328898	0.386	0.207	1.58105670552206e-19	ELF3
Successfu	0	6.88521180093251e-24	0.389128360464781	0.901	0.826	2.30916233379675e-19	RPSA
Successfu	0	9.62209732866732e-24	0.438504654290764	0.303	0.137	3.22705900208845e-19	HHLA2

Successfu	0	9.68603915959495e-24	0.40331142757222	0.29	0.124	3.24850381334495e-19	FRMD3
Successfu	0	1.40961922689094e-23	0.55361415057729	0.351	0.162	4.72758096314683e-19	GFPT2
Successfu	0	2.26212320423964e-23	0.43255275030691	0.36	0.188	7.58670880237889e-19	THSD4
Successfu	0	2.59689084778606e-23	0.55765012142804	0.408	0.232	8.7094525253049e-19	DNAJB4
Successfu	0	2.93619582803929e-23	0.51561127873095	0.661	0.512	9.84741356807817e-19	SOD1
Successfu	0	2.99089898639922e-23	0.40182110802335	0.848	0.738	1.00308770205857e-18	HINT1
Successfu	0	4.46231524446546e-23	0.53380517471756	0.562	0.363	1.49657128668883e-18	CD8B
Successfu	0	4.52854191448138e-23	0.49119003122409	0.848	0.771	1.51878238727877e-18	NPM1
Successfu	0	6.89132999982658e-23	0.68234619591080	0.525	0.376	2.31121425534184e-18	UBE2S
Successfu	0	1.03612880992295e-22	0.43768315381803	0.378	0.197	3.47496880271959e-18	HLA-DPB1
Successfu	0	1.45218416066883e-22	0.82380226828149	0.803	0.695	4.87033523805114e-18	RGS1
Successfu	0	1.53406597693106e-22	0.42969508738446	0.674	0.502	5.1449504734314e-18	EIF3K
Successfu	0	1.55678683423806e-22	0.52048295287017	0.607	0.423	5.2211516846676e-18	IPCEF1
Successfu	0	1.61533234413893e-22	0.38865478633639	0.293	0.129	5.41750161577314e-18	HLA-DQB1
Successfu	0	3.12612656297652e-22	0.49892972486682	0.511	0.334	1.04844032669107e-17	ACSL5
Successfu	0	3.23498409500252e-22	0.45654781785855	0.341	0.175	1.08494896578195e-17	PCK1
Successfu	0	3.90660601274136e-22	0.62722142588027	0.535	0.365	1.3101975245532e-17	UGP2
Successfu	0	4.31263684341295e-22	0.52299552608149	0.537	0.375	1.44637214454383e-17	SNHG8
Successfu	0	4.78026956269722e-22	0.50344615069873	0.871	0.798	1.60320680593739e-17	HSPA8
Successfu	0	5.46973014305312e-22	0.46495664166944	0.77	0.659	1.83443809537716e-17	ZFAS1
Successfu	0	8.97098458448422e-22	0.44407348630807	0.819	0.715	3.00868880994432e-17	ATP5MG
Successfu	0	9.52032339414434e-22	0.40509494932752	0.426	0.239	3.19292605992813e-17	HLA-DRB1
Successfu	0	9.88446921862095e-22	0.50689565493128	0.614	0.416	3.31505328654109e-17	UBASH3B
Successfu	0	1.74379077248868e-21	0.43828376058568	0.834	0.743	5.84832549277252e-17	RPL4
Successfu	0	3.3225532489892e-21	0.46706785410938	0.616	0.47	1.114317908646e-16	ATP5ME
Successfu	0	5.74341494701779e-21	0.48451000925745	0.71	0.595	1.92622650493083e-16	UQCRB
Successfu	0	5.91396488402091e-21	0.34218721204668	0.212	0.079	1.98342554280293e-16	HLA-DRB5
Successfu	0	6.03922382288249e-21	0.37285085509415	0.221	0.086	2.02543488571833e-16	FXYD3
Successfu	0	1.52875336731349e-20	0.46399865329523	0.33	0.176	5.12713304329597e-16	AMN
Successfu	0	1.66040490657055e-20	0.47333513066656	0.42	0.249	5.5686659756563e-16	LGALS4
Successfu	0	2.86124690584939e-20	0.42931291832405	0.57	0.402	9.59604987283768e-16	C12orf57

Successfu	0	3.45810082885532e-20	0.46854786265374	0.978	0.949	1.1597778559815e-15	JUND
Successfu	0	5.10350897217058e-20	0.46510047101204	0.765	0.65	1.71161483908657e-15	CD3G
Successfu	0	5.63933973252489e-20	0.28768471605771	0.132	0.033	1.8913217594942e-15	IGHG1
Successfu	0	7.2073828730953e-20	0.48842492583756	0.975	0.969	2.4172120679787e-15	KLF6
Successfu	0	8.62471262070304e-20	0.46680054807085	0.704	0.522	2.89255611873139e-15	CD8A
Successfu	0	2.8360235636278e-19	0.42404318757263	0.552	0.393	9.51145582769492e-15	UQCR11
Successfu	0	3.36762456495306e-19	0.39451957782052	0.662	0.524	1.12943392659396e-14	SSR4
Successfu	0	7.70849330547657e-19	0.33507941142390	0.132	0.035	2.58527448479073e-14	AC018754.1
Successfu	0	1.01022369847343e-18	0.42946783816645	0.694	0.57	3.3880882399402e-14	ATP5MC2
Successfu	0	1.13019957948295e-18	0.411158123947028	0.327	0.175	3.79046334966992e-14	ID1
Successfu	0	1.2407232119403e-18	0.34566272161468	0.297	0.147	4.16113750820537e-14	HLA-DPA1
Successfu	0	1.86652420069481e-18	0.70532745441280	0.689	0.565	6.25994886429027e-14	KLRB1
Successfu	0	2.0246969271768e-18	0.32146364920964	0.182	0.067	6.79042855436556e-14	PDK4
Successfu	0	2.81454966177481e-18	0.35922806747451	0.236	0.104	9.43943665566037e-14	TMIGD1
Successfu	0	3.78140797306315e-18	0.40627714826302	0.426	0.268	1.26820860600592e-13	TSPAN8
Successfu	0	4.29877477562596e-18	0.41935679142528	0.589	0.455	1.44172308424944e-13	EDF1
Successfu	0	6.6263474360611e-18	0.41194818055606	0.303	0.161	2.22234440310617e-13	SHROOM3
Successfu	0	6.65270145550174e-18	0.62991124828734	0.746	0.599	2.23118301414617e-13	ATF3
Successfu	0	6.97412241269638e-18	0.26951277469436	0.211	0.086	2.33898117477011e-13	SLC7A9
Successfu	0	7.80898761029193e-18	0.35746959680337	0.227	0.101	2.61897826473971e-13	MYO15B
Successfu	0	7.91779656828378e-18	0.38186298823188	0.438	0.27	2.65547061307101e-13	MTRNR2L1
Successfu	0	8.01480110305323e-18	0.48885664490375	0.8	0.698	2.68800399394199e-13	TRBC2
Successfu	0	1.39356491495439e-17	0.33781673887098	0.909	0.825	4.67373801177405e-13	CYTIP
Successfu	0	1.52024467201924e-17	0.40178120533494	0.526	0.372	5.09859658101812e-13	MZT2B
Successfu	0	1.594023117616e-17	0.35831361481156	0.717	0.584	5.34603473186053e-13	RPS10
Successfu	0	1.66852525172101e-17	0.42524397722582	0.345	0.2	5.59589998922191e-13	SMIM24
Successfu	0	1.76513882920501e-17	0.71439736714150	0.541	0.406	5.91992260538776e-13	CITED2
Successfu	0	1.79370418763466e-17	0.44416196526028	0.584	0.419	6.01572510448912e-13	SLA
Successfu	0	1.83950212824795e-17	0.29719158187504	0.143	0.046	6.16932223771797e-13	MEF2C
Successfu	0	7.15831859482497e-17	0.39790946832993	0.656	0.536	2.4007568903324e-12	RBM3
Successfu	0	1.55157528242184e-16	0.37978984107491	0.812	0.726	5.20367318218638e-12	CD3E

Successfu	0	1.61463900226848e-16	0.42527939196572	0.374	0.225	5.41517628580804e-12	GSTA1
Successfu	0	1.73076141884686e-16	0.33453528345779	0.877	0.821	5.80462764652861e-12	HMGB1
Successfu	0	1.87278167441488e-16	0.43041714955246	0.423	0.275	6.28093517965262e-12	CYB5A
Successfu	0	2.29441890294476e-16	0.377806111358603	0.439	0.294	7.69502211669613e-12	GPX4
Successfu	0	2.54071259985137e-16	0.323983869689114	0.9	0.85	8.52104191738151e-12	H3F3A
Successfu	0	2.59769567037023e-16	0.62442748864548	0.572	0.416	8.71215173928767e-12	SPRY1
Successfu	0	3.42551558977349e-16	0.42233254654329	0.589	0.455	1.14884941849823e-11	LAPTM5
Successfu	0	3.96597497399402e-16	0.54967856283990	0.326	0.187	1.33010868677811e-11	GPR34
Successfu	0	4.17327521490563e-16	0.37405287456967	0.532	0.389	1.39963304157505e-11	ATP5F1D
Successfu	0	4.34557625945244e-16	0.46415413236759	0.882	0.805	1.45741936589516e-11	CCNH
Successfu	0	4.93307494386057e-16	0.35730996626091	0.985	0.957	1.65445467467196e-11	CD69
Successfu	0	5.83148719795982e-16	0.37936805546779	0.611	0.483	1.95576417645176e-11	CIB1
Successfu	0	5.88905599740244e-16	0.37027583390835	0.374	0.228	1.97507160040883e-11	DAB1
Successfu	0	6.83570677912732e-16	0.38842584850508	0.407	0.257	2.29255933958372e-11	EPS8
Successfu	0	7.11109820163454e-16	0.39465018997004	0.486	0.339	2.38492011486419e-11	TEX14
Successfu	0	8.2404315937963e-16	0.38108610649589	0.405	0.258	2.7636759479274e-11	DGAT1
Successfu	0	9.05489246083596e-16	0.47858165350366	0.767	0.685	3.03682983351516e-11	HSPD1
Successfu	0	9.95739182741172e-16	0.28470743151000	0.179	0.073	3.33951007107734e-11	MDK
Successfu	0	1.0853368515007e-15	0.36232917929952	0.314	0.179	3.64000273256304e-11	SGK1
Successfu	0	1.13447200103934e-15	0.31801746052249	0.502	0.347	3.80479219708573e-11	MYO1E
Successfu	0	1.40108600540867e-15	0.32816738692247	0.214	0.097	4.69896224493959e-11	GCHFR
Successfu	0	1.41876000599388e-15	0.31321831987531	0.828	0.729	4.75823730810227e-11	RPL36AL
Successfu	0	2.27836216551539e-15	0.41047179816611	0.629	0.487	7.6411710307055e-11	LINC01871
Successfu	0	4.0869277554107e-15	0.38218113146571	0.291	0.162	1.37067383060964e-10	NCKAP5
Successfu	0	4.51645096791009e-15	0.59217738350753	0.463	0.329	1.51472732561769e-10	DDIT4
Successfu	0	5.06526916786667e-15	0.29855431567536	0.477	0.309	1.69878997351912e-10	CD160
Successfu	0	5.8241549458377e-15	0.35881607996389	0.572	0.441	1.95330508573505e-10	NOP53
Successfu	0	6.18095411093378e-15	0.36009574753624	0.614	0.492	2.07296838972497e-10	UQCRH
Successfu	0	6.84417657571024e-15	0.396892790354111	0.278	0.154	2.2953999399617e-10	COBL
Successfu	0	7.38527890069345e-15	0.43086917245242	0.598	0.488	2.47687483771457e-10	SAP18
Successfu	0	7.88800013009682e-15	0.50544734084725	0.774	0.689	2.64547748363187e-10	SAT1

Successfu	0	9.20296503776413e-15	0.35891313072040	0.317	0.189	3.08649041436533e-10	LARGE1
Successfu	0	1.04439691277679e-14	0.36815271719699	0.552	0.422	3.50269836607078e-10	ATP5IF1
Successfu	0	1.27382678394508e-14	0.56360574486688	0.717	0.583	4.27216026799502e-10	ELL2
Successfu	0	1.33695364384124e-14	0.36466815725112	0.278	0.153	4.48387513071474e-10	H2AFJ
Successfu	0	1.56119532594341e-14	0.41941985274790	0.722	0.65	5.23593688414902e-10	CALM2
Successfu	0	1.97995592147087e-14	0.46422141274603	0.737	0.605	6.64037616942901e-10	ATP1B3
Successfu	0	2.14980294261388e-14	0.34203346872948	0.342	0.212	7.21000910893844e-10	CTSD
Successfu	0	2.20830265269639e-14	0.52688692844012	0.46	0.319	7.40620543661316e-10	AC015849.1
Successfu	0	2.51011006235971e-14	0.31061304301509	0.277	0.151	8.418407127142e-10	C7orf50
Successfu	0	2.84487645409175e-14	0.37623155053933	0.275	0.154	9.5411466517329e-10	CDHR2
Successfu	0	3.00149972951939e-14	0.36953917826192	0.607	0.476	1.00664297928621e-09	UBL5
Successfu	0	4.36409053738519e-14	0.35575236264632	0.513	0.387	1.46362868442825e-09	RPS17
Successfu	0	5.62200382131944e-14	0.41967707137448	0.72	0.628	1.88550764159412e-09	CD55
Successfu	0	6.54217348074138e-14	0.42692286165768	0.454	0.326	2.19411414197104e-09	HMGB2
Successfu	0	6.83331949929107e-14	0.34357591439266	0.323	0.195	2.29175869367224e-09	KRT20
Successfu	0	6.83861126915298e-14	0.38212360750382	0.889	0.79	2.29353344744853e-09	NFKBIA
Successfu	0	7.75299685999106e-14	0.29452803578711	0.262	0.14	2.6002000869038e-09	SPINT2
Successfu	0	8.37697289571255e-14	0.62859787478664	0.634	0.515	2.80946916976407e-09	JMY
Successfu	0	8.44188341642211e-14	0.33192479366273	0.366	0.226	2.83123886019965e-09	SPON2
Successfu	0	8.77687674738127e-14	0.36923519145802	0.649	0.543	2.94358892353673e-09	CD37
Successfu	0	1.09037168992772e-13	0.36960020694682	0.661	0.535	3.65688857367959e-09	CD7
Successfu	0	1.19270705599611e-13	0.30220377271217	0.229	0.116	4.00010092439974e-09	B3GNT5
Successfu	0	1.23179412348756e-13	0.39401964693733	0.517	0.401	4.13119113135257e-09	MALRD1
Successfu	0	1.27558412222578e-13	0.27154788826499	0.197	0.091	4.27805402912083e-09	SLC6A19
Successfu	0	1.43562250291297e-13	0.34773889233954	0.575	0.455	4.81479075026953e-09	NDUFA4
Successfu	0	1.44803074014011e-13	0.36678174481508	0.562	0.434	4.8564054962819e-09	NDUFS5
Successfu	0	1.49563519492113e-13	0.27378054745044	0.211	0.1	5.01606131672649e-09	TFF3
Successfu	0	1.60508715332097e-13	0.29802916765082	0.931	0.904	5.38314129480785e-09	SARAF
Successfu	0	1.9542472721266e-13	0.28613267333295	0.169	0.072	6.55415450125818e-09	ARHGAP24
Successfu	0	2.01119276784935e-13	0.32934748877610	0.522	0.396	6.74513830481314e-09	SSR2
Successfu	0	2.11177650444437e-13	0.27500763059589	0.139	0.053	7.08247604060551e-09	GNG7

Successfu	0	2.86051586047179e-13	0.32594804488047	0.324	0.2	9.5935980928503e-09	MAOA
Successfu	0	3.05809091948301e-13	0.394372104316723	0.411	0.282	1.02562253257621e-08	LMO7
Successfu	0	3.73732214432586e-13	0.38229003060652	0.428	0.295	1.25342310076401e-08	CDHR5
Successfu	0	3.92636123613761e-13	0.281975515812425	0.196	0.093	1.31682303137583e-08	PRSS3
Successfu	0	4.81030158846621e-13	0.314936703901725	0.311	0.187	1.6132789467398e-08	DEDD2
Successfu	0	5.19460892326909e-13	0.337949043885953	0.305	0.184	1.74216794068599e-08	ALKBH7
Successfu	0	6.49933444752117e-13	0.445922238764592	0.369	0.244	2.17974678700965e-08	MRPL18
Successfu	0	6.97728231949514e-13	0.252341090981945	0.2	0.096	2.34004094431228e-08	OOEP
Successfu	0	8.26864437847028e-13	0.407218540607488	0.329	0.211	2.77313795165136e-08	EPB41L3
Successfu	0	1.13314241309395e-12	0.356597647986155	0.716	0.632	3.8003330250345e-08	EEF2
Successfu	0	1.20585562322258e-12	0.371528566395803	0.294	0.169	4.04419858916389e-08	TNFSF8
Successfu	0	1.34101203182363e-12	0.300144520426238	0.752	0.664	4.49748615233011e-08	TMA7
Successfu	0	1.45493755767089e-12	0.392857044008909	0.372	0.246	4.87956958091664e-08	ZBED4
Successfu	0	1.53459639634437e-12	0.36412098451572	0.653	0.555	5.14672939405976e-08	SLC25A6
Successfu	0	2.24708587748449e-12	0.311669750176091	0.435	0.316	7.53627661590749e-08	VPS28
Successfu	0	2.30859969184823e-12	0.31978400153105	0.235	0.127	7.74258164652059e-08	MYO7B
Successfu	0	2.36210399205239e-12	0.312901394136248	0.78	0.706	7.9220243685453e-08	SRP14
Successfu	0	2.36862189543548e-12	0.329720703628367	0.686	0.604	7.94388411291152e-08	OST4
Successfu	0	2.49781668197146e-12	0.327625812372981	0.647	0.544	8.37717758799587e-08	PPP1R2
Successfu	0	2.96674807556947e-12	0.36998000316874	0.502	0.398	9.94987969584491e-08	ATP5MD
Successfu	0	3.0883803045712e-12	0.353731541605406	0.432	0.308	1.03578098654709e-07	C9orf16
Successfu	0	3.15995876963536e-12	0.365354356666415	0.583	0.461	1.05978697216031e-07	ZBTB38
Successfu	0	3.45791134213973e-12	0.264842819986669	0.176	0.083	1.15971430592682e-07	EPS8L3
Successfu	0	4.06587395209633e-12	0.262103194816602	0.238	0.127	1.36361280605407e-07	CYBRD1
Successfu	0	4.24978936847853e-12	0.28715040206691	0.744	0.635	1.42529435840033e-07	CYBA
Successfu	0	4.25116471271029e-12	0.326909308889581	0.254	0.143	1.42575562134878e-07	RHOB
Successfu	0	5.03880691373429e-12	0.389491789653107	0.329	0.212	1.68991506272821e-07	MME
Successfu	0	5.2744861807725e-12	0.346038388139106	0.52	0.406	1.76895717530748e-07	ELOB
Successfu	0	5.37850374347863e-12	0.321585949336502	0.451	0.33	1.80384258548786e-07	PRKG1
Successfu	0	5.95574651804678e-12	0.28441333857439	0.245	0.136	1.99743826722253e-07	HEPH
Successfu	0	6.13748945773428e-12	0.27881050287384	0.265	0.153	2.05839121433492e-07	NR1H4

Successfu	0	6.30940098962631e-12	0.31307677718284[illegible]	0.175	0.079	2.11604690390087e-07	LINC02446
Successfu	0	6.50451703220998e-12	0.29950939547655[illegible]	0.268	0.153	2.18148492226258e-07	FCGRT
Successfu	0	7.63482170407775e-12	0.28589121974467	0.604	0.506	2.5605665031136e-07	COX5B
Successfu	0	7.98185132938593e-12	0.72182294142884[illegible]	0.463	0.343	2.67695329884945e-07	MRPL1
Successfu	0	9.22609148720789e-12	0.44765147738089[illegible]	0.339	0.222	3.09424656297978e-07	RBKS
Successfu	0	1.02900226043772e-11	0.34086269020866[illegible]	0.484	0.371	3.45106778105604e-07	UXT
Successfu	0	1.31739684429211e-11	0.43151920376160[illegible]	0.433	0.319	4.41828553638689e-07	CHMP1B
Successfu	0	1.38277696419664e-11	0.26529352295933[illegible]	0.332	0.211	4.63755738252269e-07	AC105402.3
Successfu	0	1.98639611910074e-11	0.30157497120364[illegible]	0.342	0.221	6.66197530424007e-07	TUBA4A
Successfu	0	2.01789922989653e-11	0.37067026995142[illegible]	0.306	0.192	6.76763043722699e-07	CKS2
Successfu	0	2.29170120719752e-11	0.33129820489990[illegible]	0.662	0.595	7.68590750869905e-07	SERP1
Successfu	0	2.73555546583891e-11	0.27956737012959[illegible]	0.133	0.054	9.17450592133052e-07	ADAM28
Successfu	0	3.41093785746005e-11	0.48993255522942	0.194	0.094	1.14396033863495e-06	XCL1
Successfu	0	4.03266262238091e-11	0.29336075021875[illegible]	0.374	0.26	1.35247439029411e-06	ATP5PO
Successfu	0	4.16215093723665e-11	0.30440165719200[illegible]	0.477	0.362	1.39590218133043e-06	UQCRQ
Successfu	0	4.98464737243167e-11	0.33578515891319[illegible]	0.475	0.365	1.67175103576613e-06	MGST3
Successfu	0	5.07077726517078e-11	0.3046022578982	0.701	0.593	1.70063727919298e-06	HCST
Successfu	0	5.43478486997246e-11	0.31449919952531[illegible]	0.396	0.277	1.82271814969136e-06	PRDX5
Successfu	0	6.98878541328641e-11	0.283111595476516	0.749	0.68	2.343898851908e-06	OAZ1
Successfu	0	7.837477589494e-11	0.31724394319877[illegible]	0.604	0.514	2.6285332339645e-06	COX6B1
Successfu	0	1.01691194596104e-10	0.25235565528401[illegible]	0.251	0.145	3.41051928436415e-06	ERRFI1
Successfu	0	1.09025608209371e-10	0.33338844241896[illegible]	0.299	0.191	3.65650084812587e-06	NFIB
Successfu	0	1.41745238629485e-10	0.27123672831880[illegible]	0.215	0.119	4.75385181315565e-06	HKDC1
Successfu	0	1.48997098315983e-10	0.39944432871439	0.592	0.495	4.99706468332145e-06	GADD45B
Successfu	0	1.7045276885698e-10	0.26078021501449[illegible]	0.206	0.111	5.71664496192541e-06	MATN2
Successfu	0	1.78359446225918e-10	0.25935341148273[illegible]	0.299	0.19	5.98181910752485e-06	NR5A2
Successfu	0	1.85483053002313e-10	0.29317829054050[illegible]	0.581	0.449	6.22073063159157e-06	ITGAE
Successfu	0	2.02016997268217e-10	0.34373286705403[illegible]	0.438	0.336	6.77524605438146e-06	DDT
Successfu	0	2.09977840026082e-10	0.25514742696763[illegible]	0.193	0.102	7.04223679879473e-06	MISP
Successfu	0	2.10280804786199e-10	0.27052896573965[illegible]	0.685	0.573	7.05239763091954e-06	COTL1
Successfu	0	2.47243365518216e-10	0.30527979072203[illegible]	0.478	0.376	8.29204799274991e-06	SPCS1

Successfu	0	2.64282475761116e-10	0.34891929790478	0.52	0.412	8.86350567207629e-06	AC022217.3
Successfu	0	2.67898983519964e-10	0.28897700473108{illegible}	0.894	0.839	8.98479610929255e-06	ARHGDIB
Successfu	0	3.60623236137615e-10	0.41860558643006[illegible]	0.735	0.675	1.20945820935833e-05	SQSTM1
Successfu	0	3.86114749298844e-10	0.26591648006744	0.208	0.115	1.29495164619846e-05	GNG12
Successfu	0	4.21531413665012e-10	0.31152279826283[illegible]	0.564	0.459	1.41373205514972e-05	TOMM20
Successfu	0	4.64568201461807e-10	0.25498615698127	0.194	0.103	1.55806883406261e-05	SPINK2
Successfu	0	4.70609063671906e-10	0.56858708700470[illegible]	0.469	0.378	1.57832867774284e-05	HIPK2
Successfu	0	5.54692092861e-10	0.29593955406902[illegible]	0.348	0.241	1.86032634103722e-05	SNHG7
Successfu	0	5.66680655056561e-10	0.28168015091179[illegible]	0.202	0.112	1.90053358092869e-05	AGPAT2
Successfu	0	6.60644412593407e-10	0.38013187001753	0.17	0.084	2.21566923095577e-05	AC120193.1
Successfu	0	6.842473797061e-10	0.33906028957737[illegible]	0.505	0.409	2.29482886205832e-05	SLC25A5
Successfu	0	7.84344457615456e-10	0.39068393930743[illegible]	0.426	0.315	2.63053444195072e-05	NDUFAF6
Successfu	0	9.07038300354313e-10	0.44042748150838[illegible]	0.41	0.317	3.04202505172829e-05	AC016831.5
Successfu	0	1.09116094397386e-09	0.41442359079923[illegible]	0.614	0.536	3.65953557389952e-05	TUBA1A
Successfu	0	1.20136630057154e-09	0.27525910344999[illegible]	0.342	0.234	4.02914229885683e-05	BRI3
Successfu	0	1.21087372157395e-09	0.34310484319040[illegible]	0.365	0.267	4.06102828741473e-05	C6orf48
Successfu	0	1.38707877177832e-09	0.39246060577378[illegible]	0.605	0.526	4.65198478479014e-05	DYNLL1
Successfu	0	1.54504004865479e-09	0.29450814214814	0.212	0.122	5.18175531517845e-05	TJP1
Successfu	0	1.56283025237683e-09	0.33918576877513[illegible]	0.315	0.214	5.2414201004214e-05	HOOK2
Successfu	0	1.77138848923836e-09	0.27136222417616[illegible]	0.499	0.391	5.94088271520762e-05	GSTP1
Successfu	0	1.77883002246925e-09	0.31633350982336[illegible]	0.425	0.322	5.96584012935738e-05	WDR83OS
Successfu	0	1.94556959691767e-09	0.26282923126626[illegible]	0.525	0.426	6.5250513141425e-05	ATP5PF
Successfu	0	1.98444446661719e-09	0.25889938031597[illegible]	0.628	0.548	6.65542985214074e-05	PRR13
Successfu	0	2.13234624279454e-09	0.52174165723527[illegible]	0.353	0.235	7.15146282908432e-05	CRTAM
Successfu	0	2.76455001975051e-09	0.30255923282558	0.221	0.126	9.27174785623925e-05	KLRC2
Successfu	0	2.94153614400731e-09	0.26856563278638[illegible]	0.25	0.156	9.86532391977173e-05	MIF
Successfu	0	3.15027939039218e-09	0.27286242568894[illegible]	0.175	0.093	0.000105654070194973	TLE1
Successfu	0	3.20587934533385e-09	0.34033252232348[illegible]	0.438	0.339	0.000107518781483807	CACYBP
Successfu	0	4.40721612359077e-09	0.26601046587449[illegible]	0.181	0.098	0.000147809214352987	AC025164.1
Successfu	0	4.60850556606857e-09	1.18467166158726	0.338	0.245	0.000154560059674808	AREG
Successfu	0	4.98923406683872e-09	0.25451462478795	0.23	0.138	0.000167328932133637	MARCKSL1

Successfu	0	5.29968700880958e-09	0.29665314882117€	0.236	0.146	0.000177740902901456	CDH17
Successfu	0	5.58886692229327e-09	0.32740450738253₂	0.481	0.387	0.000187439418839872	EIF4A3
Successfu	0	5.79434993121566e-09	0.37039003014868ε	0.691	0.622	0.000194330907993111	PIK3R1
Successfu	0	5.81205285013911e-09	0.457003066650681	0.223	0.129	0.000194924628487966	KLRC1
Successfu	0	7.05194565398601e-09	0.347738702005701	0.365	0.256	0.000236508153343383	OPRM1
Successfu	0	7.32243302694293e-09	0.263222668305503	0.577	0.483	0.000245579758857612	GUK1
Successfu	0	7.41771178404811e-09	0.27638719746587	0.227	0.137	0.000248775217813405	ANKRD37
Successfu	0	7.84200966295246e-09	0.313934290392451	0.634	0.569	0.0002630053200761	PGK1
Successfu	0	8.16926160859401e-09	0.476636774090417	0.271	0.177	0.000273980695829026	BATF
Successfu	0	1.04352754947019e-08	0.306320384591167	0.499	0.4	0.000349978269541314	RSL24D1
Successfu	0	1.05579097145506e-08	0.25619839233582	0.184	0.103	0.000354091176006598	STXBP6
Successfu	0	1.09626357358965e-08	0.293543780764648	0.39	0.294	0.000367664877310497	ATP5MPL
Successfu	0	1.09757318899023e-08	0.284954009213918	0.637	0.562	0.000368104096123543	ANAPC16
Successfu	0	1.28417499058045e-08	0.391338004553174	0.583	0.508	0.000430686608340871	H2AFZ
Successfu	0	1.29809916262506e-08	0.291682638829671	0.199	0.116	0.000435356497161192	TBC1D8
Successfu	0	1.32639014997436e-08	0.280812205423437	0.381	0.29	0.000444844728498399	OCIAD2
Successfu	0	1.61545292246622e-08	0.364275349546448	0.472	0.384	0.000541790601136721	KLF9
Successfu	0	1.67181989778444e-08	0.365352601304043	0.466	0.382	0.000560694957318944	TUBB4B
Successfu	0	1.70865895561624e-08	0.251367420669943	0.297	0.203	0.000573050040534574	RHOC
Successfu	0	1.7832490624807e-08	0.290634862670608	0.487	0.392	0.000598066070574779	ENY2
Successfu	0	1.85865612666889e-08	0.338225529901237	0.39	0.302	0.000623356091762211	SERTAD1
Successfu	0	2.05081774522733e-08	0.267204056026678	0.647	0.574	0.000687803255394343	EIF3E
Successfu	0	2.09701215308127e-08	0.377790178685302	0.541	0.457	0.000703295935900396	OSTF1
Successfu	0	2.10521334525029e-08	0.376098690954204	0.49	0.376	0.000706046451730042	AC020571.1
Successfu	0	2.26575857772188e-08	0.468247429096488	0.703	0.63	0.000759890111796365	RASA1
Successfu	0	2.26737152637925e-08	0.267258441994985	0.729	0.662	0.000760431062517072	ATP1A1
Successfu	0	2.40269053224594e-08	0.298445688792577	0.235	0.145	0.000805814350704645	GAB2
Successfu	0	2.40989033404063e-08	0.292806923753656	0.555	0.462	0.000808229020230545	IL6ST
Successfu	0	3.05209543299552e-08	0.273805136472624	0.907	0.88	0.00102361176631804	CNOT6L
Successfu	0	3.21373215091183e-08	0.350692736394915	0.377	0.293	0.00107782148877281	DNAJB9
Successfu	0	3.23442720262508e-08	0.34550192750389	0.792	0.725	0.0010847621952164	PDE3B

Successfu	0	4.5445815893472e-08	0.26749805332375	0.211	0.129	0.00152416177343526	PXN
Successfu	0	4.61284758677471e-08	0.29149463396674	0.489	0.397	0.0015470568236525	RGS10
Successfu	0	4.91291382359188e-08	0.25852233203897	0.653	0.581	0.00164769303815624	CNBP
Successfu	0	5.0318256632769e-08	0.27325120665096	0.386	0.297	0.00168757369094981	CUTA
Successfu	0	5.86190804520036e-08	0.31670281747273	0.508	0.428	0.0019659667201993	COX7B
Successfu	0	7.49327643688919e-08	0.36895254424391	0.647	0.592	0.0025130950514039	CYCS
Successfu	0	7.51639385335784e-08	0.26693558668016	0.783	0.741	0.00252084817053915	SKP1
Successfu	0	7.52107498354048e-08	0.25649151988055	0.392	0.296	0.00252241812797981	REX1BD
Successfu	0	8.99382880475429e-08	0.29412791913744	0.274	0.185	0.00301635030453849	BCL2L11
Successfu	0	9.36656187377718e-08	0.29658986071529	0.324	0.234	0.00314135752122739	PLS1
Successfu	0	9.93482705592393e-08	0.35365552600156	0.426	0.325	0.00333194229801577	HECW2
Successfu	0	1.16646684081831e-07	0.33312954301907	0.647	0.594	0.00391209649073645	NAP1L4
Successfu	0	1.48508761570803e-07	0.29422504515973	0.333	0.247	0.0049806868455616	AHSA1
Successfu	0	1.64087554696449e-07	0.39785115236275	0.607	0.581	0.00550316840940952	ZFAND5
Successfu	0	2.24472171525004e-07	0.26704586539513	0.39	0.301	0.0075283476886056	RPL36A
Successfu	0	2.40473308248003e-07	0.25166982895498	0.308	0.221	0.00806499381202154	ISCU
Successfu	0	2.45092372940825e-07	0.30586008993982	0.45	0.372	0.00821990800368938	NSA2
Successfu	0	2.58279979540947e-07	0.25751091189838	0.685	0.625	0.00866219395384428	ARL6IP5
Successfu	0	3.42732287118e-07	0.27273823235943	0.29	0.21	0.0114945554453635	TMEM14C
Successfu	0	4.61385111359183e-07	0.40956147843273	0.312	0.233	0.0154739338647643	NMRK1
Successfu	0	4.85262057571767e-07	0.27152582554588	0.236	0.156	0.0162747188868419	C9orf72
Successfu	0	5.02770534568568e-07	0.27571689462248	0.217	0.138	0.0168619181883606	DNAJA4
Successfu	0	5.72393262932431e-07	0.25353876257865	0.426	0.342	0.0191969252522279	GYPC
Successfu	0	6.52814663486493e-07	0.25758902594073	0.477	0.397	0.02189409818401	RPL22L1
Successfu	0	8.35435888459479e-07	0.31619516876789	0.538	0.471	0.028018848827154	BCAS2
Successfu	0	9.05970847584116e-07	0.39263809753132	0.335	0.257	0.0303844502862761	AL691403.1
Successfu	0	1.1315620585504e-06	0.25596923275331	0.595	0.529	0.0379503283196635	ATP6V1G1
Successfu	0	1.20088422103208e-06	0.30227808954353	0.433	0.359	0.040275255004974	AKIRIN2
Successfu	0	1.24340772001604e-06	0.25853166554535	0.574	0.506	0.0417014081138979	LDHA
Successfu	0	1.25399144720167e-06	0.25433595539768	0.187	0.118	0.0420563651562497	MEP1A
Successfu	6	3.02663139300363e-28	0.83779362889958	0.923	0.868	1.01507163658556e-23	RPL19

Successfu	6	4.03461051239583e-28	1.34324665971328	0.893	0.811	1.35312767364731e-23	NFKBIA
Successfu	6	2.65218121090471e-26	0.80041163290607	0.939	0.936	8.89488534513223e-22	RPL10
Successfu	6	1.07073377243345e-25	0.80395869737760	0.923	0.893	3.5910269259873e-21	RPS3
Successfu	6	5.77077747537431e-24	1.12998434764809	0.918	0.839	1.93540334969103e-19	PPP1R15A
Successfu	6	1.00700093656722e-23	0.57921345785159	0.98	0.992	3.37727974105915e-19	B2M
Successfu	6	1.24287446263331e-23	1.21637843757584	0.827	0.666	4.16835237277959e-19	IRF1
Successfu	6	2.4169997592552e-23	0.66629689113181	0.929	0.892	8.1061337925901e-19	RPL7A
Successfu	6	1.25198666299665e-22	0.71384002634010	0.913	0.8	4.19891287035818e-18	RPS5
Successfu	6	2.94171960953612e-22	0.64520138164469	0.918	0.834	9.86593922646225e-18	RPL23A
Successfu	6	1.05144195307923e-21	0.86177973613622	0.821	0.603	3.52632602223711e-17	RPS10
Successfu	6	1.38708322971354e-21	0.70626118335160	0.923	0.84	4.65199973581326e-17	RPSA
Successfu	6	6.25726868545354e-21	0.70466226779930	0.944	0.88	2.09856277172741e-16	RPL5
Successfu	6	6.83999373164242e-21	0.68489396749775	0.939	0.891	2.29399709771823e-16	RPS27A
Successfu	6	1.20630129550865e-20	0.62737886019053	0.918	0.829	4.04569328487691e-16	RPL26
Successfu	6	3.94196278721891e-20	0.65292004820344	0.939	0.873	1.32205547957748e-15	RPL18A
Successfu	6	7.5099022851964e-20	0.60170401017254	0.934	0.872	2.51867102840917e-15	RPS9
Successfu	6	1.14748533623199e-19	0.58820363643541	0.99	0.986	3.84843632065486e-15	EEF1A1
Successfu	6	4.15079254483425e-19	0.65930251182455	0.929	0.903	1.39209280368651e-14	RPS3A
Successfu	6	1.49984918987133e-18	0.61878574150220	0.944	0.926	5.03019421299047e-14	RPL13
Successfu	6	1.67825725573679e-18	0.65057455823717	0.934	0.848	5.62853918429003e-14	RPLP0
Successfu	6	1.74324244463197e-18	0.63319139710433	0.969	0.956	5.84648651080668e-14	JUND
Successfu	6	2.51639533008989e-18	0.62174121013559	0.908	0.837	8.43948665805548e-14	RACK1
Successfu	6	2.63170932143954e-18	0.59987601588446	0.934	0.89	8.82622672224394e-14	RPL18
Successfu	6	3.10409016147499e-18	0.64715330576066	0.929	0.886	1.04104975835548e-13	RPL3
Successfu	6	4.04868119707786e-18	0.60467217198324	0.913	0.892	1.35784669987597e-13	RPS23
Successfu	6	6.82239008857761e-18	0.60929393975016	0.893	0.797	2.28809318790716e-13	RPL7
Successfu	6	1.61929207676611e-17	0.56303940295356	0.939	0.891	5.43078176705817e-13	RPL14
Successfu	6	2.35221119003141e-17	0.54316824178561	0.944	0.889	7.88884588912735e-13	RPS16
Successfu	6	2.74203942331514e-17	0.87279872127319	0.551	0.311	9.19625181791432e-13	GBP1
Successfu	6	3.8361627445808e-17	0.59404689709950	0.934	0.908	1.28657226127751e-12	RPS4X
Successfu	6	5.09806288333647e-17	0.59546952867427	0.888	0.782	1.70978832981339e-12	MYL12A

Successfu	6	7.28518353201776e-17	0.54131623026441	0.939	0.913	2.44330485296811e-12	RPL13A
Successfu	6	7.57229168731289e-17	0.64331302528766	0.923	0.869	2.539595186091e-12	RPS13
Successfu	6	1.37086415413788e-16	0.68712189460632	0.857	0.682	4.59760420014763e-12	CD48
Successfu	6	1.96352545658887e-16	0.68492067322849	0.883	0.751	6.58527167630774e-12	EEF1B2
Successfu	6	2.11966839357918e-16	0.59917144357184	0.923	0.867	7.10894385838586e-12	RPL8
Successfu	6	2.27547647498132e-16	0.56692594323079	0.908	0.893	7.63149300179237e-12	RPL30
Successfu	6	8.49094224115224e-16	0.61151717878142	0.837	0.687	2.84769220883764e-11	OAZ1
Successfu	6	9.69214945980299e-16	1.03053554559212	0.878	0.759	3.25055308582873e-11	BTG2
Successfu	6	1.19589987690363e-15	0.56459115388140	0.949	0.935	4.01080900715941e-11	RPS12
Successfu	6	1.20597322655027e-15	0.51590298117923	0.934	0.89	4.0445930072043e-11	RPL6
Successfu	6	2.75824359219077e-15	0.53713201073983	0.923	0.892	9.2505973594894e-11	RPL9
Successfu	6	4.69841830260754e-15	0.51284380615198	0.908	0.872	1.57575553032852e-10	RPS7
Successfu	6	5.1446223405329e-15	0.57449826418707	0.929	0.884	1.72540344056792e-10	HLA-C
Successfu	6	8.64858224958803e-15	0.61446103932647	0.745	0.559	2.90056151486683e-10	RAC2
Successfu	6	9.91234634846012e-15	0.54078234657676	0.949	0.914	3.32440271834656e-10	RPL11
Successfu	6	1.28770751967878e-14	0.47651453727437	0.964	0.95	4.31871347949869e-10	HLA-B
Successfu	6	1.80111613855992e-14	0.53852108741810	0.934	0.845	6.04058330550225e-10	NACA
Successfu	6	2.62668701070177e-14	0.49027579208856	0.923	0.92	8.80938289649158e-10	RPS14
Successfu	6	3.03822020042976e-14	0.49974170356679	0.934	0.938	1.01895829082013e-09	RPS2
Successfu	6	4.85390251225756e-14	0.52099820926724	0.908	0.877	1.62790182456094e-09	RPS25
Successfu	6	5.73573804618865e-14	0.5119149425213	0.934	0.879	1.92365182593075e-09	RPL21
Successfu	6	9.44833774398529e-14	0.65170814616085	0.735	0.532	3.16878351257779e-09	STAT1
Successfu	6	2.92319620552784e-13	0.56830331878653	0.913	0.832	9.80381543409927e-09	PFN1
Successfu	6	4.82089413715162e-13	1.26206132931473	0.587	0.418	1.61683147571791e-08	DUSP2
Successfu	6	5.13445893392648e-13	0.46421356813198	0.923	0.866	1.72199483726026e-08	RPL24
Successfu	6	7.68519984066e-13	0.50300685373519	0.898	0.816	2.57746232256055e-08	RPL10A
Successfu	6	8.61386706594828e-13	0.57751002507508	0.699	0.498	2.88891873657773e-08	CLIC1
Successfu	6	1.40941197757043e-12	0.49737814921668	0.923	0.893	4.72688589037571e-08	RPL32
Successfu	6	2.5751945311766e-12	0.44777689529609	0.949	0.922	8.63668741866007e-08	RPS19
Successfu	6	2.6003158470127e-12	0.50772394085886	0.847	0.756	8.7209392877112e-08	BTF3
Successfu	6	2.92989360631552e-12	0.69265336376909	0.862	0.811	9.826277176861e-08	VIM

Successfu	6	4.8368335644871e-12	0.411645949377137	0.934	0.922	1.62217724085768e-07	RPL28
Successfu	6	6.08268485225315e-12	0.471299648054095	0.821	0.719	2.04001084574866e-07	HNRNPA1
Successfu	6	6.63508950968079e-12	0.544881185889769	0.862	0.768	2.22527631975674e-07	EEF1D
Successfu	6	7.4293626761287e-12	0.428481949872827	0.939	0.94	2.49165965432004e-07	RPL41
Successfu	6	9.07403510468664e-12	0.858869861251164	0.827	0.745	3.04324989340981e-07	ZFP36
Successfu	6	9.24567619716989e-12	0.471348064950566	0.954	0.956	3.10081488300684e-07	RPLP1
Successfu	6	1.76098199868849e-11	0.415588641074156	0.388	0.193	5.90598142720145e-07	PSMA2
Successfu	6	1.804292556316e-11	0.42206021928388	0.949	0.919	6.05123637537259e-07	RPS18
Successfu	6	3.59359328551742e-11	0.561847149056062	0.362	0.183	1.20521931609683e-06	CTSW
Successfu	6	3.80656960629976e-11	0.433013001124775	0.934	0.881	1.27664731456081e-06	RPL29
Successfu	6	8.916063854152e-11	0.369410121728617	0.934	0.895	2.9902694954055e-06	RPS15
Successfu	6	1.03121555924166e-10	0.482350198038091	0.98	0.97	3.45849074258468e-06	KLF6
Successfu	6	1.11380302475667e-10	0.438837592829075	0.449	0.256	3.73547258442893e-06	EIF5A
Successfu	6	1.21148716721599e-10	0.48671025431515	0.923	0.916	4.06308566140898e-06	HLA-A
Successfu	6	1.35262713840069e-10	0.870869968635379	0.929	0.902	4.53644089676822e-06	JUN
Successfu	6	1.40057715728333e-10	0.4538824069266	0.934	0.872	4.69725567009682e-06	HSP90AB1
Successfu	6	1.84403723921738e-10	0.451304136892111	0.495	0.305	6.18453209288726e-06	PSMD8
Successfu	6	1.85823590600968e-10	0.448885688001452	0.878	0.765	6.23215158157526e-06	YBX1
Successfu	6	1.9693875238248e-10	0.610043980603571	0.872	0.792	6.6049318774036e-06	GAPDH
Successfu	6	2.34074884201055e-10	0.395126746961628	0.939	0.899	7.85040346633499e-06	RPS15A
Successfu	6	2.36447405188083e-10	0.510414856623068	0.556	0.359	7.92997307519792e-06	EMP3
Successfu	6	2.42074761081643e-10	0.468523794509646	0.857	0.747	8.11870333715615e-06	RPL36AL
Successfu	6	2.75745797764331e-10	0.413025682831365	0.888	0.801	9.24796256542014e-06	RPL27
Successfu	6	3.34178603481173e-10	0.413747235381453	0.944	0.946	1.12076820035516e-05	EIF1
Successfu	6	5.00635280113316e-10	0.39269957282953	0.954	0.976	1.67903060244404e-05	TPT1
Successfu	6	6.54563530567489e-10	0.682920341128838	0.745	0.62	2.19527516881724e-05	MYADM
Successfu	6	6.89215672367392e-10	0.375445606405531	0.944	0.923	2.31149152198576e-05	RPS6
Successfu	6	8.56785622649856e-10	0.475876842940264	0.867	0.754	2.87348762124309e-05	S100A10
Successfu	6	9.34371394557844e-10	0.391090006312667	0.929	0.894	3.1336947830681e-05	ACTG1
Successfu	6	9.57691612659287e-10	0.396367259664767	0.903	0.84	3.21190613053672e-05	UBA52
Successfu	6	1.33095415644222e-09	0.309928212393775	0.281	0.128	4.46375404987593e-05	ALYREF

Successfu	6	1.50710912778476e-09	0.33705010656950	0.342	0.171	5.05454259276454e-05	DPH3
Successfu	6	1.64397640245797e-09	0.46339588944444	0.709	0.536	5.51356805856354e-05	EIF3K
Successfu	6	1.97591178106166e-09	0.46041706978427	0.862	0.758	6.62681293132458e-05	SH3BGRL3
Successfu	6	2.19147970828451e-09	0.38548761373785	0.913	0.88	7.3497846456446e-05	RPS20
Successfu	6	2.64561297227174e-09	0.37488623722528	0.949	0.897	8.87285678640495e-05	CALM1
Successfu	6	2.66382762544563e-09	0.55230055535127	0.628	0.474	8.93394509021955e-05	TUBA1B
Successfu	6	2.67543797348252e-09	0.38515823695163	0.893	0.836	8.97288387546567e-05	FAU
Successfu	6	2.89381125327560e-09	0.33143133453039	0.949	0.909	9.70526418123569e-05	FTL
Successfu	6	3.25097370222695e-09	0.49163740956634	0.541	0.353	0.000109031156025287	PDIA6
Successfu	6	6.73075946742741e-09	0.40079247688284	0.908	0.816	0.000225736211018581	RPS26
Successfu	6	7.6011392951951e-09	0.36581356440194	0.923	0.902	0.000254927009682253	RPL12
Successfu	6	8.00919873845505e-09	0.50055466613524	0.337	0.186	0.000268612507290305	GBP4
Successfu	6	9.38098604300415e-09	0.42127160770237	0.934	0.828	0.000314619509910273	HMGB1
Successfu	6	9.82834877480563e-09	0.37102664303761	0.469	0.301	0.000329623161209431	PRELID1
Successfu	6	1.06245961496685e-08	0.43187032282183	0.745	0.627	0.000356327705667583	SUMO2
Successfu	6	1.06314782232153e-08	0.37238446737283	0.939	0.902	0.000356558516650196	RPS8
Successfu	6	1.20022722876873e-08	0.34953585833577	0.908	0.85	0.000402532207984456	RPL35A
Successfu	6	1.42597748665399e-08	0.37612385017188	0.878	0.785	0.000478244329474016	NPM1
Successfu	6	1.45922274046272e-08	0.40609488298543	0.515	0.337	0.000489394122696388	UBXN1
Successfu	6	1.66310453059797e-08	0.29442859728998	0.255	0.119	0.000557771997471947	MRPS7
Successfu	6	1.69653881164113e-08	0.50668203537519	0.464	0.309	0.000568985186648202	BST2
Successfu	6	1.94186924861817e-08	0.48932463908885	0.566	0.407	0.000651264108601563	GTF3A
Successfu	6	1.9567877346239e-08	0.51721167075319	0.526	0.36	0.000656267470438162	HOPX
Successfu	6	2.01402502696547e-08	1.11933676266332	0.663	0.501	0.000675463713543678	CCL4
Successfu	6	2.17533656057873e-08	0.39239356937016	0.321	0.168	0.000729564375686896	PRF1
Successfu	6	2.19244767474754e-08	0.33496678004269	0.954	0.948	0.000735303101156829	ACTB
Successfu	6	2.88884012811504e-08	0.50914282811263	0.745	0.623	0.000968859202167223	RARRES3
Successfu	6	2.95164034085541e-08	0.46280339002679	0.704	0.55	0.000989921137516088	SSR4
Successfu	6	3.19497119043574e-08	0.32564004382536	0.893	0.876	0.00107152943784834	RPL15
Successfu	6	3.26589292898232e-08	0.38712367786515	0.893	0.84	0.00109531517052209	HLA-E
Successfu	6	3.85036404642859e-08	0.43148052365515	0.699	0.561	0.00129133509389122	CORO1A

Successfu	6	3.96555799857889e-08	0.41063063908923{	0.862	0.762	0.00132996884156339	NCL
Successfu	6	4.06218091236687e-08	0.35524488919575{	0.888	0.762	0.0013623742343896	PTGES3
Successfu	6	4.11112445216673e-08	0.50821752340493{	0.25	0.119	0.00137878891876768	GZMB
Successfu	6	4.33271016413806e-08	0.54759887931312{	0.689	0.583	0.00145310433484862	HSPA5
Successfu	6	4.57985563251248e-08	0.37291387707622{	0.485	0.313	0.00153599198203203	PSMB3
Successfu	6	4.65564683905791e-08	0.45441796381557{	0.689	0.565	0.00156141083688324	PPIB
Successfu	6	4.69523977908419e-08	0.36953397377643{	0.893	0.841	0.00157468951710925	CFL1
Successfu	6	5.24856577037508e-08	0.37797132080548	0.49	0.316	0.00176026398806839	EVI2B
Successfu	6	5.24870116660639e-08	0.42544192708644{	0.643	0.493	0.00176030939725645	RAN
Successfu	6	5.49143524072011e-08	0.38652201482344{	0.827	0.743	0.00184171755103271	CD3E
Successfu	6	5.67625174076422e-08	0.50630619409730{	0.327	0.178	0.0019037013088175	APOBEC3G
Successfu	6	8.32720769937212e-08	0.31919305227443{	0.923	0.906	0.00279277891821542	RPLP2
Successfu	6	8.85934010712794e-08	0.86563458132065	0.388	0.227	0.00297124548512857	MIR155HG
Successfu	6	9.42787984061559e-08	0.37071718984995{	0.878	0.789	0.00316192234094566	PPIA
Successfu	6	1.12484305773948e-07	0.42866848356759	0.73	0.589	0.00377249864704665	TAGLN2
Successfu	6	1.13081132397957e-07	0.41986894563600{	0.5	0.356	0.00379251501836268	SRSF9
Successfu	6	1.82737944892939e-07	0.32026765260720{	0.321	0.174	0.00612866519581939	SNRPN
Successfu	6	1.99099754275505e-07	0.42523568842041{	0.679	0.553	0.00667740755889187	PSMB9
Successfu	6	2.11176762298318e-07	0.37388431963159{	0.51	0.358	0.00708244625396099	RABAC1
Successfu	6	2.40565560647309e-07	0.35224932021357{	0.515	0.351	0.00806808777298944	HIGD2A
Successfu	6	2.45974964181244e-07	0.37355301557508{	0.883	0.825	0.00824950834871055	RPL22
Successfu	6	2.55139637585876e-07	0.31472305068088{	0.403	0.246	0.00855687316535512	PPP1R11
Successfu	6	2.76564681422669e-07	0.28768390291701	0.24	0.115	0.00927542628555347	MATK
Successfu	6	3.06974293619754e-07	0.58797233875929{	0.974	0.964	0.0102953038594193	CD69
Successfu	6	3.17058798304354e-07	0.33985928028500{	0.607	0.45	0.0106335179775314	SNRPB
Successfu	6	3.27095303710638e-07	0.42808150632306	0.755	0.658	0.0109701222958474	CYBA
Successfu	6	3.29817314698951e-07	0.35375737400436	0.765	0.656	0.0110614131003734	CHCHD2
Successfu	6	3.39881293320826e-07	0.25696850953838{	0.26	0.131	0.0113989388153939	MRPL43
Successfu	6	3.65655429917228e-07	0.46471826473701{	0.444	0.296	0.012263351808564	BHLHE40
Successfu	6	3.69479244395316e-07	0.37141995185881{	0.735	0.63	0.0123915948985301	ARPC3
Successfu	6	3.74547336517796e-07	0.36647646059317{	0.684	0.522	0.0125615685721339	RBM8A

Successfu	6	4.05593199837278e-07	0.30919034290870	0.821	0.722	0.0136027847361426	MYL12B
Successfu	6	4.52475189078504e-07	0.38023747896454	0.628	0.475	0.0151751128913149	EIF3F
Successfu	6	4.91203070199003e-07	0.48420420799366	0.398	0.249	0.0164739685683341	GBP5
Successfu	6	5.61066422352628e-07	0.40248731523937	0.622	0.499	0.0188170456728624	GUK1
Successfu	6	5.68818405485724e-07	0.34705073733796	0.862	0.76	0.0190770316831802	RPL4
Successfu	6	6.48253307738392e-07	0.43091605352489	0.622	0.458	0.0217411194349302	DEFA5
Successfu	6	6.98278886798781e-07	0.32237842011789	0.362	0.22	0.0234188773054575	ICAM3
Successfu	6	7.36046735283788e-07	0.35292899569394	0.423	0.278	0.0246855354079477	EIF3I
Successfu	6	8.57050339696004e-07	0.66953910857633	0.883	0.863	0.0287437542927246	DUSP1
Successfu	6	1.11176558370117e-06	0.28488288978337	0.357	0.214	0.0372863941461697	FDX1
Successfu	6	1.13173412212539e-06	0.39753487014634	0.617	0.503	0.0379560989878414	SEC61B
Successfu	6	1.18104244461005e-06	0.28370280769956	0.842	0.736	0.0396098015073319	ATP5MG
Successfu	6	1.32641677023646e-06	0.39464425427028	0.378	0.245	0.0444853656401905	OSTC
Successfu	6	1.3499401298432e-06	0.44841033307972	0.592	0.467	0.0452742920746812	NOP53
Successfu	6	1.37873545667139e-06	0.31321054444236	0.577	0.418	0.046240029745845	SSR2
Successfu	6	1.38079734734025e-06	0.47479116543390	0.536	0.415	0.0463091814350973	PSME2
Successfu	12	1.60380876951054e-29	0.57551033834541	0.791	0.429	5.37885385118445e-25	DEFA5
Successfu	12	1.8099784860066e-27	1.23135876750277	0.381	0.13	6.07030584636894e-23	HBB
Successfu	12	1.68341731911785e-25	1.13506462675597	0.629	0.364	5.64584500485745e-21	IL2RA
Successfu	12	1.30848661189187e-17	0.46513976026092	0.676	0.434	4.38840239896296e-13	OLFM4
Successfu	12	9.05306634301072e-16	1.30410384207898	0.77	0.579	3.03621739011894e-11	TRAF1
Successfu	12	2.51866114410089e-14	0.58379867889860	0.698	0.476	8.44708574508557e-10	NR4A1
Successfu	12	2.70025717072078e-14	0.69790029367980	0.849	0.758	9.05612249916336e-10	BTG2
Successfu	12	3.88856835245011e-13	0.94297238790839	0.583	0.41	1.30414805404472e-08	EGR1
Successfu	12	4.10185340293348e-13	0.28493531207684	0.86	0.592	1.37567959427583e-08	MT1G
Successfu	12	1.42043044102081e-12	0.52614228434418	0.885	0.788	4.7638396130956e-08	RASGEF1B
Successfu	12	2.25775346267487e-12	0.51781950038743	0.903	0.838	7.57205356311897e-08	PPP1R15A
Successfu	12	2.43451964721335e-12	0.28931540873245	0.572	0.327	8.16489199282413e-08	MT1H
Successfu	12	2.47964868549e-12	0.48659351281012	0.227	0.092	8.31624576139637e-08	AL137186.1
Successfu	12	2.71279503198056e-12	0.59433104283811	0.917	0.784	9.0981719782564e-08	NFKB1
Successfu	12	1.43650166903104e-11	0.42007839586342	0.957	0.927	4.81773929759629e-07	FOSB

Successfu	12	1.93218023405407e-11	0.41790493574409	0.932	0.857	6.48014606897053e-07	REL
Successfu	12	2.37184216530085e-11	0.48437102234381	0.799	0.622	7.95468425398599e-07	TAB2
Successfu	12	5.39912131439598e-11	0.50492439785230	0.827	0.715	1.81075730642212e-06	HIVEP2
Successfu	12	5.73642264097745e-11	0.43791674158219	0.77	0.612	1.92388142533102e-06	MYADM
Successfu	12	7.47065918706889e-11	0.58795093378915	0.676	0.501	2.50550967815917e-06	ZNF267
Successfu	12	3.31172514462669e-10	0.51498445447567	0.748	0.582	1.1106863790049e-05	GPR174
Successfu	12	3.61824543042762e-10	0.43377148262620	0.734	0.575	1.21348715245681e-05	LINC01004
Successfu	12	5.18510423228954e-10	0.32726958075308	0.993	0.94	1.73898025742527e-05	CD96
Successfu	12	8.01319302527304e-10	0.34463909394388	0.935	0.843	2.68746467681607e-05	GPBP1
Successfu	12	8.20735820490783e-10	0.39416210951952	0.935	0.864	2.75258379476199e-05	TANK
Successfu	12	8.80153708784651e-10	0.36926105483663	0.291	0.147	2.95185950852196e-05	ICAM1
Successfu	12	9.5270361360819e-10	0.43148564621289	0.978	0.946	3.19517737931915e-05	STAT4
Successfu	12	2.44897478660018e-09	0.43517819525653	0.971	0.895	8.21337163929969e-05	DDX3X
Successfu	12	2.95597138714551e-09	0.49255804937457	0.957	0.966	9.91373683820861e-05	CD69
Successfu	12	6.78142779744227e-09	0.35132192115131	0.655	0.467	0.000227435525470619	G3BP1
Successfu	12	6.92334361390763e-09	0.39200426063008	0.64	0.481	0.000232195098123234	KDM6B
Successfu	12	8.64559187083715e-09	0.42369507963122	0.665	0.478	0.000289955860164136	ADAMTS17
Successfu	12	1.09084253726002e-08	0.34322007498552	0.752	0.556	0.000365846770146267	MED13
Successfu	12	1.98178911517028e-08	0.38710405227795	0.428	0.261	0.000664652433445807	CA10
Successfu	12	2.20326817070611e-08	0.33085796251210	0.608	0.444	0.000738932079091414	SPTY2D1
Successfu	12	2.22393647012278e-08	0.34709536689660	0.46	0.292	0.000745863813349778	IL18RAP
Successfu	12	3.17739984479473e-08	0.39130706658231	0.845	0.724	0.00106563635994726	SOS1
Successfu	12	3.81923398167776e-08	0.38845802185664	0.633	0.472	0.00128089469277509	NFKBID
Successfu	12	4.84569558773766e-08	0.62010546505400	0.752	0.632	0.00162514938621546	BIRC3
Successfu	12	4.86776845492706e-08	0.38277193965797	0.32	0.183	0.00163255218441344	WHRN
Successfu	12	5.01773387384288e-08	0.44097160693301	0.543	0.392	0.00168284758660942	ARL5B
Successfu	12	5.12691007985274e-08	0.49608068621488	0.55	0.409	0.00171946310258101	INSIG1
Successfu	12	5.16326070122533e-08	0.61661475211975	0.374	0.228	0.00173165437397695	AC105402.3
Successfu	12	5.43871572954303e-08	0.42785078519212	0.701	0.571	0.00182403648137414	PFKFB3
Successfu	12	5.67966004829292e-08	0.26749333647062	0.392	0.237	0.00190484438699648	REG3A
Successfu	12	6.29015009200328e-08	0.29488219086695	0.831	0.665	0.00210959053785606	BBX

Successfu	12	6.66495998141601e-08	0.26662319985397[illegible]	0.302	0.169	0.0022352942785673	SACS
Successfu	12	6.96465332063378e-08	0.34710455509002[illegible]	0.968	0.944	0.00233580543067416	TNFAIP3
Successfu	12	7.07849385322614e-08	0.33083658236082[illegible]	0.626	0.458	0.00237398526849498	SLC16A7
Successfu	12	1.22920909267173e-07	0.51013785847826[illegible]	0.676	0.539	0.00412252145500245	ICOS
Successfu	12	1.50830648559108e-07	0.36636829621358[illegible]	0.975	0.933	0.00505855829137537	DENND4A
Successfu	12	1.62469080981128e-07	0.26961158352984[illegible]	0.748	0.58	0.00544888803794507	HNRNPF
Successfu	12	1.88796313039544e-07	0.34486714808455[illegible]	0.522	0.38	0.00633185074672022	MAPK6
Successfu	12	2.19369266335046e-07	0.37542373830694[illegible]	0.446	0.306	0.00735720645434478	C21orf91
Successfu	12	2.56408119847945e-07	0.46934070649857[illegible]	0.442	0.299	0.00859941552346037	ZSWIM4
Successfu	12	2.7542408966321e-07	0.28839557372718	0.586	0.434	0.00923717311912474	PPP4R2
Successfu	12	2.95081262150725e-07	0.78698725482674[illegible]	0.353	0.227	0.009896435370011	RBBP8
Successfu	12	3.5392556976279e-07	0.42695876575154[illegible]	0.881	0.792	0.0118699557587045	RALGAPA1
Successfu	12	4.15698983731993e-07	0.32611926842365[illegible]	0.464	0.317	0.0139417125164036	CATSPERB
Successfu	12	4.7509107993651e-07	0.33140601609631[illegible]	0.644	0.465	0.0159336046389107	FCHSD2
Successfu	12	4.83916854522401e-07	0.36634616851025[illegible]	0.647	0.506	0.0162296034669723	ZBTB1
Successfu	12	6.13612964812648e-07	0.35612502764792[illegible]	0.946	0.881	0.0205793516138866	KMT2E
Successfu	12	6.53867987393507e-07	0.43467955423969[illegible]	0.478	0.346	0.0219294245612035	NSUN6
Successfu	12	7.46514770337827e-07	0.31397976602771	0.518	0.365	0.02503661236759	STXBP3
Successfu	12	7.55202627798217e-07	0.34058243443942[illegible]	0.543	0.401	0.0253279857310966	TMEM71
Successfu	12	7.79034833006724e-07	0.34034249085913[illegible]	0.406	0.268	0.0261272702293795	DAAM1
Successfu	12	8.26178527412578e-07	0.26406106981683[illegible]	0.561	0.387	0.027708375452363	AC020571.1
Successfu	12	1.09536987608658e-06	0.368383111925944	0.568	0.415	0.0367365149041916	HIVEP1
Successfu	12	1.09855854540958e-06	0.27745764671173[illegible]	0.281	0.163	0.0368434564959465	AC025180.1
Successfu	12	1.4193856462727e-06	0.25223069134521	0.342	0.212	0.0476033558046938	ALMS1
Successfu	12	1.47535095517451e-06	0.27697140766500[illegible]	0.928	0.783	0.0494803203346426	PDE7A
Successfu	19	2.19328055438619e-33	0.69527519916696[illegible]	0.227	0.043	7.35582432330039e-29	C1QB
Successfu	19	3.96055160762013e-25	0.959474411789547	0.746	0.582	1.32828979816364e-20	MT2A
Successfu	19	6.26155734089041e-25	0.83769356374058[illegible]	0.852	0.806	2.10000110098783e-20	S100A6
Successfu	19	2.06332547858405e-24	0.81931078709653[illegible]	0.898	0.896	6.91998099007518e-20	ACTG1
Successfu	19	8.58273985797294e-24	0.83578509551647[illegible]	0.86	0.845	2.87847929356696e-19	RPSA
Successfu	19	4.28318906814094e-22	1.12476581224197	0.508	0.284	1.43649594967311e-17	LGALS1

Successfu	19	1.14302786582144e-21	0.74567031023782	0.947	0.935	3.83348685639194e-17	RPS12
Successfu	19	2.11655983736232e-21	0.62566601728392	0.92	0.918	7.09851838254575e-17	TMSB10
Successfu	19	6.14690965065348e-20	0.64107816487136	0.894	0.899	2.06155055863616e-15	RPS15
Successfu	19	8.79486970658929e-20	0.73282101090711	0.848	0.835	2.94962340219592e-15	RPL26
Successfu	19	1.42113825419371e-19	0.59756331796137	0.951	0.938	4.76621347691487e-15	RPL41
Successfu	19	1.12481519374589e-18	1.01937654422042	0.78	0.733	3.77240519678496e-14	S100A4
Successfu	19	3.83998356908495e-18	0.77840431835715	0.837	0.838	1.28785368939971e-13	PFN1
Successfu	19	7.26596798691217e-18	0.64282223334095	0.837	0.846	2.4368603434506e-13	CFL1
Successfu	19	8.36766890819077e-18	0.59982652988598	0.898	0.893	2.80634879842902e-13	RPL18
Successfu	19	1.02451291070836e-17	0.63329049685610	0.905	0.892	3.43601139993369e-13	RPS23
Successfu	19	1.25628748931191e-17	0.62542420177319	0.924	0.924	4.21333698165429e-13	RPS19
Successfu	19	2.07564506615235e-17	0.60589475144729	0.894	0.905	6.96129842286176e-13	RPL12
Successfu	19	2.07861098285935e-17	0.61829819437026	0.886	0.876	6.97124551431368e-13	RPS9
Successfu	19	3.74905644219777e-17	0.77672699858828	0.939	0.95	1.25735854958429e-12	ACTB
Successfu	19	5.23789767133442e-17	0.57455545721569	0.92	0.923	1.75668612101214e-12	RPS28
Successfu	19	5.53231973159805e-17	0.56896011556162	0.936	0.938	1.85542939158335e-12	RPS2
Successfu	19	9.69521462157513e-17	0.59610831421565	0.909	0.893	3.25158107978387e-12	RPS27A
Successfu	19	2.29649257680879e-16	0.66102429398903	0.962	0.955	7.70197680410131e-12	RPLP1
Successfu	19	3.52645174743519e-16	1.02592512341022	0.761	0.606	1.18270138705482e-11	MT1G
Successfu	19	6.09605734984032e-16	0.74712297182067	0.777	0.761	2.04449571398945e-11	S100A10
Successfu	19	8.35195592046603e-16	0.58229270941587	0.883	0.895	2.8010789766059e-11	RPL6
Successfu	19	1.07141686488299e-15	0.57666674809202	0.879	0.886	3.59331788144458e-11	RPL29
Successfu	19	1.13559982894187e-15	0.64759922749130	0.875	0.852	3.80857470630525e-11	RPLP0
Successfu	19	1.50287619639531e-15	0.79026517826951	0.633	0.511	5.04034618747059e-11	CD63
Successfu	19	1.57132815846819e-15	0.55966138408583	0.92	0.921	5.26992037787063e-11	RPS14
Successfu	19	1.62530412456992e-15	0.57519231858384	0.894	0.884	5.4509449729826e-11	RPL5
Successfu	19	3.55862958161137e-15	0.56354438916037	0.905	0.894	1.19349318908082e-10	RPL7A
Successfu	19	4.19935726906784e-15	0.54843089610746	0.909	0.891	1.40838044089997e-10	RPS16
Successfu	19	4.87169273458706e-15	0.51136490002636	0.898	0.895	1.63386830932581e-10	RPL14
Successfu	19	7.93514116325831e-15	0.54975604469116	0.917	0.893	2.66128764333357e-10	RPS3
Successfu	19	1.20786715433464e-14	0.55643709386170	0.924	0.903	4.05094486220753e-10	RPS8

Successfu	19	2.04612342461326e-14	0.80715380165285(	0.458	0.305	6.86228874146796e-10	BST2
Successfu	19	2.28755651951021e-14	0.66421243769827(	0.837	0.806	7.67200705513334e-10	RPS5
Successfu	19	4.23151649022996e-14	0.70795269273626	0.773	0.793	1.41916600049332e-09	MYL12A
Successfu	19	4.24543490008138e-14	0.67498479673475(	0.799	0.694	1.42383395678929e-09	REG1A
Successfu	19	6.28654034697436e-14	0.65614693014245(	0.686	0.613	2.10837990156826e-09	RPS10
Successfu	19	7.47546318658523e-14	0.58562522630104	0.777	0.754	2.50712084351695e-09	RPL36AL
Successfu	19	1.0582322285807e-13	0.49741639380369(	0.924	0.916	3.54909924821397e-09	RPL11
Successfu	19	1.10020004045363e-13	0.51725892745057	0.928	0.922	3.68985089567338e-09	RPL28
Successfu	19	1.21221333671174e-13	0.917180633117947	0.648	0.585	4.06552108866384e-09	IFITM2
Successfu	19	3.48659496533514e-13	0.47328060574724(	0.924	0.925	1.1693342194741e-08	RPS6
Successfu	19	5.367536516291e-13	0.54658679926202(	0.833	0.841	1.80016439683368e-08	FAU
Successfu	19	5.57093368345302e-13	0.49688515565310(	0.894	0.909	1.86837973875647e-08	RPLP2
Successfu	19	5.94707449547868e-13	0.38343597796415(	0.144	0.042	1.99452984429364e-08	C1QA
Successfu	19	6.53023220721201e-13	0.79964471611787(	0.341	0.191	2.19010927765476e-08	AGR2
Successfu	19	7.80444794856705e-13	0.49165449691369(	0.924	0.902	2.61745575299042e-08	RPS3A
Successfu	19	1.29788228365451e-12	0.42396725318241	0.973	0.987	4.3528376029205e-08	EEF1A1
Successfu	19	1.52843360084392e-12	0.58162341985417(	0.795	0.796	5.12606061051034e-08	PPIA
Successfu	19	2.05987895796683e-12	0.68891693690809(	0.754	0.768	6.90842204922914e-08	SH3BGRL3
Successfu	19	2.67871337103065e-12	0.50674471254593(	0.845	0.845	8.9838689037626e-08	UBA52
Successfu	19	3.10166197936817e-12	0.51174894196001(	0.928	0.921	1.0402353946405e-07	RPS18
Successfu	19	3.23261664308024e-12	0.35530684089368(	0.106	0.025	1.08415496975625e-07	SPINK4
Successfu	19	3.81541285895856e-12	0.460204119015284	0.898	0.894	1.27961316463752e-07	RPL9
Successfu	19	4.97778045757065e-12	0.56308869946614	0.807	0.826	1.66944800986004e-07	SERF2
Successfu	19	5.05597932190535e-12	0.57498663608810(	0.852	0.874	1.69567434498062e-07	RPL8
Successfu	19	7.42422181743194e-12	0.45923797135854(	0.867	0.851	2.48993551313032e-07	NACA
Successfu	19	8.9036169312604e-12	0.53913434601319(	0.769	0.768	2.98609504640611e-07	HINT1
Successfu	19	9.88535927906941e-12	0.36364831934809(	0.977	0.993	3.3153517950143e-07	B2M
Successfu	19	1.16666174173509e-11	0.44888538169393(	0.943	0.936	3.91275014943115e-07	RPL10
Successfu	19	2.03890393021155e-11	0.46302082742276(	0.886	0.871	6.83807600114349e-07	RPL19
Successfu	19	2.65228116201169e-11	0.43747246410507	0.917	0.929	8.89522056115482e-07	RPL13
Successfu	19	3.15880626120695e-11	0.58102227899776(	0.727	0.746	1.05940044388359e-06	ATP5MG

Successfu	19	3.93077787566607e-11	0.469502349977468	0.875	0.879	1.31830428394089e-06	RPL18A
Successfu	19	6.72687139833124e-11	0.648095768831917	0.58	0.497	2.25605812957233e-06	DBI
Successfu	19	7.07279064468509e-11	0.459689504433434	0.871	0.88	2.37207252641449e-06	RPS25
Successfu	19	7.44648733490019e-11	0.580519253540191	0.682	0.63	2.49740292237882e-06	PSME1
Successfu	19	7.9551067526365e-11	0.485165008226456	0.92	0.932	2.66798370269923e-06	RPL39
Successfu	19	9.72929111158711e-11	0.445871336535675	0.867	0.872	3.26300965300409e-06	RPL24
Successfu	19	1.29940225906361e-10	0.437821158737262	0.894	0.903	4.35793529644754e-06	RPS15A
Successfu	19	2.56331201475565e-10	0.503559556314125	0.807	0.808	8.5968358350875e-06	RPL27
Successfu	19	3.51725963988707e-10	0.459497489422668	0.867	0.876	1.17961853802532e-05	RPS7
Successfu	19	3.7881442003438e-10	0.444016929358188	0.864	0.885	1.2704678019113e-05	RPS20
Successfu	19	3.88547912814674e-10	0.445541904998547	0.886	0.895	1.30311198999786e-05	RPL30
Successfu	19	4.01376871295147e-10	0.471603415250618	0.864	0.875	1.34613775094967e-05	RPS13
Successfu	19	5.46679496942263e-10	0.489712659836815	0.822	0.795	1.83345369684496e-05	GAPDH
Successfu	19	8.47929480519122e-10	0.971422730704776	0.602	0.541	2.84378589176503e-05	NKG7
Successfu	19	8.83213032740404e-10	0.439845230594827	0.856	0.88	2.96211986920477e-05	RPL15
Successfu	19	9.20437137283586e-10	0.4819253599906	0.799	0.85	3.08696207102169e-05	ARPC2
Successfu	19	1.21441838330032e-09	0.480612871835355	0.807	0.825	4.07291637391262e-05	RPL10A
Successfu	19	1.42803379914316e-09	0.457716753486411	0.871	0.905	4.78933975556633e-05	CALM1
Successfu	19	1.48906754535872e-09	0.363465923437101	0.867	0.89	4.99403473362407e-05	HLA-C
Successfu	19	1.57645609462109e-09	0.568848549667773	0.629	0.585	5.28711845014021e-05	COX7A2
Successfu	19	2.05063358449157e-09	0.417141778903948	0.913	0.91	6.87741491566781e-05	RPS4X
Successfu	19	2.24239021991375e-09	0.598172985882157	0.477	0.363	7.52052831954674e-05	EMP3
Successfu	19	2.73904206089748e-09	0.365461629549896	0.902	0.914	9.18619926383798e-05	FTL
Successfu	19	2.99371991730796e-09	0.418620842462349	0.894	0.887	0.000100403378586674	RPS11
Successfu	19	3.45410994596846e-09	0.557730423155955	0.587	0.518	0.00011584393936789	UQCRH
Successfu	19	4.41830510241038e-09	0.386404496669762	0.966	0.976	0.000148181116524639	TPT1
Successfu	19	7.38351530399116e-09	0.418470045603316	0.841	0.865	0.000247628336265256	RPL35
Successfu	19	1.00781318369428e-08	0.45176572049809	0.86	0.872	0.000338000385547386	RPL27A
Successfu	19	1.03640327476742e-08	0.500244408169395	0.78	0.828	0.000347588930291498	MYL6
Successfu	19	1.03845898954155e-08	0.463949770026716	0.769	0.762	0.000348278375912444	BTF3
Successfu	19	1.51475397563941e-08	0.81114912896101	0.447	0.344	0.000508018188349945	MT1H

Successfu	19	1.76181145644953e-08	0.478571422993982	0.311	0.191	0.000590876326264042	NPC2
Successfu	19	1.87069316892191e-08	0.41801372904732	0.852	0.902	0.000627393074993029	RPS24
Successfu	19	2.16741785779175e-08	0.5767655234572	0.5	0.411	0.000726908601146196	GSTP1
Successfu	19	2.36773987497408e-08	0.608246473736227	0.322	0.205	0.000794092599268806	FABP5
Successfu	19	2.38525365013977e-08	0.67504776330985	0.564	0.546	0.000799966369183876	PSMA7
Successfu	19	2.64456127347821e-08	0.581017568259033	0.348	0.238	0.000886932959899122	C1QBP
Successfu	19	2.95180876987998e-08	0.768784953964938	0.583	0.556	0.000989977625242347	CKLF
Successfu	19	3.9630636345623e-08	0.550898305176028	0.568	0.508	0.0013291322817595	CLIC1
Successfu	19	4.02622564167984e-08	0.61618348458654	0.591	0.56	0.00135031555570658	PSMB9
Successfu	19	4.27168973851235e-08	0.488911749647209	0.72	0.727	0.00143263930450227	SRP14
Successfu	19	4.49621757691515e-08	0.368969797826763	0.837	0.857	0.0015079414509458	RPL35A
Successfu	19	4.95768836999301e-08	0.41413581729103	0.826	0.844	0.00166270952552825	RACK1
Successfu	19	5.75807849511462e-08	0.581446165656454	0.492	0.421	0.00193114436569154	TRMT112
Successfu	19	6.55727413392697e-08	0.460883027181283	0.841	0.812	0.00219917859903643	VIM
Successfu	19	6.94702539160518e-08	0.482272099808683	0.788	0.8	0.00232989337583654	ATP5F1E
Successfu	19	7.2473576398577e-08	0.617458645254893	0.617	0.589	0.00243061880525548	ARPC5
Successfu	19	7.71457298654178e-08	0.433489266276701	0.78	0.786	0.00258731348822638	PFDN5
Successfu	19	7.82354486189514e-08	0.393501169904956	0.811	0.845	0.00262386047578239	RPL37A
Successfu	19	7.9333210033679e-08	0.508606713708675	0.652	0.666	0.00266067719810953	CHCHD2
Successfu	19	8.37420855405275e-08	0.456350781979038	0.727	0.709	0.00280854206485821	COX4I1
Successfu	19	1.23006604337882e-07	0.390217657958783	0.875	0.884	0.00412539549628387	RPL21
Successfu	19	1.6729557857753e-07	0.465781343940174	0.439	0.344	0.0056107591143332	PPP1CA
Successfu	19	2.01080507180709e-07	0.487211041193265	0.496	0.41	0.00674383804982661	GTF3A
Successfu	19	2.02551094439188e-07	0.424978557418448	0.803	0.79	0.00679315860530149	RPL23
Successfu	19	2.24346781772523e-07	0.430760906890368	0.936	0.949	0.00752414236708688	TMSB4X
Successfu	19	2.44821013560672e-07	0.302314625715983	0.924	0.933	0.00821080715279783	RPS27
Successfu	19	3.0892655785992e-07	0.362558618597224	0.883	0.897	0.010360778897506	RPL32
Successfu	19	3.27868761889301e-07	0.316951169269659	0.924	0.914	0.0109960625362434	RPL13A
Successfu	19	4.42892469615527e-07	0.548861492593192	0.64	0.632	0.0148537276459655	RARRES3
Successfu	19	5.40906603279701e-07	0.524018842266697	0.633	0.622	0.0181409256607946	HCST
Successfu	19	6.40877121677617e-07	0.521668519642627	0.576	0.53	0.0214937369068239	SNRPD2

Successfu	19	7.673014140813e-07	0.54698418088688€	0.436	0.36	0.0257337548254586	KRT10
Successfu	19	1.02578394575687e-06	0.353780261445452	0.807	0.805	0.0344027419727939	RPL7
Successfu	19	1.4114763500616e-06	0.650583753971598	0.424	0.355	0.0473380938283658	MT1X
Successfu	22	2.11443268383537e-15	0.606727489214475	0.993	0.994	7.09138433504707e-11	MBNL1
Successfu	22	1.23935221628109e-13	0.75059758969202	0.967	0.938	4.15653946296351e-09	STK17B
Successfu	22	1.16644710237196e-12	0.658483844353151	0.98	0.885	3.91203029193507e-08	THEMIS
Successfu	22	1.83197339090057e-12	0.565209617725581	0.961	0.94	6.14407235840234e-08	ANKRD44
Successfu	22	4.41144530410559e-12	0.49433856491024	0.98	0.937	1.47951052609093e-07	SKAP1
Successfu	22	4.62791889543394e-12	0.874718178324039	0.836	0.756	1.55211143915063e-07	ITGA1
Successfu	22	1.8899998664616e-11	0.746416925398995	0.684	0.475	6.33868155213892e-07	GRAP2
Successfu	22	1.70352563953474e-09	0.670585603271846	0.816	0.803	5.71328428987162e-05	LCP1
Successfu	22	3.77295348662794e-09	0.67431289942766	0.75	0.591	0.000126537314034528	GPR174
Successfu	22	2.42992745941835e-08	0.494712054259767	0.493	0.301	0.000814949071339725	LZTFL1
Successfu	22	4.18897356402438e-08	0.437265176993259	0.309	0.156	0.0014048979539025	TIMP1
Successfu	22	6.26091744806409e-08	0.591870250849341	0.763	0.659	0.00209978649373174	GSK3B
Successfu	22	1.05082764236589e-07	0.331184916963116	0.993	0.992	0.00352426574696674	PTPRC
Successfu	22	1.3398828754428e-07	0.547762841533772	0.928	0.885	0.00449369918766005	PTPN22
Successfu	22	1.4790799760606e-07	0.617512903603916	0.625	0.482	0.00496053842371204	CYFIP2
Successfu	22	1.63330169523738e-07	0.644058008422699	0.697	0.594	0.00547776722548712	LNPEP
Successfu	22	4.41115043222249e-07	0.488562782728874	0.684	0.539	0.0147941163195878	MSH3
Successfu	22	6.81184008122666e-07	0.495957848516788	0.783	0.663	0.022845549264418	ZEB1
Successfu	22	7.26762121077256e-07	0.468845318977141	0.895	0.848	0.024374148016689	DOCK8
Successfu	22	8.90535862291537e-07	0.413344135818683	0.164	0.065	0.0298667917495336	CISH
Successfu	22	1.1257482485966e-06	0.484644115968178	0.763	0.656	0.0377553447614326	ROCK1
Successfu	22	1.20062576985504e-06	0.549283184297579	0.559	0.416	0.0402665870693984	CBL
Successfu	26	4.25595602042852e-129	1.50503016557368	1	0.995	1.42736253013132e-124	MT-ND3
Successfu	26	2.32175281187458e-97	0.988919962522526	1	0.994	7.78669458046496e-93	MT-ND2
Successfu	26	2.09643376958365e-87	1.07186761200965	0.976	0.565	7.03101957642966e-83	MT1G
Successfu	26	2.35797963437763e-68	0.862216274803922	0.771	0.287	7.90819209777569e-64	MT1H
Successfu	26	1.26095067409231e-60	0.674777248600012	1	0.995	4.22897637077078e-56	MT-ND1
Successfu	26	1.21661112726931e-56	1.05714655031895	0.938	0.574	4.0802703986358e-52	MT-ATP8

Successfu	26	5.52439901454332e-51	0.808197761115263	0.853	0.408	1.85277294149754e-46	DEFA5
Successfu	26	5.4717142551645e-43	0.68109953633532(	1	0.93	1.83510352689707e-38	SKAP1
Successfu	26	3.00151828729465e-42	0.37391764615794	0.247	0.041	1.00664920319288e-37	MT1B
Successfu	26	1.09992383317446e-37	0.45434464276460(	0.376	0.104	3.6889245517005e-33	MT1M
Successfu	26	3.60424952875428e-35	0.54360190328616	0.932	0.639	1.20879320695361e-30	PLCG2
Successfu	26	1.57121891031049e-34	0.54267470831918	0.6	0.251	5.26955398139932e-30	AC124319.2
Successfu	26	1.16680442541687e-33	0.29987466670754	0.124	0.01	3.91322868196309e-29	AC074099.1
Successfu	26	1.22221388347297e-33	0.33817958277920	1	0.999	4.09906092239165e-29	MT-ND4
Successfu	26	4.89426052444747e-33	0.49928923357788	0.532	0.21	1.64143709468919e-28	REG3A
Successfu	26	9.3030934123703e-33	0.49715075557920	0.882	0.554	3.12007146864075e-28	MT2A
Successfu	26	1.16128344288552e-32	0.99792970671671	0.856	0.594	3.89471241074947e-28	OSBPL3
Successfu	26	5.99364726040946e-30	0.49661731981387(	0.624	0.28	2.01014941819612e-25	MT1E
Successfu	26	9.35884465688598e-29	0.85608050873108	0.518	0.235	3.13876932102642e-24	SPDYA
Successfu	26	5.03991438976098e-27	0.48814016753187	0.953	0.763	1.69028648803804e-22	RNF213
Successfu	26	7.98145262415915e-26	0.51162797448884	0.991	0.933	2.6768195810905e-21	ANKRD44
Successfu	26	8.73081388867306e-26	0.53144629910465	0.974	0.856	2.92814036198317e-21	UTRN
Successfu	26	1.06259007157483e-25	0.59180658881038	0.574	0.275	3.56371458204768e-21	MTRNR2L1
Successfu	26	1.30387122694128e-25	0.48252884364776	0.965	0.743	4.37292332091566e-21	MT-ND4L
Successfu	26	3.92592531747758e-24	0.66703608676887(	0.544	0.277	1.31667683297563e-19	ZSWIM4
Successfu	26	2.41680696210259e-23	0.33276506366213(	0.4	0.157	8.10548718949968e-19	NTS
Successfu	26	4.91910441002135e-23	0.49387637336578(	0.997	0.95	1.64976923703296e-18	ARHGAP15
Successfu	26	8.16542292499783e-23	0.46853658557373	0.553	0.267	2.73851954058577e-18	AC090627.1
Successfu	26	3.01577283541565e-22	0.64386272095422	0.929	0.769	1.0114298935417e-17	DPYD
Successfu	26	4.31211758877343e-22	0.52346989859046	0.959	0.834	1.44619799692283e-17	KLF12
Successfu	26	5.65700241892003e-22	0.70595641175555	0.668	0.397	1.8972454712574e-17	EPG5
Successfu	26	7.87817700524879e-22	0.40022539467254	1	0.993	2.64218300402034e-17	MBNL1
Successfu	26	1.64682264339425e-21	0.44521311295630(	0.653	0.361	5.52311378141563e-17	APOB
Successfu	26	4.92367535136806e-21	0.47150651641942	0.697	0.414	1.65130223934182e-16	CUBN
Successfu	26	3.38330985761404e-20	0.44892196579358(	0.982	0.839	1.1346944600466e-15	LRBA
Successfu	26	1.1191958612111e-19	0.57178748802810	0.979	0.924	3.75355907932978e-15	JMJD1C
Successfu	26	2.87382775616769e-19	0.51820879116199	0.938	0.836	9.63824352863521e-15	DOCK8

Successfu	26	2.92130772332268e-18	0.45843384538747ε	0.718	0.473	9.79748184247962e-14	VWA8
Successfu	26	3.11834339808053e-18	0.39013919103990	0.865	0.594	1.04583000884825e-13	MTRNR2L8
Successfu	26	4.20449933867386e-18	0.50129928351027ε	0.932	0.799	1.41010498820444e-13	FOXN3
Successfu	26	5.54098813068957e-18	0.27327157000951	0.259	0.092	1.85833659927067e-13	CDH23
Successfu	26	6.17872799467323e-18	0.54883535833514	0.994	0.909	2.07222179485351e-13	NEAT1
Successfu	26	7.27011614717882e-18	0.42947548985188ε	0.947	0.832	2.43825155344083e-13	ARID1B
Successfu	26	9.25942159983868e-18	0.45826504956479ε	0.956	0.832	3.1054248161539e-13	RAB8B
Successfu	26	1.23651568399924e-17	0.59983813323597	0.532	0.302	4.14702630099667e-13	CATSPERB
Successfu	26	1.27809388926926e-17	0.46138239486061	0.988	0.932	4.28647128583125e-13	SIK3
Successfu	26	5.80671314179922e-17	0.41214678116457	0.991	0.943	1.94745545349662e-12	RABGAP1L
Successfu	26	1.14997000577761e-16	0.46867568220125	0.929	0.742	3.85676940537694e-12	NCOA2
Successfu	26	1.37143750694981e-16	0.51526403131344	0.891	0.707	4.59952711080826e-12	DNAJB6
Successfu	26	1.44947361228857e-16	0.30230979144187ε	0.353	0.156	4.86124460089342e-12	AL627171.2
Successfu	26	2.43343180311481e-16	0.69530040482401	0.697	0.506	8.16124358128644e-12	INPP4B
Successfu	26	2.53997460639975e-16	0.45647961979218	0.841	0.617	8.51856683494348e-12	TNIK
Successfu	26	3.27534652647789e-16	0.46462130486221	0.759	0.532	1.09848571805015e-11	DLG1
Successfu	26	3.33708803206506e-16	0.45210728588351	0.618	0.365	1.11919258419398e-11	MAF
Successfu	26	3.83800891038407e-16	0.44004343733749	0.924	0.748	1.28719142836461e-11	KDM4C
Successfu	26	5.04042794186403e-16	0.46479362477560ε	0.409	0.205	1.69045872314236e-11	NFE2L3
Successfu	26	6.36379749384111e-16	0.41456180135245	0.576	0.334	2.13429040348443e-11	NLRC5
Successfu	26	6.50851507126341e-16	0.67953545793491	0.903	0.743	2.18282578460032e-11	FBXO11
Successfu	26	9.40312934258288e-16	0.40693072114360ε	0.941	0.797	3.15362151891545e-11	ZFAND3
Successfu	26	1.00721410396305e-15	0.51537591456814	0.935	0.883	3.37799466187127e-11	THEMIS
Successfu	26	1.09576463143532e-15	0.49008817028414	0.888	0.721	3.67497542090776e-11	TBC1D5
Successfu	26	1.10936714457332e-15	0.63312596639858	0.656	0.433	3.72059552947001e-11	ITPKB
Successfu	26	1.14823175059052e-15	0.43267875753318	0.879	0.669	3.85093964513048e-11	CDKAL1
Successfu	26	1.23760849507266e-15	0.32370353534898	0.391	0.189	4.15069137077467e-11	NFATC1
Successfu	26	1.28448088135888e-15	0.88119458892319	0.712	0.552	4.3078919799014e-11	YES1
Successfu	26	1.28725358366302e-15	0.47210750330877	0.362	0.174	4.31719106888904e-11	PUS10
Successfu	26	1.50938572204967e-15	0.56940790002470	0.388	0.195	5.06217783461018e-11	CD44-AS1
Successfu	26	1.70258572461643e-15	0.34386379585855	0.679	0.417	5.71013200321859e-11	SI

Successfu	26	2.26365754530286e-15	0.68999516766294€	0.676	0.449	7.59185467543673e-11	ARHGAP25
Successfu	26	2.26758760895467e-15	0.37283426904108€	0.956	0.862	7.60503532291217e-11	ATXN1
Successfu	26	2.27178522302769e-15	0.51888767264271‹	0.812	0.611	7.61911328099027e-11	TMEM131L
Successfu	26	2.35395877675661e-15	0.49769773426473‹	0.838	0.64	7.89470694548632e-11	PRKCB
Successfu	26	2.38923542755819e-15	0.324606335501112	0.415	0.205	8.01301777694466e-11	NOL4L
Successfu	26	4.46082261451894e-15	0.36490177833228‹	0.971	0.873	1.49607068845736e-10	SYNE2
Successfu	26	4.89665285507875e-15	0.39368590431123€	0.818	0.587	1.64223943453631e-10	STK10
Successfu	26	6.05729724014691e-15	0.41769538695215‹	0.929	0.793	2.03149634840047e-10	LPP
Successfu	26	7.21803094574695e-15	0.40074655590933‹	0.785	0.569	2.42078321858461e-10	CARD11
Successfu	26	7.43005226558878e-15	0.35205088605600€	0.55	0.306	2.49189092883317e-10	MT-ND6
Successfu	26	7.60721305517314e-15	0.38663467200879€	0.462	0.251	2.55130711444397e-10	SKAP1-AS1
Successfu	26	8.27181335613124e-15	0.40280713770378‹	0.744	0.519	2.7742007633793e-10	MSI2
Successfu	26	8.75459709096211e-15	0.25990610215808‹	0.585	0.326	2.93611677236687e-10	MT1X
Successfu	26	1.32579409261918e-14	0.50158606881239‹	0.918	0.832	4.44644822782622e-10	MGAT5
Successfu	26	1.529450980074e-14	0.49054705672138‹	0.947	0.862	5.12947269697218e-10	EML4
Successfu	26	1.90614220283565e-14	0.41692509102866‹	0.906	0.705	6.3928197198702e-10	AKT3
Successfu	26	2.30988530942869e-14	0.43118133982544‹	0.835	0.631	7.74689335076193e-10	UBR5
Successfu	26	2.98566527765877e-14	0.55809265337482‹	0.532	0.322	1.0013324208212e-09	GPHN
Successfu	26	3.16549143762926e-14	0.49006581999812‹	0.679	0.456	1.0616425183521e-09	RUNX1
Successfu	26	3.73075010826212e-14	0.38747242578327‹	0.894	0.703	1.25121897130895e-09	DOCK2
Successfu	26	3.84090468492274e-14	0.40017709533401‹	0.968	0.867	1.28816261322939e-09	DOCK10
Successfu	26	4.49568276191708e-14	0.25983631543750‹	0.371	0.176	1.50776208469175e-09	MT1F
Successfu	26	5.46571570120648e-14	0.3097031428085	0.991	0.961	1.83309173187063e-09	HNRNPC
Successfu	26	6.3493939979191e-14	0.49195693903436‹	0.903	0.802	2.12945975902211e-09	BRAF
Successfu	26	9.69180109759208e-14	0.56306388812045€	0.75	0.561	3.25043625211043e-09	RIPOR2
Successfu	26	1.23448726838842e-13	0.33428748344746‹	0.976	0.93	4.14022340072108e-09	ZBTB20
Successfu	26	1.37174488497384e-13	0.30162083514152‹	0.444	0.235	4.60055799522525e-09	USP32
Successfu	26	1.42225322727527e-13	0.43072477069823‹	0.603	0.384	4.76995287363582e-09	SNTB1
Successfu	26	1.73439528054653e-13	0.32128356241708‹	0.497	0.278	5.81681489189696e-09	FAM193A
Successfu	26	1.99410431261042e-13	0.37330088967417‹	0.765	0.535	6.68782704363283e-09	NR3C2
Successfu	26	2.3334264246065e-13	0.34355412344515‹	0.95	0.85	7.82584554284528e-09	FOXP1

Successfu	26	2.5840513277304e-13	0.34366344325933{	0.841	0.648	8.66639134294223e-09	DENND1B
Successfu	26	3.30154713151391e-13	0.41521447714128	0.868	0.679	1.10727287696713e-08	ELMO1
Successfu	26	3.32381423487469e-13	0.38479040679385‥	0.615	0.394	1.11474081809227e-08	ANTXR2
Successfu	26	3.77651175143917e-13	0.57770309172380‥	0.668	0.48	1.26656651119767e-08	USP36
Successfu	26	4.2178429181079e-13	0.42507730246167‥	0.906	0.757	1.41458015787503e-08	SH3KBP1
Successfu	26	5.45967806497709e-13	0.32379205702676{	0.979	0.924	1.83106682943202e-08	PITPNC1
Successfu	26	5.58308057633849e-13	0.43818190576002	0.797	0.608	1.8724535636924e-08	EIF4G3
Successfu	26	5.61807752399923e-13	0.49505394243739‥	0.747	0.579	1.88419083999886e-08	ANK3
Successfu	26	6.18339682231437e-13	0.67828396740958‥	0.674	0.504	2.07378762626779e-08	USP12
Successfu	26	6.25400854103216e-13	0.47630981545200‥	0.779	0.618	2.09746938449137e-08	ZHX2
Successfu	26	6.8752471978503e-13	0.45166612166677‥	0.674	0.466	2.30582040521503e-08	JAZF1
Successfu	26	6.89722345987375e-13	0.38974323467592‥	0.479	0.276	2.31319080397246e-08	CD28
Successfu	26	8.17833108723828e-13	0.36054585504720‥	0.991	0.959	2.74284868003797e-08	CDC42SE2
Successfu	26	8.5131765431493e-13	0.42189919866809‥	0.438	0.252	2.85514914904141e-08	DPYD-AS1
Successfu	26	8.58593025191974e-13	0.35977764557350‥	0.915	0.751	2.87954928788884e-08	MACF1
Successfu	26	8.63845515980879e-13	0.26035622697633‥	0.765	0.488	2.89716509149667e-08	ANPEP
Successfu	26	9.01510340086527e-13	0.35666054000493	0.288	0.133	3.02348537858219e-08	DOK6
Successfu	26	9.36001551061014e-13	0.35866621392360‥	0.347	0.174	3.13916200194843e-08	DACH1
Successfu	26	1.00176528228335e-12	0.39461427605030‥	0.882	0.707	3.3597204037219e-08	PAN3
Successfu	26	1.16618581848593e-12	0.26896196511413‥	0.488	0.268	3.91115399803812e-08	GSE1
Successfu	26	1.26208190927428e-12	0.51217430659782‥	0.435	0.247	4.23277030732409e-08	SAMD3
Successfu	26	1.28789767658824e-12	0.36268330558885‥	0.847	0.636	4.31935122774165e-08	CEP85L
Successfu	26	1.31010637178233e-12	0.38465924215551‥	0.841	0.637	4.39383474968357e-08	GSK3B
Successfu	26	1.66042289505321e-12	0.41760912607280‥	0.862	0.685	5.56872630542946e-08	MYCBP2
Successfu	26	2.35095990786437e-12	0.25712766524893‥	0.321	0.155	7.88464933899553e-08	IQCN
Successfu	26	2.56494200291994e-12	0.41053726081814‥	0.897	0.738	8.6023024893929e-08	FTX
Successfu	26	2.96698332460615e-12	0.39650240073686‥	0.856	0.691	9.95066867406411e-08	ARL15
Successfu	26	3.14440593720593e-12	0.39959509900362	0.956	0.901	1.05457086322012e-07	ARIH1
Successfu	26	3.24573717843001e-12	0.40271778044181‥	0.694	0.474	1.08855533490186e-07	EPSTI1
Successfu	26	3.93984980890434e-12	0.3807221923868	0.924	0.831	1.32134682891034e-07	ESYT2
Successfu	26	4.34732323173677e-12	0.35175720719401‥	0.365	0.19	1.45800526545988e-07	AC090772.2

Successfu	26	4.4712830639479e-12	0.40532462363964	0.612	0.395	1.49957891398685e-07	MCTP2
Successfu	26	5.73554533281511e-12	0.42913645285513	0.682	0.477	1.92358719371953e-07	STK39
Successfu	26	5.74021362500486e-12	0.26926812462313	0.4	0.217	1.92515284555413e-07	AC009554.1
Successfu	26	5.92354626599724e-12	0.44029089303765	0.9	0.787	1.98663894669015e-07	CDK17
Successfu	26	6.24814247499095e-12	0.33918890800342	0.765	0.542	2.09550202326246e-07	MAP3K5
Successfu	26	6.41847970507761e-12	0.52067927451002	0.835	0.655	2.15262972348893e-07	JARID2
Successfu	26	6.80535224785051e-12	0.31796286791302	0.897	0.745	2.2823790368841e-07	EXOC4
Successfu	26	8.10588241990582e-12	0.49686211537379	0.703	0.511	2.71855084598801e-07	MLLT3
Successfu	26	8.58834303520893e-12	0.37083091886404	0.912	0.765	2.88035848714837e-07	MED13L
Successfu	26	9.14238747590169e-12	0.32122102255703	0.729	0.504	3.06617391166791e-07	EHMT1
Successfu	26	1.02030903690131e-11	0.32345591859252	0.644	0.423	3.42191244795961e-07	PTK2B
Successfu	26	1.04063564361278e-11	0.31141886670428	0.897	0.755	3.49008382154855e-07	VPS13B
Successfu	26	1.21987352134913e-11	0.38835543947820	0.571	0.365	4.0912118159007e-07	SLCO3A1
Successfu	26	1.30160022851979e-11	0.33083882973893	0.418	0.241	4.36530684640968e-07	ZSWIM5
Successfu	26	1.37072015980695e-11	0.50108028476244	0.65	0.452	4.59712127196055e-07	PRKX
Successfu	26	1.42027214167769e-11	0.33851403290706	0.468	0.266	4.76330870875864e-07	CLEC16A
Successfu	26	1.48167754321644e-11	0.34642944414947	0.924	0.775	4.96925014443928e-07	PPP3CA
Successfu	26	1.66966581746366e-11	0.29360202932195	0.691	0.462	5.59972521860962e-07	XYLT1
Successfu	26	1.67260259524405e-11	0.46712371608333	0.853	0.723	5.60957458392948e-07	MAML2
Successfu	26	1.68488974294465e-11	0.43784233462466	0.932	0.846	5.65078321988776e-07	CD44
Successfu	26	1.74760978928418e-11	0.34950400344783	0.753	0.529	5.8611337113013e-07	AC245297.3
Successfu	26	1.80488041593295e-11	0.29345099570780	0.385	0.21	6.05320793895592e-07	AC026347.1
Successfu	26	1.92479605850977e-11	0.28916461516568	0.521	0.313	6.45538102103006e-07	PI4KA
Successfu	26	1.9489672308167e-11	0.41661912800497	0.465	0.277	6.53644629871304e-07	SNAPC1
Successfu	26	2.03084242525912e-11	0.32586589107633	0.676	0.448	6.81103932583404e-07	SMAD3
Successfu	26	2.04337839300312e-11	0.48664772006449	0.615	0.424	6.85308245445387e-07	PCED1B
Successfu	26	2.84449630694932e-11	0.45744816040317	0.782	0.621	9.53987171424663e-07	MBD5
Successfu	26	2.90759238200032e-11	0.30358830584279	0.721	0.482	9.75148333075267e-07	PREX1
Successfu	26	3.08803334976949e-11	0.31454792977891	0.918	0.762	1.03566462484569e-06	ANKRD11
Successfu	26	3.2127723010711e-11	0.45287540089963	0.482	0.299	1.07749957433322e-06	ULK4
Successfu	26	3.28667268480869e-11	0.31430202204689	0.685	0.469	1.10228428503114e-06	VPS13A

Successfu	26	3.29080095952766e-11	0.33150449046192	0.821	0.635	1.10366882580639e-06	SLFN12L
Successfu	26	3.92164971699965e-11	0.46675158148204	0.626	0.433	1.31524288208734e-06	UST
Successfu	26	4.50769712708158e-11	0.37018089764097	0.779	0.582	1.51179146248062e-06	TRAPPC9
Successfu	26	4.67745072150289e-11	0.36366342226041	0.868	0.729	1.56872342297764e-06	TCF12
Successfu	26	4.77243731792531e-11	0.31475088041074	0.735	0.508	1.60058002768579e-06	PATJ
Successfu	26	5.07439380524932e-11	0.43942149782141	0.526	0.328	1.70185019440452e-06	ZNF827
Successfu	26	5.77501612614355e-11	0.38655597794590	0.456	0.267	1.93682490838603e-06	ELK3
Successfu	26	6.23452174160262e-11	0.34598131054163	0.676	0.457	2.09093390169869e-06	NFATC3
Successfu	26	6.35720747218151e-11	0.33549242811364	0.879	0.704	2.13208024202024e-06	CYTH1
Successfu	26	6.860434136734e-11	0.40605159210636	0.638	0.439	2.30085240077785e-06	FUT8
Successfu	26	6.92345271380071e-11	0.40876066733032	0.95	0.899	2.32198757115448e-06	SAMSN1
Successfu	26	7.04289615502101e-11	0.30567335700118	0.25	0.118	2.36204651247095e-06	SWT1
Successfu	26	7.11874543196358e-11	0.26957991393988	0.379	0.204	2.38748484297195e-06	ENPEP
Successfu	26	8.23418333171345e-11	0.39446715522056	0.768	0.571	2.76158040579006e-06	SMG6
Successfu	26	8.40030131670805e-11	0.36661612298665	0.741	0.523	2.81729305559755e-06	CDK6
Successfu	26	8.78022088591287e-11	0.42139252097237	0.985	0.944	2.94471048071746e-06	STAT4
Successfu	26	8.82224774196298e-11	0.28257876133532	0.926	0.779	2.95880544769954e-06	STAG1
Successfu	26	1.02441701355611e-10	0.36685248875523	0.497	0.306	3.43568978006448e-06	ESR2
Successfu	26	1.07802841837181e-10	0.33836886608381	0.9	0.727	3.61549170953538e-06	FAM49B
Successfu	26	1.14870069105107e-10	0.32064033727994	0.797	0.593	3.85251237764709e-06	ITPR2
Successfu	26	1.47246991715147e-10	0.27228586877219	0.479	0.291	4.93836960814259e-06	AC001226.2
Successfu	26	1.47380004198623e-10	0.51424385116280	0.774	0.611	4.94283058081341e-06	SP140
Successfu	26	1.62252137356203e-10	0.32330720487501	0.703	0.505	5.44161218265234e-06	UBR1
Successfu	26	1.65699860966901e-10	0.47271212317742	0.638	0.448	5.55724193710792e-06	TBL1X
Successfu	26	1.75290944242627e-10	0.35387963577351	0.906	0.776	5.87890768800922e-06	BIRC6
Successfu	26	2.03372019523361e-10	0.29648261482548	0.606	0.399	6.82069079077449e-06	BAZ2B
Successfu	26	2.13288115922094e-10	0.36383397727557	0.824	0.651	7.15325683179519e-06	FRYL
Successfu	26	2.29781897983171e-10	0.26001135479390	0.447	0.264	7.70642529455959e-06	NCOA6
Successfu	26	2.30355374733249e-10	0.39712887539693	0.882	0.784	7.72565855780369e-06	USP15
Successfu	26	2.38649562325813e-10	0.46457009032133	0.659	0.46	8.00382902128312e-06	PNPLA8
Successfu	26	2.51870487442934e-10	0.65437892850429	0.765	0.624	8.44723240786112e-06	NR4A3

Successfu	26	2.62249944347486e-10	0.28901434539515	0.768	0.555	8.79533863352599e-06	WDR26
Successfu	26	2.96310697105874e-10	0.31026157118314	0.541	0.342	9.9376681595368e-06	SBF2
Successfu	26	3.2614673446835e-10	0.32565708572266	0.538	0.352	1.09383091805995e-05	METTL15
Successfu	26	3.49640609177437e-10	0.49869466050829	0.632	0.466	1.17262467505929e-05	CAMK1D
Successfu	26	3.79924666458279e-10	0.48843085571614	0.374	0.218	1.27419134636778e-05	AF165147.1
Successfu	26	3.94235850742671e-10	0.46817030180347	0.297	0.156	1.32218819622077e-05	SETBP1
Successfu	26	4.13238001558398e-10	0.27473094890496	0.685	0.487	1.38591760962655e-05	MKL1
Successfu	26	4.15864492028649e-10	0.31467057581812	0.721	0.52	1.39472633336568e-05	MYO5A
Successfu	26	4.20998988089193e-10	0.44219032185469	0.582	0.391	1.41194640625354e-05	NDRG1
Successfu	26	4.51853165048273e-10	0.31653003215884	0.918	0.796	1.5154251449389e-05	CHST11
Successfu	26	4.55323690256298e-10	0.26010083800426	0.729	0.509	1.52706459238157e-05	INPP5D
Successfu	26	5.09215754112189e-10	0.44372768502551	0.838	0.696	1.70780779614146e-05	RFX3
Successfu	26	5.40812360709383e-10	0.36381027120643	0.844	0.701	1.81377649534713e-05	USP9X
Successfu	26	5.41436531413273e-10	0.28048627859260	0.75	0.551	1.81586983905383e-05	TBC1D22A
Successfu	26	5.58722932131518e-10	0.42839055818177	0.882	0.753	1.87384496978268e-05	ZSWIM6
Successfu	26	5.77261267143402e-10	0.47419764829715	0.653	0.456	1.93601883774554e-05	AL031772.1
Successfu	26	5.89060431492069e-10	0.32601603957763	0.509	0.323	1.9755908751381e-05	DGKH
Successfu	26	6.18718670068659e-10	0.38241810309691	0.368	0.213	2.07505867567627e-05	CCDC7
Successfu	26	6.19236388342948e-10	0.42327054923709	0.412	0.249	2.07679499922458e-05	FAM168A
Successfu	26	7.01105302129304e-10	0.25219850972017	0.262	0.129	2.35136696228126e-05	GAREM1
Successfu	26	7.13345213338115e-10	0.37920448273170	0.409	0.242	2.39241717649337e-05	HLCS
Successfu	26	7.17470088002578e-10	0.49530160122619	0.709	0.528	2.40625118114305e-05	RCOR1
Successfu	26	7.93296873779633e-10	0.34474391882695	0.491	0.3	2.66055905528213e-05	ABR
Successfu	26	8.22419102785133e-10	0.42032429511806	0.682	0.52	2.75822918692078e-05	NSMCE2
Successfu	26	8.32049928774653e-10	0.40135344212656	0.621	0.445	2.79052905112443e-05	FARS2
Successfu	26	8.64593986796388e-10	0.30263370139072	0.841	0.662	2.89967531291773e-05	CAMK2D
Successfu	26	9.45308494387865e-10	0.34344423097460	0.762	0.576	3.17037562847802e-05	SRPK2
Successfu	26	9.54942308170872e-10	0.31094656736835	0.844	0.678	3.20268551314347e-05	TRAF3
Successfu	26	1.04417603587861e-09	0.25167788649164	0.579	0.382	3.50195758912967e-05	RALGAPA2
Successfu	26	1.06528789210346e-09	0.27502605125866	0.791	0.585	3.57276253253658e-05	SFMBT2
Successfu	26	1.11963388844444e-09	0.26720177464507	0.603	0.402	3.75502813506496e-05	MIR4435-2HG

Successfu	26	1.17527243290897e-09	0.44371047420305ɛ	0.362	0.211	3.9416286854901e-05	AP3M2
Successfu	26	1.19489171662832e-09	0.25493600127859ʒ	0.479	0.29	4.00742783922807e-05	AGPAT3
Successfu	26	1.31352909599168e-09	0.28142624631583ɛ	0.812	0.627	4.4053138821369e-05	XRN1
Successfu	26	1.34195664568235e-09	0.27377004169818	0.371	0.208	4.50065419828947e-05	PRELID2
Successfu	26	1.35430834893219e-09	0.39569455811481ɛ	0.665	0.485	4.54207934064876e-05	ETV6
Successfu	26	1.49196666027727e-09	0.44508305880003ɛ	0.894	0.787	5.0037577852379e-05	CHD2
Successfu	26	1.67543139377306e-09	0.27205491157905ɛ	0.406	0.233	5.6190618084361e-05	MAML3
Successfu	26	1.67576471764042e-09	0.35019748964909ʒ	0.603	0.42	5.62017971002244e-05	TANC2
Successfu	26	1.81747544901221e-09	0.32251338242027ʌ	0.356	0.198	6.09544916089713e-05	AC131571.1
Successfu	26	1.93076399204286e-09	0.27868069569279	0.65	0.451	6.47539627651333e-05	ERCC1
Successfu	26	2.03545855506548e-09	0.36842517568657ɛ	0.718	0.529	6.82652090197862e-05	CHORDC1
Successfu	26	2.07601339915797e-09	0.31095889707673ɪ	0.788	0.586	6.96253373809599e-05	AP3B1
Successfu	26	2.07854620342103e-09	0.31125488143300ɛ	0.815	0.668	6.97102825703345e-05	COP1
Successfu	26	2.16587121800819e-09	0.29137433269467ɛ	0.356	0.196	7.26389889095587e-05	NAALADL2
Successfu	26	2.18837754781484e-09	0.27738722616470ɜ	0.932	0.781	7.33938061986139e-05	KMT2C
Successfu	26	2.27546284069285e-09	0.38045427648373ɪ	0.803	0.641	7.63144727511567e-05	NCOA1
Successfu	26	2.29962934540302e-09	0.254117630941198	0.882	0.746	7.71249689861267e-05	USP34
Successfu	26	2.32093588713785e-09	0.32136092016231ɛ	0.603	0.401	7.78395477828293e-05	XKR6
Successfu	26	2.3255586106578e-09	0.288401420897117	0.659	0.47	7.79945846842415e-05	ITFG1
Successfu	26	2.6341173147665e-09	0.37795905589227ʌ	0.471	0.299	8.8343026502639e-05	ATP10A
Successfu	26	2.84281679888809e-09	0.29660395354733ɪ	0.797	0.598	9.53423898011086e-05	CLASP1
Successfu	26	2.86358560675376e-09	0.29580839404546ɛ	0.721	0.521	9.60389340793076e-05	RERE
Successfu	26	3.12633882699696e-09	0.31988154473931ɛ	0.324	0.178	0.000104851151579824	SIL1
Successfu	26	3.18429170957386e-09	0.38078562628825ɜ	0.676	0.487	0.000106794775355688	BORCS5
Successfu	26	3.19258419926322e-09	0.25895395098451ɪ	0.756	0.556	0.00010707288887489	LCOR
Successfu	26	3.26299756864259e-09	0.27945874418039ʒ	0.65	0.463	0.000109434412457135	SENP7
Successfu	26	3.26713567573425e-09	0.31629007561744ʌ	0.312	0.169	0.000109573196292775	TGFBR3
Successfu	26	3.35274485088602e-09	0.41332057613007ɜ	0.818	0.689	0.000112444356809015	ZCCHC7
Successfu	26	3.35695849436398e-09	0.27162054100208	0.765	0.575	0.000112585673983979	PSPC1
Successfu	26	3.38630305360915e-09	0.28980249568829ʌ	0.821	0.669	0.000113569831811944	SMARCA2
Successfu	26	3.39639369124795e-09	0.29275550740435ɛ	0.482	0.309	0.000113908251617074	SORL1

Successfu	26	3.54380027609122e-09	0.30109576418673€	0.629	0.446	0.000118851973659547	PPP2R5E
Successfu	26	3.72796190591759e-09	0.46824703421561€	0.606	0.455	0.000125028386400664	PPP2R2B
Successfu	26	3.73734130786243e-09	0.29541628974081€	0.444	0.277	0.00012534295278309	DOPEY1
Successfu	26	3.76299696769267e-09	0.27021568547223€	0.403	0.238	0.000126203392302477	ANKS1A
Successfu	26	3.80986748850345e-09	0.43088636014950:	0.935	0.888	0.000127775335829429	RORA
Successfu	26	4.30470175029356e-09	0.32387730275011€	0.868	0.688	0.000144371087301345	KAT6A
Successfu	26	4.32975007416698e-09	0.45568430180698€	0.735	0.625	0.000145211157987412	PLCB1
Successfu	26	4.36692694157048e-09	0.26833980659121!	0.276	0.143	0.000146457995766391	ADCY9
Successfu	26	4.56115735317283e-09	0.25235463747981i	0.541	0.346	0.00015297209531071	PPP6R2
Successfu	26	4.63049800385549e-09	0.38182169027178i	0.353	0.203	0.000155297642053306	NEO1
Successfu	26	4.65803916252087e-09	0.28043446078826′	0.694	0.497	0.000156221317432625	GATAD2A
Successfu	26	6.14331868104694e-09	0.31708692677947	0.809	0.648	0.000206034621924952	ZEB1
Successfu	26	6.45447042829618e-09	0.44125880715213′	0.509	0.337	0.000216470029224197	NSUN6
Successfu	26	6.45558156442094e-09	0.2574408364258	0.624	0.417	0.000216507294507549	ITGAL
Successfu	26	6.88031689950775e-09	0.29322196208346€	0.568	0.384	0.000230752068175691	IGF2R
Successfu	26	7.28365666466985e-09	0.37650219449153€	0.674	0.501	0.000244279277219698	SGMS1
Successfu	26	8.18174331064503e-09	0.27228344368833′	0.444	0.279	0.000274399307152413	BMPR2
Successfu	26	8.23078086505349e-09	0.27892409996271:	0.826	0.669	0.000276043928652164	ERBIN
Successfu	26	8.27674111909581e-09	0.26409326822502i	0.868	0.654	0.000277585343652235	BBX
Successfu	26	9.65407133296835e-09	0.25240333189959′	0.224	0.11	0.000323778244365092	PGAP1
Successfu	26	9.71114408970967e-09	0.28172010459470€	0.515	0.342	0.000325692350480683	HMBOX1
Successfu	26	9.79064792259184e-09	0.44223149695361	0.518	0.349	0.000328358750027885	FAM13A
Successfu	26	9.84521548294098e-09	0.32405038183347€	0.397	0.238	0.000330188836866874	ICE2
Successfu	26	1.00641281937415e-08	0.32940905132300′	0.594	0.425	0.000337530731361701	DOCK9
Successfu	26	1.02163693167674e-08	0.31070172405045:	0.753	0.569	0.000342636594145744	DYM
Successfu	26	1.0310469439287e-08	0.26625270780638!	0.724	0.522	0.000345792524054806	PCNX2
Successfu	26	1.0912850310184e-08	0.37073424030199:	0.841	0.722	0.00036599517370295	SOS1
Successfu	26	1.12907690890346e-08	0.30045022285520⁄	0.862	0.705	0.000378669813708042	AAK1
Successfu	26	1.16930881130909e-08	0.36973293196197′	0.709	0.517	0.000392162789136841	UBR2
Successfu	26	1.27183530795105e-08	0.31736514403438€	0.974	0.941	0.000426548125580624	CD96
Successfu	26	1.35734823611252e-08	0.30335765829303i	0.8	0.631	0.000455227451427415	NUP98

Successfu	26	1.49328712967228e-08	0.343768848444034	0.9	0.761	0.000500818637549488	DYRK1A
Successfu	26	1.51907994642715e-08	0.287309826793903	0.838	0.688	0.000509469032432739	MAP3K2
Successfu	26	1.54614037635042e-08	0.261381207921838	0.618	0.426	0.000518544559420405	VPS13D
Successfu	26	1.58486026735735e-08	0.560489762001503	0.374	0.23	0.000531530436466307	HOMER1
Successfu	26	1.65731165068531e-08	0.342910438828183	0.768	0.602	0.000555829181406839	COG5
Successfu	26	1.77184830583183e-08	0.303619486699283	0.871	0.705	0.000594242484809879	HIVEP2
Successfu	26	1.79780751936738e-08	0.333341473527525	0.791	0.646	0.000602948685845433	RPS6KA5
Successfu	26	1.85536296522291e-08	0.385939432380629	0.559	0.385	0.000622251631276461	TRPS1
Successfu	26	1.88703401996575e-08	0.343809397977795	0.818	0.677	0.000632873469616112	MPP7
Successfu	26	2.02913512859146e-08	0.308832814861392	0.388	0.23	0.000680531339427004	BMPR1A
Successfu	26	2.29119855039594e-08	0.291163189968715	0.744	0.561	0.00076842216983179	RICTOR
Successfu	26	2.52144405630672e-08	0.291714427977101	0.685	0.515	0.000845641907604149	UVRAG
Successfu	26	2.58875086303106e-08	0.466223341543906	0.7	0.531	0.000868215264443357	ICOS
Successfu	26	2.65897421761122e-08	0.313078498726239	0.956	0.893	0.000891766773102452	PRKCH
Successfu	26	2.81870790013038e-08	0.326725544297033	0.653	0.466	0.000945338255545727	UBR4
Successfu	26	2.94135684845247e-08	0.329374239593233	0.621	0.431	0.00098647225983399	MAP4K4
Successfu	26	2.96615699293889e-08	0.255222719104496	0.679	0.498	0.000994789732291845	MYO9B
Successfu	26	2.99421319801439e-08	0.359890336270734	0.609	0.446	0.00100419922235007	PHF21A
Successfu	26	3.06658700236689e-08	0.305098697426667	0.447	0.289	0.00102847194885381	SLC20A2
Successfu	26	3.15255059641777e-08	0.320524644168368	0.629	0.462	0.00105730241902659	FCHSD2
Successfu	26	3.20616791141831e-08	0.257300423384249	0.962	0.902	0.00107528459413147	ARID4B
Successfu	26	3.29582978703333e-08	0.319520978069415	0.659	0.475	0.00110535539397524	PHTF2
Successfu	26	3.35896811493981e-08	0.346317210700422	0.582	0.394	0.00112653072638851	REPS1
Successfu	26	3.36992406313929e-08	0.415165959678197	0.491	0.333	0.00113020513229565	USP37
Successfu	26	3.61598216456593e-08	0.485232077967544	0.121	0.046	0.00121272809835212	FAM19A1
Successfu	26	3.66015327698188e-08	0.255732096772108	0.424	0.253	0.00122754220603418	AGAP1
Successfu	26	3.79262687044044e-08	0.284392464537039	0.679	0.473	0.00127197119980831	RFTN1
Successfu	26	3.82447314876787e-08	0.365811186286323	0.565	0.393	0.00128265180463377	MEF2A
Successfu	26	3.88637157658771e-08	0.255604436244855	0.521	0.338	0.00130341129935599	DGLUCY
Successfu	26	3.98001544404264e-08	0.483125285092446	0.897	0.784	0.00133481757962302	RASGEF1B
Successfu	26	4.00829361204607e-08	0.465318500501131	0.959	0.928	0.00134430151160801	GNG2

Successfu	26	4.04453922431114e-08	0.303824370710991	0.694	0.511	0.00135645756504947	PTPRA
Successfu	26	4.04671602276369e-08	0.250648064676013	0.9	0.797	0.00135718761971449	PCNX1
Successfu	26	4.38993252439838e-08	0.27009478918865	0.659	0.48	0.00147229557003273	TUT7
Successfu	26	4.55564781080575e-08	0.339603566679756	0.791	0.614	0.00152787316278803	DHX36
Successfu	26	4.63655758597691e-08	0.443478818176835	0.806	0.615	0.00155500868318494	AP001011.1
Successfu	26	4.72109260714945e-08	0.273271006385574	0.653	0.469	0.00158336003858578	AGO3
Successfu	26	4.81319781185062e-08	0.25468904188961	0.709	0.517	0.00161425028213846	MAN2A1
Successfu	26	5.00420408374753e-08	0.279271264989054	0.409	0.256	0.00167830996560725	DIP2A
Successfu	26	5.06980588321393e-08	0.420416927711134	0.826	0.696	0.00170031149711229	PPP1R16B
Successfu	26	5.14253290450275e-08	0.292088425467045	0.353	0.215	0.00172470268551213	PLEKHA5
Successfu	26	5.36328216342665e-08	0.280214509108407	0.503	0.338	0.00179873757197003	HIP1
Successfu	26	5.36831889969119e-08	0.38797560625094	0.982	0.896	0.00180042679257843	GLS
Successfu	26	6.36167766479904e-08	0.368545601311014	0.268	0.152	0.0021335794552203	KIAA0825
Successfu	26	6.36699385243278e-08	0.398948990077162	0.376	0.235	0.00213536239822891	DISC1
Successfu	26	6.39177181835311e-08	0.320698771036148	0.6	0.431	0.00214367243243926	ZNF831
Successfu	26	6.63596066993685e-08	0.302692456688886	0.762	0.621	0.00222556848948342	UBE2H
Successfu	26	6.65706109235396e-08	0.346417699312451	0.844	0.688	0.00223264514915367	BTBD9
Successfu	26	6.88404681637566e-08	0.483219116148381	0.844	0.74	0.00230877162127607	RORA-AS1
Successfu	26	6.88495694227557e-08	0.292089188948317	0.462	0.297	0.00230907685930038	FAM122C
Successfu	26	6.95219004651534e-08	0.4267242403686	0.609	0.457	0.00233162549780031	ARID5B
Successfu	26	8.18633222837679e-08	0.317136162312867	0.535	0.374	0.00274553210275301	RASGRF2
Successfu	26	9.00419855182154e-08	0.276666879198968	0.8	0.611	0.00301982811030991	ARID2
Successfu	26	9.25731989006427e-08	0.332667751044127	0.3	0.173	0.00310471994472975	SAMD4A
Successfu	26	9.30756099902681e-08	0.390475305801683	0.603	0.446	0.00312156980785361	SIK2
Successfu	26	9.42268883153697e-08	0.333043101026261	0.241	0.128	0.00316018138032087	PZP
Successfu	26	9.86433105025317e-08	0.393815906723312	0.703	0.535	0.00330829934763391	TNKS
Successfu	26	1.00214483521972e-07	0.325256309819074	0.868	0.772	0.00336099334835991	ELOVL5
Successfu	26	1.08835178062346e-07	0.253910495568438	0.129	0.053	0.00365011420185496	ZC3H12B
Successfu	26	1.09374022239863e-07	0.458137915199712	0.141	0.061	0.00366818595788052	EDARADD
Successfu	26	1.11921856103926e-07	0.31822250213908	0.638	0.463	0.00375363521001346	DOCK11
Successfu	26	1.27871772062106e-07	0.322379787529517	0.518	0.358	0.00428856349141892	ACYP2

Successfu	26	1.28670608424721e-07	0.402490782789679	0.779	0.622	0.0043153548653483	RAPGEF2
Successfu	26	1.30845469428136e-07	0.256986819676329	0.435	0.269	0.00438829535368083	CERS6
Successfu	26	1.33682511155292e-07	0.329228557901543	0.297	0.17	0.00448344405912617	CD40LG
Successfu	26	1.46034576238506e-07	0.253140814778424	0.871	0.697	0.00489770761788702	UBAC2
Successfu	26	1.49070709392083e-07	0.259289268588212	0.609	0.431	0.00499953345159168	CHD9
Successfu	26	1.55503855892708e-07	0.287438801555766	0.656	0.496	0.00521528831892963	SCAPER
Successfu	26	1.60898784542859e-07	0.27228046842295	0.732	0.547	0.00539622343599841	NABP1
Successfu	26	1.64120634552806e-07	0.279349884112335	0.438	0.281	0.00550427784163202	CPQ
Successfu	26	1.89287623507617e-07	0.288833813648767	0.812	0.646	0.00634832831719847	PAPOLA
Successfu	26	1.89465902416376e-07	0.373013269158848	0.679	0.535	0.00635430743524041	ZNF407
Successfu	26	1.91190266365407e-07	0.30420919532511	0.756	0.592	0.00641213915336304	ARHGEF3
Successfu	26	1.96814425624758e-07	0.442101087101207	0.494	0.35	0.00660076220660312	SIDT1
Successfu	26	1.96838114896372e-07	0.293987545515412	0.721	0.548	0.00660155669739454	CCNY
Successfu	26	2.21055496356235e-07	0.341989728586578	0.697	0.549	0.00741375923679542	NCALD
Successfu	26	2.29857924061377e-07	0.263511119945785	0.568	0.408	0.00770897505717046	ATG7
Successfu	26	2.31393144606742e-07	0.314541147527358	0.538	0.379	0.0077604632838209	UBR3
Successfu	26	2.31550417479233e-07	0.358650802516256	0.576	0.404	0.00776573790141851	PYHIN1
Successfu	26	2.3633388126681e-07	0.250784825224289	0.285	0.165	0.00792616570992626	ATG14
Successfu	26	2.44199531288837e-07	0.334245826204526	0.841	0.73	0.00818996388036501	BCL2
Successfu	26	2.4525381007065e-07	0.271172280857086	0.862	0.734	0.00822532228214947	ASH1L
Successfu	26	2.55955869013062e-07	0.270190886016141	0.671	0.495	0.00858424793496007	PBRM1
Successfu	26	2.56464068177609e-07	0.257969356931399	0.812	0.654	0.00860129191854064	KANSL1
Successfu	26	2.57047866066333e-07	0.260002010207094	0.224	0.118	0.00862087133213266	IFNG-AS1
Successfu	26	2.62305946812355e-07	0.332392094027607	0.544	0.392	0.00879721684419276	SCFD2
Successfu	26	2.70463396960009e-07	0.463693856976342	0.335	0.206	0.00907080140724479	ARHGAP10
Successfu	26	2.88151851989449e-07	0.252628868026173	0.282	0.159	0.00966403681202214	AC025180.1
Successfu	26	3.16469485341507e-07	0.264305492956319	0.609	0.431	0.0106137535993835	PPP3CC
Successfu	26	3.29078749805567e-07	0.300623700413492	0.468	0.324	0.0110366431109791	RIN3
Successfu	26	3.47220121863306e-07	0.35232676767898	0.647	0.519	0.0116450684470515	TSPAN5
Successfu	26	3.5992626604354e-07	0.275985876199438	0.65	0.473	0.0120712071105682	DIAPH2
Successfu	26	3.63507721909459e-07	0.270984215359739	0.341	0.207	0.0121913219773994	MAST2

Successfu	26	3.70364436388105e-07	0.26138834724656٤	0.532	0.365	0.0124212824675843	LUZP1
Successfu	26	3.70847740633301e-07	0.28988580652746	0.606	0.434	0.0124374915253596	MAN1A2
Successfu	26	3.72359054879319e-07	0.31444913519134	0.794	0.681	0.0124881779825426	PACS1
Successfu	26	3.72813412958171e-07	0.27757383161940	0.841	0.712	0.0125034162437911	FAF1
Successfu	26	3.84227401325124e-07	0.3408398912849	0.506	0.353	0.012886218585642	ELP4
Successfu	26	3.85150872805041e-07	0.33126606530703	0.779	0.649	0.0129171899721355	FBXL17
Successfu	26	3.85240891436896e-07	0.25113468399740	0.871	0.713	0.0129202090170106	CLEC2D
Successfu	26	3.95183489703001e-07	0.29045299659653	0.538	0.38	0.0132536638776593	MCPH1
Successfu	26	4.22053150218484e-07	0.30840588364859	0.794	0.666	0.0141548185520275	ADK
Successfu	26	4.65454778806868e-07	0.29540399163146	0.891	0.795	0.0156104223716247	ARAP2
Successfu	26	4.85469947324872e-07	0.3210137316302	0.806	0.665	0.0162816910933816	VTI1A
Successfu	26	5.25075560552612e-07	0.27521061920358	0.571	0.41	0.0176099841498135	PIBF1
Successfu	26	5.25106791840376e-07	0.25158457679777	0.244	0.136	0.0176110315847425	PLEKHG2
Successfu	26	5.26158654628283e-07	0.26344886832498	0.341	0.207	0.0176463089589233	AC092652.2
Successfu	26	5.86710442093357e-07	0.29101678427605	0.653	0.461	0.019677094806927	ATF6
Successfu	26	5.8782428423827e-07	0.29837081250513	0.403	0.266	0.0197144508447831	FAM222B
Successfu	26	6.35686151279161e-07	0.30687541030546	0.382	0.25	0.0213196421416005	BBS9
Successfu	26	6.51449883964597e-07	0.25350461867187	0.615	0.447	0.0218483262084047	OSBPL9
Successfu	26	6.54626191609442e-07	0.25326200794003	0.253	0.142	0.0219548532141975	IGF1R
Successfu	26	6.92151372523092e-07	0.31195912229058	0.621	0.462	0.0232133727316795	GLCCI1
Successfu	26	6.98735891135291e-07	0.26985587951849	0.894	0.765	0.0234342043168954	NIPBL
Successfu	26	7.11107909135344e-07	0.36785971756729	0.55	0.406	0.0238491370565812	KAT2B
Successfu	26	7.28293992396808e-07	0.27612805145229	0.9	0.846	0.0244255239170042	GPBP1
Successfu	26	7.30701208407296e-07	0.26544051005159	0.697	0.533	0.0245062571275639	UBAP1
Successfu	26	7.58247764985122e-07	0.45036602449515	0.715	0.557	0.025430113542071	FBXO34
Successfu	26	7.66740245045861e-07	0.45044814575338	0.315	0.195	0.0257149343383481	CRIM1
Successfu	26	7.71735592065904e-07	0.42359385457257	0.853	0.72	0.0258824682867063	TTC39C
Successfu	26	7.73365671791562e-07	0.40632645275361	0.679	0.518	0.0259371379005454	WWOX
Successfu	26	8.10909603248144e-07	0.26080936627894	0.8	0.613	0.0271962862737363	ACAP2
Successfu	26	8.41465694020426e-07	0.30698189308675	0.653	0.485	0.028221076446057	ASXL2
Successfu	26	8.7750758323657e-07	0.25563660495650	0.326	0.201	0.0294298493265881	TESPA1

Successfu	26	9.88729405187209e-07	0.30474775236584[illegible]	0.356	0.231	0.0331600067911686	A2M
Successfu	26	1.08923177973764e-06	0.26164208763926[illegible]	0.674	0.535	0.036530655428841	SPIDR
Successfu	26	1.12367079883175e-06	0.26107305067777[illegible]	0.391	0.252	0.0376856712512193	WDFY1
Successfu	26	1.14764227609231e-06	0.35588752085946[illegible]	0.291	0.178	0.038489626655584	AC009299.3
Successfu	26	1.31807375081709e-06	0.25759380130646[illegible]	0.6	0.441	0.0442055574549034	AFF1
Successfu	26	1.33900156518679e-06	0.25132396438865[illegible]	0.856	0.702	0.0449074344932346	CSNK1A1
Successfu	26	1.34482961746074e-06	0.29087203706346[illegible]	0.418	0.282	0.0451028957103984	KIAA1328
Successfu	26	1.35034696943037e-06	0.30280376259281	0.95	0.844	0.0452879366607559	TNRC6B
Successfu	26	1.36087134092255e-06	0.25914270677287[illegible]	0.235	0.132	0.0456409030318606	AC015923.1
Successfu	26	1.37511062106149e-06	0.26701838174047[illegible]	0.712	0.542	0.0461184600091604	FOSL2
Successfu	26	1.43554693527871e-06	0.26829218791786[illegible]	0.338	0.215	0.0481453731153773	UBE2O
Successfu	26	1.43863238582795e-06	0.25956513480921[illegible]	0.741	0.588	0.0482488529558979	TLK1
Successfu	33	2.53866082184637e-19	0.82989836219833[illegible]	1	0.936	8.51416066430837e-15	SIK3
Successfu	33	2.87311385752595e-17	0.79715442551703[illegible]	0.913	0.725	9.63584925537053e-13	CLEC2D
Successfu	33	5.66074653032686e-16	0.72620245692991[illegible]	0.993	0.935	1.89850117134102e-11	DENND4A
Successfu	33	1.03400058606823e-15	0.59470095217324[illegible]	0.986	0.937	3.46783116555564e-11	SKAP1
Successfu	33	1.98574727180444e-15	0.81128954067714[illegible]	0.978	0.799	6.65979920017774e-11	HERC1
Successfu	33	2.68589939568456e-15	0.83125436476522[illegible]	0.928	0.793	9.00796939324687e-11	PDE4D
Successfu	33	5.1412260692953e-15	0.69927420811676	1	0.994	1.72426439912026e-10	AC016831.7
Successfu	33	1.42847730806924e-14	0.71385263851188[illegible]	0.993	0.947	4.79082719580262e-10	STAT4
Successfu	33	1.48668174350228e-14	0.81766384094492[illegible]	0.957	0.869	4.98603323135795e-10	EML4
Successfu	33	2.41383621305004e-14	0.57389285052627[illegible]	0.514	0.226	8.09552389132724e-10	AL137856.1
Successfu	33	3.25392472819482e-14	0.61386320360336[illegible]	1	0.961	1.09130127534198e-09	CDC42SE2
Successfu	33	5.33441128922494e-14	0.80338594898091[illegible]	0.957	0.837	1.78905485818026e-09	ESYT2
Successfu	33	1.28416661760779e-13	0.52756220962971[illegible]	0.978	0.955	4.306838002133e-09	ARHGAP15
Successfu	33	1.58761405692153e-13	0.61297328113188[illegible]	0.993	0.929	5.32454002410343e-09	JMJD1C
Successfu	33	1.5924793894639e-13	0.597192211175547	0.986	0.904	5.34085737638404e-09	ARIH1
Successfu	33	1.61643098372109e-13	0.67709254679999	0.964	0.807	5.4211862332038e-09	BRAF
Successfu	33	1.63559826269966e-13	0.57721389665098[illegible]	0.993	0.865	5.48546945344213e-09	UTRN
Successfu	33	1.78439085704577e-13	0.65376455767478[illegible]	0.964	0.79	5.9844900563601e-09	PDE7A
Successfu	33	6.14742102650111e-13	0.66542520740952[illegible]	0.942	0.758	2.06172206386794e-08	FBXW7

Successfu	33	7.43709910005915e-13	0.57272231046997€	0.507	0.229	2.49425429617784e-08	AC109587.1
Successfu	33	7.91538675040026e-13	0.711279551153964	0.732	0.46	2.65466240834924e-08	TBL1X
Successfu	33	8.20200441637555e-13	0.62218320147575	0.674	0.371	2.75078824116403e-08	MSL2
Successfu	33	1.25029565947146e-12	0.67316600002872	0.942	0.846	4.19324158273538e-08	KLF12
Successfu	33	1.46056247820372e-12	0.52539948095243	0.971	0.792	4.89843443939963e-08	KMT2C
Successfu	33	1.55904854133526e-12	0.80288372407949	0.978	0.883	5.2287369979302e-08	PTPN22
Successfu	33	1.92184884340837e-12	0.80722805789181€	0.899	0.719	6.44549665102298e-08	CYTH1
Successfu	33	2.99653196326272e-12	0.54849773945546€	0.993	0.87	1.00497688983905e-07	PRRC2C
Successfu	33	4.82053212389281e-12	0.97686196662746	0.652	0.403	1.61671006371117e-07	PRMT9
Successfu	33	6.15532934981746e-12	0.54467586602666	0.819	0.539	2.06437435734178e-07	ZNF407
Successfu	33	7.7697744753521e-12	0.49480277464174	0.971	0.801	2.60582696354359e-07	WIPF1
Successfu	33	9.10053049874567e-12	0.44341155515116€	1	0.994	3.05213591866932e-07	MBNL1
Successfu	33	1.18465426598342e-11	0.63909588321360	0.696	0.408	3.9730934772552e-07	ANTXR2
Successfu	33	1.24040504821215e-11	0.65849088308862	0.688	0.408	4.1600704506939e-07	SEPT11
Successfu	33	1.65249660635905e-11	0.67903554958274€	0.899	0.64	5.54214311840699e-07	NUP98
Successfu	33	2.51777364328302e-11	0.74086270167590	0.899	0.703	8.44410924484259e-07	PPP1R16B
Successfu	33	2.90428643305263e-11	0.5311400609045	0.971	0.852	9.74039583917191e-07	LRBA
Successfu	33	2.92094692495779e-11	0.47451485149785	1	0.981	9.79627179692342e-07	FYN
Successfu	33	3.60108555388088e-11	0.54667953151982	0.79	0.498	1.20773207306057e-06	BTAF1
Successfu	33	3.78843012102324e-11	0.695756116098207	0.609	0.322	1.27056369398878e-06	TMEM184C
Successfu	33	5.48753600316906e-11	0.66029802296159	0.942	0.794	1.84040982474284e-06	CDK17
Successfu	33	5.76930464115863e-11	0.41015014583575	0.268	0.094	1.93490939055178e-06	AC024145.1
Successfu	33	5.80969670330319e-11	0.55784981150406	0.978	0.929	1.94845608035382e-06	PITPNC1
Successfu	33	5.9671109095066e-11	0.47885879393340€	0.978	0.912	2.00124965683032e-06	PPP2R5C
Successfu	33	1.90536661091625e-10	0.50928787690923	0.949	0.828	6.39021853969093e-06	XIST
Successfu	33	1.94408567138586e-10	0.70649675313107	0.928	0.828	6.5200745246939e-06	CAMK4
Successfu	33	2.22260789043683e-10	0.52685822727334	0.681	0.425	7.45418234294705e-06	RELL1
Successfu	33	2.32985893137767e-10	1.03147429912374	0.717	0.501	7.81388088405442e-06	BORCS5
Successfu	33	2.81173906378336e-10	0.43706658508746	0.986	0.875	9.43001047211663e-06	DOCK10
Successfu	33	3.02243097445073e-10	0.49177339834589	0.935	0.7	1.01366290021129e-05	KAT6A
Successfu	33	3.4309552533695e-10	0.48348287499647	0.804	0.589	1.15067377287506e-05	PSPC1

Successfu	33	3.58144614640173e-10	0.58754090017746	0.906	0.763	1.20114540858021e-05	ZSWIM6
Successfu	33	3.81221754467773e-10	0.48442616899315	0.659	0.402	1.27854152013402e-05	PSMA3-AS1
Successfu	33	4.32226349858716e-10	0.35066592274913	0.565	0.292	1.44960073215616e-05	AC090627.1
Successfu	33	4.42988155608788e-10	0.49386367082511	0.449	0.221	1.48569367628075e-05	CCDC7
Successfu	33	7.6853375841575e-10	0.60781168238585	0.986	0.929	2.57750851897474e-05	GNG2
Successfu	33	9.35436457509666e-10	0.57179908995987	0.899	0.681	3.13726679119592e-05	IVNS1ABP
Successfu	33	9.78568543886757e-10	0.43720807574800	1	0.962	3.2819231824874e-05	YWHAZ
Successfu	33	1.03611548511846e-09	0.53750335400252	0.906	0.728	3.47492411399029e-05	G3BP2
Successfu	33	1.19508018290347e-09	0.51297995870849	0.986	0.906	4.00805991742166e-05	ARID4B
Successfu	33	1.24328427261955e-09	0.63638230046352	0.761	0.501	4.16972679351146e-05	HECTD1
Successfu	33	1.24536118155375e-09	0.46767390832735	0.696	0.409	4.17669233069498e-05	MTFR1
Successfu	33	1.3281878542147e-09	0.47824567103925	0.993	0.933	4.45447642546525e-05	ZBTB20
Successfu	33	1.46335338855617e-09	0.83852905363582	0.877	0.735	4.90779459453969e-05	PDE3B
Successfu	33	1.59073544797314e-09	0.39828726021352	1	0.992	5.33500854541233e-05	PTPRC
Successfu	33	1.76430124261303e-09	0.44460954077980	0.899	0.676	5.91711350747559e-05	COP1
Successfu	33	2.02384869381677e-09	0.26726806492336	0.326	0.135	6.78758374932269e-05	RBM14-RBM4
Successfu	33	2.18182915789316e-09	0.48981371743895	0.986	0.952	7.31741862974209e-05	PARP8
Successfu	33	2.26716707964058e-09	0.88641814936712	0.833	0.633	7.60362495169859e-05	RAPGEF2
Successfu	33	2.27479784503725e-09	0.65842984767028	0.855	0.614	7.62921701268591e-05	GPCPD1
Successfu	33	2.31023745576897e-09	0.31992198215057	0.341	0.143	7.74807437915799e-05	ZNF26
Successfu	33	2.85191511184946e-09	0.50821689547049	0.819	0.553	9.56475290212071e-05	DUSP16
Successfu	33	2.93379849864149e-09	0.31490756803331	0.529	0.266	9.83937340474383e-05	SKAP1-AS1
Successfu	33	2.94602174951075e-09	0.41390064508628	0.471	0.236	9.88036774350915e-05	NLK
Successfu	33	2.96953657531336e-09	0.51274604013713	0.87	0.659	9.95923176628595e-05	ZEB1
Successfu	33	3.17910370898211e-09	0.61364522905267	0.572	0.326	0.000106620780191842	MT-ND6
Successfu	33	3.73550762222979e-09	0.44061153407389	0.428	0.21	0.000125281454634343	CD44-AS1
Successfu	33	3.85539622801593e-09	0.34447182011980	0.435	0.209	0.000129302278695198	EIF4G1
Successfu	33	3.86312584028344e-09	0.50677839558997	0.978	0.905	0.000129561514431426	RAB11A
Successfu	33	4.42396270681559e-09	0.50974449627418	0.826	0.592	0.000148370861261181	POLR2A
Successfu	33	6.50045172796431e-09	0.45984829069457	0.457	0.24	0.000218012150052467	POLR3E
Successfu	33	6.56261463306649e-09	0.45830681333834	0.862	0.68	0.000220096969563784	TBL1XR1

Successfu	33	7.36902703237424e-09	0.52835222297644{	0.645	0.382	0.000247142428611767	AP4B1-AS1
Successfu	33	7.52475878852757e-09	0.35496796600745{	0.862	0.629	0.000252365360249638	AP001011.1
Successfu	33	7.8179254728224e-09	0.74993422312025{	0.957	0.853	0.000262197584507518	CD44
Successfu	33	8.06802246139192e-09	0.56230935692286{	0.855	0.648	0.000270585337310162	UBR5
Successfu	33	8.50423960530396e-09	0.66239569609463{	0.855	0.703	0.000285215187882684	RANBP2
Successfu	33	8.8281888943999e-09	0.38462095603526{	0.63	0.36	0.000296079799140384	TTC14
Successfu	33	1.02853998902314e-08	0.44520565933863{	0.862	0.664	0.000344951741518582	DENND1B
Successfu	33	1.05647647051378e-08	0.77689343589562{	0.754	0.556	0.000354321078680912	BACH2
Successfu	33	1.11711079167236e-08	0.47336418208044{	0.906	0.737	0.000374656617311075	BCLAF1
Successfu	33	1.19908629006996e-08	0.51311786064515{	0.92	0.742	0.000402149559963664	SIPA1L1
Successfu	33	1.20790844945737e-08	0.54547162580501{	0.783	0.528	0.000405108335779012	FAM214A
Successfu	33	1.21968008940639e-08	0.39646750977453{	0.601	0.337	0.000409056308385114	CCNT2
Successfu	33	1.24188149874197e-08	0.66273702678869{	0.891	0.718	0.000416502217048082	HIVEP2
Successfu	33	1.33513789389699e-08	0.40972388840706{	0.667	0.416	0.000447778546855171	PTBP2
Successfu	33	1.50502367493041e-08	0.57244857773219{	0.862	0.674	0.00050475484009816	ADK
Successfu	33	1.53832563677586e-08	0.40362101610476{	0.964	0.884	0.000515923652061887	KMT2E
Successfu	33	1.61358477035155e-08	0.50203995926490{	0.457	0.239	0.000541164060280502	SEC23B
Successfu	33	1.61551020960222e-08	0.42187753134250{	0.804	0.552	0.000541809814096394	MON2
Successfu	33	1.6223372970603e-08	0.51504830969387{	0.87	0.685	0.000544099482688083	RNF149
Successfu	33	1.80655534098743e-08	0.34909489590415{	0.942	0.755	0.000605882530260363	USP34
Successfu	33	2.07172350442768e-08	0.49808359425747	0.971	0.94	0.000694814628914954	PABPC1
Successfu	33	2.3085856795482e-08	0.41853450300036{	0.928	0.784	0.000774253465206876	DPYD
Successfu	33	2.34101843829914e-08	0.57879659446356{	0.536	0.299	0.000785130763836765	FBXO33
Successfu	33	2.34424500494436e-08	0.63222408748735{	0.63	0.371	0.00078621288975824	GATA3
Successfu	33	2.36707559453707e-08	0.40005320294044{	0.804	0.547	0.000793869812895842	CDK13
Successfu	33	2.56028941578734e-08	0.65116504035871{	0.71	0.508	0.000858669864266759	RNF125
Successfu	33	2.90955263406214e-08	0.54101425641605{	0.754	0.555	0.000975805762411762	TM9SF3
Successfu	33	3.0362055088705e-08	0.34285484356916{	0.732	0.461	0.00101828260356499	SNRK
Successfu	33	3.56400848889344e-08	0.44063755638689{	0.717	0.488	0.00119529716700508	ATP9B
Successfu	33	3.62409476024571e-08	0.45544063814004{	0.674	0.432	0.00121544890069121	AP1G1
Successfu	33	3.83781537057398e-08	0.40026747302948{	0.891	0.744	0.0012871265189831	LINC01934

Successfu	33	4.08243621013646e-08	0.52929457609173[illegible]	0.964	0.818	0.00136916745615557	PPP1CB
Successfu	33	4.4478575588621e-08	0.50807313098890[illegible]	0.565	0.328	0.00149172246809117	TMEM156
Successfu	33	4.45955188588686e-08	0.35367810533681[illegible]	0.536	0.297	0.00149564451148874	SP4
Successfu	33	4.54051228092142e-08	0.53489293070765[illegible]	0.884	0.706	0.00152279700877543	RFX3
Successfu	33	4.55911957382952e-08	0.51417332324758[illegible]	0.928	0.772	0.00152903752267094	DYRK1A
Successfu	33	5.15026997650045e-08	0.55620518479775[illegible]	0.761	0.558	0.00172729754471872	VPS37B
Successfu	33	5.25254200436219e-08	0.43316566456991	0.949	0.773	0.00176159753742299	WNK1
Successfu	33	5.5768931430051e-08	0.42406982365227[illegible]	0.978	0.802	0.00187037842230105	LPP
Successfu	33	5.68964388605916e-08	0.56749726766577[illegible]	0.399	0.207	0.00190819276650652	AC010275.1
Successfu	33	5.76746554388544e-08	0.47944183127128[illegible]	0.957	0.86	0.0019342925941083	REL
Successfu	33	6.72213237920266e-08	0.42368492118846[illegible]	0.783	0.584	0.00225446875733699	LUC7L3
Successfu	33	7.10305269868896e-08	0.43746961355361[illegible]	0.855	0.668	0.0023822218140863	HIPK1
Successfu	33	7.30821258820262e-08	0.42293760880275[illegible]	0.855	0.651	0.00245102833783139	PCM1
Successfu	33	8.06353322933264e-08	0.27864719215277[illegible]	0.471	0.238	0.00270434777445358	SEC23A
Successfu	33	9.25417408769317e-08	0.47132382826203[illegible]	0.587	0.381	0.00310366490553054	SAFB
Successfu	33	9.6172798156226e-08	0.61289922434134	0.804	0.625	0.00322544330456351	SSBP2
Successfu	33	1.00001997271657e-07	0.59882745484103[illegible]	0.848	0.7	0.00335386698449682	MAP3K2
Successfu	33	1.0412612250584e-07	0.43219076767239[illegible]	0.899	0.692	0.00349218189660088	CHD1
Successfu	33	1.06475023909797e-07	0.42464153323985[illegible]	0.928	0.801	0.00357095935188677	ARAP2
Successfu	33	1.12945132051123e-07	0.45304219943301[illegible]	0.717	0.494	0.00378795383873057	USP36
Successfu	33	1.15857926875977e-07	0.41643415913741[illegible]	0.92	0.715	0.0038856431515665	PAFAH1B1
Successfu	33	1.16897279696443e-07	0.37446147086124[illegible]	0.862	0.646	0.0039205009664593	DIAPH1
Successfu	33	1.17479115766365e-07	0.41462640314027[illegible]	0.884	0.69	0.00394001458457234	TRAF3
Successfu	33	1.2112420818043e-07	0.37856595248279[illegible]	0.797	0.552	0.00406226369395528	ASCC3
Successfu	33	1.33361254430947e-07	0.25651405736315[illegible]	0.406	0.203	0.00447266975110509	GNL1
Successfu	33	1.3557329911217e-07	0.43187207079549[illegible]	0.428	0.234	0.00454685730562396	TSIX
Successfu	33	1.35923108211832e-07	0.51101286265970[illegible]	0.848	0.639	0.00455858920320841	ATP2B1
Successfu	33	1.40137706905122e-07	0.44950734807911[illegible]	0.986	0.875	0.00469993841418398	YPEL5
Successfu	33	1.42763273238424e-07	0.31655491557272[illegible]	0.609	0.356	0.00478799465787025	P2RY10
Successfu	33	1.47572893444716e-07	0.43470978062496[illegible]	0.79	0.555	0.00494929970034887	TENT5C
Successfu	33	1.50169201375468e-07	0.47940504402123[illegible]	0.877	0.673	0.00503637467573045	MKLN1

Successfu	33	1.54758847330125e-07	0.846777172921767	0.717	0.556	0.00519030222175772	AUTS2
Successfu	33	1.58532354539091e-07	0.64037997831201	0.949	0.793	0.00531685810653203	RALGAPA1
Successfu	33	1.59477389784313e-07	0.546559671649236	0.761	0.569	0.00534855269858629	FBXO34
Successfu	33	1.97520082892916e-07	0.444790966338694	0.841	0.625	0.00662442854006262	ARID2
Successfu	33	2.1296063953523e-07	0.481442056269715	0.652	0.453	0.00714227392873255	RSRC1
Successfu	33	2.36577888172871e-07	0.375674207435813	0.841	0.642	0.00793434921354175	SMG1
Successfu	33	2.39188782148669e-07	0.409941101120369	0.333	0.162	0.00802191337570205	AGO4
Successfu	33	2.64989623781718e-07	0.451185547267416	0.486	0.28	0.00888722200239126	GALC
Successfu	33	2.690918838742e-07	0.46400030990711	0.949	0.789	0.0090248036013729	USP15
Successfu	33	2.76866198190466e-07	0.394985033625705	0.725	0.494	0.00928553855491184	PHF20L1
Successfu	33	2.7932712963139e-07	0.328947200225067	0.638	0.39	0.00936807327357756	SAFB2
Successfu	33	2.97219642845264e-07	0.300483551486595	0.5	0.273	0.00996815238174445	PHF12
Successfu	33	3.03917363776741e-07	0.434738641145339	0.928	0.823	0.0101927805463443	KDM6A
Successfu	33	3.28957099022589e-07	0.644135740685822	0.841	0.704	0.0110325631870196	ZNF644
Successfu	33	3.51976096218616e-07	0.473433875052749	0.681	0.459	0.0118045743149799	SUSD6
Successfu	33	3.54758795342305e-07	0.424173349465693	0.623	0.389	0.0118979004781902	APOB
Successfu	33	3.67737181359332e-07	0.399688497935222	0.42	0.232	0.0123331695884293	MTMR6
Successfu	33	3.83527954975588e-07	0.449613214102522	0.862	0.655	0.0128627605539713	RPS6KA5
Successfu	33	3.91955074252368e-07	0.562684493930151	0.652	0.446	0.0131453892802759	MAP4K4
Successfu	33	4.1293682350896e-07	0.362641260059941	0.667	0.455	0.0138490751868435	DDHD1
Successfu	33	4.18779424571262e-07	0.515030856481438	0.478	0.29	0.014045024341271	AHCYL2
Successfu	33	4.24706089030797e-07	0.352135433514412	0.92	0.787	0.0142437928139149	BIRC6
Successfu	33	4.49621965707574e-07	0.673855083305807	0.92	0.84	0.0150794214859006	MGAT5
Successfu	33	4.64790585016747e-07	0.389666465683117	0.507	0.303	0.0155881466402917	IP6K1
Successfu	33	4.90702929086071e-07	0.694916768427749	0.638	0.449	0.0164571948356886	UST
Successfu	33	4.95387441343161e-07	0.385704395949315	0.551	0.333	0.0166143040077669	SH2D2A
Successfu	33	5.13035628540991e-07	0.29492023023478	0.449	0.24	0.0172061889100078	AKAP11
Successfu	33	5.16303582157935e-07	0.413185575074664	0.79	0.525	0.0173157895384128	INPP5D
Successfu	33	5.87443081437012e-07	0.310220359591375	0.826	0.563	0.0197016660652345	CREBBP
Successfu	33	6.02712492034213e-07	0.322810047411933	0.855	0.622	0.0202137715578434	OGT
Successfu	33	6.362937447893e-07	0.473459830278822	0.754	0.519	0.0213400196127435	PSME4

Successfu	33	6.41465255276538e-07	0.59299881196080	0.645	0.457	0.0215134617314645	SIK2
Successfu	33	6.448525144911e-07	0.41556947139053	0.935	0.888	0.0216270636310025	THEMIS
Successfu	33	6.55219420618658e-07	0.39963797244984	0.884	0.723	0.0219747489287086	PAN3
Successfu	33	6.62042305818112e-07	0.28446306783460	0.275	0.122	0.0222035748525278	AL157786.1
Successfu	33	7.0486494391637e-07	0.39071266679198	0.986	0.944	0.0236397604890672	CD96
Successfu	33	7.40434010120308e-07	0.41238617105986	0.928	0.759	0.0248326758314149	NCOA2
Successfu	33	7.58511164058472e-07	0.33014980201223	0.688	0.449	0.025438947420193	YTHDF3
Successfu	33	7.65537092368495e-07	0.34137503360611	0.522	0.307	0.0256745830038546	CDK8
Successfu	33	8.0776701128237e-07	0.60208157388394	0.725	0.554	0.0270908900243881	BCAS3
Successfu	33	8.30453031790905e-07	0.76829550104632	0.783	0.601	0.0278517337802034	BICDL1
Successfu	33	8.5822009113044e-07	0.43037289115934	0.754	0.541	0.0287829854163327	RCOR1
Successfu	33	8.81560507510595e-07	0.42707528504888	0.71	0.52	0.0295657763008903	ETF1
Successfu	33	9.62968960483852e-07	0.43067725590587	0.848	0.726	0.0322960529967074	AKT3
Successfu	33	9.76968614920354e-07	0.32760447292441	0.239	0.103	0.0327655734071988	AL365356.1
Successfu	33	9.78713784671524e-07	0.43114606067724	0.79	0.594	0.0328241029103136	CMIP
Successfu	33	1.01134786004163e-06	0.37325957504852	0.543	0.323	0.0339185845300763	COPB1
Successfu	33	1.02373069887965e-06	0.37878393654817	0.797	0.611	0.0343338801790258	ITPR2
Successfu	33	1.16974625928746e-06	0.35218924189899	0.993	0.938	0.0392309500439829	ANKRD44
Successfu	33	1.18426986585724e-06	0.42290704741521	0.797	0.598	0.03971804276112	TLK1
Successfu	33	1.20201339516785e-06	0.29690267203132	0.428	0.232	0.0403131252471393	AC009554.1
Successfu	33	1.20850646227417e-06	0.33920526606975	0.572	0.363	0.0405308897317511	RPS6KB1
Successfu	33	1.23632169970895e-06	0.40783154063743	0.652	0.462	0.0414637571648387	NBAS
Successfu	33	1.25897002597161e-06	0.39222437194548	0.558	0.352	0.0422233367310359	ZDHHC14
Successfu	33	1.31944202100893e-06	0.37455879569045	0.906	0.704	0.0442514465005974	PICALM
Successfu	33	1.33425003398133e-06	0.39745814313888	0.732	0.532	0.0447480776396658	MAN2A1
Successfu	33	1.34276093633829e-06	0.37653484204726	0.761	0.529	0.0450335162829134	CDC73
Successfu	33	1.351716651019e-06	0.37857987095204	0.71	0.494	0.0453338730418753	STK39
Successfu	33	1.4274149513989e-06	0.35316972829411	0.71	0.491	0.0478726426400162	CPSF6
Successfu	33	1.43679061781701e-06	0.46873894284042	0.957	0.931	0.048187083740347	SYTL3
Successfu	33	1.46755232783862e-06	0.39149343991582	0.928	0.796	0.0492187699710517	TRA2A
Successfu	59	1.51735050561571e-07	1.00506394440802	0.214	0.034	0.00508889012573396	PCLAF

Successfu	59	2.15062447732962e-07	1.23909832538453	1	0.947	0.00721276437206807	TMSB4X
Successfu	64	2.37045073073908e-82	0.61873897426936	0.246	0.003	7.95001766075272e-78	TFPI2
Successfu	64	1.40053634876786e-23	0.96551397631741	0.541	0.119	4.69711880649764e-19	NRG1
Successfu	64	1.96344276046998e-22	0.53019733334460	0.328	0.047	6.58499433006423e-18	IGFBP5
Successfu	64	1.2191301460258e-20	0.40247966554022	0.246	0.028	4.08871868374132e-16	FLT1
Successfu	64	5.69184371070853e-19	0.49811241668803	0.295	0.044	1.90893054369743e-14	COL4A1
Successfu	64	6.40966657700085e-19	1.2751694616095	0.721	0.237	2.14967397659455e-14	A2M
Successfu	64	8.88464967826843e-19	0.38183963253022	0.213	0.023	2.97973380909767e-14	COL1A1
Successfu	64	7.4141780282689e-18	0.69353188698416	0.541	0.138	2.48656702712082e-13	IFITM3
Successfu	64	2.20528038368126e-17	0.60770664598195	0.443	0.102	7.39606935079021e-13	MCTP1
Successfu	64	1.64897076888981e-16	0.52026636736141	0.393	0.084	5.53031816470265e-12	TCF4
Successfu	64	1.03931476247111e-15	0.85012288691008	0.246	0.037	3.48565385037562e-11	EPHA4
Successfu	64	1.69036360597635e-15	1.30041384837881	0.902	0.531	5.66914146172349e-11	FNDC3B
Successfu	64	3.71548997226525e-15	1.19970729863443	0.885	0.531	1.24610102689832e-10	AHNAK
Successfu	64	3.86097507878564e-14	1.03186235027177	0.918	0.586	1.29489382192313e-09	ARL4C
Successfu	64	5.34846840001278e-14	0.88649276652443	0.475	0.14	1.79376933199628e-09	RASA3
Successfu	64	1.03527526892437e-13	0.30097999777457	0.164	0.019	3.47210619691854e-09	TPM2
Successfu	64	1.75204427027065e-13	0.43249590108484	0.328	0.07	5.8760060736337e-09	COL3A1
Successfu	64	3.69818632182425e-13	0.57349311827899	0.508	0.157	1.24029772861342e-08	TIMP1
Successfu	64	4.75463210249097e-13	1.31312845099503	0.869	0.559	1.59460851453342e-08	BACH2
Successfu	64	8.02636178280879e-13	0.33770382406977	0.148	0.017	2.69188121471841e-08	TGFBI
Successfu	64	1.50849066763571e-12	0.42268041248918	0.18	0.026	5.05917600111664e-08	RGS9
Successfu	64	2.57662418687797e-12	1.15792810240269	1	0.855	8.64148219795134e-08	CD44
Successfu	64	3.02084647333683e-12	0.64010834841290	0.361	0.095	1.01313149022771e-07	ADGRL2
Successfu	64	3.99862313283246e-12	0.55297705249660	0.279	0.058	1.34105822628935e-07	IGFBP7
Successfu	64	6.22301073381775e-12	0.34382334500247	0.213	0.037	2.0870733399078e-07	COL6A3
Successfu	64	7.17244099786135e-12	1.03449701055262	0.852	0.478	2.40549326186274e-07	RUNX1
Successfu	64	8.68990929089449e-12	0.34917923969088	0.197	0.032	2.91442177798019e-07	FBXL7
Successfu	64	1.74230173874132e-11	0.26775565441908	0.164	0.023	5.84333157139065e-07	PRSS23
Successfu	64	2.06259916687024e-11	0.30527733708744	0.131	0.015	6.91754508584942e-07	FBN1
Successfu	64	3.68973186759742e-11	1.03137800763797	0.885	0.548	1.23746227375482e-06	FAM129A

Successfu	64	5.87625804069671e-11	0.89672405899710[illegible]	0.557	0.221	1.97077942168886e-06	KLF2
Successfu	64	1.12865662513089e-10	0.86745715281573[illegible]	0.574	0.231	3.78528858936398e-06	AGPAT4
Successfu	64	1.14664802093913e-10	0.87564281888334[illegible]	0.443	0.151	3.84562813262564e-06	P2RY8
Successfu	64	1.43999335867404e-10	0.33023818504120[illegible]	0.115	0.013	4.82944972632098e-06	MMP1
Successfu	64	1.59170234517677e-10	0.89859974286210[illegible]	0.672	0.308	5.33825132525386e-06	LMNA
Successfu	64	2.22056019687898e-10	0.40329074759293[illegible]	0.246	0.053	7.44731478829273e-06	IGFBP4
Successfu	64	2.24936971081384e-10	0.82144790270150[illegible]	0.934	0.661	7.54393613612745e-06	PRKCB
Successfu	64	3.26527953891734e-10	0.30051870642641[illegible]	0.115	0.013	1.0951094517621e-05	LINC00334
Successfu	64	3.30221691681169e-10	0.37450835731359[illegible]	0.246	0.054	1.1074975095603e-05	A2M-AS1
Successfu	64	3.64241123209622e-10	0.92930594041464	0.918	0.733	1.22159187902043e-05	IQGAP2
Successfu	64	3.84986721106385e-10	0.25232302743935[illegible]	0.164	0.026	1.29116846524659e-05	GNG11
Successfu	64	4.51300041903276e-10	0.309329111497228	0.18	0.031	1.51357008053521e-05	CLSPN
Successfu	64	5.45938405549911e-10	1.58820094925819	0.246	0.057	1.83096822453329e-05	GNLY
Successfu	64	6.64820959749997e-10	0.51948259225292[illegible]	0.295	0.078	2.22967653480954e-05	RBPMS
Successfu	64	6.78458640999161e-10	0.63849184352473	0.443	0.16	2.27541459018299e-05	RASSF2
Successfu	64	9.17526953938722e-10	0.28659343506756[illegible]	0.115	0.014	3.07720189811969e-05	CCR2
Successfu	64	9.26334043565101e-10	0.894887166113787	0.951	0.699	3.10673911530863e-05	ELMO1
Successfu	64	1.07304992510312e-09	1.08653538465777	0.885	0.645	3.59879483881086e-05	ATP2B1
Successfu	64	1.31535950274843e-09	0.68539480560688[illegible]	0.738	0.378	4.41145270031767e-05	SPTBN1
Successfu	64	2.04198512467752e-09	0.27532757490042[illegible]	0.18	0.034	6.84840971114347e-05	SYNE3
Successfu	64	2.20292768943071e-09	0.26803971790729[illegible]	0.131	0.019	7.38817888481271e-05	FAM155A
Successfu	64	3.32594668817032e-09	0.74692566091579[illegible]	0.164	0.029	0.000111545600027856	CSGALNACT1
Successfu	64	3.50568724984869e-09	0.45171538754684[illegible]	0.213	0.047	0.000117573738985425	LATS2
Successfu	64	3.52333573486656e-09	0.71231105713852[illegible]	0.41	0.137	0.000118165633875955	PZP
Successfu	64	4.14595497369745e-09	1.22003224079674	0.541	0.233	0.000139047037907865	MIR155HG
Successfu	64	5.20489719045357e-09	0.87407280566121[illegible]	0.639	0.346	0.000174561841973432	RUNX2
Successfu	64	5.40583750186005e-09	0.75877542645696[illegible]	0.459	0.17	0.000181300978137382	STX11
Successfu	64	6.00114124854376e-09	0.82649936666332[illegible]	0.951	0.703	0.000201266275193661	CEMIP2
Successfu	64	9.56091271881158e-09	0.69351835506367[illegible]	0.738	0.41	0.000320653890763503	AC008569.1
Successfu	64	9.93778677838834e-09	0.94468220854508[illegible]	0.902	0.66	0.000333293492973588	CCND3
Successfu	64	1.49239371880404e-08	0.69098170025336[illegible]	0.262	0.071	0.000500519005412498	CENPF

Successfu	64	2.26705334285569e-08	0.670765427306638	0.885	0.662	0.000760324350126942	FKBP5
Successfu	64	3.14873977282253e-08	0.60408051791077	0.885	0.621	0.00105602434500922	GPCPD1
Successfu	64	3.4436603913345e-08	0.94356147726654	0.852	0.666	0.00115493482204576	NAMPT
Successfu	64	3.91234877938176e-08	0.72681476881323	0.721	0.379	0.00131212353362905	IGLC3
Successfu	64	4.96394342532761e-08	0.57162439451595	0.59	0.266	0.00166480734598637	SAMD3
Successfu	64	5.55324208177227e-08	0.93996664208355	0.803	0.564	0.00186244632938478	NCALD
Successfu	64	6.3856606560041e-08	0.95957749050012	0.656	0.387	0.00214162287081066	SLCO3A1
Successfu	64	6.86093307818206e-08	0.69786353719123	0.213	0.052	0.0023010197357607	FAM19A1
Successfu	64	7.98575941539698e-08	0.26444669717066	0.148	0.028	0.00267826399273584	GNA15
Successfu	64	8.75451004153931e-08	0.69251135076853	0.852	0.581	0.00293608757773145	RIPOR2
Successfu	64	9.17430341467046e-08	0.68816969235106	0.328	0.108	0.00307687787921218	GZMK
Successfu	64	1.00091605203666e-07	0.98084823109126	0.754	0.473	0.00335687225532054	PRKX
Successfu	64	1.01415094759165e-07	0.25389157862026	0.18	0.04	0.00340125944803286	HPSE2
Successfu	64	1.0261145555506e-07	0.86007809414719	0.852	0.62	0.00344138299640559	TLE4
Successfu	64	1.4097899928298e-07	0.40079523180142	0.213	0.055	0.00472815367795257	COL4A2
Successfu	64	1.64580875214389e-07	1.09805067783778	0.197	0.049	0.00551971339294016	PDE7B
Successfu	64	1.77852576259472e-07	0.77274017293746	0.836	0.525	0.00596481970259017	INPP4B
Successfu	64	1.86586033025738e-07	0.50500247633571	0.344	0.123	0.00625772237561721	ITGB2
Successfu	64	1.90040680492658e-07	0.45796674226172	0.328	0.11	0.00637358434236277	LMNB1
Successfu	64	1.91515059611691e-07	0.57074505472813	0.902	0.726	0.00642303206925688	DOCK2
Successfu	64	1.98979667090875e-07	0.73152473606376	0.967	0.723	0.00667338007489376	CYTH1
Successfu	64	2.19176897681906e-07	0.67315832819195	0.967	0.809	0.00735075479445575	CHST11
Successfu	64	2.43756553285096e-07	0.58983869399355	1	0.956	0.00817510728407554	ARHGAP15
Successfu	64	2.520299423889e-07	0.25563376221527	0.18	0.041	0.00845258020783893	FGL2
Successfu	64	2.77392052177028e-07	0.66001936914919	1	0.953	0.00930317464591316	PARP8
Successfu	64	2.95461658569206e-07	1.14302650285924	0.721	0.43	0.00990919310509402	LYST
Successfu	64	3.07704854346599e-07	0.49861750380332	0.393	0.151	0.0103198054050762	PLXNC1
Successfu	64	3.23963870165363e-07	0.41167337970603	0.148	0.03	0.0108651002776059	TYMS
Successfu	64	3.58652070040084e-07	0.71697276814390	0.754	0.458	0.0120284731250043	RAP1GDS1
Successfu	64	3.76410499195933e-07	0.65099079223689	0.951	0.788	0.0126240553220332	RASA2
Successfu	64	4.07004568460156e-07	0.25743353347344	0.213	0.055	0.0136501192170167	CALD1

Successfu	64	4.11930457470583e-07	0.94856026770717€	0.803	0.619	0.0138153236826484	ITGA4
Successfu	64	4.82505948040648e-07	0.776316078311954	0.885	0.726	0.0161822844853872	ARHGAP26
Successfu	64	4.98446285508496e-07	0.64781824999140€	0.246	0.073	0.016716891523384	MIR181A1HG
Successfu	64	5.26771458479356e-07	0.58457844032721€	0.197	0.05	0.0176668611744807	SLC1A4
Successfu	64	5.61363330945679e-07	0.64811844480991∶	0.885	0.642	0.0188270033932562	CNOT2
Successfu	64	5.87543604036263e-07	0.52856331752080€	0.787	0.48	0.0197050373921682	DOCK11
Successfu	64	6.01562070788438e-07	0.80412280557738	0.656	0.387	0.0201751887301026	EZH2
Successfu	64	6.2170662301594e-07	0.48188167133498¢	0.557	0.268	0.0208507967227086	N4BP2
Successfu	64	6.49034361190733e-07	0.85207796099954	0.787	0.576	0.0217673144056148	PTPRJ
Successfu	64	6.649429987301e-07	0.43399464070171	0.197	0.05	0.0223008582914101	GZMH
Successfu	64	7.28594768868371e-07	0.64535267213499¢	0.836	0.622	0.0244356113583074	NFATC2
Successfu	64	7.78010235452805e-07	0.64690761562253¢	0.443	0.195	0.0260929072766162	SYNJ2
Successfu	64	8.05984532186321e-07	0.55469805767373	0.803	0.482	0.0270311092404648	CAMK1D
Successfu	64	8.50623472942923e-07	0.31906779254476€	0.164	0.038	0.0285282100355598	HS6ST1
Successfu	64	8.54025694069414e-07	0.57601973015455	0.951	0.769	0.0286423137277	MACF1
Successfu	64	8.73172666241213e-07	0.81078265793245	0.672	0.419	0.0292844648803978	ANTXR2
Successfu	64	9.14871663821811e-07	0.28679765461003	0.197	0.051	0.0306829658612559	LDB2
Successfu	64	9.2767037978431e-07	0.59025951374645	0.295	0.101	0.0311122091972062	AL355916.2
Successfu	64	9.380818870229e-07	0.57144530820655	0.918	0.711	0.031461390326974	PICALM
Successfu	64	1.033435791905e-06	0.39696105151376	0.131	0.026	0.0346593695889098	EFNA5
Successfu	64	1.0377129521273e-06	1.01129583022513	0.902	0.658	0.0348028169884455	ITK
Successfu	64	1.31419054537644e-06	1.05681792800018	0.721	0.488	0.0440753225108351	XYLT1
Successfu	64	1.37606416056904e-06	0.96227473490782	0.607	0.347	0.0461504398171646	DUSP10
Successfu	64	1.39330396485674e-06	0.61751685886183	0.492	0.253	0.0467286283733653	FLNA
Successfu	191	1.49159161822499e-11ٍ	0.82614332651947	0.573	0.072	5.00249996920298e-11	NRG1
Successfu	191	1.87456473604876e-65	1.70953123697299	0.649	0.197	6.28691521176032e-61	A2M
Successfu	191	1.33716906864421e-57	1.36868287840686	0.846	0.441	4.48459762241894e-53	RUNX1
Successfu	191	1.92002939723603e-54	0.46961665747818	0.312	0.046	6.4393945924502e-50	COL3A1
Successfu	191	8.69888527133963e-53	1.42541062400416	0.437	0.106	2.91743214230188e-48	PZP
Successfu	191	9.54189832922953e-50	1.0281145760178	0.978	0.86	3.200161861657e-45	EML4
Successfu	191	3.90241339730236e-49	1.06676391087745	0.943	0.701	1.30879140518727e-44	CYTH1

Successfu	191	1.11621880114415e-47	1.49109605723475	0.824	0.497	3.74357461527726e-43	PHACTR2
Successfu	191	5.53550230980221e-46	1.18435408206907	0.846	0.492	1.85649676466147e-41	INPP4B
Successfu	191	1.1885773947375e-43	0.88832204426346	0.659	0.258	3.98625086647064e-39	CD28
Successfu	191	2.13600426614141e-43	1.24763908932223	0.656	0.275	7.16373110778505e-39	BTBD11
Successfu	191	1.89348673748075e-42	0.43003558583281	0.337	0.071	6.35037582016294e-38	ADGRL2
Successfu	191	1.02146825556491e-41	0.85501091002082	0.91	0.588	3.4258002355136e-37	COG5
Successfu	191	2.19333758759042e-41	0.99675171432444	0.946	0.784	7.35601560126074e-37	CDK17
Successfu	191	3.82366316742767e-41	0.84375395092640	0.989	0.933	1.28238015309189e-36	SIK3
Successfu	191	6.64382367131505e-41	0.96065864195144	0.871	0.535	2.22820558288564e-36	MAP3K5
Successfu	191	1.6778426760463e-40	0.79263179299144	0.993	0.857	5.62714876692406e-36	UTRN
Successfu	191	3.04471818716228e-39	0.74920753319364	0.996	0.952	1.02113758561049e-34	ARHGAP15
Successfu	191	4.59821997100989e-36	0.38295460193939	0.28	0.057	1.5421510138773e-31	RBPMS
Successfu	191	6.35459007884853e-35	0.30192641915624	0.222	0.037	2.13120242064422e-30	IGFBP4
Successfu	191	1.49530907248791e-34	0.71586430196909	0.943	0.752	5.01496756730996e-30	MACF1
Successfu	191	5.65468309003206e-34	0.89024774210736	0.889	0.618	1.89646761473495e-29	TNIK
Successfu	191	1.22125541051082e-33	1.32048882124622	0.38	0.119	4.09584639577119e-29	AL357793.1
Successfu	191	1.44071079056194e-33	0.67507790850343	0.398	0.126	4.83185584938662e-29	PLXNC1
Successfu	191	1.53311130763103e-33	1.0805329820417	0.842	0.614	5.14174870353295e-29	PLCB1
Successfu	191	3.28668602829332e-33	0.82811027149042	0.864	0.56	1.10228876016901e-28	PAG1
Successfu	191	5.37200997778746e-33	0.78202950252504	0.384	0.117	1.80166470635036e-28	RASA3
Successfu	191	1.03458344650869e-32	0.81728281390183	0.401	0.127	3.46978596290085e-28	P2RY8
Successfu	191	1.11930186810747e-32	0.74398907927764	0.91	0.679	3.75391460525882e-28	ELMO1
Successfu	191	1.9493298655876e-32	0.72333366805257	0.975	0.875	6.5376625032077e-28	SYNE2
Successfu	191	4.14329204287434e-32	0.51228258196808	0.204	0.034	1.3895772853392e-27	AL357793.2
Successfu	191	5.5334513895885e-32	0.57984216095236	0.24	0.049	1.85580892704019e-27	EPB41L4A
Successfu	191	1.13476301298998e-31	0.82879251751531	0.473	0.176	3.8057681929658e-27	LRIG1
Successfu	191	2.54751042970456e-31	1.03192537000585	0.882	0.62	8.54384047914316e-27	ATP2B1
Successfu	191	1.85229802579134e-30	0.29497018542613	0.204	0.036	6.21223711889898e-26	PRR16
Successfu	191	3.52487933847388e-30	0.77753477515604	0.753	0.416	1.18217403253737e-25	ZNF831
Successfu	191	1.21904438006496e-29	0.80829738769222	0.964	0.845	4.08843104186186e-25	CD44
Successfu	191	2.7125383187439e-29	0.51975656663827	0.305	0.08	9.09731101340329e-25	PLEK

Successfu	191	4.23177115097743e-29	0.70455269622187	0.305	0.087	1.41925140861481e-24	KIAA1324L
Successfu	191	4.99556456742032e-29	0.90320251451447	0.867	0.596	1.67541244462143e-24	GPCPD1
Successfu	191	6.73946818792976e-29	0.32378643457587	0.208	0.04	2.26028284086788e-24	A2M-AS1
Successfu	191	2.72322871029118e-28	0.59403760529695	0.996	0.933	9.13316444857456e-24	SKAP1
Successfu	191	3.37644749882839e-28	0.82072139098171	0.975	0.834	1.13239296215707e-23	ANKRD12
Successfu	191	1.08290279650372e-27	0.67713421692623	0.982	0.835	3.63183939891417e-23	KLF12
Successfu	191	1.89508886574173e-27	0.68999707998582	0.423	0.159	6.35574903792462e-23	TGFBR3
Successfu	191	2.02917054066223e-27	0.73165840524753	0.251	0.063	6.805432159273e-23	FRY
Successfu	191	5.33432125283914e-27	0.67168806035575	0.892	0.628	1.78902466177719e-22	DIAPH1
Successfu	191	1.13773932451727e-26	0.59674665693774	0.201	0.041	3.81575014656602e-22	COLQ
Successfu	191	1.40279114349393e-26	0.91714026294183	0.283	0.08	4.70468093704994e-22	NELL2
Successfu	191	1.62146846930296e-26	0.62495689863638	0.932	0.704	5.43808095234826e-22	DOCK2
Successfu	191	2.12110300945048e-26	0.95093567286483	0.871	0.6	7.11375527309502e-22	OSBPL3
Successfu	191	3.56392179130371e-26	0.71785166642736	0.871	0.639	1.19526809036744e-21	CEP85L
Successfu	191	5.95386426763403e-26	0.64637886293536	0.939	0.782	1.9968069980791e-21	PDE7A
Successfu	191	6.56702960947046e-26	1.33325623341258	0.326	0.108	2.2024503904242e-21	IFNG-AS1
Successfu	191	6.86483206284998e-26	0.75886450247410	0.76	0.444	2.30232737723863e-21	PRKX
Successfu	191	8.10537100245306e-26	0.55286344112752	0.606	0.294	2.71837932680271e-21	ACSL4
Successfu	191	2.58647297620564e-25	0.61916524193066	1	0.993	8.67451306759849e-21	MBNL1
Successfu	191	4.36995019116313e-25	0.25133556928936	0.165	0.029	1.46559389511229e-20	RGS5
Successfu	191	5.73719189609335e-25	0.93195502007584	0.932	0.807	1.92413941811179e-20	RNF19A
Successfu	191	5.8579552059099e-25	0.37716377087383	0.219	0.051	1.96464101695806e-20	PHACTR2-AS1
Successfu	191	7.64627979028387e-25	0.54216510769862	0.287	0.084	2.5644093160654e-20	RGPD2
Successfu	191	1.42438576719175e-24	0.63300246988516	0.545	0.261	4.77710498600768e-20	ELK3
Successfu	191	3.60337029966663e-24	1.26790540289043	0.337	0.119	1.20849833110219e-19	SLC4A10
Successfu	191	4.11720342637581e-24	0.62695200722534	0.935	0.796	1.38082768513792e-19	LPP
Successfu	191	5.08743451550593e-24	0.94533210517166	0.434	0.184	1.70622378781038e-19	MAST4
Successfu	191	5.63440209092544e-24	0.30226403398101	0.219	0.053	1.88966577325458e-19	AL024508.1
Successfu	191	6.1145149517769e-24	0.84723627524461	0.577	0.293	2.05068602452694e-19	ST6GAL1
Successfu	191	7.38883425662606e-24	0.54351228179161	0.971	0.844	2.47806723298725e-19	LRBA
Successfu	191	1.24858210528734e-23	0.54336434778813	0.878	0.611	4.18749466471267e-19	AP001011.1

Successfu	191	2.30164947520941e-23	0.74954871550300	0.638	0.341	7.71927200995731e-19	SYNE1
Successfu	191	2.86223653353572e-23	0.57622047587467	0.978	0.796	9.59936888617208e-19	ZFAND3
Successfu	191	6.67594819493345e-23	0.53684225954561	0.964	0.811	2.23897950561678e-18	KDM6A
Successfu	191	1.31776884548338e-22	0.86287848048125	0.803	0.56	4.41953315398215e-18	RIPOR2
Successfu	191	2.33137984180953e-22	0.46141161891004	0.297	0.095	7.81898171346079e-18	C22orf34
Successfu	191	3.27652521047635e-22	0.78493165949361	0.921	0.733	1.09888102508956e-17	RORA-AS1
Successfu	191	3.342830772974e-22	0.28386794859328	0.229	0.061	1.12111858464002e-17	LRRC37A2
Successfu	191	3.67610087537439e-22	0.67588453254279	0.505	0.249	1.23289071158306e-17	ATF7IP2
Successfu	191	4.08722587823398e-22	0.83728450122081	0.961	0.886	1.37077381504211e-17	RORA
Successfu	191	5.22112463773284e-22	0.82514257616001	0.462	0.211	1.75106078100284e-17	AF165147.1
Successfu	191	7.13396340636521e-22	0.52762707593995	0.43	0.188	2.39258864722676e-17	XRRA1
Successfu	191	7.68397240419673e-22	0.545373522111227	0.559	0.275	2.5770506649195e-17	AC090627.1
Successfu	191	8.38792166244395e-22	0.60746771746611	0.685	0.391	2.81314116715045e-17	MTFR1
Successfu	191	9.31718591419316e-22	0.70544556720457	0.896	0.691	3.1247978119021e-17	TNFAIP8
Successfu	191	9.35350764747328e-22	0.61814848099436	0.892	0.69	3.13697939480959e-17	KAT6A
Successfu	191	9.39036773406154e-22	0.67288083697404	0.943	0.772	3.14934153064956e-17	DPYD
Successfu	191	1.45924905977016e-21	0.73961879936426	0.599	0.328	4.89402949665716e-17	HIVEP3
Successfu	191	1.4786219968647e-21	0.46979330919013	0.993	0.934	4.95900245308482e-17	ANKRD44
Successfu	191	1.60346298180714e-21	0.66910043731804	0.835	0.568	5.3776941483848e-17	CARD11
Successfu	191	2.2767422816277e-21	0.61349534806854	0.713	0.424	7.63573826412299e-17	MAP4K4
Successfu	191	2.69497324083959e-21	0.46800719436561	0.996	0.862	9.03840125512782e-17	PRRC2C
Successfu	191	3.06495565017803e-21	0.65571085994183	0.796	0.538	1.02792482595671e-16	ATP2B4
Successfu	191	3.18275674807428e-21	0.73604661026129	0.864	0.638	1.06743295816915e-16	TIPARP
Successfu	191	4.4815213581129e-21	0.42236855319053	0.484	0.214	1.50301263308391e-16	TSIX
Successfu	191	1.41140150523237e-20	0.73691089920721	0.892	0.706	4.73355836824832e-16	HIVEP2
Successfu	191	1.94112293264317e-20	0.41453048717173	0.616	0.321	6.51013809149866e-16	MYO1F
Successfu	191	2.1989201758865e-20	0.62204365711247	0.993	0.944	7.37473848588816e-16	STAT4
Successfu	191	2.43558152782206e-20	0.65494746290641	0.624	0.364	8.16845332800961e-16	SLCO3A1
Successfu	191	2.44055926998798e-20	0.48198225856990	0.971	0.833	8.1851476796857e-16	ARID1B
Successfu	191	2.60546825788017e-20	0.55955841167214	0.301	0.107	8.7382194432785e-16	AL356272.1
Successfu	191	2.98001490792352e-20	0.59915885523817	0.968	0.878	9.99437399819391e-16	KMT2E

Successfu	191	3.3176360506767e-20	0.71161236770258€	0.746	0.512	1.11266877867595e-15	ATM
Successfu	191	3.32896921002925e-20	0.50780654241976€	0.444	0.2	1.11646969365961e-15	DPP4
Successfu	191	3.92160185838725e-20	0.57028506790249€	0.441	0.194	1.31522683126592e-15	CD44-AS1
Successfu	191	8.07592594730243e-20	0.56242853313572	0.409	0.173	2.70850404420629e-15	KLRG1
Successfu	191	9.90833792605607e-20	0.926672515668	0.699	0.462	3.32305837364068e-15	CAMK1D
Successfu	191	1.05515491213724e-19	0.92568973354037	0.846	0.622	3.53877854432589e-15	CNOT2
Successfu	191	1.4357058461087e-19	0.71394027909468€	0.785	0.547	4.81507026667936e-15	NEK7
Successfu	191	1.55003049703072e-19	0.71075936235866€	0.846	0.647	5.19849228094162e-15	NAMPT
Successfu	191	1.67646944751568e-19	0.77007050677833€	0.896	0.732	5.62254323307808e-15	NFAT5
Successfu	191	1.72277957510206e-19	1.12835927315069	0.516	0.292	5.77785813897729e-15	CYTH3
Successfu	191	2.20351661132394e-19	0.51782359389251€	0.609	0.333	7.39015401105822e-15	FAM118A
Successfu	191	2.23431073186512e-19	0.87701092999298	0.556	0.309	7.49343133252924e-15	HNRNPLL
Successfu	191	2.25784348875002e-19	0.38825398596903	0.358	0.139	7.57235549256981e-15	CD5
Successfu	191	2.28890526426959e-19	0.911230928099427	0.763	0.541	7.67653047530734e-15	BACH2
Successfu	191	2.69748930589846e-19	0.70715794578728€	0.738	0.494	9.04683963412225e-15	MECP2
Successfu	191	2.98472065419571e-19	0.53014307484794€	0.373	0.157	1.00101561300416e-14	PIK3CB
Successfu	191	3.08961293179712e-19	0.33578555438966€	0.262	0.086	1.03619438506612e-14	CD4
Successfu	191	3.54860786308907e-19	0.69105872437920	0.86	0.695	1.19013210512281e-14	ARL15
Successfu	191	3.79202351310248e-19	0.58452686954027€	0.871	0.696	1.27176884582431e-14	RFX3
Successfu	191	4.85929133116959e-19	0.53273128709594	0.703	0.415	1.62970912664766e-14	DOCK9
Successfu	191	8.09828674943532e-19	0.89515473605605€	0.724	0.464	2.71600341002562e-14	XYLT1
Successfu	191	8.82036905925655e-19	0.48909729074412	0.391	0.162	2.95817537509346e-14	CD40LG
Successfu	191	9.49269821995827e-19	0.55515383014588€	0.95	0.795	3.18366112900961e-14	CHST11
Successfu	191	9.97335563293786e-19	0.52773817281603	1	0.994	3.3448640121747e-14	AC016831.7
Successfu	191	1.11251139742057e-18	0.48067546023318	0.939	0.754	3.7311407246691e-14	VPS13B
Successfu	191	1.32263473403127e-18	0.78535381823984	0.921	0.822	4.43585237099406e-14	CAMK4
Successfu	191	1.72617175941376e-18	0.79518326253084€	0.86	0.657	5.78923484672185e-14	JARID2
Successfu	191	2.0054618303809e-18	0.52863695415014	0.939	0.794	6.72591788673146e-14	WIPF1
Successfu	191	2.02980544516404e-18	0.87030386900251	0.52	0.29	6.80756150199116e-14	LMNA
Successfu	191	2.84218145002196e-18	0.47678072943395	0.803	0.548	9.53210814708365e-14	CREBBP
Successfu	191	4.23742636160351e-18	0.58149394615776	0.763	0.511	1.42114805315459e-13	FNDC3B

Successfu	191	4.94727241781632e-18	0.53329936741299	0.993	0.949	1.65921622348724e-13	PARP8
Successfu	191	5.7918310876542e-18	0.921192138102352	0.283	0.107	1.94246431017746e-13	PLCL1
Successfu	191	9.13835555825134e-18	0.598927953939354	0.659	0.396	3.06482168712633e-13	KAT2B
Successfu	191	9.27269264940137e-18	0.270253390440687	0.265	0.09	3.10987566075623e-13	MCTP1
Successfu	191	1.02468827412198e-17	0.399884556251317	1	0.991	3.43659953375029e-13	PTPRC
Successfu	191	1.2206277078946e-17	0.506672294028959	0.935	0.78	4.09374120673691e-13	USP15
Successfu	191	1.26166677001746e-17	0.523424964008979	0.871	0.65	4.23137801328457e-13	FRYL
Successfu	191	1.34870586520413e-17	0.646250123741734	0.749	0.5	4.52328973072162e-13	SREBF2
Successfu	191	1.37046938076921e-17	0.681564374092645	0.767	0.527	4.59628020922377e-13	SPIDR
Successfu	191	1.44635222475118e-17	0.467348424396016	0.978	0.868	4.85077609137051e-13	DOCK10
Successfu	191	1.52401557904967e-17	0.479127531852358	0.993	0.981	5.11124344901679e-13	FYN
Successfu	191	1.81255375433896e-17	0.681564731102105	0.914	0.723	6.07894278130202e-13	SLC2A3
Successfu	191	2.14898177415132e-17	0.529792094535406	0.781	0.492	7.2072550741487e-13	SGMS1
Successfu	191	2.76807151404346e-17	0.430769717852234	0.491	0.249	9.28355824379895e-13	GOLGA8A
Successfu	191	2.81915776146216e-17	0.525542043601507	0.849	0.611	9.45489130039178e-13	TMEM131L
Successfu	191	3.00496100269145e-17	0.367876247993409	0.272	0.095	1.00780382108266e-12	PTPN13
Successfu	191	3.17452633314785e-17	0.437439938013791	0.692	0.407	1.06467264161113e-12	SAMHD1
Successfu	191	3.54427968475615e-17	0.562817717841856	0.803	0.567	1.18868052067352e-12	ARL4C
Successfu	191	4.46361563024336e-17	0.856873788225023	0.703	0.519	1.49700741007102e-12	AHNAK
Successfu	191	5.074644818174e-17	0.622396693659662	0.484	0.246	1.7019343791192e-12	SLC39A8
Successfu	191	5.58425397251715e-17	0.491528502831775	0.896	0.686	1.8728470973028e-12	MYCBP2
Successfu	191	5.88413106323085e-17	0.569133260704999	0.247	0.085	1.97341987598636e-12	MSC-AS1
Successfu	191	6.54090032770636e-17	0.537629844814716	0.348	0.15	2.19368715190616e-12	SPART
Successfu	191	8.52378273481217e-17	0.657012075896509	0.663	0.414	2.85870625360131e-12	GPR183
Successfu	191	8.91851747044204e-17	0.558943567384507	0.907	0.732	2.99109238923685e-12	NEDD9
Successfu	191	1.43051653543553e-16	0.663323004281335	0.953	0.798	4.79766635654369e-12	BRAF
Successfu	191	1.46704961463609e-16	0.48446516139222	0.717	0.458	4.92019099756652e-12	NFATC3
Successfu	191	1.56450708782431e-16	0.377015844224689	0.376	0.166	5.24704387114518e-12	ATP11A
Successfu	191	1.5925960461408e-16	0.854242063799982	0.427	0.215	5.34124861954702e-12	AGPAT4
Successfu	191	1.60110853526208e-16	0.628507424226476	0.703	0.474	5.36979780556196e-12	PHTF2
Successfu	191	1.63306230501256e-16	0.514563171472929	0.738	0.476	5.47696435855113e-12	USP36

Successfu	191	1.98645813145490e-16	0.623574212417782	0.423	0.201	6.66218328127343e-12	FRMD4B
Successfu	191	2.10868149060676e-16	0.595547836761696	0.258	0.093	7.07209598319695e-12	RAP1GAP2
Successfu	191	2.307915942981e-16	0.51266797575414	0.595	0.348	7.74028848956968e-12	SLC25A26
Successfu	191	2.38535970849814e-16	0.433671895565589	0.434	0.204	8.00001939036107e-12	LINC00861
Successfu	191	2.45560691232962e-16	0.523114475967927	0.724	0.459	8.23561446257106e-12	SENP7
Successfu	191	2.72317454870408e-16	0.294545396246594	0.287	0.108	9.13298280144375e-12	ITGB2
Successfu	191	2.76292773871628e-16	0.452282938964689	0.925	0.823	9.26630705010668e-12	XIST
Successfu	191	3.47257649682959e-16	0.524964176762334	0.892	0.691	1.16463270550671e-11	PPP1R16B
Successfu	191	3.47895371917894e-16	0.477449293165824	0.9	0.704	1.16677149833823e-11	AAK1
Successfu	191	4.08708819623981e-16	0.693386504729026	0.885	0.734	1.37072763925491e-11	SIPA1L1
Successfu	191	4.99371673125831e-16	0.802325401774453	0.43	0.218	1.67479271732941e-11	CCR6
Successfu	191	5.05367868998622e-16	0.461109820700563	0.552	0.305	1.69490275904758e-11	SORL1
Successfu	191	5.35688960293246e-16	0.552516028801597	0.889	0.751	1.79659363503149e-11	EXOC4
Successfu	191	6.49357504651217e-16	0.448561818982301	0.857	0.626	2.17781519909925e-11	XRN1
Successfu	191	6.67695391545665e-16	0.287570180068494	0.315	0.126	2.23931680416585e-11	TAF5L
Successfu	191	7.04255541687528e-16	0.581051644058088	0.903	0.806	2.36193223571163e-11	FOXN3
Successfu	191	7.20293420658524e-16	0.513535427298477	0.918	0.763	2.41572007420456e-11	DYRK1A
Successfu	191	8.18245949216681e-16	0.733909027581729	0.688	0.47	2.7442332644829e-11	JAZF1
Successfu	191	9.64349903290555e-16	0.445998277156133	0.272	0.102	3.23423670565586e-11	IL23R
Successfu	191	1.02605170908159e-15	0.607248214864348	0.753	0.514	3.44117222191782e-11	DLEU2
Successfu	191	1.16492597387918e-15	0.406476895098797	0.946	0.76	3.906928731196e-11	HNRNPH1
Successfu	191	1.30805908115176e-15	0.541596502148728	0.857	0.646	4.38696854636679e-11	ZEB1
Successfu	191	1.44547826872816e-15	0.434853292525558	0.441	0.216	4.84784501766051e-11	FAM117A
Successfu	191	1.52363871489282e-15	0.584916965563783	0.742	0.505	5.10997952200754e-11	PSME4
Successfu	191	1.58601322597731e-15	0.436947259274607	0.864	0.636	5.31917115728269e-11	ATRX
Successfu	191	1.91725269760687e-15	0.516335820422318	0.703	0.449	6.43008209723392e-11	MBNL2
Successfu	191	1.96714392103222e-15	0.50011385369453	0.312	0.13	6.59740728235788e-11	ARHGAP31
Successfu	191	2.49054099975316e-15	0.390576811371291	0.384	0.177	8.35277640497216e-11	SYNJ2
Successfu	191	2.68016557300777e-15	0.558254952584353	0.824	0.617	8.98873929875346e-11	ZHX2
Successfu	191	2.77260411269711e-15	0.431085942012687	0.201	0.065	9.29875967316356e-11	AC006369.1
Successfu	191	2.94197977335362e-15	0.409160820101971	0.706	0.443	9.86681176387336e-11	FLI1

Successfu	191	2.94544475629786e-15	0.49909843920759З	0.95	0.855	9.87843262367177e-11	REL
Successfu	191	3.22180063002199e-15	0.42593082222889	0.713	0.459	1.08052749529677e-10	ATF6
Successfu	191	3.35663852857831e-15	0.44044704604745!	0.964	0.89	1.12574942971459e-10	SMCHD1
Successfu	191	3.77132239291361e-15	0.51083803289196‹	0.914	0.761	1.26482610413537e-10	SH3KBP1
Successfu	191	4.15994624337861e-15	0.68406204887451	0.158	0.043	1.39516277110432e-10	FAM19A1
Successfu	191	4.34929120089572e-15	0.54891669071209	0.921	0.768	1.45866528295641e-10	ELOVL5
Successfu	191	5.37212961234736e-15	0.71967192174822!	0.649	0.409	1.80170482938906e-10	LYST
Successfu	191	5.47961746692953e-15	0.41618022364023	0.821	0.571	1.83775410605883e-10	SETX
Successfu	191	5.59882425959317e-15	0.39697193777680	0.935	0.77	1.87773368018236e-10	RNF213
Successfu	191	5.78368120129887e-15	1.31313509725787	0.208	0.071	1.93973100129161e-10	FAAH2
Successfu	191	5.82934994786141e-15	0.78381301425516	0.649	0.455	1.95504738551376e-10	RGCC
Successfu	191	5.90912044327696e-15	0.57886586987713	0.789	0.615	1.98180081426623e-10	NSD3
Successfu	191	6.17130670988294e-15	0.58915560168676!	0.839	0.649	2.06973284436054e-10	ZNF331
Successfu	191	6.50704172001719e-15	0.52134474239050!	0.616	0.393	2.18233165205937e-10	LTB
Successfu	191	6.69149147570124e-15	0.46692548599723‹	0.885	0.71	2.24419241112068e-10	FAF1
Successfu	191	7.04153111066192e-15	0.51214964468647	0.606	0.374	2.3615887038938e-10	MAF
Successfu	191	7.18358274726425e-15	0.47284159914146	0.642	0.413	2.40922998177748e-10	WDR7
Successfu	191	7.90946884369886e-15	0.42759656923036	0.194	0.062	2.65267766079972e-10	LINC00299
Successfu	191	8.21374048409801e-15	0.44688304533499!	0.398	0.194	2.75472428355679e-10	AC079793.1
Successfu	191	1.02099391028443e-14	0.37047205310961	0.315	0.135	3.42420937631191e-10	AL035634.1
Successfu	191	1.04977035259423e-14	0.51501658710778!	0.921	0.814	3.52071980853054e-10	PPP1CB
Successfu	191	1.09296548404852e-14	0.53735793191167	0.9	0.801	3.66558764040192e-10	SLC38A1
Successfu	191	1.09334158134247e-14	0.40760576115632!	0.545	0.304	3.66684899550637e-10	IFNGR1
Successfu	191	1.29061900345905e-14	0.51348771702872	0.839	0.636	4.32847801380096e-10	UBR5
Successfu	191	1.54229314726248e-14	0.49365906349939	0.638	0.413	5.17254275728889e-10	OXSR1
Successfu	191	1.73749287702705e-14	0.56249203885354‹	0.835	0.64	5.82720361097331e-10	TC2N
Successfu	191	1.8330481235029e-14	0.67809919820115	0.767	0.594	6.14767679660402e-10	SFMBT2
Successfu	191	1.98357491831803e-14	0.4845651482617	0.67	0.443	6.65251356105501e-10	FARS2
Successfu	191	2.13448650557977e-14	0.44824637437196!	0.946	0.762	7.15864084241343e-10	WNK1
Successfu	191	2.16471471573409e-14	0.39832546583817	0.72	0.492	7.26002021362898e-10	ATAD2B
Successfu	191	3.02673524067014e-14	0.56165233687544	0.907	0.805	1.01510646501595e-09	CREM

Successfu	191	3.05178200806046e-14	0.44697688029045	0.756	0.52	1.02350664986332e-09	AGO2
Successfu	191	3.09637718467353e-14	0.39306627765421	0.771	0.515	1.03846298019581e-09	HBP1
Successfu	191	3.43522539008311e-14	0.313643053565144	0.204	0.068	1.15210589132607e-09	CASS4
Successfu	191	3.85928635666154e-14	0.469449147466122	0.86	0.703	1.29432745829715e-09	UBAC2
Successfu	191	3.86902436234671e-14	0.484649543168046	0.799	0.591	1.29759339064384e-09	FNDC3A
Successfu	191	4.25324620273255e-14	0.510742844060458	0.971	0.893	1.42645371147244e-09	PRKCH
Successfu	191	4.53209297548357e-14	0.488827627615317	0.387	0.196	1.51997334211768e-09	AC010275.1
Successfu	191	5.10382392713876e-14	0.439061123776864	0.742	0.515	1.7117204686838e-09	PPP1R15B
Successfu	191	5.26519599880154e-14	0.436121413519486	0.706	0.459	1.76584143407806e-09	DOCK11
Successfu	191	5.32054224520837e-14	0.36128361871578	0.95	0.734	1.78440345819798e-09	SRRM2
Successfu	191	5.39407760643241e-14	0.387738027530989	0.538	0.296	1.8090657476453e-09	ANKRD36C
Successfu	191	5.61716917636221e-14	0.388652196135953	0.839	0.611	1.88388619836836e-09	ARID2
Successfu	191	6.06690925066171e-14	0.281871862984149	0.204	0.07	2.03472002448692e-09	CD101
Successfu	191	6.23892022475675e-14	0.473117534241957	0.978	0.933	2.09240906497892e-09	DENND4A
Successfu	191	6.25051422154756e-14	0.444985616066163	0.48	0.266	2.09629745962262e-09	KCTD20
Successfu	191	6.50013349470598e-14	0.421142565522549	0.928	0.787	2.18001477145449e-09	TRA2A
Successfu	191	6.67559956070441e-14	0.510139921585945	0.738	0.515	2.23886258066905e-09	INPP5D
Successfu	191	7.04603089858528e-14	0.552425728259583	0.244	0.095	2.36309784276753e-09	RFX2
Successfu	191	8.18603661817647e-14	0.715791858300876	0.742	0.542	2.74543296100402e-09	AUTS2
Successfu	191	8.23584956086386e-14	0.416142932234196	0.821	0.601	2.76213922572252e-09	CLASP1
Successfu	191	9.08218111702078e-14	0.402918400503994	0.832	0.625	3.04598190302643e-09	TRIP12
Successfu	191	9.88671519910204e-14	0.650008182869354	0.624	0.407	3.31580654347484e-09	SNX25
Successfu	191	1.03026345642218e-13	0.406862847591599	0.645	0.391	3.4552975801487e-09	AC010894.3
Successfu	191	1.05515620196218e-13	0.469896963217905	0.749	0.528	3.53878287014076e-09	RCOR1
Successfu	191	1.18389743792442e-13	0.453071724944475	0.892	0.753	3.97055522731091e-09	FBXW7
Successfu	191	1.28405042447629e-13	0.523640541129848	0.756	0.559	4.30644831360857e-09	PTPRJ
Successfu	191	1.29436187289701e-13	0.385739017501262	0.975	0.931	4.34103084932201e-09	ZBTB20
Successfu	191	1.50817385212099e-13	0.689193034539799	0.867	0.79	5.05811346524336e-09	NFKB1
Successfu	191	1.55506929494777e-13	0.387387243144647	0.996	0.959	5.21539140139584e-09	CDC42SE2
Successfu	191	1.6850708866888e-13	0.618732170357336	0.771	0.572	5.65139073977691e-09	GPRIN3
Successfu	191	1.9892649392453e-13	0.425614710952236	0.559	0.325	6.67159675324088e-09	TNIP1

Successfu	191	2.15687294841507e-13	0.439870548178015	0.566	0.34	7.23372049439447e-09	NR1D2
Successfu	191	2.18439528794787e-13	0.590868876837371	0.642	0.425	7.32602491671955e-09	AC058822.1
Successfu	191	2.32490287759083e-13	0.724698519461236	0.742	0.594	7.79725927086413e-09	BICDL1
Successfu	191	2.36414891331886e-13	0.289809506275725	0.33	0.149	7.9288826254888e-09	RAB29
Successfu	191	2.61294470904826e-13	0.637574386907399	0.523	0.303	8.76329396520606e-09	TP53INP1
Successfu	191	2.65831595025906e-13	0.478867993368694	0.964	0.834	8.91546003397885e-09	RAB8B
Successfu	191	2.67896242455268e-13	0.503178152740846	0.792	0.599	8.98470417946478e-09	ITPR2
Successfu	191	2.75556467968904e-13	0.500812228750908	0.792	0.605	9.24161282274109e-09	PDE4B
Successfu	191	2.77388723745591e-13	0.552338382380153	0.885	0.718	9.30306301697962e-09	IQGAP2
Successfu	191	3.06958300120852e-13	0.519731843206665	0.495	0.29	1.02947674694531e-08	CA5B
Successfu	191	3.09784139578043e-13	0.62075940870643	0.821	0.665	1.03895404731684e-08	FOXO1
Successfu	191	3.09996837045141e-13	0.304627425000953	0.523	0.29	1.03966739208199e-08	ANKRD36
Successfu	191	3.25930753207478e-13	0.535432911854869	0.262	0.108	1.09310656010724e-08	SLC41A1
Successfu	191	3.68008499249425e-13	0.451602368055751	0.925	0.783	1.23422690478272e-08	STAG1
Successfu	191	3.7498414970103e-13	0.464317806154179	0.814	0.603	1.25762184126731e-08	NFATC2
Successfu	191	3.81669844101227e-13	0.695988960434008	0.849	0.734	1.2800443231467e-08	MAN1A1
Successfu	191	4.29702991613601e-13	0.398480521280925	0.814	0.566	1.4411378932737e-08	INPP4A
Successfu	191	4.36334283869229e-13	0.502997447253283	0.581	0.362	1.46337792124062e-08	SPTBN1
Successfu	191	5.31436397212827e-13	0.389383687672006	0.606	0.371	1.78233138897238e-08	SEL1L3
Successfu	191	5.55261855400112e-13	0.280080353717079	0.43	0.214	1.8622372106409e-08	IL4R
Successfu	191	6.45510605890306e-13	0.359120477981944	0.466	0.261	2.16491347003491e-08	CERK
Successfu	191	6.46135851940899e-13	0.427488218184714	0.86	0.675	2.16701042023939e-08	STAG2
Successfu	191	6.54430437752672e-13	0.356826111748923	0.28	0.12	2.19482880213491e-08	C11orf65
Successfu	191	7.3748852377656e-13	0.457295448675918	0.978	0.894	2.47338901104183e-08	DDX3X
Successfu	191	7.7985415237203e-13	0.566829899320695	0.602	0.41	2.61547485622531e-08	RANBP9
Successfu	191	8.60827020893645e-13	0.687787332457027	0.409	0.216	2.88704166267311e-08	WDFY2
Successfu	191	8.73866158248339e-13	0.433987736769403	0.434	0.228	2.93077232153328e-08	FAM196B
Successfu	191	1.25529864680663e-12	0.306367689218508	0.341	0.164	4.21002060166009e-08	MIATNB
Successfu	191	1.33569623064168e-12	0.44010069136111	0.563	0.346	4.47965801832605e-08	STK38
Successfu	191	1.41684733353391e-12	0.427888949316494	0.645	0.417	4.75182258720602e-08	SPOCK2
Successfu	191	1.49883168761526e-12	0.749164820696698	0.588	0.386	5.02678171392406e-08	ADAM19

Successfu	191	1.516167691769e-12	0.38483325447183	0.821	0.611	5.08492320465486e-08	OGA
Successfu	191	1.67646092489856e-12	0.45241182089747	0.849	0.654	5.62251464992479e-08	HELZ
Successfu	191	1.70586453612346e-12	0.55803392417034	0.437	0.245	5.72112848125084e-08	ZC3H12D
Successfu	191	1.78639052190877e-12	0.46737900530520	0.839	0.683	5.99119653237765e-08	TRAF3
Successfu	191	1.81851448707833e-12	0.36818831897384	0.642	0.429	6.09893388676332e-08	SEPT2
Successfu	191	1.85752635055488e-12	0.45544783797592	0.785	0.593	6.22977187449096e-08	USP47
Successfu	191	1.96652265375036e-12	0.44205492948114	0.864	0.689	6.59532367614796e-08	PLCL2
Successfu	191	1.9878075639223e-12	0.27876333025303	0.516	0.285	6.6667090078826e-08	MPRIP
Successfu	191	2.07543797003076e-12	0.34059370801547	0.301	0.135	6.96060386388915e-08	SH3BP5
Successfu	191	2.18955058467596e-12	0.43179105425570	0.122	0.032	7.34331475088625e-08	EPHA4
Successfu	191	2.53235723946714e-12	0.39200479747228	0.921	0.739	8.49301970972488e-08	FTX
Successfu	191	2.56198165391527e-12	0.38995894700727	0.943	0.762	8.59237407090104e-08	NIPBL
Successfu	191	2.59635137585396e-12	0.79139217724571	0.928	0.865	8.70764324433903e-08	TANK
Successfu	191	2.75035185983866e-12	0.57055035291432	0.803	0.651	9.22413006752691e-08	PRKCB
Successfu	191	2.80774548002818e-12	0.46625327128573	0.742	0.546	9.41661679091852e-08	TENT5C
Successfu	191	2.91913453048989e-12	0.47820952997861	0.824	0.673	9.79019338835699e-08	TBL1XR1
Successfu	191	3.41956686876261e-12	0.36770405424146	0.613	0.382	1.1468543364456e-07	EXOC2
Successfu	191	3.72608049725677e-12	0.55270443441962	0.864	0.683	1.24965287716997e-07	CHD1
Successfu	191	3.78980680976669e-12	0.33663994853305	0.491	0.274	1.27102540785955e-07	ARHGEF6
Successfu	191	4.16773283693942e-12	0.42012027337565	0.38	0.198	1.39777423885274e-07	TESPA1
Successfu	191	4.2013170801133e-12	0.56926714842730	0.502	0.312	1.4090377223284e-07	FAM102B
Successfu	191	4.30358646700369e-12	0.52375554143941	0.767	0.581	1.4433368293037e-07	SRPK2
Successfu	191	4.49494269189591e-12	0.45196687697430	0.849	0.663	1.50751388000805e-07	RBMS1
Successfu	191	5.17900504451103e-12	0.38291934526979	0.434	0.236	1.73693471182811e-07	VAV1
Successfu	191	5.28371941120588e-12	0.40073693976580	0.72	0.507	1.77205381613023e-07	PHIP
Successfu	191	5.37202635288195e-12	0.58900799547493	0.907	0.773	1.80167019822955e-07	CDC14A
Successfu	191	5.43547757908613e-12	0.44301831023368	0.796	0.581	1.82295047047391e-07	ASAP1
Successfu	191	5.60038006282509e-12	0.41389496229633	0.968	0.903	1.87825546547028e-07	ARID4B
Successfu	191	5.71128745883866e-12	0.36240633654216	0.928	0.794	1.91545158794531e-07	HERC1
Successfu	191	6.10729315342082e-12	0.39620912485401	0.957	0.878	2.04826397779428e-07	CNOT6L
Successfu	191	6.16309903731001e-12	0.41534724682874	0.849	0.637	2.06698015513303e-07	SLFN12L

Successfu	191	6.4003832153258e-12	0.56355058228807	0.652	0.458	2.14656052275597e-07	SMAD3
Successfu	191	6.43937955355934e-12	0.36014586979266	0.219	0.085	2.15963911467273e-07	AL121935.1
Successfu	191	6.52858820865639e-12	0.25210180034461	0.419	0.216	2.18955791341918e-07	NCOR2
Successfu	191	6.55957819443724e-12	0.34213053385447	0.577	0.339	2.19995133485036e-07	ATF2
Successfu	191	6.59085710025299e-12	0.42464635066700	0.48	0.277	2.21044165428285e-07	AC124319.2
Successfu	191	8.11017008422815e-12	0.34989775597402	1	0.923	2.71998884284844e-07	JMJD1C
Successfu	191	8.49824756990546e-12	0.55903900062688	0.717	0.548	2.85014226999489e-07	PBX4
Successfu	191	9.02931617523595e-12	0.35270499930479	0.749	0.509	3.02825205885063e-07	CCDC88C
Successfu	191	9.16871087627105e-12	0.36726653089502	0.387	0.205	3.07500225368378e-07	NOTCH2
Successfu	191	9.32400332932721e-12	0.25452118850754	0.165	0.056	3.12708423658976e-07	B3GNTL1
Successfu	191	9.77040600995933e-12	0.41683659462023	0.681	0.456	3.27679876762016e-07	PTEN
Successfu	191	1.03463100223386e-11	0.37102911463736	0.606	0.375	3.4699454552919e-07	UBR3
Successfu	191	1.04078541593392e-11	0.38589066060439	0.581	0.383	3.49058612795917e-07	KBTBD2
Successfu	191	1.05100362727675e-11	0.38563594517906	0.366	0.189	3.52485596516078e-07	RNF19B
Successfu	191	1.13475198670771e-11	0.87600717269631	0.208	0.084	3.80573121302031e-07	AL136962.1
Successfu	191	1.1493558969544e-11	0.48456148027251	0.728	0.51	3.85470980720567e-07	CHIC2
Successfu	191	1.16451350267798e-11	0.88872134967252	0.376	0.217	3.90554538528141e-07	PCAT1
Successfu	191	1.18804951947224e-11	0.32608986809365	0.703	0.476	3.98448047840601e-07	RFTN1
Successfu	191	1.2316886789338e-11	0.42954855168530	0.706	0.494	4.13083749140817e-07	NUP153
Successfu	191	1.23344485300395e-11	0.38650303253911	0.326	0.162	4.13672734800464e-07	KIF5C
Successfu	191	1.28689924851429e-11	0.41591040756128	0.814	0.609	4.31600269966721e-07	PRKCQ
Successfu	191	1.29341212413237e-11	0.58951948867056	0.534	0.349	4.33784558191513e-07	SBF2
Successfu	191	1.37844964819563e-11	0.35017638492788	0.319	0.151	4.6230444301185e-07	PIK3CG
Successfu	191	1.40841815284872e-11	0.29540528285976	0.527	0.303	4.72355280102405e-07	TRRAP
Successfu	191	1.43322390137436e-11	0.41236376669654	0.91	0.736	4.80674632042934e-07	VPS13C
Successfu	191	1.53013298720159e-11	0.37155855379016	0.685	0.467	5.13176001247671e-07	UBR4
Successfu	191	1.55074990639473e-11	0.50458432140783	0.656	0.445	5.20090503606666e-07	RBPJ
Successfu	191	1.5839112817049e-11	0.39804268565554	0.677	0.451	5.31212165658191e-07	SLC16A7
Successfu	191	1.63881710359606e-11	0.32238052856951	0.165	0.056	5.49626480204045e-07	C1orf141
Successfu	191	1.68072507358224e-11	0.34509272748307	0.606	0.376	5.63681575178011e-07	PBXIP1
Successfu	191	1.70065887009901e-11	0.36650463158873	0.616	0.397	5.70366971853806e-07	ADD3

Successfu	191	1.78122409167936e-11	0.417617298939785	0.785	0.566	5.97386935867422e-07	ITSN2
Successfu	191	1.86674560835558e-11	0.351282414141557	0.323	0.159	6.26069142130296e-07	STX11
Successfu	191	1.91151043930926e-11	0.379993516242664	0.914	0.767	6.41082371135541e-07	ANKRD11
Successfu	191	1.97116715990629e-11	0.647500201476094	0.505	0.318	6.6109004208937e-07	PAM
Successfu	191	2.18889424433782e-11	0.42870549788982	0.832	0.625	7.34111351666019e-07	NFE2L2
Successfu	191	2.20216944916278e-11	0.458862636665685	0.14	0.043	7.38563589860212e-07	SEMA4A
Successfu	191	2.25234892314371e-11	0.370040452108528	0.76	0.585	7.55392781843938e-07	CMIP
Successfu	191	2.43612787609501e-11	0.354578795847339	0.982	0.91	8.17028567084745e-07	FNBP1
Successfu	191	2.54824420488518e-11	0.437326601124006	0.703	0.524	8.54630141434392e-07	RNGTT
Successfu	191	2.9335044107432e-11	0.420475287060441	0.663	0.447	9.83838709275053e-07	CD6
Successfu	191	2.9833912628969e-11	0.408296711299378	0.581	0.363	1.00056976175036e-06	LUZP1
Successfu	191	3.01051641839861e-11	0.309016767356848	0.384	0.2	1.00966699640253e-06	TBC1D31
Successfu	191	3.16280567951226e-11	0.299638343500911	0.477	0.265	1.06074176879482e-06	EP400
Successfu	191	3.30992783567038e-11	0.29538504144537	0.595	0.38	1.11008359752713e-06	SAFB2
Successfu	191	3.48023071064475e-11	0.419533382652332	0.864	0.729	1.16719977573604e-06	TBC1D5
Successfu	191	3.52065132567894e-11	0.476924674235259	0.749	0.561	1.1807560416062e-06	STIM1
Successfu	191	3.56727464689335e-11	0.331506828479345	0.663	0.437	1.19639257107509e-06	YTHDF3
Successfu	191	3.58976537168284e-11	0.411571224392172	0.373	0.197	1.20393551035499e-06	NFATC1
Successfu	191	3.66449157286601e-11	0.616811131146076	0.803	0.6	1.2289971837078e-06	ITGA4
Successfu	191	3.8525340045218e-11	0.453673949726457	0.38	0.204	1.29206285443652e-06	ARHGAP10
Successfu	191	3.99010227058549e-11	0.587966896257941	0.835	0.716	1.33820049950896e-06	ARHGAP26
Successfu	191	4.01752482963875e-11	0.504397236152329	0.882	0.751	1.34739747736424e-06	OSBPL8
Successfu	191	4.08434657791414e-11	0.304384809022433	0.957	0.846	1.36980815530085e-06	TNRC6B
Successfu	191	4.17411301906524e-11	0.366711477455276	0.538	0.325	1.3999140243341e-06	RPRD2
Successfu	191	4.17481706587607e-11	0.400664219732166	0.774	0.56	1.40015014755352e-06	ARFGEF1
Successfu	191	4.20308067926346e-11	0.395169031779026	0.344	0.175	1.40962919821138e-06	AC009299.3
Successfu	191	4.28770374304803e-11	0.381383365731346	0.233	0.097	1.43801008134345e-06	GZMK
Successfu	191	4.36262346269529e-11	0.324742629150977	0.602	0.375	1.46313665691875e-06	LMBR1
Successfu	191	4.42671760042151e-11	0.343785149285438	0.756	0.542	1.48463254882937e-06	MON2
Successfu	191	4.69917532322267e-11	0.438997614078743	0.573	0.361	1.57600941990242e-06	GATA3
Successfu	191	4.71403544571552e-11	0.408463265279386	0.803	0.635	1.58099320778407e-06	NUP98

Successfu	191	4.9549156966776e-11	0.29602509017941	0.287	0.134	1.66177962635173e-06	RRM1
Successfu	191	4.97501561416944e-11	0.40662841596747	0.344	0.179	1.66852073668015e-06	AC096558.2
Successfu	191	5.01702094143359e-11	0.31274605703639	0.943	0.784	1.682608483338e-06	KMT2C
Successfu	191	5.18423468426108e-11	0.39893451402883	0.556	0.351	1.73868862840748e-06	RNF168
Successfu	191	5.50683873145096e-11	0.37910134135555	0.459	0.273	1.84688357375402e-06	SLC7A5
Successfu	191	5.51242091102255e-11	0.57160548756498	0.642	0.464	1.84875572513874e-06	GLCCI1
Successfu	191	5.98525408026328e-11	0.66843882499158	0.581	0.405	2.0073345134387e-06	ANTXR2
Successfu	191	6.23949933280832e-11	0.40317023745795	0.803	0.62	2.09260328623725e-06	UBE2H
Successfu	191	6.35906389792257e-11	0.37646534457133	0.907	0.742	2.13270285008527e-06	LRRFIP1
Successfu	191	6.88623723114964e-11	0.49858431842056	0.728	0.548	2.30950624258297e-06	DUSP16
Successfu	191	7.32113891923102e-11	0.34842309648533	0.319	0.155	2.4553635707317e-06	AL589990.1
Successfu	191	7.64545373868428e-11	0.39915213532372	0.803	0.623	2.56413227487993e-06	RAPGEF1
Successfu	191	7.64696810585586e-11	0.36235920990250	0.659	0.442	2.56464016334194e-06	FUT8
Successfu	191	7.78187694298263e-11	0.61911309493546	0.846	0.74	2.60988588913752e-06	LINC01934
Successfu	191	8.54611315003261e-11	0.37148693624521	0.975	0.937	2.86619542825794e-06	PABPC1
Successfu	191	9.4531310494776e-11	0.271113763354125	0.419	0.226	3.1703910913738e-06	LINS1
Successfu	191	1.00295919298758e-10	0.35841912371705	0.857	0.732	3.36372454144173e-06	BCLAF1
Successfu	191	1.00324923253425e-10	0.29501524605989	0.384	0.204	3.36469727607336e-06	SAMD9L
Successfu	191	1.07064839439744e-10	0.45068554507043	0.993	0.946	3.59074058513015e-06	CELF2
Successfu	191	1.08703954051919e-10	0.43806603487267	0.634	0.403	3.64571321099326e-06	PTBP2
Successfu	191	1.1217768280071e-10	0.55383626802155	0.767	0.561	3.7622151257702e-06	SMC5
Successfu	191	1.13775286196653e-10	0.47469461856041	0.495	0.309	3.81579554846334e-06	PIK3R5
Successfu	191	1.14006498352329e-10	0.38044826277041	0.86	0.683	3.82354994174042e-06	PUM2
Successfu	191	1.15169453435323e-10	0.38987592692398	0.703	0.494	3.86255312931388e-06	RASGRP1
Successfu	191	1.17675075589914e-10	0.34147295692237	0.742	0.516	3.94658668513453e-06	CDC73
Successfu	191	1.34251290002552e-10	0.34658297333006	0.262	0.12	4.50251976410559e-06	IL4I1
Successfu	191	1.40693407693927e-10	0.34689474925584	0.513	0.317	4.71857550723894e-06	ANO6
Successfu	191	1.41516834040364e-10	0.45487465920138	0.667	0.46	4.74619158004573e-06	AC087286.2
Successfu	191	1.45339376540272e-10	0.42123657084138	0.796	0.615	4.87439201040765e-06	USP25
Successfu	191	1.47052526783999e-10	0.39822379353707	0.792	0.57	4.93184764328175e-06	ABI1
Successfu	191	1.49833277775263e-10	0.35181432009096	0.692	0.479	5.02510847002677e-06	RBL2

Successfu	191	1.62552868214016e-10	0.408061777788197	0.824	0.61	5.45169809416167e-06	LPXN
Successfu	191	1.68499774325792e-10	0.269583280125056	0.548	0.324	5.6511454313384e-06	SFSWAP
Successfu	191	1.72533528513485e-10	0.41889495658917	0.896	0.783	5.78642947928526e-06	PPP3CA
Successfu	191	1.82923900132817e-10	0.368726132227911	0.853	0.709	6.13490176265441e-06	GNB1
Successfu	191	1.89212857789357e-10	0.2804747572428	0.484	0.286	6.34582082453946e-06	FAM193A
Successfu	191	1.9009194503718e-10	0.354638540438623	0.534	0.331	6.37530365265694e-06	SLC30A7
Successfu	191	1.98465454585378e-10	0.499790437738544	0.534	0.332	6.6561344158844e-06	USP37
Successfu	191	1.99976304024506e-10	0.299693299977464	0.602	0.384	6.70680528437389e-06	SENP5
Successfu	191	2.04395161802478e-10	0.348008299032604	0.394	0.223	6.85500493653151e-06	AC009554.1
Successfu	191	2.08413448766415e-10	0.354878382089338	0.781	0.598	6.98977024472802e-06	STK10
Successfu	191	2.2438682350007e-10	0.349591997697989	0.638	0.438	7.52548528654534e-06	EFR3A
Successfu	191	2.45714495253627e-10	0.301212330382077	0.649	0.426	8.24077274181615e-06	DYNC1H1
Successfu	191	2.53626421191492e-10	0.519715400056148	0.556	0.372	8.50612291392027e-06	EZH2
Successfu	191	2.55833389132887e-10	1.10545737778904	0.484	0.322	8.58014020473877e-06	CRADD
Successfu	191	2.56678439036174e-10	0.391387156632087	0.771	0.571	8.6084814883952e-06	DYM
Successfu	191	2.8690797060225e-10	0.330396135284892	0.91	0.757	9.62231951805827e-06	SFPQ
Successfu	191	2.93750432089283e-10	0.421436685699055	0.659	0.457	9.85180199141038e-06	N4BP1
Successfu	191	2.99583565932822e-10	0.388701151648716	0.154	0.055	1.0047433634255e-05	IL26
Successfu	191	3.02298596917538e-10	0.32873938520442	0.297	0.146	1.01384903434204e-05	TBXAS1
Successfu	191	3.10718034079219e-10	0.458942523663715	0.688	0.515	1.04208614269488e-05	ITPR1
Successfu	191	3.1283200671897e-10	0.311374762255485	0.677	0.45	1.04917598413408e-05	SNRK
Successfu	191	3.15060654228365e-10	0.407922936713683	0.735	0.541	1.05665042215109e-05	EPC2
Successfu	191	3.18761915058139e-10	0.440020185868708	0.703	0.516	1.06906371072199e-05	MLLT3
Successfu	191	3.30145266334744e-10	0.325003791608367	0.462	0.274	1.10724119423346e-05	TGFBR1
Successfu	191	3.35775773027262e-10	0.596235406386722	0.767	0.59	1.12612478757883e-05	AC009313.1
Successfu	191	3.82306963344828e-10	0.321176639436505	0.986	0.906	1.28218109366589e-05	PPP2R5C
Successfu	191	3.848962172321e-10	0.347163271537718	0.495	0.294	1.29086493335302e-05	AC001226.2
Successfu	191	3.96826462965817e-10	0.485440882549371	0.642	0.469	1.33087659149476e-05	STAT5B
Successfu	191	4.27211866859413e-10	0.388043512277934	0.563	0.368	1.4327831590731e-05	PHKB
Successfu	191	4.34501056083253e-10	0.392225957519466	0.982	0.958	1.45722964189201e-05	AKAP13
Successfu	191	4.55636193783292e-10	0.325310712128696	0.161	0.059	1.52811266671041e-05	CC2D2A

Successfu	191	4.61781664649493e-10	0.42528964579202	0.778	0.604	1.54872334690147e-05	DNAJC1
Successfu	191	5.43631508275468e-10	0.27274286775057	0.616	0.391	1.82323135245426e-05	LUC7L
Successfu	191	5.54892483647141e-10	0.29776344847423	0.706	0.471	1.86099841165578e-05	INO80D
Successfu	191	5.67798369852241e-10	0.35378661513522	0.728	0.514	1.90428217281044e-05	UVRAG
Successfu	191	5.80203920725427e-10	0.36848219909694	0.842	0.695	1.94588790932894e-05	ZNF644
Successfu	191	5.87301546683485e-10	0.34404627558721	0.441	0.26	1.96969192726707e-05	SKAP1-AS1
Successfu	191	5.9549885227618e-10	0.40273398388266	0.799	0.615	1.99718405076385e-05	CYLD
Successfu	191	6.03987811338107e-10	0.29941825940414	0.613	0.389	2.02565432166575e-05	UBA6
Successfu	191	6.05462521744786e-10	0.28362927480143	0.473	0.28	2.03060020542766e-05	TRIM38
Successfu	191	6.1466123674209e-10	0.25727632287950	0.477	0.288	2.06145085578562e-05	ATP11C
Successfu	191	6.34971639666882e-10	0.28423985681091	0.226	0.101	2.12956788511479e-05	PRKX-AS1
Successfu	191	6.39654107967956e-10	0.26168409812814	0.308	0.155	2.14527194730293e-05	APOL3
Successfu	191	6.62059013626555e-10	0.41920922263642	0.606	0.397	2.22041351990074e-05	RFX7
Successfu	191	6.69602592119556e-10	0.37426927845248	0.609	0.383	2.24571317345057e-05	ERO1B
Successfu	191	6.75595468757613e-10	0.31486184793068	0.401	0.223	2.26581208311928e-05	HPCAL1
Successfu	191	7.23640304233938e-10	0.31314167602577	0.749	0.544	2.42694485233978e-05	EIF3A
Successfu	191	7.33890598056776e-10	0.35202163990312	0.878	0.713	2.46132228776282e-05	PAN3
Successfu	191	7.35733766425109e-10	0.29739198709988	0.885	0.734	2.46750390583653e-05	ASH1L
Successfu	191	7.45944738006758e-10	0.37505936870898	0.778	0.582	2.50174946232706e-05	POLR2A
Successfu	191	7.47069401084804e-10	0.40637510023043	0.857	0.675	2.50552135735822e-05	RNF149
Successfu	191	8.112804337159e-10	0.33208349003563	0.491	0.298	2.72087231859638e-05	STK26
Successfu	191	9.53123947258564e-10	0.35269481969375	0.656	0.449	3.19658709431577e-05	NBAS
Successfu	191	1.09643629513573e-09	0.35646838560331	0.609	0.411	3.6772280466262e-05	B4GALT1
Successfu	191	1.1391359014717e-09	0.28196217990996	0.946	0.775	3.82043398635579e-05	BIRC6
Successfu	191	1.14088785832351e-09	0.48407772142399	0.789	0.675	3.82630969924539e-05	SMYD3
Successfu	191	1.22096288199074e-09	0.32606665719860	0.563	0.362	4.09486531362056e-05	PER1
Successfu	191	1.2526421530408e-09	0.32463024243130	0.419	0.245	4.20111125286823e-05	PREP
Successfu	191	1.28062149869711e-09	0.40872219792509	0.918	0.834	4.29494838233038e-05	ESYT2
Successfu	191	1.36245684348855e-09	0.32568173478666	0.814	0.608	4.56940776169189e-05	MARCH6
Successfu	191	1.37549277517287e-09	0.37422328615011	0.613	0.418	4.61312766937477e-05	NRF1
Successfu	191	1.40052467619972e-09	0.32724968616667	0.47	0.277	4.69707965903863e-05	NPAT

Successfu	191	1.40134417123643e-09	0.48835112362731З	0.566	0.391	4.69982808149275e-05	SPATA5
Successfu	191	1.43403351903208e-09	0.326280311023011	0.581	0.383	4.80946161612979e-05	USP7
Successfu	191	1.48350445212527e-09	0.67355382632870ὲ	0.28	0.144	4.97537723153772e-05	AFF3
Successfu	191	1.48701544406818e-09	0.35309998668551‌2	0.971	0.901	4.98715239631585e-05	RAB11A
Successfu	191	1.49717118668237e-09	0.46539166855470‌9	0.978	0.936	5.02121272589532e-05	CBLB
Successfu	191	1.5380225175458e-09	0.34964597342180‌3	0.738	0.526	5.15821991934511e-05	MSI2
Successfu	191	1.58460628128847e-09	0.28685051523565‌1	0.663	0.449	5.31445254618529e-05	PRKAR2A
Successfu	191	1.79659614951934e-09	0.26785366670648	0.297	0.15	6.02542416625795e-05	RASSF2
Successfu	191	1.79972046903472e-09	0.33280051466471‌6	0.495	0.304	6.03590250904865e-05	SPAG1
Successfu	191	1.80786394388076e-09	0.28792239552929‌2	0.305	0.155	6.0632140949873e-05	MTMR12
Successfu	191	1.94808024860899e-09	0.34705223323262‌9	0.903	0.747	6.53347153778482e-05	USP34
Successfu	191	2.00765167738843e-09	0.38777173066278‌7	0.885	0.726	6.73326219562531e-05	WAC
Successfu	191	2.01717919239058e-09	0.37058039587480‌7	0.914	0.769	6.76521557543954e-05	MED13L
Successfu	191	2.07819936306599e-09	0.37483849782259‌5	0.76	0.59	6.96986502385072e-05	TRAPPC9
Successfu	191	2.17402738729326e-09	0.42428477754246‌3	0.803	0.653	7.29125305150415e-05	CCSER2
Successfu	191	2.3146715002937e-09	0.27208523135694‌3	0.398	0.226	7.76294527768501e-05	IKBKB
Successfu	191	2.55247853486338e-09	0.36587261469167‌8	0.9	0.698	8.56050251022481e-05	USP9X
Successfu	191	2.84719816076456e-09	0.40793241124392‌8	0.939	0.884	9.54893319157217e-05	THEMIS
Successfu	191	2.85109788047701e-09	0.29022520365077‌4	0.832	0.645	9.5620120715438e-05	KMT2A
Successfu	191	2.92689042379105e-09	0.40857024707107‌4	0.724	0.553	9.81620510331042e-05	CCNY
Successfu	191	2.94367747161565e-09	0.34620331087315‌4	0.781	0.593	9.87250550430456e-05	AP3B1
Successfu	191	3.03873708141326e-09	0.30512301127809‌6	0.993	0.961	0.000101913164236438	HNRNPC
Successfu	191	3.08002186211517e-09	0.48026281863084‌1	0.81	0.671	0.000103297773211618	MGAT4A
Successfu	191	3.13170426561688e-09	0.36853688653605‌8	0.814	0.644	0.000105031097660259	NCOA1
Successfu	191	3.22412087144044e-09	0.30269467066378‌6	0.412	0.242	0.000108130565786369	CLIP4
Successfu	191	3.32842918751064e-09	0.40863307391259‌8	0.946	0.853	0.000111628858090732	FOXP1
Successfu	191	3.3680456546765e-09	0.33156021729466‌6	0.462	0.271	0.00011295751516654	HDAC4
Successfu	191	3.38856089794561e-09	0.41488369820044‌4	0.835	0.694	0.0001136455553953	BTBD9
Successfu	191	3.42749725886748e-09	0.33068804639244‌2	0.9	0.753	0.000114951403067897	SRSF5
Successfu	191	3.4848952758537e-09	0.26721939820890‌9	0.211	0.094	0.000116876417761581	SMCO4
Successfu	191	3.52175892449944e-09	0.40457367513770‌8	0.867	0.77	0.000118112750809862	FAM172A

Successfu	191	3.5619709398364e-09	0.344209917639797	0.491	0.315	0.000119461381380233	BTRC
Successfu	191	3.56422757017648e-09	0.325603699542402	0.459	0.289	0.000119537064248579	WDR48
Successfu	191	3.69827568535534e-09	0.357077088239164	0.896	0.752	0.000124032769935447	NCOA2
Successfu	191	3.77208513144898e-09	0.251630183427102	0.423	0.239	0.000126508191138536	KAT7
Successfu	191	3.78155924841953e-09	0.448277328937696	0.573	0.404	0.000126825934073494	THADA
Successfu	191	4.24646294279683e-09	0.442370351551935	0.523	0.338	0.00014241787417552	AL139807.1
Successfu	191	4.2487157647801e-09	0.324844642022836	0.986	0.945	0.000142493429319195	RABGAP1L
Successfu	191	4.38295721091975e-09	0.324889632689151	0.731	0.527	0.000146995618939827	RBM33
Successfu	191	4.55592362290612e-09	0.263212014410748	0.599	0.393	0.000152796566465025	SEC24B
Successfu	191	4.68181690018888e-09	0.328047411012466	0.918	0.754	0.000157018775198535	KDM4C
Successfu	191	4.7304319675821e-09	0.312950220864953	0.283	0.144	0.000158649227328768	SLC36A4
Successfu	191	4.98265821673172e-09	0.305696445408466	0.505	0.319	0.000167108391272749	NCKAP1L
Successfu	191	5.0584140713915e-09	0.287412529424715	0.545	0.352	0.000169649091126328	RPS6KB1
Successfu	191	5.0717102199217e-09	0.390471597002916	0.645	0.446	0.000170095017355734	PHF21A
Successfu	191	5.20723607123648e-09	0.313485404602813	0.498	0.303	0.000174640283357129	TBC1D23
Successfu	191	5.29564082806164e-09	0.435227677369247	0.516	0.333	0.000177605202091531	RUNX2
Successfu	191	5.34450114965989e-09	0.315869341277797	0.513	0.32	0.000179243879557293	PAXBP1
Successfu	191	5.66783062823211e-09	0.45946260451949	0.47	0.301	0.000190087703609649	SLC9A9
Successfu	191	5.80346033731428e-09	0.305509811580099	0.724	0.521	0.000194636452792846	MARCH7
Successfu	191	6.07098262595288e-09	0.275680333396557	0.405	0.242	0.000203608615309208	DBF4
Successfu	191	6.09462797895916e-09	0.56263634455974	0.305	0.162	0.000204401633158332	PTPRM
Successfu	191	6.179758461326e-09	0.329238371240682	0.412	0.246	0.000207256739275952	USP32
Successfu	191	6.42308697026363e-09	0.540588858262262	0.864	0.793	0.000215417490808702	PDE4D
Successfu	191	6.44395285532901e-09	0.483480731796385	0.738	0.559	0.000216117290862024	FBXO34
Successfu	191	6.46860718393562e-09	0.26931539745731	0.455	0.27	0.000216944147734833	FGD3
Successfu	191	6.78896947686457e-09	0.283945834408099	0.563	0.36	0.000227688458315084	YLPM1
Successfu	191	6.85109346725079e-09	0.334123736598353	0.455	0.276	0.000229771972704657	CRTC3
Successfu	191	6.98201962137976e-09	0.408842623009641	0.846	0.675	0.000234162974061834	BAZ1A
Successfu	191	7.2917326179857e-09	0.351611809036477	0.326	0.174	0.000244550128542004	ZDBF2
Successfu	191	7.60126866136544e-09	0.370569625672966	0.749	0.563	0.000254931348364874	PPM1B
Successfu	191	7.62775151536975e-09	0.29545830023411	0.659	0.466	0.000255819530322471	GNA13

Successfu	191	7.91264489340296e-09	0.40284144038274	0.369	0.212	0.000265374284434949	KLF2
Successfu	191	8.00488695139797e-09	0.28131005604347	0.588	0.389	0.000268467898575985	TNFRSF1B
Successfu	191	8.24097802506181e-09	0.386390377289067	0.699	0.52	0.000276385921004523	ASXL1
Successfu	191	8.31257939426582e-09	0.398566177372519	0.297	0.156	0.000278787287724887	EED
Successfu	191	8.44719717997159e-09	0.34201923626051	0.774	0.579	0.000283302099021887	KIAA1109
Successfu	191	8.4695983433988e-09	0.269635012619935	0.244	0.119	0.000284053389240909	PLEKHM3
Successfu	191	8.47279659301956e-09	0.372072046131257	0.323	0.176	0.00028416065213669	EVA1C
Successfu	191	8.90972497373583e-09	0.315459321916839	0.86	0.701	0.000298814356169152	PPP6R3
Successfu	191	9.0392158147615e-09	0.261271345824141	0.677	0.458	0.000303157219995471	AL031772.1
Successfu	191	9.1045997706202e-09	0.300388820943565	0.312	0.168	0.00030535006710706	KIF16B
Successfu	191	9.13661119173276e-09	0.324522021377745	0.792	0.606	0.000306423666148333	SND1
Successfu	191	9.55305442577600e-09	0.295244237731659	0.419	0.251	0.000320390339331675	KSR1
Successfu	191	9.81378341523889e-09	0.289012045172869	0.971	0.87	0.000329134668180282	RAP1B
Successfu	191	9.9211746988522e-09	0.311781888136421	0.358	0.203	0.000332736357050105	VCL
Successfu	191	9.93787328861874e-09	0.333744321711294	0.609	0.401	0.000333296394353695	COL4A3BP
Successfu	191	9.94586221393612e-09	0.726838702646334	0.774	0.674	0.00033356432693099	SNX9
Successfu	191	9.964757643217e-09	0.320394718483681	0.695	0.504	0.000334198041838212	TMEM131
Successfu	191	1.00334833283568e-08	0.32909682778056	0.62	0.414	0.00033650296386643	GALNT10
Successfu	191	1.01453260715506e-08	0.251260407778904	0.226	0.108	0.000340253945787663	MICAL3
Successfu	191	1.02512443560916e-08	0.350143673570149	0.81	0.668	0.000343806233214599	MKLN1
Successfu	191	1.10569574972673e-08	0.368508147375248	0.47	0.301	0.000370828240543352	MAP3K1
Successfu	191	1.12058750686463e-08	0.291836206929952	0.283	0.147	0.000375822638052258	HEG1
Successfu	191	1.14306706884239e-08	0.417631994059005	0.817	0.692	0.000383361833548359	ZCCHC7
Successfu	191	1.14745589828983e-08	0.308591238502391	0.735	0.537	0.000384833759168442	MARK3
Successfu	191	1.17117272647602e-08	0.25890168049699	0.767	0.542	0.000392787909005527	RSF1
Successfu	191	1.2677294407811e-08	0.307464035174869	0.778	0.539	0.000425171099849165	PPP4R3A
Successfu	191	1.283710814939e-08	0.349244838847541	0.631	0.455	0.000430530933114241	WDR70
Successfu	191	1.29466578080225e-08	0.370210946530155	0.631	0.444	0.000434205009565458	MICU2
Successfu	191	1.39604576082898e-08	0.383372933269758	0.581	0.403	0.000468205827266822	HDLBP
Successfu	191	1.41802249530643e-08	0.264353973369795	0.842	0.663	0.000475576384475871	CSDE1
Successfu	191	1.43887687233827e-08	0.45577065435598	0.717	0.551	0.000482570525444809	VPS37B

Successfu	191	1.46824204870476e-08	0.31446978741568\u0035	0.595	0.402	0.000492419018294601	USP24
Successfu	191	1.53104244485628e-08	0.25812440982153\u0036	0.33	0.185	0.000513481015155899	CPT1A
Successfu	191	1.53666799531675e-08	0.26196998276195	0.43	0.262	0.000515367712269333	PTAR1
Successfu	191	1.54531870251703e-08	0.55306130308987\u0033	0.602	0.456	0.00051826898645016	MAPK8
Successfu	191	1.65149430474497e-08	0.50166611959462\u0039	0.695	0.553	0.000553878159925367	NCALD
Successfu	191	1.68953842331888e-08	0.30659438779417\u0034	0.498	0.311	0.000566637396412687	ESR2
Successfu	191	1.69692031805478e-08	0.25515292108616\u0039	0.308	0.167	0.000569113136269212	NBPF19
Successfu	191	1.73186592751543e-08	0.36851787193689\u0038	0.326	0.185	0.000580833194770124	SLC4A4
Successfu	191	1.766069323488e-08	0.50503603342640\u0031	0.28	0.146	0.000592304329711406	PHLDB2
Successfu	191	1.7768235204019e-08	0.78669343869126\u0038	0.86	0.786	0.000595911072272389	FAM107B
Successfu	191	1.79288667003327e-08	0.32810148039009\u0035	0.491	0.313	0.000601298331395757	UHRF1BP1L
Successfu	191	1.84531790659082e-08	0.33198260245141\u0034	0.817	0.668	0.000618882719512428	VTI1A
Successfu	191	1.8521017722349e-08	0.35830735145975\u0034	0.674	0.498	0.00062115789237214	SCAPER
Successfu	191	1.86288570290978e-08	0.35380433262736	0.971	0.928	0.00062477460704188	GNG2
Successfu	191	1.86481495790041e-08	0.29710753566745\u0039	0.491	0.31	0.000625421640580639	MTMR3
Successfu	191	1.86543821602841e-08	0.53508775713486	0.47	0.309	0.000625630668891607	AC008014.1
Successfu	191	1.90001518355144e-08	0.31070302623732\u0038	0.71	0.507	0.000637227092259482	ETF1
Successfu	191	1.96469078457222e-08	0.29894837682711\u0035	0.455	0.29	0.000658917995329833	TAF2
Successfu	191	1.96842010829116e-08	0.46345478420351\u0037	0.76	0.609	0.000660168735918688	TLE4
Successfu	191	1.97324102748369e-08	0.31296766373939\u0035	0.832	0.698	0.000661785575797479	EIF4G2
Successfu	191	2.29232259733784e-08	0.40546855067164\u0033	0.62	0.452	0.000768799152695163	UMAD1
Successfu	191	2.30910422086735e-08	0.26092021726493\u0035	0.315	0.171	0.000774427373594493	CEBPD
Successfu	191	2.43269666028471e-08	0.44692149882231\u0034	0.441	0.28	0.000815877805926287	CDK19
Successfu	191	2.43471624324044e-08	0.37545172009602\u0035	0.609	0.419	0.000816555133657979	TBC1D15
Successfu	191	2.48380639081606e-08	0.27276471847495\u0032	0.638	0.448	0.000833018987351891	BDP1
Successfu	191	2.68865552069291e-08	0.35128409391725\u0037	0.627	0.449	0.000901721288529987	PLEKHA2
Successfu	191	2.68984479129832e-08	0.31007026305486	0.366	0.208	0.00090212014610563	AC092652.2
Successfu	191	2.73796079344732e-08	0.27795080072997\u0036	0.427	0.256	0.000918257290906362	TTC7A
Successfu	191	2.86846590552396e-08	0.27473766105832\u0033	0.613	0.425	0.000962026095394625	WAPL
Successfu	191	2.88313891418086e-08	0.34875414168754\u0037	0.67	0.488	0.000966947129037976	TNRC6C
Successfu	191	2.96473017554094e-08	0.36581532631730\u0039	0.602	0.44	0.000994311206272919	MAN1A2

Successfu	191	3.03226099365836e-08	0.266900645524997	0.416	0.254	0.00101695969205314	ILKAP
Successfu	191	3.03956241567958e-08	0.34004911772661	0.477	0.303	0.00101940844297062	HECTD4
Successfu	191	3.06335741439059e-08	0.251709252133546	0.233	0.113	0.00102738880963831	AC098829.1
Successfu	191	3.15406067040679e-08	0.294713871401617	0.964	0.906	0.00105780886764103	PIP4K2A
Successfu	191	3.19787855028861e-08	0.328349008459835	0.563	0.38	0.00107250450819579	RAB3GAP2
Successfu	191	3.27304532903563e-08	0.284002882315434	0.735	0.562	0.00109771394245197	CNOT4
Successfu	191	3.29063500509985e-08	0.308939719923322	0.419	0.253	0.00110361316801039	FAM168A
Successfu	191	3.33547843308371e-08	0.378432928550131	0.581	0.393	0.00111865275688761	SNTB1
Successfu	191	3.33863386874317e-08	0.345270516969156	0.914	0.794	0.00111971102689909	ARAP2
Successfu	191	3.35617437275832e-08	0.333940805544312	0.728	0.545	0.00112559376113568	ASCC3
Successfu	191	3.38629515960922e-08	0.284056239430996	0.842	0.68	0.00113569567062974	CDKAL1
Successfu	191	3.45545440025269e-08	0.35750294116589	0.452	0.286	0.00115889029675675	BRWD3
Successfu	191	3.51180969878225e-08	0.293660181890224	0.441	0.276	0.00117779073677759	ISCA1
Successfu	191	3.57283619510609e-08	0.330572009751286	0.516	0.343	0.00119825780311468	C1GALT1
Successfu	191	3.68084618511347e-08	0.331410807895393	0.663	0.486	0.00123448219356336	STK39
Successfu	191	3.76291388033186e-08	0.326617179425072	0.233	0.113	0.0012620060571857	TTN
Successfu	191	3.85586593936277e-08	0.320735709762459	0.591	0.398	0.00129318031874349	BRD1
Successfu	191	4.0008221550123e-08	0.30764005130988	0.943	0.842	0.00134179573434803	GPBP1
Successfu	191	4.03512415511669e-08	0.34302467961185	0.616	0.433	0.00135329993914304	PITPNB
Successfu	191	4.04448701614501e-08	0.31708790377335	0.455	0.294	0.00135644005547471	FBXO33
Successfu	191	4.1763704752753e-08	0.273768557087904	0.835	0.675	0.00140067112999783	IVNS1ABP
Successfu	191	4.2018665889227e-08	0.485933219682239	0.685	0.552	0.00140922201659289	NR3C2
Successfu	191	4.22643454526659e-08	0.410928000790267	0.882	0.75	0.00141746161779151	FBXO11
Successfu	191	4.26230768604541e-08	0.284305349696678	0.616	0.427	0.00142949275174591	NDEL1
Successfu	191	4.39682262465231e-08	0.353990462857846	0.667	0.492	0.00147460637185589	GNAI3
Successfu	191	4.49056753008149e-08	0.303779168382023	0.437	0.268	0.00150604653823873	CNOT9
Successfu	191	4.911663118639e-08	0.387413215674872	0.455	0.277	0.00164727357672915	FAM126A
Successfu	191	5.10540873846843e-08	0.315968841081896	0.57	0.392	0.00171225198270754	CUL1
Successfu	191	5.12624202165768e-08	0.412497092320793	0.781	0.671	0.00171923904922355	BBX
Successfu	191	5.12689876469575e-08	0.409755932302569	0.323	0.18	0.00171945930770366	SLC45A4
Successfu	191	5.26100296380398e-08	0.38810014768353	0.581	0.416	0.00176443517400058	EPG5

Successfu	191	5.38999879461327e-08	0.41454950239384[illegible]	0.419	0.269	0.0018076977957374	FOCAD
Successfu	191	5.41667531700818e-08	0.365511123925518	0.416	0.261	0.0018166445678182	RPS6KC1
Successfu	191	5.42202941392296e-08	0.25181508556562[illegible]	0.584	0.396	0.00181844022484148	C18orf25
Successfu	191	5.62429727956183e-08	0.28194149652467[illegible]	0.455	0.285	0.00188627682161945	KDM3A
Successfu	191	5.64656016659535e-08	0.28145534881918	0.427	0.257	0.00189374334867275	AFDN
Successfu	191	5.73862088839375e-08	0.31421431837061[illegible]	0.728	0.54	0.0019246186735495	CDK13
Successfu	191	5.74498121347525e-08	0.29826102564061[illegible]	0.828	0.656	0.00192675179937533	KANSL1
Successfu	191	6.04860116949004e-08	0.34747699089266	0.67	0.495	0.00202857986022357	PREX1
Successfu	191	6.18877129603892e-08	0.29603536030013[illegible]	0.452	0.288	0.00207559011726553	SNX29
Successfu	191	6.79972903129574e-08	0.31818900788193[illegible]	0.821	0.671	0.00228049312251597	COP1
Successfu	191	7.06510801158923e-08	0.33706584668164[illegible]	0.681	0.513	0.0023694959249268	KIAA0232
Successfu	191	7.07697580377102e-08	0.30504998501195[illegible]	0.688	0.497	0.00237347614506872	TRPM7
Successfu	191	7.09041310876644e-08	0.42120628963513[illegible]	0.953	0.865	0.00237798274841809	ATXN1
Successfu	191	7.10952481129244e-08	0.25707667850030[illegible]	0.548	0.369	0.00238439243121126	TP53BP2
Successfu	191	7.17556692690367e-08	0.26947481553695[illegible]	0.384	0.227	0.00240654163594495	MTMR1
Successfu	191	7.36152098389041e-08	0.25610920617861[illegible]	0.753	0.549	0.00246890690757717	DHX9
Successfu	191	7.4689702884655e-08	0.27035823928507	0.72	0.534	0.00250494325534556	UBAP1
Successfu	191	8.19228121211957e-08	0.760511925115602	0.52	0.394	0.00274752727292066	AOAH
Successfu	191	8.4862149468012e-08	0.25014779737871[illegible]	0.624	0.422	0.00284610676885819	RNF138
Successfu	191	8.6916922778516e-08	0.57992796320564	0.71	0.532	0.00291501975614587	QKI
Successfu	191	8.72108345410042e-08	0.41280696774361[illegible]	0.566	0.397	0.0029248769688362	TAF3
Successfu	191	8.9008714323898e-08	0.30342667676733	0.703	0.518	0.00298517426099489	GLG1
Successfu	191	8.97319706159159e-08	0.36133569088624	0.588	0.408	0.00300943083051659	NAA50
Successfu	191	9.84232272809676e-08	0.32381381250100[illegible]	0.373	0.227	0.00330091819654909	PIK3CA
Successfu	191	9.92526955484999e-08	0.26538439596710[illegible]	0.591	0.413	0.00332873690330559	PRKCE
Successfu	191	9.9458488163508e-08	0.25573388002875[illegible]	0.502	0.322	0.00333563877602773	FBXW4
Successfu	191	1.00571061595886e-07	0.40074145268691[illegible]	0.857	0.769	0.00337295226380282	NR3C1
Successfu	191	1.01664195017644e-07	0.26427122306185[illegible]	0.297	0.164	0.00340961377250174	ALG13-AS1
Successfu	191	1.02428670324915e-07	0.25971051016250[illegible]	0.358	0.206	0.00343525274535699	VTA1
Successfu	191	1.03913667212637e-07	0.33487000908169[illegible]	0.581	0.421	0.00348505657097743	RELL1
Successfu	191	1.04002038585182e-07	0.26478801255545[illegible]	0.688	0.491	0.00348802037006985	BRD4

Successfu	191	1.04868127882709e-07	0.35735217046926	0.649	0.468	0.00351706727293029	SCAF8
Successfu	191	1.0567371795759e-07	0.36685714051801	0.33	0.194	0.00354408515286167	MCF2L2
Successfu	191	1.06942441255646e-07	0.47979113043962	0.756	0.589	0.00358663559483184	REV3L
Successfu	191	1.07205360073074e-07	0.44705556217605	0.792	0.651	0.00359545336613075	FBXL17
Successfu	191	1.10822264147258e-07	0.28806968568236	0.638	0.454	0.00371675709497074	RB1CC1
Successfu	191	1.11140068454619e-07	0.32582467484335	0.287	0.158	0.00372741561583102	IL21R
Successfu	191	1.13714996865154e-07	0.27394584824938	0.645	0.473	0.00381377356486352	FAM13B
Successfu	191	1.14615034807358e-07	0.47304221547109	0.781	0.626	0.00384395903736916	NR4A3
Successfu	191	1.15453265905199e-07	0.41141390357219	0.627	0.444	0.00387207163192855	SLA
Successfu	191	1.16559688040611e-07	0.29022110737498	0.341	0.194	0.003909178817506	RNF24
Successfu	191	1.20694412021157e-07	0.28800784338468	0.685	0.509	0.00404784919036555	TENT2
Successfu	191	1.21481503031452e-07	0.31464973218143	0.778	0.592	0.00407424664866883	RBM6
Successfu	191	1.23452576127826e-07	0.30855030152342	0.76	0.569	0.00414035249817502	ZNF638
Successfu	191	1.23941926664147e-07	0.36984125176126	0.462	0.304	0.00415676433646215	DMXL1
Successfu	191	1.25715779211551e-07	0.33327115754260	0.76	0.605	0.00421625580319	SETD2
Successfu	191	1.27686942881561e-07	0.29399775308198	0.724	0.546	0.00428236469036179	FOSL2
Successfu	191	1.34736044445913e-07	0.36589991450587	0.828	0.693	0.00451877745862703	CEMIP2
Successfu	191	1.3507754549894e-07	0.37296856398521	0.534	0.369	0.00453023072094345	PHLDA1
Successfu	191	1.36555021768263e-07	0.34418640679167	0.455	0.292	0.004579782320064	SP4
Successfu	191	1.38093073689696e-07	0.2941100803172	0.738	0.558	0.00463136550540502	TBC1D22A
Successfu	191	1.41648445848519e-07	0.52336254213001	0.556	0.407	0.00475060557686764	SEPT11
Successfu	191	1.42986701482225e-07	0.29683413877695	0.455	0.287	0.00479548799431086	IL18R1
Successfu	191	1.47022809889652e-07	0.36356939008047	0.634	0.466	0.00493085099807916	FCHSD2
Successfu	191	1.48714794777827e-07	0.34065947113501	0.559	0.383	0.00498759678725877	SMAD2
Successfu	191	1.4954139933988e-07	0.29365534166222	0.624	0.442	0.0050153194510609	SUCO
Successfu	191	1.55258456580398e-07	0.54098014006714	0.183	0.086	0.00520705811679339	KIT
Successfu	191	1.57639608977186e-07	0.35808526984388	0.806	0.662	0.00528691720587686	HIPK1
Successfu	191	1.58341251527101e-07	0.3313150581381	0.975	0.943	0.00531044889371591	TNFAIP3
Successfu	191	1.58793747157837e-07	0.28247814583122	0.674	0.503	0.00532562469217953	SMARCC1
Successfu	191	1.58862176750226e-07	0.27497824099245	0.781	0.587	0.00532791968384908	TLK1
Successfu	191	1.65118294251908e-07	0.25918614103733	0.323	0.184	0.00553773735262051	EXT2

Successfu	191	1.71918409202124e-07	0.414811466081091	0.849	0.747	0.00576579960782083	CRY1
Successfu	191	1.75292036483936e-07	0.281171468737917	0.9	0.729	0.00587894431959826	TCF12
Successfu	191	1.78150466866604e-07	0.267415215576013	0.538	0.36	0.00597481035777217	TACC1
Successfu	191	1.78955459401622e-07	0.349498900661871	0.649	0.492	0.00600180819741159	ETV6
Successfu	191	1.87413220322965e-07	0.331168187271343	0.577	0.407	0.0062854645831916	CSGALNACT2
Successfu	191	1.91239172880625e-07	0.258774920030382	0.444	0.28	0.00641377938007039	GSE1
Successfu	191	1.96070028833016e-07	0.252763132912548	0.832	0.677	0.0065757966270017	ATP11B
Successfu	191	1.96183319478157e-07	0.352273599647497	0.645	0.479	0.00657959616865841	GBF1
Successfu	191	1.98494555757278e-07	0.319504682812368	0.48	0.326	0.00665711041098758	SNX13
Successfu	191	1.9909861531701e-07	0.395630787141927	0.774	0.653	0.00667736936050188	RPS6KA5
Successfu	191	2.0155074556193e-07	0.332461954589091	0.799	0.652	0.00675960890465602	TAOK3
Successfu	191	2.02880600669056e-07	0.314851498167046	0.767	0.619	0.00680420958523881	OGT
Successfu	191	2.09405728965087e-07	0.293018437057054	0.821	0.645	0.00702304933803109	GSK3B
Successfu	191	2.12345210632737e-07	0.31866111409003	0.695	0.521	0.00712163367420075	ITCH
Successfu	191	2.13234328911206e-07	0.281887955129171	0.548	0.373	0.00715145292302401	CLIP1
Successfu	191	2.15638689128893e-07	0.350288448283658	0.509	0.346	0.00723209035600482	HOOK3
Successfu	191	2.16447855126724e-07	0.27332636548192	0.545	0.378	0.00725922816524007	ARNTL
Successfu	191	2.19687501441995e-07	0.301392534438221	0.677	0.492	0.00736787942336164	TAOK1
Successfu	191	2.23105288322954e-07	0.302705370176447	0.907	0.817	0.00748250515977525	JAK1
Successfu	191	2.29071803876674e-07	0.404756117321903	0.401	0.257	0.00768261015841588	SAMD3
Successfu	191	2.40093593474315e-07	0.462876772948551	0.237	0.127	0.00805225893794159	SVIL
Successfu	191	2.4759563450673e-07	0.287269284105427	0.487	0.326	0.0083038623900867	AHCTF1
Successfu	191	2.6569765975703e-07	0.351714449154957	0.649	0.5	0.00891096811293127	XPR1
Successfu	191	2.700365574003e-07	0.326548388447298	0.753	0.565	0.00905648606209127	RICTOR
Successfu	191	2.82869622915592e-07	0.301402732514689	0.502	0.336	0.00948688141334311	SP140L
Successfu	191	2.86602433596359e-07	0.356209824856028	0.466	0.304	0.0096120724179547	LINC02328
Successfu	191	2.90129974487334e-07	0.284334467780525	0.548	0.378	0.00973037908435619	AP4B1-AS1
Successfu	191	2.94710818687491e-07	0.294754404225205	0.577	0.402	0.00988401143714107	CNTRL
Successfu	191	2.98028517949067e-07	0.264544838600221	0.667	0.495	0.00999528043497582	BTAF1
Successfu	191	3.21163872427728e-07	0.300323311861037	0.692	0.514	0.0107711939534812	SMAP1
Successfu	191	3.33063470127352e-07	0.381759185004657	0.717	0.569	0.0111702826611311	STK24

Successfu	191	3.34715959865063e-07	0.34354517452720	0.789	0.61	0.0112257038619545	ZNF292
Successfu	191	3.52528003365756e-07	0.36326689619778	0.717	0.564	0.0118230841768807	EXOC6B
Successfu	191	3.6709874628819e-07	0.28999991621693	0.462	0.302	0.0123117577530133	TCF20
Successfu	191	3.76265614070078e-07	0.50757859749421	0.394	0.252	0.0126191961646823	BBS9
Successfu	191	3.77595130461148e-07	0.30996148574506	0.896	0.778	0.012663785485406	RASA2
Successfu	191	3.82861867109855e-07	0.25296757260643	0.849	0.65	0.0128404212991303	FUBP1
Successfu	191	3.88775758091143e-07	0.34736407491321	0.903	0.743	0.0130387613748608	CD247
Successfu	191	3.94483836627603e-07	0.29335340450440	0.853	0.706	0.0132301989128165	CSNK1A1
Successfu	191	3.96433170925301e-07	0.31304206699355	0.423	0.276	0.0132955756864927	TMEM245
Successfu	191	3.99372680976266e-07	0.26749147522263	0.419	0.267	0.013394160974582	PLEKHG1
Successfu	191	4.00105361126924e-07	0.27756713333611	0.312	0.183	0.0134187336014748	CCL4L2
Successfu	191	4.09832160678978e-07	0.30530105489376	0.667	0.503	0.0137449510048516	TUT4
Successfu	191	4.18323591441744e-07	0.36082536178239	0.48	0.331	0.0140297366097732	RSU1
Successfu	191	4.2012653517541e-07	0.37527503037264	0.441	0.3	0.0140902037367129	AGTPBP1
Successfu	191	4.45256118844355e-07	0.29276922945793	0.376	0.23	0.014932999713802	MAN1C1
Successfu	191	4.49551135239932e-07	0.25017499778377	0.602	0.426	0.0150770459736769	DENND4C
Successfu	191	4.63897177270697e-07	0.53281737462216	0.703	0.597	0.0155581835313046	IMMP2L
Successfu	191	4.7091406887308e-07	0.32436751526549	0.505	0.345	0.0157935160418654	TET2
Successfu	191	4.73186174841942e-07	0.26893295902624	0.265	0.147	0.0158697179318491	DUSP6
Successfu	191	4.85998660252155e-07	0.25346160591914	0.538	0.375	0.0162994230675368	TNKS2
Successfu	191	5.00041693916687e-07	0.25547836349472	0.978	0.926	0.0167703983305779	PITPNC1
Successfu	191	5.21540232136121e-07	0.27154207024375	0.437	0.283	0.0174914163053812	WDR37
Successfu	191	5.27809154824361e-07	0.41118995981481	0.803	0.672	0.0177016634344994	CAMK2D
Successfu	191	5.37813685484275e-07	0.33186986723490	0.652	0.494	0.0180371953837716	FOXJ3
Successfu	191	5.38884559770613e-07	0.26907779032765	0.778	0.606	0.0180731103655868	PTPN4
Successfu	191	5.44588366983398e-07	0.26541513578881	0.588	0.404	0.0182644046518892	USP8
Successfu	191	5.47463206637266e-07	0.38998098658625	0.649	0.467	0.0183608210242006	PNPLA8
Successfu	191	5.48131762627301e-07	0.32373426059419	0.724	0.58	0.0183832430549944	UBE2K
Successfu	191	5.51433943994418e-07	0.41538343851610	0.538	0.397	0.0184939916136848	SCFD2
Successfu	191	5.68002434004418e-07	0.28883553636237	0.735	0.541	0.0190496656316402	RYBP
Successfu	191	5.72530471147069e-07	0.37174339140926	0.409	0.262	0.0192015269413304	DPYD-AS1

Successfu	191	5.79720607992771e-07	0.381590383847752	0.47	0.327	0.0194426697508616	PCCA
Successfu	191	5.81986565403264e-07	0.301848700097294	0.642	0.466	0.0195186654304947	CLINT1
Successfu	191	5.88711587842248e-07	0.296252403745962	0.796	0.637	0.0197442092330533	HUWE1
Successfu	191	5.89887076421537e-07	0.368813124666646	0.817	0.693	0.0197836327690255	GOLGB1
Successfu	191	6.226709341539e-07	0.268853108232608	0.573	0.396	0.0208831377896535	SLAMF1
Successfu	191	6.27408535087773e-07	0.259898349442439	0.498	0.329	0.0210420274497737	ARHGAP35
Successfu	191	6.31018266398742e-07	0.286171122919837	0.853	0.692	0.021163090618481	TAX1BP1
Successfu	191	6.33872874499328e-07	0.256132217478531	0.846	0.728	0.0212588284649585	CUL3
Successfu	191	6.48206111083755e-07	0.257626028380946	0.477	0.313	0.021739536553527	R3HDM1
Successfu	191	7.4619500953666e-07	0.358169817569778	0.677	0.549	0.0250258882298405	DLG1
Successfu	191	8.05522342583927e-07	0.335293348286025	0.738	0.581	0.0270156083255798	SMG6
Successfu	191	8.29322629906401e-07	0.480215599868075	0.527	0.397	0.0278138223618009	SUMF1
Successfu	191	8.43370629534934e-07	0.614832098462336	0.577	0.463	0.0282849641733426	PPP2R2B
Successfu	191	8.71307923634699e-07	0.612718570798561	0.667	0.531	0.0292219251428605	SERINC5
Successfu	191	9.89630754445673e-07	0.298548513234366	0.573	0.421	0.033190236242599	UBE2G1
Successfu	191	1.00464432734524e-06	0.266713375384485	0.441	0.289	0.0336937614505047	SEC63
Successfu	191	1.02820429770124e-06	0.266852097396866	0.412	0.269	0.0344839157363043	WASHC4
Successfu	191	1.05701397744653e-06	0.250662097125358	0.659	0.458	0.0354501347756019	UBE3A
Successfu	191	1.07091891221124e-06	0.328415997901341	0.814	0.675	0.0359164784777404	ERBIN
Successfu	191	1.0990582958376e-06	0.369605770540379	0.448	0.31	0.0368602171258015	PPP1R12B
Successfu	191	1.11377182721686e-06	0.440252150456127	0.315	0.197	0.037353679541199	FAM69A
Successfu	191	1.12056734507584e-06	0.260082974049449	0.455	0.302	0.0375815876191535	PIK3C3
Successfu	191	1.1214076918679e-06	0.363693015968045	0.674	0.516	0.0376097711698656	SYTL2
Successfu	191	1.15531371072109e-06	0.253421708191196	0.477	0.325	0.0387469112301639	PI4KA
Successfu	191	1.18461770857386e-06	0.288103186719393	0.588	0.409	0.03972970871015	TRIM33
Successfu	191	1.20947152188643e-06	0.376705309828087	0.799	0.695	0.0405632559010271	CCDC91
Successfu	191	1.21102541291593e-06	0.265887364976638	0.344	0.208	0.0406153702983745	TMEM168
Successfu	191	1.29047454936272e-06	0.275975191010545	0.534	0.365	0.0432799354365268	LPGAT1
Successfu	191	1.31117236043035e-06	0.264012579418982	0.832	0.63	0.0439740986241131	SMG1
Successfu	191	1.33974834522452e-06	0.252037825998535	0.577	0.392	0.0449324800021398	ERC1
Successfu	191	1.35958703424961e-06	0.311952587793635	0.129	0.055	0.0455978299546634	DOCK3

Successfu	191	1.36459581628649e-06	0.33584760348253€	0.552	0.403	0.0457658144866163	REPS1
Successfu	191	1.36467812888251e-06	0.25821278924026З	0.534	0.371	0.0457685750864618	GIGYF2
Successfu	191	1.38375880313311e-06	0.29947343496448	0.91	0.815	0.0464085027394782	CCNH
Successfu	191	1.43002861507492e-06	0.41699620862613€	0.857	0.723	0.0479602996923827	G3BP2
Successfu	191	1.45856940420345e-06	0.45899167198954?	0.735	0.622	0.0489175006781752	SSBP2
Successfu	191	1.47106351678893e-06	0.31030109507171	0.401	0.264	0.0493365282260671	JAK2
Recoverec	0	1.11356637137856e-16€	1.86235432311295	0.747	0.042	3.73467889632941e-162	REG1B
Recoverec	0	3.283709188632e-50	1.74275351180392	1	0.765	1.1012903876834e-45	IGKC
Recoverec	0	1.46257063791424e-42	1.47933209211432	0.99	0.625	4.9051694054368e-38	PHGR1
Recoverec	0	2.34173499262036e-40	1.38030127230435	0.909	0.413	7.85371081825015e-36	APOA4
Recoverec	0	1.38248231160215e-34	1.351652219847	0.98	0.754	4.63656917665128e-30	FABP6
Recoverec	0	5.31522434633759e-34	1.25922192340094	0.96	0.511	1.7826199412747e-29	REG1A
Recoverec	0	9.68391926972175e-32	1.18041351008737	0.788	0.308	3.24779284467928e-27	JCHAIN
Recoverec	0	8.38593305766106e-27	1.11729547916542	0.576	0.184	2.81247422887837e-22	PDE3A
Recoverec	0	1.10693263185384e-25	1.28948088203307	0.768	0.34	3.71243066071142e-21	IGLC2
Recoverec	0	6.32896593688441e-25	1.08133088867381	0.677	0.266	2.12260859591229e-20	KRT19
Recoverec	0	9.56349048649437e-25	0.97165587634199З	0.768	0.348	3.20740343936048e-20	APOC3
Recoverec	0	1.64916868995899e-24	0.87386906971585€	0.556	0.171	5.53098195238445e-20	HLA-DRA
Recoverec	0	1.42057640538457e-23	0.37163866990649?	0.172	0.017	4.76432914837877e-19	C11orf86
Recoverec	0	4.05716687787411e-22	0.65792640607576€	1	1	1.36069262750142e-17	MT-CO2
Recoverec	0	6.73688832306581e-22	0.33437402475634€	0.141	0.012	2.25941760578981e-17	IGHG4
Recoverec	0	2.94066660251686e-21	0.88806202114653	0.859	0.461	9.86240765152104e-17	CD74
Recoverec	0	4.38776321126378e-21	0.64398156898542З	0.404	0.105	1.47156802579365e-16	CLDN3
Recoverec	0	5.56543618475747e-21	0.96510264673763€	0.869	0.564	1.86653598764396e-16	IGHA1
Recoverec	0	1.26429199901299e-19	0.77724685541760€	0.495	0.163	4.24018250628976e-15	CLDN4
Recoverec	0	1.32801344702765e-19	0.89823042826621€	0.616	0.239	4.45389149864132e-15	IGLC3
Recoverec	0	1.20092079688516e-18	0.55942519610221€	0.273	0.056	4.02764816859346e-14	HLA-DRB5
Recoverec	0	5.27236053072483e-18	0.80158493476715€	0.505	0.181	1.76824427479449e-13	ADIRF
Recoverec	0	7.5473503349005e-18	0.84119801623565€	0.525	0.196	2.53123035531893e-13	SLC5A1
Recoverec	0	8.61807947296991e-18	0.85728229576221€	0.475	0.168	2.89033149364465e-13	MUC17
Recoverec	0	2.135225940508e-17	1.020469411296	0.485	0.182	7.16112075927574e-13	GUCA2A

Recoverec	0	3.69916957607677e-17	0.93860570672706	0.788	0.49	1.24062749242463e-12	FABP1
Recoverec	0	8.15290427221788e-17	0.528861118999233	1	1	2.73432103481643e-12	MT-CO3
Recoverec	0	3.07381559413251e-16	1.26150265237938	0.303	0.075	1.03089627396016e-11	ADAM28
Recoverec	0	9.80355102063366e-16	0.68161977594582(	0.485	0.183	3.28791494130012e-11	MUC13
Recoverec	0	2.02072571755046e-15	0.26207808842514!	0.131	0.016	6.77710991152073e-11	MS4A1
Recoverec	0	2.4850589090711e-15	0.53680728962770:	1	1	8.33439056924265e-11	MT-ATP6
Recoverec	0	2.57676125330313e-15	0.57734279646691!	1	1	8.64194189132803e-11	MT-CYB
Recoverec	0	3.10634956903833e-15	0.57692156103195!	0.384	0.119	1.04180751846407e-10	PMP22
Recoverec	0	3.49227798123849e-15	0.54802159054443	0.434	0.146	1.17124018934777e-10	IFI27
Recoverec	0	4.5548930647884e-15	0.82650324951268(	0.566	0.252	1.52762003606874e-10	CYP3A5
Recoverec	0	4.67879042537578e-15	0.77513195794046(	0.687	0.371	1.56917273286253e-10	KRT8
Recoverec	0	1.01832065860869e-14	0.63909763154215{	0.202	0.039	3.41524382484183e-10	BANK1
Recoverec	0	1.0424563782615e-14	0.77800144141346!	0.798	0.517	3.49619020141343e-10	ALDOB
Recoverec	0	1.41166019945955e-14	0.69344520922120:	0.98	0.878	4.73442597694744e-10	FTL
Recoverec	0	3.60013890557471e-14	0.80700716632683	0.545	0.243	1.20741458615164e-09	EPCAM
Recoverec	0	4.4163891990806e-14	0.58246592938526!	0.374	0.122	1.48116860958765e-09	HLA-DRB1
Recoverec	0	4.69238916729682e-14	0.76905880710578!	0.545	0.245	1.57373347892801e-09	GDA
Recoverec	0	6.07421843945277e-14	0.53720792015479(	1	0.998	2.03717138022367e-09	MT-ND4
Recoverec	0	7.10713400765589e-14	0.68440242177464	0.636	0.328	2.38359060348763e-09	SELENOP
Recoverec	0	1.11997625741092e-13	0.71602909730296!	0.374	0.122	3.75617637210474e-09	LYN
Recoverec	0	3.8774758648214e-13	0.74186764068744!	0.949	0.843	1.3004278555438e-08	RPL8
Recoverec	0	1.61162238738723e-12	1.43815670898456	0.323	0.105	5.40505916281929e-08	AC120193.1
Recoverec	0	1.74090530470246e-12	0.40004641930190{	0.192	0.041	5.83864821091111e-08	MIR3142HG
Recoverec	0	1.91861476375063e-12	0.61378429213770	0.455	0.187	6.43465019466685e-08	FGD4
Recoverec	0	2.24034061763555e-12	0.77202431093190	0.97	0.899	7.51365436342611e-08	RPS27
Recoverec	0	3.66391382112685e-12	0.53830852215516	0.414	0.158	1.22880341732952e-07	TMC5
Recoverec	0	6.94068788381387e-12	0.27203370728874	0.101	0.013	2.3277679024735e-07	LCN2
Recoverec	0	7.21733283898083e-12	0.80332431354915!	0.859	0.607	2.42054908753739e-07	FKBP5
Recoverec	0	7.54756447401546e-12	0.53634717826910	0.273	0.081	2.5313021732953e-07	CYP3A4
Recoverec	0	1.05846774598349e-11	1.08648815308257	0.727	0.464	3.54988912647944e-07	HSPA1A
Recoverec	0	1.92443678571467e-11	0.64738083088735!	0.485	0.222	6.45417609192987e-07	TSPAN8

Recoverec	0	1.98846449421841e-11	0.63742569192651i	0.374	0.145	6.66891222070969e-07	ELF3
Recoverec	0	2.46196629666802e-11	0.89424136576084{	0.667	0.413	8.25694256576521e-07	H1FX
Recoverec	0	2.52265078901469e-11	0.75191176746855{	0.899	0.762	8.46046621619748e-07	RPS5
Recoverec	0	7.70499412431217e-11	1.00313540339146	0.404	0.155	2.58410092941182e-06	IKZF2
Recoverec	0	1.287001695927e-10	0.43088212223951{	0.222	0.062	4.31634628779999e-06	FXYD3
Recoverec	0	1.32746199455321e-10	0.71683484170124{	0.869	0.758	4.45204203733256e-06	RPS21
Recoverec	0	1.75376957876181e-10	0.56815609652720:	0.404	0.17	5.88179241325136e-06	IGHM
Recoverec	0	2.0350362867514e-10	0.54662239017217{	0.465	0.216	6.82510469850685e-06	LGALS4
Recoverec	0	2.97027338982672e-10	0.88715419985646{	0.707	0.44	9.96170289480085e-06	HSPA1B
Recoverec	0	3.02876865578601e-10	0.73547981925477{	0.98	0.904	1.01578843177751e-05	RPS19
Recoverec	0	3.12041759047841e-10	0.78445183578937:	0.869	0.841	1.04652565149465e-05	RPS29
Recoverec	0	3.17302348595831e-10	0.66379704014183{	0.939	0.853	1.0641686167207e-05	RPS15
Recoverec	0	3.20686073299533e-10	0.75109191049243:	0.788	0.541	1.07551695263197e-05	HSPE1
Recoverec	0	3.30936342379446e-10	0.63478652146727{	0.636	0.369	1.10989430507219e-05	LGALS3
Recoverec	0	3.61755413676411e-10	0.71079445688799{	0.859	0.757	1.21325530638795e-05	UBB
Recoverec	0	4.28823230665516e-10	0.53985442946031i	0.515	0.249	1.43818735100601e-05	RBP2
Recoverec	0	4.5863318181796e-10	0.36929262486499i	0.354	0.134	1.53816396518107e-05	SGK1
Recoverec	0	4.59213411185115e-10	0.72673169642974{	0.909	0.78	1.54010993843264e-05	RPL10A
Recoverec	0	4.84743874226682e-10	0.35743736586504{	0.192	0.047	1.62573400538145e-05	KIR2DL4
Recoverec	0	5.28709117483173e-10	0.63384462469686i	0.535	0.275	1.77318463821507e-05	MYO1E
Recoverec	0	7.3666104808901e-10	0.54134416133950	0.323	0.121	2.47061382308092e-05	CDHR2
Recoverec	0	1.23876474229322e-09	0.59044865251524:	0.364	0.146	4.15456919270301e-05	HLA-DPB1
Recoverec	0	1.30003001443357e-09	0.61239347928717:	0.192	0.051	4.36004066240731e-05	ARHGAP24
Recoverec	0	2.15849127137184e-09	0.29179840599402{	1	1	7.23914802592687e-05	MT-CO1
Recoverec	0	2.26002609032751e-09	0.53446324839199{	0.434	0.205	7.57967550174039e-05	NEDD4L
Recoverec	0	2.5498078397688e-09	0.50408295826993	0.677	0.412	8.55154553301659e-05	ANPEP
Recoverec	0	2.90284192363765e-09	0.66600989990392	0.889	0.79	9.73555124349597e-05	RPL22
Recoverec	0	3.03545651647675e-09	0.62654381977906{	0.364	0.15	0.000101803140649597	TIGIT
Recoverec	0	3.43823383956856e-09	0.74826123505452{	0.929	0.889	0.00011531148651145	RPS8
Recoverec	0	3.77901551583605e-09	0.38421214851121	0.263	0.089	0.00012674062237011	PRAP1
Recoverec	0	4.04324945816743e-09	0.41248517064823{	0.283	0.103	0.000135602500328019	MYO15B

Recoverec	0	4.19530012782237e-09	0.484187003435742	0.364	0.154	0.000140701975686907	MYH14
Recoverec	0	4.26380856500531e-09	0.738545067739752	0.677	0.484	0.000142999611653148	CD7
Recoverec	0	4.8007905160107e-09	0.571042435895441	0.535	0.298	0.000161008912325967	ACSL5
Recoverec	0	5.24357486014266e-09	0.65237083053549	0.98	0.847	0.000175859013659465	CCL5
Recoverec	0	5.87600635804918e-09	0.298758674695092	0.152	0.036	0.000197069501236253	TMEM54
Recoverec	0	5.98472815555703e-09	0.577543513443706	0.949	0.863	0.000200715812881072	RPS4X
Recoverec	0	6.04363329993913e-09	0.607437735987245	0.98	0.902	0.000202691373613359	RPL10
Recoverec	0	6.53358170952358e-09	0.555156075151796	0.333	0.137	0.000219123263374002	PCK1
Recoverec	0	7.25229447159909e-09	0.329238058589186	0.212	0.063	0.00024322745198849	HLA-DQB1
Recoverec	0	1.07923717568058e-08	0.626198644465427	0.929	0.892	0.000361954563979754	RPL28
Recoverec	0	1.12377224059088e-08	0.586839493710638	0.919	0.852	0.000376890734049368	RPL29
Recoverec	0	1.29678152549384e-08	0.418704778947803	1	0.995	0.000434914588020124	MT-ND3
Recoverec	0	2.05561719305268e-08	0.654605097227692	0.909	0.834	0.000689412894206009	UBC
Recoverec	0	2.07741989255178e-08	0.468572297076293	0.283	0.107	0.000696725083564016	HLA-DPA1
Recoverec	0	2.13590829403197e-08	0.566491763846303	0.414	0.206	0.000716340923652441	SLC15A1
Recoverec	0	2.36063012596674e-08	0.624234745313621	0.929	0.835	0.000791708131646725	RPL18A
Recoverec	0	2.46209742069066e-08	0.697844425424204	0.364	0.162	0.000825738232951233	KLRC2
Recoverec	0	2.79052981494641e-08	0.42790828979953	0.313	0.126	0.000935887889336727	FCGRT
Recoverec	0	2.81460357654708e-08	0.601994717834552	0.929	0.853	0.000943961747502361	RPL3
Recoverec	0	3.8549834319314e-08	0.39843335750889	0.232	0.079	0.00129288434340115	STXBP6
Recoverec	0	5.38533831603864e-08	0.567664555575211	0.96	0.873	0.00180613476443304	RPS27A
Recoverec	0	5.53466687595678e-08	0.477138868143624	0.273	0.108	0.00185621657685838	CXADR
Recoverec	0	5.54495397444714e-08	0.397111788030341	0.283	0.11	0.00185966666395008	LRRC75A
Recoverec	0	7.38818798459389e-08	0.478627112523143	0.192	0.06	0.0024778504862731	MOGAT3
Recoverec	0	7.44320663385324e-08	0.523092032314364	0.505	0.288	0.0024963026408617	CHCHD10
Recoverec	0	7.61410912325271e-08	0.670214339715868	0.485	0.256	0.00255361991775649	AC015849.1
Recoverec	0	7.74082322812992e-08	0.566379847706094	0.939	0.893	0.00259611729425021	RPS18
Recoverec	0	9.81588460225434e-08	0.315169363455588	0.192	0.06	0.00329205137790406	SLC1A1
Recoverec	0	1.13137102717166e-07	0.467437277100888	0.263	0.101	0.0037943921509283	SERPINA1
Recoverec	0	1.14736326373066e-07	0.571805126250617	0.495	0.279	0.00384802691389988	PIK3IP1
Recoverec	0	1.19140455182944e-07	0.369895139850801	0.232	0.083	0.00399573258592557	TMPRSS2

Recoverec	0	1.42818984502199e-07	0.56327436229969€	0.838	0.71	0.00478986310223474	EEF1D
Recoverec	0	1.65216427891312e-07	0.41706960816871ͅ	0.455	0.236	0.00554102855861883	CYB5A
Recoverec	0	1.74287540106643e-07	0.35757979821272ͅ	0.253	0.095	0.00584525552009658	ST14
Recoverec	0	1.81745020492757e-07	0.39231703965419ͅ	0.192	0.062	0.0060953644972861	SLC6A19
Recoverec	0	1.86123664516362e-07	0.82309037294335€	0.949	0.942	0.00624221546054976	HSP90AA1
Recoverec	0	1.98366446344973e-07	0.51192997486196€	0.434	0.218	0.0066528138775177	GFOD1
Recoverec	0	2.11984418384268e-07	0.564467740111778	0.98	0.905	0.00710953342377157	RPS12
Recoverec	0	2.1205565327477e-07	0.54408036134250ͅ	0.717	0.504	0.00711192249952924	SOD1
Recoverec	0	2.18677761941982e-07	0.47142281414575ͅ	0.374	0.186	0.00733401478001019	CTSD
Recoverec	0	2.3863562589101e-07	0.54738757588548ͅ	0.939	0.892	0.00800336162113271	RPL11
Recoverec	0	2.41946838505139e-07	0.58851240055526ͅ	0.899	0.811	0.00811441306978537	RPLP0
Recoverec	0	2.63385458827722e-07	0.46117012160853ͅ	0.293	0.124	0.00883342151816416	ERRFI1
Recoverec	0	2.82179893628764e-07	0.37998393589027€	0.212	0.074	0.0094637492725215	SLC2A2
Recoverec	0	3.00455640066899e-07	0.52337442008582	0.596	0.381	0.0100766812565637	ANXA2
Recoverec	0	3.29321364744829e-07	0.56490916457686ͅ	0.97	0.895	0.0110447799308121	RPL13
Recoverec	0	3.53250277995414e-07	0.964587486111738	0.808	0.704	0.0118473078234102	DNAJB1
Recoverec	0	3.66192468304272e-07	0.54479171410420ͅ	0.889	0.872	0.0122813630019887	RPL37
Recoverec	0	3.75155786018682e-07	0.52068056015518ͅ	0.96	0.91	0.0125819747514945	RPS2
Recoverec	0	4.28469728533901e-07	0.61478857888468ͅ	0.97	0.918	0.01437001775557	ACTB
Recoverec	0	5.19719770176121e-07	0.33840623565397€	0.202	0.069	0.0174303616521667	SLC7A9
Recoverec	0	5.97773921534117e-07	0.35099394980152ͅ	0.253	0.099	0.0200481417804112	FRMD3
Recoverec	0	6.2925584112734e-07	0.27645314571288ͅ	0.162	0.049	0.0211039823997287	HLA-DMA
Recoverec	0	6.46828259410643e-07	0.40719601955005ͅ	0.556	0.335	0.0216933261641142	MALRD1
Recoverec	0	6.56502431118599e-07	0.41269487297748ͅ	0.323	0.151	0.0220177785348556	THSD4
Recoverec	0	6.70088849471517e-07	0.49834067670568ͅ	0.939	0.879	0.0224734398335757	RPS28
Recoverec	0	6.85660038175358e-07	0.53026810653095ͅ	0.909	0.839	0.0229956663603252	RPL15
Recoverec	0	7.29172763372714e-07	0.37152438696165ͅ	0.364	0.175	0.0244549961379941	PTPRG
Recoverec	0	8.12831240118839e-07	0.49843676149488ͅ	0.313	0.141	0.0272607341311056	LARGE1
Recoverec	0	8.51518431106257e-07	0.38244428107178ͅ	0.343	0.162	0.0285582251424417	SMIM24
Recoverec	0	8.62589732834293e-07	0.50775323100323	0.182	0.058	0.0289295344597965	TRDC
Recoverec	0	9.24778339875305e-07	0.34442300870648ͅ	0.263	0.103	0.031015215962738	ENTPD1

Recoverec	0	1.03417930728075e-06	0.54689172344436	0.96	0.875	0.0346843056075817	RPL30
Recoverec	0	1.18018725352158e-06	0.52047375781356	0.838	0.713	0.0395811201086069	MTRNR2L12
Recoverec	0	1.25289787009539e-06	0.47425202998862	0.99	0.946	0.0420196887672592	RPLP1
Recoverec	0	1.30828731578302e-06	0.54716996376955	0.677	0.513	0.0438773399967308	NR4A1
Recoverec	0	1.36051671946318e-06	0.36198069021259	0.343	0.161	0.045629009737356	C15orf48
Recoverec	0	1.41081600621432e-06	0.43414999581524	0.707	0.504	0.047315947216416	MTRNR2L8
Recoverec	0	1.41160071658775e-06	0.57143690231995	0.606	0.429	0.0473422648329201	PCSK5
Recoverec	0	1.42028168538187e-06	0.52641366645296	0.889	0.836	0.0476334071643371	RPS7
Recoverec	13	2.25359984552092e-46	1.00863648277786	0.902	0.807	7.55812316190808e-42	RPL19
Recoverec	13	5.57527343748174e-39	0.79928999327867	0.761	0.444	1.86983520546263e-34	PPDPF
Recoverec	13	1.33910248787407e-36	0.83655218942565	0.795	0.569	4.49108192383206e-32	RPS10
Recoverec	13	4.41566950844221e-35	0.816209111211728	0.945	0.899	1.48092723974135e-30	RPL10
Recoverec	13	1.44567921069817e-34	0.73754774517054	0.945	0.891	4.84851893683953e-30	RPL13
Recoverec	13	4.53154112887131e-34	0.75101696290853	0.92	0.854	1.51978826380086e-29	RPL7A
Recoverec	13	1.13797734714932e-32	0.78538444805141	0.917	0.858	3.81654842686939e-28	RPS4X
Recoverec	13	1.42366249267348e-32	1.01368096963517	0.917	0.786	4.77467926792832e-28	JUNB
Recoverec	13	4.02714235607133e-32	0.76216801291803	0.924	0.845	1.3506230033792e-27	RPL3
Recoverec	13	5.36079232497767e-32	0.72618107224339	0.905	0.79	1.79790252995101e-27	RPL26
Recoverec	13	1.13119126787169e-31	0.77750407104	0.881	0.683	3.79378927418806e-27	GAPDH
Recoverec	13	6.29321379640324e-31	0.71758043101671	0.887	0.802	2.11061804303772e-26	RPL23A
Recoverec	13	3.88531231398827e-30	0.77341746606243	0.92	0.861	1.30305604386539e-25	RPS3
Recoverec	13	5.20162109751456e-30	0.68131859703339	0.899	0.83	1.74451968368443e-25	RPL18A
Recoverec	13	7.78404648774883e-30	0.66257515333175	0.936	0.874	2.6106135110612e-25	HLA-A
Recoverec	13	8.23782890586753e-30	0.67513069669573	0.969	0.913	2.76280305844985e-25	H3F3B
Recoverec	13	8.64865749529346e-30	0.65406180242890	0.917	0.826	2.90058675077152e-25	RPS7
Recoverec	13	9.94942473263397e-30	0.64162814312290	0.966	0.869	3.33683806683078e-25	FTL
Recoverec	13	1.04515800415556e-29	0.54146946479783	0.991	0.979	3.50525091433693e-25	B2M
Recoverec	13	3.83518417675604e-29	0.65523058821624	0.927	0.842	1.28624406920044e-24	RPS9
Recoverec	13	1.0607571528666e-28	0.69309004023433	0.908	0.8	3.55756733928401e-24	RPLP0
Recoverec	13	2.24471437888199e-28	0.70724784952404	0.872	0.752	7.52832308389441e-24	RPS5
Recoverec	13	6.19090439581245e-28	0.64124584027727	0.927	0.848	2.07630551626758e-23	HLA-C

Recoverec	13	7.15215192655351e-28	0.70216418234983	0.887	0.762	2.39868871312751e-23	RACK1
Recoverec	13	1.42618836340488e-27	0.61271705996898	0.887	0.786	4.78315053318728e-23	FAU
Recoverec	13	7.29152471985781e-27	0.65954685462299	0.927	0.87	2.44543156054591e-22	RPL30
Recoverec	13	1.24351739799312e-26	0.60313203886355	0.899	0.813	4.17050864938934e-22	NACA
Recoverec	13	3.49893956918443e-26	0.58394180270855	0.948	0.901	1.17347435271307e-21	RPL41
Recoverec	13	6.24394684268397e-26	0.59711883382973	0.945	0.866	2.09409489209935e-21	RPS27A
Recoverec	13	1.10811363563503e-25	0.63362460349956	0.896	0.85	3.71639151119278e-21	RPL6
Recoverec	13	1.19912294890569e-25	0.46862709280098	0.951	0.734	4.02161854603991e-21	FABP6
Recoverec	13	1.9834562099248e-25	0.60277026723606	0.92	0.849	6.6521154368458e-21	RPL14
Recoverec	13	2.55883483658535e-25	0.60451647187133	0.917	0.797	8.58182027493996e-21	PNRC1
Recoverec	13	5.98996802059289e-25	0.61372783444283	0.914	0.846	2.00891547474644e-20	RPL29
Recoverec	13	1.03377168271827e-24	0.69516193761591	0.862	0.719	3.46706346950052e-20	EEF1B2
Recoverec	13	1.37574929158302e-24	0.57154184474687	0.96	0.883	4.61398797411112e-20	RPL11
Recoverec	13	1.86307719744156e-24	0.57879137558072	0.96	0.922	6.2483883047795e-20	HLA-B
Recoverec	13	3.58206499503482e-24	0.58209900275701	0.93	0.863	1.20135295803478e-19	RPS23
Recoverec	13	3.69516357961543e-24	0.64266640975337	0.917	0.836	1.23928396133142e-19	RPL8
Recoverec	13	2.54310205187847e-23	0.52065163917300	0.933	0.75	8.52905566159001e-19	IGKC
Recoverec	13	4.84109089463138e-23	0.64947343401795	0.872	0.771	1.62360506424147e-18	RPL10A
Recoverec	13	8.91065209551608e-23	0.55390749964355	0.924	0.886	2.98845449979418e-18	RPS14
Recoverec	13	1.05801541514665e-22	0.85093422654971	0.275	0.091	3.54837209931883e-18	GZMB
Recoverec	13	1.15542774752405e-22	0.39032139287791	1	1	3.87507357964618e-18	MT-CYB
Recoverec	13	1.81101942376211e-22	0.57879422229550	0.963	0.904	6.07379694341335e-18	RPS2
Recoverec	13	6.57370924274322e-22	0.57292733966790	0.939	0.878	2.20469060583122e-17	RPS3A
Recoverec	13	1.00379318820386e-21	0.52287749778943	0.89	0.766	3.3665215945981e-17	NPM1
Recoverec	13	1.28076524229358e-21	0.54176918789665	0.56	0.306	4.29543046960422e-17	SERTAD1
Recoverec	13	1.32791465463789e-21	0.50212893328031	0.914	0.867	4.45356016872457e-17	CALM1
Recoverec	13	1.45226864092422e-21	0.55166271178664	0.945	0.901	4.87061856793163e-17	RPS19
Recoverec	13	1.60363195189507e-21	0.46274340943232	0.927	0.833	5.37826084026569e-17	RPL24
Recoverec	13	2.81071675904809e-21	0.51967481084604	0.777	0.548	9.42658186649547e-17	CYBA
Recoverec	13	3.68513393563415e-21	0.55804923311181	0.893	0.847	1.23592021933298e-16	RPL18
Recoverec	13	4.65723097558236e-21	0.500411119625142	0.911	0.848	1.56194212459081e-16	RPS15

Recoverec	13	7.63999373638688e-21	0.48254748797243[illegible]	0.936	0.886	2.56230109930943e-16	RPL28
Recoverec	13	1.21095114231208e-20	0.54138582738956[illegible]	0.954	0.9	4.06128794108627e-16	RPS12
Recoverec	13	1.33041913993808e-20	0.59419307876797[illegible]	0.92	0.844	4.46195971152434e-16	RPL5
Recoverec	13	2.29325013013971e-20	0.51076544864406[illegible]	0.642	0.403	7.69110228646256e-16	CLIC1
Recoverec	13	8.11542650889748e-20	0.49019328897783[illegible]	0.862	0.694	2.72175174255404e-15	RPL36AL
Recoverec	13	1.43906560138926e-19	0.66707167168887[illegible]	0.856	0.711	4.82633821393928e-15	ZFP36
Recoverec	13	1.45062990703383e-19	0.50580113171353[illegible]	0.853	0.695	4.86512258221005e-15	BTF3
Recoverec	13	2.64820497235851e-19	0.48397745621758[illegible]	0.936	0.881	8.88154983629596e-15	RPL13A
Recoverec	13	3.05672199919103e-19	0.50870378864658[illegible]	0.685	0.45	1.02516342408869e-14	LDHA
Recoverec	13	3.49426185071602e-19	0.52645745389841	0.859	0.76	1.17190553949314e-14	RPL7
Recoverec	13	4.22131968924765e-19	0.55313269847514[illegible]	0.881	0.792	1.41574619737988e-14	RPSA
Recoverec	13	5.58719990561967e-19	0.49673579096809[illegible]	0.902	0.835	1.87383510434673e-14	RPS25
Recoverec	13	7.10962316995694e-19	0.53802774737930[illegible]	0.648	0.426	2.38442541874016e-14	NOP53
Recoverec	13	9.77219785785739e-19	0.5106320984972	0.832	0.708	3.27739971756821e-14	MYL12A
Recoverec	13	1.31890698162714e-18	0.69121754567951[illegible]	0.661	0.414	4.42335023498111e-14	DUSP2
Recoverec	13	1.53638776759002e-18	0.53475756668450[illegible]	0.82	0.657	5.15273729494342e-14	SH3BGRL3
Recoverec	13	2.03254786178506e-18	0.54146222751180[illegible]	0.835	0.696	6.81675901885472e-14	EEF1D
Recoverec	13	3.84774081419442e-18	0.49722821360763[illegible]	0.899	0.83	1.29045531426452e-13	RPS13
Recoverec	13	4.80110776688232e-18	0.49977082700245[illegible]	0.988	0.967	1.61019552285699e-13	EEF1A1
Recoverec	13	5.88977876839054e-18	0.48347256227060[illegible]	0.902	0.832	1.97531400334282e-13	RPL15
Recoverec	13	6.62973056985344e-18	0.43542510715788[illegible]	0.813	0.629	2.22347903851745e-13	OAZ1
Recoverec	13	6.86169049990563e-18	0.39053642081814[illegible]	0.434	0.221	2.30127375985835e-13	EIF5A
Recoverec	13	7.02391230531593e-18	0.48357081634806[illegible]	0.676	0.44	2.35567970895686e-13	EIF3K
Recoverec	13	1.22052572410451e-17	0.47220320672009[illegible]	0.92	0.883	4.09339917350169e-13	RPS15A
Recoverec	13	1.27966602944751e-17	0.49248501780848[illegible]	0.804	0.617	4.29174392956107e-13	COX4I1
Recoverec	13	1.46580240587356e-17	0.48889536634189	0.924	0.891	4.91600810881874e-13	RPS18
Recoverec	13	1.86869315686561e-17	0.50768057175540[illegible]	0.691	0.449	6.26722310949589e-13	CORO1A
Recoverec	13	2.88855847885007e-17	0.40358130980658[illegible]	0.976	0.945	9.68764742636737e-13	SRGN
Recoverec	13	3.55372727097312e-17	0.41693814209713[illegible]	0.486	0.265	1.19184905213897e-12	LY6E
Recoverec	13	3.61731935743473e-17	0.6269908988732	0.578	0.346	1.21317656609646e-12	DDIT4
Recoverec	13	6.65524714525985e-17	0.41869827701060[illegible]	0.914	0.825	2.23203678757725e-12	UBC

Recoverec	13	7.82193899989788e-17	0.37351129454276	0.523	0.29	2.62332190178575e-12	EIF3G
Recoverec	13	1.15328208905964e-16	0.52975749929673	0.905	0.823	3.86787747028823e-12	RPL21
Recoverec	13	1.33386094925418e-16	0.49038400853668	0.716	0.506	4.47350285160868e-12	RARRES3
Recoverec	13	1.71375271590042e-16	0.4345319317005	0.838	0.69	5.74758385858681e-12	HNRNPA1
Recoverec	13	1.73435929492136e-16	0.47479101107354	0.908	0.859	5.81669420330726e-12	RPL32
Recoverec	13	4.75508662800875e-16	0.38046804555086	0.963	0.939	1.59476095330158e-11	PTMA
Recoverec	13	5.19841320232871e-16	0.39817001843821	0.654	0.421	1.743443819797e-11	PSMB9
Recoverec	13	7.80735326958107e-16	0.43771094567933	0.979	0.966	2.6184301395521e-11	TPT1
Recoverec	13	9.97574898392257e-16	0.41750080117380	0.749	0.539	3.34566669422795e-11	SLC25A6
Recoverec	13	1.17234908431864e-15	0.40362364426337	0.639	0.417	3.93182435898784e-11	EDF1
Recoverec	13	1.62874782760245e-15	0.41276121037646	0.927	0.872	5.4624944642131e-11	RPL12
Recoverec	13	1.94797770541587e-15	0.33423214132292	0.969	0.938	6.53312762842375e-11	EIF1
Recoverec	13	2.52959352638918e-15	0.46686259145756	0.936	0.883	8.48375076880402e-11	RPS8
Recoverec	13	3.67826123535925e-15	0.37973702011406	0.667	0.439	1.23361525311478e-10	COX5B
Recoverec	13	3.73508272616404e-15	0.37555616427929	0.514	0.301	1.2526720447009e-10	CCDC85B
Recoverec	13	4.57631368682396e-15	0.43882270275606	0.893	0.822	1.53480408428702e-10	RPL35A
Recoverec	13	5.06074887609355e-15	0.36519812560667	0.679	0.446	1.69727395806425e-10	SAP18
Recoverec	13	5.16958952500124e-15	0.44886529331912	0.899	0.868	1.73377693489492e-10	RPL9
Recoverec	13	8.45710419528111e-15	0.28976646448126	0.367	0.179	2.83634360501338e-10	TBC1D10C
Recoverec	13	1.01675025090117e-14	0.36158651707827	0.624	0.409	3.40997699147235e-10	GUK1
Recoverec	13	1.02927283939575e-14	0.79137204991427	0.627	0.416	3.45197524876547e-10	GADD45B
Recoverec	13	1.06340655479542e-14	0.47244231282686	0.688	0.485	3.56645290347287e-10	RAC2
Recoverec	13	1.33079284697464e-14	0.41728486587032	0.838	0.735	4.46321305018354e-10	PFDN5
Recoverec	13	1.37451357157112e-14	0.36088731896794	0.284	0.123	4.60984361633521e-10	LAG3
Recoverec	13	1.83796948775023e-14	0.34393673868878	0.477	0.269	6.16418206801673e-10	CHCHD10
Recoverec	13	2.23796025393474e-14	0.37872488847936	0.498	0.295	7.50567109964635e-10	PAXX
Recoverec	13	2.2774711623694e-14	0.38522751189708	0.572	0.356	7.63818278435449e-10	EID1
Recoverec	13	2.78481655447728e-14	0.38850939735530	0.404	0.215	9.3397177604059e-10	SOCS3
Recoverec	13	2.93708395186631e-14	0.42625482865671	0.869	0.772	9.85039215776924e-10	HLA-E
Recoverec	13	3.59835060721094e-14	0.36062485738009	0.437	0.241	1.2068148266464e-09	TAF10
Recoverec	13	5.05845814459171e-14	0.46742172277763	0.829	0.635	1.69650569253317e-09	IRF1

Recoverec	13	5.31237118861957e-14	0.349667349811214	0.572	0.355	1.78166304923923e-09	TPI1
Recoverec	13	9.22193721770996e-14	0.424948745902912	0.505	0.304	3.09285330407557e-09	EVI2B
Recoverec	13	9.34391901364167e-14	0.422006122441943	0.92	0.78	3.13376355879514e-09	BTG2
Recoverec	13	9.38354810836979e-14	0.423897390245595	0.471	0.282	3.14705436458506e-09	LIMD2
Recoverec	13	9.52836696273046e-14	0.29889810556196	0.391	0.201	3.19562371196054e-09	NDUFA12
Recoverec	13	9.83875464512156e-14	0.330358649458557	0.612	0.39	3.29972153288087e-09	APRT
Recoverec	13	1.05418597954485e-13	0.320325354545873	0.465	0.27	3.53552893819752e-09	C9orf16
Recoverec	13	1.13993790279244e-13	0.40960314765638	0.538	0.346	3.82312373838527e-09	GPSM3
Recoverec	13	1.22783089708438e-13	0.444900927848912	0.667	0.476	4.11789926264159e-09	GMFG
Recoverec	13	1.24401212198597e-13	0.498400372693169	0.694	0.498	4.17216785471653e-09	H2AFZ
Recoverec	13	1.67836245752341e-13	0.325595211275054	0.376	0.193	5.62889201004201e-09	TUBA4A
Recoverec	13	2.59128140275954e-13	0.316791711164234	0.22	0.088	8.69063956857493e-09	IER5L
Recoverec	13	2.79430441268561e-13	0.363981646108123	0.394	0.217	9.371538139265e-09	ARPC1B
Recoverec	13	3.05332503340648e-13	0.271378167372005	0.48	0.273	1.02402414970386e-08	VPS28
Recoverec	13	3.10385417888607e-13	0.367344112695647	0.911	0.879	1.04097061451481e-08	RPS24
Recoverec	13	4.76938398718785e-13	0.372713645930894	0.911	0.862	1.59955600162306e-08	RPS16
Recoverec	13	5.21812191705019e-13	0.35925953951304	0.755	0.565	1.75005372854029e-08	OST4
Recoverec	13	5.61361590987838e-13	0.407002362596351	0.792	0.644	1.88269450385501e-08	MYL12B
Recoverec	13	5.83102801513216e-13	0.350084073020283	0.859	0.755	1.95561017571502e-08	RPL27
Recoverec	13	7.47192797321857e-13	0.330932561922503	0.327	0.162	2.50593520365805e-08	DNAAF2
Recoverec	13	8.22325469290072e-13	0.448020874690354	0.817	0.702	2.75791515890504e-08	CD3D
Recoverec	13	9.52141008471087e-13	0.586520323618745	0.606	0.423	3.19329051421033e-08	NKG7
Recoverec	13	1.16510289807058e-12	0.420739623891919	0.682	0.491	3.90752209954912e-08	TERF2IP
Recoverec	13	1.29406717224826e-12	0.258812605833377	0.254	0.11	4.3400424822862e-08	PYCARD
Recoverec	13	1.44220988865543e-12	1.1191847229552	0.636	0.458	4.83688352457257e-08	CCL4
Recoverec	13	1.51627716196579e-12	0.277377112416744	0.287	0.132	5.08529034580088e-08	BAX
Recoverec	13	1.51732625301407e-12	0.3487122581074	0.489	0.3	5.0888087873586e-08	ARL6IP4
Recoverec	13	1.60681858406365e-12	0.356995288817715	0.755	0.576	5.38894816723265e-08	PPIB
Recoverec	13	1.69090985470146e-12	0.398675013890168	0.948	0.916	5.67097347069777e-08	ACTB
Recoverec	13	2.07879683697273e-12	0.365926271523442	0.982	0.942	6.97186883183915e-08	RPLP1
Recoverec	13	2.19083373543268e-12	0.365569422719973	0.774	0.596	7.34761818189413e-08	SRSF2

Recoverec	13	2.75812027968156e-12	0.46704174564506	0.621	0.416	9.25018379399602e-08	TUBA1B
Recoverec	13	3.05683737825903e-12	0.51567503185701‹	0.789	0.632	1.02520211992051e-07	ID2
Recoverec	13	3.07596344527391e-12	0.26320474127732‹	0.257	0.115	1.03161662027597e-07	TMEM238
Recoverec	13	3.32178837280669e-12	0.28865255680559	0.303	0.145	1.11406138447191e-07	PSMB8-AS1
Recoverec	13	4.39672712411581e-12	0.50263036583969‹	0.749	0.588	1.47457434288596e-07	TRAC
Recoverec	13	4.41515135418408e-12	0.50501449263353‹	0.853	0.742	1.48075346116626e-07	NFKBIA
Recoverec	13	4.82014818650494e-12	0.45073258066660‹	0.82	0.722	1.61658129879003e-07	CD2
Recoverec	13	5.01636883444515e-12	0.311198555305788	0.823	0.686	1.68238977969622e-07	HINT1
Recoverec	13	5.42302796003657e-12	0.27910565220031‹	0.309	0.152	1.81877511723706e-07	TSTD1
Recoverec	13	5.61598935786403e-12	0.34857558432381	0.294	0.144	1.88349051084044e-07	H2AFX
Recoverec	13	7.02292069694e-12	0.30708930317663‹	0.81	0.67	2.35534714333974e-07	ATP5MG
Recoverec	13	7.34282691495845e-12	0.28677469148890‹	0.364	0.192	2.46263729073877e-07	DCXR
Recoverec	13	8.54437920562913e-12	0.45308992149975‹	0.492	0.298	2.8656138979839e-07	GZMA
Recoverec	13	8.54950926702527e-12	0.32743745555672‹	0.505	0.312	2.86733441797494e-07	COX6A1
Recoverec	13	8.93313925290141e-12	0.601113629737984	0.939	0.879	2.99599624263808e-07	JUN
Recoverec	13	1.02474523904581e-11	0.40498699120947‹	0.927	0.889	3.43679058271184e-07	RPS6
Recoverec	13	1.05136591390729e-11	0.26005066429337‹	0.358	0.186	3.52607100206227e-07	CCDC107
Recoverec	13	1.05541147112039e-11	0.33663156295336	0.875	0.804	3.53963899184356e-07	UBA52
Recoverec	13	1.09326561704622e-11	0.33797608767349‹	0.398	0.224	3.66659422644961e-07	NSMCE3
Recoverec	13	1.10898255483079e-11	0.31627543466074‹	0.431	0.252	3.7193056923915e-07	JAK3
Recoverec	13	1.15621329979528e-11	0.356316041191964	0.737	0.551	3.87770816485342e-07	HCST
Recoverec	13	1.30225592259246e-11	0.27173402541664‹	0.532	0.326	4.36750591319058e-07	ATP5MC3
Recoverec	13	1.31908359844316e-11	0.26349028142706‹	0.661	0.458	4.42394257245868e-07	RBM8A
Recoverec	13	1.35835844492881e-11	0.32900323212920‹	0.569	0.375	4.55566255260225e-07	SNRPB
Recoverec	13	1.4330803748212e-11	0.41780322102182‹	0.535	0.356	4.80626496107533e-07	LGALS3
Recoverec	13	1.49400109361145e-11	0.35125310594835‹	0.547	0.354	5.01058086775407e-07	C19orf53
Recoverec	13	1.55370853083882e-11	0.25714877282320‹	0.306	0.153	5.21082767072722e-07	PSMA2
Recoverec	13	1.58424816728016e-11	0.50326902308833‹	0.905	0.844	5.3132515034242e-07	CCL5
Recoverec	13	1.59766786170215e-11	0.32929505826307‹	0.963	0.918	5.35825847457667e-07	FTH1
Recoverec	13	1.9392638481629e-11	0.33489519718854‹	0.96	0.927	6.50390309396873e-07	BTG1
Recoverec	13	2.03078065375704e-11	0.3156793111184	0.361	0.202	6.81083215657036e-07	VAMP2

Recoverec	13	2.06265114374467e-11	0.445060268286071	0.456	0.283	6.91771940589086e-07	GIMAP7
Recoverec	13	2.52643708508326e-11	0.374209250715947	0.92	0.84	8.47316469595223e-07	ACTG1
Recoverec	13	2.54403380051506e-11	0.332568504808642	0.661	0.476	8.5321805601674e-07	PSMA7
Recoverec	13	2.68373977830639e-11	0.341471150189553	0.685	0.528	9.00072646848396e-07	ATP5MC2
Recoverec	13	3.27026766226376e-11	0.32846359845036	0.434	0.262	1.09678236857002e-06	PSMB8
Recoverec	13	3.47467898046628e-11	0.352116414966312	0.578	0.391	1.16533783646878e-06	SSR2
Recoverec	13	3.55176941685581e-11	0.354421171649517	0.89	0.769	1.1911924270251e-06	PFN1
Recoverec	13	3.75190600755733e-11	0.272650560127323	0.3	0.15	1.25831423681458e-06	FAM89B
Recoverec	13	4.17711860845916e-11	0.364533006459287	0.547	0.358	1.40092203890503e-06	ERP29
Recoverec	13	4.28289226465338e-11	0.272616524241859	0.315	0.165	1.43639640771945e-06	SNRPN
Recoverec	13	4.57731214933106e-11	0.324001312294798	0.731	0.543	1.53513894864265e-06	ARPC3
Recoverec	13	5.28349458922064e-11	0.266262269457503	0.56	0.365	1.77197841533282e-06	SPCS1
Recoverec	13	5.97826487550017e-11	0.3155172300427	0.554	0.365	2.00499047394525e-06	LAPTM5
Recoverec	13	7.81716680115607e-11	0.297052806370802	0.651	0.455	2.62172140177172e-06	UQCRH
Recoverec	13	9.57573999972109e-11	0.271493317159285	0.474	0.293	3.21151168110646e-06	C6orf48
Recoverec	13	9.60010801554473e-11	0.322850848194	0.917	0.852	3.21968422625339e-06	RPS20
Recoverec	13	1.19549027445329e-10	0.313812271627868	0.508	0.322	4.00943528246143e-06	GSTP1
Recoverec	13	1.21785480878691e-10	0.412654181864092	0.85	0.758	4.08444145770954e-06	IL32
Recoverec	13	1.2250007320686e-10	0.512901639421576	0.801	0.671	4.10840745521168e-06	CXCR4
Recoverec	13	1.32844893181548e-10	0.371041282135219	0.404	0.236	4.45535202752276e-06	LYAR
Recoverec	13	1.39113642228274e-10	0.257615771161736	0.37	0.208	4.66559333305185e-06	NDUFB10
Recoverec	13	1.42573844785705e-10	0.336761679485571	0.887	0.779	4.78164160642298e-06	RPL22
Recoverec	13	1.4890650939173e-10	0.348640712919592	0.636	0.457	4.99402651197985e-06	IFITM2
Recoverec	13	1.53763117413842e-10	0.258842486224345	0.394	0.226	5.15690743182545e-06	SNHG7
Recoverec	13	1.69647782659114e-10	0.36863064961436	0.514	0.338	5.68964733482136e-06	NSA2
Recoverec	13	1.74078426657864e-10	0.384023821786168	0.795	0.661	5.83824227325144e-06	CD48
Recoverec	13	1.74386116965461e-10	0.34646935913069	0.685	0.512	5.84856159078763e-06	CKLF
Recoverec	13	1.85777541892429e-10	0.32286404088758	0.453	0.284	6.23060719998827e-06	UBALD2
Recoverec	13	2.85138746506809e-10	0.315886459251605	0.557	0.377	9.56298328034537e-06	CBX3
Recoverec	13	2.86568531753e-10	0.39283332058222	0.569	0.401	9.61093541793211e-06	H1FX
Recoverec	13	2.9591142788307e-10	0.367051439585901	0.514	0.339	9.9242774683424e-06	TUBB4B

Recoverec	13	3.05946254031844e-10	0.26599251794612	0.361	0.203	1.026082546772e-05	SCAND1
Recoverec	13	3.29693209097037e-10	0.280843940853067	0.557	0.367	1.10572508466964e-05	UXT
Recoverec	13	4.19476969161048e-10	0.3248585104741	0.667	0.491	1.40684185917232e-05	SEC61B
Recoverec	13	4.89085138887238e-10	0.29770911695738З	0.902	0.824	1.64029373880002e-05	H3F3A
Recoverec	13	5.45136612956915e-10	0.32817764362369ξ	0.587	0.413	1.8282791725349e-05	EIF3F
Recoverec	13	5.98145479528622e-10	0.32971399131003	0.355	0.204	2.00606030924309e-05	GBP5
Recoverec	13	8.33096143862433e-10	0.27293767589045ε	0.905	0.827	2.79403784728583e-05	RPL35
Recoverec	13	8.53743376524213e-10	0.27914033799495ž	0.138	0.05	2.86328453618691e-05	GZMH
Recoverec	13	9.47648944349269e-10	0.32557822343853ɕ	0.339	0.193	3.17822502955858e-05	GABARAPL2
Recoverec	13	9.67937238345023e-10	0.36187273720335ε	0.774	0.672	3.24626790996154e-05	CD3E
Recoverec	13	1.20443410470135e-09	0.34251542168771	0.826	0.715	4.03943110034738e-05	YBX1
Recoverec	13	1.43793541925309e-09	0.45709489651699	0.838	0.714	4.82254780909103e-05	TXNIP
Recoverec	13	1.50883235805148e-09	0.26886607393228ž	0.364	0.212	5.06032196243307e-05	NXT1
Recoverec	13	1.55248115007903e-09	0.38703772797196ɕ	0.927	0.853	5.20671128113505e-05	ZFP36L2
Recoverec	13	1.68718754092774e-09	0.27241524624030ʴ	0.483	0.316	5.65848957476346e-05	UBXN1
Recoverec	13	1.72340559735292e-09	0.32324207969439ε	0.679	0.505	5.77995769240221e-05	CD37
Recoverec	13	1.86290009854961e-09	0.32548249805735	0.859	0.784	6.24779435051569e-05	CFL1
Recoverec	13	2.26740977229974e-09	0.29850581766878	0.826	0.721	7.60443889433888e-05	S100A6
Recoverec	13	2.47359554126869e-09	0.25602193288159	0.538	0.354	8.29594472630693e-05	CD81
Recoverec	13	2.49854802626287e-09	0.30842325430018	0.575	0.389	8.37963037048042e-05	ITM2A
Recoverec	13	2.75029679733872e-09	0.25062504708272ε	0.388	0.235	9.22394539891458e-05	REX1BD
Recoverec	13	3.41853954272024e-09	0.29780806912416З	0.511	0.347	0.000114650979183751	SLC25A5
Recoverec	13	3.60932610485294e-09	0.45554903051664З	0.774	0.636	0.000121049578904558	TRBC2
Recoverec	13	3.6863305944442e-09	0.26521467830325	0.578	0.403	0.00012363215547647	ABRACL
Recoverec	13	3.69593271257943e-09	0.28495886070978	0.621	0.449	0.000123954191314489	AES
Recoverec	13	4.11657945246458e-09	0.25772020369797ɕ	0.676	0.5	0.000138061841676757	SSR4
Recoverec	13	4.22623278082638e-09	0.26336002866303ʴ	0.657	0.478	0.000141739395003355	DAZAP2
Recoverec	13	4.28827156682330e-09	0.27593957990243З	0.581	0.403	0.00014382005180812	C12orf57
Recoverec	13	4.33670092800389e-09	0.25819078031060З	0.434	0.274	0.000145444275723395	NDUFB11
Recoverec	13	4.92952948419616e-09	0.30973757471081	0.486	0.322	0.000165326559840971	RSL1D1
Recoverec	13	6.27091483274986e-09	0.25617668120991З	0.284	0.156	0.000210313941660765	NDUFB5

Recoverec	13	7.6706810469021e-09	0.27666487139507	0.398	0.239	0.000257259300951003	GZMM
Recoverec	13	8.38132194581682e-09	0.26518662840663	0.752	0.596	0.000281092775418804	CHCHD2
Recoverec	13	8.732319843427e-09	0.353411871803692	0.884	0.803	0.000292864542908855	HMGB1
Recoverec	13	8.86460420246756e-09	0.29258004104494	0.52	0.361	0.000297301095742357	PARK7
Recoverec	13	9.31238106442371e-09	0.31445351583435	0.431	0.278	0.000312318636138642	SBDS
Recoverec	13	1.01210127125085e-08	0.594614911405621	0.425	0.278	0.00033943852435211	AREG
Recoverec	13	1.08519771847837e-08	0.30896358631439	0.394	0.247	0.000363953610823275	TMBIM4
Recoverec	13	1.17517409810945e-08	0.2909817015425	0.547	0.393	0.000394129889023948	BUB3
Recoverec	13	1.29638122086712e-08	0.35487866291611	0.706	0.546	0.000434780333854416	HMGN2
Recoverec	13	1.44803686658621e-08	0.26857871805813	0.82	0.679	0.000485642604315685	COX7C
Recoverec	13	1.78042168014219e-08	0.28431561207568	0.657	0.496	0.000597117823086089	PTP4A2
Recoverec	13	2.31522458628357e-08	0.27210214273655	0.523	0.361	0.000776480021747783	HNRNPA0
Recoverec	13	2.41339790046963e-08	0.2603439195331	0.477	0.319	0.000809405387859504	HIGD2A
Recoverec	13	2.41944975465897e-08	0.37622175301789	0.566	0.404	0.000811435058717524	CST7
Recoverec	13	2.68768793840758e-08	0.26975910558464	0.352	0.215	0.000901396780783135	MDH2
Recoverec	13	2.71209880022007e-08	0.31791387500170	0.758	0.625	0.000909583695617807	RPS4Y1
Recoverec	13	2.72548029132047e-08	0.25323764494347	0.303	0.169	0.000914071580103059	TBCC
Recoverec	13	2.99078921299543e-08	0.33954386925633	0.749	0.616	0.00100305088625441	CALM2
Recoverec	13	3.04183260015867e-08	0.28685032467086	0.563	0.399	0.00102016981744122	SNHG8
Recoverec	13	3.17376522477737e-08	0.25258276816089	0.667	0.492	0.00106441738108583	HMGN1
Recoverec	13	3.18122904300935e-08	0.27313181661419	0.364	0.226	0.00106692059644448	ARL6IP1
Recoverec	13	3.42306008327085e-08	0.37398492695417	0.517	0.371	0.00114802589072738	UBE2S
Recoverec	13	3.42826740386719e-08	0.48277077976204	0.45	0.302	0.00114977232190898	HMGB2
Recoverec	13	3.85267913909987e-08	0.45090199545875	0.801	0.69	0.00129211152967131	ZFP36L1
Recoverec	13	4.0157646877823e-08	0.30343037219393	0.734	0.609	0.00134680716098843	CD3G
Recoverec	13	4.28172772414203e-08	0.28777994488641	0.388	0.251	0.00143600584412275	DUT
Recoverec	13	4.4008316148366e-08	0.29875694435285	0.453	0.306	0.0014759509069839	CUTA
Recoverec	13	4.42580531445672e-08	0.36794315749713	0.56	0.414	0.0014843265863625	ANP32B
Recoverec	13	4.856687076704e-08	0.25752450040604	0.933	0.884	0.00162883571178499	RPL34
Recoverec	13	5.20811832170936e-08	0.30141091491644	0.813	0.7	0.00174669872273489	RPL4
Recoverec	13	6.04089857652331e-08	0.26866387569805	0.453	0.305	0.00202599656459439	SF3B2

Recoverec	13	6.47868938094007e-08	0.26786124387044¦	0.422	0.283	0.00217282284457968	PSMD8
Recoverec	13	6.48948083968346e-08	0.42525374907569¿	0.761	0.625	0.00217644208401304	SQSTM1
Recoverec	13	6.60607453788576e-08	0.28250253828037ː	0.56	0.404	0.00221554527851613	MAP1LC3B
Recoverec	13	6.95705043872852e-08	0.39159261567951⁊	0.902	0.848	0.00233325557614077	DUSP1
Recoverec	13	7.33009908363647e-08	0.27858590034787	0.566	0.41	0.00245836863067	GSTK1
Recoverec	13	7.50179490040061e-08	0.28420494058764ꞁ	0.832	0.72	0.00251595197369636	RPL31
Recoverec	13	7.51098737536485e-08	0.34181138857301¦	0.774	0.652	0.00251903494594986	S100A4
Recoverec	13	7.82775247300667e-08	0.67414468768853⁊	0.162	0.075	0.00262527162439698	GZMK
Recoverec	13	1.10318328588352e-07	0.25007156082252ƚ	0.422	0.279	0.00369985610419616	DRAP1
Recoverec	13	1.13897970428416e-07	0.26818931369278ꞓ	0.688	0.537	0.0038199101322282	PNN
Recoverec	13	1.17383613450262e-07	0.31098631044284ƚ	0.826	0.718	0.00393681162789488	PPIA
Recoverec	13	1.33430171998653e-07	0.25477472588377ˈ	0.691	0.538	0.00447498110849081	ARL6IP5
Recoverec	13	1.41169949103261e-07	0.35795604510667¿	0.239	0.128	0.00473455775302516	TNFSF9
Recoverec	13	1.55709806376385e-07	0.29506845863559¦	0.853	0.697	0.0052221954862512	MTRNR2L12
Recoverec	13	1.64973965309468e-07	0.28984586714915¿	0.621	0.476	0.00553289684854893	RAN
Recoverec	13	1.69133120518779e-07	0.28271905328418ꞓ	0.235	0.13	0.0056723865959588	CD27
Recoverec	13	2.00385595054777e-07	0.29724816130684ꞁ	0.413	0.271	0.00672053208694712	HOPX
Recoverec	13	2.08995998977261e-07	0.2873089976571	0.474	0.332	0.00700930781369938	HERPUD1
Recoverec	13	2.1268812080199e-07	0.33345091884526	0.602	0.439	0.00713313419545715	IER5
Recoverec	13	2.66487617573597e-07	0.25923345298895¦	0.471	0.324	0.00893746171818331	FXYD5
Recoverec	13	3.13629122868543e-07	0.25463074317561ꞓ	0.547	0.407	0.0105184935227652	S100A11
Recoverec	13	3.26216036600626e-07	0.27382804416247¦	0.872	0.785	0.0109406334355118	ITM2B
Recoverec	13	3.33477179362679e-07	0.34957813546580ː	0.731	0.623	0.0111841576414655	ARL4C
Recoverec	13	3.36100763051935e-07	0.26373891701191ꞓ	0.67	0.514	0.0112721473912358	TRIR
Recoverec	13	3.80297529172091e-07	0.29833761899122ϵ	0.443	0.305	0.0127544185333736	MIDN
Recoverec	13	3.94633090483599e-07	0.27321983946203ꞓ	0.648	0.502	0.0132352045886389	LBH
Recoverec	13	5.53305163919118e-07	0.39053698925482ꞁ	0.743	0.605	0.0185567485875194	IER2
Recoverec	13	5.6126456301598e-07	0.25113810428972ϵ	0.502	0.367	0.0188236909144299	TMEM50A
Recoverec	13	5.81768027273984e-07	0.35972240453820¦	0.963	0.955	0.0195113360987149	FOS
Recoverec	13	6.29185307418159e-07	0.26287328933920ƚ	0.645	0.497	0.0211016168401902	PSME1
Recoverec	13	1.30130932251324e-06	0.28080686048696ː	0.67	0.532	0.0436433120584491	YWHAB

Recoverec	13	1.43452831932075e-06	0.28254195431828	0.599	0.458	0.0481112107733795	KHDRBS1
Recoverec	13	1.43742774724332e-06	0.25970276615285	0.719	0.566	0.0482084517870466	GCC2
Recoverec	20	2.26784402630317e-46	1.61676277069672	0.508	0.169	7.60589529541558e-42	AC105402.3
Recoverec	20	2.40647888542973e-33	0.56416815588103	0.723	0.351	8.07084888595422e-29	OLFM4
Recoverec	20	9.65488250168161e-25	0.82376389976034	0.926	0.686	3.23805449341398e-20	MTRNR2L12
Recoverec	20	5.25152040843785e-24	0.58582107666717	1	0.989	1.76125491458189e-19	MT-ND1
Recoverec	20	2.97479392319027e-22	0.25542217922265	0.818	0.489	9.97686385959552e-18	DEFA5
Recoverec	20	4.29677223090309e-20	0.52068569277635	1	1	1.44105147080028e-15	MT-CO3
Recoverec	20	2.08408117817846e-19	0.57430128270347	0.32	0.127	6.9895914553749e-15	XIST
Recoverec	20	2.91486079172058e-18	0.25004111829128	0.794	0.489	9.77586012327248e-14	REG1A
Recoverec	20	1.11822558588966e-17	0.29052626363977	1	0.994	3.75030496995673e-13	MT-ND3
Recoverec	20	6.7784304372794e-17	0.32825323377745	0.886	0.603	2.27335000005477e-12	MT1G
Recoverec	20	4.37900776774512e-16	0.98589658185815	0.702	0.482	1.46863162514636e-11	MTRNR2L8
Recoverec	20	3.76104319793965e-15	0.28326589757666	0.32	0.139	1.261378667725e-10	DACH1
Recoverec	20	5.4987925460585e-15	0.3239988224005	0.406	0.198	1.8441850440971e-10	CERS6
Recoverec	20	1.72574711577762e-13	0.58287220840881	0.775	0.563	5.78781067689500e-09	VPS37B
Recoverec	20	2.19478759672154e-11	0.43884135096970	1	0.998	7.36087864188471e-07	MT-ND4
Recoverec	20	2.61621473578102e-11	0.64330911573980	0.895	0.732	8.77426098086238e-07	MT-ND4L
Recoverec	20	2.63110939464093e-11	0.36650623924821	0.926	0.75	8.82421468774674e-07	RFX3
Recoverec	20	2.84765134652425e-11	0.41284775136183	0.726	0.537	9.55045308597302e-07	ATM
Recoverec	20	7.32907367827122e-11	0.87391793462920	0.117	0.036	2.4580247302186e-06	NELL2
Recoverec	20	1.0510092760264e-10	0.33066732911513	0.434	0.249	3.52487490993735e-06	CA5B
Recoverec	20	2.44774902393501e-10	0.57363002860100	0.797	0.596	8.20926067647323e-06	RCOR1
Recoverec	20	4.34597467816483e-10	0.26199442629373	0.532	0.328	1.45755298756292e-05	PCNX4
Recoverec	20	9.6472575565432e-10	0.29018658927262	0.394	0.221	3.23549723931346e-05	AP3M2
Recoverec	20	1.12960112067689e-09	0.52541356000338	0.72	0.537	3.78845623852616e-05	BORCS5
Recoverec	20	1.17364693325943e-09	0.34764121148401	0.542	0.341	3.93617708476548e-05	ZSWIM4
Recoverec	20	3.29636856603545e-09	0.30144261170685	0.178	0.077	0.000110553608967697	RASA3
Recoverec	20	3.50052132917001e-09	0.32767388056679	0.898	0.739	0.000117400484337704	HIVEP2
Recoverec	20	4.36827443460095e-09	0.29762804512040	0.68	0.484	0.000146503187987647	PRKAR2A
Recoverec	20	6.11461874351274e-09	0.26575315701532	0.978	0.917	0.00020507208341993	PPP2R5C

Recovered	20	7.2278292636665e-09	0.345806175949949	0.723	0.537	0.000242406937844847	SP140
Recovered	20	1.58277621934225e-08	0.353551490360489	1	1	0.000530831488443005	MT-ATP6
Recovered	20	1.84647133876985e-08	0.337039281432738	0.585	0.405	0.000619269557596632	PRKCE
Recovered	20	2.59061746778635e-08	0.258616566037042	0.56	0.366	0.000868841286346186	HSD17B12
Recovered	20	2.65401891367166e-08	0.287055293120369	0.634	0.444	0.000890104863267202	PRMT9
Recovered	20	2.88119425644361e-08	0.299300450204717	0.203	0.097	0.000966294929726057	SVIL
Recovered	20	2.89514130222868e-08	0.275377347346893	0.483	0.31	0.000970972489941456	SORL1
Recovered	20	4.52801208608392e-08	0.445450899940242	0.788	0.651	0.00151860469343083	NR4A3
Recovered	20	6.12864109156715e-08	0.273992247442102	0.708	0.52	0.00205542364928979	BIRC3
Recovered	20	6.98349777774758e-08	0.34012256883684	0.28	0.154	0.00234212548470098	KLF2
Recovered	20	8.58801831674218e-08	0.306750407153838	0.317	0.187	0.00288024958306899	TMCC3
Recovered	20	9.53262215928239e-08	0.273594451534228	0.997	0.972	0.00319705081978013	FYN
Recovered	20	1.51584603422111e-07	0.288702363867895	0.228	0.118	0.00508384442957077	SAMD12
Recovered	20	2.50331037003353e-07	0.367113213855875	0.877	0.724	0.00839560231901844	CYTH1
Recovered	20	2.66696704188042e-07	0.262073706415085	0.929	0.791	0.00894447406505856	CHD2
Recovered	20	3.26487165062461e-07	0.276944084973868	0.825	0.631	0.0109497265418648	USP9X
Recovered	20	3.36566499805064e-07	0.406898441376624	0.785	0.637	0.0112877672704622	CEMIP2
Recovered	20	3.92166039935872e-07	0.336537795933647	0.757	0.576	0.0131524646473693	DUSP16
Recovered	20	3.99476415473949e-07	0.261160070905865	0.825	0.647	0.0133976400221653	STAT3
Recovered	20	6.41454825277121e-07	0.251594378701624	0.902	0.774	0.0215131119301441	CREM
Recovered	20	7.66437939981661e-07	0.257756298227428	0.431	0.281	0.025704795631105	ZFX
Recovered	20	8.98397490112313e-07	0.458372415547862	1	0.979	0.0301304550233868	MT-ND5
Recovered	20	1.40754642054833e-06	0.29791304124688	0.48	0.338	0.04720629185235	CD28
Recovered	26	2.49302615046949e-28	1.2284518565398	0.974	0.94	8.36111110344458e-24	HSP90AA1
Recovered	26	8.54131674303566e-26	1.52768885315527	0.75	0.456	2.8645868092793e-21	HSPA1A
Recovered	26	8.95996126373168e-25	0.676647396633186	0.218	0.038	3.00499180863033e-20	C1QB
Recovered	26	5.44875123138851e-24	1.10208909842057	0.801	0.511	1.82740218798308e-19	REG1A
Recovered	26	1.58268872225751e-21	1.15947837858918	0.718	0.432	5.30802143670723e-17	HSPA1B
Recovered	26	5.33083021136543e-21	0.351330366682531	0.109	0.01	1.78785383628774e-16	SPINK4
Recovered	26	1.58020691394769e-17	0.686594732992625	0.865	0.626	5.29969794799778e-13	MT1G
Recovered	26	1.21416156380221e-16	0.791610016969813	0.705	0.488	4.07205505267986e-12	MT2A

Recoverec	26	4.72209183762909e-16	0.89537103725010З	0.891	0.879	1.58369516050405e-11	HSP90AB1
Recoverec	26	8.15301770389502e-16	0.68624146218090	0.929	0.764	2.73435907753231e-11	IGKC
Recoverec	26	1.69188903270708e-15	1.09293233567833	0.801	0.702	5.674257437893e-11	DNAJB1
Recoverec	26	4.31312376478963e-14	0.62256798017198	0.917	0.88	1.44653544823514e-09	FTL
Recoverec	26	3.16016379317965e-12	0.67791274486699	0.936	0.89	1.05985573295659e-07	TMSB10
Recoverec	26	1.4226136881668e-11	0.75965955456906	0.795	0.633	4.77116178737382e-07	HSPH1
Recoverec	26	2.25139694338157e-11	0.89676632370664	0.442	0.242	7.55073506871312e-07	IGLC3
Recoverec	26	2.98312796108187e-11	0.64760204923581	0.917	0.907	1.00048145558764e-06	RPL41
Recoverec	26	1.00369732625926e-10	0.57419044176676	0.833	0.729	3.36620009280832e-06	S100A6
Recoverec	26	8.36631924859006e-10	0.70358970940948	0.75	0.639	2.80589614959214e-05	HSPD1
Recoverec	26	1.15786788100043e-09	0.62841958021439	0.769	0.709	3.88325729929926e-05	S100A10
Recoverec	26	2.82871812950311e-09	0.75745638104545	0.314	0.16	9.48695486272755e-05	AGR2
Recoverec	26	3.53596696191127e-09	0.57755114308394	0.885	0.888	0.00011858925996858	RPS15A
Recoverec	26	3.73593600386302e-09	0.54991641639956	0.833	0.782	0.000125295821697558	PFN1
Recoverec	26	4.63018584670264e-09	0.60322590918277	0.744	0.685	0.000155287172926713	ATP5MG
Recoverec	26	5.07041694956293e-09	0.54591799785904	0.872	0.85	0.000170051643654442	ACTG1
Recoverec	26	7.27986150995956e-09	0.63994880255584	0.853	0.857	0.000244151995321024	RPS15
Recoverec	26	9.34897910254225e-09	0.59401390908308	0.821	0.849	0.000313546061141062	RPL8
Recoverec	26	9.90424512459195e-09	0.76777877673508	0.628	0.545	0.000332168572988565	HSPE1
Recoverec	26	1.2577363689373e-08	0.43397885055742	0.974	0.981	0.000421819623414191	B2M
Recoverec	26	1.3167684095348e-08	0.64149928478870	0.487	0.321	0.00044161778918978	MT1H
Recoverec	26	3.70807637636775e-08	0.47062361567548	0.904	0.899	0.00124361465510621	DNAJA1
Recoverec	26	4.48919491453718e-08	0.58332813426790	0.821	0.813	0.00150558619043748	UBA52
Recoverec	26	4.50896911878691e-08	0.54359881080417	0.962	0.921	0.00151221806305875	FTH1
Recoverec	26	5.96704131713978e-08	0.51003402325475	0.872	0.852	0.00200122631694234	RPS9
Recoverec	26	7.5606220960678e-08	0.53460770224032	0.84	0.834	0.00253568143857922	RPL21
Recoverec	26	7.90866085591288e-08	0.55731857076441	0.853	0.874	0.00265240667785606	RPS23
Recoverec	26	8.07546622851859e-08	0.25600049068773	0.128	0.038	0.00270834986372057	C1QA
Recoverec	26	8.53125180476668e-08	0.570509111761506	0.821	0.795	0.00286121123028265	ITM2B
Recoverec	26	9.70073478321557e-08	0.48533717632021	0.436	0.285	0.00325343243159484	MT1X
Recoverec	26	9.78380703353364e-08	0.51995241257568	0.917	0.906	0.00328129320290651	RPS19

Recoverec	26	1.05450335037214e-07	0.51658020941725	0.763	0.7	0.00353659333647809	HINT1
Recoverec	26	1.07364043342162e-07	0.59028873487338	0.795	0.766	0.00360077528560944	RPS5
Recoverec	26	1.10543485133452e-07	0.48797549977309	0.827	0.838	0.0037074074044057	RPL35
Recoverec	26	1.22431035944721e-07	0.50878156382708	0.788	0.783	0.00410609208351405	SERF2
Recoverec	26	1.5335931830417e-07	0.55448710742418	0.763	0.722	0.00514336481728524	MYL12A
Recoverec	26	1.7651167518584e-07	0.51048152603060	0.904	0.893	0.00591984856238269	RPL11
Recoverec	26	1.82227715569205e-07	0.54356976973826	0.942	0.948	0.00611155312475999	RPLP1
Recoverec	26	1.84317770145215e-07	0.56591315883926	0.596	0.488	0.00618164937513022	RAN
Recoverec	26	2.63657990909527e-07	0.60301836352328	0.744	0.763	0.00884256169912371	UBB
Recoverec	26	2.64263885066682e-07	0.52781725891792	0.487	0.354	0.00886288217736638	ELOB
Recoverec	26	2.85244677376389e-07	0.45712855261630	0.974	0.967	0.00956653598984934	TPT1
Recoverec	26	2.8840530823224e-07	0.47989621598222	0.853	0.883	0.00967253722749288	RPS28
Recoverec	26	3.30749969468849e-07	0.49309174942748	0.904	0.912	0.0110926924760463	RPL39
Recoverec	26	3.45378024727174e-07	0.41047732828058	0.885	0.871	0.0115832881933	RPL37
Recoverec	26	3.75819184577823e-07	0.52537045106086	0.564	0.47	0.012604223812371	COX6B1
Recoverec	26	3.95561010899958e-07	0.52865495470980	0.776	0.802	0.0132663251835628	FAU
Recoverec	26	5.22592044471601e-07	0.47643058420628	0.846	0.854	0.0175266919874886	RPL18
Recoverec	26	6.08663529524892e-07	0.50029872429478	0.859	0.881	0.0204133574532058	RPL12
Recoverec	26	6.83682660412726e-07	0.42370443897574	0.917	0.927	0.022929349064922	HLA-B
Recoverec	26	6.97924299595021e-07	0.43487603397192	0.795	0.767	0.0234069851598178	PTGES3
Recoverec	26	8.39533629946638e-07	0.49485668253717	0.904	0.889	0.0281562788811503	RPS8
Recoverec	26	8.79213458976896e-07	0.47058042892889	0.878	0.866	0.0294870609871671	RPS4X
Recoverec	26	9.06596222087406e-07	0.53214513956037	0.917	0.907	0.0304054240963674	RPS12
Recoverec	26	9.53166818448377e-07	0.58210024556828	0.885	0.892	0.0319673087571217	RPS14
Recoverec	26	1.05197821799018e-06	0.45077919884876	0.897	0.877	0.0352812454749545	RPL30
Recoverec	26	1.18209439620516e-06	0.42799717935643	0.865	0.895	0.0396450818599286	RPL28
Recoverec	33	1.4767939411896e-30	0.56345642878354	0.977	0.957	4.95287151996167e-26	STAT4
Recoverec	33	2.1536873640127e-26	0.63010612855882	0.905	0.836	7.22303668142579e-22	TANK
Recoverec	33	3.86294744747402e-25	0.63124722648818	0.644	0.482	1.29555531493384e-20	PPP1R15B
Recoverec	33	1.55520047072095e-23	0.48056610227093	0.967	0.937	5.21583133870393e-19	TNFAIP3
Recoverec	33	2.38987783199534e-22	0.56604304768737	0.964	0.949	8.01517227294598e-18	CD69

Recoverec	33	3.00693390982529e-21	0.68086972940437!	0.469	0.288	1.00846549467721e-16	IL2RA
Recoverec	33	7.69449810044439e-19	0.40134002965775	0.974	0.937	2.58058077292704e-14	DENND4A
Recoverec	33	2.11658499636376e-18	0.46242320529665:	0.812	0.705	7.09860276080477e-14	ZFP36
Recoverec	33	3.54781443445716e-18	0.362803598716061	0.908	0.83	1.18986600502824e-13	GPBP1
Recoverec	33	2.426834674471e-17	0.52258273174629!	0.844	0.73	8.13911813124084e-13	NFKBIA
Recoverec	33	1.76245668858065e-16	0.532461002072827	0.83	0.748	5.91092724216177e-12	CDC14A
Recoverec	33	2.13331950480584e-16	0.483652154085594	0.286	0.146	7.15472695521782e-12	AC009299.3
Recoverec	33	2.70783411557433e-16	0.53466988518423:	0.844	0.776	9.08153405681318e-12	NFKB1
Recoverec	33	4.02540534939806e-16	0.501880115530243	0.852	0.749	1.35004044608112e-11	RFX3
Recoverec	33	6.42206505595922e-16	0.574328039189648	0.858	0.781	2.1538321784676e-11	BTG2
Recoverec	33	5.24634794316982e-15	0.518144371693725	0.561	0.416	1.7595201731803e-10	ZNF267
Recoverec	33	7.65982950421758e-15	0.280194786981285	0.99	0.99	2.56895361912449e-10	PTPRC
Recoverec	33	8.54127322898782e-15	0.468869278882353	0.698	0.543	2.86457221553794e-10	AC009313.1
Recoverec	33	1.07950588710925e-14	0.33659803853873	0.28	0.146	3.620446844187e-10	TIMP1
Recoverec	33	3.02993310447389e-14	0.509097220238586	0.589	0.455	1.01617896457845e-09	GPR65
Recoverec	33	3.47229475287715e-14	0.429219646245692	0.882	0.816	1.16453821421994e-09	PPP1R15A
Recoverec	33	7.67871768105253e-14	0.380928846831607	0.958	0.923	2.5752883358714e-09	FOSB
Recoverec	33	1.42541189611488e-13	0.301044362147992	0.964	0.916	4.78054641719007e-09	RAB11A
Recoverec	33	9.91274144069185e-13	0.390225149871418	0.861	0.769	3.32453522437923e-08	CLEC2D
Recoverec	33	1.6406351907576e-12	0.50257783675742!	0.698	0.612	5.50236230276285e-08	PDE4B
Recoverec	33	2.35388237737941e-12	0.57717085976938	0.653	0.546	7.89445071725507e-08	TRAF1
Recoverec	33	2.5974392110932e-12	0.378381567165778	0.854	0.806	8.71129162616439e-08	CYTIP
Recoverec	33	2.07948759638661e-11	0.366607811936805	0.896	0.868	6.97418550076142e-07	TSC22D3
Recoverec	33	2.43731574242757e-11	0.36571613675741	0.795	0.714	8.17426953695357e-07	GOLGB1
Recoverec	33	2.81096428434753e-11	0.427587160798804	0.868	0.807	9.42741201684475e-07	ANKRD28
Recoverec	33	1.04398480390594e-10	0.360947600667801	0.767	0.685	3.50131623533975e-06	TIPARP
Recoverec	33	1.34117859898192e-10	0.30464873150778:	0.941	0.865	4.49804478526557e-06	THEMIS
Recoverec	33	1.43199105017283e-10	0.3233451680339	0.896	0.831	4.80261158406964e-06	CD44
Recoverec	33	1.68303617595576e-10	0.253401038632624	0.925	0.86	5.64456672692043e-06	PRRC2C
Recoverec	33	2.06617136506679e-10	0.29031841333832:	0.83	0.754	6.92952552416099e-06	NCL
Recoverec	33	2.48281581275791e-10	0.48901493006662:	0.262	0.153	8.32686767282747e-06	AL589990.1

Recoverec	33	3.07023341693968e-10	0.26869164367345	0.97	0.944	1.02969488337323e-05	CD96
Recoverec	33	3.79492876972536e-10	0.43235834659652	0.894	0.817	1.27274321079049e-05	RALGAPA1
Recoverec	33	4.84459372540643e-10	0.33486159033364	0.733	0.655	1.62477984362681e-05	MYADM
Recoverec	33	8.24559725033599e-10	0.29882327604298	0.906	0.863	2.76540840581769e-05	MCL1
Recoverec	33	9.46898345321495e-10	0.54216006121241	0.889	0.818	3.17570767053923e-05	BRAF
Recoverec	33	1.09451863731147e-09	0.33983514572259	0.832	0.764	3.67079660581521e-05	LCP1
Recoverec	33	1.44263221183697e-09	0.36295292596804	0.844	0.789	4.83829991205885e-05	VIM
Recoverec	33	1.5050602877101e-09	0.28981153304509	0.87	0.792	5.04767119292212e-05	USP15
Recoverec	33	1.53686735412768e-09	0.29595738657743	0.806	0.719	5.1543457322734e-05	MORF4L2
Recoverec	33	1.63631339811218e-09	0.34854583064268	0.812	0.744	5.48786787458864e-05	HIVEP2
Recoverec	33	1.65895821179855e-09	0.35519305572334	0.852	0.796	5.56381405072997e-05	PDE4D
Recoverec	33	1.97908615655662e-09	0.28228268654317	0.941	0.899	6.63745915185961e-05	IL7R
Recoverec	33	2.40221569637194e-09	0.34527797791846	0.519	0.431	8.05655100249221e-05	CDC37
Recoverec	33	2.41855339530872e-09	0.36815469081662	0.974	0.95	8.11134437718639e-05	FOS
Recoverec	33	2.62942036143001e-09	0.29191186515577	0.953	0.928	8.81855000816396e-05	GNG2
Recoverec	33	3.17503694528907e-09	0.28929588180102	0.741	0.654	0.000106484389071105	BAZ1A
Recoverec	33	3.36302925224148e-09	0.33411931079277	0.811	0.727	0.000112789275061675	IVNS1ABP
Recoverec	33	1.74736011737829e-08	0.32244220563803	0.823	0.759	0.000586029636166331	NFKBIZ
Recoverec	33	2.34139619359169e-08	0.40582993509783	0.564	0.476	0.000785257455406779	ARL5B
Recoverec	33	2.52932372077472e-08	0.34192065046402	0.898	0.832	0.000848284589473425	ANXA1
Recoverec	33	2.59390013961447e-08	0.35536289126565	0.806	0.757	0.0008699422288239	HNRNPU
Recoverec	33	4.96042777907976e-08	0.29888717438655	0.851	0.781	0.00166362826854777	ARAP2
Recoverec	33	7.229552748632e-08	0.27149411866292	0.92	0.888	0.0024246474008362	SAMSN1
Recoverec	33	7.82778045029757e-08	0.43592807557575	0.42	0.327	0.0026252810074208	FAS
Recoverec	33	8.67264159183754e-08	0.34874766474796	0.646	0.584	0.00290863053707047	LPXN
Recoverec	33	1.66425898000922e-07	0.27931221024322	0.63	0.554	0.00558159176715491	TAF7
Recoverec	33	2.29982756968824e-07	0.45157844620246	0.373	0.276	0.00771316170322041	PHLDA1
Recoverec	33	2.35144831102103e-07	0.28471345058089	0.764	0.694	0.00788628734550233	ZEB1
Recoverec	33	3.12144630057646e-07	0.27926387013044	0.776	0.712	0.0104687066028733	SMAP2
Recoverec	33	3.91521672909216e-07	0.29878353646263	0.653	0.569	0.0131308538660293	HSPA5
Recoverec	33	5.96205164456178e-07	0.31758211988579	0.696	0.594	0.0199955288055313	RGCC

Recoverec	33	7.44891752279992e-07	0.30183720013095	0.701	0.618	0.0249821795879664	ARL4C
Recoverec	33	8.48862597815375e-07	0.34961094884112	0.811	0.755	0.028469153805532	RGS1
Recoverec	33	9.84637897893858e-07	0.31490854146868	0.451	0.362	0.0330227858195642	MT-ND6
Recoverec	33	9.9433535971198e-07	0.42202646914116	0.302	0.22	0.0333480192940204	HIST1H1D
Recoverec	40	2.01189205466664e-88	1.84979388683602	1	0.995	6.74748357294098e-84	MT-ND3
Recoverec	40	2.23811446466658e-62	1.16039208297836	1	0.992	7.50618829159879e-58	MT-ND2
Recoverec	40	4.72500726247914e-59	1.21304811527392	0.984	0.613	1.58467293569026e-54	MT1G
Recoverec	40	1.97581966415746e-51	0.96794626345671	1	0.99	6.62650398965128e-47	MT-ND1
Recoverec	40	7.07232171253429e-46	0.89155345053508	0.789	0.293	2.37191525594975e-41	MT1H
Recoverec	40	5.14127967277038e-41	1.40730580104357	0.903	0.531	1.72428237665373e-36	PLCG2
Recoverec	40	1.16647575813286e-36	1.01039025639392	0.973	0.671	3.91212639762597e-32	MT-ATP8
Recoverec	40	1.94152063255919e-35	0.62623005178984	1	0.998	6.511471897477e-31	MT-ND4
Recoverec	40	1.92621772272858e-30	0.45064763367631	1	1	6.46014899848713e-26	MT-CO3
Recoverec	40	3.19600453190102e-30	0.90063220920348	0.865	0.483	1.07187599990896e-25	MTRNR2L8
Recoverec	40	2.2682370474765e-29	0.67842054206035	0.584	0.217	7.60721340982669e-25	MT1E
Recoverec	40	1.5297170669717e-26	0.82690480132162	0.957	0.737	5.13036509920969e-22	MT-ND4L
Recoverec	40	1.9406387557924e-25	0.70226104067072	0.659	0.3	6.50851425917656e-21	APOB
Recoverec	40	5.18220616879006e-25	0.38714449409719	0.238	0.046	1.73800830488881e-20	MT1B
Recoverec	40	5.50780971907068e-25	0.79855437886902	0.292	0.072	1.84720922358192e-20	AL360012.1
Recoverec	40	8.44061121093933e-25	0.48075600664296	1	1	2.83081218792483e-20	MT-CYB
Recoverec	40	1.02654101397508e-23	0.46345618768228	1	0.98	3.44281325266963e-19	MT-ND5
Recoverec	40	1.66814209600354e-22	0.37079390727408	1	1	5.59461496157667e-18	MT-CO1
Recoverec	40	8.17764453085081e-22	0.56152203058109	0.746	0.396	2.74261842275675e-17	ANPEP
Recoverec	40	3.36415970627402e-21	0.56739635342586	0.843	0.502	1.12827188229018e-16	ALDOB
Recoverec	40	5.13988654327002e-19	0.33826506286899	1	1	1.7238151488819e-14	MT-CO2
Recoverec	40	8.05032955099618e-19	0.52824090567958	1	0.994	2.6999195248131e-14	MBNL1
Recoverec	40	1.91231958646283e-18	1.23833259537811	0.632	0.342	6.41353742907904e-14	CROCC
Recoverec	40	2.50639800357526e-18	0.70429397958008	0.73	0.429	8.40595762439072e-14	GRAP2
Recoverec	40	4.05676666442236e-18	0.35625004617323	0.265	0.074	1.36055840391397e-13	CYP3A4
Recoverec	40	4.11662234403375e-18	0.64518168378154	0.924	0.722	1.38063280174204e-13	IKZF1
Recoverec	40	5.90280013969134e-18	0.64799426254555	0.67	0.366	1.97968111084968e-13	CUBN

Recoverec	40	1.00124810551839e-17	0.53501711838222٤	0.773	0.479	3.35798589628759e-13	MT2A
Recoverec	40	1.26754329031151e-17	1.30888638762236	0.508	0.245	4.25108668704675e-13	AC015849.1
Recoverec	40	2.20753199738316e-17	0.42617292067714٤	0.497	0.216	7.40362081282366e-13	REG3A
Recoverec	40	5.72576606119987e-17	0.33208472600216	0.319	0.105	1.92030742160521e-12	MT1M
Recoverec	40	8.6922690849425e-17	0.43027296149601	0.481	0.202	2.91521320570802e-12	HNF4G
Recoverec	40	4.6689745936243e-16	0.27403020145182	0.195	0.047	1.56588069920972e-11	AATK
Recoverec	40	5.82516756758388e-16	0.55122219003828٤	0.919	0.702	1.95364469881628e-11	MTRNR2L12
Recoverec	40	5.82548844499773e-16	0.47125480952006	0.319	0.111	1.95375231468334e-11	MIR29B2CHG
Recoverec	40	7.97334741194019e-16	0.61764754079054	0.957	0.877	2.6741012550165e-11	THEMIS
Recoverec	40	1.20886837256905e-15	0.45781471490846	0.454	0.195	4.05430274792208e-11	SLC15A1
Recoverec	40	2.08871911246795e-15	0.31623709305440	0.795	0.511	7.00514615939502e-11	DEFA5
Recoverec	40	4.03720929694179e-15	0.53820965863810٤	0.778	0.484	1.35399925400834e-10	NFATC3
Recoverec	40	5.12343655330202e-15	0.4003837393043	0.362	0.136	1.71829815124643e-10	NTS
Recoverec	40	1.01607708533862e-14	0.55424601224809	0.968	0.871	3.40771932880867e-10	PRKCH
Recoverec	40	1.38929575524881e-14	0.64988701564189	0.519	0.24	4.65942010395347e-10	AC007952.4
Recoverec	40	2.14815213234768e-14	0.45029076753964	0.627	0.342	7.20447262146766e-10	SI
Recoverec	40	3.00051205055751e-14	0.45239238215335	0.481	0.224	1.00631173151598e-09	AL645568.1
Recoverec	40	4.12441803205317e-14	0.52839294921511	0.811	0.556	1.38324731958999e-09	CARD11
Recoverec	40	4.56273432222006e-14	0.27936539347306	0.341	0.128	1.53024983698616e-09	PBX1
Recoverec	40	8.20866930628917e-14	0.51461042201894	0.297	0.111	2.75302351194326e-09	ARRDC3-AS1
Recoverec	40	9.65112226660326e-14	0.64499658924441	0.492	0.25	3.2367933857734e-09	RASSF3
Recoverec	40	1.17625220435779e-13	0.69163499030422	0.838	0.66	3.94491464297515e-09	VMP1
Recoverec	40	1.31760027509301e-13	0.81816410103908	0.681	0.432	4.41896780260695e-09	ERCC1
Recoverec	40	1.52278392299217e-13	0.34710938697515	0.303	0.112	5.10711272093113e-09	MYO1A
Recoverec	40	2.83665575842123e-13	0.48140979682837	0.595	0.32	9.51357608259312e-09	SELENOP
Recoverec	40	3.55923195707546e-13	0.53813044186150	0.627	0.363	1.19369521376397e-08	MT-ND6
Recoverec	40	3.85934008130994e-13	0.75429392773670	0.757	0.489	1.29434547646973e-08	NABP1
Recoverec	40	3.99288883738994e-13	0.38668961797636	0.659	0.379	1.33913505828384e-08	OLFM4
Recoverec	40	4.18381903534015e-13	0.32726858437421	0.27	0.095	1.40316922807238e-08	A1CF
Recoverec	40	6.01882410917008e-13	0.46903400085029	0.919	0.742	2.01859322973346e-08	DOCK2
Recoverec	40	7.60966794256077e-13	1.13457236381552	0.6	0.372	2.55213043457603e-08	AC022217.3

Recoverec	40	1.07880709597024e-12	0.42522067583055	0.541	0.281	3.618103238465e-08	TCF20
Recoverec	40	1.32995780656753e-12	0.26388097186084	0.205	0.063	4.46041249166617e-08	ACE2
Recoverec	40	1.33832867136707e-12	0.43944368702691	0.292	0.114	4.48848669803087e-08	KMT2E-AS1
Recoverec	40	1.3929610285296e-12	0.311111455673615	0.146	0.036	4.67171269748256e-08	FP671120.1
Recoverec	40	1.64545310366433e-12	0.32485982290932	0.265	0.095	5.51852061906942e-08	MTRNR2L1
Recoverec	40	2.43219417469395e-12	0.26961440299154	0.173	0.048	8.15709282308858e-08	GIMAP5
Recoverec	40	2.52579331359473e-12	0.30975965558988	0.276	0.102	8.471005615134e-08	TRPM2
Recoverec	40	2.69971061090319e-12	0.48077075031956	0.578	0.324	9.05428944684713e-08	MALRD1
Recoverec	40	2.76061210148432e-12	0.28567709275334	0.205	0.063	9.25854086595811e-08	ARHGEF38
Recoverec	40	3.11388926756519e-12	0.36270515587588	0.395	0.178	1.04433618255601e-07	SOX6
Recoverec	40	3.14674509069677e-12	0.40805288317772	0.432	0.206	1.05535536851788e-07	NOL4L
Recoverec	40	3.45284913214477e-12	0.65568043392716	0.346	0.155	1.15801654193871e-07	HIST1H2AC
Recoverec	40	4.05396692794727e-12	0.46509160214245	0.735	0.482	1.35961942829495e-07	GATAD2A
Recoverec	40	4.05999177150684e-12	0.54756471254609	0.892	0.754	1.36164004032796e-07	LPP
Recoverec	40	4.13580916989158e-12	0.89221928259640	0.319	0.136	1.38706767939824e-07	POLG2
Recoverec	40	4.4687002419777e-12	0.25456838892739	0.254	0.091	1.49871268715448e-07	MEP1A
Recoverec	40	4.92988224709093e-12	0.40302676070217	0.276	0.102	1.65338390802936e-07	AC098829.1
Recoverec	40	5.4542898015653e-12	0.32133260110458	0.303	0.119	1.82925971364897e-07	AL627171.2
Recoverec	40	5.71436885742581e-12	0.36363482046386	0.492	0.237	1.91648502740347e-07	GDA
Recoverec	40	6.61255651716838e-12	0.50526647854394	0.551	0.311	2.21771920472793e-07	CEP192
Recoverec	40	6.64189649788701e-12	0.40578733116296	0.73	0.458	2.22755924746135e-07	APOA1
Recoverec	40	6.84362431155531e-12	0.59524537852101	0.892	0.762	2.29521472160942e-07	CHST11
Recoverec	40	8.63261359792926e-12	0.85913733979294	0.708	0.456	2.89520594847352e-07	GALM
Recoverec	40	1.19622271473384e-11	0.56962072177006	0.714	0.455	4.01189174067435e-07	SCFD2
Recoverec	40	1.22839018812333e-11	0.45464523059982	0.557	0.315	4.11977501292803e-07	RREB1
Recoverec	40	1.35845673804782e-11	0.37490934762150	0.384	0.179	4.55599220806478e-07	ENPEP
Recoverec	40	1.40972969528239e-11	0.46586917625451	0.551	0.322	4.72795145203809e-07	DGLUCY
Recoverec	40	1.43380697594671e-11	0.54973480483290	0.319	0.139	4.80870183593006e-07	BAIAP2
Recoverec	40	1.53679521403428e-11	0.49064782238243	0.584	0.352	5.15410378882817e-07	ABR
Recoverec	40	1.66157606167676e-11	0.48189588990145	0.708	0.462	5.57259379565151e-07	ST8SIA4
Recoverec	40	1.74651412751128e-11	0.25114628665578	0.157	0.043	5.85745908084733e-07	PTCH2

Recoverec	40	1.88290532361748e-11	0.3083627994947	0.351	0.155	6.31488787434829e-07	SMIM24
Recoverec	40	2.05581402959176e-11	1.0697959572529	0.503	0.269	6.89478909244485e-07	TEX14
Recoverec	40	2.19551901490977e-11	0.50887876743568{	0.486	0.264	7.36333167220439e-07	AVL9
Recoverec	40	2.21445323896536e-11	0.46171662345809{	0.486	0.254	7.42683327284203e-07	MAN1C1
Recoverec	40	2.26299928365066e-11	0.43132024277708	0.238	0.086	7.58964699750759e-07	AC090825.1
Recoverec	40	2.37231356783468e-11	0.47538146108010{	0.568	0.335	7.95626524380394e-07	KIAA1468
Recoverec	40	2.49391099856181e-11	0.25979467034118{	0.303	0.122	8.3640787069766e-07	RAPGEF5
Recoverec	40	2.66687916576652e-11	0.30384533528461	0.465	0.231	8.94417934614775e-07	CDHR5
Recoverec	40	3.00287791339753e-11	0.30146267122006{	0.319	0.134	1.00710519459526e-06	PTGR1
Recoverec	40	3.06429444437298e-11	0.28221795396270:	0.205	0.068	1.02770307075381e-06	ST3GAL2
Recoverec	40	3.21145506643573e-11	0.63609772254338	0.822	0.658	1.07705780018121e-06	SFMBT2
Recoverec	40	3.22810718588755e-11	0.56359979856583:	0.395	0.197	1.08264258800297e-06	IQCN
Recoverec	40	3.57560814341839e-11	0.32042545282088{	0.189	0.06	1.19918745913966e-06	AC138150.1
Recoverec	40	3.9335312850332e-11	0.40561263343892{	0.416	0.202	1.31922772237443e-06	CDYL2
Recoverec	40	5.71034070935131e-11	0.42104435288134	0.595	0.348	1.91513406710224e-06	PPP6R2
Recoverec	40	5.88458086316272e-11	0.50947286567926:	0.503	0.275	1.97357072988751e-06	PIK3CD
Recoverec	40	6.15424317685343e-11	0.42899594986291(	0.978	0.929	2.0640100766531e-06	CELF2
Recoverec	40	6.64905784927393e-11	0.55185949354365:	0.643	0.415	2.22996102148949e-06	PTK2B
Recoverec	40	6.97078986657448e-11	0.55189637296292!	0.789	0.547	2.33786350545175e-06	LCP2
Recoverec	40	7.45792273811124e-11	0.52017009682041	0.957	0.894	2.50123812790775e-06	PIP4K2A
Recoverec	40	7.91096102280726e-11	0.57696969407677!	0.832	0.637	2.6531781078291e-06	GSK3B
Recoverec	40	8.00317861251856e-11	0.63022339736556:	0.919	0.786	2.68410604306648e-06	MGAT5
Recoverec	40	8.12561731945917e-11	0.28963293610099!	0.249	0.094	2.72516953660022e-06	AL669831.5
Recoverec	40	8.73563436560585e-11	0.32357535292002	0.292	0.12	2.92975705353689e-06	LIMD1
Recoverec	40	8.89795345652686e-11	0.53580660943883:	0.724	0.506	2.98419563024998e-06	ARHGEF3
Recoverec	40	9.43507268799015e-11	0.42118685390917!	0.627	0.374	3.16433467809814e-06	TPD52
Recoverec	40	1.05577081023482e-10	0.371101108700267	0.362	0.167	3.54084414336554e-06	BCL2L1
Recoverec	40	1.30245402266939e-10	0.65522339302608{	0.849	0.627	4.36817030122859e-06	PRKCA
Recoverec	40	1.32286964530605e-10	0.49623176737570{	0.822	0.643	4.43664021642744e-06	SUCLG2
Recoverec	40	1.47367950135433e-10	0.29518861737714:	0.357	0.161	4.94242631164215e-06	PRELID2
Recoverec	40	2.07610454651135e-10	0.46972513459899:	0.568	0.357	6.96283942808975e-06	SPON1

Recoverec	40	2.37171921044355e-10	0.33552652277137	0.346	0.163	7.95427188798559e-06	MME
Recoverec	40	2.5650577628908e-10	0.29932622446524‹	0.422	0.212	8.60269072518317e-06	TMEM164
Recoverec	40	2.68404721918768e-10	0.44078548644818‹	0.384	0.192	9.00175756371165e-06	C1orf21
Recoverec	40	3.00203263770027e-10	0.25479503615211‹	0.195	0.066	1.00682170603192e-05	C6orf99
Recoverec	40	3.22930567080172e-10	0.28207694194026‹	0.324	0.143	1.08304453587348e-05	TMEM161B-AS1
Recoverec	40	3.29476649534211e-10	0.35983566739692‹	0.314	0.137	1.10499878720784e-05	MGAM
Recoverec	40	3.33603551027927e-10	0.46567689946292‹	0.838	0.624	1.11883958943746e-05	PHGR1
Recoverec	40	3.36445577150828e-10	0.31179496122451‹	0.324	0.143	1.12837117664845e-05	RBM47
Recoverec	40	3.59595395700481e-10	0.43806609630391‹	0.984	0.959	1.20601103810027e-05	SKAP1
Recoverec	40	4.26115331457679e-10	0.35205883673811‹	0.438	0.227	1.42910559864276e-05	CCM2
Recoverec	40	4.70931430663761e-10	0.54383343000838‹	0.632	0.408	1.57940983216012e-05	PDS5B
Recoverec	40	4.81874251250058e-10	0.28408390081952‹	0.362	0.171	1.61610986384245e-05	AC026979.2
Recoverec	40	6.18678950858033e-10	0.44565899754454‹	0.876	0.735	2.07492546538767e-05	LINC01934
Recoverec	40	6.22581542298731e-10	0.39597623914300‹	0.335	0.158	2.08801397656148e-05	MIATNB
Recoverec	40	6.65521995738577e-10	0.39315514019611‹	0.87	0.699	2.23202766930804e-05	AAK1
Recoverec	40	6.70222653740752e-10	0.25438693288769‹	0.184	0.062	2.24779273611573e-05	SLC10A2
Recoverec	40	6.75656792911057e-10	0.44348746629100‹	0.205	0.075	2.2660177520651e-05	HIST1H2BD
Recoverec	40	6.82147429608654e-10	0.43962183296845‹	0.962	0.861	2.28778604942151e-05	NEAT1
Recoverec	40	6.91569844559244e-10	0.48794947962188‹	0.751	0.55	2.31938694468279e-05	INPP4A
Recoverec	40	6.98437472950025e-10	0.29783279914854‹	0.335	0.154	2.34241959677979e-05	RALGPS1
Recoverec	40	6.99075484449463e-10	0.35263615545032‹	0.551	0.316	2.34455935974661e-05	ZFYVE28
Recoverec	40	7.63804069597303e-10	0.50967229180789‹	0.859	0.68	2.56164608861544e-05	ERBIN
Recoverec	40	7.76576197531864e-10	0.54543515420059‹	0.714	0.528	2.60448125128236e-05	NCOA3
Recoverec	40	8.25341222043467e-10	0.49480424670766‹	0.87	0.664	2.76802939048938e-05	DENND1B
Recoverec	40	8.42543771637184e-10	0.39453684634358‹	0.686	0.454	2.82572330131679e-05	PTEN
Recoverec	40	8.72679677090874e-10	0.44036747403221‹	0.973	0.94	2.92679310102737e-05	ANKRD44
Recoverec	40	8.93607790758972e-10	0.48893437001603‹	0.595	0.382	2.99698180864744e-05	KAT2B
Recoverec	40	1.12945991538703e-09	0.47556094599309‹	0.881	0.715	3.78798266422503e-05	ZNF609
Recoverec	40	1.27354478102961e-09	0.50012545464570‹	0.714	0.495	4.27121448661711e-05	FUT8
Recoverec	40	1.34532824000621e-09	0.25308445651337‹	0.222	0.083	4.51196185133283e-05	KIAA1211
Recoverec	40	1.36618596402952e-09	0.36317791334604‹	0.281	0.122	4.58191448616219e-05	FKBP15

Recoverec	40	1.39971232187497e-09	0.44266022848108	0.881	0.75	4.69435518510428e-05	SSH2
Recoverec	40	1.4810437778347e-09	0.49538749763026	0.924	0.816	4.96712462210202e-05	PPP3CA
Recoverec	40	1.5628977001761e-09	0.47897444825403	0.859	0.708	5.24164630685061e-05	TBC1D5
Recoverec	40	1.64668681510229e-09	0.41693778904833	0.73	0.514	5.52265824049007e-05	GATAD2B
Recoverec	40	1.70961707549288e-09	0.57239372090382	0.27	0.119	5.73371374778802e-05	AL035530.2
Recoverec	40	1.77938940213041e-09	0.32680541323531	0.259	0.11	5.96771617686496e-05	SCIN
Recoverec	40	1.82650233952848e-09	0.26283950025365	0.281	0.12	6.12572354631061e-05	ZDHHC13
Recoverec	40	1.85846056316037e-09	0.41973586507946	0.741	0.54	6.23290503672725e-05	ZNF407
Recoverec	40	2.01295524630077e-09	0.40425051723927	0.768	0.541	6.75104930504352e-05	MLLT3
Recoverec	40	2.05230113337916e-09	0.34807632422706	0.622	0.391	6.88300754112704e-05	ATP5ME
Recoverec	40	2.09821196445169e-09	0.377711183110284	0.578	0.338	7.03698328637809e-05	ITGAL
Recoverec	40	2.19568528085372e-09	0.32527355888412	0.254	0.106	7.36388929492721e-05	AL139383.1
Recoverec	40	2.22551801224885e-09	0.33093419749297	0.427	0.229	7.46394230948021e-05	GIT2
Recoverec	40	2.28560895003918e-09	0.44195958101126	0.881	0.72	7.66547529664141e-05	UBAC2
Recoverec	40	2.37390344412401e-09	0.45577139224839	0.914	0.788	7.9615973709031e-05	MED13L
Recoverec	40	2.43699097006996e-09	0.38891492710120	0.281	0.125	8.17318031542065e-05	COX10
Recoverec	40	2.58481528246724e-09	0.40996569575057	0.53	0.317	8.66895349433863e-05	WWP2
Recoverec	40	2.75646519051697e-09	0.33907118613937	0.341	0.167	9.24463295595582e-05	CAMK2G
Recoverec	40	2.80110297360411e-09	0.58945351343762	0.541	0.329	9.39433915287346e-05	RIN3
Recoverec	40	2.80537376523183e-09	0.57885307667676	0.886	0.797	9.4086625338345e-05	DYRK1A
Recoverec	40	2.84442573301026e-09	0.40860758588748	0.989	0.938	9.5396350233698e-05	RABGAP1L
Recoverec	40	3.05698281360545e-09	0.52819076042780	0.73	0.517	0.0001025250896027	NSMCE2
Recoverec	40	3.15294425544669e-09	0.39951014248917	0.784	0.575	0.000105743444439171	PCNX2
Recoverec	40	3.23430059131535e-09	0.31005003726651	0.195	0.071	0.000108471973231534	LINC01135
Recoverec	40	3.63999099543889e-09	0.471820628545	0.838	0.65	0.000122078018005029	TAOK3
Recoverec	40	3.70333982647524e-09	0.25683195424055	0.249	0.103	0.000124202611100327	HEPH
Recoverec	40	3.76895146886051e-09	0.49468457168751	0.724	0.502	0.000126403094362644	XYLT1
Recoverec	40	4.239994312918e-09	0.33050901889847	0.303	0.141	0.000142200929266644	PSD4
Recoverec	40	4.38771270763393e-09	0.49338331336233	0.4	0.217	0.000147155108788627	FAR2
Recoverec	40	4.4706363775432e-09	0.54732186582939	0.314	0.148	0.000149936202830044	MIR222HG
Recoverec	40	4.67725754215904e-09	0.44671269423216	0.886	0.776	0.00015686586344893	WNK1

Recoverec	40	4.68367018755219e-09	0.37798349881613	0.324	0.163	0.000157080930750125	FMNL3
Recoverec	40	4.69857710702405e-09	0.25981549205447	0.178	0.063	0.000157580879015373	ARRB1
Recoverec	40	4.80768931715292e-09	0.31634455508021	0.303	0.141	0.000161240284318675	ALPK1
Recoverec	40	4.92596726081416e-09	0.25607959167865	0.378	0.186	0.000165207089993185	PDE3A
Recoverec	40	5.35312869854133e-09	0.35396187460703	0.519	0.305	0.000179533230291679	TIAM2
Recoverec	40	5.45318934747906e-09	0.27861830415991	0.368	0.181	0.000182889064335753	DNAJC5
Recoverec	40	6.00491524289461e-09	0.55957034827322	0.778	0.606	0.000201392847416199	FOXO1
Recoverec	40	6.0743186175596e-09	0.27374908847632	0.281	0.126	0.000203720497795714	NHSL1
Recoverec	40	6.09769156777827e-09	0.41407882249483	0.443	0.249	0.000204504379800148	NUDCD3
Recoverec	40	6.60407581317503e-09	0.34315597479285	0.416	0.221	0.000221487494622264	LINC00278
Recoverec	40	7.40475146325722e-09	0.42262567167620	0.946	0.848	0.000248340554574721	ARID1B
Recoverec	40	7.75081950909894e-09	0.37637959627355	0.13	0.038	0.00025994698469616	HIST2H2BF
Recoverec	40	7.88526010814571e-09	0.39979255858133	0.557	0.333	0.000264455853506991	HMBOX1
Recoverec	40	9.68860616607678e-09	0.44353617038156	0.589	0.383	0.000324936473597883	UHRF2
Recoverec	40	1.03976013190912e-08	0.46116988726746	0.724	0.52	0.000348714753039681	PPP2R2B
Recoverec	40	1.12515580666106e-08	0.37373705846849	0.638	0.413	0.000377354754437988	ITPKB
Recoverec	40	1.15453341525267e-08	0.33103829649447	0.486	0.279	0.000387207416807442	MT1X
Recoverec	40	1.36930935676516e-08	0.44537252785212	0.973	0.907	0.000459238972071899	HNRNPA2B1
Recoverec	40	1.38420916622891e-08	0.49854240062123	0.811	0.639	0.000464236070169851	SMURF2
Recoverec	40	1.57596088109689e-08	0.34320830494007	0.357	0.184	0.000528545760302273	DAB1
Recoverec	40	1.62528005938693e-08	0.34244037978159	0.254	0.111	0.00054508642631719	TMEM107
Recoverec	40	1.64748572933929e-08	0.46336314490127	0.886	0.712	0.000552533763905812	BCL11B
Recoverec	40	1.72571239356338e-08	0.35195749494453	0.476	0.274	0.000578769422553285	LINC02328
Recoverec	40	1.82844548995414e-08	0.47380780907289	0.822	0.725	0.000613224048420819	TNFAIP8
Recoverec	40	1.85563885758753e-08	0.32551672439335	0.697	0.445	0.000622344160057704	RNF111
Recoverec	40	1.88229933466141e-08	0.37673938154159	0.519	0.32	0.000631285550858743	FBXW4
Recoverec	40	1.92594180577564e-08	0.37787010915574	0.157	0.055	0.000645922362821036	AC103591.3
Recoverec	40	1.96298055628432e-08	0.41441829837704	0.411	0.227	0.000658344418966637	BCKDHB
Recoverec	40	2.0516736882645e-08	0.29060985716423	0.292	0.137	0.000688090321570147	LARGE1
Recoverec	40	2.06154606001772e-08	0.37161287754624	0.762	0.54	0.000691401317608742	PHC3
Recoverec	40	2.12210805606567e-08	0.33077393109637	0.595	0.37	0.000711712599843305	CD58

Recoverec	40	2.20080132737984e-08	0.42634607332342	0.703	0.512	0.00073810474917665	SGMS1
Recoverec	40	2.26811743189733e-08	0.37176880365305	0.832	0.628	0.000760681224309725	KDM2A
Recoverec	40	2.34918956823641e-08	0.39675782024582	0.486	0.288	0.000787871197395127	CDK19
Recoverec	40	2.61144086633306e-08	0.35774069458812	0.449	0.257	0.000875825037750781	LCLAT1
Recoverec	40	2.68006575346688e-08	0.27736433533697	0.286	0.136	0.000898840452397722	LINC00511
Recoverec	40	2.69571796022942e-08	0.46924376971259	0.719	0.539	0.000904089889501744	MYO5A
Recoverec	40	2.8038242679743e-08	0.41540594786265	0.459	0.26	0.000940346582993219	LZTFL1
Recoverec	40	2.90042962337825e-08	0.44782620470051	0.557	0.35	0.000972746087088597	OAT
Recoverec	40	3.05642926179421e-08	0.49282444411208	0.368	0.203	0.00102506524582054	TRAF3IP2-AS1
Recoverec	40	3.17486187604468e-08	0.30883091823668	0.276	0.13	0.00106478517598787	MACC1
Recoverec	40	3.1825620002715e-08	0.36223235813444	0.654	0.422	0.00106736764365106	AC116366.3
Recoverec	40	3.19090546977464e-08	0.41232636547817	0.589	0.387	0.00107016587645302	ZDHHC14
Recoverec	40	3.28438176233469e-08	0.54132865781621	0.67	0.501	0.00110151595545181	PREX1
Recoverec	40	3.3136953292451e-08	0.31504607258335	0.481	0.277	0.00111134713952222	SH3D19
Recoverec	40	3.35955859394703e-08	0.43451919191707	0.708	0.533	0.00112672876123796	MSI2
Recoverec	40	3.60462575188105e-08	0.45194319257005	0.492	0.298	0.00120891938466587	ANKH
Recoverec	40	3.70478639741495e-08	0.36666972969868	0.595	0.385	0.00124251126196503	XKR6
Recoverec	40	3.71947293980099e-08	0.37942514745414	0.47	0.279	0.00124743683455046	MAPK14
Recoverec	40	3.81380860605996e-08	0.25159828667066	0.216	0.09	0.00127907513030039	SLC41A2
Recoverec	40	3.86479879281253e-08	0.61784357095532	0.297	0.144	0.00129617621913347	WDR74
Recoverec	40	3.86993442532299e-08	0.40778072408769	0.578	0.378	0.00129789860756483	PHF14
Recoverec	40	4.44178363845653e-08	0.45956561849470	0.73	0.536	0.00148968539666555	STIM1
Recoverec	40	4.7451427764925e-08	0.39740734777837	0.551	0.36	0.00159142598438006	CDYL
Recoverec	40	4.74755463569894e-08	0.44858145428457	0.481	0.294	0.00159223487372071	PBX3
Recoverec	40	4.77876251426332e-08	0.35782944988989	0.454	0.269	0.00160270137203363	TRAK1
Recoverec	40	5.14440760850119e-08	0.30844252400709	0.465	0.275	0.00172533142373913	AGPAT3
Recoverec	40	5.3685234387471e-08	0.44580407382023	0.919	0.803	0.001800495390887	MACF1
Recoverec	40	5.47418880484992e-08	0.28898864083595	0.249	0.11	0.00183593344137057	PAPSS2
Recoverec	40	5.93840134682909e-08	0.40085674445878	0.524	0.319	0.00199162104369954	MTM1
Recoverec	40	6.29324904712282e-08	0.30276315717837	0.286	0.138	0.00211062986542405	PRKCZ
Recoverec	40	6.38875529675959e-08	0.34837375895951	0.395	0.22	0.00214266075142723	MRPS31

Recoverec	40	6.54578884378456e-08	0.40466043839606¿	0.649	0.437	0.00219532666242847	DENND4C
Recoverec	40	6.59807020493673e-08	0.28695859869581¹	0.314	0.155	0.00221286078533168	ZNF512
Recoverec	40	6.70066268018719e-08	0.26328719004870ȣ	0.265	0.119	0.00224726824968118	TMEM273
Recoverec	40	6.91913698767352e-08	0.46601349207846ȣ	0.595	0.414	0.00232054016292594	SNTB1
Recoverec	40	7.00892867907968e-08	0.50287231198739²	0.481	0.305	0.00235065450038974	BZW2
Recoverec	40	7.19597158558302e-08	0.26843199999737¹	0.341	0.173	0.00241338495037283	ST7L
Recoverec	40	7.50684348369705e-08	0.42433853905261³	0.838	0.667	0.00251764516756231	VTI1A
Recoverec	40	7.56835675854188e-08	0.28624130135803⁵	0.427	0.229	0.00253827548967978	NPIPB5
Recoverec	40	7.65119121261644e-08	0.37103130376534²	0.941	0.8	0.0025660565088873	NCOA2
Recoverec	40	7.74269247064261e-08	0.46726698144459⁵	0.778	0.618	0.00259674420080412	EIF4G3
Recoverec	40	7.80209420656798e-08	0.79356410116494⁶	0.265	0.128	0.00261666635499877	HEATR9
Recoverec	40	7.98791622730311e-08	0.30711259319665²	0.411	0.223	0.00267898734431292	TCF7L2
Recoverec	40	8.14017532598075e-08	0.35128043983398⁷	0.665	0.464	0.00273005200082742	MYO9B
Recoverec	40	8.68635714885189e-08	0.33786449185797⁴	0.422	0.236	0.00291323046058195	CCDC7
Recoverec	40	9.17919106323812e-08	0.45432014342218	0.222	0.097	0.0030785170987888	PTGDR
Recoverec	40	9.38380104305998e-08	0.49562700656745⁴	0.805	0.654	0.00314713919382146	ATP1A1
Recoverec	40	9.61980637475986e-08	0.38871510788094³	0.4	0.231	0.00322629066196696	AKAP10
Recoverec	40	1.0261411214838e-07	0.29619402201846⁹	0.378	0.205	0.00344147209323238	MARS
Recoverec	40	1.02682258501098e-07	0.28572843962286ȣ	0.254	0.117	0.00344375758560984	NDFIP2
Recoverec	40	1.04133656050795e-07	0.42198603467355ȣ	0.611	0.396	0.00349243455663156	ANKIB1
Recoverec	40	1.04167612004472e-07	0.35517450483543⁹	0.681	0.484	0.00349357337140599	NUTM2B-AS1
Recoverec	40	1.04847594253816e-07	0.50157709410114³	0.686	0.508	0.00351637861608448	STK39
Recoverec	40	1.06142032325045e-07	0.43750077325241⁵	0.605	0.403	0.00355979148011737	MIR4435-2HG
Recoverec	40	1.09165982115563e-07	0.26955836646926ȣ	0.351	0.182	0.00366120870819174	MINDY3
Recoverec	40	1.09172359177747e-07	0.39956159630164²	0.232	0.102	0.00366142258210328	PIK3C2B
Recoverec	40	1.11074673002603e-07	0.47375523118646ȣ	0.681	0.507	0.0037252223831613	PTPRJ
Recoverec	40	1.12416579788393e-07	0.48273799316372¹	0.562	0.369	0.00377022725294312	SLCO3A1
Recoverec	40	1.12494034288068e-07	0.25690099995013²	0.578	0.365	0.00377282492195323	PIGR
Recoverec	40	1.12887932230693e-07	0.63388662292407⁴	0.595	0.396	0.00378603547115299	SLC20A1
Recoverec	40	1.15236474232246e-07	0.32399554317344⁹	0.773	0.559	0.00386480087280108	RAPGEF1
Recoverec	40	1.16937274438183e-07	0.40626946001400ȣ	0.151	0.055	0.00392184231010777	HIST1H4H

Recoverec	40	1.19850174646329e-07	0.37941004397387	0.438	0.261	0.00401953515728857	TBC1D2B
Recoverec	40	1.20006196586003e-07	0.360254111348185	0.892	0.738	0.00402476782110139	RNF213
Recoverec	40	1.25222831703462e-07	0.26315197410459	0.124	0.039	0.0041997233296707	NUP210L
Recoverec	40	1.25294028661517e-07	0.26704467797495	0.303	0.146	0.0042021113324995	THSD4
Recoverec	40	1.27639097230071e-07	0.29549585543009	0.195	0.08	0.00428076004290211	AL391839.1
Recoverec	40	1.28305578678087e-07	0.32312714737980	0.497	0.292	0.00430311249770567	ACSL5
Recoverec	40	1.31086748972265e-07	0.29315368195448	0.319	0.162	0.00439638738703183	PTK2
Recoverec	40	1.35896543257907e-07	0.44581056126369	0.838	0.688	0.0045576982677837	ZCCHC7
Recoverec	40	1.46529734241075e-07	0.26154076679453	0.33	0.169	0.00491431422697717	INPP5B
Recoverec	40	1.48447375175245e-07	0.30681936799393	0.286	0.141	0.00497862806862737	PTER
Recoverec	40	1.49906414660929e-07	0.44071879753325	0.303	0.155	0.00502756133489825	NXF1
Recoverec	40	1.53001722596407e-07	0.40389928711293	0.827	0.662	0.00513137177243829	KANSL1
Recoverec	40	1.57704524211099e-07	0.34546511808889	0.389	0.22	0.00528909433299182	FBXO42
Recoverec	40	1.58320718468823e-07	0.26423522543855	0.33	0.17	0.0053097602560074	PACSIN2
Recoverec	40	1.59480623020098e-07	0.43887808523742	0.87	0.717	0.00534866113484805	CEP85L
Recoverec	40	1.71658851021719e-07	0.3005663720679	0.384	0.212	0.00575709454556642	KSR1
Recoverec	40	1.74117846749457e-07	0.34717083467532	0.724	0.524	0.0058395643442833	DLEU2
Recoverec	40	1.77933343257142e-07	0.47888937918931	0.595	0.401	0.00596752846615804	SMAD3
Recoverec	40	1.80781926863083e-07	0.34822241568424	0.319	0.166	0.00606306426313409	PLB1
Recoverec	40	1.9044650340295e-07	0.41042784850584	0.768	0.575	0.00638719483112815	SLFN12L
Recoverec	40	1.96153974515468e-07	0.500051118331488	0.578	0.396	0.00657861199729976	EPAS1
Recoverec	40	1.97938052984564e-07	0.48253586679233	0.405	0.228	0.00663844642099631	SAE1
Recoverec	40	2.05424110604944e-07	0.32550129202344	0.459	0.278	0.00688951382146863	APC
Recoverec	40	2.08332484794604e-07	0.39051290879696	0.908	0.74	0.00698705487504144	CD247
Recoverec	40	2.13675531132038e-07	0.30194422828642	0.746	0.538	0.00716624996310629	AC245297.3
Recoverec	40	2.15783397114416e-07	0.39659732452263	0.6	0.414	0.00723694357242327	MVB12B
Recoverec	40	2.2349949862723e-07	0.35936051310243	0.886	0.747	0.00749572618496003	TCF12
Recoverec	40	2.23636523728863e-07	0.30240035597754	0.503	0.309	0.00750032173281861	ZNF91
Recoverec	40	2.27801558509492e-07	0.30887227761891	0.584	0.374	0.00764000866929133	XPO6
Recoverec	40	2.2963856930218e-07	0.33425655800405	0.249	0.116	0.00770161833725651	PEX3
Recoverec	40	2.42250959512388e-07	0.28006977078225	0.443	0.261	0.00812461268012647	HDAC4

Recoverec	40	2.42369714403256e-07	0.27929016348508€	0.465	0.276	0.00812859548165638	NLRC3
Recoverec	40	2.58526682346395e-07	0.49996692690081	0.778	0.667	0.0086704678725334	PLCL2
Recoverec	40	2.59043376778333e-07	0.30239214642623€	0.827	0.612	0.00868779677039175	ZNF292
Recoverec	40	2.60800230485498e-07	0.27721725869303€	0.265	0.128	0.00874671813002265	MBNL1-AS1
Recoverec	40	2.76265094922071e-07	0.36658323000133€	0.33	0.178	0.00926537875349641	TTC13
Recoverec	40	2.81731294815363e-07	0.36823844456778€	0.605	0.412	0.00944870416551764	ANKRD36
Recoverec	40	2.82060373655713e-07	0.46143004385106	0.897	0.744	0.0094597408116653	NEDD9
Recoverec	40	2.88345809066911e-07	0.34943462642079€	0.357	0.194	0.00967054174448606	RUFY3
Recoverec	40	2.97162030103144e-07	0.28721510800938€	0.373	0.208	0.00996622016559925	FEZ2
Recoverec	40	3.02980878654136e-07	0.47385360816818€	0.654	0.482	0.0101613727083024	UMAD1
Recoverec	40	3.16006224789072e-07	0.35920139206416€	0.276	0.142	0.0105982167669759	ZNF720
Recoverec	40	3.22140198295788e-07	0.45150060944360€	0.735	0.525	0.0108039379704441	FOXO3
Recoverec	40	3.27629108188736e-07	0.49063716876569€	0.249	0.121	0.0109880250304338	LRRC23
Recoverec	40	3.32352518487499e-07	0.33217658489640€	0.497	0.301	0.0111464387650337	PIK3C3
Recoverec	40	3.41827651707382e-07	0.49760205890286€	0.232	0.111	0.0114642157829622	CSRNP2
Recoverec	40	3.42897423648398e-07	0.37663806109674€	0.6	0.412	0.01150009379432	GALNT10
Recoverec	40	3.74386781646511e-07	0.39277406530382€	0.67	0.469	0.0125561838828607	FLI1
Recoverec	40	3.95395609261429e-07	0.36296988075925€	0.551	0.351	0.0132607779434098	LUZP1
Recoverec	40	3.98066441708005e-07	0.31441591567680€	0.497	0.308	0.0133503523220031	ARMH3
Recoverec	40	4.00911066058262e-07	0.25379545972464€	0.47	0.281	0.013445755333462	ME2
Recoverec	40	4.02619234032108e-07	0.32933376044592€	0.665	0.47	0.0135030438709688	ARHGAP25
Recoverec	40	4.10748981815046e-07	0.33141170540018	0.838	0.657	0.013775699352113	FRYL
Recoverec	40	4.12439325148891e-07	0.41619492955645€	0.935	0.812	0.0138323900868435	FYB1
Recoverec	40	4.13401942984939e-07	0.36162189720129€	0.503	0.309	0.0138646743638289	ARHGEF12
Recoverec	40	4.44375757016821e-07	0.27196903250823€	0.292	0.148	0.0149034741388301	EPB41L3
Recoverec	40	4.5785722040537e-07	0.29676270884603€	0.476	0.295	0.0153556154579553	FABP2
Recoverec	40	4.64492255078933e-07	0.26308565880657	0.351	0.191	0.0155781412508372	KLHL2
Recoverec	40	4.66053068081022e-07	0.30264539183763€	0.168	0.066	0.0156304877973013	EPHA1-AS1
Recoverec	40	5.11362602759526e-07	0.29042065632152€	0.265	0.132	0.017150078971349	CD244
Recoverec	40	5.18201219756436e-07	0.28023752285769€	0.611	0.409	0.0173794325081913	ZZEF1
Recoverec	40	5.36670484022866e-07	0.26673669465211€	0.243	0.116	0.0179988546931589	PLCG1

Recoverec	40	5.4172085576364e-07	0.27039632589255[illegible]	0.524	0.324	0.018168234060601	ARHGAP18
Recoverec	40	5.72996426656236e-07	0.27570007590956[illegible]	0.941	0.854	0.0192171541571968	ATXN1
Recoverec	40	5.97278562631599e-07	0.30229836045171[illegible]	0.384	0.22	0.0200315284335386	SERGEF
Recoverec	40	5.97550385706642e-07	0.49687916848570[illegible]	0.838	0.705	0.0200406448358294	TRA2B
Recoverec	40	6.0550158854392e-07	0.27364626525784[illegible]	0.276	0.137	0.020307312276586	NR5A2
Recoverec	40	6.200428925956e-07	0.30305811111441	0.908	0.796	0.0207949985318712	ZFAND3
Recoverec	40	6.3543467961222e-07	0.29048977290385[illegible]	0.222	0.1	0.0213112082848346	MTERF1
Recoverec	40	6.66212704588285e-07	0.44128155403005[illegible]	0.427	0.263	0.0223434416864819	MAGED1
Recoverec	40	6.87949236014443e-07	0.47414967590725[illegible]	0.405	0.25	0.0230724414774524	NLK
Recoverec	40	7.08248679185613e-07	0.37235393647800[illegible]	0.546	0.353	0.0237532442025271	HIP1
Recoverec	40	7.11087555578006e-07	0.27891099950357[illegible]	0.378	0.211	0.0238484544389752	AL354733.3
Recoverec	40	7.17406954785335e-07	0.68765140844535[illegible]	0.481	0.321	0.0240603944495906	TNF
Recoverec	40	7.35525232374581e-07	0.33835792575149[illegible]	0.708	0.507	0.0246680452433787	SPPL3
Recoverec	40	7.56677512751461e-07	0.68824684134742[illegible]	0.286	0.156	0.0253774504226585	SLC9A1
Recoverec	40	7.62068549283282e-07	0.34015828034701[illegible]	0.416	0.253	0.0255582550058627	CYP3A5
Recoverec	40	7.74020817525327e-07	0.40875360931806[illegible]	0.368	0.211	0.025959110178164[illegible]	CPEB3
Recoverec	40	7.86319814722757e-07	0.36410445900269	0.449	0.281	0.0263715939461718	KDM3B
Recoverec	40	7.88165345615579e-07	0.33544063855419[illegible]	0.578	0.38	0.0264334893612553	ACTN4
Recoverec	40	8.00092242964427e-07	0.34086787471178[illegible]	0.384	0.22	0.0268334936445409	GPD2
Recoverec	40	8.08513036991316e-07	0.34247869263984[illegible]	0.676	0.483	0.0271159102346148	DOCK11
Recoverec	40	8.13621351003323e-07	0.25262465068985[illegible]	0.265	0.132	0.0272872328699494	AP2A2
Recoverec	40	8.35211466623509e-07	0.45763255997658[illegible]	0.195	0.086	0.0280113221676192	SLC10A1
Recoverec	40	8.44729147188327e-07	0.28968214978689[illegible]	0.838	0.639	0.0283305261384021	PICALM
Recoverec	40	8.53737692187903e-07	0.29731241117428[illegible]	0.422	0.259	0.0286326547205979	MFSD14C
Recoverec	40	8.99712741227075e-07	0.52177935264083[illegible]	0.249	0.124	0.0301745659152736	KCNQ1OT1
Recoverec	40	9.17257163270615e-07	0.27522488773018[illegible]	0.211	0.094	0.0307629707417699	ZSWIM8
Recoverec	40	9.23073419555528e-07	0.39940283373388[illegible]	0.449	0.28	0.0309580363450533	AUH
Recoverec	40	9.3189275811126e-07	0.41536445334057[illegible]	0.703	0.515	0.0312538193215355	TOX
Recoverec	40	1.04848204925534e-06	0.31333220348941[illegible]	0.686	0.524	0.0351639909679255	FAM214A
Recoverec	40	1.04939864554817e-06	0.42768446114080[illegible]	0.432	0.272	0.0351947317743946	HLCS
Recoverec	40	1.10296179509997e-06	0.51491661610895[illegible]	0.503	0.336	0.0369911326840628	KLHL18

Recoverec	40	1.13143819267168e-06	0.30184912010421	0.378	0.22	0.0379461741058228	CHSY1
Recoverec	40	1.1762077728143e-06	0.472409828041341	0.481	0.308	0.039447656284646	MAGI3
Recoverec	40	1.17663221839842e-06	0.290039194907011	0.665	0.446	0.0394618913406463	TNRC6A
Recoverec	40	1.26933643318432e-06	0.367883327258559	0.681	0.495	0.0425710052961359	MNAT1
Recoverec	40	1.29943364804672e-06	0.313690966516772	0.627	0.412	0.0435804056881908	SUSD6
Recoverec	40	1.34348097666265e-06	0.312442395490166	0.768	0.591	0.0450576649953121	STIM2
Recoverec	40	1.35039680894613e-06	0.351899308753034	0.124	0.045	0.0452896081784354	IFIT2
Recoverec	40	1.36068015550787e-06	0.323127023581932	0.157	0.064	0.045634491055423	NCEH1
Recoverec	40	1.45225749610956e-06	0.275167582222214	0.243	0.122	0.0487058119045225	LANCL1
Recoverec	47	1.04852555993925e-46	0.699609152948744	0.823	0.382	3.51654502292425e-42	APOA4
Recoverec	47	5.7820112930095e-32	0.61086594500802	0.546	0.222	1.93917094744953e-27	RBP2
Recoverec	47	8.34185131621615e-27	0.649804536812887	0.606	0.29	2.79769009443257e-22	APOB
Recoverec	47	9.02939867251581e-27	0.536244363199969	0.684	0.341	3.02827972678835e-22	PIGR
Recoverec	47	8.46366339594171e-20	0.819343850613485	0.904	0.735	2.83854342973093e-15	RORA-AS1
Recoverec	47	5.70414962220696e-19	0.396018612917751	0.418	0.182	1.91305770029577e-14	SLC5A1
Recoverec	47	2.20973101325026e-18	0.617572019985849	0.968	0.922	7.41099587223872e-14	RAB11A
Recoverec	47	6.43295175909186e-18	0.444315245549933	0.784	0.489	2.15748336096423e-13	MTFR1
Recoverec	47	1.16520475295525e-17	0.656631761049537	0.968	0.895	3.90786370046133e-13	RORA
Recoverec	47	4.27100589885286e-17	0.578512151342017	0.876	0.641	1.43240995835727e-12	AP001011.1
Recoverec	47	4.5252681672536e-17	0.344037784735139	0.33	0.132	1.51768443793351e-12	AC025180.1
Recoverec	47	9.78714488897577e-17	0.61398017690992	0.543	0.306	3.28241265286469e-12	SORL1
Recoverec	47	1.40482517206727e-16	0.434853882359898	0.986	0.942	4.71150266207923e-12	ZBTB20
Recoverec	47	3.75123450536299e-16	0.584344004904144	0.83	0.62	1.25808902840864e-11	RASA1
Recoverec	47	3.97523692741382e-16	0.460441265194556	0.372	0.17	1.33321496071605e-11	INKA2
Recoverec	47	5.88514751059116e-16	0.556716426942018	0.493	0.268	1.97376077210206e-11	DPYD-AS1
Recoverec	47	6.14953574797045e-16	0.511180656202159	0.918	0.79	2.06243129915433e-11	PCNX1
Recoverec	47	7.7167927272823e-16	0.584496945380104	0.957	0.806	2.58805794487594e-11	HERC1
Recoverec	47	1.62277561287403e-15	0.31519294805084	0.323	0.136	5.44246485045691e-11	THSD4
Recoverec	47	3.59940176882426e-15	0.38141389966804	0.45	0.223	1.20716736522828e-10	AC090772.2
Recoverec	47	4.29117000046625e-15	0.415837999528003	0.986	0.953	1.43917259475637e-10	ARHGAP15
Recoverec	47	6.0833323962559e-15	0.470199547035318	0.933	0.864	2.0402280190563e-10	TNRC6B

Recoverec	47	6.40073085378258e-15	0.613894884109044	0.883	0.731	2.1466771137416e-10	MAML2
Recoverec	47	1.27866189172026e-14	0.416292034574617	0.663	0.406	4.28837625245141e-10	PCSK5
Recoverec	47	2.18399125255775e-14	0.564461118489187	0.546	0.324	7.32466986282818e-10	RUNX2
Recoverec	47	2.82115685115182e-14	0.654492437046528	0.855	0.67	9.46159584739296e-10	RBMS1
Recoverec	47	3.22455856550265e-14	0.561014207264908	0.798	0.574	1.08145245169828e-09	DUSP16
Recoverec	47	4.92061768164901e-14	0.781711645473362	0.535	0.33	1.65027675807145e-09	SPDYA
Recoverec	47	1.3678304979815e-13	0.47696728737917	0.915	0.812	4.58742992413036e-09	AKT3
Recoverec	47	1.67397826515064e-13	0.336449391655737	0.245	0.095	5.6141883056622e-09	AL157786.1
Recoverec	47	1.77008382823955e-13	0.449329982194034	0.95	0.848	5.93650714314981e-09	ATXN1
Recoverec	47	1.84136831634447e-13	0.501789984387824	0.929	0.779	6.17558105935608e-09	MGAT5
Recoverec	47	1.86981549177529e-13	0.441821082820308	0.876	0.702	6.27098719631597e-09	ADK
Recoverec	47	3.87563133808369e-13	0.392882245687207	0.972	0.938	1.29980923816651e-08	RABGAP1L
Recoverec	47	4.25647540825029e-13	0.532506919423857	0.855	0.666	1.42753672241898e-08	ATP2B1
Recoverec	47	6.29593903088998e-13	0.601966983725861	0.755	0.557	2.11153203217988e-08	AC009313.1
Recoverec	47	6.49295416279085e-13	0.459757204791578	0.858	0.652	2.1776069671168e-08	UBR5
Recoverec	47	6.66001093978724e-13	0.422563065626688	0.55	0.327	2.23363446898584e-08	AL157402.2
Recoverec	47	6.78661253390361e-13	0.470946341038813	0.596	0.381	2.27609411162059e-08	AC010894.3
Recoverec	47	7.3731006324806e-13	0.383604301491027	0.986	0.886	2.47279049012134e-08	UTRN
Recoverec	47	7.60180052804308e-13	0.623771066652138	0.727	0.552	2.54949186109509e-08	BCAS3
Recoverec	47	9.66963634117698e-13	0.532053367629078	0.862	0.725	3.24300263610394e-08	PLCB1
Recoverec	47	1.15990319786519e-12	0.432699367782484	0.975	0.942	3.89008334500027e-08	DENND4A
Recoverec	47	1.51616203026348e-12	0.280776597146934	0.188	0.066	5.08490421709766e-08	AL137186.1
Recoverec	47	1.72702963262356e-12	0.469499446929043	0.787	0.598	5.79211198189289e-08	EPB41
Recoverec	47	2.0031222105895e-12	0.573836595216451	0.879	0.738	6.71807126987505e-08	TTC39C
Recoverec	47	2.72378776719776e-12	0.635792776023108	0.784	0.623	9.13503941362784e-08	BACH2
Recoverec	47	2.98174618439121e-12	0.544433035227942	0.908	0.78	1.00001803532112e-07	PDE3B
Recoverec	47	3.66387193699962e-12	0.588187666095539	0.521	0.318	1.22878937023093e-07	BTBD11
Recoverec	47	5.90365281347069e-12	0.276835524494566	0.33	0.156	1.9799670805818e-07	MME
Recoverec	47	6.22012360432342e-12	0.466056642698532	0.986	0.959	2.08610505441799e-07	STAT4
Recoverec	47	7.31881543441564e-12	0.606915095981599	0.61	0.428	2.45458432039432e-07	AL031772.1
Recoverec	47	9.08188253979337e-12	0.512489810883142	0.663	0.462	3.0458817661959e-07	FARS2

Recoverec	47	1.14377321151904e-11	0.49486600786856	0.415	0.231	3.83598659679254e-07	ZBTB20-AS5
Recoverec	47	1.41599890804208e-11	0.45295879249571ξ	0.936	0.813	4.74897713779151e-07	CCNH
Recoverec	47	1.43104279771024e-11	0.39082173421676	0.351	0.182	4.79943133496062e-07	CD44-AS1
Recoverec	47	1.4822073857529e-11	0.67202778339217	0.709	0.52	4.97102713033808e-07	ANK3
Recoverec	47	2.00621380065433e-11	0.32299060916541	0.426	0.235	6.7284398446345e-07	GDA
Recoverec	47	2.09792460207088e-11	0.40940967661610	0.855	0.673	7.03601953042531e-07	TC2N
Recoverec	47	2.70630984029458e-11	0.47100920348995	0.613	0.406	9.07642194237997e-07	AC058822.1
Recoverec	47	2.75593838542357e-11	0.40747448056606	0.897	0.724	9.24286615703358e-07	CYTH1
Recoverec	47	2.78304128110934e-11	0.44480705700345	0.911	0.793	9.33376384858451e-07	DPYD
Recoverec	47	3.06276591738908e-11	0.31686393363295	0.621	0.396	1.02719043337395e-06	ANPEP
Recoverec	47	3.22946039319215e-11	0.52198222543502	0.326	0.169	1.08309642666878e-06	TTC39C-AS1
Recoverec	47	3.5720293547588e-11	0.25695327784712	0.28	0.127	1.19798720499901e-06	PCK1
Recoverec	47	4.02755292240639e-11	0.41328123511008	0.911	0.769	1.35076069911666e-06	SIPA1L1
Recoverec	47	8.54891210113972e-11	0.34955387877973	0.45	0.264	2.86713414048024e-06	SKAP1-AS1
Recoverec	47	9.34529152842585e-11	0.38695441712963	0.89	0.717	3.13422387280346e-06	ELMO1
Recoverec	47	1.05948518828905e-10	0.33112885465817	0.993	0.973	3.55330142448381e-06	FYN
Recoverec	47	1.25823314938733e-10	0.37371174655169	0.73	0.527	4.21986233641524e-06	STIM1
Recoverec	47	1.45523777266292e-10	0.40521674338983	0.848	0.695	4.88057644195689e-06	AAK1
Recoverec	47	1.53736384685891e-10	0.39978700866933	0.915	0.779	5.15601086959542e-06	COG5
Recoverec	47	1.65366632683096e-10	0.37408492345636	0.259	0.121	5.54606612692569e-06	AL356272.1
Recoverec	47	1.6673144184216e-10	0.35076472195788	0.965	0.848	5.59183909650237e-06	PDE7A
Recoverec	47	1.7428856167403e-10	0.36553400165149	0.738	0.551	5.84528978142362e-06	EPC2
Recoverec	47	1.82847560916522e-10	0.36874367036265	0.968	0.849	6.13234149801832e-06	ESYT2
Recoverec	47	2.19389626677699e-10	0.38211381619247	0.777	0.582	7.35788929951666e-06	PAG1
Recoverec	47	2.41319081980204e-10	0.45954277669038	0.617	0.441	8.09335937145207e-06	DOCK9
Recoverec	47	2.78174865745625e-10	0.42534391021164	0.479	0.298	9.32942864737677e-06	USP3-AS1
Recoverec	47	3.03942658266208e-10	0.412487276702	0.848	0.682	1.01936288729321e-05	PACS1
Recoverec	47	3.13398181097964e-10	0.36310741702977	0.723	0.495	1.05107481976635e-05	STK39
Recoverec	47	3.91272659771105e-10	0.61776340975345	0.691	0.536	1.31225024634033e-05	COMMD10
Recoverec	47	4.04025209201069e-10	0.55562442409107	0.372	0.198	1.35501974661855e-05	HBB
Recoverec	47	4.55615974602661e-10	0.27742398873241	0.691	0.477	1.52804485562241e-05	FABP1

Recoverec	47	6.98957918854715e-10	0.26158247235936(	0.369	0.194	2.34416506825494e-05	SLC15A1
Recoverec	47	7.15901664043608e-10	0.36472267284551	0.989	0.922	2.40099100086945e-05	JMJD1C
Recoverec	47	7.78373828888821e-10	0.85317528948777(	0.479	0.319	2.61051014732733e-05	AC013652.1
Recoverec	47	8.53795500380167e-10	0.35450017261885(	0.908	0.791	2.863459349175e-05	ZFAND3
Recoverec	47	8.79722707546349e-10	0.33581610974248(	0.972	0.946	2.95041401656895e-05	PABPC1
Recoverec	47	8.84769880191913e-10	0.39668742741331(	0.312	0.166	2.96734122418764e-05	AC010275.1
Recoverec	47	9.05603663680646e-10	0.38866059511371(	0.879	0.707	3.03721356725215e-05	TBL1XR1
Recoverec	47	9.32020054581153e-10	0.38120835999429(	0.784	0.596	3.12580885905427e-05	FKBP5
Recoverec	47	1.04485434862028e-09	0.60874524302561(	0.624	0.467	3.5042325144027e-05	CAMK1D
Recoverec	47	1.06549840258128e-09	0.44419940663091(	0.816	0.687	3.57346854257708e-05	LINC00513
Recoverec	47	1.10245285598896e-09	0.38092871192798(	0.897	0.737	3.69740638841578e-05	DOCK2
Recoverec	47	1.2026862913271e-09	0.26830022023716(	0.358	0.193	4.03356928385284e-05	AC079793.1
Recoverec	47	1.38707275948442e-09	0.45263889264625	0.78	0.6	4.65196462075884e-05	LINC01004
Recoverec	47	1.47487038557579e-09	0.34893824329897(	0.379	0.212	4.94642029914409e-05	AC006059.1
Recoverec	47	1.67668244100671e-09	0.33902351966050(	0.929	0.81	5.62325757064831e-05	FOXN3
Recoverec	47	1.84366622966724e-09	0.39322563157236(	0.943	0.86	6.18328780105799e-05	PPP1CB
Recoverec	47	1.99132886084636e-09	0.32907943586585(	0.972	0.891	6.67851873350652e-05	KLF12
Recoverec	47	1.99913342542158e-09	0.38771430354525(	0.862	0.679	6.70469368217889e-05	CCDC91
Recoverec	47	2.03048316144169e-09	0.35308343970048(	0.461	0.289	6.80983442684313e-05	HCG18
Recoverec	47	2.0744741431596e-09	0.26869828327486(	0.482	0.286	6.95737138132868e-05	FABP2
Recoverec	47	2.09892989173617e-09	0.36502555690321(	0.926	0.834	7.03939107090477e-05	FOXP1
Recoverec	47	2.13111875827139e-09	0.41679844054119(	0.706	0.52	7.14734609149059e-05	TANC2
Recoverec	47	2.14664687285749e-09	0.50310983184266(	0.433	0.264	7.19942428218945e-05	HIF1A-AS2
Recoverec	47	2.37989621279349e-09	0.30350625982999(	0.578	0.38	7.98169591846682e-05	AC114760.2
Recoverec	47	2.39970730914969e-09	0.29131672145292(	0.72	0.503	8.04813837342622e-05	ALDOB
Recoverec	47	2.51325831191136e-09	0.46953778951781(	0.798	0.659	8.42896572648831e-05	OSBPL3
Recoverec	47	2.64625937515505e-09	0.25601065499307(	0.989	0.936	8.87502469239502e-05	ANKRD44
Recoverec	47	2.96327697355092e-09	0.36519066879041(	0.596	0.409	9.93823831389506e-05	ITPKB
Recoverec	47	3.10590247302455e-09	0.38874435447195(	0.433	0.259	0.000104165757140297	AL137856.1
Recoverec	47	3.34429518717895e-09	0.33456831621566	0.73	0.533	0.000112160971987608	TTC33
Recoverec	47	3.46328452932934e-09	0.31124660300408	0.56	0.375	0.000116151636544647	SEL1L3

Recoverec	47	3.754381207295e-09	0.28328064813907‹	0.493	0.313	0.00012591443693026	ST6GAL1
Recoverec	47	3.91509270607939e-09	0.28390646201684€	0.326	0.179	0.000131304379176491	AC096558.2
Recoverec	47	4.26097021102357e-09	0.27998477766920‹	0.312	0.163	0.000142904418937309	AC015923.1
Recoverec	47	5.30514279636872e-09	0.40155647576158‹	0.894	0.717	0.000177923879104614	IKZF1
Recoverec	47	5.32677842729904e-09	0.60482281491717	0.848	0.756	0.000178649494894755	RNF19A
Recoverec	47	5.93923630444419e-09	0.32216627701984‹	0.94	0.83	0.000199190107178449	RAB8B
Recoverec	47	6.56705789421717e-09	0.38753709392006€	0.472	0.299	0.000220245987656255	IRS2
Recoverec	47	6.7384062465596e-09	0.55223336842084‹	0.918	0.844	0.000225992668697116	TANK
Recoverec	47	7.28913020830746e-09	0.30172865600488‹	0.465	0.289	0.000244462848926216	AC073167.1
Recoverec	47	7.7182421471709e-09	0.4148132375849	0.887	0.817	0.000258854405131818	CAMK4
Recoverec	47	1.00609575261842e-08	0.37458167914772‹	0.957	0.842	0.000337424393513164	ARID1B
Recoverec	47	1.07067234951759e-08	0.36199568245621€	0.968	0.889	0.000359082092581208	PIP4K2A
Recoverec	47	1.08653711820574e-08	0.39922165467459€	0.493	0.326	0.000364402818703842	AC001226.2
Recoverec	47	1.12818807771323e-08	0.26235789360926€	0.245	0.119	0.000378371717503462	PLCG1-AS1
Recoverec	47	1.16177380407602e-08	0.30119039852729‹	0.982	0.943	0.000389635698411015	HNRNPC
Recoverec	47	1.17038598796325e-08	0.30240234741754‹	0.972	0.864	0.000392524052643115	LRBA
Recoverec	47	1.18531771189523e-08	0.45714542531292‹	0.56	0.382	0.000397531854215422	ZDHHC14
Recoverec	47	1.20667197395368e-08	0.37804993013076‹	0.805	0.672	0.000404693646624586	SSBP2
Recoverec	47	1.57090423630951e-08	0.31121042756235€	0.738	0.548	0.000526849862773484	BABAM2
Recoverec	47	2.05735253645827e-08	0.32793332863726‹	0.216	0.101	0.000689994893677373	RPL36A-HNRNPH‹
Recoverec	47	2.09458286457676e-08	0.31260877713619‹	0.961	0.838	0.000702481201121753	ANKRD12
Recoverec	47	2.34265092556904e-08	0.33126196949149‹	0.979	0.889	0.000785678267417346	FNBP1
Recoverec	47	2.40236652765755e-08	0.37974513164087‹	0.358	0.208	0.000805705686045788	AC092652.2
Recoverec	47	2.41355323734585e-08	0.38225089786356‹	0.84	0.686	0.000809457484741052	PRKCB
Recoverec	47	2.50548651984012e-08	0.34365866196388‹	0.738	0.542	0.000840290069023979	EXOC6B
Recoverec	47	2.71469157024361e-08	0.30067647037853€	0.184	0.082	0.0009104532588283	COL10A1
Recoverec	47	2.85294379210975e-08	0.31044575383441‹	0.323	0.18	0.000956820288997768	AF241726.2
Recoverec	47	2.96176166229386e-08	0.46175473572833‹	0.578	0.422	0.000993315626300115	ERC1
Recoverec	47	3.03371615947178e-08	0.43950563284689‹	0.89	0.841	0.00101744772556365	CD44
Recoverec	47	3.61141000072594e-08	0.40624828134534‹	0.621	0.441	0.00121119468604346	OSBPL9
Recoverec	47	4.04776943886188e-08	0.42314147439949‹	0.298	0.164	0.0013575409144055	AC009299.3

Recoverec	47	4.13786567619426e-08	0.27899844015067\u0039	0.833	0.663	0.00138775739048203	HELZ
Recoverec	47	4.13845067882396e-08	0.30201600809261	0.535	0.361	0.00138795358866398	VPS53
Recoverec	47	4.19471305443881e-08	0.35483403040330\u0039	0.266	0.139	0.00140682286419769	AC137810.1
Recoverec	47	4.39812741482511e-08	0.43735389853594\u0037	0.252	0.128	0.00147504397238405	AL357793.1
Recoverec	47	5.190022788271e-08	0.30808526465074\u0032	0.869	0.762	0.00174062984273033	RFX3
Recoverec	47	5.24198935455344e-08	0.39645422092713\u0039	0.379	0.232	0.00175805838973013	AC010609.1
Recoverec	47	5.3671895386738e-08	0.33257410252608\u0033	0.926	0.847	0.00180004802748042	CDK17
Recoverec	47	5.72621473113779e-08	0.40295981658863\u0038	0.684	0.532	0.00192045789652899	RFTN1
Recoverec	47	6.32153186769845e-08	0.28576984623114	0.872	0.709	0.00212011535778871	ZNF609
Recoverec	47	6.92903353490645e-08	0.25186083433673\u0039	0.667	0.477	0.00232385926693693	MECP2
Recoverec	47	8.28240839944658e-08	0.30470664251180\u0035	0.982	0.945	0.0027777541290064	PARP8
Recoverec	47	8.3447032973704e-08	0.27506174867371\u0034	0.415	0.253	0.00279864659187209	POLR3E
Recoverec	47	8.41378248918966e-08	0.28180112910359\u0036	0.606	0.419	0.00282181437122443	AKNA
Recoverec	47	9.85277538959339e-08	0.35685863723619\u0034	0.816	0.64	0.00330442381016183	ZHX2
Recoverec	47	1.00770623477501e-07	0.44597220453718\u0033	0.734	0.585	0.00337964517018844	ICOS
Recoverec	47	1.03986993712803e-07	0.41157915326185\u0034	0.645	0.48	0.00348751579513997	RUNX1
Recoverec	47	1.06555754457612e-07	0.36173077082996\u0031	0.812	0.666	0.00357366689299941	RPS6KA5
Recoverec	47	1.09723163912936e-07	0.34899410433931\u0036	0.89	0.756	0.00367989547131206	IQGAP2
Recoverec	47	1.10207641472732e-07	0.32240071025675	0.83	0.721	0.00369614387971248	GOLGB1
Recoverec	47	1.11697011980798e-07	0.29925355718870\u0033	0.798	0.648	0.00374609438781201	CCND3
Recoverec	47	1.18918533727107e-07	0.31344355037419	0.965	0.931	0.00398828978413972	ARIH1
Recoverec	47	1.26657910127843e-07	0.33018200211719\u0033	0.78	0.617	0.0042478529898676	CLASP1
Recoverec	47	1.28119061603745e-07	0.49746708413822\u0034	0.305	0.17	0.00429685708806642	GFPT2
Recoverec	47	1.30838642380052e-07	0.25802533584242\u0033	0.344	0.201	0.00438806638814218	DPP4
Recoverec	47	1.42389486279762e-07	0.31212788013968	0.869	0.764	0.00477545859085065	FAM172A
Recoverec	47	1.47954749226581e-07	0.53993870829404\u0033	0.443	0.289	0.00496210637956109	CYTH3
Recoverec	47	1.48932300049869e-07	0.29888558874964	0.996	0.986	0.00499489147907251	AC016831.7
Recoverec	47	1.50204395213088e-07	0.35250188460279\u0033	0.546	0.362	0.00503755500665655	CHD7
Recoverec	47	1.60332192704465e-07	0.31055184040021\u0034	0.897	0.786	0.00537722107892235	STAG1
Recoverec	47	1.611003157155e-07	0.29387099835507	0.376	0.234	0.00540298238846644	AC009554.1
Recoverec	47	1.72631255660662e-07	0.39495538112086\u0038	0.387	0.248	0.00578970705234728	DAAM1

Recoverec	47	1.7467192659867e-07	0.32417888371551(	0.794	0.639	0.0058581470742662	ATP8A1
Recoverec	47	1.77213933668074e-07	0.54077462149105:	0.812	0.705	0.00594340090735985	RAPGEF2
Recoverec	47	1.92552837902737e-07	0.38197213003540	0.461	0.314	0.00645783707758199	IL2RA
Recoverec	47	2.02531539772892e-07	0.37067913476094(	0.365	0.224	0.00679250278090326	AC109587.1
Recoverec	47	2.11361972092977e-07	0.35270856592966	0.695	0.521	0.00708865782005426	FOXO3
Recoverec	47	2.13503155007178e-07	0.61816999297368	0.216	0.11	0.00716046881263073	DOK6
Recoverec	47	2.15305542959394e-07	0.49056741167935!	0.706	0.554	0.00722091729977215	TRAF1
Recoverec	47	2.21954837513735e-07	0.29935305295582:	0.734	0.559	0.00744392134053566	ABCC1
Recoverec	47	2.26075710528959e-07	0.37946495468510:	0.518	0.354	0.00758212717972024	SPON1
Recoverec	47	2.27635626245393e-07	0.26107922536954:	0.652	0.474	0.00763444363301799	MLLT10
Recoverec	47	2.30652554317279e-07	0.37422105752733	0.791	0.665	0.00773562536669289	VTI1A
Recoverec	47	2.5803037763806e-07	0.26585262853178!	0.989	0.936	0.00865382280522526	CBLB
Recoverec	47	2.68803110282482e-07	0.31181669974776	0.908	0.785	0.00901511871265389	SLC38A1
Recoverec	47	2.74966753178075e-07	0.32329022617376!	0.631	0.482	0.00922183496808627	NUTM2B-AS1
Recoverec	47	2.77529548488532e-07	0.35070669204907	0.738	0.578	0.00930778599720839	KIAA1109
Recoverec	47	2.98960889673608e-07	0.33444540598176{	0.89	0.738	0.0100265503178735	NEDD9
Recoverec	47	3.35922003133073e-07	0.30546866151695⁄	0.862	0.744	0.011266152141077	TCF12
Recoverec	47	3.49798010649431e-07	0.36004561921496⁄	0.745	0.573	0.0117315256811606	RICTOR
Recoverec	47	3.6126026267095e-07	0.32596234597064:	0.613	0.445	0.0121159466894583	RASGRF2
Recoverec	47	3.67932380362799e-07	0.34002297407178!	0.844	0.713	0.0123397161726075	FTX
Recoverec	47	4.18706177466674e-07	0.2796726540196	0.929	0.77	0.0140425677798773	VPS13B
Recoverec	47	4.30342557487086e-07	0.38121204189559(	0.631	0.488	0.0144328286930019	STAT5B
Recoverec	47	4.31201090468107e-07	0.26417548390655	0.965	0.899	0.0144616221721194	PITPNC1
Recoverec	47	4.3725269013817e-07	0.33132987615247	0.709	0.541	0.014664580721854	PATJ
Recoverec	47	4.89805671479876e-07	0.26363493447148!	0.709	0.525	0.0164271026100921	TTC17
Recoverec	47	5.25758711423362e-07	0.25661863638832:	0.535	0.368	0.0176328956637167	LONP2
Recoverec	47	5.53647760159553e-07	0.39806188757144(	0.777	0.658	0.0185682385802311	FBXL17
Recoverec	47	5.80370195656567e-07	0.26768168975749!	0.578	0.403	0.01946445562193	ARNTL
Recoverec	47	6.25026311321185e-07	0.38031123027647:	0.5	0.351	0.0209621324290899	ZSWIM4
Recoverec	47	6.29113384323957e-07	0.30774416754195:	0.84	0.726	0.0210992046834569	RPS6KA3
Recoverec	47	6.46367856787398e-07	0.34246415893595	0.493	0.333	0.0216778851809357	IQCB1

Recoverec	47	6.63954334632107e-07	0.27812116368897	0.727	0.573	0.0222677004748916	DYM
Recoverec	47	7.2003649057655e-07	0.25411737808884	0.585	0.416	0.0241485838209563	IGF2R
Recoverec	47	7.38301147404627e-07	0.29400467217000	0.699	0.515	0.0247611438816564	FAM214A
Recoverec	47	7.50167935099964e-07	0.30446055401332	0.433	0.275	0.0251591322073826	AUH
Recoverec	47	7.74681110818302e-07	0.29565625410229	0.358	0.22	0.0259812550946242	NFATC1
Recoverec	47	8.05383967411036e-07	0.28581573458172	0.684	0.509	0.0270109674990313	FOXJ3
Recoverec	47	8.10150114174764e-07	0.40670735381033	0.95	0.894	0.0271708145291932	GLS
Recoverec	47	8.44853820180314e-07	0.35242774102081	0.879	0.8	0.0283347074212074	PDE4D
Recoverec	47	8.49453303233488e-07	0.26838008561909	0.993	0.956	0.0284889648838447	SKAP1
Recoverec	47	8.6137388569792e-07	0.28372756361133	0.72	0.528	0.0288887573785368	SENP7
Recoverec	47	8.70099054144096e-07	0.27980696997672	0.826	0.664	0.0291813820778847	MGAT4A
Recoverec	47	8.82704931839625e-07	0.29480113584126	0.957	0.882	0.0296041580040374	ARID4B
Recoverec	47	9.08959034730607e-07	0.27640848052854	0.652	0.492	0.0304846681067951	PRKAR2A
Recoverec	47	9.66673028449568e-07	0.29625503729324	0.869	0.765	0.0324202800281416	CRY1
Recoverec	47	9.89551144483625e-07	0.36614858890090	0.291	0.172	0.0331875662836918	KIF5C
Recoverec	47	1.17722960769789e-06	0.28362761616615	0.645	0.486	0.0394819265829719	WDR70
Recoverec	47	1.20512635247265e-06	0.34233503332483	0.699	0.569	0.0404175276092277	SPIDR
Recoverec	47	1.24369962743334e-06	0.31333243767787	0.911	0.778	0.0417111981048593	MAPRE2
Recoverec	47	1.25587750201551e-06	0.73595971310835	0.571	0.446	0.0421196196625962	C15orf53
Recoverec	47	1.27959526010161e-06	0.36144325778209	0.84	0.662	0.0429150658332877	ODF2L
Recoverec	47	1.37197912727521e-06	0.29120606071904	0.684	0.533	0.0460134359705559	PCGF5
Recoverec	47	1.39679262522283e-06	0.45740796991419	0.688	0.581	0.0468456310647233	NR3C2
Recoverec	98	3.91618388808845e-36	1.03210672488595	0.903	0.926	1.3134097523871e-31	TMSB4X
Recoverec	98	5.90051002394614e-30	0.88891755556427	0.891	0.914	1.97891305183106e-25	RPL39
Recoverec	98	1.17230859463494e-24	0.69286742919015	0.951	0.985	3.93168856468665e-20	B2M
Recoverec	98	5.87297313729251e-21	0.71213422453658	0.858	0.884	1.96967773078516e-16	RPS28
Recoverec	98	2.57552552985171e-20	0.76367548138488	0.843	0.899	8.63779752201665e-16	TMSB10
Recoverec	98	3.94858574105847e-17	0.71972339254861	0.846	0.909	1.32427668583619e-12	RPS27
Recoverec	98	4.92709005207338e-17	0.67997319024788	0.869	0.877	1.65244746166437e-12	RPS27A
Recoverec	98	6.9175259747801e-17	0.79076348374906	0.79	0.838	2.31999986142175e-12	RPL37A
Recoverec	98	1.4201417051057e-16	0.64478490911010	0.876	0.911	4.76287125058351e-12	RPL41

Recoverec	98	5.08646664528447e-16	0.64169675657178	0.91	0.956	1.70589918349551e-11	JUND
Recoverec	98	1.73506637733311e-15	0.63639077709694	0.85	0.887	5.81906561629978e-11	RPLP2
Recoverec	98	6.83338518353206e-14	0.57521592013838	0.929	0.95	2.29178072285298e-09	RPLP1
Recoverec	98	8.91070575189342e-14	0.59337816853502	0.869	0.898	2.98847249507001e-09	RPS18
Recoverec	98	3.26683334954548e-13	0.74741627801510	0.783	0.85	1.09563056877056e-08	RPS29
Recoverec	98	3.31475644932176e-13	0.64929035402834	0.805	0.861	1.11170301797353e-08	RPS11
Recoverec	98	4.27239181325677e-12	0.59990146937176	0.873	0.893	1.43287476633006e-07	RPL34
Recoverec	98	5.40318218128488e-12	0.62675127531228	0.828	0.878	1.81211923995932e-07	RPL37
Recoverec	98	1.12067697391162e-11	0.57973144789785	0.88	0.911	3.7585264351048e-07	RPS12
Recoverec	98	1.48020339495442e-11	0.57897963883344	0.839	0.9	4.96430614599814e-07	RPL28
Recoverec	98	1.58257215547598e-11	0.94847000725250	0.685	0.712	5.30763049503535e-07	CD52
Recoverec	98	2.05156413419146e-11	0.81084234790821	0.697	0.77	6.88053579325131e-07	ATP5F1E
Recoverec	98	5.17064380783608e-11	0.60250644359583	0.816	0.879	1.73413052027206e-06	RPL9
Recoverec	98	2.05765152573831e-10	0.62844376399404	0.764	0.85	6.90095168702113e-06	RPL27A
Recoverec	98	2.42976583669133e-10	0.52547575828504	0.854	0.913	8.14894866309538e-06	RPS19
Recoverec	98	2.98701524038441e-10	0.53963933279993	0.82	0.847	1.00178517132012e-05	RPS25
Recoverec	98	3.42430769094287e-10	0.55064068591428	0.805	0.844	1.14844431338842e-05	RPS13
Recoverec	98	1.11343662193923e-09	0.64251541466166	0.768	0.819	3.73424374265979e-05	UBA52
Recoverec	98	2.67492686656049e-09	0.51306113642240	0.824	0.878	8.97116972507058e-05	RPS23
Recoverec	98	1.36715931896005e-08	0.4885291080244	0.861	0.891	0.00045851789239282	RPS15A
Recoverec	98	2.21832429843418e-08	0.40543288649225	0.899	0.948	0.000743981603208854	PTMA
Recoverec	98	2.67484335671974e-08	1.01836196955738	0.509	0.44	0.000897088964976665	NKG7
Recoverec	98	3.94088222759753e-08	0.46091291676316	0.828	0.884	0.00132169308149166	RPL30
Recoverec	98	7.45356294395993e-08	0.64360018305190	0.745	0.849	0.00249977594014528	RPL35
Recoverec	98	9.07195829619403e-08	0.64715805261787	0.693	0.795	0.00304255337337755	SERF2
Recoverec	98	1.2082895243421e-07	0.44959995062001	0.831	0.886	0.00405236140673852	RPL12
Recoverec	98	1.52523856097048e-07	0.41582746685008	0.805	0.863	0.00511534508578281	RPS15
Recoverec	98	2.65085797174401e-07	0.45787887143669	0.843	0.872	0.00889044746563507	RPS16
Recoverec	98	3.02458787429952e-07	0.55432258365702	0.783	0.854	0.0101438628128257	RPL24
Recoverec	98	3.95593283347665e-07	0.32675769796082	0.921	0.979	0.013267407536914	DDX5
Recoverec	98	4.42449047453733e-07	0.71891811279603	0.659	0.725	0.0148388561535033	CD3D

Recoverec	98	5.26288164287873e-07	0.45452011477530	0.775	0.809	0.0176506524538867	RPL26
Recoverec	98	7.62847675961618e-07	0.48255128541364	0.787	0.84	0.0255843853564008	RPL21
Recoverec	121	4.101437608243e-106	0.70457668519027	0.299	0.006	1.37554014505254e-10	TFPI2
Recoverec	121	7.70025561736552e-58	1.21300614841581	0.496	0.071	2.58251172895205e-53	NRG1
Recoverec	121	5.3011240558259e-50	0.64863400643413	0.291	0.024	1.77789098584289e-45	IGFBP5
Recoverec	121	5.11589086717261e-40	1.19499461071741	0.718	0.231	1.71576747903235e-35	IGLC3
Recoverec	121	1.61508423661841e-31	0.75391399984678	0.333	0.056	5.41666951277083e-27	COL3A1
Recoverec	121	3.91937616050133e-31	1.10066302311738	0.778	0.336	1.31448037670893e-26	IGLC2
Recoverec	121	2.29241937418664e-28	0.76747018022286	0.35	0.069	7.68831609714716e-24	ADGRL2
Recoverec	121	3.00588166551746e-26	0.90614572511634	0.53	0.162	1.00811259298125e-21	IGHM
Recoverec	121	4.12539411279562e-25	0.51398026922317	0.231	0.033	1.38357467754939e-20	SPARC
Recoverec	121	1.02218616200855e-21	0.40047566910430	0.205	0.031	3.42820795014426e-17	TMTC1
Recoverec	121	5.50969995572951e-20	0.55482384002623	0.316	0.075	1.84784317115256e-15	TCF4
Recoverec	121	3.34471786753765e-19	0.26132822011183	0.128	0.013	1.12175147841478e-14	PXDN
Recoverec	121	1.11698614812199e-18	0.50251319948982	0.171	0.025	3.74614814357153e-14	COL6A3
Recoverec	121	3.27642076485837e-18	0.35115732054002	0.162	0.023	1.0988459961182e-13	GNG11
Recoverec	121	2.40182091580225e-17	0.38890760569389	0.214	0.041	8.05522698741758e-13	ADAMTS9
Recoverec	121	3.45119716925339e-17	0.46844883185079	0.222	0.045	1.1574625066242e-12	COL4A1
Recoverec	121	3.6132726219688e-17	0.31648986168253	0.179	0.029	1.2118193719559e-12	C11orf96
Recoverec	121	9.92588115514555e-17	0.38647550828524	0.162	0.025	3.32894202181272e-12	SH3RF3
Recoverec	121	1.18106619578171e-16	0.38920435899985	0.231	0.049	3.96105980741268e-12	PRR16
Recoverec	121	1.76200859415716e-16	0.46916500349845	0.462	0.164	5.90942442308427e-12	TIMP1
Recoverec	121	1.92006139681931e-16	0.34978877874573	0.188	0.034	6.43950191265261e-12	RGS5
Recoverec	121	3.38436619323299e-16	0.54075736415352	0.248	0.058	1.13504873388648e-11	RBPMS
Recoverec	121	4.98315134544022e-16	0.752161351112227	0.812	0.565	1.67124929823374e-11	IGHA1
Recoverec	121	7.90936935520886e-16	0.32225224617641	0.171	0.029	2.65264429434995e-11	HPSE2
Recoverec	121	4.61099648382458e-15	0.28244792605025	0.128	0.017	1.54643600074509e-10	COL1A2
Recoverec	121	1.49952828705367e-14	0.66521931558995	0.222	0.052	5.02911796912061e-10	ADGRL3
Recoverec	121	1.87219581391118e-14	0.26175944034640	0.137	0.021	6.2789703206953e-10	THSD7A
Recoverec	121	2.49952453546527e-14	0.50371250646471	0.214	0.049	8.38290538704343e-10	CALD1
Recoverec	121	4.32537458231684e-14	0.26366044620518	0.137	0.021	1.45064412741742e-09	SLCO2A1

Recoverec	121	1.68289594371741e-13	0.50459393844575	0.419	0.16	5.64409641603945e-09	IFITM3
Recoverec	121	2.28025683637261e-13	0.27268389317484	0.12	0.017	7.64752537782648e-09	MAP1B
Recoverec	121	5.23597341248033e-13	0.2750388374095	0.145	0.026	1.75604076307765e-08	LAMC1
Recoverec	121	1.04007061914087e-12	0.71175216453616	0.581	0.309	3.48818884247465e-08	AC090627.1
Recoverec	121	1.23844635959715e-12	0.59484412838135	0.966	0.895	4.15350140081692e-08	PIP4K2A
Recoverec	121	1.89228013507333e-12	0.61474832920536	0.419	0.172	6.34632911700892e-08	PECAM1
Recoverec	121	1.0467917305292e-11	0.50676026782118	0.188	0.046	3.51073010584882e-07	COL4A2
Recoverec	121	3.48080322444178e-11	0.32979164125505	0.12	0.021	1.16739178541329e-06	PRSS23
Recoverec	121	3.48892428605616e-11	0.25793482403461	0.128	0.024	1.17011542705752e-06	RAI14
Recoverec	121	4.70508298348945e-11	0.63953316829916	0.932	0.847	1.57799073100269e-06	DOCK8
Recoverec	121	4.81494284080007e-11	0.56793921371651	0.59	0.316	1.61483552994753e-06	ACSL4
Recoverec	121	8.73917442227199e-11	0.63603192413152	0.803	0.58	2.93094431774158e-06	PCNX2
Recoverec	121	8.97081902121261e-11	0.40703000584142	0.179	0.045	3.00863328333428e-06	IGFBP4
Recoverec	121	9.13217244463786e-11	0.28002904538224	0.111	0.019	3.06274799448265e-06	COL15A1
Recoverec	121	1.56289505178453e-10	0.64045544611308	0.863	0.759	5.24163742467496e-06	ANKRD11
Recoverec	121	2.4945502260217e-10	0.48480262354812	1	0.94	8.36622254803156e-06	ANKRD44
Recoverec	121	2.92691080900282e-10	0.43350788132192	0.282	0.099	9.81627347123367e-06	SPARCL1
Recoverec	121	4.92376425812734e-10	0.77490752297657	0.821	0.679	1.65133205689075e-05	DNAJB6
Recoverec	121	5.11840216408515e-10	0.37683699258312	0.205	0.059	1.71660971779088e-05	SOX5
Recoverec	121	6.99259962042678e-10	0.59129189778136	0.889	0.72	2.34517806069873e-05	ZNF609
Recoverec	121	7.10989045313143e-10	0.36739279417593	0.162	0.041	2.38451506017122e-05	TCIM
Recoverec	121	1.1896401618651e-09	0.25855318340384	0.128	0.027	3.98981517486317e-05	NXN
Recoverec	121	1.19147212783888e-09	0.49227918221604	0.35	0.148	3.99595922234603e-05	ZBTB16
Recoverec	121	1.41043063696351e-09	0.60583957574245	0.726	0.52	4.73030227024823e-05	LDLR
Recoverec	121	1.59222317981972e-09	0.55295886436117	0.915	0.845	5.33999810047938e-05	SYNE2
Recoverec	121	1.73430514837197e-09	0.53846982427451	0.983	0.902	5.81651260660991e-05	PITPNC1
Recoverec	121	1.93542183168594e-09	0.57232840736346	0.795	0.658	6.49101773910829e-05	MYH9
Recoverec	121	2.06630136236881e-09	0.35214199682027	0.214	0.066	6.92996150911253e-05	IGFBP7
Recoverec	121	2.65658996733797e-09	0.25447667965725	0.12	0.025	8.90967143245809e-05	FBXL7
Recoverec	121	2.95012687219966e-09	0.47548727256146	0.932	0.805	9.89413550398324e-05	MACF1
Recoverec	121	4.43855786048686e-09	0.85341726255037	0.821	0.668	0.000148860353525008	OSBPL3

Recoverec	121	5.0454526186297e-09	0.635195166769787	0.632	0.403	0.000169214389923603	MAPKAP1
Recoverec	121	5.70598441311307e-09	0.537714469594797	0.564	0.322	0.000191367305246986	PPP1R12B
Recoverec	121	7.98342222358357e-09	0.562562358279551	0.701	0.469	0.000267748014534546	MAPK1
Recoverec	121	8.48801579584438e-09	0.345098245644182	0.162	0.044	0.000284671073761029	LDB2
Recoverec	121	1.17321450227918e-08	0.404962762967428	0.983	0.956	0.000393472679774393	ARHGAP15
Recoverec	121	1.39395792248585e-08	0.711778607172099	0.897	0.77	0.000467505608043305	DDX3Y
Recoverec	121	2.28929298686339e-08	0.370306501263674	0.256	0.097	0.000767783081934243	MCTP1
Recoverec	121	2.36111120678608e-08	0.531219477738678	0.761	0.588	0.000791869476531917	R3HDM2
Recoverec	121	2.64607243198891e-08	0.252449467937049	0.128	0.031	0.000887439772240439	SCGB2B2
Recoverec	121	2.9720886082307e-08	0.279744363201441	0.12	0.028	0.000996779077428413	CACNA1C
Recoverec	121	4.30879644605027e-08	0.392625763859708	0.983	0.932	0.00144508415207634	ARIH1
Recoverec	121	4.73520347783094e-08	0.653253273419583	0.615	0.401	0.00158809254239494	AP4B1-AS1
Recoverec	121	4.97132864646864e-08	0.563826660874577	0.675	0.49	0.00166728420145265	PRRC2B
Recoverec	121	5.03554647127488e-08	0.575095463657107	0.897	0.766	0.00168882157553617	CHST11
Recoverec	121	6.33532737949241e-08	0.420949195780531	0.966	0.897	0.00212474209653416	KLF12
Recoverec	121	6.9969630215344e-08	0.276858907321551	0.188	0.062	0.00234664145816221	DLC1
Recoverec	121	7.09350525555868e-08	0.598275574080018	0.581	0.373	0.00237901979260927	ATP10A
Recoverec	121	7.23071584205261e-08	0.585013840503644	0.897	0.74	0.0024250374791076	ITGA1
Recoverec	121	7.92807234421712e-08	0.438646410386088	0.427	0.209	0.00265891690280354	A2M
Recoverec	121	9.80887083009978e-08	0.51971899733466	0.915	0.79	0.00328969909899886	MGAT5
Recoverec	121	9.81206524471162e-08	0.464171113618894	0.299	0.13	0.00329077044177138	HEATR9
Recoverec	121	1.02331086535607e-07	0.541006140264595	0.641	0.417	0.00343197998023119	MVB12B
Recoverec	121	1.08319714014755e-07	1.01723344808787	0.504	0.302	0.00363282656862685	GRIP1
Recoverec	121	1.19156591253551e-07	0.50641795912757	0.897	0.802	0.00399627375746159	DPYD
Recoverec	121	1.20771541205282e-07	0.485044725157869	1	0.987	0.00405043594894274	AC016831.7
Recoverec	121	1.33003482735216e-07	0.516933295244147	0.726	0.502	0.00446067080397369	TSPAN5
Recoverec	121	1.40087400038387e-07	0.582168996270091	0.949	0.875	0.00469825122248742	PRKCH
Recoverec	121	1.529997258317e-07	0.842214722817791	0.556	0.369	0.00513130480494356	FAM13A
Recoverec	121	1.65349801180947e-07	0.293109912744555	0.171	0.055	0.0055455016320066	GPC6
Recoverec	121	1.67469515987159e-07	0.46589378420655	0.88	0.73	0.00561659262717734	ELMO1
Recoverec	121	1.86699329558365e-07	0.55102903233899	0.65	0.423	0.00626152211472846	APOA4

Recoverec	121	1.89234953749429e-07	0.36714227771759	0.991	0.93	0.00634656187884836	CELF2
Recoverec	121	1.95619352642162e-07	0.51215054702360	0.65	0.452	0.00656068184891283	ERCC6L2
Recoverec	121	2.04637774119936e-07	0.58227465445630	0.821	0.731	0.00686314166843442	ARL15
Recoverec	121	2.13192018443015e-07	0.51905851562524	0.735	0.526	0.00715003391454184	FAM214A
Recoverec	121	2.32129511271201e-07	0.47517470946315	0.35	0.174	0.00778515954901355	PTPRG
Recoverec	121	2.36181846771512e-07	0.74637054000179	0.692	0.557	0.00792106677702298	PNPLA8
Recoverec	121	2.38467663361621e-07	0.30187336578604	0.103	0.024	0.00799772849382205	HYAL2
Recoverec	121	2.45819636347032e-07	0.45887848780629	0.966	0.897	0.00824429896380677	DOCK10
Recoverec	121	2.49250946201809e-07	0.50993525821743	0.838	0.606	0.00835937823371628	FKBP5
Recoverec	121	2.82768696520666e-07	0.64982443549005	0.564	0.368	0.0094834965439101	AL139807.1
Recoverec	121	2.88189754942238e-07	0.33681608074391	0.915	0.768	0.00966530800125276	IGKC
Recoverec	121	3.93525715049183e-07	0.29989471787428	0.188	0.066	0.0131980654313195	PID1
Recoverec	121	4.86438496165403e-07	0.34791486413858	0.214	0.083	0.0163141742843953	AC124014.1
Recoverec	121	6.03229018853775e-07	0.66262633044224	0.932	0.84	0.0202310948343179	RASGEF1B
Recoverec	121	6.55081227828254e-07	0.44837007381733	0.821	0.631	0.021970114218904	PRKCQ
Recoverec	121	6.67910658743815e-07	0.29046792745750	0.103	0.025	0.0224003876729501	LIMCH1
Recoverec	121	8.00165681130507e-07	0.35726140244488	0.197	0.073	0.026835956613755	PRTFDC1
Recoverec	121	8.25150727268266e-07	0.66836843982815	0.88	0.787	0.0276739050911231	SDCBP
Recoverec	121	9.75000334810435e-07	0.48151505005836	0.607	0.4	0.0326995612288724	EPAS1
Recoverec	121	9.77522078866966e-07	0.90851412935228	0.479	0.332	0.0327841354810403	SNAPC1
Recoverec	121	1.02029159669851e-06	0.44235783206143	0.872	0.72	0.0342185395700746	TBL1XR1
Recoverec	121	1.08512859079409e-06	0.36972728321538	0.915	0.812	0.0363930426780521	BIRC6
Recoverec	121	1.1965030427754e-06	0.45673576536812	0.769	0.584	0.0401283190486015	RICTOR
Recoverec	121	1.31405554552475e-06	0.36875202629963	0.846	0.706	0.0440707948858092	AAK1
Recoverec	121	1.32509103101295e-06	0.32770605218161	0.205	0.079	0.0444409029981123	ZNF346
Recoverec	183	2.00635463244243e-42	0.63227467868620	0.571	0.077	6.72891216628541e-38	NRG1
Recoverec	183	2.70782791081121e-36	0.42002623593365	0.414	0.047	9.08151324727865e-32	PRR16
Recoverec	183	5.22110756301795e-31	0.48849325830895	0.429	0.059	1.75105505448496e-26	COL3A1
Recoverec	183	2.72226047076902e-13	0.56918924683547	0.286	0.06	9.12991716686515e-09	SOX5
Recoverec	183	6.15130135972625e-13	0.39373263524278	0.214	0.036	2.06302345002499e-08	RGS5
Recoverec	183	6.7048889673078e-13	0.75722466471237	0.529	0.178	2.24868566185569e-08	TP53INP1

Recoverec	183	2.10015549248992e-12	0.80323407689140	0.971	0.731	7.04350149071269e-08	IKZF1
Recoverec	183	4.21599890880018e-12	0.60750606236715	0.529	0.184	1.4139617140334e-07	XAF1
Recoverec	183	1.26571432026245e-11	0.25252528308375	0.157	0.023	4.24495268729621e-07	SLCO2A1
Recoverec	183	1.58394699941619e-11	0.40503996867073	0.471	0.151	5.31224144664201e-07	DNAJC13
Recoverec	183	6.64834254406896e-11	0.39220387296918	0.214	0.044	2.22972112242985e-06	MICU3
Recoverec	183	1.04822266160208e-10	0.99312739355842	0.414	0.145	3.51552916248105e-06	XIST
Recoverec	183	2.23150183185599e-10	0.28822898999487	0.186	0.035	7.4840108436786e-06	TMTC1
Recoverec	183	3.74370842137078e-10	0.97204015050401	0.871	0.61	1.25556493035933e-05	FKBP5
Recoverec	183	3.84748585150645e-10	0.77869578375310	0.757	0.366	1.29036980487823e-05	AL139807.1
Recoverec	183	6.9010259819772e-10	1.13159761291231	0.857	0.621	2.31446609383551e-05	INPP4B
Recoverec	183	7.06913733949906e-10	0.80373893126564	0.857	0.567	2.37084728092119e-05	RAPGEF1
Recoverec	183	8.93134948839613e-10	0.30481853386088	0.271	0.07	2.9953959914183e-05	AC010260.1
Recoverec	183	9.47094625162118e-10	0.90829038632752	0.6	0.263	3.17636595386871e-05	EPSTI1
Recoverec	183	1.10573170215563e-09	0.69529528203931	0.971	0.859	3.70840298268954e-05	ESYT2
Recoverec	183	1.31628472338922e-09	1.0295478002669	0.929	0.722	4.41455570530276e-05	BCL2
Recoverec	183	1.70944151126479e-09	0.58504066649105	0.557	0.223	5.73312494047986e-05	MFHAS1
Recoverec	183	1.7822190242826e-09	0.91247449602342	0.857	0.587	5.97720616363899e-05	MDFIC
Recoverec	183	2.02595970565563e-09	1.05664640311048	0.729	0.437	6.79466366082787e-05	UBASH3B
Recoverec	183	2.1871525903497e-09	0.28635263799449	0.543	0.201	7.33527235751482e-05	AHSA1
Recoverec	183	3.09492841327431e-09	0.64200113163139	0.357	0.119	0.000103797709124394	ST8SIA1
Recoverec	183	3.27321077351006e-09	0.51413057646666	0.271	0.075	0.00010977694292198	IFI44L
Recoverec	183	4.15334202013474e-09	0.79397910458257	0.571	0.264	0.000139294784671279	TESPA1
Recoverec	183	6.50821193364417e-09	0.65784572563178	1	0.799	0.000218272411830558	PCNX1
Recoverec	183	6.75901709326358e-09	0.37767201483061	0.557	0.216	0.000226683915273874	AC092652.2
Recoverec	183	7.01303166817198e-09	0.47747217089538	0.486	0.188	0.000235203056087152	SAMD9L
Recoverec	183	8.27495564578365e-09	0.57789893963804	0.971	0.749	0.000277525462448292	DOCK2
Recoverec	183	8.9817436935325e-09	0.36093856997433	0.643	0.28	0.000301229719993693	FAM196B
Recoverec	183	1.01419994361193e-08	0.62669132365092	0.943	0.657	0.000340142377088569	CCND3
Recoverec	183	1.16705500036855e-08	0.27030093312149	0.457	0.165	0.000391406906023604	ATP11A
Recoverec	183	1.30414589926297e-08	0.56364591314717	0.643	0.302	0.000437384451694816	SMURF1
Recoverec	183	1.41090539173709e-08	0.36347453492347	0.429	0.156	0.000473189450280785	MRPS27

Recoverec	183	1.44739246456176e-08	0.293941386665633	0.657	0.279	0.000485426484764721	NLRC3
Recoverec	183	1.86747953040813e-08	0.332486444317331	0.371	0.128	0.00062631528490828	SLC38A9
Recoverec	183	1.8697934031344e-08	0.517856683319234	0.514	0.216	0.000627091311543215	LRIG1
Recoverec	183	1.93140351168794e-08	0.58099262922609	0.886	0.536	0.000647754109749901	MSI2
Recoverec	183	2.21332920608645e-08	0.502266335104894	0.986	0.956	0.000742306349137272	ARHGAP15
Recoverec	183	2.31273512920019e-08	0.52682582474115	0.557	0.24	0.00077564510763116	AC010609.1
Recoverec	183	2.65274633632775e-08	0.252789779810591	0.286	0.084	0.000889678066277601	AC124014.1
Recoverec	183	3.21324555606355e-08	0.297251141153179	0.686	0.305	0.00107765829459259	PIK3C3
Recoverec	183	3.34032361950822e-08	0.347546403862644	0.557	0.234	0.00112027773551067	CEACAM21
Recoverec	183	3.81026340301552e-08	0.707494826100196	0.943	0.599	0.00127788614010334	MAP3K5
Recoverec	183	4.82380542899147e-08	0.389518078523791	0.6	0.265	0.00161780786477516	POLR2J3.1
Recoverec	183	5.0207908157607e-08	0.354811990931844	0.471	0.186	0.00168387282378982	GARS
Recoverec	183	6.19036723481271e-08	0.811595291641134	0.829	0.572	0.00207612536321149	RIPOR2
Recoverec	183	6.56402263820856e-08	0.78106715422471	0.943	0.767	0.00220144191240239	CHST11
Recoverec	183	6.85249923866335e-08	0.4103690186297	0.657	0.327	0.00229819119466291	PI4KA
Recoverec	183	8.24418004822334e-08	0.31064386326135	0.4	0.15	0.00276493310457314	MTMR12
Recoverec	183	8.46894785709394e-08	0.688600334430999	0.514	0.228	0.00284031573231217	NDUFAF6
Recoverec	183	8.97131969369425e-08	0.54958054735057	0.871	0.531	0.00300880119887118	CHORDC1
Recoverec	183	9.36300959575386e-08	0.268324028566957	0.143	0.029	0.00314016615822393	EFCAB8
Recoverec	183	1.04648760322912e-07	0.643098088104547	0.986	0.988	0.00350971012370981	AC016831.7
Recoverec	183	1.13465262058156e-07	0.501176953438117	0.829	0.513	0.00380539795890645	STK39
Recoverec	183	1.19583100930084e-07	0.363718074810969	0.529	0.224	0.00401057803899316	CLSTN1
Recoverec	183	1.21611880378097e-07	0.26964600362509	0.371	0.135	0.00407861924412063	AP2A2
Recoverec	183	1.22406973124002e-07	0.331810592439811	0.314	0.104	0.00410528506463279	RFX2
Recoverec	183	1.26167667125161e-07	0.396959205758848	0.429	0.168	0.00423141122004365	GAN
Recoverec	183	1.31720661612376e-07	0.284081232627503	0.371	0.132	0.00441764754915585	MBNL1-AS1
Recoverec	183	1.42524774353975e-07	0.26593325701526	0.5	0.201	0.00477999588228363	SAMD4B
Recoverec	183	1.46039850393539e-07	0.546802102358509	0.986	0.931	0.00489788450249852	CELF2
Recoverec	183	1.56523053316747e-07	0.484621300865445	0.714	0.381	0.00524947016213705	RANBP9
Recoverec	183	1.69827711243678e-07	0.333330584505085	0.5	0.216	0.00569568177969048	ARID5A
Recoverec	183	1.90016732114082e-07	0.526630495689553	0.414	0.168	0.00637278116164207	PAQR3

Recoverec	183	1.95784398400322e-07	0.57615240862887(	0.657	0.345	0.00656621715355	DIP2B
Recoverec	183	2.10076150520321e-07	0.92621463024627	0.814	0.573	0.00704553393615053	AC009313.1
Recoverec	183	2.46445410484858e-07	0.26332648682520(	0.329	0.114	0.00826528617684118	AC092683.1
Recoverec	183	2.47797228581728e-07	0.38930336642920	0.486	0.207	0.00831062345217401	HK1
Recoverec	183	2.75941283754431e-07	0.30773595967867	0.486	0.206	0.0092545187745561	YEATS2
Recoverec	183	3.0457761020463e-07	0.69179138472323	0.971	0.754	0.0102149238910429	SSH2
Recoverec	183	3.05730878245019e-07	0.55777512033855	0.143	0.031	0.0102536021945814	TOX2
Recoverec	183	3.33435611443765e-07	0.56537360994655	0.986	0.875	0.011182763536601	PRKCH
Recoverec	183	3.43075373548992e-07	0.41348665443240	0.871	0.506	0.0115060618780861	HBP1
Recoverec	183	3.70005503837335e-07	0.48474828758826	0.743	0.401	0.0124092445876965	LRCH1
Recoverec	183	4.09827654512944e-07	0.40622862948707	0.357	0.132	0.0137447998770551	DEAF1
Recoverec	183	4.10444152630277e-07	0.70243011997972	0.571	0.28	0.0137654759909142	AC104365.1
Recoverec	183	4.11493185231758e-07	0.56373999329613	0.6	0.304	0.0138006584463027	SAMHD1
Recoverec	183	4.2362086239582e-07	0.51572034975893	0.929	0.727	0.014207396483031	UBAC2
Recoverec	183	4.27919116067564e-07	0.48772778403926	0.829	0.476	0.014351551314674	NCK2
Recoverec	183	4.53461240220135e-07	0.44221173244740	0.457	0.198	0.0152081830745029	VSIR
Recoverec	183	4.967716222397e-07	0.27465162676062	0.386	0.145	0.0166607266666751	ELP3
Recoverec	183	4.98504355540703e-07	0.62632552521993	0.9	0.752	0.0167188390761241	NEDD9
Recoverec	183	4.98557253414297e-07	0.44176536039450	0.414	0.171	0.0167206131650087	XPO7
Recoverec	183	4.98679376570928e-07	0.30701845952100	0.429	0.174	0.0167247089314358	EXOC5
Recoverec	183	5.05779063991615e-07	0.78852774205119	0.857	0.583	0.0169628182481508	ITK
Recoverec	183	5.39996588680664e-07	0.50394681607848	0.929	0.601	0.0181104055911721	SEPT6
Recoverec	183	5.51394866393342e-07	0.27963724342369	0.414	0.164	0.0184926810290999	FAM160B1
Recoverec	183	5.51933837082795e-07	0.36141380799682	0.414	0.169	0.0185107570280828	SYNJ2
Recoverec	183	5.91223025406916e-07	0.51776575627516	0.686	0.37	0.0198284378260971	SNX25
Recoverec	183	6.18916731819845e-07	0.89532392482255	0.886	0.664	0.020757229351774	NAMPT
Recoverec	183	6.41336795358781e-07	0.30022090519369(	0.486	0.209	0.0215091534427428	FBXO28
Recoverec	183	6.66908238491483e-07	0.27269677096334	0.243	0.074	0.0223667685025274	UBE2E2
Recoverec	183	6.68557868308304e-07	0.57242932813530	0.786	0.506	0.0224220937873239	INPP5D
Recoverec	183	6.72081097658338e-07	0.68122278228827	0.314	0.118	0.0225402558532653	TMEM200A
Recoverec	183	6.78050692753229e-07	0.4526077644003	0.529	0.257	0.0227404641335578	FAM168A

Recoverec	183	7.19031707941364e-07	0.37087257956600ŧ	0.657	0.323	0.0241148854209375	XPO4
Recoverec	183	7.26225474921267e-07	0.47732967259720	0.529	0.25	0.0243561499779094	ABHD15-AS1
Recoverec	183	7.67621637815534e-07	0.56103326246739	0.586	0.312	0.0257444944890574	RNF144A
Recoverec	183	7.86658304320147e-07	0.47686964727908	0.943	0.744	0.0263829462102891	RNF213
Recoverec	183	8.43330046832873e-07	0.28577453862470	0.243	0.076	0.0282836031106809	NEMP2
Recoverec	183	8.7211318038097e-07	0.40558008943454	0.614	0.312	0.029248931843617	CRTC3
Recoverec	183	9.34959297841052e-07	0.47957049279075	0.714	0.407	0.0313566649309932	SMAD3
Recoverec	183	9.39705912041858e-07	0.54651452789174	0.886	0.619	0.0315158568780598	TNIK
Recoverec	183	9.58806961494359e-07	0.52208372469272	0.471	0.221	0.0321564678745978	ZC3H12D
Recoverec	183	9.72908236078256e-07	0.47158004535844	0.557	0.278	0.0326293964215926	CLEC16A
Recoverec	183	9.8408744832301e-07	0.46095423750853	0.629	0.342	0.0330043248418571	HMBOX1
Recoverec	183	1.03183296268152e-06	0.74599369669431	0.9	0.696	0.0346056139024128	PACS1
Recoverec	183	1.12932377775072e-06	0.47598774684626	0.971	0.845	0.0378752608582035	SYNE2
Recoverec	183	1.17723043528703e-06	0.61668492587608	0.943	0.722	0.0394819543386564	ZNF609
Recoverec	183	1.183352616748e-06	0.25066548475729	0.586	0.267	0.0396872800604945	JAK3
Recoverec	183	1.21632362420188e-06	0.25404594368351	0.186	0.051	0.0407930617084825	SGPP2
Recoverec	183	1.22571703966833e-06	0.44872369991971	0.6	0.297	0.0411080980763965	KLF9
Recoverec	183	1.2599022047266e-06	1.00068495257076	0.743	0.485	0.0422546001421208	SERINC5
Recoverec	183	1.26420481057443e-06	0.37965275769372	0.6	0.294	0.0423989009370453	CDK5RAP2
Recoverec	183	1.27250308555524e-06	0.55598080809256	0.343	0.139	0.0426772084833516	CD40LG
Recoverec	183	1.31739133867971e-06	0.31195607777973	0.243	0.078	0.0441826707166402	PRKX-AS1
Recoverec	183	1.34915510249716e-06	0.81355446949147	0.329	0.132	0.0452479638275498	TNFSF8
Recoverec	183	1.36450311817777e-06	0.50893926592477	0.943	0.793	0.0457627055774459	MED13L
Recoverec	183	1.38579038008449e-06	0.28023521503783	0.5	0.218	0.0464766377672735	RRP1B
Recoverec	183	1.41856174769134e-06	0.34001847192992	0.386	0.156	0.0475757238940721	FARP2
Rejected	0	7.54529003627085e-29	1.89305235445758	0.82	0.049	2.53053937236452e-29	REG1B
Rejected	0	3.52174074465175e-10	1.45783803593491	0.996	0.841	1.18112141094131e-104	IGKC
Rejected	0	8.15619360407802e-10	1.52615665227564	0.989	0.603	2.73542421093569e-10	PHGR1
Rejected	0	5.51882376577792e-99	1.48424509324577	1	0.718	1.8509031145666e-94	FABP6
Rejected	0	4.95141137757644e-88	1.35243412454706	0.923	0.432	1.66060434781159e-83	APOA4
Rejected	0	2.17598932767831e-79	0.80768264385474	0.471	0.08	7.29783300716751e-75	CYP3A4

Rejected	0	5.03902004428329e-75	1.17566186479805	0.805	0.33	1.68998654245173e-70	APOC3
Rejected	0	6.78870619684284e-74	1.67782165016809	0.785	0.292	2.27679628429715e-69	HSPA1B
Rejected	0	8.74473325271182e-74	1.00122565014678	0.594	0.146	2.93280863829449e-69	GUCA2A
Rejected	0	8.8824268368503e-71	1.08383783381863	0.64	0.188	2.97898831254285e-66	PDE3A
Rejected	0	4.76872126476307e-67	1.10669236040296	0.893	0.452	1.59933373777624e-62	ALDOB
Rejected	0	2.20417190012646e-65	1.01600491860427	0.946	0.54	7.39235171864414e-61	REG1A
Rejected	0	1.07554270536352e-60	1.02805365240911	0.67	0.242	3.60715512524817e-56	KRT19
Rejected	0	2.27084203641211e-58	0.900387118639464	0.67	0.232	7.61595002171892e-54	GDA
Rejected	0	1.56427290782187e-54	1.79792751272794	0.793	0.417	5.24625847825297e-50	HSPA1A
Rejected	0	3.51703107621467e-54	1.42977046399983	0.931	0.769	1.17954188234088e-49	TXNIP
Rejected	0	2.80846468847402e-52	0.856074271431597	0.782	0.348	9.41902887220417e-48	KRT8
Rejected	0	6.04230062707657e-52	0.945486057101004	0.866	0.456	2.02646678430894e-47	FABP1
Rejected	0	6.9516932094531e-51	1.05960431137922	0.946	0.871	2.33145886858638e-46	RPS29
Rejected	0	7.26877471347276e-51	1.20358527447846	0.992	0.939	2.4378016634045e-46	HSP90AA1
Rejected	0	1.48794270958362e-49	0.867120038396835	0.559	0.182	4.99026225940155e-45	SLC5A1
Rejected	0	1.07666820045121e-47	0.764807281587773	0.605	0.21	3.61092981067326e-43	RBP2
Rejected	0	4.78497495550055e-46	0.916967643066634	0.931	0.648	1.60478490057577e-41	AC105402.3
Rejected	0	2.78156851025433e-45	1.41129428993938	0.897	0.651	9.32882446969098e-41	DNAJB1
Rejected	0	2.65579396265127e-44	0.943566970169578	0.92	0.812	8.90700179193985e-40	RPS21
Rejected	0	3.62800182117197e-44	0.529774728054627	1	1	1.21675925078465e-39	MT-CO2
Rejected	0	5.05017817596759e-44	1.25944889425561	0.862	0.561	1.69372875665601e-39	HSPH1
Rejected	0	6.97958896519139e-44	0.645057007334179	0.448	0.125	2.34081454714589e-39	AMN
Rejected	0	8.80724104182521e-44	0.805875866794646	0.556	0.195	2.95377250060734e-39	FGD4
Rejected	0	2.27156928082573e-43	0.658061035621159	0.487	0.149	7.61838905403333e-39	CLDN4
Rejected	0	8.48370005732756e-43	0.504874366338817	0.36	0.081	2.84526332522652e-38	CLDN3
Rejected	0	1.36548942608751e-42	0.687966348543573	0.51	0.162	4.57957843721229e-38	MUC17
Rejected	0	8.39794852243205e-42	0.795745946255035	0.697	0.323	2.81650397545326e-37	SELENOP
Rejected	0	1.00722103613745e-40	0.764015809976915	0.766	0.38	3.37801791099777e-36	JCHAIN
Rejected	0	1.03363714204459e-40	0.874083434138915	0.977	0.932	3.46661224698915e-36	RPS27
Rejected	0	3.40654985419871e-40	0.821569798312138	0.935	0.901	1.14248869010116e-35	RPL37
Rejected	0	4.93440325464254e-39	0.860791211050611	0.962	0.764	1.65490016354201e-34	MTRNR2L8

Rejected	0	9.15741551313746e-39	0.738283663982679	0.617	0.25	3.07121401479604e-34	CYP3A5
Rejected	0	1.79114443589156e-38	0.83513528073847	1	0.994	6.00714020909311e-34	MT-ND3
Rejected	0	2.82065731610297e-38	1.27690212827909	0.831	0.586	9.45992050674615e-34	ZNF331
Rejected	0	3.09041239308243e-38	1.91774959004789	0.824	0.527	1.03646250839199e-33	ATP1B3
Rejected	0	8.3762665207592e-38	0.599211159887438	0.502	0.167	2.80923226573222e-33	ADIRF
Rejected	0	1.9443534924504e-37	0.783502046977408	0.743	0.37	6.52097274298016e-33	PCSK5
Rejected	0	3.88682140949509e-37	0.674365702495039	0.973	0.884	1.30356216431646e-32	FTL
Rejected	0	5.04377536909906e-36	0.772331295487397	0.992	0.933	1.69158138328844e-31	RPL39
Rejected	0	8.9559724033719e-36	0.78803200333177	0.962	0.918	3.00365402464287e-31	RPS4X
Rejected	0	1.67430367828661e-35	0.786137879056862	0.977	0.934	5.61527967623764e-31	RPL13
Rejected	0	3.16454153858633e-35	0.361903596691902	0.157	0.017	1.06132394121108e-30	MS4A1
Rejected	0	4.58568409910506e-35	0.310388667120363	0.13	0.011	1.53794673315785e-30	CD79A
Rejected	0	5.77321652313025e-35	0.786762643118517	0.962	0.928	1.93622135752742e-30	RPS18
Rejected	0	6.18884243788967e-35	0.388558485910669	0.264	0.053	2.07561397681944e-30	FXYD3
Rejected	0	8.2035300282061e-35	0.603241583081889	0.517	0.193	2.75129990085976e-30	LGALS4
Rejected	0	8.22928289735499e-35	0.426240074614862	0.253	0.049	2.75993689811492e-30	TM4SF20
Rejected	0	3.14124831514393e-34	0.630653208909493	0.923	0.656	1.05351185993297e-29	IGHA1
Rejected	0	3.17817693652874e-34	0.496572894553564	0.333	0.087	1.06589698097301e-29	HHLA2
Rejected	0	1.04377816023614e-33	0.551715274171133	0.429	0.142	3.50062319379996e-29	SMIM24
Rejected	0	1.96469526235746e-33	0.501406400208285	0.31	0.077	6.58919497089445e-29	SERPINA1
Rejected	0	9.80030876484637e-33	0.586529430321017	0.345	0.097	3.28682755355418e-28	CDHR2
Rejected	0	1.40960832126926e-32	0.800024928500494	0.943	0.923	4.72754438787285e-28	RPL34
Rejected	0	2.07013650807851e-32	0.608994225194649	0.441	0.154	6.94282382079371e-28	MUC13
Rejected	0	2.73129188510972e-32	0.789668442980648	0.962	0.912	9.16020672428097e-28	RPS8
Rejected	0	6.50935582128431e-32	0.713422855677794	0.973	0.921	2.18310775534233e-27	RPL11
Rejected	0	6.74585413880934e-32	0.707019194189466	0.977	0.919	2.26242456107388e-27	RPS15A
Rejected	0	9.88383741036371e-32	0.48452507511486	0.36	0.104	3.31484139068778e-27	PDE9A
Rejected	0	1.32990403981685e-31	0.598220538533244	0.414	0.139	4.46023216873775e-27	ELF3
Rejected	0	1.5023167291377e-31	0.555835296704344	0.418	0.14	5.038469846182e-27	TMC5
Rejected	0	2.24736955316282e-31	0.419347685181359	1	1	7.53722800739747e-27	MT-CO3
Rejected	0	7.02194636089862e-31	0.80037150120428	0.943	0.892	2.35502037051818e-26	RPL21

Rejected	0	3.37572708449621e-30	0.474869080900447	0.368	0.113	1.13215134959834e-25	DPEP1
Rejected	0	4.46712553649341e-30	0.692142512726318	0.602	0.282	1.49818456242916e-25	FABP2
Rejected	0	4.73221832039827e-30	0.650862416133046	0.977	0.932	1.58709138029517e-25	RPS28
Rejected	0	5.77097839091615e-30	1.21275580427934	0.87	0.747	1.93547073274546e-25	JUNB
Rejected	0	7.73381143304147e-30	0.652614216184188	0.977	0.874	2.59376567841345e-25	RPL8
Rejected	0	1.05065544005505e-29	0.58569259375181	0.51	0.207	3.52368821485662e-25	SLC15A1
Rejected	0	1.57320557485154e-29	0.73850892710243	0.912	0.871	5.27621685693709e-25	RPL35A
Rejected	0	1.78266644599707e-29	1.03926415643034	0.923	0.82	5.97870672658499e-25	DUSP1
Rejected	0	2.67516656491011e-29	0.830391101885797	0.506	0.209	8.97197362539552e-25	HLA-DRA
Rejected	0	3.01621632845952e-29	0.806262253593795	0.889	0.743	1.01157863223875e-24	UBB
Rejected	0	3.20960982388817e-29	0.676840939515742	0.943	0.895	1.07643894273562e-24	RPL29
Rejected	0	3.63362220027351e-29	0.316766780641439	0.226	0.046	1.21864421352773e-24	MDK
Rejected	0	8.91450610357451e-29	0.567711459570225	0.502	0.201	2.98974705701682e-24	CDHR5
Rejected	0	1.11279406316643e-28	0.630029060642292	0.414	0.151	3.73208872904758e-24	DNAJB4
Rejected	0	5.64192571108652e-28	0.431542923756825	0.284	0.074	1.8921890449842e-23	PRAP1
Rejected	0	6.88203980120703e-28	0.618821385139447	0.954	0.91	2.30809850852882e-23	RPLP2
Rejected	0	7.73499642242727e-28	0.715748644101416	0.95	0.909	2.59416310015366e-23	RPL32
Rejected	0	8.28261352447629e-28	0.631608828762617	0.973	0.936	2.77782292383886e-23	RPL41
Rejected	0	1.53295160074172e-27	0.606966178774258	0.199	0.039	5.14121307856759e-23	BANK1
Rejected	0	1.91123298276179e-27	0.350574025616412	0.264	0.067	6.40989317758649e-23	TMPRSS2
Rejected	0	1.99190708917313e-27	0.681467049587178	0.946	0.907	6.68045799566885e-23	RPL30
Rejected	0	3.7322361604284e-27	0.706978717275409	0.881	0.832	1.25171736348448e-22	RPL22
Rejected	0	3.85153911107418e-27	0.684714731235224	0.954	0.9	1.29172918707206e-22	RPL3
Rejected	0	4.04368642160531e-27	0.363429624604336	0.241	0.056	1.35617155207799e-22	SLC6A19
Rejected	0	4.4850348593444e-27	0.706773932577163	0.985	0.937	1.50419099112693e-22	RPS12
Rejected	0	5.5925556241022e-27	0.680904519023682	0.893	0.85	1.8756313052114e-22	RPL36
Rejected	0	1.11469225391518e-26	0.484995908726107	0.337	0.105	3.73845488118073e-22	FRMD3
Rejected	0	1.50649570704897e-26	0.745897157655411	0.762	0.503	5.05248530230083e-22	HSPE1
Rejected	0	1.76164196671515e-26	0.619269290897599	0.946	0.906	5.90819482796928e-22	RPS23
Rejected	0	2.18195005773223e-26	0.581682530851963	0.72	0.417	7.31782410362235e-22	ATP5ME
Rejected	0	2.84324916412273e-26	0.587220562668403	0.973	0.929	9.5356890466348e-22	RPL28

Rejected	0	4.67973038602501e-26	0.376480816778922	0.272	0.073	1.56948797686507e-21	PRSS3
Rejected	0	5.93888696384844e-26	0.657999242695674	0.985	0.927	1.99178390993549e-21	RPS14
Rejected	0	1.70093132691923e-25	0.660341172534627	0.939	0.832	5.70458348422173e-21	UBC
Rejected	0	1.80804695939502e-25	0.643941539346772	0.946	0.88	6.06382789241903e-21	RPL19
Rejected	0	2.52251423109648e-25	0.749818712467947	0.992	0.94	8.46000822825138e-21	BTG1
Rejected	0	3.33383807657321e-25	0.622682736735962	0.989	0.96	1.11810261412112e-20	RPLP1
Rejected	0	4.28770048042891e-25	0.54188853626748	0.421	0.164	1.43800898712625e-20	EPB41L3
Rejected	0	7.26623776047546e-25	0.604345913031837	0.939	0.891	2.43695082010826e-20	RPS7
Rejected	0	8.15670695993373e-25	0.595809617511277	0.985	0.93	2.73559638022257e-20	RPS19
Rejected	0	1.11499235430615e-24	0.4785867062011	0.337	0.113	3.73946135787195e-20	SHROOM3
Rejected	0	1.47069154204891e-24	0.611231278537395	0.927	0.855	4.93240529372362e-20	UBA52
Rejected	0	1.58727639290064e-24	0.608751656857381	0.966	0.923	5.32340756651017e-20	RPL12
Rejected	0	1.61545443228058e-24	0.516791094681475	0.487	0.208	5.4179110749826e-20	TSPAN8
Rejected	0	1.9356646632986e-24	0.607929631611218	0.943	0.886	6.49183214777085e-20	RPL24
Rejected	0	2.76871246853228e-24	0.612681547884942	0.954	0.9	9.28570787696356e-20	RPL6
Rejected	0	2.77663870283211e-24	0.60330062431409	0.95	0.909	9.31229088155834e-20	RPS27A
Rejected	0	4.85071754276702e-24	0.548305691862164	0.582	0.296	1.6268336494932e-19	MALRD1
Rejected	0	5.27007215047104e-24	0.527557958930948	0.479	0.206	1.76747679782498e-19	NEDD4L
Rejected	0	9.32794192079696e-24	0.599677284820171	0.939	0.842	3.12840516139688e-19	RPL26
Rejected	0	9.3742584696508e-24	0.601283825814006	0.92	0.847	3.14393880555148e-19	RPL37A
Rejected	0	1.00536020825847e-23	0.295480405263425	0.123	0.017	3.37177706645727e-19	ZG16
Rejected	0	1.01625473239354e-23	0.624294193156629	0.663	0.377	3.40831512150147e-19	ANPEP
Rejected	0	1.10272095803232e-23	0.640327662336344	0.954	0.893	3.69830554904879e-19	RPL18A
Rejected	0	1.32490766116317e-23	0.297200098287567	0.134	0.02	4.44347531400904e-19	IGHG4
Rejected	0	1.59677735190941e-23	0.487381477622725	1	0.999	5.35527188283378e-19	MT-CYB
Rejected	0	1.78292761658793e-23	0.855263609316809	0.943	0.808	5.97958264051261e-19	CXCR4
Rejected	0	2.45902767102411e-23	0.523048643425045	0.513	0.232	8.24708700308066e-19	EPCAM
Rejected	0	2.48791173628049e-23	0.470486284409195	0.421	0.169	8.34395838113752e-19	CST3
Rejected	0	2.61839885349155e-23	0.578333835390287	0.977	0.906	8.78158607483996e-19	RPL9
Rejected	0	2.7085476034748e-23	0.550262654290508	0.801	0.502	9.08392695253379e-19	IGLC2
Rejected	0	3.01338210985592e-23	0.599606998719912	0.862	0.664	1.01062809200348e-18	CD52

Rejected	0	3.31249753983121e-23	0.470277458055078	0.364	0.133	1.11094542490859e-18	THSD4
Rejected	0	3.8453968226206e-23	0.405905242446947	0.337	0.116	1.2896691863705e-18	COBL
Rejected	0	3.88734215568502e-23	0.39440862295325	0.337	0.115	1.30373681217364e-18	NR1H4
Rejected	0	4.20018642501744e-23	0.686252323049756	0.847	0.636	1.40865852322235e-18	HSPD1
Rejected	0	5.83494359253823e-23	0.649835784343677	0.889	0.656	1.95692338206547e-18	MTRNR2L12
Rejected	0	1.45524916665054e-22	0.729347076626684	0.437	0.196	4.88061465511257e-18	HOOK2
Rejected	0	1.47226627514085e-22	0.776338887240207	0.789	0.541	4.93768663356738e-18	CD74
Rejected	0	1.50781998902907e-22	0.559490359575044	0.946	0.9	5.05692667920571e-18	RPS15
Rejected	0	2.58410000039263e-22	0.389467728292542	0.322	0.109	8.66655458131679e-18	NCKAP5
Rejected	0	1.06153621414811e-21	0.444441549634075	0.349	0.128	3.56018015500992e-17	PCK1
Rejected	0	1.06186668399183e-21	0.550701884949939	0.372	0.145	3.56128848477179e-17	PMP22
Rejected	0	1.19689241173578e-21	0.376417456913662	0.264	0.08	4.01413777047944e-17	HLA-DQB1
Rejected	0	1.27279915174811e-21	0.3430979438683	0.238	0.067	4.26871379513282e-17	SLC2A2
Rejected	0	1.37102914694798e-21	0.732059816253853	0.962	0.894	4.59815755303413e-17	H3F3B
Rejected	0	1.44006621619543e-21	0.568316556895303	0.931	0.893	4.82969407587623e-17	RPS11
Rejected	0	1.52344839259213e-21	0.758022230283404	0.28	0.087	5.10934121907549e-17	IKZF2
Rejected	0	1.58078446205065e-21	0.601844258211132	0.874	0.76	5.30163492882546e-17	RPL31
Rejected	0	1.58233022496553e-21	0.351887104675089	0.249	0.072	5.30681910848938e-17	HLA-DRB5
Rejected	0	2.39299347071761e-21	0.591338412046587	0.935	0.889	8.02562150209272e-17	RPS13
Rejected	0	2.83739315315623e-21	0.594492943804743	0.345	0.126	9.51604915705536e-17	LYN
Rejected	0	3.4424673135619e-21	0.339016199356385	0.307	0.103	1.15453468762239e-16	CDH17
Rejected	0	4.27196208347259e-21	0.552756385618452	0.931	0.863	1.43273064355504e-16	NACA
Rejected	0	5.45670584143684e-21	0.691037788764001	0.567	0.296	1.83007000510109e-16	PRMT9
Rejected	0	9.13040262845096e-21	0.290520566455528	0.207	0.053	3.06215443352988e-16	GUCA2B
Rejected	0	1.14055439210633e-20	0.407840427780032	0.429	0.18	3.82519132024619e-16	GSTA1
Rejected	0	1.67207902468341e-20	0.484170305393896	0.364	0.144	5.60781863298322e-16	C9orf72
Rejected	0	1.98806314219584e-20	0.386709492004636	0.287	0.095	6.6675661662964e-16	CXADR
Rejected	0	2.25959507957891e-20	0.497505254736185	0.939	0.897	7.57822997789176e-16	RPL15
Rejected	0	2.29306294046326e-20	0.557789485534222	0.923	0.869	7.69047448972568e-16	RPL35
Rejected	0	2.38618290072066e-20	0.917956068822537	0.705	0.49	8.00278021243695e-16	JMY
Rejected	0	2.81893948603902e-20	0.555300942624243	0.935	0.852	9.45415924827767e-16	FAU

Rejected	0	3.47550370853987e-20	0.420976004447075	0.291	0.102	1.1656144337701e-15	CYBRD1
Rejected	0	4.0843895968903e-20	0.535837658954666	0.992	0.947	1.36982258300507e-15	RPL10
Rejected	0	8.99895995665925e-20	0.542094242490689	0.854	0.717	3.01807119026438e-15	TOMM7
Rejected	0	9.04811972696535e-20	0.498441371943986	0.663	0.383	3.03455839402964e-15	LGALS3
Rejected	0	1.16404682840584e-19	0.572838524666079	0.889	0.844	3.9039802531075e-15	RPL10A
Rejected	0	1.69575320041799e-19	0.276945699259661	0.153	0.032	5.68721708356185e-15	SFN
Rejected	0	2.16472557384712e-19	0.527246004678525	0.904	0.85	7.26005662956848e-15	RPL23A
Rejected	0	2.53255151024066e-19	0.536517968905203	0.234	0.071	8.49367125504513e-15	ARHGAP24
Rejected	0	2.68792930485998e-19	0.458671399655201	0.387	0.165	9.0147773026394e-15	SGK1
Rejected	0	3.38521164407914e-19	0.581976857067209	0.651	0.402	1.13533228119126e-14	PIK3IP1
Rejected	0	3.59297027778953e-19	0.5248382077516	0.885	0.775	1.20501037176505e-14	PFDN5
Rejected	0	3.79396893380876e-19	0.500108399767578	0.996	0.974	1.27242130102078e-14	TPT1
Rejected	0	4.32091234042965e-19	0.485522501759464	0.448	0.207	1.4491475807333e-14	CARMIL1
Rejected	0	4.52967053869305e-19	0.745458110534614	0.613	0.368	1.51916090526688e-14	NR4A1
Rejected	0	4.59140469396349e-19	0.544041197217283	0.946	0.88	1.53986530626147e-14	RPL27A
Rejected	0	4.61162751872216e-19	0.506523980689544	0.962	0.918	1.54664763722904e-14	RPS3A
Rejected	0	5.52095381726853e-19	0.419193081346129	0.375	0.155	1.85161749123552e-14	MME
Rejected	0	6.93436736572497e-19	0.680264256591284	0.548	0.312	2.32564812711684e-14	DDIT4
Rejected	0	7.04557374691755e-19	0.386063817526905	0.226	0.068	2.36294452324121e-14	SLC7A9
Rejected	0	7.17625850562078e-19	0.583733199749534	0.356	0.144	2.4067735776151e-14	CCR6
Rejected	0	8.27148357489933e-19	0.497348837760644	0.954	0.904	2.77409016134974e-14	RPL18
Rejected	0	8.48141873086005e-19	0.372192277221367	0.257	0.085	2.84449821395584e-14	ST14
Rejected	0	8.58863124452398e-19	0.504252928204337	0.828	0.62	2.88045514678845e-14	CIRBP
Rejected	0	9.42950794639842e-19	0.381034534041108	0.287	0.103	3.1624683750631e-14	ERRFI1
Rejected	0	9.535043000856e-19	0.354071218876121	0.349	0.14	3.19786272162708e-14	FCGRT
Rejected	0	9.57023193946272e-19	0.72562786945504	0.858	0.691	3.20966438785701e-14	NR4A2
Rejected	0	1.03871218418731e-18	1.13979242010491	0.582	0.345	3.48363292332742e-14	LTB
Rejected	0	1.05317192276913e-18	0.479295081964003	0.379	0.162	3.53212799458312e-14	HLA-DRB1
Rejected	0	1.22259272707257e-18	0.504537912189682	0.502	0.262	4.10033148805599e-14	LMO7
Rejected	0	2.02091532486316e-18	0.59915513795249	0.249	0.081	6.77774581652606e-14	CD160
Rejected	0	2.63642198675543e-18	0.545401140122865	0.709	0.476	8.84203205918037e-14	LAPTM5

Rejected	0	2.81907428195904e-18	0.458469483411989	0.372	0.156	9.45461132683424e-14	AC026979.2
Rejected	0	3.70697366479622e-18	0.266962906840765	0.203	0.057	1.24324482769935e-13	SLC1A1
Rejected	0	4.08394581825165e-18	0.447080677071923	0.839	0.657	1.36967374852524e-13	ZFAS1
Rejected	0	5.03956681001795e-18	0.297352591946735	0.215	0.063	1.69016991674382e-13	STXBP6
Rejected	0	8.12160905652351e-18	0.323526526725381	0.23	0.071	2.72382524537685e-13	AGR3
Rejected	0	9.32975511259431e-18	0.500307280083209	0.893	0.828	3.12901326966188e-13	RPL7
Rejected	0	9.76983672995814e-18	0.267041890851544	0.211	0.062	3.27660784249336e-13	SPINK1
Rejected	0	1.10006846958339e-17	0.423746160618466	0.345	0.143	3.68940963328878e-13	MAOA
Rejected	0	1.61843760979897e-17	0.57920241036606	0.851	0.781	5.4279160557438e-13	EEF1D
Rejected	0	1.62067590600432e-17	0.796725433904304	0.962	0.874	5.4354228535573e-13	JUN
Rejected	0	2.37460453330898e-17	0.51663451076475	0.529	0.29	7.96394868381166e-13	ACSL5
Rejected	0	2.46026518964547e-17	0.549296556342441	0.874	0.815	8.25123739303296e-13	RPL23
Rejected	0	2.6218583404829e-17	0.380904570340507	0.414	0.188	8.79318850231154e-13	SOX6
Rejected	0	2.73272155891653e-17	0.429783136931251	0.49	0.25	9.16500156429427e-13	CYB5A
Rejected	0	3.05696082501023e-17	0.461453377077163	0.517	0.273	1.02524352149193e-12	PRKG1
Rejected	0	3.43153842428905e-17	0.411548668187534	0.341	0.145	1.15086935673806e-12	KRT20
Rejected	0	4.31031229604192e-17	0.292028841000924	0.241	0.08	1.44559253784654e-12	BTNL8
Rejected	0	4.37597011363007e-17	0.262633083632267	0.13	0.027	1.46761285670925e-12	HES4
Rejected	0	6.80188065792062e-17	0.529009392342381	0.862	0.771	2.28121473505342e-12	RPL38
Rejected	0	9.91138965121105e-17	0.453571790643843	0.759	0.533	3.32408186122316e-12	PLCG2
Rejected	0	1.22400879235361e-16	0.356806869219156	0.674	0.409	4.10508068779554e-12	IGLC3
Rejected	0	1.29012364259022e-16	0.455570861112229	0.368	0.162	4.32681667251907e-12	CKS2
Rejected	0	1.85494456762331e-16	0.447918433037534	0.448	0.223	6.22111309089504e-12	EPS8
Rejected	0	1.95591998522702e-16	0.492050543972502	0.969	0.952	6.55976444645438e-12	TMSB4X
Rejected	0	1.9975695305355e-16	0.465554496972554	0.95	0.904	6.69944869150998e-12	RPS16
Rejected	0	2.36371649708167e-16	0.516887729717778	0.931	0.853	7.9274323879125e-12	RACK1
Rejected	0	3.14628423654107e-16	0.351183453485365	0.287	0.112	1.05520080725114e-11	HKDC1
Rejected	0	4.08658345827336e-16	0.455326526293879	0.927	0.888	1.37055836023572e-11	RPS20
Rejected	0	6.75720209418006e-16	0.499657110479714	0.897	0.834	2.26623043834611e-11	RPS5
Rejected	0	1.32549534096311e-15	0.498053315841489	0.946	0.897	4.44544627452207e-11	RPS25
Rejected	0	1.32601136660288e-15	0.474396980095704	0.613	0.39	4.44717692131275e-11	SNHG8

Rejected	0	1.33478020692166e-15	0.42120372863082	0.958	0.9	4.47658585797388e-11	RPL7A
Rejected	0	1.44691815499175e-15	0.29259900147843	0.238	0.083	4.85267410821132e-11	SLC5A12
Rejected	0	1.50282743364253e-15	0.51477652311950	0.831	0.687	5.0401826469503e-11	COX4I1
Rejected	0	1.65710068336388e-15	0.58932505724108	0.985	0.928	5.55758427186578e-11	CD69
Rejected	0	1.68868097922455e-15	0.47257453565510	0.828	0.721	5.66349826812329e-11	COX7C
Rejected	0	1.71242057945149e-15	0.41093965659245	0.253	0.094	5.7431161393644e-11	HEPH
Rejected	0	2.5597211142531e-15	0.44372547576436	0.897	0.755	8.58479267298205e-11	SRSF3
Rejected	0	3.11378954914488e-15	0.35586809865208	0.307	0.126	1.04430273899221e-10	RHOB
Rejected	0	3.21864956749258e-15	0.32341328210693	0.295	0.12	1.07947069194566e-10	CDH1
Rejected	0	3.425529733585e-15	0.32668040424801	0.552	0.31	1.14885416204974e-10	MYO1E
Rejected	0	4.64220133262211e-15	0.28540508981769	0.153	0.04	1.5569014829348e-10	ADAM28
Rejected	0	5.5017920141077e-15	0.82141262914562	0.782	0.658	1.84519100569144e-10	ZFP36
Rejected	0	8.61366451070753e-15	0.45383894454721	0.985	0.943	2.88885080360109e-10	RPS2
Rejected	0	1.01614263145242e-14	0.38994736527761	0.471	0.249	3.40793915736513e-10	CHCHD10
Rejected	0	1.01893204239729e-14	0.45463157884266	0.943	0.897	3.41729428379203e-10	RPS9
Rejected	0	1.14609021526605e-14	0.42628778281281	0.954	0.917	3.84375736395927e-10	RPS3
Rejected	0	1.16216804492615e-14	0.35208957515363	0.375	0.176	3.89767918907333e-10	C15orf48
Rejected	0	1.20287777746629e-14	0.298803111960113	0.195	0.063	4.03421149006645e-10	KHK
Rejected	0	1.23021723440046e-14	0.42851809988476	0.398	0.195	4.12590256073227e-10	SOCS1
Rejected	0	1.72558023008759e-14	0.55983693068967	0.843	0.775	5.78725097566776e-10	EEF1B2
Rejected	0	1.88260252245993e-14	0.93229553782577	0.82	0.711	6.31387233982613e-10	CCL5
Rejected	0	1.97924650053695e-14	0.33555463044542	0.341	0.154	6.63799691350083e-10	RHOC
Rejected	0	2.1752597534231e-14	0.45539139064326	0.663	0.435	7.2953861610304e-10	EDF1
Rejected	0	3.0238943494788e-14	0.49760880970364	0.992	0.948	1.0141536869282e-09	FTH1
Rejected	0	3.22797057406623e-14	0.31741152993485	0.31	0.136	1.08259677113033e-09	ITM2C
Rejected	0	3.81997997567625e-14	0.62803576328953	0.502	0.305	1.2811448842423e-09	UBE2S
Rejected	0	3.84846248317984e-14	0.44632529749974	0.946	0.864	1.29069734760886e-09	HLA-A
Rejected	0	4.03080837649488e-14	0.52520650578456	0.513	0.31	1.35185251330885e-09	CHMP1B
Rejected	0	4.30208763289871e-14	0.40337209327375	0.475	0.265	1.44283415032157e-09	GPX4
Rejected	0	6.37050198703326e-14	0.33910193272922	0.333	0.151	2.13653895641121e-09	LARGE1
Rejected	0	6.93012024436266e-14	0.39273821868822	0.981	0.952	2.32422372755435e-09	SRGN

Rejected	0	7.99282290813957e-14	0.3431681953173	0.268	0.11	2.68063294693185e-09	ANKRD37
Rejected	0	8.85445145591328e-14	0.460929865976552	0.95	0.893	2.96960592928420e-09	SRSF7
Rejected	0	8.9662338697284e-14	0.626222399243201	0.598	0.391	3.00709551522951e-09	GADD45B
Rejected	0	1.05412333521247e-13	0.641364783274586	0.916	0.823	3.53531884163558e-09	CCNH
Rejected	0	1.25406495541675e-13	0.253110685709022	0.134	0.035	4.2058830474767e-09	FAM3D
Rejected	0	1.28943993228859e-13	0.349233706871182	0.375	0.176	4.32452364490949e-09	RGPD2
Rejected	0	1.49018779458869e-13	0.581348770533523	0.927	0.9	4.99779182549153e-09	TSC22D3
Rejected	0	1.50520654870062e-13	0.302128808009501	0.529	0.291	5.04816172303213e-09	APOB
Rejected	0	1.88146280677677e-13	0.540234125680207	0.571	0.365	6.31004996136794e-09	TSPYL2
Rejected	0	1.969146930674e-13	0.40517148591145	0.632	0.422	6.60412497609446e-09	ATP5F1D
Rejected	0	2.48819554712613e-13	0.411222972524916	0.203	0.071	8.34491022595161e-09	TIAM1
Rejected	0	2.56587724212031e-13	0.420312620495391	0.885	0.721	8.6054390946231e-09	GAPDH
Rejected	0	2.85367826796082e-13	0.250927500626855	0.418	0.208	9.570666175087e-09	IFI27
Rejected	0	3.0272682175894e-13	0.388203222593186	0.716	0.481	1.01528521481513e-08	APOA1
Rejected	0	3.08929533299668e-13	0.28024891876935	0.241	0.093	1.03608786878043e-08	TMIGD1
Rejected	0	3.93026836015298e-13	0.756029714242905	0.793	0.662	1.31813340262811e-08	SLC2A3
Rejected	0	4.93257435886787e-13	0.423405144194168	0.946	0.924	1.65428678847711e-08	RPS6
Rejected	0	6.99546582738634e-13	0.332100017058531	0.172	0.056	2.34613932918883e-08	GNG7
Rejected	0	7.5127603089303e-13	0.512035367774674	0.349	0.171	2.51962955240904e-08	HLA-DPB1
Rejected	0	7.55282961710754e-13	0.348296737520172	0.333	0.16	2.53306799698553e-08	DEDD2
Rejected	0	7.84560827951113e-13	0.271897320662154	0.176	0.058	2.63126010478244e-08	SLC10A2
Rejected	0	8.27249022341979e-13	0.382950321845967	0.839	0.724	2.77442777113053e-08	ATP5MG
Rejected	0	1.07427823943317e-12	0.424719286512869	0.985	0.939	3.60291435941096e-08	ACTB
Rejected	0	1.21771243521624e-12	0.361203779768729	0.337	0.166	4.08396396522821e-08	H2AFJ
Rejected	0	1.23469196642271e-12	0.492380347260905	0.977	0.892	4.14090991698849e-08	EZR
Rejected	0	1.29298785529146e-12	0.7357559475476	0.33	0.16	4.3364226690765e-08	AC015849.1
Rejected	0	1.49634414447302e-12	0.329608588501935	0.345	0.167	5.01843899173362e-08	CCSER1
Rejected	0	1.59547384303273e-12	0.250260629569106	0.234	0.092	5.35090017476316e-08	MYO7B
Rejected	0	1.62159527442812e-12	0.26428438159674	0.291	0.129	5.43850623137701e-08	TNFRSF1A
Rejected	0	1.73975748712308e-12	0.300755900818391	0.28	0.123	5.83479866031339e-08	ACE
Rejected	0	1.83862449287384e-12	0.57087461485112	0.866	0.689	6.16637882420029e-08	CEMIP2

Rejected	0	2.15889394737213e-12	0.320977002455889	0.176	0.059	7.24049852069664e-08	EBF1
Rejected	0	2.18534345116109e-12	0.324703397797012	0.605	0.376	7.32920486650406e-08	UQCR11
Rejected	0	2.32479382797053e-12	0.272219662440403	0.222	0.086	7.79689354024758e-08	MALL
Rejected	0	2.89225439147294e-12	0.438771170054252	0.563	0.369	9.70004277812196e-08	LIMD2
Rejected	0	3.5404193977224e-12	0.317089652799345	0.234	0.095	1.18738585760814e-07	MAP1LC3A
Rejected	0	3.63552138839179e-12	0.522474313548735	0.824	0.732	1.21928116323884e-07	CD3D
Rejected	0	3.73524292246005e-12	0.363046859244224	0.276	0.123	1.25272577133465e-07	HLA-DPA1
Rejected	0	3.74801432342134e-12	0.532290567064716	0.893	0.828	1.25700904378905e-07	YPEL5
Rejected	0	4.19256745241254e-12	0.77355989733004	0.218	0.086	1.40610327219012e-07	XCL1
Rejected	0	4.62266469480336e-12	1.05378239007794	0.625	0.432	1.55034928534315e-07	ATF3
Rejected	0	4.774243501271e-12	0.345858158173225	0.299	0.136	1.60118578545627e-07	SPINT2
Rejected	0	5.07616153101436e-12	0.279327925614757	0.264	0.113	1.7024430542716e-07	MTTP
Rejected	0	5.21536059045046e-12	0.428936179692395	0.69	0.49	1.74912763482528e-07	PEBP1
Rejected	0	5.40843311821774e-12	0.483849681231811	0.602	0.395	1.81388029918787e-07	NSA2
Rejected	0	5.44544533187079e-12	0.28632049467999	0.264	0.113	1.82629345540282e-07	RAPGEF5
Rejected	0	5.6557009212068e-12	0.916746849832367	0.69	0.563	1.89680897495434e-07	KLRB1
Rejected	0	5.75957552986527e-12	0.306831078546702	0.238	0.098	1.93164644120621e-07	SLC26A3
Rejected	0	6.9323589752268e-12	0.274486544136296	0.23	0.094	2.32497455311156e-07	SH3RF1
Rejected	0	6.97013364633207e-12	0.362453184662313	0.387	0.208	2.33764342230685e-07	HIST1H1C
Rejected	0	7.42119206095889e-12	0.51429416721365	0.268	0.117	2.48891939340439e-07	XCL2
Rejected	0	8.34363000711253e-12	0.834296226949141	0.766	0.676	2.7982866317854e-07	RGS1
Rejected	0	9.01053135409778e-12	0.313244917491784	0.13	0.037	3.02195200553731e-07	MARCH1
Rejected	0	1.08285135272215e-11	0.288686211857316	0.291	0.135	3.63166686675956e-07	CRYL1
Rejected	0	1.45419085742638e-11	0.30839088630516	0.318	0.16	4.8770652976366e-07	MIF
Rejected	0	1.4858284873783e-11	0.622494829438112	0.759	0.633	4.98317158096933e-07	SAT1
Rejected	0	1.53881346854645e-11	0.266932196562169	0.249	0.104	5.16087261081109e-07	MATN2
Rejected	0	1.79369815304242e-11	0.304827604630673	0.295	0.137	6.01570486567366e-07	MYH14
Rejected	0	2.14440686356641e-11	0.350986857467358	0.977	0.928	7.19191173902902e-07	RPL13A
Rejected	0	2.22626948514777e-11	0.292840133975867	0.176	0.063	7.4664625992886e-07	HLA-DQA1
Rejected	0	2.29249635819919e-11	0.292165519104265	0.226	0.092	7.68857428612844e-07	NPAS2
Rejected	0	2.39428211863925e-11	0.558073491105331	0.563	0.357	8.02994336949231e-07	TXK

Rejected	0	2.3977329738267e-11	0.347063712147432	0.632	0.442	8.04151684761998e-07	CIB1
Rejected	0	2.52326134381209e-11	0.338166597001338	0.9	0.812	8.46251389487699e-07	SERF2
Rejected	0	2.74674986133965e-11	0.336415424368327	0.314	0.151	9.21204968496091e-07	RALGPS2
Rejected	0	2.76929942453251e-11	0.306698009321999	0.314	0.152	9.28767640999714e-07	ID1
Rejected	0	3.17376773020519e-11	0.322597163271405	0.261	0.116	1.06441822135622e-06	TRAPPC2B
Rejected	0	3.31856759137345e-11	0.56944348746649	0.9	0.863	1.11298119879483e-06	HSP90AB1
Rejected	0	4.41747875408323e-11	0.386378813015423	0.9	0.832	1.48153402454443e-06	H3F3A
Rejected	0	5.1722592612507e-11	0.424535449076591	0.709	0.563	1.73467231103826e-06	CD37
Rejected	0	5.2302251729257e-11	0.305435870274483	0.245	0.11	1.75411291849582e-06	COQ7
Rejected	0	5.27835613739568e-11	0.288672373024451	1	0.985	1.77025508135976e-06	B2M
Rejected	0	6.08953904514699e-11	0.741053861988026	0.774	0.618	2.0423096049614e-06	ELL2
Rejected	0	6.12630409498379e-11	0.298062376615801	1	1	2.05463986737566e-06	MT-ATP6
Rejected	0	6.36563571693445e-11	0.392870153188179	0.575	0.399	2.13490690674548e-06	RPS17
Rejected	0	6.57713883716505e-11	0.410609529071118	0.751	0.601	2.20584082320842e-06	UQCRB
Rejected	0	6.67747999677918e-11	0.284058068530501	0.184	0.068	2.2394932413198e-06	AC104695.2
Rejected	0	7.31734214601825e-11	0.264425151310797	0.211	0.085	2.4540902089316e-06	MS4A8
Rejected	0	7.36607545706244e-11	0.332584083624471	0.287	0.139	2.4704343867896e-06	PTGR1
Rejected	0	8.2919349994409e-11	0.385159304130652	0.943	0.898	2.78094916011249e-06	RPL14
Rejected	0	9.02746509821091e-11	0.326415069265078	0.226	0.097	3.02763124463798e-06	TMIGD2
Rejected	0	1.12857065809131e-10	0.621224105330131	0.759	0.648	3.78500027310665e-06	TRBC2
Rejected	0	1.15211135658405e-10	0.616857408295578	0.636	0.459	3.86395106771159e-06	IPCEF1
Rejected	0	1.2619272075104e-10	0.561711123408049	0.506	0.329	4.23225146854839e-06	EIF4A3
Rejected	0	1.49036553729374e-10	0.317331464614918	0.245	0.112	4.99838793897574e-06	TJP1
Rejected	0	1.68077439193965e-10	0.278433452218677	0.215	0.089	5.63698115568718e-06	AC025164.1
Rejected	0	1.95712625050551e-10	0.291843266612653	0.33	0.171	6.56381001894537e-06	HIST1H2AC
Rejected	0	2.04303939881515e-10	0.260770780228302	0.245	0.111	6.85194553574625e-06	FAM173A
Rejected	0	2.27544999433675e-10	0.254600482096378	0.184	0.071	7.63140419100658e-06	USP6NL
Rejected	0	2.51860108067966e-10	0.316049118195528	0.326	0.169	8.44688430438344e-06	PLS1
Rejected	0	2.6583678367206e-10	0.25900826405193	0.299	0.143	8.91563405079356e-06	ARHGAP32
Rejected	0	2.80599763412506e-10	0.291876547334497	0.264	0.124	9.41075486532862e-06	MUC3A
Rejected	0	3.00375097163973e-10	0.398846654540015	0.563	0.394	1.00739800086853e-05	ELOB

Rejected	0	3.16944812232764e-10	0.376043394846016	0.594	0.403	1.06296951126624e-05	OSTF1
Rejected	0	3.41526866172783e-10	0.343859928424574	0.874	0.828	1.14541280377028e-05	RPL27
Rejected	0	4.26300886053336e-10	0.495685743963945	0.245	0.111	1.42972791164568e-05	CCL4L2
Rejected	0	4.5473386892103e-10	0.689776583770958	0.448	0.29	1.52508644958735e-05	NAF1
Rejected	0	4.57675630834287e-10	0.29981623506134	0.368	0.201	1.53495253069203e-05	DAB1
Rejected	0	4.71334195392462e-10	0.573757973158551	0.41	0.251	1.58076062450724e-05	TMEM184C
Rejected	0	5.28285855436022e-10	0.604814903702263	0.743	0.63	1.77176510196133e-05	ID2
Rejected	0	6.7947408211578e-10	0.325701697007488	0.935	0.911	2.2788201765999e-05	RPS24
Rejected	0	9.40204779995481e-10	0.621672990632441	0.977	0.944	3.15325879114884e-05	FOS
Rejected	0	9.77781364578444e-10	0.337303552929263	0.51	0.334	3.27928314052318e-05	PIGR
Rejected	0	1.0392514832446e-09	0.41129123841223	0.908	0.872	3.48544162450575e-05	DNAJA1
Rejected	0	1.0851126218264e-09	0.365199891516706	0.586	0.413	3.63925071108138e-05	MZT2B
Rejected	0	1.35487720784497e-09	0.377070374056552	0.529	0.345	4.54398717967047e-05	AC022217.3
Rejected	0	1.4583804861742e-09	0.309099531180557	0.1	0.028	4.89111647453104e-05	ARL4D
Rejected	0	1.46055577276023e-09	0.42352964270587	0.958	0.912	4.89841195068327e-05	SARAF
Rejected	0	1.55217386630016e-09	0.331751206858065	0.636	0.462	5.20568071279746e-05	PRR13
Rejected	0	1.57659077140151e-09	0.251691477477242	0.222	0.099	5.28757012912638e-05	GNG12
Rejected	0	1.67581102645436e-09	0.304953798394881	0.471	0.292	5.62033502052262e-05	HSD17B11
Rejected	0	1.85386345964357e-09	0.339059800648435	0.345	0.19	6.21748727095262e-05	AC007952.4
Rejected	0	1.86815568299049e-09	0.747150260187874	0.747	0.649	6.26542052961351e-05	RANBP2
Rejected	0	2.14648627241225e-09	0.419843409242938	0.973	0.946	7.19888566041619e-05	PTMA
Rejected	0	2.15931621077319e-09	0.409670280480877	0.877	0.806	7.24191470769112e-05	PNRC1
Rejected	0	2.84612434181198e-09	0.333258519900018	0.766	0.647	9.545331817569e-05	RPS10
Rejected	0	3.71611054571987e-09	0.263610726355616	0.241	0.111	0.000124630915482353	TNFSF9
Rejected	0	3.87804871394211e-09	0.353933985837544	0.728	0.58	0.000130061997768191	SLC25A6
Rejected	0	4.02556486555735e-09	0.304509228241193	0.184	0.078	0.000135009394461062	SPSB1
Rejected	0	4.46891648084146e-09	0.250307693381749	0.241	0.116	0.000149878520934461	MYO1A
Rejected	0	5.1305311595458e-09	0.479649511038021	0.69	0.543	0.000172067754028847	CYCS
Rejected	0	5.18289837404559e-09	0.315439369936331	0.333	0.188	0.000173824045668741	SELENOH
Rejected	0	5.9632243203591e-09	0.311770604096404	0.613	0.433	0.000199994617256204	UXT
Rejected	0	6.12819972183695e-09	0.305314982128084	0.318	0.172	0.000205527562270968	DST

Rejected	0	6.29420791723292e-09	0.4231746001172	0.728	0.573	0.000211095145128158	TRAC
Rejected	0	7.05818224061587e-09	0.431250152312442	0.966	0.892	0.000236717315985775	IL7R
Rejected	0	7.28113891104713e-09	0.639078476867333	0.686	0.54	0.000244194836798699	VPS37B
Rejected	0	8.05854235172824e-09	0.484506021693608	0.341	0.197	0.000270267393392262	NMRK1
Rejected	0	8.38621944221158e-09	0.252920170506854	1	0.992	0.000281257027652892	MT-ND1
Rejected	0	1.13592714271352e-08	0.261579580420516	1	0.998	0.00038096724512326	MT-ND4
Rejected	0	1.33413745124433e-08	0.4563273893443	0.682	0.572	0.000447443018398323	CD3G
Rejected	0	1.34994032773368e-08	0.341089353573534	0.341	0.199	0.000452742987115323	CTSD
Rejected	0	1.43143900653998e-08	0.440178356786317	0.674	0.526	0.000480076014013377	SOD1
Rejected	0	1.46628592111476e-08	0.295077071489249	0.418	0.255	0.00049176297222347	ARRDC3
Rejected	0	1.55944639621518e-08	0.268778853633105	0.483	0.313	0.000523007132362647	VPS28
Rejected	0	1.60594285653113e-08	0.376852073335109	0.575	0.413	0.000538601115223409	H1FX
Rejected	0	1.66903093496532e-08	0.360532999207631	0.318	0.177	0.00055975959496867	PIM2
Rejected	0	1.7131751975861e-08	0.453403541582985	0.513	0.35	0.000574564697766426	EGR1
Rejected	0	1.74175715675236e-08	0.423175284531627	0.291	0.152	0.000584150515231608	ENTPD1
Rejected	0	1.80376046707342e-08	0.344715649943073	0.862	0.803	0.000604945185447083	ITM2B
Rejected	0	1.86528953020682e-08	0.326729819283453	0.805	0.702	0.000625580802640762	BTG2
Rejected	0	1.98646540393096e-08	0.372384745644376	0.402	0.252	0.000666220767170364	FAM53C
Rejected	0	2.02913358779297e-08	0.327336475690478	0.383	0.236	0.000680530822674006	MED29
Rejected	0	2.31422362392821e-08	0.369776140937143	0.356	0.205	0.000776144318993042	CD8B
Rejected	0	2.58268005543133e-08	0.283230795740049	0.64	0.478	0.000866179236990559	NDUFS5
Rejected	0	2.6524778912623e-08	0.494687432106149	0.977	0.955	0.000889588035171549	KLF6
Rejected	0	2.68057896623383e-08	0.290012056503895	0.525	0.368	0.000899012573695502	ATP5MD
Rejected	0	3.06598576821409e-08	0.304170490790891	0.851	0.737	0.00102827030694364	HINT1
Rejected	0	3.74516514514686e-08	0.294457095851464	0.831	0.687	0.00125605348637935	OAZ1
Rejected	0	3.89536447928019e-08	0.29303065407091	0.874	0.779	0.00130642733906099	BTF3
Rejected	0	4.17283293299695e-08	0.306111069289254	0.579	0.406	0.00139948470906852	DBI
Rejected	0	5.01600647090055e-08	0.250563837069552	0.437	0.272	0.00168226825021063	REX1BD
Rejected	0	5.16730543518775e-08	0.271518420651762	0.556	0.391	0.00173301089685327	GPSM3
Rejected	0	5.20902465825214e-08	0.446433935858794	0.448	0.29	0.0017470026898846	ITGAE
Rejected	0	5.34281510940578e-08	0.413613037412724	0.812	0.723	0.00179187333139251	DNAJB6

Rejected	0	6.47855149941563e-08	0.449170582037943	0.36	0.224	0.00217277660187401	WDR45B
Rejected	0	6.62960582339937e-08	0.31997862206394	0.567	0.404	0.00222343720105168	ATP5IF1
Rejected	0	7.82540790049171e-08	0.298307183191615	0.364	0.224	0.00262448530166691	MZT2A
Rejected	0	1.00964918167187e-07	0.323192468960092	0.739	0.597	0.00338616142549111	ATP1A1
Rejected	0	1.02562039273717e-07	0.292127686604224	0.648	0.497	0.00343972567316193	SLC25A3
Rejected	0	1.12703130659743e-07	0.629105249428515	0.559	0.428	0.00377983759606648	RGS2
Rejected	0	1.40601603077749e-07	0.312992280709567	0.59	0.433	0.00471549656402153	HERPUD1
Rejected	0	1.44639044169513e-07	0.291854214235921	0.943	0.905	0.00485090426335713	RPL5
Rejected	0	1.64983177017184e-07	0.302371363982656	0.284	0.158	0.00553320579080232	ADI1
Rejected	0	1.90058381277927e-07	0.592731651123615	0.383	0.254	0.00637417799129911	TRGC2
Rejected	0	1.98271751961918e-07	0.277380385998668	0.552	0.396	0.00664963801729879	PRDX1
Rejected	0	2.02616570100898e-07	0.251531986744157	0.28	0.15	0.00679535452804393	DAPK2
Rejected	0	2.06050697371947e-07	0.334597393453098	0.927	0.861	0.00691052828846036	ACTG1
Rejected	0	2.07597600000371e-07	0.337417674836096	0.793	0.678	0.00696240830881245	SEPT6
Rejected	0	2.07787034945045e-07	0.386980418806821	0.494	0.35	0.00696876157798693	TRMO
Rejected	0	2.13199857867542e-07	0.28002958450243	0.494	0.336	0.00715029683316161	VAMP8
Rejected	0	2.34197632427415e-07	0.568714284649038	0.176	0.083	0.00785452019635064	ICA1
Rejected	0	2.56813813250122e-07	0.253157777529091	0.383	0.239	0.00861302166878259	SCAND1
Rejected	0	2.78391753211226e-07	0.339815409591513	0.843	0.774	0.00933670261919809	HSPA8
Rejected	0	2.84683357927034e-07	0.324187734932539	0.782	0.672	0.00954771045815686	CD3E
Rejected	0	3.3630278384081e-07	0.287287473870885	0.192	0.094	0.0112789227644531	PASK
Rejected	0	3.48391168717431e-07	0.290553149484346	0.958	0.945	0.0116843430164452	JUND
Rejected	0	3.90038954173537e-07	0.250881875500463	0.996	0.977	0.0130811264450721	EEF1A1
Rejected	0	4.27637620084632e-07	0.428662019842851	0.59	0.467	0.0143421105023984	BCAS2
Rejected	0	4.48820982291142e-07	0.300393495852172	0.418	0.275	0.0150525581040803	YPEL3
Rejected	0	4.54550044950357e-07	0.410447941590287	0.713	0.588	0.0152446994075451	IER2
Rejected	0	5.20185398293857e-07	0.276423213568595	0.421	0.285	0.0174459778879794	PTP4A1
Rejected	0	6.01224655523965e-07	0.250601506480056	0.441	0.297	0.0201638724969627	FAM102A
Rejected	0	6.36552504450359e-07	0.265440520031955	0.732	0.591	0.0213486978942561	ATP5MC2
Rejected	0	6.66576019633629e-07	0.270197617729202	0.264	0.148	0.0223556265464727	NR5A2
Rejected	0	6.80582557469731e-07	0.542458678474332	0.295	0.171	0.0228253778124198	CRTAM

Rejected	0	6.89749633339486e-07	0.28887415985909	0.893	0.78	0.0231328232029397	ATP5F1E
Rejected	0	7.24792660535246e-07	0.628925583858585	0.521	0.39	0.0243080962490311	TRBC1
Rejected	0	7.78455192315559e-07	0.265658409126378	0.651	0.492	0.0261078302398792	HMGN1
Rejected	0	8.31805514203218e-07	0.285631980996904	0.33	0.201	0.0278970933353475	HNF4G
Rejected	0	8.33935911910903e-07	0.341461736684777	0.18	0.088	0.0279685426136679	GPR34
Rejected	0	8.59190038169986e-07	0.317664390406895	0.674	0.534	0.028815515500145	HMGN2
Rejected	0	8.59211372273636e-07	0.260125651399297	0.398	0.259	0.0288162310033132	NSMCE3
Rejected	0	9.31964575198536e-07	0.306729566887523	0.755	0.63	0.0312562279230085	ISG20
Rejected	0	9.37097689142173e-07	0.260255907865177	0.536	0.385	0.0314283822984502	GYPC
Rejected	0	1.01011364469441e-06	0.252922564868772	0.613	0.465	0.0338771914157611	CCT4
Rejected	0	1.07440373747903e-06	0.273461712690703	0.138	0.06	0.0360333525475718	AC013264.1
Rejected	0	1.23370773061515e-06	0.309298475570932	0.674	0.562	0.041376089869371	ANAPC16
Rejected	0	1.26048029853506e-06	0.250285551473904	0.579	0.436	0.0422739882522689	C12orf57
Rejected	0	1.26274042823461e-06	0.409724157873326	0.395	0.252	0.0423497884821322	KLRD1
Rejected	0	1.2928597863439e-06	0.343150882739244	0.575	0.443	0.0433599315144016	PPDPF
Rejected	0	1.41367168745384e-06	0.296590638975461	0.494	0.369	0.0474117210538269	UBXN1
Rejected	0	1.45560409661065e-06	0.423550409078612	0.912	0.882	0.0488180501921281	FOSB
Rejected	0	1.45697034328819e-06	0.257645704037861	0.556	0.405	0.0488638713731993	SLC20A1
Rejected	6	1.51627496731265e-71	1.73109205280479	0.526	0.074	5.08528298537317e-67	CD160
Rejected	6	4.45404908156578e-62	3.64655124166864	0.578	0.108	1.49379898097553e-57	XCL2
Rejected	6	7.30275237956278e-45	2.03627260523369	0.948	0.709	2.44919709305777e-40	CCL5
Rejected	6	4.15890476305241e-44	3.55238142532404	0.489	0.105	1.39481347943252e-39	CCL4L2
Rejected	6	1.44878471672136e-43	3.37378468768441	0.43	0.081	4.8589341829401e-39	XCL1
Rejected	6	9.10809877832871e-38	1.42089887518682	0.8	0.37	3.05467416827588e-33	NR4A1
Rejected	6	9.509203394978e-36	1.45876983185372	0.978	0.931	3.18919663460772e-31	CD69
Rejected	6	3.00938647164487e-32	0.970798488110428	0.378	0.082	1.00928803486026e-27	EGR2
Rejected	6	3.76130110593825e-32	1.62885025131635	0.919	0.701	1.26146516490957e-27	BTG2
Rejected	6	1.66815624283449e-31	1.3352161688063	0.948	0.695	5.59466240721832e-27	NR4A2
Rejected	6	3.14269010844696e-27	1.61389876598021	0.83	0.494	1.05399540857094e-22	NR4A3
Rejected	6	2.78639992166976e-26	1.22352238177169	0.896	0.658	9.34502805729603e-22	ZFP36
Rejected	6	1.18247955170041e-25	1.89288251618113	0.274	0.052	3.96579992049284e-21	CCL3L1

Rejected	6	2.46967972926352e-24	1.11823475686952	0.948	0.898	8.282811876004e-20	H3F3B
Rejected	6	3.45018286028687e-24	1.52520085840771	0.681	0.323	1.15712232768301e-19	SPRY1
Rejected	6	4.69920560470498e-24	1.25604308019618	0.637	0.301	1.57601957570596e-19	BCL2A1
Rejected	6	2.75489471441578e-23	1.14163156345561	0.8	0.476	9.23936589320763e-19	MAP3K8
Rejected	6	4.38236222621524e-21	1.01281326383817	0.519	0.204	1.46975664342807e-16	CD8B
Rejected	6	1.90672020557125e-20	1.03313791895403	0.822	0.65	6.39475822544486e-16	RPS10
Rejected	6	5.62365145345487e-20	0.905449145649661	0.948	0.943	1.8860602244597e-15	ACTB
Rejected	6	1.64967706623042e-19	0.742431941695082	0.526	0.213	5.53268694472359e-15	HOPX
Rejected	6	2.60738718512895e-19	1.02399005637014	0.452	0.166	8.74465514148546e-15	AC020571.1
Rejected	6	5.2573306404202e-18	0.80525104009388	0.948	0.954	1.76320355018413e-13	TMSB4X
Rejected	6	6.79801155840631e-18	0.865264064375394	0.985	0.956	2.27991711645831e-13	KLF6
Rejected	6	7.43815096795444e-18	0.516724049228653	0.333	0.098	2.49460707163256e-13	TMIGD2
Rejected	6	1.32832435051661e-17	0.952710337675919	0.822	0.588	4.45493420676261e-13	IER2
Rejected	6	6.17252539296978e-17	0.403990166085056	0.17	0.031	2.07014156629421e-12	SPINK2
Rejected	6	6.28662786753101e-17	0.668534541703608	0.4	0.146	2.10840925421255e-12	CAPG
Rejected	6	1.04332048460818e-16	0.892912182256891	0.889	0.729	3.4990882412789e-12	GAPDH
Rejected	6	7.47368856901989e-16	1.09120303617423	0.43	0.18	2.50652567227789e-11	MYC
Rejected	6	7.76260309184142e-16	0.743175564107332	0.615	0.314	2.60342182494177e-11	ALOX5AP
Rejected	6	9.04073061170006e-16	0.682930868916872	0.274	0.08	3.03208023255197e-11	TNFSF14
Rejected	6	1.08788586431372e-15	0.907112212816544	0.333	0.112	3.64855161173534e-11	TNFSF9
Rejected	6	1.43395855480245e-15	0.934408754662513	0.985	0.942	4.80921020109646e-11	HSP90AA1
Rejected	6	3.45741307725959e-15	1.05697318772458	0.956	0.947	1.15954719785132e-10	FOS
Rejected	6	4.75739761218766e-15	1.18960021509181	0.652	0.398	1.5955360111755e-10	GADD45B
Rejected	6	6.30319253324627e-15	1.19699870900917	0.615	0.353	2.11396471180013e-10	EGR1
Rejected	6	9.98154177667462e-15	1.0518584116204	0.519	0.253	3.34760948106113e-10	TRGC2
Rejected	6	1.47060511811762e-14	0.848431203383735	0.607	0.344	4.93211544514288e-10	NEU1
Rejected	6	1.85217912824762e-14	1.04966126417289	0.563	0.288	6.21183836031688e-10	TNIP3
Rejected	6	2.09300514981092e-14	0.921072414221747	0.756	0.573	7.01952067143585e-10	CD3G
Rejected	6	2.48029072407859e-14	0.708111481455472	0.548	0.251	8.31839903041476e-10	KLRD1
Rejected	6	2.5774233906897e-14	0.461775758396871	0.23	0.061	8.64416256769512e-10	FASLG
Rejected	6	2.77272592536805e-14	0.539526238210425	1	0.999	9.29916820849938e-10	MT-CYB

Rejected	6	3.92197949072677e-14	1.641658329152	0.2	0.048	1.31535348159994e-09	IL2
Rejected	6	4.85930798630228e-14	0.728521822775148	0.926	0.894	1.62971471244606e-09	RPS7
Rejected	6	6.13294647601508e-14	0.940904566652758	0.785	0.652	2.05686758912594e-09	TRBC2
Rejected	6	6.55968507807268e-14	2.3285525859241	0.444	0.205	2.19998718148402e-09	IFNG
Rejected	6	8.02771341654918e-14	2.38345633966358	0.637	0.409	2.69233452564226e-09	CCL4
Rejected	6	9.64485587822339e-14	1.44153490448442	0.407	0.171	3.23469176443856e-09	CRTAM
Rejected	6	1.30760351429219e-13	0.736984336170459	0.748	0.516	4.38544066623316e-09	RAC2
Rejected	6	1.62170055580524e-13	1.4353600305933	0.585	0.328	5.43885932405963e-09	HSPA1B
Rejected	6	1.85706755467645e-13	0.654575371810705	0.956	0.843	6.22823316487387e-09	REL
Rejected	6	1.91176204572726e-13	0.666799259414124	0.748	0.442	6.41166754896008e-09	ITGA1
Rejected	6	2.11321612765559e-13	0.664908764879358	0.889	0.818	7.08730424893132e-09	PFN1
Rejected	6	2.4285749646238e-13	0.618774081377229	0.948	0.937	8.1449547163553e-09	RPL13
Rejected	6	2.78237755282461e-13	0.569580918260064	0.207	0.052	9.33153783666318e-09	CA10
Rejected	6	3.63047611856879e-13	0.502411621808678	1	0.985	1.2175890806456e-08	B2M
Rejected	6	3.87161649517331e-13	0.722159580787842	0.837	0.736	1.29846274015122e-08	CD3D
Rejected	6	4.06974761212928e-13	0.62586181920425	0.948	0.946	1.36491195415592e-08	JUND
Rejected	6	4.26061512212921e-13	0.775137022124912	0.837	0.705	1.4289250996597e-08	SH3BGRL3
Rejected	6	4.27350399573005e-13	0.675388319941587	0.919	0.923	1.43324777008794e-08	RPS4X
Rejected	6	4.76864456974707e-13	0.650081092789257	0.896	0.906	1.59930801580177e-08	RPL6
Rejected	6	5.05422215415633e-13	0.689767642673552	0.637	0.35	1.69508502606095e-08	NKG7
Rejected	6	5.1066264564263e-13	0.629209376299383	0.978	0.962	1.71266038095625e-08	RPLP1
Rejected	6	5.22186715767974e-13	0.69554444720416	0.919	0.865	1.75130980734263e-08	ACTG1
Rejected	6	6.579578489363e-13	0.541580970152872	0.556	0.292	2.20665903376256e-08	ITGAE
Rejected	6	8.70201555823513e-13	0.637263869733011	0.948	0.881	2.9184819779209e-08	FOSB
Rejected	6	9.3090517472366e-13	0.721908272131849	0.896	0.897	3.12206977498821e-08	RPL21
Rejected	6	1.10717167687099e-12	0.718679962822468	0.889	0.735	3.71323236988993e-08	NFKBIZ
Rejected	6	1.19188998280073e-12	0.71056895188895	0.904	0.738	3.99736062431708e-08	FABP6
Rejected	6	1.42469982784262e-12	0.659614705642207	0.904	0.886	4.77815828261857e-08	RPL19
Rejected	6	2.223303398287e-12	0.973458266247252	0.474	0.254	7.45651493717495e-08	INSIG1
Rejected	6	2.26537687793881e-12	0.7646759274104	0.667	0.436	7.59762097323118e-08	CKLF
Rejected	6	3.90198799535242e-12	0.562711230862262	0.926	0.835	1.30864873388129e-07	PPP1R15A

Rejected	6	4.3139837448614e-12	0.636267538837579	0.459	0.222	1.44682386835161e-07	SERTAD1
Rejected	6	4.76816785236052e-12	0.714602329482891	0.748	0.541	1.59914813432467e-07	CORO1A
Rejected	6	5.10187595807798e-12	0.59273558805094	0.933	0.922	1.71106715882019e-07	RPS3A
Rejected	6	6.17533700793357e-12	0.341975271908284	1	1	2.07108452572076e-07	MALAT1
Rejected	6	1.02518600255798e-11	0.48240428431003	0.585	0.322	3.43826881537895e-07	GSTP1
Rejected	6	1.03968225909574e-11	0.636832767328326	0.874	0.81	3.48688636055528e-07	PNRC1
Rejected	6	1.17069401608028e-11	0.589505867942133	0.926	0.904	3.92627359113006e-07	RPL7A
Rejected	6	1.40735050035181e-11	0.951249922197658	0.667	0.498	4.71997210807989e-07	SERPINB9
Rejected	6	1.6568409667413e-11	0.491080868621742	0.526	0.282	5.55671323425696e-07	SSBP4
Rejected	6	1.73947156972161e-11	0.60308428956852	0.259	0.09	5.83383975053235e-07	IL23A
Rejected	6	1.94445477772391e-11	1.21802556888701	0.444	0.218	6.52131243353043e-07	TNF
Rejected	6	2.10496460944291e-11	0.588761182169674	0.867	0.855	7.05963030714964e-07	RPL23A
Rejected	6	2.33327550052298e-11	0.61065914801181	0.896	0.829	7.82533937365398e-07	MCL1
Rejected	6	3.0042233877696e-11	0.762887685107188	0.585	0.345	1.00755643979017e-06	ARL5B
Rejected	6	3.29859520272443e-11	0.498617517255369	0.341	0.139	1.10628285908972e-06	ABI3
Rejected	6	3.92510002209566e-11	0.724435260041543	0.822	0.755	1.31640004541044e-06	MYL12A
Rejected	6	3.96474193491573e-11	0.574987992999338	0.919	0.932	1.32969515013204e-06	RPS18
Rejected	6	5.04228005521671e-11	0.930309303782184	0.933	0.88	1.69107988491858e-06	JUN
Rejected	6	6.21006608915320e-11	0.552522978271109	0.896	0.831	2.0827319649802e-06	RPL7
Rejected	6	7.50343841803055e-11	0.666718114409674	0.637	0.447	2.51650317663909e-06	PPDPF
Rejected	6	8.98050513976058e-11	0.638593024164313	0.926	0.864	3.0118818137729e-06	HSP90AB1
Rejected	6	1.51837350443354e-10	0.523662655222171	0.815	0.696	5.09232105916922e-06	OAZ1
Rejected	6	1.73980507392745e-10	0.469084678373099	0.97	0.954	5.83495825693789e-06	SRGN
Rejected	6	1.84162266672785e-10	0.558833230838438	0.926	0.92	6.17643409967186e-06	RPS3
Rejected	6	2.24953008561932e-10	0.587340674777105	0.948	0.941	7.54447400115007e-06	RPS12
Rejected	6	2.76025931470822e-10	0.532232847987131	0.459	0.24	9.25735768966844e-06	GZMM
Rejected	6	2.97730687128638e-10	0.724733281594818	0.556	0.333	9.98529178492026e-06	CD8A
Rejected	6	4.3540706976931e-10	0.550798899755662	0.844	0.827	1.46026823059231e-05	CFL1
Rejected	6	5.13060727126226e-10	0.924757156234621	0.652	0.444	1.72070306663594e-05	YES1
Rejected	6	5.75935098222773e-10	0.417924235135735	0.948	0.919	1.93157113241953e-05	HLA-B
Rejected	6	6.81887120158339e-10	0.377412286231594	0.281	0.112	2.28691302358704e-05	PDLIM2

Rejected	6	8.78778017086176e-10	0.68247144720749	0.756	0.579	2.94724571370362e-05	TRAC
Rejected	6	9.61945743249998e-10	0.60500318896011	0.867	0.839	3.22617363371184e-05	RPS5
Rejected	6	9.78541564046213e-10	0.38165380545604	1	1	3.28183269749819e-05	MT-CO1
Rejected	6	1.0910395611081e-09	0.51077477389373	0.97	0.895	3.65912848004433e-05	SRSF7
Rejected	6	1.16649579379367e-09	0.47360456386540	0.919	0.927	3.91219359322521e-05	RPL11
Rejected	6	1.1803079632001e-09	0.73339274782472	0.867	0.776	3.95851684698051e-05	HSPA8
Rejected	6	1.59284054583389e-09	0.48902730374112	0.941	0.907	5.34206862261769e-05	RPS16
Rejected	6	2.20941727190875e-09	0.48022536518039	0.948	0.952	7.40994364652758e-05	RPL10
Rejected	6	2.26642355812963e-09	0.63491185927698	0.904	0.882	7.60113132925515e-05	RPSA
Rejected	6	2.47385390193670e-09	0.52682158064631	0.919	0.927	8.29681121631529e-05	RPS6
Rejected	6	3.02214224115663e-09	0.77197069188764	0.585	0.4	0.000101356606483911	HMGCS1
Rejected	6	3.0648066247652e-09	0.91080166079045	0.741	0.635	0.000102787484581375	ZFP36L1
Rejected	6	3.16990592839262e-09	0.60287964088494	0.822	0.675	0.000106312305026432	CD3E
Rejected	6	3.3407930870702e-09	0.37292564327104	0.444	0.237	0.00011204351855416	ARPC1B
Rejected	6	3.83529385797848e-09	0.55895972538760	0.852	0.67	0.000128628085408882	MTRNR2L12
Rejected	6	4.33552902894797e-09	0.49879676842995	0.578	0.371	0.000145404972572857	TPI1
Rejected	6	4.40079036742419e-09	0.54984005019149	0.674	0.469	0.000147593707342672	CLIC1
Rejected	6	4.52194266156661e-09	0.63818921561494	0.77	0.663	0.000151656912983621	CD48
Rejected	6	4.77827809098222e-09	0.47625968499090	0.904	0.913	0.000160253890615362	RPS27A
Rejected	6	4.89474355432123e-09	1.43539839174405	0.637	0.445	0.000164159909324825	HSPA1A
Rejected	6	5.15333931740436e-09	0.50595351730978	0.926	0.904	0.000172832694027108	RPL3
Rejected	6	5.39030257627925e-09	0.79833538302836	0.356	0.167	0.000180779967803253	AC015849.1
Rejected	6	5.43681939686327e-09	0.62371208399224	0.815	0.78	0.000182340048932	EEF1B2
Rejected	6	7.13299433688612e-09	0.44165606532809	0.919	0.9	0.000239226364070487	RPL15
Rejected	6	8.20318014030786e-09	0.43643976893365	0.926	0.87	0.000275118255545645	HLA-A
Rejected	6	1.01337297616238e-08	0.50137891726518	0.193	0.063	0.000339865028745339	KLRC1
Rejected	6	1.05683857137706e-08	0.40016150955370	0.911	0.834	0.000354442520068439	H3F3A
Rejected	6	1.13876334676145e-08	0.48557191086083	0.681	0.491	0.000381918451236854	RARRES3
Rejected	6	1.17734725459001e-08	0.72528103933035	0.659	0.517	0.000394858722244399	COTL1
Rejected	6	1.22929469527639e-08	0.44460350016756	0.948	0.898	0.000412280854901795	CALM1
Rejected	6	1.52045532209709e-08	0.56124421594819	0.941	0.875	0.000509930305924923	RPLP0

Rejected	6	1.58179856934638e-08	0.441015388504582	0.919	0.934	0.00053050360418739	RPL28
Rejected	6	1.8967452467561e-08	0.611486986306056	0.881	0.835	0.000636130420857062	VIM
Rejected	6	1.89834873264631e-08	0.435085314472569	0.867	0.86	0.00063666819795492	RACK1
Rejected	6	2.00744410730164e-08	0.524889578960379	0.919	0.883	0.000673256604706824	RPL8
Rejected	6	2.1105070038485e-08	0.396497667247058	0.978	0.904	0.00070782183895071	PABPC1
Rejected	6	2.37797246353687e-08	0.45268191889113	0.896	0.899	0.000797524404820997	RPL18A
Rejected	6	2.66516269318821e-08	0.532105221712694	0.585	0.369	0.000893842264041462	NFKBID
Rejected	6	2.71119477641613e-08	0.433671695987893	0.941	0.899	0.000909280504114443	RPS9
Rejected	6	2.81930399617312e-08	0.686009654646933	0.733	0.578	0.00094553817423654	PTGER4
Rejected	6	3.0014531287739e-08	0.453471140893797	0.859	0.658	0.00100662735032819	MT-ATP8
Rejected	6	3.05834669663647e-08	0.577589597567852	0.615	0.392	0.00102570831511794	ZEB2
Rejected	6	3.39871095959949e-08	0.534554597928541	0.867	0.763	0.00113985968163048	SDCBP
Rejected	6	3.5867992191989e-08	0.486682623285418	0.911	0.937	0.00120294072213493	RPS19
Rejected	6	3.66845237868695e-08	0.515985105252466	0.859	0.848	0.00123032555876403	RPL10A
Rejected	6	3.80310932417986e-08	0.625186311524661	0.696	0.55	0.00127548680514344	CYCS
Rejected	6	3.89162359972893e-08	0.777605030774406	0.844	0.755	0.00130517272287709	JUNB
Rejected	6	3.90861368091381e-08	0.585920071645863	0.37	0.187	0.00131087085630487	HECW2
Rejected	6	4.63780005670294e-08	0.426353518259287	0.933	0.898	0.00155542538301703	RPL29
Rejected	6	4.93049523372271e-08	0.757404221139696	0.289	0.136	0.00165358949148592	EGR3
Rejected	6	5.29216708822314e-08	0.558859118646263	0.881	0.91	0.00177488699804828	RPL5
Rejected	6	5.34922293309719e-08	0.425046987504417	0.911	0.911	0.00179402238730214	RPL30
Rejected	6	5.53795841559343e-08	0.732913088829193	0.674	0.488	0.00185732049342172	PFKFB3
Rejected	6	5.5508055803525e-08	0.450819425454574	0.904	0.891	0.00186162917553862	RPS20
Rejected	6	5.62539590047728e-08	0.52252922988665	0.356	0.18	0.00188664527710207	RBKS
Rejected	6	7.75680819644082e-08	0.414689885068973	0.511	0.29	0.00260147833292232	MYBL1
Rejected	6	8.55595570084742e-08	0.327750100632276	0.267	0.113	0.00286949642295021	CD9
Rejected	6	9.93594005796082e-08	0.482590492486334	0.948	0.775	0.0033323155766389	MTRNR2L8
Rejected	6	1.08485467891316e-07	0.519195359647966	0.296	0.133	0.00363838562213895	HDAC9
Rejected	6	1.33772725998709e-07	0.409671299343107	0.904	0.934	0.00448646968454471	RPS14
Rejected	6	1.35669298627652e-07	0.522880693622225	0.896	0.839	0.00455007693737418	UBC
Rejected	6	1.40229220717362e-07	0.91556675657196	0.607	0.443	0.0047030076044189	ATF3

Rejected	6	1.46588797249833e-07	0.477205513276641	0.341	0.175	0.00491629508216489	H2AFJ
Rejected	6	1.53512342408134e-07	0.50000935584799	0.867	0.851	0.00514849693968401	RPL26
Rejected	6	1.74423285737181e-07	0.42511555805327	0.926	0.909	0.00584980815705359	RPS23
Rejected	6	1.86229480020475e-07	0.438083723036906	0.548	0.351	0.0062457643009267	COX6A1
Rejected	6	1.93002740703676e-07	0.44232740452878	0.8	0.639	0.00647292591771989	CHCHD2
Rejected	6	1.96821101561224e-07	0.298864246895569	0.37	0.193	0.00660098610416033	UBE2M
Rejected	6	1.98932573314838e-07	0.385944180692853	0.926	0.933	0.00667180064383302	RPL13A
Rejected	6	2.18435160463511e-07	0.488895989621191	0.919	0.873	0.00732587841162522	DNAJA1
Rejected	6	2.21827614655405e-07	0.496331062079474	0.593	0.407	0.00743965454031296	GUK1
Rejected	6	2.23853674112183e-07	0.479282949077452	0.978	0.946	0.00750760452237438	TNFAIP3
Rejected	6	2.31182510212436e-07	0.43185182743491	0.933	0.912	0.00775339902750466	RPL32
Rejected	6	2.56043925795238e-07	0.380591293674266	0.43	0.246	0.0085872011833207	PPP1R14B
Rejected	6	2.75039969535369e-07	0.423613326565953	0.644	0.451	0.0092242904982772	CIB1
Rejected	6	2.79664815964457e-07	0.579137783205687	0.563	0.364	0.00937939859781597	GPR65
Rejected	6	3.13810028665586e-07	0.38189100882612	0.437	0.249	0.0105245607413864	DYNLT3
Rejected	6	3.16908907874091e-07	0.463467695056379	0.563	0.399	0.0106284909522813	CSRNP1
Rejected	6	3.19624541610533e-07	0.446451066277881	0.4	0.231	0.0107195678765341	TRG-AS1
Rejected	6	3.3958699354011e-07	1.04932603883766	0.667	0.522	0.0113890685893482	TRAF1
Rejected	6	3.48509064671384e-07	0.399935635875284	0.911	0.893	0.0116882970109489	RPS13
Rejected	6	3.74884927081166e-07	0.346217976037929	0.719	0.507	0.0125728906844482	LCK
Rejected	6	4.15760283244754e-07	0.332916616432102	0.163	0.057	0.0139437683794626	AC103591.3
Rejected	6	4.26665232193877e-07	0.502449845034535	0.563	0.392	0.0143094985573183	SLC25A5
Rejected	6	4.66924514706453e-07	0.42398519317172	0.356	0.194	0.015659714374225	SELENOH
Rejected	6	4.87044959144194e-07	0.392249891862146	0.97	0.979	0.016334513839778	EEF1A1
Rejected	6	5.34234010024874e-07	0.729622151363936	0.704	0.569	0.0179171402282142	SELENOK
Rejected	6	5.53484253657397e-07	0.61368994447546	0.83	0.686	0.0185627548991618	RASGEF1B
Rejected	6	6.6016428127073e-07	0.416796688278917	0.911	0.902	0.0221405896652577	RPL14
Rejected	6	6.65141854162632e-07	0.365454563941283	0.593	0.413	0.0223075275049064	OSTF1
Rejected	6	7.91613957217215e-07	0.4384216063994	0.756	0.661	0.026549148897151	MYL12B
Rejected	6	8.8147916084115e-07	0.504184724806385	0.215	0.091	0.0295630480962905	GPR34
Rejected	6	9.83170997990362e-07	0.374159599737477	0.97	0.938	0.0329735889306008	RPL41

Rejected	6	1.02477774525211e-06	0.452060127735111	0.704	0.545	0.0343689960202652	ARPC5
Rejected	6	1.03615113778336e-06	0.313386318862592	0.304	0.153	0.0347504368589782	RGL4
Rejected	6	1.07584481517238e-06	0.55846353110652	0.674	0.519	0.0360816834112514	CD7
Rejected	6	1.08588313184578e-06	0.385447580818066	0.452	0.272	0.0364183484758436	AC044849.1
Rejected	6	1.08684880475427e-06	0.350808247681923	0.919	0.895	0.0364507352138485	RPS11
Rejected	6	1.17077330358112e-06	0.343940882036713	0.526	0.341	0.0392653950555036	COX7A2L
Rejected	6	1.23308296812047e-06	0.401636424906563	0.422	0.247	0.0413551365848243	NRBF2
Rejected	6	1.37687558265454e-06	0.294173024562167	0.296	0.15	0.046177653291068	COMMD7
Rejected	6	1.38878977337871e-06	0.376925838435529	0.896	0.902	0.0465772314195753	RPS25
Rejected	6	1.45962546012513e-06	0.420135789284381	0.83	0.738	0.0489529186816766	YBX1
Rejected	6	1.49019464213352e-06	0.322934080560402	0.4	0.226	0.049978147907874	DZIP3
Rejected	14	8.30628509777422e-84	1.4612198017657	0.884	0.381	2.78576189609152e-79	STAT1
Rejected	14	5.17041103715247e-49	0.767528818364725	0.641	0.231	1.7340524536402e-44	GBP1
Rejected	14	1.72094638514607e-36	0.663465032195054	0.62	0.245	5.77170998650289e-32	SNTB1
Rejected	14	3.58578769923011e-36	0.944686834940332	0.634	0.278	1.20260147856779e-31	FRMD4B
Rejected	14	5.86786971045842e-36	0.546424517142885	0.528	0.185	1.96796614349355e-31	GBP4
Rejected	14	1.75606174285065e-35	1.27712824850836	0.574	0.245	5.88947987317251e-31	CD38
Rejected	14	5.04716345767254e-35	0.83914684921653	0.697	0.316	1.69271768043422e-30	ARL5B
Rejected	14	4.98225076212722e-33	0.779131259527221	0.958	0.762	1.67094726060223e-28	MTRNR2L8
Rejected	14	4.30799141629031e-31	0.851450435538235	0.877	0.581	1.44481416119544e-26	ICOS
Rejected	14	1.03260973893795e-30	0.664336589503811	0.577	0.245	3.46316654245008e-26	GBP5
Rejected	14	4.49472258501364e-30	0.374903347004517	0.514	0.189	1.50744006056188e-25	PARP9
Rejected	14	2.4046110604842e-29	0.738883364723667	0.944	0.647	8.06458457465191e-25	MTRNR2L12
Rejected	14	4.86587857477673e-29	0.771013958591543	0.616	0.288	1.63191835640862e-24	PRMT9
Rejected	14	1.37599299462114e-28	0.787372924820591	0.93	0.645	4.61480530536039e-24	AC105402.3
Rejected	14	2.81026711510087e-28	0.834992223438508	0.75	0.419	9.4250738506253e-24	YES1
Rejected	14	4.88210655706805e-28	0.471441349767661	0.754	0.349	1.63736089710948e-23	OLFM4
Rejected	14	1.13911135069919e-27	0.684187214316385	0.574	0.252	3.82035164797495e-23	IRF4
Rejected	14	2.11070262211354e-27	0.577721207579347	0.975	0.858	7.07887445404437e-23	RHOH
Rejected	14	6.50900415498692e-27	0.728271656234179	0.982	0.895	2.18298981349951e-22	SAMSN1
Rejected	14	7.2255641853291e-27	0.333791652253292	0.88	0.463	2.42330971647568e-22	MT1G

Rejected	14	3.86918572063842e-26	0.4511973186721115	0.736	0.374	1.29764750698771e-21	RASSF5
Rejected	14	6.18560817710136e-26	0.848539847550758	0.824	0.542	2.07452927043625e-21	PDE4B
Rejected	14	8.27062281841502e-26	0.6017549929641922	0.606	0.284	2.77380148084003e-21	BCL2A1
Rejected	14	1.63312263261213e-25	0.62934127410364	0.342	0.11	5.47716668525457e-21	CSF1
Rejected	14	1.91169092627294e-25	0.7534876441149622	0.768	0.449	6.41142902853417e-21	EPSTI1
Rejected	14	2.02399192377752e-24	0.6365538374886522	0.521	0.235	6.78806411396504e-20	TMEM184C
Rejected	14	2.51044246978802e-24	0.5419576890209488	0.134	0.02	8.41952195517507e-20	HIST1H1B
Rejected	14	1.20196042434869e-23	0.2564814297966344	0.12	0.016	4.03113487118063e-19	FAM111B
Rejected	14	4.79838496474249e-22	0.4029430387956544	0.415	0.165	1.60928234947534e-17	CKAP2
Rejected	14	6.31093021116587e-22	0.5277513557229166	0.655	0.338	2.11655977422081e-17	DUSP5
Rejected	14	8.47075029888434e-22	0.68062213208935	0.93	0.767	2.84092023523983e-17	CREM
Rejected	14	9.28804504552382e-22	0.8864457271008144	0.982	0.928	3.11502454736778e-17	CD69
Rejected	14	9.80560030084836e-22	0.3646132112773588	0.718	0.361	3.28860222889852e-17	APOL6
Rejected	14	2.64073825903857e-21	0.6365678377344244	0.87	0.634	8.85650797316355e-17	IRF1
Rejected	14	3.87074734602488e-21	0.3698069977454333	0.19	0.047	1.29817124490982e-16	KIF23
Rejected	14	4.06175603698071e-21	0.4622775051915566	0.673	0.358	1.36223173968259e-16	PSME2
Rejected	14	4.24491992360131e-21	0.3332842488168388	0.398	0.155	1.42366124397741e-16	SAMD9L
Rejected	14	6.11698883853653e-21	0.6540996456647655	0.813	0.526	2.05151571666838e-16	CYTOR
Rejected	14	6.82254015492246e-21	0.6525654642056911	0.563	0.276	2.28814351715789e-16	PCBP3
Rejected	14	9.95937429029929e-21	0.4250274033580599	0.511	0.233	3.34017494948058e-16	TNFSF10
Rejected	14	1.10558774137345e-20	0.5313209048209477	0.975	0.815	3.70792016701828e-16	MCL1
Rejected	14	1.53096185537139e-20	0.5096683683468855	0.634	0.324	5.13453987054457e-16	HIVEP3
Rejected	14	1.75429000488721e-20	0.6039116196905499	0.968	0.835	5.88353781839073e-16	REL
Rejected	14	2.05880950805379e-20	0.5365302530569922	0.782	0.471	6.9048353281108e-16	ZNF267
Rejected	14	2.3688975121215e-20	0.3755899411466466	0.518	0.234	7.94480847615308e-16	INSIG1
Rejected	14	2.74403717078232e-20	0.4938320575779711	0.761	0.469	9.20295186336973e-16	RARRES3
Rejected	14	3.61429861604379e-20	0.2681257932533677	0.201	0.052	1.21216346984877e-15	PCLAF
Rejected	14	4.28149972892327e-20	0.2651546723546911	0.197	0.051	1.43592937908629e-15	AC017002.5
Rejected	14	5.19479132701312e-20	0.3889500705840011	0.366	0.143	1.74222911525366e-15	HIST1H1E
Rejected	14	6.47393503571275e-20	0.4818082977756833	0.972	0.952	2.17122833227734e-15	TMSB4X
Rejected	14	9.8705415312486e-20	0.3805553732386155	0.229	0.066	3.31038221875015e-15	AIM2

Rejected	14	1.00125956484318e-19	0.265147179149145	0.5	0.219	3.35802432857107e-15	TAP1
Rejected	14	1.02536384755595e-19	0.883149640627568	0.683	0.408	3.43886527193314e-15	ADAM19
Rejected	14	1.23162987188324e-19	0.562288537782731	0.88	0.62	4.13064026432202e-15	TRAF3
Rejected	14	1.5378066923046e-19	0.62713924468386	0.908	0.723	5.15749608465117e-15	NFKBIZ
Rejected	14	1.66270462378261e-19	0.611393521387662	0.454	0.205	5.57637876724211e-15	HIST1H1D
Rejected	14	1.7631370387145e-19	0.463930912858432	0.394	0.163	5.91320900044069e-15	HIST2H2AC
Rejected	14	2.52901818676183e-19	0.314480657148465	0.25	0.078	8.48182119476181e-15	EGR2
Rejected	14	3.03465478162025e-19	0.520960801746034	0.856	0.553	1.0177625206598e-14	PTGER4
Rejected	14	3.28089301071914e-19	0.269124894042711	0.423	0.177	1.10034589793499e-14	UBE2L6
Rejected	14	3.33832380117177e-19	0.459017832754302	0.511	0.243	1.11960703643699e-14	LZTFL1
Rejected	14	5.06594660457769e-19	0.601892734525808	0.743	0.44	1.69901717224327e-14	USP12
Rejected	14	6.77080712310885e-19	0.729210902181116	0.894	0.732	2.27079329294824e-14	IL32
Rejected	14	7.42880063664266e-19	0.690959683247432	0.729	0.464	2.49147115751722e-14	MAP3K8
Rejected	14	7.8139031511115e-19	0.389716139774869	0.648	0.337	2.62062683881978e-14	LDLR
Rejected	14	9.06883365119133e-19	0.572549841170576	0.845	0.604	3.04150542993655e-14	TIPARP
Rejected	14	1.21248558687938e-18	0.298289505507363	0.539	0.248	4.06643416127606e-14	MT1E
Rejected	14	1.69124625223433e-18	0.744189608937593	0.796	0.549	5.67210168074349e-14	FBXO34
Rejected	14	3.07828184988465e-18	0.365865689311965	0.574	0.293	1.03239416681431e-13	YARS
Rejected	14	4.74492375694445e-18	0.60006097666055	0.768	0.5	1.59135252960403e-13	TRAF1
Rejected	14	5.31828051833746e-18	0.281696274250897	0.268	0.092	1.78364492024002e-13	SLC1A4
Rejected	14	5.47429602731107e-18	0.881893944401562	0.616	0.351	1.83596940163959e-13	GZMA
Rejected	14	6.29983659002287e-18	0.545933075028142	0.912	0.716	2.11283919556187e-13	GAPDH
Rejected	14	7.52994396328403e-18	0.488555673231688	0.511	0.255	2.5253926064062e-13	VAV1
Rejected	14	8.28373933985257e-18	0.568446124791604	0.919	0.81	2.77820049979976e-13	PFN1
Rejected	14	1.26130929044953e-17	0.301879922906443	0.461	0.207	4.23017909830964e-13	CEACAM21
Rejected	14	3.00804113375077e-17	0.415101663775044	0.581	0.307	1.00883683543733e-12	BHLHE40
Rejected	14	3.11020707324497e-17	0.302028520494702	0.356	0.148	1.0431012482249e-12	PUS10
Rejected	14	3.35964328018073e-17	0.559130625796535	0.937	0.751	1.12675716330701e-12	RAB8B
Rejected	14	3.4196931364879e-17	0.406120203242478	0.437	0.201	1.14689668411531e-12	HIST1H1C
Rejected	14	4.12510877562249e-17	0.355466063832644	0.609	0.32	1.38347898116827e-12	NUB1
Rejected	14	5.25878129848176e-17	0.634019087474445	0.782	0.527	1.76369007188481e-12	VPS37B

Rejected	14	6.14548839932989e-17	0.417857181386358	0.775	0.475	2.06107389936726e-12	SPPL2A
Rejected	14	6.53955102343881e-17	0.657680006748724	0.637	0.375	2.19323462224091e-12	MIR4435-2HG
Rejected	14	7.60752399720495e-17	0.414703047834396	0.743	0.454	2.5514113981826e-12	CD28
Rejected	14	7.86157388929311e-17	0.43466089895346	0.704	0.432	2.63661465099112e-12	PARP14
Rejected	14	8.66225962853501e-17	0.290351888489717	0.405	0.177	2.90514863421807e-12	LMNB1
Rejected	14	1.12512809755537e-16	0.254770089983323	0.5	0.236	3.77345461358119e-12	IDH2
Rejected	14	1.20399488208085e-16	0.324726715719127	0.521	0.264	4.03795803552274e-12	SLC39A8
Rejected	14	1.43425969545749e-16	0.296892077578112	0.525	0.265	4.81022016662532e-12	CNN2
Rejected	14	1.78240936540989e-16	0.712199632661651	0.968	0.918	5.97784452971169e-12	GNG2
Rejected	14	1.79691016310648e-16	0.35697654451722	0.708	0.397	6.02647730502652e-12	ABRACL
Rejected	14	2.0753978573173e-16	0.306744292937724	0.56	0.281	6.96046933387077e-12	PSMB8
Rejected	14	4.75043241069027e-16	0.42897653773959	0.352	0.151	1.5932000218973e-11	SETBP1
Rejected	14	5.35059578629401e-16	0.473143498403172	0.947	0.76	1.79448281480729e-11	HSPA8
Rejected	14	5.46055337697308e-16	0.471450234339124	0.201	0.064	1.83136039156923e-11	MKI67
Rejected	14	7.04891529216239e-16	0.516707309476705	0.817	0.585	2.36406521068542e-11	ZNF331
Rejected	14	7.12828988825233e-16	0.677021749146891	0.923	0.792	2.39068586272207e-11	NFKB1
Rejected	14	8.32179845639142e-16	0.420772575802762	0.908	0.732	2.79096476630455e-11	TPM3
Rejected	14	8.55606735109248e-16	0.462619661694393	0.831	0.546	2.86953386820939e-11	LPXN
Rejected	14	9.1945872477426e-16	0.259899163593299	0.482	0.233	3.08368067114791e-11	SNX5
Rejected	14	1.19206388381695e-15	0.250963866428551	0.489	0.24	3.99794385354528e-11	XAF1
Rejected	14	1.28001159667925e-15	0.306197740849101	0.585	0.301	4.29290289294288e-11	CNOT9
Rejected	14	1.50214259822739e-15	0.715350333350574	0.732	0.485	5.03788584593502e-11	NR4A3
Rejected	14	1.70858954534341e-15	0.604100920299543	0.838	0.566	5.73026761717274e-11	BIRC3
Rejected	14	1.99178605037136e-15	0.404186665744947	0.356	0.159	6.68005205573548e-11	AC020571.1
Rejected	14	2.07332256129691e-15	0.327316824073932	0.651	0.358	6.95350920607759e-11	CALCOCO2
Rejected	14	2.37704845543726e-15	0.363156051560847	0.525	0.263	7.97214510984547e-11	AHR
Rejected	14	2.45587414423056e-15	0.298053423907845	0.669	0.365	8.23651070492047e-11	CTSC
Rejected	14	4.20070190745434e-15	0.334406358110735	0.391	0.174	1.40883140572204e-10	CD4
Rejected	14	4.53818395676314e-15	0.312001091810106	0.447	0.216	1.52201613541922e-10	ZSWIM4
Rejected	14	4.64366030109565e-15	0.29848710013644	0.271	0.104	1.55739079178146e-10	CASS4
Rejected	14	4.8172098423786e-15	0.315452786257158	0.687	0.393	1.61559583693694e-10	GDI2

Rejected	14	5.03863685565216e-15	0.376152193356876	0.986	0.893	1.68985802864862e-10	HNRNPA2B1
Rejected	14	5.44720561654991e-15	0.348955226576936	0.764	0.472	1.82688381967851e-10	ETF1
Rejected	14	7.14130542075489e-15	0.427066398490522	0.764	0.495	2.39505101201278e-10	COTL1
Rejected	14	8.09568885770371e-15	0.651060642043667	0.606	0.359	2.71513212909667e-10	IL2RA
Rejected	14	8.65733947277633e-15	0.557157091811367	0.835	0.579	2.90349851237973e-10	RAB11FIP1
Rejected	14	1.06283091778034e-14	0.452056420489693	0.285	0.118	3.56452233205171e-10	DUSP4
Rejected	14	1.13993618390832e-14	0.261642192067969	0.493	0.247	3.82311797359173e-10	CHMP4B
Rejected	14	1.16564606587968e-14	0.268309340498801	0.518	0.262	3.90934377574727e-10	KSR1
Rejected	14	1.19978437028578e-14	0.414513535656922	0.933	0.753	4.02383682106445e-10	RNF213
Rejected	14	1.24597807650637e-14	0.335215452206462	0.743	0.43	4.17876127298705e-10	EIF3J
Rejected	14	1.72327833266963e-14	0.285632824871639	0.423	0.202	5.7795308721074e-10	CGAS
Rejected	14	1.86914561751698e-14	0.390984098993296	0.764	0.503	6.26874057202844e-10	PSMB9
Rejected	14	2.05320713900895e-14	0.411391011771576	0.155	0.044	6.88604610280822e-10	DIAPH3
Rejected	14	2.10824845931916e-14	0.353308905888021	0.532	0.28	7.07064368286461e-10	TP53INP1
Rejected	14	2.41502946998466e-14	0.296825310420033	0.468	0.235	8.09952583643455e-10	CHSY1
Rejected	14	2.66800449115042e-14	0.360585013865832	0.437	0.213	8.9479534624203e-10	CD40LG
Rejected	14	2.93613339654523e-14	0.341030173779546	0.694	0.418	9.84720418533338e-10	CKLF
Rejected	14	3.61887589187572e-14	0.266481101293092	0.155	0.044	1.21369859661728e-09	TNFRSF9
Rejected	14	3.93240715236563e-14	0.310557710034804	0.187	0.059	1.31885071076039e-09	PLAUR
Rejected	14	5.45579697768761e-14	0.358808659491377	0.299	0.125	1.82976519037687e-09	EGR3
Rejected	14	5.47884053171455e-14	0.677228756792766	0.736	0.51	1.83749353752642e-09	DLEU2
Rejected	14	5.73433084822464e-14	0.487886021007608	0.884	0.631	1.92317987987758e-09	RANBP2
Rejected	14	5.75031080285822e-14	0.413425741639156	0.289	0.122	1.92853923706259e-09	NUSAP1
Rejected	14	5.87672135202554e-14	0.324799810038764	0.581	0.318	1.97093480704232e-09	IL4R
Rejected	14	7.0442393142854e-14	0.616748783012586	0.894	0.694	2.36249698122504e-09	NAMPT
Rejected	14	7.19387828154336e-14	0.338831853425665	0.525	0.279	2.41268289806401e-09	UCP2
Rejected	14	7.19670599779755e-14	0.416877541653159	0.954	0.823	2.41363125754134e-09	ARHGDIB
Rejected	14	7.7950915582782e-14	0.371738769005387	0.877	0.65	2.61431780681534e-09	SLC2A3
Rejected	14	9.60505864796009e-14	0.425668224467143	0.937	0.769	3.22134456935285e-09	LCP1
Rejected	14	1.19474848369015e-13	0.404417295355836	0.673	0.413	4.00694746460001e-09	GIMAP4
Rejected	14	1.23406903063442e-13	0.369431116828575	0.257	0.104	4.1388207149417e-09	ZFY

Rejected	14	1.42182902681009e-13	0.26449822417659	0.588	0.323	4.76853019011568e-09	PPP1CA
Rejected	14	1.42991080248736e-13	0.29552895925726	0.613	0.348	4.79563484938211e-09	M6PR
Rejected	14	1.42991265615631e-13	0.35409319389524	0.602	0.337	4.79564106621703e-09	RNF168
Rejected	14	1.89039140927901e-13	0.36752187505465	0.887	0.724	6.33999470843995e-09	CD3D
Rejected	14	1.91312418078501e-13	0.3890888000582	0.75	0.489	6.41623587751675e-09	TPM4
Rejected	14	1.99101509366569e-13	0.49454735823236	0.356	0.167	6.677466421136e-09	CCDC138
Rejected	14	2.25195750372365e-13	0.25896419847623	0.327	0.14	7.55261507598838e-09	AC243960.1
Rejected	14	2.44012216462965e-13	0.47990044087752	0.975	0.939	8.18368171573492e-09	ACTB
Rejected	14	2.53537210335708e-13	0.26922854261106	0.581	0.317	8.50313096023899e-09	CMC2
Rejected	14	2.55711209678652e-13	0.25332849454713	0.123	0.031	8.57604255020263e-09	DTL
Rejected	14	2.6095567684476e-13	0.3580143455204	0.461	0.239	8.75193149001955e-09	RBBP8
Rejected	14	4.55780308926818e-13	0.63907256678343	0.553	0.313	1.52859600007876e-08	GRAMD1B
Rejected	14	4.59400247439629e-13	0.37824510342212	0.923	0.784	1.54073654986303e-08	ARPC2
Rejected	14	4.67246514181625e-13	0.55334304570453	0.905	0.72	1.56705135926233e-08	NFAT5
Rejected	14	5.07505919293992e-13	0.37094217313811	0.993	0.994	1.70207335212819e-08	MT-ND3
Rejected	14	6.35303022819397e-13	0.33250156044572	0.817	0.573	2.13067927793169e-08	ARL6IP5
Rejected	14	6.6813005524987e-13	0.64636623105398	0.651	0.434	2.24077457929701e-08	ITGA1
Rejected	14	7.2740578795342e-13	0.37522103782088	0.965	0.902	2.43957353163818e-08	STK17B
Rejected	14	7.49766999784914e-13	0.46989660219279	0.694	0.432	2.51456856387864e-08	ARHGAP25
Rejected	14	7.58350669509155e-13	0.27412042506037	0.444	0.227	2.54335647539981e-08	ETV3
Rejected	14	8.1710181877491e-13	0.54580908513154	0.69	0.475	2.74039607980729e-08	PFKFB3
Rejected	14	8.91395436897458e-13	0.41986261335334	0.68	0.435	2.98956201626669e-08	CDK6
Rejected	14	9.70896143385602e-13	0.39509451003909	0.757	0.497	3.25619148568663e-08	S100A11
Rejected	14	1.05429795332538e-12	0.38107468910143	0.852	0.616	3.53590447586266e-08	CLEC2B
Rejected	14	1.06828236172911e-12	0.26452530932724	0.423	0.21	3.58280538476708e-08	MBOAT1
Rejected	14	1.52790514000796e-12	0.37859126263418	0.863	0.614	5.1242882585587e-08	ZFP36L1
Rejected	14	1.56128908335096e-12	0.40159450597112	0.771	0.518	5.23625132774245e-08	ENO1
Rejected	14	2.0253166458481e-12	0.58283838496490	0.412	0.21	6.79250696684536e-08	HIST1H4C
Rejected	14	2.33623541561976e-12	0.26150696781954	0.567	0.317	7.83526633690554e-08	BST2
Rejected	14	2.34490068722134e-12	0.56069268594419	0.835	0.589	7.86432792480294e-08	OSBPL3
Rejected	14	2.59147699426697e-12	0.25999085179536	0.613	0.351	8.69129554337257e-08	JPT1

Rejected	14	2.64544249313619e-12	0.35959392013948	0.342	0.163	8.87228503348016e-08	MIR222HG
Rejected	14	2.70547026919867e-12	0.27992398238355	0.637	0.367	9.07360618883849e-08	SERPINB1
Rejected	14	2.72736117137233e-12	0.33653832068822	0.609	0.351	9.14702389654851e-08	SLAMF1
Rejected	14	4.02302432771244e-12	0.48438783420855	0.665	0.425	1.3492418990282e-07	NUP58
Rejected	14	4.63689744592649e-12	0.26907676506198	0.475	0.25	1.55512266541483e-07	CASP3
Rejected	14	4.79328397966533e-12	0.29911012228737	0.151	0.049	1.60757158110016e-07	TPX2
Rejected	14	4.87649245709793e-12	0.69557846900094	0.915	0.789	1.6354780402615e-07	TANK
Rejected	14	5.03189579974087e-12	0.75386752431189	0.556	0.344	1.68759721331709e-07	EGR1
Rejected	14	5.35805246647891e-12	0.50129199721556	0.405	0.213	1.7969836362077e-07	PHF6
Rejected	14	5.73297277418252e-12	0.33714904458599	0.613	0.352	1.92272440900533e-07	SLC7A5
Rejected	14	6.40434857119843e-12	0.38572093182916	0.366	0.183	2.14789042380853e-07	NFE2L3
Rejected	14	7.19086451505706e-12	0.46205972538303	0.972	0.869	2.41167214105984e-07	GLS
Rejected	14	7.32512190697147e-12	0.32248299121299	0.768	0.501	2.45669938516009e-07	CAP1
Rejected	14	7.42213480799451e-12	0.32292746876086	0.62	0.383	2.4892355719052e-07	GIMAP7
Rejected	14	8.32100440816033e-12	0.26943030576888	0.627	0.377	2.79069845840881e-07	ZC3H12D
Rejected	14	8.70375750744739e-12	0.37068010315186	0.75	0.529	2.91906619284771e-07	ARPC5
Rejected	14	8.71396923720050e-12	0.4187601641479	0.958	0.857	2.92249100277231e-07	ACTG1
Rejected	14	9.11520658241195e-12	0.33532257032400	0.908	0.677	3.05705798360932e-07	EIF4G2
Rejected	14	9.62866330963893e-12	0.33331980984693	0.732	0.47	3.22926110078671e-07	CD6
Rejected	14	1.04696297305256e-11	0.34259313597530	0.863	0.635	3.51130441902367e-07	MAN1A1
Rejected	14	1.16145384240812e-11	0.46340140240177	0.887	0.662	3.89528389666836e-07	BAZ1A
Rejected	14	1.34215340584658e-11	0.35595685514411	0.165	0.058	4.50131409252825e-07	TOP2A
Rejected	14	1.37123125811144e-11	0.33776713280999	0.69	0.435	4.59883539345416e-07	SKIL
Rejected	14	1.58983402876696e-11	0.42684164014753	0.303	0.143	5.33198536567865e-07	PPM1L
Rejected	14	1.72771368363898e-11	0.34597772930984	0.761	0.555	5.79440615218842e-07	PSME1
Rejected	14	1.73383214889341e-11	0.26691233825451	0.609	0.357	5.81492626095872e-07	CLIP1
Rejected	14	1.87386771940415e-11	0.28361995603167	0.761	0.479	6.28457755733765e-07	PNPLA8
Rejected	14	2.04584798701268e-11	0.32689580378708	0.331	0.161	6.86136497884311e-07	AC009299.3
Rejected	14	2.27040329996898e-11	0.30846086556243	0.873	0.626	7.61447858743597e-07	CYLD
Rejected	14	2.27397860454377e-11	0.41880162030329	0.761	0.538	7.62646944391891e-07	TENT5C
Rejected	14	2.32159138507256e-11	0.29949426543998	0.757	0.503	7.78615318725636e-07	PHF20L1

Rejected	14	2.46125210764102e-11	0.293951580712307	0.581	0.341	8.25454731860644e-07	ITGAL
Rejected	14	2.54509338848298e-11	0.410879091912625	0.676	0.426	8.53573420629423e-07	BTBD11
Rejected	14	2.82673737002448e-11	0.260926888708168	0.602	0.362	9.48031179158809e-07	AP3M2
Rejected	14	2.87646165251184e-11	0.300831201119375	0.415	0.215	9.6470770901942e-07	GALC
Rejected	14	3.17012303027849e-11	0.385998922290259	0.746	0.527	1.0631958618948e-06	TUT7
Rejected	14	3.61497787279466e-11	0.305527463744538	0.479	0.262	1.21239127897787e-06	AL606489.1
Rejected	14	3.97650153355925e-11	0.378458040032297	0.775	0.531	1.3336390843251e-06	LCP2
Rejected	14	4.14254744738085e-11	0.264504402793911	0.123	0.036	1.38932756290259e-06	ASPM
Rejected	14	5.01023020751422e-11	0.407171138136187	0.155	0.053	1.68033100699612e-06	DPF3
Rejected	14	5.41453275109119e-11	0.404159387275333	0.201	0.081	1.81592599406096e-06	C15orf53
Rejected	14	5.68260094108672e-11	0.357148718265627	0.725	0.483	1.90583070362166e-06	GLCCI1
Rejected	14	6.01088084093218e-11	0.318039941516808	0.838	0.625	2.01592921643183e-06	IFITM2
Rejected	14	6.54495404714594e-11	0.309838908299146	0.743	0.485	2.19504668833181e-06	UBR4
Rejected	14	6.61784232785951e-11	0.323562807822539	0.563	0.344	2.21949195991752e-06	PHLDA1
Rejected	14	6.70965273197259e-11	0.538946330576747	0.884	0.692	2.25028333324897e-06	BTG2
Rejected	14	6.84147840670116e-11	0.277215840883062	0.599	0.354	2.29449502803944e-06	NFKBID
Rejected	14	7.25481605300263e-11	0.383348481638256	0.877	0.643	2.43312020785602e-06	MT-ATP8
Rejected	14	8.28554679734125e-11	0.325837869687374	0.937	0.81	2.77880668489231e-06	MYL6
Rejected	14	1.02784691393002e-10	0.615177054237651	0.743	0.5	3.4471929799385e-06	TSC22D2
Rejected	14	1.15468521419027e-10	0.316141698027981	0.722	0.477	3.87258327135132e-06	PPP1R15B
Rejected	14	1.16263843898203e-10	0.349859882345036	0.778	0.527	3.89925679665793e-06	RCOR1
Rejected	14	1.2568836401029e-10	0.607897140088577	0.387	0.21	4.21533635217712e-06	TNF
Rejected	14	1.30291728214624e-10	0.347046832615037	0.887	0.7	4.36972398086206e-06	CD2
Rejected	14	1.31800756350735e-10	0.291806215094976	0.746	0.502	4.42033376649096e-06	RAC2
Rejected	14	1.40285120551371e-10	0.46529765796175	0.894	0.719	4.70488237305189e-06	MT-ND4L
Rejected	14	1.5232786041928e-10	0.291282832900041	0.799	0.556	5.10877178274183e-06	CD3G
Rejected	14	1.7950435258905e-10	0.319626111922694	0.662	0.413	6.02021697713155e-06	SPTY2D1
Rejected	14	2.25929410634866e-10	0.267093690359337	0.306	0.15	7.57722057387212e-06	PAK1
Rejected	14	2.67939536493007e-10	0.326692506384067	0.176	0.068	8.98615617490247e-06	NCAPG2
Rejected	14	2.96935161183507e-10	0.288301316203404	0.947	0.829	9.95861143577244e-06	UBC
Rejected	14	3.02710296724716e-10	0.420224145517426	0.768	0.531	1.01522979315535e-05	ATP1B3

Rejected	14	3.09456414375544e-10	0.325757286314516	0.908	0.683	1.0378549225327e-05	NR4A2
Rejected	14	3.22365621148947e-10	0.296834870992294	0.954	0.841	1.08114982020934e-05	GPBP1
Rejected	14	3.46350977865171e-10	0.25846881639362	0.197	0.079	1.16159190956421e-05	AL031777.3
Rejected	14	3.55471843170593e-10	0.324655206774149	0.504	0.306	1.19218146762554e-05	USP37
Rejected	14	3.61751698789426e-10	0.325390575490809	0.746	0.498	1.21324284739998e-05	PHTF2
Rejected	14	4.86026602552725e-10	0.436792617920918	0.718	0.494	1.63003601964133e-05	BORCS5
Rejected	14	5.25685799969126e-10	0.444304643430192	0.532	0.31	1.76304503593645e-05	MYO1E
Rejected	14	5.2691066528929e-10	0.335491687713772	0.859	0.694	1.76715298924722e-05	SH3BGRL3
Rejected	14	5.36070205984253e-10	0.628489636918807	0.278	0.136	1.79787225682999e-05	CCL20
Rejected	14	5.45769349211154e-10	0.334841953449647	0.919	0.728	1.83040124338437e-05	STAT3
Rejected	14	7.17804973572802e-10	0.522284128921332	0.729	0.479	2.40737432036846e-05	SERPINB9
Rejected	14	7.25409561995898e-10	0.28421022263157	0.718	0.472	2.43287858902184e-05	LAPTM5
Rejected	14	7.26158663855925e-10	0.262533269203586	0.236	0.104	2.43539092684e-05	AC098829.1
Rejected	14	7.57472979119281e-10	0.250960030021143	0.401	0.22	2.54041287737024e-05	ICE2
Rejected	14	7.88827655388893e-10	0.259902101347975	0.634	0.407	2.64557019064327e-05	IL2RG
Rejected	14	7.91301098573167e-10	0.493725209439319	0.859	0.695	2.65386562439469e-05	JARID2
Rejected	14	8.37225900470773e-10	0.433109184964992	0.926	0.807	2.80788822499888e-05	ARAP2
Rejected	14	9.84710313457039e-10	0.364703905469656	0.947	0.82	3.30252144927222e-05	YPEL5
Rejected	14	1.05822148478779e-09	0.325069958402007	0.975	0.892	3.5490632156813e-05	FNBP1
Rejected	14	1.05926059056082e-09	0.350909194492285	0.972	0.9	3.55254816862287e-05	PABPC1
Rejected	14	1.08098188612345e-09	0.71210087673048	0.359	0.2	3.62539704968084e-05	IFNG
Rejected	14	1.15264967302947e-09	0.334668637060635	0.905	0.691	3.86575647340625e-05	EVL
Rejected	14	1.3475335875573e-09	0.294902264553802	0.75	0.527	4.51935814594968e-05	CORO1A
Rejected	14	1.39424692493916e-09	0.395139443350479	0.623	0.403	4.67602533686095e-05	MAF
Rejected	14	1.39431451250726e-09	0.567300582191358	0.634	0.429	4.67625201204685e-05	ATF3
Rejected	14	1.46654109445471e-09	0.273579203582485	0.549	0.33	4.9184855225822e-05	EPG5
Rejected	14	1.51914680304843e-09	0.281577656839854	0.736	0.5	5.09491454806382e-05	LDHA
Rejected	14	1.82135527839435e-09	0.357437866260446	0.764	0.547	6.10846133267896e-05	LIMS1
Rejected	14	1.88281844900516e-09	0.716372043579748	0.366	0.205	6.31459651427351e-05	MIR155HG
Rejected	14	1.90133902992392e-09	0.324380126535129	0.88	0.685	6.37671083855886e-05	ACTR3
Rejected	14	2.01690964692612e-09	0.331949100089328	0.852	0.615	6.76431157386082e-05	SP140

Rejected	14	2.23323369503154e-09	0.37544857314600[illegible]	0.736	0.506	7.48981916639678e-05	FAM177A1
Rejected	14	2.55999870682143e-09	0.45664064214624[illegible]	0.338	0.186	8.58572366293772e-05	CTLA4
Rejected	14	2.68277437911918e-09	0.29994521602405[illegible]	0.923	0.817	8.99748871268989e-05	CFL1
Rejected	14	2.69351637132713e-09	0.27095557731215[illegible]	0.549	0.322	9.03351520615694e-05	EIF4A3
Rejected	14	3.13008126557307e-09	0.27986117792390[illegible]	0.437	0.252	0.00010497666548479	ARHGAP10
Rejected	14	3.1660853295275e-09	0.31962587707006[illegible]	0.842	0.634	0.000106184169781693	HSPD1
Rejected	14	4.04144554410917e-09	0.39033335022625[illegible]	0.634	0.385	0.000135542000658333	CMTM6
Rejected	14	4.58064476585252e-09	0.26456015562027[illegible]	0.806	0.577	0.000153625664157162	ARPC3
Rejected	14	5.09713596064316e-09	0.34527177369784[illegible]	0.93	0.787	0.00017094774584805	CLEC2D
Rejected	14	5.59850873073737e-09	0.32920747430551[illegible]	0.57	0.362	0.00018776278581147	TESPA1
Rejected	14	5.84000853623347e-09	0.26560942879492[illegible]	0.236	0.111	0.000195862206288198	CENPF
Rejected	14	5.98552313229989e-09	0.28080736727899[illegible]	0.725	0.497	0.000200742474811074	RUNX1
Rejected	14	8.22833471587669e-09	0.26789128283005[illegible]	0.451	0.265	0.000275961889701072	KIF1B
Rejected	14	8.27640803698863e-09	0.325087001451124	0.88	0.677	0.000277574172744525	PTPN1
Rejected	14	1.47124786522159e-08	0.32431749291393[illegible]	0.915	0.774	0.000493427109038018	HIVEP2
Rejected	14	1.50352081274665e-08	0.26020046577335[illegible]	0.746	0.525	0.000504250810178971	SLFN5
Rejected	14	1.58217349292705e-08	0.25556945552489[illegible]	0.701	0.483	0.000530629346057873	H2AFV
Rejected	14	1.62662142233428e-08	0.30570107409368	0.778	0.567	0.00054553629262247	N4BP1
Rejected	14	1.63471715009594e-08	0.27903810987210[illegible]	0.771	0.542	0.000548251437799175	STK24
Rejected	14	1.77735227317255e-08	0.37586758461716[illegible]	0.898	0.707	0.000596088405376609	VMP1
Rejected	14	2.03198961624892e-08	0.30380134153391	0.673	0.454	0.000681488677497562	FOSL2
Rejected	14	2.39721623159029e-08	0.35367951694360[illegible]	0.507	0.322	0.000803978379750752	NFATC1
Rejected	14	2.86945087882123e-08	0.47192327632810[illegible]	0.873	0.719	0.000962356435739064	ELOVL5
Rejected	14	3.53772193870643e-08	0.33941710454289[illegible]	0.606	0.425	0.00118648118380336	SPTBN1
Rejected	14	4.2106097418429e-08	0.27755727088885[illegible]	0.592	0.387	0.00141215429521927	HMGCS1
Rejected	14	4.56594185761014e-08	0.32699867364796[illegible]	0.947	0.813	0.00153132558020529	CDK17
Rejected	14	4.64903927445047e-08	0.32077427981577[illegible]	0.852	0.653	0.0015591947918652	TAB2
Rejected	14	4.82880085038473e-08	0.40247526013776[illegible]	0.894	0.776	0.00161948322920203	PDE4D
Rejected	14	5.07919620746206e-08	0.45919597407524	0.278	0.152	0.00170346082405863	CCR6
Rejected	14	5.44565875052204e-08	0.30357151877667[illegible]	0.616	0.411	0.00182636503175008	RILPL2
Rejected	14	5.51485000919138e-08	0.27290646141139[illegible]	0.616	0.4	0.00184957039608261	MAP3K1

Rejected	14	7.04893420469416e-08	0.2512044104340866	0.637	0.434	0.00236407155357033	RB1
Rejected	14	7.68781837877294e-08	0.3982765830572022	0.732	0.539	0.00257834052787287	PRKX
Rejected	14	7.80395573134983e-08	0.3986448733897755	0.873	0.673	0.00261729067318011	CNOT2
Rejected	14	8.1978842049821e-08	0.2982259376291688	0.866	0.655	0.0027494064046669	G3BP2
Rejected	14	8.43097625774439e-08	0.2886992247954222	0.335	0.189	0.00282758081732231	CPEB2
Rejected	14	8.8936438086994e-08	0.2728771212967077	0.489	0.299	0.0029827502605616	NSUN6
Rejected	14	9.48486750190297e-08	0.25178490938757	0.743	0.515	0.00318103486278822	TMEM123
Rejected	14	9.52392684249587e-08	0.5022406750597244	0.82	0.678	0.00319413458443626	RASGEF1B
Rejected	14	1.00036967961497e-07	0.30876184769199	0.567	0.381	0.00335503983149268	EZH2
Rejected	14	1.04332325884871e-07	0.30803281026435	0.926	0.815	0.00349909754552681	BRAF
Rejected	14	1.0517329153434e-07	0.2502453126943822	0.447	0.273	0.00352730185147869	SPDYA
Rejected	14	1.17608367597548e-07	0.2920086972231377	0.792	0.597	0.00394434943248656	PBX4
Rejected	14	1.17904318232586e-07	0.2557478027102499	0.261	0.135	0.00395427502488445	SLAMF7
Rejected	14	1.46491850689733e-07	0.3174466355961333	0.866	0.71	0.00491304368843226	CHD1
Rejected	14	1.80003122701814e-07	0.3629204578516211	0.785	0.598	0.00603694472917344	NCOA3
Rejected	14	1.91351882869648e-07	0.2581832046004899	0.785	0.557	0.00641755944768226	GTF2B
Rejected	14	2.09909453329263e-07	0.2619769010494477	0.859	0.642	0.00703994324575683	CAPZB
Rejected	14	2.23270672351606e-07	0.2814426844432422	0.75	0.543	0.00748805180932817	MLLT3
Rejected	14	2.43526331232686e-07	0.3543680658286944	0.405	0.243	0.00816738609688183	ALCAM
Rejected	14	2.60393467848551e-07	0.2880587100924011	0.711	0.477	0.00873307612470469	CHORDC1
Rejected	14	2.72424804984097e-07	0.2659829743829588	0.845	0.669	0.00913658310955663	XRN1
Rejected	14	2.74231911172708e-07	0.3267632708702011	0.965	0.918	0.00919718983691028	TMSB10
Rejected	14	2.86983898465243e-07	0.2587459262664599	0.655	0.454	0.00962486598672732	OGDH
Rejected	14	3.00240262035059e-07	0.3043282300540022	0.764	0.575	0.0100694579081318	SFMBT2
Rejected	14	3.25921040968724e-07	0.2767074334984444	0.926	0.78	0.0109307398720091	HLA-E
Rejected	14	3.56020255130015e-07	0.3177980987178611	0.401	0.241	0.0119402073165504	GRIP1
Rejected	14	3.60228243213873e-07	0.2549351689110077	0.535	0.343	0.0120813348209069	STOM
Rejected	14	4.9599710191964e-07	0.4381708339653811	0.81	0.695	0.0166347508041809	BICDL1
Rejected	14	6.04534464409998e-07	0.2856758046963477	0.415	0.262	0.0202748768673825	DDX3Y
Rejected	14	7.11047373120804e-07	0.26337785162591	0.641	0.441	0.0238471067997255	RANBP9
Rejected	14	7.47163079879121e-07	0.2726033713493899	0.979	0.94	0.025058355372986	DENND4A

Rejected	14	8.0202085717002e-07	0.30054842663747{	0.764	0.54	0.0268981755077681	IFRD1
Rejected	14	8.13120326495707e-07	0.34589190736933	0.31	0.183	0.027270429510013	ZDBF2
Rejected	14	9.2669067614014e-07	0.40732809625173	0.521	0.338	0.031079351896388	TUBB
Rejected	14	9.3880031827609e-07	0.25941297100249	0.87	0.671	0.0314854850743435	RNF149
Rejected	14	1.03756695778686e-06	0.26013778284127	0.771	0.575	0.0347979206302556	RAB27A
Rejected	14	1.12793252729945e-06	0.46906842018180	0.542	0.399	0.0378286011005691	IL12RB2
Rejected	14	1.16938420847034e-06	0.27498335007756	0.827	0.659	0.0392188075836784	TNIK
Rejected	14	1.18733430585844e-06	0.28335092455136	0.915	0.76	0.0398208179498805	NCL
Rejected	14	1.22583596955468e-06	0.30251728881289	0.986	0.941	0.0411120867469247	STAT4
Rejected	20	4.17504348550149e-68	1.22390773815315	0.782	0.454	1.40022608416749e-63	MT1G
Rejected	20	2.40197792447964e-51	0.92444221819535	0.961	0.916	8.05575356311981e-47	TMSB10
Rejected	20	2.24306655307538e-48	0.93774805089817	0.795	0.538	7.52279660570422e-44	REG1A
Rejected	20	1.08297670962639e-41	0.62125530984663	0.225	0.044	3.63208728874499e-37	C1QB
Rejected	20	1.03876850087765e-39	0.73199717864993	0.945	0.939	3.48382179824346e-35	RPL41
Rejected	20	4.03253981695695e-39	0.7854586925348	0.931	0.885	1.35243320381102e-34	FTL
Rejected	20	5.22641164293645e-38	0.94832869589787	0.471	0.22	1.75283393680803e-33	MT1H
Rejected	20	3.61164193732747e-37	0.48098055753709	0.2	0.038	1.21127247294089e-32	C1QA
Rejected	20	3.60899249936682e-30	0.68890499459605	0.878	0.846	1.21038390443764e-25	RPL26
Rejected	20	5.49176989335213e-30	0.55710968043358	0.984	0.987	1.84182978683244e-25	B2M
Rejected	20	1.14921107573511e-26	0.76727380720621	0.446	0.247	3.85422410580042e-22	MT1E
Rejected	20	5.1481759554364e-26	0.78451450978243	0.336	0.148	1.72659525193426e-21	AGR2
Rejected	20	1.40203939954123e-25	0.71417181711749	0.818	0.779	4.70215973818138e-21	S100A6
Rejected	20	1.42678756507741e-25	0.60330813798608	0.915	0.927	4.78516013575662e-21	RPS15A
Rejected	20	4.27632571511976e-25	0.7025331767801	0.478	0.283	1.43419411833686e-20	MT1X
Rejected	20	2.3163556300793e-24	0.834039451110244	0.685	0.572	7.76859351215994e-20	MT2A
Rejected	20	2.38512385180783e-24	0.34865396470193	0.106	0.015	7.9992283741931e-20	SPINK4
Rejected	20	5.03124171787488e-24	0.56415089422443	0.876	0.858	1.68737784734088e-19	FAU
Rejected	20	6.09830532078578e-23	0.49802776511580	0.92	0.901	2.04524963848514e-18	RPS15
Rejected	20	1.51509089464187e-22	0.55787075722556	0.926	0.908	5.0813118424499e-18	RPL30
Rejected	20	2.33839837231855e-22	0.54235844768944	0.913	0.91	7.84252046108194e-18	RPS23
Rejected	20	2.93425354698238e-22	0.56147492056738	0.816	0.786	9.8408995458695e-18	ATP5F1E

Rejected	20	1.33868611696821e-21	1.079545571118	0.352	0.17	4.48968549908798e-17	GZMK
Rejected	20	4.31192263455901e-21	0.324205907435872	0.115	0.022	1.4461326131784e-16	C1QC
Rejected	20	3.2647344263717e-20	0.486903945537255	0.91	0.898	1.09492663191654e-15	RPL29
Rejected	20	1.37187895955121e-19	0.522876327009597	0.908	0.914	4.60100765454285e-15	RPS27A
Rejected	20	1.88339150035855e-19	0.388702146141472	0.117	0.025	6.31651841390252e-15	MT1B
Rejected	20	4.29633765637717e-19	0.528224803799406	0.885	0.887	1.44090572319578e-14	RPL19
Rejected	20	6.1963688876709e-19	0.478704310971046	0.901	0.91	2.07813819754707e-14	RPL18
Rejected	20	7.31940024094099e-19	0.486987641889307	0.848	0.856	2.45478045280679e-14	HMGB1
Rejected	20	1.27067577268782e-17	0.465422620560757	0.952	0.932	4.2615924064404e-13	RPS19
Rejected	20	2.72020414032938e-17	0.454668323885886	0.915	0.913	9.12302064583669e-13	RPS24
Rejected	20	3.41851026717851e-17	0.456503858536825	0.94	0.931	1.14649997340633e-12	RPS14
Rejected	20	1.99580890426067e-16	0.474771858195148	0.892	0.905	6.69354390310943e-12	RPL14
Rejected	20	2.96492764601362e-16	0.420784402992231	0.943	0.95	9.94377433920049e-12	PTMA
Rejected	20	1.17453356800106e-15	0.685582212918254	0.841	0.818	3.93915068036196e-11	CXCR4
Rejected	20	2.33440463636537e-15	0.437696804451921	0.878	0.874	7.82912626944219e-11	RPL35A
Rejected	20	4.61499930370343e-15	0.389816008059148	0.945	0.935	1.54777846647605e-10	RPS28
Rejected	20	8.60355896147386e-15	0.53712442694824	0.784	0.78	2.8854616044991e-10	RPL36AL
Rejected	20	8.73572341417801e-15	0.568666318266955	0.68	0.645	2.92978691864702e-10	CYBA
Rejected	20	9.0508551872966e-15	0.420177865262184	0.878	0.873	3.03547581271553e-10	RPL35
Rejected	20	1.17690735580258e-14	0.717599184534932	0.637	0.566	3.94711188989068e-10	HCST
Rejected	20	1.54896419443719e-14	0.496379060983018	0.526	0.422	5.19491611530346e-10	UQCR10
Rejected	20	1.57836929697583e-14	0.41173545525879	0.853	0.855	5.29353494819753e-10	RPL36
Rejected	20	1.79803438325171e-14	0.616412672032596	0.782	0.758	6.03024771454959e-10	S100A10
Rejected	20	2.83282347479528e-14	0.383909470555081	0.936	0.937	9.50072336976841e-10	RPS27
Rejected	20	3.12273789876476e-14	0.434769355051796	0.841	0.852	1.04730383648772e-09	HLA-C
Rejected	20	3.25841685600047e-14	0.451992409770748	0.818	0.822	1.09280784516544e-09	SERF2
Rejected	20	3.95857302875167e-14	0.415413111532294	0.855	0.863	1.32762622238274e-09	UBA52
Rejected	20	6.60563139295737e-14	0.411474723981259	0.89	0.883	2.21539665657004e-09	RPL8
Rejected	20	1.03741719639325e-13	0.371952835034249	0.924	0.935	3.47928979326367e-09	RPL28
Rejected	20	1.62558618456304e-13	0.370931165280838	0.931	0.926	5.45189094578752e-09	RPL11
Rejected	20	2.59412417989613e-13	0.594529667189368	0.361	0.224	8.70017367453565e-09	FABP5

Rejected	20	3.88446122823713e-13	0.64102683626075	0.49	0.374	1.30277060672617e-08	OLFM4
Rejected	20	8.88139383088642e-13	0.36163912983121	0.899	0.925	2.97864186300269e-08	HLA-B
Rejected	20	1.07074512258258e-12	0.39013037210965	0.979	0.96	3.59106499211745e-08	RPLP1
Rejected	20	1.10318212891333e-12	0.42347900633402	0.887	0.895	3.69985222394951e-08	RPS13
Rejected	20	1.30940581820274e-12	0.36258472695434	0.908	0.9	4.39148523308835e-08	RPS9
Rejected	20	1.36997833708137e-12	0.38989332128732	0.945	0.941	4.59463334690351e-08	RPS12
Rejected	20	3.62184346421267e-12	0.52410765742543	0.745	0.733	1.21469386102765e-07	ATP5MG
Rejected	20	4.16906682563796e-12	0.46010392716435	0.752	0.727	1.39822163198246e-07	TOMM7
Rejected	20	4.19501310762585e-12	0.49391093331252	0.561	0.486	1.40692349603556e-07	COX6B1
Rejected	20	4.54502060031923e-12	0.39602523548353	0.903	0.906	1.52430900893506e-07	RPL7A
Rejected	20	1.09955791458717e-11	0.38507141778318	0.871	0.87	3.68769733394244e-07	NACA
Rejected	20	1.66764456048848e-11	0.42513787666330	0.743	0.729	5.59294632696627e-07	COX7C
Rejected	20	2.41193991028774e-11	0.41492690615780	0.841	0.836	8.08916407112301e-07	RPL22
Rejected	20	2.8709099675194e-11	0.34294936352406	0.945	0.924	9.62845784906656e-07	RPL12
Rejected	20	3.14071300875899e-11	0.43427451935418	0.786	0.786	1.05333232887759e-06	PFDN5
Rejected	20	3.95364651484759e-11	0.33644822228052	0.906	0.916	1.32597396814959e-06	RPLP2
Rejected	20	4.90211725617866e-11	0.39292862035323	0.901	0.92	1.6440720853772e-06	RPS8
Rejected	20	5.53221431502202e-11	0.34007354836263	0.933	0.94	1.85539403697209e-06	RPL39
Rejected	20	6.34528748213974e-11	0.30534024265966	0.952	0.946	2.12808251576003e-06	RPS2
Rejected	20	6.50849087371892e-11	0.59579169935777	0.699	0.681	2.18281766922785e-06	CD52
Rejected	20	8.37475278192877e-11	0.34976620138960	0.949	0.952	2.80872458800327e-06	RPL10
Rejected	20	9.88146527898266e-11	0.46038759893853	0.545	0.463	3.31404582526521e-06	RPS27L
Rejected	20	1.14537860810401e-10	0.33337415550566	0.901	0.916	3.84137077585922e-06	RPL9
Rejected	20	1.15555236808722e-10	0.36308956635501	0.853	0.854	3.87549153209092e-06	RPL37A
Rejected	20	1.1906410460211e-10	0.34785230442064	0.897	0.903	3.99317194014555e-06	RPS25
Rejected	20	1.25122861393326e-10	0.41408909346307	0.917	0.921	4.19637052540937e-06	RPS3
Rejected	20	2.00466637661894e-10	0.32031179307694	0.924	0.941	6.72325009390461e-06	RPL13
Rejected	20	2.18602792936171e-10	0.76105257090666	0.471	0.361	7.33150046949329e-06	GZMA
Rejected	20	2.18753203393612e-10	0.44004057233941	0.671	0.66	7.33654493541497e-06	TMA7
Rejected	20	2.98979049293574e-10	0.38716887998764	0.784	0.789	1.00271593552079e-05	BTF3
Rejected	20	3.06581337737e-10	0.45477639511181	0.687	0.653	1.02821249050235e-05	RPS10

Rejected	20	3.72776897892457e-10	0.357933751908217	0.871	0.906	1.25021916015172e-05	CALM1
Rejected	20	4.15305718990296e-10	0.441800640873091	0.186	0.093	1.39285232034965e-05	MT1M
Rejected	20	6.18892789788296e-10	0.377490833994498	0.653	0.608	2.07564263839199e-05	UQCRB
Rejected	20	6.32326114638724e-10	0.311392266170077	0.899	0.905	2.12069532327535e-05	RPL37
Rejected	20	8.56012617641582e-10	0.351206309467915	0.885	0.902	2.87089511704634e-05	RPL18A
Rejected	20	8.70104405355137e-10	0.477896829595973	0.492	0.418	2.91815615468006e-05	MZT2B
Rejected	20	9.44541821114602e-10	0.446133105167738	0.611	0.586	3.16780435965415e-05	SSR4
Rejected	20	9.81768285787473e-10	0.340897489953111	0.906	0.895	3.29265447687403e-05	RPL21
Rejected	20	9.95324966937804e-10	0.341661270314676	0.844	0.858	3.33812087411601e-05	RPL23A
Rejected	20	1.21508987844014e-09	0.482496485462653	0.531	0.478	4.07516843431254e-05	TMEM258
Rejected	20	1.27260517311265e-09	0.387967698535042	0.736	0.751	4.26806322958522e-05	HINT1
Rejected	20	1.44677014608687e-09	0.341601604807364	0.924	0.922	4.85217771594614e-05	RPS3A
Rejected	20	1.85844034661234e-09	0.336296870436004	0.11	0.041	6.23283723446845e-05	LAIR2
Rejected	20	1.90575671673424e-09	0.428048139526166	0.949	0.944	6.39152687658331e-05	BTG1
Rejected	20	1.91995647980317e-09	0.380821778044167	0.92	0.912	6.43915004196386e-05	RPL32
Rejected	20	2.06760222253155e-09	0.435996796993849	0.531	0.468	6.93432433392632e-05	TXN
Rejected	20	2.45722810774398e-09	0.432398659456996	0.177	0.09	8.24105162775176e-05	TFF3
Rejected	20	2.49957219766508e-09	0.416761982189613	0.6	0.567	8.38306523652916e-05	COX7A2
Rejected	20	3.47472415623006e-09	0.516810549434097	0.807	0.827	0.000116535298751644	MYL6
Rejected	20	6.46218026254981e-09	0.471620980531653	0.621	0.612	0.000216728601645396	OST4
Rejected	20	6.60056154075505e-09	0.45315628839326	0.745	0.762	0.000221369632953843	MYL12A
Rejected	20	7.4871053877455e-09	0.431230205459964	0.543	0.493	0.000251102540494209	COX5B
Rejected	20	7.72765021708988e-09	0.301418258069964	0.933	0.931	0.00025916993298076	RPS18
Rejected	20	8.28294596360394e-09	0.25400320347575	0.959	0.945	0.000277793441727349	EIF1
Rejected	20	9.07647326797391e-09	0.504851642713329	0.216	0.116	0.000304406760461309	GNLY
Rejected	20	9.22389876836606e-09	0.54129631358782	0.457	0.397	0.000309351116893461	ATP5MC3
Rejected	20	1.08591280366203e-08	0.924546515684672	0.52	0.401	0.000364193436092171	CCL4
Rejected	20	1.54946441712234e-08	0.353727881551666	0.775	0.791	0.000519659376214491	EEF1D
Rejected	20	1.80495184783222e-08	0.390607670294979	0.545	0.488	0.00060534475072597	CD63
Rejected	20	1.93166159360687e-08	0.506926801897551	0.133	0.061	0.000647840665263873	GZMH
Rejected	20	2.34623272738619e-08	0.423864363265845	0.676	0.664	0.000786879532110779	MYL12B

Rejected	20	2.3633552442145e-08	0.338827510386291	0.899	0.911	0.000792622081804657	RPL5
Rejected	20	2.65616956186775e-08	0.279444737643525	0.892	0.885	0.000890826147659206	RPL27A
Rejected	20	2.70254401014322e-08	0.328880747674619	0.906	0.929	0.000906379210121833	RPL34
Rejected	20	2.95722291386993e-08	0.387393055107441	0.745	0.715	0.000991793420853696	CD2
Rejected	20	3.43638565616049e-08	1.04362607464434	0.248	0.158	0.00115249502136311	GZMB
Rejected	20	3.82638496951875e-08	0.765875475407985	0.752	0.734	0.0012832929910772	S100A4
Rejected	20	5.19559851661396e-08	0.390564314604095	0.692	0.703	0.00174249983050199	COX4I1
Rejected	20	5.82278651881982e-08	0.373639093195106	0.805	0.809	0.00195284614268179	ITM2B
Rejected	20	5.91043278058082e-08	0.42695238491922	0.462	0.401	0.0019822409459512	ELOB
Rejected	20	6.00055225966192e-08	0.276994985631535	0.876	0.895	0.00201246521684542	RPL24
Rejected	20	6.31940339402498e-08	0.294493791536788	0.749	0.759	0.0021194015102881	UBB
Rejected	20	6.40396423373266e-08	0.330736675299647	0.897	0.907	0.00214776152470926	RPL6
Rejected	20	6.49620437314083e-08	0.502180670251144	0.375	0.271	0.00217869702266397	RPS4Y1
Rejected	20	7.98977237374352e-08	0.29533199483277	0.867	0.901	0.0026796098587061	RPS7
Rejected	20	1.05781752872538e-07	0.35323573960286	0.653	0.646	0.00354770842783917	CHCHD2
Rejected	20	1.21746499710899e-07	0.455615502287532	0.497	0.441	0.00408313410730414	C12orf57
Rejected	20	1.25182214672859e-07	0.268608922029892	0.885	0.893	0.0041983611156983 5	RPS20
Rejected	20	1.34146373090513e-07	0.43055852633326	0.57	0.551	0.00449900106070962	PSMA7
Rejected	20	1.66107930309727e-07	0.363771932318037	0.313	0.226	0.00557092776672761	NDUFA13
Rejected	20	1.70360471929455e-07	0.518217198039164	0.706	0.744	0.00571354950757005	RPS26
Rejected	20	1.80769637150482e-07	0.396924608517554	0.17	0.091	0.00606265209075286	AL158071.4
Rejected	20	2.39050965198081e-07	0.396960970313129	0.552	0.522	0.00801729127081323	DYNLL1
Rejected	20	2.44023305811368e-07	0.276690541605928	0.811	0.825	0.00818405363030167	RPS21
Rejected	20	2.55346529365796e-07	0.457049685538363	0.421	0.355	0.00856381190187008	LSM7
Rejected	20	2.89958708494795e-07	0.341918665277322	0.837	0.841	0.00972463516549845	RPS5
Rejected	20	3.08917351861047e-07	0.330159706369211	0.8	0.846	0.0103604701467158	H3F3A
Rejected	20	3.70599854315678e-07	0.293101025346447	0.94	0.919	0.0124291779140392	RPS4X
Rejected	20	4.37960096207104e-07	0.363254146015017	0.913	0.895	0.0146883057065938	ZFP36L2
Rejected	20	4.5440569771868e-07	0.406311262753727	0.66	0.664	0.0152398582900891	TAGLN2
Rejected	20	4.70572498945254e-07	0.453080275588696	0.549	0.52	0.0157820604696259	RAN
Rejected	20	4.90701231556359e-07	0.286387726670866	0.855	0.862	0.0164571379039372	RACK1

Rejected	20	5.10527904168168e-07	0.401840059577712	0.605	0.581	0.017122084849992	COMMD6
Rejected	20	5.76803655037361e-07	0.526619035079477	0.51	0.446	0.019344840982643	LITAF
Rejected	20	8.21395622466539e-07	0.37688910347207	0.513	0.479	0.0275479663862828	RBX1
Rejected	20	1.11949105664248e-06	0.30720167349449	0.823	0.834	0.0375454910576755	RPL27
Rejected	20	1.42611611053472e-06	0.421230464026482	0.699	0.714	0.0478290821151134	SH3BGRL3
Rejected	23	6.06171739507021e-23	0.651750851928068	0.814	0.641	2.03297877995865e-18	MT-ATP8
Rejected	23	9.20289497804282e-22	4.45449791681994	0.337	0.156	3.086466917736e-17	HBB
Rejected	23	2.85755185004525e-21	0.396282373421738	0.986	0.962	9.58365739468176e-17	DDX5
Rejected	23	3.95027441442761e-19	1.26023078638748	0.094	0.314	1.32484303311073e-14	IGHM
Rejected	23	5.32473989716018e-19	0.594234141763656	0.72	0.583	1.78581126670958e-14	HSPA5
Rejected	23	1.02196647568627e-18	0.49620427528389	0.998	0.992	3.42747116615661e-14	MBNL1
Rejected	23	1.2314518760958e-17	0.503330871062879	0.94	0.87	4.1300433020501e-13	PDE3B
Rejected	23	3.22159192445673e-17	0.438621954934154	0.822	0.714	1.0804574996243e-12	HNRNPDL
Rejected	23	3.61160741251472e-15	0.305366037478723	1	0.993	1.21126089400919e-10	PTPRC
Rejected	23	2.2060291009623e-14	0.478815484671354	0.894	0.829	7.39858039880736e-10	PPP1R15A
Rejected	23	4.73621050585282e-14	0.49684790862272	0.887	0.782	1.58843027945292e-09	RFX3
Rejected	23	1.26550920821693e-12	0.46274683590281	0.846	0.754	4.24426478251795e-08	ATP2B1
Rejected	23	4.81595181238751e-12	0.392477596437807	0.971	0.941	1.61517391883852e-07	STAT4
Rejected	23	2.84800947902959e-11	0.488142176021949	0.875	0.804	9.55165419076943e-07	ANXA1
Rejected	23	4.01664580568205e-11	0.359405383363721	0.73	0.592	1.34710267030964e-06	EPC2
Rejected	23	6.27771029849976e-11	0.361797624644679	0.518	0.385	2.10541847991085e-06	COPS2
Rejected	23	6.80965876905855e-11	0.373653203142974	0.79	0.716	2.28382335796686e-06	ARL4C
Rejected	23	7.3688983805367e-11	0.322920392674101	0.981	0.956	2.4713811388644e-06	PARP8
Rejected	23	1.84150208204254e-10	0.411830124180623	0.757	0.67	6.17602968275427e-06	USP47
Rejected	23	2.57259832397102e-10	0.325410080453529	0.904	0.826	8.62798025893401e-06	USP15
Rejected	23	3.40093457414984e-10	0.312364435416462	0.863	0.779	1.14060543747837e-05	KAT6A
Rejected	23	3.61899613576709e-10	0.326155925564534	0.908	0.876	1.21373892401357e-05	ANKRD12
Rejected	23	6.0456912602829e-10	0.337691161088496	0.882	0.784	2.02760393487368e-05	HERC1
Rejected	23	6.15316003010067e-10	0.329147192060229	0.964	0.94	2.06364681089516e-05	DENND4A
Rejected	23	1.24851593561434e-09	0.459772413918874	0.655	0.542	4.18727274486336e-05	SYTL2
Rejected	23	2.03796098828796e-09	0.49051434187061	0.658	0.542	6.83491356252015e-05	ANTXR2

Rejected	23	2.80479645624904e-09	0.31755021003971	0.643	0.483	9.40672635496802e-05	MT1G
Rejected	23	3.66420220988998e-09	0.336157361488645	0.619	0.513	0.00012289001371529	PBXIP1
Rejected	23	4.20231127905635e-09	0.379984948249244	0.494	0.366	0.000140937115676992	CEBPB
Rejected	23	4.34669462587461e-09	0.33418026223628	0.855	0.775	0.000145779444362583	LCP1
Rejected	23	5.83660049713228e-09	0.371506385818979	0.398	0.272	0.000195747907472822	CCDC173
Rejected	23	6.41620313008347e-09	0.353704912412134	0.422	0.306	0.00021518662057674	ECD
Rejected	23	1.07051483934664e-08	0.320770269489764	0.369	0.252	0.000359029266820075	NUS1
Rejected	23	1.80906853538152e-08	0.271399152835062	0.776	0.7	0.000606725405396253	SF3B1
Rejected	23	2.50900361487011e-08	0.37447924794708	0.692	0.627	0.000841469632355137	AP3B1
Rejected	23	3.6596091201978e-08	0.250470334243033	0.822	0.738	0.00122735970673194	SRRM2
Rejected	23	5.30786839390352e-08	0.28782641315828	0.588	0.457	0.00178015290194736	IL18R1
Rejected	23	1.18218496394126e-07	0.363512664020844	0.52	0.417	0.0039648119320662	BOD1L1
Rejected	23	1.34549841395385e-07	0.352909634109859	0.788	0.709	0.00451253258071843	SCAF11
Rejected	23	1.38420165445539e-07	0.312755279516288	0.581	0.493	0.00464233550871248	CNTRL
Rejected	23	1.4408093483595e-07	0.272532267458551	0.301	0.194	0.00483218639252808	AL157938.2
Rejected	23	1.64287254806862e-07	0.269668312465339	0.822	0.745	0.00550986595171254	CCSER2
Rejected	23	1.76668910414306e-07	0.328052641162188	0.711	0.628	0.005925121917475	GPR183
Rejected	23	1.79820120489415e-07	0.375336264184896	0.817	0.74	0.00603080720097401	ADK
Rejected	23	1.97198418694521e-07	0.290918696212022	0.564	0.467	0.00661364056617686	WBP11
Rejected	23	2.31992280212042e-07	0.380221876258849	0.451	0.363	0.00778055709375145	MT-ND6
Rejected	23	2.46222016445356e-07	0.426150625056203	0.405	0.297	0.00825779398754435	CD44-AS1
Rejected	23	2.99761666270512e-07	0.388211691130992	0.766	0.663	0.0100534067633804	FAM129A
Rejected	23	3.57714852119115e-07	0.29509201378909	0.39	0.284	0.0119970407103709	WDR75
Rejected	23	3.87032061716033e-07	0.330252271816631	0.764	0.706	0.0129802812858323	MKLN1
Rejected	23	5.64326241295824e-07	0.281102618074526	0.807	0.771	0.0189263734805793	NCL
Rejected	23	5.89706138211779e-07	0.392082183602713	0.583	0.513	0.0197775644633467	TMEM87A
Rejected	23	7.15590856789225e-07	0.654826153061655	0.795	0.766	0.023999486154997	ANKRD28
Rejected	23	8.33042894911844e-07	0.496561660413299	0.754	0.696	0.0279385926095534	ANK3
Rejected	23	8.5378951555742e-07	0.325625643004234	0.713	0.64	0.0286343927727648	DHX9
Rejected	23	9.01920791883896e-07	0.306692845120874	0.335	0.235	0.0302486195182021	KLHDC2
Rejected	23	9.54468551267364e-07	0.266730843571412	0.267	0.172	0.0320109662724049	ABCG1

Rejected	23	1.00155665941126e-06	0.356755388657996	0.655	0.588	0.0335902072433349	PDIA3
Rejected	23	1.24857740233164e-06	0.251410806486627	0.771	0.677	0.0418747889193986	CFLAR
Rejected	23	1.29156815925779e-06	0.330985330182751	0.648	0.573	0.0433166129251877	MON2
Rejected	23	1.4134105108793e-06	0.330702896134064	0.824	0.808	0.0474029617138699	NFKBIA
Rejected	27	6.36022903541501e-17	2.37374196116548	0.579	0.5	2.13309361389749e-12	DEFA5
Rejected	27	1.76560672071944e-16	0.754831706155497	0.888	0.944	5.92149181994885e-12	RPL39
Rejected	27	1.79319709178312e-16	0.431542999309529	0.987	0.995	6.01402440642222e-12	MT-ND3
Rejected	27	4.95300649906525e-15	2.23359478048305	0.288	0.117	1.6611393196565e-10	GNLY
Rejected	27	5.3821528012813e-15	0.634365436809396	0.837	0.882	1.80506640649372e-10	RPS29
Rejected	27	5.80593671419302e-15	0.555597704405929	0.876	0.907	1.94719505520606e-10	RPL37
Rejected	27	5.28797225340599e-14	0.487757241068711	0.97	0.988	1.7734801343473e-09	B2M
Rejected	27	2.12753913076704e-13	0.568645751389613	0.901	0.944	7.13534073676651e-09	RPL41
Rejected	27	1.50167207604049e-12	0.587709413597801	0.923	0.957	5.03630780862461e-08	TMSB4X
Rejected	27	1.11996115691187e-11	0.588579110250637	0.876	0.943	3.75612572805104e-07	RPS28
Rejected	27	1.22780382389704e-11	0.5662572927974	0.863	0.944	4.11780846458591e-07	RPS27
Rejected	27	1.40083678949972e-10	0.486468091488724	0.884	0.941	4.69812642462417e-06	RPS19
Rejected	27	1.77059981064334e-10	0.525630012633049	0.897	0.914	5.93823764493562e-06	RPS27A
Rejected	27	2.88336794546473e-10	0.552000114583376	0.807	0.859	9.67023941549961e-06	RPL36
Rejected	27	3.82084574008785e-10	0.535874885446701	0.88	0.914	1.28143524431066e-05	RPL30
Rejected	27	9.98182050307724e-10	0.63863977845884	0.79	0.836	3.34770296032204e-05	RPL27
Rejected	27	3.77698698426325e-09	0.688723209967104	0.807	0.823	0.000126672589478221	CXCR4
Rejected	27	1.36776640193928e-08	0.540802918177274	0.803	0.901	0.000458721495882396	RPL24
Rejected	27	3.57406360947161e-08	0.490153827465379	0.845	0.92	0.00119866945334459	RPS24
Rejected	27	6.11053796629121e-08	0.529991452706828	0.785	0.869	0.00204935222313475	UBA52
Rejected	27	7.73360977716245e-08	0.393746861973552	0.901	0.93	0.00259369804706474	RPL12
Rejected	27	8.93987772427028e-08	0.513852943787442	0.858	0.93	0.00299825619116577	TMSB10
Rejected	27	1.00684758661898e-07	0.625138600974169	0.674	0.737	0.00337676543600274	COX7C
Rejected	27	1.01608346351546e-07	0.46388934758652	0.906	0.95	0.0034077407199381 4	JUND
Rejected	27	1.27880710077025e-07	0.449238143258829	0.854	0.909	0.00428886325456328	RPS15
Rejected	27	1.31934683404893e-07	0.442601317703799	0.854	0.916	0.00442482541203331	RPS23
Rejected	27	1.33176063949512e-07	0.445329044871645	0.888	0.929	0.00446645883273873	RPL34

Rejected	27	1.71838929621983e-07	0.317117136222451	1	1	0.00576313402166208	MALAT1
Rejected	27	1.97307752057955e-07	0.47992125147231	0.803	0.894	0.00661730738851969	RPL27A
Rejected	27	3.32053662642629e-07	0.39151740442131	0.893	0.928	0.0111364157377085	RPS15A
Rejected	27	3.4071462285185e-07	0.49077377806984	0.794	0.882	0.0114268870212053	RPL35
Rejected	27	5.16357473634612e-07	0.40294895271764	0.906	0.945	0.0173175969507576	RPS12
Rejected	27	1.27826751468359e-06	0.51091305275507	0.764	0.863	0.0428705359074582	RPL37A
Rejected	30	1.32317424588075e-11	0.76292944869071	0.288	0.012	4.43766178583485e-10	TFPI2
Rejected	30	2.45774685029847e-93	0.98515370689517	0.602	0.178	8.24279138653102e-89	NRG1
Rejected	30	1.07782169842566e-64	0.57923059114512	0.325	0.061	3.61479841217998e-60	COL4A1
Rejected	30	1.06698848324934e-55	0.65098991907809	0.282	0.054	3.57846597512163e-51	IGFBP5
Rejected	30	1.10997796556994e-54	0.69477558030576	0.988	0.954	3.72264410092848e-50	SIK3
Rejected	30	3.28245195865446e-52	0.51530494807070	0.292	0.062	1.10086873789353e-47	COL4A2
Rejected	30	1.57896521399441e-47	0.67586416856373	0.975	0.911	5.29553353469446e-43	CD44
Rejected	30	5.75937660374771e-47	0.35446037141134	0.155	0.014	1.93157972536491e-42	MMP1
Rejected	30	3.65578457402021e-45	0.62417134096229	1	0.988	1.2260770304349e-40	AC016831.7
Rejected	30	3.20390632511025e-44	0.47655549130396	0.261	0.058	1.07452610331547e-39	LDB2
Rejected	30	4.25060198133614e-43	0.66246356614261	0.992	0.938	1.42556689250051e-38	SYTL3
Rejected	30	6.20800363086438e-42	0.57636252463523	0.994	0.979	2.0820402577193e-37	FYN
Rejected	30	5.33138190498749e-41	0.65912473329137	0.675	0.381	1.7880388632947e-36	IGLC3
Rejected	30	1.72214626551971e-39	0.67723903458737	0.967	0.827	5.77573414530002e-35	IQGAP2
Rejected	30	2.14746001429108e-39	0.71317891216645	0.542	0.263	7.20215139592944e-35	CD44-AS1
Rejected	30	2.22507000710192e-39	0.55228479487190	0.766	0.479	7.46243978981841e-35	IGLC2
Rejected	30	1.46617089796528e-38	0.50648468403106	0.319	0.101	4.91724395759597e-34	RBPMS
Rejected	30	1.00066388371235e-36	0.69875689254599	0.93	0.769	3.35602653319449e-32	RFX3
Rejected	30	1.95213280002613e-34	0.63565893972377	0.99	0.914	6.54706298472764e-30	RAB11A
Rejected	30	2.40869358967417e-34	0.53482345798420	0.509	0.245	8.07827656104922e-30	TIMP1
Rejected	30	1.14775338034693e-33	0.70994482253394	0.874	0.667	3.84933528700755e-29	RAPGEF2
Rejected	30	2.19961065934766e-33	0.82444545790623	0.896	0.709	7.37705422932019e-29	RORA-AS1
Rejected	30	8.12066526998027e-33	0.63942623100361	0.923	0.782	2.72350871824598e-28	DYRK1A
Rejected	30	9.01895696358457e-33	0.57088886242218	0.874	0.667	3.02477778644699e-28	SND1
Rejected	30	1.08345303573001e-32	0.59030329277330	0.876	0.639	3.63368479123129e-28	IGHA1

Rejected	30	1.61433703419004e-32	0.52144308512124	0.992	0.933	5.41416354526657e-28	DENND4A
Rejected	30	5.03013287218774e-31	0.67363914907340	0.466	0.231	1.68700596267432e-26	AL035634.1
Rejected	30	1.24129081333009e-30	0.46416804943291	0.271	0.089	4.16304112974646e-26	IGFBP4
Rejected	30	3.83250848299707e-29	0.46861857976786	0.288	0.102	1.28534669502756e-24	COL3A1
Rejected	30	5.78235737634913e-29	0.44645320046173	0.346	0.135	1.93928701687997e-24	SPARCL1
Rejected	30	7.33938460143316e-29	0.78174009600536	0.89	0.69	2.46148280762865e-24	AP001011.1
Rejected	30	1.56676089252186e-28	0.57605214095326	0.981	0.938	5.25460268133981e-24	STAT4
Rejected	30	1.62618366246057e-28	0.42201243088527	0.319	0.12	5.45389476716026e-24	TCF4
Rejected	30	2.57384763071544e-28	0.341161021101217	0.201	0.053	8.63217018389344e-24	ADAMTS9
Rejected	30	5.32436770122985e-28	0.37439438825331	0.257	0.083	1.78568643963847e-23	IGFBP7
Rejected	30	6.35824232202863e-28	0.62543438120651	0.793	0.62	2.13242730996196e-23	AHNAK
Rejected	30	1.84501658510232e-27	0.39260260857219	0.195	0.051	6.18781662311616e-23	CEMIP
Rejected	30	6.1193123652823e-27	0.50621243638906	0.308	0.124	2.05229498106838e-22	ADGRL2
Rejected	30	1.14808032529695e-26	0.33334132178618	0.184	0.047	3.85043179498092e-22	GNG11
Rejected	30	1.1559147639648e-26	0.28983328512510	0.213	0.061	3.87670693538514e-22	SPARC
Rejected	30	3.57128872080899e-26	0.33528674565515	0.197	0.054	1.19773881118492e-21	TMTC1
Rejected	30	5.54370368311599e-26	0.46950058131690	0.969	0.852	1.85924734124344e-21	SYNE2
Rejected	30	9.95558558894048e-26	0.561182951105834	0.439	0.226	3.33890429481886e-21	AF241726.2
Rejected	30	2.14983602703732e-25	0.58034377517310	0.888	0.741	7.21012006747777e-21	ATP2B1
Rejected	30	2.68352472993436e-25	0.45211231074199	0.451	0.228	9.00000523925385e-21	IFITM3
Rejected	30	3.0249853218648e-25	0.57734965163544	0.615	0.364	1.01451957724702e-20	A2M
Rejected	30	4.72731023933403e-25	0.37994091828238	0.226	0.073	1.58544530806785e-20	SOX5
Rejected	30	4.84681714390934e-25	0.38100016593716	0.209	0.063	1.62552553372431e-20	RGS5
Rejected	30	6.08512603717067e-25	0.55556224358971	0.758	0.583	2.0408295703463e-20	AGO2
Rejected	30	8.00977862599073e-25	0.64075049630475	0.841	0.677	2.68631955558477e-20	ZHX2
Rejected	30	1.37288478820763e-24	0.49762447272151	0.94	0.808	4.60438100269076e-20	CYTH1
Rejected	30	1.64711346810239e-24	0.59012176570262	0.946	0.848	5.5240891493218e-20	RORA
Rejected	30	2.06518612833119e-24	0.45674848043110	0.969	0.875	6.92622123719713e-20	UTRN
Rejected	30	6.67396196733433e-24	0.70497475615073	0.805	0.653	2.23831336460459e-19	MPP7
Rejected	30	1.21934597464122e-23	0.62325042382301	0.882	0.78	4.08944252975173e-19	SNX9
Rejected	30	3.74401298833198e-23	0.71612019217966	0.71	0.545	1.25566707602678e-18	SSBP2

Rejected	30	5.45209375040137e-23	0.386258905105191	1	0.951	1.82852320200961e-18	PARP8
Rejected	30	6.18525470595856e-23	0.444547386577435	0.942	0.782	2.07441072328438e-18	ELMO1
Rejected	30	1.68649688340172e-22	0.680140510712279	0.872	0.744	5.6561732475527e-18	LINC00513
Rejected	30	1.99407162106874e-22	0.748892941737212	0.762	0.584	6.68771740274033e-18	CAMK1D
Rejected	30	6.03237469255241e-22	0.550557998548854	0.58	0.381	2.02313782438823e-17	AC001226.2
Rejected	30	7.33533013204126e-22	0.432229211115979	0.93	0.808	2.460123019684e-17	STAG1
Rejected	30	1.01205122827728e-21	0.639456230828743	0.607	0.397	3.39421740939634e-17	PRKCE
Rejected	30	1.54720930069902e-21	0.389044259074059	0.288	0.121	5.18903055268438e-17	MCTP1
Rejected	30	1.59205744794533e-21	0.467812680461987	0.957	0.866	5.33944226891906e-17	PITPNC1
Rejected	30	2.37704885456855e-21	0.502524078622751	0.894	0.77	7.97214644845201e-17	LRRFIP1
Rejected	30	3.15851485683611e-21	0.395545460350412	0.952	0.857	1.0593027126857e-16	ARID1B
Rejected	30	3.8837306976532e-21	0.48571345829222	0.694	0.494	1.30252560137893e-16	RELB
Rejected	30	5.30923035484723e-21	0.500004126184444	0.731	0.536	1.78060967640866e-16	DENND1A
Rejected	30	5.34980520544832e-21	0.550906626200009	0.896	0.765	1.79421766980326e-16	HIVEP2
Rejected	30	3.21030382361836e-20	0.48016548274822	0.961	0.861	1.07667169636512e-15	ZBTB20
Rejected	30	3.58693988132663e-20	0.394656032745434	0.917	0.812	1.20298789739933e-15	MACF1
Rejected	30	4.10045066389138e-20	0.63233639671375	0.81	0.609	1.37520914365589e-15	PLCB1
Rejected	30	5.53372306172371e-20	0.537504426960179	0.7	0.511	1.8559000404409e-15	FNDC3B
Rejected	30	7.82113135549167e-20	0.396351664995313	0.203	0.07	2.6230510340048e-15	CALD1
Rejected	30	1.0491152913168e-19	0.38089175530438	0.888	0.751	3.51852286401828e-15	PPP6R3
Rejected	30	1.42023019866413e-19	0.324526435682554	0.172	0.054	4.76316804027977e-15	COL6A3
Rejected	30	1.6374527920168e-19	0.38704007992175	0.952	0.852	5.49168917386596e-15	DOCK8
Rejected	30	2.0195302934808e-19	0.547969430392873	0.909	0.8	6.77310069827591e-15	MGAT5
Rejected	30	2.12073433035367e-19	0.320744325838552	0.923	0.805	7.11251879714014e-15	BRAF
Rejected	30	2.1608698536181e-19	0.512029641605573	0.826	0.678	7.24712531506438e-15	ANK3
Rejected	30	3.1355406292675e-19	0.466229111511902	0.822	0.703	1.05159761624373e-14	GNB1
Rejected	30	3.22608145253298e-19	0.302178131888568	0.211	0.077	1.08196319755051e-14	CLIC4
Rejected	30	3.60325565522499e-19	0.519731097101966	0.795	0.648	1.20845988164936e-14	TAB2
Rejected	30	3.67005433117704e-19	0.532815135053381	0.491	0.308	1.23086282159015e-14	ESR2
Rejected	30	4.4003364413723e-19	0.417467648948373	0.886	0.76	1.47578483570744e-14	EXOC4
Rejected	30	5.31668361561098e-19	0.463837245771132	0.455	0.279	1.78310935100361e-14	AC090627.1

Rejected	30	5.54017068791422e-19	0.39678307730707	0.969	0.894	1.85806244531267e-14	ARID4B
Rejected	30	1.55495039901212e-18	0.39918955947202	0.948	0.866	5.21499264820684e-14	PDE3B
Rejected	30	1.59656760613033e-18	0.585862113011916	0.896	0.749	5.3545684374399e-14	SIPA1L1
Rejected	30	3.61856166247511e-18	0.41883082377378	0.894	0.782	1.2135932103609e-13	TANK
Rejected	30	4.83100957513488e-18	0.42781593233554	0.932	0.802	1.62022399130874e-13	DPYD
Rejected	30	5.03542767882904e-18	0.39743314380273	0.892	0.776	1.68878173492568e-13	KMT2C
Rejected	30	5.04396551427981e-18	0.44036336389392	0.528	0.341	1.69164515417916e-13	ACSL4
Rejected	30	1.11263043437776e-17	0.37326147744985	0.95	0.857	3.73153995081612e-13	PRRC2C
Rejected	30	1.4801161488601e-17	0.56366465684768	0.573	0.402	4.964013540047e-13	AC008569.1
Rejected	30	2.33742985411015e-17	0.25771495454659	0.13	0.036	7.83927224471464e-13	FBXL7
Rejected	30	3.6751350475622e-17	0.37841704036021	0.826	0.68	1.23256679225141e-12	PUM1
Rejected	30	4.18165915645881e-17	0.57786812980675	0.46	0.278	1.40244484789316e-12	DPYD-AS1
Rejected	30	6.23865289624264e-17	0.43722552647542	0.642	0.466	2.09231940834186e-12	AC058822.1
Rejected	30	6.88491247111644e-17	0.452117448031824	0.524	0.325	2.30906194456303e-12	ZBTB16
Rejected	30	7.41165524428205e-17	0.52870234330097	0.845	0.735	2.48572093582731e-12	AKT3
Rejected	30	7.50376630205956e-17	0.50075262507730	0.675	0.522	2.51661314238473e-12	XYLT1
Rejected	30	8.72976533457537e-17	0.63852078173141	0.882	0.801	2.92778869790989e-12	SCML4
Rejected	30	1.0829506739606e-16	0.30671298052825	0.242	0.104	3.63199997032906e-12	LRMDA
Rejected	30	1.50883537789937e-16	0.35721689695165	0.636	0.446	5.06033209039892e-12	APOA4
Rejected	30	1.66742924359136e-16	0.431190470370972	0.205	0.083	5.59222419715669e-12	PRR16
Rejected	30	1.94946120509567e-16	0.51231539318460	0.617	0.437	6.53810298964987e-12	AC009313.1
Rejected	30	2.16911813589743e-16	0.41730487137644	0.907	0.826	7.27478840417279e-12	CHST11
Rejected	30	2.90100052418772e-16	0.36055940286662	0.851	0.702	9.72937555802079e-12	USP9X
Rejected	30	3.14696204239397e-16	0.35721512868812	0.917	0.808	1.05542812977809e-11	PCNX1
Rejected	30	3.82295636334067e-16	0.50624940466249	0.799	0.655	1.28214310513719e-11	TLE4
Rejected	30	3.8858674329529e-16	0.37725680465044	0.267	0.126	1.30324221966374e-11	PLCG1-AS1
Rejected	30	4.12714615136929e-16	0.29543578407084	0.979	0.947	1.38416227624623e-11	SKAP1
Rejected	30	4.13203532730406e-16	0.25663295011499	0.164	0.056	1.38580200807124e-11	TIMP3
Rejected	30	5.55753770481196e-16	0.37510776794456	0.865	0.729	1.86388699543984e-11	CUL3
Rejected	30	5.95286829769211e-16	0.43145475142722	0.741	0.566	1.99647296967998e-11	TNKS
Rejected	30	6.41852426871431e-16	0.36372740492668	0.863	0.741	2.15264466924141e-11	KDM4C

Rejected	30	6.45593574364925e-16	0.431773826390293	0.857	0.728	2.16519172970508e-11	ZSWIM6
Rejected	30	7.76376418879383e-16	0.306374012723092	0.226	0.097	2.60381123363768e-11	TTC28
Rejected	30	9.30320081088125e-16	0.339204457545027	0.946	0.846	3.12010748795335e-11	KLF12
Rejected	30	1.03048182399746e-15	0.393175251897081	0.925	0.864	3.4560299413227e-11	DOCK10
Rejected	30	1.81925010351339e-15	0.402758404971546	0.789	0.619	6.10140099716322e-11	ABCC1
Rejected	30	2.42288774984857e-15	0.250364825139841	0.112	0.03	8.12588093544214e-11	FGF7
Rejected	30	2.72045424824208e-15	0.378173292115428	0.942	0.855	9.12385945775427e-11	ESYT2
Rejected	30	2.98627201639309e-15	0.471910209838335	0.518	0.34	1.00153590885791e-10	DISC1
Rejected	30	3.1276615946672e-15	0.376883829945509	0.973	0.9	1.04895514561948e-10	FOXP1
Rejected	30	3.20559566299742e-15	0.31188236376984	0.919	0.798	1.07509267345607e-10	KDM6A
Rejected	30	3.90117262371519e-15	0.376205282937193	0.934	0.836	1.3083752745416e-10	SLC38A1
Rejected	30	5.52125136108689e-15	0.347903093872892	0.354	0.201	1.85171728148132e-10	PTPRG
Rejected	30	6.11220168021829e-15	0.353317885042339	0.981	0.922	2.04991019951161e-10	CELF2
Rejected	30	7.25426617726244e-15	0.494249252342842	0.696	0.529	2.43293579053028e-10	ANTXR2
Rejected	30	8.25638954961271e-15	0.509910427685043	0.429	0.277	2.76902792714911e-10	FAM196B
Rejected	30	1.02435286890469e-14	0.336682974108658	0.381	0.217	3.43547465173254e-10	PTPRM
Rejected	30	1.19331064282054e-14	0.473534116511518	0.482	0.324	4.00212523389153e-10	SMURF1
Rejected	30	1.3808318583997e-14	0.427923251966884	0.795	0.663	4.63103388670092e-10	UBR5
Rejected	30	1.41069863902444e-14	0.252202595148363	0.178	0.069	4.73120109556016e-10	WWTR1
Rejected	30	1.50927849260664e-14	0.382653883428714	0.393	0.242	5.06181820850415e-10	IFFO2
Rejected	30	1.96413855696724e-14	0.363678959391724	0.886	0.761	6.58732789235675e-10	ANKRD11
Rejected	30	1.96830880467912e-14	0.305297568573781	0.969	0.914	6.60131406913282e-10	GNG2
Rejected	30	2.10294062933598e-14	0.345237474184317	0.861	0.736	7.05284228266702e-10	BCLAF1
Rejected	30	2.20676932094266e-14	0.336332552933001	1	0.983	7.4010629485775e-10	MT-ND5
Rejected	30	2.29580671446379e-14	0.354381430676201	0.754	0.589	7.69967655896867e-10	MSI2
Rejected	30	2.54584524079683e-14	0.397692981567027	0.329	0.186	8.53825576858441e-10	AC090772.2
Rejected	30	2.92711585443831e-14	0.549932940242659	0.834	0.756	9.81696115261519e-10	ANKRD28
Rejected	30	3.56677071394199e-14	0.286152935007096	0.973	0.891	1.19622356204186e-09	ARIH1
Rejected	30	3.60187308967148e-14	0.353272339569573	0.841	0.731	1.20799619681402e-09	SRRM2
Rejected	30	3.70455275216115e-14	0.607650418092865	0.555	0.401	1.24243290201981e-09	UST
Rejected	30	4.26056191182017e-14	0.316413922175178	0.211	0.095	1.42890725398625e-09	AL355916.2

Rejected	30	4.48175162219488e-14	0.361828807477896	0.863	0.757	1.50308985905172e-09	FTX
Rejected	30	4.68782232056234e-14	0.431295282503945	0.588	0.429	1.5722018498702e-09	RALGAPA2
Rejected	30	5.0451400534421e-14	0.378260811850858	0.607	0.452	1.69203907112341e-09	DCP1A
Rejected	30	5.23542080782635e-14	0.482698284996879	0.644	0.484	1.7558554305288e-09	HIVEP1
Rejected	30	5.84006782120856e-14	0.348318159772103	0.87	0.741	1.95864194587693e-09	DOCK2
Rejected	30	6.41612903141017e-14	0.353394588280045	0.646	0.488	2.15184135455434e-09	PRRC2B
Rejected	30	8.07780660057961e-14	0.334753492894198	0.983	0.953	2.70913477770239e-09	CDC42SE2
Rejected	30	8.171028794584e-14	0.401084690762105	0.847	0.729	2.74039963712758e-09	CDKAL1
Rejected	30	8.7693728380414e-14	0.36196237003463	0.665	0.495	2.94107226242232e-09	OSBPL9
Rejected	30	9.746897017653e-14	0.28411012903659	0.203	0.089	3.26891432178046e-09	GRB10
Rejected	30	1.18009220936661e-13	0.41158991280868	0.845	0.726	3.95779325177374e-09	TBC1D5
Rejected	30	1.24009218623888e-13	0.44044030446177	0.491	0.324	4.15902117420795e-09	EPAS1
Rejected	30	1.47953633975925e-13	0.329245545678573	0.536	0.364	4.96206897628458e-09	HIPK2
Rejected	30	1.71851741724677e-13	0.394062278413164	0.685	0.543	5.7635637139622e-09	NUP153
Rejected	30	1.73462517310193e-13	0.334577949574628	0.861	0.719	5.81758590554925e-09	ZNF644
Rejected	30	1.76709578697544e-13	0.511019020055643	0.631	0.484	5.92648585035824e-09	SIK2
Rejected	30	1.84398381306227e-13	0.511304326733617	0.72	0.576	6.18435291224823e-09	TBL1X
Rejected	30	1.94924217620759e-13	0.369393164491866	0.735	0.586	6.53736841056503e-09	CDK13
Rejected	30	2.11306521550443e-13	0.373838409498477	0.269	0.141	7.08679811975875e-09	AC010260.1
Rejected	30	2.49230137371968e-13	0.466658748687196	0.474	0.319	8.35868034718105e-09	PKP4
Rejected	30	2.74331169007916e-13	0.393287232255935	0.76	0.632	9.2005187461875e-09	RPS6KA5
Rejected	30	2.93356566874386e-13	0.283719597754604	0.203	0.093	9.83859253983315e-09	GLIS3
Rejected	30	3.00092631654393e-13	0.402823944909883	0.406	0.256	1.0064506680425e-08	AC109587.1
Rejected	30	3.08336636712216e-13	0.299643188813908	0.168	0.067	1.03409941220543e-08	CLMP
Rejected	30	3.22422038998986e-13	0.464286055149167	0.395	0.247	1.0813390343948e-08	AC092652.2
Rejected	30	3.42505332669338e-13	0.592295974593822	0.344	0.205	1.14869438470643e-08	SAMD4A
Rejected	30	3.45026593374159e-13	0.357008823292864	0.766	0.634	1.15715018885825e-08	CREBBP
Rejected	30	4.10585358607664e-13	0.256360772421667	0.994	0.969	1.37702117569838e-08	ARHGAP15
Rejected	30	4.22837391231909e-13	0.421083149823663	0.288	0.155	1.41811204271358e-08	AC009299.3
Rejected	30	4.56155537877024e-13	0.395678432666805	0.404	0.256	1.52985444293196e-08	SKAP1-AS1
Rejected	30	4.70173564734944e-13	0.384676690255699	0.785	0.646	1.57686810140806e-08	CRY1

Rejected	30	4.88533533274537e-13	0.356747490950705	0.957	0.891	1.63844376389614e-08	EML4
Rejected	30	5.12145953427746e-13	0.457751425999435	0.472	0.333	1.71763509860598e-08	SLC9A8
Rejected	30	7.48921374590748e-13	0.344325194188112	0.762	0.631	2.51173250610245e-08	MARCH6
Rejected	30	7.81393543913998e-13	0.268803041189198	0.975	0.913	2.62063766757877e-08	JMJD1C
Rejected	30	9.47056835426879e-13	0.263150165981579	0.319	0.178	3.17623921465467e-08	NFIB
Rejected	30	9.51388534402982e-13	0.333158586573665	0.424	0.281	3.19076686668072e-08	ATG13
Rejected	30	9.53017453047894e-13	0.392678967060679	0.19	0.085	3.19622993403203e-08	BNC2
Rejected	30	1.10142819510518e-12	0.262383588453164	0.13	0.046	3.69396988074375e-08	MAPK10
Rejected	30	1.17151609092725e-12	0.461879413699789	0.758	0.64	3.92903066575182e-08	EXT1
Rejected	30	1.22421628698297e-12	0.502399289679176	0.799	0.652	4.10577658328347e-08	FAM129A
Rejected	30	1.43169190611311e-12	0.336587861262025	0.478	0.335	4.80160831472215e-08	DIDO1
Rejected	30	1.54039479749517e-12	0.473963306528544	0.696	0.538	5.16617607183929e-08	QKI
Rejected	30	1.54476646283593e-12	0.31461928937682	0.487	0.324	5.18083776305913e-08	LMNA
Rejected	30	1.75637507894064e-12	0.347682316719322	0.586	0.438	5.8905307397511e-08	AC010894.3
Rejected	30	1.9556506848781e-12	0.419191478476515	0.468	0.322	6.55886126694418e-08	ERAP1
Rejected	30	1.99500136075157e-12	0.359888327059938	0.909	0.809	6.69083556368862e-08	CDK17
Rejected	30	2.11525792283461e-12	0.531393170978963	0.874	0.771	7.09415202160273e-08	PPP3CA
Rejected	30	2.59761135233652e-12	0.445373707104091	0.716	0.596	8.71186895346622e-08	PBX4
Rejected	30	2.60220061931738e-12	0.368549333197519	0.702	0.584	8.72726043706664e-08	TBC1D22A
Rejected	30	2.62884092373822e-12	0.39129229230358	0.665	0.53	8.81660669003325e-08	RCOR1
Rejected	30	2.79772306364593e-12	0.351909688659259	0.671	0.538	9.38300361085571e-08	SCAF8
Rejected	30	2.94648505255313e-12	0.367151261684696	0.836	0.733	9.8819215692527e-08	ADK
Rejected	30	3.22181923708041e-12	0.511498781195922	0.569	0.426	1.08053373573203e-07	BTBD11
Rejected	30	3.49987814325675e-12	0.38453740844205	0.679	0.527	1.17378913168545e-07	XPR1
Rejected	30	3.69402691321807e-12	0.265400498888086	0.143	0.055	1.23890274615508e-07	GPC6
Rejected	30	3.72895328259607e-12	0.347397120158854	0.888	0.791	1.25061635191707e-07	ZFAND3
Rejected	30	3.96443998683189e-12	0.291295492132054	0.967	0.88	1.32959388278368e-07	TNRC6B
Rejected	30	4.29250858196349e-12	0.325977995978355	0.762	0.615	1.43962152821892e-07	ARFGEF1
Rejected	30	4.97182069562719e-12	0.323762133881289	0.364	0.228	1.66744922489945e-07	RPRD1B
Rejected	30	5.35371772596001e-12	0.388435128485389	0.28	0.159	1.79552985093247e-07	SAP30L-AS1
Rejected	30	5.79472180160505e-12	0.310140852772045	0.94	0.862	1.9434337978223e-07	JAK1

Rejected	30	6.14938400289536e-12	0.375617905629386	0.896	0.785	2.06238040689105e-07	NFKB1
Rejected	30	6.41971753189876e-12	0.390024749644481	0.646	0.494	2.15304486584821e-07	SAMHD1
Rejected	30	6.47034421175554e-12	0.482064562220845	0.592	0.458	2.17002404173857e-07	DOCK9
Rejected	30	6.5206449394684e-12	0.401899986785938	0.663	0.527	2.18689389979891e-07	PATJ
Rejected	30	7.22362371226321e-12	0.329049325000601	0.915	0.834	2.42265892061883e-07	FOXN3
Rejected	30	7.29837480864017e-12	0.427638068307969	0.524	0.377	2.44772894332174e-07	ZC3H12D
Rejected	30	7.73003783799685e-12	0.41308429789073	0.663	0.519	2.59250009010738e-07	FARS2
Rejected	30	7.73146750574959e-12	0.296809134387827	0.89	0.783	2.5929795720783e-07	USP34
Rejected	30	7.74335470116814e-12	0.416187227727887	0.849	0.744	2.59696629967777e-07	MAML2
Rejected	30	8.63569479149707e-12	0.442341525902175	0.522	0.384	2.89623931917229e-07	ELK3
Rejected	30	9.24104500752367e-12	0.561788102687224	0.286	0.162	3.09926167462329e-07	AC104078.2
Rejected	30	1.0680763752342e-11	0.424623647623536	0.621	0.489	3.58211454726044e-07	SMAD3
Rejected	30	1.12906042350301e-11	0.407832489135778	0.669	0.522	3.7866428483444e-07	RFTN1
Rejected	30	1.2364368996077e-11	0.828027647600047	0.489	0.344	4.1467620739043e-07	FILIP1L
Rejected	30	1.40319045827726e-11	0.25352522137443	0.983	0.93	4.70602015897028e-07	RABGAP1L
Rejected	30	1.417552517549e-11	0.351958389115045	0.615	0.47	4.75418763335585e-07	EFR3A
Rejected	30	1.52520937880222e-11	0.299684730185798	0.304	0.174	5.1152472146269e-07	APP
Rejected	30	1.5334310399016e-11	0.462842019246151	0.665	0.536	5.14282102162199e-07	SYTL2
Rejected	30	1.53406193330012e-11	0.378163461997668	0.48	0.333	5.14493691190195e-07	HIF1A-AS2
Rejected	30	1.63565359877905e-11	0.472450394134724	0.458	0.321	5.48565503958519e-07	ULK4
Rejected	30	1.65578600317828e-11	0.322134087635952	0.741	0.605	5.5531750974593e-07	DYM
Rejected	30	1.77401803232705e-11	0.379642096462953	0.23	0.117	5.94970167681846e-07	AP002518.2
Rejected	30	2.21458592908623e-11	0.25156744726821	0.942	0.867	7.4272782889694e-07	LRBA
Rejected	30	2.23751476596998e-11	0.326597131956896	0.598	0.458	7.50417702211012e-07	PTBP2
Rejected	30	2.41766782941519e-11	0.344081133134221	0.381	0.247	8.10837436629268e-07	AC009554.1
Rejected	30	2.74565499870751e-11	0.362682575486051	0.501	0.362	9.20837773466523e-07	SNX29
Rejected	30	2.90236453478938e-11	0.351818977087815	0.706	0.565	9.73395017677664e-07	RNGTT
Rejected	30	3.34483167914101e-11	0.3051875666867	0.872	0.732	1.12178964855031e-06	CCND3
Rejected	30	3.49618152819246e-11	0.300218880242118	0.884	0.806	1.17254936092519e-06	CHD2
Rejected	30	3.74374191654592e-11	0.334894917631388	0.818	0.717	1.25557616397117e-06	PHF20
Rejected	30	4.30235717256102e-11	0.31041713646544	0.725	0.59	1.44292454853351e-06	MPZL3

Rejected	30	4.43053714329727e-11	0.31982212817356[illegible]	0.884	0.785	1.48591354711904e-06	NCOA2
Rejected	30	4.47515186863178e-11	0.40602473397694[illegible]	0.677	0.546	1.50087643370173e-06	MAP4
Rejected	30	4.89630549538811e-11	0.40012798251955[illegible]	0.658	0.53	1.64212293704327e-06	MBNL2
Rejected	30	4.91882112425226e-11	0.40480273869389[illegible]	0.41	0.271	1.64967422865172e-06	PLPP1
Rejected	30	5.94187167135242e-11	0.31603432534574	0.273	0.155	1.99278492113817e-06	KAZN
Rejected	30	6.44721888560997e-11	0.28567310645970[illegible]	0.161	0.071	2.16226826985587e-06	ADGRL3
Rejected	30	7.37508058391342e-11	0.46173506169644[illegible]	0.689	0.566	2.47345452623288e-06	LINC01004
Rejected	30	7.51407187456567e-11	0.30600264129156[illegible]	0.952	0.899	2.52006942529184e-06	KMT2E
Rejected	30	7.75964867378118e-11	0.33761173824372[illegible]	0.778	0.692	2.60243097221273e-06	SUCLG2
Rejected	30	9.33780850135373e-11	0.42707506743577[illegible]	0.429	0.3	3.13171421518401e-06	RPGR
Rejected	30	9.90873379491142e-11	0.32151593051253[illegible]	0.671	0.533	3.32319114013739e-06	BRD4
Rejected	30	1.04666788134577e-10	0.27923108155258	0.178	0.083	3.51031474045746e-06	TPST1
Rejected	30	1.17856149526879e-10	0.33863760907620[illegible]	0.783	0.678	3.95265954283248e-06	HIPK1
Rejected	30	1.20858462214764e-10	0.32391490087758[illegible]	0.615	0.469	4.05335110575875e-06	SLC25A26
Rejected	30	1.22007317667027e-10	0.30768006738800[illegible]	0.613	0.47	4.09188141991676e-06	YLPM1
Rejected	30	1.32007558868779e-10	0.31933742737550[illegible]	0.925	0.852	4.42726950934112e-06	NAP1L1
Rejected	30	1.32421042173268e-10	0.30298700570434[illegible]	0.696	0.59	4.44113691240706e-06	TM9SF3
Rejected	30	1.44797627315212e-10	0.31397175927904	0.234	0.126	4.85622282489757e-06	AC108449.1
Rejected	30	1.48134224111033e-10	0.32252401012251	0.673	0.556	4.96812560823581e-06	TBC1D15
Rejected	30	1.50786128708885e-10	0.30194607802106[illegible]	0.406	0.275	5.05706518463859e-06	TTC7A
Rejected	30	1.51771376110467e-10	0.28646327345688[illegible]	0.19	0.095	5.09010841199283e-06	SP2-AS1
Rejected	30	1.76569474761752e-10	0.44874221504364[illegible]	0.644	0.531	5.92178704455964e-06	MAPK8
Rejected	30	1.80092383848029e-10	0.31423447703536[illegible]	0.7	0.564	6.03993836949521e-06	SMG6
Rejected	30	1.80233806212556e-10	0.31235253576786[illegible]	0.845	0.727	6.04468139275669e-06	RIPOR2
Rejected	30	1.90830434450875e-10	0.40395633775048[illegible]	0.886	0.822	6.40007111061345e-06	FAM107B
Rejected	30	2.25957503766114e-10	0.32464540557764[illegible]	0.702	0.544	7.57816276130792e-06	DIAPH2
Rejected	30	2.27723183650398e-10	0.33961921883038[illegible]	0.828	0.711	7.63738013326705e-06	ZEB1
Rejected	30	2.44325674447706e-10	0.38801017380200[illegible]	0.646	0.535	8.19419446962718e-06	ARHGEF7
Rejected	30	2.80969733092934e-10	0.33636038992639[illegible]	0.89	0.827	9.42316290847082e-06	ADGRE5
Rejected	30	2.89106649353868e-10	0.38972066001298[illegible]	0.754	0.636	9.69605880603001e-06	ZNF609
Rejected	30	3.30567309338143e-10	0.37022453742278[illegible]	0.814	0.718	1.10865664205826e-05	ELOVL5

Rejected	30	3.46268685999359e-10	0.310610871783696	0.445	0.322	1.16131591910465e-05	EP400
Rejected	30	3.54983216114399e-10	0.361090742107394	0.776	0.687	1.19054271020447e-05	PPP1R16B
Rejected	30	3.57929784201791e-10	0.329791233188942	0.52	0.388	1.20042491025597e-05	IP6K1
Rejected	30	3.72302140891542e-10	0.369327868046526	0.253	0.147	1.24862692012205e-05	AC026470.2
Rejected	30	3.78865356220764e-10	0.270608547076713	0.629	0.477	1.2706386316932e-05	APOA1
Rejected	30	3.91865298444945e-10	0.328699488168327	0.714	0.597	1.31423783792466e-05	CMIP
Rejected	30	4.05995472541665e-10	0.259893956433382	0.182	0.088	1.36162761581024e-05	AC093495.1
Rejected	30	4.6079718582702e-10	0.342913225241854	0.371	0.248	1.54542160182666e-05	NUS1
Rejected	30	4.80154447011137e-10	0.334090957442606	0.737	0.615	1.61034198438595e-05	ZNF831
Rejected	30	5.45171745154048e-10	0.306202416482603	0.896	0.778	1.82839699889765e-05	LPP
Rejected	30	5.4820453068654e-10	0.297459127382333	0.826	0.726	1.83856835501652e-05	PIAS1
Rejected	30	6.22561170105824e-10	0.323186931694062	0.685	0.574	2.08794565230091e-05	WDR33
Rejected	30	6.63111920864072e-10	0.290091589813129	0.812	0.691	2.22394476019393e-05	UBE2H
Rejected	30	6.76679479791009e-10	0.380103427577769	0.507	0.381	2.26944763932308e-05	AL157402.2
Rejected	30	7.47126020310344e-10	0.281416217402522	0.909	0.799	2.50571124691683e-05	ARAP2
Rejected	30	7.78854869414119e-10	0.312434193220007	0.545	0.424	2.61212346104107e-05	USP10
Rejected	30	8.43905801536639e-10	0.397927448097412	0.369	0.242	2.83029127719358e-05	LINC02320
Rejected	30	8.44122657449942e-10	0.401082956848065	0.557	0.436	2.83101856855561e-05	CA5B
Rejected	30	9.27911923819713e-10	0.2920821363441	0.605	0.48	3.11203101010655e-05	ELF2
Rejected	30	9.93085603969068e-10	0.431784436157682	0.789	0.69	3.33061049859146e-05	BICDL1
Rejected	30	1.01120127752513e-09	0.283778313661322	0.642	0.529	3.39136684456378e-05	ATP9B
Rejected	30	1.03435315100412e-09	0.391538067313223	0.758	0.638	3.46901359783762e-05	USP3
Rejected	30	1.05628646935858e-09	0.277033590485094	0.826	0.708	3.54257356093479e-05	PICALM
Rejected	30	1.09580030736222e-09	0.26797930645731	0.944	0.892	3.6750950708314e-05	PIP4K2A
Rejected	30	1.12148282827511e-09	0.366216896052061	0.609	0.493	3.76122910946907e-05	CUX1
Rejected	30	1.13211475810244e-09	0.290147323971054	0.83	0.695	3.79688647572395e-05	CASK
Rejected	30	1.14831803536761e-09	0.307676137235445	0.516	0.387	3.85122902701589e-05	WIPF2
Rejected	30	1.16311795439593e-09	0.284993957150422	0.534	0.388	3.90086499545309e-05	SBF2
Rejected	30	1.24043027173665e-09	0.300171129156005	0.71	0.601	4.16015504535039e-05	LUC7L3
Rejected	30	1.30809313803594e-09	0.340486343315573	0.735	0.625	4.38708276634495e-05	BABAM2
Rejected	30	1.40886089712871e-09	0.444706900525046	0.814	0.72	4.72503767679028e-05	CDC14A

Rejected	30	1.47378942198343e-09	0.294055285672153	0.83	0.729	4.94279496344801e-05	PRKCB
Rejected	30	1.67278267249318e-09	0.321220171945342	0.34	0.226	5.61017852700763e-05	KIAA0556
Rejected	30	1.68680071508434e-09	0.360146742302361	0.453	0.329	5.65719223824987e-05	INTS6L
Rejected	30	1.74593835500386e-09	0.36747700019439	0.298	0.184	5.85552805501193e-05	ZBTB20-AS5
Rejected	30	1.75719792319207e-09	0.270467947241674	0.818	0.704	5.89329039480157e-05	STAG2
Rejected	30	2.16449270077027e-09	0.257987640414099	0.818	0.692	7.25927561984334e-05	MKLN1
Rejected	30	2.3109320200292e-09	0.272640359307941	0.592	0.478	7.75040380877393e-05	AP1G1
Rejected	30	2.3160075309828e-09	0.292712616646605	0.6	0.482	7.76742605741011e-05	ARID1A
Rejected	30	2.35045347667272e-09	0.288204407239299	0.458	0.342	7.88295087006496e-05	TNIP1
Rejected	30	2.36834046849806e-09	0.349732916865167	0.609	0.474	7.94294026324879e-05	GPHN
Rejected	30	2.50702191868123e-09	0.458687109731358	0.418	0.303	8.4080501108731e-05	DAAM1
Rejected	30	2.75836482143976e-09	0.325355886161113	0.725	0.623	9.25100393814467e-05	HUWE1
Rejected	30	2.86770250532096e-09	0.405528264109532	0.484	0.352	9.61770066234545e-05	SUMF1
Rejected	30	2.91081031997202e-09	0.276541221689044	0.547	0.425	9.76227565112217e-05	KBTBD2
Rejected	30	3.04831525199571e-09	0.253553728966752	0.157	0.073	0.000102234396921432	SDK1
Rejected	30	3.29744717102626e-09	0.524937663331087	0.362	0.251	0.000110589783221879	ZDHHC14
Rejected	30	3.63065724042137e-09	0.254994799258479	0.584	0.452	0.000121764982529252	IGF2R
Rejected	30	3.64358429343697e-09	0.404010428807366	0.648	0.535	0.000122198530033289	NCALD
Rejected	30	4.11743310789085e-09	0.294477617645572	0.54	0.424	0.000138090471572443	SREK1
Rejected	30	4.24613076955099e-09	0.255958124774462	0.232	0.131	0.000142406733749201	MAK
Rejected	30	4.38233784168342e-09	0.259740452565498	0.81	0.693	0.000146974846534378	NUP98
Rejected	30	4.49018304674375e-09	0.282684519370486	0.683	0.554	0.000150591759021692	RYBP
Rejected	30	4.77573374407453e-09	0.340854298449563	0.764	0.654	0.000160168558308772	RAPGEF1
Rejected	30	4.91030090555141e-09	0.300274139581885	0.76	0.674	0.000164681671770383	SETD2
Rejected	30	5.15754585910242e-09	0.319119448125974	0.613	0.481	0.000172973773022577	RELL1
Rejected	30	5.20119429368954e-09	0.387360766833936	0.801	0.721	0.00017443765422176	GPRIN3
Rejected	30	5.7298503340963e-09	0.323281960776255	0.538	0.42	0.000192167720504922	SRPK1
Rejected	30	5.96002396396644e-09	0.270345661629614	0.625	0.5	0.000199887283703507	NBAS
Rejected	30	6.55793742823472e-09	0.302126172981728	0.729	0.64	0.000219940105468136	SENP6
Rejected	30	6.56816495939862e-09	0.323528261540814	0.282	0.172	0.000220283116408311	RFX2
Rejected	30	6.91718958337882e-09	0.276563513983072	0.623	0.509	0.000231988704247359	PBXIP1

Rejected	30	7.08344814371265e-09	0.32088140377877[illegible]	0.843	0.748	0.000237564683843835	BACH2
Rejected	30	7.47592525775368e-09	0.31191697296807[illegible]	0.383	0.268	0.000250727581294543	ARMH4
Rejected	30	7.68341685429349e-09	0.305611345833407	0.663	0.561	0.000257686434459295	PBRM1
Rejected	30	8.52328037651412e-09	0.32476052758492[illegible]	0.652	0.544	0.000285853777267531	RB1CC1
Rejected	30	9.01119033888908e-09	0.28044086095843[illegible]	0.764	0.665	0.000302217301585662	USP47
Rejected	30	9.6120263664227e-09	0.30740688886599	0.797	0.708	0.000322368140277085	GOLGB1
Rejected	30	9.6431403946052e-09	0.29073323766168[illegible]	0.195	0.104	0.000323411642554269	AC025034.1
Rejected	30	9.78845451425711e-09	0.27599336794084[illegible]	0.934	0.869	0.000328285187499155	ANKRD12
Rejected	30	9.88528328275548e-09	0.34664254758905[illegible]	0.491	0.387	0.000331532630737053	WDR48
Rejected	30	9.9552060291726e-09	0.34962948478083[illegible]	0.354	0.243	0.000333877699806391	AC079793.1
Rejected	30	9.98876829401972e-09	0.28677105638626[illegible]	0.621	0.515	0.000335003311044833	BDP1
Rejected	30	1.18022191024652e-08	0.31469092555466[illegible]	0.518	0.403	0.000395822824258477	KPNA1
Rejected	30	1.18867114399527e-08	0.28653805712342[illegible]	0.625	0.52	0.000398656528273133	TTC19
Rejected	30	1.27327939069408e-08	0.30686196184597[illegible]	0.586	0.465	0.000427032442050981	CHD6
Rejected	30	1.28219835092028e-08	0.25555955181734[illegible]	0.716	0.588	0.000430023682931644	HBP1
Rejected	30	1.3408677409747e-08	0.40171534490091	0.669	0.557	0.000449700222968094	IMMP2L
Rejected	30	1.37429068833195e-08	0.31653737801903	0.265	0.164	0.000460909611052768	CFLAR-AS1
Rejected	30	1.4007043493504e-08	0.35708524467738[illegible]	0.677	0.57	0.000469768224685137	N4BP1
Rejected	30	1.43172553093169e-08	0.30905033948866[illegible]	0.752	0.64	0.000480172108563872	TTC39C
Rejected	30	1.46022290585882e-08	0.29817614689650[illegible]	0.509	0.395	0.000489729558166929	TP53BP2
Rejected	30	1.55926790038943e-08	0.26381044034215[illegible]	0.698	0.589	0.000522947268432607	YWHAE
Rejected	30	1.61337171521546e-08	0.25826915281446[illegible]	0.841	0.738	0.00054109260584896	ASH1L
Rejected	30	1.77613283781325e-08	0.27881503132337[illegible]	0.739	0.628	0.000595679431145807	TRAF3
Rejected	30	1.78462178876091e-08	0.25357989895799[illegible]	0.839	0.75	0.000598526455514633	FAM172A
Rejected	30	1.79275126088466e-08	0.25609772734541[illegible]	0.675	0.574	0.000601252917875496	RBM33
Rejected	30	1.88980525467148e-08	0.28276403798172[illegible]	0.522	0.409	0.000633802886311722	ARNTL
Rejected	30	1.92385560912317e-08	0.32474064199760[illegible]	0.333	0.217	0.00064522269418773	WHRN
Rejected	30	1.95888187587521e-08	0.27283825603638[illegible]	0.749	0.65	0.000656969803531028	DIAPH1
Rejected	30	2.02847553274922e-08	0.26039483546923[illegible]	0.321	0.216	0.000680310124173433	SART3
Rejected	30	2.03227925287989e-08	0.32156416422431[illegible]	0.617	0.482	0.000681585815830856	PIK3R5
Rejected	30	2.11127620580698e-08	0.43953081368182[illegible]	0.226	0.128	0.000708079813903545	PHLDB2

Rejected	30	2.14637113358221e-08	0.270925459616989	0.729	0.616	0.000719849950780803	ARID2
Rejected	30	2.27356717983625e-08	0.256112857089463	0.203	0.113	0.000762508960773481	PDE9A
Rejected	30	2.2838106264132e-08	0.308627787808482	0.246	0.147	0.00076594440788646	AC010275.1
Rejected	30	2.31444901398069e-08	0.519380919289442	0.615	0.521	0.000776219910308843	RAD51B
Rejected	30	2.34135472557282e-08	0.28697317952551	0.658	0.542	0.000785243547862611	GBF1
Rejected	30	2.74012987828686e-08	0.328789227682582	0.317	0.207	0.000918984758579846	PELI2
Rejected	30	2.8033481718654e-08	0.259577409821951	0.176	0.093	0.000940186909880218	AC024145.1
Rejected	30	2.88089190046516e-08	0.253088145278037	0.298	0.189	0.000966193525578004	PLEKHG1
Rejected	30	2.93657727556467e-08	0.299752032350564	0.42	0.31	0.000984869286678879	KAT7
Rejected	30	2.94594269649812e-08	0.283589482064061	0.379	0.273	0.000988010261551539	RRP12
Rejected	30	2.98703468812215e-08	0.32349132921928	0.563	0.456	0.00100179169370241	NAA50
Rejected	30	3.10961700851576e-08	0.453618020532082	0.427	0.313	0.00104290335231602	ADAMTS17
Rejected	30	3.19851925438586e-08	0.303680707326171	0.716	0.628	0.00107271938753593	TRAPPC9
Rejected	30	3.52253368189768e-08	0.325405799897204	0.497	0.38	0.00118138734623484	AC104365.1
Rejected	30	3.77880350383262e-08	0.305026980309167	0.383	0.271	0.00126733511911539	CCDC173
Rejected	30	4.10492008062899e-08	0.338123612855365	0.458	0.34	0.00137670809664135	ABHD15-AS1
Rejected	30	4.18353094580133e-08	0.251644540010373	0.654	0.557	0.00140307260860285	EHMT1
Rejected	30	4.34398475323333e-08	0.266740065727609	0.814	0.703	0.0014568856065394	PAFAH1B1
Rejected	30	4.45502191209384e-08	0.253299753570537	0.445	0.33	0.00149412524887803	RBM23
Rejected	30	4.46433501063847e-08	0.293934310161303	0.273	0.172	0.00149724867586793	AC005920.2
Rejected	30	4.58989818415602e-08	0.290674551546499	0.472	0.361	0.00153936005300225	EXOC6
Rejected	30	4.75052510179924e-08	0.335187107190152	0.685	0.594	0.00159323110864143	ATP2B4
Rejected	30	5.05895424567336e-08	0.283232638616813	0.706	0.593	0.00169667207491393	EPC2
Rejected	30	5.149723968046e-08	0.273570802234663	0.621	0.526	0.00172711442440327	ADNP
Rejected	30	5.3028966074335e-08	0.275569478674996	0.611	0.497	0.00177848546420105	PPIL4
Rejected	30	5.42794549933816e-08	0.252521283706686	0.613	0.491	0.00182042436156803	SCFD1
Rejected	30	5.56608393052243e-08	0.35428222957444	0.23	0.137	0.00186675322861861	PBX1
Rejected	30	6.18376248129343e-08	0.375343622977722	0.402	0.286	0.00207391026097619	FER
Rejected	30	6.92167704885581e-08	0.254124716315983	0.932	0.838	0.00232139204864526	PPP1CB
Rejected	30	7.62696266748584e-08	0.317516867727686	0.393	0.288	0.0025579307394214	AC073167.1
Rejected	30	7.68121214527857e-08	0.254737377272762	0.803	0.718	0.00257612492928353	GPCPD1

Rejected	30	7.77862396996624e-08	0.56415521969080	0.48	0.38	0.00260879490704728	AGPAT4
Rejected	30	8.1462142093847e-08	0.29345224359682	0.288	0.183	0.00273207732154344	MCC
Rejected	30	8.15172718925284e-08	0.37088712061261	0.468	0.363	0.00273392626473162	ECPAS
Rejected	30	8.70915335306855e-08	0.25311332880138	0.712	0.617	0.00292087585155213	STK10
Rejected	30	8.8912216253839e-08	0.25556405016094	0.876	0.798	0.00298193790872125	NIPBL
Rejected	30	9.2208791274108e-08	0.28136676991875	0.648	0.543	0.00309249844175103	MECP2
Rejected	30	9.22434802674759e-08	0.37842251246970	0.516	0.403	0.00309366184121061	SEPT11
Rejected	30	9.98468437655137e-08	0.33114785490481	0.863	0.785	0.0033486634462078	HERC1
Rejected	30	1.10517034866059e-07	0.31161143900478	0.613	0.511	0.00370652031533788	SGMS1
Rejected	30	1.11883005367735e-07	0.26587007606559	0.594	0.473	0.00375233223402309	RUNX2
Rejected	30	1.19260218057239e-07	0.31726499588895	0.445	0.319	0.00399974919320369	PLCL1
Rejected	30	1.23480401292893e-07	0.27840623126899	0.547	0.435	0.00414128569856105	SORL1
Rejected	30	1.31778389423572e-07	0.29323494320325	0.687	0.59	0.00441958362448776	MARK3
Rejected	30	1.37365904025975e-07	0.42833166184128	0.348	0.245	0.00460697768922315	NFIA
Rejected	30	1.44269631301333e-07	0.28126150140841	0.166	0.088	0.00483851489458409	RHD
Rejected	30	1.48501235613497e-07	0.32123172455135	0.311	0.211	0.00498043444000545	IFIH1
Rejected	30	1.48604265601057e-07	0.33884155360978	0.261	0.169	0.00498388985972825	AC093019.2
Rejected	30	1.56285416282619e-07	0.28641663797362	0.505	0.4	0.00524150029128648	TP53BP1
Rejected	30	1.59056804429805e-07	0.29335797448355	0.23	0.144	0.0053344471069668	FAM217A
Rejected	30	1.64518604738975e-07	0.31484054049606	0.874	0.796	0.00551762496573576	RALGAPA1
Rejected	30	1.70439235097838e-07	0.33605333948060	0.48	0.369	0.00571619106671129	SIDT1
Rejected	30	1.73296312560811e-07	0.29898846159495	0.588	0.488	0.00581201173066447	SEPT2
Rejected	30	1.75965974523938e-07	0.32892886354173	0.547	0.426	0.00590154685358385	GRK5
Rejected	30	1.86896336199006e-07	0.39573383960639	0.453	0.34	0.00626812932344225	AC008014.1
Rejected	30	2.03789364509206e-07	0.25713007934876	0.236	0.148	0.00683468770690977	ELMO1-AS1
Rejected	30	2.10194955047642e-07	0.27007911149304	0.484	0.374	0.00704951840238781	SPAG1
Rejected	30	2.13279470189751e-07	0.30236571979414	0.71	0.595	0.00715296687122388	ASAP1
Rejected	30	2.27843569759545e-07	0.26914175246968	0.52	0.42	0.00764141764259561	PDXDC1
Rejected	30	2.35879300506096e-07	0.31097848466601	0.511	0.403	0.00791091998037345	ATG7
Rejected	30	2.53009693078706e-07	0.43704204552294	0.443	0.326	0.00848543908647365	PCAT1
Rejected	30	2.54835907921945e-07	0.25580268608045	0.389	0.287	0.0085466866798862	MTMR1

Rejected	30	2.64143469012929e-07	0.33717454582985[illegible]	0.398	0.296	0.0088588436637556	APOB
Rejected	30	2.65026146453261e-07	0.30389218518957[illegible]	0.449	0.341	0.00888844689974948	PDE8A
Rejected	30	2.85632234105726e-07	0.30442997686128[illegible]	0.269	0.172	0.00957953386743783	ELOVL6
Rejected	30	2.88162924963501e-07	0.32243234959822[illegible]	0.518	0.407	0.00966440817742591	SNX25
Rejected	30	2.90380369766778e-07	0.42326918801450[illegible]	0.458	0.356	0.00973877684123822	USP3-AS1
Rejected	30	2.95557106995283e-07	0.284648612065117	0.186	0.106	0.00991239425440781	MB21D2
Rejected	30	3.08953548667085e-07	0.26938192843681[illegible]	0.511	0.395	0.0103616841151967	HMBOX1
Rejected	30	3.13049674177161e-07	0.31094736374990[illegible]	0.17	0.091	0.0104990599725536	IL1R1
Rejected	30	3.13268202173396e-07	0.28718055145074[illegible]	0.805	0.71	0.0105063889644914	ARHGAP26
Rejected	30	3.18248835499641e-07	0.26563335112868[illegible]	0.712	0.6	0.010673429444987	ODF2L
Rejected	30	3.3709567506009e-07	0.27805238364039[illegible]	0.685	0.572	0.0113055147501653	TUT4
Rejected	30	3.53590702486578e-07	0.28639746720338[illegible]	0.638	0.538	0.0118587249799949	PCNX2
Rejected	30	3.65193883377354e-07	0.319736250443247	0.619	0.527	0.0122478724607097	AKNA
Rejected	30	3.76843208472226e-07	0.25636466232918[illegible]	0.735	0.662	0.0126385675257415	LPIN2
Rejected	30	4.1641933547466e-07	0.28321907872029[illegible]	0.458	0.359	0.0139658716731492	MT-ND6
Rejected	30	4.83765238689933e-07	0.256424380619311	0.172	0.098	0.016224518575183	AC025917.1
Rejected	30	5.28727887053299e-07	0.30675467235051[illegible]	0.586	0.473	0.0177324758759935	FTO
Rejected	30	5.53149687402576e-07	0.27372577027663[illegible]	0.526	0.419	0.0185515342161076	MTFR1
Rejected	30	5.80697880168651e-07	0.310276384244147	0.77	0.701	0.0194754455050962	SMYD3
Rejected	30	5.89972712450995e-07	0.279795597128271	0.424	0.32	0.0197865048301815	MAP4K3
Rejected	30	6.09000054211519e-07	0.30066864683139[illegible]	0.677	0.57	0.0204246438181459	ABLIM1
Rejected	30	6.1968890187135e-07	0.27696009736665[illegible]	0.472	0.361	0.0207831263909613	PCCA
Rejected	30	6.23381643182873e-07	0.32846867282726[illegible]	0.518	0.42	0.0209069735490672	SREBF2
Rejected	30	6.73756879816997e-07	0.27109173174963[illegible]	0.41	0.312	0.0225964582353024	POLR3E
Rejected	30	6.81423061333329e-07	0.31503825443178[illegible]	0.348	0.245	0.0228535666309972	FAR2
Rejected	30	6.81888443550083e-07	0.30551705078408[illegible]	0.288	0.194	0.0228691746197827	AL157938.2
Rejected	30	6.84098391567342e-07	0.32884959442390[illegible]	0.402	0.307	0.0229432918563855	AC124319.2
Rejected	30	7.13101007127726e-07	0.26963722148765[illegible]	0.576	0.482	0.0239159815770497	RAB21
Rejected	30	7.17509637979866e-07	0.28406245961072[illegible]	0.236	0.147	0.0240638382385687	TSIX
Rejected	30	7.49708308490136e-07	0.30638406646963[illegible]	0.356	0.267	0.0251437172501422	OSBP
Rejected	30	7.89898649949981e-07	0.35749176867901[illegible]	0.532	0.418	0.0264916209220225	RASGRF2

Rejected	30	8.03799606082693e-07	0.321827916965179	0.294	0.196	0.0269578311888013	SPON1
Rejected	30	8.32319163115345e-07	0.479951730916666	0.311	0.216	0.0279143200925624	PRKN
Rejected	30	8.73564417137034e-07	0.2993336164084	0.611	0.52	0.0292976034219418	ATAD2B
Rejected	30	9.57173634660941e-07	0.280557007440871	0.613	0.508	0.0321016893592586	MYO5A
Rejected	30	9.83688529783296e-07	0.260593765270803	0.472	0.37	0.0329909459118722	ARMH3
Rejected	30	1.1181889861397e-06	0.259270606333015	0.317	0.225	0.0375018222171534	GSE1
Rejected	30	1.17674527206967e-06	0.276781393332584	0.439	0.332	0.0394656829346724	FAM69A
Rejected	30	1.19188968462551e-06	0.255389577215837	0.772	0.656	0.0399735962429703	KAT6B
Rejected	30	1.4094547403777e-06	0.362459412673501	0.675	0.578	0.0472702930827873	PELI1
Rejected	30	1.47297607458491e-06	0.265830800966632	0.462	0.361	0.0494006715894286	TBC1D23
Rejected	34	3.23087078957332e-76	1.01727401113172	0.984	0.721	1.0835694454071e-71	CCND3
Rejected	34	3.74734166882618e-62	0.953969474291431	0.973	0.767	1.25678344889092e-57	IKZF1
Rejected	34	1.12025925051386e-58	0.763132564215582	1	0.908	3.75712547437337e-54	ANKRD44
Rejected	34	2.54209854124222e-52	0.914947005633148	0.989	0.823	8.52569008761815e-48	FKBP5
Rejected	34	1.24953837443776e-51	0.63692942764032	1	0.969	4.19070180018937e-47	ARHGAP15
Rejected	34	1.04395659879953e-49	0.390366937573333	0.609	0.199	3.50122164105388e-45	NRG1
Rejected	34	2.66461324648384e-45	0.870705862657133	0.962	0.868	8.9365799060575e-41	PDE3B
Rejected	34	8.71762807389812e-41	0.687981450975208	0.981	0.822	2.92371810342395e-36	RASA2
Rejected	34	2.71795648853307e-39	0.627749331750416	0.964	0.732	9.11548247124222e-35	DOCK2
Rejected	34	2.56096848948094e-38	0.65408570487914	0.981	0.803	8.58897612002118e-34	PCNX1
Rejected	34	4.63105318988364e-38	0.25372147600454	0.306	0.073	1.55316261882318e-33	PRR16
Rejected	34	9.57108057141157e-38	0.755751050151045	0.986	0.888	3.20994900204001e-33	PIP4K2A
Rejected	34	8.62501227664063e-37	0.312019065363754	0.279	0.064	2.89265661733974e-32	AC124014.1
Rejected	34	2.13970150413534e-35	0.669728656320463	0.921	0.674	7.17613090456909e-31	SUCLG2
Rejected	34	3.84015606724071e-35	0.6844456257524	0.959	0.774	1.28791154183119e-30	LPP
Rejected	34	3.96740566798679e-35	0.600855653407611	0.727	0.376	1.33058851292941e-30	HDAC4
Rejected	34	5.31672001271895e-35	0.678117674240878	0.954	0.789	1.78312155786568e-30	ELMO1
Rejected	34	2.4414377748095e-34	1.04958318346658	0.869	0.679	8.18809400915612e-30	ANK3
Rejected	34	2.71588639680189e-34	0.49585598438973	0.68	0.325	9.10853979759417e-30	USP3-AS1
Rejected	34	1.7778750966229e-33	0.560659192362087	0.497	0.199	5.96263749905389e-29	RNF157
Rejected	34	1.24977127113385e-32	0.725398487513782	0.88	0.608	4.19148288912871e-28	MAP3K5

Rejected	34	1.49053414076656e-32	0.566336587833366	0.656	0.314	4.99895340130288e-28	ABHD15-AS1
Rejected	34	1.9080040649486e-32	0.539350419274098	1	0.922	6.39906403302461e-28	CELF2
Rejected	34	9.67378299014688e-32	0.543703868416997	0.809	0.473	3.24439333923546e-27	ST3GAL1
Rejected	34	3.04306468062519e-30	0.581228603980685	0.634	0.316	1.02058303258808e-25	MVB12B
Rejected	34	7.05552359477323e-30	0.399922255022838	0.473	0.189	2.36628150321505e-25	AC096558.2
Rejected	34	8.32078032295379e-30	0.600805316214877	0.964	0.821	2.79062330471224e-25	CHST11
Rejected	34	9.71377710430672e-30	0.540988730299618	1	0.953	3.25780656524239e-25	PARP8
Rejected	34	1.74165961927691e-29	0.256954762212719	0.336	0.104	5.84117803113092e-25	COL3A1
Rejected	34	2.66492977949953e-29	0.546588027643571	0.839	0.529	8.93764149448552e-25	DOCK11
Rejected	34	2.99217678869344e-29	0.535258468034461	0.847	0.523	1.003516251392e-24	PREX1
Rejected	34	4.49122323881609e-29	0.593109916380232	0.986	0.858	1.50626644983414e-24	PRKCH
Rejected	34	4.56310664370969e-29	0.544926716749099	0.77	0.436	1.53037470616735e-24	DOCK9
Rejected	34	4.70477154773832e-29	0.652371576218001	0.888	0.643	1.57788628168048e-24	PLCL2
Rejected	34	5.11833804769468e-29	0.401233465980217	0.462	0.183	1.71658821443584e-24	HERC3
Rejected	34	5.1999509597464e-29	0.533377744360339	0.869	0.563	1.74395955287975e-24	INPP5D
Rejected	34	1.73214231240384e-28	0.558662628554076	0.825	0.518	5.80925888733999e-24	INPP4A
Rejected	34	2.21556276519806e-28	0.601389367950211	0.943	0.758	7.43055440192127e-24	EXOC4
Rejected	34	2.31538040801867e-28	0.715402937149493	0.697	0.364	7.76532281241301e-24	A2M
Rejected	34	2.53352117315201e-28	0.610592382578286	0.743	0.43	8.4969233105172e-24	PTK2B
Rejected	34	2.79474856441782e-28	0.626131909435066	0.954	0.831	9.37302773534447e-24	SSH2
Rejected	34	5.07826238769363e-28	0.675148026084731	0.833	0.57	1.70314763958469e-23	STIM1
Rejected	34	5.78256359321036e-28	0.642878609523833	0.762	0.451	1.93935617789089e-23	KAT2B
Rejected	34	6.1973447690644e-28	0.485752587618554	0.984	0.882	2.07846548864882e-23	TNRC6B
Rejected	34	6.67737565066419e-28	0.797266281027431	0.76	0.483	2.23945824571976e-23	RUNX1
Rejected	34	7.46344460403235e-28	0.516729725697449	0.549	0.249	2.50309005130037e-23	SLAMF6
Rejected	34	1.22653614412963e-27	0.487907815279341	0.784	0.462	4.11355692018196e-23	PIK3R5
Rejected	34	1.34449294408089e-27	0.83970109232046	0.899	0.682	4.50916043585848e-23	RBMS1
Rejected	34	2.34476726610724e-27	0.68132488328915	0.904	0.724	7.86388045707047e-23	RIPOR2
Rejected	34	5.25187507498795e-27	0.493745255441269	0.749	0.433	1.76137386264946e-22	USP24
Rejected	34	5.44851650176238e-27	0.617121759018628	0.874	0.6	1.82732346436107e-22	TC2N
Rejected	34	7.71993920986088e-27	0.639188232013097	0.921	0.716	2.58911321220314e-22	FOXO1

Rejected	34	8.40171316779986e-27	0.57413044523905	0.918	0.706	2.81776656221672e-22	COG5
Rejected	34	1.01407154686743e-26	0.489179427441289	0.986	0.877	3.400993153884e-22	UTRN
Rejected	34	1.54711142340437e-26	0.507019792735415	0.814	0.495	5.18870229181357e-22	FLI1
Rejected	34	1.22182197974096e-25	0.604688354130677	0.855	0.593	4.09774655565525e-21	DYM
Rejected	34	1.48602713396654e-25	0.639846870232537	0.891	0.692	4.98383780189697e-21	CASK
Rejected	34	4.08430397899003e-25	0.570560564825582	0.918	0.72	1.36979386847368e-20	TNFAIP8
Rejected	34	5.13089971652007e-25	0.450576390006845	0.852	0.534	1.7208011469265e-20	CCDC88C
Rejected	34	5.35385507882126e-25	0.437828882175101	0.443	0.188	1.79557591633507e-20	AL645568.1
Rejected	34	5.62729062713436e-25	0.679728526000563	0.904	0.753	1.88728073052832e-20	THEMIS
Rejected	34	6.47488934829841e-25	0.531136924561567	0.74	0.453	2.17154838963232e-20	FTO
Rejected	34	7.90147432611368e-25	0.473612237171325	0.546	0.26	2.64999645949201e-20	SNTB2
Rejected	34	8.74124499268525e-25	0.753279354535921	0.888	0.72	2.93163874564678e-20	INPP4B
Rejected	34	8.76410113293478e-25	0.550732797478551	0.762	0.47	2.93930423796367e-20	NFATC3
Rejected	34	1.04362164084367e-24	0.50619810342531	0.986	0.855	3.50009825906151e-20	SYNE2
Rejected	34	1.49303850115608e-24	0.49055380611105	0.962	0.776	5.00735252517727e-20	VPS13B
Rejected	34	1.72844319777257e-24	0.45214052536479	0.937	0.726	5.79685279668964e-20	AAK1
Rejected	34	2.37644514097963e-24	0.463104802715277	0.585	0.302	7.97012171381747e-20	ESR2
Rejected	34	3.75551842780187e-24	0.465355424150465	0.833	0.553	1.25952577031619e-19	SEMA4D
Rejected	34	3.91237896925323e-24	0.515552554504337	0.984	0.832	1.31213365870815e-19	IQGAP2
Rejected	34	3.94504513157116e-24	0.575103449011349	0.852	0.611	1.32308923622633e-19	BABAM2
Rejected	34	3.95596352097198e-24	0.449002220428807	0.639	0.34	1.32675104566358e-19	RREB1
Rejected	34	4.65674805646612e-24	0.394477779934432	0.505	0.236	1.56178016317761e-19	NUDCD3
Rejected	34	4.85993229606944e-24	0.498054904630588	0.978	0.828	1.62992409345577e-19	FOXN3
Rejected	34	5.38274865393886e-24	0.816237637969098	0.503	0.258	1.80526624355801e-19	RNF130
Rejected	34	6.2248638770421e-24	0.556900128697337	0.383	0.161	2.08769484708238e-19	USP9Y
Rejected	34	1.37267954347353e-23	0.397946954948121	0.445	0.195	4.60369265290151e-19	TG
Rejected	34	1.73030512831383e-23	0.542054589137559	0.94	0.738	5.80309733933894e-19	FAM172A
Rejected	34	4.82807582460793e-23	0.582543014783799	0.861	0.669	1.61924007005701e-18	VTI1A
Rejected	34	5.203009044857e-23	0.532199768672767	0.904	0.741	1.74498517346414e-18	RPS6KA3
Rejected	34	5.39103257108655e-23	0.511364609998851	0.765	0.463	1.80804450369101e-18	MAP4K4
Rejected	34	6.49033310910932e-23	0.490080106171905	0.798	0.512	2.17672791813308e-18	PATJ

Rejected	34	7.54020796659704e-23	0.35221585031755	0.555	0.27	2.52883494783731e-18	CRACR2A
Rejected	34	7.72308591902707e-23	0.53093052178132	0.88	0.651	2.5901685555233e-18	NCOA1
Rejected	34	1.06705253992237e-22	0.64148695440502	0.593	0.324	3.57868080839165e-18	ZBTB16
Rejected	34	1.31189324638704e-22	0.51378023327520	0.784	0.507	4.39982756973286e-18	AFF1
Rejected	34	1.48407128756781e-22	0.33879053473205	0.426	0.186	4.97727828424492e-18	TTC13
Rejected	34	1.52065130911889e-22	0.45374975920926	0.79	0.508	5.09996036052294e-18	VWA8
Rejected	34	1.55051592524971e-22	0.54638028624817	0.617	0.341	5.20012031010249e-18	ATF7IP2
Rejected	34	1.61368930284965e-22	0.40243205547053	0.716	0.422	5.41199118389717e-18	NIN
Rejected	34	1.63524129155934e-22	0.61323477618959	0.847	0.639	5.48427224363171e-18	ATP8A1
Rejected	34	1.72034493244378e-22	0.50783474760098	0.926	0.73	5.76969283442996e-18	CCSER2
Rejected	34	1.87987197882388e-22	0.40619439622876	0.719	0.411	6.30471464257953e-18	XPO6
Rejected	34	1.9267121333943e-22	0.54025144802888	0.899	0.752	6.46180715297779e-18	TCF12
Rejected	34	1.96769013203583e-22	0.56513834648458	0.915	0.72	6.59923916482177e-18	PRKCB
Rejected	34	2.14749926221754e-22	0.40459251308055	0.628	0.332	7.2022830256252e-18	TRRAP
Rejected	34	2.41126114610064e-22	0.60144233130022	0.893	0.696	8.08688763179232e-18	BTBD9
Rejected	34	2.52417563798926e-22	0.49846801594930	0.929	0.707	8.46558025468838e-18	UBAC2
Rejected	34	3.23473270816438e-22	0.60368947327117	0.732	0.516	1.08486465566417e-17	FARS2
Rejected	34	3.90078552922517e-22	0.61376404438483	0.511	0.271	1.30824545079154e-17	CPQ
Rejected	34	4.21690904982731e-22	0.47349473008378	0.964	0.861	1.41426695713108e-17	DOCK10
Rejected	34	5.68188870386294e-22	0.65313752038349	0.675	0.432	1.90559183350155e-17	SCFD2
Rejected	34	6.27012461137549e-22	0.48251000115628	0.85	0.61	2.10287439216311e-17	TRAPPC9
Rejected	34	7.70412766520279e-22	0.63120586284802	0.563	0.305	2.58381033635571e-17	TRERF1
Rejected	34	9.04749620285554e-22	0.47229630377932	0.97	0.859	3.03434927651369e-17	ARID1B
Rejected	34	1.13741929411202e-21	0.48791153858194	0.967	0.848	3.81467682859289e-17	KLF12
Rejected	34	1.40887146285339e-21	0.38211730750242	0.615	0.326	4.72507311211771e-17	NLRC5
Rejected	34	1.83304610826998e-21	0.27159821049197	0.467	0.214	6.14767003791587e-17	IQSEC1
Rejected	34	2.01673866729146e-21	0.40600449173364	0.623	0.336	6.76373814236211e-17	LONP2
Rejected	34	2.08137130629685e-21	0.31405612788954	0.568	0.285	6.98050308705839e-17	AC124319.2
Rejected	34	2.22332350933431e-21	0.65429657223208	0.833	0.644	7.45658238560541e-17	FBXL17
Rejected	34	2.4773679257245e-21	0.50353260707225	0.568	0.305	8.30859654929483e-17	RASA3
Rejected	34	2.74435568180933e-21	0.45161966396576	0.923	0.721	9.20402008565212e-17	EPS15

Rejected	34	2.79233638956789e-21	0.512520552654599	0.866	0.592	9.3649377833328e-17	NCK2
Rejected	34	2.91630655065999e-21	0.780753596207101	0.497	0.273	9.78070890960347e-17	UTY
Rejected	34	3.21028873080141e-21	0.601686054737226	0.943	0.841	1.07666663453618e-16	PPP1CB
Rejected	34	3.44648133328672e-21	0.479816978380819	0.932	0.789	1.1558809095577e-16	ZFAND3
Rejected	34	3.72563877375766e-21	0.541091636587654	0.874	0.642	1.24950473194284e-16	RAPGEF1
Rejected	34	4.72201684635016e-21	0.465240830611216	0.708	0.42	1.58367000992892e-16	UBR3
Rejected	34	4.88812726284125e-21	0.397207583029854	0.705	0.408	1.6393801214117e-16	TMEM181
Rejected	34	5.20242147855616e-21	0.504542878272012	0.863	0.627	1.74478811547817e-16	TTC39C
Rejected	34	5.57258783286571e-21	0.444054178688103	0.842	0.607	1.8689345073865e-16	KIAA1109
Rejected	34	6.3655766833244e-21	0.473518937619811	0.918	0.694	2.13488710805334e-16	COP1
Rejected	34	7.52338547055825e-21	0.439933159423223	0.984	0.864	2.52319301911582e-16	LRBA
Rejected	34	7.79817046766496e-21	0.507191372576398	0.885	0.661	2.61535041144548e-16	SMARCA2
Rejected	34	8.61696174679866e-21	0.384123522504608	0.541	0.28	2.88995663064134e-16	CLIP4
Rejected	34	9.12715176006591e-21	0.429744605546023	0.874	0.596	3.0610641572909e-16	STK10
Rejected	34	9.42180193269354e-21	0.429119080902648	0.656	0.373	3.15988393218676e-16	CRTC3
Rejected	34	1.00317514082128e-20	0.40301390063955	0.719	0.424	3.36444878728641e-16	TBC1D1
Rejected	34	1.14182140670662e-20	0.494788164602975	0.765	0.514	3.82944063381267e-16	MAN1A2
Rejected	34	1.25418383679592e-20	0.356566719826534	0.536	0.267	4.20628175184616e-16	FAM196B
Rejected	34	1.32052398453816e-20	0.473781369567337	0.691	0.417	4.42877333934406e-16	MAPKAP1
Rejected	34	1.72423798130116e-20	0.56161960244504	0.883	0.726	5.78274934168782e-16	TBC1D5
Rejected	34	1.90030959819641e-20	0.38730677841454	0.694	0.398	6.37325833043112e-16	RPTOR
Rejected	34	1.93497058475476e-20	0.45650889451964	0.5	0.255	6.4895043471505e-16	TTC39B
Rejected	34	1.94280066580047e-20	0.43332026997194	0.88	0.631	6.51576487296162e-16	NKTR
Rejected	34	2.06486641419985e-20	0.724494895127511	0.423	0.201	6.92514897994346e-16	PZP
Rejected	34	2.67768131905466e-20	0.577382785174305	0.768	0.531	8.98040760784553e-16	TSPAN5
Rejected	34	3.13377364954152e-20	0.303490213136335	0.664	0.368	1.05100500658323e-15	ANKRD36
Rejected	34	3.41974001292844e-20	0.466683423912145	0.929	0.752	1.14691240553594e-15	FTX
Rejected	34	3.72931591591499e-20	0.913059070458799	0.536	0.317	1.25073797187957e-15	PCAT1
Rejected	34	4.10067665781988e-20	0.561188349356286	0.664	0.397	1.37528493749963e-15	SPATA5
Rejected	34	4.36462280367186e-20	0.418049135942439	0.981	0.853	1.46380719589547e-15	DOCK8
Rejected	34	4.91255426260512e-20	0.364611271965664	0.954	0.744	1.64757244859251e-15	RNF213

Rejected	34	5.56043776718819e-20	0.475738671722774	0.822	0.588	1.86485961835957e-15	RNF38
Rejected	34	5.75436224383505e-20	0.450881786130235	0.888	0.668	1.9298980093374e-15	RASA1
Rejected	34	5.78002703601424e-20	0.518361644453309	0.762	0.532	1.93850546733846e-15	RAP1GDS1
Rejected	34	6.25544981159449e-20	0.387150689095391	0.699	0.421	2.09795275781256e-15	ARHGAP25
Rejected	34	6.45987042380199e-20	0.383515335936694	0.549	0.282	2.16651134273471e-15	PIK3CD
Rejected	34	6.951598190648e-20	0.799302626345609	0.691	0.463	2.33142700117953e-15	RUNX2
Rejected	34	7.65408426414711e-20	0.410706768655781	0.836	0.584	2.56702678050966e-15	MSI2
Rejected	34	8.09920082118035e-20	0.325705734130185	0.579	0.313	2.71630997140747e-15	FBXW4
Rejected	34	8.83075536831577e-20	0.38002804219127	0.962	0.755	2.96165873542574e-15	ANKRD11
Rejected	34	8.99212521420438e-20	0.446973628656315	0.814	0.549	3.01577895433986e-15	GLG1
Rejected	34	1.03187277050613e-19	0.487021416021407	0.959	0.866	3.46069489772346e-15	ZBTB20
Rejected	34	1.10520600068578e-19	0.417145847583982	0.65	0.378	3.70663988509997e-15	HMBOX1
Rejected	34	1.33873243573077e-19	0.414768084109974	0.995	0.947	4.48984084295386e-15	SKAP1
Rejected	34	1.38676101170372e-19	0.386649870754499	0.907	0.672	4.65091908105193e-15	SND1
Rejected	34	1.38927417939977e-19	0.488228993988835	0.923	0.76	4.65934774287096e-15	SH3KBP1
Rejected	34	1.69923929991046e-19	0.464710403451289	0.852	0.604	5.69890876403969e-15	USP25
Rejected	34	1.89731786962666e-19	0.487403525796708	0.844	0.615	6.36322467115388e-15	RERE
Rejected	34	2.09001824399886e-19	0.277477343220858	0.544	0.274	7.00950318672336e-15	SFI1
Rejected	34	2.09485280628285e-19	0.612777613162408	0.781	0.561	7.02571734171141e-15	ETV6
Rejected	34	3.18215940286908e-19	0.456911962949528	0.937	0.781	1.06723262053423e-14	NCOA2
Rejected	34	3.23701626206698e-19	0.441233410458882	0.566	0.322	1.08563051397202e-14	GRAP2
Rejected	34	4.18768503025035e-19	0.386450785265138	0.751	0.479	1.40446580544536e-14	PRRC2B
Rejected	34	4.50481405216709e-19	0.482015207992567	0.863	0.626	1.5108245368158e-14	CLASP1
Rejected	34	4.58157099154778e-19	0.467346897165101	0.607	0.354	1.5365672791453e-14	SIDT1
Rejected	34	5.05300574706199e-19	0.431860063686349	0.896	0.654	1.69467706744965e-14	RICTOR
Rejected	34	5.32314141757097e-19	0.366354936252264	0.686	0.398	1.78527516862495e-14	MTFR1
Rejected	34	5.4025418355425e-19	0.466511309307599	0.986	0.915	1.81190448080424e-14	JMJD1C
Rejected	34	5.5166525845931e-19	0.366976881003431	0.404	0.187	1.85017494382083e-14	PRMT3
Rejected	34	5.99558515323695e-19	0.469383260363034	0.631	0.365	2.01079934869261e-14	AC104365.1
Rejected	34	6.8183482081928e-19	0.457107320470409	0.88	0.695	2.2867376220637e-14	PACS1
Rejected	34	6.85638042401297e-19	0.301431605699938	0.423	0.193	2.29949286660547e-14	INKA2

Rejected	34	7.86827063078558e-19	0.45771008254713[illegible]	0.956	0.805	2.63886060415287e-14	DPYD
Rejected	34	8.20398757313907e-19	0.51276276401302[illegible]	0.82	0.577	2.75145335227938e-14	PAG1
Rejected	34	9.27826844135136e-19	0.34700361592676[illegible]	0.68	0.393	3.11174566986042e-14	ADD1
Rejected	34	9.62582536753352e-19	0.374111653927121	0.833	0.571	3.22830931176339e-14	MARK3
Rejected	34	1.09289035958803e-18	0.28807162258002[illegible]	0.478	0.232	3.66533568798633e-14	CREB3L2
Rejected	34	1.18139608012186e-18	0.36759089495467[illegible]	0.779	0.5	3.96216617351268e-14	SLA
Rejected	34	1.28674403028176e-18	0.39614213752270[illegible]	0.79	0.512	4.31548212875898e-14	TAOK1
Rejected	34	1.38267972865574e-18	0.50748712162071[illegible]	0.877	0.685	4.63723127396564e-14	CCDC91
Rejected	34	1.4825246597106e-18	0.33363104577315[illegible]	0.546	0.297	4.97209120373742e-14	AGPAT3
Rejected	34	1.49526594344297e-18	0.31682740102044[illegible]	0.801	0.502	5.01482292111902e-14	AKNA
Rejected	34	1.5143937846093e-18	0.35841780921952[illegible]	0.587	0.324	5.07897387482268e-14	DENND6A
Rejected	34	1.53451298438864e-18	0.397891108272321	0.79	0.492	5.14644964704263e-14	NSD1
Rejected	34	1.58123928892025e-18	0.45672050071129	0.839	0.597	5.30316032718075e-14	STIM2
Rejected	34	1.66923225527111e-18	0.46484983759383[illegible]	0.678	0.419	5.59827113772824e-14	SORL1
Rejected	34	1.71414261656865e-18	0.35252056042905[illegible]	0.464	0.231	5.74889150744793e-14	AC079793.1
Rejected	34	1.93847795550587e-18	0.30033078942310[illegible]	0.464	0.233	6.50126736717559e-14	HK1
Rejected	34	2.14633303557589e-18	0.34375289521291[illegible]	0.448	0.222	7.19837173471441e-14	WDR59
Rejected	34	2.80329856821533e-18	0.38422392935686	0.73	0.462	9.40170273808056e-14	MLLT10
Rejected	34	3.54944859637e-18	0.62860624003060[illegible]	0.885	0.743	1.19041407025057e-13	MAML2
Rejected	34	3.62864478366225e-18	0.38564756849019[illegible]	0.432	0.212	1.21697488754465e-13	ANKUB1
Rejected	34	3.86152337267517e-18	0.40749442446734	0.571	0.315	1.2950777087278e-13	MTR
Rejected	34	3.96247762584087e-18	0.39455439245573[illegible]	0.956	0.772	1.32893574615451e-13	KMT2C
Rejected	34	3.99153615172425e-18	0.47640768172193[illegible]	0.776	0.522	1.33868139456528e-13	HERC4
Rejected	34	4.18919205681176e-18	0.28904080434203[illegible]	0.402	0.182	1.40497123201353e-13	AC090772.2
Rejected	34	5.0821330002924e-18	0.42022891764448[illegible]	1	0.93	1.70444576563806e-13	RABGAP1L
Rejected	34	5.3506972449414e-18	0.34970553942505[illegible]	0.727	0.459	1.79451684200845e-13	SEC24B
Rejected	34	1.08963279138713e-17	0.28443814929954	0.582	0.308	3.65441045575415e-13	MGA
Rejected	34	1.14495460682657e-17	0.40692925593968[illegible]	0.749	0.502	3.83994876037496e-13	ATAD2B
Rejected	34	1.34723920419574e-17	0.37070634202288[illegible]	0.544	0.304	4.51837084303169e-13	CLEC16A
Rejected	34	1.37573286636743e-17	0.39085901878416[illegible]	0.833	0.581	4.6139328872231e-13	ZNF638
Rejected	34	1.49123890805915e-17	0.47127338920773[illegible]	0.88	0.676	5.00131704984877e-13	CEP85L

Rejected	34	1.59258073530617e-17	0.331998167346627	0.429	0.211	5.34119727006984e-13	PACSIN2
Rejected	34	1.60258463975545e-17	0.389041403375433	0.77	0.523	5.37474836481183e-13	CARD11
Rejected	34	1.82178464879754e-17	0.336833088726769	0.503	0.263	6.1099013551372e-13	ITGA6
Rejected	34	1.95795636016667e-17	0.435001132939545	0.94	0.771	6.56659404072697e-13	FAM49B
Rejected	34	1.97166059723349e-17	0.380445694394284	0.803	0.523	6.61255531100168e-13	GATAD2B
Rejected	34	2.11068800184786e-17	0.35494048823854	0.473	0.241	7.07882542059735e-13	GPR155
Rejected	34	2.35517246953729e-17	0.31658231672149	0.478	0.244	7.89877742833417e-13	PARG
Rejected	34	2.42825662334877e-17	0.326767896415718	0.694	0.419	8.1438870633871e-13	WWP1
Rejected	34	2.66907885278805e-17	0.392608865389856	0.653	0.385	8.95155665648056e-13	AL031772.1
Rejected	34	3.13162407500264e-17	0.281794886474854	0.391	0.184	1.05028408227439e-12	PIGL
Rejected	34	3.13404926025642e-17	0.644523958916647	0.634	0.407	1.0510974409048e-12	RASGRF2
Rejected	34	3.47630417145193e-17	0.513098525341857	0.596	0.366	1.16588289302155e-12	ST6GAL1
Rejected	34	3.57122259778219e-17	0.442801872932409	0.809	0.574	1.19771663484419e-12	SLFN12L
Rejected	34	3.80585551390412e-17	0.408994393757699	0.817	0.571	1.27640782225316e-12	TBC1D22A
Rejected	34	3.91747241951294e-17	0.418879451111672	0.738	0.486	1.31384190005625e-12	MKL1
Rejected	34	3.97907270041519e-17	0.550700085360172	0.399	0.199	1.33450140226525e-12	PELI2
Rejected	34	4.53011078636595e-17	0.330870946334995	0.505	0.265	1.51930855553141e-12	CBFB
Rejected	34	4.83874039759762e-17	0.358802680260695	0.926	0.729	1.62281675454629e-12	ASH1L
Rejected	34	4.98719250240523e-17	0.306937058826419	0.582	0.321	1.67260462145667e-12	NUP214
Rejected	34	5.18807762500985e-17	0.386175936202622	0.97	0.869	1.7399774738758e-12	PITPNC1
Rejected	34	5.23543440195884e-17	0.578761095798	0.555	0.337	1.75585998972896e-12	SRBD1
Rejected	34	6.56266472143461e-17	0.389414272418885	0.929	0.737	2.20098649427474e-12	KDM4C
Rejected	34	7.24032299652474e-17	0.534349759062947	0.765	0.515	2.42825952657447e-12	XYLT1
Rejected	34	7.34109480445839e-17	0.390389162065106	0.915	0.754	2.46205637551925e-12	PAN3
Rejected	34	7.9693145037512e-17	0.300853657624942	0.44	0.221	2.67274869826808e-12	ANKFY1
Rejected	34	8.8283131047629e-17	0.428372749576807	0.852	0.629	2.96083964907538e-12	USP3
Rejected	34	9.53305170030349e-17	0.369941381120733	0.607	0.358	3.19719487924779e-12	MAP2K1
Rejected	34	1.01481659366565e-16	0.258554021589279	0.541	0.293	3.40349189183584e-12	NUP210
Rejected	34	1.06236732301107e-16	0.314351688282499	0.609	0.348	3.56296752791452e-12	DIS3L2
Rejected	34	1.08461959759013e-16	0.391074864067042	0.642	0.407	3.63759720639779e-12	CYFIP2
Rejected	34	1.16805938044234e-16	0.422044726928138	0.587	0.348	3.91743755012751e-12	EXOC6

Rejected	34	1.19487726753621e-16	0.35046217438519[illegible]	0.923	0.701	4.00737937986295e-12	BPTF
Rejected	34	1.30448716360629e-16	0.34206281610218[illegible]	0.937	0.762	4.37498904930276e-12	MYCBP2
Rejected	34	1.35287060755181e-16	0.44592686421677[illegible]	0.768	0.522	4.53725744360727e-12	PCNX2
Rejected	34	1.41806073581377e-16	0.34648820885180[illegible]	0.516	0.281	4.75589209577223e-12	ZFYVE28
Rejected	34	1.4367550156199e-16	0.39285780579282[illegible]	0.94	0.759	4.81858897138601e-12	KANSL1
Rejected	34	1.5451739235607e-16	0.40170638024104[illegible]	0.847	0.589	5.18220430483786e-12	ATM
Rejected	34	1.60853586086808e-16	0.55871867154848[illegible]	0.765	0.542	5.39470757017936e-12	MAN2A1
Rejected	34	1.82849574763442e-16	0.37152751254252[illegible]	0.418	0.214	6.1324090384163e-12	SERGEF
Rejected	34	1.917426392822e-16	0.36795474798165[illegible]	0.809	0.565	6.43066463624644e-12	TNKS
Rejected	34	2.04450852386606e-16	0.41014380493658[illegible]	0.91	0.719	6.85687268734199e-12	ZNF644
Rejected	34	2.20657445803994e-16	0.40488772069329[illegible]	0.921	0.768	7.40040941737434e-12	PPP3CA
Rejected	34	2.73080968251815e-16	0.32401948651300[illegible]	0.555	0.313	9.15858951322938e-12	ANKRD36C
Rejected	34	2.84690883209628e-16	0.38330544481726[illegible]	0.732	0.489	9.54796284108451e-12	NBAS
Rejected	34	2.92539408784264e-16	0.34163196290592[illegible]	0.645	0.385	9.81118669180663e-12	FAM120A
Rejected	34	3.12660848454445e-16	0.44994771752933[illegible]	0.582	0.354	1.04860195354652e-11	MAP2K5
Rejected	34	3.31005065654244e-16	0.44601038277526[illegible]	0.702	0.46	1.1101247891912e-11	SLC16A7
Rejected	34	3.42859389655879e-16	0.36165023487257[illegible]	0.727	0.459	1.14988182102789e-11	EFR3A
Rejected	34	3.61213134507713e-16	0.33578991556520[illegible]	0.88	0.604	1.21143661051197e-11	ARFGEF1
Rejected	34	3.62554336044512e-16	0.41256452047717	0.311	0.139	1.21593473222608e-11	EVA1C
Rejected	34	3.95729896702734e-16	0.41563096668253	0.538	0.308	1.32719892756163e-11	VRK2
Rejected	34	4.15181301156494e-16	0.47640293854371[illegible]	0.656	0.418	1.39243504781865e-11	SLCO3A1
Rejected	34	4.19226385385834e-16	0.53236467291600[illegible]	0.762	0.562	1.40600145130701e-11	MBD5
Rejected	34	4.347902318119e-16	0.39474971335895[illegible]	0.757	0.493	1.45819947945075e-11	SGMS1
Rejected	34	5.04094578668625e-16	0.30372192634751[illegible]	0.538	0.303	1.69063239793883e-11	ERICH1
Rejected	34	5.42148438930226e-16	0.47435808182248[illegible]	0.76	0.54	1.81825743448419e-11	MAP4
Rejected	34	5.50921155655675e-16	0.32404835659045[illegible]	0.891	0.634	1.847679371838e-11	TRIP12
Rejected	34	5.60703103645547e-16	0.44966255969725[illegible]	0.866	0.719	1.88048606900644e-11	CAMK4
Rejected	34	5.96538802686999e-16	0.40034707151137[illegible]	0.902	0.692	2.00067183645166e-11	PPP1R12A
Rejected	34	6.00685681555959e-16	0.46322000126375[illegible]	0.743	0.506	2.01457963880238e-11	ADAM10
Rejected	34	6.13931986986722e-16	0.32562221678945[illegible]	0.571	0.328	2.05900509795607e-11	NR1D2
Rejected	34	6.21507958515419e-16	0.34327464939625[illegible]	0.962	0.776	2.08441339126901e-11	USP34

Rejected	34	6.83421636869308e-16	0.262208661049131	0.533	0.29	2.29205948573228e-11	CDK5RAP2
Rejected	34	8.21680499047573e-16	0.344819950274952	0.26	0.109	2.75575205770575e-11	KCNQ1
Rejected	34	9.11673696337531e-16	0.306709159782246	0.724	0.442	3.05757124277681e-11	TOP2B
Rejected	34	9.54056396231726e-16	0.385210567569739	0.825	0.585	3.19971434168196e-11	CMIP
Rejected	34	9.97213238819667e-16	0.471904788728279	0.874	0.702	3.3444537603534e-11	GMDS
Rejected	34	1.05135401522914e-15	0.447007547030845	0.801	0.572	3.52603109627549e-11	MALT1
Rejected	34	1.08430469317208e-15	0.428611707306093	0.73	0.497	3.63654107996053e-11	MCUB
Rejected	34	1.14331912452704e-15	0.374336159540662	0.888	0.665	3.83446367983879e-11	HELZ
Rejected	34	1.17390918009698e-15	0.3741568555241	0.923	0.695	3.93705660820924e-11	AP001011.1
Rejected	34	1.26320240689304e-15	0.426031414965638	0.902	0.727	4.23652823223788e-11	CDKAL1
Rejected	34	1.42907806890451e-15	0.285279137328229	0.44	0.227	4.79284202749194e-11	AC006059.1
Rejected	34	1.86578986821913e-15	0.331872445176635	0.579	0.337	6.25748606003333e-11	DGKD
Rejected	34	1.89783762471233e-15	0.296171480226072	0.68	0.412	6.36496782576021e-11	PARP4
Rejected	34	2.07004547200321e-15	0.359360144221679	0.978	0.89	6.94251850400435e-11	EML4
Rejected	34	2.11934481623787e-15	0.267726851690537	0.434	0.219	7.10785864469858e-11	STAMBPL1
Rejected	34	2.44434884744899e-15	0.337193140062456	0.344	0.162	8.19785716457443e-11	DOK6
Rejected	34	2.51657492082849e-15	0.412779812105594	0.667	0.44	8.4400889694746e-11	STX8
Rejected	34	2.62335050735151e-15	0.403557939018269	0.997	0.959	8.79819293155548e-11	CBLB
Rejected	34	2.63698589481253e-15	0.34786141345431	0.486	0.269	8.84392329402225e-11	TANGO6
Rejected	34	3.00996309834539e-15	0.40373212565572	0.943	0.779	1.00948142392308e-10	WNK1
Rejected	34	3.15305006533412e-15	0.491113666605511	0.626	0.405	1.05746993091176e-10	PRKCE
Rejected	34	3.52985738499628e-15	0.325542987170099	0.557	0.331	1.18384356978005e-10	SEL1L3
Rejected	34	3.6566272467024e-15	0.399867504302598	0.833	0.652	1.22635964599905e-10	KAT6B
Rejected	34	3.91420948317173e-15	0.415027513391451	0.844	0.607	1.31274757646613e-10	DLG1
Rejected	34	4.02076048661884e-15	0.470479209823326	0.675	0.445	1.34848265200223e-10	IL18R1
Rejected	34	4.19863130001306e-15	0.352879322595471	0.59	0.357	1.40813696539838e-10	KIAA1468
Rejected	34	4.33446895384817e-15	0.40594244093778	0.658	0.426	1.4536941977416e-10	RALGAPA2
Rejected	34	5.20444477784169e-15	0.298797764095173	0.883	0.63	1.74546668959254e-10	CAPZB
Rejected	34	5.38267012545505e-15	0.410449044148294	0.839	0.637	1.80523990667512e-10	BBX
Rejected	34	5.50276740290687e-15	0.347194543116271	0.492	0.276	1.84551813158691e-10	NOL10
Rejected	34	5.53433626825739e-15	0.335847770463834	0.557	0.321	1.85610569764816e-10	TARSL2

Rejected	34	5.95087829092979e-15	0.334979124796578	0.549	0.317	1.99580556121203e-10	CEP192
Rejected	34	6.23426035386975e-15	0.317609945859655	0.41	0.214	2.09084623748084e-10	LPCAT3
Rejected	34	6.43178062765286e-15	0.305813314115656	0.705	0.451	2.15709058690222e-10	NRF1
Rejected	34	6.51426705433359e-15	0.409687027833259	0.77	0.561	2.1847548846824e-10	RNGTT
Rejected	34	6.52802564747455e-15	0.373848894090673	0.863	0.619	2.18936924165001e-10	CRYBG1
Rejected	34	6.69653087521093e-15	0.370709508250586	0.44	0.239	2.24588252492824e-10	MAGED1
Rejected	34	7.33273641060427e-15	0.403376178014518	0.885	0.669	2.45925313738846e-10	BCL11B
Rejected	34	8.03538198126025e-15	0.334328574734831	0.973	0.864	2.69490640887506e-10	SMAP2
Rejected	34	8.61430552343063e-15	0.371744406520518	0.863	0.636	2.88906578644817e-10	DIAPH1
Rejected	34	8.65625787261092e-15	0.352430451324763	0.801	0.556	2.90313576531625e-10	SLC4A7
Rejected	34	9.64946116140152e-15	0.427488735291546	0.333	0.163	3.23623628431084e-10	MAP2K6
Rejected	34	9.85193979051732e-15	0.339256806213555	0.358	0.181	3.3041435669437e-10	SFMBT1
Rejected	34	1.09151221309131e-14	0.471071194185698	0.5	0.285	3.66071366026564e-10	LRIG1
Rejected	34	1.10243907614999e-14	0.298689548621171	0.675	0.426	3.69736017359183e-10	UBE3C
Rejected	34	1.1258415188874e-14	0.405740248331732	0.697	0.462	3.77584728604455e-10	MNAT1
Rejected	34	1.13030356896328e-14	0.355112438653645	0.32	0.15	3.79081210958905e-10	TTN
Rejected	34	1.19191785030247e-14	0.311425092145839	0.705	0.451	3.99745408634443e-10	CHD6
Rejected	34	1.38142371346931e-14	0.366415518488498	0.639	0.411	4.63301885023339e-10	RFX7
Rejected	34	1.40493093125705e-14	0.432857054125521	0.995	0.934	4.7118573572499e-10	CNOT6L
Rejected	34	1.40584027245765e-14	0.259556895076382	0.511	0.282	4.71490710576847e-10	EIF4E3
Rejected	34	1.44401899825598e-14	0.330648746916976	0.59	0.358	4.84295091635092e-10	ARHGEF6
Rejected	34	1.63757471018644e-14	0.334065250791587	0.891	0.7	5.49209806302327e-10	MYH9
Rejected	34	1.64485817792089e-14	0.345389469014932	0.475	0.264	5.51652535711108e-10	KPNA3
Rejected	34	1.68663915608636e-14	0.352607808456388	0.937	0.813	5.65665040168243e-10	STAG1
Rejected	34	1.8177480945899e-14	0.381820285067105	0.475	0.273	6.09636355963560e-10	CCM2
Rejected	34	1.97657311011718e-14	0.292383939868855	0.393	0.201	6.62903089671101e-10	ZC3H6
Rejected	34	2.04166518885481e-14	0.2666189315799	0.495	0.273	6.84733671038128e-10	DIP2A
Rejected	34	2.08302475605785e-14	0.278553018287286	0.511	0.288	6.98604842686682e-10	ERAP2
Rejected	34	2.08306763643597e-14	0.420272410668262	0.339	0.166	6.98619223907896e-10	ELOVL6
Rejected	34	2.12420445538826e-14	0.270100512475038	0.35	0.17	7.12415690248116e-10	RAD54L2
Rejected	34	2.1293369009835e-14	0.313436363560231	0.317	0.15	7.14137009851845e-10	TUBGCP3

Rejected	34	2.15103151883313e-14	0.391654314681559	0.719	0.485	7.21412950786255e-10	COA1
Rejected	34	2.20337603386305e-14	0.423862838727021	0.956	0.873	7.38968254236991e-10	ATXN1
Rejected	34	2.20375832270412e-14	0.271553779705109	0.235	0.093	7.39096466268507e-10	ZMAT1
Rejected	34	2.41228174018847e-14	0.335591900659159	0.574	0.349	8.09031050024407e-10	MAP3K4
Rejected	34	2.46456561994989e-14	0.383323820962459	0.391	0.205	8.26566017618793e-10	C22orf34
Rejected	34	2.50145185018609e-14	0.604242163838232	0.434	0.254	8.3893692151541e-10	DDX3Y
Rejected	34	2.69742145180243e-14	0.322499356800724	0.328	0.158	9.04661206505498e-10	FBXL4
Rejected	34	2.77788976845316e-14	0.384151214383553	0.833	0.663	9.31648670543821e-10	ANKRD17
Rejected	34	2.82490701032804e-14	0.288567036270345	0.366	0.183	9.47417313123817e-10	CKAP5
Rejected	34	2.88381673620347e-14	0.373823337453463	0.801	0.562	9.67174456987921e-10	ZNF148
Rejected	34	3.04717804700817e-14	0.544130488077581	0.913	0.805	1.0219625734056e-09	MGAT5
Rejected	34	3.27489588157612e-14	0.253230265555573	0.456	0.244	1.098334580763e-09	EXOC1
Rejected	34	3.31404826351131e-14	0.359861124863982	0.609	0.38	1.11146550661642e-09	AC118549.1
Rejected	34	3.45973761326969e-14	0.326453701444618	0.863	0.622	1.16032680073839e-09	MARCH6
Rejected	34	3.48758177331467e-14	0.350148889407924	0.896	0.699	1.16966517513427e-09	EMB
Rejected	34	3.91212809271658e-14	0.381305102177747	0.839	0.651	1.31204951973529e-09	TAOK3
Rejected	34	3.96981412223192e-14	0.494350335756865	0.653	0.437	1.33139626031414e-09	RSRC1
Rejected	34	4.01824672806838e-14	0.25802132395211	0.38	0.192	1.34763958765957e-09	ABHD2
Rejected	34	4.64047798991868e-14	0.347335129170629	0.331	0.162	1.55632350825893e-09	BMT2
Rejected	34	4.89068046806473e-14	0.268078982737311	0.582	0.339	1.64023641537955e-09	KLF9
Rejected	34	5.05536595945267e-14	0.291205674528787	0.699	0.449	1.69546863548124e-09	RBM26
Rejected	34	5.05539520765768e-14	0.309596989947274	0.79	0.536	1.69547844474423e-09	PHC3
Rejected	34	5.08739075976043e-14	0.253852597092942	0.607	0.364	1.70620911300845e-09	SFSWAP
Rejected	34	5.11833883592289e-14	0.350447682598723	0.669	0.422	1.71658847879182e-09	SYNE1
Rejected	34	5.25241668247624e-14	0.42456664209363	0.404	0.221	1.76155550696888e-09	CLYBL
Rejected	34	5.49698176710471e-14	0.31622918683023	0.866	0.624	1.84357774505158e-09	CREBBP
Rejected	34	5.54819229630326e-14	0.371191891135758	0.91	0.726	1.86075273233419e-09	FAF1
Rejected	34	6.66752311270214e-14	0.340762004059977	0.579	0.348	2.23615390153804e-09	NFKBID
Rejected	34	6.74420375008964e-14	0.33697967715183	0.88	0.68	2.26187105370506e-09	ATP11B
Rejected	34	6.77064256466414e-14	0.334702730969673	0.451	0.246	2.27073810333706e-09	AC092652.2
Rejected	34	6.89578568766883e-14	0.38190292166156	0.555	0.346	2.31270860393037e-09	R3HDM1

Rejected	34	6.94053241129125e-14	0.334215843881901	0.959	0.81	2.32771576009886e-09	MACF1
Rejected	34	7.19988319417409e-14	0.281569791886975	0.35	0.171	2.41469682566211e-09	KATNAL1
Rejected	34	7.23046063039693e-14	0.458088937991834	0.872	0.721	2.42495188622252e-09	ARL15
Rejected	34	7.55825375301805e-14	0.446531923879292	0.601	0.39	2.53488714368719e-09	CD226
Rejected	34	7.62157587340376e-14	0.350334648884747	0.628	0.405	2.55612411642215e-09	CHD9
Rejected	34	7.77426202701488e-14	0.257573754533422	0.628	0.378	2.60733199862025e-09	C16orf72
Rejected	34	8.04556931421014e-14	0.284570774010025	0.697	0.441	2.6983230365998e-09	IGF2R
Rejected	34	8.50226377019402e-14	0.339492849285982	0.831	0.614	2.85148922324767e-09	ACAP2
Rejected	34	9.8794676481757e-14	0.30129927967723	0.358	0.177	3.31337585984517e-09	MCC
Rejected	34	1.04259505534933e-13	0.367823492893045	0.426	0.233	3.49665529663059e-09	TGFBR3
Rejected	34	1.11124087389167e-13	0.325343993358006	0.705	0.47	3.72687964285788e-09	THADA
Rejected	34	1.19516265010615e-13	0.256390555216722	0.454	0.248	4.00833649592602e-09	SLC30A9
Rejected	34	1.20021081616317e-13	0.429720294993524	0.571	0.373	4.02526703524805e-09	XKR6
Rejected	34	1.23058856951148e-13	0.485575992124847	0.544	0.345	4.12714794442759e-09	DISC1
Rejected	34	1.24296200595885e-13	0.318517379408252	0.716	0.473	4.1686459755848e-09	RNF216
Rejected	34	1.25765048212429e-13	0.321098968550416	0.762	0.515	4.21790818694844e-09	RFTN1
Rejected	34	1.31297584507955e-13	0.36255467901392	0.754	0.512	4.40345838922778e-09	WDR70
Rejected	34	1.55347510524466e-13	0.455581414300045	0.866	0.69	5.21004480796955e-09	NEDD9
Rejected	34	1.59129938474467e-13	0.268703111940409	0.35	0.173	5.33689987655667e-09	ELP3
Rejected	34	1.67435287692803e-13	0.281939790434279	0.607	0.373	5.61544467864123e-09	ATR
Rejected	34	1.69696492320147e-13	0.411007801709608	0.713	0.518	5.69128095943309e-09	PTPRA
Rejected	34	1.80406727488678e-13	0.366845483747665	0.508	0.304	6.05048082651528e-09	AVL9
Rejected	34	1.83969988129026e-13	0.299804248456586	0.779	0.534	6.16998546187128e-09	PRKDC
Rejected	34	1.85520182662008e-13	0.414523746287045	0.809	0.602	6.22197588611843e-09	NEK7
Rejected	34	1.89375323011726e-13	0.320095884546143	0.697	0.472	6.35126958316728e-09	SLMAP
Rejected	34	1.94176478795692e-13	0.332067490038538	0.377	0.202	6.51229074584993e-09	EXT2
Rejected	34	1.98992839653011e-13	0.334869109584337	0.358	0.187	6.67382185628269e-09	L3MBTL3
Rejected	34	2.02693141078774e-13	0.299301002493086	0.44	0.245	6.79792256549992e-09	PIK3CB
Rejected	34	2.02804602577764e-13	0.303969791128965	0.574	0.358	6.80166076125305e-09	FAM120B
Rejected	34	2.03033374776801e-13	0.459213369369237	0.574	0.377	6.80933332326434e-09	CHD7
Rejected	34	2.05088830837379e-13	0.701625530160262	0.833	0.674	6.87826920862401e-09	BCL2

Rejected	34	2.14427109759857e-13	0.446163168167376	0.574	0.368	7.19145640712609e-09	DGKH
Rejected	34	2.16948762476223e-13	0.383195239746592	0.727	0.522	7.27602759592755e-09	SPPL3
Rejected	34	2.46535319361486e-13	0.331118019852149	0.858	0.624	8.26830154074553e-09	SENP6
Rejected	34	2.54871536482979e-13	0.273080975374231	0.546	0.323	8.54788159056614e-09	TAF1
Rejected	34	2.58903552459973e-13	0.274187528035607	0.954	0.813	8.68310734240258e-09	BIRC6
Rejected	34	2.62166624684846e-13	0.518155328198722	0.822	0.696	8.79254425868035e-09	SMYD3
Rejected	34	2.82651148617619e-13	0.270025769747607	0.648	0.401	9.47955422233771e-09	USP48
Rejected	34	2.94937683053121e-13	0.480381207544422	0.806	0.63	9.89162001423557e-09	SRPK2
Rejected	34	2.98365023401573e-13	0.420611492370558	0.757	0.539	1.0006566154842e-08	STK39
Rejected	34	3.54736306483861e-13	0.326618229976016	0.732	0.491	1.18971462468557e-08	PHTF2
Rejected	34	3.64305762296031e-13	0.311097650687968	0.568	0.347	1.22180866558843e-08	TXK
Rejected	34	3.67890910123327e-13	0.346625869033733	0.503	0.298	1.23383253437161e-08	SIPA1L3
Rejected	34	3.73477653556526e-13	0.303127273666599	0.421	0.235	1.25256935449788e-08	CASD1
Rejected	34	3.90109127385993e-13	0.30563830425462	0.653	0.411	1.30834799142714e-08	RAB3GAP2
Rejected	34	4.3336721426385e-13	0.326856269234926	0.669	0.435	1.4534269631981e-08	EXOC2
Rejected	34	4.43292641798032e-13	0.253024813595951	0.486	0.276	1.48671486206224e-08	MTMR1
Rejected	34	4.58361240910009e-13	0.29913883701036	0.577	0.347	1.53725192976399e-08	RNF13
Rejected	34	4.64489830470751e-13	0.346210220013404	0.41	0.221	1.55780599343281e-08	ATXN7L1
Rejected	34	4.68292852401268e-13	0.278279420576543	0.38	0.201	1.57056056838337e-08	XPO7
Rejected	34	4.84764425520193e-13	0.264307441245923	0.546	0.326	1.62580293030962e-08	DGLUCY
Rejected	34	4.92118163280659e-13	0.452294266716735	0.484	0.291	1.65046589601067e-08	GMDS-DT
Rejected	34	5.44933672629509e-13	0.373304380160788	0.675	0.457	1.82759855126485e-08	ITPKB
Rejected	34	5.66058145329562e-13	0.312521491546103	0.874	0.657	1.89844580780629e-08	UBR5
Rejected	34	5.73399285355404e-13	0.532055815094179	0.467	0.284	1.92306652322495e-08	INVS
Rejected	34	5.83303452287537e-13	0.278118179333075	0.918	0.699	1.95628311828194e-08	USP9X
Rejected	34	5.89845107722876e-13	0.391461751598189	0.202	0.082	1.97822252228098e-08	C12orf42
Rejected	34	5.92746981076653e-13	0.319865530204494	0.434	0.243	1.98795482513488e-08	STX18
Rejected	34	5.98019937062357e-13	0.282373250929761	0.822	0.58	2.00563926491973e-08	EPC2
Rejected	34	6.05978306129325e-13	0.2927335856243	0.847	0.593	2.03233004309653e-08	OGA
Rejected	34	6.06753719358767e-13	0.470997530165164	0.505	0.312	2.03493062398543e-08	CEP128
Rejected	34	6.92374078574373e-13	0.35236258551687	0.814	0.588	2.32208418472273e-08	PRKCQ

Rejected	34	6.97935763722247e-13	0.25633503366368	0.716	0.461	2.34073696437167e-08	YLPM1
Rejected	34	7.08356376181481e-13	0.41954273542746	0.776	0.566	2.37568561443745e-08	SFMBT2
Rejected	34	7.74867882723086e-13	0.27215402373677	0.631	0.386	2.59875190507669e-08	TRIM22
Rejected	34	7.86778985023367e-13	0.30032158006497	0.967	0.836	2.63869935997137e-08	SLC38A1
Rejected	34	7.8735771247024e-13	0.26954243300941	0.716	0.47	2.64064029608269e-08	ARID1A
Rejected	34	7.95587115596132e-13	0.32530480996386	0.754	0.532	2.66824006828631e-08	SCAF8
Rejected	34	8.18928178978124e-13	0.34642955952722	0.738	0.52	2.74652132665683e-08	ATP9B
Rejected	34	9.63398472497729e-13	0.29555204560817	0.579	0.356	3.23104579706289e-08	SPG11
Rejected	34	9.75568850746164e-13	0.30704737302532	0.902	0.736	3.27186281163248e-08	BCLAF1
Rejected	34	9.83599415775954e-13	0.35604115862090	0.41	0.218	3.29879572062939e-08	TNFSF8
Rejected	34	1.00593739318459e-12	0.27313248846001	0.683	0.44	3.37371282926247e-08	ZZEF1
Rejected	34	1.0400405438918e-12	0.36324476535812	0.374	0.2	3.48808797610433e-08	ABL2
Rejected	34	1.1244746919285e-12	0.41772921981749	0.787	0.588	3.77126322178981e-08	OSBPL3
Rejected	34	1.14678777362573e-12	0.41826905700148	0.475	0.276	3.84609683518598e-08	EHBP1
Rejected	34	1.21684164319198e-12	0.39289853384958	0.331	0.174	4.08104350293727e-08	LINC01184
Rejected	34	1.25210117699794e-12	0.34765127459796	0.776	0.576	4.19929692741568e-08	PTPN4
Rejected	34	1.29041185367115e-12	0.35897150179202	0.724	0.512	4.32778327484231e-08	NUTM2B-AS1
Rejected	34	1.33322648446445e-12	0.29232621338254	0.91	0.709	4.47137498359688e-08	VPS13C
Rejected	34	1.34691214910016e-12	0.35280801784010	0.538	0.333	4.51727396565212e-08	PDE8A
Rejected	34	1.35109534760825e-12	0.49370196665338	0.516	0.325	4.53130357680856e-08	FAM69A
Rejected	34	1.36043361891323e-12	0.38157084415763	0.366	0.196	4.56262227111121e-08	ATRN
Rejected	34	1.41836796948431e-12	0.33812230018262	0.951	0.797	4.75692249605647e-08	MED13L
Rejected	34	1.42947609670161e-12	0.30814923472670	0.65	0.426	4.79417693311785e-08	AMBRA1
Rejected	34	1.43806693663117e-12	0.43195822135700	0.577	0.387	4.82298889207361e-08	ATXN10
Rejected	34	1.57713796696128e-12	0.52019953769167	0.743	0.58	5.28940531359475e-08	LINC01934
Rejected	34	1.6281560135753e-12	0.26483530844870	0.413	0.229	5.46050963832883e-08	SHPRH
Rejected	34	1.65484338396748e-12	0.25971300589341	0.388	0.209	5.55001374115012e-08	ZNF236
Rejected	34	1.74441711648766e-12	0.31301324798191	0.686	0.444	5.85042612527631e-08	VPS13A
Rejected	34	1.84146672788363e-12	0.28953373009893	0.462	0.268	6.17591111197612e-08	CMC1
Rejected	34	1.95543432921413e-12	0.27750542695607	0.541	0.334	6.55813565331833e-08	WWP2
Rejected	34	1.98160415669383e-12	0.54684455073912	0.445	0.272	6.64590402071978e-08	APBA2

Rejected	34	1.99737051438314e-12	0.39050122614606	0.82	0.634	6.69878123113819e-08	FNDC3A
Rejected	34	2.12504466747919e-12	0.30360599151411	0.678	0.444	7.1269748057917e-08	OPA1
Rejected	34	2.30972635187433e-12	0.28896524311126	0.456	0.263	7.74636023891611e-08	SLC25A12
Rejected	34	2.32999974993979e-12	0.35050512119952	0.997	0.899	7.81435316134807e-08	SMCHD1
Rejected	34	2.33221284074039e-12	0.51179297123663	0.374	0.21	7.82177542527513e-08	PRKN
Rejected	34	2.52093457280314e-12	0.27297001080185	0.538	0.315	8.45471037026715e-08	AL139807.1
Rejected	34	2.62797279298666e-12	0.31773398708279	0.809	0.579	8.81369515311865e-08	HBP1
Rejected	34	2.87033559173735e-12	0.29982884291311	0.333	0.173	9.62653150756872e-08	LINC00426
Rejected	34	2.99287947084518e-12	0.33085513516923	0.945	0.782	1.00375191693206e-07	PDE7A
Rejected	34	3.43001079392482e-12	0.38294014932330	0.508	0.317	1.15035702006651e-07	NT5DC1
Rejected	34	3.8236102483889e-12	0.31865460366550	0.697	0.466	1.28236240510467e-07	CD2AP
Rejected	34	3.88325732395293e-12	0.26475470442842	0.954	0.784	1.30236684130733e-07	DYRK1A
Rejected	34	3.88616631639981e-12	0.39999083721174	0.708	0.518	1.30334245919417e-07	SCAPER
Rejected	34	3.94527583826548e-12	0.32415408940433	0.702	0.484	1.32316661063748e-07	PTEN
Rejected	34	4.03154410257547e-12	0.3548837526853	0.71	0.497	1.35209926112176e-07	MYO5A
Rejected	34	4.3108424933275e-12	0.32387761097581	0.97	0.905	1.44577035541218e-07	FOXP1
Rejected	34	4.33989957183292e-12	0.32084005426252	0.582	0.364	1.45551551840132e-07	POU2F1
Rejected	34	4.37802524732598e-12	0.30492554547334	0.563	0.36	1.46830210744819e-07	ARMH3
Rejected	34	4.47408018023044e-12	0.26803573870215	0.128	0.041	1.50051701084569e-07	CALCB
Rejected	34	4.53636738200197e-12	0.42618622942810	0.396	0.224	1.52140689257582e-07	PTPRM
Rejected	34	4.84553293542585e-12	0.29551763954663	0.776	0.56	1.62509483588312e-07	LNPEP
Rejected	34	4.89925562459828e-12	0.26536882007608	0.459	0.267	1.64311235137777e-07	RCOR3
Rejected	34	5.07787796722581e-12	0.30260463492923	0.358	0.192	1.70301871264819e-07	VPS50
Rejected	34	5.40673220589489e-12	0.37304679692085	0.833	0.623	1.81330984721303e-07	AHNAK
Rejected	34	5.50234635639552e-12	0.35117101023110	0.309	0.16	1.84537692100793e-07	ALPK1
Rejected	34	6.16993566127865e-12	0.32867891710076	0.73	0.511	2.06927302207963e-07	TNRC6A
Rejected	34	6.56038024479676e-12	0.32416561161645	0.817	0.607	2.20022032649994e-07	ARID2
Rejected	34	6.58019482410877e-12	0.28250729414223	0.399	0.217	2.2068657401096e-07	RNF144A
Rejected	34	6.60972711513855e-12	0.37247742060493	0.47	0.292	2.21677027987517e-07	TMEM65
Rejected	34	6.75765557531728e-12	0.25538745528760	0.883	0.677	2.26638252684991e-07	NCOR1
Rejected	34	7.1820956782523e-12	0.30388308757296	0.664	0.439	2.40873124857226e-07	UBL3

Rejected	34	8.23733663610942e-12	0.29665696200141€	0.852	0.638	2.76263796101838e-07	ITSN2
Rejected	34	8.64645976436305e-12	0.39465444913352€	0.858	0.681	2.89984967577208e-07	TMEM131L
Rejected	34	9.09529672409743e-12	0.51233191342293€	0.888	0.72	3.0503806153278e-07	RORA-AS1
Rejected	34	9.4697993030637e-12	0.35757321932504€	0.765	0.568	3.1759812902615e-07	SENP7
Rejected	34	9.89628770824322e-12	0.30353763762659⁄	0.921	0.753	3.31901697159061e-07	PPP6R3
Rejected	34	1.04363880517895e-11	0.40979147270053€	0.915	0.767	3.50015582480916e-07	MAPRE2
Rejected	34	1.0933267505824e-11	0.28040997092089€	0.557	0.356	3.66679925610324e-07	TYW1
Rejected	34	1.10851493946039e-11	0.36784016261638⁄	0.418	0.24	3.71773740396225e-07	LINC02320
Rejected	34	1.1479620073498e-11	0.29086641335134⁄	0.727	0.501	3.85003498024977e-07	BRWD1
Rejected	34	1.18400362969617e-11	0.35266302864254⁄	0.653	0.463	3.97091137327503e-07	PPP2R5E
Rejected	34	1.19967834536028e-11	0.43337812607444⁄	0.669	0.471	4.02348123466931e-07	FCHSD2
Rejected	34	1.21257368210101e-11	0.29532666422983⁄	0.423	0.242	4.06672961503036e-07	PREP
Rejected	34	1.32188063720596e-11	0.26612022396951⁄	0.445	0.262	4.43332328106135e-07	MIB1
Rejected	34	1.38236588580379e-11	0.32913848652991€	0.358	0.193	4.63617870780876e-07	GPD2
Rejected	34	1.40582416001082e-11	0.32457222848199⁄	0.568	0.361	4.71485306784429e-07	ANO6
Rejected	34	1.72627534652267e-11	0.30249211510479⁄	0.661	0.44	5.78958225716773e-07	FBXW11
Rejected	34	1.84356371760359e-11	0.39542182706564⁄	0.869	0.736	6.18294399609893e-07	AKT3
Rejected	34	1.96567377101609e-11	0.27493192758421⁄	0.88	0.652	6.59247669323378e-07	ROCK1
Rejected	34	1.99557760406532e-11	0.25902263251446⁄	0.563	0.342	6.69276816851427e-07	ATP2C1
Rejected	34	2.01622559246701e-11	0.2760542420739	0.705	0.487	6.76201739201585e-07	FAM208B
Rejected	34	2.03248879153942e-11	0.36559767739040⁄	0.825	0.68	6.8165609090649e-07	ZCCHC7
Rejected	34	2.03595147763116e-11	0.26521535431904	0.713	0.481	6.8281740656794e-07	SUCO
Rejected	34	2.05018746591597e-11	0.25278256151448€	0.355	0.189	6.87591872318898e-07	CYREN
Rejected	34	2.32677926938149e-11	0.25138896767169€	0.322	0.17	7.80355231365164e-07	ORC4
Rejected	34	2.32762170586263e-11	0.39699396323476⁄	0.885	0.713	7.80637767712207e-07	CDC14A
Rejected	34	2.35635692796915e-11	0.30680117940749⁄	0.874	0.662	7.90274986502294e-07	FRYL
Rejected	34	2.40715525881125e-11	0.31572460056468€	0.478	0.293	8.07311730700116e-07	ABL1
Rejected	34	2.47247667019301e-11	0.46858137415086⁄	0.811	0.659	8.29219225649331e-07	CAMK2D
Rejected	34	2.64077491077304e-11	0.37378135428697⁄	0.874	0.707	8.85663089575063e-07	TBL1XR1
Rejected	34	2.73749783851732e-11	0.29164288935090⁄	0.59	0.381	9.1810202508194e-07	AEBP2
Rejected	34	2.83155996404575e-11	0.26037640546821⁄	0.369	0.203	9.49648580741662e-07	TGFBR1

Rejected	34	2.86485402645218e-11	0.259592414165817	0.792	0.54	9.60814743391531e-07	EHMT1
Rejected	34	2.9463419649844e-11	0.344414261029006	0.612	0.403	9.88144168216469e-07	RCSD1
Rejected	34	3.14405906685935e-11	0.369901728285125	0.716	0.512	1.05445452984329e-06	KIAA0232
Rejected	34	3.16003966121721e-11	0.252338687453193	0.762	0.543	1.05981410157903e-06	UBR1
Rejected	34	3.17980023508723e-11	0.36769096172795	0.56	0.374	1.06644140284356e-06	PHKB
Rejected	34	3.21603427074121e-11	0.264716175342511	0.492	0.299	1.07859357372119e-06	NLRP1
Rejected	34	3.32050357601479e-11	0.344893697795914	0.287	0.145	1.11363048932384e-06	MEGF9
Rejected	34	3.61780288990867e-11	0.336080159697209	0.825	0.624	1.21333873321757e-06	GSK3B
Rejected	34	3.6823822527545e-11	0.340950672297784	0.735	0.545	1.2349973599288e-06	PHIP
Rejected	34	3.86273650270963e-11	0.329053206717464	0.757	0.545	1.29548456827876e-06	ITCH
Rejected	34	4.27713583450781e-11	0.290999198767838	0.615	0.399	1.43446581617723e-06	ARNTL
Rejected	34	4.33997672993805e-11	0.390569511876778	0.56	0.363	1.45554139568662e-06	AGTPBP1
Rejected	34	4.37429400575426e-11	0.28479420633178	0.948	0.813	1.46705072364986e-06	CYTH1
Rejected	34	4.99887800144412e-11	0.299162100436143	0.497	0.305	1.67652370412433e-06	ATP10A
Rejected	34	5.30910336447539e-11	0.434771272219854	0.598	0.407	1.78056708637776e-06	PCED1B
Rejected	34	5.35895441320611e-11	0.467488847354637	0.243	0.116	1.79728613110107e-06	IFNG-AS1
Rejected	34	5.37838855894101e-11	0.465424000871607	0.404	0.242	1.80380395489763e-06	KIAA1328
Rejected	34	5.66948276118714e-11	0.320096473161928	0.751	0.528	1.90143112844694e-06	PCGF5
Rejected	34	6.05956046437377e-11	0.410083082259055	0.525	0.333	2.03225538854168e-06	HIF1A-AS2
Rejected	34	6.17469215600428e-11	0.301586829984273	0.596	0.394	2.07086825528072e-06	RPRD2
Rejected	34	6.34941454275971e-11	0.293925446921943	0.459	0.284	2.12946664935075e-06	ROCK2
Rejected	34	6.57291756319802e-11	0.313952876503158	0.716	0.476	2.20442509234535e-06	CLINT1
Rejected	34	6.57841364228658e-11	0.268141555277477	0.697	0.468	2.20626836735007e-06	TNRC6C
Rejected	34	6.73401056292107e-11	0.34396937961781	0.571	0.392	2.25845246259247e-06	GALNT10
Rejected	34	7.23441814697728e-11	0.262866679600056	0.631	0.417	2.42627915813324e-06	ZNF451
Rejected	34	7.45438028963767e-11	0.412044930063556	0.76	0.585	2.50005006153868e-06	ZNF407
Rejected	34	7.80551719426426e-11	0.331887026139041	0.754	0.562	2.61781435661235e-06	DENND1B
Rejected	34	8.1687946143728e-11	0.256011156314596	0.787	0.579	2.73965033776835e-06	TLK1
Rejected	34	8.44351171742167e-11	0.285183663489388	0.831	0.655	2.83178495978888e-06	FAM129A
Rejected	34	8.51689085867294e-11	0.321684127361101	0.497	0.317	2.85639485618173e-06	MED27
Rejected	34	9.36323962473919e-11	0.262467821753817	0.407	0.232	3.14024330534503e-06	DPP4

Rejected	34	1.00164871323094e-10	0.298418065645626	0.563	0.374	3.35932945443393e-06	SNX13
Rejected	34	1.04807190333633e-10	0.257701412199167	0.292	0.151	3.5150235494094e-06	FARSB
Rejected	34	1.05386148690528e-10	0.299148084754808	0.694	0.476	3.53444065478294e-06	PPP3CC
Rejected	34	1.07015206701424e-10	0.422929407717401	0.317	0.174	3.58907600235237e-06	ZNRF1
Rejected	34	1.07545202604257e-10	0.358223561150368	0.866	0.691	3.60685100494156e-06	OXNAD1
Rejected	34	1.1211993966079e-10	0.410180492571476	0.836	0.669	3.76027853634357e-06	ARID5B
Rejected	34	1.1310455399763e-10	0.255604279614728	0.669	0.464	3.79330053197253e-06	VPS13D
Rejected	34	1.20824621965168e-10	0.277485631523176	0.511	0.324	4.05221617146781e-06	BTRC
Rejected	34	1.23774358790754e-10	0.262666451438783	0.448	0.257	4.15114444512431e-06	AC109587.1
Rejected	34	1.25997241385095e-10	0.300128592459003	0.975	0.914	4.22569548157332e-06	CD44
Rejected	34	1.40184199863418e-10	0.598287832791953	0.604	0.452	4.70149769501932e-06	TRPS1
Rejected	34	1.4093813640696e-10	0.34832439207194	0.792	0.62	4.72678321881662e-06	KIF13B
Rejected	34	1.436783565024e-10	0.265662352721438	0.484	0.296	4.81868472037748e-06	THOC1
Rejected	34	1.44065384591452e-10	0.288334260965415	0.462	0.283	4.83166486842812e-06	BRWD3
Rejected	34	1.44731127224077e-10	0.347434045701078	0.645	0.463	4.8539925448411e-06	PIBF1
Rejected	34	1.45566940377596e-10	0.263889985901286	0.536	0.341	4.88202404638383e-06	SLC39A11
Rejected	34	1.46114035754388e-10	0.277731075115469	0.645	0.427	4.90037253113066e-06	GIGYF2
Rejected	34	1.52551189145214e-10	0.362597308876074	0.735	0.545	5.1162617815522e-06	DENND1A
Rejected	34	1.58269723238112e-10	0.438448265235623	0.628	0.451	5.3080499779598e-06	UMAD1
Rejected	34	1.59007756658513e-10	0.285146294818116	0.65	0.453	5.33280214281322e-06	DENND4C
Rejected	34	1.86059250479236e-10	0.299811589179272	0.249	0.123	6.24005514257262e-06	ORC5
Rejected	34	1.87054674471599e-10	0.418833378116698	0.475	0.298	6.27343967242847e-06	RASSF3
Rejected	34	1.87159793728599e-10	0.40641401982799	0.713	0.542	6.27696516206974e-06	MLLT3
Rejected	34	1.92423064560171e-10	0.293912710376202	0.306	0.162	6.45348473921901e-06	BMPR1A
Rejected	34	1.94973911302863e-10	0.264976152157997	0.311	0.166	6.53903503727543e-06	LARS2
Rejected	34	2.04838848992157e-10	0.317232596099632	0.776	0.567	6.86988531749896e-06	CCNY
Rejected	34	2.13295569371451e-10	0.25762140911442	0.705	0.495	7.15350680557973e-06	GALM
Rejected	34	2.14160168360119e-10	0.269256207260848	0.47	0.286	7.18250372646166e-06	DPYD-AS1
Rejected	34	2.15896406287142e-10	0.265360530232655	0.642	0.431	7.24073367405816e-06	MAP2K4
Rejected	34	2.51905097397014e-10	0.265895041818444	0.732	0.517	8.44839315650106e-06	TMEM131
Rejected	34	2.61692584595918e-10	0.318096221326353	0.574	0.374	8.7766459021779e-06	LRCH1

Rejected	34	2.99479528112654e-10	0.280111616268503	0.481	0.304	1.00439444138422e-05	TCF20
Rejected	34	3.21813146893948e-10	0.256865622855114	0.437	0.262	1.07929693205292e-05	SGK3
Rejected	34	3.41632356768569e-10	0.28119288537292	0.683	0.469	1.14576659813043e-05	CHCHD3
Rejected	34	3.42215559603526e-10	0.265225884070944	0.205	0.094	1.1477225437983e-05	LINC00278
Rejected	34	3.44165181607999e-10	0.259748768393373	0.746	0.555	1.15426118607691e-05	PPM1B
Rejected	34	3.65769970368568e-10	0.25187634477386	0.885	0.672	1.2267193266221e-05	HECA
Rejected	34	3.72981377313203e-10	0.295031612000755	0.366	0.212	1.25090494323302e-05	NOTCH2
Rejected	34	3.74066925273327e-10	0.300397254195063	0.855	0.688	1.25454565398168e-05	GOLGA4
Rejected	34	3.81810350412156e-10	0.359320463316053	0.861	0.741	1.28051555321229e-05	NR3C1
Rejected	34	4.03833044896126e-10	0.40133483811646	0.546	0.37	1.35437526597263e-05	SLC9A9
Rejected	34	4.53782799427038e-10	0.265729131192874	0.667	0.459	1.5218967527184e-05	GATAD2A
Rejected	34	5.31678294844241e-10	0.27030540871985	0.628	0.419	1.78314266524862e-05	GRK5
Rejected	34	5.32570798074961e-10	0.308638469250911	0.85	0.656	1.7861359425838e-05	USP47
Rejected	34	5.64698479893279e-10	0.309300119670967	0.855	0.688	1.89388576186608e-05	CSDE1
Rejected	34	5.79205535615546e-10	0.258880086462064	0.628	0.439	1.94253952534742e-05	TULP4
Rejected	34	6.05497458692771e-10	0.330321930937593	0.404	0.246	2.03071737696382e-05	TPK1
Rejected	34	6.11643368461456e-10	0.305884159710574	0.896	0.675	2.05132952914603e-05	TOP1
Rejected	34	6.15912296054153e-10	0.308123760772454	0.844	0.668	2.06564665850642e-05	ERBIN
Rejected	34	6.29953842998055e-10	0.292815929753953	0.891	0.711	2.11273919864688e-05	RAP1A
Rejected	34	6.72194700851453e-10	0.327474827024707	0.792	0.636	2.2544065877156e-05	ZNF609
Rejected	34	6.80989518915609e-10	0.429767758219684	0.396	0.242	2.28390264853917e-05	NFIA
Rejected	34	6.85283195551461e-10	0.461959814445294	0.623	0.454	2.29830278124049e-05	NR3C2
Rejected	34	6.85594544576489e-10	0.355481477275282	0.716	0.55	2.29934698360063e-05	DIAPH2
Rejected	34	7.00868102908139e-10	0.354042751342447	0.94	0.86	2.35057144353331e-05	RHOH
Rejected	34	7.08262307016761e-10	0.263120496364642	0.492	0.31	2.37537012527281e-05	AGPS
Rejected	34	7.34462134113205e-10	0.305422227232488	0.585	0.398	2.46323910538887e-05	MAP3K1
Rejected	34	7.48121415196932e-10	0.294815551836796	0.251	0.127	2.50904960228747e-05	IL15
Rejected	34	7.67993159408239e-10	0.307896851584608	0.585	0.406	2.57569545802335e-05	TMEM135
Rejected	34	8.25115479447486e-10	0.284785207911436	0.257	0.131	2.76727229497098e-05	CNNM2
Rejected	34	8.30561472611922e-10	0.33484370549881	0.62	0.427	2.78553706684586e-05	STXBP5
Rejected	34	8.48718746463617e-10	0.273600507015482	0.65	0.456	2.84643293188968e-05	STRN3

Rejected	34	8.62359296599336e-10	0.277491713181487	0.536	0.351	2.89218060893485e-05	MTMR3
Rejected	34	8.70764483676397e-10	0.331466435265782	0.658	0.462	2.9203699253539e-05	WDR7
Rejected	34	8.88799305608927e-10	0.409618842769795	0.921	0.784	2.98085511115122e-05	OSBPL8
Rejected	34	9.77373888214039e-10	0.528964853577617	0.615	0.466	3.27791654629224e-05	UBASH3B
Rejected	34	9.790406143421e-10	0.26384972665584	0.557	0.357	3.28350641238053e-05	ZMYND11
Rejected	34	1.08661454466929e-09	0.375250452167904	0.563	0.391	3.64428785991187e-05	SBF2
Rejected	34	1.12817234529473e-09	0.334207802230243	0.396	0.248	3.78366441164947e-05	DTNBP1
Rejected	34	1.12995871457371e-09	0.269746375039367	0.874	0.71	3.78965553693729e-05	ZEB1
Rejected	34	1.21086981805727e-09	0.251056487932142	0.544	0.354	4.06101519580047e-05	TBCK
Rejected	34	1.29349524030801e-09	0.286708417892062	0.639	0.437	4.33812433694501e-05	AC010894.3
Rejected	34	1.33420241101649e-09	0.26243313234357	0.754	0.549	4.47464804606712e-05	FOXJ3
Rejected	34	1.33451215402143e-09	0.413010878653241	0.612	0.447	4.47568686215709e-05	GTDC1
Rejected	34	1.45231312062675e-09	0.36669883203162	0.839	0.71	4.870767743958e-05	ARHGAP26
Rejected	34	1.50179238710602e-09	0.260208852295477	0.344	0.197	5.03671130787616e-05	SLC25A13
Rejected	34	1.69169843305799e-09	0.298922674081318	0.833	0.627	5.6736182047899e-05	PDS5A
Rejected	34	1.78189569208984e-09	0.316150856214518	0.631	0.449	5.97612177213092e-05	LMBR1
Rejected	34	1.83530565633552e-09	0.265165467939088	0.481	0.301	6.15524811021808e-05	OPRM1
Rejected	34	1.89286193207342e-09	0.322433100478103	0.768	0.593	6.34828034778783e-05	CAMK1D
Rejected	34	1.89370661960751e-09	0.304876460298773	0.795	0.633	6.35111326083966e-05	RPS6KA5
Rejected	34	2.13032076654296e-09	0.456686579197258	0.79	0.641	7.14466978683176e-05	EXT1
Rejected	34	2.17352204506815e-09	0.259223466052096	0.71	0.492	7.28955823474957e-05	HERPUD2
Rejected	34	2.18374263949238e-09	0.264598844058685	0.142	0.057	7.32383606432953e-05	ANKRD6
Rejected	34	2.30666850554516e-09	0.287719969185236	0.792	0.589	7.73610483389735e-05	GPATCH8
Rejected	34	2.76028408098081e-09	0.450030019449441	0.645	0.476	9.25744075079346e-05	GPHN
Rejected	34	2.79928199066415e-09	0.318088702022967	0.779	0.596	9.38823194028944e-05	LCOR
Rejected	34	2.98005470404723e-09	0.298397341052534	0.855	0.69	9.9945074664336e-05	JARID2
Rejected	34	3.05863728815886e-09	0.299344097876899	0.929	0.793	0.000102580577370272	CD247
Rejected	34	3.08962201671261e-09	0.264278375004236	0.82	0.642	0.000103619743196507	SOS1
Rejected	34	3.24854239831673e-09	0.334460805672166	0.705	0.53	0.000108949614954747	ITFG1
Rejected	34	3.40016285080657e-09	0.275375806412262	0.44	0.278	0.000114034661690351	RETREG1
Rejected	34	3.47433629301738e-09	0.38462808133918	0.604	0.434	0.000116522290595217	PAM

Rejected	34	3.83346059359689e-09	0.342359043489148	0.309	0.173	0.000128566601388052	C1QTNF3-AMACR
Rejected	34	4.06866283149011e-09	0.294887938663607	0.47	0.306	0.000136454814042515	VPS54
Rejected	34	4.44080536204226e-09	0.527906016691177	0.18	0.085	0.000148935730232173	CSGALNACT1
Rejected	34	4.7129575433179e-09	0.274146325546192	0.634	0.44	0.000158063170087796	PDS5B
Rejected	34	4.7566934032403e-09	0.442841127139203	0.825	0.685	0.000159529983357873	RAPGEF2
Rejected	34	5.18072603196693e-09	0.289061842588067	0.787	0.585	0.000173751189660107	ASCC3
Rejected	34	5.23294702017828e-09	0.251480317124276	0.257	0.137	0.000175502577162739	SLC4A4
Rejected	34	5.85613462661381e-09	0.251318564414103	0.492	0.319	0.000196403043107374	NSMAF
Rejected	34	6.51396316093878e-09	0.436537163430473	0.645	0.491	0.000218465296491565	COMMD10
Rejected	34	7.78791272278863e-09	0.274742138842199	0.497	0.336	0.000261191016896885	RAB30
Rejected	34	8.39309670838243e-09	0.352090852153404	0.768	0.618	0.00028148767740573	SPIDR
Rejected	34	8.39808980369342e-09	0.460818417996537	0.926	0.856	0.00028165513583627	RORA
Rejected	34	9.57071563292033e-09	0.565949260231002	0.689	0.56	0.000320982660896882	IMMP2L
Rejected	34	1.00016699571994e-08	0.255007764220105	0.932	0.769	0.000335436007024553	KAT6A
Rejected	34	1.04207473877403e-08	0.453442959084797	0.923	0.861	0.000349491025890033	RNF19A
Rejected	34	1.17618608856789e-08	0.304085604598856	0.369	0.226	0.000394469290383899	NDUFAF2
Rejected	34	1.22529336213833e-08	0.336739666657815	0.806	0.632	0.000410938887793952	SERINC5
Rejected	34	1.29613591606925e-08	0.364758352331682	0.727	0.57	0.000434698063531306	ARHGEF3
Rejected	34	1.51733590079426e-08	0.476969674802498	0.746	0.588	0.00050888411440838	GABPB1
Rejected	34	1.6083242819295e-08	0.27360059981915	0.885	0.711	0.000539399797673516	GPRIN3
Rejected	34	1.63469877438097e-08	0.296571919324827	0.467	0.304	0.000548245274951891	CDK8
Rejected	34	1.85019857950628e-08	0.339236236707671	0.601	0.441	0.000620519599594816	ST8SIA4
Rejected	34	1.90627375376337e-08	0.315179539543631	0.314	0.184	0.000639326091537158	AVEN
Rejected	34	1.97416919105068e-08	0.705741759336267	0.227	0.125	0.000662096863294576	LEF1
Rejected	34	2.15445635126389e-08	0.412449041403498	0.505	0.355	0.000722561571086884	SUMF1
Rejected	34	2.28745134116471e-08	0.278396414518656	0.82	0.659	0.000767165430799821	TLE4
Rejected	34	2.31069215361201e-08	0.254097735269811	0.257	0.141	0.000774959934478394	ANKRD26
Rejected	34	2.4010637830459e-08	0.322197664024137	0.765	0.592	0.000805268771557934	ASAP1
Rejected	34	2.44422632087399e-08	0.259942995786029	0.555	0.386	0.000819744623494718	TPD52
Rejected	34	2.49365631423233e-08	0.265383935782083	0.757	0.55	0.000836322454667237	SMURF2
Rejected	34	2.55564448436034e-08	0.306134612854002	0.426	0.276	0.000857112047164771	WDPCP

Rejected	34	2.56794379099741e-08	0.25662872857852	0.568	0.395	0.000861236988624712	PHF14
Rejected	34	2.68300636493103e-08	0.25138032644776	0.41	0.261	0.000899826674670568	TDRD3
Rejected	34	2.7297601626894e-08	0.39354430915578	0.156	0.07	0.00091550696336277	AC139720.1
Rejected	34	2.88049267520201e-08	0.26429432011062	0.421	0.271	0.00096605963340925	IL18RAP
Rejected	34	2.9205724418448e-08	0.31284058290658	0.743	0.565	0.00097950158554591	TTC17
Rejected	34	2.94111040065172e-08	0.32532767051537	0.404	0.267	0.000986389606170575	CDK19
Rejected	34	3.10112087256679e-08	0.48087979832454	0.541	0.406	0.00104005391824145	PPP2R2B
Rejected	34	3.25911595770394e-08	0.26560802844504	0.893	0.725	0.00109304230989475	STAT3
Rejected	34	3.3487609324261e-08	0.25170926050925	0.492	0.328	0.00112310744151707	PPP1R12B
Rejected	34	3.67665529737581e-08	0.25408753148843	0.792	0.618	0.0012330766536339	ITPR2
Rejected	34	4.2812271954881e-08	0.31844375698185	0.617	0.448	0.0014358379768228	TAF3
Rejected	34	4.74166080137803e-08	0.40636115642899	0.694	0.548	0.00159025819956617	MDFIC
Rejected	34	5.05236953001973e-08	0.34872365634187	0.74	0.59	0.00169446369297802	PRKCA
Rejected	34	5.1258647485567e-08	0.25646935806065	0.24	0.129	0.00171911251937095	KANSL1L
Rejected	34	5.3670575632757e-08	0.26187791651093	0.601	0.438	0.0018000037655714	DIP2B
Rejected	34	5.37107573154278e-08	0.26577648994530	0.227	0.121	0.00180135137884482	AC027097.2
Rejected	34	5.43258854457917e-08	0.26348242573832	0.587	0.422	0.00182198154608096	ANKIB1
Rejected	34	5.54826295825752e-08	0.26700615394692	0.448	0.293	0.00186077643094041	RHBDD1
Rejected	34	5.71223024679032e-08	0.25885801710587	0.434	0.279	0.00191576778016854	SP4
Rejected	34	5.98970866768688e-08	0.34515730147014	0.53	0.374	0.00200882849296883	HIPK2
Rejected	34	6.23002628078193e-08	0.26538958891795	0.5	0.347	0.00208942621404864	PRIM2
Rejected	34	6.37663795797249e-08	0.25857594217445	0.959	0.898	0.00213859683834481	ARIH1
Rejected	34	6.39933817805969e-08	0.33780498695259	0.626	0.463	0.00214621003815766	PHF21A
Rejected	34	6.47414688783854e-08	0.34141546690154	0.754	0.625	0.00217129938324329	DNAJC1
Rejected	34	6.53168614145831e-08	0.263180975111449	0.292	0.168	0.00219059689812229	PLCXD2
Rejected	34	7.3254569669487e-08	0.29692156209037	0.287	0.167	0.00245681175757526	HIBADH
Rejected	34	7.44235401996609e-08	0.28403686749678	0.473	0.321	0.00249601669121623	UBE2E1
Rejected	34	7.57393654010089e-08	0.48110226667213	0.443	0.301	0.00254014683681904	CRADD
Rejected	34	7.95176443806403e-08	0.25150068619922	0.658	0.491	0.00266686275723791	CUX1
Rejected	34	8.12907992951312e-08	0.25985566208691	0.918	0.768	0.00272633082676011	HIVEP2
Rejected	34	8.83593221224176e-08	0.30891230248249	0.844	0.694	0.00296339494534164	MAP3K2

Rejected	34	8.8979242914246e-08	0.25887360511136	0.954	0.813	0.00298418584885798	CCNH
Rejected	34	1.05073669383727e-07	0.287793954779479	0.284	0.163	0.00352396072379144	MBNL3
Rejected	34	1.10438118230441e-07	0.262553204010229	0.847	0.675	0.00370387360921253	EPB41
Rejected	34	1.2627621283057e-07	0.326939419445742	0.301	0.182	0.00423505162591166	ENOX2
Rejected	34	1.62311942810735e-07	0.278733679564934	0.224	0.12	0.00544361793798642	UBE3D
Rejected	34	1.68503991437836e-07	0.264526626180346	0.363	0.23	0.00565128686484216	RFX3-AS1
Rejected	34	1.78755002080246e-07	0.338551250307391	0.634	0.48	0.00599508525976728	GNAQ
Rejected	34	1.92587932568041e-07	0.33220420016797	0.164	0.08	0.00645901408246697	ST8SIA1
Rejected	34	2.16356784531069e-07	0.382395387532207	0.194	0.099	0.00725617383960298	DLGAP1
Rejected	34	2.37947672904173e-07	0.262285594113453	0.776	0.593	0.00798028905386016	NCOA3
Rejected	34	2.42555959879469e-07	0.280489047601739	0.541	0.391	0.00813484178243761	PDSS2
Rejected	34	2.99796714346334e-07	0.252296880010618	0.508	0.337	0.0100545822057474	NDRG1
Rejected	34	3.08508059366961e-07	0.257672917260926	0.79	0.642	0.0103467432950491	EIF4G3
Rejected	34	3.53883817374193e-07	0.258515096042381	0.448	0.3	0.0118685554670957	DOPEY1
Rejected	34	3.57000506904593e-07	0.305833023939307	0.489	0.346	0.0119730830005662	VAV3
Rejected	34	4.12981526063304e-07	0.539765188438277	0.705	0.549	0.0138505744211111	WWOX
Rejected	34	4.16485263612309e-07	0.304311195402803	0.582	0.418	0.0139680827710296	PVT1
Rejected	34	4.19376412729113e-07	0.684633423865414	0.508	0.386	0.014065046130109	AOAH
Rejected	34	4.49240687393687e-07	0.36023567726638	0.667	0.511	0.0150666341738095	FOXN2
Rejected	34	4.52334901642681e-07	0.361238050016048	0.781	0.638	0.0151704079312922	HIF1A
Rejected	34	8.13442223734536e-07	0.272075783226772	0.391	0.258	0.0272812252996089	NUBPL
Rejected	34	1.02676536065921e-06	0.271288719951531	0.773	0.616	0.0344356566657887	ZNF831
Rejected	34	1.05462806282572e-06	0.316118841877332	0.577	0.433	0.0353701159710491	BTBD11
Rejected	34	1.16498314142914e-06	0.277074710480568	0.803	0.657	0.0390712045972506	TNIK
Rejected	34	1.27109253068873e-06	0.314335000051545	0.268	0.159	0.0426299012942385	SVIL
Rejected	34	1.27610663964772e-06	0.259377999693255	0.391	0.264	0.0427980644805051	GFOD1
Rejected	34	1.48846921923959e-06	0.259742897644156	0.139	0.068	0.0499202806748572	C9orf84

Supplemental Digital Conte

Transplan	Day	Pathway identifie
Successfu	0	R-HSA-156902
Successfu	0	R-HSA-156842
Successfu	0	R-HSA-975956
Successfu	0	R-HSA-72689
Successfu	0	R-HSA-1799339
Successfu	0	R-HSA-192823
Successfu	0	R-HSA-156827
Successfu	0	R-HSA-72706
Successfu	0	R-HSA-72649
Successfu	0	R-HSA-72702
Successfu	0	R-HSA-6791226
Successfu	0	R-HSA-927802
Successfu	0	R-HSA-975957
Successfu	0	R-HSA-9735869
Successfu	0	R-HSA-72613
Successfu	0	R-HSA-72764
Successfu	0	R-HSA-72737
Successfu	0	R-HSA-9711097
Successfu	0	R-HSA-8868773
Successfu	0	R-HSA-72766
Successfu	6	R-HSA-975956
Successfu	6	R-HSA-9735869
Successfu	6	R-HSA-72689
Successfu	6	R-HSA-192823
Successfu	6	R-HSA-1799339
Successfu	6	R-HSA-72706
Successfu	6	R-HSA-156827
Successfu	6	R-HSA-72649
Successfu	6	R-HSA-72702

Successfu	6	R-HSA-156842
Successfu	6	R-HSA-6791226
Successfu	6	R-HSA-927802
Successfu	6	R-HSA-156902
Successfu	6	R-HSA-975957
Successfu	6	R-HSA-72613
Successfu	6	R-HSA-72764
Successfu	6	R-HSA-72737
Successfu	6	R-HSA-8868773
Successfu	6	R-HSA-72766
Successfu	6	R-HSA-72695
Successfu	12	R-HSA-5357956
Successfu	12	R-HSA-73887
Successfu	12	R-HSA-75893
Successfu	12	R-HSA-9648895
Successfu	12	R-HSA-168643
Successfu	12	R-HSA-168638
Successfu	12	R-HSA-1280215
Successfu	12	R-HSA-451927
Successfu	12	R-HSA-5357905
Successfu	12	R-HSA-449147
Successfu	12	R-HSA-5661231
Successfu	12	R-HSA-9031628
Successfu	12	R-HSA-168164
Successfu	12	R-HSA-8877330
Successfu	12	R-HSA-937061
Successfu	12	R-HSA-166166
Successfu	12	R-HSA-5660526
Successfu	12	R-HSA-168256
Successfu	12	R-HSA-8950505
Successfu	12	R-HSA-187037

Successfu	19	R-HSA-975956
Successfu	19	R-HSA-72689
Successfu	19	R-HSA-192823
Successfu	19	R-HSA-1799339
Successfu	19	R-HSA-72706
Successfu	19	R-HSA-156827
Successfu	19	R-HSA-72649
Successfu	19	R-HSA-72702
Successfu	19	R-HSA-6791226
Successfu	19	R-HSA-927802
Successfu	19	R-HSA-156902
Successfu	19	R-HSA-72764
Successfu	19	R-HSA-975957
Successfu	19	R-HSA-156842
Successfu	19	R-HSA-9735869
Successfu	19	R-HSA-72613
Successfu	19	R-HSA-72737
Successfu	19	R-HSA-8868773
Successfu	19	R-HSA-72766
Successfu	19	R-HSA-72312
Successfu	22	R-HSA-449147
Successfu	22	R-HSA-168256
Successfu	22	R-HSA-1280215
Successfu	22	R-HSA-373755
Successfu	22	R-HSA-6785807
Successfu	22	R-HSA-416700
Successfu	22	R-HSA-1266695
Successfu	22	R-HSA-202427
Successfu	22	R-HSA-202403
Successfu	22	R-HSA-9680350
Successfu	22	R-HSA-9607240

Successfu	22	R-HSA-5632927
Successfu	22	R-HSA-1433557
Successfu	22	R-HSA-5632928
Successfu	22	R-HSA-8950505
Successfu	22	R-HSA-5423599
Successfu	22	R-HSA-9020591
Successfu	22	R-HSA-6783783
Successfu	22	R-HSA-447115
Successfu	22	R-HSA-9706377
Successfu	26	R-HSA-5661231
Successfu	26	R-HSA-5660526
Successfu	26	R-HSA-9013149
Successfu	26	R-HSA-428359
Successfu	26	R-HSA-1368082
Successfu	26	R-HSA-4839726
Successfu	26	R-HSA-3247509
Successfu	26	R-HSA-5607763
Successfu	26	R-HSA-9012999
Successfu	26	R-HSA-8934593
Successfu	26	R-HSA-2025928
Successfu	26	R-HSA-6785470
Successfu	26	R-HSA-4086398
Successfu	26	R-HSA-426496
Successfu	26	R-HSA-442982
Successfu	26	R-HSA-9013148
Successfu	26	R-HSA-8868766
Successfu	26	R-HSA-194315
Successfu	26	R-HSA-9716542
Successfu	26	R-HSA-8936459
Successfu	33	R-HSA-428359
Successfu	33	R-HSA-3247509

Successfu	33	R-HSA-4839726
Successfu	33	R-HSA-2122947
Successfu	33	R-HSA-2894858
Successfu	33	R-HSA-2644606
Successfu	33	R-HSA-2894862
Successfu	33	R-HSA-2644602
Successfu	33	R-HSA-2644603
Successfu	33	R-HSA-1368082
Successfu	33	R-HSA-5663202
Successfu	33	R-HSA-5621575
Successfu	33	R-HSA-9614399
Successfu	33	R-HSA-9705683
Successfu	33	R-HSA-9694516
Successfu	33	R-HSA-9005891
Successfu	33	R-HSA-9697154
Successfu	33	R-HSA-9005895
Successfu	33	R-HSA-9675151
Successfu	33	R-HSA-1169408
Successfu	59	R-HSA-5656169
Successfu	59	R-HSA-110313
Successfu	59	R-HSA-73893
Successfu	59	R-HSA-114608
Successfu	59	R-HSA-76005
Successfu	59	R-HSA-76002
Successfu	59	R-HSA-73894
Successfu	59	R-HSA-109582
Successfu	64	R-HSA-216083
Successfu	64	R-HSA-1474228
Successfu	64	R-HSA-8939246
Successfu	64	R-HSA-428359
Successfu	64	R-HSA-381426

Successfu	64	R-HSA-1474244
Successfu	64	R-HSA-8948216
Successfu	64	R-HSA-1442490
Successfu	64	R-HSA-8941333
Successfu	64	R-HSA-2022090
Successfu	64	R-HSA-4419969
Successfu	64	R-HSA-1650814
Successfu	64	R-HSA-8957275
Successfu	64	R-HSA-352238
Successfu	64	R-HSA-8940973
Successfu	64	R-HSA-3000171
Successfu	64	R-HSA-2214320
Successfu	64	R-HSA-8951911
Successfu	64	R-HSA-1474290
Successfu	64	R-HSA-375165
Successfu	191	R-HSA-400253
Successfu	191	R-HSA-5663202
Successfu	191	R-HSA-4839726
Successfu	191	R-HSA-3247509
Successfu	191	R-HSA-383280
Successfu	191	R-HSA-1660499
Successfu	191	R-HSA-1483255
Successfu	191	R-HSA-9022692
Successfu	191	R-HSA-8939246
Successfu	191	R-HSA-8986944
Successfu	191	R-HSA-9013149
Successfu	191	R-HSA-6802957
Successfu	191	R-HSA-3214841
Successfu	191	R-HSA-5607763
Successfu	191	R-HSA-1368082
Successfu	191	R-HSA-428359

Successfu	191	R-HSA-8878171
Successfu	191	R-HSA-9006335
Successfu	191	R-HSA-9022537
Successfu	191	R-HSA-350054
Recoverec	0	R-HSA-975956
Recoverec	0	R-HSA-72689
Recoverec	0	R-HSA-192823
Recoverec	0	R-HSA-1799339
Recoverec	0	R-HSA-72706
Recoverec	0	R-HSA-156827
Recoverec	0	R-HSA-6791226
Recoverec	0	R-HSA-927802
Recoverec	0	R-HSA-156902
Recoverec	0	R-HSA-3371568
Recoverec	0	R-HSA-975957
Recoverec	0	R-HSA-156842
Recoverec	0	R-HSA-72613
Recoverec	0	R-HSA-72764
Recoverec	0	R-HSA-72737
Recoverec	0	R-HSA-8868773
Recoverec	0	R-HSA-72312
Recoverec	0	R-HSA-9735869
Recoverec	0	R-HSA-2408557
Recoverec	0	R-HSA-72766
Recoverec	13	R-HSA-9735869
Recoverec	13	R-HSA-975956
Recoverec	13	R-HSA-72689
Recoverec	13	R-HSA-1799339
Recoverec	13	R-HSA-192823
Recoverec	13	R-HSA-72706
Recoverec	13	R-HSA-156827

Recoverec	13	R-HSA-72649
Recoverec	13	R-HSA-72702
Recoverec	13	R-HSA-156842
Recoverec	13	R-HSA-6791226
Recoverec	13	R-HSA-927802
Recoverec	13	R-HSA-156902
Recoverec	13	R-HSA-975957
Recoverec	13	R-HSA-72613
Recoverec	13	R-HSA-72764
Recoverec	13	R-HSA-72737
Recoverec	13	R-HSA-8868773
Recoverec	13	R-HSA-72312
Recoverec	13	R-HSA-72695
Recoverec	20	R-HSA-8868766
Recoverec	20	R-HSA-6785470
Recoverec	20	R-HSA-72306
Recoverec	20	R-HSA-163200
Recoverec	20	R-HSA-72312
Recoverec	20	R-HSA-6799198
Recoverec	20	R-HSA-611105
Recoverec	20	R-HSA-1428517
Recoverec	20	R-HSA-389359
Recoverec	20	R-HSA-174490
Recoverec	20	R-HSA-1475029
Recoverec	20	R-HSA-174495
Recoverec	20	R-HSA-2892247
Recoverec	20	R-HSA-389513
Recoverec	20	R-HSA-9006925
Recoverec	20	R-HSA-175474
Recoverec	20	R-HSA-6796648
Recoverec	20	R-HSA-388841

Recoverec	20	R-HSA-9670439
Recoverec	20	R-HSA-9669938
Recoverec	26	R-HSA-975956
Recoverec	26	R-HSA-72689
Recoverec	26	R-HSA-192823
Recoverec	26	R-HSA-1799339
Recoverec	26	R-HSA-72706
Recoverec	26	R-HSA-72649
Recoverec	26	R-HSA-72702
Recoverec	26	R-HSA-6791226
Recoverec	26	R-HSA-927802
Recoverec	26	R-HSA-3371568
Recoverec	26	R-HSA-156902
Recoverec	26	R-HSA-975957
Recoverec	26	R-HSA-156827
Recoverec	26	R-HSA-3371571
Recoverec	26	R-HSA-72764
Recoverec	26	R-HSA-3371511
Recoverec	26	R-HSA-72613
Recoverec	26	R-HSA-72737
Recoverec	26	R-HSA-3371556
Recoverec	26	R-HSA-3371453
Recoverec	33	R-HSA-6785807
Recoverec	33	R-HSA-428359
Recoverec	33	R-HSA-449147
Recoverec	33	R-HSA-1280215
Recoverec	33	R-HSA-6804115
Recoverec	33	R-HSA-9024446
Recoverec	33	R-HSA-6791312
Recoverec	33	R-HSA-9648895
Recoverec	33	R-HSA-9006931

Recoverec	33	R-HSA-5603029
Recoverec	33	R-HSA-3700989
Recoverec	33	R-HSA-209560
Recoverec	33	R-HSA-381183
Recoverec	33	R-HSA-168928
Recoverec	33	R-HSA-73887
Recoverec	33	R-HSA-9029558
Recoverec	33	R-HSA-381033
Recoverec	33	R-HSA-8877330
Recoverec	33	R-HSA-6803211
Recoverec	33	R-HSA-1810476
Recoverec	40	R-HSA-6785470
Recoverec	40	R-HSA-8868766
Recoverec	40	R-HSA-5661231
Recoverec	40	R-HSA-5660526
Recoverec	40	R-HSA-163200
Recoverec	40	R-HSA-611105
Recoverec	40	R-HSA-202433
Recoverec	40	R-HSA-193648
Recoverec	40	R-HSA-1855204
Recoverec	40	R-HSA-72306
Recoverec	40	R-HSA-2022377
Recoverec	40	R-HSA-6799198
Recoverec	40	R-HSA-5250913
Recoverec	40	R-HSA-9013106
Recoverec	40	R-HSA-1428517
Recoverec	40	R-HSA-204998
Recoverec	40	R-HSA-3214815
Recoverec	40	R-HSA-73854
Recoverec	40	R-HSA-8964043
Recoverec	40	R-HSA-9617629

Recoverec	47	R-HSA-8939246
Recoverec	47	R-HSA-428359
Recoverec	47	R-HSA-9022692
Recoverec	47	R-HSA-8986944
Recoverec	47	R-HSA-8941333
Recoverec	47	R-HSA-9005891
Recoverec	47	R-HSA-9675151
Recoverec	47	R-HSA-9005895
Recoverec	47	R-HSA-9697154
Recoverec	47	R-HSA-8878171
Recoverec	47	R-HSA-9022535
Recoverec	47	R-HSA-975577
Recoverec	47	R-HSA-9022537
Recoverec	47	R-HSA-164944
Recoverec	47	R-HSA-9022534
Recoverec	47	R-HSA-400253
Recoverec	47	R-HSA-9707616
Recoverec	47	R-HSA-164952
Recoverec	47	R-HSA-6811438
Recoverec	47	R-HSA-8941332
Recoverec	98	R-HSA-975956
Recoverec	98	R-HSA-72689
Recoverec	98	R-HSA-192823
Recoverec	98	R-HSA-1799339
Recoverec	98	R-HSA-72706
Recoverec	98	R-HSA-72649
Recoverec	98	R-HSA-72702
Recoverec	98	R-HSA-927802
Recoverec	98	R-HSA-156902
Recoverec	98	R-HSA-975957
Recoverec	98	R-HSA-156827

Recoverec	98	R-HSA-72764
Recoverec	98	R-HSA-72613
Recoverec	98	R-HSA-72737
Recoverec	98	R-HSA-156842
Recoverec	98	R-HSA-6791226
Recoverec	98	R-HSA-9735869
Recoverec	98	R-HSA-72695
Recoverec	98	R-HSA-72662
Recoverec	98	R-HSA-2408557
Recoverec	121	R-HSA-1474244
Recoverec	121	R-HSA-1474228
Recoverec	121	R-HSA-8948216
Recoverec	121	R-HSA-2022090
Recoverec	121	R-HSA-216083
Recoverec	121	R-HSA-3000171
Recoverec	121	R-HSA-1442490
Recoverec	121	R-HSA-2173782
Recoverec	121	R-HSA-1474290
Recoverec	121	R-HSA-1650814
Recoverec	121	R-HSA-8957275
Recoverec	121	R-HSA-3000178
Recoverec	121	R-HSA-381426
Recoverec	121	R-HSA-375165
Recoverec	121	R-HSA-419037
Recoverec	121	R-HSA-2243919
Recoverec	121	R-HSA-3000157
Recoverec	121	R-HSA-2214320
Recoverec	121	R-HSA-109582
Recoverec	121	R-HSA-5690714
Recoverec	183	R-HSA-1483255
Recoverec	183	R-HSA-1306955

Recoverec	183	R-HSA-164952
Recoverec	183	R-HSA-1660516
Recoverec	183	R-HSA-111453
Recoverec	183	R-HSA-6806834
Recoverec	183	R-HSA-1358803
Recoverec	183	R-HSA-8949664
Recoverec	183	R-HSA-512988
Recoverec	183	R-HSA-8847993
Recoverec	183	R-HSA-6785631
Recoverec	183	R-HSA-164938
Recoverec	183	R-HSA-1963642
Recoverec	183	R-HSA-1660499
Recoverec	183	R-HSA-8949215
Recoverec	183	R-HSA-186797
Recoverec	183	R-HSA-2173788
Recoverec	183	R-HSA-3304347
Recoverec	183	R-HSA-3315487
Recoverec	183	R-HSA-3311021
Rejected	0	R-HSA-975956
Rejected	0	R-HSA-72689
Rejected	0	R-HSA-192823
Rejected	0	R-HSA-1799339
Rejected	0	R-HSA-72706
Rejected	0	R-HSA-156827
Rejected	0	R-HSA-72649
Rejected	0	R-HSA-72702
Rejected	0	R-HSA-156842
Rejected	0	R-HSA-6791226
Rejected	0	R-HSA-927802
Rejected	0	R-HSA-156902
Rejected	0	R-HSA-975957

Rejected	0	R-HSA-9735869
Rejected	0	R-HSA-72613
Rejected	0	R-HSA-72764
Rejected	0	R-HSA-72737
Rejected	0	R-HSA-8868773
Rejected	0	R-HSA-72312
Rejected	0	R-HSA-9633012
Rejected	6	R-HSA-975956
Rejected	6	R-HSA-72689
Rejected	6	R-HSA-192823
Rejected	6	R-HSA-156827
Rejected	6	R-HSA-1799339
Rejected	6	R-HSA-72706
Rejected	6	R-HSA-927802
Rejected	6	R-HSA-975957
Rejected	6	R-HSA-72649
Rejected	6	R-HSA-72702
Rejected	6	R-HSA-156842
Rejected	6	R-HSA-6791226
Rejected	6	R-HSA-156902
Rejected	6	R-HSA-9735869
Rejected	6	R-HSA-72613
Rejected	6	R-HSA-72764
Rejected	6	R-HSA-72737
Rejected	6	R-HSA-8868773
Rejected	6	R-HSA-72766
Rejected	6	R-HSA-72312
Rejected	14	R-HSA-1280215
Rejected	14	R-HSA-168256
Rejected	14	R-HSA-913531
Rejected	14	R-HSA-909733

Rejected	14	R-HSA-449147
Rejected	14	R-HSA-447115
Rejected	14	R-HSA-9020591
Rejected	14	R-HSA-8950505
Rejected	14	R-HSA-8877330
Rejected	14	R-HSA-2559584
Rejected	14	R-HSA-8856828
Rejected	14	R-HSA-2559583
Rejected	14	R-HSA-3928662
Rejected	14	R-HSA-140342
Rejected	14	R-HSA-5668541
Rejected	14	R-HSA-5357801
Rejected	14	R-HSA-8878171
Rejected	14	R-HSA-194315
Rejected	14	R-HSA-109581
Rejected	14	R-HSA-202403
Rejected	20	R-HSA-975956
Rejected	20	R-HSA-72689
Rejected	20	R-HSA-192823
Rejected	20	R-HSA-1799339
Rejected	20	R-HSA-72706
Rejected	20	R-HSA-156827
Rejected	20	R-HSA-72649
Rejected	20	R-HSA-72702
Rejected	20	R-HSA-6791226
Rejected	20	R-HSA-927802
Rejected	20	R-HSA-156902
Rejected	20	R-HSA-975957
Rejected	20	R-HSA-156842
Rejected	20	R-HSA-72613
Rejected	20	R-HSA-72764

Rejected	20	R-HSA-72737
Rejected	20	R-HSA-8868773
Rejected	20	R-HSA-9735869
Rejected	20	R-HSA-72695
Rejected	20	R-HSA-72662
Rejected	23	R-HSA-9648895
Rejected	23	R-HSA-9029569
Rejected	23	R-HSA-8950505
Rejected	23	R-HSA-9020591
Rejected	23	R-HSA-9024446
Rejected	23	R-HSA-447115
Rejected	23	R-HSA-5218900
Rejected	23	R-HSA-449147
Rejected	23	R-HSA-381183
Rejected	23	R-HSA-381033
Rejected	23	R-HSA-1810476
Rejected	23	R-HSA-140534
Rejected	23	R-HSA-72163
Rejected	23	R-HSA-5210891
Rejected	23	R-HSA-9616222
Rejected	23	R-HSA-1606322
Rejected	23	R-HSA-72172
Rejected	23	R-HSA-9725371
Rejected	23	R-HSA-2173788
Rejected	23	R-HSA-5357769
Rejected	27	R-HSA-975956
Rejected	27	R-HSA-72689
Rejected	27	R-HSA-192823
Rejected	27	R-HSA-1799339
Rejected	27	R-HSA-72706
Rejected	27	R-HSA-72649

Rejected	27	R-HSA-72702
Rejected	27	R-HSA-927802
Rejected	27	R-HSA-156902
Rejected	27	R-HSA-975957
Rejected	27	R-HSA-156827
Rejected	27	R-HSA-72764
Rejected	27	R-HSA-72613
Rejected	27	R-HSA-72737
Rejected	27	R-HSA-156842
Rejected	27	R-HSA-6791226
Rejected	27	R-HSA-72766
Rejected	27	R-HSA-9735869
Rejected	27	R-HSA-72695
Rejected	27	R-HSA-72662
Rejected	30	R-HSA-4839726
Rejected	30	R-HSA-3247509
Rejected	30	R-HSA-8939246
Rejected	30	R-HSA-9022692
Rejected	30	R-HSA-428359
Rejected	30	R-HSA-2032785
Rejected	30	R-HSA-3214841
Rejected	30	R-HSA-5620916
Rejected	30	R-HSA-9022534
Rejected	30	R-HSA-2980766
Rejected	30	R-HSA-199992
Rejected	30	R-HSA-397795
Rejected	30	R-HSA-8939243
Rejected	30	R-HSA-9022537
Rejected	30	R-HSA-8963901
Rejected	30	R-HSA-9707616
Rejected	30	R-HSA-392451

Rejected	30	R-HSA-164944
Rejected	30	R-HSA-5621575
Rejected	30	R-HSA-9012999
Rejected	34	R-HSA-8939246
Rejected	34	R-HSA-383280
Rejected	34	R-HSA-4839726
Rejected	34	R-HSA-3247509
Rejected	34	R-HSA-8986944
Rejected	34	R-HSA-8878171
Rejected	34	R-HSA-8939243
Rejected	34	R-HSA-8941333
Rejected	34	R-HSA-2151201
Rejected	34	R-HSA-1660499
Rejected	34	R-HSA-8941284
Rejected	34	R-HSA-428359
Rejected	34	R-HSA-400253
Rejected	34	R-HSA-9012999
Rejected	34	R-HSA-350054
Rejected	34	R-HSA-8951911
Rejected	34	R-HSA-194315
Rejected	34	R-HSA-9022692
Rejected	34	R-HSA-9716542
Rejected	34	R-HSA-9707616

nt, Table S3

Pathway name (only top 20 per transplant and per day is shown)	#Entities foun
Peptide chain elongation	88
Eukaryotic Translation Elongation	90
Nonsense Mediated Decay (NMD) independent of the Exon Junction Complex (EJC)	86
Formation of a pool of free 40S subunits	88
SRP-dependent cotranslational protein targeting to membrane	90
Viral mRNA Translation	86
L13a-mediated translational silencing of Ceruloplasmin expression	88
GTP hydrolysis and joining of the 60S ribosomal subunit	88
Translation initiation complex formation	36
Ribosomal scanning and start codon recognition	36
Major pathway of rRNA processing in the nucleolus and cytosol	86
Nonsense-Mediated Decay (NMD)	87
Nonsense Mediated Decay (NMD) enhanced by the Exon Junction Complex (EJC)	87
SARS-CoV-1 modulates host translation machinery	35
Eukaryotic Translation Initiation	88
Eukaryotic Translation Termination	86
Cap-dependent Translation Initiation	88
Cellular response to starvation	89
rRNA processing in the nucleus and cytosol	86
Translation	98
Nonsense Mediated Decay (NMD) independent of the Exon Junction Complex (EJC)	69
SARS-CoV-1 modulates host translation machinery	28
Formation of a pool of free 40S subunits	72
Viral mRNA Translation	69
SRP-dependent cotranslational protein targeting to membrane	72
GTP hydrolysis and joining of the 60S ribosomal subunit	72
L13a-mediated translational silencing of Ceruloplasmin expression	72
Translation initiation complex formation	29
Ribosomal scanning and start codon recognition	29

Eukaryotic Translation Elongation	72
Major pathway of rRNA processing in the nucleolus and cytosol	70
Nonsense-Mediated Decay (NMD)	70
Peptide chain elongation	70
Nonsense Mediated Decay (NMD) enhanced by the Exon Junction Complex (EJC)	70
Eukaryotic Translation Initiation	72
Eukaryotic Translation Termination	69
Cap-dependent Translation Initiation	72
rRNA processing in the nucleus and cytosol	70
Translation	80
Formation of the ternary complex, and subsequently, the 43S complex	29
TNFR1-induced NF-kappa-B signaling pathway	4
Death Receptor Signaling	6
TNF signaling	4
Response of EIF2AK1 (HRI) to heme deficiency	3
Nucleotide-binding domain, leucine rich repeat containing receptor (NLR) signaling pathways	4
NOD1/2 Signaling Pathway	3
Cytokine Signaling in Immune system	14
Interleukin-2 family signaling	3
Regulation of TNFR1 signaling	3
Signaling by Interleukins	10
Metallothioneins bind metals	2
NGF-stimulated transcription	3
Toll Like Receptor 3 (TLR3) Cascade	4
RUNX1 and FOXP3 control the development of regulatory T lymphocytes (Tregs)	2
TRIF(TICAM1)-mediated TLR4 signaling	4
MyD88-independent TLR4 cascade	4
Response to metal ions	2
Immune System	24
Gene and protein expression by JAK-STAT signaling after Interleukin-12 stimulation	3
Signaling by NTRK1 (TRKA)	4

Nonsense Mediated Decay (NMD) independent of the Exon Junction Complex (EJC)	72
Formation of a pool of free 40S subunits	72
Viral mRNA Translation	72
SRP-dependent cotranslational protein targeting to membrane	73
GTP hydrolysis and joining of the 60S ribosomal subunit	72
L13a-mediated translational silencing of Ceruloplasmin expression	72
Translation initiation complex formation	30
Ribosomal scanning and start codon recognition	30
Major pathway of rRNA processing in the nucleolus and cytosol	72
Nonsense-Mediated Decay (NMD)	72
Peptide chain elongation	73
Eukaryotic Translation Termination	73
Nonsense Mediated Decay (NMD) enhanced by the Exon Junction Complex (EJC)	72
Eukaryotic Translation Elongation	73
SARS-CoV-1 modulates host translation machinery	31
Eukaryotic Translation Initiation	72
Cap-dependent Translation Initiation	72
rRNA processing in the nucleus and cytosol	73
Translation	76
rRNA processing	73
Signaling by Interleukins	9
Immune System	15
Cytokine Signaling in Immune system	10
Semaphorin interactions	4
Interleukin-4 and Interleukin-13 signaling	4
Other semaphorin interactions	2
Interleukin-7 signaling	2
Phosphorylation of CD3 and TCR zeta chains	2
TCR signaling	3
Signaling by CSF1 (M-CSF) in myeloid cells	2
FLT3 Signaling	2

Defective Mismatch Repair Associated With MSH3	1
Signaling by SCF-KIT	2
Defective Mismatch Repair Associated With MSH2	1
Gene and protein expression by JAK-STAT signaling after Interleukin-12 stimulation	2
Diseases of Mismatch Repair (MMR)	1
Interleukin-12 signaling	2
Interleukin-10 signaling	2
Interleukin-12 family signaling	2
FLT3 signaling by CBL mutants	1
Metallothioneins bind metals	8
Response to metal ions	8
RAC1 GTPase cycle	19
Insulin-like Growth Factor-2 mRNA Binding Proteins (IGF2BPs/IMPs/VICKZs) bind RNA	5
RORA activates gene expression	6
Chromatin organization	20
Chromatin modifying enzymes	20
CLEC7A (Dectin-1) induces NFAT activation	5
RHO GTPase cycle	29
Regulation of RUNX1 Expression and Activity	6
Calcineurin activates NFAT	4
tRNA processing in the mitochondrion	7
Ca2+ pathway	9
Post-transcriptional silencing by small RNAs	3
Ras activation upon Ca2+ influx through NMDA receptor	5
CDC42 GTPase cycle	13
rRNA processing in the mitochondrion	6
Signaling by Rho GTPases	37
Signaling by Rho GTPases, Miro GTPases and RHOBTB3	37
RUNX1 regulates genes involved in megakaryocyte differentiation and platelet function	8
Insulin-like Growth Factor-2 mRNA Binding Proteins (IGF2BPs/IMPs/VICKZs) bind RNA	5
Chromatin modifying enzymes	13

Chromatin organization	13
NOTCH1 Intracellular Domain Regulates Transcription	6
Signaling by NOTCH1 HD+PEST Domain Mutants in Cancer	6
Constitutive Signaling by NOTCH1 PEST Domain Mutants	6
Constitutive Signaling by NOTCH1 HD+PEST Domain Mutants	6
Signaling by NOTCH1 PEST Domain Mutants in Cancer	6
Signaling by NOTCH1 in Cancer	6
RORA activates gene expression	4
Diseases of signal transduction by growth factor receptors and second messengers	18
CD209 (DC-SIGN) signaling	4
Regulation of localization of FOXO transcription factors	3
SARS-CoV-2-host interactions	11
SARS-CoV-2 Infection	14
Loss of function of MECP2 in Rett syndrome	3
Disorders of Nervous System Development	3
Pervasive developmental disorders	3
Disorders of Developmental Biology	3
ISG15 antiviral mechanism	6
Termination of translesion DNA synthesis	1
Translesion synthesis by Y family DNA polymerases bypasses lesions on DNA template	1
DNA Damage Bypass	1
Platelet degranulation	1
Response to elevated platelet cytosolic Ca2+	1
Platelet activation, signaling and aggregation	1
DNA Repair	1
Hemostasis	1
Integrin cell surface interactions	10
Degradation of the extracellular matrix	12
RUNX1 regulates transcription of genes involved in differentiation of myeloid cells	5
Insulin-like Growth Factor-2 mRNA Binding Proteins (IGF2BPs/IMPs/VICKZs) bind RNA	5
Regulation of Insulin-like Growth Factor (IGF) transport and uptake by Insulin-like Growth Factor Binding Proteins (I(	10

Extracellular matrix organization	15
Collagen chain trimerization	6
Collagen degradation	7
RUNX2 regulates genes involved in differentiation of myeloid cells	3
Assembly of collagen fibrils and other multimeric structures	6
Depolymerization of the Nuclear Lamina	4
Collagen biosynthesis and modifying enzymes	6
Post-translational protein phosphorylation	7
Breakdown of the nuclear lamina	2
RUNX2 regulates osteoblast differentiation	4
Non-integrin membrane-ECM interactions	5
Anchoring fibril formation	3
RUNX3 regulates RUNX1-mediated transcription	2
Collagen formation	6
NCAM signaling for neurite out-growth	5
Circadian Clock	24
Diseases of signal transduction by growth factor receptors and second messengers	59
Chromatin organization	37
Chromatin modifying enzymes	37
Nuclear Receptor transcription pathway	19
Synthesis of PIPs at the plasma membrane	16
PI Metabolism	21
Regulation of MECP2 expression and activity	11
RUNX1 regulates transcription of genes involved in differentiation of myeloid cells	6
Transcriptional Regulation by MECP2	17
RAC1 GTPase cycle	25
Oncogenic MAPK signaling	15
PKMTs methylate histone lysines	10
CLEC7A (Dectin-1) induces NFAT activation	6
RORA activates gene expression	7
Insulin-like Growth Factor-2 mRNA Binding Proteins (IGF2BPs/IMPs/VICKZs) bind RNA	5

Transcriptional regulation by RUNX1	29
Signaling by Erythropoietin	8
Loss of MECP2 binding ability to the NCoR/SMRT complex	4
Notch-HLH transcription pathway	7
Nonsense Mediated Decay (NMD) independent of the Exon Junction Complex (EJC)	38
Formation of a pool of free 40S subunits	38
Viral mRNA Translation	38
SRP-dependent cotranslational protein targeting to membrane	38
GTP hydrolysis and joining of the 60S ribosomal subunit	38
L13a-mediated translational silencing of Ceruloplasmin expression	38
Major pathway of rRNA processing in the nucleolus and cytosol	38
Nonsense-Mediated Decay (NMD)	38
Peptide chain elongation	38
Attenuation phase	16
Nonsense Mediated Decay (NMD) enhanced by the Exon Junction Complex (EJC)	38
Eukaryotic Translation Elongation	39
Eukaryotic Translation Initiation	38
Eukaryotic Translation Termination	38
Cap-dependent Translation Initiation	38
rRNA processing in the nucleus and cytosol	38
rRNA processing	45
SARS-CoV-1 modulates host translation machinery	16
Selenocysteine synthesis	38
Translation	39
SARS-CoV-1 modulates host translation machinery	29
Nonsense Mediated Decay (NMD) independent of the Exon Junction Complex (EJC)	71
Formation of a pool of free 40S subunits	74
SRP-dependent cotranslational protein targeting to membrane	75
Viral mRNA Translation	71
GTP hydrolysis and joining of the 60S ribosomal subunit	74
L13a-mediated translational silencing of Ceruloplasmin expression	74

Translation initiation complex formation	30
Ribosomal scanning and start codon recognition	30
Eukaryotic Translation Elongation	74
Major pathway of rRNA processing in the nucleolus and cytosol	71
Nonsense-Mediated Decay (NMD)	72
Peptide chain elongation	72
Nonsense Mediated Decay (NMD) enhanced by the Exon Junction Complex (EJC)	72
Eukaryotic Translation Initiation	74
Eukaryotic Translation Termination	71
Cap-dependent Translation Initiation	74
rRNA processing in the nucleus and cytosol	71
rRNA processing	72
Formation of the ternary complex, and subsequently, the 43S complex	30
rRNA processing in the mitochondrion	7
tRNA processing in the mitochondrion	7
tRNA processing	7
Respiratory electron transport, ATP synthesis by chemiosmotic coupling, and heat production by uncoupling proteins	6
rRNA processing	7
Complex I biogenesis	4
Respiratory electron transport	5
The citric acid (TCA) cycle and respiratory electron transport	6
CD28 dependent Vav1 pathway	2
Membrane binding and targetting of GAG proteins	2
Reversible hydration of carbon dioxide	2
Synthesis And Processing Of GAG, GAGPOL Polyproteins	2
POU5F1 (OCT4), SOX2, NANOG activate genes related to proliferation	2
CTLA4 inhibitory signaling	2
Intracellular signaling by second messengers	6
Assembly Of The HIV Virion	2
TP53 Regulates Transcription of DNA Repair Genes	3
Costimulation by the CD28 family	3

Signaling by phosphorylated juxtamembrane, extracellular and kinase domain KIT mutants	2
Signaling by KIT in disease	2
Nonsense Mediated Decay (NMD) independent of the Exon Junction Complex (EJC)	30
Formation of a pool of free 40S subunits	30
Viral mRNA Translation	30
SRP-dependent cotranslational protein targeting to membrane	30
GTP hydrolysis and joining of the 60S ribosomal subunit	30
Translation initiation complex formation	14
Ribosomal scanning and start codon recognition	14
Major pathway of rRNA processing in the nucleolus and cytosol	30
Nonsense-Mediated Decay (NMD)	30
Attenuation phase	19
Peptide chain elongation	30
Nonsense Mediated Decay (NMD) enhanced by the Exon Junction Complex (EJC)	30
L13a-mediated translational silencing of Ceruloplasmin expression	30
HSF1-dependent transactivation	19
Eukaryotic Translation Termination	30
HSF1 activation	16
Eukaryotic Translation Initiation	30
Cap-dependent Translation Initiation	30
Cellular response to heat stress	20
Regulation of HSF1-mediated heat shock response	17
Interleukin-4 and Interleukin-13 signaling	12
Insulin-like Growth Factor-2 mRNA Binding Proteins (IGF2BPs/IMPs/VICKZs) bind RNA	5
Signaling by Interleukins	19
Cytokine Signaling in Immune system	21
TP53 regulates transcription of additional cell cycle genes whose exact role in the p53 pathway remain uncertain	4
NR1H2 and NR1H3-mediated signaling	5
TP53 Regulates Transcription of Cell Cycle Genes	4
Response of EIF2AK1 (HRI) to heme deficiency	3
Signaling by Nuclear Receptors	9

IkBA variant leads to EDA-ID	2
Transcriptional Regulation by TP53	9
NF-kB is activated and signals survival	2
ATF6 (ATF6-alpha) activates chaperone genes	2
DDX58/IFIH1-mediated induction of interferon-alpha/beta	4
Death Receptor Signaling	5
NR1H2 & NR1H3 regulate gene expression linked to lipogenesis	2
ATF6 (ATF6-alpha) activates chaperones	2
RUNX1 and FOXP3 control the development of regulatory T lymphocytes (Tregs)	2
TP53 Regulates Transcription of Death Receptors and Ligands	2
RIP-mediated NFkB activation via ZBP1	2
tRNA processing in the mitochondrion	12
rRNA processing in the mitochondrion	11
Metallothioneins bind metals	7
Response to metal ions	7
Respiratory electron transport, ATP synthesis by chemiosmotic coupling, and heat production by uncoupling proteins	12
Respiratory electron transport	10
Generation of second messenger molecules	6
NRAGE signals death through JNK	7
Synthesis of IP3 and IP4 in the cytosol	5
tRNA processing	12
Metabolism of Angiotensinogen to Angiotensins	4
Complex I biogenesis	6
Positive epigenetic regulation of rRNA expression	7
RHOC GTPase cycle	7
The citric acid (TCA) cycle and respiratory electron transport	14
Cell death signalling via NRAGE, NRIF and NADE	7
HDACs deacetylate histones	6
RNA Polymerase I Promoter Clearance	7
Plasma lipoprotein clearance	5
Regulation of FOXO transcriptional activity by acetylation	3

RUNX1 regulates transcription of genes involved in differentiation of myeloid cells	5
Insulin-like Growth Factor-2 mRNA Binding Proteins (IGF2BPs/IMPs/VICKZs) bind RNA	5
Regulation of MECP2 expression and activity	7
Transcriptional Regulation by MECP2	9
RUNX2 regulates genes involved in differentiation of myeloid cells	3
Loss of function of MECP2 in Rett syndrome	4
Disorders of Developmental Biology	4
Pervasive developmental disorders	4
Disorders of Nervous System Development	4
Transcriptional regulation by RUNX1	14
Loss of phosphorylation of MECP2 at T308	3
N-Glycan antennae elongation	4
Loss of MECP2 binding ability to the NCoR/SMRT complex	3
Nef and signal transduction	3
Loss of MECP2 binding ability to 5hmC-DNA	2
Circadian Clock	8
Heme signaling	6
The role of Nef in HIV-1 replication and disease pathogenesis	4
Intra-Golgi traffic	5
RUNX2 regulates genes involved in cell migration	3
Nonsense Mediated Decay (NMD) independent of the Exon Junction Complex (EJC)	37
Formation of a pool of free 40S subunits	37
Viral mRNA Translation	37
SRP-dependent cotranslational protein targeting to membrane	37
GTP hydrolysis and joining of the 60S ribosomal subunit	37
Translation initiation complex formation	15
Ribosomal scanning and start codon recognition	15
Nonsense-Mediated Decay (NMD)	37
Peptide chain elongation	37
Nonsense Mediated Decay (NMD) enhanced by the Exon Junction Complex (EJC)	37
L13a-mediated translational silencing of Ceruloplasmin expression	37

Eukaryotic Translation Termination	37
Eukaryotic Translation Initiation	37
Cap-dependent Translation Initiation	37
Eukaryotic Translation Elongation	37
Major pathway of rRNA processing in the nucleolus and cytosol	37
SARS-CoV-1 modulates host translation machinery	15
Formation of the ternary complex, and subsequently, the 43S complex	15
Activation of the mRNA upon binding of the cap-binding complex and eIFs, and subsequent binding to 43S	15
Selenocysteine synthesis	37
Extracellular matrix organization	17
Degradation of the extracellular matrix	11
Collagen chain trimerization	7
Assembly of collagen fibrils and other multimeric structures	8
Integrin cell surface interactions	8
Non-integrin membrane-ECM interactions	7
Collagen degradation	7
Binding and Uptake of Ligands by Scavenger Receptors	10
Collagen formation	8
Collagen biosynthesis and modifying enzymes	7
Post-translational protein phosphorylation	8
ECM proteoglycans	7
Regulation of Insulin-like Growth Factor (IGF) transport and uptake by Insulin-like Growth Factor Binding Proteins (I(	8
NCAM signaling for neurite out-growth	6
NCAM1 interactions	5
Crosslinking of collagen fibrils	4
Laminin interactions	4
Anchoring fibril formation	3
Hemostasis	18
CD22 mediated BCR regulation	5
PI Metabolism	6
GRB7 events in ERBB2 signaling	2

The role of Nef in HIV-1 replication and disease pathogenesis	3
Synthesis of PIPs at the early endosome membrane	3
BH3-only proteins associate with and inactivate anti-apoptotic BCL-2 members	2
Signaling by MET	4
Downregulation of ERBB2:ERBB3 signaling	2
Processing of SMDT1	2
Interleukin-3, Interleukin-5 and GM-CSF signaling	3
ERBB2 Activates PTK6 Signaling	2
ERBB2 Regulates Cell Motility	2
Nef-mediates down modulation of cell surface receptors by recruiting them to clathrin adapters	2
PI3K events in ERBB2 signaling	2
Synthesis of PIPs at the plasma membrane	3
Mitochondrial calcium ion transport	2
Signaling by PDGF	3
Downregulation of TGF-beta receptor signaling	2
Loss of Function of SMAD4 in Cancer	1
SMAD2/3 MH2 Domain Mutants in Cancer	1
SMAD4 MH2 Domain Mutants in Cancer	1
Nonsense Mediated Decay (NMD) independent of the Exon Junction Complex (EJC)	79
Formation of a pool of free 40S subunits	79
Viral mRNA Translation	79
SRP-dependent cotranslational protein targeting to membrane	79
GTP hydrolysis and joining of the 60S ribosomal subunit	79
L13a-mediated translational silencing of Ceruloplasmin expression	79
Translation initiation complex formation	32
Ribosomal scanning and start codon recognition	32
Eukaryotic Translation Elongation	82
Major pathway of rRNA processing in the nucleolus and cytosol	79
Nonsense-Mediated Decay (NMD)	80
Peptide chain elongation	80
Nonsense Mediated Decay (NMD) enhanced by the Exon Junction Complex (EJC)	80

SARS-CoV-1 modulates host translation machinery	33
Eukaryotic Translation Initiation	79
Eukaryotic Translation Termination	79
Cap-dependent Translation Initiation	79
rRNA processing in the nucleus and cytosol	79
rRNA processing	86
Response of EIF2AK4 (GCN2) to amino acid deficiency	81
Nonsense Mediated Decay (NMD) independent of the Exon Junction Complex (EJC)	54
Formation of a pool of free 40S subunits	53
Viral mRNA Translation	53
L13a-mediated translational silencing of Ceruloplasmin expression	54
SRP-dependent cotranslational protein targeting to membrane	53
GTP hydrolysis and joining of the 60S ribosomal subunit	53
Nonsense-Mediated Decay (NMD)	54
Nonsense Mediated Decay (NMD) enhanced by the Exon Junction Complex (EJC)	54
Translation initiation complex formation	23
Ribosomal scanning and start codon recognition	22
Eukaryotic Translation Elongation	55
Major pathway of rRNA processing in the nucleolus and cytosol	53
Peptide chain elongation	54
SARS-CoV-1 modulates host translation machinery	23
Eukaryotic Translation Initiation	54
Eukaryotic Translation Termination	53
Cap-dependent Translation Initiation	54
rRNA processing in the nucleus and cytosol	53
Translation	57
rRNA processing	56
Cytokine Signaling in Immune system	85
Immune System	135
Interferon Signaling	37
Interferon alpha/beta signaling	23

Signaling by Interleukins	51
Interleukin-12 family signaling	18
Interleukin-12 signaling	16
Gene and protein expression by JAK-STAT signaling after Interleukin-12 stimulation	15
RUNX1 and FOXP3 control the development of regulatory T lymphocytes (Tregs)	7
Formation of Senescence-Associated Heterochromatin Foci (SAHF)	6
Clathrin-mediated endocytosis	16
Cellular Senescence	18
EPHB-mediated forward signaling	9
Apoptosis induced DNA fragmentation	5
TNFR2 non-canonical NF-kB pathway	12
Programmed Cell Death	19
Transcriptional regulation by RUNX1	20
Signaling by Rho GTPases	38
Apoptosis	16
TCR signaling	13
Nonsense Mediated Decay (NMD) independent of the Exon Junction Complex (EJC)	72
Formation of a pool of free 40S subunits	72
Viral mRNA Translation	72
SRP-dependent cotranslational protein targeting to membrane	73
GTP hydrolysis and joining of the 60S ribosomal subunit	72
L13a-mediated translational silencing of Ceruloplasmin expression	72
Translation initiation complex formation	29
Ribosomal scanning and start codon recognition	29
Major pathway of rRNA processing in the nucleolus and cytosol	72
Nonsense-Mediated Decay (NMD)	72
Peptide chain elongation	72
Nonsense Mediated Decay (NMD) enhanced by the Exon Junction Complex (EJC)	72
Eukaryotic Translation Elongation	73
Eukaryotic Translation Initiation	72
Eukaryotic Translation Termination	72

Cap-dependent Translation Initiation	72
rRNA processing in the nucleus and cytosol	72
SARS-CoV-1 modulates host translation machinery	29
Formation of the ternary complex, and subsequently, the 43S complex	29
Activation of the mRNA upon binding of the cap-binding complex and eIFs, and subsequent binding to 43S	29
Response of EIF2AK1 (HRI) to heme deficiency	4
NR1H3 & NR1H2 regulate gene expression linked to cholesterol transport and efflux	5
Gene and protein expression by JAK-STAT signaling after Interleukin-12 stimulation	5
Interleukin-12 signaling	5
NR1H2 and NR1H3-mediated signaling	5
Interleukin-12 family signaling	5
CASP8 activity is inhibited	2
Signaling by Interleukins	10
ATF6 (ATF6-alpha) activates chaperone genes	2
ATF6 (ATF6-alpha) activates chaperones	2
RIP-mediated NFkB activation via ZBP1	2
Caspase activation via Death Receptors in the presence of ligand	2
mRNA Splicing - Major Pathway	5
Uptake and function of anthrax toxins	2
Transcriptional regulation of granulopoiesis	3
ZBP1(DAI) mediated induction of type I IFNs	2
mRNA Splicing	5
Nuclear events stimulated by ALK signaling in cancer	2
Downregulation of TGF-beta receptor signaling	2
Caspase activation via extrinsic apoptotic signalling pathway	2
Nonsense Mediated Decay (NMD) independent of the Exon Junction Complex (EJC)	27
Formation of a pool of free 40S subunits	27
Viral mRNA Translation	27
SRP-dependent cotranslational protein targeting to membrane	27
GTP hydrolysis and joining of the 60S ribosomal subunit	27
Translation initiation complex formation	11

Ribosomal scanning and start codon recognition	11
Nonsense-Mediated Decay (NMD)	27
Peptide chain elongation	27
Nonsense Mediated Decay (NMD) enhanced by the Exon Junction Complex (EJC)	27
L13a-mediated translational silencing of Ceruloplasmin expression	27
Eukaryotic Translation Termination	27
Eukaryotic Translation Initiation	27
Cap-dependent Translation Initiation	27
Eukaryotic Translation Elongation	27
Major pathway of rRNA processing in the nucleolus and cytosol	27
Translation	28
SARS-CoV-1 modulates host translation machinery	11
Formation of the ternary complex, and subsequently, the 43S complex	11
Activation of the mRNA upon binding of the cap-binding complex and eIFs, and subsequent binding to 43S	11
Chromatin organization	24
Chromatin modifying enzymes	24
RUNX1 regulates transcription of genes involved in differentiation of myeloid cells	5
Regulation of MECP2 expression and activity	8
Insulin-like Growth Factor-2 mRNA Binding Proteins (IGF2BPs/IMPs/VICKZs) bind RNA	5
YAP1- and WWTR1 (TAZ)-stimulated gene expression	5
PKMTs methylate histone lysines	7
VxPx cargo-targeting to cilium	5
Loss of MECP2 binding ability to 5hmC-DNA	2
Nuclear Envelope Breakdown	8
trans-Golgi Network Vesicle Budding	9
G-protein beta:gamma signalling	6
RUNX1 interacts with co-factors whose precise effect on RUNX1 targets is not known	6
Loss of MECP2 binding ability to the NCoR/SMRT complex	3
Chylomicron remodeling	4
Heme signaling	8
G beta:gamma signalling through PI3Kgamma	5

Nef and signal transduction	3
CD209 (DC-SIGN) signaling	5
RHO GTPase cycle	28
RUNX1 regulates transcription of genes involved in differentiation of myeloid cells	7
Nuclear Receptor transcription pathway	17
Chromatin organization	31
Chromatin modifying enzymes	31
Transcriptional Regulation by MECP2	16
Transcriptional regulation by RUNX1	29
RUNX1 interacts with co-factors whose precise effect on RUNX1 targets is not known	9
RUNX2 regulates genes involved in differentiation of myeloid cells	4
Transcriptional activation of mitochondrial biogenesis	14
Synthesis of PIPs at the plasma membrane	12
RUNX2 regulates chondrocyte maturation	4
Insulin-like Growth Factor-2 mRNA Binding Proteins (IGF2BPs/IMPs/VICKZs) bind RNA	5
Circadian Clock	15
RHO GTPase cycle	42
Notch-HLH transcription pathway	7
RUNX3 regulates RUNX1-mediated transcription	3
Signaling by Rho GTPases	58
Regulation of MECP2 expression and activity	8
Signaling by Rho GTPases, Miro GTPases and RHOBTB3	59
Heme signaling	11

#Entities tota	Entities ratio	Entities pValue	Entities FDR	#Reactions foun	#Reactions tota
97	0.00636817226890756	1.1102230246251565E-1	3.4416913763379853E-1	5	5
102	0.00669642857142857	1.1102230246251565E-1	3.4416913763379853E-1	9	9
101	0.0066307773109243	1.1102230246251565E-1	3.4416913763379853E-1	1	1
106	0.00695903361344537	1.1102230246251565E-1	3.4416913763379853E-1	2	2
119	0.0078125	1.1102230246251565E-1	3.4416913763379853E-1	5	5
114	0.00748424369747899	1.1102230246251565E-1	3.4416913763379853E-1	2	2
120	0.00787815126050420	1.1102230246251565E-1	3.4416913763379853E-1	3	3
120	0.00787815126050420	1.1102230246251565E-1	3.4416913763379853E-1	3	3
62	0.00407037815126050	1.1102230246251565E-1	3.4416913763379853E-1	2	2
64	0.00420168067226890	1.1102230246251565E-1	3.4416913763379853E-1	2	2
189	0.01240808823529411	1.1102230246251565E-1	3.4416913763379853E-1	6	7
124	0.00814075630252100	1.1102230246251565E-1	3.4416913763379853E-1	5	6
124	0.00814075630252100	1.1102230246251565E-1	3.4416913763379853E-1	4	5
41	0.00269170168067226	1.1102230246251565E-1	3.4416913763379853E-1	2	3
130	0.00853466386554621	1.1102230246251565E-1	3.4416913763379853E-1	13	21
106	0.00695903361344537	1.1102230246251565E-1	3.4416913763379853E-1	3	5
130	0.00853466386554621	1.1102230246251565E-1	3.4416913763379853E-1	10	18
176	0.01155462184873949	1.1102230246251565E-1	3.4416913763379853E-1	14	28
207	0.01358981092436974	1.1102230246251565E-1	3.4416913763379853E-1	7	15
339	0.0222557773109243	1.1102230246251565E-1	3.4416913763379853E-1	41	99
101	0.0066307773109243	1.1102230246251565E-1	2.220446049250313E-15	1	1
41	0.00269170168067226	1.1102230246251565E-1	2.220446049250313E-15	3	3
106	0.00695903361344537	1.1102230246251565E-1	2.220446049250313E-15	2	2
114	0.00748424369747899	1.1102230246251565E-1	2.220446049250313E-15	2	2
119	0.0078125	1.1102230246251565E-1	2.220446049250313E-15	5	5
120	0.00787815126050420	1.1102230246251565E-1	2.220446049250313E-15	3	3
120	0.00787815126050420	1.1102230246251565E-1	2.220446049250313E-15	3	3
62	0.00407037815126050	1.1102230246251565E-1	2.220446049250313E-15	2	2
64	0.00420168067226890	1.1102230246251565E-1	2.220446049250313E-15	2	2

102	0.006696428571428571	1.1102230246251565E-1(	2.220446049250313E-15	8	9
189	0.012408088235294117	1.1102230246251565E-1(	2.220446049250313E-15	6	7
124	0.008140756302521009	1.1102230246251565E-1(	2.220446049250313E-15	5	6
97	0.006368172268907563	1.1102230246251565E-1(	2.220446049250313E-15	4	5
124	0.008140756302521009	1.1102230246251565E-1(	2.220446049250313E-15	4	5
130	0.008534663865546218	1.1102230246251565E-1(	2.220446049250313E-15	13	21
106	0.006959033613445379	1.1102230246251565E-1(	2.220446049250313E-15	3	5
130	0.008534663865546218	1.1102230246251565E-1(	2.220446049250313E-15	10	18
207	0.013589810924369748	1.1102230246251565E-1(	2.220446049250313E-15	7	15
339	0.02225577731092437	1.1102230246251565E-1(	2.220446049250313E-15	42	99
54	0.0035451680672268907	1.1102230246251565E-1(	2.220446049250313E-15	1	3
33	0.002166491596638655(	3.877595522339128E-5	0.01942675356691903	5	5
172	0.011292016806722689	4.232134896041684E-4	0.07451891579971504	27	119
64	0.004201680672268907	4.811790530714344E-4	0.07451891579971504	23	58
29	0.0019038865546218487	6.065394785085276E-4	0.07451891579971504	3	20
72	0.004726890756302521	7.451891579971504E-4	0.07451891579971504	7	46
41	0.002691701680672269	0.001633572344655554	0.12688103145430252	6	18
1039	0.06821165966386554	0.0017870567810465143	0.12688103145430252	130	745
47	0.003085609243697479	0.002402497214827548	0.14635003228197274	21	59
54	0.0035451680672268907	0.003543360358561398	0.14635003228197274	16	41
658	0.043198529411764705	0.0036690084414943147	0.14635003228197274	104	505
16	0.001050420168067226(	0.0037173541021684464	0.14635003228197274	4	27
56	0.003676470588235294	0.003920437880611027	0.14635003228197274	24	37
116	0.007615546218487395	0.0041733883993690934	0.14635003228197274	23	73
17	0.0011160714285714285	0.00418142949377065	0.14635003228197274	3	20
121	0.007943802521008403	0.004838473563786305	0.14999268047737546	23	70
121	0.007943802521008403	0.004838473563786305	0.14999268047737546	23	72
21	0.001378676470588235‹	0.006289323114635259	0.18219440660259412	4	31
2627	0.1724658613445378	0.008056670997901416	0.18219440660259412	245	1664
73	0.004792542016806723	0.008116441096888027	0.18219440660259412	36	36
143	0.009388130252100840	0.008609888346955197	0.18219440660259412	26	102

101	0.006531298499741335	1.1102230246251565E-1(	1.7763568394002505E-1!	1	1
106	0.006854630108639421	1.1102230246251565E-1(	1.7763568394002505E-1!	2	2
114	0.007371960682876358	1.1102230246251565E-1(	1.7763568394002505E-1!	2	2
119	0.007695292291774444	1.1102230246251565E-1(	1.7763568394002505E-1!	5	5
120	0.007759958613554061	1.1102230246251565E-1(	1.7763568394002505E-1!	3	3
120	0.007759958613554061	1.1102230246251565E-1(	1.7763568394002505E-1!	3	3
62	0.004009311950336265	1.1102230246251565E-1(	1.7763568394002505E-1!	2	2
64	0.004138644593895499	1.1102230246251565E-1(	1.7763568394002505E-1!	2	2
189	0.012221934816347646	1.1102230246251565E-1(	1.7763568394002505E-1!	6	7
124	0.00801862390067253	1.1102230246251565E-1(	1.7763568394002505E-1!	5	6
97	0.006272633212622866	1.1102230246251565E-1(	1.7763568394002505E-1!	4	5
106	0.006854630108639421	1.1102230246251565E-1(	1.7763568394002505E-1!	4	5
124	0.00801862390067253	1.1102230246251565E-1(	1.7763568394002505E-1!	4	5
102	0.006595964821520952	1.1102230246251565E-1(	1.7763568394002505E-1!	7	9
41	0.002651319192964304:	1.1102230246251565E-1(	1.7763568394002505E-1!	2	3
130	0.008406621831350233	1.1102230246251565E-1(	1.7763568394002505E-1!	13	21
130	0.008406621831350233	1.1102230246251565E-1(	1.7763568394002505E-1!	10	18
207	0.013385928608380756	1.1102230246251565E-1(	1.7763568394002505E-1!	8	15
339	0.02192188308329022	1.1102230246251565E-1(	1.7763568394002505E-1!	42	99
247	0.015972581479565442	1.1102230246251565E-1(	1.7763568394002505E-1!	8	21
658	0.043198529411764705	8.360720445965697E-7	1.764112014098762E-4	14	505
2627	0.1724658613445378	3.97813708885586E-6	2.924613856514213E-4	70	1664
1039	0.06821165966386554	4.178019795020305E-6	2.924613856514213E-4	27	745
71	0.004661239495798319	6.501455177887472E-6	3.3807566925014854E-4	7	41
211	0.013852415966386554	4.3132384375377253E-4	0.01734937716535767	2	47
19	0.001247373949579831!	4.956964904387906E-4	0.01734937716535767	2	9
31	0.002035189075630252:	0.001303073923539233	0.03573914545824364	2	26
33	0.002166491596638655(	0.0014735456125581914	0.03573914545824364	4	7
135	0.008862920168067227	0.0015538758894888538	0.03573914545824364	12	52
41	0.002691701680672269	0.0022556156511784886	0.04736792867474826	7	14
48	0.003151260504201680!	0.00306901032882112	0.0552603058461294	4	43

2	1.3130252100840336E-4	0.003408268312995788	0.0552603058461294	1	1
51	0.003348214285714285	0.0034537691153830874	0.0552603058461294	2	39
3	1.9695378151260505E-4	0.005108211153417641	0.07662316730126462	1	2
73	0.004792542016806723	0.006915356281568141	0.08820322097039579	1	36
5	3.2825630252100843E-4	0.008499736190052176	0.08820322097039579	2	6
84	0.005514705882352941	0.009051956525877647	0.08820322097039579	1	56
86	0.005646008403361344	0.009468364038783705	0.08820322097039579	1	15
96	0.006302521008403361	0.011675988925910263	0.08820322097039579	1	114
7	4.5955882352941176E-4	0.011880142940949079	0.08820322097039579	1	1
16	0.0010504201680672268	5.905048960919146E-8	6.696325521682311E-5	17	27
21	0.0013786764705882354	4.5398293846421467E-7	2.574083261092097E-4	17	31
191	0.012539390756302522	1.379382018151798E-5	0.005214064028613796	3	6
13	8.534663865546219E-4	6.468598266928804E-5	0.018306133095408517	2	3
26	0.0017069327731092437	1.9554285587441989E-4	0.031939145644581	4	4
254	0.016675420168067227	1.971552200282778E-4	0.031939145644581	23	85
254	0.016675420168067227	1.971552200282778E-4	0.031939145644581	23	85
18	0.0011817226890756302	2.898135787380296E-4	0.04086371460206217	5	6
460	0.030199579831932773	3.582667114191418E-4	0.045141605638811866	40	91
32	0.0021008403361344537	5.81377197624966E-4	0.058362121618384055	19	19
12	7.878151260504202E-4	6.053533216286988E-4	0.058362121618384055	2	3
45	0.0029543067226890755	6.208736342381282E-4	0.058362121618384055	2	3
81	0.005317752100840336	0.0011928643646318404	0.10377919972297012	12	27
7	4.5955882352941176E-4	0.0014740941995692314	0.11646297245545734	2	3
27	0.0017725840336134454	0.0017518806538308063	0.11646297245545734	2	2
159	0.010438550420168067	0.0017952194593912063	0.11646297245545734	3	6
40	0.0026260504201680674	0.001802363693125364	0.11646297245545734	1	6
708	0.04648109243697479	0.0018486186104040847	0.11646297245545734	56	203
724	0.047531512605042014	0.0026768396067889944	0.15793353680055067	56	212
76	0.004989495798319327	0.0030469211116248562	0.17062758225099195	33	33
13	8.534663865546219E-4	2.3895911139826254E-6	0.0022940074694233203	2	3
254	0.016675420168067227	2.2496040179542653E-4	0.07198732857453649	16	85

254	0.016675420168067227	2.249604017954265 3E-4	0.07198732857453649	16	85
57	0.003742121848739496	3.175793293699325E-4	0.0762190390487838	14	18
68	0.004464285714285714	7.942554727081719E-4	0.07853273914429693	13	21
68	0.004464285714285714	7.942554727081719E-4	0.07853273914429693	13	21
68	0.004464285714285714	7.942554727081719E-4	0.07853273914429693	13	21
68	0.004464285714285714	7.942554727081719E-4	0.07853273914429693	13	21
68	0.004464285714285714	7.942554727081719E-4	0.07853273914429693	14	39
26	0.0017069327731092437	8.180493660864263E-4	0.07853273914429693	1	4
498	0.03269432773109244	9.960148350451892E-4	0.08665329064893146	92	478
30	0.0019695378151260504	0.0013810801013616203	0.10336883065207525	5	11
14	9.191176470588235E-4	0.0014838664815577385	0.10336883065207525	4	5
248	0.016281512605042018	0.0020920119386821368	0.10336883065207525	9	67
365	0.023962710084033615	0.002104483417471048	0.10336883065207525	12	210
16	0.0010504201680672268	0.0021646578796288285	0.10336883065207525	2	5
16	0.0010504201680672268	0.0021646578796288285	0.10336883065207525	2	5
16	0.0010504201680672268	0.0021646578796288285	0.10336883065207525	2	5
16	0.0010504201680672268	0.0021646578796288285	0.10336883065207525	2	5
83	0.005449054621848739	0.0021654382796696403	0.10336883065207525	10	16
32	0.0027531618342940723	0.0054987437685023455	0.016484861455378086	2	9
39	0.0033554159855459006	0.006699573154655747	0.016484861455378086	2	28
48	0.004129742751441108	0.008242430727689043	0.016484861455378086	2	34
128	0.011012647337176289	0.021904016272979665	0.022754719414934788	1	11
133	0.011442828873784738	0.022754719414934788	0.022754719414934788	1	14
265	0.022799621440247785	0.045079420142677074	0.045079420142677074	1	117
314	0.02701540049901058	0.05330096913389937	0.05330096913389937	2	333
726	0.06246235911554676	0.12102317192481404	0.12102317192481404	1	338
86	0.005646008403361344	4.134850783987787E-8	2.2534936772733438E-5	24	55
148	0.009716386554621849	8.862297340428427E-8	2.410544876596532E-5	37	105
11	7.221638655462185E-4	1.609874422658919E-7	2.9138727050126434E-5	7	7
13	8.534663865546219E-4	3.647998344513681E-7	4.961277748538606E-5	2	3
127	0.008337710084033613	1.4038036639796303E-6	1.530145993737797E-4	7	14

328	0.02153361344537815	2.9684976483590475E-6	2.671647883523143E-4	132	319
44	0.002888655462184874	9.358600854936228E-6	7.206122658300895E-4	5	28
69	0.004529936974789916	1.124891219805857E-5	7.649260294679827E-4	17	34
6	3.939075630252101E-4	4.131053481881697E-5	0.002478632089129018	2	2
67	0.004398634453781513	9.53597895160252E-5	0.0051494286338653605	18	26
23	0.0015099789915966387	1.2167260359075005E-4	0.0059619575759467525	3	6
76	0.004989495798319327	1.8776341919690687E-4	0.007935854352631	27	51
109	0.007155987394957983	1.9355742323490244E-4	0.007935854352631	1	1
3	1.9695378151260505E-4	5.014549062865026E-4	0.01869015835424248	2	3
34	0.002232142857142857	5.304548821296606E-4	0.01869015835424248	21	24
61	0.004004726890756302	5.497105398306612E-4	0.01869015835424248	3	22
15	9.847689075630252E-4	6.015097012387338E-4	0.019248310439639482	1	4
4	2.626050420168067E-4	8.852338380476166E-4	0.026467525202070186	2	2
104	0.006827731092436975	9.657917061492993E-4	0.026467525202070186	45	77
70	0.004595588235294118	0.0010130890493251776	0.026467525202070186	8	23
105	0.006893382352941176	5.1044015902945716E-8	7.896509260185702E-5	52	59
498	0.03269432773109244	1.030626244902244E-6	5.074086089125185E-4	214	478
254	0.016675420168067227	1.3145300749028976E-6	5.074086089125185E-4	37	85
254	0.016675420168067227	1.3145300749028976E-6	5.074086089125185E-4	37	85
86	0.005646008403361344	1.987965208671838E-6	6.14281249479598E-4	2	2
69	0.004529936974789916	6.9357391759838904E-6	0.0015881976318168345	21	33
112	0.007352941176470588	7.186414623605586E-6	0.0015881976318168345	37	89
39	0.0025603991596638656	3.314514849162009E-5	0.006397013658882678	13	14
11	7.221638655462185E-4	6.46140323677935E-5	0.01104899953489269	7	7
100	0.006565126050420168	1.643045801803522E-4	0.02530290534777424	76	77
191	0.012539390756302522	3.258565024222282E-4	0.045619910339111946	3	6
93	0.006105567226890756	6.605019425784953E-4	0.0845442486500474	41	46
48	0.0031512605042016808	7.953759406065242E-4	0.09464973693217638	13	22
18	0.0011817226890756302	8.724128661927733E-4	0.09596541528120506	5	6
26	0.0017069327731092437	0.0011423545206212804	0.11766251562399188	4	4
13	8.534663865546219E-4	0.0012547564239439035	0.11914012284244946	2	3

259	0.017003676470588234	0.0013092321191477962	0.11914012284244946	126	132
35	0.002297794117647059	0.0014622904995862385	0.12184463194167605	25	25
8	5.252100840336134E-4	0.001504254715329334	0.12184463194167605	1	1
28	0.001838235294117647	0.001733464314848443	0.1270149136781613	2	2
101	0.00663077731092437	1.1102230246251565E-1(	2.9976021664879227E-1!	1	1
106	0.006959033613445379	1.1102230246251565E-1(	2.9976021664879227E-1!	2	2
114	0.007484243697478992	1.1102230246251565E-1(	2.9976021664879227E-1!	2	2
119	0.0078125	1.1102230246251565E-1(	2.9976021664879227E-1!	5	5
120	0.007878151260504201	1.1102230246251565E-1(	2.9976021664879227E-1!	3	3
120	0.007878151260504201	1.1102230246251565E-1(	2.9976021664879227E-1!	3	3
189	0.012408088235294117	1.1102230246251565E-1(	2.9976021664879227E-1!	6	7
124	0.008140756302521009	1.1102230246251565E-1(	2.9976021664879227E-1!	5	6
97	0.006368172268907563	1.1102230246251565E-1(	2.9976021664879227E-1!	4	5
47	0.003085609243697479	1.1102230246251565E-1(	2.9976021664879227E-1!	4	5
124	0.008140756302521009	1.1102230246251565E-1(	2.9976021664879227E-1!	4	5
102	0.006696428571428571	1.1102230246251565E-1(	2.9976021664879227E-1!	6	9
130	0.008534663865546218	1.1102230246251565E-1(	2.9976021664879227E-1!	13	21
106	0.006959033613445379	1.1102230246251565E-1(	2.9976021664879227E-1!	3	5
130	0.008534663865546218	1.1102230246251565E-1(	2.9976021664879227E-1!	10	18
207	0.013589810924369748	1.1102230246251565E-1(	2.9976021664879227E-1!	7	15
247	0.016215861344537816	1.1102230246251565E-1(	2.9976021664879227E-1!	8	21
41	0.002691701680672269	1.1102230246251565E-1(	2.9976021664879227E-1!	1	3
112	0.007352941176470588	1.1102230246251565E-1(	2.9976021664879227E-1!	2	7
339	0.02225577731092437	1.1102230246251565E-1(	2.9976021664879227E-1!	27	99
41	0.002691701680672269	1.1102230246251565E-1(	2.6645352591003757E-1!	3	3
101	0.00663077731092437	1.1102230246251565E-1(	2.6645352591003757E-1!	1	1
106	0.006959033613445379	1.1102230246251565E-1(	2.6645352591003757E-1!	2	2
119	0.0078125	1.1102230246251565E-1(	2.6645352591003757E-1!	5	5
114	0.007484243697478992	1.1102230246251565E-1(	2.6645352591003757E-1!	2	2
120	0.007878151260504201	1.1102230246251565E-1(	2.6645352591003757E-1!	3	3
120	0.007878151260504201	1.1102230246251565E-1(	2.6645352591003757E-1!	3	3

62	0.004070378151260504	1.1102230246251565E-1	2.6645352591003757E-1	2	2
64	0.004201680672268907	1.1102230246251565E-1	2.6645352591003757E-1	2	2
102	0.006696428571428571	1.1102230246251565E-1	2.6645352591003757E-1	8	9
189	0.012408088235294117	1.1102230246251565E-1	2.6645352591003757E-1	6	7
124	0.008140756302521009	1.1102230246251565E-1	2.6645352591003757E-1	5	6
97	0.006368172268907563	1.1102230246251565E-1	2.6645352591003757E-1	4	5
124	0.008140756302521009	1.1102230246251565E-1	2.6645352591003757E-1	4	5
130	0.008534663865546218	1.1102230246251565E-1	2.6645352591003757E-1	13	21
106	0.006959033613445379	1.1102230246251565E-1	2.6645352591003757E-1	3	5
130	0.008534663865546218	1.1102230246251565E-1	2.6645352591003757E-1	10	18
207	0.013589810924369748	1.1102230246251565E-1	2.6645352591003757E-1	7	15
247	0.016215861344537816	1.1102230246251565E-1	2.6645352591003757E-1	8	21
54	0.003545168067226890	1.1102230246251565E-1	2.6645352591003757E-1	1	3
40	0.002626050420168067	7.257189293952138E-10	2.85207539252319E-7	1	6
45	0.002954306722689075	1.626396239906569E-9	3.187736630216875E-7	1	3
184	0.01207983193277311	1.909568458513533E-5	0.0025015346806527283	1	54
153	0.010044642857142858	6.596873934827663E-5	0.0064649364561311096	11	31
247	0.016215861344537816	1.2029952002778899E-4	0.009383362562167541	1	21
59	0.003873424369747899	1.5041970268225846E-4	0.0097772806743468	6	13
118	0.007746848739495798	1.9387031953910228E-4	0.010856737894189727	8	19
238	0.015625	6.956796215128458E-4	0.034088301454129444	11	67
17	0.001116071428571428	0.002701889390561707	0.09456612866965974	5	6
17	0.001116071428571428	0.002701889390561707	0.09456612866965974	2	4
17	0.001116071428571428	0.002701889390561707	0.09456612866965974	2	8
18	0.001181722689075630	0.0030204184044606563	0.096653388942741	2	5
21	0.001378676470588235	0.004075868822725259	0.12227606468175778	2	16
25	0.001641281512605042	0.005710575690557107	0.1278369331533875	5	5
369	0.02422531512605042	0.006140079212958671	0.1278369331533875	11	116
27	0.001772584033613445	0.0066227587652830655	0.1278369331533875	2	16
86	0.005646008403361344	0.006863115041083723	0.1278369331533875	3	17
87	0.005711659663865546	0.007082949176043685	0.1278369331533875	16	35

28	0.001838235294117647	0.007102051841854862	0.1278369331533875	7	11
28	0.001838235294117647	0.007102051841854862	0.1278369331533875	7	26
101	0.00663077731092437	1.1102230246251565E-1	1.5543122344752192E-1	1	1
106	0.006959033613445379	1.1102230246251565E-1	1.5543122344752192E-1	2	2
114	0.007484243697478992	1.1102230246251565E-1	1.5543122344752192E-1	2	2
119	0.0078125	1.1102230246251565E-1	1.5543122344752192E-1	5	5
120	0.007878151260504201	1.1102230246251565E-1	1.5543122344752192E-1	3	3
62	0.004070378151260504	1.1102230246251565E-1	1.5543122344752192E-1	2	2
64	0.004201680672268907	1.1102230246251565E-1	1.5543122344752192E-1	2	2
189	0.012408088235294117	1.1102230246251565E-1	1.5543122344752192E-1	6	7
124	0.008140756302521009	1.1102230246251565E-1	1.5543122344752192E-1	5	6
47	0.003085609243697479	1.1102230246251565E-1	1.5543122344752192E-1	4	5
97	0.006368172268907563	1.1102230246251565E-1	1.5543122344752192E-1	4	5
124	0.008140756302521009	1.1102230246251565E-1	1.5543122344752192E-1	4	5
120	0.007878151260504201	1.1102230246251565E-1	1.5543122344752192E-1	2	3
59	0.003873424369747899	1.1102230246251565E-1	1.5543122344752192E-1	5	8
106	0.006959033613445379	1.1102230246251565E-1	1.5543122344752192E-1	3	5
43	0.002823004201680672	1.1102230246251565E-1	1.5543122344752192E-1	4	7
130	0.008534663865546218	1.1102230246251565E-1	1.5543122344752192E-1	12	21
130	0.008534663865546218	1.1102230246251565E-1	1.5543122344752192E-1	10	18
135	0.008862920168067227	1.1102230246251565E-1	1.5543122344752192E-1	16	29
113	0.00741859243697479	1.1102230246251565E-1	1.5543122344752192E-1	7	14
211	0.013852415966386554	1.5121741414603207E-8	7.3225024224132795E-6	4	47
13	8.534663865546219E-4	3.0294324160351493E-8	7.3225024224132795E-6	2	3
658	0.043198529411764705	4.520063223711901E-8	7.3225024224132795E-6	115	505
1039	0.06821165966386554	2.8390451034532305E-6	3.463635026212941E-4	117	745
28	0.001838235294117647	3.739006465719452E-5	0.0036268362717478686	5	14
85	0.005580357142857143	2.504320214079536E-4	0.020284993734044243	6	60
65	0.004267331932773109	9.040623981293461E-4	0.051762948136188536	5	42
29	0.001903886554621848	9.442827252319486E-4	0.051762948136188536	3	20
386	0.02534138655462185	9.58573113633121E-4	0.051762948136188536	15	196

8	5.252100840336134E-4	0.0012935275530205903	0.062089322544988335	2	2
484	0.03177521008403361	0.004369315707244969	0.15183223185941674	10	259
15	9.847689075630252E-4	0.004415132716677816	0.15183223185941674	3	4
15	9.847689075630252E-4	0.004415132716677816	0.15183223185941674	1	5
105	0.006893382352941176	0.005056434508991403	0.15183223185941674	9	53
172	0.011292016806722689	0.005464146383440949	0.15183223185941674	18	119
17	0.0011160714285714285	0.005623415994793213	0.15183223185941674	2	8
17	0.0011160714285714285	0.005623415994793213	0.15183223185941674	2	10
17	0.0011160714285714285	0.005623415994793213	0.15183223185941674	3	20
18	0.0011817226890756302	0.006277970911246156	0.1569492727811539	2	7
19	0.0012473739495798318	0.006965532405544428	0.15701844844324486	2	4
45	0.0029543067226890755	8.237291737600572E-9	9.036309036147827E-6	2	3
40	0.0026260504201680674	2.5345437748569566E-8	1.3889299886216122E-5	1	6
16	0.0010504201680672268	4.338662146219363E-7	1.5836116833700675E-4	15	27
21	0.0013786764705882354	2.585863992266013E-6	7.085267338808876E-4	15	31
153	0.010044642857142858	0.0013861824163290404	0.30357394917605984	12	31
118	0.0077468487394957986	0.001954873701811244	0.31527572339497356	9	19
47	0.003085609243697479	0.00220041571667628	0.31527572339497356	17	17
64	0.004201680672268907	0.002301282652518055	0.31527572339497356	4	7
39	0.0025603991596638656	0.00497728034523226	0.5042148075367666	4	12
184	0.01207983193277311	0.005980285600273194	0.5042148075367666	2	54
26	0.0017069327731092437	0.00629099084307605	0.5042148075367666	6	20
59	0.0038734243697478993	0.006549876317158865	0.5042148075367666	6	13
79	0.0051864495798319326	0.007097251370309365	0.5042148075367666	6	7
79	0.0051864495798319326	0.007097251370309365	0.5042148075367666	3	6
238	0.015625	0.007492356115196608	0.5042148075367666	14	67
81	0.005317752100840336	0.008073153373159614	0.5042148075367666	4	17
63	0.004136029411764706	0.008866499378953452	0.5042148075367666	5	5
84	0.0055147058823529415	0.009717661876186723	0.5042148075367666	6	10
46	0.0030199579831932774	0.009728999975557429	0.5042148075367666	25	36
16	0.0010504201680672268	0.010398759171049266	0.5042148075367666	5	9

11	7.221638655462185E-4	2.0005249242727174E-6	0.0017244524847230824	7	7
13	8.534663865546219E-4	4.479872814044938E-6	0.0019308251828533685	2	3
39	0.002560399159663865(	8.073025684729451E-6	0.0023169583715173525	13	14
100	0.006565126050420168	9.491888838386586E-5	0.02040756100253116	76	77
6	3.939075630252101E-4	1.8728650713384365E-4	0.020617477047622512	2	2
16	0.001050420168067226{	2.1702607418550013E-4	0.020617477047622512	5	5
16	0.001050420168067226{	2.1702607418550013E-4	0.020617477047622512	5	5
16	0.001050420168067226{	2.1702607418550013E-4	0.020617477047622512	5	5
16	0.001050420168067226{	2.1702607418550013E-4	0.020617477047622512	5	5
259	0.017003676470588234	2.8219905148363367E-4	0.022893941363439696	124	132
7	4.5955882352941176E-4	2.935120687620474E-4	0.022893941363439696	1	1
19	0.001247373949579831{	4.138887674688396E-4	0.028538407539470345	3	6
8	5.252100840336134E-4	4.3240011423439917E-4	0.028538407539470345	1	1
9	5.908613445378151E-4	6.076202940540343E-4	0.03537362390561083	2	5
2	1.3130252100840336E-4	6.205898930808917E-4	0.03537362390561083	1	1
105	0.006893382352941176	6.862651456130964E-4	0.03637205271749411	49	59
70	0.004595588235294118	0.0017757756564416027	0.08878878282208014	7	25
29	0.0019038865546218487	0.0019549999425851894	0.09121913417065086	5	20
49	0.003216911764705882	0.002027091870458908	0.09121913417065086	6	10
14	9.191176470588235E-4	0.0021419412297694063	0.09210347288008447	7	7
101	0.00663077731092437	1.1102230246251565E-1(	1.2212453270876722E-1!	1	1
106	0.006959033613445379	1.1102230246251565E-1(	1.2212453270876722E-1!	2	2
114	0.007484243697478992	1.1102230246251565E-1(	1.2212453270876722E-1!	2	2
119	0.0078125	1.1102230246251565E-1(	1.2212453270876722E-1!	5	5
120	0.007878151260504201	1.1102230246251565E-1(	1.2212453270876722E-1!	3	3
62	0.004070378151260504!	1.1102230246251565E-1(	1.2212453270876722E-1!	2	2
64	0.004201680672268907	1.1102230246251565E-1(	1.2212453270876722E-1!	2	2
124	0.008140756302521009	1.1102230246251565E-1(	1.2212453270876722E-1!	5	6
97	0.006368172268907563	1.1102230246251565E-1(	1.2212453270876722E-1!	4	5
124	0.008140756302521009	1.1102230246251565E-1(	1.2212453270876722E-1!	4	5
120	0.007878151260504201	1.1102230246251565E-1(	1.2212453270876722E-1!	2	3

106	0.006959033613445379	1.1102230246251565E-16	1.2212453270876722E-15	3	5
130	0.008534663865546218	1.1102230246251565E-16	1.2212453270876722E-15	12	21
130	0.008534663865546218	1.1102230246251565E-16	1.2212453270876722E-15	10	18
102	0.006696428571428571	1.1102230246251565E-16	1.2212453270876722E-15	4	9
189	0.012408088235294117	1.1102230246251565E-16	1.2212453270876722E-15	3	7
41	0.002691701680672269	1.1102230246251565E-16	1.2212453270876722E-15	1	3
54	0.00354516806722689	1.1102230246251565E-16	1.2212453270876722E-15	1	3
66	0.004332983193277311	1.1102230246251565E-16	1.2212453270876722E-15	2	6
112	0.007352941176470588	1.1102230246251565E-16	1.2212453270876722E-15	2	7
328	0.02153361344537815	1.1580337799799167E-8	7.585121258868455E-6	107	319
148	0.009716386554621849	1.5655359697497317E-7	4.073271163851366E-5	18	105
44	0.002888655462184874	2.154642679830232E-7	4.073271163851366E-5	6	28
67	0.004398634453781513	2.4989393643259916E-7	4.073271163851366E-5	19	26
86	0.005646008403361344	1.5914420624651981E-6	2.0311227222069572E-4	13	55
61	0.004004726890756302	1.8634153414742727E-6	2.0311227222069572E-4	5	22
69	0.004529936974789916	4.154506440534611E-6	3.3652865211608596E-4	14	34
168	0.011029411764705883	4.154674717482543E-6	3.3652865211608596E-4	4	33
104	0.006827731092436975	6.340249018688127E-6	4.564979293455451E-4	47	77
76	0.004989495798319327	7.74751500043358E-6	5.035884750281827E-4	28	51
109	0.007155987394957983	8.88393031439616E-6	5.241518885493734E-4	1	1
79	0.00518644957983193	9.930180689954327E-6	5.362297572575336E-4	9	23
127	0.008337710084033613	2.628656124925577E-5	0.0013143280624627884	5	14
70	0.004595588235294118	5.2232736155843895E-5	0.002402705863168819	3	23
44	0.002888655462184874	6.0665543488935825E-5	0.0026086183700242405	2	10
24	0.00157563025210084	7.963772842978223E-5	0.003185509137191289	12	13
31	0.00203518907563025	2.1093830644935974E-4	0.00801565564507567	13	15
15	9.847689075630252E-4	3.8642193542237635E-4	0.013911189675205549	1	4
803	0.05271796218487395	4.3570909703771754E-4	0.014814109299282396	58	338
72	0.004726890756302521	5.799531841663708E-4	0.018558501893323864	3	4
112	0.007352941176470588	5.500002978222618E-4	0.23100012508534995	16	89
6	3.939075630252101E-4	0.0013752458592615202	0.28880163044491924	1	1

29	0.00190388655462184[illegible]	0.002342822247601184	0.2966190495502885	10	20
31	0.00203518907563025[illegible]	0.0028249433290503667	0.2966190495502885	5	13
11	7.221638655462185E-4	0.004489545503146397	0.37712182226429736	4	5
88	0.005777310924369748	0.008307521708997623	0.38761874127627194	9	51
16	0.00105042016806722[illegible]	0.009226572414027578	0.38761874127627194	2	8
17	0.00111607142857142[illegible]	0.010355719915745132	0.38761874127627194	1	5
50	0.003282563025210084	0.010487492598652115	0.38761874127627194	7	38
18	0.00118172268907563[illegible]	0.011542802712238509	0.38761874127627194	2	2
19	0.00124737394957983[illegible]	0.012786717265495184	0.38761874127627194	2	2
22	0.001444327731092437	0.016848636882577428	0.38761874127627194	9	15
22	0.001444327731092437	0.016848636882577428	0.38761874127627194	4	7
69	0.004529936974789916	0.02441062214731804	0.38761874127627194	7	33
27	0.00177258403361344[illegible]	0.024656353778105822	0.38761874127627194	2	10
70	0.004595588235294118	0.025325772500441435	0.38761874127627194	3	31
28	0.001838235294117647	0.026364478976906747	0.38761874127627194	12	28
3	1.9695378151260505E-[illegible]	0.026432727108524956	0.38761874127627194	1	1
3	1.9695378151260505E-[illegible]	0.026432727108524956	0.38761874127627194	1	1
3	1.9695378151260505E-[illegible]	0.026432727108524956	0.38761874127627194	1	1
101	0.00663077731092437	1.1102230246251565E-1[illegible]	3.4416913763379853E-1[illegible]	1	1
106	0.006959033613445379	1.1102230246251565E-1[illegible]	3.4416913763379853E-1[illegible]	2	2
114	0.007484243697478992	1.1102230246251565E-1[illegible]	3.4416913763379853E-1[illegible]	2	2
119	0.0078125	1.1102230246251565E-1[illegible]	3.4416913763379853E-1[illegible]	5	5
120	0.007878151260504201	1.1102230246251565E-1[illegible]	3.4416913763379853E-1[illegible]	3	3
120	0.007878151260504201	1.1102230246251565E-1[illegible]	3.4416913763379853E-1[illegible]	3	3
62	0.00407037815126050[illegible]	1.1102230246251565E-1[illegible]	3.4416913763379853E-1[illegible]	2	2
64	0.004201680672268907	1.1102230246251565E-1[illegible]	3.4416913763379853E-1[illegible]	2	2
102	0.006696428571428571	1.1102230246251565E-1[illegible]	3.4416913763379853E-1[illegible]	8	9
189	0.012408088235294117	1.1102230246251565E-1[illegible]	3.4416913763379853E-1[illegible]	6	7
124	0.008140756302521009	1.1102230246251565E-1[illegible]	3.4416913763379853E-1[illegible]	5	6
97	0.006368172268907563	1.1102230246251565E-1[illegible]	3.4416913763379853E-1[illegible]	4	5
124	0.008140756302521009	1.1102230246251565E-1[illegible]	3.4416913763379853E-1[illegible]	4	5

41	0.002691701680672269	1.1102230246251565E-1(	3.4416913763379853E-1!	2	3
130	0.008534663865546218	1.1102230246251565E-1(	3.4416913763379853E-1!	13	21
106	0.006959033613445379	1.1102230246251565E-1(	3.4416913763379853E-1!	3	5
130	0.008534663865546218	1.1102230246251565E-1(	3.4416913763379853E-1!	10	18
207	0.013589810924369748	1.1102230246251565E-1(	3.4416913763379853E-1!	7	15
247	0.016215861344537816	1.1102230246251565E-1(	3.4416913763379853E-1!	8	21
115	0.007549894957983193	1.1102230246251565E-1(	3.4416913763379853E-1!	6	16
101	0.006531298499741335	1.1102230246251565E-1(	2.7755575615628914E-1!	1	1
106	0.006854630108639421	1.1102230246251565E-1(	2.7755575615628914E-1!	2	2
114	0.007371960682876358	1.1102230246251565E-1(	2.7755575615628914E-1!	2	2
120	0.007759958613554061	1.1102230246251565E-1(	2.7755575615628914E-1!	3	3
119	0.007695292291774444	1.1102230246251565E-1(	2.7755575615628914E-1!	5	5
120	0.007759958613554061	1.1102230246251565E-1(	2.7755575615628914E-1!	3	3
124	0.00801862390067253	1.1102230246251565E-1(	2.7755575615628914E-1!	6	6
124	0.00801862390067253	1.1102230246251565E-1(	2.7755575615628914E-1!	5	5
62	0.004009311950336265	1.1102230246251565E-1(	2.7755575615628914E-1!	2	2
64	0.004138644593895499	1.1102230246251565E-1(	2.7755575615628914E-1!	2	2
102	0.006595964821520952	1.1102230246251565E-1(	2.7755575615628914E-1!	8	9
189	0.012221934816347646	1.1102230246251565E-1(	2.7755575615628914E-1!	6	7
97	0.006272633212622866	1.1102230246251565E-1(	2.7755575615628914E-1!	4	5
41	0.0026513191929643043	1.1102230246251565E-1(	2.7755575615628914E-1!	2	3
130	0.008406621831350233	1.1102230246251565E-1(	2.7755575615628914E-1!	13	21
106	0.006854630108639421	1.1102230246251565E-1(	2.7755575615628914E-1!	3	5
130	0.008406621831350233	1.1102230246251565E-1(	2.7755575615628914E-1!	10	18
207	0.013385928608380756	1.1102230246251565E-1(	2.7755575615628914E-1!	7	15
339	0.02192188308329022	1.1102230246251565E-1(	2.7755575615628914E-1!	42	99
247	0.015972581479565442	1.1102230246251565E-1(	2.7755575615628914E-1!	8	21
1039	0.06821165966386554	1.1102230246251565E-1(	1.3999912340523224E-1:	354	745
2627	0.1724658613445378	1.5420997812043424E-1:	9.715228621587357E-11	638	1664
322	0.021139705882352942	7.673861546209082E-13	3.2230218494078144E-1(	47	79
129	0.008469012605042016	4.546918397352329E-12	1.4322792951659835E-9	13	25

658	0.043198529411764705	6.764566684580586E-11	1.7046708045143077E-8	254	505
96	0.00630252100840336[illegible]	4.469261627448873E-10	9.385449417642633E-8	80	114
84	0.00551470588235294[illegible]	3.344056920262517E-9	6.013019619999227E-7	48	56
73	0.004792542016806723	3.829948802547278E-9	6.013019619999227E-7	36	36
17	0.00111607142857142[illegible]	6.579419973284928E-7	9.211187962598899E-5	20	20
17	0.00111607142857142[illegible]	9.972444260042401E-6	0.0012565279767653426	1	2
161	0.010569852941176471	1.6161995935792817E-5	0.0017664413738904816	19	35
199	0.013064600840336135	1.682325117990935E-5	0.0017664413738904816	23	90
52	0.00341386554621848[illegible]	1.94907758134244E-5	0.0018906052539021667	9	26
13	8.534663865546219E-4	3.711505889580913E-5	0.0033403553006228215	5	12
104	0.006827731092436975	4.570223071931334E-5	0.0038389873804223207	23	44
237	0.015559348739495799	4.96944648198383E-5	0.0038761682559473876	69	193
259	0.017003676470588234	5.27692236929056E-5	0.0039049225532750143	125	132
708	0.04648109243697479	1.0294087793272233E-4	0.007205861455290563	105	203
192	0.012605042016806723	1.2492003108588445E-4	0.00824421973112388	52	142
135	0.008862920168067227	1.3459989772779402E-4	0.00824421973112388	30	52
101	0.00663077731092437	1.110223024625156[illegible]	1.887379141862766E-15	1	1
106	0.006959033613445379	1.110223024625156[illegible]	1.887379141862766E-15	2	2
114	0.007484243697478992	1.110223024625156[illegible]	1.887379141862766E-15	2	2
119	0.0078125	1.110223024625156[illegible]	1.887379141862766E-15	5	5
120	0.007878151260504201	1.110223024625156[illegible]	1.887379141862766E-15	3	3
120	0.007878151260504201	1.110223024625156[illegible]	1.887379141862766E-15	3	3
62	0.00407037815126050[illegible]	1.110223024625156[illegible]	1.887379141862766E-15	2	2
64	0.004201680672268907	1.110223024625156[illegible]	1.887379141862766E-15	2	2
189	0.012408088235294117	1.110223024625156[illegible]	1.887379141862766E-15	6	7
124	0.008140756302521009	1.110223024625156[illegible]	1.887379141862766E-15	5	6
97	0.006368172268907563	1.110223024625156[illegible]	1.887379141862766E-15	4	5
124	0.008140756302521009	1.110223024625156[illegible]	1.887379141862766E-15	4	5
102	0.006696428571428571	1.110223024625156[illegible]	1.887379141862766E-15	6	9
130	0.008534663865546218	1.110223024625156[illegible]	1.887379141862766E-15	13	21
106	0.006959033613445379	1.110223024625156[illegible]	1.887379141862766E-15	3	5

130	0.008534663865546218	1.1102230246251565E-1(	1.887379141862766E-15	10	18
207	0.013589810924369748	1.1102230246251565E-1(	1.887379141862766E-15	7	15
41	0.002691701680672269	1.1102230246251565E-1(	1.887379141862766E-15	1	3
54	0.0035451680672268907	1.1102230246251565E-1(	1.887379141862766E-15	1	3
66	0.004332983193277311	1.1102230246251565E-1(	1.887379141862766E-15	2	6
29	0.0019038865546218487	2.0410886244937032E-5	0.005620864311639329	11	20
66	0.004332983193277311	3.1577889391232183E-5	0.005620864311639329	6	40
73	0.004792542016806723	5.0761245547925604E-5	0.006040588220203147	36	36
84	0.0055147058823529415	9.779108501384304E-5	0.0073356242563688	41	56
85	0.005580357142857143	1.0331865149815211E-4	0.0073356242563688	6	60
96	0.0063025210084033615	1.813190303793233E-4	0.010697822792380074	55	114
12	7.878151260504202E-4	0.001976571566166374	0.10080514987448508	2	2
658	0.043198529411764705	0.0028180367897467917	0.1191953602008683	75	505
15	9.847689075630252E-4	0.003056291287201751	0.1191953602008683	1	5
17	0.0011160714285714285	0.0038984130194013034	0.13644445567904562	2	10
19	0.0012473739495798318	0.004835900905560853	0.150875643828709	2	4
20	0.0013130252100840337	0.005339753118407176	0.150875643828709	7	9
213	0.013983718487394959	0.005989916253452354	0.150875643828709	10	10
22	0.001444327731092437	0.006416384025712429	0.150875643828709	6	20
70	0.004595588235294118	0.006559810601248217	0.150875643828709	6	27
23	0.0015099789915966387	0.006988652806778517	0.15375036174912737	2	11
224	0.014705882352941176	0.007361857167200836	0.15459900051121755	15	15
27	0.0017725840336134454	0.009498228196331504	0.18046633573029858	1	9
28	0.001838235294117647	0.010179512252093015	0.18323122053767427	4	28
32	0.0021008403361344537	0.01311294650257211	0.22292009054372586	7	17
101	0.006531298499741335	1.1102230246251565E-1(	1.4432899320127035E-1!	1	1
106	0.006854630108639421	1.1102230246251565E-1(	1.4432899320127035E-1!	2	2
114	0.007371960682876358	1.1102230246251565E-1(	1.4432899320127035E-1!	2	2
119	0.007695292291774444	1.1102230246251565E-1(	1.4432899320127035E-1!	5	5
120	0.007759958613554061	1.1102230246251565E-1(	1.4432899320127035E-1!	3	3
62	0.004009311950336265	1.1102230246251565E-1(	1.4432899320127035E-1!	2	2

64	0.0041386445938954999	1.1102230246251565E-16	1.4432899320127035E-15	2	2
124	0.00801862390067253	1.1102230246251565E-16	1.4432899320127035E-15	5	6
97	0.00627263321262286	1.1102230246251565E-16	1.4432899320127035E-15	4	5
124	0.00801862390067253	1.1102230246251565E-16	1.4432899320127035E-15	4	5
120	0.00775995861355406	1.1102230246251565E-16	1.4432899320127035E-15	2	3
106	0.00685463010863942	1.1102230246251565E-16	1.4432899320127035E-15	3	5
130	0.00840662183135023	1.1102230246251565E-16	1.4432899320127035E-15	12	21
130	0.00840662183135023	1.1102230246251565E-16	1.4432899320127035E-15	10	18
102	0.00659596482152095	1.1102230246251565E-16	1.4432899320127035E-15	4	9
189	0.01222193481634764	1.1102230246251565E-16	1.4432899320127035E-15	3	7
339	0.02192188308329022	1.1102230246251565E-16	1.4432899320127035E-15	37	99
41	0.00265131919296430	1.1102230246251565E-16	1.4432899320127035E-15	1	3
54	0.00349198137609932	1.1102230246251565E-16	1.4432899320127035E-15	1	3
66	0.00426797723745473	1.1102230246251565E-16	1.4432899320127035E-15	2	6
254	0.01667542016806722	2.071313802942676E-5	0.01331854775292140	29	85
254	0.01667542016806722	2.071313802942676E-5	0.01331854775292140	29	85
11	7.221638655462185E-4	5.400848222256404E-5	0.0231156303911257	6	7
39	0.00256039915966386	9.567262535470356E-5	0.03019439195027373	13	14
13	8.534663865546219E-4	1.1748790642129858E-4	0.03019439195027373	2	3
18	0.00118172268907563	5.17338066519657E-4	0.1107103462352065	5	9
48	0.00315126050420168	0.00186310826424018	0.280332804412344	11	22
25	0.00164128151260504	0.00218561529068594	0.280332804412344	9	10
2	1.3130252100840336E-4	0.00240257061998638	0.280332804412344	1	1
65	0.00426733193277310	0.00257918777956489	0.280332804412344	9	13
80	0.00525210084033613	0.00259513922805942	0.280332804412344	15	19
39	0.00256039915966386	0.00299134548992097	0.280332804412344	13	15
39	0.00256039915966386	0.00299134548992097	0.280332804412344	4	5
8	5.252100840336134E-4	0.00308058026826751	0.280332804412344	1	1
17	0.00111607142857142	0.00340681815982346	0.289579543584994	3	3
70	0.00459558823529411	0.00401125850496331	0.3017557215282604	5	25
29	0.00190388655462184	0.00409352669994167	0.3017557215282604	3	4

9	5.908613445378151E-4	0.004273243107789537	0.30175572152826047	2	5
30	0.00196953781512605[illegible]	0.004713051790545486	0.30175572152826047	5	11
460	0.030199579831932773	0.004720194098156827	0.30175572152826047	38	91
11	7.221638655462185E-4	3.1286197232915924E-6	0.004455154485967228	7	7
86	0.005646008403361344	7.727772416643575E-6	0.005502173960650225	2	2
254	0.016675420168067227	3.8277386240737066E-5	0.013626749501702395	33	85
254	0.016675420168067227	3.8277386240737066E-5	0.013626749501702395	33	85
100	0.006565126050420168	1.6285585139907965E-4	0.04625106179733862	16	77
259	0.017003676470588234	2.7932699273380823E-4	0.06406499990712478	126	132
39	0.00256039915966386[illegible]	3.4883536297269924E-4	0.06406499990712478	5	5
6	3.939075630252101E-4	3.5991572981530773E-4	0.06406499990712478	2	2
88	0.005777310924369748	4.3063697195855344E-4	0.06804064156945144	25	32
69	0.004529936974789916	5.039681996260592E-4	0.07156348434690041	17	33
7	4.5955882352941176E-4	6.389041367790282E-4	0.08241863364449464	4	4
13	8.534663865546219E-4	8.076689231603096E-4	0.08852913249664118	2	3
105	0.006893382352941176	8.121938761159742E-4	0.08852913249664118	54	59
460	0.030199579831932773	9.621405803755811E-4	0.09251408442865516	49	91
28	0.001838235294117647	9.841923875388847E-4	0.09251408442865516	2	2
4	2.626050420168067E-4	0.0014474195066881412	0.1151542654615445	2	2
708	0.04648109243697479	0.001514376058529554	0.1151542654615445	88	203
39	0.00256039915966386[illegible]	0.001535607201444189	0.1151542654615445	7	14
724	0.047531512605042014	0.001556138722453304	0.1151542654615445	90	212
70	0.004595588235294118	0.00187624524712926	0.13321341254617747	12	25

Reactions ratio	Species identifie	Species na
3.494792758789404E-4	9606	Homo sapie
6.290626965820927E-4	9606	Homo sapie
6.989585517578807E-5	9606	Homo sapie
1.3979171035157615E-4	9606	Homo sapie
3.494792758789404E-4	9606	Homo sapie
1.3979171035157615E-4	9606	Homo sapie
2.0968756552736424E-4	9606	Homo sapie
2.0968756552736424E-4	9606	Homo sapie
1.3979171035157615E-4	9606	Homo sapie
1.3979171035157615E-4	9606	Homo sapie
4.892709862305165E-4	9606	Homo sapie
4.193751310547285E-4	9606	Homo sapie
3.494792758789404E-4	9606	Homo sapie
2.0968756552736424E-4	9606	Homo sapie
0.0014678129586915495	9606	Homo sapie
3.494792758789404E-4	9606	Homo sapie
0.0012581253931641854	9606	Homo sapie
0.001957083944922066	9606	Homo sapie
0.0010484378276368212	9606	Homo sapie
0.006919689662403019	9606	Homo sapie
6.989585517578807E-5	9606	Homo sapie
2.0968756552736424E-4	9606	Homo sapie
1.3979171035157615E-4	9606	Homo sapie
1.3979171035157615E-4	9606	Homo sapie
3.494792758789404E-4	9606	Homo sapie
2.0968756552736424E-4	9606	Homo sapie
2.0968756552736424E-4	9606	Homo sapie
1.3979171035157615E-4	9606	Homo sapie
1.3979171035157615E-4	9606	Homo sapie

6.290626965820927E-4	9606	Homo sapie
4.892709862305165E-4	9606	Homo sapie
4.193751310547285E-4	9606	Homo sapie
3.494792758789404E-4	9606	Homo sapie
3.494792758789404E-4	9606	Homo sapie
0.0014678129586915495	9606	Homo sapie
3.494792758789404E-4	9606	Homo sapie
0.0012581253931641854	9606	Homo sapie
0.0010484378276368212	9606	Homo sapie
0.006919689662403019	9606	Homo sapie
2.0968756552736424E-4	9606	Homo sapie
3.494792758789404E-4	9606	Homo sapie
0.008317606765918781	9606	Homo sapie
0.0040539596001957084	9606	Homo sapie
0.0013979171035157615	9606	Homo sapie
0.0032152093380862514	9606	Homo sapie
0.0012581253931641854	9606	Homo sapie
0.05207241210596212	9606	Homo sapie
0.004123855455371497	9606	Homo sapie
0.0028657300622073113	9606	Homo sapie
0.03529740686377298	9606	Homo sapie
0.001887188089746278	9606	Homo sapie
0.002586146641504159	9606	Homo sapie
0.00510239742783253	9606	Homo sapie
0.0013979171035157615	9606	Homo sapie
0.004892709862305165	9606	Homo sapie
0.0050325015726567415	9606	Homo sapie
0.00216677151044943	9606	Homo sapie
0.11630670301251136	9606	Homo sapie
0.0025162507863283707	9606	Homo sapie
0.007129377227930384	9606	Homo sapie

6.792094002580996E-5	9606	Homo sapie
1.3584188005161992E-4	9606	Homo sapie
1.3584188005161992E-4	9606	Homo sapie
3.396047001290498E-4	9606	Homo sapie
2.0376282007742986E-4	9606	Homo sapie
2.0376282007742986E-4	9606	Homo sapie
1.3584188005161992E-4	9606	Homo sapie
1.3584188005161992E-4	9606	Homo sapie
4.754465801806697E-4	9606	Homo sapie
4.0752564015485973E-4	9606	Homo sapie
3.396047001290498E-4	9606	Homo sapie
3.396047001290498E-4	9606	Homo sapie
3.396047001290498E-4	9606	Homo sapie
6.112884602322896E-4	9606	Homo sapie
2.0376282007742986E-4	9606	Homo sapie
0.001426339740542009	9606	Homo sapie
0.0012225769204645793	9606	Homo sapie
0.0010188141003871494	9606	Homo sapie
0.006724173062555186	9606	Homo sapie
0.001426339740542009	9606	Homo sapie
0.03529740686377298	9606	Homo sapie
0.11630670301251136	9606	Homo sapie
0.05207241210596212	9606	Homo sapie
0.0028657300622073113	9606	Homo sapie
0.0032851051932620396	9606	Homo sapie
6.290626965820927E-4	9606	Homo sapie
0.00181729223457049	9606	Homo sapie
4.892709862305165E-4	9606	Homo sapie
0.00363458446914098	9606	Homo sapie
9.78541972461033E-4	9606	Homo sapie
0.0030055217725588872	9606	Homo sapie

6.989585517578807E-5	9606	Homo sapie
0.002725938351855735	9606	Homo sapie
1.3979171035157615E-4	9606	Homo sapie
0.0025162507863283707	9606	Homo sapie
4.193751310547285E-4	9606	Homo sapie
0.003914167889844132	9606	Homo sapie
0.0010484378276368212	9606	Homo sapie
0.00796812749003984	9606	Homo sapie
6.989585517578807E-5	9606	Homo sapie
0.001887188089746278	9606	Homo sapie
0.0021667715104494 3	9606	Homo sapie
4.193751310547285E-4	9606	Homo sapie
2.0968756552736424E-4	9606	Homo sapie
2.795834207031523E-4	9606	Homo sapie
0.005941147689941986	9606	Homo sapie
0.005941147689941986	9606	Homo sapie
4.193751310547285E-4	9606	Homo sapie
0.006360522820996715	9606	Homo sapie
0.0013280212483399733	9606	Homo sapie
2.0968756552736424E-4	9606	Homo sapie
2.0968756552736424E-4	9606	Homo sapie
0.001887188089746278	9606	Homo sapie
2.0968756552736424E-4	9606	Homo sapie
1.3979171035157615E-4	9606	Homo sapie
4.193751310547285E-4	9606	Homo sapie
4.193751310547285E-4	9606	Homo sapie
0.01418885860068498	9606	Homo sapie
0.014817921297267072	9606	Homo sapie
0.0023065632208010066	9606	Homo sapie
2.0968756552736424E-4	9606	Homo sapie
0.005941147689941986	9606	Homo sapie

0.00594114768994198€	9606	Homo sapie
0.00125812539316418⁄	9606	Homo sapie
0.001467812958691549	9606	Homo sapie
0.001467812958691549	9606	Homo sapie
0.001467812958691549	9606	Homo sapie
0.001467812958691549	9606	Homo sapie
0.002725938351855735	9606	Homo sapie
2.795834207031523E-4	9606	Homo sapie
0.0334102187740267	9606	Homo sapie
7.688544069336689E-4	9606	Homo sapie
3.494792758789404E-4	9606	Homo sapie
0.004683022296777801	9606	Homo sapie
0.014678129586915495	9606	Homo sapie
3.494792758789404E-4	9606	Homo sapie
3.494792758789404E-4	9606	Homo sapie
3.494792758789404E-4	9606	Homo sapie
3.494792758789404E-4	9606	Homo sapie
0.0011183336828126092	9606	Homo sapie
6.457167455876022E-4	9606	Homo sapie
0.002008896541828096	9606	Homo sapie
0.0024393743722198307	9606	Homo sapie
7.892093557181805E-4	9606	Homo sapie
0.001004448270914048	9606	Homo sapie
0.008394317692638828	9606	Homo sapie
0.023891519586741284	9606	Homo sapie
0.024250251112067727	9606	Homo sapie
0.0038442720346683443	9606	Homo sapie
0.007339064793457748	9606	Homo sapie
4.892709862305165E-4	9606	Homo sapie
2.0968756552736424E-4	9606	Homo sapie
9.78541972461033E-4	9606	Homo sapie

0.022296777801076396	9606	Homo sapie
0.001957083944922066	9606	Homo sapie
0.0023764590759767948	9606	Homo sapie
1.3979171035157615E-4	9606	Homo sapie
0.00181729223457049	9606	Homo sapie
4.193751310547285E-4	9606	Homo sapie
0.003564688613965192	9606	Homo sapie
6.989585517578807E-5	9606	Homo sapie
2.0968756552736424E-4	9606	Homo sapie
0.001677500524218914	9606	Homo sapie
0.0015377088138673377	9606	Homo sapie
2.795834207031523E-4	9606	Homo sapie
1.3979171035157615E-4	9606	Homo sapie
0.005381980848535682	9606	Homo sapie
0.0016076046690431257	9606	Homo sapie
0.004123855455371497	9606	Homo sapie
0.0334102187740267	9606	Homo sapie
0.005941147689941986	9606	Homo sapie
0.005941147689941986	9606	Homo sapie
1.3979171035157615E-4	9606	Homo sapie
0.0023065632208010066	9606	Homo sapie
0.006220731110645139	9606	Homo sapie
9.78541972461033E-4	9606	Homo sapie
4.892709862305165E-4	9606	Homo sapie
0.005381980848535682	9606	Homo sapie
4.193751310547285E-4	9606	Homo sapie
0.0032152093380862514	9606	Homo sapie
0.0015377088138673377	9606	Homo sapie
4.193751310547285E-4	9606	Homo sapie
2.795834207031523E-4	9606	Homo sapie
2.0968756552736424E-4	9606	Homo sapie

0.009226252883204026	9606	Homo sapie
0.00174739637939470[illegible]	9606	Homo sapie
6.989585517578807E-5	9606	Homo sapie
1.3979171035157615E-4	9606	Homo sapie
6.989585517578807E-5	9606	Homo sapie
1.3979171035157615E-4	9606	Homo sapie
1.3979171035157615E-4	9606	Homo sapie
3.494792758789404E-4	9606	Homo sapie
2.0968756552736424E-4	9606	Homo sapie
2.0968756552736424E-4	9606	Homo sapie
4.892709862305165E-4	9606	Homo sapie
4.193751310547285E-4	9606	Homo sapie
3.494792758789404E-4	9606	Homo sapie
3.494792758789404E-4	9606	Homo sapie
3.494792758789404E-4	9606	Homo sapie
6.290626965820927E-4	9606	Homo sapie
0.00146781295869154[illegible]	9606	Homo sapie
3.494792758789404E-4	9606	Homo sapie
0.00125812539316418[illegible]	9606	Homo sapie
0.00104843782763682[illegible]	9606	Homo sapie
0.00146781295869154[illegible]	9606	Homo sapie
2.0968756552736424E-4	9606	Homo sapie
4.892709862305165E-4	9606	Homo sapie
0.006919689662403019	9606	Homo sapie
2.0968756552736424E-4	9606	Homo sapie
6.989585517578807E-5	9606	Homo sapie
1.3979171035157615E-4	9606	Homo sapie
3.494792758789404E-4	9606	Homo sapie
1.3979171035157615E-4	9606	Homo sapie
2.0968756552736424E-4	9606	Homo sapie
2.0968756552736424E-4	9606	Homo sapie

1.3979171035157615E-4	9606	Homo sapie
1.3979171035157615E-4	9606	Homo sapie
6.290626965820927E-4	9606	Homo sapie
4.892709862305165E-4	9606	Homo sapie
4.193751310547285E-4	9606	Homo sapie
3.494792758789404E-4	9606	Homo sapie
3.494792758789404E-4	9606	Homo sapie
0.0014678129586915495	9606	Homo sapie
3.494792758789404E-4	9606	Homo sapie
0.0012581253931641854	9606	Homo sapie
0.0010484378276368212	9606	Homo sapie
0.0014678129586915495	9606	Homo sapie
2.0968756552736424E-4	9606	Homo sapie
4.193751310547285E-4	9606	Homo sapie
2.0968756552736424E-4	9606	Homo sapie
0.003774376179492556	9606	Homo sapie
0.00216677151044943	9606	Homo sapie
0.0014678129586915495	9606	Homo sapie
9.08646117285245E-4	9606	Homo sapie
0.0013280212483399733	9606	Homo sapie
0.004683022296777801	9606	Homo sapie
4.193751310547285E-4	9606	Homo sapie
2.795834207031523E-4	9606	Homo sapie
5.591668414063046E-4	9606	Homo sapie
3.494792758789404E-4	9606	Homo sapie
0.0011183336828126092	9606	Homo sapie
3.494792758789404E-4	9606	Homo sapie
0.008107919200391417	9606	Homo sapie
0.0011183336828126092	9606	Homo sapie
0.0011882295379883974	9606	Homo sapie
0.0024463549311525825	9606	Homo sapie

7.688544069336689E-4	9606	Homo sapie
0.00181729223457049	9606	Homo sapie
6.989585517578807E-5	9606	Homo sapie
1.3979171035157615E-4	9606	Homo sapie
1.3979171035157615E-4	9606	Homo sapie
3.494792758789404E-4	9606	Homo sapie
2.0968756552736424E-4	9606	Homo sapie
1.3979171035157615E-4	9606	Homo sapie
1.3979171035157615E-4	9606	Homo sapie
4.892709862305165E-4	9606	Homo sapie
4.193751310547285E-4	9606	Homo sapie
3.494792758789404E-4	9606	Homo sapie
3.494792758789404E-4	9606	Homo sapie
3.494792758789404E-4	9606	Homo sapie
2.0968756552736424E-4	9606	Homo sapie
5.591668414063046E-4	9606	Homo sapie
3.494792758789404E-4	9606	Homo sapie
4.892709862305165E-4	9606	Homo sapie
0.001467812958691549	9606	Homo sapie
0.001258125393164185	9606	Homo sapie
0.002026979800097854	9606	Homo sapie
9.78541972461033E-4	9606	Homo sapie
0.003285105193262039	9606	Homo sapie
2.0968756552736424E-4	9606	Homo sapie
0.03529740686377298	9606	Homo sapie
0.05207241210596212	9606	Homo sapie
9.78541972461033E-4	9606	Homo sapie
0.004193751310547285	9606	Homo sapie
0.002935625917383099	9606	Homo sapie
0.001397917103515761	9606	Homo sapie
0.013699587614454462	9606	Homo sapie

1.3979171035157615E-4	9606	Homo sapie
0.01810302649052911	9606	Homo sapie
2.795834207031523E-4	9606	Homo sapie
3.494792758789404E-4	9606	Homo sapie
0.003704480324316768	9606	Homo sapie
0.008317606765918781	9606	Homo sapie
5.591668414063046E-4	9606	Homo sapie
6.989585517578808E-4	9606	Homo sapie
0.0013979171035157615	9606	Homo sapie
4.892709862305165E-4	9606	Homo sapie
2.795834207031523E-4	9606	Homo sapie
2.0968756552736424E-4	9606	Homo sapie
4.193751310547285E-4	9606	Homo sapie
0.001887188089746278	9606	Homo sapie
0.00216677151044943	9606	Homo sapie
0.00216677151044943	9606	Homo sapie
0.0013280212483399733	9606	Homo sapie
0.0011882295379883974	9606	Homo sapie
4.892709862305165E-4	9606	Homo sapie
8.38750262109457E-4	9606	Homo sapie
0.003774376179492556	9606	Homo sapie
0.0013979171035157615	9606	Homo sapie
9.08646117285245E-4	9606	Homo sapie
4.892709862305165E-4	9606	Homo sapie
4.193751310547285E-4	9606	Homo sapie
0.004683022296777801	9606	Homo sapie
0.0011882295379883974	9606	Homo sapie
3.494792758789404E-4	9606	Homo sapie
6.989585517578808E-4	9606	Homo sapie
0.0025162507863283707	9606	Homo sapie
6.290626965820927E-4	9606	Homo sapie

4.892709862305165E-4	9606	Homo sapie
2.0968756552736424E-4	9606	Homo sapie
9.78541972461033E-4	9606	Homo sapie
0.005381980848535682	9606	Homo sapie
1.3979171035157615E-4	9606	Homo sapie
3.494792758789404E-4	9606	Homo sapie
3.494792758789404E-4	9606	Homo sapie
3.494792758789404E-4	9606	Homo sapie
3.494792758789404E-4	9606	Homo sapie
0.009226252883204026	9606	Homo sapie
6.989585517578807E-5	9606	Homo sapie
4.193751310547285E-4	9606	Homo sapie
6.989585517578807E-5	9606	Homo sapie
3.494792758789404E-4	9606	Homo sapie
6.989585517578807E-5	9606	Homo sapie
0.004123855455371497	9606	Homo sapie
0.00174739637939470[illegible]	9606	Homo sapie
0.00139791710351576[illegible]	9606	Homo sapie
6.989585517578808E-4	9606	Homo sapie
4.892709862305165E-4	9606	Homo sapie
6.989585517578807E-5	9606	Homo sapie
1.3979171035157615E-4	9606	Homo sapie
1.3979171035157615E-4	9606	Homo sapie
3.494792758789404E-4	9606	Homo sapie
2.0968756552736424E-4	9606	Homo sapie
1.3979171035157615E-4	9606	Homo sapie
1.3979171035157615E-4	9606	Homo sapie
4.193751310547285E-4	9606	Homo sapie
3.494792758789404E-4	9606	Homo sapie
3.494792758789404E-4	9606	Homo sapie
2.0968756552736424E-4	9606	Homo sapie

3.494792758789404E-4	9606	Homo sapie
0.001467812958691549	9606	Homo sapie
0.001258125393164185	9606	Homo sapie
6.290626965820927E-4	9606	Homo sapie
4.892709862305165E-4	9606	Homo sapie
2.0968756552736424E-4	9606	Homo sapie
2.0968756552736424E-4	9606	Homo sapie
4.193751310547285E-4	9606	Homo sapie
4.892709862305165E-4	9606	Homo sapie
0.022296777801076396	9606	Homo sapie
0.007339064793457748	9606	Homo sapie
0.001957083944922066	9606	Homo sapie
0.00181729223457049	9606	Homo sapie
0.003844272034668344	9606	Homo sapie
0.001537708813867337	9606	Homo sapie
0.002376459075976794	9606	Homo sapie
0.002306563220801006	9606	Homo sapie
0.005381980848535682	9606	Homo sapie
0.003564688613965192	9606	Homo sapie
6.989585517578807E-5	9606	Homo sapie
0.001607604669043125	9606	Homo sapie
9.78541972461033E-4	9606	Homo sapie
0.001607604669043125	9606	Homo sapie
6.989585517578808E-4	9606	Homo sapie
9.08646117285245E-4	9606	Homo sapie
0.001048437827636821	9606	Homo sapie
2.795834207031523E-4	9606	Homo sapie
0.02362479904941637	9606	Homo sapie
2.795834207031523E-4	9606	Homo sapie
0.006220731110645139	9606	Homo sapie
6.989585517578807E-5	9606	Homo sapie

0.0013979171035157615	9606	Homo sapie
9.08646117285245E-4	9606	Homo sapie
3.494792758789404E-4	9606	Homo sapie
0.003564688613965192	9606	Homo sapie
5.591668414063046E-4	9606	Homo sapie
3.494792758789404E-4	9606	Homo sapie
0.0026560424966799467	9606	Homo sapie
1.3979171035157615E-4	9606	Homo sapie
1.3979171035157615E-4	9606	Homo sapie
0.0010484378276368212	9606	Homo sapie
4.892709862305165E-4	9606	Homo sapie
0.0023065632208010066	9606	Homo sapie
6.989585517578808E-4	9606	Homo sapie
0.00216677151044943	9606	Homo sapie
0.001957083944922066	9606	Homo sapie
6.989585517578807E-5	9606	Homo sapie
6.989585517578807E-5	9606	Homo sapie
6.989585517578807E-5	9606	Homo sapie
6.989585517578807E-5	9606	Homo sapie
1.3979171035157615E-4	9606	Homo sapie
1.3979171035157615E-4	9606	Homo sapie
3.494792758789404E-4	9606	Homo sapie
2.0968756552736424E-4	9606	Homo sapie
2.0968756552736424E-4	9606	Homo sapie
1.3979171035157615E-4	9606	Homo sapie
1.3979171035157615E-4	9606	Homo sapie
6.290626965820927E-4	9606	Homo sapie
4.892709862305165E-4	9606	Homo sapie
4.193751310547285E-4	9606	Homo sapie
3.494792758789404E-4	9606	Homo sapie
3.494792758789404E-4	9606	Homo sapie

2.0968756552736424E-4	9606	Homo sapie
0.0014678129586915495	9606	Homo sapie
3.494792758789404E-4	9606	Homo sapie
0.0012581253931641854	9606	Homo sapie
0.0010484378276368212	9606	Homo sapie
0.0014678129586915495	9606	Homo sapie
0.0011183336828126092	9606	Homo sapie
6.792094002580996E-5	9606	Homo sapie
1.3584188005161992E-4	9606	Homo sapie
1.3584188005161992E-4	9606	Homo sapie
2.0376282007742986E-4	9606	Homo sapie
3.396047001290498E-4	9606	Homo sapie
2.0376282007742986E-4	9606	Homo sapie
4.0752564015485973E-4	9606	Homo sapie
3.396047001290498E-4	9606	Homo sapie
1.3584188005161992E-4	9606	Homo sapie
1.3584188005161992E-4	9606	Homo sapie
6.112884602322896E-4	9606	Homo sapie
4.754465801806697E-4	9606	Homo sapie
3.396047001290498E-4	9606	Homo sapie
2.0376282007742986E-4	9606	Homo sapie
0.001426339740542009	9606	Homo sapie
3.396047001290498E-4	9606	Homo sapie
0.0012225769204645793	9606	Homo sapie
0.0010188141003871494	9606	Homo sapie
0.006724173062555186	9606	Homo sapie
0.001426339740542009	9606	Homo sapie
0.05207241210596212	9606	Homo sapie
0.11630670301251136	9606	Homo sapie
0.005521772558887258	9606	Homo sapie
0.0017473963793947019	9606	Homo sapie

0.03529740686377298	9606	Homo sapie
0.00796812749003984	9606	Homo sapie
0.003914167889844132	9606	Homo sapie
0.002516250786328707	9606	Homo sapie
0.00139791710351576[illegible]	9606	Homo sapie
1.3979171035157615E-4	9606	Homo sapie
0.00244635493115258[illegible]	9606	Homo sapie
0.006290626965820926	9606	Homo sapie
0.00181729223457049	9606	Homo sapie
8.38750262109457E-4	9606	Homo sapie
0.00307541762773467[illegible]	9606	Homo sapie
0.0134899000489271	9606	Homo sapie
0.009226252883204026	9606	Homo sapie
0.01418885860068498	9606	Homo sapie
0.009925211434961907	9606	Homo sapie
0.00363458446914098	9606	Homo sapie
6.989585517578807E-5	9606	Homo sapie
1.3979171035157615E-4	9606	Homo sapie
1.3979171035157615E-4	9606	Homo sapie
3.494792758789404E-4	9606	Homo sapie
2.0968756552736424E-4	9606	Homo sapie
2.0968756552736424E-4	9606	Homo sapie
1.3979171035157615E-4	9606	Homo sapie
1.3979171035157615E-4	9606	Homo sapie
4.892709862305165E-4	9606	Homo sapie
4.193751310547285E-4	9606	Homo sapie
3.494792758789404E-4	9606	Homo sapie
3.494792758789404E-4	9606	Homo sapie
6.290626965820927E-4	9606	Homo sapie
0.00146781295869154[illegible]	9606	Homo sapie
3.494792758789404E-4	9606	Homo sapie

0.0012581253931641854	9606	Homo sapie
0.0010484378276368212	9606	Homo sapie
2.0968756552736424E-4	9606	Homo sapie
2.0968756552736424E-4	9606	Homo sapie
4.193751310547285E-4	9606	Homo sapie
0.0013979171035157615	9606	Homo sapie
0.002795834207031523	9606	Homo sapie
0.0025162507863283707	9606	Homo sapie
0.003914167889844132	9606	Homo sapie
0.004193751310547285	9606	Homo sapie
0.00796812749003984	9606	Homo sapie
1.3979171035157615E-4	9606	Homo sapie
0.03529740686377298	9606	Homo sapie
3.494792758789404E-4	9606	Homo sapie
6.989585517578808E-4	9606	Homo sapie
2.795834207031523E-4	9606	Homo sapie
6.290626965820927E-4	9606	Homo sapie
6.989585517578808E-4	9606	Homo sapie
0.0013979171035157615	9606	Homo sapie
0.001887188089746278	9606	Homo sapie
7.688544069336689E-4	9606	Homo sapie
0.0010484378276368212	9606	Homo sapie
6.290626965820927E-4	9606	Homo sapie
0.001957083944922066	9606	Homo sapie
0.0011882295379883974	9606	Homo sapie
6.792094002580996E-5	9606	Homo sapie
1.3584188005161992E-4	9606	Homo sapie
1.3584188005161992E-4	9606	Homo sapie
3.396047001290498E-4	9606	Homo sapie
2.0376282007742986E-4	9606	Homo sapie
1.3584188005161992E-4	9606	Homo sapie

1.3584188005161992E-4	9606	Homo sapie
4.0752564015485973E-4	9606	Homo sapie
3.396047001290498E-4	9606	Homo sapie
3.396047001290498E-4	9606	Homo sapie
2.0376282007742986E-4	9606	Homo sapie
3.396047001290498E-4	9606	Homo sapie
0.001426339740542009	9606	Homo sapie
0.0012225769204645793	9606	Homo sapie
6.112884602322896E-4	9606	Homo sapie
4.754465801806697E-4	9606	Homo sapie
0.006724173062555186	9606	Homo sapie
2.0376282007742986E-4	9606	Homo sapie
2.0376282007742986E-4	9606	Homo sapie
4.0752564015485973E-4	9606	Homo sapie
0.005941147689941986	9606	Homo sapie
0.005941147689941986	9606	Homo sapie
4.892709862305165E-4	9606	Homo sapie
9.78541972461033E-4	9606	Homo sapie
2.0968756552736424E-4	9606	Homo sapie
6.290626965820927E-4	9606	Homo sapie
0.0015377088138673377	9606	Homo sapie
6.989585517578808E-4	9606	Homo sapie
6.989585517578807E-5	9606	Homo sapie
9.08646117285245E-4	9606	Homo sapie
0.0013280212483399733	9606	Homo sapie
0.0010484378276368212	9606	Homo sapie
3.494792758789404E-4	9606	Homo sapie
6.989585517578807E-5	9606	Homo sapie
2.0968756552736424E-4	9606	Homo sapie
0.0017473963793947019	9606	Homo sapie
2.795834207031523E-4	9606	Homo sapie

3.494792758789404E-4	9606	Homo sapie
7.688544069336689E-4	9606	Homo sapie
0.006360522820996715	9606	Homo sapie
4.892709862305165E-4	9606	Homo sapie
1.3979171035157615E-4	9606	Homo sapie
0.005941147689941986	9606	Homo sapie
0.005941147689941986	9606	Homo sapie
0.005381980848535682	9606	Homo sapie
0.009226252883204026	9606	Homo sapie
3.494792758789404E-4	9606	Homo sapie
1.3979171035157615E-4	9606	Homo sapie
0.0022366673656252184	9606	Homo sapie
0.0023065632208010066	9606	Homo sapie
2.795834207031523E-4	9606	Homo sapie
2.0968756552736424E-4	9606	Homo sapie
0.004123855455371497	9606	Homo sapie
0.006360522820996715	9606	Homo sapie
1.3979171035157615E-4	9606	Homo sapie
1.3979171035157615E-4	9606	Homo sapie
0.01418885860068498	9606	Homo sapie
9.78541972461033E-4	9606	Homo sapie
0.014817921297267072	9606	Homo sapie
0.0017473963793947019	9606	Homo sapie

Submitted entities found

RPL4;RPL5;RPL30;RPL3;RPL32;RPL31;RPLP1;RPL34;RPLP0;RPL10A;RPL8;RPL9;RPL6;RPL7;RPS15;RPS4X;RPL7A;RPS14;RPS17;RPS
RPL4;RPL5;RPL30;RPL3;RPL32;RPL31;RPLP1;RPL34;RPLP0;RPL10A;RPL8;RPL9;RPL6;RPL7;RPS15;RPS4X;EEF1B2;RPL7A;RPS14;RPS
RPL4;RPL5;RPL30;RPL3;RPL32;RPL31;RPLP1;RPL34;RPLP0;RPL10A;RPL8;RPL9;RPL6;RPL7;RPS15;RPS4X;RPL7A;RPS14;RPS17;RPS
RPL4;RPL5;RPL30;RPL3;RPL32;RPL31;RPLP1;RPL34;RPLP0;RPL10A;RPL8;RPL9;RPL6;RPL7;RPS15;RPS4X;RPL7A;RPS14;RPS17;RPS
RPL4;RPL5;RPL30;RPL3;RPL32;RPL31;RPLP1;RPL34;RPLP0;SRP14;RPL10A;RPL8;RPL9;RPL6;RPL7;RPS15;RPS4X;RPL7A;RPS14;RPS
RPL4;RPL5;RPL30;RPL3;RPL32;RPL31;RPLP1;RPL34;RPLP0;RPL10A;RPL8;RPL9;RPL6;RPL7;RPS15;RPS4X;RPL7A;RPS14;RPS17;RPS
RPL4;RPL5;RPL30;RPL3;RPL32;RPL31;RPLP1;RPL34;RPLP0;RPL10A;RPL8;RPL9;RPL6;RPL7;RPS15;RPS4X;RPL7A;RPS14;RPS17;RPS
RPL4;RPL5;RPL30;RPL3;RPL32;RPL31;RPLP1;RPL34;RPLP0;RPL10A;RPL8;RPL9;RPL6;RPL7;RPS15;RPS4X;RPL7A;RPS14;RPS17;RPS
RPS15;RPS4X;RPS14;RPS17;RPS16;RPS15A;UBB;RPS19;RPS18;RPS3;RPS2;RPS11;RPS27A;RPS10;RPS13;RPS12;RPS9;RPS7;RPS8;
RPS15;RPS4X;RPS14;RPS17;RPS16;RPS15A;UBB;RPS19;RPS18;RPS3;RPS2;RPS11;RPS27A;RPS10;RPS13;RPS12;RPS9;RPS7;RPS8;
RPL4;RPL5;RPL30;RPL3;RPL32;RPL31;RPLP1;RPL34;RPLP0;RPL10A;RPL8;RPL9;RPL6;RPL7;RPS15;RPS4X;RPL7A;RPS14;RPS17;RPS
RPL4;RPL5;RPL30;RPL3;RPL32;RPL31;RPLP1;RPL34;RPLP0;EIF4A3;RPL10A;RPL8;RPL9;RPL6;RPL7;RPS15;RPS4X;RPL7A;RPS14;RPS
RPL4;RPL5;RPL30;RPL3;RPL32;RPL31;RPLP1;RPL34;RPLP0;EIF4A3;RPL10A;RPL8;RPL9;RPL6;RPL7;RPS15;RPS4X;RPL7A;RPS14;RPS
RPS15;RPS4X;RPS14;RPS17;RPS16;RPS15A;UBB;RPS19;RPS18;RPS3;RPS2;RPS11;RPS27A;RPS10;RPS13;RPS12;RPS9;RPS7;RPS8;
RPL4;RPL5;RPL30;RPL3;RPL32;RPL31;RPLP1;RPL34;RPLP0;RPL10A;RPL8;RPL9;RPL6;RPL7;RPS15;RPS4X;RPL7A;RPS14;RPS17;RPS
RPL4;RPL5;RPL30;RPL3;RPL32;RPL31;RPLP1;RPL34;RPLP0;RPL10A;RPL8;RPL9;RPL6;RPL7;RPS15;RPS4X;RPL7A;RPS14;RPS17;RPS
RPL4;RPL5;RPL30;RPL3;RPL32;RPL31;RPLP1;RPL34;RPLP0;RPL10A;RPL8;RPL9;RPL6;RPL7;RPS15;RPS4X;RPL7A;RPS14;RPS17;RPS
RPL4;RPL5;RPL30;RPL3;RPL32;RPL31;RPLP1;RPL34;RPLP0;RPL10A;RPL8;RPL9;RPL6;RPL7;RPS15;RPS4X;RPL7A;RPS14;RPS17;RPS
RPL4;RPL5;RPL30;RPL3;RPL32;RPL31;RPLP1;RPL34;RPLP0;RPL10A;RPL8;RPL9;RPL6;RPL7;RPS15;RPS4X;RPL7A;RPS14;RPS17;RPS
RPL4;RPL5;RPL30;RPL3;RPL32;RPL31;RPLP1;RPL34;RPLP0;SRP14;RPL10A;RPL8;RPL9;RPL6;RPL7;RPS15;RPS4X;EEF1B2;RPL7A;RPS
RPL4;RPL5;RPL30;RPL3;RPL32;RPLP1;RPLP0;RPL10A;RPL8;RPL9;RPL6;RPL7;RPS15;RPS4X;RPS14;RPL7A;RPS16;RPS19;RPL18A;RP
RPS15;RPS4X;RPS14;RPS16;RPS15A;RPS19;RPS18;RPS3;RPS2;HNRNPA1;RPS27A;RPS10;RPS13;RPS12;RPS9;RPS7;RPS8;RPS5;RPS
RPL4;RPL5;RPL30;RPL3;RPL32;RPLP1;RPLP0;RPL10A;RPL8;RPL9;RPL6;RPL7;RPS15;RPS4X;RPS14;RPL7A;RPS16;RPS19;RPL18A;RP
RPL4;RPL5;RPL30;RPL3;RPL32;RPLP1;RPLP0;RPL10A;RPL8;RPL9;RPL6;RPL7;RPS15;RPS4X;RPS14;RPL7A;RPS16;RPS19;RPL18A;RP
RPL4;RPL5;RPL30;RPL3;RPL32;RPLP1;RPLP0;RPL10A;RPL8;RPL9;RPL6;RPL7;RPS15;RPS4X;RPS14;RPL7A;RPS16;RPS19;RPL18A;RP
RPL4;RPL5;RPL30;RPL3;RPL32;RPLP1;RPLP0;RPL10A;RPL8;RPL9;RPL6;RPL7;RPS15;RPS4X;RPS14;RPL7A;RPS16;RPS19;RPL18A;RP
RPL4;RPL5;RPL30;RPL3;RPL32;RPLP1;RPLP0;RPL10A;RPL8;RPL9;RPL6;RPL7;RPS15;RPS4X;RPS14;RPL7A;RPS16;RPS19;RPL18A;RP
RPS15;RPS4X;RPS14;RPS16;RPS15A;RPS19;RPS18;RPS3;RPS2;RPS27A;RPS10;RPS13;RPS12;RPS9;RPS7;RPS8;RPS5;RPS6;RPS3A;
RPS15;RPS4X;RPS14;RPS16;RPS15A;RPS19;RPS18;RPS3;RPS2;RPS27A;RPS10;RPS13;RPS12;RPS9;RPS7;RPS8;RPS5;RPS6;RPS3A;

RPL4;RPL5;RPL30;RPL3;RPL32;RPLP1;RPLP0;RPL10A;RPL8;RPL9;RPL6;RPL7;RPS15;RPS4X;EEF1B2;RPS14;RPL7A;RPS16;RPS19;RP
RPL4;RPL5;RPL30;RPL3;RPL32;RPLP1;RPLP0;RPL10A;RPL8;RPL9;RPL6;RPL7;RPS15;RPS4X;RPS14;RPL7A;RPS16;RPS19;RPL18A;RP
RPL4;RPL5;RPL30;RPL3;RPL32;RPLP1;RPLP0;RPL10A;RPL8;RPL9;RPL6;RPL7;RPS15;RPS4X;RPS14;RPL7A;RPS16;RPS19;RPL18A;RP
RPL4;RPL5;RPL30;RPL3;RPL32;RPLP1;RPLP0;RPL10A;RPL8;RPL9;RPL6;RPL7;RPS15;RPS4X;RPS14;RPL7A;RPS16;RPS19;RPL18A;RP
RPL4;RPL5;RPL30;RPL3;RPL32;RPLP1;RPLP0;RPL10A;RPL8;RPL9;RPL6;RPL7;RPS15;RPS4X;RPS14;RPL7A;RPS16;RPS19;RPL18A;RP
RPL4;RPL5;RPL30;RPL3;RPL32;RPLP1;RPLP0;RPL10A;RPL8;RPL9;RPL6;RPL7;RPS15;RPS4X;RPS14;RPL7A;RPS16;RPS19;RPL18A;RP
RPL4;RPL5;RPL30;RPL3;RPL32;RPLP1;RPLP0;RPL10A;RPL8;RPL9;RPL6;RPL7;RPS15;RPS4X;RPS14;RPL7A;RPS16;RPS19;RPL18A;RP
RPL4;RPL5;RPL30;RPL3;RPL32;RPLP1;RPLP0;RPL10A;RPL8;RPL9;RPL6;RPL7;RPS15;RPS4X;RPS14;RPL7A;RPS16;RPS19;RPL18A;RP
RPL4;RPL5;RPL30;RPL3;RPL32;RPLP1;RPLP0;RPL10A;RPL8;RPL9;RPL6;RPL7;RPS15;RPS4X;RPS14;RPL7A;RPS16;RPS19;RPL18A;RP
RPL4;RPL5;RPL30;RPL3;RPL32;RPLP1;RPLP0;RPL10A;RPL8;RPL9;RPL6;RPL7;RPS15;RPS4X;EEF1B2;RPS14;RPL7A;RPS16;RPS19;RP
RPS15;RPS4X;RPS14;RPS16;RPS15A;RPS19;RPS18;RPS3;RPS2;RPS27A;RPS10;RPS13;RPS12;RPS9;RPS7;RPS8;RPS5;RPS6;RPS3A;
TNFAIP3;TAB2;TRAF1;BIRC3
TNFAIP3;TAB2;TRAF1;SOS1;NFKB1;BIRC3
TNFAIP3;TAB2;TRAF1;BIRC3
PPP1R15A
TNFAIP3;TAB2;NFKB1;BIRC3
TNFAIP3;TAB2;BIRC3
EGR1;IL18RAP;IL2RA;HNRNPF;REL;STAT4;TAB2;SOS1;NFKB1;BIRC3;ICAM1
IL2RA;STAT4;SOS1
TNFAIP3;TRAF1;BIRC3
IL18RAP;IL2RA;HNRNPF;STAT4;TAB2;SOS1;NFKB1;ICAM1
MT1G;MT1H
EGR1;FOSB
TAB2;TANK;NFKB1;BIRC3
IL2RA
TAB2;TANK;NFKB1;BIRC3
TAB2;TANK;NFKB1;BIRC3
MT1G;MT1H
REG3A;EGR1;CD96;DDX3X;DEFA5;TNFAIP3;HBB;OLFM4;TANK;NFKB1;ICAM1;IL18RAP;IL2RA;HNRNPF;REL;STAT4;TAB2;ICOS;SOS1;BI
HNRNPF;STAT4
EGR1;FOSB;SOS1

RPL5;RPL30;RPL32;RPLP1;RPLP0;RPL10A;RPL8;RPL9;RPL6;RPL7;RPS4X;RPS15;RPL7A;RPS14;RPS16;RPS19;RPL18A;RPS18;RPL36A
RPL5;RPL30;RPL32;RPLP1;RPLP0;RPL10A;RPL8;RPL9;RPL6;RPL7;RPS4X;RPS15;RPL7A;RPS14;RPS16;RPS19;RPL18A;RPS18;RPL36A
RPL5;RPL30;RPL32;RPLP1;RPLP0;RPL10A;RPL8;RPL9;RPL6;RPL7;RPS4X;RPS15;RPL7A;RPS14;RPS16;RPS19;RPL18A;RPS18;RPL36A
RPL5;RPL30;RPL32;RPLP1;RPLP0;SRP14;RPL10A;RPL8;RPL9;RPL6;RPL7;RPS4X;RPS15;RPL7A;RPS14;RPS16;RPS19;RPL18A;RPS18;
RPL5;RPL30;RPL32;RPLP1;RPLP0;RPL10A;RPL8;RPL9;RPL6;RPL7;RPS4X;RPS15;RPL7A;RPS14;RPS16;RPS19;RPL18A;RPS18;RPL36A
RPL5;RPL30;RPL32;RPLP1;RPLP0;RPL10A;RPL8;RPL9;RPL6;RPL7;RPS4X;RPS15;RPL7A;RPS14;RPS16;RPS19;RPL18A;RPS18;RPL36A
RPS4X;RPS15;RPS14;RPS16;RPS15A;RPS19;RPS18;RPS3;RPS2;RPS11;RPS27A;RPS10;RPS13;RPS12;RPS9;RPS7;RPS8;RPS5;RPS6;
RPS4X;RPS15;RPS14;RPS16;RPS15A;RPS19;RPS18;RPS3;RPS2;RPS11;RPS27A;RPS10;RPS13;RPS12;RPS9;RPS7;RPS8;RPS5;RPS6;
RPL5;RPL30;RPL32;RPLP1;RPLP0;RPL10A;RPL8;RPL9;RPL6;RPL7;RPS4X;RPS15;RPL7A;RPS14;RPS16;RPS19;RPL18A;RPS18;RPL36A
RPL5;RPL30;RPL32;RPLP1;RPLP0;RPL10A;RPL8;RPL9;RPL6;RPL7;RPS4X;RPS15;RPL7A;RPS14;RPS16;RPS19;RPL18A;RPS18;RPL36A
RPL5;RPL30;RPL32;RPLP1;RPLP0;RPL10A;RPL8;RPL9;RPL6;RPL7;RPS4X;RPS15;RPL7A;RPS14;RPS16;RPS19;RPL18A;RPS18;RPL36A
RPL5;RPL30;RPL32;RPLP1;RPLP0;RPL10A;RPL8;RPL9;RPL6;RPL7;RPS4X;RPS15;RPL7A;RPS14;TRMT112;RPS16;RPS19;RPL18A;RPS1
RPL5;RPL30;RPL32;RPLP1;RPLP0;RPL10A;RPL8;RPL9;RPL6;RPL7;RPS4X;RPS15;RPL7A;RPS14;RPS16;RPS19;RPL18A;RPS18;RPL36A
RPL5;RPL30;RPL32;RPLP1;RPLP0;RPL10A;RPL8;RPL9;RPL6;RPL7;RPS4X;RPS15;RPL7A;RPS14;RPS16;RPS19;RPL18A;RPS18;RPL36A
RPS4X;RPS15;RPS14;RPS16;RPS15A;RPS19;RPS18;RPS3;RPS2;RPS11;RPS27A;RPS10;RPS13;RPS12;RPS9;RPS7;RPS8;RPS5;RPS6;
RPL5;RPL30;RPL32;RPLP1;RPLP0;RPL10A;RPL8;RPL9;RPL6;RPL7;RPS4X;RPS15;RPL7A;RPS14;RPS16;RPS19;RPL18A;RPS18;RPL36A
RPL5;RPL30;RPL32;RPLP1;RPLP0;RPL10A;RPL8;RPL9;RPL6;RPL7;RPS4X;RPS15;RPL7A;RPS14;RPS16;RPS19;RPL18A;RPS18;RPL36A
RPL5;RPL30;RPL32;RPLP1;RPLP0;RPL10A;RPL8;RPL9;RPL6;RPL7;RPS4X;RPS15;RPL7A;RPS14;TRMT112;RPS16;RPS19;RPL18A;RPS1
RPL5;RPL30;RPL32;RPLP1;RPLP0;SRP14;RPL10A;RPL8;RPL9;RPL6;RPL7;RPS4X;RPS15;RPL7A;RPS14;TRMT112;RPS16;RPS19;RPL18
RPL5;RPL30;RPL32;RPLP1;RPLP0;RPL10A;RPL8;RPL9;RPL6;RPL7;RPS4X;RPS15;RPL7A;RPS14;TRMT112;RPS16;RPS19;RPL18A;RPS1
ZEB1;CISH;TIMP1;LCP1;CBL
CYFIP2;ZEB1;PTPRC;CISH;ROCK1;GRAP2;PTPN22;LNPEP;TIMP1;LCP1;CBL
ZEB1;CISH;GRAP2;TIMP1;LCP1;CBL
GSK3B;PTPRC;ROCK1;ITGA1
ZEB1;TIMP1
PTPRC;ITGA1
CISH
PTPRC;PTPN22
PTPRC;GRAP2;PTPN22
GRAP2;CBL
GRAP2;CBL

MSH3
GRAP2;CBL
MSH3
LCP1
MSH3
LCP1
TIMP1
LCP1
CBL
MT2A;MT1M;MT1F;MT1G;MT1X;MT1H;MT1B;MT1E
MT2A;MT1M;MT1F;MT1G;MT1X;MT1H;MT1B;MT1E
PLEKHG2;DOCK9;RASGRF2;DOCK8;FAM13A;ARAP2;ERBIN;MYO9B;ARHGAP15;ARHGAP25;MPP7;DOCK10;DOCK11;ABR;PREX1;ARHG
CD44
NCOA1;NCOA2;CHD9;NCOA6;RORA;TBL1X
NCOA1;NCOA2;PBRM1;KDM4C;KMT2C;EHMT1;ARID5B;ARID4B;ASH1L;ELP4;SMARCA2;ARID1B;PHF21A;GATAD2A;KAT2B;KANSL1;KAT
NCOA1;NCOA2;PBRM1;KDM4C;KMT2C;EHMT1;ARID5B;ARID4B;ASH1L;ELP4;SMARCA2;ARID1B;PHF21A;GATAD2A;KAT2B;KANSL1;KAT
PPP3CA;PPP3CC;NFATC3;ITPR2;NFATC1
DOCK9;RASGRF2;DOCK8;FAM13A;MSI2;ARHGAP15;DOCK10;PREX1;STK10;DOCK11;ABR;EPSTI1;PTK2B;PLEKHG2;USP9X;ARAP2;ERB
CDK6;AGO3;RUNX1;TNRC6B
PPP3CA;PPP3CC;NFATC3;NFATC1
MT-ND6;MT-ND4L;MT-ND4;MT-ND2;MT-ND3;MT-ATP8;MT-ND1
PPP3CA;GNG2;PPP3CC;AGO3;ITPR2;NFATC1;PLCB1;TNRC6B
AGO3;TNRC6B
DLG1;CAMK2D;RASGRF2
DOCK10;DOCK11;ABR;PREX1;ARHGAP10;PLEKHG2;DOCK9;RASGRF2;DOCK8;ARAP2;MYO9B;CDC42SE2
MT-ND4L;MT-ND4;MT-ND2;MT-ND3;MT-ATP8;MT-ND1
DOCK9;RASGRF2;DOCK8;FAM13A;MSI2;ARHGAP15;DOCK10;PREX1;STK10;DOCK11;ABR;PPP2R5E;EPSTI1;PTK2B;CLASP1;NCOA2;PL
DOCK9;RASGRF2;DOCK8;FAM13A;MSI2;ARHGAP15;DOCK10;PREX1;STK10;DOCK11;ABR;PPP2R5E;EPSTI1;PTK2B;CLASP1;NCOA2;PL
KAT2B;NR4A3;AGO3;KMT2C;RUNX1;TNRC6B
CD44
NCOA2;CREBBP;TBL1XR1;KMT2C;KAT6A;MSL2;ARID4B;TBL1X;ARID2;OGT;RCOR1;KDM6A

NCOA2;CREBBP;TBL1XR1;KMT2C;KAT6A;MSL2;ARID4B;TBL1X;ARID2;OGT;RCOR1;KDM6A
CDK8;CREBBP;TBL1XR1;FBXW7;TBL1X
CDK8;CREBBP;TBL1XR1;FBXW7;TBL1X
CDK8;CREBBP;TBL1XR1;FBXW7;TBL1X
CDK8;CREBBP;TBL1XR1;FBXW7;TBL1X
CDK8;CREBBP;TBL1XR1;FBXW7;TBL1X
CDK8;CREBBP;TBL1XR1;FBXW7;TBL1X
NCOA2;CREBBP;TBL1XR1;TBL1X
RANBP2;CREBBP;CPSF6;FBXW7;BRAF;PPP2R5C;DUSP16;PPP1CB;EML4;CDK8;POLR2A;TBL1XR1;AKT3;PSME4;FYN;BIRC6;TBL1X
CREBBP;RPS6KA5;REL;FYN
AKT3;YWHAZ
RANBP2;SEC23A;CREBBP;TRAF3;AKT3;G3BP2;NUP98;YWHAZ
RANBP2;SEC23A;CREBBP;MAN2A1;TRAF3;MGAT5;AKT3;G3BP2;NUP98;YWHAZ;PARP8
TBL1XR1;CAMK4;TBL1X
TBL1XR1;CAMK4;TBL1X
TBL1XR1;CAMK4;TBL1X
TBL1XR1;CAMK4;TBL1X
RANBP2;ARIH1;NUP98;EIF4G1
PCLAF
PCLAF
PCLAF
TMSB4X
TMSB4X
TMSB4X
PCLAF
TMSB4X
COL1A1;COL3A1;COL4A2;ITGA4;COL4A1;ITGB2;COL6A3;CD44;FBN1
COL1A1;COL3A1;COL4A2;MMP1;COL4A1;COL6A3;TIMP1;A2M;CD44;GZMH;FBN1
PRKCB;RUNX2;RUNX1
CD44
IGFBP5;IGFBP4;MMP1;COL4A1;IGFBP7;TIMP1;PRSS23;GZMH;FBN1

ITGA4;MMP1;ITGB2;GZMH;COL1A1;COL3A1;COL4A2;COL4A1;COL6A3;TIMP1;A2M;CD44;FBN1
COL1A1;COL3A1;COL4A2;COL4A1;COL6A3
COL1A1;COL3A1;COL4A2;MMP1;COL4A1;COL6A3
RUNX2;RUNX1
COL1A1;COL3A1;COL4A2;COL4A1;COL6A3
PRKCB;LMNA;LMNB1
COL1A1;COL3A1;COL4A2;COL4A1;COL6A3
IGFBP5;IGFBP4;COL4A1;IGFBP7;TIMP1;PRSS23;FBN1
LMNA;LMNB1
COL1A1;RUNX2
COL1A1;COL3A1;COL4A2;COL4A1
COL1A1;COL4A2;COL4A1
RUNX1
COL1A1;COL3A1;COL4A2;COL4A1;COL6A3
COL3A1;COL4A2;COL4A1;COL6A3;SPTBN1
NCOA1;ATF2;NCOA2;CPT1A;CREBBP;CRTC3;CUL1;CREM;RORA;NR3C1;ARNTL;PPP1CB;PER1;TBL1XR1;NAMPT;CRY1;BTRC
GSK3B;WDR48;PTEN;PIK3CB;RBPJ;DUSP16;PPP1CB;MECP2;MPRIP;CNTRL;LRRFIP1;JAK2;KSR1;FBXW7;AP3B1;NRG1;PPP2R5C;DUS
ATF2;SETD2;KDM3A;KMT2A;TRRAP;KMT2C;ARID4B;PHF21A;TAF5L;NSD3;EP400;KAT7;SMYD3;JAK2;ARID2;KDM6A;NCOA1;NCOA2;BRI
ATF2;SETD2;KDM3A;KMT2A;TRRAP;KMT2C;ARID4B;PHF21A;TAF5L;NSD3;EP400;KAT7;SMYD3;JAK2;ARID2;KDM6A;NCOA1;NCOA2;BRI
NCOR2;NR4A3;NR1D2;RORA;NR3C1;ESR2;NR3C2
MTMR1;MTMR3;PLEKHA2;PTEN;SYNJ2;PIK3CB;PTPN13;PIK3CG;PIK3R5;INPP4A;INPP4B;PIK3CA;INPP5D;PIP4K2A;SBF2
MTMR1;TNFAIP8;MTMR3;MTMR12;PLEKHA2;PITPNB;PTEN;SYNJ2;PIK3CB;PTPN13;PIK3CG;PIK3R5;INPP4A;INPP4B;PIK3CA;INPP5D;PI
MECP2;TNRC6C;NCOR2;CAMK2D;TBL1XR1;CAMK4;AGO2;TNRC6B
CREBBP;PRKCB;RUNX2;RUNX1
TNRC6C;NCOR2;MECP2;CAMK2D;TBL1XR1;CAMK4;AGO2;PTEN;SLC2A3;PTPN4;TNRC6B
PLEKHG1;DOCK3;FAM13B;WIPF1;DOCK9;ARAP2;ERBIN;ARHGAP26;ARHGAP15;IQGAP2;VAV1;ARHGAP35;GNA13;DOCK10;PREX1;DOC
CAMK2D;KSR1;AP3B1;BRAF;DUSP16;DUSP6;QKI;SND1;RAP1B;PPP1CB;MPRIP;LMNA;JAK2;MARK3;VCL
NSD3;SETD2;KMT2A;EED;KMT2C;SMYD3;ASH1L;NFKB1;EZH2
PPP3CA;ITPR1;NFATC3;NFATC2;ITPR2;NFATC1
NCOA1;NCOA2;CPT1A;CREBBP;TBL1XR1;RORA
CD44

SMARCC1;CREBBP;AUTS2;KMT2A;PRKCB;CCNH;KMT2C;TCF12;NFATC2;GATA3;ARID1B;RUNX2;RUNX1;TNRC6C;KAT2B;ITCH;NR4A3;
STAT5B;PIK3CA;RAPGEF1;PIK3CB;JAK2;PIK3CG;VAV1;PIK3R5
NCOR2;MECP2;TBL1XR1
HDAC4;NOTCH2;NCOR2;KAT2B;CREBBP;TBL1XR1;RBPJ
RPL30;RPL3;RPL10;RPLP1;RPLP0;RPL11;RPL10A;RPL8;RPS4X;RPS15;UBB;RPS19;RPL18A;RPS18;UBC;RPL13;RPS2;RPL15;RPL37;RP
RPL30;RPL3;RPL10;RPLP1;RPLP0;RPL11;RPL10A;RPL8;RPS4X;RPS15;UBB;RPS19;RPL18A;RPS18;UBC;RPL13;RPS2;RPL15;RPL37;RP
RPL30;RPL3;RPL10;RPLP1;RPLP0;RPL11;RPL10A;RPL8;RPS4X;RPS15;UBB;RPS19;RPL18A;RPS18;UBC;RPL13;RPS2;RPL15;RPL37;RP
RPL30;RPL3;RPL10;RPLP1;RPLP0;RPL11;RPL10A;RPL8;RPS4X;RPS15;UBB;RPS19;RPL18A;RPS18;UBC;RPL13;RPS2;RPL15;RPL37;RP
RPL30;RPL3;RPL10;RPLP1;RPLP0;RPL11;RPL10A;RPL8;RPS4X;RPS15;UBB;RPS19;RPL18A;RPS18;UBC;RPL13;RPS2;RPL15;RPL37;RP
RPL30;RPL3;RPL10;RPLP1;RPLP0;RPL11;RPL10A;RPL8;RPS4X;RPS15;UBB;RPS19;RPL18A;RPS18;UBC;RPL13;RPS2;RPL15;RPL37;RP
RPL30;RPL3;RPL10;RPLP1;RPLP0;RPL11;RPL10A;RPL8;RPS4X;RPS15;UBB;RPS19;RPL18A;RPS18;UBC;RPL13;RPS2;RPL15;RPL37;RP
RPL30;RPL3;RPL10;RPLP1;RPLP0;RPL11;RPL10A;RPL8;RPS4X;RPS15;UBB;RPS19;RPL18A;RPS18;UBC;RPL13;RPS2;RPL15;RPL37;RP
RPL30;RPL3;RPL10;RPLP1;RPLP0;RPL11;RPL10A;RPL8;RPS4X;RPS15;UBB;RPS19;RPL18A;RPS18;UBC;RPL13;RPS2;RPL15;RPL37;RP
DNAJB1;HSP90AA1;UBB;HSPA1B;HSPA1A
RPL30;RPL3;RPL10;RPLP1;RPLP0;RPL11;RPL10A;RPL8;RPS4X;RPS15;UBB;RPS19;RPL18A;RPS18;UBC;RPL13;RPS2;RPL15;RPL37;RP
RPL30;RPL3;RPL10;RPLP1;RPLP0;RPL11;RPL10A;RPL8;RPS4X;RPS15;UBB;RPS19;RPL18A;RPS18;UBC;RPL13;RPS2;RPL15;RPL37;RP
RPL30;RPL3;RPL10;RPLP1;RPLP0;RPL11;RPL10A;RPL8;RPS4X;RPS15;UBB;RPS19;RPL18A;RPS18;UBC;RPL13;RPS2;RPL15;RPL37;RP
RPL30;RPL3;RPL10;RPLP1;RPLP0;RPL11;RPL10A;RPL8;RPS4X;RPS15;UBB;RPS19;RPL18A;RPS18;UBC;RPL13;RPS2;RPL15;RPL37;RP
RPL30;RPL3;RPL10;RPLP1;RPLP0;RPL11;RPL10A;RPL8;RPS4X;RPS15;UBB;RPS19;RPL18A;RPS18;UBC;RPL13;RPS2;RPL15;RPL37;RP
RPL30;RPL3;RPL10;RPLP1;RPLP0;RPL11;RPL10A;RPL8;RPS4X;RPS15;UBB;RPS19;RPL18A;RPS18;UBC;RPL13;RPS2;RPL15;RPL37;RP
MT-ND4;RPL30;RPL3;RPL10;MT-CO1;RPLP1;RPLP0;RPL11;RPL10A;RPL8;MT-ND3;RPS4X;RPS15;MT-ATP6;UBB;RPS19;RPL18A;RPS18;
RPS7;RPS8;RPS5;RPS4X;RPS15;RPS28;RPS27;UBB;RPS19;RPS18;RPS29;RPS2;RPS27A;RPS21;RPS12
RPL30;RPL3;RPL10;RPLP1;RPLP0;RPL11;RPL10A;RPL8;RPS4X;RPS15;UBB;RPS19;RPL18A;RPS18;UBC;RPL13;RPS2;RPL15;RPL37;RP
RPL30;RPL3;RPL10;RPLP1;RPLP0;RPL11;RPL10A;RPL8;RPS4X;RPS15;UBB;RPS19;RPL18A;RPS18;UBC;RPL13;RPS2;RPL15;RPL37;RP
RPS4Y1;RPS15;RPS4X;RPS14;RPS16;RPS15A;RPS19;RPS18;RPS3;RPS2;HNRNPA1;RPS27A;RPS10;RPS13;RPS12;RPS9;RPS7;RPS8;
RPL4;RPL5;RPL30;RPL3;RPL32;RPL31;RPL34;RPLP1;RPLP0;RPL10A;RPL8;RPL9;RPL6;RPL7;RPS15;RPS4X;RPS14;RPL7A;RPS16;RPS
RPL4;RPL5;RPL30;RPL3;RPL32;RPL31;RPL34;RPLP1;RPLP0;RPL10A;RPL8;RPL9;RPL6;RPL7;RPS15;RPS4X;RPS14;RPL7A;RPS16;RPS
RPL4;RPL5;RPL30;RPL3;RPL32;RPL31;RPL34;RPLP1;RPLP0;RPL10A;RPL8;RPL9;RPL6;RPL7;RPS15;RPS4X;RPS14;RPL7A;RPS16;RPS
RPL4;RPL5;RPL30;RPL3;RPL32;RPL31;RPL34;RPLP1;RPLP0;RPL10A;RPL8;RPL9;RPL6;RPL7;RPS15;RPS4X;RPS14;RPL7A;RPS16;RPS
RPL4;RPL5;RPL30;RPL3;RPL32;RPL31;RPL34;RPLP1;RPLP0;RPL10A;RPL8;RPL9;RPL6;RPL7;RPS15;RPS4X;RPS14;RPL7A;RPS16;RPS
RPL4;RPL5;RPL30;RPL3;RPL32;RPL31;RPL34;RPLP1;RPLP0;RPL10A;RPL8;RPL9;RPL6;RPL7;RPS15;RPS4X;RPS14;RPL7A;RPS16;RPS

RPS4Y1;RPS15;RPS4X;RPS14;RPS16;RPS15A;RPS19;RPS18;RPS3;RPS2;RPS27A;RPS10;RPS13;RPS12;RPS9;RPS7;RPS8;RPS5;RPS6
RPS4Y1;RPS15;RPS4X;RPS14;RPS16;RPS15A;RPS19;RPS18;RPS3;RPS2;RPS27A;RPS10;RPS13;RPS12;RPS9;RPS7;RPS8;RPS5;RPS6
RPL4;RPL5;RPL30;RPL3;RPL32;RPL31;RPL34;RPLP1;RPLP0;RPL10A;RPL8;RPL9;RPL6;RPL7;RPS15;RPS4X;EEF1B2;RPS14;RPL7A;RPS
RPL4;RPL5;RPL30;RPL3;RPL32;RPL31;RPL34;RPLP1;RPLP0;RPL10A;RPL8;RPL9;RPL6;RPL7;RPS15;RPS4X;RPS14;RPL7A;RPS16;RPS
RPL4;RPL5;RPL30;RPL3;RPL32;RPL31;RPL34;RPLP1;RPLP0;RPL10A;RPL8;RPL9;RPL6;RPL7;RPS15;RPS4X;RPS14;RPL7A;RPS16;RPS
RPL4;RPL5;RPL30;RPL3;RPL32;RPL31;RPL34;RPLP1;RPLP0;RPL10A;RPL8;RPL9;RPL6;RPL7;RPS15;RPS4X;RPS14;RPL7A;RPS16;RPS
RPL4;RPL5;RPL30;RPL3;RPL32;RPL31;RPL34;RPLP1;RPLP0;RPL10A;RPL8;RPL9;RPL6;RPL7;RPS15;RPS4X;RPS14;RPL7A;RPS16;RPS
RPL4;RPL5;RPL30;RPL3;RPL32;RPL31;RPL34;RPLP1;RPLP0;RPL10A;RPL8;RPL9;RPL6;RPL7;RPS15;RPS4X;RPS14;RPL7A;RPS16;RPS
RPL4;RPL5;RPL30;RPL3;RPL32;RPL31;RPL34;RPLP1;RPLP0;RPL10A;RPL8;RPL9;RPL6;RPL7;RPS15;RPS4X;RPS14;RPL7A;RPS16;RPS
RPL4;RPL5;RPL30;RPL3;RPL32;RPL31;RPL34;RPLP1;RPLP0;RPL10A;RPL8;RPL9;RPL6;RPL7;RPS15;RPS4X;RPS14;RPL7A;RPS16;RPS
RPL4;RPL5;RPL30;RPL3;RPL32;RPL31;RPL34;RPLP1;RPLP0;RPL10A;RPL8;RPL9;RPL6;RPL7;RPS15;RPS4X;RPS14;RPL7A;RPS16;RPS
RPL4;RPL5;RPL30;RPL3;RPL32;RPL31;RPL34;RPLP1;RPLP0;RPL10A;RPL8;RPL9;RPL6;RPL7;RPS15;RPS4X;RPS14;RPL7A;RPS16;RPS
RPS4Y1;RPS15;RPS4X;RPS14;RPS16;RPS15A;RPS19;RPS18;RPS3;RPS2;RPS27A;RPS10;RPS13;RPS12;RPS9;RPS7;RPS8;RPS5;RPS6
MT-ATP6;MT-ND4L;MT-ND4;MT-ND5;MT-CO3;MT-ND3;MT-ND1
MT-ATP6;MT-ND4L;MT-ND4;MT-ND5;MT-CO3;MT-ND3;MT-ND1
MT-ATP6;MT-ND4L;MT-ND4;MT-ND5;MT-CO3;MT-ND3;MT-ND1
MT-ATP6;MT-ND4;MT-ND5;MT-CO3;MT-ND3;MT-ND1
MT-ATP6;MT-ND4L;MT-ND4;MT-ND5;MT-CO3;MT-ND3;MT-ND1
MT-ND4;MT-ND5;MT-ND3;MT-ND1
MT-ND4;MT-ND5;MT-CO3;MT-ND3;MT-ND1
MT-ATP6;MT-ND4;MT-ND5;MT-CO3;MT-ND3;MT-ND1
CD28;FYN
VPS37B
CA5B
VPS37B
STAT3
FYN;PPP2R5C
PRKAR2A;PRKCE;CD28;FYN;PPP2R5C;RCOR1
VPS37B
ATM
CD28;FYN;PPP2R5C

STAT3;FYN
STAT3;FYN
RPL30;RPL12;RPLP1;RPL11;RPL8;RPS4X;RPS15;RPS14;RPS15A;UBB;RPS19;RPL35;RPL37;RPL18;RPL39;RPS12;RPL41;RPS9;RPL21;R
RPL30;RPL12;RPLP1;RPL11;RPL8;RPS4X;RPS15;RPS14;RPS15A;UBB;RPS19;RPL35;RPL37;RPL18;RPL39;RPS12;RPL41;RPS9;RPL21;R
RPL30;RPL12;RPLP1;RPL11;RPL8;RPS4X;RPS15;RPS14;RPS15A;UBB;RPS19;RPL35;RPL37;RPL18;RPL39;RPS12;RPL41;RPS9;RPL21;R
RPL30;RPL12;RPLP1;RPL11;RPL8;RPS4X;RPS15;RPS14;RPS15A;UBB;RPS19;RPL35;RPL37;RPL18;RPL39;RPS12;RPL41;RPS9;RPL21;R
RPL30;RPL12;RPLP1;RPL11;RPL8;RPS4X;RPS15;RPS14;RPS15A;UBB;RPS19;RPL35;RPL37;RPL18;RPL39;RPS12;RPL41;RPS9;RPL21;R
RPS9;RPS8;RPS5;RPS4X;RPS15;RPS14;RPS28;RPS15A;UBB;RPS19;FAU;UBA52;RPS12;RPS23
RPS9;RPS8;RPS5;RPS4X;RPS15;RPS14;RPS28;RPS15A;UBB;RPS19;FAU;UBA52;RPS12;RPS23
RPL30;RPL12;RPLP1;RPL11;RPL8;RPS4X;RPS15;RPS14;RPS15A;UBB;RPS19;RPL35;RPL37;RPL18;RPL39;RPS12;RPL41;RPS9;RPL21;R
RPL30;RPL12;RPLP1;RPL11;RPL8;RPS4X;RPS15;RPS14;RPS15A;UBB;RPS19;RPL35;RPL37;RPL18;RPL39;RPS12;RPL41;RPS9;RPL21;R
DNAJB1;HSP90AA1;HSPH1;HSP90AB1;UBB;PTGES3;HSPA1B;HSPA1A
RPL30;RPL12;RPLP1;RPL11;RPL8;RPS4X;RPS15;RPS14;RPS15A;UBB;RPS19;RPL35;RPL37;RPL18;RPL39;RPS12;RPL41;RPS9;RPL21;R
RPL30;RPL12;RPLP1;RPL11;RPL8;RPS4X;RPS15;RPS14;RPS15A;UBB;RPS19;RPL35;RPL37;RPL18;RPL39;RPS12;RPL41;RPS9;RPL21;R
RPL30;RPL12;RPLP1;RPL11;RPL8;RPS4X;RPS15;RPS14;RPS15A;UBB;RPS19;RPL35;RPL37;RPL18;RPL39;RPS12;RPL41;RPS9;RPL21;R
DNAJB1;HSP90AA1;HSPH1;HSP90AB1;UBB;PTGES3;HSPA1B;HSPA1A
RPL30;RPL12;RPLP1;RPL11;RPL8;RPS4X;RPS15;RPS14;RPS15A;UBB;RPS19;RPL35;RPL37;RPL18;RPL39;RPS12;RPL41;RPS9;RPL21;R
DNAJB1;HSP90AA1;HSPH1;HSP90AB1;UBB;PTGES3;HSPA1B;HSPA1A
RPL30;RPL12;RPLP1;RPL11;RPL8;RPS4X;RPS15;RPS14;RPS15A;UBB;RPS19;RPL35;RPL37;RPL18;RPL39;RPS12;RPL41;RPS9;RPL21;R
RPL30;RPL12;RPLP1;RPL11;RPL8;RPS4X;RPS15;RPS14;RPS15A;UBB;RPS19;RPL35;RPL37;RPL18;RPL39;RPS12;RPL41;RPS9;RPL21;R
DNAJB1;HSP90AA1;HSPH1;HSP90AB1;UBB;PTGES3;HSPA1B;HSPA1A
DNAJB1;HSPH1;UBB;HSPA1B;HSPA1A
ZEB1;ANXA1;VIM;TIMP1;FOS;MCL1
CD44
NFKBIA;ZEB1;ANXA1;IL2RA;STAT4;VIM;TIMP1;LCP1;FOS;IL7R;NFKB1;MCL1
ANXA1;FOS;NFKB1;NFKBIA;ZEB1;IL2RA;STAT4;VIM;TIMP1;LCP1;IL7R;CD44;MCL1
BTG2;RGCC
ARL4C;FAS
BTG2;RGCC
PPP1R15A
ARL4C;GNG2;FAS;FOSB;FOS

NFKBIA;NFKB1
BTG2;RGCC;TAF7;FAS;FOS
NFKBIA;NFKB1
HSPA5
NFKBIA;TNFAIP3;TANK;NFKB1
NFKBIA;TNFAIP3;FAS;TRAF1;NFKB1
FAS
HSPA5
IL2RA
FAS
NFKBIA;NFKB1
MT-ND6;MT-ND4L;MT-ND4;MT-ND5;MT-CO1;MT-CO2;MT-CO3;MT-ND2;MT-CYB;MT-ND3;MT-ATP8;MT-ND1
MT-ND4L;MT-ND4;MT-ND5;MT-CO1;MT-CO2;MT-CO3;MT-CYB;MT-ND2;MT-ND3;MT-ATP8;MT-ND1
MT2A;MT1M;MT1G;MT1X;MT1H;MT1B;MT1E
MT2A;MT1M;MT1G;MT1X;MT1H;MT1B;MT1E
MT-ND6;MT-ND4;MT-ND5;MT-CO1;MT-CO2;MT-CO3;MT-CYB;MT-ND2;MT-ND3;MT-ATP8;ATP5ME;MT-ND1
MT-ND6;MT-ND4;MT-ND5;MT-CO1;MT-CO2;MT-CO3;MT-CYB;MT-ND2;MT-ND3;MT-ND1
FYB1;GRAP2;PLCG2;LCP2;PLCG1;CD247
TIAM2;ABR;PREX1;ARHGEF12;MAGED1;ARHGEF38;ARHGEF3
ITPKB;INPP5B;PLCG2;PTEN;PLCG1
MT-ND6;MT-ND4L;MT-ND4;MT-ND5;MT-CO1;MT-CO2;MT-CO3;MT-ND2;MT-CYB;MT-ND3;MT-ATP8;MT-ND1
ACE2;ENPEP;MME;ANPEP
MT-ND6;MT-ND4;MT-ND5;MT-ND2;MT-ND3;MT-ND1
KAT2B;GSK3B;HIST1H4H;GATAD2B;HIST1H2BD;HIST1H2AC;GATAD2A
FMNL3;ABR;PREX1;ARHGEF12;ERBIN;ARHGAP18;MYO9B
MT-ND6;MT-ND4;MT-ND5;MT-CO1;MT-ND2;MT-ND3;MT-ATP8;MT-ND1;SUCLG2;MT-CO2;ME2;MT-CO3;MT-CYB;ATP5ME
TIAM2;ABR;PREX1;ARHGEF12;MAGED1;ARHGEF38;ARHGEF3
HIST1H4H;HIST2H2BF;GATAD2B;HIST1H2BD;HIST1H2AC;GATAD2A
KAT2B;HIST1H4H;MNAT1;GATAD2B;HIST1H2BD;HIST1H2AC;GATAD2A
CUBN;NCEH1;APOA1;APOB;AP2A2
KAT2B;FOXO3;FOXO1

PRKCB;RUNX2;RUNX1
CD44
MECP2;TBL1XR1;CAMK4;TNRC6B
MECP2;TBL1XR1;CAMK4;FKBP5;TNRC6B
RUNX2;RUNX1
MECP2;TBL1XR1;CAMK4
MECP2;TBL1XR1;CAMK4
MECP2;TBL1XR1;CAMK4
MECP2;TBL1XR1;CAMK4
CCND3;CCNH;PCGF5;PRKCB;IL2RA;TCF12;ARID1B;RUNX2;RUNX1;TNRC6B
MECP2;CAMK4
ST6GAL1;MGAT4A;MGAT5
MECP2;TBL1XR1
ELMO1;FYN;DOCK2
MECP2
PPP1CB;TBL1XR1;CRY1;RORA;ARNTL
TBL1XR1;HBB;RORA;APOB;ARNTL
PACS1;ELMO1;FYN;DOCK2
CYTH3;COG5;VTI1A;CYTH1
AKT3;RUNX2
RPL30;RPL12;RPL34;RPLP1;RPL9;RPS15;RPS16;RPS15A;RPS19;RPS18;RPL35;RPLP2;RPL37;RPS11;RPS27A;RPL39;RPS13;RPS12;RP
RPL30;RPL12;RPL34;RPLP1;RPL9;RPS15;RPS16;RPS15A;RPS19;RPS18;RPL35;RPLP2;RPL37;RPS11;RPS27A;RPL39;RPS13;RPS12;RP
RPL30;RPL12;RPL34;RPLP1;RPL9;RPS15;RPS16;RPS15A;RPS19;RPS18;RPL35;RPLP2;RPL37;RPS11;RPS27A;RPL39;RPS13;RPS12;RP
RPL30;RPL12;RPL34;RPLP1;RPL9;RPS15;RPS16;RPS15A;RPS19;RPS18;RPL35;RPLP2;RPL37;RPS11;RPS27A;RPL39;RPS13;RPS12;RP
RPL30;RPL12;RPL34;RPLP1;RPL9;RPS15;RPS16;RPS15A;RPS19;RPS18;RPL35;RPLP2;RPL37;RPS11;RPS27A;RPL39;RPS13;RPS12;RP
RPS15;RPS25;RPS28;RPS16;RPS15A;RPS27;RPS19;RPS29;RPS18;RPS11;RPS27A;UBA52;RPS13;RPS23;RPS12
RPS15;RPS25;RPS28;RPS16;RPS15A;RPS27;RPS19;RPS29;RPS18;RPS11;RPS27A;UBA52;RPS13;RPS23;RPS12
RPL30;RPL12;RPL34;RPLP1;RPL9;RPS15;RPS16;RPS15A;RPS19;RPS18;RPL35;RPLP2;RPL37;RPS11;RPS27A;RPL39;RPS13;RPS12;RP
RPL30;RPL12;RPL34;RPLP1;RPL9;RPS15;RPS16;RPS15A;RPS19;RPS18;RPL35;RPLP2;RPL37;RPS11;RPS27A;RPL39;RPS13;RPS12;RP
RPL30;RPL12;RPL34;RPLP1;RPL9;RPS15;RPS16;RPS15A;RPS19;RPS18;RPL35;RPLP2;RPL37;RPS11;RPS27A;RPL39;RPS13;RPS12;RP
RPL30;RPL12;RPL34;RPLP1;RPL9;RPS15;RPS16;RPS15A;RPS19;RPS18;RPL35;RPLP2;RPL37;RPS11;RPS27A;RPL39;RPS13;RPS12;RP

RPL30;RPL12;RPL34;RPLP1;RPL9;RPS15;RPS16;RPS15A;RPS19;RPS18;RPL35;RPLP2;RPL37;RPS11;RPS27A;RPL39;RPS13;RPS12;RP
RPL30;RPL12;RPL34;RPLP1;RPL9;RPS15;RPS16;RPS15A;RPS19;RPS18;RPL35;RPLP2;RPL37;RPS11;RPS27A;RPL39;RPS13;RPS12;RP
RPL30;RPL12;RPL34;RPLP1;RPL9;RPS15;RPS16;RPS15A;RPS19;RPS18;RPL35;RPLP2;RPL37;RPS11;RPS27A;RPL39;RPS13;RPS12;RP
RPL30;RPL12;RPL34;RPLP1;RPL9;RPS15;RPS16;RPS15A;RPS19;RPS18;RPL35;RPLP2;RPL37;RPS11;RPS27A;RPL39;RPS13;RPS12;RP
RPL30;RPL12;RPL34;RPLP1;RPL9;RPS15;RPS16;RPS15A;RPS19;RPS18;RPL35;RPLP2;RPL37;RPS11;RPS27A;RPL39;RPS13;RPS12;RP
RPS15;RPS25;RPS28;RPS16;RPS15A;RPS27;RPS19;RPS29;RPS18;RPS11;RPS27A;UBA52;RPS13;RPS23;RPS12
RPS15;RPS25;RPS28;RPS16;RPS15A;RPS27;RPS19;RPS29;RPS18;RPS11;RPS27A;UBA52;RPS13;RPS23;RPS12
RPS15;RPS25;RPS28;RPS16;RPS15A;RPS27;RPS19;RPS29;RPS18;RPS11;RPS27A;UBA52;RPS13;RPS23;RPS12
RPL30;RPL12;RPL34;RPLP1;RPL9;RPS15;RPS16;RPS15A;RPS19;RPS18;RPL35;RPLP2;RPL37;RPS11;RPS27A;RPL39;RPS13;RPS12;RP
COL15A1;SPARC;ITGA1;LAMC1;PRRC2B;COL3A1;COL1A2;COL4A2;COL4A1;PXDN;PECAM1;COL6A3;TIMP1;A2M;ADAMTS9
COL3A1;COL15A1;COL1A2;COL4A2;COL4A1;COL6A3;LAMC1;TIMP1;A2M;ADAMTS9
COL3A1;COL15A1;COL1A2;COL4A2;COL4A1;COL6A3
COL3A1;COL15A1;COL1A2;COL4A2;COL4A1;PXDN;COL6A3
COL3A1;COL1A2;COL4A2;COL4A1;ITGA1;PECAM1;COL6A3
COL3A1;COL1A2;COL4A2;COL4A1;LAMC1;PRRC2B
COL3A1;COL15A1;COL1A2;COL4A2;COL4A1;COL6A3
IGHM;COL3A1;SPARC;COL1A2;IGKC;COL4A2;COL4A1;IGLC3;IGLC2;IGHA1
COL3A1;COL15A1;COL1A2;COL4A2;COL4A1;PXDN;COL6A3
COL3A1;COL15A1;COL1A2;COL4A2;COL4A1;COL6A3
SPARC;IGFBP5;IGFBP4;COL4A1;SPARCL1;IGFBP7;LAMC1;TIMP1;PRSS23
COL3A1;SPARC;COL1A2;COL4A2;COL4A1;COL6A3;LAMC1
SPARC;IGFBP5;IGFBP4;COL4A1;SPARCL1;IGFBP7;LAMC1;TIMP1;PRSS23
COL3A1;COL4A2;COL4A1;COL6A3;MAPK1;CACNA1C
COL3A1;COL4A2;COL4A1;COL6A3;CACNA1C
COL1A2;COL4A2;COL4A1;PXDN
COL4A2;COL4A1;ITGA1;LAMC1
COL1A2;COL4A2;COL4A1
IGHM;PRKCH;SPARC;DOCK8;ITGA1;GNG11;DOCK10;COL1A2;IGKC;IGLC3;PECAM1;MAPK1;PRKCQ;TIMP1;IGLC2;IGHA1;A2M
IGHM;IGKC;IGLC3;IGLC2
INPP4B;MTMR12;INPP5D;PI4KA;PIK3C3;SYNJ2
NRG1

PACS1;DOCK2;AP2A2
INPP4B;MTMR12;PIK3C3
BCL2
COL3A1;RAPGEF1;LRIG1;RANBP9
NRG1
MICU3
INPP5D;RAPGEF1;JAK3
NRG1
NRG1
PACS1;AP2A2
NRG1
INPP4B;INPP5D;SYNJ2
MICU3
COL3A1;NCK2;RAPGEF1
SMAD3;SMURF1
SMAD3
SMAD3
SMAD3
RPL5;RPL30;RPL3;RPL32;RPL31;RPLP1;RPL34;RPL10A;RPL8;RPL9;RPL6;RPL7;RPS15;RPS4X;RPL7A;RPS14;RPS17;RPS16;RPS19;RP
RPL5;RPL30;RPL3;RPL32;RPL31;RPLP1;RPL34;RPL10A;RPL8;RPL9;RPL6;RPL7;RPS15;RPS4X;RPL7A;RPS14;RPS17;RPS16;RPS19;RP
RPL5;RPL30;RPL3;RPL32;RPL31;RPLP1;RPL34;RPL10A;RPL8;RPL9;RPL6;RPL7;RPS15;RPS4X;RPL7A;RPS14;RPS17;RPS16;RPS19;RP
RPL5;RPL30;RPL3;RPL32;RPL31;RPLP1;RPL34;RPL10A;RPL8;RPL9;RPL6;RPL7;RPS15;RPS4X;RPL7A;RPS14;RPS17;RPS16;RPS19;RP
RPL5;RPL30;RPL3;RPL32;RPL31;RPLP1;RPL34;RPL10A;RPL8;RPL9;RPL6;RPL7;RPS15;RPS4X;RPL7A;RPS14;RPS17;RPS16;RPS19;RP
RPL5;RPL30;RPL3;RPL32;RPL31;RPLP1;RPL34;RPL10A;RPL8;RPL9;RPL6;RPL7;RPS15;RPS4X;RPL7A;RPS14;RPS17;RPS16;RPS19;RP
RPS15;RPS4X;RPS14;RPS17;RPS16;RPS15A;RPS19;UBB;RPS18;RPS3;RPS2;RPS11;RPS27A;RPS10;RPS13;RPS12;RPS9;RPS7;RPS8;
RPS15;RPS4X;RPS14;RPS17;RPS16;RPS15A;RPS19;UBB;RPS18;RPS3;RPS2;RPS11;RPS27A;RPS10;RPS13;RPS12;RPS9;RPS7;RPS8;
RPL5;RPL30;RPL3;RPL32;RPL31;RPLP1;RPL34;RPL10A;RPL8;RPL9;RPL6;RPL7;RPS15;RPS4X;EEF1B2;RPL7A;RPS14;RPS17;RPS16;R
RPL5;RPL30;RPL3;RPL32;RPL31;RPLP1;RPL34;RPL10A;RPL8;RPL9;RPL6;RPL7;RPS15;RPS4X;RPL7A;RPS14;RPS17;RPS16;RPS19;RP
RPL5;RPL30;RPL3;RPL32;RPL31;RPLP1;RPL34;EIF4A3;RPL10A;RPL8;RPL9;RPL6;RPL7;RPS15;RPS4X;RPL7A;RPS14;RPS17;RPS16;RP
RPL5;RPL30;RPL3;RPL32;RPL31;RPLP1;RPL34;RPL10A;RPL8;RPL9;RPL6;RPL7;RPS15;RPS4X;RPL7A;RPS14;RPS17;RPS16;RPS19;RP
RPL5;RPL30;RPL3;RPL32;RPL31;RPLP1;RPL34;EIF4A3;RPL10A;RPL8;RPL9;RPL6;RPL7;RPS15;RPS4X;RPL7A;RPS14;RPS17;RPS16;RP

RPS15;RPS4X;RPS14;RPS17;RPS16;RPS15A;RPS19;UBB;RPS18;RPS3;RPS2;RPS11;RPS27A;RPS10;RPS13;RPS12;RPS9;RPS7;RPS8;F
RPL5;RPL30;RPL3;RPL32;RPL31;RPLP1;RPL34;RPL10A;RPL8;RPL9;RPL6;RPL7;RPS15;RPS4X;RPL7A;RPS14;RPS17;RPS16;RPS19;RPI
RPL5;RPL30;RPL3;RPL32;RPL31;RPLP1;RPL34;RPL10A;RPL8;RPL9;RPL6;RPL7;RPS15;RPS4X;RPL7A;RPS14;RPS17;RPS16;RPS19;RPI
RPL5;RPL30;RPL3;RPL32;RPL31;RPLP1;RPL34;RPL10A;RPL8;RPL9;RPL6;RPL7;RPS15;RPS4X;RPL7A;RPS14;RPS17;RPS16;RPS19;RPI
RPL5;RPL30;RPL3;RPL32;RPL31;RPLP1;RPL34;RPL10A;RPL8;RPL9;RPL6;RPL7;RPS15;RPS4X;RPL7A;RPS14;RPS17;RPS16;RPS19;RPI
RPL5;MT-ND4;RPL30;RPL3;RPL32;RPL31;RPLP1;RPL34;RPL10A;RPL8;MT-ND3;RPL9;RPL6;MT-ND1;RPL7;RPS15;RPS4X;MT-ATP6;RPL7
RPL5;RPL30;RPL3;RPL32;RPL31;RPLP1;RPL34;RPL10A;RPL8;RPL9;RPL6;RPL7;RPS15;RPS4X;RPL7A;RPS14;RPS17;RPS16;RPS19;RPI
RPL5;RPL30;RPL3;RPL32;RPL10;RPLP1;RPLP0;RPL11;RPL10A;RPL8;RPL6;RPL7;RPS4X;RPS14;RPL7A;RPS16;RPS19;RPL18A;RPS18;
RPL5;RPL30;RPL3;RPL32;RPL10;RPLP1;RPLP0;RPL11;RPL10A;RPL8;RPL6;RPL7;RPS4X;RPS14;RPL7A;RPS16;RPS19;RPL18A;RPS18;F
RPL5;RPL30;RPL3;RPL32;RPL10;RPLP1;RPLP0;RPL11;RPL10A;RPL8;RPL6;RPL7;RPS4X;RPS14;RPL7A;RPS16;RPS19;RPL18A;RPS18;F
RPL5;RPL30;RPL3;RPL32;RPL10;RPLP1;RPLP0;RPL11;RPL10A;RPL8;RPL6;RPL7;RPS4X;RPS14;RPL7A;RPS16;RPS19;RPL18A;RPS18;F
RPL5;RPL30;RPL3;RPL32;RPL10;RPLP1;RPLP0;RPL11;RPL10A;RPL8;RPL6;RPL7;RPS4X;RPS14;RPL7A;RPS16;RPS19;RPL18A;RPS18;F
RPL5;RPL30;RPL3;RPL32;RPL10;RPLP1;RPLP0;RPL11;RPL10A;RPL8;RPL6;RPL7;RPS4X;RPS14;RPL7A;RPS16;RPS19;RPL18A;RPS18;F
RPL5;RPL30;RPL3;RPL32;RPL10;RPLP1;RPLP0;RPL11;RPL10A;RPL8;RPL6;RPL7;RPS4X;RPS14;RPL7A;RPS16;RPS19;RPL18A;RPS18;F
RPL5;RPL30;RPL3;RPL32;RPL10;RPLP1;RPLP0;RPL11;RPL10A;RPL8;RPL6;RPL7;RPS4X;RPS14;RPL7A;RPS16;RPS19;RPL18A;RPS18;F
RPS9;RPS7;RPS5;RPS6;RPS3A;RPSA;RPS4X;RPS25;RPS14;RPS16;RPS19;RPS18;RPS3;RPS20;PABPC1;RPS11;RPS27A;RPS10;RPS1
RPS9;RPS7;RPS5;RPS6;RPS3A;RPSA;RPS4X;RPS25;RPS14;RPS16;RPS19;RPS18;RPS3;RPS20;RPS11;RPS27A;RPS10;RPS13;RPS23;
RPL5;RPL30;RPL3;RPL32;RPL10;RPLP1;RPLP0;RPL11;RPL10A;RPL8;RPL6;RPL7;RPS4X;EEF1B2;RPS14;RPL7A;RPS16;RPS19;RPL18A;
RPL5;RPL30;RPL3;RPL32;RPL10;RPLP1;RPLP0;RPL11;RPL10A;RPL8;RPL6;RPL7;RPS4X;RPS14;RPL7A;RPS16;RPS19;RPL18A;RPS18;F
RPL5;RPL30;RPL3;RPL32;RPL10;RPLP1;RPLP0;RPL11;RPL10A;RPL8;RPL6;RPL7;RPS4X;RPS14;RPL7A;RPS16;RPS19;RPL18A;RPS18;F
RPS9;RPS7;RPS5;RPS6;RPS3A;RPSA;RPS4X;EEF1A1;RPS25;RPS14;RPS16;RPS19;RPS18;RPS3;RPS20;RPS11;RPS27A;RPS10;RPS13
RPL5;RPL30;RPL3;RPL32;RPL10;RPLP1;RPLP0;RPL11;RPL10A;RPL8;RPL6;RPL7;RPS4X;RPS14;RPL7A;RPS16;RPS19;RPL18A;RPS18;F
RPL5;RPL30;RPL3;RPL32;RPL10;RPLP1;RPLP0;RPL11;RPL10A;RPL8;RPL6;RPL7;RPS4X;RPS14;RPL7A;RPS16;RPS19;RPL18A;RPS18;F
RPL5;RPL30;RPL3;RPL32;RPL10;RPLP1;RPLP0;RPL11;RPL10A;RPL8;RPL6;RPL7;RPS4X;RPS14;RPL7A;RPS16;RPS19;RPL18A;RPS18;F
RPL5;RPL30;RPL3;RPL32;RPL10;RPLP1;RPLP0;RPL11;RPL10A;RPL8;RPL6;RPL7;RPS4X;RPS14;RPL7A;RPS16;RPS19;RPL18A;RPS18;F
RPL5;RPL30;RPL3;RPL10;RPL32;RPLP1;RPL11;RPLP0;RPL10A;RPL8;RPL6;RPL7;RPS4X;EEF1B2;RPS14;RPL7A;RPS16;RPS19;RPL18A;
RPL5;RPL30;RPL3;RPL10;RPL32;MT-CO1;RPLP1;RPL11;RPLP0;RPL10A;RPL8;RPL6;RPL7;RPS4X;RPS14;RPL7A;RPS16;RPS19;RPL18A
IFITM2;CSF1;EIF4A3;UBE2L6;TNF;LMNB1;CASP3;CFL1;MAP3K8;DUSP4;GBP5;IL4R;VAV1;HLA-E;IFNG;TRAF3;IRF4;IRF1;PSME1;PSME2;
IFITM2;CSF1;EIF4A3;UBE2L6;TNF;ACTB;ACTG1;CFL1;COTL1;MAP3K8;GBP5;IL4R;TAP1;OLFM4;HLA-E;PSME1;PSME2;UBR4;CNN2;RNF
RANBP2;GBP5;PTPN1;EGR1;IFITM2;STAT1;EIF4A3;UBE2L6;PSMB8;HLA-E;BST2;IFNG;IRF4;IRF1;UBC;XAF1;GBP1;GBP4;NUP58;EIF4G2
BST2;PTPN1;EGR1;IFITM2;IRF4;STAT1;IRF1;UBC;XAF1;PSMB8;HLA-E

CSF1;IL2RG;TNF;LMNB1;CNN2;CASP3;CFL1;UBC;STAT4;MAP3K8;N4BP1;MCL1;IL12RB2;IL32;DUSP4;HSPA8;IL4R;YES1;CCL20;STAT1;S
CNN2;IFNG;STAT1;HNRNPA2B1;CFL1;STAT3;STAT4;PSME2;LCP1;IL12RB2;LMNB1
CNN2;IFNG;HNRNPA2B1;CFL1;STAT4;PSME2;LCP1;IL12RB2;LMNB1
CNN2;IFNG;HNRNPA2B1;CFL1;STAT4;PSME2;LCP1;LMNB1
IFNG;IL2RA;CTLA4;RUNX1
RB1;HIST1H1D;HIST1H1E;HIST1H1B;HIST1H1C;LMNB1
ACTR3;HSPA8;FNBP1;M6PR;CD3G;ARPC5;CD3D;ACTB;ACTG1;CD4;ARPC2;ARPC3;UBC;LDLR
RB1;STAT3;H2AFV;NFKB1;HIST2H2AC;LMNB1;CDK6;UBC;HIST1H1D;HIST1H1E;TNIK;HIST1H4C;HIST1H1B;EZH2;HIST1H1C
ACTR3;PAK1;YES1;ARPC2;ARPC3;CFL1;ARPC5;ACTB;ACTG1
CASP3;HIST1H1D;HIST1H1E;HIST1H1B;HIST1H1C
CD40LG;TRAF3;TNFRSF9;UBC;PSME1;REL;PSME2;TNF;PSMB8;BIRC3;PSMB9
STAT3;PSMB8;PSMB9;LMNB1;ARHGAP10;STK24;IRF1;CASP3;UBC;HIST1H1D;TNFSF10;PSME1;HIST1H1E;PSME2;CHMP4B;HIST1H1B;
H2AFV;PSMB8;RUNX1;HIST2H2AC;PSMB9;NR4A2;NR4A3;CDK6;IFNG;IL2RA;UBC;PSME1;CTLA4;PSME2;HIST1H4C
ACTB;ACTG1;HIST2H2AC;LMNB1;PAK1;CAPZB;CFL1;ARHGDIB;RAC2;EPSTI1;STOM;SPTBN1;ACTR3;IL32;RANBP2;TPM4;TPM3;FNBP1;
STAT3;PSMB8;PSMB9;LMNB1;ARHGAP10;STK24;CASP3;UBC;HIST1H1D;TNFSF10;PSME1;HIST1H1E;PSME2;HIST1H1B;HIST1H1C
CD3G;CD3D;NFKB1;PSMB8;PSMB9;PAK1;CD4;UBC;PSME1;PSME2;TAB2;EVL;LCP2
RPL5;RPL30;RPL32;RPL34;RPLP1;RPL8;RPL9;RPL6;RPS4X;RPS15;RPL7A;RPS14;RPS19;RPL18A;RPS18;RPL36AL;RPL36;RPLP2;RPL3
RPL5;RPL30;RPL32;RPL34;RPLP1;RPL8;RPL9;RPL6;RPS4X;RPS15;RPL7A;RPS14;RPS19;RPL18A;RPS18;RPL36AL;RPL36;RPLP2;RPL3
RPL5;RPL30;RPL32;RPL34;RPLP1;RPL8;RPL9;RPL6;RPS4X;RPS15;RPL7A;RPS14;RPS19;RPL18A;RPS18;RPL36AL;RPL36;RPLP2;RPL3
RPL5;RPL30;RPL32;RPL34;RPLP1;RPL8;RPL9;RPL6;RPS4X;RPS15;RPL7A;RPS14;RPS19;RPL18A;RPS18;RPL36AL;RPL36;RPLP2;RPL3
RPL5;RPL30;RPL32;RPL34;RPLP1;RPL8;RPL9;RPL6;RPS4X;RPS15;RPL7A;RPS14;RPS19;RPL18A;RPS18;RPL36AL;RPL36;RPLP2;RPL3
RPL5;RPL30;RPL32;RPL34;RPLP1;RPL8;RPL9;RPL6;RPS4X;RPS15;RPL7A;RPS14;RPS19;RPL18A;RPS18;RPL36AL;RPL36;RPLP2;RPL3
RPS27L;RPS4Y1;RPS4X;RPS15;RPS14;RPS15A;UBB;RPS19;RPS18;RPS3;RPS2;RPS27A;RPS10;RPS13;RPS12;RPS9;RPS7;RPS8;RPS5
RPS27L;RPS4Y1;RPS4X;RPS15;RPS14;RPS15A;UBB;RPS19;RPS18;RPS3;RPS2;RPS27A;RPS10;RPS13;RPS12;RPS9;RPS7;RPS8;RPS5
RPL5;RPL30;RPL32;RPL34;RPLP1;RPL8;RPL9;RPL6;RPS4X;RPS15;RPL7A;RPS14;RPS19;RPL18A;RPS18;RPL36AL;RPL36;RPLP2;RPL3
RPL5;RPL30;RPL32;RPL34;RPLP1;RPL8;RPL9;RPL6;RPS4X;RPS15;RPL7A;RPS14;RPS19;RPL18A;RPS18;RPL36AL;RPL36;RPLP2;RPL3
RPL5;RPL30;RPL32;RPL34;RPLP1;RPL8;RPL9;RPL6;RPS4X;RPS15;RPL7A;RPS14;RPS19;RPL18A;RPS18;RPL36AL;RPL36;RPLP2;RPL3
RPL5;RPL30;RPL32;RPL34;RPLP1;RPL8;RPL9;RPL6;RPS4X;RPS15;RPL7A;RPS14;RPS19;RPL18A;RPS18;RPL36AL;RPL36;RPLP2;RPL3
RPL5;RPL30;RPL32;RPL34;RPLP1;RPL8;RPL9;RPL6;RPS4X;RPS15;RPL7A;RPS14;RPS19;RPL18A;RPS18;RPL36AL;RPL36;RPLP2;RPL3
RPL5;RPL30;RPL32;RPL34;RPLP1;RPL8;RPL9;RPL6;RPS4X;RPS15;RPL7A;RPS14;RPS19;RPL18A;RPS18;RPL36AL;RPL36;RPLP2;RPL3
RPL5;RPL30;RPL32;RPL34;RPLP1;RPL8;RPL9;RPL6;RPS4X;RPS15;RPL7A;RPS14;RPS19;RPL18A;RPS18;RPL36AL;RPL36;RPLP2;RPL3

RPL5;RPL30;RPL32;RPL34;RPLP1;RPL8;RPL9;RPL6;RPS4X;RPS15;RPL7A;RPS14;RPS19;RPL18A;RPS18;RPL36AL;RPL36;RPLP2;RPL3
RPL5;RPL30;RPL32;RPL34;RPLP1;RPL8;RPL9;RPL6;RPS4X;RPS15;RPL7A;RPS14;RPS19;RPL18A;RPS18;RPL36AL;RPL36;RPLP2;RPL3
RPS27L;RPS4Y1;RPS4X;RPS15;RPS14;RPS15A;UBB;RPS19;RPS18;RPS3;RPS2;RPS27A;RPS10;RPS13;RPS12;RPS9;RPS7;RPS8;RPS5
RPS27L;RPS4Y1;RPS4X;RPS15;RPS14;RPS15A;UBB;RPS19;RPS18;RPS3;RPS2;RPS27A;RPS10;RPS13;RPS12;RPS9;RPS7;RPS8;RPS5
RPS27L;RPS4Y1;RPS4X;RPS15;RPS14;RPS15A;UBB;RPS19;RPS18;RPS3;RPS2;RPS27A;RPS10;RPS13;RPS12;RPS9;RPS7;RPS8;RPS5
PPP1R15A;CEBPB
ARL4C;ABCG1
HNRNPDL;STAT4;LCP1
HNRNPDL;STAT4;LCP1
ARL4C;ABCG1
HNRNPDL;STAT4;LCP1
CFLAR
NFKBIA;ANXA1;HNRNPDL;STAT4;LCP1;IL18R1
HSPA5
HSPA5
NFKBIA;DHX9
CFLAR
SRRM2;DDX5;DHX9;WBP11;SF3B1
ANTXR2
CEBPB
NFKBIA;DHX9
SRRM2;DDX5;DHX9;WBP11;SF3B1
CEBPB
PPP1R15A;USP15
CFLAR
RPL30;RPL41;RPL34;RPL12;RPS15;RPS28;RPS15A;RPS27;RPS19;RPS29;RPL27A;RPL37A;RPL36;RPL35;RPL24;RPL27;RPL37;RPS27A
RPL30;RPL41;RPL34;RPL12;RPS15;RPS28;RPS15A;RPS27;RPS19;RPS29;RPL27A;RPL37A;RPL36;RPL35;RPL24;RPL27;RPL37;RPS27A
RPL30;RPL41;RPL34;RPL12;RPS15;RPS28;RPS15A;RPS27;RPS19;RPS29;RPL27A;RPL37A;RPL36;RPL35;RPL24;RPL27;RPL37;RPS27A
RPL30;RPL41;RPL34;RPL12;RPS15;RPS28;RPS15A;RPS27;RPS19;RPS29;RPL27A;RPL37A;RPL36;RPL35;RPL24;RPL27;RPL37;RPS27A
RPL30;RPL41;RPL34;RPL12;RPS15;RPS28;RPS15A;RPS27;RPS19;RPS29;RPL27A;RPL37A;RPL36;RPL35;RPL24;RPL27;RPL37;RPS27A
RPS15;RPS28;RPS15A;RPS27;RPS19;RPS29;RPS27A;RPS24;RPS12;RPS23

RPS15;RPS28;RPS15A;RPS27;RPS19;RPS29;RPS27A;RPS24;RPS12;RPS23
RPL30;RPL41;RPL34;RPL12;RPS15;RPS28;RPS15A;RPS27;RPS19;RPS29;RPL27A;RPL37A;RPL36;RPL35;RPL24;RPL27;RPL37;RPS27A
RPL30;RPL41;RPL34;RPL12;RPS15;RPS28;RPS15A;RPS27;RPS19;RPS29;RPL27A;RPL37A;RPL36;RPL35;RPL24;RPL27;RPL37;RPS27A
RPL30;RPL41;RPL34;RPL12;RPS15;RPS28;RPS15A;RPS27;RPS19;RPS29;RPL27A;RPL37A;RPL36;RPL35;RPL24;RPL27;RPL37;RPS27A
RPL30;RPL41;RPL34;RPL12;RPS15;RPS28;RPS15A;RPS27;RPS19;RPS29;RPL27A;RPL37A;RPL36;RPL35;RPL24;RPL27;RPL37;RPS27A
RPL30;RPL41;RPL34;RPL12;RPS15;RPS28;RPS15A;RPS27;RPS19;RPS29;RPL27A;RPL37A;RPL36;RPL35;RPL24;RPL27;RPL37;RPS27A
RPL30;RPL41;RPL34;RPL12;RPS15;RPS28;RPS15A;RPS27;RPS19;RPS29;RPL27A;RPL37A;RPL36;RPL35;RPL24;RPL27;RPL37;RPS27A
RPL30;RPL41;RPL34;RPL12;RPS15;RPS28;RPS15A;RPS27;RPS19;RPS29;RPL27A;RPL37A;RPL36;RPL35;RPL24;RPL27;RPL37;RPS27A
RPL30;RPL41;RPL34;RPL12;RPS15;RPS28;RPS15A;RPS27;RPS19;RPS29;RPL27A;RPL37A;RPL36;RPL35;RPL24;RPL27;RPL37;RPS27A
RPL30;RPL41;RPL34;RPL12;RPS15;RPS28;RPS15A;RPS27;RPS19;RPS29;RPL27A;RPL37A;RPL36;RPL35;RPL24;RPL27;RPL37;RPS27A
RPL30;RPL41;RPL34;RPL12;RPS15;RPS28;RPS15A;RPS27;RPS19;RPS29;RPL27A;RPL37A;RPL36;RPL35;RPL24;RPL27;RPL37;RPS27A
RPS15;RPS28;RPS15A;RPS27;RPS19;RPS29;RPS27A;RPS24;RPS12;RPS23
RPS15;RPS28;RPS15A;RPS27;RPS19;RPS29;RPS27A;RPS24;RPS12;RPS23
RPS15;RPS28;RPS15A;RPS27;RPS19;RPS29;RPS27A;RPS24;RPS12;RPS23
NCOA2;SETD2;PBRM1;CREBBP;KDM4C;PHF20;KMT2C;ATP2B4;EHMT1;ARID4B;ASH1L;ARID1A;ARID1B;NFKB1;KAT6B;EP400;KAT7;SM
NCOA2;SETD2;PBRM1;CREBBP;KDM4C;PHF20;KMT2C;ATP2B4;EHMT1;ARID4B;ASH1L;ARID1A;ARID1B;NFKB1;KAT6B;EP400;KAT7;SM
CREBBP;PRKCB;RUNX2
MECP2;AGO2;TBL1X;TNRC6B;HIPK2
CD44
WWTR1;HIPK1;RUNX2;HIPK2
SETD2;KMT2C;EHMT1;SMYD3;ASH1L;NFKB1
GBF1;EXOC4;EXOC6;ASAP1;RAB11A
MECP2
PRKCB;LMNA;NUP153;NUP98;LPIN2
APP;AP1G1;GOLGB1;GBF1;SNX9;PUM1;IGF2R;PICALM
GNG2;GNB1;AKT3;PLCB1;GNG11;PIK3R5
PBRM1;RYBP;ARID2;ARID1A;ARID1B;HIPK2
MECP2;TBL1X
APOA1;HBP1;APOA4;APOB
NCOA2;CREBBP;APOA1;RORA;TBL1X;APOB;ARNTL
GNG2;GNB1;AKT3;GNG11;PIK3R5

ELMO1;FYN;DOCK2
CREBBP;RPS6KA5;FYN;NFKB1;RELB
MTMR1;DOCK9;CUL3;RASGRF2;WIPF2;DOCK8;MSI2;ARHGAP15;IQGAP2;DOCK10;STK10;SCFD1;PLEKHG1;RALGAPA1;USP9X;ARAP2;
CREBBP;CBFB;PRKCB;RUNX2;RUNX1
NCOR1;NR1D2;RORA;NR3C1;ESR2;NR3C2
TRRAP;KMT2C;EHMT1;PHF21A;NSD1;UTY;SMYD3;ARID2;NCOA1;NCOA2;CREBBP;KDM4C;PRMT3;ARID5B;ELP3;ASH1L;ARID1A;SMAR
TRRAP;KMT2C;EHMT1;PHF21A;NSD1;UTY;SMYD3;ARID2;NCOA1;NCOA2;CREBBP;KDM4C;PRMT3;ARID5B;ELP3;ASH1L;ARID1A;SMAR
TNRC6C;CAMK2D;NCOR1;TBL1XR1;CAMK4;PTEN;OPRM1;PTPN4;TNRC6A;FKBP5;TNRC6B;HIPK2
CREBBP;CBFB;PCGF5;PRKCB;CCNH;KMT2C;TCF12;ARID1A;SMARCA2;PHC3;ARID1B;RUNX2;RUNX1;HIPK2;TNRC6C;KAT2B;ITCH;CCN
CBFB;PCGF5;ARID2;ARID1A;SMARCA2;ARID1B;PHC3;RUNX1;HIPK2
CBFB;RUNX2;RUNX1
NCOA1;NCOA2;CREBBP;NCOR1;CRTC3;CHD9;TBL1XR1;CAMK4;NRF1;GABPB1
INPP4A;MTMR1;INPP4B;MTMR3;INPP5D;PTEN;PIP4K2A;PIK3CD;PIK3CB;SBF2;PIK3R5
HDAC4;CBFB;RUNX2
CD44
NCOA1;NCOA2;CREBBP;CRTC3;CHD9;RORA;NR3C1;HIF1A;ARNTL;PPP1CB;NCOR1;TBL1XR1;BTRC
CYFIP2;ITSN2;MTMR1;ROCK1;ROCK2;DOCK9;RASGRF2;DOCK8;MTR;MSI2;ARHGAP15;IQGAP2;DOCK10;PREX1;STK10;DOCK11;CAPZ
NOTCH2;HDAC4;KAT2B;CREBBP;NCOR1;MAML2;TBL1XR1
CBFB;RUNX1
CYFIP2;ITSN2;DOCK9;RASGRF2;DOCK8;MTR;MSI2;PPP1CB;DOCK10;PREX1;DOCK11;STK10;CAPZB;PPP2R5E;NCK2;ANKFY1;EMB;VAV
TNRC6C;CAMK2D;NCOR1;TBL1XR1;CAMK4;TNRC6A;TNRC6B;HIPK2
CYFIP2;ITSN2;DOCK9;RASGRF2;DOCK8;MTR;MSI2;PPP1CB;DOCK10;PREX1;DOCK11;STK10;CAPZB;PPP2R5E;NCK2;ANKFY1;EMB;VAV
NCOA1;NCOA2;CREBBP;NCOR1;CRTC3;CHD9;TBL1XR1;RORA;EMB;ARNTL

PS9;RPS7;	R-HSA-156930;R-HSA-156915;R-HSA-156912;R-HSA-156923;R-HSA-156907
RPS12;RPS	R-HSA-156930;R-HSA-156915;R-HSA-156912;R-HSA-156913;R-HSA-156923;R-HSA-156907;R-HSA-1569
PS9;RPS7;	R-HSA-927789
PS9;RPS7;	R-HSA-72673;R-HSA-72676
PS12;RPS	R-HSA-1799326;R-HSA-1799330;R-HSA-1799329;R-HSA-1799335;R-HSA-1799332
PS9;RPS7;	R-HSA-192704;R-HSA-192841
PS9;RPS7;	R-HSA-156832;R-HSA-156823;R-HSA-156826
PS9;RPS7;	R-HSA-72672;R-HSA-72619;R-HSA-72671
RPS23	R-HSA-156808;R-HSA-157849
RPS23	R-HSA-72697;R-HSA-72621
PS9;RPS7;	R-HSA-6791223;R-HSA-6791222;R-HSA-6791221;R-HSA-6791218;R-HSA-6791228;R-HSA-6791227
PS12;RPS	R-HSA-927836;R-HSA-927789;R-HSA-927832;R-HSA-927813;R-HSA-927889
PS12;RPS	R-HSA-927836;R-HSA-927832;R-HSA-927813;R-HSA-927889
23	R-HSA-9729232;R-HSA-9727944
PS9;RPS7;	R-HSA-72673;R-HSA-72672;R-HSA-156832;R-HSA-72676;R-HSA-72619;R-HSA-156808;R-HSA-72621;R-H
PS9;RPS7;	R-HSA-141671;R-HSA-141691;R-HSA-141673
PS9;RPS7;	R-HSA-72673;R-HSA-72672;R-HSA-72691;R-HSA-72676;R-HSA-72697;R-HSA-72619;R-HSA-156808;R-H
TP6V1G1;F	R-HSA-9633008;R-HSA-9639286;R-HSA-9635892;R-HSA-9645598;R-HSA-9640195;R-HSA-9640167;R-HS
PS9;RPS7;	R-HSA-6791223;R-HSA-6791222;R-HSA-6791221;R-HSA-6791218;R-HSA-6791228;R-HSA-6791227;R-HS
RPL39;RPS	R-HSA-5389842;R-HSA-156930;R-HSA-5419281;R-HSA-1799326;R-HSA-156808;R-HSA-5389848;R-HSA-
A;RPL24;R	R-HSA-927789
	R-HSA-9729232;R-HSA-9727869;R-HSA-9727944
A;RPL24;R	R-HSA-72673;R-HSA-72676
A;RPL24;R	R-HSA-192704;R-HSA-192841
RPL13A;R	R-HSA-1799326;R-HSA-1799330;R-HSA-1799329;R-HSA-1799335;R-HSA-1799332
A;RPL24;R	R-HSA-72672;R-HSA-72619;R-HSA-72671
A;RPL24;R	R-HSA-156832;R-HSA-156823;R-HSA-156826
	R-HSA-156808;R-HSA-157849
	R-HSA-72697;R-HSA-72621

3A;RPSA;E	R-HSA-156915;R-HSA-156912;R-HSA-156913;R-HSA-156923;R-HSA-156907;R-HSA-156910;R-HSA-1569
A;NCL;RPL	R-HSA-6791223;R-HSA-6791222;R-HSA-6791221;R-HSA-6791218;R-HSA-6791228;R-HSA-6791227
A;RPL24;RI	R-HSA-927836;R-HSA-927789;R-HSA-927832;R-HSA-927813;R-HSA-927889
A;EEF1A1;I	R-HSA-156915;R-HSA-156912;R-HSA-156923;R-HSA-156907
A;RPL24;RI	R-HSA-927836;R-HSA-927832;R-HSA-927813;R-HSA-927889
A;RPL24;RI	R-HSA-72673;R-HSA-72672;R-HSA-156832;R-HSA-72676;R-HSA-72619;R-HSA-156808;R-HSA-72621;R-H
A;RPL24;RI	R-HSA-141671;R-HSA-141691;R-HSA-141673
A;RPL24;RI	R-HSA-72673;R-HSA-72672;R-HSA-72691;R-HSA-72676;R-HSA-72697;R-HSA-72619;R-HSA-156808;R-H
A;NCL;RPL	R-HSA-6791223;R-HSA-6791222;R-HSA-6791221;R-HSA-6791218;R-HSA-6791228;R-HSA-6791227;R-HS
5;RPL22;RI	R-HSA-5389842;R-HSA-5419281;R-HSA-1799326;R-HSA-5368279;R-HSA-156808;R-HSA-5389848;R-HSA
	R-HSA-72691
	R-HSA-5357776;R-HSA-5357904;R-HSA-5357780;R-HSA-5357860;R-HSA-5357757
	R-HSA-5357776;R-HSA-5357904;R-HSA-5357845;R-HSA-5357780;R-HSA-9793680;R-HSA-9817362;R-HS
	R-HSA-9817397;R-HSA-5634228;R-HSA-5357776;R-HSA-5357904;R-HSA-5693108;R-HSA-5357845;R-HS
	R-HSA-9654752;R-HSA-9650710;R-HSA-9654774
	R-HSA-727819;R-HSA-688136;R-HSA-9603905;R-HSA-688985;R-HSA-688137;R-HSA-706479;R-HSA-168
	R-HSA-727819;R-HSA-688136;R-HSA-688985;R-HSA-688137;R-HSA-706479;R-HSA-168184
	R-HSA-450054;R-HSA-727819;R-HSA-452100;R-HSA-452097;R-HSA-8950415;R-HSA-450063;R-HSA-452
	R-HSA-451942;R-HSA-450054;R-HSA-912527;R-HSA-919404;R-HSA-452100;R-HSA-508292;R-HSA-4520
	R-HSA-9817397;R-HSA-5634228;R-HSA-5693108;R-HSA-5357845;R-HSA-9793680;R-HSA-9817362;R-HS
	R-HSA-450054;R-HSA-727819;R-HSA-452100;R-HSA-452097;R-HSA-8950415;R-HSA-450063;R-HSA-452
	R-HSA-5662597;R-HSA-5662618;R-HSA-5662619;R-HSA-5662617
	R-HSA-9613202;R-HSA-9621042;R-HSA-9031624;R-HSA-9620723;R-HSA-9613207;R-HSA-9620663;R-HS
	R-HSA-168166;R-HSA-5684275;R-HSA-5684273;R-HSA-450337;R-HSA-9013985;R-HSA-9013986;R-HSA-
	R-HSA-8877396;R-HSA-8877391;R-HSA-8877385
	R-HSA-936941;R-HSA-168166;R-HSA-5684275;R-HSA-5684273;R-HSA-450337;R-HSA-166245;R-HSA-53
	R-HSA-936941;R-HSA-168166;R-HSA-5684275;R-HSA-5684273;R-HSA-450337;R-HSA-166245;R-HSA-53
	R-HSA-5662597;R-HSA-5662618;R-HSA-5662619;R-HSA-5662617
	R-HSA-450054;R-HSA-452100;R-HSA-688136;R-HSA-688137;R-HSA-452097;R-HSA-8950415;R-HSA-450
	R-HSA-8950344;R-HSA-8950218;R-HSA-8950732;R-HSA-8950415;R-HSA-9617941;R-HSA-8950721;R-HS
	R-HSA-9613202;R-HSA-9031624;R-HSA-9613207;R-HSA-9613210;R-HSA-9031616;R-HSA-9612483;R-HS

S3A;RPSA	R-HSA-927789
S3A;RPSA	R-HSA-72673;R-HSA-72676
S3A;RPSA	R-HSA-192704;R-HSA-192841
13A;RPS3/	R-HSA-1799326;R-HSA-1799330;R-HSA-1799329;R-HSA-1799335;R-HSA-1799332
S3A;RPSA	R-HSA-72672;R-HSA-72619;R-HSA-72671
S3A;RPSA	R-HSA-156832;R-HSA-156823;R-HSA-156826
	R-HSA-156808;R-HSA-157849
	R-HSA-72697;R-HSA-72621
S3A;RPSA	R-HSA-6791223;R-HSA-6791222;R-HSA-6791221;R-HSA-6791218;R-HSA-6791228;R-HSA-6791227
S3A;RPSA	R-HSA-927836;R-HSA-927789;R-HSA-927832;R-HSA-927813;R-HSA-927889
S3A;RPSA	R-HSA-156915;R-HSA-156912;R-HSA-156923;R-HSA-156907
PL13A;RPS	R-HSA-141671;R-HSA-141691;R-HSA-141673;R-HSA-6800138
S3A;RPSA	R-HSA-927836;R-HSA-927832;R-HSA-927813;R-HSA-927889
S3A;RPSA	R-HSA-156915;R-HSA-156912;R-HSA-156913;R-HSA-156923;R-HSA-156907;R-HSA-156908;R-HSA-1569
	R-HSA-9729232;R-HSA-9727944
S3A;RPSA	R-HSA-72673;R-HSA-72672;R-HSA-156832;R-HSA-72676;R-HSA-72619;R-HSA-156808;R-HSA-72621;R-H
S3A;RPSA	R-HSA-72673;R-HSA-72672;R-HSA-72691;R-HSA-72676;R-HSA-72697;R-HSA-72619;R-HSA-156808;R-H
PL13A;RPS	R-HSA-6791223;R-HSA-6791222;R-HSA-6790982;R-HSA-6791221;R-HSA-6791218;R-HSA-6791228;R-HS
RPS6;RPL1	R-HSA-5389842;R-HSA-5419281;R-HSA-1799326;R-HSA-5368279;R-HSA-156808;R-HSA-5389848;R-HSA
PL13A;RPS	R-HSA-6791223;R-HSA-6791222;R-HSA-6790982;R-HSA-6791221;R-HSA-6791218;R-HSA-6791228;R-HS
	R-HSA-879917;R-HSA-1112690;R-HSA-6789615;R-HSA-8983011;R-HSA-6785047;R-HSA-8950426;R-HSA
	R-HSA-389381;R-HSA-2424484;R-HSA-2424485;R-HSA-202241;R-HSA-2424486;R-HSA-2424487;R-HSA-
	R-HSA-6789615;R-HSA-9680348;R-HSA-9733316;R-HSA-8950426;R-HSA-912734;R-HSA-9706304;R-HSA
	R-HSA-373746;R-HSA-434990;R-HSA-9680443;R-HSA-399951;R-HSA-419087;R-HSA-419197;R-HSA-419
	R-HSA-6790036;R-HSA-6789615
	R-HSA-373746;R-HSA-434990
	R-HSA-9012671;R-HSA-8983011
	R-HSA-202214;R-HSA-8855375;R-HSA-202233;R-HSA-8852200
	R-HSA-202241;R-HSA-8855381;R-HSA-202375;R-HSA-202214;R-HSA-202325;R-HSA-8855375;R-HSA-20
	R-HSA-9733316;R-HSA-389159;R-HSA-9680389;R-HSA-9680388;R-HSA-9817983;R-HSA-9680348;R-HSA
	R-HSA-9706293;R-HSA-9706304;R-HSA-9706354;R-HSA-9706350

	R-HSA-5578663
	R-HSA-1433501;R-HSA-205244
	R-HSA-5577259
	R-HSA-8950426
	R-HSA-5578663;R-HSA-5577259
	R-HSA-8950426
	R-HSA-6785047
	R-HSA-8950426
	R-HSA-9706356
	R-HSA-5662614;R-HSA-5662615;R-HSA-5662610;R-HSA-5662608;R-HSA-5662622;R-HSA-5662623;R-HS
	R-HSA-5662614;R-HSA-5662615;R-HSA-5662610;R-HSA-5662608;R-HSA-5662622;R-HSA-5662623;R-HS
	R-HSA-9013145;R-HSA-9013144;R-HSA-9013143
	R-HSA-428287;R-HSA-428296
	R-HSA-1368081;R-HSA-5663264;R-HSA-1801587;R-HSA-5663293
	R-HSA-5637686;R-HSA-5159245;R-HSA-5244692;R-HSA-3318486;R-HSA-3301237;R-HSA-5205799;R-HS
	R-HSA-5637686;R-HSA-5159245;R-HSA-5244692;R-HSA-3318486;R-HSA-3301237;R-HSA-5205799;R-HS
	R-HSA-2730872;R-HSA-169683;R-HSA-169680;R-HSA-2730849;R-HSA-2730867
	R-HSA-9018766;R-HSA-8981637;R-HSA-9013145;R-HSA-9013144;R-HSA-9014296;R-HSA-9018778;R-HS
	R-HSA-8938440;R-HSA-8935785;R-HSA-8938507;R-HSA-8937134;R-HSA-8937728;R-HSA-8936068;R-HS
	R-HSA-2025882;R-HSA-2025890
	R-HSA-6786854;R-HSA-6785722
	R-HSA-2730872;R-HSA-169680;R-HSA-4420052;R-HSA-398193;R-HSA-3965444;R-HSA-4518575;R-HSA-
	R-HSA-426522;R-HSA-426489
	R-HSA-442732;R-HSA-442760
	R-HSA-9013161;R-HSA-9013157;R-HSA-9013159
	R-HSA-6785722
;NUP98;HN	R-HSA-9018766;R-HSA-5666001;R-HSA-5666129;R-HSA-8981637;R-HSA-5666008;R-HSA-5625797;R-HS
;NUP98;HN	R-HSA-9018766;R-HSA-5666001;R-HSA-5666129;R-HSA-8981637;R-HSA-5666008;R-HSA-5625797;R-HS
	R-HSA-8936584;R-HSA-8937097;R-HSA-8937037;R-HSA-8934735;R-HSA-8938176;R-HSA-8938328;R-HS
	R-HSA-428287;R-HSA-428296
	R-HSA-5637686;R-HSA-5159245;R-HSA-4754187;R-HSA-5244692;R-HSA-3318486;R-HSA-5205799;R-HS

	R-HSA-5637686;R-HSA-5159245;R-HSA-4754187;R-HSA-5244692;R-HSA-3318486;R-HSA-5205799;R-HS
	R-HSA-4396371;R-HSA-1980078;R-HSA-1980047;R-HSA-1852623;R-HSA-1912393;R-HSA-1912394;R-HS
	R-HSA-2220964;R-HSA-2220967;R-HSA-2220966;R-HSA-2220971;R-HSA-4396393;R-HSA-4396392;R-HS
	R-HSA-2220964;R-HSA-2220967;R-HSA-2220966;R-HSA-2220971;R-HSA-4396393;R-HSA-4396392;R-HS
	R-HSA-2220964;R-HSA-2220967;R-HSA-2220966;R-HSA-2220971;R-HSA-4396393;R-HSA-4396392;R-HS
	R-HSA-2220964;R-HSA-2220967;R-HSA-2220966;R-HSA-2220971;R-HSA-4396393;R-HSA-4396392;R-HS
	R-HSA-2220964;R-HSA-2220967;R-HSA-2220966;R-HSA-2220971;R-HSA-4396393;R-HSA-4396392;R-HS
	R-HSA-1801587
	R-HSA-6802919;R-HSA-9656211;R-HSA-6802918;R-HSA-9670418;R-HSA-9656209;R-HSA-9670417;R-HS
	R-HSA-5660980;R-HSA-5660660;R-HSA-5624486;R-HSA-5624473;R-HSA-5660666
	R-HSA-9614562;R-HSA-199299;R-HSA-9614423;R-HSA-9614564
	R-HSA-9729500;R-HSA-9756494;R-HSA-9768142;R-HSA-9705320;R-HSA-9708889;R-HSA-9729481;R-HS
	R-HSA-9694656;R-HSA-9696980;R-HSA-9729500;R-HSA-9756494;R-HSA-9729279;R-HSA-9768142;R-HS
	R-HSA-9005561;R-HSA-9005585
	R-HSA-9005561;R-HSA-9005585
	R-HSA-9005561;R-HSA-9005585
	R-HSA-9005561;R-HSA-9005585
	R-HSA-1169395;R-HSA-1678843;R-HSA-1169394;R-HSA-1678842;R-HSA-1169398;R-HSA-1169403;R-HSA
Q15004	R-HSA-5653838;R-HSA-5655193
Q15004	R-HSA-5653838;R-HSA-5655193
Q15004	R-HSA-5653838;R-HSA-5655193
P62328	R-HSA-481007
P62328	R-HSA-481007
P62328	R-HSA-481007
Q15004	R-HSA-5653838;R-HSA-5655193
P62328	R-HSA-481007
	R-HSA-4085087;R-HSA-114563;R-HSA-202727;R-HSA-4539779;R-HSA-216069;R-HSA-199050;R-HSA-26
	R-HSA-1474197;R-HSA-1564179;R-HSA-3791295;R-HSA-1564112;R-HSA-1604690;R-HSA-1602454;R-HS
	R-HSA-8939066;R-HSA-8938251;R-HSA-8939054;R-HSA-8938382;R-HSA-8938228;R-HSA-8938389;R-HS
	R-HSA-428287;R-HSA-428296
	R-HSA-381543;R-HSA-381537;R-HSA-8952289;R-HSA-381500;R-HSA-381518;R-HSA-381545;R-HSA-381

R-HSA-8944265;R-HSA-2002460;R-HSA-1564179;R-HSA-216069;R-HSA-8944262;R-HSA-216082;R-HSA-
R-HSA-8944265;R-HSA-8944240;R-HSA-8944262;R-HSA-8944214;R-HSA-8944247
R-HSA-1474197;R-HSA-1564179;R-HSA-1564112;R-HSA-1564120;R-HSA-1454822;R-HSA-2485111;R-HS
R-HSA-8938382;R-HSA-8938371
R-HSA-2395302;R-HSA-2395340;R-HSA-1474266;R-HSA-2396234;R-HSA-2559639;R-HSA-2213201;R-HS
R-HSA-5229194;R-HSA-5223304;R-HSA-5244669
R-HSA-8948232;R-HSA-8944265;R-HSA-2002460;R-HSA-8948234;R-HSA-8948224;R-HSA-8948226;R-HS
R-HSA-8952289
R-HSA-264871;R-HSA-264865
R-HSA-9008137;R-HSA-8937864;R-HSA-8985227;R-HSA-9008877;R-HSA-8939852;R-HSA-8985485;R-HS
R-HSA-2465890;R-HSA-2327738;R-HSA-2731075
R-HSA-2396234
R-HSA-8951926;R-HSA-8951910
R-HSA-8948232;R-HSA-8944265;R-HSA-2002460;R-HSA-8948234;R-HSA-2395302;R-HSA-8948224;R-HS
R-HSA-392053;R-HSA-392054;R-HSA-392051;R-HSA-391868;R-HSA-375151;R-HSA-391871;R-HSA-3918
R-HSA-549385;R-HSA-1368087;R-HSA-1368081;R-HSA-400256;R-HSA-400267;R-HSA-5663174;R-HSA-1
AMK2D;CU R-HSA-9664664;R-HSA-5339713;R-HSA-1839112;R-HSA-9664646;R-HSA-1839107;R-HSA-9724090;R-HS
OGT;RCOR R-HSA-8865605;R-HSA-4754187;R-HSA-5159245;R-HSA-5244692;R-HSA-3321975;R-HSA-3301237;R-HS
OGT;RCOR R-HSA-8865605;R-HSA-4754187;R-HSA-5159245;R-HSA-5244692;R-HSA-3321975;R-HSA-3301237;R-HS
R-HSA-382096;R-HSA-376419
R-HSA-6809254;R-HSA-1675994;R-HSA-199456;R-HSA-1675836;R-HSA-6809793;R-HSA-1676177;R-HSA
R-HSA-1676185;R-HSA-6809254;R-HSA-1675994;R-HSA-1483219;R-HSA-1676177;R-HSA-1676048;R-HS
R-HSA-9023592;R-HSA-9005995;R-HSA-9022314;R-HSA-9022625;R-HSA-9021888;R-HSA-9018594;R-HS
R-HSA-8939066;R-HSA-8938251;R-HSA-8939054;R-HSA-8938382;R-HSA-8938228;R-HSA-8938389;R-HS
R-HSA-9022728;R-HSA-9022734;R-HSA-9021953;R-HSA-9021952;R-HSA-9021954;R-HSA-9017988;R-HS
R-HSA-9013145;R-HSA-9013144;R-HSA-9013143
R-HSA-6802919;R-HSA-9656211;R-HSA-6802918;R-HSA-9656209;R-HSA-6802916;R-HSA-9652816;R-HS
R-HSA-5637686;R-HSA-5159245;R-HSA-5244692;R-HSA-5638332;R-HSA-5638333;R-HSA-5638141;R-HS
R-HSA-169683;R-HSA-2730872;R-HSA-169680;R-HSA-2730849;R-HSA-2730867
R-HSA-1368081;R-HSA-5663264;R-HSA-1801587;R-HSA-5663293
R-HSA-428287;R-HSA-428296

	R-HSA-8936584;R-HSA-8937097;R-HSA-8931981;R-HSA-8938382;R-HSA-8936068;R-HSA-8938887;R-HS
	R-HSA-9027273;R-HSA-9027272;R-HSA-9027275;R-HSA-9027274;R-HSA-9029149;R-HSA-9029151;R-HS
	R-HSA-9005585
	R-HSA-212356;R-HSA-350058
	R-HSA-927789
	R-HSA-72673;R-HSA-72676
	R-HSA-192704;R-HSA-192841
	R-HSA-1799326;R-HSA-1799330;R-HSA-1799329;R-HSA-1799335;R-HSA-1799332
	R-HSA-72672;R-HSA-72619;R-HSA-72671
	R-HSA-156832;R-HSA-156823;R-HSA-156826
	R-HSA-6791223;R-HSA-6791222;R-HSA-6791221;R-HSA-6791218;R-HSA-6791228;R-HSA-6791227
	R-HSA-927836;R-HSA-927789;R-HSA-927832;R-HSA-927813;R-HSA-927889
	R-HSA-156915;R-HSA-156912;R-HSA-156923;R-HSA-156907
	R-HSA-3371554;R-HSA-5324617;R-HSA-5082369;R-HSA-5082384
	R-HSA-927836;R-HSA-927832;R-HSA-927813;R-HSA-927889
	R-HSA-156915;R-HSA-156912;R-HSA-156913;R-HSA-156923;R-HSA-156907;R-HSA-156910
	R-HSA-72673;R-HSA-72672;R-HSA-156832;R-HSA-72676;R-HSA-72619;R-HSA-156808;R-HSA-72621;R-H
	R-HSA-141671;R-HSA-141691;R-HSA-141673
	R-HSA-72673;R-HSA-72672;R-HSA-72691;R-HSA-72676;R-HSA-72697;R-HSA-72619;R-HSA-156808;R-H
	R-HSA-6791223;R-HSA-6791222;R-HSA-6791221;R-HSA-6791218;R-HSA-6791228;R-HSA-6791227;R-HS
O3;MT-CY	R-HSA-6791223;R-HSA-6791222;R-HSA-6791221;R-HSA-6791218;R-HSA-6791228;R-HSA-6791227;R-HS
	R-HSA-9729232
	R-HSA-2408529;R-HSA-5333615
	R-HSA-1799326;R-HSA-156808;R-HSA-156823;R-HSA-156826;R-HSA-157849;R-HSA-72671;R-HSA-7267
	R-HSA-9729232;R-HSA-9727869;R-HSA-9727944
A;RPS3A;R	R-HSA-927789
A;RPS3A;R	R-HSA-72673;R-HSA-72676
;RPS6;RPL	R-HSA-1799326;R-HSA-1799330;R-HSA-1799329;R-HSA-1799335;R-HSA-1799332
A;RPS3A;R	R-HSA-192704;R-HSA-192841
A;RPS3A;R	R-HSA-72672;R-HSA-72619;R-HSA-72671
A;RPS3A;R	R-HSA-156832;R-HSA-156823;R-HSA-156826

	R-HSA-156808;R-HSA-157849
	R-HSA-72697;R-HSA-72621
2;RPL13A;I	R-HSA-156915;R-HSA-156912;R-HSA-156913;R-HSA-156923;R-HSA-156907;R-HSA-156910;R-HSA-1569
A;RPS3A;R	R-HSA-6791223;R-HSA-6791222;R-HSA-6791221;R-HSA-6791218;R-HSA-6791228;R-HSA-6791227
A;RPS3A;R	R-HSA-927836;R-HSA-927789;R-HSA-927832;R-HSA-927813;R-HSA-927889
A;RPS3A;R	R-HSA-156915;R-HSA-156912;R-HSA-156923;R-HSA-156907
A;RPS3A;R	R-HSA-927836;R-HSA-927832;R-HSA-927813;R-HSA-927889
A;RPS3A;R	R-HSA-72673;R-HSA-72672;R-HSA-156832;R-HSA-72676;R-HSA-72619;R-HSA-156808;R-HSA-72621;R-I
A;RPS3A;R	R-HSA-141671;R-HSA-141691;R-HSA-141673
A;RPS3A;R	R-HSA-72673;R-HSA-72672;R-HSA-72691;R-HSA-72676;R-HSA-72697;R-HSA-72619;R-HSA-156808;R-H
A;RPS3A;R	R-HSA-6791223;R-HSA-6791222;R-HSA-6791221;R-HSA-6791218;R-HSA-6791228;R-HSA-6791227;R-HS
A;RPS3A;R	R-HSA-6791223;R-HSA-6791222;R-HSA-6791221;R-HSA-6791218;R-HSA-6791228;R-HSA-6791227;R-HS
	R-HSA-72691
	R-HSA-6785722
	R-HSA-6785722
	R-HSA-6785722
	R-HSA-6799191;R-HSA-164834;R-HSA-164832;R-HSA-163217;R-HSA-6799202;R-HSA-6799199;R-HSA-6
	R-HSA-6785722
	R-HSA-6799191;R-HSA-6799202;R-HSA-6799199;R-HSA-6799197;R-HSA-6799196;R-HSA-6799179
	R-HSA-6799191;R-HSA-163217;R-HSA-6799202;R-HSA-6799199;R-HSA-6799197;R-HSA-6799196;R-HSA
	R-HSA-6799191;R-HSA-164834;R-HSA-164832;R-HSA-163217;R-HSA-6799202;R-HSA-6799199;R-HSA-6
	R-HSA-389348;R-HSA-389350;R-HSA-388814;R-HSA-389352;R-HSA-389354
	R-HSA-3149434;R-HSA-184269
	R-HSA-1475028;R-HSA-1475032
	R-HSA-3149434;R-HSA-184269
	R-HSA-2972956;R-HSA-452515
	R-HSA-388833;R-HSA-388829;R-HSA-389532;R-HSA-390329;R-HSA-388809
	R-HSA-8944104;R-HSA-2316434;R-HSA-6807077;R-HSA-8857925;R-HSA-111925;R-HSA-8951727;R-HSA
	R-HSA-3149434;R-HSA-184269
	R-HSA-6797606;R-HSA-6797712;R-HSA-6797616
	R-HSA-389381;R-HSA-389348;R-HSA-389158;R-HSA-389350;R-HSA-388833;R-HSA-388832;R-HSA-3888

R-HSA-9670418;R-HSA-9670417;R-HSA-9670416;R-HSA-9670426;R-HSA-9670414;R-HSA-9670413;R-HS
R-HSA-9670418;R-HSA-9670417;R-HSA-9670416;R-HSA-9670426;R-HSA-9670414;R-HSA-9670413;R-HS
R-HSA-927789
R-HSA-72673;R-HSA-72676
R-HSA-192704;R-HSA-192841
R-HSA-1799326;R-HSA-1799330;R-HSA-1799329;R-HSA-1799335;R-HSA-1799332
R-HSA-72672;R-HSA-72619;R-HSA-72671
R-HSA-156808;R-HSA-157849
R-HSA-72697;R-HSA-72621
R-HSA-6791223;R-HSA-6791222;R-HSA-6791221;R-HSA-6791218;R-HSA-6791228;R-HSA-6791227
R-HSA-927836;R-HSA-927789;R-HSA-927832;R-HSA-927813;R-HSA-927889
R-HSA-3371554;R-HSA-5324617;R-HSA-5082369;R-HSA-5082384
R-HSA-156915;R-HSA-156912;R-HSA-156923;R-HSA-156907
R-HSA-927836;R-HSA-927832;R-HSA-927813;R-HSA-927889
R-HSA-156823;R-HSA-156826
R-HSA-3371554;R-HSA-5324617;R-HSA-5082356;R-HSA-5082369;R-HSA-5082384
R-HSA-141671;R-HSA-141691;R-HSA-141673
R-HSA-3371586;R-HSA-4793819;R-HSA-5082409;R-HSA-5324632
R-HSA-72673;R-HSA-72672;R-HSA-72691;R-HSA-72676;R-HSA-156823;R-HSA-156826;R-HSA-72697;R-I
R-HSA-72673;R-HSA-72672;R-HSA-72691;R-HSA-72676;R-HSA-72697;R-HSA-72619;R-HSA-156808;R-H
R-HSA-5251955;R-HSA-5082409;R-HSA-5251959;R-HSA-5324632;R-HSA-5082369;R-HSA-3371518;R-HS
R-HSA-5251955;R-HSA-5251942;R-HSA-5251959;R-HSA-3371467;R-HSA-5252041;R-HSA-3371518;R-HS
R-HSA-6785895;R-HSA-6790036;R-HSA-6789615;R-HSA-6790041
R-HSA-428287;R-HSA-428296
R-HSA-450054;R-HSA-6785895;R-HSA-452100;R-HSA-8950415;R-HSA-452097;R-HSA-450063;R-HSA-89
R-HSA-450054;R-HSA-6785895;R-HSA-452100;R-HSA-8950415;R-HSA-452097;R-HSA-450063;R-HSA-89
R-HSA-6798020;R-HSA-6798031;R-HSA-6803917;R-HSA-6798044;R-HSA-6803914
R-HSA-9605063;R-HSA-9618407;R-HSA-9618405;R-HSA-9618394;R-HSA-9618392;R-HSA-9028533
R-HSA-6798020;R-HSA-6798031;R-HSA-6803917;R-HSA-6798044;R-HSA-6803914
R-HSA-9654752;R-HSA-9650710;R-HSA-9654774
R-HSA-9021601;R-HSA-9618394;R-HSA-9021600;R-HSA-9618392;R-HSA-9625465;R-HSA-9009378;R-HS

R-HSA-5228811;R-HSA-9630921
R-HSA-6805399;R-HSA-6799815;R-HSA-6798020;R-HSA-6800001;R-HSA-6798031;R-HSA-6803917;R-HS
R-HSA-209536;R-HSA-193691;R-HSA-193705
R-HSA-1791150
R-HSA-168166;R-HSA-937337;R-HSA-933527;R-HSA-933525;R-HSA-933538;R-HSA-9705145;R-HSA-168
R-HSA-5357776;R-HSA-5634228;R-HSA-5693108;R-HSA-209536;R-HSA-83586;R-HSA-83650;R-HSA-535
R-HSA-9605063;R-HSA-9028533
R-HSA-381158;R-HSA-1791150
R-HSA-8877396;R-HSA-8877391;R-HSA-8877385
R-HSA-6799815;R-HSA-6800001
R-HSA-9604480;R-HSA-168140
R-HSA-6786854;R-HSA-6785722
R-HSA-6785722
R-HSA-5662614;R-HSA-5662615;R-HSA-5662610;R-HSA-5662608;R-HSA-5662622;R-HSA-5662621;R-HS
R-HSA-5662614;R-HSA-5662615;R-HSA-5662610;R-HSA-5662608;R-HSA-5662622;R-HSA-5662621;R-HS
R-HSA-164834;R-HSA-6799191;R-HSA-164832;R-HSA-163217;R-HSA-6799202;R-HSA-6799199;R-HSA-1
R-HSA-6799191;R-HSA-163217;R-HSA-6799202;R-HSA-6799199;R-HSA-164651;R-HSA-6799197;R-HSA-
R-HSA-430183;R-HSA-202241;R-HSA-430180;R-HSA-202407;R-HSA-202375;R-HSA-202245;R-HSA-2022
R-HSA-205115;R-HSA-205039;R-HSA-204958;R-HSA-205132
R-HSA-1855213;R-HSA-1855221;R-HSA-1855205;R-HSA-1855153
R-HSA-6786854;R-HSA-6785722
R-HSA-2022399;R-HSA-2022396;R-HSA-2022378;R-HSA-2022379;R-HSA-2022393;R-HSA-2022368
R-HSA-6799191;R-HSA-6799202;R-HSA-6799199;R-HSA-6799197;R-HSA-6799196;R-HSA-6799179
R-HSA-427383;R-HSA-5250930;R-HSA-5250947;R-HSA-5250938;R-HSA-427404;R-HSA-427336
R-HSA-9013109;R-HSA-9013111;R-HSA-9013110
R-HSA-164834;R-HSA-164832;R-HSA-6799202;R-HSA-9012268;R-HSA-164651;R-HSA-164840;R-HSA-67
R-HSA-205115;R-HSA-205039;R-HSA-204958;R-HSA-205132
R-HSA-3777129;R-HSA-3782637;R-HSA-3782655;R-HSA-9679787;R-HSA-3769447
R-HSA-73718;R-HSA-73769;R-HSA-73736;R-HSA-73739;R-HSA-74986;R-HSA-73758
R-HSA-349637;R-HSA-8933258;R-HSA-174657;R-HSA-349638;R-HSA-8865667;R-HSA-6784738;R-HSA-2
R-HSA-9626962;R-HSA-9620515;R-HSA-9626928;R-HSA-9626945;R-HSA-9620532

R-HSA-8939066;R-HSA-8938251;R-HSA-8938382;R-HSA-8939054;R-HSA-8938228;R-HSA-8938389;R-HS
R-HSA-428287;R-HSA-428296
R-HSA-9023592;R-HSA-9005995;R-HSA-9022314;R-HSA-9022625;R-HSA-9021888;R-HSA-9018594;R-HS
R-HSA-9022728;R-HSA-9022734;R-HSA-9021953;R-HSA-9021952;R-HSA-9021954;R-HSA-9017988;R-HS
R-HSA-8938382;R-HSA-8938371
R-HSA-9005561;R-HSA-9022462;R-HSA-9005585;R-HSA-9022465;R-HSA-9023461
R-HSA-9005561;R-HSA-9022462;R-HSA-9005585;R-HSA-9022465;R-HSA-9023461
R-HSA-9005561;R-HSA-9022462;R-HSA-9005585;R-HSA-9022465;R-HSA-9023461
R-HSA-9005561;R-HSA-9022462;R-HSA-9005585;R-HSA-9022465;R-HSA-9023461
R-HSA-8936584;R-HSA-8937097;R-HSA-8931981;R-HSA-8938382;R-HSA-8936068;R-HSA-8938887;R-HS
R-HSA-9005561
R-HSA-975902;R-HSA-975903;R-HSA-975916
R-HSA-9005585
R-HSA-200952;R-HSA-200908
R-HSA-9022462
R-HSA-549385;R-HSA-1368087;R-HSA-1368081;R-HSA-400256;R-HSA-5663174;R-HSA-1368069;R-HSA-
R-HSA-400342;R-HSA-1368069;R-HSA-9707504;R-HSA-1368065;R-HSA-9707586;R-HSA-9707516;R-HSA
R-HSA-182263;R-HSA-182279;R-HSA-200952;R-HSA-182286;R-HSA-200908
R-HSA-8849748;R-HSA-8847635;R-HSA-8847883;R-HSA-6811433;R-HSA-8847880;R-HSA-8847544
R-HSA-8939833;R-HSA-8939963;R-HSA-8940001;R-HSA-8939667;R-HSA-8939829;R-HSA-8939670;R-HS
R-HSA-927789
R-HSA-72673;R-HSA-72676
R-HSA-192704;R-HSA-192841
R-HSA-1799326;R-HSA-1799330;R-HSA-1799329;R-HSA-1799335;R-HSA-1799332
R-HSA-72672;R-HSA-72619;R-HSA-72671
R-HSA-156808;R-HSA-157849
R-HSA-72697;R-HSA-72621
R-HSA-927836;R-HSA-927789;R-HSA-927832;R-HSA-927813;R-HSA-927889
R-HSA-156915;R-HSA-156912;R-HSA-156923;R-HSA-156907
R-HSA-927836;R-HSA-927832;R-HSA-927813;R-HSA-927889
R-HSA-156823;R-HSA-156826

R-HSA-141671;R-HSA-141691;R-HSA-141673
R-HSA-72673;R-HSA-72672;R-HSA-72691;R-HSA-72676;R-HSA-156823;R-HSA-156826;R-HSA-72697;R-H
R-HSA-72673;R-HSA-72672;R-HSA-72691;R-HSA-72676;R-HSA-72697;R-HSA-72619;R-HSA-156808;R-H
R-HSA-156915;R-HSA-156912;R-HSA-156923;R-HSA-156907
R-HSA-6791223;R-HSA-6791221;R-HSA-6791218
R-HSA-9729232
R-HSA-72691
R-HSA-156808;R-HSA-157849
R-HSA-2408529;R-HSA-5333615
R-HSA-8944265;R-HSA-2002460;R-HSA-8944262;R-HSA-2426676;R-HSA-2002440;R-HSA-1458433;R-HS
R-HSA-1564112;R-HSA-2168038;R-HSA-1454781;R-HSA-1592310;R-HSA-1602454;R-HSA-1454822;R-HS
R-HSA-8944216;R-HSA-8944265;R-HSA-8944240;R-HSA-8944262;R-HSA-8944214;R-HSA-8944247
R-HSA-2395302;R-HSA-2395340;R-HSA-1474266;R-HSA-2396234;R-HSA-2213201;R-HSA-2559639;R-HS
R-HSA-2484965;R-HSA-4085087;R-HSA-114563;R-HSA-210304;R-HSA-4539779;R-HSA-2467436;R-HSA-
R-HSA-2328129;R-HSA-2426355;R-HSA-2465890;R-HSA-2327738;R-HSA-2731075
R-HSA-1564112;R-HSA-2168038;R-HSA-1454822;R-HSA-2485111;R-HSA-1474213;R-HSA-1454757;R-HS
R-HSA-3221843;R-HSA-2247513;R-HSA-2197770;R-HSA-2203516
R-HSA-8948232;R-HSA-8944265;R-HSA-2002460;R-HSA-8948234;R-HSA-2395302;R-HSA-8948224;R-HS
R-HSA-8948232;R-HSA-8944265;R-HSA-2002460;R-HSA-8948234;R-HSA-8948224;R-HSA-8948226;R-HS
R-HSA-8952289
R-HSA-4086204;R-HSA-2396370;R-HSA-2327909;R-HSA-2424243;R-HSA-2424252;R-HSA-2396124;R-HS
R-HSA-381543;R-HSA-381537;R-HSA-8952289;R-HSA-381518;R-HSA-381545
R-HSA-525833;R-HSA-198756;R-HSA-375151
R-HSA-525833;R-HSA-375151
R-HSA-2559639;R-HSA-2395302;R-HSA-2395223;R-HSA-2243926;R-HSA-2395314;R-HSA-2395340;R-HS
R-HSA-4084507;R-HSA-2426530;R-HSA-4084505;R-HSA-3907292;R-HSA-4084501;R-HSA-216051;R-HSA
R-HSA-2396234
R-HSA-1011598;R-HSA-114563;R-HSA-210304;R-HSA-425861;R-HSA-8858498;R-HSA-392195;R-HSA-41
R-HSA-5690740;R-HSA-5690702;R-HSA-5690701
R-HSA-1675961;R-HSA-1676185;R-HSA-1676024;R-HSA-1675994;R-HSA-1675836;R-HSA-1676177;R-HS
R-HSA-1306953

	R-HSA-167601;R-HSA-167537;R-HSA-182198;R-HSA-182263;R-HSA-182279;R-HSA-182186;R-HSA-1821
	R-HSA-6809680;R-HSA-1675939;R-HSA-1676162;R-HSA-6809707;R-HSA-6809720
	R-HSA-114352;R-HSA-508163;R-HSA-9692376;R-HSA-6790025
	R-HSA-8875431;R-HSA-8875558;R-HSA-8851877;R-HSA-8875443;R-HSA-8851859;R-HSA-8875490;R-HS
	R-HSA-1358798;R-HSA-1358792
	R-HSA-8953461
	R-HSA-508247;R-HSA-453111;R-HSA-912527;R-HSA-912734;R-HSA-913374;R-HSA-453104;R-HSA-9134
	R-HSA-8848005;R-HSA-8847995
	R-HSA-6785636;R-HSA-6785648
	R-HSA-167601;R-HSA-167537;R-HSA-182198;R-HSA-182263;R-HSA-182279;R-HSA-182186;R-HSA-1821
	R-HSA-1306979;R-HSA-1250346;R-HSA-1250462;R-HSA-1250189
	R-HSA-1676203;R-HSA-1675994;R-HSA-1675949;R-HSA-1675836;R-HSA-1676177;R-HSA-1676164;R-HS
	R-HSA-8949178;R-HSA-8953461
	R-HSA-382052;R-HSA-382054;R-HSA-382058
	R-HSA-178215;R-HSA-2167924;R-HSA-2187382;R-HSA-178218;R-HSA-2187401;R-HSA-2187368;R-HSA-
	R-HSA-3311014
	R-HSA-3315483
	R-HSA-3311014
;RPS5;RPL	R-HSA-927789
;RPS5;RPL	R-HSA-72673;R-HSA-72676
;RPS5;RPL	R-HSA-192704;R-HSA-192841
;RPS5;RPL	R-HSA-1799326;R-HSA-1799330;R-HSA-1799329;R-HSA-1799335;R-HSA-1799332
;RPS5;RPL	R-HSA-72672;R-HSA-72619;R-HSA-72671
;RPS5;RPL	R-HSA-156832;R-HSA-156823;R-HSA-156826
	R-HSA-156808;R-HSA-157849
	R-HSA-72697;R-HSA-72621
21;RPS8;R	R-HSA-156915;R-HSA-156912;R-HSA-156913;R-HSA-156923;R-HSA-156907;R-HSA-156910;R-HSA-1569
;RPS5;RPL	R-HSA-6791223;R-HSA-6791222;R-HSA-6791221;R-HSA-6791218;R-HSA-6791228;R-HSA-6791227
21;RPS8;RI	R-HSA-927836;R-HSA-927789;R-HSA-927832;R-HSA-927813;R-HSA-927889
;RPS5;RPL	R-HSA-156915;R-HSA-156912;R-HSA-156923;R-HSA-156907
21;RPS8;RI	R-HSA-927836;R-HSA-927832;R-HSA-927813;R-HSA-927889

	R-HSA-9729232;R-HSA-9727944
;RPS5;RPI	R-HSA-72673;R-HSA-72672;R-HSA-156832;R-HSA-72676;R-HSA-72619;R-HSA-156808;R-HSA-72621;R-I
;RPS5;RPI	R-HSA-141671;R-HSA-141691;R-HSA-141673
;RPS5;RPI	R-HSA-72673;R-HSA-72672;R-HSA-72691;R-HSA-72676;R-HSA-72697;R-HSA-72619;R-HSA-156808;R-H
;RPS5;RPI	R-HSA-6791223;R-HSA-6791222;R-HSA-6791221;R-HSA-6791218;R-HSA-6791228;R-HSA-6791227;R-HS
13;RPS12;I	R-HSA-6791223;R-HSA-6791222;R-HSA-6791221;R-HSA-6791218;R-HSA-6791228;R-HSA-6791227;R-HS
;RPS5;RPI	R-HSA-9633008;R-HSA-9635892;R-HSA-9633742;R-HSA-9634669;R-HSA-1791173;R-HSA-9633005
;;RPL13A;F	R-HSA-927789
;;RPL13A;F	R-HSA-72673;R-HSA-72676
;;RPL13A;F	R-HSA-192704;R-HSA-192841
;;RPL13A;F	R-HSA-156832;R-HSA-156823;R-HSA-156826
;;RPL13A;F	R-HSA-1799326;R-HSA-1799330;R-HSA-1799329;R-HSA-1799335;R-HSA-1799332
;;RPL13A;F	R-HSA-72672;R-HSA-72619;R-HSA-72671
;;RPL13A;F	R-HSA-927836;R-HSA-927789;R-HSA-927832;R-HSA-927830;R-HSA-927813;R-HSA-927889
;;RPL13A;F	R-HSA-927836;R-HSA-927832;R-HSA-927830;R-HSA-927813;R-HSA-927889
	R-HSA-156808;R-HSA-157849
	R-HSA-72697;R-HSA-72621
S5;RPS6;F	R-HSA-156915;R-HSA-156912;R-HSA-156913;R-HSA-156923;R-HSA-156907;R-HSA-156910;R-HSA-1569
;;RPL13A;F	R-HSA-6791223;R-HSA-6791222;R-HSA-6791221;R-HSA-6791218;R-HSA-6791228;R-HSA-6791227
;;RPL13A;F	R-HSA-156915;R-HSA-156912;R-HSA-156923;R-HSA-156907
	R-HSA-9729232;R-HSA-9727944
;;RPL13A;F	R-HSA-72673;R-HSA-72672;R-HSA-156832;R-HSA-72676;R-HSA-72619;R-HSA-156808;R-HSA-72621;R-I
;;RPL13A;F	R-HSA-141671;R-HSA-141691;R-HSA-141673
;;RPL13A;F	R-HSA-72673;R-HSA-72672;R-HSA-72691;R-HSA-72676;R-HSA-72697;R-HSA-72619;R-HSA-156808;R-H
;;RPL13A;F	R-HSA-6791223;R-HSA-6791222;R-HSA-6791221;R-HSA-6791218;R-HSA-6791228;R-HSA-6791227;R-HS
S5;RPS6;F	R-HSA-5389842;R-HSA-5419281;R-HSA-1799326;R-HSA-5368279;R-HSA-156808;R-HSA-5389848;R-HS/
'S5;RPS6;F	R-HSA-6791223;R-HSA-6791222;R-HSA-6791221;R-HSA-6791218;R-HSA-6791228;R-HSA-6791227;R-HS
'TPN1;YES	R-HSA-450054;R-HSA-452100;R-HSA-9732753;R-HSA-452097;R-HSA-8950415;R-HSA-450063;R-HSA-11
NFKB1;BS	R-HSA-741386;R-HSA-688136;R-HSA-688137;R-HSA-936960;R-HSA-936991;R-HSA-936986;R-HSA-2037
	R-HSA-997326;R-HSA-9730967;R-HSA-9689680;R-HSA-9732753;R-HSA-9734547;R-HSA-873924;R-HSA-
	R-HSA-9710963;R-HSA-909725;R-HSA-997311;R-HSA-997309;R-HSA-909726;R-HSA-913529;R-HSA-909

	R-HSA-450054;R-HSA-452100;R-HSA-452097;R-HSA-8950415;R-HSA-450063;R-HSA-1112602;R-HSA-45
	R-HSA-8950415;R-HSA-9617941;R-HSA-8984963;R-HSA-8984964;R-HSA-8950405;R-HSA-8950533;R-HS
	R-HSA-8950344;R-HSA-8950218;R-HSA-8950732;R-HSA-8950415;R-HSA-9617941;R-HSA-8950721;R-HS
	R-HSA-8950344;R-HSA-8950218;R-HSA-8950732;R-HSA-8950415;R-HSA-9617941;R-HSA-8950721;R-HS
	R-HSA-8877414;R-HSA-8939082;R-HSA-8877348;R-HSA-8877345;R-HSA-8877391;R-HSA-8877421;R-HS
	R-HSA-4647594
	R-HSA-8868071;R-HSA-8866279;R-HSA-8868230;R-HSA-8866277;R-HSA-8863471;R-HSA-8867756;R-HS
	R-HSA-3790137;R-HSA-182594;R-HSA-3857305;R-HSA-3209109;R-HSA-3857308;R-HSA-6804879;R-HSA
	R-HSA-3928619;R-HSA-3928623;R-HSA-4093336;R-HSA-3928620;R-HSA-3928627;R-HSA-3928595;R-HS
	R-HSA-211219;R-HSA-211186;R-HSA-211239;R-HSA-211190;R-HSA-211247
	R-HSA-5676598;R-HSA-5668534;R-HSA-5676596;R-HSA-5668404;R-HSA-5676597;R-HSA-5676595;R-HS
	R-HSA-211715;R-HSA-351877;R-HSA-201603;R-HSA-351876;R-HSA-9710101;R-HSA-114306;R-HSA-964
	R-HSA-8936584;R-HSA-8937097;R-HSA-8931981;R-HSA-8938382;R-HSA-8938887;R-HSA-8877192;R-HS
IST1H4C;F	R-HSA-5668947;R-HSA-5623632;R-HSA-5671763;R-HSA-5666129;R-HSA-9018766;R-HSA-9018754;R-HS
	R-HSA-211715;R-HSA-351877;R-HSA-201603;R-HSA-351876;R-HSA-114306;R-HSA-211716;R-HSA-2016
	R-HSA-202307;R-HSA-202241;R-HSA-202375;R-HSA-202245;R-HSA-202500;R-HSA-202248;R-HSA-2025
PL26;RPL2	R-HSA-927789
PL26;RPL2	R-HSA-72673;R-HSA-72676
PL26;RPL2	R-HSA-192704;R-HSA-192841
L27;RPL26	R-HSA-1799326;R-HSA-1799330;R-HSA-1799329;R-HSA-1799335;R-HSA-1799332
PL26;RPL2	R-HSA-72672;R-HSA-72619;R-HSA-72671
PL26;RPL2	R-HSA-156832;R-HSA-156823;R-HSA-156826
	R-HSA-156808;R-HSA-157849
	R-HSA-72697;R-HSA-72621
PL26;RPL2	R-HSA-6791223;R-HSA-6791222;R-HSA-6791221;R-HSA-6791218;R-HSA-6791228;R-HSA-6791227
PL26;RPL2	R-HSA-927836;R-HSA-927789;R-HSA-927832;R-HSA-927813;R-HSA-927889
PL26;RPL2	R-HSA-156915;R-HSA-156912;R-HSA-156923;R-HSA-156907
PL26;RPL2	R-HSA-927836;R-HSA-927832;R-HSA-927813;R-HSA-927889
PL27;RPL2	R-HSA-156915;R-HSA-156912;R-HSA-156913;R-HSA-156923;R-HSA-156907;R-HSA-156910
PL26;RPL2	R-HSA-72673;R-HSA-72672;R-HSA-156832;R-HSA-72676;R-HSA-72619;R-HSA-156808;R-HSA-72621;R-H
PL26;RPL2	R-HSA-141671;R-HSA-141691;R-HSA-141673

PL26;RPL2!	R-HSA-72673;R-HSA-72672;R-HSA-72691;R-HSA-72676;R-HSA-72697;R-HSA-72619;R-HSA-156808;R-H
PL26;RPL2!	R-HSA-6791223;R-HSA-6791222;R-HSA-6791221;R-HSA-6791218;R-HSA-6791228;R-HSA-6791227;R-HS
	R-HSA-9729232
	R-HSA-72691
	R-HSA-156808;R-HSA-157849
	R-HSA-9654752;R-HSA-9635927;R-HSA-9650710;R-HSA-1791118;R-HSA-9653894;R-HSA-9654774;R-HS
	R-HSA-9024361;R-HSA-9618407;R-HSA-9618405;R-HSA-9618394;R-HSA-9618392;R-HSA-9024386
	R-HSA-8950344;R-HSA-8950218;R-HSA-8950732;R-HSA-8950415;R-HSA-9617941;R-HSA-8950721;R-HS
	R-HSA-8950344;R-HSA-8950218;R-HSA-8950732;R-HSA-8950415;R-HSA-9617941;R-HSA-8950721;R-HS
	R-HSA-9024361;R-HSA-9618407;R-HSA-9618405;R-HSA-9618394;R-HSA-9618392;R-HSA-9024386
	R-HSA-8950415;R-HSA-9617941;R-HSA-8984963;R-HSA-8950533;R-HSA-8984964;R-HSA-8950681;R-HS
	R-HSA-3465429;R-HSA-5675456
	R-HSA-6785895;R-HSA-8950415;R-HSA-9617941;R-HSA-8984963;R-HSA-8987266;R-HSA-8848395;R-HS
	R-HSA-1791150
	R-HSA-381158;R-HSA-1791150
	R-HSA-9604480;R-HSA-168140
	R-HSA-3371360;R-HSA-141159;R-HSA-141156;R-HSA-3465459;R-HSA-73945;R-HSA-3465448;R-HSA-33
	R-HSA-9770132;R-HSA-9770119;R-HSA-9770129;R-HSA-9770145;R-HSA-9770131;R-HSA-9770141;R-HS
	R-HSA-5210959;R-HSA-5210943;R-HSA-5210892;R-HSA-5210918;R-HSA-5210932;R-HSA-5210912
	R-HSA-9617217;R-HSA-9617194;R-HSA-9618584;R-HSA-9622377;R-HSA-9617209;R-HSA-9634445
	R-HSA-9604480;R-HSA-168140
	R-HSA-9770132;R-HSA-9770129;R-HSA-9770131;R-HSA-75079;R-HSA-9770141;R-HSA-75081;R-HSA-97
	R-HSA-9725357
	R-HSA-178178;R-HSA-2179291;R-HSA-2179293;R-HSA-178189
	R-HSA-3371360;R-HSA-141159;R-HSA-141156;R-HSA-3465459;R-HSA-73945;R-HSA-3465448;R-HSA-33
	R-HSA-927789
	R-HSA-72673;R-HSA-72676
	R-HSA-192704;R-HSA-192841
	R-HSA-1799326;R-HSA-1799330;R-HSA-1799329;R-HSA-1799335;R-HSA-1799332
	R-HSA-72672;R-HSA-72619;R-HSA-72671
	R-HSA-156808;R-HSA-157849

R-HSA-72697;R-HSA-72621
R-HSA-927836;R-HSA-927789;R-HSA-927832;R-HSA-927813;R-HSA-927889
R-HSA-156915;R-HSA-156912;R-HSA-156923;R-HSA-156907
R-HSA-927836;R-HSA-927832;R-HSA-927813;R-HSA-927889
R-HSA-156823;R-HSA-156826
R-HSA-141671;R-HSA-141691;R-HSA-141673
R-HSA-72673;R-HSA-72672;R-HSA-72691;R-HSA-72676;R-HSA-156823;R-HSA-156826;R-HSA-72697;R-I
R-HSA-72673;R-HSA-72672;R-HSA-72691;R-HSA-72676;R-HSA-72697;R-HSA-72619;R-HSA-156808;R-H
R-HSA-156915;R-HSA-156912;R-HSA-156923;R-HSA-156907
R-HSA-6791223;R-HSA-6791221;R-HSA-6791218
R-HSA-5389842;R-HSA-5419281;R-HSA-1799326;R-HSA-5368279;R-HSA-156808;R-HSA-5389848;R-HSA
R-HSA-9729232
R-HSA-72691
R-HSA-156808;R-HSA-157849
R-HSA-5159245;R-HSA-4754187;R-HSA-5244692;R-HSA-3321975;R-HSA-3697008;R-HSA-3777129;R-HS
R-HSA-5159245;R-HSA-4754187;R-HSA-5244692;R-HSA-3321975;R-HSA-3697008;R-HSA-3777129;R-HS
R-HSA-8939066;R-HSA-8938251;R-HSA-8938382;R-HSA-8939054;R-HSA-8938228;R-HSA-8938231
R-HSA-9023592;R-HSA-9005995;R-HSA-9022314;R-HSA-9022625;R-HSA-9021888;R-HSA-9018594;R-HS
R-HSA-428287;R-HSA-428296
R-HSA-2032781;R-HSA-2032794;R-HSA-1989766;R-HSA-2064932;R-HSA-2032800
R-HSA-5637686;R-HSA-5651654;R-HSA-8865237;R-HSA-5159245;R-HSA-5244692;R-HSA-5638141;R-HS
R-HSA-5623525;R-HSA-5623524;R-HSA-5623508;R-HSA-5623527;R-HSA-5620918;R-HSA-5623521;R-HS
R-HSA-9022462
R-HSA-5229194;R-HSA-5195402;R-HSA-5221130;R-HSA-2993898;R-HSA-5223304;R-HSA-2990880;R-HS
R-HSA-5671702;R-HSA-421831;R-HSA-5229132;R-HSA-432706;R-HSA-8874979;R-HSA-432707;R-HSA-4
R-HSA-392295;R-HSA-8964271;R-HSA-392300;R-HSA-8964614;R-HSA-8964634;R-HSA-8964604;R-HSA-
R-HSA-8938217;R-HSA-8878054;R-HSA-8878050;R-HSA-8937989
R-HSA-9005585
R-HSA-174690;R-HSA-174739;R-HSA-174757
R-HSA-400342;R-HSA-1368069;R-HSA-1368065;R-HSA-9707586;R-HSA-549475
R-HSA-392295;R-HSA-392300;R-HSA-9603279

	R-HSA-200952;R-HSA-200908
	R-HSA-5660980;R-HSA-5660660;R-HSA-5624486;R-HSA-5624473;R-HSA-5660666
	R-HSA-9018766;R-HSA-8981637;R-HSA-9018758;R-HSA-9013145;R-HSA-9014296;R-HSA-9013144;R-HS
	R-HSA-8939066;R-HSA-8938251;R-HSA-8939054;R-HSA-8938382;R-HSA-8938228;R-HSA-8938389;R-HS
	R-HSA-382096;R-HSA-376419
/D3	R-HSA-8865605;R-HSA-4754187;R-HSA-5159245;R-HSA-5244692;R-HSA-3321975;R-HSA-3301237;R-HS
/D3	R-HSA-8865605;R-HSA-4754187;R-HSA-5159245;R-HSA-5244692;R-HSA-3321975;R-HSA-3301237;R-HS
	R-HSA-9615570;R-HSA-9022728;R-HSA-9615571;R-HSA-9022314;R-HSA-9022734;R-HSA-9017921;R-HS
	R-HSA-8936584;R-HSA-8937097;R-HSA-8931981;R-HSA-8938382;R-HSA-8936068;R-HSA-8938887;R-HS
	R-HSA-8878054;R-HSA-8938217;R-HSA-8878050;R-HSA-8937989;R-HSA-8878056
	R-HSA-8938382;R-HSA-8938371
	R-HSA-1368140;R-HSA-2466367;R-HSA-1592249;R-HSA-1592250;R-HSA-1592251;R-HSA-2466369;R-HS
	R-HSA-6809254;R-HSA-1675994;R-HSA-199456;R-HSA-6809793;R-HSA-1676145;R-HSA-1676048;R-HSA
	R-HSA-8878257;R-HSA-8878266;R-HSA-9008326;R-HSA-8986294
	R-HSA-428287;R-HSA-428296
	R-HSA-1368087;R-HSA-1368081;R-HSA-400256;R-HSA-400267;R-HSA-5663174;R-HSA-1368069;R-HSA-
'25;SLC4A	R-HSA-9018766;R-HSA-9018754;R-HSA-8981637;R-HSA-9693214;R-HSA-9018756;R-HSA-9018758;R-HS
	R-HSA-212356;R-HSA-350058
	R-HSA-8951926;R-HSA-8951910
'1R12B;DC	R-HSA-2029469;R-HSA-2029466;R-HSA-5668947;R-HSA-9018766;R-HSA-5666129;R-HSA-5666001;R-HS
	R-HSA-8986937;R-HSA-8986939;R-HSA-9022314;R-HSA-9021183;R-HSA-9021182;R-HSA-9006992;R-HS
'1R12B;DC	R-HSA-2029469;R-HSA-2029466;R-HSA-5668947;R-HSA-9018766;R-HSA-5666129;R-HSA-5666001;R-HS
	R-HSA-400342;R-HSA-1368069;R-HSA-5663271;R-HSA-1368119;R-HSA-9708423;R-HSA-1368065;R-HSA

Supplemental Digital Content, Table S3

Transplan	Day	p_val	avg_log2FC	pct.1	pct.2	p_val_adj	Gene
Successfu	0	1.96089868754244e-139	2.06021227746969	0.796	0.084	6.57646201827985e-135	REG1B
Successfu	0	2.71380199918341e-75	1.0865376378369	0.93	0.538	9.10154914486132e-71	APOA4
Successfu	0	1.25105247138826e-68	1.11069250320427	0.986	0.814	4.19577977854193e-64	FABP6
Successfu	0	3.08533700684619e-63	1.67979139314	0.806	0.376	1.03476032535607e-58	FKBP5
Successfu	0	1.98151723489368e-61	1.1025040006961	0.968	0.759	6.64561250238641e-57	PHGR1
Successfu	0	1.72625702781116e-50	0.771976865536466	0.94	0.689	5.78952081987308e-46	REG1A
Successfu	0	4.19412719337242e-47	1.81127746618401	0.683	0.254	1.40662637811324e-42	CXCR4
Successfu	0	4.15015495151397e-46	1.72153307742777	0.771	0.367	1.39187896763876e-41	HLA-DRA
Successfu	0	8.59448368235166e-46	1.41360703374685	0.94	0.848	2.8824179373871e-41	TMSB4X
Successfu	0	1.96399583592753e-45	0.998990122725307	0.662	0.262	6.58684923453374e-41	GUCA2A
Successfu	0	2.23814863898867e-44	1.89521286331467	0.937	0.817	7.50630290544019e-40	RPS27
Successfu	0	2.87317362855904e-43	0.738062171082544	0.817	0.518	9.63604971546131e-39	APOC3
Successfu	0	2.22695961066873e-42	1.8100108294023	0.845	0.669	7.4687771422608e-38	TXNIP
Successfu	0	1.19357328542631e-41	1.52015452625984	0.796	0.434	4.00300608466277e-37	MGAT5
Successfu	0	1.40740583200682e-38	1.22590562152277	0.796	0.494	4.72015767938447e-34	SMAP2
Successfu	0	2.03137688509017e-38	1.35359691449489	0.577	0.214	6.81283179721541e-34	CD52
Successfu	0	4.97342640006297e-38	0.761422515455098	0.504	0.158	1.66798774605312e-33	CYP3A4
Successfu	0	6.6034908825798e-37	1.2167618769049	0.507	0.139	2.21467877219961e-32	MS4A1
Successfu	0	2.54531312112838e-36	1.07168018187814	0.944	0.896	8.53647114564036e-32	FTH1
Successfu	0	4.65595848669596e-36	1.02162117865105	0.451	0.12	1.56151535726809e-31	LAPTM5
Successfu	0	2.09310330102148e-35	1.52686328629221	0.908	0.836	7.01984985096584e-31	RPL11
Successfu	0	2.69125419721146e-35	1.43757882645746	0.574	0.224	9.02592832660779e-31	HLA-DPB1
Successfu	0	8.33278891648334e-35	1.05015239771384	0.651	0.252	2.79465074681018e-30	CD83
Successfu	0	1.18183422018244e-34	0.903233161430399	0.69	0.365	3.96363560764787e-30	KRT19
Successfu	0	1.22077544064107e-34	1.58796578661981	0.849	0.747	4.09423667282202e-30	RPS29
Successfu	0	3.3302631099353e-34	0.963423339455192	0.651	0.298	1.1169036418101e-29	PDE3A
Successfu	0	2.3266252409717e-33	1.03906141434404	0.835	0.445	7.80303573317088e-29	CD69
Successfu	0	2.38704124365032e-33	1.43310413950767	0.919	0.856	8.00565892295445e-29	RPS18
Successfu	0	1.0549690852877e-32	1.28549795289987	0.905	0.838	3.5381553182379e-28	RPL12

Successfu	0	2.0071661827692e-32	1.32984836881695	0.951	0.881	6.73163394377133e-28	RPS2
Successfu	0	4.41273391114916e-32	1.58814010215334	0.93	0.851	1.47994269912121e-27	RPS12
Successfu	0	6.36497911842377e-32	1.48643529543384	0.919	0.814	2.13468669673697e-27	RPL30
Successfu	0	1.72188158401833e-31	1.52854406419314	0.877	0.736	5.77484645648066e-27	BTG1
Successfu	0	1.81994608625686e-31	1.12348817442864	0.592	0.247	6.10373518408824e-27	HLA-DQB1
Successfu	0	2.32011827332707e-31	0.69578610208830€	0.81	0.582	7.78121266508432e-27	FABP1
Successfu	0	2.6390581100243e-31	1.24752405554541	0.88	0.798	8.85087308939951e-27	RPL3
Successfu	0	1.58294217359237e-30	1.16856768575984	0.838	0.739	5.3088714617941e-26	RPL22
Successfu	0	2.67499068227725e-30	0.99136753428599‡	0.93	0.833	8.97138375022146e-26	ACTB
Successfu	0	6.7146685155849e-30	1.3118239630996	0.919	0.852	2.25196552675686e-25	RPL28
Successfu	0	9.43636063295829e-30	1.66143647506759	0.838	0.7	3.16476662908155e-25	RPL21
Successfu	0	1.35175671910902e-29	1.21119931461204	0.937	0.849	4.53352168454784e-25	RPS28
Successfu	0	1.72413705330802e-29	1.11026851244552	0.898	0.838	5.78241084938442e-25	RPL29
Successfu	0	2.21888602363453e-29	1.36004453456486	0.845	0.722	7.44169994606548e-25	RPS7
Successfu	0	4.13280143028355e-29	1.37207570607362	0.887	0.845	1.3860589436885e-24	RPL34
Successfu	0	6.83886174407836e-29	1.18474568230972	0.919	0.827	2.293617451729e-24	RPS4X
Successfu	0	7.11757782422869e-29	1.37465671214891	0.884	0.794	2.38709325068982e-24	RPS3A
Successfu	0	2.02542254881488e-28	1.49409842222268	0.785	0.664	6.79286214421535e-24	RPS21
Successfu	0	3.10493505169963e-28	1.27184257014981	0.863	0.768	1.04133311763902e-23	RPS13
Successfu	0	4.48816638186876e-28	1.32875515689753	0.88	0.773	1.50524124115115e-23	RPS3
Successfu	0	4.88682152455349e-28	1.14518841905415	0.486	0.172	1.63894220290475e-23	HLA-DQA1
Successfu	0	5.21368524390873e-28	0.48716123810824€	0.609	0.241	1.74856575710211e-23	BANK1
Successfu	0	6.86386563922506e-28	1.13565112263076	0.961	0.899	2.3020032580833e-23	RPL10
Successfu	0	9.80459273604024e-28	1.38356467977254	0.891	0.79	3.28826431181317e-23	RPS27A
Successfu	0	1.17112658596876e-27	1.12661055427294	0.866	0.804	3.92772434402204e-23	RPL8
Successfu	0	1.41636947578569e-27	0.86831308289900‡	0.891	0.663	4.75021994789004e-23	MTRNR2L8
Successfu	0	2.26411643298482e-27	1.06449325487743	0.982	0.952	7.59339369294449e-23	EEF1A1
Successfu	0	2.28783570842723e-27	0.73279348723146‡	0.556	0.2	7.67294339892326e-23	AFF3
Successfu	0	6.25321916463104e-27	1.07754689703803	0.961	0.885	2.09720464343396e-22	RPL41
Successfu	0	7.82884078768615e-27	1.33750294188941	0.915	0.865	2.62563662337418e-22	RPL13
Successfu	0	8.31919722696775e-27	1.26008136828532	0.859	0.816	2.79009236598044e-22	RPL37

Successfu	0	1.40224397340049e-26	1.22138345986763	0.933	0.847	4.70284583799056e-22	RPS14
Successfu	0	2.35763815295197e-26	1.34442992461197	0.863	0.79	7.90704683737031e-22	RPL18A
Successfu	0	6.1366271612852e-26	1.15982239208223	0.813	0.759	2.05810201735183e-21	RPL37A
Successfu	0	6.97895736132459e-26	1.35424924835727	0.887	0.808	2.34060271984104e-21	RPS23
Successfu	0	1.00513005509787e-25	1.48646424203483	0.926	0.875	3.37100517878725e-21	RPS8
Successfu	0	1.12366877056263e-25	1.18075821295282	0.82	0.727	3.76856032271295e-21	UBA52
Successfu	0	1.50325171431934e-25	1.39681267376264	0.859	0.797	5.04160559948419e-21	RPL32
Successfu	0	1.88923408078431e-25	1.31004323751522	0.894	0.858	6.33611326013443e-21	RPL13A
Successfu	0	2.17344932099551e-25	0.510501720994422	0.736	0.472	7.28931433275475e-21	SELENOP
Successfu	0	2.49697910678819e-25	0.960796717752492	0.792	0.681	8.37436852834623e-21	PFDN5
Successfu	0	3.92912380741807e-25	1.15824329790725	0.866	0.783	1.31774954253187e-20	RPL27A
Successfu	0	4.66087415378529e-25	0.772479458375279	0.5	0.211	1.56316397369651e-20	HLA-DRB5
Successfu	0	5.06262930944043e-25	1.18357905685802	0.926	0.883	1.69790461780013e-20	RPS19
Successfu	0	6.18737238515289e-25	0.972519271538282	0.451	0.169	2.07512095053258e-20	VPREB3
Successfu	0	8.69387296323082e-25	1.05453157680591	0.923	0.854	2.91575111440835e-20	RPL18
Successfu	0	1.1204408200544e-24	1.43572233813488	0.595	0.348	3.75773442229844e-20	CD37
Successfu	0	1.38028573749809e-24	0.770054292962387	0.408	0.134	4.6292023064211e-20	LTB
Successfu	0	1.4744494319053e-24	1.17095346387537	0.87	0.789	4.94500850472398e-20	RPS16
Successfu	0	1.83603993704034e-24	1.26825195515628	0.803	0.669	6.15771074084591e-20	RPSA
Successfu	0	1.89253355385807e-24	0.586147192752025	0.57	0.285	6.34717903292918e-20	SLC5A1
Successfu	0	1.94202474333083e-24	1.3699224707864	0.831	0.734	6.51316258418293e-20	RPL19
Successfu	0	1.9983057813426e-24	1.16786757238028	0.813	0.712	6.70191792946683e-20	RPL26
Successfu	0	2.10462921843688e-24	0.519692221536854	0.208	0.029	7.05850547279362e-20	LINC01857
Successfu	0	2.15210355877175e-24	1.23802554903325	0.866	0.796	7.2177249154087e-20	RPS25
Successfu	0	2.23262080569516e-24	1.29136229446053	0.806	0.677	7.48776365814041e-20	RPS5
Successfu	0	2.28642948357876e-24	1.02212904580348	0.873	0.809	7.66822720202645e-20	RPL6
Successfu	0	2.36715928800891e-24	0.379009008152537	0.831	0.604	7.93897882012428e-20	ALDOB
Successfu	0	3.19105003166908e-24	1.19828366529504	0.813	0.748	1.07021435962118e-19	RPL35A
Successfu	0	3.80830421942048e-24	1.14960893291481	0.845	0.787	1.27722906910924e-19	RPL36
Successfu	0	4.54309281962487e-24	1.17924629926777	0.88	0.819	1.52366246984579e-19	RPL9
Successfu	0	4.66314484239904e-24	1.18816105494429	0.905	0.844	1.56392551724379e-19	RPS6

Successfu	0	6.15255587607803e-24	0.97778137552290€	0.426	0.151	2.06344418971905e-19	MARCH1
Successfu	0	6.77855438339285e-24	0.91234202995122€	0.831	0.766	2.27339156910229e-19	NACA
Successfu	0	9.24304396319479e-24	1.23895006026497	0.789	0.68	3.09993208437627e-19	RPL10A
Successfu	0	9.37956402204978e-24	1.16987768437043	0.898	0.841	3.14571818171506e-19	RPS15A
Successfu	0	1.06103791900177e-23	0.93464155086329€	0.606	0.288	3.55850897274815e-19	ARHGAP24
Successfu	0	1.36653966869751e-23	1.0194594822753	0.817	0.627	4.58310074087771e-19	CELF2
Successfu	0	1.39209890891637e-23	0.94528619670347€	0.563	0.274	4.66882132072373e-19	CCND3
Successfu	0	1.65331812527727e-23	1.25513229735772	0.532	0.269	5.54489832855491e-19	HLA-DPA1
Successfu	0	1.93952706403128e-23	0.97829181304318€	0.82	0.709	6.50478586734811e-19	RACK1
Successfu	0	2.54285115820332e-23	0.99779694900489€	0.602	0.324	8.5282142143823e-19	HLA-DRB1
Successfu	0	3.07206266186741e-23	0.83537341226580€	0.285	0.07	1.03030837553709e-18	ZBTB16
Successfu	0	3.67964027096214e-23	1.07713281371449	0.845	0.777	1.23407775407528e-18	RPL35
Successfu	0	1.44407269239859e-22	0.77481443188618€	0.405	0.152	4.84313099576639e-18	TNFRSF13C
Successfu	0	1.99423102724964e-22	0.60034996219916€	0.458	0.194	6.68825201918985e-18	CLDN3
Successfu	0	2.25651980290916e-22	0.71786018526237€	0.275	0.069	7.56791611499674e-18	TNFSF9
Successfu	0	2.4654244891017e-22	1.06698378480987	0.588	0.319	8.26854065154929e-18	CEMIP2
Successfu	0	7.54757818026859e-22	1.13306289894299	0.813	0.757	2.53130677009848e-17	FAU
Successfu	0	7.8023885120678e-22	1.03469209067591	0.838	0.79	2.6167650591773e-17	RPS9
Successfu	0	9.98441334294172e-22	1.09084515445458	0.87	0.806	3.34857254695579e-17	RPS15
Successfu	0	1.27354839470697e-21	1.14448074246754	0.933	0.885	4.27122660616823e-17	RPL39
Successfu	0	1.27903178955102e-21	0.34713942645690€	0.634	0.358	4.28961681579621e-17	GDA
Successfu	0	1.80550394696331e-21	0.99078776237112€	0.757	0.604	6.05529913732556e-17	ARID5B
Successfu	0	5.88136969468023e-21	0.93757366114738€	0.634	0.373	1.97249376820185e-16	SNX9
Successfu	0	1.03649255785591e-20	0.74691605718177€	0.754	0.632	3.47618874053715e-16	CIRBP
Successfu	0	1.03798955440976e-20	0.82023640791568€	0.778	0.677	3.48120936757945e-16	EEF1D
Successfu	0	1.75037775828593e-20	0.82202606571339€	0.486	0.217	5.87041692573934e-16	GPR183
Successfu	0	1.76805614931613e-20	0.61678017365940€	0.535	0.283	5.92970671357642e-16	ACSL5
Successfu	0	2.85903966826881e-20	0.78779157394856€	0.915	0.806	9.58864723943994e-16	MTRNR2L12
Successfu	0	3.6765103753411e-20	1.07435020770505	0.824	0.768	1.2330280496819e-15	RPL15
Successfu	0	4.39160419961198e-20	1.05907781890827	0.901	0.86	1.47285621646586e-15	RPLP2
Successfu	0	5.77237433991957e-20	1.06396220840142	0.782	0.725	1.93593890612223e-15	RPS26

Successfu	0	9.76454851329262e-20	0.520204157254872	0.31	0.099	3.27483428038808e-15	PDE9A
Successfu	0	1.06031771085297e-19	1.09484389305493	0.768	0.668	3.55609353865869e-15	RPL7
Successfu	0	1.140830502013e-19	1.10012826700401	0.824	0.77	3.82611733765121e-15	RPL5
Successfu	0	1.31674342020722e-19	0.624436504073631	0.303	0.093	4.41609408269098e-15	FCMR
Successfu	0	1.43100981012652e-19	0.678509517842851	0.507	0.266	4.7993207012023e-15	CLDN4
Successfu	0	2.42486302540273e-19	0.989947079469325	0.81	0.652	8.13250561459567e-15	SSH2
Successfu	0	2.56464984750097e-19	0.743189775084655	0.746	0.693	8.60132265854875e-15	COX7C
Successfu	0	3.29404365272739e-19	1.08618448183335	0.845	0.818	1.10475636025171e-14	RPS11
Successfu	0	6.06649078385927e-19	0.716245362599489	0.366	0.139	2.03457967909072e-14	TRIM22
Successfu	0	7.85289411519491e-19	0.418579560115273	1	1	2.63370362835407e-14	MT-ATP6
Successfu	0	1.16521350022117e-18	0.752066275937257	0.62	0.361	3.90789303704177e-14	REL
Successfu	0	1.24577428840014e-18	0.819984448762407	0.665	0.419	4.1780778084364e-14	PIP4K2A
Successfu	0	1.65402312743584e-18	1.0460324818302	0.754	0.69	5.54726276479433e-14	EEF1B2
Successfu	0	1.80444775029852e-18	0.743926593492164	0.634	0.369	6.05175686495117e-14	BACH2
Successfu	0	2.74274314652255e-18	0.799405431402572	0.863	0.835	9.19861196480732e-14	RPS24
Successfu	0	3.70333897267062e-18	0.72277996498622	0.972	0.948	1.24202582465427e-13	TPT1
Successfu	0	4.1536449102335e-18	1.00992866279806	0.743	0.67	1.39304942999411e-13	RPL38
Successfu	0	4.89107670610756e-18	0.448512917257859	0.676	0.467	1.64036930569435e-13	PCSK5
Successfu	0	5.82794303206229e-18	0.72470546183387	0.387	0.162	1.95457553409305e-13	ABHD15-AS1
Successfu	0	6.5054408626505e-18	0.999184962966929	0.454	0.223	2.18179475651572e-13	SESN3
Successfu	0	7.31342471524104e-18	0.279703657151354	0.169	0.029	2.45277638099754e-13	LCN2
Successfu	0	1.3714935500123e-17	0.560557853956885	0.363	0.148	4.59971506803127e-13	SERPINA1
Successfu	0	1.52850625466474e-17	0.886493571922183	0.842	0.79	5.1263042768946e-13	RPL14
Successfu	0	1.54666878109127e-17	0.57978740104849	0.687	0.491	5.1872177580239e-13	LGALS3
Successfu	0	1.55983131900551e-17	0.443536746213914	0.282	0.088	5.23136227768067e-13	BCL2A1
Successfu	0	3.0147964985103e-17	0.531449997421746	0.627	0.406	1.01110244967038e-12	FABP2
Successfu	0	3.61178467817974e-17	1.35283454228426	0.563	0.344	1.21132034536792e-12	ZNF331
Successfu	0	4.0354551820839e-17	0.824428921427471	0.585	0.384	1.3534109589673e-12	ARHGDIB
Successfu	0	4.16012468374776e-17	0.629665840314988	0.363	0.148	1.39522261643532e-12	IRF8
Successfu	0	4.41713852768783e-17	0.517220425660442	0.278	0.084	1.48141991941594e-12	LY86
Successfu	0	4.80548128267368e-17	0.319333608007262	1	0.999	1.6116623125831e-12	MT-CO3

Successfu	0	6.19370627777962e-17	0.87681032844695	0.493	0.261	2.07724521144173e-12	PRKCE
Successfu	0	6.73501025415503e-17	0.91844301476376	0.518	0.28	2.25878773903851e-12	SLC2A3
Successfu	0	7.29239659987282e-17	0.65530961315221	0.845	0.747	2.44572397166535e-12	TSC22D3
Successfu	0	7.62632784771903e-17	0.58034499730760	0.542	0.312	2.55771783356801e-12	SLC15A1
Successfu	0	9.68267285016731e-17	1.05854303252647	0.792	0.739	3.24737482048911e-12	RPL23A
Successfu	0	1.14236902022682e-16	0.51825462104588	0.342	0.133	3.83127722003671e-12	TRBC2
Successfu	0	1.15206732317206e-16	0.49763491065618	0.623	0.387	3.86380338845446e-12	MYO1E
Successfu	0	1.24012140930719e-16	0.37782487505537	0.486	0.26	4.15911918253447e-12	MUC17
Successfu	0	1.25703864263366e-16	0.53558640534436	0.292	0.096	4.21585619966478e-12	LINC00926
Successfu	0	2.73451136448355e-16	0.67312012059647	0.722	0.602	9.17100421420494e-12	COX4I1
Successfu	0	3.1957627799669e-16	0.45783664150671	0.303	0.104	1.0717949211453e-11	CD24
Successfu	0	5.70044896140208e-16	0.98379212768832	0.627	0.407	1.91181657267503e-11	SAMSN1
Successfu	0	7.31484837346873e-16	0.68630885838320	0.373	0.171	2.45325384749394e-11	LIMD2
Successfu	0	1.18562192858309e-15	0.92876378704370	0.729	0.657	3.97633882408197e-11	RPL27
Successfu	0	1.39296521383912e-15	0.68975551878752	0.412	0.202	4.67172673417363e-11	COTL1
Successfu	0	1.69788116778493e-15	0.48553209566023	0.225	0.066	5.69435386051711e-11	ABCG1
Successfu	0	1.79951082404474e-15	0.80092464658830	0.627	0.477	6.03519940168124e-11	CD79A
Successfu	0	1.84417483352619e-15	0.43634403331511	0.264	0.086	6.18499355668013e-11	HLA-DMB
Successfu	0	2.44136700308916e-15	0.74131078107836	0.817	0.816	8.18785665496044e-11	RPL24
Successfu	0	2.68404250246232e-15	1.15028007888342	0.792	0.797	9.00174174475812e-11	RPS20
Successfu	0	2.77491691022653e-15	0.87784855053532	0.461	0.278	9.30651633351774e-11	RCSD1
Successfu	0	3.31810388017244e-15	0.44223835511396	0.426	0.216	1.11282567933223e-10	PMP22
Successfu	0	3.45426702255086e-15	0.42101688523150	0.504	0.283	1.15849207402311e-10	MUC13
Successfu	0	4.10025941119011e-15	0.91133637473162	0.708	0.643	1.37514500132494e-10	RPL31
Successfu	0	4.25304931203563e-15	0.77112007101519	0.954	0.924	1.42638767827051e-10	RPLP1
Successfu	0	4.94681257986535e-15	0.59198004542077	0.352	0.164	1.65906200303524e-10	PRAP1
Successfu	0	5.66946670770403e-15	0.43946700936932	0.324	0.119	1.90142574442978e-10	RUBCNL
Successfu	0	6.67814917550183e-15	0.38455021627118	0.782	0.683	2.2397176704798e-10	GAPDH
Successfu	0	6.92833044245245e-15	0.29826653223609	1	1	2.3236234637897e-10	MT-CO2
Successfu	0	7.30183713685673e-15	0.72314025293070	0.877	0.796	2.44889013895901e-10	SMCHD1
Successfu	0	8.05294328377519e-15	0.37955206702907	0.665	0.506	2.70079611851252e-10	KRT8

Successfu	0	9.92632947647241e-15	0.58441510270641	0.609	0.446	3.32909237981932e-10	EIF3K
Successfu	0	2.24148583408851e-14	1.0503843963991	0.75	0.691	7.51749519036606e-10	RPL23
Successfu	0	3.32103990717156e-14	0.91789096626968	0.342	0.158	1.1138103640672e-09	LINC01781
Successfu	0	3.5639195130224e-14	0.44097992980724	0.211	0.062	1.19526732627745e-09	SPOCK2
Successfu	0	4.3405578507702e-14	0.71600363596904	0.356	0.166	1.45573629199131e-09	RILPL2
Successfu	0	5.15234964690643e-14	0.55553760379029	0.831	0.759	1.72799502457948e-09	ACTG1
Successfu	0	6.84650563918252e-14	0.54364218047572	0.62	0.457	2.29618106126903e-09	MYL12A
Successfu	0	6.88262082163036e-14	0.87839221594043	0.673	0.555	2.30829337115839e-09	TOMM7
Successfu	0	8.87378710299998e-14	0.63845231078094	0.799	0.765	2.97609071860413e-09	RPLP0
Successfu	0	9.73452759544003e-14	0.75253186867023	0.296	0.118	3.26476586495868e-09	AP000787.1
Successfu	0	1.27879383856677e-13	0.86961388110748	0.863	0.817	4.28881877578522e-09	CD74
Successfu	0	1.38775206243517e-13	0.80256963566408	0.87	0.876	4.65424286699506e-09	PTMA
Successfu	0	1.40028031402928e-13	0.59855021170065	0.715	0.523	4.6962601171914e-09	PTPRC
Successfu	0	1.71115557920638e-13	0.36861907279208	0.335	0.128	5.73887358154237e-09	AP002075.1
Successfu	0	1.71447639276448e-13	0.30489687753976	0.246	0.087	5.7500109260535e-09	GUCA2B
Successfu	0	1.91969202676355e-13	0.59506548962536	0.746	0.638	6.43826311935960e-09	OAZ1
Successfu	0	2.00331522671544e-13	0.65495014210588	0.665	0.536	6.71871860735826e-09	KIAA1551
Successfu	0	2.95346087041346e-13	0.43506091713112	0.405	0.22	9.90531706719265e-09	HLA-DMA
Successfu	0	4.08737976988967e-13	0.52682475452342	0.588	0.423	1.3708254272256e-08	PRR13
Successfu	0	4.12651304906691e-13	0.35419163246880	0.465	0.225	1.38394994639606e-08	RIPOR2
Successfu	0	5.28019491913972e-13	0.78207737588133	0.842	0.836	1.77087177198108e-08	RPL7A
Successfu	0	5.61561238069403e-13	0.41009285104056	0.401	0.208	1.88336408023716e-08	THSD4
Successfu	0	6.23446947346537e-13	0.71955472400941	0.697	0.609	2.09091637201082e-08	BTF3
Successfu	0	7.86818665167536e-13	0.58536230502278	0.775	0.717	2.63883243923888e-08	ZFP36L2
Successfu	0	9.48246109257776e-13	0.30676064820490	0.155	0.036	3.18022780122873e-08	CXCR5
Successfu	0	1.42997105319337e-12	0.67461144089016	0.387	0.211	4.79583691819992e-08	SKI
Successfu	0	1.52088320092274e-12	0.80912479694227	0.528	0.385	5.1007380792547e-08	COMMD6
Successfu	0	2.22557317005154e-12	0.62616470410111	0.479	0.298	7.46412729771884e-08	BCL2L11
Successfu	0	2.61975039549569e-12	0.30123102037652	0.246	0.084	8.78611887641346e-08	CD22
Successfu	0	2.78379197106613e-12	0.54700760976564	0.356	0.179	9.3362815125616e-08	CORO1A
Successfu	0	2.97703241926112e-12	0.51834674121879	0.412	0.225	9.98437132771794e-08	DPEP1

Successfu	0	3.02490165493424e-12	0.43975035734434	0.415	0.227	1.01449151703184e-07	AKNA
Successfu	0	3.71135443050551e-12	0.41037572219425	0.289	0.125	1.24471404890294e-07	TMPRSS2
Successfu	0	3.96635706324966e-12	0.77821042698104	0.592	0.446	1.33023683187267e-07	RPS10
Successfu	0	4.5142990017551e-12	0.53737996891314	0.486	0.308	1.51400559920863e-07	LGALS4
Successfu	0	6.16142945230212e-12	0.64468770839366	0.261	0.105	2.06642020971308e-07	JADE3
Successfu	0	6.35706852366689e-12	0.66242139473148	0.401	0.236	2.1320336414674e-07	PARVB
Successfu	0	6.43676095176914e-12	0.50690864454362	0.736	0.658	2.15876088800433e-07	CFL1
Successfu	0	6.59372153361634e-12	0.54796640412984	0.493	0.312	2.21140232794425e-07	LMO7
Successfu	0	7.43376231969316e-12	0.57572186623004	0.665	0.583	2.49313520677869e-07	HNRNPA1
Successfu	0	8.96475244749341e-12	0.60529778281906	0.391	0.211	3.00659867584034e-07	RASSF6
Successfu	0	1.00031286255566e-11	0.54842997039068	0.715	0.613	3.35484927843916e-07	EEF2
Successfu	0	1.5351498660969e-11	0.54735260791709	0.56	0.396	5.14858562091577e-07	CD53
Successfu	0	1.70656227117505e-11	0.56150824519969	0.366	0.191	5.72346854506689e-07	PARP15
Successfu	0	1.85371326650506e-11	0.33827631658360	0.261	0.1	6.21698355320465e-07	JAM3
Successfu	0	1.8870771753815e-11	0.49164973861237	0.482	0.318	6.32887943079448e-07	ADIRF
Successfu	0	2.15994605704277e-11	0.39347668050342	0.831	0.764	7.24402708611003e-07	HLA-A
Successfu	0	2.95693750580137e-11	0.36865231071578	0.25	0.104	9.91697700695663e-07	TM4SF20
Successfu	0	2.97622363090886e-11	0.46634369223198	0.313	0.146	9.98165881334213e-07	TRAF5
Successfu	0	3.02085576715205e-11	0.45242100274641	0.479	0.324	1.01313460718746e-06	CIB1
Successfu	0	5.46584764250038e-11	0.71884377194404	0.975	0.904	1.83313598234178e-06	FTL
Successfu	0	5.55053956361429e-11	0.38931872978969	0.426	0.246	1.86153995884496e-06	ELF3
Successfu	0	5.55685478746882e-11	0.61451992277559	0.68	0.572	1.86365795862129e-06	PFN1
Successfu	0	6.522477533028e-11	0.36185726280614	0.415	0.249	2.18750851502693e-06	AMN
Successfu	0	7.05434035159189e-11	0.26997011338776	0.169	0.05	2.36588466711689e-06	SHISAL2A
Successfu	0	8.30152257990064e-11	0.66854402381782	0.813	0.759	2.78416464284708e-06	ANKRD44
Successfu	0	8.56948452118508e-11	0.52671196141485	0.493	0.35	2.87403371871505e-06	CLIC1
Successfu	0	9.26407525980424e-11	0.58324392804167	0.655	0.58	3.10698556063315e-06	SLC25A6
Successfu	0	9.94504477760233e-11	0.27219756643659	0.37	0.192	3.33536911751227e-06	HHLA2
Successfu	0	1.0635343231884e-10	0.27421563037512	0.151	0.042	3.56688141310927e-06	C11orf86
Successfu	0	1.28054560675376e-10	0.91131094266720	0.567	0.41	4.29469385593075e-06	ATP1B3
Successfu	0	1.4479688047974e-10	0.37369591974950	0.268	0.12	4.85619777752952e-06	SLC2A2

Successfu	0	1.45531810152284e-10	0.53207854956609	0.391	0.203	4.8808458488873e-06	ZEB2
Successfu	0	1.53595731830047e-10	0.27747859432161	0.278	0.124	5.15129365411611e-06	B3GNT5
Successfu	0	1.67537838873208e-10	0.32096132589058	0.549	0.377	5.61888404012966e-06	CYP3A5
Successfu	0	1.71974533099762e-10	0.46330017851804	0.377	0.2	5.76768189109981e-06	SWAP70
Successfu	0	2.02512074299992e-10	0.27559597160395	1	0.994	6.79184994787313e-06	MT-ND1
Successfu	0	2.05638188860916e-10	0.32626160927595	0.261	0.108	6.89669357801741e-06	CYBB
Successfu	0	2.37547832820838e-10	0.68587166472267	0.493	0.321	7.96687921714528e-06	LDLRAD4
Successfu	0	2.46290623429425e-10	0.31231969557924	0.313	0.153	8.26009492857605e-06	TMIGD1
Successfu	0	2.50315464852177e-10	0.68862441624196	0.69	0.622	8.39508006021232e-06	SEPT7
Successfu	0	3.29110250196514e-10	0.29162810607287	0.405	0.234	1.10376995710907e-05	TMC5
Successfu	0	3.44636541424865e-10	0.64671467411791	0.525	0.389	1.15584203263071e-05	EIF3F
Successfu	0	3.70231803553804e-10	0.36259385683306	0.479	0.328	1.24168342275875e-05	EIF3L
Successfu	0	3.73350676958701e-10	0.53737662639511	0.454	0.272	1.25214350038409e-05	LCP1
Successfu	0	3.87456300417655e-10	0.88979610214788	0.532	0.399	1.29945094034073e-05	CREM
Successfu	0	4.34610333581901e-10	0.91629209475785	0.655	0.513	1.45759613676698e-05	AP001011.1
Successfu	0	4.68943276747645e-10	0.49358677257642	0.553	0.423	1.57274196155625e-05	TOMM20
Successfu	0	4.80377458943799e-10	0.32289767527095	0.208	0.081	1.61108992180571e-05	RBM38
Successfu	0	4.89546704060112e-10	0.59647604159627	0.514	0.405	1.6418417360768e-05	RPS17
Successfu	0	5.04594062433545e-10	0.55617616649031	0.637	0.527	1.69230756658962e-05	ARPC3
Successfu	0	5.56163915642287e-10	0.33282784570294	0.18	0.059	1.8652625402811e-05	FAM129C
Successfu	0	5.73462217152063e-10	0.38364441745151	0.475	0.31	1.92327758388459e-05	SEPT9
Successfu	0	6.15752713660193e-10	0.29748187942133	0.176	0.058	2.06511145107355e-05	CNR1
Successfu	0	6.22612644558601e-10	0.87668437098450	0.296	0.138	2.08811828732064e-05	IL7R
Successfu	0	6.33748920024013e-10	0.56242507524229	0.419	0.25	2.12546712797653e-05	C15orf48
Successfu	0	6.91660384350931e-10	0.5378787081274	0.507	0.355	2.31969059703615e-05	CHPT1
Successfu	0	7.14590686977851e-10	0.54484785285281	0.535	0.409	2.39659424598632e-05	NOP53
Successfu	0	7.51141393960472e-10	0.39057648543398	0.25	0.113	2.51917800706463e-05	FXYD3
Successfu	0	8.4347704349937e-10	0.33029180285979	0.366	0.205	2.82885330848819e-05	CDHR2
Successfu	0	8.53594910910668e-10	0.41231534080569	0.401	0.254	2.8627866122122e-05	C7orf50
Successfu	0	9.16525582032059e-10	0.78332395379885	0.732	0.643	3.07384349701912e-05	CCNH
Successfu	0	1.02209708041775e-09	0.25413288531755	0.211	0.078	3.42790918830504e-05	CR1

Successfu	0	1.04394781832815e-09	0.25927558165642	0.426	0.26	3.50119219310895e-05	IFI27
Successfu	0	1.11138772778571e-09	0.40956720330062	0.479	0.314	3.72737216144773e-05	CST3
Successfu	0	1.20475163130512e-09	0.33776101405644	0.475	0.303	4.04049602107111e-05	LPGAT1
Successfu	0	1.24539378983643e-09	0.59668079135736	0.574	0.444	4.17680169235342e-05	STK17A
Successfu	0	1.30069330961634e-09	0.27467483388478	0.208	0.078	4.36226522179129e-05	CCR6
Successfu	0	1.49920980579827e-09	0.51501193368246	0.306	0.157	5.02804984668625e-05	FRMD3
Successfu	0	1.56942689092485e-09	0.36384023496988	0.335	0.184	5.26354390678375e-05	GPSM3
Successfu	0	1.77717839482604e-09	0.33578037206140	0.764	0.672	5.96030090056756e-05	HINT1
Successfu	0	2.01993064981345e-09	0.44229785442528	0.299	0.151	6.77444341334435e-05	CXADR
Successfu	0	2.06251154818909e-09	0.46822068140505	0.637	0.542	6.91725123031657e-05	S100A10
Successfu	0	2.07620371450389e-09	0.35136728508588	0.331	0.179	6.96317201770313e-05	TJP1
Successfu	0	2.39337688163498e-09	1.43949751330167	0.415	0.278	8.02690738562739e-05	CRYBG1
Successfu	0	2.77326976965028e-09	0.66137390454910	0.683	0.644	9.30099215345312e-05	RPL4
Successfu	0	2.87975772645303e-09	0.59122110152803	0.461	0.302	9.65813146297818e-05	SLC4A7
Successfu	0	2.88465231389616e-09	0.30784046146261	0.282	0.143	9.67454693034495e-05	THEMIS2
Successfu	0	3.43588527379329e-09	0.61355387937556	0.482	0.34	0.000115232720312479	BLK
Successfu	0	3.45504476330548e-09	0.90233755524163	0.539	0.41	0.000115875291271739	NR4A2
Successfu	0	3.693858656909e-09	0.46769795592887	0.44	0.291	0.000123884631635414	FAM102A
Successfu	0	3.89534439727313e-09	0.49689496544295	0.648	0.561	0.000130642060395746	CCNI
Successfu	0	4.15207669777213e-09	0.84868695569338	0.458	0.295	0.000139252348289882	FYN
Successfu	0	4.62994404503884e-09	0.50069540670387	0.718	0.639	0.000155279063382513	SF1
Successfu	0	4.65869035704416e-09	0.26598746516915	0.18	0.067	0.000156243157194547	KCNK5
Successfu	0	4.86154657295005e-09	0.37146814164674	0.303	0.154	0.000163046548963599	PKIG
Successfu	0	4.95153640997142e-09	0.57583962003991	0.408	0.265	0.000166064628117621	GNA12
Successfu	0	5.14860502017541e-09	0.67472947415474	0.616	0.504	0.000172673915166643	RANBP2
Successfu	0	5.21993325106896e-09	0.51956341067925	0.785	0.707	0.000175066121374351	ANKRD11
Successfu	0	5.51620206652808e-09	0.28235059062766	0.127	0.036	0.000185002384907219	C19orf33
Successfu	0	5.78682886984716e-09	0.45311835370940	0.327	0.181	0.000194078666636934	SGPP1
Successfu	0	7.0475329052253e-09	0.38631142858737	0.254	0.115	0.000236360158575446	PIK3R5
Successfu	0	7.46206908698699e-09	0.58499196028542	0.574	0.467	0.00025026287303937	CD48
Successfu	0	9.23738001331061e-09	0.30589438981962	0.687	0.53	0.000309803250886411	ATP1A1

Successfu	0	1.02114158315809e-08	0.62493816363301	0.451	0.309	0.000342470464159561	CMTM7
Successfu	0	1.13764225187321e-08	0.44468745660362	0.342	0.191	0.000381542458433239	TBC1D8
Successfu	0	1.17481210755104e-08	0.52868873749723	0.401	0.232	0.000394008484630468	EBF1
Successfu	0	1.39526583079927e-08	0.42986490725272	0.905	0.833	0.000467944254333459	EZR
Successfu	0	1.50696440008296e-08	0.40486443703136	0.729	0.651	0.000505405720499824	PPIA
Successfu	0	1.60407494754211e-08	0.57776419144986	0.444	0.3	0.000537974655906673	PIKFYVE
Successfu	0	1.7156630980032e-08	0.34116963388087	0.254	0.122	0.000575399089808314	SLC6A19
Successfu	0	1.82445410409056e-08	0.25153896281766	0.155	0.052	0.000611885417429891	CNR2
Successfu	0	1.9897366842725e-08	0.43332758069870	0.486	0.346	0.000667317889171311	CYB5A
Successfu	0	2.15916534622195e-08	0.29469268574505	0.415	0.263	0.000724140873815918	SMAD3
Successfu	0	2.17908996375747e-08	0.78082013081960	0.525	0.382	0.000730823192044981	SYTL3
Successfu	0	2.32314075102911e-08	0.49479136135552	0.423	0.284	0.000779134945080145	CCDC6
Successfu	0	2.45599368058412e-08	0.57180941247723	0.56	0.455	0.000823691160594302	RBM3
Successfu	0	2.67346781935103e-08	0.27705000707659	0.803	0.746	0.000896627637253949	HLA-C
Successfu	0	3.14095707983411e-08	0.50888594915986	0.394	0.261	0.00105341418543476	CYFIP2
Successfu	0	3.18104138951688e-08	0.44943866991575	0.363	0.223	0.00106685766121617	ZCCHC10
Successfu	0	3.20597352828605e-08	0.41748007068387	0.511	0.395	0.00107521940191658	GPX4
Successfu	0	3.43051885436672e-08	1.00241172380475	0.282	0.148	0.00115052741337751	SCML4
Successfu	0	4.15885105109934e-08	0.28633872623980	0.479	0.336	0.0013947954655177	CDHR5
Successfu	0	4.31422321356921e-08	0.30092274888440	0.25	0.123	0.00144690418136684	CLDN15
Successfu	0	4.45766994479693e-08	0.41626972899846	0.25	0.126	0.00149501334608599	PRSS3
Successfu	0	4.74529058742801e-08	0.41540308100803	0.25	0.119	0.00159147555721161	GRK3
Successfu	0	5.10901019787403e-08	0.44158727681064	0.335	0.202	0.00171345984016299	CTSD
Successfu	0	5.36722566434013e-08	0.32572764590312	0.366	0.221	0.00180006014330639	PCK1
Successfu	0	5.36846307017191e-08	0.34326313781136	0.359	0.214	0.00180047514447426	DEK
Successfu	0	5.5028891457283e-08	0.60429629742656	0.542	0.399	0.00184555896169436	LY9
Successfu	0	6.28404709393484e-08	0.45519852853418	0.447	0.318	0.00210754371436386	SH3BP5
Successfu	0	6.57452068356596e-08	0.38542672164713	0.183	0.076	0.00220496274685435	SFN
Successfu	0	6.58499113171942e-08	0.37018696928167	0.155	0.057	0.00220847432575606	IPCEF1
Successfu	0	6.97033889460469e-08	0.33108896429959	0.204	0.089	0.00233771225847252	CCR7
Successfu	0	7.05885010781416e-08	0.47990093067067	0.711	0.649	0.00236739714915871	SARAF

Successfu	0	8.25524073351101e-08	0.375959074434389	0.352	0.213	0.00276864263720492	IFITM2
Successfu	0	9.6262863320539e-08	0.465778463549144	0.746	0.716	0.00322846391004424	ATP5F1E
Successfu	0	1.01258678682073e-07	0.482496498946231	0.444	0.32	0.00339601356563937	SP110
Successfu	0	1.05935648523198e-07	0.467098614131143	0.627	0.557	0.00355286978017103	UQCRB
Successfu	0	1.11653187945645e-07	0.412800499062906	0.838	0.778	0.00374462461732104	ELF1
Successfu	0	1.18880876017203e-07	0.434675257072324	0.514	0.408	0.00398702681986495	DDT
Successfu	0	1.22655219645968e-07	0.489579947790121	0.303	0.171	0.00411361075648648	PRMT9
Successfu	0	1.27733214652063e-07	0.250353702732749	0.789	0.736	0.00428391655300087	MYL6
Successfu	0	1.33664556694337e-07	0.27309260995276	0.335	0.193	0.00448284190241469	TUBA1A
Successfu	0	1.4088435064634e-07	0.276266323215619	0.306	0.171	0.00472497935197694	B4GALT1
Successfu	0	1.43843892528903e-07	0.654134315675642	0.567	0.462	0.00482423646763434	SP140
Successfu	0	1.4388063720643e-07	0.257329692348805	0.764	0.622	0.00482546881062925	LPP
Successfu	0	1.43978313701818e-07	0.32107563999863	0.606	0.439	0.00482874468493156	NR3C2
Successfu	0	1.57543867823281e-07	0.296183598468406	0.268	0.142	0.0052837062390572	KLF9
Successfu	0	1.75240211966616e-07	0.252033124280948	0.151	0.057	0.00587720622893636	TNF
Successfu	0	1.75343159706084e-07	0.674859794214134	0.602	0.468	0.00588065889022264	RGS1
Successfu	0	1.75826751030639e-07	0.47051054188956	0.585	0.489	0.00589687757606558	EIF4B
Successfu	0	1.82032426865083e-07	0.700517531094874	0.514	0.387	0.00610500353220116	GNG2
Successfu	0	1.84884472644358e-07	0.561004553584914	0.69	0.664	0.00620065544354647	NPM1
Successfu	0	1.91253438447111e-07	0.402491297151215	0.711	0.615	0.00641425781863921	VMP1
Successfu	0	2.04428775137771e-07	0.498701163604331	0.602	0.52	0.00685613226057055	ZFAS1
Successfu	0	2.05693355274019e-07	0.315186639045029	0.285	0.158	0.00689854374918006	GNG12
Successfu	0	2.08675995892014e-07	0.69447847086662	0.447	0.33	0.00699857555022638	USP36
Successfu	0	2.17266317812137e-07	0.305419752249257	0.458	0.33	0.00728667776678344	TSPAN8
Successfu	0	2.18132404501348e-07	0.313062939614076	0.687	0.647	0.00731572458216619	SRP14
Successfu	0	2.18675636452251e-07	0.366985148976908	0.208	0.094	0.0073339434953356	MCOLN2
Successfu	0	2.22775907566513e-07	0.470795686895823	0.405	0.269	0.00747145838796572	STK39
Successfu	0	2.30587428018513e-07	0.282841563503951	0.863	0.802	0.00773344116088489	YWHAZ
Successfu	0	2.3970923384873e-07	0.491186780054418	0.711	0.662	0.00803936828481871	NAP1L1
Successfu	0	2.40869542057227e-07	0.331970260102978	0.176	0.075	0.00807828270151529	ANKRD9
Successfu	0	2.5727733462062e-07	0.596907123764788	0.472	0.357	0.00862856724850636	CMTM6

Successfu	0	2.6495787982103e-07	1.22508395892228	0.616	0.517	0.0088861573734377	LINC00513
Successfu	0	3.45022292213437e-07	0.592608087827413	0.489	0.365	0.0115713576362542	GNA13
Successfu	0	3.66646736939949e-07	0.46591201666751	0.549	0.484	0.012296598263492	CHCHD10
Successfu	0	4.02521150540702e-07	0.504082830394515	0.782	0.78	0.0134997543468341	H3F3A
Successfu	0	4.06429435935703e-07	0.640447910061922	0.423	0.28	0.0136308304224116	NR4A3
Successfu	0	4.1181983227408e-07	0.300628831799989	0.264	0.139	0.0138116135348081	NFATC1
Successfu	0	5.37048993413266e-07	0.450119068583699	0.581	0.507	0.0180115491410941	EIF3E
Successfu	0	5.51746118549618e-07	0.563422484059467	0.588	0.506	0.0185044613239171	PHF20
Successfu	0	5.63122296450647e-07	0.615274748221635	0.229	0.115	0.0188859955783618	UBASH3B
Successfu	0	5.87188307674375e-07	0.288262300933274	0.183	0.086	0.0196931214627832	CAPG
Successfu	0	6.63644268613043e-07	0.334150449801523	0.444	0.329	0.0222573014807442	MTPN
Successfu	0	7.105318058046e-07	0.320265761605997	0.264	0.146	0.0238298157030747	ARHGAP4
Successfu	0	7.98644388261839e-07	0.36778714234339	0.208	0.1	0.0267849354935256	SLC44A2
Successfu	0	8.20405438095328e-07	0.38945655375127	0.627	0.543	0.0275147575828411	HMGN2
Successfu	0	8.32665252281284e-07	0.310194535750028	0.891	0.874	0.0279259272310097	TMSB10
Successfu	0	8.45957628880507e-07	0.370035953073648	0.215	0.106	0.0283717269573945	SNX8
Successfu	0	8.48800235378278e-07	0.386895872050132	0.57	0.439	0.0284670622941167	FGD4
Successfu	0	9.42633977832653e-07	0.470966989136367	0.894	0.844	0.0316140583485515	AKAP13
Successfu	0	9.82166674155874e-07	0.469457153106856	0.658	0.6	0.0329399059178397	HLA-E
Successfu	0	1.09370668912288e-06	0.50200478217319	0.394	0.264	0.0366807349398031	CARD11
Successfu	0	1.27132907261919e-06	0.283907172840851	0.18	0.081	0.0426378344375024	FCRL3
Successfu	0	1.40080602309807e-06	0.381204654727666	0.37	0.24	0.0469802324026629	CLEC2D
Successfu	0	1.44991464098868e-06	0.301812159057689	0.296	0.173	0.0486272372294784	ERRFI1
Successfu	0	1.45811567103223e-06	0.452234563677221	0.489	0.385	0.0489022833750789	TMEM243
Successfu	0	1.48134919680358e-06	0.391604493277744	0.363	0.237	0.0496814893623984	SHROOM3
Successfu	6	9.81790755709257e-39	0.864197570656634	0.762	0.23	3.29272983649771e-34	SPAG4
Successfu	6	1.03803698878082e-33	0.410136384487527	0.5	0.094	3.48136845297312e-29	TNFRSF4
Successfu	6	1.9903390886273e-32	0.498345198810144	0.667	0.189	6.67519923543822e-28	TNFRSF18
Successfu	6	1.55790699271948e-30	0.446973135766295	0.579	0.155	5.22490847218261e-26	CCR10
Successfu	6	1.85892044260463e-28	1.35374896062865	0.841	0.461	6.23444738040742e-24	DERL3
Successfu	6	2.25302743527243e-27	1.32420632691916	0.897	0.597	7.55620341241668e-23	MZB1

Successfu	6	1.00810360652892e-26	0.460709014461052	0.643	0.187	3.3809778755767e-22	NME1
Successfu	6	6.12233285675955e-26	0.921235571099222	0.786	0.357	2.05330799350002e-21	SDF2L1
Successfu	6	1.02568290825894e-25	0.899237987059905	0.762	0.34	3.43993533771882e-21	PRDX4
Successfu	6	2.84676965832097e-25	0.874670219004164	0.754	0.34	9.54749608007688e-21	SELENOM
Successfu	6	2.44711503922175e-24	1.13068304146161	0.841	0.501	8.20713441854191e-20	SEC11C
Successfu	6	9.86924002451998e-24	0.762425658166894	0.794	0.373	3.30994571942351e-19	MANF
Successfu	6	1.03966316031038e-23	0.281488665592251	0.516	0.139	3.48682230704894e-19	FAM89B
Successfu	6	1.19095131210494e-23	0.287909435916232	0.619	0.193	3.99421251053756e-19	NDUFAF3
Successfu	6	1.57403195766453e-23	0.468086633113831	0.738	0.272	5.27898837961531e-19	TMEM208
Successfu	6	3.25230434951569e-23	0.297295594601376	0.421	0.098	1.09075783274057e-18	FAM92B
Successfu	6	3.3941557612504e-23	0.252539150268665	0.587	0.173	1.13833195920816e-18	PSENEN
Successfu	6	3.40344020652847e-23	0.923492319284647	0.841	0.448	1.14144577646552e-18	FKBP11
Successfu	6	8.46608138666781e-23	0.266872754660031	0.421	0.099	2.83935437546065e-18	SYNGR1
Successfu	6	3.37380300906122e-22	0.636010224152517	0.778	0.362	1.13150605317895e-17	MYDGF
Successfu	6	4.46513768384058e-22	1.31034319794815	0.944	0.873	1.49751787640646e-17	JCHAIN
Successfu	6	6.72459754436088e-22	0.292448391697152	0.683	0.245	2.25529552442775e-17	NDUFS8
Successfu	6	8.61053353396e-22	0.449949480479274	0.69	0.256	2.8878007366195e-17	TMED9
Successfu	6	1.05057418200272e-21	0.472792843395249	0.706	0.267	3.52341569160072e-17	SRM
Successfu	6	1.98614638387791e-21	0.689955536448271	0.786	0.364	6.66113774224975e-17	KDELR2
Successfu	6	1.99937895625409e-20	0.871071294901204	0.857	0.617	6.70551714348495e-16	PPIB
Successfu	6	3.96761827957411e-20	0.32692822608321	0.627	0.22	1.33065981860356e-15	PSMB6
Successfu	6	1.54390474054194e-19	1.15378421753356	0.841	0.728	5.17794771882957e-15	SSR4
Successfu	6	1.63687797128358e-19	0.81557728530237	0.817	0.469	5.48976134009088e-15	ITM2C
Successfu	6	3.09109527145048e-19	0.558330911314665	0.675	0.277	1.03669153213906e-14	CNPY2
Successfu	6	5.9427487087936e-19	0.674937000086752	0.817	0.489	1.9930790619552e-14	RABAC1
Successfu	6	6.7808131014574e-19	0.844638768411054	0.849	0.532	2.27414909796678e-14	FKBP2
Successfu	6	1.27484039032522e-18	0.483938068094064	0.706	0.306	4.27555970107274e-14	SDC1
Successfu	6	2.80249400441064e-18	0.528460556105919	0.786	0.409	9.39900439199242e-14	KDELR1
Successfu	6	5.6406840094062e-18	0.295047542232132	0.5	0.159	1.89177260307465e-13	SLC35B1
Successfu	6	7.34410603903119e-18	0.386908688123441	0.69	0.294	2.46306628337028e-13	ATRAID
Successfu	6	1.3208952291671e-17	0.408121996100346	0.698	0.279	4.43001841958063e-13	MRPL51

Successfu	6	5.04148836595493e-17	0.871835088024365	0.865	0.729	1.69081436817396e-12	CYBA
Successfu	6	6.04317332070858e-17	0.664732143726541	0.794	0.443	2.02675946829924e-12	TNFRSF17
Successfu	6	2.74938269053497e-16	0.281715744416325	0.5	0.159	9.22087966751618e-12	UBE2M
Successfu	6	1.12030637728211e-15	0.805415534456932	0.81	0.565	3.75728352812873e-11	SPCS1
Successfu	6	2.31862811187888e-15	0.640190471499895	0.778	0.464	7.77621496161938e-11	GSTP1
Successfu	6	2.41642149010977e-15	0.398826957163168	0.738	0.32	8.10419439353014e-11	NDUFC2
Successfu	6	3.17170999021419e-15	0.512252429802365	0.714	0.344	1.06372809651804e-10	CRELD2
Successfu	6	4.57806762850229e-15	0.572611427938946	0.619	0.278	1.5353923212471e-10	DDOST
Successfu	6	5.17462860756909e-15	0.339469947261131	0.619	0.246	1.73546694240652e-10	ICAM3
Successfu	6	2.04640925088479e-14	0.293326636024544	0.69	0.292	6.86324734561742e-10	AURKAIP1
Successfu	6	3.22805461388931e-14	0.641975714629559	0.825	0.63	1.0826249564062e-09	SEC61B
Successfu	6	6.15059076114229e-14	0.25930312129317	0.349	0.1	2.0627851294719e-09	GRHPR
Successfu	6	1.19534613543798e-13	0.530866365774312	0.944	0.918	4.00895186903188e-09	FTL
Successfu	6	2.08707337518106e-13	0.269844192930241	0.643	0.264	6.99962668568225e-09	PSMD8
Successfu	6	2.2309861610858e-13	0.268533586481705	0.548	0.209	7.48228138704954e-09	AUP1
Successfu	6	2.3232370799124e-13	0.410484711971125	0.706	0.328	7.79167251861022e-09	CLPTM1L
Successfu	6	2.34152486044338e-13	0.260053828713132	0.738	0.335	7.853006076955e-09	ALG5
Successfu	6	2.40263843541574e-13	0.340660118481087	0.643	0.309	8.05796878469733e-09	TIMP1
Successfu	6	3.10778263116293e-13	0.393251810727404	0.738	0.386	1.04228813883942e-08	PLPP5
Successfu	6	3.19949869971933e-13	0.474861570663823	0.825	0.49	1.07304787391187e-08	SEC61G
Successfu	6	6.10219911296299e-13	0.272431475305579	0.714	0.336	2.04655553850553e-08	SRPRA
Successfu	6	7.12402996010861e-13	0.254922072532197	0.754	0.358	2.38925716802123e-08	PRDX5
Successfu	6	1.01243387564146e-12	0.460094668898548	0.746	0.421	3.39550073212634e-08	CUTA
Successfu	6	1.10144980366591e-12	0.4548889936772	0.762	0.424	3.69404235153472e-08	LMAN2
Successfu	6	1.14643831689944e-12	1.71702158175746	0.984	0.976	3.84492482721733e-08	IGKC
Successfu	6	1.21270760594987e-12	0.60308946379435	0.794	0.479	4.06717876883467e-08	PDIA6
Successfu	6	1.28899165548785e-12	0.563561914689618	0.817	0.565	4.32302021417515e-08	TMEM258
Successfu	6	1.53636692136399e-12	0.284390798297228	0.627	0.273	5.15266738087055e-08	NENF
Successfu	6	2.44320642162356e-12	0.620410985635891	0.794	0.499	8.19402569684109e-08	SSR2
Successfu	6	2.82813265711442e-12	0.291107081798274	0.754	0.397	9.48499130543033e-08	GUK1
Successfu	6	2.98687069020013e-12	0.798878513263912	0.873	0.746	1.00173669207932e-07	HLA-C

Successfu	6	3.20846102685132e-12	0.27192597632013[illegible]	0.69	0.343	1.0760536591854e-07	SRSF9
Successfu	6	4.12396598683266e-12	0.25236632436047[illegible]	0.659	0.286	1.38309571266394e-07	NDUFA13
Successfu	6	5.96369906280402e-12	0.52229484596664[illegible]	0.841	0.68	2.00010539168321e-07	SPCS2
Successfu	6	5.98198372583937e-12	0.56067546702914[illegible]	0.841	0.627	2.00623770197201e-07	SELENOK
Successfu	6	7.63674801935138e-12	0.28767493289180[illegible]	0.778	0.407	2.56121255073007e-07	OSTC
Successfu	6	8.29741736813516e-12	0.36478707376467[illegible]	0.77	0.428	2.78278783692517e-07	KRT10
Successfu	6	8.4253946887891e-12	0.49881883284885[illegible]	0.825	0.505	2.82570887072609e-07	LGALS1
Successfu	6	9.58605358565612e-12	0.31640197848215[illegible]	0.746	0.378	3.21497065155735e-07	REEP5
Successfu	6	1.02581199673477e-11	0.44618224779643[illegible]	0.754	0.404	3.44036827464908e-07	CD27
Successfu	6	1.06683040015863e-11	0.54688220265916	0.825	0.567	3.57793579605202e-07	SSR3
Successfu	6	1.07401024113446e-11	0.27173928961186[illegible]	0.421	0.152	3.60201554671675e-07	B4GALT3
Successfu	6	1.87195239849132e-11	0.32485177428844[illegible]	0.556	0.23	6.2781539540602e-07	SRA1
Successfu	6	2.22354472701036e-11	0.30092640486429[illegible]	0.738	0.375	7.45732430544733e-07	ATP5MC1
Successfu	6	2.267678095319e-11	0.80690233220082[illegible]	0.643	0.315	7.60533879608088e-07	MIR155HG
Successfu	6	2.29670667630025e-11	0.39077173328138[illegible]	0.349	0.122	7.70269485097577e-07	IGLV3-1
Successfu	6	3.28913592383455e-11	0.42313554401868[illegible]	0.746	0.388	1.10311040613563e-06	PSME2
Successfu	6	3.33435559705948e-11	0.60608990605687[illegible]	0.968	0.958	1.11827618014181e-06	B2M
Successfu	6	3.45871910934182e-11	0.33406282867517[illegible]	0.69	0.323	1.15998521489106e-06	MLEC
Successfu	6	4.09927105473768e-11	0.31701702245256[illegible]	0.698	0.352	1.37481352633792e-06	COPE
Successfu	6	4.31946267113686e-11	0.30897922007482[illegible]	0.619	0.273	1.44866139064588e-06	ERGIC3
Successfu	6	5.11204937055936e-11	0.36283158860985[illegible]	0.73	0.386	1.7144791178982e-06	KRTCAP2
Successfu	6	8.13174928639751e-11	0.33877731937662[illegible]	0.714	0.372	2.727226075672e-06	JTB
Successfu	6	9.83385400451408e-11	0.41972361866380[illegible]	0.778	0.46	3.29807795603393e-06	TMEM59
Successfu	6	1.11093344374488e-10	0.40003866372653[illegible]	0.754	0.399	3.72584858363158e-06	ATP5MC3
Successfu	6	1.16435498323366e-10	0.48146564498958[illegible]	0.817	0.581	3.90501374276904e-06	CD63
Successfu	6	1.7091823673617e-10	0.25720499608513	0.31	0.101	5.73225582365766e-06	SNRNP25
Successfu	6	1.8373543412841e-10	0.44940099949131[illegible]	0.81	0.511	6.1621189897986e-06	TMED10
Successfu	6	1.23557980499614e-09	0.30944421711492[illegible]	0.659	0.325	4.14388754999605e-05	UFC1
Successfu	6	1.43951567494286e-09	0.25576786741302	0.651	0.334	4.82784767062337e-05	BST2
Successfu	6	1.5760179333056e-09	0.27969833124968[illegible]	0.754	0.409	5.28564894472032e-05	EMP3
Successfu	6	1.73095976755133e-09	0.49464294796333[illegible]	0.817	0.594	5.80529286841366e-05	SPCS3

Successfu	6	1.83544388175385e-09	0.370876568711078	0.111	0.018	6.15571169062607e-05	IGKV2D-28
Successfu	6	2.1156262028485e-09	0.359382704658457	0.849	0.566	7.09538715911331e-05	CALR
Successfu	6	2.48374129999983e-09	0.322884527215592	0.794	0.489	8.32997157193943e-05	DAD1
Successfu	6	2.78653826037457e-09	0.271572783910374	0.754	0.389	9.34549201764424e-05	RGCC
Successfu	6	2.79959809476053e-09	0.328431917253307	0.548	0.25	9.38929209020786e-05	MRPL52
Successfu	6	4.15565785675178e-09	0.337954932595511	0.762	0.456	0.000139372453199741	DNAJB9
Successfu	6	6.78661084424356e-09	0.285548049947545	0.651	0.316	0.00022760935449424	NME3
Successfu	6	1.93476897707045e-08	0.303954090374013	0.825	0.517	0.000648882819529889	SELENOS
Successfu	6	2.90996245226211e-08	0.573426539754036	0.556	0.307	0.000975943207239668	DUSP2
Successfu	6	3.25609768400624e-08	0.31619062425201	0.817	0.514	0.00109203004126201	LMAN1
Successfu	6	4.09617759564918e-08	1.08059182492856	0.921	0.92	0.00137377604202882	IGHA1
Successfu	6	4.14663735943541e-08	0.369348262368162	0.857	0.522	0.00139069923760745	SLAMF7
Successfu	6	6.33248639779654e-08	0.286523540113744	0.651	0.344	0.002123789288093	COX5A
Successfu	6	6.82920472331191e-08	0.397543996079194	0.921	0.786	0.00229037868010435	HSP90B1
Successfu	6	7.11239953323806e-08	0.426160581244696	0.754	0.46	0.00238535655545738	ERLEC1
Successfu	6	8.07796833808646e-08	0.398651163556221	0.81	0.558	0.00270918902122744	CHCHD2
Successfu	6	1.20917123990079e-07	1.11117175911222	0.349	0.155	0.00405531850437927	IGKV4-1
Successfu	6	1.91325010683253e-07	0.313071154240061	0.817	0.581	0.00641665820829494	DYNLL1
Successfu	6	3.0093096991476e-07	0.53324096815972	0.817	0.547	0.0100926228690012	TXN
Successfu	6	3.13390573368847e-07	0.331130989081944	0.754	0.457	0.0105104930496444	PSMB1
Successfu	6	3.38835058750772e-07	0.345362565220214	0.857	0.607	0.0113638502003834	XBP1
Successfu	6	5.71219786380832e-07	0.387797611111638	0.849	0.708	0.0191575691956404	RPL36AL
Successfu	6	5.7748307862212e-07	0.337953972419599	0.802	0.605	0.0193676274908287	OST4
Successfu	6	6.99825997415677e-07	0.391074221106402	0.73	0.426	0.023470764301327	NPC2
Successfu	6	7.30411995195524e-07	0.455861871123412	0.857	0.672	0.0244965574948675	NFKBIA
Successfu	6	8.10744348102174e-07	0.714043967760143	0.254	0.105	0.0271907439466507	CCL3
Successfu	6	9.86090123662663e-07	0.252445680199372	0.119	0.029	0.0330714905673984	FEN1
Successfu	6	1.150027139516e-06	0.28178594851689	0.897	0.758	0.0385696102050876	RPLP0
Successfu	6	1.2176894231143e-06	0.330454137858845	0.857	0.655	0.0408388678724074	UBE2J1
Successfu	6	1.43977974046425e-06	0.271963052984847	0.746	0.428	0.0482873329356902	RPN1
Successfu	12	2.0805806149322e-23	1.31062559714836	0.921	0.741	6.97785126635961e-19	RALGPS2

Successfu	12	5.72742893905947e-22	1.35181774341619	0.822	0.565	1.92086511758176e-17	RASGEF1B
Successfu	12	4.18593202490564e-20	1.12619776241867	0.816	0.517	1.40387788251285e-15	TBC1D9
Successfu	12	5.73496353047872e-19	1.05633104005425	0.428	0.141	1.92339206885195e-14	AP002075.1
Successfu	12	1.09354581537695e-18	0.783157347368311	0.829	0.563	3.66753395561122e-14	UVRAG
Successfu	12	2.63531948968109e-18	1.62424567162468	0.592	0.291	8.83833450449246e-14	BANK1
Successfu	12	4.89028038978916e-18	0.87214198842986	0.303	0.078	1.64010223712749e-13	TMEM163
Successfu	12	7.10883436061728e-18	1.00340092598603	0.638	0.323	2.38416086786382e-13	JAZF1
Successfu	12	7.18048027601158e-18	1.11028872160803	0.914	0.754	2.40818947496876e-13	MEF2C
Successfu	12	7.48670984749069e-18	0.824817719136368	0.888	0.694	2.51089274865143e-13	ELMO1
Successfu	12	2.5158615924832e-16	0.675934690042976	0.947	0.817	8.43769660887014e-12	TNRC6B
Successfu	12	2.65865743611872e-16	0.740039157527443	0.684	0.381	8.91660530925497e-12	INPP5D
Successfu	12	4.88470001322076e-16	1.04594458209625	0.809	0.576	1.63823069043398e-11	ATP2B1
Successfu	12	5.73405161235378e-16	0.877541068413052	0.618	0.346	1.92308622975121e-11	ANGPTL1
Successfu	12	6.47582733814321e-16	0.301717890671341	0.467	0.188	2.17186297266647e-11	DEFA6
Successfu	12	9.49444152251828e-16	0.557250241564991	0.421	0.157	3.18424579782218e-11	LRRK2
Successfu	12	1.24036446397798e-15	0.47425445864689	0.388	0.137	4.15993433928935e-11	AC008937.2
Successfu	12	1.24170565938609e-15	0.788791334246546	0.414	0.155	4.16443244044906e-11	FCRL1
Successfu	12	1.33581073673472e-15	0.362884344002413	0.48	0.211	4.4800420488609e-11	HBB
Successfu	12	2.14417395114858e-15	0.756652590678113	0.961	0.813	7.19113059736212e-11	SIK3
Successfu	12	2.94496458077273e-15	0.916699050899703	0.921	0.813	9.87682221099558e-11	ARHGAP15
Successfu	12	3.42818291679497e-15	1.68990425389721	0.586	0.331	1.1497439866347e-10	ARHGAP24
Successfu	12	3.49903919613804e-15	0.730491064872865	0.48	0.196	1.17350776560077e-10	PARP15
Successfu	12	6.85408103740476e-15	0.773254547125625	0.954	0.859	2.29872169832481e-10	FOSB
Successfu	12	6.90706952738172e-15	0.658469613813996	0.355	0.121	2.31649297809328e-10	CD24
Successfu	12	2.54959416040221e-14	0.79406172551803	0.901	0.711	8.55082889515695e-10	DDX3X
Successfu	12	3.41824559354553e-14	0.612083006199601	0.783	0.526	1.1464112071633e-09	CDK13
Successfu	12	3.47588293872498e-14	0.924033889590159	0.664	0.401	1.16574161998958e-09	OSBPL10
Successfu	12	4.00324557381932e-14	0.594385786097832	0.941	0.862	1.34260850054752e-09	ZBTB20
Successfu	12	4.05819257969669e-14	0.719370531103124	0.934	0.759	1.36103662737867e-09	NCOA3
Successfu	12	4.62100110448695e-14	0.802599605513826	0.388	0.151	1.54979135042283e-09	PAX5
Successfu	12	4.90406489151084e-14	0.685226647718869	0.868	0.66	1.64472528331491e-09	DOCK8

Successfu	12	7.18153966782691e-14	0.74596660913267	0.776	0.53	2.40854477379579e-09	MAP3K1
Successfu	12	9.09252871645911e-14	1.0660505846778	0.671	0.397	3.04945228092606e-09	BACH2
Successfu	12	1.07876149851340e-13	0.86659454807934	0.836	0.632	3.61795031371424e-09	PDE7A
Successfu	12	1.56085016477249e-13	0.819569119628	0.836	0.647	5.23477928261397e-09	SETBP1
Successfu	12	1.59845107774969e-13	0.95848093110997	0.507	0.25	5.36088522455691e-09	RIPOR2
Successfu	12	1.90878021835105e-13	0.74029652532027	0.888	0.759	6.40166709630574e-09	FCHSD2
Successfu	12	1.94951972713808e-13	0.31073310698430	0.191	0.041	6.53829926087568e-09	SPIB
Successfu	12	2.1005836856192e-13	0.69174077643736	0.618	0.351	7.04493756482967e-09	PEAK1
Successfu	12	2.11082335868187e-13	1.48974783268184	0.493	0.255	7.07927938034724e-09	AFF3
Successfu	12	2.15579243792635e-13	0.97301329742468	0.757	0.537	7.23009667831738e-09	LYN
Successfu	12	2.84422107237954e-13	0.84459747580235	0.809	0.602	9.53894863254649e-09	PRKCB
Successfu	12	3.06902194305004e-13	0.70545864105688	0.849	0.707	1.02928857926012e-08	CYTIP
Successfu	12	3.4304949305388e-13	0.56518496253789	0.875	0.614	1.1505193898041e-08	MT1G
Successfu	12	3.62234414151484e-13	0.48100439886747	0.368	0.139	1.21486177818125e-08	RUBCNL
Successfu	12	4.87936849706931e-13	0.78944862445342	0.822	0.624	1.63644260654711e-08	EML4
Successfu	12	4.88664985825091e-13	0.53751058407355	0.75	0.472	1.63888462946019e-08	HBP1
Successfu	12	4.98811319141754e-13	0.76757265021393	0.612	0.339	1.67291340213761e-08	BLK
Successfu	12	5.31487082790503e-13	0.66579071743366	0.678	0.474	1.78250137826279e-08	SHOC2
Successfu	12	7.30616685028996e-13	0.58451278012813	0.553	0.281	2.45034223825025e-08	LCP1
Successfu	12	8.160709860409e-13	0.66693349671871	0.895	0.737	2.73693887298397e-08	FUT8
Successfu	12	1.11514263205142e-12	0.61136311163042	0.487	0.24	3.73996535937406e-08	SLC23A2
Successfu	12	1.29361210609477e-12	0.59997655940218	0.855	0.678	4.33851628142065e-08	PAN3
Successfu	12	1.45922075290573e-12	0.72141562045327	0.493	0.24	4.89393456109525e-08	EBF1
Successfu	12	2.09127561686858e-12	0.73333203306210	0.382	0.155	7.01372016385384e-08	KHDRBS2
Successfu	12	2.40256538217908e-12	0.74078011673440	0.862	0.712	8.05772377875219e-08	TBC1D5
Successfu	12	2.56098582978856e-12	0.56877850345268	0.684	0.419	8.58903427594488e-08	ZDHHC21
Successfu	12	2.67394551973518e-12	0.66960102654107	0.921	0.8	8.96787848408785e-08	ARID1B
Successfu	12	2.79000486265672e-12	0.94372124159633	0.441	0.196	9.35711830837812e-08	MS4A1
Successfu	12	2.98861444255265e-12	1.16722879650936	0.763	0.624	1.00232151174331e-07	KLF2
Successfu	12	3.68640841283556e-12	0.60541474225967	0.52	0.279	1.23634765349679e-07	ALOX5
Successfu	12	4.57734842279722e-12	0.93353171466785	0.77	0.578	1.53515111403773e-07	TCF4

Successfu	12	5.86527454464211e-12	0.82529077107384	0.309	0.114	1.96709577678207e-07	JAM3
Successfu	12	8.76048285745532e-12	0.68517173002392	0.447	0.212	2.93809074073337e-07	SWAP70
Successfu	12	9.28775278370174e-12	1.21412503030289	0.553	0.318	3.11492652859789e-07	CD83
Successfu	12	9.3870977767171e-12	0.50404915023063	0.322	0.119	3.14824485235538e-07	CYBB
Successfu	12	1.08297375948881e-11	0.72017821874402	0.967	0.913	3.63207739457357e-07	AC016831.7
Successfu	12	1.3048612860355e-11	0.70557983998773	0.783	0.566	4.37624378110585e-07	RFX3
Successfu	12	1.40823351146888e-11	0.31987120723535	0.289	0.096	4.72293355076434e-07	MCOLN2
Successfu	12	1.45173965220713e-11	0.37716991772343	0.243	0.073	4.86884444557227e-07	TLR10
Successfu	12	1.60293990383855e-11	0.76643109280438	0.704	0.507	5.37593984949372e-07	CCDC50
Successfu	12	1.92729295920784e-11	0.49470266509327	0.737	0.538	6.46375512659124e-07	XRCC5
Successfu	12	1.98474954138892e-11	0.83587277639350	0.507	0.278	6.65645301191016e-07	PLEKHG1
Successfu	12	2.14815001753908e-11	0.48585219095887	0.257	0.083	7.20446552882257e-07	FCRL3
Successfu	12	2.30130161511554e-11	0.51216450062242	0.934	0.803	7.71810535677448e-07	ARIH1
Successfu	12	2.6811933697775e-11	0.45543815519286	0.303	0.105	8.99218632355979e-07	LY86
Successfu	12	2.80010804032181e-11	0.68728670005512	0.158	0.034	9.39100234563128e-07	ATRNL1
Successfu	12	2.9866972785614e-11	0.64874990692262	0.638	0.41	1.00167853328392e-06	ANTXR2
Successfu	12	3.01168463805077e-11	0.50823957916347	0.697	0.468	1.01005879390947e-06	EPC2
Successfu	12	4.22876901872708e-11	0.55825193747203	0.638	0.409	1.41824455350069e-06	USP24
Successfu	12	6.80414551502514e-11	0.53200554753763	0.882	0.669	2.28197432282913e-06	ERBIN
Successfu	12	6.9699906846546e-11	0.53739106984749	0.717	0.507	2.33759547581946e-06	SREBF2
Successfu	12	8.07830704418019e-11	0.50763188764240	0.809	0.584	2.70930261647715e-06	COP1
Successfu	12	8.36778771509742e-11	0.39852258332716	0.921	0.741	2.80638864388937e-06	LRBA
Successfu	12	8.55379518995058e-11	0.664111266972996	0.691	0.484	2.86877183080562e-06	AKT3
Successfu	12	9.03267722313111e-11	0.48334953050984	0.77	0.552	3.02937928709371e-06	RICTOR
Successfu	12	9.09955221495202e-11	0.59210469920058	0.474	0.254	3.05180782185061e-06	TRIM38
Successfu	12	1.13315522396286e-10	0.44168915903129	0.401	0.195	3.80037599012663e-06	STAMBPL1
Successfu	12	1.14828035228478e-10	0.68290868033810	0.875	0.716	3.85110264549269e-06	DENND5B
Successfu	12	1.15813169767497e-10	0.57373129244312	0.75	0.536	3.8841420876623e-06	WIPF1
Successfu	12	1.18518920705415e-10	0.66286743407962	0.73	0.547	3.97488756261822e-06	XKR6
Successfu	12	1.21383197877909e-10	0.51296139294584	0.382	0.169	4.07094969042932e-06	PCNX2
Successfu	12	1.28178573065612e-10	0.80745555666746	0.855	0.67	4.2988529834745e-06	FAM214A

Successfu	12	1.28943732116434e-10	0.62210016904665	0.855	0.67	4.32451488772098e-06	PPP3CA
Successfu	12	1.36516699199762e-10	0.47082137224664	0.882	0.716	4.57849705776161e-06	MYCBP2
Successfu	12	1.60152321930127e-10	0.61672291699627	0.77	0.564	5.3711885728926e-06	BTBD9
Successfu	12	1.68718704569982e-10	1.05797992512781	0.572	0.359	5.65848791386805e-06	MAP4K4
Successfu	12	1.71258355864968e-10	0.54565264232710	0.875	0.733	5.7436627389993e-06	ZCCHC7
Successfu	12	2.02784991393516e-10	0.45329534771369	0.257	0.087	6.80100304135575e-06	CCR6
Successfu	12	2.30331400346199e-10	0.50026964558636	0.928	0.783	7.72485450481083e-06	PABPC1
Successfu	12	2.35571828476514e-10	0.40355739707310	0.309	0.119	7.90060798344531e-06	FCMR
Successfu	12	2.4400389787164e-10	0.72308940028957	0.849	0.69	8.18340272681907e-06	BRAF
Successfu	12	2.71203051334875e-10	0.68592870711261	0.539	0.309	9.09560793566905e-06	GAB2
Successfu	12	2.84061146670092e-10	0.39356285725033	0.171	0.043	9.52684273702155e-06	TNR
Successfu	12	2.95245792456671e-10	0.67375455565520	0.77	0.573	9.90195338741183e-06	NR4A1
Successfu	12	3.18154572545957e-10	0.48843566138375	0.829	0.657	1.06702680540463e-05	HIVEP2
Successfu	12	3.19271166512711e-10	0.56247270994230	0.711	0.493	1.07077163825033e-05	DOCK10
Successfu	12	3.32135541628997e-10	0.71213936712959	0.395	0.19	1.11391617951533e-05	SGPP1
Successfu	12	3.56028122652146e-10	0.57071387619495	0.579	0.352	1.19404711775077e-05	TET2
Successfu	12	4.03456100911059e-10	0.60352353236829	0.809	0.659	1.35311107123551e-05	JAK1
Successfu	12	4.58810735388673e-10	0.46077538274422	0.316	0.126	1.53875944434653e-05	GRK3
Successfu	12	4.85509972674842e-10	0.60942427428941	0.362	0.16	1.62830334635689e-05	TRAF5
Successfu	12	5.81111851157065e-10	0.69803534871490	0.816	0.626	1.94893292641056e-05	RAB11FIP1
Successfu	12	6.20289588454339e-10	0.56363697626927	0.48	0.264	2.08032722175816e-05	COL4A3
Successfu	12	6.67673850954626e-10	0.48980503216916	0.947	0.801	2.23924456133162e-05	JMJD1C
Successfu	12	6.83586969088278e-10	0.60610727409078	0.737	0.543	2.29261397692827e-05	TNFAIP8
Successfu	12	6.88098115913314e-10	0.73690647188741	0.895	0.754	2.30774346115007e-05	ANKRD44
Successfu	12	7.98963241058629e-10	0.60534089206252	0.25	0.088	2.67956291786243e-05	ADAMTS6
Successfu	12	8.53011361031143e-10	0.52783028420805	0.296	0.119	2.86082950262625e-05	KYNU
Successfu	12	1.15414704660042e-09	0.46161396367170	0.809	0.631	3.87077836488849e-05	VPS13C
Successfu	12	1.19167620714985e-09	0.40411608007470	0.803	0.633	3.99664366353917e-05	FUS
Successfu	12	1.2448007217129e-09	0.58178946486983	0.789	0.606	4.17481266048072e-05	RSRC2
Successfu	12	1.44300889354073e-09	0.79856512702816	0.586	0.383	4.83956322715689e-05	MAPRE2
Successfu	12	1.56707153794057e-09	0.35890291805184	0.842	0.675	5.25564452394507e-05	HNRNPA3

Successfu	12	1.5672721387463e-09	0.61840812134635	0.77	0.619	5.25631729892734e-05	TMEM131
Successfu	12	1.5772076569151e-09	0.60331510913912	0.592	0.369	5.28963903976188e-05	TMEM131L
Successfu	12	1.65896440223023e-09	0.83294883997078	0.461	0.252	5.56383481219975e-05	SESN3
Successfu	12	1.70077801413896e-09	0.45650787820494	0.612	0.389	5.70406930381925e-05	ZBTB1
Successfu	12	1.71644513107309e-09	0.48205885384695	0.783	0.568	5.75661368059294e-05	SETX
Successfu	12	1.86811898273171e-09	0.38196488207766	0.743	0.531	6.26529744428559e-05	KLF12
Successfu	12	1.94218388383941e-09	0.60013769588096	0.895	0.743	6.51369630962062e-05	KMT2E
Successfu	12	1.96085867396044e-09	0.52971026629661	0.849	0.698	6.57632782072854e-05	STK17B
Successfu	12	2.13730065539596e-09	0.37673143337529	0.178	0.051	7.16807893806698e-05	FCRLA
Successfu	12	2.28762796887237e-09	0.43386460498033	0.954	0.842	7.67224668200416e-05	AKAP13
Successfu	12	2.35730297606992e-09	0.74506659083334	0.882	0.758	7.90592272114331e-05	DNAJA1
Successfu	12	2.6946124787103e-09	0.59538906652492	0.77	0.596	9.03719133109859e-05	ZHX2
Successfu	12	2.76339768269405e-09	0.52014750763018	0.803	0.637	9.26788314821929e-05	SF1
Successfu	12	2.81240255974385e-09	0.36716457289370	0.928	0.818	9.43223570486894e-05	MTRNR2L12
Successfu	12	2.91911933319486e-09	1.20568332504964	0.474	0.266	9.79014241966893e-05	AC105402.3
Successfu	12	2.92896854154846e-09	0.51586512238797	0.645	0.441	9.82317469464524e-05	C6orf106
Successfu	12	2.96360828310401e-09	0.40937885117825	0.743	0.535	9.93934945987423e-05	ABI1
Successfu	12	3.13060218416681e-09	0.53138585580510	0.954	0.85	0.000104994136052586	HNRNPA2B1
Successfu	12	3.17302553414741e-09	0.46126747034892	0.941	0.861	0.000106416930364236	MBNL1
Successfu	12	3.38771104210759e-09	0.39942406746994	0.789	0.564	0.000113617052930204	TRIP12
Successfu	12	3.41618944303851e-09	0.51848695382948	0.737	0.551	0.000114572161540625	KAT6A
Successfu	12	3.52370830856513e-09	0.39671980839627	0.842	0.652	0.000118178129252657	HNRNPU
Successfu	12	3.65416043491352e-09	0.359356633361116	0.211	0.068	0.00012255323266613	CNR1
Successfu	12	3.73105101551376e-09	0.42146470014387	0.224	0.077	0.000125131988958301	OSTN
Successfu	12	3.90684670503403e-09	1.19011575027777	0.447	0.258	0.000131027824793431	GPR183
Successfu	12	4.10487236129001e-09	0.45897245274724	0.487	0.277	0.000137669209252944	PAWR
Successfu	12	4.25010517296826e-09	0.54245477430666	0.645	0.44	0.00014254002729101	XYLT1
Successfu	12	4.89417322413921e-09	0.51552155398372	0.776	0.613	0.000164140781591181	ZNF644
Successfu	12	4.96944795647962e-09	0.43652120835260	0.697	0.497	0.000166665345564414	CREBBP
Successfu	12	6.27006664024952e-09	0.45596283092140	0.651	0.455	0.000210285494980688	IFI16
Successfu	12	6.41470038215848e-09	0.55233162448520	0.592	0.373	0.000215136221416831	PLEKHA2

Successfu	12	6.56922435262278e-09	0.46913293175940	0.388	0.201	0.000220318646338263	SLC36A4
Successfu	12	6.6682921651708e-09	0.53360954869460	0.803	0.631	0.000223641182635498	ZSWIM6
Successfu	12	7.17327139325067e-09	0.71905492445058	0.388	0.191	0.000240577175986841	MARCH1
Successfu	12	7.59354614029699e-09	0.48253577624538	0.566	0.369	0.000254672350453281	NRF1
Successfu	12	7.75527560083511e-09	0.417114841187564	0.684	0.46	0.000260096433100808	EIF4G3
Successfu	12	7.79978869877993e-09	0.38954967404508	0.625	0.424	0.000261589313379681	ZNF33A
Successfu	12	7.90619785320663e-09	0.54467273814233	0.572	0.367	0.000265158063600844	PVT1
Successfu	12	8.68903240979382e-09	0.64687657470121	0.618	0.417	0.000291412768959665	MIR29B2CHG
Successfu	12	9.01544473475055e-09	0.39241186805934	0.934	0.782	0.000302359985514064	STAG1
Successfu	12	9.96806597087268e-09	0.67093654317047	0.375	0.19	0.000334308996531128	BCL11A
Successfu	12	1.06747035388732e-08	0.25432979235351	0.441	0.224	0.000358008207286729	COTL1
Successfu	12	1.07978429045157e-08	0.44387750690743	0.408	0.215	0.000362138055331647	SPOPL
Successfu	12	1.097848953431e-08	1.12185365751662	0.553	0.379	0.000368196582001688	KCNQ5
Successfu	12	1.10864457957124e-08	0.46654748016659	0.658	0.466	0.000371817219096602	CREBRF
Successfu	12	1.27425382989909e-08	0.42636730998653	0.928	0.843	0.000427359249471557	MT-ND4L
Successfu	12	1.29201925108449e-08	0.97446606668122	0.421	0.229	0.000433317416428718	RASSF6
Successfu	12	1.40228106435574e-08	0.65775042682961	0.349	0.17	0.00047029702336362	PIK3AP1
Successfu	12	1.42375431499843e-08	0.52270108144116	0.533	0.338	0.000477498722164173	COL4A4
Successfu	12	1.44182963273926e-08	0.59831999433656	0.737	0.544	0.000483560822228092	PTPRC
Successfu	12	1.45261031466645e-08	0.43851266085179	0.842	0.69	0.000487176447332834	ITPR2
Successfu	12	1.45670446706427e-08	0.47712918541045	0.711	0.516	0.000488549544164015	WNK1
Successfu	12	1.47250818552334e-08	0.50032248254353	0.474	0.269	0.000493849795260817	PCED1B
Successfu	12	1.68287899402718e-08	0.39856245689570	0.908	0.756	0.000564403957016835	ANKRD12
Successfu	12	1.68445970255012e-08	0.45958196632764	0.711	0.526	0.000564934095041258	PCM1
Successfu	12	1.7857040024608e-08	0.41734217725724	0.836	0.69	0.000598889408345303	USP34
Successfu	12	1.82920042112712e-08	0.46538445222632	0.704	0.505	0.000613477237237614	RNGTT
Successfu	12	1.84097119564092e-08	0.46057070420207	0.75	0.568	0.000617424919594051	EPB41
Successfu	12	1.86392988748507e-08	0.49172588509047	0.572	0.376	0.000625124805664743	MTFR1
Successfu	12	2.00717350732336e-08	0.52760069946047	0.658	0.448	0.00067316585088611	PPP1R16B
Successfu	12	2.17352754091004e-08	0.69068221973948	0.592	0.397	0.000728957666670409	ANK3
Successfu	12	2.19233911687805e-08	0.32726924611087	0.507	0.271	0.000735266693018559	CD52

Successfu	12	2.23886752113088e-08	0.784828359141393	0.605	0.42	0.000750871389236873	TRAK1
Successfu	12	2.25004870628562e-08	0.386715790323078	0.908	0.764	0.00075462133511407	BCL2
Successfu	12	2.41838417139279e-08	0.571595159997203	0.842	0.706	0.000811077683401714	PRDM2
Successfu	12	2.46421163717831e-08	0.51936252745632	0.737	0.567	0.000826447298876863	SPIDR
Successfu	12	2.49691279692144e-08	0.393064856293825	0.829	0.633	0.000837414613831512	ATF7IP
Successfu	12	2.49882336444274e-08	0.334031655414394	0.289	0.126	0.000838055379966806	TCOF1
Successfu	12	2.55473466671081e-08	0.724463793934412	0.75	0.585	0.00085680691252147	HDAC9
Successfu	12	2.55809461068514e-08	0.58597818157074	0.632	0.439	0.000857933770531582	FLI1
Successfu	12	2.7533467797425e-08	0.819514490296466	0.599	0.397	0.00092341744299004	REL
Successfu	12	2.78562769622792e-08	0.489445786255119	0.316	0.144	0.000934243816760921	TFEC
Successfu	12	2.8996392605801e-08	0.434441661265435	0.586	0.383	0.000972481015213355	SPATA5
Successfu	12	3.09792594598702e-08	0.429124140524326	0.763	0.581	0.00103898240376513	SEPT6
Successfu	12	3.10885109915567e-08	0.482478789861321	0.474	0.293	0.00104264648163483	P4HA1
Successfu	12	3.17373255823287e-08	0.454535838929197	0.645	0.447	0.00106440642538014	NT5DC1
Successfu	12	3.18091181801819e-08	0.607028384744686	0.651	0.463	0.00106681420552694	FAM49A
Successfu	12	3.2286825560397e-08	0.520566205855548	0.684	0.534	0.0010828355556446	PTPN2
Successfu	12	3.33446038622771e-08	0.491708171923386	0.75	0.58	0.00111831132433305	SENP6
Successfu	12	3.36519813430288e-08	0.550733793996946	0.684	0.508	0.0011286201502825	ITGA4
Successfu	12	4.11836437498359e-08	0.340836943441202	0.658	0.468	0.001381217044082	HNRNPF
Successfu	12	4.26956104228972e-08	1.05475949240275	0.855	0.699	0.00143192538236313	UBE2H
Successfu	12	4.54963661790725e-08	0.555623704263934	0.592	0.407	0.00152585712891373	CCM2
Successfu	12	4.56313415358186e-08	0.588282551819142	0.414	0.226	0.00153038393242829	VAV3
Successfu	12	4.87262513509679e-08	0.358061031758248	0.52	0.317	0.00163418101780876	UBAC2
Successfu	12	4.89979579604358e-08	0.551594074869348	0.27	0.116	0.00164329351407709	C12orf42
Successfu	12	4.90899572190379e-08	0.319398427267048	0.467	0.269	0.00164637898521209	TMPO
Successfu	12	5.152513350322e-08	0.861217535461914	0.566	0.393	0.00172804992743099	AC120193.1
Successfu	12	5.3802857698291e-08	0.468677097248937	0.487	0.293	0.00180444024148528	ITPR1
Successfu	12	5.68972891709605e-08	0.858969097695477	0.678	0.494	0.00190822128421567	INTS6
Successfu	12	5.70530950949783e-08	0.69344147695764	0.618	0.47	0.00191344670329538	SUPT3H
Successfu	12	6.87847817061041e-08	0.592674310648963	0.539	0.353	0.00230690400885932	PRKN
Successfu	12	7.27298711340435e-08	0.338812794753275	0.875	0.715	0.00243921441809355	KDM4C

Successfu	12	7.37045533611442e-08	0.34071587578558€	0.789	0.651	0.00247190331062605	PAFAH1B1
Successfu	12	7.4212779806976e-08	0.43069571824622€	0.776	0.596	0.00248894820916636	SCAF11
Successfu	12	7.46634494833604e-08	0.54411235991059	0.428	0.221	0.00250406276877294	HLA-DQA1
Successfu	12	7.9671899329314e-08	0.27444259465105	0.526	0.341	0.00267203615970653	CAPRIN1
Successfu	12	8.05986731261417e-08	1.10252000836184	0.579	0.412	0.00270311829930454	LY9
Successfu	12	8.14792391334719e-08	0.43351385863182	0.618	0.433	0.00273265072205838	AFTPH
Successfu	12	8.29677649474942e-08	0.414657624803111	0.763	0.592	0.00278257290080906	LINC01004
Successfu	12	9.15357157839144e-08	0.34021758062937	0.671	0.482	0.00306992483596092	BLNK
Successfu	12	9.17022773356978e-08	0.30701000031351	0.921	0.773	0.00307551097728463	DDX17
Successfu	12	9.40466873624808e-08	0.40751028003075	0.822	0.651	0.00315413780076288	SOS1
Successfu	12	9.47147767824179e-08	0.50131513522495	0.553	0.381	0.00317654418372873	R3HDM1
Successfu	12	9.5340731110419e-08	0.45442949675900€	0.967	0.833	0.00319753743998123	EZR
Successfu	12	9.79285421228753e-08	0.54784586582911€	0.401	0.213	0.00328432744571699	VPREB3
Successfu	12	9.82448071936251e-08	0.27716471902008	0.836	0.655	0.0032949343436598	DYRK1A
Successfu	12	1.04683623878061e-07	0.52082637731072	0.789	0.674	0.00351087937762242	PPP1R10
Successfu	12	1.06734378828488e-07	0.27881779237477	0.145	0.041	0.00357965759714982	EMILIN2
Successfu	12	1.06825663523405e-07	0.57119646589627	0.717	0.526	0.00358271910324797	RAD51B
Successfu	12	1.11211127359826e-07	0.57347657584523	0.783	0.608	0.00372979878939385	SIPA1L1
Successfu	12	1.13036417630446e-07	0.43521962588664	0.375	0.206	0.00379101537448989	SLC39A10
Successfu	12	1.17719870658326e-07	0.42845642173164	0.974	0.89	0.00394808902213894	JUND
Successfu	12	1.2026694481487e-07	0.34225682852300	0.816	0.657	0.00403351279520111	RERE
Successfu	12	1.22317213909506e-07	0.38394072005192	0.592	0.426	0.00410227472009703	SMARCA2
Successfu	12	1.23913353371717e-07	0.42947535062583	0.48	0.307	0.00415580604538064	MTMR6
Successfu	12	1.36084659674423e-07	0.38446339338050	0.375	0.188	0.00456400731616078	RILPL2
Successfu	12	1.36643474275039e-07	0.41714378786082	0.546	0.363	0.00458274884023625	RAB21
Successfu	12	1.44552056657119e-07	0.59105339822028	0.75	0.614	0.00484798687616647	UBE2E2
Successfu	12	1.44652352715651e-07	0.37198346979484	0.493	0.308	0.00485135060537751	LRMP
Successfu	12	1.45559971924914e-07	0.56004960835737€	0.428	0.249	0.00488179033841777	PIK3CG
Successfu	12	1.48192101204366e-07	0.45590705775746€	0.783	0.629	0.00497006669019201	SFPQ
Successfu	12	1.51196586362126e-07	0.32600356244447	0.763	0.6	0.00507083111341298	CUL3
Successfu	12	1.52290977819607e-07	0.74012349057271	0.862	0.712	0.00510753481411398	HSPA8

Successfu	12	1.64907125173874e-07	0.46961938548848	0.467	0.27	0.00553065516408139	CARD11
Successfu	12	1.66717805858229e-07	0.53294095735577	0.553	0.381	0.00559138177287327	ACBD6
Successfu	12	1.7099349954505e-07	0.45011843224233	0.822	0.646	0.00573477998774189	MAML2
Successfu	12	1.77344215718202e-07	0.34498502003958	0.697	0.522	0.00594777030675707	TENT2
Successfu	12	1.88818726575791e-07	0.50302089021017	0.796	0.668	0.00633260245189887	BBX
Successfu	12	1.91332802219312e-07	0.47446824412799	0.743	0.566	0.00641691952083129	TNKS
Successfu	12	2.04990460675314e-07	0.48538527720699	0.796	0.679	0.00687497007012869	RSRP1
Successfu	12	2.07362309488487e-07	0.42387407338190	0.763	0.571	0.00695451713562489	ADK
Successfu	12	2.09578846004852e-07	0.40601012820524	0.559	0.384	0.00702885533731072	KPNA1
Successfu	12	2.1357748354596e-07	0.68747693257936	0.638	0.476	0.0071629616431644	CHORDC1
Successfu	12	2.1564686231905e-07	0.45644250588382	0.717	0.588	0.00723236446845629	ACTR3
Successfu	12	2.2106439499523e-07	0.30396613939579	0.211	0.08	0.00741405767935004	CD244
Successfu	12	2.21439635284871e-07	0.47719120962744	0.507	0.329	0.007426642488184	POU2F2
Successfu	12	2.28594292350737e-07	0.44606055354711	0.546	0.365	0.007666595376859	CHIC2
Successfu	12	2.37478504501019e-07	0.73326584462971	0.52	0.333	0.00796455408395516	CXCR4
Successfu	12	2.39165966479526e-07	0.27499712309238	0.599	0.408	0.00802114818379035	LNPEP
Successfu	12	2.60561594356121e-07	0.46376893157083	0.763	0.632	0.0087387147515156	BCLAF1
Successfu	12	2.7106228301521e-07	0.29106912365055	0.237	0.096	0.0090908868477641	SNX10
Successfu	12	2.80094771080374e-07	0.52933493798695	0.888	0.794	0.00939381843249359	DUSP1
Successfu	12	2.88548616378151e-07	0.75537556276091	0.513	0.356	0.00967734349609041	TMEM65
Successfu	12	2.9239763557264e-07	0.30745563008361	0.138	0.04	0.00980643190183521	FAM177B
Successfu	12	2.93225109853963e-07	0.44788318625883	0.941	0.825	0.00983418373428222	HSP90AB1
Successfu	12	3.11820582592941e-07	0.28765160560946	0.105	0.024	0.0104578386990021	LINC01811
Successfu	12	3.14950259673768e-07	0.38716512337551	0.401	0.229	0.0105628018089388	GSAP
Successfu	12	3.52560541568297e-07	0.77716648589579	0.842	0.682	0.0118241754431175	ST6GAL1
Successfu	12	3.63865724730824e-07	0.49218976779502	0.809	0.698	0.0122033286760224	STK4
Successfu	12	3.72853074049201e-07	0.48589074097252	0.901	0.804	0.0125047463974621	YWHAZ
Successfu	12	3.76618144813575e-07	0.33917434084173	0.822	0.67	0.0126310193407577	PTGES3
Successfu	12	3.81026285436991e-07	0.57540619143728	0.638	0.472	0.0127788595609858	SKIL
Successfu	12	3.82001183119355e-07	0.30604513164751	0.309	0.149	0.0128115556794569	NFATC1
Successfu	12	3.82221553919608e-07	0.39868891464200	0.743	0.584	0.0128189464753558	ZFAND6

Successfu	12	4.00345653836212e-07	0.29736556517372	0.487	0.292	0.0134267925383589	PRKCE
Successfu	12	4.00600140821961e-07	0.34190324628757	0.25	0.113	0.0134353275228869	AC097662.1
Successfu	12	4.06301519591226e-07	0.33815282648211	0.599	0.444	0.0136265403640505	MAPKAP1
Successfu	12	4.16165062163654e-07	0.33458807176418	0.691	0.521	0.0139573438548446	SETD2
Successfu	12	4.16961424592102e-07	0.47104846433265	0.803	0.675	0.0139840522579699	CD44
Successfu	12	4.31385849344744e-07	0.38258936902992	0.651	0.469	0.014467818615324	SNX25
Successfu	12	4.33615100709167e-07	0.34187096463392	0.875	0.76	0.0145425832475841	FNBP1
Successfu	12	4.42900025141237e-07	0.36637884787573	0.816	0.634	0.0148539810431868	RAB2A
Successfu	12	4.57472415438859e-07	0.27539924188924	0.493	0.313	0.0153427098689885	GGA2
Successfu	12	4.72781436611446e-07	0.34785059390931	0.612	0.419	0.0158561438210747	DIP2B
Successfu	12	4.76726639820677e-07	0.463158111344494	0.757	0.601	0.0159884580463059	ASCC3
Successfu	12	4.88050812833711e-07	0.36685203689021	0.276	0.129	0.016368248160817	BCAR3
Successfu	12	4.91717001692735e-07	0.70041615003131	0.401	0.236	0.0164912048027709	SNED1
Successfu	12	4.95433820117739e-07	0.53134682557572	0.822	0.691	0.0166158594591087	FBXL17
Successfu	12	5.16796703806516e-07	0.28661326049242	0.789	0.603	0.0173323278522629	NCOR1
Successfu	12	5.17464276322926e-07	0.25810474385829	0.197	0.072	0.0173547168993183	FAM129C
Successfu	12	5.19410742092543e-07	0.35408339812883	0.796	0.653	0.0174199974682997	DOCK2
Successfu	12	5.29001633727388e-07	0.3359984512869	0.375	0.192	0.0177416567919491	ABHD15-AS1
Successfu	12	5.31771895634959e-07	0.34230601442303	0.822	0.672	0.0178345658358052	PRRC2C
Successfu	12	5.46679070157072e-07	0.42765808888547	0.816	0.643	0.0183345226549279	MARCH6
Successfu	12	5.65001149912217e-07	0.52934829289713	0.737	0.556	0.0189490085657559	ZEB1
Successfu	12	5.78281649875972e-07	0.32627215602107	0.671	0.492	0.0193944099735403	BRD4
Successfu	12	5.87278570996873e-07	0.35494736364072	0.257	0.118	0.0196961487140931	RGS19
Successfu	12	6.01303104423965e-07	0.46770230492268	0.77	0.606	0.020166503516171	IKZF3
Successfu	12	6.10473026642885e-07	0.32227292842228	0.658	0.482	0.0204740443675491	TAF7
Successfu	12	6.13078032027085e-07	0.28422755374553	0.158	0.051	0.0205614110381244	CXCR5
Successfu	12	6.24757846166726e-07	0.30038659094224	0.842	0.672	0.0209531286447397	VPS13B
Successfu	12	6.38938042923598e-07	0.51494986531015	0.632	0.464	0.0214287040835716	LRRFIP1
Successfu	12	6.45070695712733e-07	0.27234367362284	0.513	0.333	0.0216343809928136	MICAL3
Successfu	12	6.77389840823777e-07	0.41935522035141	0.724	0.571	0.0227183004815478	TUT4
Successfu	12	6.90169960434391e-07	0.72674729118705	0.862	0.75	0.0231469201330486	PPP1R15A

Successfu	12	7.07070354107115e-07	0.467627097437082	0.362	0.19	0.0237137255360444	TNFRSF13C
Successfu	12	7.33265437337653e-07	0.337250283782906	0.882	0.754	0.0245922562374302	GPBP1
Successfu	12	7.48060486938986e-07	0.626386969830085	0.73	0.598	0.0250884526109597	CD55
Successfu	12	7.81287819996666e-07	0.43681550955967	0.493	0.315	0.0262028309070482	PTK2
Successfu	12	8.04927234077535e-07	0.396832867077078	0.428	0.253	0.0269956495764924	NUP88
Successfu	12	8.31107474623171e-07	0.340149035144351	0.454	0.289	0.0278736824839119	PSIP1
Successfu	12	8.47181389034714e-07	0.255138352597134	0.158	0.054	0.0284127694254462	LYL1
Successfu	12	8.66550291504391e-07	0.421505701294216	0.572	0.418	0.0290623636764743	MOB1A
Successfu	12	8.71445186710588e-07	0.769891975368911	0.849	0.716	0.0292265286718997	DNAJB1
Successfu	12	8.82227372836741e-07	0.537788458047119	0.224	0.093	0.0295881416301986	CR1
Successfu	12	8.96901897943424e-07	0.487150205163274	0.664	0.514	0.0300802958532266	CYLD
Successfu	12	9.05322214762398e-07	0.423162196861357	0.651	0.48	0.0303626964387013	PTEN
Successfu	12	9.1012116374793e-07	0.376056956253016	0.605	0.458	0.0305236435897781	TRANK1
Successfu	12	9.1208140206327e-07	0.324622614994761	0.829	0.653	0.030589386062398	USP3
Successfu	12	1.0037776452302e-06	0.326967811895749	0.461	0.286	0.0336646946657305	C2CD5
Successfu	12	1.02459839362264e-06	0.380781644958855	0.73	0.602	0.0343629809253161	ZNF638
Successfu	12	1.04030202869828e-06	0.290961729628877	0.289	0.141	0.0348896494384831	L3MBTL3
Successfu	12	1.04436902617074e-06	0.39575406033174	0.691	0.529	0.0350260483997144	NEK7
Successfu	12	1.05719071476829e-06	0.505812272648554	0.645	0.464	0.0354560621918991	SKAP2
Successfu	12	1.058609610268e-06	0.306035760932003	0.368	0.209	0.0355036491091683	HIST1H1E
Successfu	12	1.06454031084451e-06	0.670998278727765	0.789	0.675	0.0357025529451031	SSH2
Successfu	12	1.08705792439741e-06	0.345560040278909	0.934	0.821	0.0364577486684405	RBM39
Successfu	12	1.10676593323066e-06	0.513431058385169	0.651	0.507	0.03711871586869	PHF20
Successfu	12	1.15901629213089e-06	0.454851456001117	0.243	0.11	0.0388710884054857	AC024145.1
Successfu	12	1.20264534897713e-06	0.261901553690544	0.724	0.56	0.0403343197139951	FUBP1
Successfu	12	1.23398057818452e-06	1.29656677959728	0.421	0.281	0.0413852406311524	AUTS2
Successfu	12	1.26045623295174e-06	0.366542076614514	0.612	0.463	0.0422731811407354	EP300
Successfu	12	1.28152485467905e-06	0.375657770341535	0.566	0.415	0.042979780576226	TRIM44
Successfu	12	1.31073773238009e-06	0.33516364256192	0.355	0.19	0.0439595220685634	WDFY4
Successfu	12	1.33345048985339e-06	0.30908044220514	0.164	0.057	0.0447212625287031	GAPT
Successfu	12	1.35876132159634e-06	0.627179389435392	0.658	0.508	0.045570137203698	REV3L

Successfu	12	1.3930313770724e-06	0.35594126698970	0.763	0.627	0.0467194863242543	CHD9
Successfu	12	1.44398654642457e-06	0.37441375726985	0.704	0.538	0.0484284207939872	PPP3CC
Successfu	12	1.462418567389e-06	0.32675931186183	0.586	0.409	0.0490465939130922	ARHGDIB
Successfu	12	1.46549068641871e-06	0.43355227733255	0.507	0.341	0.0491496266411108	SINHCAF
Successfu	12	1.46872000532076e-06	0.53475825766701	0.592	0.445	0.0492579315384476	ACYP2
Successfu	12	1.47001512536364e-06	0.525599861189611	0.48	0.33	0.0493013672744458	SP110
Successfu	19	9.98897288071089e-28	0.62931203264055	0.327	0.058	3.35010172473282e-23	C1QB
Successfu	19	1.21658039503154e-17	0.54854341602969	0.961	0.866	4.08016732885677e-13	TMSB10
Successfu	19	2.63594556532563e-16	0.32501895158279	0.163	0.023	8.84043423698911e-12	C1QC
Successfu	19	1.42687808623286e-15	0.53352498635902	0.229	0.051	4.78546372560776e-11	C1QA
Successfu	19	2.78435117868262e-15	0.72588229543073	0.895	0.611	9.33815698306578e-11	MT1G
Successfu	19	6.57612985136912e-14	1.19785553071724	0.68	0.518	2.20550242955218e-09	LGALS1
Successfu	19	4.54851183709048e-13	0.57210666416420	0.614	0.372	1.52547989992341e-08	ATP5MF
Successfu	19	8.16346422189225e-13	0.79891349497783	0.778	0.623	2.73786263073822e-08	PPIB
Successfu	19	2.21057306740674e-12	0.51138310549713	0.614	0.357	7.41381995346873e-08	MT1H
Successfu	19	2.91315002942137e-12	0.46422679122786	0.412	0.185	9.77012256867339e-08	FABP5
Successfu	19	5.69498328362388e-12	0.845411971134778	0.817	0.732	1.90998349366178e-07	CYBA
Successfu	19	6.49025126534223e-12	0.87445617428172	0.712	0.544	2.17670046937048e-07	FKBP2
Successfu	19	7.60966866435344e-12	0.615931119717286	0.837	0.786	2.55213067665086e-07	SERF2
Successfu	19	1.22657582090552e-11	0.56310272999854	0.856	0.753	4.11368998815292e-07	SUB1
Successfu	19	1.37756838658075e-11	0.61995104511775	0.595	0.401	4.62008885491451e-07	PSME2
Successfu	19	1.51111350420551e-11	0.80511745318248	0.758	0.635	5.06797247040444e-07	SEC61B
Successfu	19	4.78591010164822e-11	0.52046465657778	0.81	0.688	1.60509852989078e-06	ATP5MG
Successfu	19	1.00316709096936e-10	0.55771218607879	0.627	0.441	3.36442178969304e-06	KRT10
Successfu	19	1.50216625161454e-10	0.63137905931080	0.523	0.318	5.03796517466485e-06	TIMP1
Successfu	19	1.50321738552285e-10	0.55577231290782	0.575	0.39	5.04149046756654e-06	ATP5MC1
Successfu	19	1.71275506751707e-10	0.71810285123362	0.719	0.589	5.74423794543875e-06	CD63
Successfu	19	1.95649639965545e-10	0.95502905247017	0.732	0.614	6.56169762516445e-06	MZB1
Successfu	19	2.18467247819153e-10	0.46506661538378	0.824	0.708	7.32695455735874e-06	ATP5F1E
Successfu	19	6.77703377965591e-10	0.78903903637651	0.699	0.576	2.272881589021e-05	TMEM258
Successfu	19	1.10043076065227e-09	0.77561416924690	0.647	0.506	3.69062468507557e-05	RABAC1

Successfu	19	1.17627073088627e-09	0.83579488931283	0.817	0.729	3.94497677724638e-05	SSR4
Successfu	19	1.93231764860996e-09	0.46253096266893	0.562	0.372	6.48060692990809e-05	IL2RG
Successfu	19	3.48603976749743e-09	0.38276872808929	0.529	0.341	0.000116914801722329	PSMB3
Successfu	19	4.31950433916437e-09	0.28936671920713	0.131	0.029	0.000144867536526894	CCL2
Successfu	19	5.31847620662747e-09	0.55894401745403	0.98	0.956	0.000178371055017872	B2M
Successfu	19	6.6645898602916e-09	0.578840108771118	0.131	0.03	0.00022351701473446	APOE
Successfu	19	1.04444190625004e-08	0.52325488058925	0.797	0.712	0.000350284926518139	RPL36AL
Successfu	19	1.25041642268527e-08	0.48096711308352	0.68	0.56	0.000419364659840187	TXN
Successfu	19	1.68756431521897e-08	0.33344199222349	0.928	0.888	0.000565975320038139	RPS19
Successfu	19	1.75337746193374e-08	0.40279467251360	0.673	0.538	0.000588047733183339	COX5B
Successfu	19	1.77003292012233e-08	0.49540441878650	0.588	0.424	0.000593633640750627	EMP3
Successfu	19	2.15629080090312e-08	0.39612591045363	0.647	0.533	0.000723176808806889	ATP5PF
Successfu	19	2.49498465878867e-08	0.44084008303908	0.948	0.929	0.000836767954864546	RPLP1
Successfu	19	6.9624766513676e-08	0.44012112293627	0.549	0.363	0.00233507541933567	UQCR11
Successfu	19	7.0336625992555e-08	0.62227235622148	0.719	0.572	0.00235894976253831	SPCS1
Successfu	19	8.71437433857058e-08	0.421711011863761	0.601	0.432	0.0029226268656698	SH3BGRL3
Successfu	19	1.09814517119079e-07	0.37926932806288	0.902	0.809	0.00368295927513969	RPS15
Successfu	19	1.22940707754328e-07	0.51949140686477	0.647	0.527	0.00412318545666467	UBL5
Successfu	19	1.27599191688026e-07	0.34154187289037	0.778	0.638	0.00427942169083302	SRP14
Successfu	19	1.28254718791769e-07	0.41699870228619	0.745	0.604	0.00430140675883836	MT2A
Successfu	19	1.35133452777343e-07	0.45011528312853	0.66	0.564	0.00453210573924651	COX6B1
Successfu	19	1.76605352467736e-07	0.49543807770928	0.706	0.621	0.00592299031106294	RPS27L
Successfu	19	1.99857590430688e-07	0.63114313617075	0.614	0.46	0.0067028238678644	TNFRSF17
Successfu	19	2.0511342057155e-07	0.38261304487592	0.922	0.761	0.00687909389912863	NACA
Successfu	19	2.11160764900011e-07	0.33504833900198	0.804	0.74	0.00708190973321657	MYL6
Successfu	19	2.15395140702518e-07	0.82287004117096	0.817	0.673	0.00722392222888105	NFKBIA
Successfu	19	2.34651611456875e-07	0.57506926791673	0.405	0.236	0.00786974574504067	AGR2
Successfu	19	2.49139040591807e-07	0.71754120890652	0.634	0.482	0.00835562514336803	DERL3
Successfu	19	2.54021786469445e-07	0.45298482483833	0.732	0.556	0.00851938267461226	BIRC3
Successfu	19	2.99081405129928e-07	0.40872297738192	0.654	0.507	0.0100305921652475	SEC61G
Successfu	19	3.01278553495485e-07	0.37817145265086	0.379	0.212	0.0101042801271316	ISG15

Successfu	19	3.42032734653138e-07	0.416304654631934	0.634	0.477	0.0114710938547969	GSTP1
Successfu	19	3.46136528523669e-07	0.555786134269211	0.941	0.895	0.0116087268936268	JUND
Successfu	19	3.56137640660893e-07	0.520858720089122	0.49	0.349	0.011944144192485	BST2
Successfu	19	4.16921005938021e-07	0.429472928563924	0.137	0.041	0.0139826966971493	CFD
Successfu	19	4.90346365968728e-07	0.509926738235907	0.778	0.703	0.0164452364218592	S100A6
Successfu	19	9.09068398771723e-07	0.359660468667429	0.405	0.26	0.030488335958006	IFI27L2
Successfu	19	9.57206223975176e-07	0.412840658603884	0.575	0.44	0.0321027823396795	SEM1
Successfu	19	9.7898251672275e-07	0.531086938876337	0.464	0.314	0.0328331156458476	NOP10
Successfu	19	1.0021753587699e-06	0.341765535670604	0.556	0.418	0.0336109571824249	ATOX1
Successfu	19	1.02096649128873e-06	0.391875743410388	0.288	0.141	0.0342411741848413	MT1M
Successfu	19	1.08684617898909e-06	0.429780359597711	0.523	0.386	0.0364506471509362	PHPT1
Successfu	19	1.47291257585353e-06	0.562411818144424	0.948	0.899	0.0493985419689757	FOS
Successfu	22	5.79845595299789e-12	0.441148726449958	0.185	0.013	1.94468615751643e-07	S100A8
Successfu	22	7.39160557038458e-12	0.60415982828998	0.259	0.027	2.47899667619558e-07	TMEM72-AS1
Successfu	22	6.52918393493848e-11	1.13649401864935	0.222	0.021	2.18975770809967e-06	IGKV1-16
Successfu	22	2.61589287126022e-09	0.31503272962477	0.148	0.011	8.77318151163252e-05	AL161937.2
Successfu	22	1.13372433617254e-08	0.398923228150744	0.148	0.012	0.000380228467865548	AL356272.1
Successfu	22	6.72061738966879e-07	0.624714530851902	0.333	0.075	0.0225396066014712	AC079305.3
Successfu	22	1.1666336815061e-06	0.467417740551548	0.111	0.01	0.0391265604103517	AC084026.2
Successfu	22	1.4608241669484e-06	0.896521938850083	0.333	0.082	0.0489931209111153	AC096677.1
Successfu	26	1.7125409037447e-39	0.568532141417771	0.462	0.06	5.74351968297898e-35	MT1B
Successfu	26	5.59019576930651e-34	1.29622039981982	1	0.989	1.87483985711002e-29	MT-ND3
Successfu	26	1.12637591453033e-31	1.06632009231071	0.875	0.344	3.77763954215181e-27	MT1H
Successfu	26	3.76126889919182e-28	1.11363980081283	0.99	0.615	1.26145436341095e-23	MT1G
Successfu	26	1.33957704257914e-24	1.0352173865049	0.99	0.696	4.4926734854019e-20	MT-ATP8
Successfu	26	5.03338442426503e-23	1.28052116026311	0.846	0.423	1.68809646821001e-18	SESN1
Successfu	26	1.85801258602319e-22	0.583263880144319	0.481	0.116	6.23140261100457e-18	CECR2
Successfu	26	1.18667396848256e-21	0.906535139183354	1	0.994	3.97986715549682e-17	MT-ND2
Successfu	26	2.88747990920889e-20	0.371399605551887	0.481	0.127	9.68403011950478e-16	AC008467.1
Successfu	26	3.42882948254256e-20	0.481817721298053	0.548	0.172	1.14996083185512e-15	AC092683.1
Successfu	26	8.37305975291715e-20	0.496097889366196	0.596	0.193	2.80815677993336e-15	TET1

Successfu	26	2.41694861189158e-19	1.22588486965241	0.885	0.505	8.10596225456197e-15	IFNG-AS1
Successfu	26	2.98492055771529e-19	0.33802069209592	0.433	0.11	1.00108265664655e-14	KIF19
Successfu	26	7.01962515942275e-19	0.75342695626145	0.817	0.368	2.3542418859672e-14	WNT5B
Successfu	26	1.2883735943308e-18	0.73226358569572	0.798	0.383	4.32094736066664e-14	ENAM
Successfu	26	1.39860943198099e-18	0.54237226109729	0.663	0.267	4.69065631297784e-14	TXNDC5
Successfu	26	1.53580192506902e-18	0.66818446486969	0.923	0.446	5.15077249629648e-14	MTRNR2L1
Successfu	26	1.78288044979341e-18	0.76863263765915	0.654	0.249	5.97942445251713e-14	AMPD1
Successfu	26	2.85054891330438e-17	0.81746836918596	0.663	0.286	9.56017094544023e-13	FAAH2
Successfu	26	2.89108050515624e-17	0.75197899124349	0.721	0.323	9.696105798193e-13	MCC
Successfu	26	3.20248586741209e-17	0.37093302214118	0.452	0.13	1.07404971021267e-12	PLEKHA8
Successfu	26	8.09525665335354e-17	0.55772517456161	0.788	0.366	2.71498717640171e-12	SLC15A2
Successfu	26	8.29981868510517e-17	0.36267905174913	0.452	0.132	2.78359319061057e-12	MT1M
Successfu	26	9.79026294729964e-17	0.65242944380028	0.798	0.383	3.28345838726535e-12	AC078883.1
Successfu	26	2.07613280229073e-16	0.29764017992673	0.279	0.055	6.96293419232264e-12	ABCA6
Successfu	26	2.13104285689178e-16	0.50833393529083	0.75	0.334	7.14709153344364e-12	ANKRD36C
Successfu	26	2.75988361323564e-16	0.87987229976549	0.808	0.431	9.25609766206969e-12	LINC02362
Successfu	26	5.28756578330409e-16	0.45035997534671	0.462	0.144	1.77334381240453e-11	HTR4
Successfu	26	1.9291943205809e-15	0.85595260804897	0.952	0.702	6.47013191236421e-11	MSI2
Successfu	26	2.94307503949213e-15	0.84972712585973	0.894	0.554	9.8704850674487e-11	SAMD12
Successfu	26	3.01968067543374e-15	0.83227931872149	0.587	0.246	1.01274050492697e-10	PLCL1
Successfu	26	3.25242910350719e-15	0.65600306002185	0.942	0.673	1.09079967273424e-10	MYO1D
Successfu	26	4.47052035918455e-15	1.02013718683759	0.933	0.708	1.49932311806331e-10	FNDC3B
Successfu	26	5.32199810265792e-15	0.47426973374942	0.74	0.325	1.78489172366941e-10	MT1E
Successfu	26	6.05998166509388e-15	0.53776550574460	0.779	0.412	2.03239665083919e-10	MANEA
Successfu	26	6.08753601816574e-15	0.74374797544050	0.529	0.207	2.04163782977243e-10	AL078459.1
Successfu	26	6.72270739450599e-15	0.39762323696946	0.654	0.279	2.25466160596942e-10	KCNN3
Successfu	26	6.77765949877296e-15	0.81450959748451	0.846	0.468	2.27309144269847e-10	RASGRP3
Successfu	26	7.9349184619725e-15	0.75121497348716	0.875	0.499	2.66121295377634e-10	LINC01934
Successfu	26	8.05960856099072e-15	0.68226606035572	0.933	0.602	2.70303151918507e-10	CPEB4
Successfu	26	1.42493255804974e-14	0.83745504419549	0.942	0.756	4.77893881318722e-10	FNDC3A
Successfu	26	1.8456951087763e-14	0.27294642763115	0.269	0.059	6.19009225581395e-10	CXXC4

Successfu	26	2.4922403160162e-14	0.774813703911077	0.904	0.584	8.35847557185513e-10	TXNDC11
Successfu	26	2.49537493011771e-14	0.650105835121399	0.875	0.5	8.36898844062877e-10	MYO5A
Successfu	26	3.0327389236353e-14	0.42409957301289	0.462	0.151	1.01711998020881e-09	EFCAB13
Successfu	26	3.13330818577041e-14	0.534768898475718	0.548	0.21	1.05084889934368e-09	CRIP1
Successfu	26	3.61080231337337e-14	0.816660572320788	0.952	0.805	1.21099087985916e-09	FBXW7
Successfu	26	4.39207883871804e-14	0.455911177692737	0.567	0.226	1.47301540092926e-09	DAPK1
Successfu	26	4.47312047768993e-14	0.846540155497554	0.894	0.608	1.50019514580765e-09	XBP1
Successfu	26	4.4830531094141e-14	0.414722964940439	0.798	0.368	1.5035263518353e-09	TIAM2
Successfu	26	6.36196988140838e-14	0.457719031840484	0.519	0.199	2.13367745882674e-09	AC060809.1
Successfu	26	1.09730120004429e-13	0.830164542156445	0.923	0.697	3.68012876470853e-09	CDK14
Successfu	26	1.12853932302027e-13	0.827010094125773	0.615	0.269	3.78489518154537e-09	CHODL
Successfu	26	1.3068744084388e-13	0.410029888912161	0.567	0.219	4.38299539102204e-09	MT1F
Successfu	26	1.65242054776735e-13	0.299122288889932	0.327	0.089	5.54188803310214e-09	WDR64
Successfu	26	1.71463402900111e-13	0.535391533574215	0.413	0.134	5.75053960646391e-09	TSHR
Successfu	26	1.8385202787101e-13	0.47379482957795	0.837	0.424	6.16602931073795e-09	MT-ND6
Successfu	26	2.06621432143265e-13	0.52194562287541	0.913	0.594	6.92966959122082e-09	MT2A
Successfu	26	2.4420264203859e-13	0.663184256241472	0.769	0.416	8.19006820869025e-09	RAPGEF5
Successfu	26	2.4956317605087e-13	0.751378644760241	0.933	0.718	8.36984979839407e-09	DENND5B
Successfu	26	2.63357591371219e-13	0.344708022686789	0.423	0.141	8.83248689940795e-09	GAS6
Successfu	26	2.64030829331874e-13	0.686102019848665	0.885	0.579	8.85506595413239e-09	HDAC9
Successfu	26	2.78893279999276e-13	0.521481135931373	0.385	0.12	9.35352282461572e-09	AC104248.1
Successfu	26	3.81620771099143e-13	0.611644204667293	0.625	0.282	1.27987974211231e-08	ITGA8
Successfu	26	3.84893575208872e-13	0.502197518615322	0.394	0.127	1.29085607253551e-08	ARMC2
Successfu	26	3.88429574034973e-13	0.946592258594546	0.875	0.58	1.30271510539849e-08	CLIC4
Successfu	26	5.21882088304807e-13	0.383120899599749	0.673	0.305	1.75028814775666e-08	CRYBG3
Successfu	26	5.28341259617817e-13	0.292537569585605	0.529	0.198	1.77195091650624e-08	SLC7A7
Successfu	26	5.52054744996487e-13	0.414848978416563	0.769	0.4	1.85148120376922e-08	FBH1
Successfu	26	5.89946232114551e-13	0.804824219005938	0.913	0.648	1.97856167326578e-08	AC008014.1
Successfu	26	6.0659112346095e-13	0.800962949887047	0.865	0.542	2.03438530986333e-08	PDK1
Successfu	26	6.81682995263044e-13	0.34437939883455	0.846	0.418	2.2862284295132e-08	NPEPPS
Successfu	26	1.39152296341832e-12	0.497508248992629	0.337	0.098	4.66688971471237e-08	AF130417.1

Successfu	26	1.40209360837604e-12	0.673878824070906	0.75	0.401	4.70234154377158e-08	FER
Successfu	26	1.61062469405974e-12	0.497767457809737	0.769	0.401	5.40171309893754e-08	ANKRD36
Successfu	26	1.71681066148614e-12	0.583486487533746	0.75	0.4	5.75783959649222e-08	CCM2
Successfu	26	1.77110616531614e-12	0.610078432366577	0.529	0.216	5.93993585723728e-08	HIST1H2BC
Successfu	26	1.91667092124787e-12	0.673206910418657	0.625	0.303	6.42813093568112e-08	KAZN
Successfu	26	1.94791625843898e-12	0.43436908195625	0.375	0.12	6.53292154755266e-08	AC007262.2
Successfu	26	1.9574093543036e-12	0.363686655615337	0.5	0.189	6.56475949246341e-08	GLDC
Successfu	26	2.08424395336241e-12	0.349524196910357	0.365	0.114	6.99013737078686e-08	GPLD1
Successfu	26	2.16953602168703e-12	0.420852143090357	0.442	0.161	7.27618990953395e-08	FRMD6
Successfu	26	2.23155573970561e-12	0.607216197523098	0.567	0.25	7.48419163982469e-08	AC104123.1
Successfu	26	3.03042862845217e-12	0.258665232865507	0.462	0.165	1.01634515341029e-07	KMT2E-AS1
Successfu	26	3.04781394735862e-12	0.741461183380958	0.894	0.558	1.02217584166514e-07	EAF2
Successfu	26	3.35770036726994e-12	0.278297923082621	0.538	0.211	1.12610554917499e-07	RETREG3
Successfu	26	3.55106735650534e-12	0.503341731864306	0.76	0.403	1.19095697002476e-07	RUFY3
Successfu	26	3.56370689722181e-12	0.369335223353921	0.625	0.277	1.19519601919025e-07	ACSS2
Successfu	26	3.58817062796521e-12	0.619082867902506	0.837	0.522	1.20340066520697e-07	FCRL5
Successfu	26	3.67714148930041e-12	0.381958727507362	0.558	0.243	1.23323971268157e-07	NXPE3
Successfu	26	5.43307676631668e-12	0.392423461870999	0.587	0.251	1.82214528588729e-07	AC023590.1
Successfu	26	5.89971151496888e-12	0.618020597628627	0.904	0.586	1.97864524789026e-07	SPATS2
Successfu	26	6.18298641646404e-12	0.527127384905748	0.779	0.447	2.07364998435371e-07	PIM2
Successfu	26	6.45870126286191e-12	0.369458217031589	0.692	0.315	2.16611922953863e-07	MT1X
Successfu	26	6.58271192177473e-12	0.406439208348705	0.654	0.307	2.20770992432481e-07	METTL7A
Successfu	26	7.38192690141023e-12	0.501994330867792	0.663	0.334	2.47575064419496e-07	TPTEP2-CSNK1E
Successfu	26	7.89859557163462e-12	0.454730980147076	0.596	0.279	2.64903098281482e-07	IGF1
Successfu	26	8.38753178267575e-12	0.614670460127187	0.952	0.676	2.81301040927379e-07	ATM
Successfu	26	8.5729996005683e-12	0.595011389514201	0.875	0.513	2.8752126060386e-07	SEL1L
Successfu	26	8.65734883415002e-12	0.512744020221082	0.827	0.458	2.90350165199723e-07	ASXL2
Successfu	26	9.20603153209378e-12	0.330904385025586	0.587	0.259	3.08751885523361e-07	TIMP2
Successfu	26	9.2907589614382e-12	0.435223659408309	0.558	0.25	3.11593474048714e-07	SMOC1
Successfu	26	9.52792494684912e-12	0.410401660012216	0.683	0.32	3.19547546867426e-07	ANKRD36B
Successfu	26	1.05595662036206e-11	0.802155025303576	0.769	0.412	3.54146731337027e-07	LINC02384

Successfu	26	1.25627011701733e-11	0.59745963130715	0.673	0.344	4.2132787184527e-07	TMTC2
Successfu	26	1.29493703045914e-11	0.70351114274085	0.894	0.591	4.34295981275386e-07	BCAS3
Successfu	26	1.49518010682397e-11	0.68365355010509	0.952	0.796	5.01453504226624e-07	ATXN1
Successfu	26	1.78022432023955e-11	0.71940714169688	0.942	0.804	5.97051632521939e-07	FOXN3
Successfu	26	1.8675590220388e-11	0.29414415281153	0.385	0.129	6.26341944811372e-07	APOLD1
Successfu	26	1.95217564232798e-11	0.28292922292433	0.308	0.09	6.54720666923959e-07	AL357153.2
Successfu	26	1.97050660422984e-11	0.28180265601869	0.404	0.144	6.60868504926604e-07	PTPRS
Successfu	26	2.37267922215736e-11	0.28557260983544	0.606	0.263	7.95749157527134e-07	AC139887.2
Successfu	26	2.38069461692265e-11	0.48603587337940	0.731	0.37	7.98437360623517e-07	ESR1
Successfu	26	2.84375015511525e-11	0.50002899300041	0.577	0.257	9.53736927022554e-07	AL360012.1
Successfu	26	3.22219587399529e-11	0.40371545921665	0.769	0.411	1.08066005222054e-06	MIR29B2CHG
Successfu	26	3.7993715465414e-11	0.33882899096836	0.404	0.142	1.27423322927906e-06	APLF
Successfu	26	4.07673162546846e-11	0.48221528967560	0.442	0.183	1.36725425254961e-06	HIST1H2BG
Successfu	26	5.21601285125468e-11	0.45696981353653	0.827	0.476	1.74934639005379e-06	C11orf80
Successfu	26	5.35234341194869e-11	0.54016933046334	0.923	0.671	1.79506893349935e-06	FAM214A
Successfu	26	6.30764878613418e-11	0.45330985650134	0.769	0.433	2.11545924989368e-06	IGLL5
Successfu	26	6.37696305969055e-11	0.27951917599740	0.356	0.116	2.13870587095902e-06	CTIF
Successfu	26	6.41488528281361e-11	0.26097314277892	0.519	0.216	2.15142422615003e-06	FBXL4
Successfu	26	6.69109386627671e-11	0.42214909337910	0.692	0.365	2.24405906087188e-06	PAPSS1
Successfu	26	7.66072128012068e-11	0.27307565605161	0.519	0.22	2.56925270292687e-06	GPR155
Successfu	26	8.16486087867382e-11	0.36069232535965	0.702	0.353	2.73833104148963e-06	PAM
Successfu	26	8.47775781565519e-11	0.29478422977263	0.788	0.431	2.84327041621444e-06	INSR
Successfu	26	8.4862845011378e-11	0.43806868898074	0.135	0.02	2.8461300959916e-06	AC137770.1
Successfu	26	9.19034845859687e-11	0.61878096226246	0.827	0.539	3.08225906604422e-06	FAM69A
Successfu	26	9.5377053387997e-11	0.47582233561256	0.913	0.658	3.19875561652664e-06	PCMTD1
Successfu	26	1.17724494593569e-10	0.38550271867510	0.615	0.282	3.94824409967911e-06	KNTC1
Successfu	26	1.2010996821123e-10	0.54172980701761	0.577	0.268	4.02824811386824e-06	COL24A1
Successfu	26	1.24300743848704e-10	0.50026911395901	0.942	0.647	4.16879834719785e-06	SOS1
Successfu	26	1.25683415924315e-10	0.55126812383582	0.837	0.519	4.21517040326967e-06	IQCB1
Successfu	26	1.28557994991679e-10	0.64159550114555	0.808	0.513	4.31157803603094e-06	MCPH1
Successfu	26	1.30514517179959e-10	0.29252569829105	0.596	0.26	4.37719587718146e-06	PIK3CA

Successfu	26	1.35694481082326e-10	0.506911921155388	0.769	0.422	4.55092150653905e-06	ST8SIA4
Successfu	26	1.36999963504646e-10	0.55217024433429[illegible]	0.798	0.469	4.59470477601881e-06	CHST15
Successfu	26	1.38289941340807e-10	0.43532011907309[illegible]	0.827	0.484	4.63796805268799e-06	ARFGEF2
Successfu	26	1.42617260978597e-10	0.31185916940486[illegible]	0.5	0.21	4.7830976987002e-06	QSER1
Successfu	26	1.45115513895266e-10	0.30790051514145[illegible]	0.712	0.344	4.86688410501945e-06	TMEM65
Successfu	26	1.52161422801695e-10	0.36656606912912[illegible]	0.856	0.519	5.10318979792325e-06	INPP4A
Successfu	26	1.62623468735474e-10	0.51945266057127[illegible]	0.894	0.598	5.45406589445031e-06	SCAPER
Successfu	26	1.63613366251649e-10	0.30424141338044[illegible]	0.635	0.299	5.48726507734779e-06	CSNK2A1
Successfu	26	1.99748995810137e-10	0.88489249068589[illegible]	0.913	0.659	6.69918182148039e-06	GLCCI1
Successfu	26	2.05877160144088e-10	0.66142323317089[illegible]	0.894	0.606	6.90470819691242e-06	GMDS-DT
Successfu	26	2.07892902707855e-10	0.41430480027968[illegible]	0.817	0.487	6.97231217101605e-06	KANK1
Successfu	26	2.23117422925823e-10	0.33736513457253[illegible]	0.404	0.147	7.48291213008626e-06	AL627171.2
Successfu	26	2.26084442027719e-10	0.42482245946788[illegible]	0.933	0.673	7.58242001672563e-06	ERBIN
Successfu	26	2.35326394549531e-10	0.42647965275911[illegible]	0.894	0.585	7.89237662040219e-06	SYNE2
Successfu	26	2.43585402951912e-10	0.289096436871118	0.442	0.173	8.16936724420122e-06	LINC02100
Successfu	26	2.50455777070571e-10	0.27099190779098[illegible]	0.798	0.427	8.3997858513928e-06	NT5C2
Successfu	26	2.71196627182909e-10	0.43277926832998[illegible]	0.433	0.172	9.09539248246039e-06	AC004594.1
Successfu	26	2.72525340769926e-10	0.56109249574176[illegible]	0.865	0.64	9.13995487874177e-06	GAB1
Successfu	26	2.89387200546516e-10	0.28419053314989[illegible]	0.433	0.166	9.70546793192905e-06	FBXL14
Successfu	26	3.01838488635296e-10	0.34045816265001[illegible]	0.606	0.281	1.01230592318506e-05	AF117829.1
Successfu	26	3.14603371705304e-10	0.29117685426395[illegible]	0.625	0.301	1.05511678802525e-05	MTUS1
Successfu	26	3.16766437767998e-10	0.51883344221074[illegible]	0.933	0.691	1.06237127898631e-05	ERC1
Successfu	26	3.55334589914496e-10	0.26847820035861	0.327	0.11	1.19172114765524e-05	LIN9
Successfu	26	4.29914380664429e-10	0.27359965113678[illegible]	0.721	0.382	1.44184684987236e-05	FBXL20
Successfu	26	4.37449525340184e-10	0.37918578694969[illegible]	0.769	0.415	1.46711821808591e-05	APOB
Successfu	26	4.71668508661046e-10	0.45601528801265[illegible]	0.577	0.297	1.58188184434742e-05	AC007388.1
Successfu	26	4.99447379977565e-10	0.30722841579002	0.712	0.365	1.67504662296876e-05	ATE1
Successfu	26	5.36857320369555e-10	0.37326322790394[illegible]	0.875	0.54	1.80051208105541e-05	MED13
Successfu	26	5.54245363961571e-10	0.56918710722713[illegible]	0.721	0.408	1.85882810165432e-05	MLLT3
Successfu	26	5.58088517468836e-10	0.52517567883131[illegible]	0.885	0.607	1.87171726988698e-05	CREB3L2
Successfu	26	5.79204434961924e-10	0.84940676354865[illegible]	0.385	0.138	1.9425358339753e-05	SLC2A5

Successfu	26	6.26285605555538e-10	0.48650376540764	0.952	0.682	2.10043666391216e-05	ATRX
Successfu	26	6.64011081953057e-10	0.39702734809422[illegible]	0.49	0.212	2.22696036665416e-05	FBXO32
Successfu	26	7.76939531623972e-10	0.27552997417556[illegible]	0.683	0.332	2.60569980116048e-05	ZNF215
Successfu	26	8.58365929438965e-10	0.57395671066367[illegible]	0.856	0.606	2.8787876541524e-05	DPYD
Successfu	26	8.92167202395018e-10	0.26078831602400[illegible]	0.452	0.187	2.99215036339241e-05	LINC02340
Successfu	26	9.36987545737452e-10	0.48640806691489[illegible]	0.644	0.342	3.14246883089426e-05	EPB41L4A
Successfu	26	9.55723526689065e-10	0.41206059302854[illegible]	0.875	0.565	3.20530556380978e-05	ARID2
Successfu	26	9.59390772859605e-10	0.42437585656037[illegible]	0.529	0.234	3.21760477401654e-05	HEATR5B
Successfu	26	9.77932668266128e-10	0.30050399450514[illegible]	0.577	0.269	3.27979058283094e-05	UNC13B
Successfu	26	1.03384992238398e-09	0.36019622354295[illegible]	0.442	0.183	3.46732586969138e-05	DDAH1
Successfu	26	1.04611517593577e-09	0.30523910009200[illegible]	0.683	0.338	3.50846107705339e-05	CYB5R4
Successfu	26	1.1047602993124e-09	0.34961202652186[illegible]	0.654	0.326	3.70514509183391e-05	TMC3-AS1
Successfu	26	1.11935823893909e-09	0.42140008537866[illegible]	0.567	0.255	3.75410366175393e-05	CBR4
Successfu	26	1.16747406966291e-09	0.70697842606257[illegible]	0.74	0.423	3.91547453483545e-05	SOX5
Successfu	26	1.19821669845392e-09	0.30856265365684[illegible]	0.712	0.376	4.01857916327475e-05	AUH
Successfu	26	1.30762752212091e-09	0.511192610547026	0.971	0.782	4.3855211836891e-05	FTX
Successfu	26	1.33719522190637e-09	0.55413934817678[illegible]	0.788	0.516	4.4846853352296e-05	TTC28
Successfu	26	1.43532977789558e-09	0.53714062449933[illegible]	0.952	0.766	4.81380900910619e-05	BCL2
Successfu	26	1.44692529956015e-09	0.43677893742752[illegible]	0.529	0.243	4.85269806966482e-05	PPM1L
Successfu	26	1.53942409938733e-09	0.28562729204862[illegible]	0.51	0.229	5.16292054452521e-05	RBFOX2
Successfu	26	1.63490521784112e-09	0.55818873114791[illegible]	0.913	0.695	5.48314511959554e-05	TPD52
Successfu	26	1.76879664118293e-09	0.76044596614569[illegible]	0.529	0.253	5.93219017519932e-05	PAIP2B
Successfu	26	1.77144095370948e-09	0.30048031848502[illegible]	0.635	0.313	5.94105867055085e-05	ARHGAP12
Successfu	26	1.82508680715175e-09	0.46892705429747[illegible]	0.99	0.876	6.12097613382554e-05	RABGAP1L
Successfu	26	1.87825326144252e-09	0.2679302409119	0.452	0.192	6.29928578822594e-05	BLOC1S5-TXNDC
Successfu	26	2.0886197664162e-09	0.29334128926922[illegible]	0.471	0.193	7.00481297260666e-05	C11orf65
Successfu	26	2.10558316199382e-09	0.63704577459297[illegible]	0.385	0.151	7.06170480869489e-05	AC068051.1
Successfu	26	2.11980872165103e-09	0.40200278198917[illegible]	0.673	0.35	7.10941449067322e-05	DST
Successfu	26	2.2218664518633e-09	0.36526505613684[illegible]	0.837	0.487	7.45169570625913e-05	ATP8A1
Successfu	26	2.26350557509259e-09	0.328204111851581	0.913	0.617	7.59134499774552e-05	DLG1
Successfu	26	2.56846481318288e-09	0.87119468067959[illegible]	0.452	0.212	8.61411729045273e-05	ST3GAL6

Successfu	26	2.72027218162714e-09	0.44656472371326[illegible]	0.875	0.578	9.1232488427411e-05	ZBTB38
Successfu	26	2.99305453189066e-09	0.52850809376638[illegible]	0.817	0.502	0.000100381062890549	SLC44A1
Successfu	26	3.13744822247911e-09	0.50671894815421[illegible]	0.567	0.28	0.000105223738485504	TESK2
Successfu	26	3.63111951699093e-09	0.30846144425349[illegible]	0.846	0.5	0.000121780486360842	BAZ2B
Successfu	26	3.72640021916781e-09	0.33212050287665[illegible]	0.558	0.28	0.00012497601055045	SETDB2
Successfu	26	3.86139316818436e-09	0.45683799129954[illegible]	0.827	0.526	0.000129503404074567	KLHL6
Successfu	26	4.00659440443885e-09	0.32963465929981[illegible]	0.663	0.365	0.00013437316313607	NCOA7
Successfu	26	4.04006796053757e-09	0.29958798542672[illegible]	0.952	0.695	0.000135495799260509	MTRNR2L8
Successfu	26	4.07724220938712e-09	0.49367461810258[illegible]	0.683	0.386	0.000136742549218425	HSH2D
Successfu	26	4.11805673551553e-09	0.26581145985649	0.26	0.082	0.00013811138679572	PNPLA7
Successfu	26	4.3117300575965e-09	0.32631404632263[illegible]	0.394	0.158	0.000144606802671672	EML5
Successfu	26	4.39238249046311e-09	0.43339730548328	0.702	0.407	0.000147311723965152	EPB41L2
Successfu	26	4.73087936469832e-09	0.25733427773156[illegible]	0.365	0.136	0.000158664232133252	KCNJ3
Successfu	26	4.80824911184327e-09	0.62545644426995[illegible]	0.923	0.678	0.000161259058713	FOXO3
Successfu	26	4.93936966299178e-09	0.48620614374725[illegible]	0.654	0.321	0.000165656579757418	AC007569.1
Successfu	26	5.00391514381311e-09	0.57698780103516[illegible]	0.904	0.656	0.000167821306093204	AFF1
Successfu	26	5.1956447112046e-09	0.48186827894215	0.654	0.36	0.00017425153232438	TRAM2
Successfu	26	5.52681737965465e-09	0.27886711891941[illegible]	0.615	0.312	0.000185358401278858	ARHGAP21
Successfu	26	5.6260045329255e-09	0.40605162043476[illegible]	0.625	0.332	0.000188684940025255	C16orf74
Successfu	26	5.76733416113767e-09	0.44346853334555[illegible]	0.356	0.139	0.000193424853096235	SPAG16
Successfu	26	5.85927402723981e-09	0.50346354270420[illegible]	0.808	0.456	0.000196508332325569	EDEM3
Successfu	26	5.87892386976527e-09	0.26240918483729[illegible]	0.375	0.148	0.000197167348744188	TCP11L2
Successfu	26	6.5458761989136e-09	0.26461057378337[illegible]	0.462	0.201	0.000219535595959164	STARD5
Successfu	26	6.64061183316835e-09	0.35521904045850[illegible]	0.346	0.128	0.0002227128396608	MORC4
Successfu	26	6.71306157313795e-09	0.37094546559070[illegible]	0.279	0.089	0.0002251426590399	GPC6
Successfu	26	6.7801295999146e-09	0.31138630918655[illegible]	0.471	0.205	0.000227391986521936	KIAA0586
Successfu	26	7.32750900744822e-09	0.31465878084952[illegible]	0.49	0.223	0.000245749997091799	ALDH1L2
Successfu	26	8.15484121883371e-09	0.34986045561666[illegible]	0.894	0.612	0.000273497064797245	STRBP
Successfu	26	8.39410608402836e-09	0.86037095435106[illegible]	0.654	0.38	0.000281521529846143	CADPS2
Successfu	26	8.88613800386357e-09	0.37440239630324[illegible]	0.769	0.448	0.000298023296373576	TRANK1
Successfu	26	9.05181328764099e-09	0.52186033162881[illegible]	0.99	0.84	0.000303579714040904	MT-ND4L

Successfu	26	9.3694514908821e-09	0.40510155218617	0.625	0.318	0.000314232664101204	Z93930.2
Successfu	26	1.02345670847355e-08	0.276841233877921	0.365	0.142	0.00034324691088786	NRG2
Successfu	26	1.0347850591938e-08	0.589838119525099	0.913	0.695	0.000347046213152418	SEL1L3
Successfu	26	1.12475505430998e-08	0.68540070727776	0.5	0.238	0.000377220350114482	CYREN
Successfu	26	1.1390450299488e-08	0.264842323322911	0.587	0.287	0.000382012922144229	NMRK1
Successfu	26	1.15056351855834e-08	0.645893829972768	0.683	0.398	0.000385875992854095	ESR2
Successfu	26	1.18394107497542e-08	0.362985445322155	0.692	0.36	0.000397070157725256	STAU2
Successfu	26	1.21286806548108e-08	0.448530480148075	0.885	0.638	0.000406771691801046	RUNX2
Successfu	26	1.3010325486547e-08	0.432918907967907	0.644	0.351	0.000436340296167814	KAT2B
Successfu	26	1.34752985825627e-08	0.370783964352301	0.856	0.553	0.000451934563861986	RICTOR
Successfu	26	1.36810582979265e-08	0.406728796168501	0.913	0.64	0.000458835333195858	DENND1B
Successfu	26	1.39634541581264e-08	0.585179764140093	0.962	0.817	0.000468306325555242	MED13L
Successfu	26	1.41135676925101e-08	0.407955737679476	0.692	0.361	0.000473340833271403	TEC
Successfu	26	1.44876351360004e-08	0.420250768808552	0.808	0.509	0.00048588630719118	UBE2E1
Successfu	26	1.54156828401588e-08	0.289442775255299	0.596	0.296	0.000517011171093246	RALGPS1
Successfu	26	1.58073525925933e-08	0.304150105121654	0.163	0.038	0.000530146991250393	AC106882.1
Successfu	26	1.63677371940015e-08	0.287899021604016	0.635	0.323	0.000548941170012422	IGF2R
Successfu	26	1.83411771031472e-08	0.497585342812011	0.558	0.29	0.000615126397685349	KIAA1328
Successfu	26	1.85928023533518e-08	0.412007554773396	1	0.995	0.000623565405326714	MT-ND1
Successfu	26	2.22223486911506e-08	1.29043534476056	0.442	0.214	0.000745293130403809	DCC
Successfu	26	2.22226209167359e-08	0.292612090148704	0.5	0.216	0.000745302260305488	CYP3A4
Successfu	26	2.24853829208317e-08	0.507682180335908	0.788	0.506	0.000754114772398853	ITGA4
Successfu	26	2.33392476250681e-08	0.421689851095894	0.808	0.526	0.000782751686849536	MAP3K5
Successfu	26	2.42571963319099e-08	0.619672406942965	0.875	0.653	0.000813537850579594	MAN1A1
Successfu	26	2.43698394252555e-08	0.306381107439098	0.74	0.39	0.000817315674644219	CASC4
Successfu	26	2.51286545191612e-08	0.25077835596476	0.433	0.189	0.000842764815263628	MERTK
Successfu	26	2.5325783405066e-08	0.301318715354348	0.798	0.485	0.000849376123839105	ABCA5
Successfu	26	2.82805930277525e-08	0.502857295082013	0.51	0.257	0.000948474528964763	TTLL7
Successfu	26	2.94544779152402e-08	0.262678807536555	0.779	0.455	0.000987844280321327	PHF21A
Successfu	26	2.950047734054e-08	0.715167333360796	0.606	0.343	0.00098938700904703	UBA6-AS1
Successfu	26	3.10194643590379e-08	0.343821495106931	0.596	0.305	0.00104033079567341	LIMD1

Successfu	26	3.24248417205636e-08	0.269224286581418	0.51	0.243	0.00108746434162426	AL139383.1
Successfu	26	3.54105635069393e-08	0.265484314333821	0.837	0.5	0.00118759947889573	SNRNP70
Successfu	26	3.64222176589441e-08	0.338382063540637	0.74	0.435	0.00122152833584567	CSNK1G3
Successfu	26	3.65138588969297e-08	0.450973530121515	0.952	0.698	0.00122460179968523	TBL1XR1
Successfu	26	3.92836745986711e-08	0.253315930445681	0.231	0.073	0.00131749587869023	LINC02541
Successfu	26	3.98931820794935e-08	0.460776472105744	0.933	0.686	0.00133793754058205	AHNAK
Successfu	26	4.13489701066388e-08	0.292776879165226	0.76	0.435	0.00138676175943645	PDS5B
Successfu	26	4.17325673321678e-08	0.463989684438511	0.885	0.683	0.00139962684318624	PIAS1
Successfu	26	4.20169006961346e-08	0.513375313586075	0.798	0.485	0.00140916281554696	ERN1
Successfu	26	4.20933191288067e-08	0.256337011502874	0.606	0.31	0.00141172573694192	NUGGC
Successfu	26	4.30188529729838e-08	0.403173347108116	0.913	0.653	0.00144276629100793	SLC38A2
Successfu	26	4.86571126694684e-08	0.368575862925838	0.663	0.374	0.00163186224470863	SLC12A6
Successfu	26	5.06564871278158e-08	0.431897529835249	0.875	0.576	0.00169891726529268	KIF13B
Successfu	26	5.16474060821502e-08	0.264532783319547	0.702	0.395	0.00173215070518315	IFNAR1
Successfu	26	5.35125316139845e-08	0.672811443708852	0.712	0.439	0.00179470328526981	HIST1H2AC
Successfu	26	5.36513598083436e-08	0.668772063870434	0.875	0.686	0.00179935930525223	ST6GAL1
Successfu	26	5.49156618765677e-08	0.399035823682506	0.654	0.349	0.00184176146801633	RANBP9
Successfu	26	5.52376469157238e-08	0.511160258118007	0.913	0.664	0.00185256020225955	WWOX
Successfu	26	5.88887476259345e-08	0.259333038059205	0.51	0.246	0.00197501081787859	ARHGAP6
Successfu	26	5.88919938308848e-08	0.271639259226019	0.644	0.36	0.00197511968910022	RDX
Successfu	26	6.01137116531859e-08	0.298451066455724	0.856	0.554	0.00201609366142455	CEP350
Successfu	26	6.1130399694491e-08	0.288670495195501	0.577	0.3	0.00205019134495384	COG6
Successfu	26	6.11896860613055e-08	0.302591959430131	0.577	0.294	0.00205217969112406	PECAM1
Successfu	26	6.18119714554128e-08	0.537493240008377	0.933	0.747	0.00207304989867164	GNG7
Successfu	26	6.36485724363388e-08	0.264920326575542	0.75	0.436	0.00213464582236993	ANKIB1
Successfu	26	6.67598936797496e-08	0.289761432796527	0.837	0.501	0.00223899331423144	RNF213
Successfu	26	7.17341308287058e-08	0.453591710656479	0.558	0.305	0.00240581927973314	AEBP2
Successfu	26	7.71787411488734e-08	0.268017238505185	0.856	0.54	0.00258842062065092	GALNT2
Successfu	26	7.71810584815782e-08	0.268315012920058	0.606	0.315	0.00258849833935517	ZNF518A
Successfu	26	8.29787755219405e-08	0.256959683147	0.327	0.127	0.00278294217345484	AL357060.1
Successfu	26	9.71064147452818e-08	0.303742332571989	0.683	0.383	0.00325675493772726	LRCH1

Successfu	26	1.0398735349437e-07	0.42038196994240З	0.779	0.483	0.00348752786149419	DMXL1
Successfu	26	1.04447243622828e-07	0.37758841820622[illegible]	0.837	0.524	0.00350295165662242	PKN2
Successfu	26	1.07412615161632e-07	0.35370567375638	0.856	0.539	0.00360240428729082	COBLL1
Successfu	26	1.08396720704395e-07	0.26733588799055[illegible]	0.663	0.365	0.00363540921898401	DGKD
Successfu	26	1.17232521885269e-07	0.38966210743468[illegible]	0.577	0.312	0.00393174431898816	DENND2C
Successfu	26	1.25675319408473e-07	0.27029546364762[illegible]	0.5	0.242	0.00421489886232136	ZNF532
Successfu	26	1.34419454931678e-07	0.67266578004027[illegible]	0.779	0.48	0.00450815967949863	BLNK
Successfu	26	1.39546880525549e-07	0.28498416509462[illegible]	0.5	0.241	0.00468012327906585	P2RX1
Successfu	26	1.40635092322087e-07	0.35830790775587[illegible]	0.952	0.675	0.00471661972629817	KANSL1
Successfu	26	1.42774329694893e-07	0.27225044778440[illegible]	0.356	0.151	0.00478836546930733	L3MBTL4
Successfu	26	1.54978796584825e-07	0.30259686218117[illegible]	0.827	0.527	0.00519767887986186	PTPN2
Successfu	26	1.66768257460189e-07	0.40804053115246	0.875	0.609	0.00559307381869982	FHIT
Successfu	26	1.72389222788084e-07	0.25755458797286[illegible]	0.625	0.344	0.00578158975386677	PLXNC1
Successfu	26	1.74929285640006e-07	0.42293973654242	0.5	0.25	0.00586677838179452	PIK3CG
Successfu	26	1.77767219230183e-07	0.32306173611746[illegible]	0.856	0.577	0.00596195699854189	PHF3
Successfu	26	1.79790070436436e-07	0.49182891280041[illegible]	0.865	0.616	0.0060297993822972	POU2AF1
Successfu	26	1.83060697675465e-07	0.38432590266239[illegible]	0.99	0.929	0.00613948967863976	NEAT1
Successfu	26	1.86185358966766e-07	0.28024101138706	0.962	0.731	0.0062442845690274	PDCD4
Successfu	26	1.98926353448359e-07	0.36379110035831[illegible]	0.827	0.568	0.00667159204195107	SEC14L1
Successfu	26	2.03408246128232e-07	0.46107203173127[illegible]	0.808	0.534	0.00682190575864865	SLAMF7
Successfu	26	2.10506184381576e-07	0.30738223299913[illegible]	0.231	0.078	0.0070599564117893	PTCH1
Successfu	26	2.13447832207851e-07	0.25928371213255	0.635	0.355	0.00715861339658691	WASHC3
Successfu	26	2.13743199124093e-07	0.40928274245116[illegible]	0.25	0.09	0.00716851941222385	NCAM1
Successfu	26	2.2057798505796e-07	0.57326136989049[illegible]	0.125	0.027	0.00739774446287386	SLIT2
Successfu	26	2.42662869041077e-07	0.29615912761347[illegible]	0.433	0.205	0.00813842730189963	RNF122
Successfu	26	2.43949418199958e-07	0.28677820556125[illegible]	0.577	0.315	0.0081815755875902	GOLGA3
Successfu	26	3.01012580307805e-07	0.35830490801144[illegible]	0.519	0.257	0.0100953599183632	CLIC5
Successfu	26	3.01589928162544e-07	0.35787583098973[illegible]	0.654	0.358	0.0101147230107154	VPS54
Successfu	26	3.11369574074142e-07	0.34270837776267[illegible]	0.798	0.524	0.0104427127752986	CDK6
Successfu	26	3.30368778735538e-07	0.41578063216948[illegible]	0.875	0.657	0.0110799081012325	UBE2J1
Successfu	26	3.38030966339689e-07	0.40539192981698[illegible]	0.952	0.769	0.0113368825491005	EXOC4

Successfu	26	3.53238283560993e-07	0.36706748500571[illegible]	0.808	0.523	0.0118469055540686	LARGE1
Successfu	26	3.55546855529547e-07	0.38182829081427[illegible]	0.837	0.535	0.01192433044075	MAP3K1
Successfu	26	3.67740281208125e-07	0.32785075566746[illegible]	0.788	0.492	0.0123332735511581	SCMH1
Successfu	26	4.1205121557361e-07	0.29066441352776[illegible]	0.413	0.186	0.0138193736679077	SELENOO
Successfu	26	4.25077877288919e-07	0.37301885096377[illegible]	0.865	0.591	0.0142562618485158	SCFD1
Successfu	26	4.29708226353789e-07	0.27405358014760[illegible]	0.365	0.158	0.0144115544954534	AC079447.1
Successfu	26	5.05951690971037e-07	0.27484593522136[illegible]	0.692	0.409	0.0169686078117866	ELP4
Successfu	26	5.51313349042614e-07	0.32409248933676[illegible]	0.375	0.175	0.0184899471001912	NAV2
Successfu	26	5.5291830447849e-07	0.25546087495381[illegible]	0.481	0.226	0.0185437740955996	CORO1C
Successfu	26	5.85656348945621e-07	0.95195660093363[illegible]	0.385	0.194	0.0196417426309382	NPAS3
Successfu	26	6.16758291197295e-07	0.27273284303543[illegible]	0.346	0.149	0.0206848395701749	TBC1D20
Successfu	26	6.20835086143184e-07	0.28797291542299[illegible]	0.75	0.482	0.0208215671190701	PLCL2
Successfu	26	6.75747471613576e-07	0.28164357739197[illegible]	0.567	0.312	0.0226632187029761	HS2ST1
Successfu	26	6.81997673156988e-07	0.26589008601257[illegible]	0.913	0.714	0.0228728379623391	NCOA2
Successfu	26	7.00517123761036e-07	0.35207056704078[illegible]	0.865	0.609	0.0234939432966976	PIP5K1B
Successfu	26	7.34189726491795e-07	0.43523924575571[illegible]	0.99	0.861	0.0246232550470818	ZBTB20
Successfu	26	7.53968821776798e-07	0.50979829616228	0.933	0.694	0.0252866063447502	EIF2AK3
Successfu	26	8.23458555813032e-07	0.36366980734933[illegible]	0.663	0.399	0.0276171530448575	KRTCAP2
Successfu	26	9.02204581778289e-07	0.37952579503399[illegible]	0.596	0.314	0.0302581372636803	KIF1B
Successfu	26	9.52854548737041e-07	0.39790573155810[illegible]	0.913	0.684	0.0319568358555429	VOPP1
Successfu	26	9.99886656470403e-07	0.43397023254777	0.933	0.747	0.0335341986847044	RAPGEF2
Successfu	26	1.08228967227072e-06	0.26630964165311	0.298	0.118	0.0362978310286155	SEPT10
Successfu	26	1.11817453251129e-06	0.32615970310171[illegible]	0.923	0.652	0.0375013374713638	XRN1
Successfu	26	1.34591999843057e-06	0.25796217953695	0.269	0.104	0.0451394649073644	AC093797.1
Successfu	26	1.41864427594897e-06	0.40767847507687[illegible]	0.606	0.35	0.0475784917267766	PRCP
Successfu	33	3.56051986850928e-13	0.72843032733458[illegible]	0.721	0.228	1.19412715350064e-08	HBB
Successfu	33	2.35604222243519e-10	0.53175379753839[illegible]	0.326	0.065	7.90169440560314e-06	LINC00278
Successfu	33	1.98090524152428e-09	0.38786319251440[illegible]	0.186	0.025	6.64355999902413e-05	SEPT7-AS1
Successfu	33	4.36565651334902e-09	0.28415847783080[illegible]	0.186	0.025	0.000146415388144699	AC015923.1
Successfu	33	2.0061013746609e-08	0.82635844905727[illegible]	0.767	0.365	0.000672806279033774	P2RY8
Successfu	33	3.0812574537957e-08	0.29000539227255	0.186	0.029	0.001033392124854	LOH12CR2

Successfu	33	4.26967247249517e-08	0.82192185770189	0.884	0.445	0.00143196275382543	MT-ND6
Successfu	33	6.45985667653287e-08	0.29548104163248[illegible]	0.349	0.093	0.00216650673217559	AL080276.2
Successfu	33	8.45835352528473e-08	0.69541057604876[illegible]	0.907	0.54	0.00283676260530999	VWA8
Successfu	33	1.01110220409351e-07	0.64291601888053[illegible]	0.791	0.388	0.00339103457208882	SLC15A2
Successfu	33	1.09793570567951e-07	0.40625870636764	0.488	0.171	0.00368225676970795	PYGB
Successfu	33	1.1208980677495e-07	0.32525164504892[illegible]	0.419	0.13	0.00375926793961828	RELT
Successfu	33	1.26839848891825e-07	0.317204115433117	0.163	0.025	0.00425395485213402	FAM85B
Successfu	33	1.2805533588766e-07	0.70238513460765[illegible]	0.651	0.28	0.00429471985500036	TSIX
Successfu	33	1.47779327170405e-07	0.31218061144470[illegible]	0.465	0.154	0.00495622307464105	APLF
Successfu	33	2.06556274966942e-07	0.60149333392187	0.349	0.099	0.0069274843498413	AC092546.1
Successfu	33	3.22412967668095e-07	0.30098254015718[illegible]	0.14	0.019	0.0108130861096526	AL589666.1
Successfu	33	4.79980964063548e-07	1.5756027684588	0.86	0.6	0.0160976015727633	CROCC
Successfu	33	4.81986801839684e-07	0.52850411901687[illegible]	0.279	0.071	0.0161648733600993	TBL2
Successfu	33	6.79027759805485e-07	0.37858705988471	0.767	0.353	0.0227732330083564	PLXNC1
Successfu	33	6.91829730178527e-07	0.59445031309723[illegible]	0.465	0.166	0.0232025854907274	MOXD1
Successfu	59	2.16813653566505e-08	0.41110330643876[illegible]	0.175	0.039	0.000727149631331344	TLL1
Successfu	59	6.83013719965286e-08	0.84768450641676[illegible]	0.162	0.036	0.00229069141401958	PRSS23
Successfu	59	1.48392848425109e-07	0.86297899731269[illegible]	0.975	0.874	0.00497679935048132	JCHAIN
Successfu	59	2.09155770278147e-07	1.03011075113113	0.762	0.665	0.00701466622358848	HSPA1B
Successfu	59	9.84156464315219e-07	0.72810440007502[illegible]	1	1	0.0330066395002038	MT-CO2
Successfu	64	3.60547312697486e-44	1.03877737138877	0.319	0.011	1.20920357732483e-39	TFPI2
Successfu	64	2.16793628688564e-22	0.68221087804142[illegible]	0.511	0.087	7.27082471895705e-18	IGFBP4
Successfu	64	4.7527601191612e-15	0.55082861299448	0.574	0.157	1.59398068876428e-10	NRG1
Successfu	64	3.54704505579819e-14	1.4409870977252	1	0.81	1.1896079708136e-09	FBXW7
Successfu	64	2.07527293054135e-13	1.76878524074749	0.872	0.651	6.96005035444959e-09	GAB1
Successfu	64	6.42784508802e-13	1.2298571647781	0.957	0.775	2.15577068562015e-08	BCL2
Successfu	64	2.29473167229078e-11	1.46057746695518	0.872	0.622	7.6960710825288e-07	CREB3L2
Successfu	64	2.61837007208456e-11	1.30035042827995	0.915	0.704	8.78148954775721e-07	ERC1
Successfu	64	3.30564326622809e-11	1.70110678334929	0.83	0.634	1.10864663862758e-06	CARMIL1
Successfu	64	5.38274341120881e-11	1.63210710762526	0.851	0.558	1.80526448525121e-06	AC022217.3
Successfu	64	1.07590905399236e-10	1.17970398121753	0.936	0.67	3.60838378527956e-06	CCSER1

Successfu	64	1.85166492168412e-10	1.38855742563183	0.894	0.673	6.2101138143442e-06	GLCCI1
Successfu	64	1.91620995400252e-10	1.59051206054042	0.894	0.7	6.42658494373366e-06	AHNAK
Successfu	64	2.14960642448673e-10	0.89435847273070[illegible]	0.936	0.779	7.20935002644361e-06	EXOC4
Successfu	64	2.18338265708942e-10	1.05608463886645	0.915	0.62	7.3226287553465e-06	GMDS-DT
Successfu	64	2.39598270306913e-10	0.98834830780622[illegible]	0.957	0.803	8.03564678955325e-06	ATXN1
Successfu	64	2.969036264116e-10	0.424110819946171	0.298	0.063	9.95755382259225e-06	LINC01948
Successfu	64	3.0471007166127e-10	1.14163306664993	0.979	0.932	1.02193663833757e-05	NEAT1
Successfu	64	3.18001435751102e-10	0.82019690026276[illegible]	0.277	0.056	1.06651321522205e-05	SLC14A2
Successfu	64	3.78588312387224e-10	1.23512439224704	0.936	0.692	1.26970948208427e-05	TENT5C
Successfu	64	4.86637952667253e-10	0.39910273412651[illegible]	0.17	0.021	1.63208636565543e-05	SELE
Successfu	64	6.3983698991934e-10	0.90501366661597[illegible]	0.915	0.69	2.14588529679148e-05	KANSL1
Successfu	64	7.44022688383297e-10	1.03638448985055	0.872	0.622	2.4953032922999e-05	UBE2E2
Successfu	64	1.04573960223732e-09	0.99290728665201[illegible]	0.915	0.766	3.50720147798353e-05	FNDC3A
Successfu	64	1.06397664508983e-09	1.13968229248945	0.915	0.72	3.56836487230227e-05	FNDC3B
Successfu	64	1.08314382458958e-09	1.00335872288466	1	0.889	3.63264775890853e-05	HSP90AA1
Successfu	64	1.11403650847624e-09	0.86433025375118[illegible]	0.915	0.6	3.73625564212762e-05	TXNDC11
Successfu	64	1.34880311767912e-09	0.95816786066426[illegible]	0.894	0.743	4.52361589607222e-05	SND1
Successfu	64	1.34982287824493e-09	1.13713603195363	0.702	0.389	4.52703596905784e-05	ESR1
Successfu	64	1.40721966847324e-09	1.00480976722317	0.894	0.663	4.71953332412556e-05	MAN1A1
Successfu	64	1.45319346425571e-09	1.32412213427893	0.745	0.416	4.87372024042079e-05	ERO1B
Successfu	64	1.61477947033769e-09	1.25478233105407	0.915	0.757	5.41564738761853e-05	RAPGEF2
Successfu	64	2.82533817048274e-09	1.6402559831841	0.766	0.443	9.47561915616502e-05	BMP6
Successfu	64	3.49088133693082e-09	0.97633210696073[illegible]	0.851	0.575	0.000117077178277986	SNX29
Successfu	64	3.59877178818844e-09	1.21479314324209	0.83	0.578	0.000120695608232264	EAF2
Successfu	64	4.03732579679509e-09	1.13475556221241	0.681	0.355	0.000135403832572914	EPB41L4A
Successfu	64	4.05928635729022e-09	1.13477031448359	0.447	0.148	0.000136140345850799	A2M
Successfu	64	5.30434127836381e-09	0.87188423531747[illegible]	0.936	0.73	0.000177896997793766	RAB11A
Successfu	64	6.69415157420128e-09	0.93816268878591[illegible]	1	0.866	0.000224508455495562	FOSB
Successfu	64	6.85636369728269e-09	1.22151122669519	0.723	0.426	0.000229948725679467	LRRK1
Successfu	64	7.68249472618961e-09	1.05441035667269	0.979	0.834	0.000257655508126947	HSP90AB1
Successfu	64	7.70107011082066e-09	1.19038251345316	0.872	0.762	0.000258278489376703	ELL2

Successfu	64	7.71175787142998e-09	0.465621531020789	0.234	0.046	0.000258636935492019	QRICH2
Successfu	64	7.78748419116394e-09	1.6935429992464	0.872	0.774	0.000261176644803256	ANKRD28
Successfu	64	1.2984521842961e-08	1.01506377318235	0.915	0.709	0.000435474893569227	CDK14
Successfu	64	1.34758202993921e-08	0.815765830940322	0.681	0.33	0.000451952061201011	CCND3
Successfu	64	1.68416509748799e-08	1.37977370789793	0.553	0.258	0.000564835290395522	AL606489.1
Successfu	64	1.90576763947638e-08	0.808517875564507	0.468	0.185	0.000639156350927587	LINC02100
Successfu	64	1.97729829670048e-08	0.250197318104322	0.532	0.21	0.000663146302747408	IFITM3
Successfu	64	2.04193627220269e-08	0.962303924949081	0.872	0.586	0.000684824586971337	CD38
Successfu	64	2.17935545243841e-08	1.29149797324215	0.596	0.263	0.000730912231638793	PLCL1
Successfu	64	2.72006341192784e-08	1.07042805405141	0.872	0.587	0.000912254867092359	NR4A1
Successfu	64	2.96230202678851e-08	0.887118124212452	0.894	0.757	0.00099349685374433	GNG7
Successfu	64	4.77907786213317e-08	0.777363795613797	0.915	0.797	0.00160280713340222	STAG1
Successfu	64	4.8737209883602e-08	0.972937950652399	0.872	0.618	0.00163454854507624	NFKBIZ
Successfu	64	5.01650204195959e-08	0.803682414489209	1	0.991	0.00168243445483241	MT-ND5
Successfu	64	5.94507370663524e-08	0.675304754878372	0.766	0.451	0.00199385881973133	FLI1
Successfu	64	7.15379025060839e-08	1.12591834551416	0.872	0.696	0.00239923817424904	ARF4
Successfu	64	7.31940308020238e-08	0.848596141912023	0.872	0.651	0.00245478140503827	RUNX2
Successfu	64	7.3571196403796e-08	1.29895319086344	0.872	0.697	0.00246743078499051	OSBPL3
Successfu	64	7.3651362372161e-08	1.17532732547002	0.872	0.708	0.00247011939123753	EIF2AK3
Successfu	64	8.3589907906852e-08	0.821746781963125	0.787	0.559	0.00280343833138	MDFIC
Successfu	64	9.65998343946531e-08	0.725301103352905	0.723	0.429	0.00323976524592788	EIF2AK4
Successfu	64	1.05033979387497e-07	0.804108932466082	0.723	0.408	0.00352262960069788	NBEA
Successfu	64	1.10587251465681e-07	0.63479808606105	0.936	0.88	0.003708875239656	HNRNPC
Successfu	64	1.18388968100273e-07	1.07166486472715	0.787	0.562	0.00397052921214694	PDK1
Successfu	64	1.20124674146235e-07	1.24971469601146	0.745	0.487	0.00402874132151643	YWHAQ
Successfu	64	1.32504930061158e-07	0.62977700848446	0.213	0.044	0.00444395034439111	ADAMTS9
Successfu	64	1.3660485866214e-07	1.11292183359458	0.872	0.639	0.00458145374981086	NAMPT
Successfu	64	1.39744696767982e-07	0.52006532769126	0.234	0.052	0.00468675764020457	FGF7
Successfu	64	1.43709954026385e-07	0.681261536147985	0.766	0.481	0.00481974443813691	MAPKAPK2
Successfu	64	1.46735213896026e-07	1.04609679159367	0.745	0.431	0.00492120560364492	LINC02384
Successfu	64	1.76554538652095e-07	0.520436821904802	0.915	0.716	0.00592128611731395	MSI2

Successfu	64	1.76892745560836e-07	0.90758582152752	0.83	0.67	0.00593262890061931	DNAJC1
Successfu	64	1.77203260823861e-07	0.60969141912132[illegible]	0.766	0.479	0.00594304296151064	ASXL2
Successfu	64	1.89712553609292e-07	0.42625662431441[illegible]	0.128	0.017	0.00636257962294844	ANO2
Successfu	64	1.93332726450757e-07	0.61198655326223[illegible]	0.894	0.668	0.0064839929797055	AFF1
Successfu	64	2.12583448756587e-07	1.44576847843951	0.809	0.598	0.00712962370439842	CLIC4
Successfu	64	2.18760533682766e-07	0.96560458423438[illegible]	0.511	0.224	0.0073367907786526	BAG3
Successfu	64	2.2443416530888e-07	1.03424083378177	0.872	0.621	0.00752707303612922	CPEB4
Successfu	64	2.2581930996197e-07	0.73290977583887[illegible]	0.787	0.56	0.00757352801750455	MAP4
Successfu	64	2.26480276384203e-07	0.42720628335381[illegible]	0.298	0.086	0.00759569550937342	AC009686.1
Successfu	64	2.27071578246276e-07	0.68008998692481[illegible]	0.404	0.148	0.00761552659122361	COL10A1
Successfu	64	2.46781430212548e-07	0.46112633257762[illegible]	0.319	0.094	0.00827655560646842	COL4A1
Successfu	64	2.47903856873341e-07	0.84657016878424[illegible]	0.787	0.552	0.00831419955181812	BRD2
Successfu	64	2.53232968261476e-07	1.07983412081728	0.787	0.559	0.00849292728955338	GALNT2
Successfu	64	2.55775582000405e-07	0.90785852487992[illegible]	0.787	0.549	0.00857820146912957	FAM3C
Successfu	64	3.17204150161778e-07	0.56214408513953[illegible]	0.404	0.147	0.0106383927881257	AC009126.1
Successfu	64	3.52077322621652e-07	0.86288223394738	0.83	0.635	0.011807969246085	DLG1
Successfu	64	3.83117910502606e-07	0.73640577898072[illegible]	0.234	0.056	0.0128490084824364	LINC01358
Successfu	64	4.08916625369755e-07	0.52188767723607[illegible]	0.787	0.498	0.0137142457816508	AC116366.3
Successfu	64	4.27907873926222e-07	0.87889104963929[illegible]	0.681	0.435	0.0143511742757376	MANEA
Successfu	64	4.73918008716839e-07	1.0797881401875	0.511	0.222	0.0158942621763453	SLC16A1
Successfu	64	5.03647031512358e-07	0.26172136490637[illegible]	0.34	0.108	0.0168913141428615	COL4A2
Successfu	64	5.28984062995005e-07	0.90709203054736[illegible]	0.83	0.526	0.0177410675047265	IFNG-AS1
Successfu	64	5.36107748167014e-07	0.59892127119999[illegible]	0.872	0.676	0.0179799816580253	BBX
Successfu	64	5.67869300069579e-07	1.04055763585634	0.915	0.758	0.0190452005857335	PPP1R15A
Successfu	64	5.77246200947209e-07	0.91812700603549[illegible]	0.681	0.44	0.0193596830873675	UBALD2
Successfu	64	5.99488755054879e-07	0.86756969596053[illegible]	0.957	0.872	0.0201056538670305	JUN
Successfu	64	6.02749528986702e-07	0.85718071323439[illegible]	0.915	0.683	0.020215013703156	NFKBIA
Successfu	64	6.26644323123312e-07	0.78693030924154[illegible]	0.894	0.707	0.0210163973089097	TPD52
Successfu	64	6.60283802543769e-07	1.14170845672691	0.766	0.525	0.0221445981697129	AC007384.1
Successfu	64	7.06976822595803e-07	0.76330525409395[illegible]	0.894	0.767	0.0237105886762181	ETV6
Successfu	64	7.28738005100169e-07	0.64743904744677[illegible]	0.936	0.747	0.0244404152150495	CDKAL1

Successfu	64	7.71004520029768e-07	0.88033974365181	0.596	0.301	0.0258579495927584	NFE2L3
Successfu	64	7.73684326565867e-07	1.02578820287763	0.936	0.706	0.025947824944366	VIM
Successfu	64	7.99800946796473e-07	0.94257322930558	0.894	0.727	0.0268237241536601	DNAJB1
Successfu	64	8.20755059465344e-07	0.70696994861006	0.851	0.578	0.0275264831843487	IFRD1
Successfu	64	8.20982195852444e-07	0.80180123665711	0.553	0.287	0.0275341008844993	AC026202.2
Successfu	64	8.40264324800394e-07	0.88449135150081	0.83	0.696	0.0281807849251556	TRA2B
Successfu	64	8.45549651851616e-07	0.83823384235747	0.915	0.763	0.0283580442237995	UTRN
Successfu	64	8.64029993703766e-07	0.55250293044143	0.468	0.207	0.0289778379288369	C11orf65
Successfu	64	8.70025494336856e-07	1.17861517155987	0.851	0.693	0.0291789150290695	FOXO3
Successfu	64	8.87919903079509e-07	0.75024779417187	0.851	0.622	0.0297790577094806	ATP11B
Successfu	64	8.95891923143209e-07	0.57867662892906	0.936	0.841	0.030046423318377	XIST
Successfu	64	8.9717986520351e-07	1.19802636660741	0.426	0.185	0.0300896183191953	AC004594.1
Successfu	64	9.00667289977191e-07	0.97268425785321	0.894	0.721	0.030206579571255	MCL1
Successfu	64	9.67401569743208e-07	0.86968328648291	0.851	0.688	0.0324447138460477	CFLAR
Successfu	64	9.76890073805539e-07	0.75810582684042	0.809	0.662	0.0327629392952902	UBR5
Successfu	64	1.02947525632168e-06	1.01450872651117	0.574	0.288	0.0345265411465165	CHODL
Successfu	64	1.0304478870683e-06	0.74344600454983	0.915	0.837	0.0345591612364966	CDC42SE2
Successfu	64	1.07742793299145e-06	0.60202496304020	0.553	0.283	0.0361347780166671	TSIX
Successfu	64	1.08036157598184e-06	0.78318500168629	0.723	0.464	0.0362331665352789	PPP1R15B
Successfu	64	1.10264474168723e-06	0.84606889977106	0.872	0.72	0.0369804993467064	IER2
Successfu	64	1.21958592380115e-06	1.05173370102726	0.404	0.162	0.040902472712443	AC068051.1
Successfu	64	1.23077417224636e-06	0.81919232505457	0.596	0.313	0.0412777041887984	GALM
Successfu	64	1.23647081431525e-06	0.73089146612758	0.745	0.521	0.0414687581705048	ASAP1
Successfu	64	1.27522358840521e-06	0.82689889107636	0.702	0.478	0.0427684487079341	EDEM3
Successfu	64	1.28897703609323e-06	0.89684253517685	0.319	0.107	0.0432297118364948	HSPE1-MOB4
Successfu	64	1.31410002164676e-06	0.82687876015673	0.957	0.785	0.044072286525989	CCNL1
Successfu	64	1.3470766350567e-06	0.67480445158861	0.936	0.793	0.0451782561865315	FTX
Successfu	64	1.41879598978965e-06	1.10088809438074	0.787	0.64	0.0475835799055653	FBXO11
Successfu	64	1.42676700429164e-06	0.70402596163630	0.723	0.525	0.0478509117899331	PTK2B
Successfu	64	1.46795258827427e-06	0.78429103505193	0.851	0.6	0.0492321939055426	CROCC
Successfu	191	2.70574583442705e-53	2.0802228225117	0.588	0.106	9.07453037950143e-49	NRG1

Successfu	191	6.45806263862676e-33	1.31944425725383	0.418	0.082	2.16590504774264e-28	COL3A1
Successfu	191	2.71548342955562e-25	0.81469535340206	0.315	0.058	9.10718832604363e-21	PRR16
Successfu	191	3.82351113962362e-16	1.1342721672914	0.345	0.104	1.28232916600697e-11	ADGRL2
Successfu	191	5.12651205715862e-16	1.64684109536399	0.861	0.624	1.71932961372986e-11	APOA1
Successfu	191	1.05097850335399e-15	1.38581185093209	0.37	0.126	3.52477170454861e-11	A2M
Successfu	191	1.52247440037444e-13	0.58338363754265	0.297	0.099	5.1060746439758e-09	MB21D2
Successfu	191	3.11229419198405e-13	0.78197501474281	0.152	0.027	1.04380122610761e-08	PAPPA
Successfu	191	6.66590560805906e-13	0.79678566580121	0.218	0.055	2.23561142283085e-08	SDK1
Successfu	191	1.18080966953367e-12	1.0673400424656	0.297	0.098	3.96019946968203e-08	RBPMS
Successfu	191	6.86756126094837e-11	1.09672014621955	0.2	0.055	2.30324269569686e-06	IGFBP5
Successfu	191	1.91307684427476e-10	0.81197626864558	0.182	0.047	6.41607712032868e-06	NNMT
Successfu	191	1.95742075744257e-10	0.63318464489354	0.133	0.027	6.5647977363109e-06	NLGN1
Successfu	191	1.30840722014059e-09	0.88657891464817	0.23	0.078	4.3881361349075e-05	ADGRL3
Successfu	191	4.72303340038681e-09	0.62041270983266	0.194	0.06	0.000158401094182173	RGS5
Successfu	191	6.58263587120684e-09	0.74974351360497	0.158	0.043	0.000220768441848535	FGF7
Successfu	191	7.70958786031709e-09	0.91773252006336	0.176	0.053	0.000258564157659314	COL6A3
Successfu	191	2.20970463841681e-08	0.36202753523945	0.127	0.031	0.000741090741632231	ZYG11A
Successfu	191	2.99930215882673e-08	0.93242174306209	0.261	0.103	0.00100590595802731	BNC2
Successfu	191	4.94700490721316e-08	0.85866719237676	0.17	0.053	0.00165912650578115	COL6A1
Successfu	191	4.97379677618386e-08	0.63296086672789	0.115	0.027	0.00166811196279654	BMP5
Successfu	191	8.15636544510294e-08	0.49966517119450	0.236	0.092	0.00273548184297862	ADAMDEC1
Successfu	191	8.83717747144729e-08	0.27364974785832	0.188	0.063	0.00296381258037399	CCDC144A
Successfu	191	1.04247308690564e-07	0.29814798440063	0.109	0.025	0.00349624623886413	COL12A1
Successfu	191	1.51689690738686e-07	0.43648634360977	0.109	0.026	0.00508736884799405	TNC
Successfu	191	2.06950047464105e-07	0.66775717683949	0.218	0.089	0.00694069069185114	RGPD2
Successfu	191	5.63422420455943e-07	0.58469349192394	0.842	0.949	0.0188960611372514	NEAT1
Successfu	191	8.26754980002473e-07	0.46943143069731	0.352	0.195	0.0277277085193229	GLDC
Successfu	191	8.73061776690118e-07	1.58422399781161	0.63	0.498	0.0292807458666332	CUBN
Successfu	191	1.01119328617686e-06	0.31861401796775	0.115	0.031	0.0339134004317995	AC022395.1
Successfu	191	1.08472868692331e-06	0.37762479119956	0.345	0.569	0.0363796307020341	LGALS3
Successfu	191	1.3859574568595e-06	1.0370594437524	0.364	0.2	0.0464822411881541	IFITM3

Successfu	191	1.39410919547722e-06	1.14598271474588	0.297	0.15	0.0467556341979149	PTPRM
Recoverec	0	1.58800061838334e-203	2.08200532609457	0.774	0.082	5.32583647393405e-199	REG1B
Recoverec	0	1.28704797498465e-92	1.51995396568649	0.9	0.399	4.31650149850353e-88	APOA4
Recoverec	0	7.23839915742935e-91	1.58633755813032	0.97	0.599	2.42761430941866e-86	PHGR1
Recoverec	0	2.70064410606253e-87	1.43522016196976	0.981	0.689	9.0574202029125e-83	FABP6
Recoverec	0	1.61210696248737e-78	1.01404726952895	0.67	0.186	5.40668433079013e-74	PDE3A
Recoverec	0	8.48523590962547e-76	0.86554393598048€	0.511	0.101	2.84577841937019e-71	CYP3A4
Recoverec	0	1.57160664333005e-68	1.13797415025047	0.807	0.35	5.27085436040034e-64	APOC3
Recoverec	0	1.26896815822388e-65	1.05629605590142	0.633	0.2	4.25586540905126e-61	GUCA2A
Recoverec	0	1.84366764236885e-61	1.02189527178057	0.63	0.203	6.18329253897666e-57	SLC5A1
Recoverec	0	2.23542534014532e-59	1.13521747812858	0.944	0.575	7.49716950577937e-55	REG1A
Recoverec	0	1.59649679461926e-55	0.99619045363047‹	0.567	0.178	5.35433094979407e-51	CLDN4
Recoverec	0	1.27229361516095e-54	1.21816922564075	0.807	0.387	4.26701832652679e-50	FKBP5
Recoverec	0	1.53423976769215e-54	1.17313427921324	0.848	0.452	5.14553333288594e-50	ALDOB
Recoverec	0	2.33445131951522e-54	1.0952402631087	0.678	0.27	7.82928283539015e-50	KRT19
Recoverec	0	4.16389498744378e-54	1.32872387318322	0.907	0.716	1.39648710088889e-49	RPS26
Recoverec	0	1.62362797003561e-52	0.89616926471052‹	0.767	0.339	5.44532348590542e-48	SELENOP
Recoverec	0	2.32804586933204e-52	0.96961222842251ξ	0.848	0.463	7.80780023656581e-48	FABP1
Recoverec	0	1.89937423159318e-49	0.88144061405925ž	0.467	0.126	6.37012129791721e-45	PMP22
Recoverec	0	8.02158932176959e-46	0.62782314979406ξ	0.459	0.129	2.69028062673509e-41	DPEP1
Recoverec	0	1.37384825785721e-45	0.47397566906911ξ	0.326	0.063	4.6076122872015e-41	PDE9A
Recoverec	0	1.69858962321278e-45	0.54015862110589	0.356	0.076	5.69672987833101e-41	SLC7A9
Recoverec	0	1.33957236641255e-43	0.81968111961735ξ	0.637	0.259	4.4926578024744e-39	CYP3A5
Recoverec	0	3.30179627983756e-43	0.78538946886382	0.548	0.199	1.10735643633192e-38	ADIRF
Recoverec	0	1.05240259611099e-42	0.81094641436906‹	0.504	0.169	3.52954782683704e-38	MUC17
Recoverec	0	1.3413560615174e-42	0.88402344509617ξ	0.53	0.19	4.49863995911705e-38	MUC13
Recoverec	0	2.26474763188588e-42	0.81066111006588ξ	0.596	0.239	7.59551060781888e-38	GDA
Recoverec	0	2.86256778589611e-42	0.90338911662161‹	0.696	0.34	9.60047984033837e-38	PCSK5
Recoverec	0	5.60157605217847e-42	0.79856969408266‹	0.667	0.292	1.87865657637961e-37	FABP2
Recoverec	0	1.40038385871451e-39	0.66148065391865ξ	0.433	0.132	4.69660738535672e-35	CLDN3
Recoverec	0	2.70724305343953e-39	1.02709667840483	0.337	0.081	9.07955175262549e-35	FAM177B

Recoverec	0	2.04994056777909e-38	0.29348062425816	0.178	0.019	6.87509067621752e-34	C11orf86
Recoverec	0	2.94465831159504e-37	0.459389117491951	0.33	0.078	9.87579504542744e-33	FXYD3
Recoverec	0	7.79639757630189e-37	0.262667883526567	0.159	0.016	2.61475581914013e-32	LCN2
Recoverec	0	9.78064320842577e-37	0.756019301103218	0.741	0.39	3.28023211924184e-32	ANPEP
Recoverec	0	3.06979765569786e-36	0.74078207295829	0.552	0.223	1.02954873776795e-31	SLC15A1
Recoverec	0	3.08730821474736e-36	0.576731571827152	0.326	0.079	1.03542142906197e-31	TM4SF20
Recoverec	0	4.47435013552223e-36	0.624987501454859	0.378	0.104	1.50060754845145e-31	ZBTB16
Recoverec	0	1.89262413570148e-35	0.697548940849025	0.685	0.359	6.34748282631561e-31	KRT8
Recoverec	0	9.3214000027491e-35	0.909673586521631	0.726	0.394	3.12621113292199e-30	RPS4Y1
Recoverec	0	1.10256597882208e-34	0.261069209999141	0.126	0.009	3.6977857797735e-30	CLCA4
Recoverec	0	3.08842501430848e-34	0.61807154494272	0.311	0.078	1.03579598129878e-29	TMPRSS2
Recoverec	0	9.47670294913058e-34	0.566027784558542	0.374	0.109	3.17829663507941e-29	SERPINA1
Recoverec	0	2.71482248676958e-33	0.76880724220683	0.563	0.246	9.10497165612782e-29	EPCAM
Recoverec	0	1.10559785787831e-32	0.798404683165512	0.611	0.315	3.70795409575226e-28	LGALS3
Recoverec	0	1.12846350716599e-32	0.606017611974453	0.507	0.199	3.78464091033329e-28	CST3
Recoverec	0	1.71326901695345e-32	0.717420181144308	0.726	0.421	5.74596162905848e-28	S100A10
Recoverec	0	1.75854517010264e-32	0.977847319277046	0.915	0.854	5.89780879149024e-28	RPL8
Recoverec	0	2.61246713015863e-32	0.917035209798748	0.922	0.856	8.76169226112601e-28	RPL6
Recoverec	0	4.15606014848757e-32	1.03054817459684	0.926	0.786	1.39385945259976e-27	RPL21
Recoverec	0	8.73293773195694e-32	0.498312687142624	0.37	0.112	2.92885265654372e-27	HHLA2
Recoverec	0	1.66135453519798e-31	0.606472750701482	0.456	0.162	5.571850840147e-27	ELF3
Recoverec	0	1.7212705488204e-31	0.770607204109801	0.967	0.901	5.77279716663385e-27	FTH1
Recoverec	0	1.84911294893173e-31	0.74818281102663	0.548	0.238	6.20155500812723e-27	LMO7
Recoverec	0	2.2827323710198e-31	1.02385771954825	0.889	0.765	7.6558278259262e-27	RPL10A
Recoverec	0	2.43278133966181e-31	0.998828845298858	0.907	0.813	8.15906205695779e-27	RPS7
Recoverec	0	3.5062309527228e-31	0.48914054948988	0.407	0.137	1.17591973692417e-26	PCK1
Recoverec	0	4.76978660594033e-31	0.913055200402158	0.944	0.86	1.59969103190027e-26	RPL18A
Recoverec	0	1.14075432217381e-30	0.599472058075353	0.526	0.22	3.82586184570652e-26	TSPAN8
Recoverec	0	2.58488825006555e-30	0.897527726486957	0.959	0.915	8.66919821306984e-26	RPS19
Recoverec	0	5.30767523677132e-30	0.445774902513195	0.33	0.094	1.78008812090836e-25	PRAP1
Recoverec	0	7.07209846963052e-30	1.00307800340678	0.956	0.893	2.37184038474468e-25	RPS27

Recoverec	0	8.52612304348081e-30	0.49913365785660[illegible]	0.378	0.122	2.85949114632259e-25	CDHR2
Recoverec	0	8.75962010646354e-30	0.88811959612823[illegible]	0.933	0.84	2.93780139130574e-25	RPS29
Recoverec	0	6.00083316721552e-29	0.51929097516647[illegible]	0.426	0.154	2.01255942762074e-24	THSD4
Recoverec	0	6.69645740218473e-29	0.47100933274293[illegible]	0.293	0.079	2.24585788354472e-24	PRSS3
Recoverec	0	4.27288934642753e-28	0.81312598163207[illegible]	0.952	0.889	1.43304162900487e-23	RPL28
Recoverec	0	1.55458258195649e-27	0.52516400504817[illegible]	0.333	0.105	5.21375906336567e-23	CYBRD1
Recoverec	0	5.15543693710908e-27	0.66372936281413[illegible]	0.515	0.239	1.72903043996764e-22	ACSL5
Recoverec	0	1.45364388834507e-26	0.83650228376725[illegible]	0.952	0.873	4.87523087273169e-22	RPL11
Recoverec	0	1.53141401463325e-26	0.59290376801128[illegible]	0.7	0.416	5.13605632227698e-22	PPDPF
Recoverec	0	1.91215140575907e-26	0.77420798522271[illegible]	0.759	0.425	6.41297338463479e-22	CD52
Recoverec	0	2.18833886879194e-26	0.94515482336263	0.959	0.893	7.33925089815441e-22	RPS8
Recoverec	0	2.31436425352976e-26	0.82439606414158[illegible]	0.93	0.854	7.76191483348811e-22	RPL3
Recoverec	0	5.37767652365705e-26	0.59066009610964[illegible]	0.411	0.155	1.8035651525041e-21	TMC5
Recoverec	0	6.42499749729269e-26	0.35514378729076[illegible]	0.293	0.084	2.15481566064202e-21	SLC6A19
Recoverec	0	1.63936954348617e-25	0.60664812410231[illegible]	0.556	0.269	5.49811757494393e-21	RBP2
Recoverec	0	5.48202869130394e-25	0.79868843474272[illegible]	0.915	0.806	1.83856278248951e-20	RPL19
Recoverec	0	5.66003544797426e-25	0.51827900854699[illegible]	0.522	0.246	1.89826268854161e-20	CYB5A
Recoverec	0	9.59322474983914e-25	0.44138264438770[illegible]	0.289	0.086	3.21737571660105e-20	ABCG1
Recoverec	0	1.18521824246094e-24	0.82474445582425[illegible]	0.952	0.912	3.97498494156551e-20	RPL13
Recoverec	0	1.35953471588869e-24	0.68053859558576[illegible]	0.633	0.305	4.55960753014747e-20	LAPTM5
Recoverec	0	1.56052409513048e-24	0.48502600376223	0.419	0.168	5.23368571024861e-20	SMIM24
Recoverec	0	1.95984101276337e-24	0.3646369023731	0.319	0.102	6.57291478860578e-20	STXBP6
Recoverec	0	4.19313939252619e-24	0.74822312597592[illegible]	0.881	0.755	1.40629508946543e-19	RPS5
Recoverec	0	5.9075414050873e-24	0.44647938154394[illegible]	0.411	0.165	1.98127123643818e-19	KRT20
Recoverec	0	6.00550169541322e-24	0.54468743399882	0.415	0.168	2.01412515860768e-19	AMN
Recoverec	0	6.30501204236239e-24	0.38172058302709[illegible]	0.444	0.182	2.1145749387675e-19	MME
Recoverec	0	6.38721155945092e-24	0.49205066814800[illegible]	0.485	0.221	2.14214301280865e-19	LGALS4
Recoverec	0	8.66585431509704e-24	0.39234944982175[illegible]	0.611	0.322	2.90635422019725e-19	MALRD1
Recoverec	0	1.35233506051159e-23	0.41565119123287	0.415	0.167	4.53546132594377e-19	EPB41L3
Recoverec	0	2.10892651003261e-23	0.70323417601085[illegible]	0.937	0.858	7.07291772934737e-19	RPL29
Recoverec	0	3.57572961591009e-23	0.46180958532097[illegible]	0.278	0.085	1.19922819858393e-18	MALL

Recoverec	0	8.18430004063765e-23	0.46475250905997	0.43	0.178	2.74485054762905e-18	C15orf48
Recoverec	0	8.25065372408331e-23	0.36636792306476	0.293	0.093	2.76710424598306e-18	AGR3
Recoverec	0	9.63038435961523e-23	0.2730517517166	0.226	0.057	3.22983830652776e-18	GUCA2B
Recoverec	0	1.00251677253601e-22	0.47978269620436	0.359	0.134	3.36224075173127e-18	NR1H4
Recoverec	0	1.2011100638903e-22	0.37040980244006	0.415	0.168	4.02828293227528e-18	ID1
Recoverec	0	1.40683372143315e-22	0.39097860204959	0.278	0.085	4.7182389349425e-18	MATN2
Recoverec	0	1.4414771151958e-22	0.52689205367416	0.681	0.372	4.83442594894367e-18	MYO1E
Recoverec	0	2.77051304588727e-22	0.57888619270924	0.793	0.594	9.29174665329674e-18	GAPDH
Recoverec	0	3.15685479546384e-22	0.68506682720231	0.926	0.842	1.05874596130266e-17	RPS9
Recoverec	0	3.60211394103847e-22	0.66985289491022	0.874	0.789	1.20807697354548e-17	RPLP0
Recoverec	0	4.20697248825303e-22	0.37027598874146	0.374	0.143	1.4109344331103e-17	NCKAP5
Recoverec	0	5.95263419593949e-22	0.28496091080074	0.196	0.046	1.99639445663419e-17	TM4SF5
Recoverec	0	6.47879962076679e-22	0.78675234274830	0.944	0.856	2.17285981681277e-17	RPS27A
Recoverec	0	6.94239265321618e-22	0.71786476350760	0.856	0.688	2.32833964803564e-17	EEF1D
Recoverec	0	7.78055534628975e-22	0.28412917694503	0.222	0.058	2.60944265203866e-17	SMIM22
Recoverec	0	7.95675238810559e-22	0.63183810043864	0.989	0.922	2.66853561592285e-17	RPL10
Recoverec	0	8.47188319022257e-22	0.49416220851115	0.378	0.152	2.84130018433685e-17	CDH1
Recoverec	0	9.06584261577721e-22	0.77430937068708	0.919	0.839	3.04050229647936e-17	RPS3
Recoverec	0	9.83477707433853e-22	0.41509852559760	0.519	0.243	3.29838753519166e-17	CDHR5
Recoverec	0	2.31356278343654e-21	0.448751187011917	0.304	0.104	7.75922686308946e-17	FRMD3
Recoverec	0	2.73602348996565e-21	0.74038693150375	0.881	0.756	9.1760755806468e-17	RPS21
Recoverec	0	2.81220143290482e-21	0.79472507125643	0.978	0.906	9.43156116567617e-17	RPS12
Recoverec	0	3.2724348347532e-21	0.68822587624152	0.963	0.915	1.09750919487953e-16	RPS2
Recoverec	0	3.5631106816029e-21	0.64010783324795	0.904	0.767	1.19499606039598e-16	RACK1
Recoverec	0	3.58524309683391e-21	0.61977549955801	0.93	0.844	1.20241882981616e-16	RPL7A
Recoverec	0	3.6769749942264e-21	0.45575058619488	0.333	0.125	1.23318387356365e-16	MEP1A
Recoverec	0	4.3535100340683e-21	0.73076073595367	0.948	0.887	1.46008019522583e-16	RPL12
Recoverec	0	4.43024679626579e-21	0.33581916517670	0.296	0.098	1.48581617053162e-16	CXADR
Recoverec	0	5.53393054363226e-21	0.31093943230345	0.185	0.043	1.85596962572339e-16	LIPH
Recoverec	0	5.72431121315769e-21	0.54303773499627	0.481	0.232	1.91981949466882e-16	C7orf50
Recoverec	0	5.7273794456986e-21	0.43159230247719	0.307	0.109	1.9208485184984e-16	HKDC1

Recoverec	0	6.39573286263459e-21	0.460590108301515	0.252	0.077	2.14500088747039e-16	B3GNT5
Recoverec	0	7.46203331281371e-21	0.712371915599134	0.885	0.734	2.50261673245146e-16	RPSA
Recoverec	0	1.01970874414493e-20	0.66967300400506	0.926	0.863	3.41989918611327e-16	RPS15
Recoverec	0	1.61158584817964e-20	0.656745937821098	0.911	0.84	5.40493661762487e-16	RPL36
Recoverec	0	1.66863716067281e-20	0.27143849652874	0.226	0.063	5.59627530946446e-16	GPRC5A
Recoverec	0	1.75327026408113e-20	0.659540997142375	0.9	0.837	5.8801178116753e-16	RPL37A
Recoverec	0	3.3572335399656e-20	0.342585913007556	0.3	0.103	1.12594898463366e-15	TFF3
Recoverec	0	5.08475850923712e-20	0.600452054936541	0.719	0.385	1.70532630882795e-15	HLA-DRB5
Recoverec	0	5.95500222726638e-20	0.661401191982751	0.93	0.851	1.9971886469806e-15	RPS16
Recoverec	0	6.27551459287255e-20	0.720624520108253	0.93	0.862	2.10468208415759e-15	RPL30
Recoverec	0	6.95276080426222e-20	0.30782290320011	0.281	0.093	2.33181691853346e-15	SPINK1
Recoverec	0	8.35211714152994e-20	0.622977645831706	0.911	0.84	2.80113304692631e-15	RPL35
Recoverec	0	9.18879106629316e-20	0.42437255428805	0.341	0.134	3.0817367478134e-15	ST14
Recoverec	0	1.57623555602249e-19	0.336167374735537	0.293	0.106	5.28637880778823e-15	MDK
Recoverec	0	2.0709320996843e-19	0.650090505106911	0.896	0.828	6.94549207592119e-15	RPL15
Recoverec	0	2.90391250944333e-19	0.654248859841168	0.952	0.895	9.73914177417104e-15	RPS6
Recoverec	0	3.1171623440661e-19	0.336415220007591	0.23	0.069	1.04543390695289e-14	PDK4
Recoverec	0	3.95869894989142e-19	0.629525624751293	0.904	0.819	1.32766845381458e-14	FAU
Recoverec	0	4.31897924673577e-19	0.702158997390119	0.93	0.87	1.44849925977024e-14	RPL37
Recoverec	0	4.47330833370743e-19	0.515287779451685	0.344	0.141	1.5002581489588e-14	COBL
Recoverec	0	8.88142830922287e-19	0.604880894140301	0.919	0.815	2.97865342634717e-14	RPL35A
Recoverec	0	9.5421621497945e-19	0.272862614454375	0.17	0.041	3.20025034179808e-14	KCNK5
Recoverec	0	1.30018511060755e-18	0.692905101878237	0.911	0.84	4.36056082395562e-14	RPL5
Recoverec	0	1.356206271239e-18	0.366911187452191	0.307	0.117	4.54844459248136e-14	MYO15B
Recoverec	0	1.54190688154506e-18	0.604913687582375	0.878	0.743	5.17124729932581e-14	EEF1B2
Recoverec	0	1.55910247096628e-18	0.514072949997067	0.404	0.185	5.22891786712671e-14	SGK1
Recoverec	0	2.01278229046734e-18	0.705443193894317	0.893	0.794	6.75046924576936e-14	RPL22
Recoverec	0	2.09673110871015e-18	0.582383262987181	0.97	0.903	7.03201679239209e-14	RPS18
Recoverec	0	2.38556103969957e-18	0.43675008090491	0.411	0.188	8.00069461494441e-14	C1orf21
Recoverec	0	3.41216571160976e-18	0.605371126421296	0.944	0.878	1.14437213635968e-13	RPL18
Recoverec	0	3.54034558678837e-18	0.442168243671636	0.307	0.117	1.18736110289708e-13	DAPK2

Recoverec	0	3.6672734029607e-18	0.29045685827456	0.27	0.093	1.22993015388496e-13	TMEM45B
Recoverec	0	3.8133123736754e-18	0.27153092592773	0.293	0.106	1.27890870388326e-13	CLDN7
Recoverec	0	4.3834365558218e-18	0.701114227533451	0.922	0.871	1.47011695209152e-13	RPL32
Recoverec	0	5.60617628080918e-18	0.45487770089742	0.319	0.126	1.88019940105778e-13	LHFPL2
Recoverec	0	5.78419613160499e-18	0.46682571612672	0.367	0.164	1.93990369861768e-13	PEPD
Recoverec	0	6.28403701441704e-18	0.56123340236188	0.856	0.7	2.10754033389519e-13	COX7C
Recoverec	0	7.4408076098394e-18	0.34770602892136	0.396	0.176	2.49549805618794e-13	NFIB
Recoverec	0	7.75699726381521e-18	0.3637050979534	0.422	0.198	2.60154174233835e-13	PLS1
Recoverec	0	8.53508569654559e-18	0.33170902801510	0.981	0.904	2.86249704090746e-13	FTL
Recoverec	0	1.14732547731755e-17	0.53514331007420	0.678	0.443	3.84790018582759e-13	LSM7
Recoverec	0	1.17964284380196e-17	0.32023189187312	0.278	0.1	3.95628616954302e-13	TMIGD1
Recoverec	0	1.47728752972552e-17	0.46821609978512	0.959	0.863	4.95452691719346e-13	RPS4X
Recoverec	0	1.56939763810981e-17	0.51836329463730	0.681	0.465	5.26344579869267e-13	PEBP1
Recoverec	0	1.68523881182129e-17	0.64359350375033	0.73	0.53	5.65195392708625e-13	HMGN2
Recoverec	0	1.98230553179032e-17	0.59605805101960	0.87	0.762	6.64825629251838e-13	RPL7
Recoverec	0	1.9838663639275e-17	0.46169137428033	0.541	0.303	6.65349101134006e-13	CIB1
Recoverec	0	2.90941170918747e-17	0.52958365844496	0.893	0.798	9.75758499027294e-13	UBA52
Recoverec	0	3.18747699927585e-17	0.38030600044498	0.333	0.143	1.06901603601713e-12	CTSD
Recoverec	0	3.616008154535e-17	0.26857029792198	0.219	0.068	1.21273681486795e-12	FAM3B
Recoverec	0	5.34904156890118e-17	0.55692153956690	0.867	0.571	1.79396156137808e-12	HLA-DRA
Recoverec	0	5.35724502501742e-17	0.52146506295679	0.374	0.174	1.79671283649034e-12	DCXR
Recoverec	0	5.47501167224143e-17	0.27632041408643	0.27	0.097	1.83620941463633e-12	KHK
Recoverec	0	7.76123456607267e-17	0.621199011059458	0.926	0.875	2.60296284876945e-12	RPS24
Recoverec	0	1.19170440373524e-16	0.61462570573840	0.922	0.81	3.99673822924726e-12	NACA
Recoverec	0	1.30728378416807e-16	0.59669027922119	0.93	0.851	4.38436835534288e-12	RPS25
Recoverec	0	1.76709054148181e-16	0.54293662861156	0.985	0.965	5.9264682580217e-12	EEF1A1
Recoverec	0	2.00875212720005e-16	0.30691767113929	0.189	0.055	6.73695288420354e-12	C6orf222
Recoverec	0	2.23649314933546e-16	0.48551719941685	0.922	0.854	7.50075072424125e-12	RPL24
Recoverec	0	2.72167148115777e-16	0.89768725530243	0.878	0.72	9.12794181350692e-12	TXNIP
Recoverec	0	2.72767386245282e-16	0.49916250620204	0.878	0.723	9.14807259989426e-12	PFDN5
Recoverec	0	2.76311194324847e-16	0.41858707102640	0.785	0.613	9.26692483526673e-12	SRP14

Recoverec	0	3.22991963968328e-16	0.631645681123211	0.926	0.876	1.08325044875698e-11	RPS3A
Recoverec	0	3.3448126614906e-16	0.262246120111574	0.248	0.088	1.12178327041072e-11	LGALS2
Recoverec	0	3.62203169539627e-16	0.49093924033166	0.689	0.435	1.214756990002e-11	ARHGDIB
Recoverec	0	3.85777405373574e-16	0.48586474720368З	0.87	0.749	1.29382026214189e-11	ACTG1
Recoverec	0	4.11883944531219e-16	0.494408792674527	0.781	0.603	1.3813763731688e-11	COX4I1
Recoverec	0	5.21309755356909e-16	0.48399360052482З	0.944	0.851	1.748368657516e-11	ACTB
Recoverec	0	5.63826626101914e-16	0.287715741351568	0.252	0.09	1.8909617386206e-11	TENT5A
Recoverec	0	5.84440643115948e-16	0.366552329682185	0.541	0.302	1.96009702888227e-11	SLC20A1
Recoverec	0	6.52608875674775e-16	0.384099485989734	0.678	0.472	2.18871964723806e-11	ATP5ME
Recoverec	0	6.77712764969139e-16	0.538476586907893	0.956	0.922	2.2729130711535e-11	RPL41
Recoverec	0	7.24728282523437e-16	0.44927807175187	0.359	0.166	2.4305937139271e-11	MYH14
Recoverec	0	7.55862063678367e-16	0.298336764811935	0.193	0.059	2.53501018916451e-11	FHL2
Recoverec	0	7.93510657722169e-16	0.606062228423794	0.904	0.829	2.66127604386861e-11	RPS13
Recoverec	0	8.90249764956025e-16	0.329339399582151	0.193	0.059	2.98571966170952e-11	NPAS2
Recoverec	0	1.04178646377836e-15	0.496882036470888	0.452	0.235	3.49394344221987e-11	FAM102A
Recoverec	0	1.07071466471159e-15	0.55557439335463	0.952	0.908	3.59096284250975e-11	RPS28
Recoverec	0	1.0968874518235e-15	0.270295051836097	0.781	0.414	3.67874113592565e-11	HLA-DPB1
Recoverec	0	1.24698148055448e-15	0.433567187650645	0.304	0.129	4.18212648948363e-11	TJP1
Recoverec	0	1.32668731508112e-15	0.278205033563434	0.296	0.121	4.44944391731907e-11	MYO7B
Recoverec	0	1.38930595288097e-15	0.251442913437027	0.2	0.063	4.65945430477221e-11	EFNA1
Recoverec	0	1.47019140399495e-15	0.265457680932532	0.237	0.084	4.93072793071827e-11	SLC2A2
Recoverec	0	1.73932018423228e-15	0.531069890864207	0.493	0.262	5.83333203387823e-11	IRF8
Recoverec	0	1.76820948115959e-15	0.27125808772608З	0.326	0.14	5.93022095791303e-11	FAT1
Recoverec	0	2.22944579973795e-15	0.503044737036812	0.863	0.763	7.47711532316113e-11	HLA-C
Recoverec	0	2.30797400350344e-15	0.302365634682577	0.222	0.076	7.74048321294985e-11	TNFRSF1A
Recoverec	0	2.5010555771628e-15	0.484143316282434	0.93	0.832	8.38804019468861e-11	RPL27A
Recoverec	0	2.50393750311257e-15	0.350801920639251	0.337	0.151	8.39770559793894e-11	ACE
Recoverec	0	2.99601271669715e-15	0.517850149983271	0.5	0.255	1.00480274492589e-10	LINC00926
Recoverec	0	3.68997919914071e-15	0.400718470808991	0.415	0.209	1.23754522380781e-10	GSTA1
Recoverec	0	4.79124502083613e-15	0.503466553470492	0.941	0.867	1.60688775508802e-10	RPL9
Recoverec	0	7.07393282599413e-15	0.296745944227078	0.319	0.136	2.37245559118191e-10	TBC1D8

Recoverec	0	7.47910048251852e-15	0.46456192074125¢	0.685	0.466	2.50834071982706e-10	COMMD6
Recoverec	0	8.23529005127698e-15	0.57747296234367	0.978	0.886	2.76195157739727e-10	RPL34
Recoverec	0	8.4546484242472e-15	0.51310609966362	0.963	0.888	2.83551998852403e-10	RPS14
Recoverec	0	9.89474280848694e-15	0.45021670316970	0.322	0.146	3.31849884311035e-10	SHROOM3
Recoverec	0	1.0671550102827e-14	0.54799321633797	0.933	0.893	3.57902447348614e-10	RPL13A
Recoverec	0	1.10456473176143e-14	0.41944094199676	0.326	0.145	3.70448919738149e-10	MGAM
Recoverec	0	1.30520118379642e-14	0.41997972522203	0.6	0.391	4.37738373021642e-10	PRR13
Recoverec	0	1.41763542350453e-14	0.51990748450357	0.904	0.798	4.75446568334948e-10	H3F3A
Recoverec	0	1.42827703356233e-14	0.40851501163683	0.263	0.105	4.79015551516133e-10	ABCA1
Recoverec	0	1.45796118894891e-14	0.39544147821863	0.385	0.194	4.88971023549687e-10	MIF
Recoverec	0	1.49815801531842e-14	0.48316653737283	0.893	0.689	5.0245223517749e-10	MTRNR2L12
Recoverec	0	1.66429996529136e-14	0.42573815923463	0.893	0.748	5.58172922359417e-10	HLA-A
Recoverec	0	1.84294514081438e-14	0.54959155049279	0.874	0.783	6.18086941326326e-10	RPL26
Recoverec	0	1.940144718359e-14	0.35334981275675	0.348	0.161	6.50685735643242e-10	PLEKHA7
Recoverec	0	2.69198588766062e-14	0.45282637117869	0.504	0.275	9.02838227003617e-10	HLA-DMA
Recoverec	0	2.71427000418973e-14	0.54567880317834	0.819	0.658	9.10311874005151e-10	RPL4
Recoverec	0	2.87663743032901e-14	0.56528157003200	0.941	0.883	9.64766661383742e-10	RPS15A
Recoverec	0	3.01405028529443e-14	0.38846748243156	0.27	0.11	1.01085218468205e-09	GNG12
Recoverec	0	3.35696808569542e-14	0.29733881132775	0.341	0.156	1.12585995658053e-09	PTGR1
Recoverec	0	4.04853646402059e-14	0.45287455380004	0.396	0.193	1.35779815930323e-09	FCMR
Recoverec	0	4.17122224610558e-14	0.32144146381020	0.296	0.127	1.39894451689889e-09	ABTB2
Recoverec	0	4.48783791054421e-14	0.31412129613860	0.222	0.081	1.50513107843832e-09	BTNL3
Recoverec	0	5.48954994599423e-14	0.37858746853434	0.311	0.143	1.84108526088754e-09	CDH17
Recoverec	0	5.62959775499918e-14	0.27744949808449	0.219	0.079	1.88805449507162e-09	SLC10A2
Recoverec	0	7.24419472288567e-14	0.32774828232496	0.641	0.403	2.4295580261614e-09	ATP1B3
Recoverec	0	7.25893624632313e-14	0.49806519203029	0.948	0.904	2.43450203829185e-09	RPLP2
Recoverec	0	7.89007543233844e-14	0.42881629097739	0.622	0.426	2.64617349849767e-09	CHCHD10
Recoverec	0	7.99355410048581e-14	0.50430284083809	0.619	0.368	2.68087817422093e-09	CEMIP2
Recoverec	0	8.19719958908233e-14	0.42550239926487	0.233	0.087	2.74917679818643e-09	TNFSF9
Recoverec	0	8.74385774068648e-14	0.68386288322770	0.567	0.366	2.93251500907143e-09	H1FX
Recoverec	0	1.03640422195025e-13	0.28131264975834	0.285	0.122	3.47589247957673e-09	HEPH

Recoverec	0	1.15092886433192e-13	0.489653363789378	0.419	0.233	3.8599852251964e-09	EIF1AY
Recoverec	0	1.18034667933032e-13	0.461892529025793	0.667	0.476	3.95864669313802e-09	CNBP
Recoverec	0	1.42397364226347e-13	0.314692564404973	0.574	0.362	4.77572280142323e-09	PIGR
Recoverec	0	1.67605223784853e-13	0.347989955297753	0.244	0.097	5.62114399529639e-09	GRAMD1B
Recoverec	0	2.24564932769267e-13	0.331327000676795	0.748	0.448	7.53145871521566e-09	HLA-DQB1
Recoverec	0	2.75592008412988e-13	0.429203058395893	0.411	0.217	9.24280477815479e-09	ABCB1
Recoverec	0	3.15661631056869e-13	0.421098338692383	0.604	0.423	1.05866597823853e-08	ATP5F1D
Recoverec	0	3.59621372990383e-13	0.574237246958652	0.348	0.167	1.20609816073515e-08	IGHD
Recoverec	0	4.15446093920057e-13	0.383785280952285	0.722	0.444	1.39332310978909e-08	HLA-DPA1
Recoverec	0	4.94399465615461e-13	0.294790299373517	0.315	0.144	1.65811692778113e-08	TRAPPC6A
Recoverec	0	5.25637674726157e-13	0.452040363211529	0.889	0.764	1.76288363349658e-08	RPL23
Recoverec	0	5.47121415180623e-13	0.310451219481054	0.296	0.134	1.83493580223277e-08	ETS2
Recoverec	0	5.62353896223644e-13	0.684804116101898	0.693	0.454	1.88602249715486e-08	ZNF331
Recoverec	0	5.86054273755896e-13	0.409954789140254	0.807	0.683	1.96550882332252e-08	MYL6
Recoverec	0	6.46460703300808e-13	0.298615702228712	0.433	0.238	2.16809990673025e-08	DGAT1
Recoverec	0	6.63731565232133e-13	0.354811776339064	0.485	0.283	2.22602292347553e-08	IL32
Recoverec	0	7.61294462354283e-13	0.357086967879308	0.441	0.254	2.55322936784379e-08	FIS1
Recoverec	0	8.27062095598434e-13	0.453912438131118	0.767	0.521	2.77380085621803e-08	CD37
Recoverec	0	8.48992930478986e-13	0.450247731628997	0.441	0.259	2.84735249024042e-08	ANXA11
Recoverec	0	9.01493470626073e-13	0.355353077525661	0.567	0.369	3.02342880178572e-08	DDT
Recoverec	0	1.03135681663687e-12	0.407857879488231	0.293	0.135	3.45896449163672e-08	H2AFJ
Recoverec	0	1.04387542383836e-12	0.545168879935131	0.922	0.876	3.50094939646911e-08	RPS23
Recoverec	0	1.04857497781111e-12	0.453058784472164	0.904	0.802	3.51671076058291e-08	RPL23A
Recoverec	0	1.08328652498066e-12	0.44555337459356	0.752	0.553	3.63312634748015e-08	RPS10
Recoverec	0	1.21221930361017e-12	0.322402633830675	0.189	0.066	4.06554110044777e-08	AC093010.2
Recoverec	0	1.2689509028616e-12	0.286919933059841	0.241	0.099	4.25580753801723e-08	AGPAT2
Recoverec	0	1.28170436846964e-12	0.391209486970989	0.322	0.156	4.29858011097349e-08	POR
Recoverec	0	1.28610477943536e-12	0.309813609976151	0.941	0.87	4.3133382092703e-08	TMSB10
Recoverec	0	1.33319862876823e-12	0.282998625848641	0.448	0.24	4.47128156116289e-08	COTL1
Recoverec	0	1.34188205658202e-12	0.350969752431384	0.881	0.782	4.50040404136476e-08	SERF2
Recoverec	0	1.36355598754174e-12	0.287121670326558	0.307	0.143	4.57309407101748e-08	MTTP

Recoverec	0	1.37406455968484e-12	0.31497217973793	0.441	0.248	4.60833772027102e-08	MAGI1
Recoverec	0	1.41962441748333e-12	0.55185946252870	0.904	0.852	4.7611363713556e-08	RPL14
Recoverec	0	1.42302533937842e-12	0.34314033373802	0.3	0.137	4.77254238320733e-08	IRS2
Recoverec	0	1.5828324459027e-12	0.28993267402145	0.281	0.126	5.30850345706847e-08	ERRFI1
Recoverec	0	1.69599432840112e-12	0.28782312696031	0.807	0.505	5.68802577859168e-08	CXCR4
Recoverec	0	1.84436128470526e-12	0.28460201141349	0.419	0.228	6.18561887664451e-08	SOX6
Recoverec	0	1.89625590548287e-12	0.30552381757399	0.419	0.243	6.35966305580844e-08	FCGRT
Recoverec	0	2.43952233251677e-12	0.36326223569851	0.626	0.416	8.18166999879473e-08	SH3BGRL3
Recoverec	0	2.47078127308571e-12	0.39015002848511	0.87	0.752	8.28650623367487e-08	RPL27
Recoverec	0	2.56756704776271e-12	0.35082500928615	0.522	0.311	8.61110636478659e-08	SEPT9
Recoverec	0	2.60899878886048e-12	0.40599713940195	0.826	0.671	8.75006013808029e-08	CIRBP
Recoverec	0	3.43787026706704e-12	0.40528095781770	0.789	0.621	1.15299293016894e-07	HNRNPA1
Recoverec	0	3.45876094709831e-12	0.44648962055	0.981	0.941	1.15999924643783e-07	RPLP1
Recoverec	0	3.54367319884344e-12	0.43712517968147	0.544	0.352	1.18847711742811e-07	EIF3L
Recoverec	0	4.31478249728281e-12	0.40548532573009	0.663	0.479	1.44709175393871e-07	EIF3K
Recoverec	0	5.25684505087616e-12	0.31471151144827	0.815	0.698	1.76304069316285e-07	ATP5F1E
Recoverec	0	5.38363595966548e-12	0.47991364251004	0.374	0.205	1.80556382815261e-07	HMGB2
Recoverec	0	5.47159901593523e-12	0.25921826789576	0.426	0.231	1.83506487796436e-07	DRAM2
Recoverec	0	6.00516040761487e-12	0.45235625749093	0.819	0.684	2.01401069750587e-07	BTF3
Recoverec	0	7.32413154881119e-12	0.44063203033723	0.6	0.418	2.4563672388403e-07	UXT
Recoverec	0	7.32578004890463e-12	0.32573750800438	0.244	0.103	2.45692011280163e-07	KIAA1211
Recoverec	0	8.01708188544585e-12	0.39613224881354	0.837	0.687	2.68876892274083e-07	HINT1
Recoverec	0	9.14269476896221e-12	0.32288162450420	0.604	0.426	3.06627697161455e-07	NDUFA4
Recoverec	0	1.02907014545312e-11	0.51911690980684	0.356	0.185	3.45129545382067e-07	RASSF6
Recoverec	0	1.115910248925e-11	0.34401786032270	0.741	0.567	3.74253979284465e-07	UQCRH
Recoverec	0	1.17995719404753e-11	0.27572722208862	0.193	0.072	3.95734043739662e-07	FAM160A1
Recoverec	0	1.30327594456623e-11	0.44659195802275	0.833	0.705	4.37092686288621e-07	RPL31
Recoverec	0	1.57083439330983e-11	0.30045010201402	0.285	0.133	5.26826438828252e-07	SCIN
Recoverec	0	1.67717938298026e-11	0.28428974035502	0.204	0.079	5.62492421463919e-07	EPS8L3
Recoverec	0	1.92850443680065e-11	0.45256896553871	0.593	0.403	6.46781818014201e-07	BLK
Recoverec	0	2.03559822168404e-11	0.34978370318350	0.47	0.29	6.82698931588393e-07	VPS28

Recoverec	0	2.19904355767762e-11	0.25720852698077[illegible]	0.315	0.157	7.3751522837392e-07	FKBP8
Recoverec	0	2.36052509539072e-11	0.45501405364368[illegible]	0.6	0.413	7.91672906492141e-07	CYSTM1
Recoverec	0	2.73855808793257e-11	0.29170565086649[illegible]	0.307	0.151	9.18457611530826e-07	EIF6
Recoverec	0	2.97716853227382e-11	0.36829700926812[illegible]	0.685	0.391	9.98482782353995e-07	MS4A1
Recoverec	0	3.18059803244535e-11	0.33975221433857[illegible]	0.848	0.631	1.06670896812152e-06	SMAP2
Recoverec	0	3.29031779820839e-11	0.28638021177008[illegible]	0.393	0.207	1.10350678316313e-06	TRBC2
Recoverec	0	3.29711169029069e-11	0.27049080452140[illegible]	0.97	0.884	1.10578531868969e-06	TMSB4X
Recoverec	0	3.65044355583648e-11	0.38985674756237[illegible]	0.796	0.607	1.22428575975644e-06	PFN1
Recoverec	0	3.7604607814834e-11	0.26604909424603[illegible]	0.641	0.44	1.2611833368939e-06	MYL12B
Recoverec	0	3.91044550264157e-11	0.25675855710239[illegible]	0.263	0.118	1.31148521267593e-06	SLC5A12
Recoverec	0	4.92137492011e-11	0.25823072638516[illegible]	0.2	0.078	1.65053072070649e-06	MYRFL
Recoverec	0	5.05877691359657e-11	0.323817111737971	0.441	0.26	1.69661260128202e-06	EPS8
Recoverec	0	5.1473568168816e-11	0.28894619422392[illegible]	0.33	0.164	1.72632052924575e-06	ARPC4
Recoverec	0	5.30432860975719e-11	0.42284839001254[illegible]	0.922	0.871	1.77896572914037e-06	RPS11
Recoverec	0	5.47490209531785e-11	0.35482462455392[illegible]	0.504	0.315	1.8361726647277e-06	CCDC6
Recoverec	0	5.64127879386438e-11	0.35205004021688[illegible]	0.537	0.357	1.89197208188623e-06	RNASET2
Recoverec	0	5.73751027977098e-11	0.32366544912513	0.396	0.23	1.92424619762959e-06	NDUFB1
Recoverec	0	5.94549036596208e-11	0.59463553868942[illegible]	0.515	0.334	1.99399855893636e-06	EIF1B
Recoverec	0	6.02794575262476e-11	0.37708627748432[illegible]	0.852	0.743	2.02165244651529e-06	RPL38
Recoverec	0	6.62299411881761e-11	0.36021317610415	0.648	0.485	2.22121976756905e-06	COX5B
Recoverec	0	7.550516328888e-11	0.38006337005470[illegible]	1	0.955	2.53229216638246e-06	TPT1
Recoverec	0	7.6320715975177e-11	0.32152841641403[illegible]	0.47	0.294	2.55964417237548e-06	ATP5PO
Recoverec	0	8.24660021107903e-11	0.26268775627507[illegible]	0.215	0.088	2.76574477879169e-06	AC119396.1
Recoverec	0	8.74208838625408e-11	0.40608867225106	0.659	0.47	2.93192160298189e-06	ATP1A1
Recoverec	0	9.85387227497522e-11	0.29638724878664[illegible]	1	0.992	3.30479168358119e-06	MT-ND4
Recoverec	0	1.00570037031086e-10	0.25306991813983[illegible]	0.552	0.37	3.37291790194857e-06	COX6A1
Recoverec	0	1.01828642193249e-10	0.32910521403106[illegible]	0.63	0.455	3.41512900187718e-06	RBM3
Recoverec	0	1.09469594955708e-10	0.27922372691740[illegible]	0.519	0.302	3.67139127562453e-06	TNFRSF13C
Recoverec	0	1.21894539037891e-10	0.25926211949950[illegible]	0.789	0.533	4.08809905025279e-06	BACH2
Recoverec	0	1.28035972340379e-10	0.33220342308200[illegible]	0.8	0.643	4.29407044035163e-06	CFL1
Recoverec	0	1.34489726671094e-10	0.34063994367847[illegible]	0.263	0.121	4.51051645309515e-06	LRRC75A

Recoverec	0	1.46887096444106e-10	0.27016589605934	0.526	0.345	4.92629944054243e-06	WDR83OS
Recoverec	0	1.59258611591991e-10	0.26079863898952	0.504	0.332	5.34121531557221e-06	UQCR11
Recoverec	0	1.62361294598325e-10	0.36893335623644	0.463	0.296	5.44527309823863e-06	ACTN4
Recoverec	0	1.62641797147987e-10	0.37288486998116	0.474	0.299	5.4546805927492e-06	FGD4
Recoverec	0	1.81618028800504e-10	0.41809157962069	0.481	0.288	6.09110544991129e-06	ABLIM1
Recoverec	0	1.84573899736273e-10	0.30828345143670	0.711	0.531	6.19023944935511e-06	ANAPC16
Recoverec	0	1.96088527250474e-10	0.25299737224541	0.17	0.062	6.5764170269264e-06	CNR1
Recoverec	0	2.58249287399745e-10	0.37640025186969	0.433	0.252	8.66116460081266e-06	PLAC8
Recoverec	0	2.75902015059595e-10	0.39194662496555	0.715	0.523	9.25320178106871e-06	CD79A
Recoverec	0	2.82684980037817e-10	0.31501557660328	0.515	0.341	9.48068886050829e-06	SMDT1
Recoverec	0	3.13235853430978e-10	0.33638394077344	0.522	0.324	1.05053040523681e-05	MBP
Recoverec	0	3.22775610114183e-10	0.31787137084613	0.256	0.12	1.08252484120095e-05	IGF2BP2
Recoverec	0	3.28169398965283e-10	0.34189630182754	0.744	0.572	1.10061453024977e-05	ATP5MC2
Recoverec	0	3.93750307956232e-10	0.29309237742185	0.4	0.234	1.32055978282361e-05	JPT1
Recoverec	0	5.09803664356191e-10	0.32015997200747	0.53	0.346	1.70977952951779e-05	STK24
Recoverec	0	5.32104592709466e-10	0.33165607313504	0.804	0.659	1.78457238302901e-05	TOMM7
Recoverec	0	5.32630925458984e-10	0.26250927097959	0.348	0.197	1.78633759780434e-05	CALM3
Recoverec	0	5.87974829044795e-10	0.27512405308180	0.444	0.279	1.97194998165043e-05	ALKBH7
Recoverec	0	6.1425116123677e-10	0.31670784476559	0.263	0.13	2.06007554455588e-05	CES2
Recoverec	0	6.15817016427131e-10	0.35380031150634	0.648	0.47	2.06532710969331e-05	NOP53
Recoverec	0	6.48988800730251e-10	0.39128557739011	0.411	0.25	2.17657863988912e-05	GLO1
Recoverec	0	6.59523439909219e-10	0.41463500470775	0.959	0.928	2.21190971276754e-05	RPL39
Recoverec	0	6.61698061097426e-10	0.62739467349354	0.904	0.797	2.21920295730855e-05	BTG1
Recoverec	0	6.99535017462732e-10	0.32618407563870	0.193	0.076	2.34610054156651e-05	LINC01811
Recoverec	0	8.04127154850257e-10	0.26772437104612	0.752	0.546	2.69688165193679e-05	ACTR2
Recoverec	0	9.73737541820406e-10	0.33395277441102	0.952	0.864	3.26572096775728e-05	HLA-B
Recoverec	0	1.09799125259883e-09	0.2871084968966	0.796	0.636	3.68244306296594e-05	PPIA
Recoverec	0	1.23016227997841e-09	0.43746447771749	0.663	0.489	4.12571825459159e-05	KIAA1551
Recoverec	0	1.26607516282702e-09	0.27879192788802	0.959	0.822	4.24616288108927e-05	EZR
Recoverec	0	1.30188889791346e-09	0.31194150919086	0.478	0.296	4.36627498582217e-05	CD79B
Recoverec	0	1.33389384584827e-09	0.32544431528451	0.811	0.672	4.47361318020593e-05	YBX1

Recoverec	0	1.36461891674987e-09	0.28078237432724€	0.381	0.227	4.5766589229957e-05	SLC9A3R1
Recoverec	0	1.40243858184757e-09	0.31654468715466€	0.815	0.622	4.70349851580039e-05	HLA-E
Recoverec	0	1.50442218293878e-09	0.27785405775306€	0.567	0.384	5.04553111714007e-05	NEDD4L
Recoverec	0	1.61829335245256e-09	0.26310601849361£	1	1	5.42743224545541e-05	MT-ATP6
Recoverec	0	1.77403443946546e-09	0.25565810654063⁴	0.344	0.184	5.94975670307925e-05	TLE1
Recoverec	0	1.79809511609287e-09	0.30799794113102	0.222	0.102	6.03045140035226e-05	MS4A8
Recoverec	0	1.87618167469807e-09	0.34182627431583	0.437	0.283	6.2923381006024e-05	PSMD8
Recoverec	0	1.9467289760628e-09	0.38127765016010⁄	0.615	0.456	6.52893963991942e-05	RPS17
Recoverec	0	1.97345771475751e-09	0.52718798085015€	0.522	0.365	6.61858248375374e-05	RCSD1
Recoverec	0	2.00811521776311e-09	0.27281058379576€	0.481	0.311	6.73481681733391e-05	APRT
Recoverec	0	2.02482604087646e-09	0.31011088822393€	0.315	0.173	6.79086157589147e-05	KRT18
Recoverec	0	2.04769973179079e-09	0.45320475432221€	0.537	0.37	6.86757536047996e-05	HSPA4
Recoverec	0	2.11141928847065e-09	0.26792728039885ź	0.389	0.226	7.08127800967287e-05	CXXC5
Recoverec	0	2.14001563646506e-09	0.28717984409878€	0.474	0.303	7.17718444157652e-05	AES
Recoverec	0	2.16531482857358e-09	0.35360913953308€	0.6	0.427	7.26203287207006e-05	SNHG8
Recoverec	0	2.30596350771676e-09	0.31494804750193	0.704	0.552	7.73374041218046e-05	CALM2
Recoverec	0	2.31608083467856e-09	0.29614923366627€	0.83	0.71	7.76767190334495e-05	CALM1
Recoverec	0	2.49823056207124e-09	0.27887506871429€	0.411	0.251	8.37856565907451e-05	ATP6V1F
Recoverec	0	2.49911453961788e-09	0.26693487779529	0.233	0.11	8.38153034297045e-05	PRKCZ
Recoverec	0	2.70894069188919e-09	0.25708469289514€	0.711	0.476	9.08524529245798e-05	SNX9
Recoverec	0	3.57734765135861e-09	0.26317491885894	0.363	0.211	0.000119977085531265	UQCRFS1
Recoverec	0	4.02841645000341e-09	0.40834213743531	0.9	0.851	0.000135105030900214	RPS20
Recoverec	0	4.7394842456855e-09	0.29192706063572€	0.504	0.332	0.0001589528226318	SYF2
Recoverec	0	4.89716092871538e-09	0.32180691296925€	0.604	0.427	0.000164240983227257	EIF3F
Recoverec	0	5.28512756358417e-09	0.27806140241203£	0.526	0.365	0.000177252608227486	RIOK3
Recoverec	0	5.95863264578354e-09	0.25198394483099	0.315	0.172	0.000199840621674288	TMEM238
Recoverec	0	6.23963884814092e-09	0.25886283716181€	0.356	0.202	0.00020926500768895	ARHGAP32
Recoverec	0	6.47155040274785e-09	0.35461371180724€	0.622	0.468	0.000217042857407358	HMGN1
Recoverec	0	6.68749797541531e-09	0.29405874821221⁴	0.385	0.213	0.000224285307099479	LY86
Recoverec	0	7.3287297694494e-09	0.40753943461265€	0.559	0.377	0.000245790939007794	SAMSN1
Recoverec	0	7.89502720112573e-09	0.30122222169670	0.526	0.378	0.000264783422271355	ATP5MC3

Recoverec	0	7.89591166801773e-09	0.31047954606208€	0.496	0.336	0.000264813085521979	EIF3G
Recoverec	0	8.28206256968456e-09	0.26386096216745ε	0.419	0.253	0.000277763814462081	MAGI3
Recoverec	0	9.10581914605701e-09	0.34407111657601	0.241	0.12	0.00030539096252046	CRIP1
Recoverec	0	1.00955594424345e-08	0.27287182455157′	0.515	0.364	0.000338584872580368	MGST3
Recoverec	0	1.02501933076409e-08	0.43879550599528:	0.544	0.398	0.00034377098315166	MZT2B
Recoverec	0	1.05020564495824e-08	0.31225376438846:	0.526	0.322	0.000352217969206093	FCRL1
Recoverec	0	1.09599238892586e-08	0.29372439138099ξ	0.381	0.231	0.000367573927397955	FBL
Recoverec	0	1.10637184474233e-08	0.29861875002066ε	0.5	0.343	0.000371054989289683	ANXA2
Recoverec	0	1.19583098922266e-08	0.30678213015509ξ	0.389	0.236	0.000401057797165495	MARCKSL1
Recoverec	0	1.26403756032287e-08	0.25904248703671:	0.374	0.227	0.000423932916981084	HSD17B11
Recoverec	0	1.53952175228669e-08	1.52195751252079	0.115	0.038	0.000516324805281909	PYY
Recoverec	0	1.58983795950565e-08	0.29933655914131′	0.678	0.485	0.000533199854859004	CD53
Recoverec	0	1.65579811661581e-08	0.33370498273500′	0.444	0.279	0.00055532157235061	TPK1
Recoverec	0	1.66818361172054e-08	0.42058121728955⁊	0.526	0.362	0.000559475419698835	ETS1
Recoverec	0	1.84676407328918e-08	0.29095763090975ξ	0.37	0.223	0.000619367734899726	REX1BD
Recoverec	0	1.85545282440885e-08	0.25176790376797ξ	0.563	0.397	0.00062228176825024	PSMA7
Recoverec	0	2.24687081024939e-08	0.26482209449412	0.6	0.432	0.000753555532341441	ERP29
Recoverec	0	2.54285992801106e-08	0.31974932620522ξ	0.537	0.366	0.000852824362656348	POU2F2
Recoverec	0	2.67285375743527e-08	0.29580577148041ε	0.722	0.59	0.000896421693168642	TMBIM6
Recoverec	0	2.75126392189575e-08	0.29437137563064:	0.511	0.367	0.000922718894125396	ARL6IP4
Recoverec	0	3.04819678110287e-08	0.28031802403655ξ	0.574	0.425	0.00102230423644628	SLC25A3
Recoverec	0	3.16953163677464e-08	0.29712277235633:	0.593	0.439	0.00106299752034148	SAP18
Recoverec	0	3.55957607452866e-08	0.33954054523771€	0.522	0.358	0.00119381062387542	PDLIM1
Recoverec	0	3.64761585608261e-08	0.26830792532817′	0.507	0.315	0.00122333740581298	MARCH1
Recoverec	0	4.02862274094595e-08	0.34260426891559ς	0.667	0.53	0.00135111949485845	VMP1
Recoverec	0	4.18738358060085e-08	0.26895438597730€	0.574	0.42	0.00140436470526191	ST13
Recoverec	0	4.34091765422082e-08	0.28759905342691⁄	0.541	0.41	0.00145585696287258	UQCRQ
Recoverec	0	4.34847736186673e-08	0.31621354331161⁄	0.415	0.278	0.00145839233762286	VAMP8
Recoverec	0	4.60790424678662e-08	0.302889314411767	0.944	0.858	0.0015453989262873	CD74
Recoverec	0	4.76103437586141e-08	0.30848842404586:	0.437	0.294	0.0015967557089764	LAMTOR4
Recoverec	0	7.13782420062039e-08	0.25312734144316ς	0.126	0.047	0.00239388348040407	IPCEF1

Recoverec	0	8.55358652076969e-08	0.25742099300516	0.33	0.199	0.00286870184733574	CLTB
Recoverec	0	9.34955663074836e-08	0.25218496966493	0.493	0.366	0.00313565430282038	ELOB
Recoverec	0	1.00267060190664e-07	0.40580019782616	0.411	0.269	0.00336275666467448	GNA12
Recoverec	0	1.10973719307669e-07	0.31093350913627	0.27	0.151	0.00372183659814059	MYO5B
Recoverec	0	1.15556497483742e-07	0.36226949364205	0.344	0.21	0.00387553381260973	UCP2
Recoverec	0	1.34185922954375e-07	0.34673742075499	0.415	0.268	0.00450032748404383	GSAP
Recoverec	0	1.42262488804099e-07	0.37403652682983	0.719	0.551	0.00477119934951186	BTBD9
Recoverec	0	1.50081514426112e-07	0.42451246744109	0.719	0.61	0.00503343383082295	EEF2
Recoverec	0	1.51394270501546e-07	0.76242735199292	0.167	0.076	0.00507746104408085	PCDH9
Recoverec	0	1.6523007716952e-07	0.57305687167272	0.493	0.353	0.00554148632811135	USP36
Recoverec	0	1.87166615118457e-07	0.27001475736739	0.181	0.084	0.00627719393784282	AL157786.1
Recoverec	0	2.075404477452e-07	0.30234777105463	0.474	0.338	0.00696049153647851	GPX4
Recoverec	0	2.37715479936022e-07	0.32234957433383	0.289	0.163	0.00797250176609429	SNX8
Recoverec	0	2.73940424898854e-07	0.25536542045327	0.637	0.489	0.00918741397025776	MYL12A
Recoverec	0	2.9543867658091e-07	0.28026015667047	0.496	0.369	0.00990842233517055	RAC1
Recoverec	0	3.34903487706065e-07	0.27166492235788	0.793	0.631	0.011231993170686	JAK1
Recoverec	0	3.49627473439782e-07	0.30947649740624	0.296	0.16	0.0117258062042234	CD96
Recoverec	0	3.62716291660845e-07	0.32227111442209	0.43	0.299	0.0121647789897214	EIF3D
Recoverec	0	3.64076744175658e-07	0.30045581779055	0.378	0.238	0.0122104058461632	DEK
Recoverec	0	4.94981517500168e-07	0.25633971547107	0.396	0.255	0.0166006901339206	CACYBP
Recoverec	0	5.30980674457254e-07	0.28581086181294	0.289	0.17	0.0178080298599474	MAOA
Recoverec	0	6.06239493554279e-07	0.28740181806462	0.293	0.175	0.0203320601348234	AFG3L2
Recoverec	0	6.49598856658805e-07	0.26148226585757	0.263	0.149	0.021786246454623	NDUFS7
Recoverec	0	6.8116342836588e-07	0.27171120472393	0.719	0.556	0.0228448590605349	EXT1
Recoverec	0	8.00622680604056e-07	0.25938584838348	0.207	0.107	0.0268512834620988	MACROD2
Recoverec	0	8.41173846042739e-07	0.31621451994693	0.174	0.084	0.0282112884485814	FGD6
Recoverec	0	8.65913851205917e-07	0.26993457300557	0.2	0.101	0.029041018741744	ZNF831
Recoverec	0	9.01414237158471e-07	0.25283701778390	0.252	0.143	0.0302316306858208	PLEC
Recoverec	0	9.2226119003398e-07	0.26894502006056	0.637	0.48	0.0309307957913596	INPP5D
Recoverec	0	9.68188095389948e-07	0.28568408831653	0.685	0.545	0.0324710923431881	EIF3E
Recoverec	0	1.09848142178314e-06	0.28024953428564	0.507	0.375	0.0368408699237628	NDUFB11

Recoverec	0	1.12283144959528e-06	0.259268144430695	0.167	0.077	0.0376575211565266	LY86-AS1
Recoverec	0	1.21466166847937e-06	0.251036981383519	0.396	0.262	0.040737323037461	STK39
Recoverec	0	1.24265117809441e-06	0.263311966387439	0.474	0.335	0.0416760352109302	UBE2I
Recoverec	0	1.25654314119989e-06	0.265313142168865	0.611	0.452	0.042141943869562	CCND3
Recoverec	0	1.26823638367062e-06	0.250096449555308	0.4	0.269	0.0425341118355453	RAB14
Recoverec	0	1.32682860737821e-06	0.350890335773148	0.415	0.287	0.0444991778342503	HIST1H1C
Recoverec	13	2.28142019934982e-26	0.562937592582665	0.567	0.26	7.65142706457943e-22	CORO1A
Recoverec	13	7.81718936017115e-24	0.914853705310775	0.776	0.55	2.6217289676142e-19	RPS10
Recoverec	13	1.81184115886802e-22	0.539053996678656	0.69	0.417	6.07655287861155e-18	PPDPF
Recoverec	13	8.08547098707313e-22	1.05026612875358	0.892	0.809	2.71170525964459e-17	RPL19
Recoverec	13	8.17086965445735e-22	0.769536724115421	0.918	0.784	2.74034626471191e-17	RPLP0
Recoverec	13	6.78623253076003e-21	0.867609200975331	0.907	0.78	2.2759666661663e-16	JUNB
Recoverec	13	8.25019905269443e-20	0.589532308133772	0.66	0.412	2.76695175829266e-15	SH3BGRL3
Recoverec	13	1.17355868523374e-19	0.808582463183515	0.918	0.846	3.9358811185369e-15	RPL7A
Recoverec	13	2.16153512756719e-19	0.978197370260443	0.56	0.311	7.24935651083485e-15	DUSP2
Recoverec	13	3.77322543402383e-19	0.55718891564241	0.272	0.09	1.26546434606291e-14	TCL1A
Recoverec	13	1.26180220649602e-18	0.637485938657503	0.41	0.182	4.23183224014634e-14	HLA-DQA2
Recoverec	13	5.10063431691322e-18	0.333273166957025	0.321	0.125	1.71065073720636e-13	ARPC1B
Recoverec	13	9.73664120766596e-18	0.820414835416953	0.955	0.912	3.26547472822701e-13	RPL13
Recoverec	13	4.9195759499434e-17	0.415901050257203	0.444	0.214	1.64992738209202e-12	SOCS3
Recoverec	13	6.40089097848876e-17	0.620131220115718	0.884	0.72	2.14673081636556e-12	CYBA
Recoverec	13	1.22420295255129e-16	0.658867476962327	0.929	0.852	4.10573186226653e-12	RPL8
Recoverec	13	1.30504409547884e-16	0.488494542434693	0.836	0.588	4.37685688741695e-12	GAPDH
Recoverec	13	1.37780962534353e-16	0.387151449200303	0.534	0.305	4.62089792147713e-12	APRT
Recoverec	13	1.71269180328443e-16	0.542363029529032	0.504	0.268	5.74402576985533e-12	SERTAD1
Recoverec	13	1.92441599051769e-16	0.558781550049191	0.854	0.689	6.45410634899822e-12	PNRC1
Recoverec	13	8.87813365072201e-16	0.533988150055185	0.698	0.464	2.97754846377915e-11	NOP53
Recoverec	13	2.96826093617582e-15	0.673388960599033	0.94	0.841	9.95495352774648e-11	RPS9
Recoverec	13	3.38262662492161e-15	0.698035866013057	0.948	0.927	1.13446531746621e-10	RPL10
Recoverec	13	6.52782406283622e-15	0.426708252804582	0.616	0.371	2.18930163419401e-10	MAP3K8
Recoverec	13	8.10087950576616e-15	0.69527945392726	0.925	0.855	2.71687296864385e-10	RPL3

Recoverec	13	1.0910447103704e-14	0.77329227079315	0.914	0.839	3.65914574964026e-10	RPS3
Recoverec	13	1.12414079224599e-14	0.44923938856665	0.59	0.367	3.77014338903459e-10	EVI2B
Recoverec	13	1.16806085174283e-14	0.34317623565154	0.384	0.182	3.9174424845751e-10	CDKN2AIP
Recoverec	13	1.61642280157968e-14	0.822113205542997	0.858	0.773	5.42115879193793e-10	RACK1
Recoverec	13	2.07942974650884e-14	0.86938587961223	0.496	0.294	6.97399148384135e-10	LTB
Recoverec	13	2.42799537319011e-14	0.39409885371103	0.519	0.296	8.14301088260501e-10	UBXN1
Recoverec	13	2.4786314908011e-14	0.52208416915384	0.799	0.607	8.31283429384872e-10	PFN1
Recoverec	13	3.80404320200037e-14	0.26615107504637	0.235	0.087	1.27580000908688e-09	BIN1
Recoverec	13	5.37014929709205e-14	0.49017837526037	0.739	0.523	1.80104067125873e-09	ARPC3
Recoverec	13	5.62030484915319e-14	0.52508631084690	0.854	0.643	1.884937840309e-09	NFKBIA
Recoverec	13	6.98805232027367e-14	0.60687862638099	0.53	0.316	2.34365298717339e-09	DDIT4
Recoverec	13	7.3538717837759e-14	0.31167807839136	0.351	0.167	2.46634151884276e-09	ZNRD1
Recoverec	13	1.07093830799986e-13	0.40942363263406	0.653	0.425	3.59171289736994e-09	ERP29
Recoverec	13	1.467803132762e-13	0.42247310045573	0.59	0.365	4.92271814665721e-09	RAC2
Recoverec	13	1.95593629791541e-13	0.35654391430727	0.53	0.311	6.55981915594872e-09	FXYD5
Recoverec	13	1.98986293140895e-13	0.71863310775537	0.858	0.745	6.67360229935935e-09	EEF1B2
Recoverec	13	1.99733497482894e-13	0.32743742131043	0.437	0.235	6.69866203858131e-09	PSMB8
Recoverec	13	2.5382696939073e-13	0.33194158320192	0.713	0.472	8.51284889942632e-09	EIF3K
Recoverec	13	3.12244352788402e-13	0.50017684865454	0.549	0.316	1.04720511038174e-08	LAPTM5
Recoverec	13	3.41403285626044e-13	0.39336629328	0.944	0.694	1.14499833933263e-08	FABP6
Recoverec	13	3.74440326675615e-13	0.30757860014748	0.642	0.419	1.25579796760468e-08	ATP5F1D
Recoverec	13	4.6569500888852e-13	0.65363024943920	0.817	0.638	1.56184792081032e-08	ZFP36
Recoverec	13	5.09000877762498e-13	0.34233350353897	0.575	0.367	1.70708714383987e-08	NDUFB11
Recoverec	13	5.35450958106993e-13	0.36505395728361	0.545	0.34	1.79579542329923e-08	CD81
Recoverec	13	7.19698271562614e-13	0.43961964950168	0.772	0.568	2.41372406316669e-08	SLC25A6
Recoverec	13	7.39502228844458e-13	0.75649274149322	0.866	0.757	2.48014257509854e-08	RPS5
Recoverec	13	1.25954979476034e-12	0.37786337229910	0.713	0.503	4.22427810166723e-08	EDF1
Recoverec	13	1.35729428704171e-12	0.56054482571145	0.713	0.518	4.5520935798805e-08	IRF1
Recoverec	13	1.73231878663414e-12	0.74980593396572	0.847	0.786	5.80985074661357e-08	RPL26
Recoverec	13	2.59595635459332e-12	0.45588368033720	0.937	0.866	8.70631842203506e-08	HLA-B
Recoverec	13	2.93903164337388e-12	0.36918811339535	0.507	0.293	9.85692432554737e-08	CD79B

Recoverec	13	3.13511969943401e-12	0.616209210034153	0.981	0.904	1.05145644479618e-07	FTL
Recoverec	13	3.22223294058781e-12	0.456557273935691	0.836	0.633	1.08067248361434e-07	NCL
Recoverec	13	3.71015158068631e-12	0.707576243541424	0.955	0.916	1.24431063713057e-07	RPS2
Recoverec	13	3.71140156677494e-12	0.35510449035216	0.429	0.236	1.24472985746498e-07	GPSM3
Recoverec	13	3.73827758386721e-12	0.77768266226235	0.631	0.455	1.25374353607738e-07	HLA-DPA1
Recoverec	13	4.27985137988952e-12	0.688239527615422	0.69	0.526	1.43537655578735e-07	CD79A
Recoverec	13	4.3270877079334e-12	0.739382484524681	0.851	0.739	1.4512186754867e-07	RPSA
Recoverec	13	4.46189940324537e-12	0.356763163607484	0.728	0.508	1.49643182186043e-07	RPL22L1
Recoverec	13	4.51830316676573e-12	0.565364049038671	0.91	0.801	1.51534851606989e-07	RPL23A
Recoverec	13	6.03484337870637e-12	0.259073594890396	0.388	0.2	2.02396577235054e-07	PIM3
Recoverec	13	6.39378570907531e-12	0.47542380484546	0.653	0.439	2.14434785110968e-07	ARHGDIB
Recoverec	13	8.20334670033912e-12	0.460685987735436	0.851	0.68	2.75123841635973e-07	BTF3
Recoverec	13	1.25281488184453e-11	0.354437742675814	0.556	0.352	4.20169055073017e-07	CLIC1
Recoverec	13	1.44012832012549e-11	0.378648630566851	0.631	0.424	4.82990236003688e-07	EIF3F
Recoverec	13	1.62914364821146e-11	0.581755015134393	0.854	0.764	5.46382196737158e-07	RPL7
Recoverec	13	1.74208992368688e-11	0.442711467693982	0.925	0.856	5.84262118606104e-07	RPL6
Recoverec	13	1.7471621545882e-11	0.422715574850585	0.821	0.653	5.85963243405789e-07	OAZ1
Recoverec	13	2.77127280520962e-11	0.498613165656946	0.743	0.524	9.29429473411201e-07	NR4A2
Recoverec	13	2.82619360767903e-11	0.5980213565837	0.332	0.169	9.47848812143392e-07	IGHD
Recoverec	13	3.43160763692692e-11	0.385658586215237	0.649	0.428	1.15089256927255e-06	BCAS2
Recoverec	13	3.51242657944674e-11	0.331691005994431	0.504	0.3	1.17799762621485e-06	SNHG7
Recoverec	13	4.83478083136137e-11	0.315365784826222	0.388	0.208	1.62148879522198e-06	TRBC2
Recoverec	13	7.71494921270131e-11	0.643419254913587	0.907	0.865	2.58743966695576e-06	RPL18A
Recoverec	13	1.01037198404099e-10	0.31119700748195	0.437	0.259	3.38858556007667e-06	ICAM3
Recoverec	13	1.07608902258112e-10	0.300344956701402	0.366	0.196	3.60898736393256e-06	DUT
Recoverec	13	1.17962755101762e-10	0.318457563833516	0.664	0.463	3.9562348806029e-06	HMGN1
Recoverec	13	1.3300016487425e-10	0.265269765552687	0.612	0.399	4.46055952955259e-06	RAN
Recoverec	13	1.61245398882379e-10	0.400411773262888	0.847	0.689	5.40784818771721e-06	EEF1D
Recoverec	13	1.62528677106745e-10	0.286151262222863	0.231	0.099	5.45088677280603e-06	SPIB
Recoverec	13	1.81521550406857e-10	0.276413525537683	0.515	0.318	6.08786975754517e-06	ARL6IP5
Recoverec	13	1.93349317437616e-10	0.375008798373661	0.966	0.901	6.48454940822278e-06	FTH1

Recoverec	13	1.97866567178648e-10	0.5160780936528	0.944	0.89	6.63604893003748e-06	RPL28
Recoverec	13	2.22771513087269e-10	0.29080330134524ɛ	0.425	0.247	7.47131100592084e-06	UBE2S
Recoverec	13	2.52100723396143e-10	0.40387053093436ɛ	0.873	0.751	8.45495406125983e-06	HLA-A
Recoverec	13	2.70630927750045e-10	0.47721684092805	0.795	0.644	9.07642005488101e-06	CFL1
Recoverec	13	3.10744891572356e-10	0.47484452149507ɛ	0.959	0.922	1.04217621735537e-05	RPL41
Recoverec	13	3.34262201846789e-10	0.37539354281679ɛ	0.493	0.306	1.12104857255376e-05	NUP58
Recoverec	13	3.66751779175650e-10	0.54846074287784ɛ	0.843	0.77	1.23001211699929e-05	RPL10A
Recoverec	13	4.3830150324282e-10	0.39997471271992	1	0.998	1.46997558157577e-05	MT-CYB
Recoverec	13	4.6477203958848e-10	0.67004195040488ɛ	0.888	0.872	1.55875246637184e-05	RPS4X
Recoverec	13	8.21481447425908e-10	0.58679255266724	0.657	0.446	2.75508447837701e-05	GADD45B
Recoverec	13	9.02434147941396e-10	0.35676646499644ɛ	0.511	0.335	3.02658364536585e-05	EIF3G
Recoverec	13	9.67351728664977e-10	0.32471925582185	0.481	0.304	3.2443042275966e-05	RSL1D1
Recoverec	13	1.03424636437378e-09	0.64272736346559	0.907	0.878	3.46865545683678e-05	RPS23
Recoverec	13	1.15320445330263e-09	0.59782835523523	0.631	0.462	3.86761709548637e-05	ZNF331
Recoverec	13	1.2981923967161e-09	0.64039736963127	0.552	0.406	4.35387766010647e-05	HLA-DRB5
Recoverec	13	1.4131769380427e-09	0.64673973118295ɛ	0.675	0.533	4.7395128148076e-05	CD37
Recoverec	13	1.49693202192295e-09	0.36002977545835	0.858	0.723	5.02041061512519e-05	TXNIP
Recoverec	13	1.59140474259098e-09	0.51212316785970	0.896	0.798	5.33725322570163e-05	BTG1
Recoverec	13	1.7184249916931e-09	0.27580835095620	0.784	0.603	5.76325373714031e-05	COX4I1
Recoverec	13	1.92050132802376e-09	0.54983671518536ɛ	0.896	0.863	6.44097735392608e-05	RPL29
Recoverec	13	2.00089725783842e-09	0.31349097213948	0.799	0.6	6.71060922333851e-05	EEF2
Recoverec	13	2.20965903249949e-09	0.52425241183477	0.91	0.851	7.4107544631968e-05	RPL14
Recoverec	13	2.3120910554648e-09	0.40309824418325ɛ	0.817	0.671	7.75429098181784e-05	YBX1
Recoverec	13	3.41924704199937e-09	0.39484494097409	0.448	0.283	0.000114674707294575	HIST1H1C
Recoverec	13	4.80153411977306e-09	0.37833302935940ɛ	0.511	0.346	0.000161033851308949	PSME2
Recoverec	13	4.90545931250025e-09	0.26915484661416	0.429	0.256	0.000164519294422633	LBH
Recoverec	13	4.95761940888569e-09	0.34058744169702	0.832	0.641	0.000166268639735208	HNRNPDL
Recoverec	13	5.25591671502328e-09	0.38944479428148	0.75	0.545	0.000176272934788451	BIRC3
Recoverec	13	5.32172502264666e-09	0.38720804933525	0.795	0.62	0.000178480013809524	HNRNPA1
Recoverec	13	5.46054830720847e-09	0.55090042867709	0.892	0.842	0.000183135869127158	RPL5
Recoverec	13	5.5016737964488e-09	0.30435646426703	0.451	0.282	0.0001845151357853	HLA-DMA

Recoverec	13	5.78870237447248e-09	0.25617655395418(	0.425	0.258	0.000194141500235058	IFITM2
Recoverec	13	6.12689018463437e-09	0.34220204012232	0.433	0.269	0.000205483643012268	LIMD2
Recoverec	13	9.07462380966586e-09	0.33496194676144	0.578	0.4	0.000304344733328574	PSMB9
Recoverec	13	9.75190911064305e-09	0.35663143047573	0.884	0.816	0.000327059527752747	RPS7
Recoverec	13	1.01213075046803e-08	0.30959236140404(	0.664	0.461	0.000339448411091968	PNN
Recoverec	13	1.08951453507312e-08	0.75318382590367	0.56	0.443	0.000365401384772824	HLA-DPB1
Recoverec	13	1.11636711091655e-08	0.86680459461250	0.608	0.506	0.000374407201659192	HLA-DRB1
Recoverec	13	1.11988012607979e-08	0.45088317707960	0.91	0.865	0.000375585396684641	RPS15
Recoverec	13	1.14233969069787e-08	0.56161395283021	0.91	0.878	0.000383117885466253	RPL11
Recoverec	13	1.19609045566727e-08	0.28184461275521	0.25	0.124	0.000401144817021689	ARL4C
Recoverec	13	1.369102907951e-08	0.31480117506449	0.593	0.421	0.000459169733268606	NSA2
Recoverec	13	1.49220813281462e-08	0.51917214380016(	0.94	0.907	0.000500456763583366	RPS18
Recoverec	13	1.52656476934452e-08	0.44346974778031	0.937	0.874	0.000511979292342764	RPS24
Recoverec	13	1.67330732226967e-08	0.47838923374216	0.869	0.833	0.000561193809742802	RPS13
Recoverec	13	1.70267780041231e-08	0.58620519217232	0.914	0.878	0.00057104408070228	RPS3A
Recoverec	13	1.75048163964269e-08	0.42748443307053(	0.896	0.82	0.000587076532303366	FAU
Recoverec	13	2.00531731409511e-08	0.26424509850846	0.295	0.162	0.000672543320801218	ABRACL
Recoverec	13	2.01924255615539e-08	0.26767633614938	0.638	0.435	0.000677213568483395	BOD1L1
Recoverec	13	2.28188227904391e-08	0.44323955247483	0.825	0.697	0.000765297678745746	NPM1
Recoverec	13	2.9343108095471e-08	0.25220780812455	0.31	0.173	0.000984109159305905	TMEM238
Recoverec	13	3.02921489080449e-08	0.32541328249913	0.601	0.414	0.00101593809007801	TUBA1B
Recoverec	13	3.77927111782243e-08	0.47979624572932	0.922	0.869	0.00126749194749529	RPL9
Recoverec	13	3.8397605547125e-08	0.7443678529724	0.873	0.724	0.00128777889483948	BTG2
Recoverec	13	3.94422664120957e-08	0.40849700696885	0.91	0.853	0.00132281473092886	RPS25
Recoverec	13	3.9954527681372e-08	0.28849724443653	0.97	0.959	0.00133999494937785	TPT1
Recoverec	13	3.99734772533513e-08	0.33933756174172	0.858	0.753	0.0013406304801229	RPL27
Recoverec	13	4.08091152165702e-08	0.34409338174235(	0.851	0.764	0.00136865610613333	HLA-C
Recoverec	13	4.3423265238578e-08	0.26316392114539	0.493	0.323	0.00145632946957143	MYADM
Recoverec	13	4.41388657609067e-08	0.46426853647462	0.91	0.883	0.00148032927988929	RPL18
Recoverec	13	5.06427120149346e-08	0.43658051271381(	0.91	0.86	0.00169845527555688	RPS27A
Recoverec	13	5.89512957680777e-08	0.45408709760301	0.888	0.868	0.00197710855746979	RPL30

Recoverec	13	6.34469259043021e-08	0.39724822468339	0.892	0.829	0.00212788300097848	RPL15
Recoverec	13	6.44910838497549e-08	0.51058121120450	0.843	0.797	0.00216290197015308	RPL21
Recoverec	13	7.77908931369072e-08	0.49993411263226	0.896	0.857	0.00260895097402559	ACTB
Recoverec	13	7.80360399267408e-08	0.30004459442755	0.847	0.679	0.00261717270706303	SERP1
Recoverec	13	8.15318130929034e-08	0.28157799900438	0.963	0.916	0.0027344139475098	EIF1
Recoverec	13	8.78209787127662e-08	0.25121263218125	0.455	0.292	0.00294533998406875	VPS28
Recoverec	13	9.61951544550794e-08	0.32585699196987	0.903	0.798	0.00322619309011445	H3F3A
Recoverec	13	9.63119482903123e-08	0.82546932852972	0.657	0.597	0.00323011012176049	HLA-DRA
Recoverec	13	9.74811451685284e-08	0.41958911476283	0.944	0.917	0.00326932264666211	RPS19
Recoverec	13	1.23319312943135e-07	0.51890534816642	0.933	0.912	0.00413588311748688	RPS12
Recoverec	13	1.26979980888591e-07	0.31535813236344	0.638	0.489	0.00425865459904157	MYL12A
Recoverec	13	1.34898093275242e-07	0.73240974532508	0.896	0.865	0.00452421225226508	CD74
Recoverec	13	1.37029113108173e-07	0.35813331602883	0.698	0.525	0.00459568239542192	CYCS
Recoverec	13	1.49754032369045e-07	0.27258153740939	0.608	0.463	0.00502245073759303	PSME1
Recoverec	13	1.59588997995883e-07	0.25497830473838	0.728	0.575	0.00535229581478591	ATP5MC2
Recoverec	13	1.63241532143726e-07	0.31768179932422	0.772	0.628	0.00547479450503629	HLA-E
Recoverec	13	1.69905033139387e-07	0.25377395247936	0.493	0.333	0.00569827500142877	SYF2
Recoverec	13	1.70406582878183e-07	0.34955458612776	0.44	0.293	0.00571509597656851	PCNX4
Recoverec	13	1.72534058354124e-07	0.33726195767423	0.478	0.32	0.00578644724908061	ARL5B
Recoverec	13	1.86035228992446e-07	0.30836726300272	0.851	0.682	0.00623924950994865	ZFP36L2
Recoverec	13	2.11265612139682e-07	0.26027713288462	0.526	0.368	0.00708542609994067	SLC25A5
Recoverec	13	2.12066906073211e-07	0.43336048033147	0.854	0.818	0.00711229989588336	NACA
Recoverec	13	2.32681475112719e-07	0.31006259202426	0.993	0.974	0.00780367131233037	B2M
Recoverec	13	2.37237889284743e-07	0.30828609713048	0.955	0.945	0.0079564843308317	RPLP1
Recoverec	13	2.77595881539202e-07	0.48742883672571	0.653	0.486	0.00931001067506177	TNFAIP3
Recoverec	13	2.90973871048812e-07	0.29709278749244	0.828	0.613	0.00975868168723506	MT-ATP8
Recoverec	13	2.9194617986482e-07	0.47914140818493	0.929	0.894	0.00979129098030633	RPL13A
Recoverec	13	3.89967802185841e-07	0.27386368681016	0.399	0.256	0.0130787401497087	PLAC8
Recoverec	13	4.24650139941737e-07	0.41649009258908	0.496	0.347	0.014241916393366	YBX3
Recoverec	13	5.88651766780226e-07	0.35413166424729	0.877	0.796	0.0197422029542752	RPL22
Recoverec	13	6.66791067665945e-07	0.51591534419671	0.933	0.889	0.0223628388273805	RPL12

Recoverec	13	6.82160797384863e-07	0.42690610154990€	0.892	0.875	0.0228783088226935	RPL32
Recoverec	13	6.84296815927034e-07	0.34347643294456€	0.907	0.856	0.0229499466125609	RPL24
Recoverec	13	7.10304179185928e-07	0.32766722864732'	0.694	0.537	0.0238221815615376	REL
Recoverec	13	7.93045788304174e-07	0.35701135700843⁄	0.896	0.855	0.0265971696481454	RPS16
Recoverec	13	1.20008948139467e-06	0.31559920146868§	0.854	0.689	0.0402486010270143	HERPUD1
Recoverec	13	1.45604739237746e-06	0.34808336680350£	0.511	0.393	0.0488329174455552	GSTP1
Recoverec	20	1.81548941344023e-79	1.47030166553023	0.711	0.237	6.08878839479583e-75	AC105402.3
Recoverec	20	1.20433365972719e-54	1.49312655721249	0.38	0.083	4.03909422799306e-50	SLC38A11
Recoverec	20	8.80296414059755e-53	1.27695207795821	0.729	0.333	2.95233811347361e-48	SMAD3
Recoverec	20	5.48297327377465e-51	0.71062942673389⁄	0.392	0.088	1.83887957655854e-46	SELL
Recoverec	20	5.46152993566626e-50	1.16254518820959	0.602	0.222	1.83168790982375e-45	COL19A1
Recoverec	20	5.26193689264717e-49	1.22991142171529	0.38	0.093	1.76474839505601e-44	STEAP1B
Recoverec	20	2.04105060549163e-48	0.71968115404752‸	0.769	0.346	6.84527552069782e-44	OLFM4
Recoverec	20	8.00827419088336e-48	0.76900116459707'	0.915	0.49	2.68581499813846e-43	MT1G
Recoverec	20	8.70718506973889e-48	1.01265733161633	0.909	0.571	2.92021572868903e-43	MTRNR2L8
Recoverec	20	1.00118614381959e-46	0.92852218033036	0.818	0.455	3.35777808914213e-42	PARP14
Recoverec	20	2.56581102472083e-46	0.95952122051877‸	0.647	0.256	8.60521701470871e-42	KHDRBS2
Recoverec	20	2.6204309137671e-46	0.73124258588314'	0.611	0.212	8.7884011985921e-42	CD22
Recoverec	20	2.9452004251002e-46	0.87165565214266'	0.343	0.077	9.87761318570106e-42	P2RY14
Recoverec	20	1.30551662047774e-45	0.99474372749797'	0.76	0.37	4.37844164175823e-41	VAV3
Recoverec	20	3.98749535269125e-44	0.40809100551861⁄	0.283	0.051	1.33732619138559e-39	SDK2
Recoverec	20	4.0567888908128e-44	0.95208008660990£	0.653	0.275	1.3605658582008e-39	UST
Recoverec	20	5.46875795856496e-44	0.901122822211109	0.927	0.651	1.83411204414352e-39	RERE
Recoverec	20	2.09140056003254e-43	1.00453415673841	0.748	0.374	7.01413919823715e-39	PLEKHG1
Recoverec	20	1.09332260561861e-42	0.77053232969474‸	0.696	0.291	3.66678535472369e-38	PAX5
Recoverec	20	2.54893191535694e-42	0.90007240797601‸	0.799	0.44	8.54860785772412e-38	SNX2
Recoverec	20	3.53601993227358e-42	0.93478740252049€	0.821	0.468	1.18591036488591e-37	TMEM131L
Recoverec	20	6.70507188960629e-42	0.90088880508930⁄	0.772	0.419	2.24874701033616e-37	ITPR1
Recoverec	20	8.30743264790694e-42	0.47091022726724€	0.337	0.076	2.78614676145503e-37	CLEC17A
Recoverec	20	1.70512094353601e-41	0.55913214600362'	0.529	0.18	5.71863462043108e-37	CIITA
Recoverec	20	6.01320521101351e-41	0.63395003877213	0.556	0.197	2.01670876366971e-36	PIK3AP1

Recoverec	20	1.52007709070476e-40	0.93865514295567!	0.663	0.283	5.09803454680564e-36	AP002075.1
Recoverec	20	4.47663175933591e-40	0.64048568580468:	0.508	0.182	1.50137275944608e-35	CR2
Recoverec	20	5.02633575578342e-40	3.79751769720177	0.498	0.178	1.68573248577464e-35	HBB
Recoverec	20	4.61019289696088e-39	1.13638409920856	0.775	0.459	1.54616649378274e-34	AFF3
Recoverec	20	1.10363069278384e-38	1.1335262566545	0.398	0.122	3.70135661745844e-34	AC019197.1
Recoverec	20	1.46590295326629e-38	0.98023896144170:	0.805	0.465	4.91634532466449e-34	MAP4K4
Recoverec	20	4.91639443796827e-38	0.87598955488480(	0.742	0.395	1.6488603666058e-33	JAZF1
Recoverec	20	1.03435774415377e-37	0.80285606429923	0.717	0.352	3.46902900234292e-33	PRKCE
Recoverec	20	1.04796193066775e-37	0.73546405231374!	0.678	0.292	3.5146547230735e-33	FCRL1
Recoverec	20	1.89074969488408e-37	1.38026101029859	0.802	0.523	6.34119632670222e-33	BACH2
Recoverec	20	2.10038808721528e-37	0.52824820403087(	0.28	0.062	7.0442815669026e-33	GBP4
Recoverec	20	7.31124008469165e-37	0.66764202094661	0.684	0.312	2.45204369960388e-32	PIKFYVE
Recoverec	20	7.82778229747307e-37	0.68205935800147	0.59	0.254	2.62528162692652e-32	XIST
Recoverec	20	1.02975028810473e-36	1.03160079927811	0.881	0.632	3.45357651624566e-32	SIPA1L1
Recoverec	20	3.25249113980076e-36	0.82857394876003!	0.523	0.194	1.09082047846638e-31	CR1
Recoverec	20	3.7535200354475e-36	0.47257578881700	0.495	0.173	1.25885554948838e-31	HLA-DMB
Recoverec	20	5.08187764499389e-36	0.66982996937598	0.617	0.276	1.70436012457805e-31	BMP2K
Recoverec	20	6.26925682158834e-36	0.72587694859959	0.629	0.267	2.1025833528243e-31	LTB
Recoverec	20	7.69431400591885e-36	0.62638619288648!	0.471	0.166	2.58051903130507e-31	HLA-DQA2
Recoverec	20	1.86824245344545e-35	0.45269337468613	0.389	0.116	6.26571154036535e-31	CCR7
Recoverec	20	2.24040568806625e-35	0.70554406995587	0.881	0.578	7.5138725966366e-31	ZHX2
Recoverec	20	2.48328337412923e-35	0.81402462615386(	0.945	0.833	8.32843578015462e-31	ARHGAP15
Recoverec	20	2.79502444141303e-35	1.03929083326663	0.793	0.52	9.37395297161104e-31	BANK1
Recoverec	20	2.80045260768489e-35	0.61906931706879	0.687	0.355	9.39215795565358e-31	MICAL3
Recoverec	20	3.50995233717163e-35	0.84439965701637(	0.736	0.374	1.17716781484062e-30	MS4A1
Recoverec	20	3.73820933875399e-35	0.85443534497718!	0.96	0.853	1.25372064803131e-30	CD74
Recoverec	20	5.04820519597954e-35	0.62052242292377(	0.66	0.296	1.69306705862762e-30	WDFY4
Recoverec	20	6.48628102833737e-35	0.99823315922870:	0.857	0.618	2.17536893128379e-30	RASGEF1B
Recoverec	20	8.73909499723085e-35	1.08009898946969	0.775	0.468	2.93091768017128e-30	LY9
Recoverec	20	1.94206884644019e-34	0.73344687099808	0.553	0.224	6.51331049719112e-30	RUBCNL
Recoverec	20	3.98690116881163e-34	0.45875284679919!	0.599	0.233	1.33712691399604e-29	LINC00926

Recoverec	20	6.8101415746393e-34	0.917208346196757	0.796	0.496	2.28398528130253e-29	LRRFIP1
Recoverec	20	7.15418145042605e-34	0.98394993398613	0.818	0.529	2.39936937484389e-29	CD83
Recoverec	20	1.24622851123291e-33	0.75207858604871	0.553	0.229	4.17960118097293e-29	GRK3
Recoverec	20	1.31958169966704e-33	0.76344365705522	0.404	0.134	4.42561310434333e-29	MTSS1
Recoverec	20	2.2034706948703e-33	0.72499261719224	0.608	0.276	7.39000001645601e-29	BCL11A
Recoverec	20	2.46537040039244e-33	0.602587435755497	0.587	0.254	8.26835924883617e-29	MT1H
Recoverec	20	2.56646009887049e-33	0.387114784546581	0.435	0.139	8.60739387959186e-29	FAM129C
Recoverec	20	1.18146467995097e-32	0.66974074087633	0.623	0.297	3.96239624361956e-28	RRAS2
Recoverec	20	3.43993330167075e-32	0.46634898720132	0.547	0.217	1.15368483071433e-27	TRAF5
Recoverec	20	4.59889261029076e-32	0.84455926180384	0.435	0.159	1.54237660363932e-27	ST6GALNAC3
Recoverec	20	5.85434746088446e-32	0.51955082640583	0.596	0.272	1.96343105143143e-27	DOCK11
Recoverec	20	6.28990067169677e-32	0.814273257751247	0.921	0.667	2.10950688727366e-27	PDE7A
Recoverec	20	7.6152904264058e-32	0.48597960830317	0.438	0.149	2.55401610320798e-27	CCR6
Recoverec	20	1.24616348584367e-31	0.48734048327148	0.234	0.049	4.17938309882251e-27	NETO1
Recoverec	20	1.28176580402524e-31	0.72661890999914	0.635	0.303	4.29878615353985e-27	SWAP70
Recoverec	20	1.34260226136383e-31	0.80672979961465	0.836	0.534	4.502819464162e-27	ADAM28
Recoverec	20	1.99201233202322e-31	0.43344521824516	0.647	0.319	6.68081095913948e-27	NAA15
Recoverec	20	2.74535214199834e-31	0.81784867016155	0.729	0.414	9.20736201383403e-27	EBF1
Recoverec	20	3.8694849743395e-31	0.89641975140763	0.672	0.359	1.29774787069398e-26	AUTS2
Recoverec	20	4.01608371362722e-31	0.45509392540406	0.532	0.218	1.3469141558763e-26	HVCN1
Recoverec	20	5.26807136870693e-31	0.576995825986311	0.696	0.348	1.76680577563693e-26	LCP1
Recoverec	20	6.34217908189443e-31	0.71966965234719	0.836	0.557	2.12704002048575e-26	SLC38A1
Recoverec	20	6.77421397227872e-31	0.58705146906649	0.705	0.366	2.27193588202284e-26	UBAC2
Recoverec	20	1.05636634222357e-30	0.66050484594492	0.945	0.739	3.5428414385494e-26	UTRN
Recoverec	20	1.15270446872673e-30	0.39386891641481	0.258	0.062	3.86594024721571e-26	CHI3L2
Recoverec	20	1.421130189459e-30	0.76697373506302	0.784	0.475	4.76618642940758e-26	HLA-DRB1
Recoverec	20	1.43827151276967e-30	0.475737772052001	0.292	0.078	4.82367499952694e-26	CD244
Recoverec	20	1.51307403491687e-30	0.497129622759847	0.62	0.279	5.07454769830421e-26	TNFRSF13C
Recoverec	20	1.67857208878624e-30	0.58803841830653	0.821	0.514	5.6295950713713e-26	EIF4G3
Recoverec	20	1.81458859036082e-30	0.63519876227183	0.426	0.149	6.08576721435213e-26	IGHD
Recoverec	20	2.3118137215234e-30	0.96982766997542	0.891	0.7	7.75336085924517e-26	FOXP1

Recoverec	20	3.99944216607697e-30	0.60333814379351	0.498	0.199	1.3413329136589e-25	C12orf42
Recoverec	20	5.09894812740774e-30	0.51918030298042	0.833	0.508	1.71008522297001e-25	USP9X
Recoverec	20	5.20824529250881e-30	0.76464443163750	0.83	0.51	1.7467413062016e-25	REL
Recoverec	20	7.09071579412909e-30	0.49685135506984	0.298	0.081	2.37808426303502e-25	TCL1A
Recoverec	20	8.25414389717039e-30	0.63146127077740	0.641	0.326	2.76827478023301e-25	STX7
Recoverec	20	1.08108745177173e-29	0.62562339732235	0.544	0.247	3.62575109575203e-25	AC104365.1
Recoverec	20	1.3435420982901e-29	0.70134851700416	0.921	0.73	4.50597148924535e-25	TBC1D5
Recoverec	20	1.41126940858234e-29	0.72937314295876	0.784	0.495	4.73311534250345e-25	AC120193.1
Recoverec	20	1.53123378566078e-29	0.55195802161848	0.635	0.295	5.13545187034914e-25	LAPTM5
Recoverec	20	2.8509497505545e-29	0.62289385539630	0.593	0.277	9.56151527340969e-25	CLEC2D
Recoverec	20	2.92927136007516e-29	0.47397181028623	0.574	0.243	9.82419028742006e-25	IRF8
Recoverec	20	3.2053355556312e-29	0.52777420545922	0.757	0.454	1.07500543864759e-24	SIPA1L3
Recoverec	20	3.35636777732595e-29	0.78000708473258	0.778	0.459	1.12565862515958e-24	SNX9
Recoverec	20	3.40842235572961e-29	0.62808505847548	0.927	0.677	1.1431166896646e-24	MTRNR2L12
Recoverec	20	6.0222065867393e-29	0.76427263251640	0.909	0.706	2.01972764506063e-24	SSH2
Recoverec	20	7.89370037036654e-29	0.67266133451386	0.748	0.409	2.64738923021353e-24	HLA-DPB1
Recoverec	20	1.11402867998241e-28	0.49519996826866	0.471	0.185	3.736229386925e-24	KYNU
Recoverec	20	1.16432693336523e-28	0.81251486058930	0.805	0.518	3.90491966912031e-24	ARHGAP24
Recoverec	20	1.56878933794496e-28	0.54861334180235	0.322	0.098	5.26140568159979e-24	CHL1
Recoverec	20	1.66203005049732e-28	0.47331324675544	0.283	0.079	5.57411638335792e-24	CNKSR2
Recoverec	20	2.39250906125033e-28	0.49448248566338	0.693	0.376	8.02399688962137e-24	LRCH1
Recoverec	20	2.57625043600006e-28	0.47934085736467	0.596	0.271	8.64022871225699e-24	PARP15
Recoverec	20	2.83762640070628e-28	0.42371212384076	0.486	0.201	9.51683142268872e-24	UGT8
Recoverec	20	2.92013840654781e-28	0.72761630465317	0.547	0.257	9.79356018788004e-24	RUNX1
Recoverec	20	3.95932530577275e-28	0.58432462748400	0.717	0.375	1.32787852105007e-23	RIPOR2
Recoverec	20	5.22909131570179e-28	0.61653512382377	0.647	0.35	1.75373264546007e-23	LYST
Recoverec	20	5.27876838078911e-28	0.71833346260186	0.781	0.502	1.77039333954905e-23	NEDD9
Recoverec	20	5.83698826961943e-28	0.60752267184931	0.559	0.263	1.95760912586496e-23	STAP1
Recoverec	20	5.89698699233086e-28	0.67547679662240	0.897	0.671	1.97773149748792e-23	PTPRC
Recoverec	20	6.4607608052638e-28	0.41322923135092	0.596	0.296	2.16680995886937e-23	WIPF2
Recoverec	20	7.80886592558236e-28	0.84476525618634	0.751	0.434	2.61893745412181e-23	SLC2A3

Recoverec	20	8.00546877092545e-28	0.74845454643715!	0.857	0.617	2.68487411639298e-23	LYN
Recoverec	20	1.00081967931368e-27	0.70703090760644	0.869	0.616	3.35654904048224e-23	CD55
Recoverec	20	1.10205312963014e-27	0.70180491683069	0.924	0.725	3.69606578615357e-23	PRDM2
Recoverec	20	1.26193383755115e-27	0.60727825304037	0.465	0.185	4.23227370437904e-23	JAM3
Recoverec	20	1.27316467083703e-27	0.38884693776066	0.377	0.131	4.26993967305322e-23	AC092652.2
Recoverec	20	1.33438067144872e-27	0.67482076548545	0.657	0.354	4.47524589590472e-23	GNA13
Recoverec	20	1.36977454914113e-27	0.81799915034967	0.684	0.363	4.59394988290952e-23	MYO1E
Recoverec	20	1.42436099917331e-27	0.56071354115593	0.818	0.528	4.77702191902744e-23	SPIDR
Recoverec	20	1.54213086602789e-27	0.61748016484149	0.766	0.462	5.17199849848434e-23	XYLT1
Recoverec	20	1.86933201013334e-27	0.41063325225175	0.498	0.2	6.26936569558521e-23	TRIM22
Recoverec	20	2.93397695234554e-27	0.70984373433788	0.936	0.773	9.83997190277647e-23	ZCCHC7
Recoverec	20	4.29878404479529e-27	0.45466474608526	0.45	0.173	1.44172619294345e-22	CYBB
Recoverec	20	4.44338451316925e-27	0.41540365671082	0.447	0.181	1.4902222980267e-22	CD40
Recoverec	20	4.83586579070931e-27	0.53431426862796	0.775	0.451	1.62185266888809e-22	RAB8B
Recoverec	20	4.92266833330421e-27	0.91130588804945	0.748	0.477	1.65096450562357e-22	VPS37B
Recoverec	20	4.95492545559199e-27	0.55245841566353	0.389	0.148	1.66178289929644e-22	KLF7
Recoverec	20	5.37944159077788e-27	0.59936477303319	0.912	0.676	1.80415712071508e-22	KDM4C
Recoverec	20	5.56095781134668e-27	0.56058973461916	0.714	0.42	1.86503403076945e-22	PLEKHA2
Recoverec	20	5.66636833511116e-27	0.60346613550803	0.264	0.075	1.90038661222958e-22	SCN3A
Recoverec	20	8.03182105355045e-27	0.87274183032452	0.733	0.431	2.69371214493975e-22	GNG2
Recoverec	20	8.51866452711545e-27	0.34762896109859	0.486	0.202	2.85698970910398e-22	ABHD15-AS1
Recoverec	20	1.22054927161516e-26	0.66916817851313	0.942	0.811	4.09347814714293e-22	RALGPS2
Recoverec	20	1.27867597582468e-26	0.69274335076797	0.824	0.575	4.2884234877208e-22	SNX29
Recoverec	20	1.46691281641946e-26	0.47649962536953	0.729	0.373	4.91973220370757e-22	HLA-DRB5
Recoverec	20	2.7996869857007e-26	0.63322618376217	0.945	0.84	9.38959021264299e-22	SIK3
Recoverec	20	3.31001056799832e-26	0.49335892597287	0.644	0.346	1.11011134429528e-21	CMIP
Recoverec	20	4.96951780210147e-26	0.58686628533191	0.76	0.418	1.66667688046879e-21	HLA-DQA1
Recoverec	20	5.99200110635784e-26	0.48761351714285	0.52	0.234	2.00959733105029e-21	B4GALT1
Recoverec	20	7.03430581557346e-26	0.53407070817348	0.708	0.405	2.35916548442703e-21	DIP2B
Recoverec	20	1.10313021529853e-25	0.29761358689198	0.261	0.071	3.69967811606819e-21	ABCB4
Recoverec	20	1.14842789521475e-25	0.56173557828193	0.69	0.396	3.85159747497122e-21	USP24

Recoverec	20	1.26165844900818e-25	0.49621962676571	0.748	0.436	4.23135010628363e-21	TRAK1
Recoverec	20	1.3476059843045e-25	0.68327341639614	0.65	0.363	4.51960095016043e-21	NIN
Recoverec	20	1.35664943257145e-25	0.55930055079537	0.681	0.373	4.54993086695814e-21	PTPRK
Recoverec	20	1.74283355774343e-25	0.36481126504600	0.529	0.243	5.84511518595991e-21	NCKAP1L
Recoverec	20	2.24769598486882e-25	0.28852399809581	0.356	0.123	7.53832279405305e-21	LAT2
Recoverec	20	4.29728207764648e-25	0.50856488270331	0.313	0.104	1.44122246320108e-20	THRB
Recoverec	20	4.83403752297263e-25	0.66673490568658	0.763	0.482	1.62123950445456e-20	DLEU2
Recoverec	20	5.71630574881254e-25	0.77805327056021	0.502	0.245	1.91713462203675e-20	STAT1
Recoverec	20	6.13865740497767e-25	0.4922170704649	0.286	0.09	2.05878292048141e-20	MED12L
Recoverec	20	6.96038435925892e-25	0.32909328761264	0.38	0.139	2.33437370640826e-20	SLC44A2
Recoverec	20	8.77072035205978e-25	0.46628249206699	0.395	0.155	2.94152419167381e-20	L3MBTL3
Recoverec	20	1.08659492979075e-24	0.36383613132462	0.447	0.179	3.64422207553221e-20	FCMR
Recoverec	20	1.09909609025692e-24	0.52956567292405	0.626	0.34	3.68614846750366e-20	TBC1D1
Recoverec	20	1.2736563138746e-24	0.45676927303257	0.872	0.584	4.27158854547264e-20	PPP2R5C
Recoverec	20	1.44543394566451e-24	0.51249588363826	0.793	0.469	4.84769636696964e-20	STK17A
Recoverec	20	1.46090659853918e-24	0.6476533772616	0.918	0.704	4.89958855018071e-20	MT-ND4L
Recoverec	20	1.52908949404202e-24	0.42581557983997	0.535	0.248	5.12826034511811e-20	TRIM38
Recoverec	20	1.79635956066415e-24	0.58264399167366	0.945	0.802	6.02463069455543e-20	ARID1B
Recoverec	20	2.56166914293989e-24	0.60287062741736	0.511	0.243	8.59132597159181e-20	RFTN1
Recoverec	20	2.70060055961647e-24	0.72303023597117	0.723	0.453	9.05727415684171e-20	MALT1
Recoverec	20	4.00010151596114e-24	0.55071202479876	0.681	0.405	1.34155404642305e-19	USP6NL
Recoverec	20	4.0849705306754e-24	0.77544767699619	0.884	0.682	1.37001741657792e-19	ST6GAL1
Recoverec	20	4.89714541513587e-24	0.75657671298730	0.848	0.633	1.64240462932827e-19	ATP2B1
Recoverec	20	5.73041059026795e-24	0.55590599960005	0.818	0.56	1.92186510376406e-19	BPTF
Recoverec	20	6.61907620692541e-24	0.57862475789624	0.511	0.25	2.21990577827864e-19	MARCH3
Recoverec	20	6.74651849682921e-24	0.39331932381315	0.374	0.144	2.26264737346658e-19	TCOF1
Recoverec	20	7.96579420299388e-24	0.64411216262374	0.714	0.445	2.67156805980009e-19	RB1
Recoverec	20	9.94599901818636e-24	0.55413726665218	0.647	0.356	3.33568915071934e-19	CEMIP2
Recoverec	20	1.05936560469434e-23	0.63443591284115	0.878	0.681	3.55290036502389e-19	PRKCB
Recoverec	20	1.14452291333994e-23	0.37858343305221	0.28	0.089	3.8385009467595e-19	ADARB1
Recoverec	20	1.21842110511859e-23	0.46918735173176	0.818	0.514	4.08634070234672e-19	FAM49B

Recoverec	20	1.25005277687979e-23	0.42722671384429!	0.568	0.287	4.19242700309945e-19	CYFIP2
Recoverec	20	1.29705138716628e-23	0.46867157752111	0.544	0.259	4.35005094227826e-19	MOB3B
Recoverec	20	1.3374949461045e-23	0.45504768483555!	0.693	0.4	4.48569055024528e-19	CHD7
Recoverec	20	1.41780185280522e-23	0.7039255499847	0.869	0.638	4.75502385393815e-19	ADK
Recoverec	20	2.00227884917693e-23	0.54545514254127	0.787	0.505	6.71524280436959e-19	SKIL
Recoverec	20	2.51280360392917e-23	0.62198562673173(	0.429	0.188	8.42744072685766e-19	SESTD1
Recoverec	20	2.62901265575234e-23	0.49586981826125;	0.809	0.53	8.81718264486221e-19	BIRC3
Recoverec	20	2.74283514968713e-23	0.38501682858786	0.267	0.084	9.19892052502068e-19	ZNF318
Recoverec	20	2.74953601961824e-23	0.50136071770109!	0.751	0.457	9.22139390259566e-19	INPP5D
Recoverec	20	3.0962922529639e-23	0.46209864514071:	0.34	0.125	1.03843449579903e-18	SLC9A7
Recoverec	20	3.82943527243434e-23	0.54816736364708(	0.416	0.184	1.28431600166903e-18	SLAMF1
Recoverec	20	4.08932947621355e-23	0.44594964626633!	0.438	0.199	1.3714793197325e-18	TET3
Recoverec	20	5.31482861435117e-23	0.50238787583229	0.827	0.549	1.78248722068109e-18	WIPF1
Recoverec	20	5.63445149384855e-23	0.46747222417609!	0.699	0.41	1.88968234200693e-18	RNF213
Recoverec	20	6.36077158193533e-23	0.45245642909172{	0.611	0.337	2.13327557314947e-18	CNTRL
Recoverec	20	9.24960405104187e-23	0.39247897833646	0.802	0.51	3.10213220663842e-18	PHF20
Recoverec	20	9.68014462652757e-23	0.47681320430441	0.632	0.341	3.24652690484482e-18	GPR183
Recoverec	20	9.88381583107911e-23	0.59199913585684(	0.809	0.559	3.31483415342731e-18	MGAT5
Recoverec	20	1.0707912832234e-22	0.51534451169382:	0.912	0.665	3.59121980567464e-18	DOCK8
Recoverec	20	1.49788819606102e-22	0.45552103309365	0.796	0.528	5.02361743194946e-18	PCM1
Recoverec	20	1.52658081290548e-22	0.46296509117748(	0.553	0.272	5.11984673032241e-18	PHTF2
Recoverec	20	1.76172194085361e-22	0.40342513674655{	0.477	0.226	5.90846304523485e-18	RABEP2
Recoverec	20	1.82444528320966e-22	0.42945191347973	0.875	0.612	6.11882459082855e-18	DOCK2
Recoverec	20	1.92186104286923e-22	0.56080420540311{	0.836	0.561	6.44553756557481e-18	SETBP1
Recoverec	20	2.16036471182511e-22	0.47729948420092;	0.608	0.32	7.24543117051905e-18	CARD11
Recoverec	20	2.30566510924773e-22	0.47927549324950	0.772	0.482	7.73273964339504e-18	CHD1
Recoverec	20	2.56793117296411e-22	0.51841786612582!	0.827	0.554	8.61232756788703e-18	CHST11
Recoverec	20	2.56989846936456e-22	0.28260568703162{	0.325	0.112	8.61892548655485e-18	TLR10
Recoverec	20	2.8860662523549e-22	0.33840565286970!	0.353	0.135	9.67928899714785e-18	TMC8
Recoverec	20	3.19473702256502e-22	0.55067519653995	0.881	0.651	1.07145090262786e-17	EML4
Recoverec	20	3.26062731702848e-22	0.45924933344227	0.805	0.519	1.09354918958501e-17	KAT6A

Recoverec	20	3.75814908347701e-22	0.61136112466382€	0.86	0.623	1.26040803961652e-17	SMAP2
Recoverec	20	3.77805409583029e-22	0.48467614803073ξ	0.362	0.146	1.26708378265956e-17	KMO
Recoverec	20	4.03432932246997e-22	0.45929787697474	0.562	0.304	1.35303336816998e-17	AGO2
Recoverec	20	4.12089061078178e-22	0.44892626652189ξ	0.568	0.306	1.38206429304399e-17	EFR3A
Recoverec	20	4.46758746905425e-22	0.31102004915426	0.593	0.318	1.49833948537142e-17	PPP4R2
Recoverec	20	4.70990234024366e-22	0.63392373711691ξ	0.574	0.32	1.57960704687092e-17	GRK5
Recoverec	20	4.7534064152017e-22	0.49547079524852	0.736	0.433	1.59419744353034e-17	HLA-DPA1
Recoverec	20	5.38521298306468e-22	0.48439503858097	0.511	0.264	1.80609273026023e-17	EPSTI1
Recoverec	20	6.01006460036079e-22	0.40619069939183	0.316	0.113	2.015655465669e-17	CD72
Recoverec	20	6.58991873096108e-22	0.59953887250105ξ	0.936	0.783	2.21012694398973e-17	ANKRD44
Recoverec	20	7.82888034246417e-22	0.52596077292887	0.605	0.327	2.62564988925563e-17	PTK2
Recoverec	20	8.15908250457047e-22	0.29432338092047	0.34	0.125	2.73639309038284e-17	SHISAL2A
Recoverec	20	8.16650865918343e-22	0.44703004335264ξ	0.535	0.267	2.73888367411694e-17	INPP5A
Recoverec	20	8.79557096978354e-22	0.37295075266692€	0.821	0.517	2.949858591846e-17	PUM1
Recoverec	20	1.17524068148365e-21	0.49160460543822	0.809	0.558	3.94152219755986e-17	KDM6A
Recoverec	20	1.22269936374774e-21	0.35401797471546	0.69	0.406	4.10068912613716e-17	BRD4
Recoverec	20	1.2529122047724e-21	0.53681630542862ξ	0.456	0.216	4.20201695236569e-17	SAMD4A
Recoverec	20	1.3861021151018e-21	0.32725277893983	0.775	0.464	4.6487092736284e-17	CD53
Recoverec	20	1.40894402225649e-21	0.46250890581310	0.529	0.272	4.72531646184382e-17	NUP88
Recoverec	20	1.49835078000898e-21	0.49921702089959	0.933	0.815	5.0251688459941e-17	FCHSD2
Recoverec	20	1.58868029992528e-21	0.43397769271338€	0.711	0.44	5.32811598988939e-17	PDS5A
Recoverec	20	1.75847851442065e-21	0.51241362849661	0.857	0.62	5.89758524166398e-17	NIPBL
Recoverec	20	2.7118021896044e-21	0.42888717098713	0.663	0.398	9.09484218349522e-17	WASHC4
Recoverec	20	2.77045817375706e-21	0.25131197428853ξ	0.383	0.161	9.29156262314643e-17	ZNFX1
Recoverec	20	3.58068844379152e-21	0.39231406999558	0.538	0.275	1.2008912902788e-16	DHX15
Recoverec	20	3.8978838763426e-21	0.44707447142073€	0.65	0.375	1.30727229444778e-16	RCOR1
Recoverec	20	4.0990078880551e-21	0.45960691908646	0.544	0.277	1.37472526549592e-16	ATP8B1
Recoverec	20	4.63680692262119e-21	0.39737749342719ξ	0.429	0.193	1.55509230570869e-16	PXK
Recoverec	20	5.13475001350749e-21	0.37411358838813	0.754	0.471	1.72209245953014e-16	DMXL1
Recoverec	20	5.21094092685695e-21	0.47748035336363	0.845	0.607	1.74764536804928e-16	UVRAG
Recoverec	20	5.68070096243915e-21	0.49919181886298	0.729	0.453	1.90519348878284e-16	ADGRE5

Recoverec	20	5.87853988620605e-21	0.40747751104302	0.857	0.586	1.97154470703579e-16	VPS13C
Recoverec	20	6.66456372618239e-21	0.37674319326129	0.775	0.484	2.23516138248705e-16	ELOVL5
Recoverec	20	6.84143103393337e-21	0.33303087026100	0.301	0.104	2.29447914016057e-16	CXCR5
Recoverec	20	7.13238354806023e-21	0.53647798526556	0.781	0.524	2.39205879434844e-16	JARID2
Recoverec	20	7.23615385809862e-21	0.40499092255592	0.468	0.229	2.42686128092912e-16	SLC25A32
Recoverec	20	7.42898759980246e-21	0.42521616096772	0.31	0.113	2.49153386122175e-16	TTN
Recoverec	20	7.64446740302557e-21	0.31360870675251	0.535	0.264	2.56380147762672e-16	SMC6
Recoverec	20	7.76236426274633e-21	0.42210850779469	0.526	0.272	2.60334172643987e-16	SPTLC2
Recoverec	20	8.10645928273158e-21	0.38748819356255	0.626	0.344	2.71874431424252e-16	RCSD1
Recoverec	20	8.14973693252842e-21	0.49113094388197	0.574	0.291	2.73325877243138e-16	SESN3
Recoverec	20	1.03350116879165e-20	0.40904892169065	0.635	0.347	3.46615621989342e-16	SP110
Recoverec	20	1.05780610855113e-20	0.49746739223530	0.888	0.657	3.54767012685878e-16	PRRC2C
Recoverec	20	1.07040686479293e-20	0.71499898426820	0.821	0.609	3.58993054314253e-16	CCSER1
Recoverec	20	1.08523823442974e-20	0.34096652364664	0.283	0.096	3.63967199063047e-16	FCER2
Recoverec	20	1.15653483276563e-20	0.46095590338048	0.465	0.22	3.87878652212937e-16	FAM117B
Recoverec	20	1.23502214801268e-20	0.41149653261344	0.362	0.151	4.14201728000491e-16	RASSF2
Recoverec	20	1.24167040498252e-20	0.26210650384783	0.249	0.078	4.16431420423037e-16	AC008878.3
Recoverec	20	1.25251887784889e-20	0.41016131107772	0.906	0.646	4.20069781252961e-16	DDX3X
Recoverec	20	1.477981506365e-20	0.48970439873485	0.565	0.315	4.95685437604695e-16	ZFX
Recoverec	20	1.52428449856786e-20	0.43767401735843	0.666	0.393	5.11214535129689e-16	C6orf106
Recoverec	20	1.60126822190935e-20	0.53598169666522	0.526	0.289	5.37033336263956e-16	PDE8A
Recoverec	20	1.60856504765401e-20	0.27137599365053	0.31	0.114	5.394805456822e-16	PAQR3
Recoverec	20	1.84363867164393e-20	0.43119421156927	0.52	0.267	6.18319537695942e-16	PAWR
Recoverec	20	1.93022969784959e-20	0.36790639488008	0.742	0.463	6.47360436064795e-16	SHOC2
Recoverec	20	2.0894852679899e-20	0.34909959870260	0.55	0.292	7.00771569178452e-16	STK10
Recoverec	20	2.09608323282655e-20	0.32631450735296	0.726	0.444	7.02984394625369e-16	CNOT1
Recoverec	20	2.24945056377132e-20	0.37910243601002	0.578	0.304	7.54420730077625e-16	CERS6
Recoverec	20	2.33735984823556e-20	0.41970148516054	0.851	0.611	7.83903745901242e-16	HLA-E
Recoverec	20	2.47362591767114e-20	0.46108594796855	0.647	0.378	8.29604660268545e-16	STIM2
Recoverec	20	2.50094780485503e-20	0.38866199826539	0.602	0.334	8.3876787479228e-16	AC001226.2
Recoverec	20	2.53541333751647e-20	0.50458191062706	0.857	0.648	8.50326925136273e-16	CAMK1D

Recoverec	20	2.5900286563445e-20	0.40713050143283;	0.298	0.11	8.68643810764818e-16	IL4R
Recoverec	20	2.67109013237749e-20	0.41475536383195!	0.435	0.208	8.95830208596764e-16	FBXO21
Recoverec	20	3.22104996826116e-20	0.52799698091408;	0.605	0.349	1.08027573835543e-15	SIK2
Recoverec	20	3.5571163101708e-20	0.36965221101837(	0.444	0.208	1.19298566810508e-15	SP4
Recoverec	20	3.63273411115743e-20	0.30879451129738	0.465	0.22	1.21834636619998e-15	AKNA
Recoverec	20	4.13006868375066e-20	0.54911023641845:	0.827	0.59	1.3851424351563e-15	RFX3
Recoverec	20	4.27001752750874e-20	0.42439452899554(	0.818	0.563	1.43207847837588e-15	COP1
Recoverec	20	4.90650138860977e-20	0.43185749090906(	0.62	0.359	1.64554243571194e-15	PTBP2
Recoverec	20	5.12024552610033e-20	0.34354285144189!	0.48	0.235	1.71722794454353e-15	MT1E
Recoverec	20	5.1553247394746e-20	0.44749440705274	0.66	0.402	1.72899281112499e-15	SMARCA2
Recoverec	20	5.39536767048993e-20	0.46929443584263;	0.629	0.369	1.80949840932891e-15	MTFR1
Recoverec	20	6.00162023729638e-20	0.43367785461341{	0.769	0.489	2.01282339518446e-15	SP140
Recoverec	20	6.5462963751587e-20	0.39683427025602	0.523	0.273	2.19549687830073e-15	OXSR1
Recoverec	20	7.39182058878219e-20	0.31774833603867	0.48	0.229	2.47906878906577e-15	GNB5
Recoverec	20	1.00476125124219e-19	0.33318863141068	0.489	0.238	3.36976828441606e-15	ZNF107
Recoverec	20	1.04473209426668e-19	0.47082203125896!	0.669	0.397	3.50382249775157e-15	NFKBID
Recoverec	20	1.17993547350146e-19	0.45195359969507:	0.657	0.386	3.95726759102918e-15	PEAK1
Recoverec	20	1.21703402089636e-19	0.59356158797578	0.869	0.632	4.08168869928222e-15	ZSWIM6
Recoverec	20	1.23445383669952e-19	0.51898181832420!	0.827	0.574	4.14011127752286e-15	NSD3
Recoverec	20	1.3076718288061e-19	0.59484625063659	0.939	0.796	4.38566977944991e-15	CDC42SE2
Recoverec	20	1.31291120027296e-19	0.46227192719488	0.736	0.463	4.40324158347544e-15	RLF
Recoverec	20	1.5835375116179e-19	0.2923169419253	0.359	0.152	5.31086810646412e-15	AL354733.3
Recoverec	20	1.77850930403174e-19	0.48023129934744	0.833	0.604	5.96476450386165e-15	SP100
Recoverec	20	1.82015995181668e-19	0.37061980093817!	0.632	0.374	6.10445244640277e-15	UBR1
Recoverec	20	1.87648567510981e-19	0.43863283363206;	0.371	0.164	6.29335765718328e-15	SLC25A25
Recoverec	20	1.96249707133943e-19	0.40022716488621!	0.748	0.482	6.58182267785817e-15	PPP2R2A
Recoverec	20	1.9672597933871e-19	0.39065295404432!	0.714	0.431	6.59779589506167e-15	CCND3
Recoverec	20	2.00443587888358e-19	0.28262551153406!	0.356	0.15	6.72247705059976e-15	TSPAN13
Recoverec	20	2.01731583959867e-19	0.65546468130479	0.729	0.494	6.76567386284602e-15	CAMK2D
Recoverec	20	2.10048321121691e-19	0.32468142109927!	0.699	0.423	7.04460059377928e-15	KPNA4
Recoverec	20	2.1009311576522e-19	0.76413700314179:	0.602	0.383	7.04610291653395e-15	TMEM123

Recoverec	20	2.23297337817578e-19	0.35729752096963	0.833	0.584	7.48894611572593e-15	WAC
Recoverec	20	2.29259662445964e-19	0.47418037794501	0.842	0.583	7.68891055911275e-15	TRAF3
Recoverec	20	2.50922963678817e-19	0.33785524115778	0.599	0.336	8.41545435586017e-15	RAD21
Recoverec	20	2.62720156963646e-19	0.46803692105458	0.593	0.334	8.81110862424675e-15	TET2
Recoverec	20	2.66181787664296e-19	0.49517195516464	0.647	0.389	8.92720479468517e-15	DIAPH2
Recoverec	20	2.80510863327657e-19	0.39527236132410	0.821	0.58	9.40777333428297e-15	SCAF11
Recoverec	20	3.1513593329727e-19	0.48456831592433	0.833	0.583	1.05690289309238e-14	LPP
Recoverec	20	3.52624103825755e-19	0.50553413774722	0.948	0.82	1.18263071941082e-14	EZR
Recoverec	20	4.02889604679714e-19	0.33609691533393	0.641	0.377	1.35121115617482e-14	NCF1
Recoverec	20	4.18798942046405e-19	0.44487268411866	0.708	0.419	1.40456789183523e-14	MAPRE2
Recoverec	20	4.48309945233298e-19	0.46553006815273	0.827	0.561	1.50354189432344e-14	PARP8
Recoverec	20	5.00391100180165e-19	0.45624293240539	0.909	0.722	1.67821167178424e-14	KMT2E
Recoverec	20	5.40667027281186e-19	0.38804096006477	0.474	0.238	1.81328907609564e-14	ATP2A3
Recoverec	20	5.55425467364127e-19	0.34600131720047	0.723	0.442	1.86278593244581e-14	CD2AP
Recoverec	20	5.62906498991332e-19	0.30327443789254	0.59	0.333	1.88787581631713e-14	ILF3
Recoverec	20	5.70462513376052e-19	0.34941163182531	0.663	0.4	1.9132171773606e-14	CMTM6
Recoverec	20	5.70585577644684e-19	0.30941379978551	0.541	0.289	1.91362991030474e-14	LMBR1
Recoverec	20	6.6003311749594e-19	0.49133032940187	0.869	0.627	2.21361906945788e-14	USP15
Recoverec	20	6.92432355542691e-19	0.40287649386912	0.416	0.191	2.32227963401908e-14	AC027097.2
Recoverec	20	7.05011322551495e-19	0.35470529666522	0.544	0.298	2.3644669735732e-14	ARHGAP17
Recoverec	20	7.97636873957289e-19	0.54605904894941	0.675	0.42	2.67511454787796e-14	BAZ1A
Recoverec	20	1.14091132080785e-18	0.41340688418524	0.66	0.397	3.82638838772536e-14	ADAM19
Recoverec	20	1.15733025979992e-18	0.35350142591771	0.526	0.275	3.88145422531698e-14	ABLIM1
Recoverec	20	1.1901515548669e-18	0.36228518840568	0.751	0.489	3.99153028471259e-14	STAG2
Recoverec	20	1.20714520271712e-18	0.37342869312544	0.623	0.36	4.04852358087267e-14	LNPEP
Recoverec	20	1.22158866306934e-18	0.27198732939305	0.666	0.385	4.09696405820195e-14	MTPN
Recoverec	20	1.22594581724199e-18	0.44189518242368	0.553	0.312	4.11157708186619e-14	IFT57
Recoverec	20	1.3278320703222e-18	0.32449613079694	0.322	0.131	4.4532831974466e-14	ARL13B
Recoverec	20	1.37077083426593e-18	0.39544100388324	0.83	0.573	4.59729122396106e-14	CYTH1
Recoverec	20	1.41796816716941e-18	0.25679868038088	0.34	0.137	4.75558163905277e-14	FGD2
Recoverec	20	1.44183130235086e-18	0.54710136193186	0.933	0.775	4.8356138218243e-14	SMCHD1

Recoverec	20	1.45909796942459e-18	0.32559998139209‹	0.34	0.142	4.89352276985618e-14	NFATC1
Recoverec	20	1.66066301511385e-18	0.39310772595580‹	0.733	0.442	5.56953162008881e-14	HLA-DQB1
Recoverec	20	1.7060751967356e-18	0.34537762318648‹	0.632	0.375	5.72183499481186e-14	ZBTB1
Recoverec	20	1.76989911109067e-18	0.44148915133810‹	0.821	0.573	5.9358876387759e-14	RAB30
Recoverec	20	1.77443500058775e-18	0.31570351862025‹	0.505	0.269	5.95110010497119e-14	IP6K1
Recoverec	20	1.9063485521328e-18	0.40492511269721‹	1	1	6.39351177414298e-14	MT-CO3
Recoverec	20	2.06861789836127e-18	0.37759844873922‹	0.581	0.327	6.93773070752404e-14	MTMR6
Recoverec	20	2.36699158694948e-18	0.30665782103504‹	0.495	0.26	7.93841638431118e-14	SAMD8
Recoverec	20	2.48045012102552e-18	0.34867504973694‹	0.666	0.4	8.3189336158954e-14	TRIM44
Recoverec	20	2.61504713920587e-18	0.37837313869839‹	0.578	0.298	8.77034509546864e-14	MARCH1
Recoverec	20	2.67358163270609e-18	0.28183891016892‹	0.28	0.108	8.96665807976968e-14	C5orf15
Recoverec	20	2.71736215040725e-18	0.30266167237704‹	0.407	0.19	9.11348918003582e-14	CCT5
Recoverec	20	3.41170276376674e-18	0.35750002441921	0.812	0.56	1.14421687291209e-13	HNRNPK
Recoverec	20	3.46104925828213e-18	0.2640058467275	0.304	0.119	1.16076670024266e-13	HLA-DOB
Recoverec	20	3.46614834492706e-18	0.48532175190758‹	0.404	0.195	1.16247683192164e-13	CCNYL1
Recoverec	20	3.72821403202897e-18	0.33753870691761‹	0.541	0.288	1.25036842206188e-13	EVL
Recoverec	20	4.49293195025179e-18	0.56356733205082‹	0.705	0.454	1.50683951747545e-13	COBLL1
Recoverec	20	4.69912415844245e-18	0.28271782302372‹	0.201	0.06	1.57599226025843e-13	AL035634.1
Recoverec	20	4.72785561213982e-18	0.27963968023425‹	0.702	0.413	1.58562821519945e-13	PPP4R3A
Recoverec	20	4.78164222600552e-18	0.30238995798358‹	0.359	0.157	1.60366716975773e-13	PIGB
Recoverec	20	4.95079337607655e-18	0.52149219722152‹	0.617	0.378	1.66039708246855e-13	ANTXR2
Recoverec	20	5.10970631831358e-18	0.47662104775940‹	0.842	0.613	1.71369330503601e-13	BACH1
Recoverec	20	5.40748313056914e-18	0.46740966568163‹	0.802	0.577	1.81356169233028e-13	IQGAP1
Recoverec	20	5.47326538142744e-18	0.38597555575159‹	0.851	0.617	1.83562374362314e-13	JAK1
Recoverec	20	6.04620825006415e-18	0.44571874891848‹	0.894	0.715	2.02777732290651e-13	MYCBP2
Recoverec	20	6.81571730024265e-18	0.36422964901437‹	0.693	0.437	2.28585526815538e-13	FBXW11
Recoverec	20	7.27002944958213e-18	0.26092403953023‹	0.267	0.096	2.43822247680085e-13	IL1RAP
Recoverec	20	8.05975267608784e-18	0.27926214840335‹	0.599	0.346	2.70307985250634e-13	RNF138
Recoverec	20	8.49853756764165e-18	0.51156106917764‹	0.942	0.817	2.85023952943566e-13	DENND4A
Recoverec	20	8.58922179926475e-18	0.29831630030810‹	0.544	0.302	2.88065320703741e-13	DCP1A
Recoverec	20	8.92630551003828e-18	0.26452130909554‹	0.234	0.079	2.99370434195664e-13	LINC01215

Recoverec	20	9.12018822189893e-18	0.38283492645345[illegible]	0.845	0.597	3.05872872586046e-13	PPP6R3
Recoverec	20	9.75508326647794e-18	0.53160003949567[illegible]	0.605	0.377	3.27165982591137e-13	HMBOX1
Recoverec	20	9.85160307683184e-18	0.38098689580474[illegible]	0.796	0.539	3.30403063990786e-13	CUL3
Recoverec	20	1.01777944785179e-17	0.29178865257449[illegible]	0.593	0.334	3.41342871220534e-13	USP7
Recoverec	20	1.03111143956137e-17	0.40651808645530[illegible]	0.796	0.538	3.45814154600092e-13	TRIO
Recoverec	20	1.13559266143307e-17	0.43442310961009	0.684	0.451	3.80855066791423e-13	THRAP3
Recoverec	20	1.26399954282171e-17	0.29875162315013[illegible]	0.672	0.408	4.23920166671546e-13	MOB1A
Recoverec	20	1.30680820021339e-17	0.36737153566230[illegible]	0.778	0.525	4.38277334187565e-13	SF3B1
Recoverec	20	1.33891954704659e-17	0.31347428743784[illegible]	0.584	0.332	4.49046837688487e-13	MAPK1
Recoverec	20	1.38596675477927e-17	0.34029654322680[illegible]	0.821	0.589	4.6482553021787e-13	TMEM131
Recoverec	20	1.42654269886346e-17	0.37613916167059[illegible]	0.438	0.224	4.78433890344828e-13	SBNO1
Recoverec	20	1.61818682333535e-17	0.38599574763627[illegible]	0.441	0.232	5.42707496810210e-13	ATP10D
Recoverec	20	1.62969685167198e-17	0.40159995040656[illegible]	0.334	0.144	5.46567730113747e-13	SAV1
Recoverec	20	1.64459573219403e-17	0.41472163125279[illegible]	0.839	0.567	5.51564516663234e-13	HLA-DRA
Recoverec	20	1.67467070262512e-17	0.37842884266714[illegible]	0.252	0.093	5.61651060246413e-13	ZNF516
Recoverec	20	1.71086801901389e-17	0.44859505961779[illegible]	0.842	0.594	5.73790916216878e-13	TNFAIP8
Recoverec	20	1.78555828180418e-17	0.39747165968202[illegible]	0.809	0.559	5.98840536551485e-13	SETX
Recoverec	20	1.79331064885969e-17	0.28869975273655[illegible]	0.653	0.399	6.01440525414562e-13	PRRC2B
Recoverec	20	1.86440479027264e-17	0.33044138682308[illegible]	0.845	0.595	6.25284078561637e-13	RHOA
Recoverec	20	1.89367994927491e-17	0.41418938745183[illegible]	0.775	0.513	6.3510238138782e-13	CD37
Recoverec	20	2.03993172636557e-17	0.32463150086556	0.647	0.392	6.84152302388484e-13	FLI1
Recoverec	20	2.4347688531621e-17	0.31205760217385[illegible]	0.486	0.242	8.16572777973504e-13	LBH
Recoverec	20	2.56195795216869e-17	0.37941018813870[illegible]	0.587	0.345	8.59229457998336e-13	BDP1
Recoverec	20	2.59479044961675e-17	0.41364734171600[illegible]	0.857	0.635	8.70240820992465e-13	TBC1D22A
Recoverec	20	2.77359849367217e-17	0.42490179886391[illegible]	0.593	0.35	9.30209462807774e-13	ADIPOR2
Recoverec	20	2.80422761840223e-17	0.33581643225014[illegible]	0.632	0.382	9.40481858659741e-13	USP12
Recoverec	20	2.80714189463445e-17	0.305603111411998	0.556	0.304	9.41459248622502e-13	ARAP2
Recoverec	20	2.97622232551046e-17	0.27772891562434[illegible]	0.477	0.254	9.98165443529699e-13	CLEC16A
Recoverec	20	3.18897143360671e-17	0.36901340174594[illegible]	0.897	0.645	1.06951723940302e-12	DYRK1A
Recoverec	20	3.36231482789375e-17	0.31548863928899[illegible]	0.477	0.256	1.12765314697901e-12	STX12
Recoverec	20	3.48555063249376e-17	0.32454691301917[illegible]	0.666	0.409	1.16898397112576e-12	SYK

Recoverec	20	3.59872175225147e-17	0.38830200935723ε	0.632	0.386	1.2069393012701e-12	ZDHHC21
Recoverec	20	3.71179432092253e-17	0.289988970119401	0.605	0.359	1.244861579351e-12	XRCC6
Recoverec	20	3.88095748877864e-17	0.29478713915915	0.657	0.392	1.30159552258658e-12	RNF111
Recoverec	20	3.9301332236709e-17	0.34730313332301	0.377	0.166	1.31808808055475e-12	AP000787.1
Recoverec	20	3.98128293773974e-17	0.33152602055885	0.62	0.364	1.33524267165915e-12	SIDT1
Recoverec	20	3.99968931926677e-17	0.35730311692630	0.426	0.214	1.34141580389569e-12	USP32
Recoverec	20	4.13976983334351e-17	0.33688522915186	0.459	0.238	1.38839600670675e-12	PRPF8
Recoverec	20	4.3503588310186e-17	0.37083550591232	0.298	0.125	1.45902334474702e-12	TTC39B
Recoverec	20	4.85660844672075e-17	0.26757909457168ε	0.489	0.257	1.6288093408612e-12	AC010894.3
Recoverec	20	4.86627066324531e-17	0.34449078083081	0.796	0.545	1.63204985503921e-12	FRYL
Recoverec	20	5.11667833937742e-17	0.37116309939359	0.684	0.44	1.7160315814604e-12	PSME4
Recoverec	20	5.29378574926976e-17	0.32257724721968	0.714	0.458	1.77542986459009e-12	SKAP2
Recoverec	20	5.30016996563885e-17	0.36505047313402	0.532	0.29	1.77757100307596e-12	MANBA
Recoverec	20	5.60435963184934e-17	0.38846817201356	0.736	0.495	1.87959013332963e-12	CTSS
Recoverec	20	5.7276689473832e-17	0.34203133633988	0.483	0.254	1.92094561157338e-12	GNA12
Recoverec	20	6.45487657162617e-17	0.41958202259566	0.562	0.322	2.16483650459199e-12	RABGAP1
Recoverec	20	6.90889305378987e-17	0.39801506853983	0.723	0.478	2.31710455238005e-12	SPAG9
Recoverec	20	7.40211078048677e-17	0.43644504795857	0.799	0.558	2.48251991355965e-12	SRPK2
Recoverec	20	7.67592185553028e-17	0.29198917265497	0.508	0.277	2.57435067190775e-12	VPS41
Recoverec	20	7.72725362353633e-17	0.29305842952082	0.574	0.333	2.59156632026161e-12	KPNB1
Recoverec	20	8.12669292578874e-17	0.40679707348143	0.739	0.48	2.72553027345103e-12	CDK13
Recoverec	20	8.38980411948511e-17	0.283231531072911	0.617	0.375	2.81377250559292e-12	DHX9
Recoverec	20	8.47015604565184e-17	0.37003226578844	0.802	0.57	2.84072093459071e-12	EPS15
Recoverec	20	8.5821948717007e-17	0.35574791960007	0.83	0.575	2.87829651607098e-12	PAFAH1B1
Recoverec	20	8.64128046399883e-17	0.42058011393054	0.824	0.591	2.89811264201593e-12	CHD2
Recoverec	20	9.0070762672542e-17	0.42934817956037	0.261	0.101	3.02079323851171e-12	MT1M
Recoverec	20	9.11252244324407e-17	0.32995125985337	0.875	0.617	3.0561577770152e-12	SFPQ
Recoverec	20	9.23800723877364e-17	0.27411554980305	0.702	0.431	3.0982428677399e-12	GATAD2B
Recoverec	20	9.95981847531728e-17	0.35656128099841	0.632	0.396	3.34032392025191e-12	PTBP3
Recoverec	20	1.00300843687806e-16	0.31777963619299	0.438	0.22	3.36388969560165e-12	SLC25A33
Recoverec	20	1.04530908018718e-16	0.38629349915399	0.243	0.091	3.50575759313175e-12	IL21R

Recoverec	20	1.06380198011325e-16	0.48955244117583	0.605	0.368	3.56777908090381e-12	TOP2B
Recoverec	20	1.29682539291093e-16	0.27798283731076	0.316	0.133	4.34929300274467e-12	SRGAP2
Recoverec	20	1.29722231991942e-16	0.27765608867077	0.565	0.331	4.35062421654576e-12	PPIL4
Recoverec	20	1.34166186535708e-16	0.34212454421676	0.733	0.483	4.49966556403458e-12	LCOR
Recoverec	20	1.35280917490268e-16	0.29343119285112	0.474	0.251	4.53705141078862e-12	CEPT1
Recoverec	20	1.55626345802899e-16	0.34888676549613	0.596	0.357	5.21939638553764e-12	ADD1
Recoverec	20	1.57518686877983e-16	0.30768999556715	0.532	0.295	5.2828617205138e-12	NUP58
Recoverec	20	1.57949096181615e-16	0.25830385200925	0.429	0.215	5.29729678773902e-12	NIPA2
Recoverec	20	1.5829669192813e-16	0.31193847321846	0.465	0.248	5.30895445388561e-12	CASP4
Recoverec	20	1.68009337816786e-16	0.39549156133443	0.611	0.373	5.63469717169937e-12	RPS6KA3
Recoverec	20	1.69543387171231e-16	0.42196251962437	0.781	0.542	5.68614611894875e-12	ITSN2
Recoverec	20	2.00886524140593e-16	0.39136957704313	0.769	0.549	6.73733224662719e-12	MACF1
Recoverec	20	2.1595049566028e-16	0.28706183502693	0.59	0.336	7.24254772345448e-12	IVNS1ABP
Recoverec	20	2.3658057713368e-16	0.62720497023509	0.781	0.576	7.93443939590935e-12	ARID5B
Recoverec	20	2.4961471592988e-16	0.25267915263415	0.486	0.269	8.3715783428563e-12	TRRAP
Recoverec	20	2.51644460943564e-16	0.26874949602030	0.477	0.251	8.43965193112525e-12	C2CD5
Recoverec	20	2.61012804745192e-16	0.36005428001980	0.736	0.468	8.75384744554426e-12	DOCK10
Recoverec	20	2.7731775714684e-16	0.43231303784642	0.802	0.558	9.30068293919071e-12	PAPOLA
Recoverec	20	2.79417576358824e-16	0.41299587207471	0.815	0.582	9.37110667592223e-12	PDE4B
Recoverec	20	2.87289808518356e-16	0.50362828607958	0.805	0.595	9.63512559808863e-12	RALGAPA1
Recoverec	20	2.88241240690861e-16	0.33549711655758	0.277	0.109	9.66703473029008e-12	CPED1
Recoverec	20	2.90045671759361e-16	0.35763962752020	0.477	0.254	9.72755173946546e-12	GSAP
Recoverec	20	2.90703615114461e-16	0.36138122628672	0.714	0.443	9.74961784370879e-12	ZC3HAV1
Recoverec	20	2.95188610167926e-16	0.35611974427773	0.426	0.22	9.90003560781191e-12	SMC1A
Recoverec	20	3.07425865424016e-16	0.37994404181774	0.638	0.39	1.03104486745906e-11	BLK
Recoverec	20	3.15959635190551e-16	0.43063239757898	0.593	0.367	1.05966542450207e-11	MAP3K8
Recoverec	20	3.19586021123576e-16	0.48949656574973	0.66	0.446	1.07182759764425e-11	SSBP2
Recoverec	20	3.65048983051009e-16	0.39581134909403	0.848	0.625	1.22430127935647e-11	NCL
Recoverec	20	3.8787829349191e-16	0.28356622054276	0.629	0.383	1.30086622071317e-11	NUTM2B-AS1
Recoverec	20	4.1758291322213e-16	0.29096766951381	0.395	0.197	1.40048957436438e-11	CUL2
Recoverec	20	4.25179931191754e-16	0.31119874262886	0.523	0.293	1.42596845323091e-11	C1GALT1

Recoverec	20	4.27014165272797e-16	0.26894658003802:	0.252	0.096	1.43212010749191e-11	CDCA7L
Recoverec	20	4.32867900849311e-16	0.417113787290497	0.775	0.539	1.45175236586842e-11	SENP6
Recoverec	20	4.42726060455489e-16	0.31435584704509:	0.796	0.553	1.48481466155562e-11	MAP3K2
Recoverec	20	4.67757114819327e-16	0.30900003000247	0.389	0.191	1.56876381168106e-11	MIOS
Recoverec	20	4.74667898641587e-16	0.29929153583455	0.55	0.319	1.59194119846415e-11	USP10
Recoverec	20	4.92202668312689e-16	0.43748663964442	0.836	0.615	1.6507493089871e-11	ZNF644
Recoverec	20	4.99092470952641e-16	0.36774609036778	0.38	0.187	1.67385632908097e-11	STRN
Recoverec	20	5.37816496544298e-16	0.36458516634717:	0.502	0.279	1.80372896611027e-11	ATL2
Recoverec	20	5.62414263173632e-16	0.30095475517658	0.587	0.352	1.88622495583173e-11	ADD3
Recoverec	20	5.74480967690265e-16	0.34076587497205	0.687	0.454	1.92669426943961e-11	SREBF2
Recoverec	20	5.88860594292043e-16	0.31286168141487:	0.857	0.621	1.97492066113665e-11	RAB11FIP1
Recoverec	20	6.06998614121996e-16	0.33229782957936	0.337	0.152	2.03575195204235e-11	SNX8
Recoverec	20	6.27534023925281e-16	0.25155106000569:	0.526	0.283	2.10462360944061e-11	CD79B
Recoverec	20	6.36882608124936e-16	0.29164981754644	0.635	0.39	2.13597689112941e-11	HCLS1
Recoverec	20	6.42543343096059e-16	0.27832147346634	0.678	0.411	2.15496186407556e-11	TAGAP
Recoverec	20	6.50494120858778e-16	0.29853639289758:	0.553	0.316	2.18162718253617e-11	ARHGEF7
Recoverec	20	7.50740726909321e-16	0.28858914500141	0.495	0.269	2.51783424990848e-11	TMPO
Recoverec	20	8.02601096121897e-16	0.311527618118751	0.514	0.294	2.69176355617362e-11	WDR20
Recoverec	20	8.17867724148377e-16	0.37110956722045:	0.489	0.281	2.74296477324883e-11	JAK2
Recoverec	20	8.2134220343174e-16	0.39273074324143:	0.647	0.427	2.75461748186937e-11	BOD1L1
Recoverec	20	8.66832661159106e-16	0.28671901970308	0.638	0.395	2.90718337899541e-11	CD47
Recoverec	20	8.83577965121719e-16	0.34190991958172:	0.517	0.283	2.96334377942522e-11	LPGAT1
Recoverec	20	8.94286323459137e-16	0.37555563726539	0.547	0.32	2.99925747161725e-11	LEMD3
Recoverec	20	9.10319736884506e-16	0.394165796116067	0.264	0.104	3.05303033356326e-11	BCL6
Recoverec	20	9.80555101517078e-16	0.26140860633889	0.343	0.162	3.28858569946798e-11	PIK3C2B
Recoverec	20	9.98656823332694e-16	0.31529681152994	0.544	0.314	3.34929525409319e-11	INO80
Recoverec	20	1.02452377465391e-15	0.32122691733599	0.851	0.583	3.4360478354343e-11	ITPR2
Recoverec	20	1.08811799232848e-15	0.25814139363327	0.432	0.221	3.64933012267126e-11	MAPK8IP3
Recoverec	20	1.10394234518821e-15	0.29088053210159	0.413	0.212	3.70240183729222e-11	SLC36A4
Recoverec	20	1.13483729098437e-15	0.27227717961081	0.432	0.217	3.80601730650337e-11	SPOPL
Recoverec	20	1.28110226633556e-15	0.35669320815857:	0.416	0.213	4.29656078083619e-11	PIK3CA

Recoverec	20	1.3108905980404e-15	0.31884589120335	0.666	0.417	4.39646488770789e-11	SNX25
Recoverec	20	1.32495339159133e-15	0.39877407127409	0.617	0.387	4.44362868471902e-11	ST8SIA4
Recoverec	20	1.34872672924318e-15	0.32741689609650	0.657	0.414	4.52335970453577e-11	TGFBR2
Recoverec	20	1.35581904305508e-15	0.31090371578071	0.653	0.404	4.54714590659813e-11	NUP153
Recoverec	20	1.37353194167497e-15	0.32112427994967	0.827	0.601	4.60655142598953e-11	PUM2
Recoverec	20	1.47298933125768e-15	0.32738183430476	0.778	0.544	4.940111619172e-11	GPCPD1
Recoverec	20	1.52314554681133e-15	0.43817078336362	0.483	0.277	5.10832553489585e-11	APLP2
Recoverec	20	1.54457902946466e-15	0.30327706152775	0.565	0.325	5.18020914901857e-11	CAPZA2
Recoverec	20	1.62738050373769e-15	0.37363571939443	0.313	0.139	5.45790873343546e-11	METTL21A
Recoverec	20	2.06232747398057e-15	0.28576661424588	0.69	0.45	6.91663388223603e-11	PNN
Recoverec	20	2.13774455581581e-15	0.28887420753604	0.729	0.496	7.16956769129505e-11	CNOT4
Recoverec	20	2.1411292467276e-15	0.41368035537336	0.745	0.509	7.18091926767502e-11	AP001011.1
Recoverec	20	2.15871464819345e-15	0.43063740622017	0.72	0.5	7.23989718711119e-11	AKT3
Recoverec	20	2.1895592878945e-15	0.29212400773033	0.568	0.339	7.34334393974058e-11	FBXO34
Recoverec	20	2.219790419028e-15	0.26792720810157	0.69	0.435	7.4447331073361e-11	EMB
Recoverec	20	2.3823332589431e-15	0.27031686660230	0.21	0.071	7.98986928384335e-11	KLHL14
Recoverec	20	2.65072029424622e-15	0.28826544726549	0.486	0.267	8.88998572284296e-11	DNM1L
Recoverec	20	2.77892656080097e-15	0.34299449889745	0.881	0.654	9.31996389961429e-11	VPS13B
Recoverec	20	2.92903527050602e-15	0.34288477925364	0.602	0.374	9.82339849022307e-11	PPM1B
Recoverec	20	3.13657267040702e-15	0.39501013628790	0.207	0.071	1.05194374220111e-10	LINC01811
Recoverec	20	3.15754438608617e-15	0.29392148178393	0.371	0.179	1.05897723620558e-10	AC009554.1
Recoverec	20	3.2035382108899e-15	0.27012628594623	0.38	0.186	1.07440264516825e-10	TNFRSF10B
Recoverec	20	3.2760478966599e-15	0.29809553221924	0.669	0.422	1.0987209435818e-10	NASP
Recoverec	20	3.49424592043941e-15	0.26846514951137	0.672	0.434	1.17190019679697e-10	SMARCC1
Recoverec	20	3.54796541041005e-15	0.29615579204692	0.429	0.23	1.18991663934332e-10	FAM91A1
Recoverec	20	3.62686356695939e-15	0.33408145317411	0.647	0.405	1.21637750308684e-10	KDM5A
Recoverec	20	3.63137781204583e-15	0.27797656728097	0.492	0.277	1.21789149060393e-10	SFSWAP
Recoverec	20	3.64617853937594e-15	0.41774964846923	0.426	0.23	1.2228553585359e-10	GALNT10
Recoverec	20	3.81155176220311e-15	0.45987548040706	0.751	0.538	1.27831823000768e-10	ZEB1
Recoverec	20	4.06371218227941e-15	0.27359070527227	0.295	0.13	1.36288779169287e-10	CEP295
Recoverec	20	4.11948864452855e-15	0.35945548674756	0.736	0.489	1.38159410160199e-10	GPATCH8

Recoverec	20	4.17371113576416e-15	0.35011880024649З	0.495	0.281	1.39977924071258e-10	NCK2
Recoverec	20	4.26776537001886e-15	0.450274837209427	0.556	0.339	1.43132314979692e-10	ATF7IP2
Recoverec	20	4.29105627702092e-15	0.308648531862617	0.681	0.434	1.43913445418728e-10	RNF38
Recoverec	20	5.01081930204844e-15	0.416636073095592	0.59	0.354	1.68052857752101e-10	PIGR
Recoverec	20	5.55542966587421e-15	0.256755603963794	0.337	0.16	1.86318000134089e-10	SFT2D2
Recoverec	20	5.61790467705585e-15	0.303411454679676	0.553	0.328	1.88413287059099e-10	XPO1
Recoverec	20	5.62467306611441e-15	0.511534076169405	0.413	0.218	1.88640285291345e-10	GCH1
Recoverec	20	5.9599843617835e-15	0.338219177438395	0.407	0.216	1.99885955525495e-10	CSNK1D
Recoverec	20	6.00044629173239e-15	0.273933231262417	0.322	0.149	2.01242967732121e-10	ZNF267
Recoverec	20	6.01486591970376e-15	0.40132850961033	0.696	0.474	2.01726573215025e-10	SMG1
Recoverec	20	6.15103357250682e-15	0.30495524760293	0.593	0.37	2.06293363954734e-10	USP8
Recoverec	20	6.44975678489917e-15	0.337233115004546	0.763	0.518	2.16311943051948e-10	SUCLG2
Recoverec	20	6.6448795768034e-15	0.286518867351242	0.772	0.514	2.22855971246832e-10	CTNNB1
Recoverec	20	6.92162043253733e-15	0.364842234623526	0.736	0.52	2.32137306066437e-10	SETD2
Recoverec	20	6.93525313585791e-15	0.329288333642578	0.508	0.285	2.32594519670402e-10	DTNBP1
Recoverec	20	7.01346810227284e-15	0.449196188537115	0.875	0.682	2.35217693214027e-10	STK4
Recoverec	20	7.35979045470431e-15	0.363104918594893	0.465	0.267	2.46832652269873e-10	MTMR1
Recoverec	20	8.25425878261564e-15	0.325913616512054	0.565	0.342	2.76831331051363e-10	GPBP1L1
Recoverec	20	8.39989627176659e-15	0.299925854243509	0.605	0.372	2.81715721162508e-10	RABEP1
Recoverec	20	9.60233757180967e-15	0.390129534645495	0.255	0.102	3.22043197483353e-10	PDE7B
Recoverec	20	1.00971365471196e-14	0.439771806716687	0.729	0.49	3.38637765517297e-10	WNK1
Recoverec	20	1.04176150827387e-14	0.371401218154332	0.483	0.275	3.49385974644889e-10	CEP170
Recoverec	20	1.06993127865613e-14	0.281925255867547	0.632	0.407	3.58833552235693e-10	MARCH7
Recoverec	20	1.07008371751491e-14	0.347272241609366	0.486	0.283	3.5888467718015e-10	TTC7A
Recoverec	20	1.07255894739496e-14	0.357158142420598	0.647	0.422	3.59714819777321e-10	SMC5
Recoverec	20	1.12855679565703e-14	0.328336064336413	0.672	0.448	3.78495378127453e-10	IFI16
Recoverec	20	1.13089907815706e-14	0.320026921496482	0.547	0.333	3.79280932832315e-10	SYAP1
Recoverec	20	1.15787525177111e-14	0.367212345874628	0.599	0.374	3.88328201938995e-10	VRK2
Recoverec	20	1.18836334946495e-14	0.365783218668493	0.581	0.363	3.98553300143554e-10	DGKD
Recoverec	20	1.21150192956368e-14	0.253093119381814	0.204	0.073	4.06313517137068e-10	GBP1
Recoverec	20	1.21226435569447e-14	0.264806344228032	0.617	0.388	4.0656921961281e-10	EIF2S3

Recoverec	20	1.22181070821238e-14	0.330683220200111	0.839	0.627	4.09770875320269e-10	NFKB1
Recoverec	20	1.28560361201214e-14	0.32499304595234	0.617	0.389	4.31165739396631e-10	CSNK1G3
Recoverec	20	1.33276541753747e-14	0.37535808894382	0.763	0.529	4.46982865733717e-10	TPM3
Recoverec	20	1.35682842848062e-14	0.27859045300038	0.407	0.216	4.55053118343829e-10	IMMT
Recoverec	20	1.40759607101614e-14	0.33371129429497	0.529	0.321	4.72079570297394e-10	CAP1
Recoverec	20	1.48064132273586e-14	0.30541225225982	0.757	0.504	4.96577486819153e-10	CXCR4
Recoverec	20	1.48576206676666e-14	0.32634645936231	0.55	0.309	4.98294881952203e-10	TUBA1A
Recoverec	20	1.51160533983e-14	0.32937481071372	0.875	0.682	5.06962198872185e-10	BRAF
Recoverec	20	1.55118421580028e-14	0.30067245456899	0.884	0.66	5.20236162295098e-10	USP34
Recoverec	20	1.65166387480802e-14	0.27491161722344	0.52	0.301	5.53935030333113e-10	ANKRD13A
Recoverec	20	1.66521793958418e-14	0.32752902763768	0.635	0.414	5.58480792577743e-10	FAM13B
Recoverec	20	1.70831534598706e-14	0.33022298978273	0.465	0.255	5.72934800737141e-10	MORC3
Recoverec	20	1.73658894580949e-14	0.34277476472029	0.845	0.62	5.82417200645585e-10	HNRNPU
Recoverec	20	1.8254392992132e-14	0.36984909007220	0.672	0.44	6.12215832170124e-10	HIF1A
Recoverec	20	2.00880535047182e-14	0.38254762708049	0.581	0.364	6.7371313844124e-10	FARP2
Recoverec	20	2.03624710317719e-14	0.39570209984251	0.48	0.278	6.82916553463568e-10	ZMYND11
Recoverec	20	2.27494837413669e-14	0.28411415629104	0.386	0.2	7.62972185717964e-10	G2E3
Recoverec	20	2.28737826205169e-14	0.25467866250007	0.678	0.438	7.67140921526894e-10	EPC2
Recoverec	20	2.29834181310467e-14	0.42141809961320	0.681	0.45	7.70817877279046e-10	ITGA4
Recoverec	20	2.60263118221049e-14	0.32657342370077	0.477	0.271	8.72870445889753e-10	CEP192
Recoverec	20	2.67956051669577e-14	0.39831410581078	0.708	0.503	8.98671006089429e-10	LMBRD1
Recoverec	20	2.75697030547945e-14	0.30021646194758	0.486	0.286	9.24632701051699e-10	CDC27
Recoverec	20	2.83513992084911e-14	0.38077653330005	0.337	0.163	9.50849226654375e-10	INPP5F
Recoverec	20	2.84978293960532e-14	0.28570842839815	0.465	0.251	9.55760202284832e-10	SLC23A2
Recoverec	20	2.86530173061812e-14	0.33782698395580	0.541	0.32	9.60964894414705e-10	EPB41L2
Recoverec	20	2.90498930730022e-14	0.34746285628934	0.948	0.77	9.74275313882348e-10	PLCG2
Recoverec	20	3.03087872308623e-14	0.29623501835898	0.851	0.627	1.01649610614866e-09	ATRX
Recoverec	20	3.05402334052925e-14	0.25254988890151	0.444	0.239	1.0242583479467e-09	PLEKHF2
Recoverec	20	3.08768998460139e-14	0.32759616606131	0.748	0.519	1.03554946703561e-09	ROCK1
Recoverec	20	3.21409893587583e-14	0.36760765475491	0.872	0.684	1.07794450111404e-09	ELMO1
Recoverec	20	3.43444240484681e-14	0.27818572935698	0.827	0.597	1.15184329373752e-09	SEPT7

Recoverec	20	3.56194148716493e-14	0.29406206717383‹	0.571	0.343	1.19460393596538e-09	ARHGAP25
Recoverec	20	3.56416928386712e-14	0.26106718335986‹	0.471	0.267	1.19535109442335e-09	PDCD10
Recoverec	20	3.86351515426695e-14	0.30232899084819‹	0.453	0.259	1.29574571243805e-09	NPAT
Recoverec	20	3.96614267033999e-14	0.37032319436083‹	0.347	0.173	1.33016492877863e-09	ALPK1
Recoverec	20	4.00843909237051e-14	0.32784417063886‹	0.614	0.385	1.34435030279922e-09	USP25
Recoverec	20	4.45124096701446e-14	0.28934584446245‹	0.742	0.503	1.49285719551731e-09	RIC1
Recoverec	20	4.48354281376813e-14	0.25154606348625‹	0.474	0.268	1.50369058888155e-09	IQSEC1
Recoverec	20	4.58735245084933e-14	0.42202468984637‹	0.271	0.117	1.53850626496585e-09	LINC01572
Recoverec	20	4.62863220842412e-14	0.26093786407805‹	0.672	0.435	1.55235067006128e-09	R3HDM2
Recoverec	20	4.84950663997471e-14	0.28644930765849‹	0.511	0.304	1.62642753691472e-09	TNKS2
Recoverec	20	4.88709869880721e-14	0.36955284214079‹	0.903	0.724	1.63903516160596e-09	LRBA
Recoverec	20	4.9790256558359e-14	0.27734242454893‹	0.647	0.425	1.66986562445424e-09	PRPF4B
Recoverec	20	5.18726912153999e-14	0.28831702734879‹	0.653	0.415	1.73970631798208e-09	EP300
Recoverec	20	5.53437700748535e-14	0.36886740391898‹	0.805	0.587	1.85611936077044e-09	FAM172A
Recoverec	20	5.57166713679482e-14	0.35904672382224‹	0.438	0.236	1.86862572433825e-09	TRPS1
Recoverec	20	5.67918969569933e-14	0.61274729003908	0.356	0.188	1.90468664014364e-09	L3MBTL4
Recoverec	20	5.80235732378865e-14	0.26755759728742‹	0.441	0.242	1.94599459925224e-09	HTT
Recoverec	20	5.9241612263596e-14	0.34140520057871‹	0.581	0.359	1.98684519209648e-09	STIM1
Recoverec	20	6.01043085463624e-14	0.36199413098602‹	0.377	0.197	2.0157783000279e-09	ZMYM5
Recoverec	20	6.40031694119627e-14	0.25833708093333‹	0.307	0.144	2.1465382957384e-09	RRM2B
Recoverec	20	6.80817282277977e-14	0.31676664952300‹	0.419	0.232	2.28332500130388e-09	EMSY
Recoverec	20	7.3667211836099e-14	0.30236317590118‹	0.401	0.213	2.47065095055909e-09	DCP2
Recoverec	20	7.51394182661833e-14	0.29974902660442‹	0.626	0.408	2.52002580981125e-09	ANGPTL1
Recoverec	20	7.68490151990819e-14	0.25058835529069‹	0.31	0.145	2.57736227174681e-09	SAMD9
Recoverec	20	7.93220762582101e-14	0.26386235152894‹	0.508	0.293	2.66030379354785e-09	AEBP2
Recoverec	20	8.09370084501542e-14	0.27814429774743‹	0.775	0.544	2.71446538940127e-09	EIF4G2
Recoverec	20	8.339326300143e-14	0.27875055083972‹	0.28	0.122	2.79684325454196e-09	FAM210A
Recoverec	20	8.73685874982375e-14	0.28322084521141‹	0.702	0.487	2.93016768751589e-09	OGA
Recoverec	20	8.94892924883156e-14	0.38189550414053‹	0.584	0.358	3.00129189147313e-09	SLC4A7
Recoverec	20	8.99556501220819e-14	0.55935905216315‹	0.386	0.209	3.01693259379438e-09	LIX1-AS1
Recoverec	20	9.04306361563163e-14	0.28775026939971	0.739	0.506	3.03286267541053e-09	TSC22D2

Recoverec	20	9.12092691809777e-14	0.32160917708853(	0.438	0.237	3.05897646979163e-09	CDK19
Recoverec	20	9.75546628907352e-14	0.52699884113885(	0.404	0.218	3.27178828402948e-09	PBX3
Recoverec	20	9.8284855747217e-14	0.31214665412561(	0.632	0.413	3.29627749205016e-09	FARS2
Recoverec	20	1.04212537544432e-13	0.27353231067604	0.365	0.189	3.49508008416517e-09	FBXO28
Recoverec	20	1.16293093185293e-13	0.26436604515126(	0.556	0.326	3.90023775924835e-09	SLC12A2
Recoverec	20	1.18180333199978e-13	0.31812873839652(	0.726	0.488	3.96353201486086e-09	SPPL3
Recoverec	20	1.19770292972708e-13	0.25913041768206(	0.535	0.327	4.01685608571867e-09	PSEN1
Recoverec	20	1.21139376913641e-13	0.32896996209221	0.711	0.494	4.0627724229297e-09	TENT2
Recoverec	20	1.41982469185315e-13	0.36695258649960(	0.547	0.339	4.76180805153711e-09	RNMT
Recoverec	20	1.4209760136731e-13	0.28003743071037(	0.517	0.31	4.76566935465683e-09	PRKAR1A
Recoverec	20	1.52684984034903e-13	0.26416590547815(	0.623	0.396	5.12074899456258e-09	MAPK1IP1L
Recoverec	20	1.54267615320075e-13	0.31018251094043(	0.769	0.532	5.17382728260466e-09	TAOK3
Recoverec	20	1.54952002493287e-13	0.28822480108389(	0.511	0.301	5.19678025961985e-09	SPTBN1
Recoverec	20	1.60381597401685e-13	0.31052697690086	0.502	0.299	5.37887801365772e-09	TLN1
Recoverec	20	1.64278187822102e-13	0.25424487186911(	0.128	0.034	5.50956186317766e-09	LINC01684
Recoverec	20	1.65451830678884e-13	0.39634261724793(	0.578	0.376	5.54892349730841e-09	KCMF1
Recoverec	20	1.67856956214838e-13	0.28810341920698(	0.66	0.436	5.62958659753323e-09	TAF15
Recoverec	20	1.73955404740274e-13	0.49911441488376(	0.571	0.361	5.83411636417931e-09	ANK3
Recoverec	20	1.83565108262939e-13	0.30625627089229(	0.365	0.19	6.15640660092244e-09	CCND2
Recoverec	20	1.91773464861556e-13	0.27926668873260(	0.362	0.188	6.43169846452688e-09	RMND5A
Recoverec	20	1.97202741535155e-13	0.31023563525651	0.702	0.476	6.61378554560604e-09	STAT3
Recoverec	20	2.04111301737687e-13	0.25485010417817(	0.76	0.507	6.84548483767854e-09	PHF3
Recoverec	20	2.05086272922504e-13	0.27541682088009(	0.574	0.363	6.87818342127495e-09	HOOK3
Recoverec	20	2.07181536203818e-13	0.26892216539601(	0.584	0.36	6.94845436120363e-09	SYNCRIP
Recoverec	20	2.14491668257021e-13	0.26634807440513(	0.407	0.221	7.19362157000398e-09	PPP2R5A
Recoverec	20	2.17673722003042e-13	0.25718738630845(	0.708	0.476	7.30034128853803e-09	RAB3GAP1
Recoverec	20	2.29383467648036e-13	0.34876472106193(	0.833	0.609	7.69306273797985e-09	HNRNPA1
Recoverec	20	2.50709204622569e-13	0.43024822268790(	0.553	0.339	8.40828530463172e-09	USP36
Recoverec	20	2.53749340789163e-13	0.27816795414211(	0.413	0.22	8.51024539138694e-09	AP1S3
Recoverec	20	2.57148472635413e-13	0.26720272334036(	0.845	0.613	8.62424547524647e-09	FUS
Recoverec	20	2.63525001042339e-13	0.26176555932965(	0.489	0.283	8.83810148495796e-09	SIRT1

Recoverec	20	2.71833087449526e-13	0.27425916766038{	0.523	0.319	9.11673808688220e-09	UBR4
Recoverec	20	2.73680795331341e-13	0.27469499638299{	0.286	0.13	9.17870651382252e-09	XAF1
Recoverec	20	2.95714716755226e-13	0.25691086199685{	0.581	0.36	9.91768017053678e-09	AGFG1
Recoverec	20	3.18794106693444e-13	0.25580788611415{	0.699	0.46	1.06917167502847e-08	RC3H1
Recoverec	20	3.58210171035011e-13	0.32261915571751	0.739	0.501	1.20136527161722e-08	MAP3K1
Recoverec	20	3.72116210087785e-13	0.26060519835156{	0.462	0.268	1.24800334539241e-08	FRS2
Recoverec	20	3.72543985038917e-13	0.25833271092675	0.778	0.544	1.24943801702352e-08	ACAP2
Recoverec	20	3.76981091533646e-13	0.36472205307489{	0.578	0.371	1.26431918478554e-08	PDCD6IP
Recoverec	20	3.93762488269064e-13	0.41201386945392{	0.444	0.252	1.32060063315679e-08	E2F5
Recoverec	20	3.98881110971808e-13	0.28791962709635{	0.407	0.224	1.33776746997725e-08	ABR
Recoverec	20	4.03079595545953e-13	0.27940509565197	0.441	0.249	1.35184834754202e-08	TP53BP2
Recoverec	20	4.71345125410946e-13	0.30113414972660{	0.55	0.345	1.58079728160323e-08	JPX
Recoverec	20	4.76835533477517e-13	0.28399645460440{	0.587	0.381	1.5992110121769e-08	NEMF
Recoverec	20	4.88205767580892e-13	0.27288242498763{	0.669	0.454	1.6373445033128e-08	YWHAQ
Recoverec	20	4.92792378865255e-13	0.33680129288623{	1	0.985	1.65272708023829e-08	MT-ND1
Recoverec	20	5.0657367816409e-13	0.34658430378130{	0.556	0.358	1.69894680182672e-08	PHF14
Recoverec	20	5.3223361027081e-13	0.26282636210336{	0.596	0.386	1.78500508212624e-08	TMEM165
Recoverec	20	5.47538322963077e-13	0.43156724553835{	0.289	0.137	1.83633402755357e-08	PCAT1
Recoverec	20	5.53679516766691e-13	0.30511523622947{	0.9	0.696	1.85693036333213e-08	PPP3CA
Recoverec	20	5.95833245120872e-13	0.28284277680915{	0.696	0.476	1.99830553748638e-08	MARK3
Recoverec	20	6.04490323476997e-13	0.33565158752787	0.717	0.487	2.02733964687715e-08	PKN2
Recoverec	20	6.1481857149496e-13	0.30711946866339{	0.55	0.346	2.0619785250798e-08	UBE2R2
Recoverec	20	6.19860679608991e-13	0.27922241897590{	0.799	0.577	2.07888874727264e-08	NFAT5
Recoverec	20	6.22238589434363e-13	0.30445340285648{	0.471	0.276	2.08686378124497e-08	YTHDC2
Recoverec	20	6.40952755496761e-13	0.33365818330102{	0.289	0.136	2.14962735138504e-08	TCP11L2
Recoverec	20	6.45392806241625e-13	0.33913176206157{	0.407	0.226	2.16451839357316e-08	AGPAT5
Recoverec	20	6.47702949621965e-13	0.31359531905683{	0.948	0.817	2.17226615244215e-08	ARIH1
Recoverec	20	6.57352644422675e-13	0.37999896141870{	0.915	0.808	2.20462929886477e-08	RAB11A
Recoverec	20	6.82215814836185e-13	0.42534557850390{	0.492	0.297	2.2880153997976e-08	BAZ1B
Recoverec	20	6.82908331409772e-13	0.35363808303049{	0.666	0.448	2.29033796188209e-08	BLNK
Recoverec	20	7.13821077368201e-13	0.37292908595097{	0.924	0.811	2.39401312927747e-08	MEF2C

Recoverec	20	7.66382888807564e-13	0.279307512739072	0.76	0.55	2.57029493248281e-08	ACTR3
Recoverec	20	8.61332591412722e-13	0.292915523436454	0.672	0.452	2.88873724507999e-08	UBE3A
Recoverec	20	8.75302926000442e-13	0.383268980391072	0.723	0.513	2.93559095322028e-08	USP47
Recoverec	20	9.72534732419714e-13	0.282341424231997	0.796	0.592	3.26168698558924e-08	ARPC2
Recoverec	20	9.73892825202701e-13	0.29737450334237	0.866	0.668	3.26624175716482e-08	PAN3
Recoverec	20	9.75181947390863e-13	0.298098646998156	0.398	0.222	3.27056521515948e-08	POC1B
Recoverec	20	9.99081158186492e-13	0.337163092456163	0.422	0.236	3.35071838832586e-08	LBR
Recoverec	20	1.00596880031654e-12	0.356295960201042	0.578	0.367	3.37381816250161e-08	NRIP1
Recoverec	20	1.0281079256134e-12	0.280794726250541	0.796	0.584	3.44806836092221e-08	TUT4
Recoverec	20	1.11389396968105e-12	0.306899113621899	0.805	0.602	3.73577759551631e-08	FBXO11
Recoverec	20	1.1532843739968e-12	0.287050437107687	0.65	0.428	3.86788513351046e-08	HBP1
Recoverec	20	1.159758666989e-12	0.26083997942928	0.505	0.29	3.88959861734771e-08	ZEB2
Recoverec	20	1.25380595066153e-12	0.348752347932248	0.757	0.545	4.20501439732865e-08	ZFAND6
Recoverec	20	1.32582566913371e-12	0.2697445398653	0.404	0.226	4.44655412914063e-08	UBAP2L
Recoverec	20	1.34627247198511e-12	0.428818095353704	0.757	0.558	4.51512861654367e-08	NGLY1
Recoverec	20	1.40381498714236e-12	0.29282898919291	0.368	0.195	4.70811470387805e-08	DYNC1LI1
Recoverec	20	1.40400572402482e-12	0.316569215797442	0.69	0.462	4.70875439723444e-08	ZNF407
Recoverec	20	1.43796838279418e-12	0.299827384355264	0.596	0.388	4.82265836221512e-08	ZFR
Recoverec	20	1.44671571744838e-12	0.560028013122107	0.653	0.453	4.85199517317837e-08	ZNF331
Recoverec	20	1.45228819660935e-12	0.28168490689162	0.495	0.29	4.87068415378845e-08	CYB5R4
Recoverec	20	1.45264381570034e-12	0.368689358864854	0.863	0.701	4.87187682909579e-08	CYTIP
Recoverec	20	1.46654110723447e-12	0.322128290186075	0.447	0.261	4.91848556544295e-08	LINC01184
Recoverec	20	1.48526946583797e-12	0.263983767079879	0.739	0.514	4.98129673452739e-08	CD79A
Recoverec	20	1.49245277786743e-12	0.316210189860732	0.666	0.452	5.00538812641178e-08	NEK7
Recoverec	20	1.49792102673892e-12	0.25107855900066	0.587	0.365	5.02372753947699e-08	RFX7
Recoverec	20	1.50825119505283e-12	0.272104746712219	0.498	0.305	5.05837285796817e-08	PSIP1
Recoverec	20	1.58012129881894e-12	0.282135648217682	0.745	0.52	5.29941081197895e-08	GNB1
Recoverec	20	1.63200246777663e-12	0.27106443389247	0.65	0.438	5.47340987642925e-08	TAPBP
Recoverec	20	1.64387440203645e-12	0.27805651921433	0.711	0.501	5.51322596954984e-08	TRIP12
Recoverec	20	1.65601073875206e-12	0.264296545139943	0.526	0.317	5.55392881562667e-08	USP22
Recoverec	20	1.66548615290896e-12	0.292784766170725	0.964	0.9	5.58570745962606e-08	MBNL1

Recoverec	20	1.73936441070455e-12	0.30898636655621:	0.83	0.629	5.83348036062091e-08	CD44
Recoverec	20	1.9389852575491e-12	0.30550393839046?	0.663	0.458	6.50296875676817e-08	SH3BGRL
Recoverec	20	2.13397271714127e-12	0.36555470039882?	0.426	0.245	7.15691769874841e-08	DHRSX
Recoverec	20	2.15442502589165e-12	0.32286814517403€	0.426	0.249	7.22551065183542e-08	PACSIN2
Recoverec	20	2.33199885048153e-12	0.39167782511630£	0.809	0.627	7.82105774474495e-08	HDAC9
Recoverec	20	2.37815869019074e-12	0.35006333044124€	0.845	0.658	7.9758686151617e-08	ANKRD11
Recoverec	20	2.5015379379579e-12	0.30063996954832	0.83	0.639	8.38965793632319e-08	ARID4B
Recoverec	20	2.60449834571973e-12	0.45630594743985¿	0.459	0.281	8.73496655187481e-08	SLC9A9
Recoverec	20	2.61389599316554e-12	0.27875165205764:	0.663	0.44	8.76648438187859e-08	HERC4
Recoverec	20	2.62917389327776e-12	0.26122592337555:	0.413	0.237	8.81772340327496e-08	RNPS1
Recoverec	20	2.92245770261899e-12	0.34202850130331¿	0.325	0.168	9.80133864304358e-08	THAP12
Recoverec	20	2.95399301627718e-12	0.31024933836389!	1	0.991	9.90710177799039e-08	MT-ND4
Recoverec	20	3.01062213712417e-12	0.28259412875781?	0.711	0.492	1.0097024523487e-07	PHC3
Recoverec	20	3.10094137655477e-12	0.2579044477867	0.596	0.384	1.03999371886894e-07	PNPLA8
Recoverec	20	3.12621048863912e-12	0.25619767974261€	0.435	0.254	1.04846847367979e-07	GIT2
Recoverec	20	3.4076670942006e-12	0.31472334497710?	0.419	0.239	1.142863390053e-07	USP37
Recoverec	20	3.51449340360701e-12	0.33618846324070	0.404	0.225	1.17869079770172e-07	MBNL3
Recoverec	20	3.68066109155807e-12	0.33431522608854£	0.556	0.362	1.23442011688675e-07	MAPKAP1
Recoverec	20	4.02537312794672e-12	0.28925307064573?	0.748	0.541	1.35002963965077e-07	ACTR2
Recoverec	20	4.30807448289131e-12	0.50042744706134	0.86	0.692	1.44484202007209e-07	BCL2
Recoverec	20	4.478669991148e-12	0.25513365523681	0.38	0.209	1.50205634163121e-07	ZNF24
Recoverec	20	4.80640946437599e-12	0.26458965383789:	0.343	0.179	1.61197360616242e-07	LRRC8B
Recoverec	20	4.90544514576839e-12	0.29542049920993?	0.538	0.339	1.6451881929878e-07	KLHL5
Recoverec	20	4.9488891531832e-12	0.36076657550985?	0.45	0.272	1.65975844419458e-07	NAA50
Recoverec	20	5.10508149264955e-12	0.25066665986267€	0.368	0.2	1.71214223100481e-07	HCG18
Recoverec	20	5.2478574399425e-12	0.54151432055619!	0.784	0.652	1.76002642820791e-07	RHOH
Recoverec	20	5.34976382103864e-12	0.35314664606924£	0.818	0.629	1.79420379029994e-07	MAML2
Recoverec	20	5.603190968814e-12	0.38117982124578	0.751	0.564	1.87919818712084e-07	ARL15
Recoverec	20	5.78784823872104e-12	0.26254299097612!	0.48	0.287	1.94112854230226e-07	EXOC6
Recoverec	20	5.96010720391444e-12	0.31871539084846	0.866	0.674	1.99890075404882e-07	CD69
Recoverec	20	7.39746188768299e-12	0.31469029978847	0.69	0.483	2.48096076789112e-07	CEP85L

Recoverec	20	7.57798959710446e-12	0.33603240637849	0.532	0.34	2.54150615107689e-07	STK24
Recoverec	20	7.95004432191159e-12	0.29768896590699	0.532	0.335	2.66628586468271e-07	KIAA2026
Recoverec	20	8.02657802621617e-12	0.32718998989641	0.438	0.257	2.69195373843238e-07	PI4KA
Recoverec	20	8.1351756320041e-12	0.25320680337481	0.623	0.409	2.72837520346154e-07	UBL3
Recoverec	20	8.19837838929231e-12	0.30348349746784	0.772	0.551	2.74957214420085e-07	OSBPL8
Recoverec	20	8.33627372711811e-12	0.28822733025847	0.632	0.428	2.79581948260087e-07	PPP4R3B
Recoverec	20	8.35377683347188e-12	0.30935928038569	0.477	0.297	2.8016896744098e-07	RUBCN
Recoverec	20	8.42073358649619e-12	0.33624113760512	0.708	0.493	2.82414563023909e-07	CCNY
Recoverec	20	8.62836923074328e-12	0.27422972227868	0.413	0.235	2.89378247260668e-07	FAM102A
Recoverec	20	9.26560796694957e-12	0.26582034765912	0.289	0.142	3.10749959995555e-07	BLOC1S2
Recoverec	20	1.00216917679172e-11	0.32612523486845	0.52	0.319	3.36107498512406e-07	MBP
Recoverec	20	1.02301886352926e-11	0.26074464751381	0.775	0.57	3.43100066450442e-07	MKLN1
Recoverec	20	1.05249690398954e-11	0.27680281452777	0.392	0.223	3.52986411660013e-07	SCYL2
Recoverec	20	1.25554030913069e-11	0.25052291084956	0.66	0.428	4.21083108876251e-07	PPP1R16B
Recoverec	20	1.28425754714787e-11	0.29834096172998	0.28	0.133	4.30714296162452e-07	EGR3
Recoverec	20	1.3651275796185e-11	0.28497237122152	0.541	0.351	4.57836487652454e-07	LRCH3
Recoverec	20	1.40827680946893e-11	0.27667515481169	0.714	0.505	4.72307876359689e-07	PTEN
Recoverec	20	1.41998536867094e-11	0.29396130521473	0.483	0.298	4.76234692944861e-07	MICU1
Recoverec	20	1.44116335840423e-11	0.33295425655249	0.915	0.808	4.8333736714161e-07	JMJD1C
Recoverec	20	1.45767063957e-11	0.32552431345465	0.69	0.499	4.88873579098988e-07	HIVEP1
Recoverec	20	1.51723820404037e-11	0.31273746444855	0.578	0.371	5.0885134887106e-07	MYH9
Recoverec	20	1.58645072373987e-11	0.31642767558891	0.541	0.352	5.32063843727878e-07	RAB21
Recoverec	20	1.66348175110896e-11	0.49561586738047	0.517	0.339	5.57898509686923e-07	YBX3
Recoverec	20	1.68011305036806e-11	0.25010024277710	0.377	0.208	5.63476314832439e-07	IFNGR1
Recoverec	20	1.71924336766778e-11	0.31230081392419	0.781	0.581	5.76599840648421e-07	KLF12
Recoverec	20	1.74871879345087e-11	0.28529968364383	0.903	0.741	5.86485308947552e-07	GPBP1
Recoverec	20	1.85157991904194e-11	0.28986334187290	0.69	0.482	6.20982873248287e-07	COPA
Recoverec	20	2.19109377446518e-11	0.2866693198115	0.532	0.356	7.34849030080131e-07	RNF216
Recoverec	20	2.4417966108507e-11	0.52460780065002	0.693	0.515	8.18929747347106e-07	RAD51B
Recoverec	20	2.46724168602685e-11	0.39308022703012	0.316	0.165	8.27463516659684e-07	DDX58
Recoverec	20	2.50820522353756e-11	0.31352546883148	0.702	0.489	8.41201867870026e-07	CYLD

Recoverec	20	2.62898362915472e-11	0.31677575752464(	0.581	0.394	8.81708529545909e-07	HSD17B12
Recoverec	20	2.6338872112793e-11	0.25756513056858(	0.426	0.26	8.83353092918853e-07	TAF1
Recoverec	20	2.87069835747896e-11	0.31679531832512(	0.669	0.466	9.62774815131294e-07	RAP1A
Recoverec	20	2.91855278022728e-11	0.25872333196461(	0.498	0.312	9.78824231432625e-07	KRAS
Recoverec	20	2.97849506868977e-11	0.25568775298873(	0.644	0.437	9.98927676137174e-07	SGMS1
Recoverec	20	3.57066994916583e-11	0.28114822898522	0.684	0.478	1.19753128755123e-06	TNKS
Recoverec	20	3.63552118857791e-11	0.34683237488446(	0.629	0.434	1.21928109622526e-06	ALG13
Recoverec	20	3.67876273734417e-11	0.29529509467166(	0.322	0.172	1.23378344685049e-06	CA5B
Recoverec	20	3.80619199181394e-11	0.25238464459003(	0.444	0.268	1.27652067021456e-06	NOTCH2
Recoverec	20	4.07835473344428e-11	0.34872298949546(	0.41	0.237	1.36779861050254e-06	GALNT7
Recoverec	20	4.42428862650709e-11	0.32267252819859(	0.292	0.149	1.48381791955795e-06	RPGR
Recoverec	20	4.45042462136497e-11	0.27648561536570(	0.745	0.539	1.49258340951338e-06	CSNK1A1
Recoverec	20	4.55183482855453e-11	0.263041174948064	0.824	0.59	1.52659436480062e-06	ANKRD17
Recoverec	20	5.08226718616479e-11	0.36147077975201(	0.471	0.287	1.70449076889595e-06	FBXO33
Recoverec	20	5.31064319447764e-11	0.25212790606982(	0.532	0.341	1.78108351456391e-06	MIS18BP1
Recoverec	20	5.8394381987319e-11	0.38666054231307	0.918	0.791	1.95843078309071e-06	BTG1
Recoverec	20	5.93684088247985e-11	0.50469872446494(	0.444	0.275	1.99109769516609e-06	TPK1
Recoverec	20	6.15094755152476e-11	0.31179150979866(	0.93	0.81	2.06290478983037e-06	TNRC6B
Recoverec	20	6.41737272670189e-11	0.25753364430312(	0.669	0.468	2.15225846508128e-06	HUWE1
Recoverec	20	7.30869154969051e-11	0.28571640741764(	0.453	0.279	2.4511889719352e-06	SNRK
Recoverec	20	8.04990203422827e-11	0.37797231388914(	0.176	0.07	2.69977614423948e-06	IL13RA1
Recoverec	20	8.69399810808583e-11	0.27623998821272(	0.866	0.677	2.91579308548983e-06	RAP1B
Recoverec	20	8.712202137855e-11	0.29312496049822(	0.736	0.533	2.92189835299381e-06	AP3B1
Recoverec	20	8.84482146131704e-11	0.26062008542990(	0.508	0.326	2.96637622169651e-06	ZNF131
Recoverec	20	9.58994780748733e-11	0.27658661006005(	0.541	0.353	3.2162766956751e-06	TMEM243
Recoverec	20	1.11868234549889e-10	0.27883738090797(	0.559	0.364	3.75183685033419e-06	TNPO1
Recoverec	20	1.26200688623802e-10	0.34927962214583(	0.559	0.379	4.23251869506509e-06	FAM49A
Recoverec	20	1.34910684953085e-10	0.26192555332208(	0.274	0.139	4.52463455195657e-06	DGKA
Recoverec	20	1.37543941843389e-10	0.25172867021536(	0.687	0.482	4.61294872154359e-06	TRAPPC9
Recoverec	20	1.42884827656833e-10	0.25740699829951(	0.55	0.362	4.79207134995487e-06	MIER1
Recoverec	20	1.54279023203577e-10	0.28726332544030(	0.942	0.835	5.17420988020157e-06	YWHAZ

Recoverec	20	1.67082235070582e-10	0.257185373598458	0.793	0.583	5.60360399979719e-06	EHMT1
Recoverec	20	1.71690885884557e-10	0.252098570361379	0.553	0.361	5.75816893079626e-06	UBQLN1
Recoverec	20	1.87512264554141e-10	0.345536239043011	0.76	0.555	6.28878632861678e-06	QKI
Recoverec	20	1.87846848560216e-10	0.271458267128587	0.584	0.395	6.30000760701251e-06	THADA
Recoverec	20	2.00727168841788e-10	0.253632253960897	0.35	0.196	6.73198778861588e-06	MTM1
Recoverec	20	2.29395279224655e-10	0.254818409421124	0.711	0.509	7.69345887463649e-06	DYM
Recoverec	20	2.39315091383744e-10	0.347556989221364	0.401	0.239	8.02614953482799e-06	PELI2
Recoverec	20	2.39916694674771e-10	0.267122103246576	0.693	0.499	8.04632610600247e-06	TTC17
Recoverec	20	2.40026123310674e-10	0.314006137182014	0.626	0.433	8.04999612359338e-06	MCTP2
Recoverec	20	2.47021225131962e-10	0.25240372508751	0.611	0.426	8.28459784847575e-06	STX5
Recoverec	20	2.56101997402957e-10	0.260115510553958	0.824	0.622	8.58914878890039e-06	GRB2
Recoverec	20	2.62517852222759e-10	0.293999440469487	0.894	0.723	8.80432372784688e-06	ZFAND3
Recoverec	20	3.03007231228144e-10	0.314407434034783	0.459	0.292	1.01622565209295e-05	EEA1
Recoverec	20	3.36383271063468e-10	0.263940020108745	0.793	0.579	1.12816221449266e-05	UBR5
Recoverec	20	3.42641436132214e-10	0.267718832784354	0.836	0.662	1.14915084850022e-05	BBX
Recoverec	20	3.45100230507226e-10	0.288786452317358	0.921	0.784	1.15739715307514e-05	ELF1
Recoverec	20	3.48136845457177e-10	0.337303999749757	0.684	0.495	1.16758135229428e-05	FOXO1
Recoverec	20	3.8519061253748e-10	0.347361487823186	0.468	0.302	1.2918522763282e-05	PAK1
Recoverec	20	4.61302795241486e-10	0.26768038751616	0.231	0.109	1.54711731468089e-05	AF241726.2
Recoverec	20	4.70802571278131e-10	0.278359363262382	0.407	0.249	1.5789776635526e-05	N4BP2L1
Recoverec	20	5.46846266516706e-10	0.300934918012948	0.441	0.277	1.83401300864373e-05	AVL9
Recoverec	20	5.55259160637756e-10	0.283769020161542	0.568	0.39	1.86222817294691e-05	SDCCAG8
Recoverec	20	5.77800649016984e-10	0.340271782016411	0.264	0.135	1.93782781667316e-05	MYC
Recoverec	20	6.29643376411279e-10	0.348231347005523	0.359	0.208	2.11169795580815e-05	PHLPP1
Recoverec	20	7.0753433938938e-10	0.400916826184747	0.526	0.357	2.3729286674441e-05	SLC12A6
Recoverec	20	7.07749305696663e-10	0.254928998705192	0.353	0.204	2.37364962144547e-05	NOLC1
Recoverec	20	7.20430642022179e-10	0.367104213626396	0.714	0.534	2.41618028721398e-05	COMMD10
Recoverec	20	7.27712456431634e-10	0.310940731732231	0.246	0.12	2.44060203638041e-05	AL662796.1
Recoverec	20	7.75514764510263e-10	0.32449296546318	0.35	0.198	2.60092141721452e-05	ARHGAP32
Recoverec	20	8.69450494488298e-10	0.300639338496345	0.851	0.683	2.91596306841485e-05	STK17B
Recoverec	20	9.22262243021237e-10	0.456342531623508	0.687	0.516	3.09308311064463e-05	IRF1

Recoverec	20	9.43092386865486e-10	0.25889907108209‌2	0.374	0.221	3.16294324706947e-05	DNAJA2
Recoverec	20	1.08034385418701e-09	0.39172818059525‌9	0.568	0.401	3.6232572181724e-05	TMEM156
Recoverec	20	1.15134511649624e-09	0.29800601718580‌7	0.502	0.333	3.86138125170509e-05	FILIP1L
Recoverec	20	1.21804908628374e-09	0.28332145480709‌7	0.799	0.615	4.08509302557839e-05	PACS1
Recoverec	20	1.29004327060165e-09	0.25883760982358‌8	0.544	0.371	4.32654712094382e-05	ATXN10
Recoverec	20	1.3034963189936e-09	0.33218291774732	0.386	0.236	4.37166595464075e-05	RAB22A
Recoverec	20	1.3924361134409e-09	0.25748434589763	0.468	0.302	4.66995223725808e-05	PHF20L1
Recoverec	20	1.45928388304142e-09	0.41578105651176‌3	0.234	0.114	4.89414628694432e-05	CENPP
Recoverec	20	1.51501618907651e-09	0.28793658993506‌5	0.812	0.637	5.08106129492481e-05	STRBP
Recoverec	20	1.52055924893489e-09	0.34080449333894‌9	0.453	0.287	5.09965160907784e-05	FAM126A
Recoverec	20	1.52991720893292e-09	0.28607131819827‌2	0.757	0.563	5.13103633531921e-05	LINC01004
Recoverec	20	1.61468955179252e-09	0.30116690803415‌7	0.653	0.461	5.41534581880175e-05	ATP8A1
Recoverec	20	1.71946341820131e-09	0.27359956369372‌2	0.812	0.624	5.76673641196355e-05	ETV6
Recoverec	20	1.75625419434869e-09	0.28123118553914‌1	0.581	0.396	5.89012531700664e-05	COL4A3BP
Recoverec	20	1.75758835196563e-09	0.28767172284811‌5	0.453	0.291	5.89459981482232e-05	CDK8
Recoverec	20	1.92785918033702e-09	0.26163043862114‌7	0.347	0.196	6.4656541190143e-05	UBE2D1
Recoverec	20	2.06819551883647e-09	0.34433965440002‌2	0.751	0.576	6.93631413107376e-05	MBD5
Recoverec	20	2.12048569390066e-09	0.27971720463879‌8	0.796	0.597	7.11168492020404e-05	CHD9
Recoverec	20	2.34155410981128e-09	0.29842687976066‌5	0.757	0.575	7.85310417348507e-05	TCF4
Recoverec	20	2.80084269696797e-09	0.26635073410469	0.809	0.637	9.39346623709118e-05	ATF7IP
Recoverec	20	2.93250700399219e-09	0.29052591556358‌6	0.319	0.18	9.835041989989e-05	RNF130
Recoverec	20	3.64636007255919e-09	0.27229341785854‌8	0.55	0.378	0.00012229162411349	CCDC50
Recoverec	20	3.95540579096217e-09	0.25632942657820‌4	0.571	0.389	0.000132656399417289	PRPF40A
Recoverec	20	4.23617605329295e-09	0.26779815156669‌1	0.438	0.281	0.000142072872475339	VDAC1
Recoverec	20	4.28724230177694e-09	0.26798115855466‌3	0.517	0.35	0.000143785532316995	KIF2A
Recoverec	20	4.44528494242603e-09	0.32033936260266‌9	0.356	0.21	0.000149085966399084	NAF1
Recoverec	20	4.69237070469585e-09	0.26569257918234‌3	0.544	0.371	0.00015737272869409	RAP1GDS1
Recoverec	20	4.86544474757055e-09	0.34921536054863‌9	0.596	0.418	0.000163177285944021	SCLT1
Recoverec	20	5.00496208969647e-09	0.34493212069637	0.684	0.508	0.00016785641856424	ZMYM2
Recoverec	20	5.48561675947351e-09	0.25154657981173‌5	0.41	0.258	0.000183976614879223	USP11
Recoverec	20	5.8219904216358e-09	0.32695738815895	0.371	0.228	0.000195257914760822	INTS6L

Recoverec	20	6.26133180918545e-09	0.27486947177244(	0.502	0.346	0.000209992546216462	ATG7
Recoverec	20	7.7310895169538e-09	0.27099890779451(	0.839	0.655	0.000259285280219597	UBE2H
Recoverec	20	7.83338489723257e-09	0.33546055258011(	0.398	0.246	0.000262716062683386	ZNF106
Recoverec	20	8.02282307054005e-09	0.28990288895076(	0.407	0.256	0.000269069440139772	PTPRJ
Recoverec	20	8.16331011051671e-09	0.27436665129313(	0.544	0.369	0.000273781094486509	TAF3
Recoverec	20	8.29351077161764e-09	0.32292374375681(	0.103	0.034	0.000278147764258512	SNTG2
Recoverec	20	8.33559271410552e-09	0.26176576011218(	0.465	0.313	0.000279559108445671	CCSER2
Recoverec	20	8.9711778255894e-09	0.52842882581638(	0.584	0.405	0.000300875361914617	ATP1B3
Recoverec	20	9.40685223772334e-09	0.26570909501401(	0.997	0.953	0.000315487010348765	MT-ND5
Recoverec	20	1.16163797602251e-08	0.25947440736927(	0.35	0.213	0.000389590144398429	ATG3
Recoverec	20	1.17717213241986e-08	0.51215732043341	0.708	0.54	0.000394799989770974	LARGE1
Recoverec	20	1.23900861441298e-08	0.35224995975958(	0.322	0.19	0.000415538709101824	ABHD17B
Recoverec	20	1.26922294025138e-08	0.26127059952873(	0.553	0.382	0.000425671989701508	ADAM10
Recoverec	20	1.48673156137421e-08	0.29594699641505(	0.559	0.39	0.000498620031053681	TIPARP
Recoverec	20	1.69482271546421e-08	0.30732596061608	0.404	0.26	0.000568409642312387	ATP13A3
Recoverec	20	1.72798298289553e-08	0.25961192013764	0.365	0.227	0.000579530932803503	IPMK
Recoverec	20	2.0883917061656e-08	0.28459331823352	0.316	0.186	0.000700404810413819	IGF1R
Recoverec	20	2.09012224931768e-08	0.31793971722522(	0.565	0.408	0.000700985199976163	PDSS2
Recoverec	20	2.13868038451362e-08	0.42465904676592(	0.581	0.413	0.000717270627358179	NR3C2
Recoverec	20	2.29981839286536e-08	0.40786872625529(	0.802	0.647	0.000771313092599184	UBE2E2
Recoverec	20	2.72109792333794e-08	0.32857869606541(	0.201	0.1	0.000912601821529079	PLK2
Recoverec	20	2.75802770594551e-08	0.28508088274336(	0.264	0.143	0.000924987332020005	DSP
Recoverec	20	2.95954287646922e-08	0.25993589066223(	0.486	0.322	0.000992571489910246	WDPCP
Recoverec	20	3.27228555521989e-08	0.27355747490294(	0.672	0.509	0.00109745912950965	DPYD
Recoverec	20	3.44814575179762e-08	0.29356435679920(	0.201	0.097	0.00115643912223789	ADAMTS6
Recoverec	20	3.59590311712899e-08	0.26667954268737(	0.319	0.183	0.00120599398742272	TLE1
Recoverec	20	5.08643415195423e-08	0.34960797101597(	0.289	0.165	0.00170588828588241	MAML3
Recoverec	20	7.63989741348679e-08	0.27066809293785(	0.486	0.329	0.0025622687945352	TNS3
Recoverec	20	7.77152499818659e-08	0.25518437455149(	0.565	0.399	0.00260641405389182	ARHGAP26
Recoverec	20	8.03475485935915e-08	0.25008294979933(	0.134	0.055	0.00269469608473187	CCL25
Recoverec	20	9.98428074062987e-08	0.28298040813951(	0.103	0.037	0.00334852807479245	RGS7

Recoverec	20	1.1068873178847e-07	0.286178916410198	0.59	0.436	0.00371227868672169	CMSS1
Recoverec	20	1.23507530370327e-07	0.259195806913738	0.246	0.133	0.00414219555356004	ANKAR
Recoverec	20	1.30123118470673e-07	0.360575260549285	0.766	0.601	0.00436406914726945	TTC3
Recoverec	20	1.39483229371856e-07	0.488902562763421	0.17	0.082	0.0046779885466733	GPM6A
Recoverec	20	1.50661517257056e-07	0.328661349705395	0.45	0.307	0.00505288596576713	PRKN
Recoverec	20	2.1568860736337e-07	0.262601439980495	0.359	0.23	0.00723376451375271	WDFY2
Recoverec	20	2.35388072899582e-07	0.325371258311035	0.407	0.266	0.00789444518890617	PCED1B
Recoverec	20	2.5026980260907e-07	0.256853399781759	0.432	0.297	0.00839354863990301	MYO9A
Recoverec	20	2.54782413248303e-07	0.276557893783295	0.149	0.067	0.00854489257552157	ACSM3
Recoverec	20	2.77699689104096e-07	0.267390166213875	0.298	0.179	0.00931349217317317	SHLD1
Recoverec	20	6.24046552963924e-07	0.338415323756719	0.502	0.363	0.0209292732933041	SKAP1
Recoverec	20	1.13893465951239e-06	0.255367339810462	0.271	0.16	0.0381975906107265	IL7
Recoverec	20	1.24486194052877e-06	0.26673184712196	0.547	0.408	0.0417501797614539	ACYP2
Recoverec	20	1.42503498954506e-06	0.276589408148879	0.283	0.172	0.0477928234793623	CARS2
Recoverec	26	2.73821801080917e-43	0.675307847846762	0.339	0.039	9.1834355646518e-39	C1QB
Recoverec	26	4.14852844959197e-15	0.845173682491506	0.771	0.491	1.39133347142416e-10	CD63
Recoverec	26	4.15573851131184e-14	0.360384938865831	0.211	0.045	1.39375158192376e-09	C1QA
Recoverec	26	6.17193152748998e-14	1.0553263291056	0.44	0.172	2.06994239568959e-09	AGR2
Recoverec	26	8.47550974689911e-14	0.46548551949853	0.202	0.043	2.84251645891502e-09	MT1B
Recoverec	26	4.44983338414078e-13	0.596637112737288	0.193	0.042	1.49238512037314e-08	C11orf96
Recoverec	26	5.86715404504316e-13	0.905915020571618	0.963	0.921	1.96772612362657e-08	HSP90AA1
Recoverec	26	8.25211850440709e-13	0.472149650227782	0.468	0.195	2.76759550400805e-08	SPAG4
Recoverec	26	8.89116017944927e-13	1.00820004769789	0.844	0.534	2.98191730098369e-08	MT1G
Recoverec	26	1.22433116245839e-12	0.275240698266584	0.138	0.023	4.10616185265296e-08	C1QC
Recoverec	26	2.34596271115476e-12	1.10002273691692	0.826	0.593	7.86788974067083e-08	HSPA1A
Recoverec	26	5.90147933910122e-12	0.280580865463685	0.147	0.027	1.97923814074777e-07	SPINK4
Recoverec	26	7.99050953039183e-12	0.644356480931579	0.697	0.396	2.67985708630281e-07	LGALS1
Recoverec	26	6.71161631777874e-10	1.17005058851847	0.899	0.767	2.25094188065663e-05	IGHA1
Recoverec	26	1.18380353403562e-09	0.839528162043359	0.615	0.359	3.97024029244866e-05	HSPB1
Recoverec	26	3.28948370920801e-09	0.669026964237897	0.67	0.439	0.000110322704639418	FKBP2
Recoverec	26	4.84780702799065e-09	0.86451773822586	0.89	0.741	0.00016258575210475	JCHAIN

Recoverec	26	5.88189799959492e-09	0.50557430465432	0.835	0.594	0.000197267095110414	HSPA1B
Recoverec	26	7.28767702755172e-09	0.896237394845114	0.706	0.461	0.00024441411215003	MT2A
Recoverec	26	1.30157153455258e-08	0.639766791712465	0.734	0.568	0.000436521061258245	SEC61B
Recoverec	26	1.33328510726935e-08	1.05225769551508	0.725	0.625	0.000447157159275993	SSR4
Recoverec	26	2.22419003434695e-08	0.404161347641121	0.991	0.909	0.00074594885371928	FTL
Recoverec	26	4.30462889644457e-08	0.755432084046388	0.835	0.668	0.00144368643928958	DNAJB1
Recoverec	26	1.60755058041694e-07	0.405487601121256	0.514	0.294	0.00539140313660234	PHPT1
Recoverec	26	1.60943720819657e-07	0.651207210375003	0.431	0.229	0.00539773050884967	MT1X
Recoverec	26	5.24444748928215e-07	0.553995961744577	0.578	0.382	0.0175888279895545	DERL3
Recoverec	26	6.23348186000012e-07	0.678924273594135	0.67	0.464	0.0209058514620684	MZB1
Recoverec	26	6.97820675901224e-07	1.38457318973962	0.972	0.932	0.0234035098283752	IGKC
Recoverec	26	8.7231233907915e-07	0.567365231753772	0.569	0.376	0.0292556112280365	FKBP11
Recoverec	26	1.20147906225553e-06	0.267383390791092	0.514	0.286	0.0402952047899261	TRIB1
Recoverec	26	1.35116904610361e-06	0.45450159053917	0.606	0.408	0.0453155074682229	RABAC1
Recoverec	33	1.08280651467066e-58	1.32473360887019	0.772	0.291	3.63151648890244e-54	BMP6
Recoverec	33	1.44981843141494e-55	1.15494548636651	0.912	0.496	4.86240105527941e-51	AC008014.1
Recoverec	33	2.760034819503e-54	0.87732422270544	0.647	0.215	9.25660477764917e-50	TMC3-AS1
Recoverec	33	2.46185457948549e-53	0.883201045327644	0.507	0.141	8.25656788867844e-49	AC060809.1
Recoverec	33	3.0106401376475e-53	1.16140915656406	0.952	0.67	1.00970848936422e-48	FNDC3A
Recoverec	33	1.0946280132923e-51	1.55609513261679	0.64	0.24	3.67116343097972e-47	CADPS2
Recoverec	33	4.5707404049237e-51	0.993518844597856	0.926	0.511	1.53293491700331e-46	FNDC3B
Recoverec	33	7.33331209409214e-51	1.19257319788935	0.743	0.302	2.45944621011662e-46	LINC01934
Recoverec	33	1.72230025156276e-50	0.804111600978682	0.415	0.094	5.77625058369117e-46	AC004594.1
Recoverec	33	1.4256595385057e-48	1.2666024974422	0.926	0.537	4.78137696024042e-44	GLCCI1
Recoverec	33	2.98460944989751e-47	0.898683498462876	0.879	0.44	1.00097831730663e-42	SPATS2
Recoverec	33	3.16801397223179e-47	0.944207133967235	0.86	0.446	1.0624885260071e-42	CROCC
Recoverec	33	9.00150058774642e-46	1.00495866562728	0.699	0.286	3.0189232671184e-41	FAM129A
Recoverec	33	2.82767847282623e-45	0.796296350631486	0.824	0.371	9.48346806216459e-41	PDK1
Recoverec	33	8.4982210794059e-45	1.0744056217852	0.743	0.319	2.85013338561115e-40	TTC28
Recoverec	33	1.31521410604879e-44	0.93494044350646	0.779	0.36	4.41096506886642e-40	SEC24D
Recoverec	33	7.39513628943657e-44	0.787270312465702	0.827	0.385	2.48018080875124e-39	TXNDC11

Recoverec	33	7.93792482382609e-44	0.71165637387195?	0.665	0.257	2.6622212274148e-39	ENAM
Recoverec	33	3.38401883575057e-43	0.77249207113499?	0.812	0.388	1.13493223713403e-38	SAMD12
Recoverec	33	4.19752702865339e-43	1.11171915169789	0.934	0.724	1.40776661486977e-38	JCHAIN
Recoverec	33	5.38290798060114e-43	0.92565878498703?	0.938	0.692	1.80531967853401e-38	HSP90B1
Recoverec	33	8.79031141960597e-43	0.99222369566882?	0.868	0.492	2.94809464390745e-38	DNAJC1
Recoverec	33	3.62008403412029e-42	1.02630716175017	0.772	0.366	1.21410378336326e-37	SLAMF7
Recoverec	33	4.39729270379852e-42	0.81402400555605?	0.838	0.411	1.47476402699995e-37	CREB3L2
Recoverec	33	1.05869562517564e-41	0.88460882603745?	0.794	0.394	3.55065338771406e-37	SEC11C
Recoverec	33	2.43390060158806e-41	0.851115796231874	0.724	0.321	8.16281583760602e-37	TNFRSF17
Recoverec	33	1.08009264155904e-40	0.87193202122404?	0.967	0.722	3.62241470126072e-36	FBXW7
Recoverec	33	2.14702886286679e-40	1.02874452682909	0.868	0.456	7.20070540028263e-36	EAF2
Recoverec	33	1.01598909722208e-39	0.83529969882864?	0.93	0.572	3.4074242342634e-35	ATXN1
Recoverec	33	1.36180990001873e-39	1.20377107619199	0.919	0.692	4.56723804268283e-35	IER2
Recoverec	33	4.02594272457718e-39	0.70299653184573?	0.618	0.236	1.3502206709687e-34	ESR1
Recoverec	33	6.54356674774243e-38	0.74842496181867?	0.739	0.345	2.19458141585786e-33	EDEM3
Recoverec	33	1.19928701556276e-37	0.59800660787252?	0.445	0.138	4.02216879279437e-33	ALDH1L2
Recoverec	33	1.22555006454537e-37	0.67624437823067?	0.838	0.413	4.11024980647226e-33	RRBP1
Recoverec	33	1.68245693046594e-37	0.60257776500757?	0.614	0.254	5.64262405339665e-33	MANEA
Recoverec	33	8.59322690403872e-37	0.64598302157896?	0.493	0.168	2.8819964390765e-32	PARM1
Recoverec	33	2.14122521382955e-36	0.57037220708234?	0.393	0.111	7.18124112214156e-32	LINC02100
Recoverec	33	3.96615077865246e-36	0.72482225640802	0.846	0.426	1.33016764814446e-31	MZB1
Recoverec	33	4.98325717270609e-36	0.69852348447348?	0.735	0.344	1.67128479058217e-31	MT-ND6
Recoverec	33	5.88850021415879e-36	0.81544775689743?	0.636	0.263	1.97488520182457e-31	AC078883.1
Recoverec	33	8.84737932769118e-36	0.99603558407584?	0.787	0.413	2.96723407892107e-31	IFNG-AS1
Recoverec	33	9.12940331360415e-36	0.68434329005292?	0.456	0.149	3.06181928331656e-31	CFAP54
Recoverec	33	3.81231955618157e-35	1.02112770539083	0.875	0.572	1.27857573275217e-30	AHNAK
Recoverec	33	6.98036899504841e-35	0.77099868021646?	0.761	0.392	2.34107615355934e-30	SEL1L
Recoverec	33	8.09458971240655e-35	0.69931704856000?	0.82	0.455	2.71476349774691e-30	HDLBP
Recoverec	33	1.04936346702459e-34	0.86838447744554?	0.93	0.646	3.51935519570706e-30	ANKRD28
Recoverec	33	1.22674010871721e-34	0.60745747923791?	0.426	0.136	4.11424097661579e-30	SMOC1
Recoverec	33	1.43378689648328e-34	0.65716044287864?	0.404	0.125	4.80863449342561e-30	TTLL7

Recoverec	33	2.09017402689422e-34	0.620911607128992	0.518	0.189	7.01002565139784e-30	MCC
Recoverec	33	6.04805758680247e-34	0.555122118481896	0.449	0.145	2.02839755346181e-29	COL24A1
Recoverec	33	7.58423474651842e-34	0.752746163496861	0.849	0.464	2.54360064928735e-29	GAB1
Recoverec	33	9.20109823255741e-34	0.829848324930867	0.831	0.452	3.0858643252351e-29	RUNX2
Recoverec	33	9.52142718429335e-34	0.834217040844375	0.816	0.505	3.1932962490683e-29	HSPA5
Recoverec	33	9.89643374122812e-34	0.707644571398769	0.79	0.406	3.31906594813309e-29	ZBTB38
Recoverec	33	6.60497654727578e-33	0.706191577131244	0.776	0.446	2.21517703442535e-28	SSR3
Recoverec	33	1.50159179340768e-32	0.751040740289134	0.699	0.33	5.03603855673068e-28	SOX5
Recoverec	33	2.03088442553805e-32	0.684890320304018	0.658	0.294	6.81118018636951e-28	SLC15A2
Recoverec	33	2.85500329335578e-32	0.884725720519145	0.86	0.599	9.57511004525661e-28	SSR4
Recoverec	33	3.6149296377514e-32	0.687156047687431	0.761	0.383	1.21237510190906e-27	FCRL5
Recoverec	33	5.23527019603194e-32	0.593852463373604	0.757	0.377	1.75580491834519e-27	SELENOS
Recoverec	33	6.49015521498283e-32	0.691136523699799	0.879	0.61	2.17666825600094e-27	SPCS2
Recoverec	33	6.82421833091366e-32	0.941374521342098	0.849	0.599	2.28870634382182e-27	VIM
Recoverec	33	1.84269070053359e-31	0.701433185599854	0.724	0.369	6.18001607144955e-27	CDK6
Recoverec	33	3.39554858424299e-31	0.524301741584553	0.757	0.375	1.13879908418341e-26	KANK1
Recoverec	33	1.74214535801607e-30	0.763998566776857	0.757	0.389	5.84280710171428e-26	UAP1
Recoverec	33	1.81416212146165e-30	0.767462261316101	0.614	0.271	6.08433692295808e-26	LINC02362
Recoverec	33	3.26473066804716e-30	0.6520841769318	0.522	0.211	1.09492537144966e-25	UGGT2
Recoverec	33	3.48131030451623e-30	0.543798063146378	0.504	0.197	1.16756184992865e-25	EPB41L4A
Recoverec	33	1.30483794461123e-29	0.548197252319227	0.581	0.254	4.37616549863716e-25	NUCB2
Recoverec	33	1.46995651962811e-29	0.665226477262134	0.482	0.188	4.92994017552876e-25	TIAM1
Recoverec	33	4.19454937145881e-29	0.894291319038155	0.871	0.53	1.40676796819986e-24	OSBPL3
Recoverec	33	4.51991317081739e-29	0.759642703616939	0.967	0.878	1.51588847922874e-24	JUN
Recoverec	33	5.62334509525612e-29	0.416436268291531	0.515	0.2	1.885957478047e-24	NUGGC
Recoverec	33	6.08928696669314e-29	0.674431290672302	0.827	0.53	2.04222506288955e-24	PPIB
Recoverec	33	7.83115190133772e-29	0.662614446472051	0.603	0.263	2.62641172467065e-24	TMTC2
Recoverec	33	1.22192660257387e-28	0.616160423413347	0.835	0.493	4.09809743971225e-24	UBE2J1
Recoverec	33	1.26754678663469e-28	0.598538045718082	0.702	0.352	4.25109841301544e-24	LARP1B
Recoverec	33	1.40524782852153e-28	0.794008841292041	0.82	0.491	4.71292016729551e-24	GMDS-DT
Recoverec	33	1.53292016625892e-28	0.691318224627184	0.824	0.486	5.14110765359917e-24	PIP5K1B

Recoverec	33	2.06921753740504e-28	0.78612244768343	0.702	0.377	6.93974177694902e-24	AC007952.4
Recoverec	33	3.86528461790228e-28	0.43316413732730	0.669	0.302	1.29633915515207e-23	INPP4A
Recoverec	33	4.32342171702659e-28	0.53784052480791	0.82	0.471	1.44998917545638e-23	DNAJC3
Recoverec	33	4.5755238859225e-28	0.56193979475973	0.717	0.342	1.53453920086069e-23	FKBP11
Recoverec	33	5.05502205654159e-28	0.69737351297707	0.746	0.411	1.69535329732292e-23	FKBP2
Recoverec	33	8.07987640010564e-28	0.37480458656445	0.301	0.085	2.70982894706743e-23	PSAT1
Recoverec	33	8.97923055993949e-28	1.07432681839025	0.919	0.754	3.0114543451925e-23	IGHA1
Recoverec	33	1.01042939464111e-27	0.47424345126226	0.511	0.213	3.38877810374736e-23	MEI1
Recoverec	33	1.01835947576046e-27	0.47307561322684	0.724	0.349	3.41537400980542e-23	DERL3
Recoverec	33	1.39933929603063e-27	0.55183506144571	0.824	0.471	4.69310413102752e-23	SPCS3
Recoverec	33	1.57391717802055e-27	0.59031091753685	0.849	0.54	5.27860343164532e-23	SEC61B
Recoverec	33	2.77250755377203e-27	0.57304695917434	0.82	0.441	9.29843583384062e-23	XBP1
Recoverec	33	2.91875109882744e-27	0.64603012335368	0.71	0.396	9.78890743524747e-23	PABPC4
Recoverec	33	4.823660394104e-27	0.51432447882621	0.691	0.344	1.6177592229746e-22	ERLEC1
Recoverec	33	4.88412140409821e-27	0.70160602872364	0.89	0.619	1.63803663650646e-22	ERC1
Recoverec	33	5.21285169188554e-27	0.74929488719721	0.375	0.128	1.74828620042457e-22	PLCL1
Recoverec	33	5.8572390425533e-27	0.69706833353532	0.309	0.09	1.96440083009153e-22	ST3GAL6
Recoverec	33	5.8943445743746e-27	0.61659693640610	0.912	0.599	1.97684528335375e-22	SND1
Recoverec	33	9.68549511844407e-27	0.62555505202869	0.864	0.519	3.24832135282377e-22	CFLAR
Recoverec	33	1.37930804809622e-26	0.73164826477597	0.599	0.285	4.6259233317051e-22	HMCES
Recoverec	33	2.08290197165918e-26	0.51277324378937	0.765	0.41	6.98563663255057e-22	CPEB4
Recoverec	33	2.14487909857596e-26	0.56452286474568	0.46	0.179	7.19349552080406e-22	CRYBG3
Recoverec	33	2.55397376421365e-26	0.66845981338190	0.835	0.555	8.56551721041973e-22	SELENOK
Recoverec	33	3.29623526562383e-26	0.73458137281749	0.721	0.392	1.10549138338492e-21	RASGRP3
Recoverec	33	3.7372644338289e-26	0.72241200795819	0.772	0.454	1.25340374581754e-21	AC022217.3
Recoverec	33	7.5106468828924e-26	0.44459519147597	0.46	0.184	2.51892075158445e-21	AC139887.2
Recoverec	33	1.57684037475097e-25	0.50754488339829	0.713	0.371	5.28840724883979e-21	LGALS1
Recoverec	33	1.9756639401144e-25	0.67730451532826	0.54	0.235	6.62598172235566e-21	SLC7A5
Recoverec	33	1.97702033525972e-25	0.38563447304256	0.581	0.251	6.63053080039405e-21	PRDX4
Recoverec	33	2.31551119038664e-25	0.57754175549557	0.783	0.448	7.76576143031871e-21	TEX14
Recoverec	33	2.43823006487621e-25	0.42861327992034	0.489	0.2	8.17733599158183e-21	SLC1A4

Recoverec	33	3.19753409285705e-25	0.51162998950086	0.728	0.402	1.0723889840624e-20	LMAN1
Recoverec	33	3.63140977063712e-25	0.69770910226381	0.901	0.63	1.21790220887628e-20	MCL1
Recoverec	33	3.84732082632536e-25	0.70252404749984	0.82	0.495	1.290314458733e-20	MAN1A1
Recoverec	33	3.95689825809165e-25	0.53442726243499	0.801	0.474	1.32706453779878e-20	TRAM1
Recoverec	33	4.32413927670293e-25	0.45318987444260	0.691	0.358	1.45022983062063e-20	HM13
Recoverec	33	6.91700497099132e-25	0.47220180877530	0.713	0.386	2.31982512717107e-20	DNAJB9
Recoverec	33	8.20654818881704e-25	0.67826014657801	0.835	0.51	2.75231213156546e-20	MAN1A2
Recoverec	33	1.29487013204634e-24	0.43420166430739	0.239	0.061	4.34273544885702e-20	AC006148.1
Recoverec	33	1.30943940383355e-24	0.65985327447428	0.89	0.581	4.39159787257695e-20	FOXO3
Recoverec	33	1.42184099639647e-24	0.49434123042718	0.724	0.387	4.76857033371447e-20	USO1
Recoverec	33	1.47617315658331e-24	0.70884542702152	0.879	0.574	4.95078953254912e-20	WWOX
Recoverec	33	1.69637787879638e-24	0.62074246713175	0.868	0.545	5.6893121299073e-20	MBNL2
Recoverec	33	2.00888118132871e-24	0.55258505581192	0.691	0.372	6.73738570594024e-20	SIL1
Recoverec	33	2.20228496795268e-24	0.6465261183582	0.629	0.319	7.38602332551968e-20	MLLT3
Recoverec	33	2.51871670931581e-24	0.42568612599330	0.283	0.084	8.44727209970336e-20	TPTE2
Recoverec	33	2.95226452391123e-24	0.54833297348900	0.754	0.43	9.90130476029348e-20	VWA8
Recoverec	33	3.2434124126053e-24	0.42819174691723	0.544	0.243	1.08777565493957e-19	WNT5B
Recoverec	33	4.4014623773634e-24	0.42253894848923	0.621	0.306	1.47616245212014e-19	MANF
Recoverec	33	5.21121915660302e-24	0.54803541484721	0.449	0.186	1.74773868074152e-19	FAAH2
Recoverec	33	5.62520144429067e-24	0.52313831716971	0.555	0.26	1.8865800603862e-19	SLC7A1
Recoverec	33	7.35029295243374e-24	0.57852930967613	0.412	0.16	2.46514125038723e-19	ITGA8
Recoverec	33	7.59312109629694e-24	0.61287713899445	0.886	0.569	2.54658095327607e-19	HSPA1B
Recoverec	33	9.37759285610519e-24	0.37378732131697	0.559	0.251	3.14505709208056e-19	LINC02384
Recoverec	33	9.55192068918717e-24	0.48161723214588	0.5	0.217	3.20352316073959e-19	TIMP1
Recoverec	33	1.56381575388656e-23	0.51687656133639	0.684	0.368	5.24472527538474e-19	CCPG1
Recoverec	33	1.75699747816088e-23	1.02707995908271	0.618	0.339	5.89261814225597e-19	HSPB1
Recoverec	33	4.55256847534926e-23	0.54335673262717	0.618	0.315	1.52684041526263e-18	ERN1
Recoverec	33	4.9560746355056e-23	0.65102852722495	0.577	0.274	1.66216831125587e-18	PRDM1
Recoverec	33	5.45408890343737e-23	0.51349940090246	0.75	0.437	1.82919233643482e-18	SRP54
Recoverec	33	5.54093480921708e-23	0.33424715286617	0.368	0.135	1.85831871631523e-18	FAM227B
Recoverec	33	8.84660528127072e-23	0.50879875283417	0.504	0.233	2.96697447923257e-18	TMEM39A

Recoverec	33	9.32767839171761e-23	0.93406882725442	0.71	0.428	3.12831677901425e-18	RASSF3
Recoverec	33	1.06490095014513e-22	0.42551531947194	0.46	0.192	3.57146480659674e-18	ZNF215
Recoverec	33	1.48949229292147e-22	0.44099088328583	0.316	0.107	4.99545925200001e-18	IGF1
Recoverec	33	2.11832160793399e-22	0.72330973062360	0.39	0.152	7.104427008689e-18	UBA6-AS1
Recoverec	33	3.60890155536925e-22	0.32458149307730	0.592	0.291	1.21035340363974e-17	CDK2AP2
Recoverec	33	3.84027319639916e-22	0.67877649046244	0.897	0.721	1.28795082460835e-17	BTG2
Recoverec	33	4.42764691597919e-22	0.58253800928033	0.868	0.604	1.4849442226811e-17	GOLGB1
Recoverec	33	5.6199144138359e-22	0.68433982329176	0.798	0.523	1.88480689611228e-17	FHIT
Recoverec	33	6.88455115244716e-22	0.81019645547785	0.871	0.64	2.30894076550773e-17	NFKBIA
Recoverec	33	7.54718927775091e-22	0.82527581295302	0.592	0.302	2.5311763399721e-17	CYTOR
Recoverec	33	8.64477763235557e-22	0.57562691475486	0.757	0.487	2.89928552233941e-17	SPCS1
Recoverec	33	1.02582642626871e-21	0.39464831377617	0.607	0.317	3.44041666841999e-17	EPRS
Recoverec	33	1.04320760743234e-21	0.50059058027238	0.684	0.383	3.49870967380657e-17	RABAC1
Recoverec	33	1.18665089108551e-21	0.45492613677055	0.618	0.313	3.97978975852257e-17	GALK2
Recoverec	33	1.84371766916583e-21	0.37555078960066	0.438	0.182	6.18346031884837e-17	KCNN3
Recoverec	33	2.07028297906767e-21	0.63909084521407	0.265	0.081	6.94331505519716e-17	LINC00309
Recoverec	33	2.47997547168435e-21	0.52924985318273	0.721	0.397	8.31734173693496e-17	SEC63
Recoverec	33	2.56244079629746e-21	0.74611023829016	0.338	0.125	8.59391394262244e-17	SYT1
Recoverec	33	2.83611556048321e-21	0.29915976535269	0.357	0.135	9.51176436674859e-17	TNFRSF18
Recoverec	33	3.87669382136033e-21	0.73596882660228	0.357	0.137	1.30016557380783e-16	DCC
Recoverec	33	3.88641613234756e-21	0.64392378305146	0.89	0.617	1.30342624246672e-16	TENT5C
Recoverec	33	4.8089855349384e-21	0.38719150151964	0.64	0.333	1.61283756870764e-16	ARFGAP3
Recoverec	33	7.91069486553834e-21	0.44997852727199	0.761	0.436	2.65308884400425e-16	SFMBT2
Recoverec	33	8.18303219597248e-21	0.37733098581385	0.607	0.306	2.74442533788525e-16	PDIA4
Recoverec	33	9.42930536043062e-21	0.56737511473634	0.279	0.091	3.16240043178122e-16	AC068051.1
Recoverec	33	1.00764072649593e-20	0.32736803664152	0.324	0.116	3.37942546852204e-16	SLC38A4
Recoverec	33	1.04668545924273e-20	0.27828004936663	0.507	0.233	3.51037369320828e-16	TXNDC15
Recoverec	33	1.11657075049367e-20	0.46065649032175	0.805	0.513	3.74475498300568e-16	CDV3
Recoverec	33	1.14227735786063e-20	0.57579256894850	0.971	0.813	3.83096980279299e-16	NEAT1
Recoverec	33	1.48082488154739e-20	0.61564751051130	0.853	0.558	4.96639048773362e-16	DLG1
Recoverec	33	1.64003326622795e-20	0.61718096679960	0.632	0.346	5.50034356827531e-16	DUSP5

Recoverec	33	2.26659358866926e-20	0.73852592242835:	0.801	0.478	7.60170157767898e-16	CLIC4
Recoverec	33	2.40507957694607e-20	0.26947436832024:	0.232	0.067	8.06615588516175e-16	MOXD1
Recoverec	33	3.63135223792137e-20	0.53213917743379€	0.445	0.2	1.21788291355407e-15	Z93930.2
Recoverec	33	3.66052334060519e-20	0.46844115595233‹	0.452	0.205	1.22766631797217e-15	IFFO2
Recoverec	33	3.75150637855876e-20	0.35662882336649€	0.577	0.28	1.25818020924104e-15	IGLL5
Recoverec	33	3.87217845521659e-20	0.30485378582513!	0.335	0.123	1.29865121031054e-15	AL357060.1
Recoverec	33	4.30321723178881e-20	0.51779728613405‹	0.768	0.439	1.44321299519733e-15	DDX3Y
Recoverec	33	4.33827694911607e-20	0.29350476209341€	0.423	0.179	1.45497132319455e-15	TXNDC5
Recoverec	33	4.37911247115122e-20	0.28222816243896€	0.408	0.167	1.4686667405747e-15	AC023590.1
Recoverec	33	5.37500380165527e-20	0.56919991332925'	0.772	0.459	1.80266877499914e-15	CD38
Recoverec	33	5.91456015602019e-20	0.35720168831725€	0.239	0.071	1.98362518512605e-15	NRG2
Recoverec	33	8.58144975982427e-20	0.79221713935432!	0.39	0.169	2.87804662044986e-15	HIST1H4H
Recoverec	33	9.01423118634906e-20	0.52350453510368'	0.82	0.514	3.02319285527775e-15	LCORL
Recoverec	33	1.01059400369636e-19	0.56214016922055'	0.868	0.579	3.38933016959687e-15	PPP1R10
Recoverec	33	1.07591977488022e-19	0.32387175357025	0.551	0.268	3.60841974099329e-15	INSR
Recoverec	33	1.10608324278023e-19	0.91258482755369	0.562	0.289	3.70958197963635e-15	RGCC
Recoverec	33	1.26036132363759e-19	0.29398652311898:	0.316	0.115	4.22699980721573e-15	FRMD6
Recoverec	33	1.67355535022608e-19	0.36261790127593:	0.665	0.353	5.61276993358823e-15	PCCA
Recoverec	33	1.89902582914951e-19	0.70914866717838'	0.886	0.702	6.36895282580163e-15	RAPGEF2
Recoverec	33	3.66510321318138e-19	0.28804993355185'	0.474	0.217	1.22920231563677e-14	DTWD1
Recoverec	33	4.38440906586666e-19	0.39309641663356:	0.327	0.128	1.47044311251036e-14	AC012368.1
Recoverec	33	4.53890170526332e-19	0.53370290308819‹	0.897	0.684	1.52225685391121e-14	HERPUD1
Recoverec	33	4.87656191493739e-19	0.74290140635060‹	0.809	0.524	1.6355013350317e-14	NFKBIZ
Recoverec	33	4.97648600035247e-19	0.41231241551375'	0.452	0.205	1.66901387479821e-14	WARS
Recoverec	33	7.21153649561107e-19	0.41764907274469€	0.772	0.491	2.41860510989804e-14	TMEM258
Recoverec	33	8.04003740557576e-19	0.91356748691611:	0.882	0.753	2.696467745082e-14	PPP1R15A
Recoverec	33	8.30813393334283e-19	0.61637147658457	0.831	0.574	2.78638195856452e-14	HSPA1A
Recoverec	33	9.6890540707797e-19	0.29567483875617	0.474	0.225	3.2495149542581e-14	HSPA13
Recoverec	33	1.02858478488016e-18	0.82219043818491!	0.882	0.665	3.44966765153108e-14	HSPA8
Recoverec	33	1.10141337983456e-18	0.35358452542512!	0.647	0.347	3.69392019328916e-14	ABCA5
Recoverec	33	1.10178673775552e-18	0.51602388978013'	0.559	0.289	3.69517236108447e-14	SLC10A7

Recoverec	33	1.20493488128083e-18	0.36143120162436	0.647	0.34	4.04111060483965e-14	SLC44A1
Recoverec	33	1.25864630335135e-18	0.73153725271345	0.853	0.653	4.22124797217977e-14	DNAJB1
Recoverec	33	1.8068213392824e-18	0.28515608787195	0.279	0.099	6.05971740768531e-14	GSTM2
Recoverec	33	1.82657474499395e-18	0.49023274260485	0.735	0.474	6.12596637976072e-14	CD63
Recoverec	33	2.24787041800756e-18	0.64922508455999	0.827	0.58	7.53890780791375e-14	ARF4
Recoverec	33	2.35029719198443e-18	0.26190453067814	0.43	0.191	7.88242672247738e-14	MCEE
Recoverec	33	2.41370423771406e-18	0.46442856287145	0.261	0.089	8.09508127244541e-14	NRCAM
Recoverec	33	2.47531472479895e-18	0.40225760829142	0.566	0.293	8.30171052403071e-14	FOCAD
Recoverec	33	3.0472588669442e-18	0.35868735209488	0.919	0.601	1.02198967879575e-13	MT-ATP8
Recoverec	33	3.11162383000739e-18	0.33616347382709	0.408	0.184	1.04357640010788e-13	FKBP14
Recoverec	33	4.70147742646093e-18	0.29342544276196	0.493	0.239	1.57678149928647e-13	RDX
Recoverec	33	5.9337474481722e-18	0.27368069886830	0.375	0.158	1.99006021916799e-13	PDIA5
Recoverec	33	5.99027191586365e-18	0.36745180202012	0.529	0.267	2.00901739514235e-13	PAM
Recoverec	33	6.60073259642888e-18	0.37762757194973	0.651	0.365	2.21375369819032e-13	KDELR1
Recoverec	33	7.4290306757118e-18	0.48883008517613	0.257	0.087	2.49154830802022e-13	AC092546.1
Recoverec	33	7.87604203981284e-18	1.18288756056272	0.824	0.635	2.64146697931243e-13	HSPH1
Recoverec	33	7.94513160314006e-18	0.31816052904029	0.511	0.265	2.66463823706111e-13	CLPTM1L
Recoverec	33	8.9364952544471e-18	0.52641327645810	0.529	0.279	2.99712177843647e-13	CITED2
Recoverec	33	9.26938164234938e-18	0.65818856827795	0.368	0.161	3.10876521521113e-13	CHODL
Recoverec	33	9.94856852250966e-18	0.50324292851061	0.559	0.3	3.33655091107929e-13	ARMCX3
Recoverec	33	1.14366361006043e-17	0.28057104652572	0.544	0.268	3.83561901542067e-13	GORASP2
Recoverec	33	1.25627020612369e-17	0.42152458742022	0.559	0.305	4.21327901729765e-13	AIG1
Recoverec	33	1.51716702374432e-17	0.46560219243710	0.64	0.354	5.08827476423369e-13	SRBD1
Recoverec	33	1.62981435216895e-17	0.47318116101503	0.912	0.632	5.46607137430421e-13	TPD52
Recoverec	33	1.72910747089591e-17	0.27701034892917	0.551	0.271	5.79908063589071e-13	CHST15
Recoverec	33	2.12897795004631e-17	0.38981071405615	0.529	0.266	7.1401662488653e-13	TRIB1
Recoverec	33	2.22622940233301e-17	0.80799982518538	0.592	0.343	7.46632816954444e-13	IGHA2
Recoverec	33	2.64232393460733e-17	0.37886766359231	0.768	0.453	8.86182601188606e-13	SYNE2
Recoverec	33	2.97685705660345e-17	0.51090181531880	0.596	0.321	9.98378319643664e-13	ESR2
Recoverec	33	3.23322162371407e-17	0.40138848199713	0.54	0.289	1.08435786816123e-12	EIF2AK4
Recoverec	33	3.33537113627279e-17	0.29147501027926	0.397	0.174	1.11861677168317e-12	SWT1

Recoverec	33	4.90110378449397e-17	0.36059365149333(	0.485	0.242	1.64373218724359e-12	MTUS1
Recoverec	33	5.13646555452429e-17	0.25949962280347(	0.401	0.174	1.72266781767636e-12	ARSG
Recoverec	33	5.51109871803327e-17	0.56816476638067(	0.316	0.127	1.848312288054e-12	AL591518.1
Recoverec	33	5.73709161995287e-17	0.25439165951345(	0.658	0.37	1.92410578749979e-12	IQGAP2
Recoverec	33	6.45500716953208e-17	0.41002095015792	0.673	0.398	2.16488030451767e-12	ATG5
Recoverec	33	7.22624561881434e-17	0.27485859626527	0.765	0.435	2.42353825563795e-12	UTY
Recoverec	33	7.51379892815327e-17	0.35240444360605(	0.268	0.099	2.51997788452404e-12	LINC00571
Recoverec	33	7.51616851836964e-17	0.72461256894365(	0.893	0.631	2.52077259769081e-12	EIF2AK3
Recoverec	33	7.5479099142758e-17	0.311440819431186	0.724	0.421	2.53141802704982e-12	ARFGEF2
Recoverec	33	7.87376097909372e-17	0.28033635630943	0.71	0.416	2.64070195716845e-12	SEC61G
Recoverec	33	1.12082285824885e-16	0.32699199083480(	0.39	0.171	3.75901570199499e-12	EML6
Recoverec	33	1.14854224853456e-16	0.32626257511368(	0.412	0.189	3.85198099313522e-12	PAIP2B
Recoverec	33	1.29724115811351e-16	0.97418785367703(	0.625	0.391	4.35068739608108e-12	ATF3
Recoverec	33	1.31673914663089e-16	0.41222993738103(	0.445	0.217	4.41607974997068e-12	MB21D2
Recoverec	33	1.33439010078474e-16	0.59918790227003(	0.46	0.228	4.47527752001187e-12	AC007569.1
Recoverec	33	1.36095833295733e-16	0.45729170891668(	0.338	0.142	4.5643820570723e-12	ASNS
Recoverec	33	1.36918654030728e-16	0.38683145195889(	0.507	0.256	4.59197781888254e-12	KANSL1L
Recoverec	33	1.41925849494131e-16	0.38988676328803(	0.625	0.353	4.75990914033415e-12	C11orf80
Recoverec	33	1.63206620052697e-16	0.38282421163615(	0.456	0.222	5.47362362332737e-12	IDE
Recoverec	33	1.66353948675235e-16	0.33595858080203	0.801	0.471	5.57917873067003e-12	HIPK2
Recoverec	33	1.7856315976114e-16	0.36700840587820(	0.665	0.409	5.9886512520691e-12	CANX
Recoverec	33	2.14992444836258e-16	0.30800679184849(	0.548	0.293	7.21041661491843e-12	SEC23B
Recoverec	33	2.20153124461279e-16	0.33884090779545(	0.526	0.279	7.38349548818239e-12	AC012447.1
Recoverec	33	2.31130833434888e-16	0.45224002239710(	0.982	0.848	7.75166589173926e-12	RABGAP1L
Recoverec	33	2.52986834273114e-16	0.29981467401644(	0.574	0.315	8.48467244785169e-12	ME2
Recoverec	33	2.54826376935559e-16	0.27283240357849(	0.496	0.248	8.54636702966479e-12	CRELD2
Recoverec	33	2.76164108412513e-16	0.51968638320719(	0.68	0.401	9.26199186793885e-12	IRF4
Recoverec	33	3.74603793499851e-16	0.29338859452582(	0.522	0.264	1.2563462026398e-11	SDF2L1
Recoverec	33	4.39263226267721e-16	0.82863966233026	0.864	0.665	1.47320100825668e-11	HSPD1
Recoverec	33	4.47114892625006e-16	0.32642377431177(	0.478	0.235	1.49953392688575e-11	HIST1H2BD
Recoverec	33	5.39381862338281e-16	0.28187883259095(	0.588	0.319	1.80897888991013e-11	ECPAS

Recoverec	33	5.59759022680311e-16	0.52617416254862ŧ	0.298	0.119	1.87731981026523e-11	HIST2H2BF
Recoverec	33	5.96110926468765e-16	0.40935100940105‹	0.746	0.47	1.99923682519094e-11	USP48
Recoverec	33	7.20268107172003e-16	0.27704026944359‹	0.54	0.293	2.41563517783346e-11	MYDGF
Recoverec	33	7.41037583160845e-16	0.392511531344711	0.456	0.226	2.48529184640484e-11	XRCC4
Recoverec	33	7.92170709072156e-16	0.35671533607523ĉ	0.629	0.361	2.6567821240862e-11	DAP
Recoverec	33	9.34998192873444e-16	0.42085351128046ĉ	0.298	0.122	3.13579693925895e-11	DEPTOR
Recoverec	33	9.6656743858169e-16	0.62502858667424‹	0.423	0.213	3.24167387551527e-11	RPL36A-HNRNPH
Recoverec	33	1.09394716789971e-15	0.27039647459558‹	0.357	0.159	3.66888001170204e-11	NFXL1
Recoverec	33	1.15557032125354e-15	0.28431436624414‹	0.739	0.426	3.87555174342012e-11	SEC14L1
Recoverec	33	1.33801564898399e-15	0.26909255239334‹	0.136	0.032	4.48743688356252e-11	AC022217.2
Recoverec	33	1.46338174492462e-15	0.26493360906145‹	0.287	0.113	4.9078896961282e-11	BEX5
Recoverec	33	1.66047222026198e-15	0.67534326649175	0.562	0.328	5.56889173231464e-11	LMNA
Recoverec	33	1.80515622004678e-15	0.31028737060434‹	0.419	0.198	6.05413293079289e-11	KNTC1
Recoverec	33	1.83941886378289e-15	0.55963975301679ĉ	0.544	0.305	6.16904298535506e-11	KCNQ1OT1
Recoverec	33	1.89294844267822e-15	0.50483625300736ŧ	0.338	0.144	6.34857048705420e-11	CADM1
Recoverec	33	2.40737477609715e-15	0.52422698856170‹	0.592	0.349	8.07385352407462e-11	HIST1H2AC
Recoverec	33	2.57640040392691e-15	0.27360245418719	0.669	0.399	8.64073167469006e-11	EIF2S2
Recoverec	33	2.88776841079765e-15	0.32970241790705ŧ	0.43	0.211	9.68499769613316e-11	PGM3
Recoverec	33	2.94877055889129e-15	0.32306826082827ɛ	0.463	0.234	9.88958670040961e-11	UBA5
Recoverec	33	3.05879885590512e-15	0.36628043879784ɛ	0.871	0.675	1.02585996029346e-10	SUB1
Recoverec	33	3.62738988015616e-15	0.76029718404644‡	0.401	0.197	1.21655401800677e-10	HIST1H2BC
Recoverec	33	3.71675205887693e-15	0.387117511721442	0.651	0.406	1.24652430550615e-10	PDIA6
Recoverec	33	4.47177708206102e-15	0.28343988834919ŧ	0.29	0.114	1.49974459778163e-10	SNTB1
Recoverec	33	5.03184628560637e-15	0.29186382248621ɛ	0.496	0.256	1.68758060726666e-10	TMEM107
Recoverec	33	5.55754236164456e-15	0.32839672241368ɛ	0.162	0.045	1.86388855724835e-10	HSPA1L
Recoverec	33	5.56383064947051e-15	0.25639277073117ĉ	0.518	0.27	1.86599752321942e-10	SEC22B
Recoverec	33	5.97210135364039e-15	0.34338126926316ɛ	0.673	0.398	2.00292335198391e-10	GALNT2
Recoverec	33	6.0529237173193e-15	0.51873400599074‹	0.312	0.135	2.03002955631455e-10	SCNN1B
Recoverec	33	7.15904585459082e-15	0.45462234282604ŧ	0.915	0.66	2.40100079871267e-10	DENND5B
Recoverec	33	7.94696211680175e-15	0.31498619563081‡	0.11	0.022	2.66525215473297e-10	VWC2
Recoverec	33	8.23523587459759e-15	0.34887002982986‹	0.827	0.542	2.76193340762254e-10	SCFD1

Recoverec	33	8.30059077649987e-15	0.35954372835665	0.54	0.295	2.78385213462253e-10	CRADD
Recoverec	33	9.75082456637599e-15	0.25440036071550	0.5	0.264	3.27023154307118e-10	SEC61A1
Recoverec	33	9.76691244103622e-15	0.37888385757071	0.805	0.527	3.27562709447473e-10	MON2
Recoverec	33	1.07938403446683e-14	0.38239557759635	0.165	0.047	3.62003817479979e-10	ACVR1C
Recoverec	33	1.25456531331301e-14	0.37870217856294	0.342	0.155	4.20756114778917e-10	AL139383.1
Recoverec	33	1.26344545985691e-14	0.34603041063671	0.353	0.163	4.23734338326809e-10	KIF21A
Recoverec	33	1.31082842850174e-14	0.32978144843204	0.496	0.272	4.39625638350913e-10	SLC41A2
Recoverec	33	1.49186017567872e-14	0.38202513743102	0.287	0.118	5.00340065719128e-10	TULP2
Recoverec	33	1.65499721549104e-14	0.48921684962454	0.702	0.466	5.55052966131385e-10	FAM69A
Recoverec	33	1.71115816672659e-14	0.33412551156962	0.765	0.511	5.73888225956764e-10	PDIA3
Recoverec	33	1.95124818521892e-14	0.25978189334394	0.467	0.246	6.54409616358723e-10	MTHFD2
Recoverec	33	2.02297614862155e-14	0.26283787193677	0.566	0.309	6.78465740724697e-10	GFPT1
Recoverec	33	2.17920423637257e-14	0.61095186643777	0.312	0.135	7.30861516794632e-10	ASTL
Recoverec	33	2.72026189341975e-14	0.29642588792582	0.426	0.213	9.12321433815115e-10	AARS
Recoverec	33	2.72829785956863e-14	0.29622982374294	0.232	0.085	9.15016536142127e-10	REPS2
Recoverec	33	2.78107228503757e-14	0.29145938363092	0.489	0.265	9.32716022955899e-10	ALG5
Recoverec	33	2.91009506511769e-14	0.67899809089250	0.478	0.264	9.75987682939171e-10	MIR155HG
Recoverec	33	3.1186162549031e-14	0.34540322077852	0.243	0.092	1.0459215195694e-09	SRGAP3
Recoverec	33	3.55544577623256e-14	0.25048789331030	0.61	0.345	1.19242540443288e-09	PLPP5
Recoverec	33	4.03395750485034e-14	0.29865449892817	0.515	0.282	1.35290866797671e-09	IARS
Recoverec	33	4.13356063460313e-14	0.49868517977509	0.897	0.672	1.3863135656332e-09	CDK14
Recoverec	33	4.58731435925588e-14	0.27757541932127	0.452	0.23	1.53849348980724e-09	BTN3A1
Recoverec	33	4.67782085075523e-14	0.25456717327853	0.371	0.172	1.56884755692629e-09	SLFN11
Recoverec	33	5.19982804368036e-14	0.34926530708628	0.824	0.573	1.74391832928952e-09	XRN1
Recoverec	33	5.46494148229799e-14	0.30370370873026	0.221	0.079	1.8328320743331e-09	ARFGEF3
Recoverec	33	5.72313281922529e-14	0.30790202040371	0.46	0.246	1.91942428491178e-09	ST7
Recoverec	33	6.07687983019152e-14	0.25405003292632	0.529	0.29	2.03806395744963e-09	UFL1
Recoverec	33	6.20467413503013e-14	0.38067635974679	0.735	0.463	2.08092361140641e-09	ALCAM
Recoverec	33	6.78531535378229e-14	0.37465500707640	0.265	0.107	2.2756590633515e-09	MERTK
Recoverec	33	1.26145259829627e-13	0.41135715152247	0.555	0.318	4.23065972416603e-09	WDR74
Recoverec	33	1.36110441397342e-13	0.34553987988389	0.272	0.113	4.56487198358405e-09	SLCO3A1

Recoverec	33	1.36552002717871e-13	0.63076198272493	0.952	0.914	4.57968106715196e-09	FOS
Recoverec	33	1.48366270669064e-13	0.91254739828515	0.327	0.59	4.97590798569907e-09	IGHM
Recoverec	33	1.54611715967886e-13	0.35806650892386	0.688	0.43	5.18536773013096e-09	ERCC1
Recoverec	33	1.62330960814273e-13	0.2870966056774	0.702	0.434	5.44425576378909e-09	BICD1
Recoverec	33	1.70709110067795e-13	0.38215774716387	0.772	0.498	5.7252421334537e-09	POU2AF1
Recoverec	33	1.7451178876913e-13	0.32707305101949	0.665	0.405	5.85277637173909e-09	IQCB1
Recoverec	33	2.10545537981213e-13	0.32102447958576	0.596	0.354	7.06127625281393e-09	ITGA6
Recoverec	33	2.14631794963552e-13	0.34421566009935	0.772	0.512	7.19832113948762e-09	SCAPER
Recoverec	33	2.36933812452297e-13	0.52820284864837	0.585	0.346	7.94628620202513e-09	SESN1
Recoverec	33	2.3771059454299e-13	0.44226755304794	0.368	0.182	7.97233791978281e-09	DENND2C
Recoverec	33	2.38070412596739e-13	0.40353768262395	0.651	0.395	7.98440549766944e-09	ABHD3
Recoverec	33	2.64820273661393e-13	1.01370053304374	0.761	0.564	8.88154233805579e-09	PELI1
Recoverec	33	2.90818665296603e-13	0.34042488711508	0.61	0.361	9.75347639671746e-09	OXR1
Recoverec	33	3.1849494291317e-13	0.32058393594205	0.838	0.573	1.06816833954219e-08	PCMTD1
Recoverec	33	3.20595845883056e-13	0.42984536343063	0.151	0.044	1.07521434792259e-08	CHSY3
Recoverec	33	3.40906837459613e-13	0.26152377716604	0.43	0.221	1.14333335147205e-08	COG6
Recoverec	33	3.56233164357084e-13	0.28453280489129	0.761	0.504	1.19473478662079e-08	RPS27L
Recoverec	33	4.03848661810402e-13	0.28404696360270	0.724	0.458	1.35442764197973e-08	EVI5
Recoverec	33	4.07141567513953e-13	0.25131517596694	0.493	0.262	1.3654713891283e-08	DUSP22
Recoverec	33	4.43380044670541e-13	0.34601110336436	0.228	0.086	1.48700799381606e-08	LINC00877
Recoverec	33	4.64638256261621e-13	0.42640633595692	0.515	0.279	1.55830378385022e-08	VPS54
Recoverec	33	4.92633503085782e-13	0.27692524845272	0.312	0.142	1.6521942426491e-08	CFL2
Recoverec	33	4.94552259957319e-13	0.42115790132370	0.261	0.109	1.65862936944486e-08	DGKI
Recoverec	33	5.03728565816095e-13	0.45366253089503	0.243	0.096	1.68940486403402e-08	AC108866.1
Recoverec	33	5.18271002792397e-13	0.28346282226875	0.305	0.137	1.73817728916514e-08	CCSAP
Recoverec	33	5.38287867190381e-13	0.29525800869750	0.482	0.26	1.8053098489831e-08	CASP10
Recoverec	33	5.67091805348465e-13	0.47723377568736	0.533	0.304	1.90191249677768e-08	NBEA
Recoverec	33	6.10415807939912e-13	0.41919939450864	0.717	0.472	2.04721253666888e-08	UMAD1
Recoverec	33	6.88483867879809e-13	0.27378180534246	0.335	0.155	2.3090371960953e-08	HIST1H2BG
Recoverec	33	7.3127508581626e-13	0.28445594611734	0.489	0.275	2.45255038281057e-08	SRPRA
Recoverec	33	8.72663044802896e-13	0.35755571406327	0.857	0.62	2.92673731965995e-08	GOLGA4

Recoverec	33	8.96845571530538e-13	0.40042727544535‹	0.121	0.031	3.00784067779912e-08	AC068633.1
Recoverec	33	9.79039690708369e-13	0.49960947743733‹	0.441	0.244	3.28350331469773e-08	ICAM1
Recoverec	33	9.87585857216568e-13	0.34768286174666‹	0.787	0.495	3.31216544793293e-08	OGT
Recoverec	33	1.02176715266978e-12	0.37399840583511‹	0.794	0.521	3.42680267662392e-08	ASCC3
Recoverec	33	1.09094828381043e-12	0.29524461260042‹	0.217	0.082	3.65882235424343e-08	AC104596.1
Recoverec	33	1.10521523396751e-12	0.32085419382169‹	0.173	0.057	3.70667085168022e-08	SLC7A11
Recoverec	33	1.25460248361525e-12	0.32643275581492‹	0.688	0.438	4.20768580954881e-08	MYO5A
Recoverec	33	1.34077821386765e-12	0.42600254700763‹	0.176	0.06	4.49670197366933e-08	SPERT
Recoverec	33	1.34978488260777e-12	0.25757806246073‹	0.276	0.119	4.52690853928995e-08	ABCA10
Recoverec	33	1.69645894544469e-12	0.32721033304459‹	0.783	0.538	5.68958401123239e-08	UBXN4
Recoverec	33	1.70122786974646e-12	0.40062490461665‹	0.206	0.075	5.70557802955568e-08	HSPE1-MOB4
Recoverec	33	1.76506184640373e-12	0.30448778329385‹	0.544	0.313	5.91966442046883e-08	TARSL2
Recoverec	33	2.2211431719665e-12	0.29123688653848‹	0.559	0.337	7.44926997014125e-08	CCNC
Recoverec	33	2.30162779600391e-12	0.32938366754032‹	0.805	0.552	7.71919930223791e-08	NSMCE2
Recoverec	33	2.884983039923e-12	0.28023976637938‹	0.364	0.184	9.67565611929376e-08	GOLPH3L
Recoverec	33	2.91502179504873e-12	0.27356901292437	0.618	0.373	9.77640009623442e-08	MDFIC
Recoverec	33	2.92133845273131e-12	0.28526855779468‹	0.54	0.307	9.79758490277026e-08	PLXNC1
Recoverec	33	3.00364544322018e-12	0.25164026323779‹	0.151	0.047	1.00736260874718e-07	LINC02341
Recoverec	33	3.36740145123526e-12	0.67697635594660‹	0.206	0.078	1.12935909871528e-07	AF165147.1
Recoverec	33	3.95103629405943e-12	0.30077785544805‹	0.346	0.172	1.32509855230165e-07	ANKRD36B
Recoverec	33	4.75154616067092e-12	0.35055474585654‹	0.496	0.287	1.59357355136581e-07	CASP3
Recoverec	33	5.29996091543588e-12	0.44597639081468‹	0.559	0.339	1.77750089181889e-07	LRRK1
Recoverec	33	5.34609901639613e-12	0.27832947165654‹	0.592	0.36	1.79297468811893e-07	TFG
Recoverec	33	5.75465512303955e-12	0.32736850515682‹	0.474	0.264	1.92999623516501e-07	GLRX
Recoverec	33	6.49641988166622e-12	0.28939946247214‹	0.739	0.48	2.17876929991322e-07	SCFD2
Recoverec	33	8.64803455346689e-12	0.27612465730700‹	0.268	0.117	2.90037782854173e-07	RAPGEF4
Recoverec	33	8.67666342095645e-12	0.44383376024736‹	0.257	0.111	2.90997937812037e-07	NPAS3
Recoverec	33	9.29058941837346e-12	0.33468483167112‹	0.423	0.231	3.11587787913409e-07	AC026202.2
Recoverec	33	1.03688753885871e-11	0.30686223002314‹	0.551	0.324	3.47751342782436e-07	MAPKAPK2
Recoverec	33	1.10098371793464e-11	0.25021872180447‹	0.493	0.284	3.69247919320921e-07	COPB2
Recoverec	33	1.13928090938706e-11	0.34462325740202	0.849	0.613	3.82092031390231e-07	FBXL17

Recoverec	33	1.29548423840264e-11	0.52348184498507	0.254	0.112	4.34479503875478e-07	AC087280.2
Recoverec	33	1.36012930398553e-11	0.25416064265242	0.305	0.142	4.56160165970668e-07	AC011511.2
Recoverec	33	1.38975811678132e-11	0.32237885694182	0.511	0.298	4.66097077206121e-07	TPTEP2-CSNK1E
Recoverec	33	1.39676504159333e-11	0.42767552541166	0.246	0.104	4.68447059649571e-07	AC104248.1
Recoverec	33	1.71374968678988e-11	0.39558750615218	0.309	0.146	5.74757369955589e-07	IGLV10-54
Recoverec	33	1.91305376358553e-11	0.25022218606641	0.305	0.145	6.41599971231314e-07	ARHGAP6
Recoverec	33	1.9220575135724e-11	0.28104597429529	0.151	0.048	6.44619648901912e-07	SCART1
Recoverec	33	2.08890411238637e-11	0.28188094613843	1	0.954	7.0057666121214e-07	MT-ND5
Recoverec	33	2.71892964721813e-11	0.36320104339804	0.496	0.29	9.11874625084015e-07	MEF2C-AS1
Recoverec	33	2.72953536662458e-11	0.42158235685071	0.746	0.504	9.1543157125855e-07	DPYD
Recoverec	33	2.87161141662056e-11	0.25926856440701	0.438	0.238	9.63081036906204e-07	UBN2
Recoverec	33	3.064847807619e-11	0.32911199648387	0.467	0.26	1.02788865771926e-06	TSPYL2
Recoverec	33	3.09949240985627e-11	0.33505691684958	0.732	0.513	1.0395077644176e-06	EGR1
Recoverec	33	3.21682652173436e-11	0.27607061173662	0.739	0.483	1.07885927885927e-06	DENND1B
Recoverec	33	3.24736466101439e-11	0.25207020993854	0.364	0.184	1.08910116001101e-06	AC022182.1
Recoverec	33	3.47376119007136e-11	0.28601757241253	0.426	0.233	1.16503002792613e-06	MYO6
Recoverec	33	4.24595817050403e-11	0.32715529565797	0.849	0.679	1.42400945122364e-06	SERP1
Recoverec	33	4.28255654633324e-11	0.32492550053311	0.71	0.453	1.43628381450924e-06	ITGA4
Recoverec	33	4.89384486515379e-11	0.33832662883361	0.772	0.519	1.64129769087528e-06	MYO1D
Recoverec	33	5.48941518520964e-11	0.66012017494389	0.96	0.918	1.84104006481561e-06	HSP90AA1
Recoverec	33	5.85258781824778e-11	0.62520128282235	0.29	0.142	1.96284090248394e-06	BAG3
Recoverec	33	6.8754854378535e-11	0.29004858495291	0.434	0.239	2.30590030614731e-06	C12orf45
Recoverec	33	7.99927511753411e-11	0.31331905028731	0.533	0.318	2.68279688891859e-06	RUFY3
Recoverec	33	8.31536093564944e-11	0.26868268493324	0.647	0.404	2.78880575059811e-06	IL6ST
Recoverec	33	1.03755424446987e-10	0.34131416263594	0.868	0.668	3.47974942510305e-06	CDKAL1
Recoverec	33	1.15528328881822e-10	0.27347158082225	0.301	0.147	3.87458909403855e-06	CXorf21
Recoverec	33	1.16368022925837e-10	0.31581393817386	0.478	0.272	3.90275075288674e-06	C22orf34
Recoverec	33	1.16740042890857e-10	0.46161405456662	0.324	0.162	3.91522755847355e-06	AC084200.1
Recoverec	33	1.20523998323118e-10	0.53922384225009	0.515	0.319	4.04213385576075e-06	AL691403.1
Recoverec	33	1.30559194748373e-10	0.35527639328382	0.904	0.707	4.37869427347092e-06	EXOC4
Recoverec	33	1.34084984020559e-10	0.25638485824514	0.618	0.387	4.49694219408151e-06	ACBD3

Recoverec	33	1.48613755710254e-10	0.50545727963690!	0.805	0.606	4.98420813901051e-06	SRGN
Recoverec	33	1.60477926978396e-10	0.43535705311584	0.408	0.226	5.38210871500146e-06	DNAJB4
Recoverec	33	1.86104705754907e-10	0.37631824439462	0.301	0.145	6.24157962160806e-06	AC109492.1
Recoverec	33	2.21759080109153e-10	0.26642589002020	0.136	0.044	7.43735602870076e-06	LINC02334
Recoverec	33	3.05958250413362e-10	0.26401010649358	0.408	0.235	1.02612278023633e-05	SARS
Recoverec	33	3.07716307251688e-10	0.32336489624057	0.415	0.234	1.03201895126071e-05	AL606489.1
Recoverec	33	3.23375101772881e-10	0.33819169309172	0.438	0.26	1.08453541632589e-05	FAM13A
Recoverec	33	3.30113957622144e-10	0.25968177451917	0.57	0.349	1.10713619107315e-05	TNIP1
Recoverec	33	3.47310301213445e-10	0.29643306828086	0.346	0.179	1.16480928820965e-05	AC104123.1
Recoverec	33	3.61543543770101e-10	0.33347985973024	0.592	0.374	1.21254473709617e-05	CAB39
Recoverec	33	4.45946047136343e-10	0.29229322308107	0.919	0.677	1.49561385288587e-05	SEL1L3
Recoverec	33	5.44525139515808e-10	0.68255044036009	0.518	0.341	1.82622841290812e-05	GLA
Recoverec	33	5.62043593935054e-10	0.25032787029667	0.39	0.217	1.88498180533938e-05	IL15
Recoverec	33	6.07248661047769e-10	0.32364964354283	0.452	0.265	2.03659055942201e-05	CEBPB
Recoverec	33	6.41959871544294e-10	0.30152422542324	0.199	0.083	2.15300501718525e-05	ARMC2
Recoverec	33	6.46971242588101e-10	0.26624930000933	0.526	0.322	2.16981215339197e-05	HERC2
Recoverec	33	6.57792027285715e-10	0.44309050052320	0.82	0.617	2.20610290111083e-05	MORF4L2
Recoverec	33	7.29954683178775e-10	0.25452196674734	0.426	0.244	2.44812201644498e-05	KCNQ1
Recoverec	33	8.13748392372542e-10	0.28599745164249	0.246	0.115	2.72914935833903e-05	FAR2
Recoverec	33	8.65817320021165e-10	0.35001201215274	0.585	0.363	2.90377812788698e-05	H1FX
Recoverec	33	9.40715989251444e-10	0.31525017183192	0.235	0.107	3.15497328475149e-05	ATF5
Recoverec	33	9.62960153769186e-10	0.27718079608637	0.717	0.481	3.2295757637111e-05	GBF1
Recoverec	33	1.05069897860198e-09	0.38052188221214	0.279	0.136	3.52383423443531e-05	GRIP1
Recoverec	33	1.13694960152985e-09	0.26706179678538	0.596	0.374	3.81310157361082e-05	P2RY8
Recoverec	33	1.36050746767534e-09	0.34879735076367	0.812	0.638	4.56286994508956e-05	ZFP36
Recoverec	33	1.36063032392451e-09	0.28127230841319	0.732	0.475	4.56328198037803e-05	NBAS
Recoverec	33	1.49871486243683e-09	0.45982746179517	0.272	0.137	5.02638990564064e-05	PRR3
Recoverec	33	1.51273768993366e-09	0.26542785098964	0.21	0.092	5.07341966449952e-05	LINC02427
Recoverec	33	1.59260297662283e-09	0.32608843848209	0.261	0.128	5.34127186299763e-05	AL627171.1
Recoverec	33	1.88995572834084e-09	0.48093638144810	0.64	0.453	6.33853352170949e-05	TPM4
Recoverec	33	2.17481261195737e-09	0.30542015232091	0.114	0.035	7.29388653798263e-05	CNTN5

Recoverec	33	2.21530235221071e-09	0.28097076020945‸	0.522	0.325	7.42968102884429e-05	ARRDC3
Recoverec	33	2.96131344025231e-09	0.25894197998483‸	0.467	0.282	9.93165301591821e-05	MAGED1
Recoverec	33	3.34293710629323e-09	0.35724427337673‸	0.886	0.622	0.000112115424670862	HDAC9
Recoverec	33	3.62569252619855e-09	0.43888088762807‸	0.798	0.598	0.000121598475943647	SLC38A2
Recoverec	33	3.63396719253157e-09	0.35866501655187‸	0.938	0.713	0.000121875991703124	GLS
Recoverec	33	3.64479117105277e-09	0.37808185940144‸	0.904	0.805	0.000122239006294768	UBC
Recoverec	33	4.21049619279681e-09	0.31885840230187‸	0.838	0.7	0.000141211621314019	RPL36AL
Recoverec	33	4.46494504182567e-09	0.28941132914982‸	0.265	0.13	0.000149745326812749	LINC01376
Recoverec	33	5.19366710324761e-09	0.36363481172857‸	0.901	0.696	0.000174185207308718	FUT8
Recoverec	33	5.93524376303321e-09	0.27159533807886	0.316	0.167	0.000199056205324608	FMNL2
Recoverec	33	6.35929347164932e-09	0.26117162512481‸	0.39	0.225	0.000213277984452175	ICE1
Recoverec	33	6.49292136490443e-09	0.44886849069118‸	0.103	0.031	0.000217759596736165	LINC01695
Recoverec	33	6.63020965239361e-09	0.26186276796728‸	0.18	0.077	0.000222363971321977	IL26
Recoverec	33	6.86116205717879e-09	0.41446775689816‸	0.188	0.081	0.000230109653073662	NRXN1
Recoverec	33	7.07282567610801e-09	0.34981717299030‸	0.415	0.242	0.00023720842752531	NFE2L3
Recoverec	33	7.47960271054450e-09	0.35674532217729‸	0.599	0.402	0.000250850915706241	PPP1R15B
Recoverec	33	8.61393275145662e-09	0.48425383697728‸	0.39	0.227	0.000288894076618352	ZFAND2A
Recoverec	33	1.11603253034385e-08	0.28760001478802‸	0.261	0.133	0.000374294990026722	STX11
Recoverec	33	1.60507701784702e-08	0.28798434846484‸	0.246	0.125	0.000538310730245535	SPAG16
Recoverec	33	1.73022299490811e-08	0.32187905203878‸	0.265	0.137	0.00058028218803228	AC021678.2
Recoverec	33	1.92449966705829e-08	0.29925179650906‸	0.217	0.102	0.00064543869833801	AZIN1-AS1
Recoverec	33	2.82400609329194e-08	0.26744711688043‸	0.298	0.164	0.000947115163568251	HIST1H2BJ
Recoverec	33	2.85732008670754e-08	0.25917501283313‸	0.511	0.308	0.000958288010679976	MIR4435-2HG
Recoverec	33	3.49333769825807e-08	0.30642499941234‸	0.691	0.48	0.00117159559724179	MAP3K5
Recoverec	33	3.77244026599782e-08	0.42244064075456‸	0.901	0.699	0.00126520101641035	GNG7
Recoverec	33	4.33845434116777e-08	0.42394447367636‸	0.68	0.475	0.00145503081694085	AC007384.1
Recoverec	33	4.5194305278751e-08	0.31615047734704‸	0.272	0.143	0.00151572661043875	EML5
Recoverec	33	5.29901881646855e-08	0.52660012620996‸	0.456	0.302	0.00177718493066722	ANKRD37
Recoverec	33	5.35966584447655e-08	0.26573393774848‸	0.504	0.321	0.00179752473092055	HSH2D
Recoverec	33	8.09061929553295e-08	0.31069214838465‸	0.721	0.492	0.00271343189933584	UBE2G1
Recoverec	33	1.22972052868513e-07	0.41479181887599‸	0.673	0.485	0.0041242367091042	BRD2

Recoverec	33	1.25978441736564e-07	0.26699916484610	0.493	0.317	0.00422506497896088	AC020916.1
Recoverec	33	1.2758611003974e-07	0.38190397854377	0.375	0.227	0.00427898295851278	SLC2A13
Recoverec	33	2.19295076078284e-07	0.25326579550140	0.676	0.484	0.00735471826151348	TMEM117
Recoverec	33	2.25028152134774e-07	0.42954739742186	0.871	0.698	0.00754699416629605	SQSTM1
Recoverec	33	2.43792359909828e-07	0.29312479425656	0.173	0.08	0.00817630816665581	ATP10B
Recoverec	33	2.58280724163598e-07	0.26680685814155	0.36	0.222	0.00866221892699875	KAZN
Recoverec	33	3.33219635300497e-07	0.31315717391913	0.886	0.679	0.0111755201287081	FTX
Recoverec	33	4.98471644753281e-07	0.28868963400651	0.408	0.252	0.0167177420217356	GALM
Recoverec	33	6.38465411209922e-07	0.25054780191018	0.864	0.675	0.0214128529611584	ELL2
Recoverec	33	7.42739767160256e-07	0.27942831901198	0.246	0.136	0.0249100063110207	XKR9
Recoverec	33	9.43688633547536e-07	0.47039090695481	0.632	0.488	0.0316494293919173	TNFAIP3
Recoverec	33	9.7672991867261e-07	0.25024266580962	0.824	0.593	0.032757568012442	FAM214A
Recoverec	33	1.03637653758829e-06	0.25253820247519	0.713	0.493	0.034757996317636	CYLD
Recoverec	33	1.04147237168175e-06	0.26750286012904	0.21	0.105	0.0349289004014625	AL121985.1
Recoverec	33	1.15351568361484e-06	0.32639338044440	0.746	0.581	0.0386866089970745	HSPE1
Recoverec	33	1.44694218852499e-06	0.29904638677135	0.368	0.232	0.0485275471187511	PATL2
Recoverec	33	1.48864474243264e-06	0.511997890841411	0.221	0.119	0.0499261673717058	CCL3
Recoverec	40	2.06701700429728e-76	2.06309482613066	1	0.993	6.93236162901221e-72	MT-ND3
Recoverec	40	6.86320310519191e-64	1.20049700931274	0.879	0.264	2.30178105741926e-59	MT1H
Recoverec	40	2.26840977744044e-63	1.60729790517452	1	0.984	7.60779271157974e-59	MT-ND2
Recoverec	40	5.7111876465611e-60	1.71562424186587	1	0.614	1.91541811290366e-55	MT-ATP8
Recoverec	40	2.29541439751604e-55	0.52580571964257	0.546	0.097	7.6983608063893e-51	MT1M
Recoverec	40	3.45767311597538e-52	2.41180545914121	0.936	0.465	1.15963440963582e-47	CROCC
Recoverec	40	1.90522300997924e-50	1.31146427428218	0.95	0.523	6.38973693086838e-46	MT1G
Recoverec	40	1.33277709322732e-48	0.30627100444405	0.312	0.034	4.46986781526578e-44	MT1B
Recoverec	40	1.7773073374334e-47	0.85453496648001	0.333	0.041	5.96073334828415e-43	RAB44
Recoverec	40	3.01519924151138e-46	1.25991804058705	0.525	0.113	1.01123752161809e-41	TULP2
Recoverec	40	4.59027404727355e-46	1.34846315749706	0.638	0.169	1.5394861099746e-41	AL360012.1
Recoverec	40	9.07739298377888e-46	1.11850181672325	0.489	0.099	3.04437605889976e-41	AL353759.1
Recoverec	40	1.82174769328577e-45	0.95529338021550	0.518	0.109	6.10977741374181e-41	PTCH2
Recoverec	40	5.28909288021421e-44	1.09646196049802	1	0.986	1.77385597016624e-39	MT-ND1

Recoverec	40	1.10714351447071e-43	1.63118406215367	0.993	0.821	3.71313791883188e-39	NEAT1
Recoverec	40	2.80268687409385e-42	1.54070423839959	0.709	0.234	9.39965123833595e-38	HIST1H2BD
Recoverec	40	5.81629487309421e-42	0.60333822514857(	0.333	0.048	1.95066897453833e-37	FP671120.1
Recoverec	40	5.89700813437516e-41	0.70483990294155:	0.468	0.099	1.97773858810674e-36	SDR42E2
Recoverec	40	3.82876155155322e-40	0.91890007242921:	0.66	0.201	1.28409004915992e-35	Z93930.2
Recoverec	40	9.25451225899821e-40	1.39073275905998	0.901	0.432	3.10377832142282e-35	CREB3L2
Recoverec	40	1.75849640188986e-39	0.70433072161092(	0.745	0.239	5.8976452326582e-35	MT1E
Recoverec	40	3.90385978616833e-39	1.6355501721902	0.66	0.212	1.30927649508513e-34	POLG2
Recoverec	40	4.79623296276124e-39	0.35132037105863:	0.787	0.284	1.60856061105087e-34	IGLL5
Recoverec	40	5.51235474173926e-39	0.98611965354370(	1	0.956	1.84873353328451e-34	MT-ND5
Recoverec	40	9.39622722045335e-39	0.46767029550651!	0.277	0.034	3.15130668519565e-34	SPSB2
Recoverec	40	3.51692916089516e-38	0.71963918666523‹	0.631	0.19	1.17950770198102e-33	AC139887.2
Recoverec	40	2.4126282944673e-37	0.78335297604851:	0.355	0.062	8.09147277398444e-33	GIPC1
Recoverec	40	3.84253184845011e-36	0.33897795252262:	0.34	0.056	1.2887083313332e-31	HCG27
Recoverec	40	7.71785226896308e-36	1.09907650979982	0.624	0.194	2.58841329396484e-31	HIST1H2BC
Recoverec	40	7.93789028334661e-36	0.75429451221200!	0.482	0.118	2.66220964322878e-31	KMT2E-AS1
Recoverec	40	8.92751449385695e-36	0.86506567588899	1	0.998	2.99410981094974e-31	MT-CYB
Recoverec	40	2.78783963615156e-35	0.67408786254781:	0.319	0.051	9.3498565717251e-31	AC011452.1
Recoverec	40	3.01064057162605e-35	1.65374539924476	0.667	0.245	1.00970863491195e-30	GALM
Recoverec	40	3.05404777921946e-35	1.08075486521414	0.915	0.469	1.02426654419462e-30	RUNX2
Recoverec	40	5.59500028117047e-35	1.10472674437642	0.752	0.282	1.87645119429895e-30	LINC02362
Recoverec	40	8.83861789567152e-35	0.57861599281853(	0.326	0.055	2.96429566985031e-30	BAIAP2
Recoverec	40	1.3950766884874e-34	0.67784050659189:	0.922	0.444	4.67880819784904e-30	MT2A
Recoverec	40	3.81073337631216e-34	0.88130235024696:	1	0.992	1.27804375974757e-29	MT-ND4
Recoverec	40	8.35739815855755e-34	1.2918347615537	0.858	0.425	2.80290419441703e-29	CPEB4
Recoverec	40	1.27204708736556e-33	0.88128902906990	0.816	0.362	4.26619152160662e-29	MT-ND6
Recoverec	40	1.40843849433758e-33	0.74695619812184(	0.582	0.183	4.72362102230937e-29	TXNDC5
Recoverec	40	1.7823875612898e-33	1.22208255248621	0.929	0.535	5.97777140305375e-29	FNDC3B
Recoverec	40	2.60191046061321e-33	0.43377957061060	0.291	0.044	8.7262873028046e-29	NUP210L
Recoverec	40	3.45619700344099e-33	0.38715173836187:	0.227	0.027	1.15913935101404e-28	IGKJ5
Recoverec	40	9.13969174994889e-33	1.19257259299349	0.567	0.177	3.06526981909786e-28	SLC16A1

Recoverec	40	1.6527019706693e-32	1.05451122237293	0.986	0.718	5.54283186923069e-28	MT-ND4L
Recoverec	40	2.37055723052062e-32	1.02792366160976	0.851	0.395	7.95037483972006e-28	PDK1
Recoverec	40	4.55003650566255e-32	0.96531424573976	0.695	0.253	1.5259912432691e-27	ESR1
Recoverec	40	8.14103764438281e-32	0.73062145710551	0.695	0.251	2.73034120517311e-27	WNT5B
Recoverec	40	9.60594381796501e-32	0.83194109127997	0.61	0.21	3.22164143766911e-27	SLC1A4
Recoverec	40	9.69276485378065e-32	1.51443923490938	0.496	0.141	3.25075947666095e-27	LRRC23
Recoverec	40	1.03230432742467e-31	1.08793735664111	0.915	0.536	3.46214225331684e-27	CFLAR
Recoverec	40	1.0343139791608e-31	0.63732769262787	0.816	0.342	3.4688822233095e-27	CUBN
Recoverec	40	1.07804227948068e-31	0.50335010332012	0.433	0.106	3.61553819692232e-27	MERTK
Recoverec	40	7.71464871900703e-31	0.93501885660323	0.539	0.162	2.58733888738058e-26	CFAP54
Recoverec	40	1.04038256351578e-30	0.57981410065575	0.284	0.047	3.48923504151922e-26	LSMEM1
Recoverec	40	1.07888171283531e-30	0.90833527170414	0.78	0.326	3.61835348850708e-26	LINC01934
Recoverec	40	1.4495063305055e-30	0.889299113377482	0.844	0.381	4.86135433124933e-26	SEC24D
Recoverec	40	1.92274677491985e-30	0.58100163417692	0.504	0.141	6.44850813372619e-26	TET1
Recoverec	40	2.32281710094786e-30	0.3781682431096	0.326	0.061	7.79026399315895e-26	AL355075.4
Recoverec	40	2.35347550033486e-30	1.14003481996091	0.858	0.437	7.89308613302305e-26	RRBP1
Recoverec	40	2.46571054892672e-30	0.32127795724894	0.163	0.014	8.26950003899042e-26	KCNB1
Recoverec	40	3.03350826691862e-30	0.802176911181447	0.482	0.135	1.01737800255917e-25	TRNAU1AP
Recoverec	40	3.96655055396608e-30	1.57855498624118	0.823	0.469	1.33030172478914e-25	AC022217.3
Recoverec	40	4.75453627599211e-30	0.72457718489840	0.56	0.171	1.59457637624223e-25	HEXIM1
Recoverec	40	4.76135533775383e-30	0.35501023774447	0.248	0.036	1.59686335317588e-25	FBXL18
Recoverec	40	6.41815923855865e-30	1.14054176636796	0.901	0.484	2.1525222454278e-25	GAB1
Recoverec	40	6.60572423364201e-30	0.79289899191270	0.397	0.095	2.21542779347886e-25	GYS1
Recoverec	40	7.18568991072275e-30	1.14368185538406	0.801	0.438	2.4099366822582e-25	ERCC1
Recoverec	40	8.03465265868607e-30	0.46297813463139	0.348	0.071	2.69466180867013e-25	AC069368.1
Recoverec	40	9.41031497149545e-30	1.32294167098201	0.475	0.135	3.15603143514014e-25	ASTL
Recoverec	40	1.0125327850402e-29	1.43334358590369	0.759	0.353	3.39583245446784e-25	HIST1H2AC
Recoverec	40	1.01750418511724e-29	0.46157704794283	0.27	0.043	3.41250553604621e-25	SNX32
Recoverec	40	1.06157850650883e-29	0.65500041165168	0.511	0.152	3.56032199512931e-25	ALDH1L2
Recoverec	40	1.10349062230414e-29	1.58596406326223	0.738	0.322	3.70088684908363e-25	BMP6
Recoverec	40	1.39448965268778e-29	0.75441455803464	0.652	0.24	4.67683939718428e-25	TMC3-AS1

Recoverec	40	2.26233823554324e-29	0.66927343978082(	0.504	0.146	7.58742997436491e-25	MTO1
Recoverec	40	2.36478686119274e-29	0.93572181801757(	0.695	0.281	7.93102217506821e-25	AC078883.1
Recoverec	40	2.48085398409924e-29	0.54454882452794(	0.149	0.012	8.32028809187202e-25	LDHC
Recoverec	40	2.75512418105253e-29	0.90419531378779	0.447	0.12	9.24013547841398e-25	HIST2H2BF
Recoverec	40	4.51785798985199e-29	1.54506901582589	0.809	0.407	1.51519921263656e-24	UAP1
Recoverec	40	6.50347892291698e-29	0.78045160640522	0.56	0.19	2.1811367611679e-24	KCNN3
Recoverec	40	7.96969706427128e-29	0.32630740655015	0.234	0.033	2.6728770014153e-24	FP236383.1
Recoverec	40	9.12876080245831e-29	0.59343258585361	0.674	0.253	3.06160379792847e-24	FBH1
Recoverec	40	1.15928389743199e-28	0.74194166644499	0.596	0.203	3.88800633520742e-24	SLC35F2
Recoverec	40	1.78133933470776e-28	1.02521336236168	0.773	0.342	5.97425586074288e-24	TTC28
Recoverec	40	1.88335437789718e-28	0.71832275330098	0.596	0.214	6.31639391259156e-24	NUGGC
Recoverec	40	2.26823976980961e-28	0.69008620787450	0.674	0.263	7.60722253998746e-24	KIAA0319L
Recoverec	40	3.11684658446615e-28	1.04856500321926	0.95	0.591	1.04532800749826e-23	ATXN1
Recoverec	40	3.42143283880661e-28	0.83127359926599	0.801	0.364	1.14748014547896e-23	EDEM3
Recoverec	40	4.38665574346877e-28	0.34864443412056	0.22	0.03	1.47119660324456e-23	PPM1N
Recoverec	40	5.99143980811738e-28	0.84632177636845	0.809	0.366	2.00940908284641e-23	LARP1B
Recoverec	40	8.85780738427615e-28	0.89717706561008	0.39	0.094	2.97073144053853e-23	SLC10A1
Recoverec	40	1.14387752787382e-27	0.89760548291945	0.908	0.525	3.83633645298322e-23	MYO1D
Recoverec	40	1.16935034102542e-27	0.62739730460095	0.277	0.05	3.92176717373106e-23	NPL
Recoverec	40	1.36574820823478e-27	1.24771867979952	0.823	0.411	4.5804463407778e-23	TXNDC11
Recoverec	40	1.37912747551413e-27	0.76448923192365	0.752	0.306	4.6253177273793e-23	FAM129A
Recoverec	40	1.69190415097458e-27	0.55082739533150	0.489	0.141	5.67430814153855e-23	AL031777.3
Recoverec	40	1.71476246368711e-27	0.93525076828664	0.404	0.105	5.75097035071384e-23	SCARB1
Recoverec	40	2.66528414708548e-27	0.28831587922478	0.142	0.012	8.93882997249527e-23	THSD8
Recoverec	40	2.71312623043122e-27	0.65347382965721	0.603	0.225	9.09928275162024e-23	MEI1
Recoverec	40	4.23607584484126e-27	0.87829954575219	0.326	0.069	1.42069511684286e-22	ARMH1
Recoverec	40	5.10172336736969e-27	1.02856726949078	0.965	0.736	1.71101598294845e-22	FBXW7
Recoverec	40	6.62489711725985e-27	0.60053830571406	0.319	0.068	2.22185799518661e-22	AC006064.2
Recoverec	40	7.09756902977489e-27	0.72542109570086	0.362	0.087	2.3803827012059e-22	HIST1H2BE
Recoverec	40	7.8704629306765e-27	0.38021104644411	0.496	0.15	2.63959585769028e-22	TRG-AS1
Recoverec	40	1.0886237193509e-26	0.74830264560049	0.745	0.319	3.65102622995904e-22	INPP4A

Recoverec	40	1.4802240103597e-26	0.89799650206089	0.745	0.324	4.96437528594435e-22	ERN1
Recoverec	40	2.18032121407636e-26	0.56023763552289	0.553	0.19	7.3123612877693e-22	CRYBG3
Recoverec	40	2.20428427967167e-26	1.02262952482191	0.837	0.426	7.39272861716285e-22	ZBTB38
Recoverec	40	2.5136542706324e-26	0.92268180843440	0.511	0.167	8.43029369284693e-22	TRIM69
Recoverec	40	2.69031792151201e-26	1.50290705305209	0.723	0.367	9.02278824516699e-22	MXD1
Recoverec	40	3.0969932054953e-26	0.93973482617727	0.404	0.109	1.03866958125901e-21	DGKI
Recoverec	40	3.26701403924703e-26	0.94292524268781	0.617	0.239	1.09569116848267e-21	NFE2L3
Recoverec	40	4.66017399628764e-26	0.704111371014247	0.674	0.281	1.56292915487495e-21	ENAM
Recoverec	40	5.16044259073842e-26	0.71842648608378	0.745	0.323	1.73070923608185e-21	GALK2
Recoverec	40	5.6186969566625e-26	0.29060481721826	0.149	0.014	1.88439858532547e-21	RFTN2
Recoverec	40	6.88458322059644e-26	1.03351512016918	0.518	0.173	2.30895152052363e-21	HIST1H4H
Recoverec	40	8.53121507033929e-26	0.53913436982075	0.745	0.296	2.86119891029039e-21	APOB
Recoverec	40	8.7605360975562e-26	2.06348703901558	0.787	0.467	2.9381085963984e-21	TEX14
Recoverec	40	9.71770711675477e-26	0.93239697592695	0.78	0.362	3.25912461281722e-21	ATP2A2
Recoverec	40	1.00080921445909e-25	0.84875608888699	0.858	0.449	3.3565139434529e-21	SFMBT2
Recoverec	40	2.28942427462421e-25	0.88653635892160	0.858	0.467	7.67827113223469e-21	SPATS2
Recoverec	40	2.36681246617756e-25	0.71316053438158	0.376	0.098	7.93781564906629e-21	PARP16
Recoverec	40	2.69659340232359e-25	0.700711113083738	0.589	0.215	9.04383495271285e-21	AARS
Recoverec	40	3.13361631315701e-25	0.81623292841677	0.943	0.597	1.0509522391066e-20	MTRNR2L8
Recoverec	40	3.26678591074461e-25	0.86482756771307	0.546	0.193	1.09561465874553e-20	MRNIP
Recoverec	40	3.41214889350683e-25	0.62864616183376	0.78	0.339	1.14436649590432e-20	OAT
Recoverec	40	4.30406888718774e-25	0.25714923751009	0.163	0.018	1.44349862338502e-20	AL356356.1
Recoverec	40	4.42282719498715e-25	0.97918061421133	0.887	0.477	1.48332778465479e-20	USP48
Recoverec	40	6.12630966796151e-25	0.93620617899163	0.844	0.482	2.05464173644093e-20	EAF2
Recoverec	40	7.47735527536262e-25	0.99786682989657	0.95	0.687	2.50775541225112e-20	FNDC3A
Recoverec	40	7.57327643596226e-25	1.06207909945986	0.872	0.46	2.53992545109302e-20	XBP1
Recoverec	40	1.23983645820469e-24	1.75242105408269	0.844	0.494	4.15816351352689e-20	CLIC4
Recoverec	40	1.3552374091736e-24	0.86225312562155	0.865	0.474	4.54519522288642e-20	HDLBP
Recoverec	40	1.9342192389553e-24	0.97126413780057	0.752	0.359	6.48698448360828e-20	ITGA6
Recoverec	40	1.95682880204918e-24	0.73801260605334	0.901	0.501	6.56281243631253e-20	PIP5K1B
Recoverec	40	2.36450338014963e-24	0.26708028508997	0.206	0.031	7.93007143634581e-20	PIK3R3

[truncated: 431,280 more chars]
